# Characterizing a long-term chronic heart failure model by transcriptomic alterations and monitoring of cardiac remodeling

**DOI:** 10.18632/aging.202879

**Published:** 2021-04-23

**Authors:** Yingqi Zhu, Qiancheng Wang, Hairuo Lin, Kaitong Chen, Cankun Zheng, Lin Chen, Siyuan Ma, Wangjun Liao, Jianping Bin, Yulin Liao

**Affiliations:** 1 Department of Cardiology, State Key Laboratory of Organ Failure Research, Guangdong Provincial Key Lab of Shock and Microcirculation, Nanfang Hospital, Southern Medical University, Guangzhou 510515, China; 2 Bioland Laboratory, Guangzhou Regenerative Medicine and Health Guangdong Laboratory, Guangzhou 510005, China; 3 Department of Oncology, Nanfang Hospital, Southern Medical University, Guangzhou 510515, China

**Keywords:** transcriptomic profiling, myocardial infarction, heart failure, mice, cardiac remodeling

## Abstract

The long-term characteristics of transcriptomic alterations and cardiac remodeling in chronic heart failure (CHF) induced by myocardial infarction (MI) in mice are not well elucidated. This study aimed to reveal the dynamic changes in the transcriptome and cardiac remodeling in post-MI mice over a long time period. Monitoring C57BL/6 mice with MI for 8 months showed that approximately 44% of mice died of cardiac rupture in the first 2 weeks and others survived to 8 months with left ventricular (LV) aneurysm. The transcriptomic profiling analysis of cardiac tissues showed that the Integrin and WNT pathways were activated at 8 months after MI while the metabolism-related pathways were inversely inhibited. Subsequent differential analysis at 1 and 8 months post-MI revealed significant enrichments in biological processes, including consistent regulation of metabolism-related pathways. Moreover, echocardiographic monitoring showed a progressive increase in LV dimensions and a decrease in the LV fractional shortening during the first 4 weeks, and these parameters progressed at a lower rate till 8 months. A similar trend was found in the invasive LV hemodynamics, cardiac morphological and histological analyses. These results suggested that mouse MI model is ideal for long-term studies, and transcriptomic findings may provide new CHF therapeutic targets.

## INTRODUCTION

Chronic heart failure (CHF) induced by myocardial infarction (MI) causes high rates of hospitalization and mortality, which is a major health burden worldwide [1]. Patients with MI without timely coronary reperfusion would suffer chronic cardiac remodeling [2]. Despite strides forward in the treatment and management of MI [3, 4], the current therapies used to prevent or delay the progression of cardiac remodeling are limited. Thus, it is very necessary to use animal models of post-MI chronic cardiac remodeling to investigate and explore the pathophysiological and transcriptomic changes associated with advanced heart failure to identify new therapeutic targets. In the era of evidence-based medicine, long-term observation has increased in many clinical studies. Vallejo-Vaz *et al.* carried out a 20-year follow-up to evaluate the long-term benefits of lowering low-density lipoprotein cholesterol for the primary prevention of cardiovascular disease in patients enrolled in the WOSCOPS trial [5]. Twenty years of a human life span is comparable to approximately 8 months for mouse [6]. The mouse MI model is the most frequently used animal model for studies on post-MI remodeling, and this model is generally utilized with an observational period of 3–6 weeks [7, 8], However, the long-term (e.g., several months) characteristics as well as transcriptomic level changes of this model have not been well elucidated. After an ischemic insult, the myocardium can exhibit distinct responses, leading to myocardial stunning, hibernation, or activation of cellular mechanisms. However, in long-time ischemic myocardium, structural cardiac remodeling leads to irreversible heart failure due to progressive fibrosis, metabolism pathway switching, and unique transcriptomic alterations [9]. To elucidate the pathogenesis of heart failure and identify novel therapeutic targets, it is important to better understand the molecular bases of the long-term ischemic and failing cardiomyocyte states as well as to identify regulators of the transition between states. Uncovering the gene programs involved in long-term ischemic cardiac dysfunction would enable accurate assessment of the cardiomyocyte condition and prediction of treatment response [10].

To date, studies have rarely addressed the distinct mRNA expression profiles in mice with long-term MI lasting for several months. Very little is known about the expression profile of mRNA transcript in the advanced failing heart. In this study, transcriptomic profiling analyses were performed on cardiac tissue using RNA sequencing (RNA-seq) technology in sham and 8 months post-MI mice. We performed Weighted Gene Co-expression Network Analysis (WGCNA), Gene Set Enrichment Analysis (GSEA) and Protein-Protein Interaction Network (PPI) to detect the characteristics of transcriptomic alterations of heart in advanced heart failure mice. More importantly, we used Principal Component Analysis (PCA) and Gene Ontology (GO) analysis to compare our sequencing data with open access datasets derived from cardiac tissue of 4 weeks post-MI mice (GSE96566; https://www.ncbi.nlm.nih.gov/geo/query/acc.cgi?acc=GSE96566) [11].

In addition to the distinct transcriptomic changes, we found that the electrocardiogram (ECG), echocardiography, invasive LV (left ventricle) hemodynamics, heart and lung histology, and molecular biomarkers also showed dynamic change during post-MI remodeling in an 8-month time period.

We present the following article/case in accordance with the animal research reporting checklist.

## RESULTS

### Confirmation of MI success

**Figure 1:**
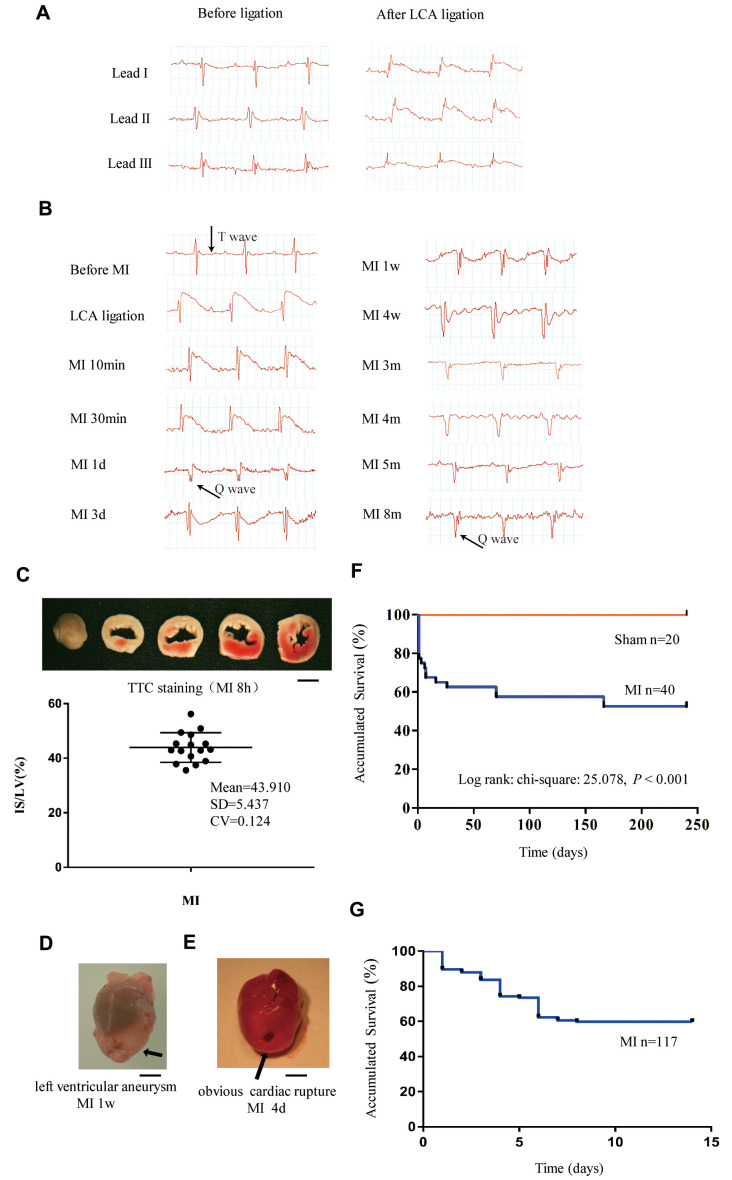
**Monitoring of long-term MI mouse model for 8 months.** (**A**) Limb leads I, II and III of electrocardiogram (ECG) reliably reflect the acute ischemia induced by left coronary ligation in C57BL/6 mice. (**B**) Lead II ECG can reflect different stages of myocardial infarction (MI) for a time period of 8 months. (**C**) Triphenyl tetrazolium chloride (TTC)-stained sections of heart in mice with MI for 8 h; the infarct size (IS)/ left ventricle (LV) was approximately 44% in the MI groups. (**D**) Representative left ventricular aneurysm formed 1 week after MI; (**E**) Example necropsy images of a dead mouse showing obvious rupture in the left ventricle free wall. (**F**): Kaplan–Meier survival analysis of mice subjected to MI or sham operation for 8 months. (**G**) Kaplan–Meier survival analysis of mice subjected to MI or sham operation for 14 days, *n* = 114 in MI group; Scale bar =2 mm for panel (**C, D** and **E**).

We generated a mouse MI model by ligation of the left coronary artery (LCA). Lead II ECG was used to monitor the evolution of ECG during the time course. As shown in Figure 1A, the ST segment with an upward roach back elevation immediately appeared in leads I, II and III when the LCA was tied, indicating the occurrence of myocardial ischemia and that the limb leads reflected the myocardial ischemia induced by LCA occlusion. Therefore, we employed lead II ECG to assess the correct ligature placement and monitor ECG changes in response to MI throughout the time course. The elevated ST segment started to fall after 30 minutes of MI (Figure 1B). One day after MI, the ST segment dropped to a baseline level, and the T wave became inverted to be recorded at day 3rd, this trend recovered during 1 month and then persisted to 8 months after MI (Figure 1B). A pathological Q wave appeared 1 day after MI and persisted to even 8 months as pointed out by the black arrows in Figure 1B. Acute infarct size was calculated from the TTC (Triphenyl tetrazolium chloride) staining of heart sections. As shown in Figure 1C, the brick-red staining indicated viable tissue, while the unstained white sections represented infarct area. Our approach showed similar infarct size (infarct area/LV area) with low dispersion among different mice (Figure 1C). In this model, the ventricular aneurysm was generally formed at one week after MI (Figure 1D). Most of the mice that survived after 2 months survived until as long as 8 months, but they exhibited decreased exercise tolerance and shortness of breath (Supplementary Figure 1 and Supplementary Video 1).

### Survival rate over eight months

Kaplan–Meier survival analysis indicated that the total mortality was 42% for mice with MI and 0% for sham-operated mice during the 8 months of observation (Figure 1F). The first 2-week mortality rate after MI remained at approximately 40% when the sample size was enlarged from 40 to 117 (Figure 1G). Most deaths occurred in the first 10 days after MI due to cardiac rupture (Figure 1E). For the MI mice that survived to 4 weeks, the mortality afterward was very low, which may have been due to the individual differences in compensation reactivity and tolerances.

### Identification of transcriptomic status in the hearts of mice with MI for 8 months

To date, studies have rarely addressed the distinct mRNA expression profiles in long-term MI models as long as 8 months. To obtain a comprehensive understanding of transcriptome alterations during the long term post-MI status of mice, RNA-seq measurements were performed on cardiac tissues in the sham group and 8-month MI group (Supplementary raw data).

**Figure 2:**
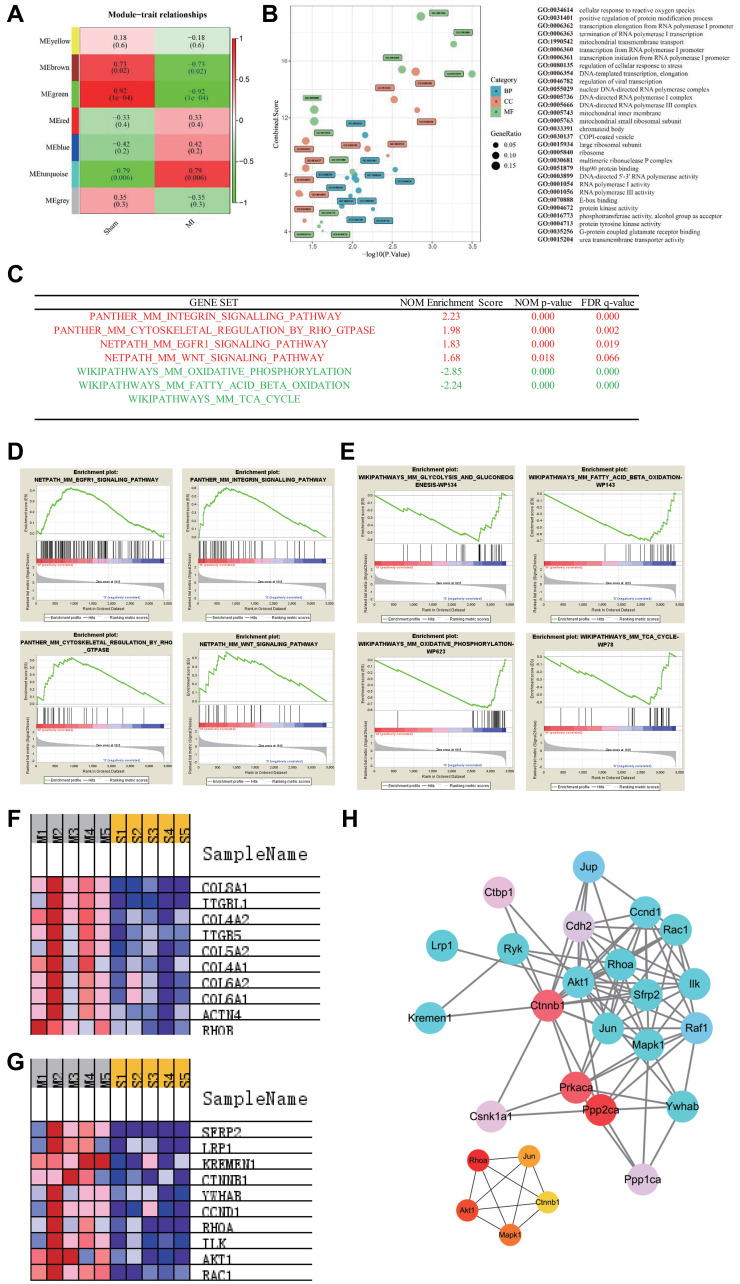
**Pathways enrichment associated with advanced heart failure.** (**A**) WGCNA identified demonstrated 7 modules by average linkage clustering. (**B**) Gene Ontology analysis of genes in the green module, which were more correlated with advanced heart failure. (**C**) List of the four significantly up-regulated (marked red) and four down-regulated gene sets (marked green) associated with advanced heart failure enriched in GSEA analysis. (**D**–**E**) Enrichment plots of four significantly up-regulated and down-regulated pathways. (**F**) Heatmap of the enriched genes in the Integrin signaling pathway. (**G**) Heatmap of the enriched genes in the WNT signaling pathway. (**H**) Protein-protein interaction network of WNT signaling pathway and hub genes involved in this network.

We used the WGCNA package to construct co-expression modules for cluster analysis. Based on the histological grade by average linkage clustering, 7 modules were identified and the green module, which contained 86 genes, had the highest significant correlation coefficient with the trait (Figure 2A). GO enrichment analysis was then performed using these 86 genes (Supplementary Table 1), and the results in Figure 2B shows that the genes related to RNA Polymerase-1 mediated biological processes were significantly upregulated. To further elucidate the potential mechanisms of gene alterations in 8 months post-MI regulation, we performed GSEA analysis and a total of 3398 genes were detected in the profiling. All the genes were grouped into 893 gene sets. According to the gene set size filters (min = 15, max = 500), 771 gene sets were filtered out and the remaining 122 gene sets were used in the analysis. The GSEA analysis showed that 9 gene sets were significantly enriched at *P* value < 0.01 in the MI group (Supplementary Table 2), while 6 gene sets were significantly enriched in the sham group (Supplementary Table 3). Gene sets enrichments revealed that the Integrin and WNT pathways were activated and that the metabolism-related pathways (fatty acid beta oxidation, glycolysis and TCA cycle pathways) were inversely inhibited 8 months after MI (Figure 2C–2E and Supplementary Figure 2). In addition, the expression of the top 10 genes involved in the abovementioned pathways are shown in Figure 2F–2G and Supplementary Tables 4–8. We then screened the WNT signaling pathway-related genes that were significantly up-regulated in the GSEA analysis. The top four contributing genes in this pathway were SFRP2, LRP1, KREMEN1, CTNNB1. We next used the String database to analyze PPI. MCC selection of the top ranked nodes in the network demonstrated that CTNNB1 was located at the core of the network, indicating that CTNNB1 is the hub gene (Figure 2H). Thus, these findings suggested that CTNNB1 should play an important role in the transcriptome regulation of long-term MI.

### Alterations of molecular signature from 1 month to 8 months after MI

**Figure 3:**
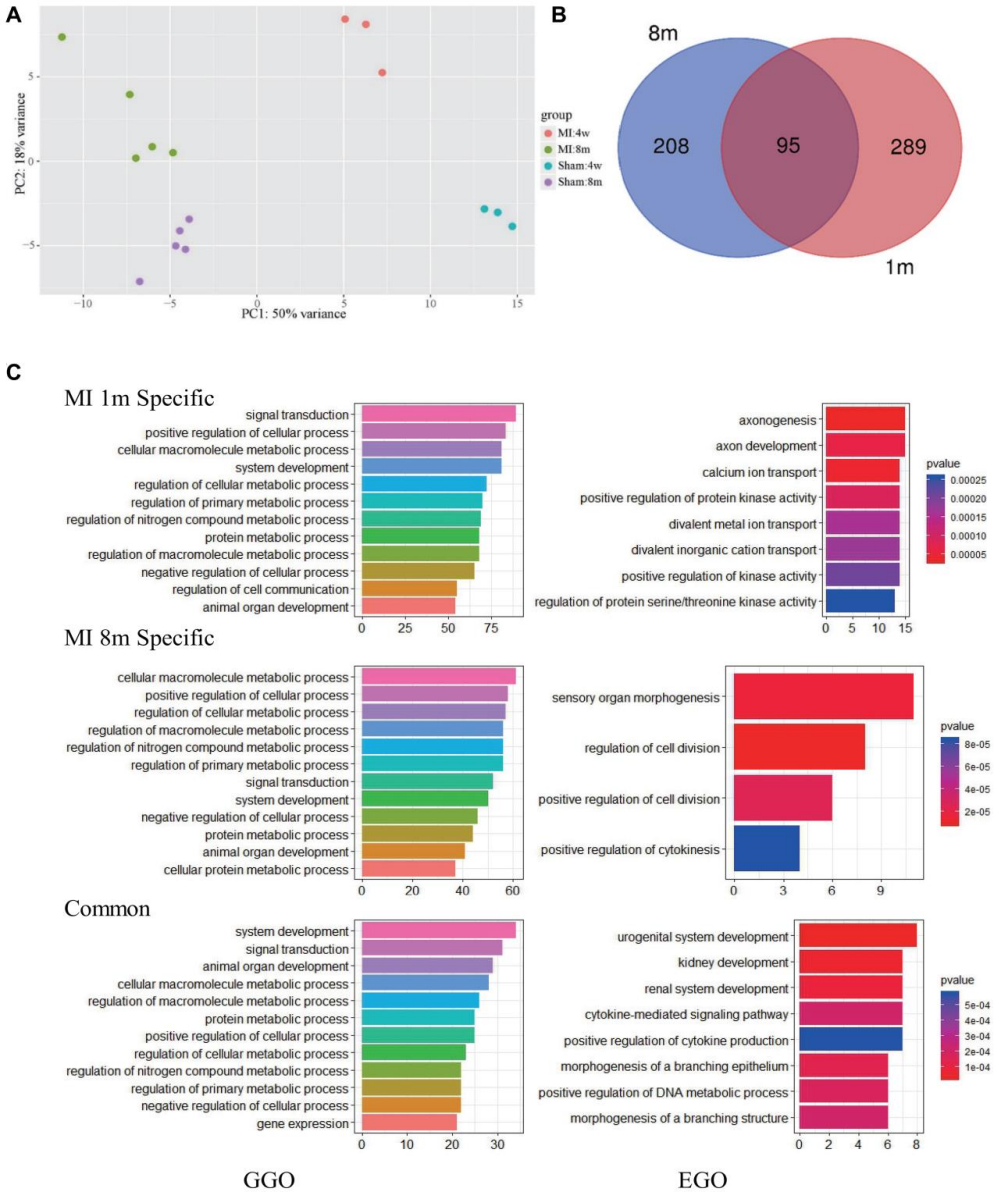
**Pathways most affected at 1 month and 8 months after MI.** (**A**) PCA analysis show that the transcriptome of mice after LCA ligation changes significantly and varies with time. (**B**) Overlap of significantly modulated genes between 1 month and 8 months post-MI. (**C**) GGO (left) and EGO (right) analysis of pathways affected by long term MI.

Global clustering of the transcriptomic profiles of mice at 1 month and 8 months after MI was performed. PCA with arbitrary parameters was used to identify the differentially expressed genes in the different status through the distribution position. The results indicated that there was a large difference in gene expression between the two time points of MI (Figure 3A and Supplementary raw data). Differential analysis demonstrated that 303 and 384 genes were differentially regulated at 1 month and 8 month post MI respectively, according to the threshold of padj (adjust *P* value) <0.05 and that 95 genes were simultaneously differentially regulated at both time points (Figure 3B and Supplementary Table 9). Most genes showed a continuous alteration trend at both time points, but some genes, including Kif2c, Pira2 and Rassf10, showed an inverted trend (marked by red in Supplementary Table 9). Enrich Gene Ontology (EGO) and Group Gene Ontology (GGO) analysis revealed that metabolism related pathways persisted throughout the time course. EGO analysis revealed more independent differences than GGO, including the enrichment of axonogenesis related biological processes at 1 month as well as sensory organ morphogenesis and cell division regulation at 8 months (Figure 3C). With regard to the common altered biological processes in EGO analysis, the urogenital and renal system development made the greatest contribution (Figure 3C).

### Verifying time-course changes of echocardiography

**Figure 4:**
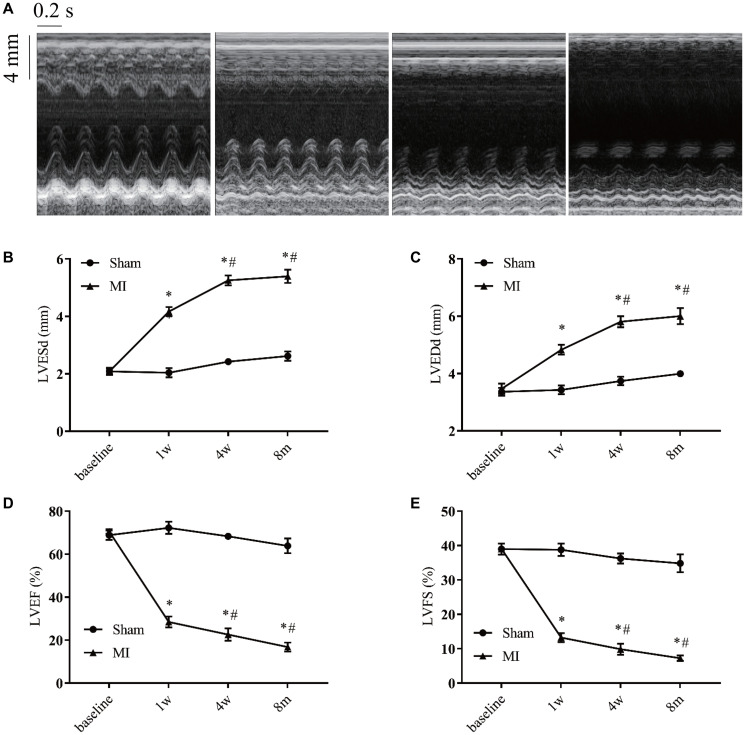
**Serial echocardiographic assessment of post-infarction remodeling.** (**A**) Representative recordings of M-mode echocardiographic images at indicated time points. (**B**) Left ventricular end-systolic diameter (LVESd). (**C**) Left ventricular end-diastolic diameter (LVEDd). (**D**) Left ventricular systolic function presented by the left ventricular ejection fraction (LVEF). (**E**) Left ventricle fractional shortening (LVFS). ^*^P < 0.05 vs the corresponding time point in the sham group. ^#^*P* < 0.05 compared with the prior time point in the MI group. *n* = 8–10 at various time points.

M-mode echocardiography was used to evaluate the cardiac remodeling and verify heart function alteration (Figure 4A). We found a progressive increase in left ventricular end-systolic (LVESd) and end-diastolic diameters (LVEDd) as well as a progressive decrease in the left ventricular ejection fraction (LVEF) and fractional shortening (LVFS) during the first 4 weeks after MI compared to the sham group (Figure 4B–4E). Four weeks later, those parameters were maintained at relatively stable levels.

### LV hemodynamic characterization in long term post-MI model

**Figure 5:**
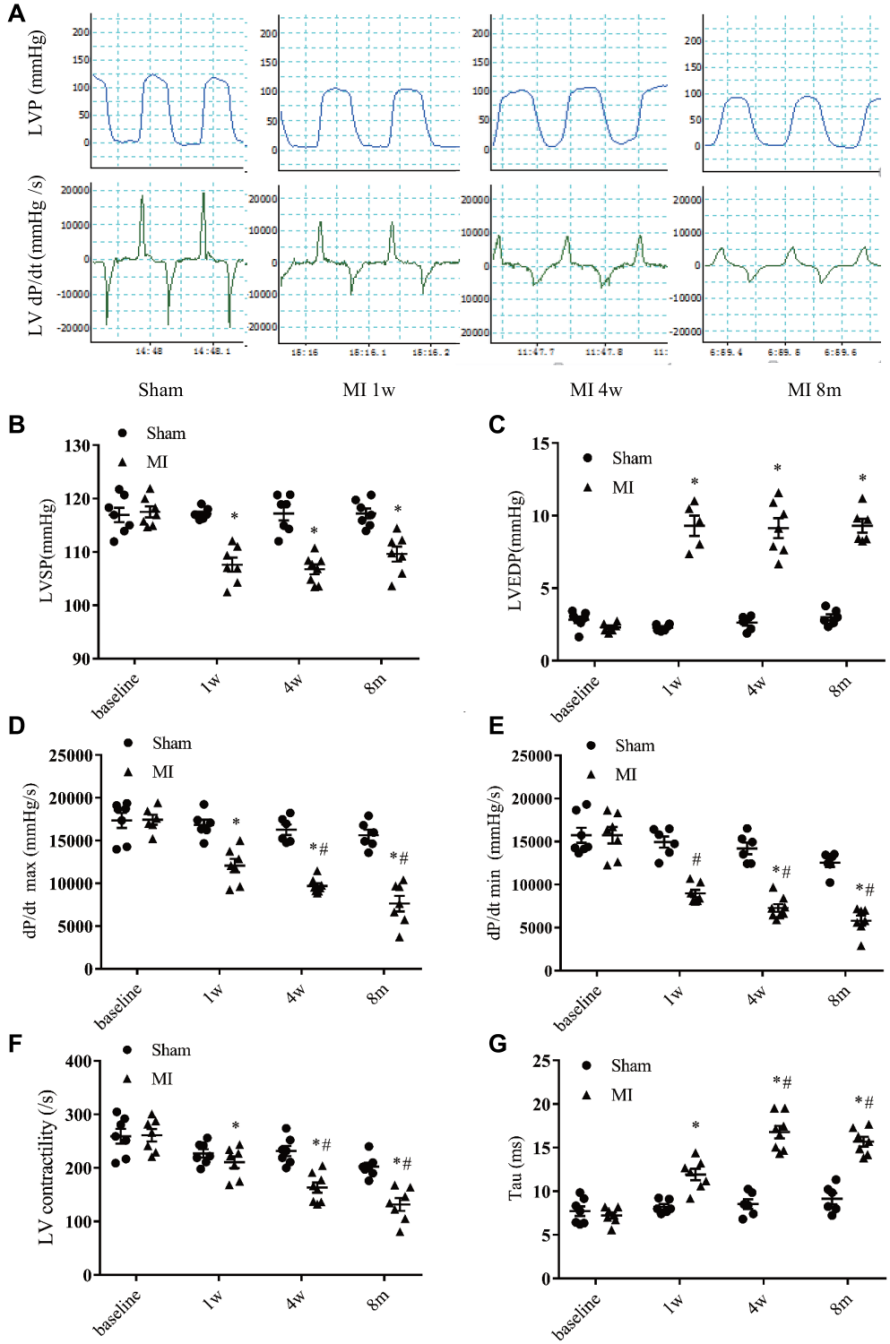
**Hemodynamics assessment of left ventricular function.** (**A**) Representative recordings of left ventricular (LV) pressure and pressure change rate (dp/dt). (**B**) LV systolic pressure. (**C**) LV end-diastolic pressure. (**D**) Maximum increasing rate of LV pressure (dp/dt max). (**E**) Maximum descending rate of the LV pressure (dp/dt min). (**F**) LV contractility index (dp/dt max divided by the pressure at the time of dp/dt max). (**G**) Exponential time constant of relaxation (Tau). ^*^P < 0.05 compared with the sham group. ^#^*P* < 0.05 compared with the prior time point in the MI group. *n* = 6–10 at various time point.

To characterize the long term post-MI cardiac efficiency alterations, the LV hemodynamics in MI or sham mice were measured at 4 time points (Figure 5A). Compared with that in the sham mice, the systolic pressure (LVSP) was decreased and end diastolic pressure (LVEDP) was significantly increased in MI mice. Moreover, the LV pressures were stable at 1 week, 4 weeks, and 8 months after MI (Figure 5B–5C). A time-dependent reduction of systolic function was evidenced by a progressive decrease in the maximum rates of change in the LV pressure (*dP/dt max*) and LV contractility in MI mice (Figure 5D–5F). After MI, *dP/dt min* was significantly decreased and persisted to 8 months, while the exponential time constant of relaxation (τ) was progressively extended (Figure 5E–5G), indicating a dysfunction of diastolic function.

### Characterizing morphological remodeling

**Figure 6:**
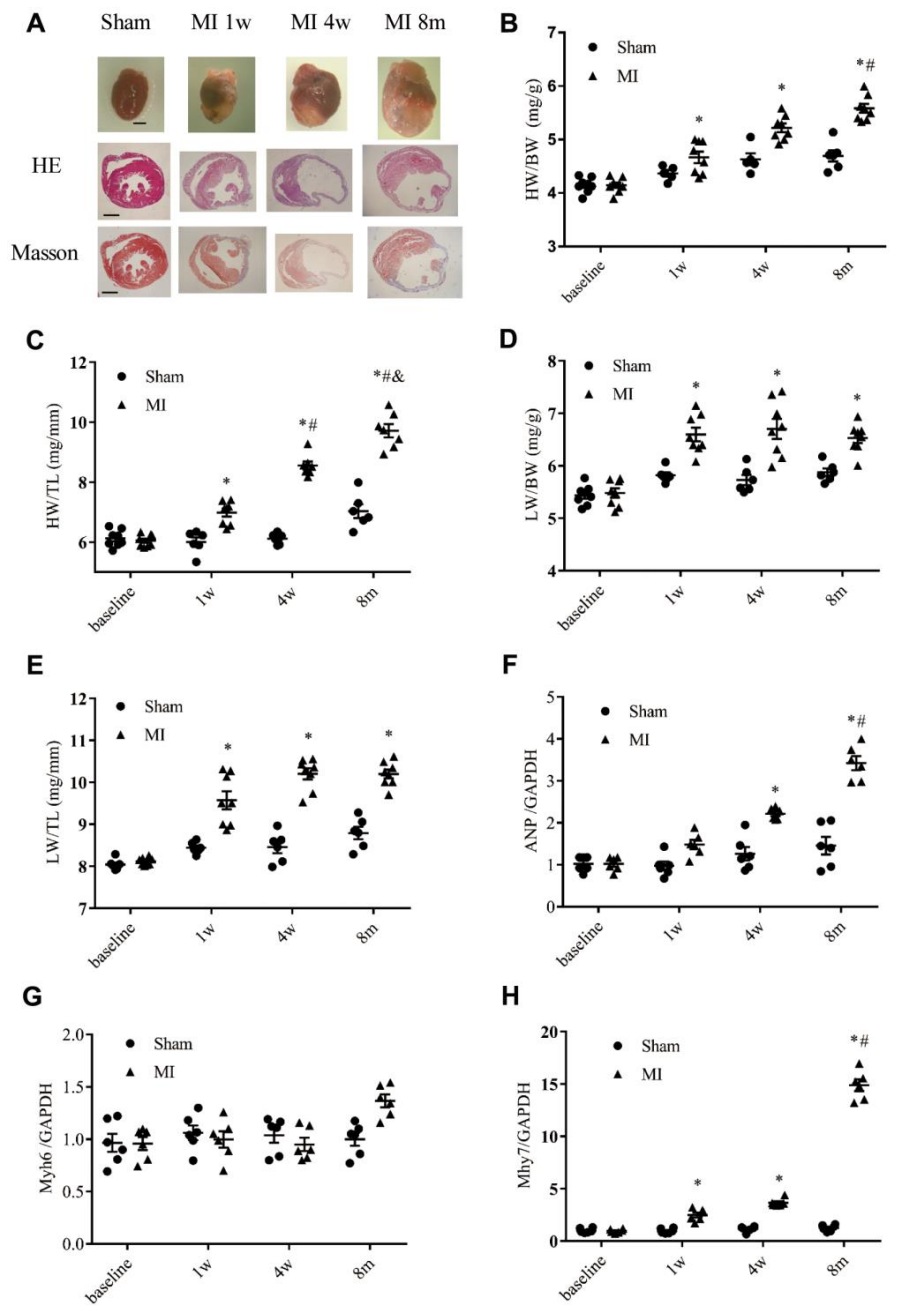
**Cardiac remodeling validated by morphological and histological analysis.** (**A**) Representative images of whole hearts and cross sections of hearts stained with hematoxylin-eosin staining (HE) and Masson's trichrome staining from different time points (scale bar = 2 mm). (**B**) Heart weight to body weight ratio (HW/BW). (**C**) Heart weight to tibia length ratio (HW/TL). (**D**) Lung weight to body weight ratio (LW/BW). (**E**) Lung weight to tibia length ratio (LW/TL); *n* = 8–10 at different time points. (**F**) Expression of the gene encoding ANP (atrial natrium peptide). (**G**) Expression of the gene encoding α-MHC (α-myosin heavy chain, Myh6). (**H**) Expression of β-MHC (Myh7); *n* = 6 at each time point. ^*^P < 0.05 vs. the corresponding time point in the sham group. ^#^*P* < 0.05 compared with the prior time point in the MI group.

Consistent with the molecular changes, a time-dependent enlargement of cardiac cavity after MI was observed (Figure 6A and Supplementary Figure 3). At 1 week to 8 months after MI, both the heart weight/body weight ratio and heart weight/tibia length ratio were significantly higher than in the sham group, and both ratios were even higher at 8-months after MI (Figure 6B–6C). Moreover, the heart weight/tibia length ratio at 8 months after MI was significantly higher than that at 4 weeks post-MI (Figure 6C). The lung weight/body weight ratio and the lung weight/tibia length ratio were significantly higher at the 3 indicated time points in the post-MI group than the corresponding time points in the sham group (Figure 6D–6E), and the lung weight/tibia length ratio was higher at 4 weeks and 8 months post MI than at 1 week post-MI (Figure 6D–6E), a liver congestion indicated by larger LiW/BW and LiW/TL was also observed (Supplementary Figure 4A and 4B). We next performed real-time PCR analysis to verify the biomarkers of heart failure in MI mice at the indicated time points based on the transcriptomic analysis (Figure 6F–6H). Both the ANP and β-MHC genes were significantly upregulated at 4 weeks and 8 months after MI (Figure 6F and 6H). In accordance with the GSEA analysis, the gene expression of α-MHC was not significantly changed (Figure 6G), and the expression levels of ANP and β-MHC were much higher at 8 months post-MI than 4 weeks post-MI (Figure 6F and 6H).

## DISCUSSION

In the present study, we characterized a long-term mouse model of heart failure. The following observations were made: (1) Limb lead II ECG is sufficient to reflect the phases of MI such as acute ischemia and old MI; (2) Most mice that survive to 4 weeks after MI can survive to as long as 8 months; (3) We first summarized the genetic alterations at 8 months after MI by WGCNA and GSEA. PCA and GO analysis suggested that the transcriptomic profiles are different at 1 month and 8 months after MI. (4) Echocardiography, invasive measurement of LV hemodynamic, histological examinations and molecular examinations successfully verified the predicted changes in molecular pathways and characterized this long-term chronic heart failure mouse model. Therefore, this long-term MI model is suitable as a chronic heart failure model to investigate the long term effects of various therapeutic approaches, which would be much more clinically relevant.

The MI mouse model has been frequently used to elucidate the mechanisms involved in myocardial injury and cardiac remodeling and to investigate the effects of therapeutic interventions at preclinical stages. Conventional treatments for post-MI heart failure can partly delay cardiac remodeling, improve quality of life, and reduce risk of heart failure-related hospitalization and death [12, 13]. However, the 5-year mortality rate of heart failure remains as high as approximately 50%. Thus, it is necessary to identify new therapeutic approaches and observe their longer-term effects in animal models. However, the observational period of post-MI heart failure in the MI mouse model is generally 3–6 weeks and occasionally, a maximum of 1 year [14]. In the present study, we observed mice with MI for 8 months and analyzed their survival rate. Interestingly, the mortality rate of mice after MI decreased significantly 4 weeks after MI and most of the surviving mice survived to 8 months with LV aneurysm. We also found that there are two dying windows, the first one occurred the first 10 days after MI, in which approximately one-quarter of mice died of cardiac rupture, which was in consistent with previous studies [15, 16]. The second one was 2–8 weeks after MI, in which an additional 20% of mice died of heart failure. Most of the mice that survived after 2 months survived until as long as 8 months, but they exhibited LV aneurysm, marked cardiac remodeling, decreased exercise tolerance and shortness of breath. During 1–8 months after MI, about 15% (4/27) mice died of decompensated heart failure (Figure 1F). Till to 8 months, about 40% (9/23) lived MI mice had obvious breath shortness and a decrease of running distance (fatigue), indicating that decompensated heart failure could occur in this 8-month MI model. These findings suggested that the long-term mouse MI model is ideal to test the interventional effects of novel therapies on cardiac remodeling.

Characterizing the transcriptional profiles in the hearts of mice at 8 months after MI might provide clues for identifying new therapeutic targets for heart failure. We observed a differential expression of the RNA Polymerase-1 pathway subunit in end-stage heart failure. RNA polymerase-1 is necessary in ribosomal RNA production, driving cell growth and driving cell division via its essential component of cellular protein synthesis machinery [17], and it also plays a vital role in determining the fate of cells when undergoing stress situations [18, 19]. It has been reported that dysregulation of polymerase-1 transcription is related to the etiology of human diseases [20].

The ability to repair injured tissue after MI is affected by numerous complex cellular and molecular pathways [21]. Our present GSEA analysis demonstrated that the up-regulated and down-regulated gene sets in the MI model were statistically enriched for growth and metabolism regulation, respectively. The activation of pathways promoting cell growth, proliferation, differentiation and regeneration, e.g., the WNT signaling pathway, were still increasing at 8 months after MI. Previous study has shown that active WNT signaling is markedly increased in the myocardium beginning 7 days post-MI, however, the observation time only lasted 3 weeks [22]. Unexpectedly, our findings showed that the WNT pathway activation was still increasing at 8 months after MI. Activation of WNT pathway may be one of the important factors by which long-term MI leads to exacerbated cardiac dysfunction. We found that CTNNB1 is the only top ranked differentially expressed gene located at the core of the network. Previous studies have shown that CTNNB1 is mostly related to the occurrence and development of tumors, including Craniopharyngioma [23], Hepatocellular [24] and Gastric cancer [25], Studies have also shown that CTNNB1 has an important relationship with heart development [26]. WNT/CTNNB1 are required for cardiogenesis, but the specific role of CTNNB1 in cardiac repair after MI remains unclear. Thus, CTNNB1 should be used as a potential target gene for the further mechanistic approaches. Another pathway that was significantly upregulated in the MI group was the Integrin signaling pathway, which has been proved to participate in multiple critical cellular processes in cardiomyocytes including adhesion, extracellular matrix organization, signaling, survival, and proliferation. Integrins are mechano-transducers and are particularly relevant for a contracting muscle cell, and they translate mechanical information to biochemical information [27]. We found that the most highly up-regulated gene in the integrin pathway was COL8A1, which has been confirmed to be involved in the progression of cardiac dilation [28] and TAC-induced cardiac pathological remodeling [29]. Due to the small size of the mouse heart, we used the whole heart rather than a special portion of the heart to perform transcript analysis. Therefore, it is unclear where the altered gene expression occurred. As shown in Supplementary Figure 5, the deposition of β-catenin and collagen-8 protein occurred in both the infarct area and border area sections in mice at 8 months post-MI, suggesting that the altered expression of their corresponding coding genes, CTNNB1 and COL8A1 occur in both the infarct and border area. Our findings indicated that CTNNB1 and COL8A1 may play an important role in the cardiac remodeling progress of long term MI. Similarly, we found that the activity of either fatty acid or oxidative phosphorylation pathways mostly processed by mitochondrial was still in decreasing phase even at 8 months after MI, and this long term persistent dysregulation of energy metabolism may provide targets for improving heart failure. Oxidative metabolism in mitochondria is the main energy source of the heart, and maladjustment to myocardial energy stress leads to metabolism dysfunction and in turn exacerbates a vicious cycle that increases myocardial damage [30]. Overall, efforts to sustain energy homeostasis are promising therapeutic strategies to combat heart failure.

Differential gene analysis using PCA showed that 95 genes were continuously differentially regulated during the 8-month monitoring period. GGO and EGO analyses indicated that genes associated with cytokine pathways and metabolic processes were commonly regulated at 1 month and 8 months after MI. Cytokine signaling is an important part of regulator processes throughout the human body as cytokines bind to receptors on target cells and activate a cascade of intercellular signals, such as the protein kinase transduction cascade [31], and these signals may have a sustained effect on post-MI cardiac remodeling.

In summary, our findings indicated that most mice with chronic MI and marked cardiac remodeling and LV aneurysm can survive for more than 8 months. The time-course of cardiac remodeling was noninvasively evaluated with ECG and echocardiography, while molecular and histological changes were examined at the indicated time points. We first addressed the transcriptomic characteristics at 8 months after MI by transcriptomic profiling analysis using RNA-seq. We also compared the similarities and differences of early and advanced transcriptomic expression by analyzing published sequencing data. Our identification of differentially expressed genes and pathways at various time points after MI will ultimately facilitate the development of new therapeutics for chronic heart failure.

## MATERIALS AND METHODS

### MI Model

The experiments were performed in accordance with our institution’s guidelines for animal research that conform to the Guide for the Care and Use of Laboratory Animals (National Institutes of Health Publication, 8th Edition, 2011). Approval for this study was granted by our university’s ethics review board. Male C57BL/6 mice aged 10–12 weeks and weighing 25–35 g were intraperitoneal anesthetized with a mixture of xylazine (5 mg/kg) and ketamine (100 mg/kg), intubated with PE-90 tubing, and ventilated with room air using a mouse mini-ventilator. The respiration rate was set between 110–130 times/minute. Thoracotomy was performed between the 3rd and 4th rib to expose the left coronary artery (LCA). An 8–0 nylon suture was then placed around the LCA at 2 mm from the tip of the left auricle and the vessel was ligated as previously described [32, 33]. Coronary occlusion was confirmed by ST segment elevation on the ECG monitor [34], and only mice with a ST elevation in lead II >1/2 of R wave were included in this study.

At the indicated time points, the mice were sacrificed by an overdose of anesthetic (pentobarbital sodium;150 mg·kg−1, i.p.), and their hearts and lungs were harvested for further analysis. Myocardial infarct size was determined using TTC staining [35].

### Exercise exhaustion test

After 3 days of acclimatization to treadmill exercise, mice from the sham 8 m and MI 8 m groups were forced to receive an exhaustion test. Animals ran uphill (20°) on the treadmill (ShangHai Biowill Co., Ltd.) starting at a warm-up speed of 5 m/min for 4 min, after which speed was increased to 14 m/min for 2 min. Every subsequent 2 min, the speed was increased by 2 m/min until the animal was exhausted. Exhaustion was defined as the inability of the animal to return to running within 10 sec of direct contact with an electric-stimulus grid. Total running distance in a period from starting to the occurrence of exhaustion for each mouse was calculated.

### Echocardiography

Transthoracic echocardiography measurements were performed using the VisualSonics Vevo 2100 high-resolution ultrasound imaging system (Fujifilm VisualSonics, Inc., Toronto, Canada) with a MS400 transducer. Ultrasound was performed to evaluate cardiac function at the indicated time points (baseline, 1-week, 4-week and 8-months in the post-MI or sham groups). The mice were immobilized with isoflurane anesthesia and placed in a supine position on a heated pad. Heart rates were approximately 400–500 beats/minutes under anaesthesia, A two-dimensional short-axis view of the LV for guided M-mode was obtained perpendicular to the ventricular septum and posterior wall at the tip of the mitral leaflets. The LVESd, LVEDd and LV wall thickness were directly measured, while the LVEF and LVFS were automatically obtained using the calculation system.

### Invasive LV hemodynamics

LV hemodynamics were evaluated before the animals were sacrificed using Power Lab software (blood pressure module; AD Instruments, Australia) [36, 37]. Briefly, mice from each group were anesthetized with pentobarbital (light anesthesia with 30 mg/kg, ip), and were ventilated. A Millar catheter (Millar Instruments, Inc., Houston, TX) was inserted via the right carotid artery and carefully introduced into the LV to measure the LVSP, LVEDP, and maximum rates of change in the LV pressure (dp/dt max and dp/dt min, respectively). Both the contractility index and the exponential time constant of relaxation (τ) were calculated using the Blood Pressure Module software.

### Histological analyses

We performed all histological analyses on fixed (4% paraformaldehyde) heart tissue. After fixation, the samples underwent a series of dehydrations, and 4-μm sections were obtained after the samples were embedded in paraffin blocks. HE (hematoxylin-eosin) staining was used to determine cardiomyocyte morphology [38]. Myocardial fibrosis and old myocardial infarct size were assessed with Masson’s trichrome staining [39]. Immunohistochemical staining was performed in different sections of the infarcted hearts to determine where the altered gene expression occurred according to a previously described protocol [40].

### Immunohistochemistry

Heart tissues were harvested from sham 8m and MI 8m mice, fixed in 4% paraformaldehyde and embedded in paraffin. Then 4 μm sections were obtained for immunostaining. After antigen retrieval by heating in citrate buffer (pH 6.0), the sections were incubated with primary antibodies overnight at 4°C. β-catenin (1:100, Santa Cruz, CA, USA), collagen-8 (1:100, Abcam, MA, USA). The sections were incubated with an HRPlabeled secondary antibody for 1 hour. Then Dako EVision + System-HRP (DAB) were used to visualize the indicated protein staining. Images were captured using an upright microscope (Olympus, Japan).

### Real-time PCR

Total tissue RNA was extracted from hearts with a total RNA isolation system (R4369-01, Omega, USA). The sequences of the primers were designed by Primer Premiers 6.0. We measured the relative expression levels of genes encoding alpha-myosin heavy chain gene (α-MHC, Myh6), beta-myosin heavy chain gene (β-MHC, Myh7), atrial natriuretic peptide gene (ANP) and glyceraldehyde-3-phosphate dehydrogenase (GAPDH) in hearts harvested from mice with MI or sham-operated mice using the TaqMan real-time PCR method and a Quantitect SYBR Green RT-PCR kit (DRR420A, Takara, Japan), as described elsewhere [41].

### Bioinformatics analysis

For each sample, the raw reads obtained from the sequencing instrument were processed to obtain clean reads by removing sequencing adapters and low quality. High-quality sequences were aligned to the *Mus musculus* 10 database using Hisat2 2.0.5 software. Subsequently, StringTie 1.3.3b software was employed to assemble and quantify the transcripts, from which we got a raw gene count matrix. Differential expression analysis was conducted using software DESeq2 1.20.0 software. PCA performed by R was used for difference pre-analysis and sample classification. The WGCNA R software package was used to perform analysis on various aspects of the weighted correlation network [42]. We screened 75% of the gene based on the absolute deviation of the median (MAD), and genes with an MAD at least greater than 1 (4437 genes) were subjected to cluster analysis. GSEA was performed to analyze ranked lists of all available genes with no threshold, and our data were analyzed by gene sets from specialized mouse databases (GSKB; http://ge-lab.org/gskb/). The Protein-protein interactions network (PPI) was analyzed by using String database (Version 11.0). The Visualized network was mapped using Cytoscape software and CytoHubba was used to calculate the top-ranked nodes in the network [43].

### Statistical analysis

All the data are expressed as the mean ± standard error of mean (SEM), and significant differences between two experimental groups were calculated using the *t* test. Survival rates were calculated according to the Kaplan–Meier method and *P* values less than 0.05 were considered to be statistically significant [44].

### Statement

No human studies were carried out by the authors for this article.

### Availability of data and materials

The datasets used and/or analyzed during the current study are available from the corresponding author on reasonable request.

## Supplementary Materials

Supplementary Figures

Supplementary Tables 1-9

Supplementary Video 1

Supplementary raw data
