# Supplementary material for: Characterizing a long-term chronic heart failure model by transcriptomic alterations and monitoring of cardiac remodeling
Source: Aging (Albany NY). 2021 Apr 23;13(10):13585–614. doi: 10.18632/aging.202879 (PMC8202904; doi:10.18632/aging.202879)
Supplement: Supplementary raw data [file aging-13-202879-s004.pdf]

Supplementary raw data. Supplementary raw data of all gene count matrix and differentially expressed genes at 1 and 8 months after MI.

1m.gene.count.matrix

gene_id	m1	m2	m3	s1	s2	s3
ENSMUSG00000045915	0	0	0	0	0	0
ENSMUSG00000086512	0	0	0	0	0	0
ENSMUSG00000086515	0	0	0	0	0	0
ENSMUSG00000086517	0	0	0	0	0	0
ENSMUSG00000062826	0	0	0	0	0	0
ENSMUSG00000086519	0	0	0	0	0	0
ENSMUSG00000101049	0	0	0	0	0	0
ENSMUSG00000112484	0	0	0	0	0	0
ENSMUSG00000112485	0	0	0	0	0	0
ENSMUSG00000089450	0	0	0	0	0	0
ENSMUSG00000112487	0	0	0	0	0	0
ENSMUSG00000112480	0	0	0	0	0	0
ENSMUSG00000093346	0	0	0	0	0	0
ENSMUSG00000112482	2	0	0	0	0	0
ENSMUSG00000093344	0	0	0	0	0	0
ENSMUSG00000049506	0	0	0	0	0	0
ENSMUSG00000039269	0	0	0	0	0	0
ENSMUSG00000100948	0	0	0	0	0	0
ENSMUSG00000108612	2	8	3	5	10	11
ENSMUSG00000009646	0	0	0	0	0	0
ENSMUSG00000036523	117	87	85	14	46	18
ENSMUSG00000089451	0	0	0	0	0	0
ENSMUSG00000100949	0	0	0	0	0	0
ENSMUSG00000094310	1	0	2	0	0	0
ENSMUSG00000089452	0	0	0	0	0	0
ENSMUSG00000109088	0	0	0	0	0	0
ENSMUSG00000109089	4	11	16	7	7	15
ENSMUSG00000055109	0	0	0	0	0	0
ENSMUSG00000112739	4	5	5	10	0	6
ENSMUSG00000109080	0	8	0	0	0	0
ENSMUSG00000109081	0	0	0	0	0	0
ENSMUSG00000109083	0	0	0	0	0	0
ENSMUSG00000112732	0	0	0	0	0	0
ENSMUSG00000055102	0	0	0	0	0	0
ENSMUSG00000109086	3	3	1	1	1	3
ENSMUSG00000113288	2	2	0	4	0	3
ENSMUSG00000106300	0	0	0	0	2	0
ENSMUSG00000106301	0	0	0	0	0	0
ENSMUSG00000106304	0	3	10	11	2	7
ENSMUSG00000091292	0	0	0	0	0	0
ENSMUSG00000113281	0	0	0	0	0	0
ENSMUSG00000113280	0	0	0	0	0	0
ENSMUSG00000113283	0	0	0	0	0	0
ENSMUSG00000113282	0	0	0	0	0	0
ENSMUSG00000113285	0	1	0	0	0	0
ENSMUSG00000113284	0	0	0	0	0	0
ENSMUSG00000113287	0	0	0	0	0	0
ENSMUSG00000097044	0	0	0	0	0	0
ENSMUSG00000097040	0	0	0	0	0	1
ENSMUSG00000097041	0	0	0	0	0	0
ENSMUSG00000097043	0	0	0	0	0	0
ENSMUSG00000097049	0	0	0	0	0	0
ENSMUSG00000100944	0	1	0	0	0	0
ENSMUSG00000071065	0	3	0	0	0	0

ENSMUSG00000046881	8	2	8	6	5	6
ENSMUSG00000046886	0	0	0	0	0	0
ENSMUSG00000114889	0	0	0	0	0	0
ENSMUSG00000114885	0	0	0	0	0	0
ENSMUSG00000114883	0	0	0	0	3	0
ENSMUSG00000110398	0	0	0	0	0	0
ENSMUSG00000110391	0	0	2	0	0	0
ENSMUSG00000109691	0	0	0	0	0	0
ENSMUSG00000080987	0	2	0	0	0	0
ENSMUSG00000096309	1	0	0	0	0	0
ENSMUSG00000060416	0	0	0	0	0	0
ENSMUSG00000089257	0	0	0	0	0	0
ENSMUSG00000100942	0	0	0	0	0	0
ENSMUSG00000105651	3	4	10	7	3	7
ENSMUSG00000114779	0	0	0	0	0	0
ENSMUSG00000109330	0	0	0	0	0	0
ENSMUSG00000109331	3	0	0	0	0	3
ENSMUSG00000105654	0	0	0	0	0	0
ENSMUSG00000109334	0	0	0	0	0	0
ENSMUSG00000109335	0	0	0	0	0	0
ENSMUSG00000114770	0	0	0	0	0	0
ENSMUSG00000080321	0	0	0	0	0	0
ENSMUSG00000114772	0	0	0	0	0	0
ENSMUSG00000109339	0	0	0	0	0	0
ENSMUSG00000114774	0	0	0	0	0	0
ENSMUSG00000114775	0	0	0	0	0	0
ENSMUSG00000114776	0	0	0	0	0	0
ENSMUSG00000114777	0	0	0	0	0	0
ENSMUSG00000049608	6	0	0	2	0	0
ENSMUSG00000025001	143	115	136	47	97	159
ENSMUSG00000110641	0	0	0	0	0	0
ENSMUSG00000025003	0	0	0	0	0	0
ENSMUSG00000110643	0	0	0	0	0	0
ENSMUSG00000025004	0	0	0	0	0	0
ENSMUSG00000110646	0	0	0	0	0	0
ENSMUSG00000110647	30	21	33	11	14	6
ENSMUSG00000070330	0	0	0	0	0	0
ENSMUSG00000070332	0	0	0	0	0	0
ENSMUSG00000070335	0	0	0	0	0	0
ENSMUSG00000070334	0	0	0	0	0	0
ENSMUSG00000070337	5	7	11	12	0	15
ENSMUSG00000070336	7	15	22	18	28	36
ENSMUSG00000108060	0	0	0	0	0	0
ENSMUSG00000108063	0	0	0	0	0	0
ENSMUSG00000050424	3	3	4	0	0	0
ENSMUSG00000108065	0	5	0	0	0	0
ENSMUSG00000108066	0	0	0	0	0	0
ENSMUSG00000108069	0	0	0	0	0	0
ENSMUSG00000108068	0	0	0	0	0	0
ENSMUSG00000040836	286	176	372	191	214	250
ENSMUSG00000083030	0	0	0	0	0	0
ENSMUSG00000083031	0	0	0	0	0	0
ENSMUSG00000083034	0	2	0	0	0	2
ENSMUSG00000083039	0	0	0	0	0	0
ENSMUSG00000100941	0	0	0	0	0	0
ENSMUSG00000082939	0	0	0	0	0	0
ENSMUSG00000083185	0	0	0	0	0	0
ENSMUSG00000060412	0	0	0	0	0	0

ENSMUSG00000107460	0	0	0	0	0	0
ENSMUSG00000087144	0	1	0	0	0	1
ENSMUSG00000079034	0	0	0	0	0	0
ENSMUSG00000082397	0	0	0	0	0	0
ENSMUSG00000087140	0	0	0	0	0	0
ENSMUSG00000111117	0	0	0	0	0	0
ENSMUSG00000111116	49	47	70	15	12	24
ENSMUSG00000111115	0	0	0	0	0	0
ENSMUSG00000111114	0	0	0	0	0	0
ENSMUSG00000111112	0	0	0	0	0	0
ENSMUSG00000111111	0	0	0	0	0	0
ENSMUSG00000111110	0	0	0	0	0	0
ENSMUSG00000079112	0	0	0	0	0	0
ENSMUSG00000079113	0	0	0	0	0	0
ENSMUSG00000114298	0	0	0	0	0	0
ENSMUSG00000079116	0	0	0	0	0	0
ENSMUSG00000111119	0	0	0	0	0	0
ENSMUSG00000009350	0	0	0	0	0	0
ENSMUSG00000108349	0	3	0	0	0	0
ENSMUSG00000093342	0	2	1	1	0	3
ENSMUSG00000001670	0	0	0	0	0	0
ENSMUSG00000112486	0	0	0	0	0	0
ENSMUSG00000001672	0	0	6	0	0	0
ENSMUSG00000026063	0	0	0	0	0	0
ENSMUSG00000093340	0	0	0	0	0	0
ENSMUSG00000108556	0	0	0	0	0	0
ENSMUSG00000031786	0	0	3	0	0	0
ENSMUSG00000026068	24	39	50	13	9	19
ENSMUSG00000026069	73	84	79	63	47	54
ENSMUSG00000112481	0	0	5	6	0	0
ENSMUSG00000112483	18	29	30	21	39	43
ENSMUSG00000041872	0	0	0	0	0	0
ENSMUSG00000053198	79	62	100	55	47	66
ENSMUSG00000053194	0	0	0	0	0	0
ENSMUSG00000101256	0	0	0	0	0	0
ENSMUSG00000078240	6	2	4	6	4	10
ENSMUSG00000081308	0	0	1	3	3	0
ENSMUSG00000090087	0	0	0	0	0	3
ENSMUSG00000081300	0	0	0	0	0	0
ENSMUSG00000081304	2	4	4	9	10	10
ENSMUSG00000081305	2	3	1	0	0	2
ENSMUSG00000081306	0	0	0	0	0	0
ENSMUSG00000053441	14	8	6	8	10	28
ENSMUSG00000073012	0	0	0	0	0	0
ENSMUSG00000092821	0	0	0	0	0	0
ENSMUSG00000092822	0	0	0	0	0	0
ENSMUSG00000027335	42	54	64	53	47	56
ENSMUSG00000042993	0	6	6	0	3	4
ENSMUSG00000059069	0	0	0	0	0	0
ENSMUSG00000036526	9	12	36	0	0	0
ENSMUSG00000090558	0	0	0	0	3	0
ENSMUSG00000084875	0	2	0	0	0	0
ENSMUSG00000090552	0	0	0	0	0	0
ENSMUSG00000090550	0	0	0	0	0	0
ENSMUSG00000084871	0	0	0	0	0	0
ENSMUSG00000090556	0	0	0	1	0	0
ENSMUSG00000090555	0	0	0	0	0	0
ENSMUSG00000098709	0	0	0	0	0	0

ENSMUSG00000098707	1	0	0	0	0	0
ENSMUSG00000098705	1	0	0	0	0	0
ENSMUSG00000030657	355	300	482	122	145	158
ENSMUSG00000098703	0	0	0	0	0	0
ENSMUSG00000098700	0	0	0	0	0	0
ENSMUSG00000093878	2	1	3	0	0	2
ENSMUSG00000034219	0	0	0	0	0	0
ENSMUSG00000078935	0	0	0	0	0	0
ENSMUSG00000078934	0	0	0	0	0	0
ENSMUSG00000086653	0	0	0	0	0	0
ENSMUSG00000086652	0	0	4	0	0	0
ENSMUSG00000086650	0	0	0	0	0	0
ENSMUSG00000093871	0	0	0	0	0	0
ENSMUSG00000043119	3	3	0	0	0	0
ENSMUSG00000086659	0	0	0	0	0	0
ENSMUSG00000086658	2	0	0	0	0	0
ENSMUSG00000109965	8	0	0	5	4	2
ENSMUSG00000077223	0	0	0	0	0	0
ENSMUSG00000031297	8	11	10	4	0	0
ENSMUSG00000077224	0	0	0	0	0	0
ENSMUSG00000077228	0	0	0	0	0	0
ENSMUSG00000031298	91	82	90	14	34	28
ENSMUSG00000114124	0	0	0	0	0	0
ENSMUSG00000082641	0	0	0	1	0	0
ENSMUSG00000111324	4	0	16	0	4	4
ENSMUSG00000082020	0	0	0	0	0	0
ENSMUSG00000082025	0	0	0	0	0	0
ENSMUSG00000030650	29	42	27	45	43	52
ENSMUSG00000111016	0	0	0	0	0	0
ENSMUSG00000095091	0	0	0	0	0	0
ENSMUSG00000073907	7	7	2	6	2	2
ENSMUSG00000101302	0	0	0	0	0	0
ENSMUSG00000101301	0	0	0	0	0	0
ENSMUSG00000101300	0	0	0	0	0	0
ENSMUSG00000101307	2	3	0	0	0	0
ENSMUSG00000111322	0	0	0	0	0	0
ENSMUSG00000073901	0	0	0	0	0	0
ENSMUSG00000073900	3	0	0	0	2	0
ENSMUSG00000037922	122	180	216	127	138	168
ENSMUSG00000037924	0	0	0	0	0	0
ENSMUSG00000071506	0	0	0	0	0	0
ENSMUSG00000043800	0	0	0	0	0	0
ENSMUSG00000103239	0	0	0	0	0	0
ENSMUSG00000043807	6	6	4	12	7	9
ENSMUSG00000112737	0	0	0	0	0	0
ENSMUSG00000103234	7	7	7	6	7	6
ENSMUSG00000103237	2	0	0	0	0	0
ENSMUSG00000103231	0	0	0	0	0	0
ENSMUSG00000103230	6	21	14	12	15	14
ENSMUSG00000087015	0	0	0	0	0	0
ENSMUSG00000087010	0	0	0	0	0	0
ENSMUSG00000109084	0	0	0	0	0	0
ENSMUSG00000112733	0	0	0	0	0	0
ENSMUSG00000099547	0	0	1	0	0	0
ENSMUSG00000099546	0	0	0	0	0	0
ENSMUSG00000099545	2	1	1	1	0	1
ENSMUSG00000099544	0	0	0	0	0	0
ENSMUSG00000099542	0	0	0	0	0	0

ENSMUSG00000099541	0	0	0	0	0	0
ENSMUSG00000019214	47	34	53	29	27	47
ENSMUSG00000099549	0	0	0	0	0	0
ENSMUSG00000099548	0	0	0	0	0	0
ENSMUSG00000081050	0	0	0	0	0	0
ENSMUSG00000083775	0	0	0	0	0	0
ENSMUSG00000089316	0	0	0	0	0	0
ENSMUSG00000089314	0	0	0	0	0	0
ENSMUSG00000075081	0	0	0	2	0	0
ENSMUSG00000075084	0	0	0	0	0	0
ENSMUSG00000075086	0	0	0	0	0	0
ENSMUSG00000026882	0	0	0	0	0	0
ENSMUSG00000102548	0	5	0	0	0	2
ENSMUSG00000102549	9	35	12	55	47	41
ENSMUSG00000102547	0	0	0	0	0	0
ENSMUSG00000102541	0	0	0	0	0	0
ENSMUSG00000084097	0	0	0	0	0	0
ENSMUSG00000084096	2	0	0	0	0	0
ENSMUSG00000084095	0	0	0	0	0	0
ENSMUSG00000084094	8	0	3	7	3	6
ENSMUSG00000084092	0	0	0	0	0	0
ENSMUSG00000068888	0	0	0	0	0	0
ENSMUSG00000000263	0	0	2	0	0	0
ENSMUSG00000068887	0	0	0	0	0	0
ENSMUSG00000068885	0	0	0	0	0	0
ENSMUSG00000065872	0	0	0	0	0	0
ENSMUSG00000022056	0	0	0	1	0	0
ENSMUSG00000064471	0	0	0	0	0	0
ENSMUSG00000106305	4	0	0	0	0	0
ENSMUSG00000064477	0	0	0	0	0	0
ENSMUSG00000022055	0	11	4	0	5	7
ENSMUSG00000107960	0	0	0	0	0	0
ENSMUSG00000107073	0	0	0	0	0	0
ENSMUSG00000107072	0	0	0	0	0	0
ENSMUSG00000107070	0	0	0	0	0	0
ENSMUSG00000106308	0	0	0	0	0	0
ENSMUSG00000107074	0	0	0	0	0	0
ENSMUSG00000023159	0	0	0	0	0	0
ENSMUSG00000075330	0	0	0	0	0	0
ENSMUSG00000079652	0	0	0	0	0	0
ENSMUSG00000079655	0	0	0	0	0	0
ENSMUSG00000079654	63	82	123	37	50	78
ENSMUSG00000075334	2	2	0	9	2	0
ENSMUSG00000075335	0	0	0	0	0	0
ENSMUSG00000023153	16	27	6	42	23	36
ENSMUSG00000068776	0	0	0	0	0	0
ENSMUSG00000111250	0	0	0	7	0	0
ENSMUSG00000113286	0	0	0	0	0	0
ENSMUSG00000037033	0	0	0	0	0	0
ENSMUSG00000085329	0	0	0	0	0	0
ENSMUSG00000085324	0	0	0	0	0	0
ENSMUSG00000085325	0	0	0	0	0	0
ENSMUSG00000085323	0	0	0	0	0	0
ENSMUSG00000085320	0	0	0	0	0	0
ENSMUSG00000085321	0	0	0	0	0	0
ENSMUSG00000074061	0	0	0	0	0	0
ENSMUSG00000074060	0	0	0	0	0	0
ENSMUSG00000074063	70	46	53	54	86	115

ENSMUSG00000074062	0	0	0	0	0	0
ENSMUSG00000074067	22	32	21	34	38	36
ENSMUSG00000103090	0	0	0	0	0	0
ENSMUSG00000088541	0	0	0	0	0	0
ENSMUSG00000101891	0	0	0	0	0	0
ENSMUSG00000088542	0	0	0	0	0	0
ENSMUSG00000101897	0	5	0	2	0	0
ENSMUSG00000101896	0	0	0	1	0	0
ENSMUSG00000088547	0	0	0	0	0	0
ENSMUSG00000101894	0	0	0	0	0	0
ENSMUSG00000034764	0	0	0	0	0	2
ENSMUSG00000034762	6	7	2	0	0	3
ENSMUSG00000111253	0	0	0	0	0	0
ENSMUSG00000088898	0	0	0	0	0	0
ENSMUSG00000034768	12	23	6	32	31	28
ENSMUSG00000021799	32	38	31	38	73	71
ENSMUSG00000094888	1	0	0	0	0	0
ENSMUSG00000094885	0	0	0	0	0	0
ENSMUSG00000094884	2	0	0	0	0	0
ENSMUSG00000094887	0	0	0	0	0	0
ENSMUSG00000039717	0	0	2	0	0	0
ENSMUSG00000039710	0	0	0	0	0	0
ENSMUSG00000021790	0	0	0	0	0	0
ENSMUSG00000094883	0	0	0	0	0	0
ENSMUSG00000062591	51	47	47	15	24	30
ENSMUSG00000044292	0	0	0	0	0	0
ENSMUSG00000062598	0	0	0	0	0	0
ENSMUSG00000103792	0	0	0	0	0	0
ENSMUSG00000103790	0	0	0	0	0	0
ENSMUSG00000036892	0	0	0	0	0	0
ENSMUSG00000103797	0	0	0	0	0	0
ENSMUSG00000103794	0	0	1	0	0	0
ENSMUSG00000103795	0	0	0	0	0	0
ENSMUSG00000103798	2	0	0	0	0	0
ENSMUSG00000103799	0	0	0	0	0	0
ENSMUSG00000036899	0	0	0	0	0	0
ENSMUSG00000097282	3	3	13	5	5	12
ENSMUSG00000097283	0	0	0	0	3	0
ENSMUSG00000097280	0	0	0	0	3	0
ENSMUSG00000097281	0	0	0	0	0	0
ENSMUSG00000097286	0	0	0	0	0	0
ENSMUSG00000097287	70	67	66	54	72	95
ENSMUSG00000097288	0	0	0	0	0	0
ENSMUSG00000097289	4	6	8	0	0	0
ENSMUSG00000063646	5	8	13	4	9	5
ENSMUSG00000059366	0	2	0	0	0	0
ENSMUSG00000112980	37	22	46	26	23	22
ENSMUSG00000106679	0	0	0	0	0	0
ENSMUSG00000106673	2	5	5	6	6	3
ENSMUSG00000106670	0	0	0	0	0	0
ENSMUSG00000066031	0	0	0	0	0	0
ENSMUSG00000066030	0	0	0	0	0	0
ENSMUSG00000106674	0	0	0	0	0	0
ENSMUSG00000106675	1	0	0	1	0	1
ENSMUSG00000111257	0	0	0	0	0	0
ENSMUSG00000069910	47	27	35	3	13	21
ENSMUSG00000109530	0	0	0	0	0	0
ENSMUSG00000109533	0	0	0	0	0	0

ENSMUSG00000109534	0	0	0	0	0	0
ENSMUSG00000109535	0	0	0	0	0	0
ENSMUSG00000109537	0	0	0	0	0	0
ENSMUSG00000100854	0	0	0	0	0	0
ENSMUSG00000114577	0	0	0	0	0	0
ENSMUSG00000114572	0	0	1	1	0	2
ENSMUSG00000100851	0	0	0	0	0	0
ENSMUSG00000100852	0	0	0	0	0	0
ENSMUSG00000100853	0	0	0	0	0	0
ENSMUSG00000100858	0	0	0	0	0	0
ENSMUSG00000100859	0	0	0	0	0	0
ENSMUSG00000114578	0	0	0	0	0	0
ENSMUSG00000101139	0	0	0	0	0	0
ENSMUSG00000066697	0	2	0	0	0	0
ENSMUSG00000054526	0	0	0	0	0	0
ENSMUSG00000112801	0	2	0	0	0	0
ENSMUSG00000112802	2	9	6	8	12	4
ENSMUSG00000112803	0	0	0	0	0	0
ENSMUSG00000112805	0	6	10	0	0	0
ENSMUSG00000054520	287	285	420	97	131	147
ENSMUSG00000108668	2	0	8	4	0	3
ENSMUSG00000108667	0	0	0	0	0	0
ENSMUSG00000112809	0	0	0	0	0	0
ENSMUSG00000108665	0	0	0	0	0	0
ENSMUSG00000108663	0	0	0	0	0	0
ENSMUSG00000108662	0	0	0	0	0	0
ENSMUSG00000108661	0	0	0	2	0	0
ENSMUSG00000105329	0	0	0	0	0	0
ENSMUSG00000067341	0	4	6	4	0	0
ENSMUSG00000081589	0	0	0	1	0	0
ENSMUSG00000058188	0	0	0	0	0	0
ENSMUSG00000066964	0	0	0	0	0	0
ENSMUSG00000110396	0	0	0	0	0	0
ENSMUSG00000060699	0	0	0	0	0	0
ENSMUSG00000060691	0	5	0	0	0	0
ENSMUSG00000091936	0	0	0	0	0	0
ENSMUSG00000075228	0	0	0	0	0	0
ENSMUSG00000110395	0	0	0	0	0	0
ENSMUSG00000091933	0	0	0	0	0	0
ENSMUSG00000091930	0	0	0	0	0	0
ENSMUSG00000110392	0	0	0	0	0	0
ENSMUSG00000083115	0	0	0	0	0	0
ENSMUSG00000091938	0	0	9	10	8	0
ENSMUSG00000110393	26	38	15	17	21	25
ENSMUSG00000096629	5	4	4	0	2	13
ENSMUSG00000083114	0	0	0	0	0	0
ENSMUSG00000110390	4	0	0	3	3	5
ENSMUSG00000083113	0	0	0	0	0	0
ENSMUSG00000023931	20	33	12	4	12	0
ENSMUSG00000023930	0	0	0	0	0	0
ENSMUSG00000096627	0	0	0	0	0	0
ENSMUSG00000096626	0	0	0	0	0	0
ENSMUSG00000096624	0	0	0	0	1	0
ENSMUSG00000104680	0	0	0	0	0	0
ENSMUSG00000083110	0	0	0	0	0	0
ENSMUSG00000093554	0	0	0	0	0	0
ENSMUSG00000056961	0	0	0	0	0	0
ENSMUSG00000056966	5	6	3	13	8	12

ENSMUSG00000109974	0	0	0	0	0	0
ENSMUSG00000111054	0	0	0	0	0	0
ENSMUSG00000111055	49	36	49	58	61	60
ENSMUSG00000111052	5	5	0	0	0	0
ENSMUSG00000010461	242	102	136	21	33	43
ENSMUSG00000100613	0	0	0	0	0	0
ENSMUSG00000110989	2	1	0	2	1	1
ENSMUSG00000110988	0	0	0	0	0	0
ENSMUSG00000110983	0	0	0	0	0	0
ENSMUSG00000110982	0	0	2	0	2	2
ENSMUSG00000110981	0	0	2	0	0	2
ENSMUSG00000110987	0	0	0	0	0	0
ENSMUSG00000110986	0	0	3	0	3	0
ENSMUSG00000110985	0	0	0	0	0	0
ENSMUSG00000024184	0	5	8	2	0	7
ENSMUSG00000064860	0	0	0	0	0	0
ENSMUSG00000108932	0	0	0	0	0	0
ENSMUSG00000091268	0	0	0	0	0	0
ENSMUSG00000045775	25	17	20	22	13	14
ENSMUSG00000018259	0	0	0	0	0	0
ENSMUSG00000108936	0	0	0	0	0	0
ENSMUSG00000026885	96	94	94	90	103	134
ENSMUSG00000081146	0	0	0	0	0	0
ENSMUSG00000081147	0	0	0	0	0	0
ENSMUSG00000081144	0	0	0	0	0	0
ENSMUSG00000081145	0	0	0	0	0	0
ENSMUSG00000081141	0	0	0	0	0	0
ENSMUSG00000108939	0	0	0	0	0	0
ENSMUSG00000081148	0	0	0	0	0	0
ENSMUSG00000027134	75	45	91	40	46	64
ENSMUSG00000056380	0	0	0	0	0	0
ENSMUSG00000090718	0	0	0	0	0	0
ENSMUSG00000017165	0	0	0	0	0	0
ENSMUSG00000109333	0	0	0	0	0	0
ENSMUSG00000090710	0	0	0	0	0	0
ENSMUSG00000090713	0	0	0	0	0	0
ENSMUSG00000090715	0	0	0	0	0	0
ENSMUSG00000090714	0	0	0	0	0	0
ENSMUSG00000105652	0	0	0	0	0	0
ENSMUSG00000008496	82	63	111	22	50	32
ENSMUSG00000042200	0	11	10	0	5	0
ENSMUSG00000094416	0	0	0	0	0	0
ENSMUSG00000094415	0	0	0	0	0	0
ENSMUSG00000094412	0	0	0	0	0	0
ENSMUSG00000114771	11	21	21	12	17	10
ENSMUSG00000056032	0	0	0	0	2	0
ENSMUSG00000094370	0	0	14	0	0	0
ENSMUSG00000094419	0	0	0	0	0	0
ENSMUSG00000086496	0	0	0	0	0	0
ENSMUSG00000105871	2	0	4	0	0	3
ENSMUSG00000064743	0	0	0	0	0	0
ENSMUSG00000028370	261	151	214	121	78	111
ENSMUSG00000054994	0	0	0	0	0	0
ENSMUSG00000048067	0	0	0	0	0	0
ENSMUSG00000077021	0	0	0	0	0	0
ENSMUSG00000077022	0	0	0	0	0	0
ENSMUSG00000077025	1	0	0	0	0	0
ENSMUSG00000064746	0	0	0	0	0	0

ENSMUSG00000031480	382	419	467	298	265	399
ENSMUSG00000055602	4	0	9	0	12	4
ENSMUSG00000104057	4	2	2	2	0	0
ENSMUSG00000046456	38	48	42	12	14	14
ENSMUSG00000083312	0	0	0	0	0	0
ENSMUSG00000046450	0	2	0	0	0	0
ENSMUSG00000110642	0	0	0	0	0	0
ENSMUSG00000025002	0	0	0	0	0	0
ENSMUSG00000092715	0	1	0	0	0	0
ENSMUSG00000092714	0	0	0	0	0	0
ENSMUSG00000077792	0	0	0	0	0	0
ENSMUSG00000077793	0	0	0	0	0	0
ENSMUSG00000092719	0	0	0	0	0	1
ENSMUSG00000092718	0	0	0	0	0	0
ENSMUSG00000077796	0	0	0	0	0	0
ENSMUSG00000077794	0	0	0	0	0	0
ENSMUSG00000110645	0	0	0	0	0	0
ENSMUSG00000049334	0	0	0	0	0	0
ENSMUSG00000049336	0	0	0	3	6	7
ENSMUSG00000029423	0	0	0	0	0	0
ENSMUSG00000077178	0	0	0	0	0	0
ENSMUSG00000029420	0	0	0	0	3	0
ENSMUSG00000006218	4	6	4	4	6	0
ENSMUSG00000113369	0	0	2	0	0	0
ENSMUSG00000113366	0	0	0	0	0	0
ENSMUSG00000113367	0	0	0	0	0	0
ENSMUSG00000113364	0	0	0	0	0	0
ENSMUSG00000113365	0	0	0	0	0	0
ENSMUSG00000113362	0	0	0	0	0	0
ENSMUSG00000112147	0	0	0	0	0	0
ENSMUSG00000113360	0	0	0	0	0	0
ENSMUSG00000113361	74	51	69	67	61	77
ENSMUSG00000071749	0	0	0	0	0	0
ENSMUSG00000109276	1	0	0	0	0	0
ENSMUSG00000109275	3	3	0	0	0	0
ENSMUSG00000109273	0	0	0	0	0	0
ENSMUSG00000109272	1	0	0	0	0	0
ENSMUSG00000109271	0	0	0	0	0	0
ENSMUSG00000109278	0	0	0	0	0	0
ENSMUSG00000106531	0	0	0	0	2	0
ENSMUSG00000106534	0	0	0	0	0	0
ENSMUSG00000040838	10	2	7	2	4	4
ENSMUSG00000050425	4	0	0	0	4	0
ENSMUSG00000108062	0	0	0	0	0	0
ENSMUSG00000047324	0	0	0	0	0	0
ENSMUSG00000050423	3	14	11	24	2	18
ENSMUSG00000063529	0	5	0	0	0	0
ENSMUSG00000082551	0	0	0	3	2	0
ENSMUSG00000082552	0	0	0	0	0	0
ENSMUSG00000082553	0	0	0	0	0	0
ENSMUSG00000082555	0	0	0	0	2	0
ENSMUSG00000082556	0	0	0	0	3	0
ENSMUSG00000082558	0	0	0	0	0	0
ENSMUSG00000093474	0	0	0	0	0	0
ENSMUSG00000085937	0	0	0	0	0	0
ENSMUSG00000085934	0	0	0	0	0	0
ENSMUSG00000084259	0	0	0	0	1	0
ENSMUSG00000080391	0	0	0	0	0	0

ENSMUSG00000083206	0	0	3	0	9	2
ENSMUSG00000084257	0	0	0	0	0	0
ENSMUSG00000083204	0	0	0	0	0	0
ENSMUSG00000084251	0	0	0	0	0	0
ENSMUSG00000083202	2	0	0	0	0	0
ENSMUSG00000084253	0	0	0	2	2	2
ENSMUSG00000083200	0	0	0	0	0	0
ENSMUSG00000041248	0	0	0	0	0	0
ENSMUSG00000085931	0	0	0	0	0	0
ENSMUSG00000025215	10	10	6	0	0	0
ENSMUSG00000025216	0	0	0	0	0	0
ENSMUSG00000080958	0	0	0	0	0	0
ENSMUSG00000080959	0	0	0	0	0	0
ENSMUSG00000080952	0	0	0	1	0	0
ENSMUSG00000080953	0	0	0	0	0	3
ENSMUSG00000080954	0	0	0	0	0	0
ENSMUSG00000080955	0	0	0	0	0	0
ENSMUSG00000099150	0	0	0	0	0	0
ENSMUSG00000099152	0	0	0	0	0	0
ENSMUSG00000099153	1	0	1	0	2	2
ENSMUSG00000099154	0	0	0	0	0	0
ENSMUSG00000099158	2	0	4	1	2	4
ENSMUSG00000099159	0	0	0	0	0	0
ENSMUSG00000085938	0	6	0	0	0	0
ENSMUSG00000068574	6	5	5	11	7	10
ENSMUSG00000044149	201	190	182	123	200	167
ENSMUSG00000080649	1	0	0	0	0	0
ENSMUSG00000085129	13	16	10	8	16	9
ENSMUSG00000006711	0	4	2	0	4	0
ENSMUSG00000085124	0	0	0	0	0	0
ENSMUSG00000085126	0	0	0	0	0	0
ENSMUSG00000085127	8	3	0	4	10	0
ENSMUSG00000085120	0	0	0	0	0	0
ENSMUSG00000085121	0	0	0	0	0	0
ENSMUSG00000085122	0	0	0	0	0	0
ENSMUSG00000085123	0	0	0	0	0	0
ENSMUSG00000074665	0	0	0	0	0	0
ENSMUSG00000074664	0	0	0	0	0	0
ENSMUSG00000079324	0	0	0	0	0	0
ENSMUSG00000041930	11	0	17	4	6	6
ENSMUSG00000060070	0	0	0	0	0	0
ENSMUSG00000045236	0	0	0	0	0	0
ENSMUSG00000045232	0	2	0	0	0	3
ENSMUSG00000045238	3	0	0	0	4	10
ENSMUSG00000036492	16	33	20	22	16	32
ENSMUSG00000047496	330	266	342	124	213	203
ENSMUSG00000103802	0	0	0	0	0	0
ENSMUSG00000103801	0	0	0	0	0	0
ENSMUSG00000103807	0	1	0	3	0	0
ENSMUSG00000019785	2	0	0	0	0	2
ENSMUSG00000047751	0	0	0	0	0	0
ENSMUSG00000089922	0	0	0	0	0	0
ENSMUSG00000089924	0	0	0	0	0	0
ENSMUSG00000089926	0	0	0	0	0	0
ENSMUSG00000089927	0	0	0	0	0	0
ENSMUSG00000048501	0	0	0	0	0	0
ENSMUSG00000048500	0	0	0	0	0	0
ENSMUSG00000048503	288	290	240	203	272	385

ENSMUSG00000037737	0	0	0	0	0	0
ENSMUSG00000031488	93	55	94	32	32	35
ENSMUSG00000076983	0	0	0	0	0	0
ENSMUSG00000037738	4	0	0	0	4	0
ENSMUSG00000067441	0	0	0	0	0	0
ENSMUSG00000031738	0	0	0	0	0	0
ENSMUSG00000004821	0	0	0	0	0	0
ENSMUSG00000082772	0	0	0	0	0	0
ENSMUSG00000043308	0	0	0	0	0	0
ENSMUSG00000087199	0	0	0	0	0	0
ENSMUSG00000087192	7	13	0	3	11	9
ENSMUSG00000087196	0	0	3	0	0	0
ENSMUSG00000087195	0	0	0	0	0	0
ENSMUSG00000085979	0	0	0	0	3	0
ENSMUSG00000085978	0	0	0	0	0	0
ENSMUSG00000113232	0	0	0	0	0	0
ENSMUSG00000094952	2	1	2	0	0	1
ENSMUSG00000085973	0	0	0	0	0	0
ENSMUSG00000085972	0	0	0	0	0	0
ENSMUSG00000037884	0	0	0	0	0	0
ENSMUSG00000085970	0	0	0	0	0	0
ENSMUSG00000085976	0	5	6	0	6	8
ENSMUSG00000094950	0	0	0	0	0	0
ENSMUSG00000081918	0	0	0	0	0	0
ENSMUSG00000079114	0	0	0	0	0	0
ENSMUSG00000015787	0	0	0	0	0	0
ENSMUSG00000082618	0	0	0	0	0	0
ENSMUSG00000014782	4	0	4	4	4	7
ENSMUSG00000040650	0	0	0	0	0	0
ENSMUSG00000101516	0	0	0	0	0	0
ENSMUSG00000101517	5	6	4	13	10	10
ENSMUSG00000040657	0	0	0	0	0	0
ENSMUSG00000101515	0	0	0	0	0	0
ENSMUSG00000021961	0	0	0	0	0	0
ENSMUSG00000100641	0	0	0	0	0	0
ENSMUSG00000100640	0	0	0	0	0	0
ENSMUSG00000100643	0	0	0	0	0	0
ENSMUSG00000100645	0	0	0	0	0	0
ENSMUSG00000100644	0	0	0	0	0	0
ENSMUSG00000100647	0	0	0	0	0	0
ENSMUSG00000100646	0	0	0	0	0	0
ENSMUSG00000100649	0	0	0	0	0	0
ENSMUSG00000100648	2	0	0	0	2	2
ENSMUSG00000059910	0	0	0	0	0	0
ENSMUSG00000107918	0	0	0	0	0	0
ENSMUSG00000107914	0	0	3	0	0	0
ENSMUSG00000107916	0	0	0	0	0	0
ENSMUSG00000107917	0	2	0	0	0	0
ENSMUSG00000084754	0	0	0	0	0	0
ENSMUSG00000084757	0	0	0	0	0	0
ENSMUSG00000084750	0	0	0	0	0	0
ENSMUSG00000089107	0	0	0	0	0	0
ENSMUSG00000084752	0	0	0	0	0	0
ENSMUSG00000084753	0	0	0	0	0	0
ENSMUSG00000089109	0	0	0	0	0	0
ENSMUSG00000084758	5	0	1	0	6	4
ENSMUSG00000075270	81	70	32	175	189	191
ENSMUSG00000021359	0	0	0	0	2	0

ENSMUSG00000022431	8	12	0	8	4	2
ENSMUSG00000022435	0	0	0	0	0	0
ENSMUSG00000032113	54	62	74	25	31	35
ENSMUSG00000032110	5	0	0	0	0	0
ENSMUSG00000032118	93	65	71	60	95	94
ENSMUSG00000020364	168	157	114	57	66	187
ENSMUSG00000088722	0	0	0	0	0	0
ENSMUSG00000113111	0	0	0	0	0	0
ENSMUSG00000113110	0	0	0	0	1	0
ENSMUSG00000060811	3	0	2	0	0	2
ENSMUSG00000102739	0	0	0	0	0	0
ENSMUSG00000102738	0	0	0	0	0	0
ENSMUSG00000112218	16	0	11	34	28	0
ENSMUSG00000112215	0	0	0	0	0	0
ENSMUSG00000112217	0	0	0	0	0	0
ENSMUSG00000112216	6	9	7	7	9	6
ENSMUSG00000112211	0	0	0	0	0	0
ENSMUSG00000112213	3	6	3	5	5	1
ENSMUSG00000112212	0	0	0	0	0	0
ENSMUSG00000036912	2	0	4	0	0	4
ENSMUSG00000036916	545	538	590	376	433	563
ENSMUSG00000036915	0	0	0	0	0	0
ENSMUSG00000028176	0	3	0	3	0	0
ENSMUSG00000028177	5	5	10	11	21	16
ENSMUSG00000028174	0	0	0	0	0	0
ENSMUSG00000028175	33	46	12	3	5	22
ENSMUSG00000028172	0	0	0	0	3	0
ENSMUSG00000028179	72	112	37	137	136	157
ENSMUSG00000039691	4	0	4	6	16	4
ENSMUSG00000105422	0	0	0	0	0	3
ENSMUSG00000105423	0	0	0	0	0	0
ENSMUSG00000026065	0	6	4	9	7	14
ENSMUSG00000105426	0	0	0	0	0	0
ENSMUSG00000105424	0	0	0	0	2	0
ENSMUSG00000105425	2	2	0	0	0	1
ENSMUSG00000024295	0	1	1	2	1	2
ENSMUSG00000082275	7	0	0	0	0	3
ENSMUSG00000103023	0	0	0	0	0	0
ENSMUSG00000113169	0	0	0	0	0	0
ENSMUSG00000113164	0	0	0	0	0	0
ENSMUSG00000113166	0	0	0	0	0	0
ENSMUSG00000113167	0	0	0	2	0	0
ENSMUSG00000113160	0	0	0	0	0	0
ENSMUSG00000113161	9	12	9	7	3	9
ENSMUSG00000113162	0	0	0	0	0	0
ENSMUSG00000113163	0	0	0	0	0	0
ENSMUSG00000069038	3	2	6	3	4	7
ENSMUSG00000103026	6	0	2	2	4	4
ENSMUSG00000069036	0	0	0	0	0	0
ENSMUSG00000103027	0	0	0	0	0	0
ENSMUSG00000039963	0	0	5	0	0	0
ENSMUSG00000039962	0	3	0	3	0	0
ENSMUSG00000021187	118	70	75	16	8	16
ENSMUSG00000050097	0	2	0	0	0	0
ENSMUSG00000024128	0	0	0	0	0	0
ENSMUSG00000008813	0	0	0	0	0	0
ENSMUSG00000106738	0	0	2	0	0	2
ENSMUSG00000106735	33	27	30	32	23	38

ENSMUSG00000106733	0	0	0	0	0	0
ENSMUSG00000106730	0	0	0	0	0	0
ENSMUSG00000051062	0	0	0	0	3	0
ENSMUSG00000059280	0	0	0	0	0	0
ENSMUSG00000059136	1	1	3	2	3	1
ENSMUSG00000059134	3	3	1	0	1	3
ENSMUSG00000051839	19	22	25	8	5	16
ENSMUSG00000055884	331	274	382	239	281	319
ENSMUSG00000051706	0	0	0	0	0	0
ENSMUSG00000055882	5	1	4	6	12	8
ENSMUSG00000105289	24	20	29	22	23	31
ENSMUSG00000109706	0	0	0	0	0	0
ENSMUSG00000109705	1	0	0	0	0	0
ENSMUSG00000109704	0	1	0	0	0	0
ENSMUSG00000109703	1	0	0	1	0	0
ENSMUSG00000109702	0	0	0	0	0	0
ENSMUSG00000105280	0	0	0	0	0	0
ENSMUSG00000105281	14	9	5	20	21	15
ENSMUSG00000089487	0	0	0	0	0	0
ENSMUSG00000106888	19	6	14	14	4	10
ENSMUSG00000089485	0	0	0	0	0	0
ENSMUSG00000089484	0	0	0	0	0	0
ENSMUSG00000089481	0	0	0	0	0	0
ENSMUSG00000089480	0	0	0	0	0	0
ENSMUSG00000106880	0	0	0	0	0	0
ENSMUSG00000106883	3	0	0	0	2	0
ENSMUSG00000106884	0	0	0	0	0	0
ENSMUSG00000106887	2	0	0	0	2	0
ENSMUSG00000089488	0	0	0	0	0	0
ENSMUSG00000104779	2	2	0	2	0	3
ENSMUSG00000104778	0	0	0	0	0	0
ENSMUSG00000108258	8	4	12	0	0	0
ENSMUSG00000108259	0	0	0	0	0	0
ENSMUSG00000022986	2	0	0	0	0	0
ENSMUSG00000108252	0	0	0	0	0	0
ENSMUSG00000104770	0	0	0	0	0	0
ENSMUSG00000108250	0	0	0	0	0	0
ENSMUSG00000104772	0	0	0	0	0	0
ENSMUSG00000104775	0	0	0	0	0	0
ENSMUSG00000108257	3	4	6	5	3	4
ENSMUSG00000108254	0	0	0	0	0	0
ENSMUSG00000104482	0	0	1	0	0	0
ENSMUSG00000104481	2	4	2	2	4	4
ENSMUSG00000104480	0	0	0	0	0	0
ENSMUSG00000104487	0	0	0	0	0	0
ENSMUSG00000104489	0	0	0	0	0	0
ENSMUSG00000114304	0	0	0	0	0	0
ENSMUSG00000114307	0	0	0	0	0	0
ENSMUSG00000096295	0	0	0	0	0	0
ENSMUSG00000114301	1	3	2	1	2	0
ENSMUSG00000096293	0	0	0	0	0	0
ENSMUSG00000114303	0	0	0	0	0	0
ENSMUSG00000096291	0	0	0	0	0	0
ENSMUSG00000114308	0	0	0	0	0	0
ENSMUSG00000096298	0	0	0	0	0	0
ENSMUSG00000096299	10	13	12	4	14	9
ENSMUSG00000097749	0	0	0	0	0	0
ENSMUSG00000097742	4	0	0	0	0	2

ENSMUSG00000097744	0	0	0	0	0	0
ENSMUSG00000050074	0	0	0	0	2	0
ENSMUSG00000054757	0	0	0	0	0	0
ENSMUSG00000054753	0	0	0	0	0	0
ENSMUSG00000054759	2	1	0	0	3	4
ENSMUSG00000091745	7	4	4	0	6	6
ENSMUSG00000091744	0	0	0	0	0	0
ENSMUSG00000091741	0	0	0	0	0	0
ENSMUSG00000091740	0	0	0	0	0	0
ENSMUSG00000091743	1	5	0	0	0	0
ENSMUSG00000103410	0	0	0	0	0	0
ENSMUSG00000091749	2	0	0	0	0	0
ENSMUSG00000025983	4	15	7	13	12	8
ENSMUSG00000058665	0	0	0	0	0	0
ENSMUSG00000058669	0	0	0	0	0	0
ENSMUSG00000009292	66	58	175	29	39	50
ENSMUSG00000044309	4	0	6	0	0	0
ENSMUSG00000110534	7	9	12	0	12	4
ENSMUSG00000110535	0	0	0	0	0	0
ENSMUSG00000110533	0	0	0	2	0	0
ENSMUSG00000027584	5	5	6	0	2	2
ENSMUSG00000026182	0	3	3	0	0	5
ENSMUSG00000038599	0	0	3	0	0	0
ENSMUSG00000080427	0	0	0	0	0	0
ENSMUSG00000080428	0	0	0	0	0	0
ENSMUSG00000095248	2	0	0	0	0	2
ENSMUSG00000095245	1	2	2	1	0	2
ENSMUSG00000095246	3	1	4	0	1	2
ENSMUSG00000095247	0	0	7	0	7	10
ENSMUSG00000095240	0	0	0	0	0	0
ENSMUSG00000095241	0	0	0	0	0	0
ENSMUSG00000095242	0	0	0	0	0	0
ENSMUSG00000095243	0	0	0	0	0	0
ENSMUSG00000113945	0	0	0	0	0	0
ENSMUSG00000113940	19	27	28	18	13	33
ENSMUSG00000113941	10	3	7	6	4	6
ENSMUSG00000047544	0	0	0	0	0	0
ENSMUSG00000047545	0	0	0	0	0	0
ENSMUSG00000094596	1	1	0	0	0	0
ENSMUSG00000094594	5	2	2	2	0	0
ENSMUSG00000114468	0	0	0	0	0	0
ENSMUSG00000086860	0	0	0	0	0	0
ENSMUSG00000086861	0	0	0	0	0	0
ENSMUSG00000086862	4	0	0	0	0	0
ENSMUSG00000086868	0	0	0	0	0	0
ENSMUSG00000081486	0	0	0	0	0	0
ENSMUSG00000048745	0	0	0	0	0	0
ENSMUSG00000081484	0	0	0	0	0	0
ENSMUSG00000081483	0	0	0	0	0	0
ENSMUSG00000081481	0	0	0	0	0	0
ENSMUSG00000077619	0	0	0	0	0	0
ENSMUSG00000077618	0	0	0	0	0	0
ENSMUSG00000077616	0	1	0	0	0	0
ENSMUSG00000077615	0	0	0	0	1	0
ENSMUSG00000077612	0	0	0	0	0	0
ENSMUSG00000094625	0	0	0	0	0	0
ENSMUSG00000094624	0	0	0	0	0	0
ENSMUSG00000094626	0	0	5	0	0	5

ENSMUSG00000094621	1	0	1	0	0	0
ENSMUSG00000094620	0	0	0	0	0	0
ENSMUSG00000094628	26	26	29	20	18	14
ENSMUSG00000013083	0	0	0	0	0	0
ENSMUSG00000044899	1	0	0	0	0	0
ENSMUSG00000090900	1	0	1	2	1	0
ENSMUSG00000044897	0	0	0	0	0	0
ENSMUSG00000091185	0	0	0	0	2	0
ENSMUSG00000090907	0	0	0	0	0	0
ENSMUSG00000048299	2	2	0	0	0	0
ENSMUSG00000105178	0	0	0	0	0	0
ENSMUSG00000048292	9	5	5	0	4	0
ENSMUSG00000048294	0	0	0	0	0	0
ENSMUSG00000092502	0	0	0	0	0	0
ENSMUSG00000092503	0	0	0	0	0	0
ENSMUSG00000099772	0	0	0	0	0	0
ENSMUSG00000099774	0	0	0	0	0	0
ENSMUSG00000099776	0	0	0	0	0	0
ENSMUSG00000099777	0	0	0	0	0	0
ENSMUSG00000099778	0	0	0	0	0	0
ENSMUSG00000099779	0	5	5	0	0	5
ENSMUSG00000092508	0	0	0	0	0	0
ENSMUSG00000092509	0	0	0	0	0	0
ENSMUSG00000086284	0	0	0	0	0	0
ENSMUSG00000046793	10	5	0	0	0	7
ENSMUSG00000086286	0	0	0	0	0	0
ENSMUSG00000086283	19	13	44	0	0	6
ENSMUSG00000007646	12	51	27	30	33	59
ENSMUSG00000097798	0	0	0	0	0	0
ENSMUSG00000086138	0	0	0	0	0	0
ENSMUSG00000086139	3	2	7	2	0	6
ENSMUSG00000086136	0	0	0	0	0	0
ENSMUSG00000086137	0	0	0	0	0	0
ENSMUSG00000043670	0	0	0	0	0	0
ENSMUSG00000043671	144	105	170	90	78	93
ENSMUSG00000086132	0	0	0	0	0	0
ENSMUSG00000105174	2	0	0	0	0	0
ENSMUSG00000098829	0	0	0	0	0	0
ENSMUSG00000098828	0	0	0	0	0	0
ENSMUSG00000076251	0	0	0	0	0	0
ENSMUSG00000000037	53	29	15	16	15	50
ENSMUSG00000076253	0	0	0	0	0	0
ENSMUSG00000098823	1	0	0	0	0	0
ENSMUSG00000098821	0	0	0	0	0	0
ENSMUSG00000098820	0	0	0	0	0	0
ENSMUSG00000070828	0	0	0	0	0	0
ENSMUSG00000087425	0	0	0	0	0	0
ENSMUSG00000070821	0	0	0	0	0	0
ENSMUSG00000070820	0	0	0	0	0	0
ENSMUSG00000070823	0	0	2	0	0	0
ENSMUSG00000087428	0	0	0	0	0	0
ENSMUSG00000041138	0	0	0	0	0	3
ENSMUSG00000049168	0	0	0	0	0	0
ENSMUSG00000037577	100	86	143	45	72	86
ENSMUSG00000083502	4	3	2	4	5	4
ENSMUSG00000083505	4	3	5	3	4	8
ENSMUSG00000083507	0	0	0	0	0	0
ENSMUSG00000049160	3	0	0	0	0	4

ENSMUSG00000083509	0	0	0	0	0	0
ENSMUSG00000037578	0	0	0	0	0	0
ENSMUSG00000037579	7	0	0	0	0	2
ENSMUSG00000026841	0	5	2	0	2	6
ENSMUSG00000079423	0	0	0	0	0	0
ENSMUSG00000079421	0	0	0	0	0	0
ENSMUSG00000079424	0	0	0	0	0	0
ENSMUSG00000050917	0	0	0	0	0	0
ENSMUSG00000110847	0	0	0	0	0	0
ENSMUSG00000005649	0	0	0	0	0	0
ENSMUSG00000071158	0	0	0	0	0	0
ENSMUSG00000084193	3	4	1	3	3	4
ENSMUSG00000084191	0	0	0	0	0	0
ENSMUSG00000084196	0	0	0	0	0	0
ENSMUSG00000084197	0	0	0	0	0	0
ENSMUSG00000052865	0	0	0	0	0	0
ENSMUSG00000084198	2	0	0	0	0	0
ENSMUSG00000084199	0	0	1	4	0	0
ENSMUSG00000000690	0	2	2	0	0	0
ENSMUSG00000084873	0	0	0	0	0	0
ENSMUSG00000099370	9	7	3	6	6	5
ENSMUSG00000080852	0	0	0	0	0	0
ENSMUSG00000080850	54	66	52	105	113	103
ENSMUSG00000105172	0	0	0	0	0	1
ENSMUSG00000110848	0	0	0	0	0	0
ENSMUSG00000105236	0	0	0	0	0	0
ENSMUSG00000104820	0	0	0	0	0	0
ENSMUSG00000105173	5	8	3	5	11	9
ENSMUSG00000075707	14	6	39	2	0	0
ENSMUSG00000104824	7	0	0	7	0	0
ENSMUSG00000104826	0	0	0	0	0	0
ENSMUSG00000074259	0	0	5	0	0	0
ENSMUSG00000034532	5	22	6	6	9	8
ENSMUSG00000074252	13	10	9	11	5	6
ENSMUSG00000078773	11	30	22	5	13	21
ENSMUSG00000074254	0	0	0	0	0	0
ENSMUSG00000032053	23	31	19	8	5	7
ENSMUSG00000020411	0	0	6	3	6	4
ENSMUSG00000081812	0	0	2	1	2	2
ENSMUSG00000081814	0	0	0	0	0	0
ENSMUSG00000065151	0	0	0	0	0	0
ENSMUSG00000082096	0	0	0	0	0	0
ENSMUSG00000065155	0	0	0	0	0	0
ENSMUSG00000073705	24	21	31	20	10	28
ENSMUSG00000006527	773	890	822	608	788	854
ENSMUSG00000006522	13	16	46	0	0	2
ENSMUSG00000006529	0	0	0	0	0	0
ENSMUSG00000075605	0	0	0	0	0	0
ENSMUSG00000079180	0	0	0	0	0	0
ENSMUSG00000079183	0	0	0	0	1	0
ENSMUSG00000098016	0	0	0	2	2	0
ENSMUSG00000098017	0	0	0	0	0	0
ENSMUSG00000098015	0	0	0	0	0	0
ENSMUSG00000098012	0	0	0	0	0	0
ENSMUSG00000098013	1	0	0	0	0	0
ENSMUSG00000098010	0	0	0	0	0	0
ENSMUSG00000077938	0	0	0	0	0	0
ENSMUSG00000088577	0	0	0	0	0	0

ENSMUSG00000063216	0	0	0	0	0	0
ENSMUSG00000104188	0	0	0	0	3	0
ENSMUSG00000111037	0	0	0	0	2	0
ENSMUSG00000086655	0	0	0	0	0	0
ENSMUSG00000099601	0	0	0	0	0	0
ENSMUSG00000108407	0	0	0	0	0	0
ENSMUSG00000035910	0	0	0	0	0	0
ENSMUSG00000035916	0	0	0	0	0	0
ENSMUSG00000035031	0	2	0	0	0	0
ENSMUSG00000035914	188	149	246	112	126	100
ENSMUSG00000104187	8	0	6	2	5	8
ENSMUSG00000108403	0	0	0	0	0	0
ENSMUSG00000078938	0	0	0	0	0	0
ENSMUSG00000084942	0	0	0	0	0	0
ENSMUSG00000084945	0	0	0	0	0	0
ENSMUSG00000084944	0	0	0	0	0	0
ENSMUSG00000084947	4	0	0	12	4	0
ENSMUSG00000084949	0	0	2	0	0	0
ENSMUSG00000038891	0	0	0	0	0	0
ENSMUSG00000073380	0	0	0	5	10	12
ENSMUSG00000110095	0	0	0	0	0	0
ENSMUSG00000030368	0	0	0	0	0	0
ENSMUSG00000030366	0	0	0	0	0	0
ENSMUSG00000030364	0	0	0	0	0	0
ENSMUSG00000030363	0	0	0	0	0	0
ENSMUSG00000110090	0	0	0	0	0	0
ENSMUSG00000030651	0	0	0	0	0	0
ENSMUSG00000088796	0	0	0	0	0	0
ENSMUSG00000101495	0	0	0	0	0	0
ENSMUSG00000088792	0	0	0	0	0	0
ENSMUSG00000101721	0	0	0	0	0	0
ENSMUSG00000101720	0	0	0	0	0	0
ENSMUSG00000101723	0	0	0	0	0	0
ENSMUSG00000101722	5	0	0	0	0	0
ENSMUSG00000101725	0	0	0	0	0	0
ENSMUSG00000101727	0	0	0	0	0	0
ENSMUSG00000101726	0	0	0	0	0	0
ENSMUSG00000101728	0	0	0	0	0	0
ENSMUSG00000061845	0	0	0	0	0	0
ENSMUSG00000061847	0	0	0	0	0	0
ENSMUSG00000061843	0	0	0	0	0	0
ENSMUSG00000088463	0	0	0	0	0	0
ENSMUSG00000088460	0	0	0	0	0	0
ENSMUSG00000095407	7	10	6	0	0	0
ENSMUSG00000058270	3	0	0	2	0	0
ENSMUSG00000088469	0	0	0	0	0	0
ENSMUSG00000058275	0	0	0	0	2	0
ENSMUSG00000107891	0	6	0	1	2	0
ENSMUSG00000107890	0	0	0	0	0	0
ENSMUSG00000107893	1	0	2	0	0	0
ENSMUSG00000059891	0	5	0	0	0	0
ENSMUSG00000107894	21	13	24	18	14	10
ENSMUSG00000107898	0	0	0	0	0	0
ENSMUSG00000059898	0	0	0	0	0	0
ENSMUSG00000023391	0	0	0	0	0	0
ENSMUSG00000029379	0	0	0	0	0	0
ENSMUSG00000107769	0	3	0	0	0	0
ENSMUSG00000107762	0	0	0	0	0	0

ENSMUSG00000107766	6	6	0	5	0	0
ENSMUSG00000107764	0	0	0	0	0	0
ENSMUSG00000103658	0	2	0	0	0	2
ENSMUSG00000111979	0	0	0	0	0	0
ENSMUSG00000111978	0	0	1	0	0	0
ENSMUSG00000111975	0	0	0	0	0	0
ENSMUSG00000103652	0	0	0	0	0	2
ENSMUSG00000036036	67	74	61	20	34	55
ENSMUSG00000103650	0	0	0	0	0	0
ENSMUSG00000036030	19	13	17	7	8	10
ENSMUSG00000111970	0	0	0	0	0	0
ENSMUSG00000111973	1	0	2	2	0	0
ENSMUSG00000111972	0	4	0	3	0	0
ENSMUSG00000058856	0	0	0	0	0	0
ENSMUSG00000094021	0	0	0	0	0	0
ENSMUSG00000094020	0	0	0	0	0	0
ENSMUSG00000094027	0	0	2	0	0	2
ENSMUSG00000094026	0	0	0	0	0	0
ENSMUSG00000094025	0	0	0	0	0	0
ENSMUSG00000094024	0	0	0	0	0	0
ENSMUSG00000095998	0	0	0	0	0	0
ENSMUSG00000089732	0	0	0	3	0	0
ENSMUSG00000089737	0	0	4	0	0	0
ENSMUSG00000095992	0	0	0	0	0	0
ENSMUSG00000095993	0	0	0	0	0	0
ENSMUSG00000095994	0	0	0	1	0	0
ENSMUSG00000095996	0	0	0	0	0	0
ENSMUSG00000095997	0	0	0	0	0	0
ENSMUSG00000106388	0	0	0	0	0	0
ENSMUSG00000106389	0	0	0	2	0	0
ENSMUSG00000095262	8	6	9	5	4	6
ENSMUSG00000106382	0	0	0	0	0	0
ENSMUSG00000106383	50	38	50	45	55	104
ENSMUSG00000106381	0	0	0	0	0	0
ENSMUSG00000106386	118	103	82	152	128	150
ENSMUSG00000106387	0	0	1	0	0	0
ENSMUSG00000106384	91	98	89	113	98	130
ENSMUSG00000028707	0	0	0	0	0	0
ENSMUSG00000112409	2	0	0	0	1	0
ENSMUSG00000112404	0	0	0	0	0	0
ENSMUSG00000112407	0	0	0	0	0	0
ENSMUSG00000112400	0	0	0	0	0	0
ENSMUSG00000112401	0	2	0	0	0	0
ENSMUSG00000112402	4	0	0	0	0	0
ENSMUSG00000112403	0	0	0	0	0	0
ENSMUSG00000114806	0	0	0	0	0	0
ENSMUSG00000114807	0	0	0	0	0	0
ENSMUSG00000114804	0	0	0	0	0	2
ENSMUSG00000114805	0	2	9	0	7	9
ENSMUSG00000114802	0	0	0	0	0	0
ENSMUSG00000114800	28	41	14	78	99	95
ENSMUSG00000114808	0	0	0	0	0	0
ENSMUSG00000114809	0	0	0	0	0	0
ENSMUSG00000110314	0	0	0	0	0	0
ENSMUSG00000110315	0	1	0	3	0	0
ENSMUSG00000110310	0	0	0	0	0	0
ENSMUSG00000099501	0	0	0	0	0	0
ENSMUSG00000092073	0	0	0	0	0	0

ENSMUSG00000092077	2	8	2	2	3	4
ENSMUSG00000038135	17	4	0	10	7	6
ENSMUSG00000092075	0	0	0	0	0	0
ENSMUSG00000109000	0	0	0	0	0	0
ENSMUSG00000109001	0	0	0	0	0	0
ENSMUSG00000109003	0	0	0	0	4	0
ENSMUSG00000109004	0	0	0	0	0	0
ENSMUSG00000075033	40	40	57	35	46	47
ENSMUSG00000109007	0	0	0	0	0	0
ENSMUSG00000109009	0	2	0	0	0	0
ENSMUSG00000096444	0	0	0	0	0	0
ENSMUSG00000046807	13	4	21	3	14	24
ENSMUSG00000046804	0	0	0	0	0	0
ENSMUSG00000029074	5	0	0	0	0	0
ENSMUSG00000106968	4	6	0	0	6	8
ENSMUSG00000066189	0	0	0	0	0	0
ENSMUSG00000106963	0	0	0	2	4	0
ENSMUSG00000106960	0	0	0	0	0	0
ENSMUSG00000088926	0	0	0	0	0	0
ENSMUSG00000088925	0	0	0	0	0	0
ENSMUSG00000088924	0	0	0	0	0	0
ENSMUSG00000088923	0	0	0	0	0	0
ENSMUSG00000088928	0	0	0	0	0	0
ENSMUSG00000096318	0	0	0	0	0	0
ENSMUSG00000096313	0	0	0	0	0	0
ENSMUSG00000096311	0	0	0	0	0	0
ENSMUSG00000096316	0	0	0	0	0	0
ENSMUSG00000096314	0	0	0	0	0	0
ENSMUSG00000082924	0	2	0	1	0	0
ENSMUSG00000113464	0	0	0	0	0	0
ENSMUSG00000113461	0	0	0	0	0	0
ENSMUSG00000113460	0	3	0	0	0	0
ENSMUSG00000113463	0	0	0	0	0	0
ENSMUSG00000034303	240	224	202	165	155	207
ENSMUSG00000108357	0	0	0	0	0	0
ENSMUSG00000108355	0	0	0	0	0	0
ENSMUSG00000034308	136	177	195	143	150	180
ENSMUSG00000113468	0	0	0	0	0	0
ENSMUSG00000108351	0	0	0	0	0	0
ENSMUSG00000111199	0	0	0	0	0	0
ENSMUSG00000093848	0	0	0	0	0	0
ENSMUSG00000109914	0	0	0	0	0	0
ENSMUSG00000111190	0	0	0	0	0	0
ENSMUSG00000114118	0	0	6	0	4	5
ENSMUSG00000104528	6	8	0	8	5	6
ENSMUSG00000104529	0	0	0	0	0	0
ENSMUSG00000104522	0	0	0	0	0	0
ENSMUSG00000098136	0	0	1	0	0	0
ENSMUSG00000098131	0	0	0	0	0	0
ENSMUSG00000023243	27	39	22	15	18	29
ENSMUSG00000095502	0	0	0	0	0	0
ENSMUSG00000098138	4	5	3	4	12	3
ENSMUSG00000096256	8	11	9	4	3	3
ENSMUSG00000114344	0	5	13	0	0	4
ENSMUSG00000114347	0	0	0	0	0	0
ENSMUSG00000026394	0	0	0	0	0	0
ENSMUSG00000106699	0	0	0	0	0	0
ENSMUSG00000082021	0	0	0	0	0	0

ENSMUSG00000026398	68	77	74	43	87	84
ENSMUSG00000082022	0	0	0	0	0	0
ENSMUSG00000044515	0	0	0	0	0	0
ENSMUSG00000082024	1	2	1	1	1	2
ENSMUSG00000044518	0	0	0	0	0	0
ENSMUSG00000044519	0	0	0	0	0	0
ENSMUSG00000082027	0	0	0	0	0	0
ENSMUSG00000109077	0	0	0	0	0	0
ENSMUSG00000095033	0	0	0	0	0	0
ENSMUSG00000045648	0	0	0	2	0	0
ENSMUSG00000090003	0	0	0	0	0	0
ENSMUSG00000095030	0	0	0	0	0	0
ENSMUSG00000095037	0	0	0	0	0	0
ENSMUSG00000045009	23	26	38	16	33	34
ENSMUSG00000095035	0	0	0	0	0	0
ENSMUSG00000095034	0	0	0	0	0	0
ENSMUSG00000095236	0	0	0	0	0	0
ENSMUSG00000053117	7	11	12	16	11	6
ENSMUSG00000080383	1	1	1	1	0	1
ENSMUSG00000107579	0	0	0	0	0	0
ENSMUSG00000080657	0	1	0	0	0	0
ENSMUSG00000080653	0	0	0	0	0	0
ENSMUSG00000106849	0	0	0	0	0	0
ENSMUSG00000080659	0	1	0	0	0	1
ENSMUSG00000005364	2	0	0	4	0	0
ENSMUSG00000099506	0	0	0	0	0	0
ENSMUSG00000081381	0	0	0	0	0	0
ENSMUSG00000081383	0	0	0	0	0	0
ENSMUSG00000081384	2	0	0	0	0	0
ENSMUSG00000081386	0	0	0	0	0	0
ENSMUSG00000073906	12	7	2	4	8	9
ENSMUSG00000098254	0	0	0	0	0	1
ENSMUSG00000081389	0	0	0	0	0	0
ENSMUSG00000098252	0	1	0	1	1	0
ENSMUSG00000028883	158	227	168	100	106	102
ENSMUSG00000035836	4	0	0	0	0	0
ENSMUSG00000101306	0	0	0	0	0	0
ENSMUSG00000101802	0	0	0	0	0	0
ENSMUSG00000098784	0	0	5	0	0	0
ENSMUSG00000061525	0	0	0	0	0	0
ENSMUSG00000098782	0	0	0	0	0	0
ENSMUSG00000073909	0	0	2	0	0	0
ENSMUSG00000098780	0	0	0	0	0	0
ENSMUSG00000047730	0	0	0	0	0	0
ENSMUSG00000031210	0	6	0	6	0	0
ENSMUSG00000031212	0	0	0	0	0	0
ENSMUSG00000101806	0	0	0	0	0	0
ENSMUSG00000093060	0	0	0	0	0	0
ENSMUSG00000093064	0	0	0	0	1	2
ENSMUSG00000093065	0	0	0	0	0	0
ENSMUSG00000052790	0	0	0	0	0	0
ENSMUSG00000081524	0	0	1	0	0	0
ENSMUSG00000081525	0	0	0	0	0	0
ENSMUSG00000081526	0	0	0	0	0	0
ENSMUSG00000081523	0	0	0	0	0	0
ENSMUSG00000081529	3	2	4	0	1	0
ENSMUSG00000077408	0	0	0	0	0	0
ENSMUSG00000077409	6	3	2	1	5	4

ENSMUSG00000077406	0	0	0	0	0	1
ENSMUSG00000077407	1	0	0	0	0	0
ENSMUSG00000077404	0	0	0	0	0	0
ENSMUSG00000077402	1	0	0	4	0	2
ENSMUSG00000077403	0	0	0	0	0	0
ENSMUSG00000040424	21	30	18	38	32	47
ENSMUSG00000106467	0	0	0	0	0	0
ENSMUSG00000004415	14	6	11	3	0	23
ENSMUSG00000095316	0	0	0	0	0	0
ENSMUSG00000030167	5	0	14	5	0	2
ENSMUSG00000030160	0	0	0	0	0	0
ENSMUSG00000030162	8	22	27	3	0	0
ENSMUSG00000048155	0	0	0	0	0	0
ENSMUSG00000048153	3	3	0	3	0	0
ENSMUSG00000092331	0	0	0	0	0	0
ENSMUSG00000092330	0	0	0	0	0	0
ENSMUSG00000092332	0	0	0	0	0	0
ENSMUSG00000092336	0	0	0	0	0	0
ENSMUSG00000092339	1	1	0	0	0	2
ENSMUSG00000092338	0	0	0	1	3	2
ENSMUSG00000106958	0	0	0	0	0	0
ENSMUSG00000092290	0	0	0	0	0	0
ENSMUSG00000072584	12	13	4	10	21	17
ENSMUSG00000070985	0	0	0	0	0	0
ENSMUSG00000070983	0	0	0	0	0	0
ENSMUSG00000070980	5	0	0	2	5	2
ENSMUSG00000040284	0	0	0	0	0	0
ENSMUSG00000082517	0	2	0	0	0	0
ENSMUSG00000043880	0	0	0	0	0	0
ENSMUSG00000041771	0	0	0	0	0	0
ENSMUSG00000100031	0	0	0	0	0	0
ENSMUSG00000100036	0	0	0	0	0	0
ENSMUSG00000068806	2	5	0	0	2	0
ENSMUSG00000068808	0	0	0	0	0	0
ENSMUSG00000070677	0	0	0	0	0	0
ENSMUSG00000095492	0	0	0	0	0	0
ENSMUSG00000064165	0	0	0	0	0	0
ENSMUSG00000095494	0	0	0	0	0	0
ENSMUSG00000084548	0	0	0	0	0	0
ENSMUSG00000084546	0	0	0	0	0	0
ENSMUSG00000075002	0	0	0	0	0	0
ENSMUSG00000024734	0	0	0	0	0	0
ENSMUSG00000085630	0	0	0	0	0	0
ENSMUSG00000107569	0	0	0	0	0	0
ENSMUSG00000107563	0	0	0	0	0	0
ENSMUSG00000107565	0	0	0	0	0	0
ENSMUSG00000107564	0	0	0	0	0	0
ENSMUSG00000065698	0	0	0	0	0	0
ENSMUSG00000033533	0	0	0	0	0	0
ENSMUSG00000065694	2	0	2	1	1	1
ENSMUSG00000061536	211	123	222	174	140	194
ENSMUSG00000101813	1	1	1	1	0	2
ENSMUSG00000101812	0	0	0	0	0	0
ENSMUSG00000101811	0	0	0	0	0	0
ENSMUSG00000101810	0	0	0	0	0	0
ENSMUSG00000101817	0	0	0	0	0	0
ENSMUSG00000101816	0	0	0	0	0	0
ENSMUSG00000101815	0	0	0	0	0	0

ENSMUSG00000101819	3	1	1	2	2	4
ENSMUSG00000101818	0	0	0	0	0	0
ENSMUSG00000074359	0	0	0	0	0	0
ENSMUSG00000089873	0	0	0	0	0	0
ENSMUSG00000103718	0	0	0	0	0	0
ENSMUSG00000103719	0	0	0	0	0	0
ENSMUSG00000075918	9	10	2	3	3	2
ENSMUSG00000103712	0	0	0	0	0	0
ENSMUSG00000103713	0	0	3	0	0	0
ENSMUSG00000103716	0	0	2	2	0	0
ENSMUSG00000103714	0	0	0	0	0	0
ENSMUSG00000065340	0	0	0	0	0	0
ENSMUSG00000068117	0	0	0	0	0	0
ENSMUSG00000065344	0	0	0	0	0	0
ENSMUSG00000068113	2	0	0	0	0	0
ENSMUSG00000065348	0	0	0	0	0	0
ENSMUSG00000075088	0	0	0	0	0	0
ENSMUSG00000078520	0	0	0	0	0	0
ENSMUSG00000078521	14	8	6	4	5	14
ENSMUSG00000089311	0	0	0	0	0	0
ENSMUSG00000089310	0	0	0	0	0	0
ENSMUSG00000083957	0	0	0	0	0	0
ENSMUSG00000030945	0	0	0	0	0	0
ENSMUSG00000089630	0	0	2	0	0	0
ENSMUSG00000089634	3	6	7	6	5	7
ENSMUSG00000083955	0	0	0	0	0	0
ENSMUSG00000083954	2	2	0	0	0	0
ENSMUSG00000097208	14	15	18	9	10	13
ENSMUSG00000097209	0	3	0	0	0	0
ENSMUSG00000073510	0	0	0	0	0	0
ENSMUSG00000097201	0	11	0	0	0	0
ENSMUSG00000063971	0	0	0	0	0	0
ENSMUSG00000083958	0	0	0	0	0	0
ENSMUSG00000075085	0	0	0	0	0	0
ENSMUSG00000035270	24	19	46	17	17	42
ENSMUSG00000035277	0	0	0	0	0	0
ENSMUSG00000062372	0	0	0	0	0	0
ENSMUSG00000048830	0	0	0	0	0	0
ENSMUSG00000048834	5	0	0	0	0	0
ENSMUSG00000077872	0	0	0	0	0	0
ENSMUSG00000069441	6	0	0	6	12	2
ENSMUSG00000102048	0	0	0	0	0	0
ENSMUSG00000102041	0	0	0	0	3	0
ENSMUSG00000102043	0	0	0	0	0	0
ENSMUSG00000102042	0	0	0	0	0	0
ENSMUSG00000102045	0	0	0	0	0	0
ENSMUSG00000102044	0	0	0	0	0	0
ENSMUSG00000102047	0	0	2	0	0	1
ENSMUSG00000102046	0	0	0	0	0	0
ENSMUSG00000096159	0	0	0	0	0	0
ENSMUSG00000096158	0	0	0	0	0	0
ENSMUSG00000096151	2	0	0	1	0	0
ENSMUSG00000089697	0	0	0	0	0	0
ENSMUSG00000096153	0	0	0	0	0	0
ENSMUSG00000096152	0	0	0	0	0	0
ENSMUSG00000096154	0	0	0	0	0	0
ENSMUSG00000096157	3	0	1	1	0	2
ENSMUSG00000091401	0	0	1	2	4	0

ENSMUSG00000063394	0	0	0	0	0	0
ENSMUSG00000069996	0	2	1	2	0	0
ENSMUSG00000063020	0	0	0	0	0	0
ENSMUSG00000032726	3	0	3	0	0	0
ENSMUSG00000021278	0	0	12	0	3	0
ENSMUSG00000105905	0	0	0	2	0	0
ENSMUSG00000105907	0	0	2	0	0	0
ENSMUSG00000105900	0	0	0	0	0	0
ENSMUSG00000105903	0	0	0	0	0	0
ENSMUSG00000057074	0	0	0	0	0	0
ENSMUSG00000020891	0	0	0	0	0	0
ENSMUSG00000060613	0	0	0	0	0	0
ENSMUSG00000104051	2	0	1	0	0	0
ENSMUSG00000101020	0	0	0	0	0	0
ENSMUSG00000101021	0	0	0	0	0	0
ENSMUSG00000101026	0	2	0	0	0	0
ENSMUSG00000101027	0	0	0	0	0	0
ENSMUSG00000060615	0	0	0	0	0	0
ENSMUSG00000101025	0	0	0	0	0	0
ENSMUSG00000104058	0	0	0	0	0	0
ENSMUSG00000104059	0	0	0	0	1	0
ENSMUSG00000101028	28	23	22	27	27	24
ENSMUSG00000101029	0	0	0	0	0	0
ENSMUSG00000089699	0	0	0	0	0	0
ENSMUSG00000059460	0	0	0	0	0	0
ENSMUSG00000091311	0	0	0	0	0	0
ENSMUSG00000083397	3	12	13	12	13	18
ENSMUSG00000091318	2	6	0	0	1	1
ENSMUSG00000109960	0	0	2	0	0	0
ENSMUSG00000102542	7	7	0	18	8	11
ENSMUSG00000094043	0	0	0	0	0	0
ENSMUSG00000108889	0	0	0	0	0	0
ENSMUSG00000110026	0	0	0	0	0	0
ENSMUSG00000108882	0	0	0	0	0	0
ENSMUSG00000112359	0	0	0	0	0	0
ENSMUSG00000056306	0	3	0	14	8	4
ENSMUSG00000026809	0	7	3	0	3	0
ENSMUSG00000026805	13	10	0	10	5	9
ENSMUSG00000026807	0	0	0	0	2	3
ENSMUSG00000021852	20	18	13	29	24	25
ENSMUSG00000100373	0	0	0	0	0	0
ENSMUSG00000100372	0	0	0	0	0	0
ENSMUSG00000100375	0	0	0	0	0	0
ENSMUSG00000100377	0	0	0	0	0	0
ENSMUSG00000100376	0	0	0	0	0	0
ENSMUSG00000100379	0	0	0	0	0	0
ENSMUSG00000095284	0	0	0	0	0	0
ENSMUSG00000110902	0	3	4	6	0	13
ENSMUSG00000110901	0	0	0	0	0	0
ENSMUSG00000105182	1	0	0	0	1	0
ENSMUSG00000110909	0	0	0	0	0	0
ENSMUSG00000013150	214	186	177	131	205	171
ENSMUSG00000068889	0	0	0	0	0	0
ENSMUSG00000088344	2	1	2	0	3	0
ENSMUSG00000088343	1	0	0	0	0	0
ENSMUSG00000088340	0	0	0	0	0	0
ENSMUSG00000046723	0	0	0	0	0	0
ENSMUSG00000086415	0	0	0	0	0	0

ENSMUSG00000083778	0	0	0	0	0	0
ENSMUSG00000086417	24	31	39	34	23	38
ENSMUSG00000086416	0	0	0	0	0	0
ENSMUSG00000086410	2	0	3	2	0	0
ENSMUSG00000086413	0	0	0	0	0	0
ENSMUSG00000095543	0	0	0	1	0	0
ENSMUSG00000095542	0	0	0	0	0	0
ENSMUSG00000095541	1	1	2	0	1	1
ENSMUSG00000095540	0	0	0	0	0	0
ENSMUSG00000095546	0	0	0	0	0	0
ENSMUSG00000095544	0	0	0	0	0	0
ENSMUSG00000095549	0	0	0	0	0	0
ENSMUSG00000093260	0	0	0	0	0	0
ENSMUSG00000093261	0	0	0	0	0	0
ENSMUSG00000027869	0	6	0	0	0	0
ENSMUSG00000027868	70	64	97	24	35	28
ENSMUSG00000052554	0	0	0	0	0	0
ENSMUSG00000052557	901	880	1042	512	624	736
ENSMUSG00000097949	0	0	0	0	0	0
ENSMUSG00000097945	0	0	0	0	0	0
ENSMUSG00000097947	0	0	0	0	0	0
ENSMUSG00000097941	0	0	0	0	0	0
ENSMUSG00000097940	0	0	0	0	0	0
ENSMUSG00000097943	5	2	5	9	5	8
ENSMUSG00000103983	231	258	247	170	251	263
ENSMUSG00000112679	3	0	1	0	0	1
ENSMUSG00000112678	0	0	0	0	0	0
ENSMUSG00000004651	4	0	0	0	0	0
ENSMUSG00000112672	0	0	0	0	0	0
ENSMUSG00000112671	0	0	0	0	0	0
ENSMUSG00000112670	7	4	0	4	4	4
ENSMUSG00000112677	3	0	0	0	4	0
ENSMUSG00000004654	0	0	2	0	0	0
ENSMUSG00000055022	0	0	0	0	0	0
ENSMUSG00000024986	52	70	61	39	36	68
ENSMUSG00000018973	0	0	0	0	0	0
ENSMUSG00000060084	1	0	0	0	0	0
ENSMUSG00000024989	19	36	33	5	12	28
ENSMUSG00000106029	0	0	0	0	0	0
ENSMUSG00000106028	0	0	0	0	0	0
ENSMUSG00000106027	1	0	0	0	1	0
ENSMUSG00000106026	0	0	0	0	0	0
ENSMUSG00000106024	50	65	42	22	32	46
ENSMUSG00000106023	0	0	0	0	0	0
ENSMUSG00000106021	0	0	0	0	0	0
ENSMUSG00000106020	0	0	0	0	0	0
ENSMUSG00000028519	0	1	0	0	0	1
ENSMUSG00000110293	0	0	0	0	0	0
ENSMUSG00000110292	0	0	0	2	0	0
ENSMUSG00000110297	0	0	0	0	0	0
ENSMUSG00000110296	0	0	0	0	0	0
ENSMUSG00000110294	0	0	0	0	0	0
ENSMUSG00000110125	6	0	0	0	0	0
ENSMUSG00000110126	1	3	1	1	1	0
ENSMUSG00000110121	0	0	0	0	0	0
ENSMUSG00000025163	0	4	11	0	0	3
ENSMUSG00000110123	13	14	24	11	16	11
ENSMUSG00000110122	0	0	0	0	0	0

ENSMUSG00000100391	0	0	0	0	0	0
ENSMUSG00000077869	0	0	0	0	0	0
ENSMUSG00000097683	0	0	0	2	0	0
ENSMUSG00000087084	0	0	0	0	0	0
ENSMUSG00000003354	0	6	5	0	0	11
ENSMUSG00000051237	0	0	0	0	0	0
ENSMUSG00000105598	20	23	12	23	11	9
ENSMUSG00000003352	203	167	246	75	109	92
ENSMUSG00000105592	0	0	0	0	0	2
ENSMUSG00000105593	0	0	0	0	0	0
ENSMUSG00000105590	9	0	2	12	0	6
ENSMUSG00000105591	0	0	0	0	0	0
ENSMUSG00000114636	1	0	0	0	0	0
ENSMUSG00000114630	1	2	4	2	1	2
ENSMUSG00000114639	0	0	0	0	0	0
ENSMUSG00000082882	0	0	0	0	0	0
ENSMUSG00000060081	0	0	0	0	0	0
ENSMUSG00000082886	0	0	0	0	0	0
ENSMUSG00000082887	0	0	0	0	0	0
ENSMUSG00000082889	0	0	0	0	0	0
ENSMUSG00000050545	136	163	105	131	101	172
ENSMUSG00000070471	5	0	10	3	5	6
ENSMUSG00000108147	0	0	0	0	0	0
ENSMUSG00000108144	0	0	0	0	0	0
ENSMUSG00000108145	8	21	19	8	13	22
ENSMUSG00000108140	0	0	0	0	0	0
ENSMUSG00000105048	0	0	0	0	0	1
ENSMUSG00000105040	0	0	0	0	0	0
ENSMUSG00000105041	0	6	16	8	5	9
ENSMUSG00000105043	0	2	1	0	0	0
ENSMUSG00000105044	4	4	6	0	5	5
ENSMUSG00000105045	0	0	4	0	0	0
ENSMUSG00000105047	0	0	0	0	0	1
ENSMUSG00000111656	4	0	12	5	3	6
ENSMUSG00000111657	0	0	0	0	0	0
ENSMUSG00000101666	0	0	0	0	0	0
ENSMUSG00000111652	5	4	0	2	0	3
ENSMUSG00000111658	0	0	0	0	0	0
ENSMUSG00000057534	0	0	0	0	0	0
ENSMUSG00000037995	57	53	102	21	55	42
ENSMUSG00000025838	0	0	0	0	0	0
ENSMUSG00000025839	0	0	0	0	0	0
ENSMUSG00000085824	0	0	0	0	0	0
ENSMUSG00000104317	0	1	0	0	0	0
ENSMUSG00000104314	0	0	0	0	0	4
ENSMUSG00000104313	0	0	0	0	0	0
ENSMUSG00000104312	0	0	0	0	0	0
ENSMUSG00000104311	0	0	0	0	0	0
ENSMUSG00000104310	0	0	0	0	0	0
ENSMUSG00000104319	0	0	0	0	0	0
ENSMUSG00000017922	0	0	0	0	0	0
ENSMUSG00000076117	1	1	1	2	0	1
ENSMUSG00000089253	3	0	0	0	2	0
ENSMUSG00000044724	0	0	4	0	0	0
ENSMUSG00000044726	0	0	4	0	0	0
ENSMUSG00000053091	172	201	199	155	194	253
ENSMUSG00000083828	0	0	0	0	0	0
ENSMUSG00000001444	0	0	6	0	0	0

ENSMUSG00000083821	0	0	0	0	0	0
ENSMUSG00000083825	0	0	0	0	0	0
ENSMUSG00000042894	0	0	0	0	0	0
ENSMUSG00000027478	113	113	113	74	57	119
ENSMUSG00000092906	1	0	0	0	0	0
ENSMUSG00000027470	44	17	72	0	0	0
ENSMUSG00000078057	0	0	0	0	0	0
ENSMUSG00000099496	0	0	0	0	0	0
ENSMUSG00000090215	41	36	42	44	37	40
ENSMUSG00000077972	0	0	0	0	0	0
ENSMUSG00000086824	3	5	8	0	3	0
ENSMUSG00000098429	0	0	0	0	0	0
ENSMUSG00000098423	0	0	0	0	0	0
ENSMUSG00000098421	0	0	0	0	0	0
ENSMUSG00000098420	0	0	0	0	0	0
ENSMUSG00000098425	0	3	2	1	0	0
ENSMUSG00000098424	20	12	13	30	27	29
ENSMUSG00000042761	7	5	0	0	7	0
ENSMUSG00000032978	0	0	0	0	0	0
ENSMUSG00000095514	0	0	0	0	0	2
ENSMUSG00000074882	0	0	0	0	0	0
ENSMUSG00000046650	0	0	0	0	0	0
ENSMUSG00000074887	0	0	0	0	0	0
ENSMUSG00000074885	0	0	0	0	0	6
ENSMUSG00000086995	0	0	0	0	0	0
ENSMUSG00000086996	2	0	0	0	0	0
ENSMUSG00000086997	10	10	5	3	9	0
ENSMUSG00000086999	0	0	0	0	0	0
ENSMUSG00000052058	0	0	0	0	0	0
ENSMUSG00000111095	0	1	0	0	0	0
ENSMUSG00000093747	0	0	0	0	0	0
ENSMUSG00000093746	0	0	0	0	1	0
ENSMUSG00000043388	5	8	8	0	0	0
ENSMUSG00000093743	3	0	0	0	0	0
ENSMUSG00000093741	0	0	0	0	0	0
ENSMUSG00000093740	0	1	4	0	0	2
ENSMUSG00000043383	0	3	0	0	0	0
ENSMUSG00000049985	17	24	32	18	12	14
ENSMUSG00000043385	12	25	20	16	36	29
ENSMUSG00000101899	0	0	0	2	0	0
ENSMUSG00000101898	0	0	0	0	0	0
ENSMUSG00000028813	0	0	0	0	0	0
ENSMUSG00000049670	42	40	56	44	23	55
ENSMUSG00000049676	49	36	57	8	18	16
ENSMUSG00000033316	0	0	3	0	0	0
ENSMUSG00000103620	1	0	4	0	7	5
ENSMUSG00000101220	0	0	0	0	0	0
ENSMUSG00000101221	0	0	0	0	0	0
ENSMUSG00000101222	0	0	0	0	0	0
ENSMUSG00000101223	3	0	8	0	0	0
ENSMUSG00000101224	0	0	0	0	0	4
ENSMUSG00000101226	0	0	0	0	2	2
ENSMUSG00000101227	0	0	0	0	0	0
ENSMUSG00000101229	0	0	0	0	0	0
ENSMUSG00000096852	0	0	1	0	1	0
ENSMUSG00000088890	0	0	0	0	0	0
ENSMUSG00000088896	0	0	0	0	0	0
ENSMUSG00000051528	2	0	0	0	2	2

ENSMUSG00000087112	0	2	0	0	2	0
ENSMUSG00000087117	0	0	0	0	0	0
ENSMUSG00000087116	0	0	0	0	0	0
ENSMUSG00000087115	0	0	0	0	0	0
ENSMUSG00000087114	0	0	0	0	0	0
ENSMUSG00000099464	0	0	0	0	0	0
ENSMUSG00000101598	0	0	0	0	0	0
ENSMUSG00000099467	0	0	0	0	0	0
ENSMUSG00000099460	0	0	0	0	0	0
ENSMUSG00000099461	0	0	0	0	0	0
ENSMUSG00000099462	2	0	0	0	0	0
ENSMUSG00000099463	0	0	0	0	0	0
ENSMUSG00000101592	0	0	0	0	0	0
ENSMUSG00000101593	0	0	0	0	0	0
ENSMUSG00000101590	0	0	0	0	0	0
ENSMUSG00000099468	0	0	0	0	0	0
ENSMUSG00000099469	0	0	0	0	0	0
ENSMUSG00000014704	17	8	0	10	12	15
ENSMUSG00000101595	0	2	0	0	0	0
ENSMUSG00000096591	2	1	1	1	2	3
ENSMUSG00000094661	7	0	6	0	0	0
ENSMUSG00000082699	0	0	0	0	0	3
ENSMUSG00000082698	0	0	0	0	0	0
ENSMUSG00000082696	0	0	0	0	0	0
ENSMUSG00000100175	0	0	0	0	0	0
ENSMUSG00000100174	25	30	27	18	24	8
ENSMUSG00000089094	0	0	0	0	0	0
ENSMUSG00000030468	8	24	10	0	8	15
ENSMUSG00000100171	0	0	0	0	0	0
ENSMUSG00000082690	0	0	0	0	0	0
ENSMUSG00000094662	0	0	0	0	0	0
ENSMUSG00000094889	0	0	1	0	1	0
ENSMUSG00000042031	0	0	0	0	0	0
ENSMUSG00000083128	0	0	0	0	0	0
ENSMUSG00000083129	0	0	0	0	0	0
ENSMUSG00000083126	0	0	0	0	0	0
ENSMUSG00000083124	0	0	0	0	0	0
ENSMUSG00000050635	0	0	2	0	0	0
ENSMUSG00000089187	0	0	0	0	0	0
ENSMUSG00000083120	0	0	0	0	0	0
ENSMUSG00000000183	31	52	43	14	22	28
ENSMUSG00000094886	0	0	0	0	0	0
ENSMUSG00000094881	0	0	0	0	0	0
ENSMUSG00000041523	0	3	5	0	0	0
ENSMUSG00000094880	0	0	0	0	1	1
ENSMUSG00000111034	0	5	10	0	6	0
ENSMUSG00000064739	0	0	0	0	0	0
ENSMUSG00000060890	0	0	3	0	5	3
ENSMUSG00000064732	0	0	0	0	0	0
ENSMUSG00000064735	0	0	0	0	0	2
ENSMUSG00000064737	0	0	0	0	0	0
ENSMUSG00000101016	0	0	0	0	0	0
ENSMUSG00000107334	0	3	0	0	0	4
ENSMUSG00000107336	29	20	35	24	26	16
ENSMUSG00000107337	0	0	0	0	0	0
ENSMUSG00000079598	0	10	0	0	12	4
ENSMUSG00000079594	0	0	0	0	0	0
ENSMUSG00000079595	0	0	0	0	0	0

ENSMUSG00000087801	0	0	0	0	0	0
ENSMUSG00000087800	0	0	0	0	0	0
ENSMUSG00000087802	0	0	0	2	0	2
ENSMUSG00000087805	0	1	1	1	0	0
ENSMUSG00000087804	0	0	0	0	0	0
ENSMUSG00000087806	0	0	0	0	0	0
ENSMUSG00000085407	4	0	7	0	6	0
ENSMUSG00000091497	0	0	0	0	0	0
ENSMUSG00000085405	0	0	0	0	0	0
ENSMUSG00000085404	0	0	0	0	0	0
ENSMUSG00000074146	0	0	0	3	0	0
ENSMUSG00000091491	0	0	0	0	0	0
ENSMUSG00000015890	2	0	0	0	0	0
ENSMUSG00000091490	0	0	0	0	1	0
ENSMUSG00000036449	0	0	2	0	0	0
ENSMUSG00000079042	0	0	0	0	0	3
ENSMUSG00000088750	0	0	0	0	0	0
ENSMUSG00000088751	0	0	0	0	0	0
ENSMUSG00000088754	0	0	0	0	0	0
ENSMUSG00000094968	1	0	0	0	0	0
ENSMUSG00000094963	0	0	0	0	0	0
ENSMUSG00000094960	0	0	0	0	0	0
ENSMUSG00000077799	0	0	0	0	0	0
ENSMUSG00000094967	0	0	0	0	0	0
ENSMUSG00000094964	1	0	0	1	0	0
ENSMUSG00000094965	0	0	1	0	1	0
ENSMUSG00000104277	13	19	16	15	8	4
ENSMUSG00000062497	0	1	0	0	0	0
ENSMUSG00000050504	0	0	0	0	0	0
ENSMUSG00000061769	0	0	0	0	0	0
ENSMUSG00000105915	0	0	0	0	0	0
ENSMUSG00000059203	0	0	0	0	0	0
ENSMUSG00000059206	0	0	0	0	0	0
ENSMUSG00000063728	0	0	0	0	0	0
ENSMUSG00000078315	0	0	0	0	0	0
ENSMUSG00000078314	11	16	40	20	25	22
ENSMUSG00000024215	2	0	0	0	0	0
ENSMUSG00000024211	0	0	5	2	5	8
ENSMUSG00000103528	0	0	0	0	0	0
ENSMUSG00000103521	0	0	0	0	0	0
ENSMUSG00000107626	0	0	0	0	0	0
ENSMUSG00000103523	0	0	0	0	0	0
ENSMUSG00000103524	0	0	0	0	0	0
ENSMUSG00000103526	0	0	0	0	0	0
ENSMUSG00000022900	0	3	0	0	0	4
ENSMUSG00000022902	0	0	0	0	0	0
ENSMUSG00000097435	2	0	0	0	0	0
ENSMUSG00000097434	0	0	0	0	1	0
ENSMUSG00000097437	0	0	0	0	0	0
ENSMUSG00000051037	85	105	86	64	48	83
ENSMUSG00000051036	0	0	0	0	6	0
ENSMUSG00000051034	286	195	215	198	279	347
ENSMUSG00000051038	0	0	0	0	0	0
ENSMUSG00000100951	0	0	0	0	0	0
ENSMUSG00000105396	0	0	0	0	0	0
ENSMUSG00000100953	0	0	0	0	0	0
ENSMUSG00000100952	0	0	0	0	0	0
ENSMUSG00000114437	0	0	0	0	0	0

ENSMUSG00000100957	0	0	0	0	0	0
ENSMUSG00000114434	0	0	0	0	0	0
ENSMUSG00000100959	0	0	0	0	0	0
ENSMUSG00000114439	0	0	0	0	0	0
ENSMUSG00000058550	0	0	0	0	0	0
ENSMUSG00000050783	0	0	0	0	0	2
ENSMUSG00000054200	0	0	6	0	0	0
ENSMUSG00000050788	26	11	14	5	4	12
ENSMUSG00000093827	0	0	4	2	4	0
ENSMUSG00000051788	0	0	0	0	0	0
ENSMUSG00000105206	0	0	0	0	0	0
ENSMUSG00000105205	0	0	0	0	0	0
ENSMUSG00000114389	2	0	0	0	0	0
ENSMUSG00000114388	0	0	0	0	0	0
ENSMUSG00000114385	0	0	0	0	0	0
ENSMUSG00000114384	0	0	0	0	0	0
ENSMUSG00000093824	0	0	0	0	0	0
ENSMUSG00000114381	0	0	0	0	0	0
ENSMUSG00000114383	0	1	0	0	0	0
ENSMUSG00000105209	0	0	0	0	0	0
ENSMUSG00000066803	0	0	0	0	0	0
ENSMUSG00000066804	0	0	0	0	0	0
ENSMUSG00000091873	1	0	0	0	0	0
ENSMUSG00000065570	2	2	1	2	0	0
ENSMUSG00000091879	0	0	0	0	0	0
ENSMUSG00000086791	0	0	0	0	0	0
ENSMUSG00000080300	0	0	0	0	0	0
ENSMUSG00000065572	0	0	0	0	0	0
ENSMUSG00000071714	79	86	149	14	16	34
ENSMUSG00000106395	11	9	6	12	10	19
ENSMUSG00000065574	0	0	0	0	0	0
ENSMUSG00000096473	2	4	1	1	1	1
ENSMUSG00000096470	0	0	0	0	0	0
ENSMUSG00000096476	0	0	0	0	0	0
ENSMUSG00000096477	0	0	0	0	0	0
ENSMUSG00000096475	0	0	0	0	0	1
ENSMUSG00000096479	0	0	0	0	0	0
ENSMUSG00000108434	12	4	7	8	7	7
ENSMUSG00000108432	0	0	0	0	0	0
ENSMUSG00000108433	0	0	0	0	0	0
ENSMUSG00000108430	0	0	0	0	0	0
ENSMUSG00000108431	0	0	0	0	0	0
ENSMUSG00000108438	0	0	1	1	0	0
ENSMUSG00000071716	8	3	3	3	5	0
ENSMUSG00000026109	24	14	8	5	4	4
ENSMUSG00000021337	0	0	0	0	0	0
ENSMUSG00000021336	0	0	0	0	0	0
ENSMUSG00000021335	0	0	0	0	0	0
ENSMUSG00000026100	20	8	10	7	12	7
ENSMUSG00000081442	0	0	0	0	0	0
ENSMUSG00000106677	0	0	0	0	0	0
ENSMUSG00000091296	0	0	0	0	0	0
ENSMUSG00000091293	0	0	0	0	0	2
ENSMUSG00000045030	0	0	0	1	0	0
ENSMUSG00000000982	14	0	13	0	0	4
ENSMUSG00000045034	0	5	17	6	7	0
ENSMUSG00000111265	0	0	0	0	0	0
ENSMUSG00000111267	0	0	0	0	0	0

ENSMUSG00000111266	0	0	0	0	0	0
ENSMUSG00000111263	0	0	0	0	0	0
ENSMUSG00000111262	0	0	0	0	0	0
ENSMUSG00000027270	0	0	0	0	0	0
ENSMUSG00000027273	0	0	0	0	0	0
ENSMUSG00000091129	0	0	0	0	0	0
ENSMUSG00000017000	0	0	0	0	0	0
ENSMUSG00000017003	0	0	0	0	0	0
ENSMUSG00000017004	0	0	0	0	0	0
ENSMUSG00000111454	0	0	0	0	0	0
ENSMUSG00000098172	0	0	0	0	0	0
ENSMUSG00000099730	0	0	0	0	0	0
ENSMUSG00000014603	0	0	0	0	0	0
ENSMUSG00000090457	0	0	0	0	0	0
ENSMUSG00000090455	0	0	0	0	0	0
ENSMUSG00000081779	0	0	0	0	2	0
ENSMUSG00000081778	0	0	0	0	0	0
ENSMUSG00000090451	11	13	16	8	5	12
ENSMUSG00000081775	0	0	0	0	0	0
ENSMUSG00000081774	0	0	0	0	0	0
ENSMUSG00000081777	0	0	0	0	3	1
ENSMUSG00000081771	14	8	2	2	6	8
ENSMUSG00000081770	0	0	0	0	0	0
ENSMUSG00000103213	0	0	0	0	0	0
ENSMUSG00000053522	7	13	0	0	0	0
ENSMUSG00000053528	0	0	0	0	0	0
ENSMUSG00000042567	9	2	2	0	0	0
ENSMUSG00000094513	1	0	0	0	0	0
ENSMUSG00000094511	0	0	0	0	0	0
ENSMUSG00000094510	1	0	0	0	0	0
ENSMUSG00000094516	0	0	0	0	0	0
ENSMUSG00000094515	1	0	1	0	0	0
ENSMUSG00000094514	0	0	1	0	0	3
ENSMUSG00000111841	0	0	0	0	0	0
ENSMUSG00000111843	0	0	0	0	0	0
ENSMUSG00000044814	5	11	0	11	4	6
ENSMUSG00000111849	0	0	0	0	0	0
ENSMUSG00000090989	0	0	0	0	0	0
ENSMUSG00000044819	0	0	0	0	0	0
ENSMUSG00000008601	0	3	0	0	3	0
ENSMUSG00000051176	0	0	0	0	0	0
ENSMUSG00000093187	2	2	0	0	1	1
ENSMUSG00000046719	0	6	3	5	6	0
ENSMUSG00000093180	0	0	0	0	0	0
ENSMUSG00000093183	0	0	0	0	0	0
ENSMUSG00000086204	0	0	0	0	0	0
ENSMUSG00000086205	0	0	0	0	0	0
ENSMUSG00000086207	0	0	0	0	0	0
ENSMUSG00000046716	0	0	0	0	0	0
ENSMUSG00000046717	0	0	0	0	0	0
ENSMUSG00000086202	0	0	0	0	0	0
ENSMUSG00000086203	0	0	0	0	0	0
ENSMUSG00000051172	1	0	0	0	0	0
ENSMUSG00000081252	0	0	0	0	0	0
ENSMUSG00000028236	0	0	3	0	0	0
ENSMUSG00000028238	13	19	5	3	0	3
ENSMUSG00000109538	0	0	0	2	0	0
ENSMUSG00000029544	103	156	169	62	162	108

ENSMUSG00000029546	0	0	0	0	0	0
ENSMUSG00000029541	0	0	0	0	0	0
ENSMUSG00000114576	0	0	0	0	2	2
ENSMUSG00000092588	0	0	0	0	0	0
ENSMUSG00000092583	0	0	0	0	0	0
ENSMUSG00000092580	0	0	0	0	0	0
ENSMUSG00000114574	0	0	0	0	0	0
ENSMUSG00000092584	0	0	0	0	0	0
ENSMUSG00000100850	0	0	0	0	0	0
ENSMUSG00000114573	15	12	10	5	9	9
ENSMUSG00000057195	0	0	0	0	0	0
ENSMUSG00000114570	0	0	0	0	0	0
ENSMUSG00000071665	0	0	0	0	0	0
ENSMUSG00000096808	2	0	1	1	1	1
ENSMUSG00000096803	0	0	0	0	0	0
ENSMUSG00000114838	0	0	0	0	0	0
ENSMUSG00000096800	0	0	0	0	0	0
ENSMUSG00000096804	0	0	0	0	0	0
ENSMUSG00000076126	2	0	0	0	2	0
ENSMUSG00000076120	0	0	0	0	0	0
ENSMUSG00000106276	0	0	0	0	0	0
ENSMUSG00000106274	0	0	0	0	0	0
ENSMUSG00000106275	0	0	0	0	0	0
ENSMUSG00000081361	0	0	0	0	0	0
ENSMUSG00000106270	0	0	0	0	0	0
ENSMUSG00000047048	0	0	0	0	0	0
ENSMUSG00000047046	0	0	0	0	0	0
ENSMUSG00000103219	0	4	6	0	0	0
ENSMUSG00000026830	0	0	0	0	0	0
ENSMUSG00000109426	1	0	0	0	0	0
ENSMUSG00000109424	0	2	0	0	0	0
ENSMUSG00000109422	1	0	0	0	2	2
ENSMUSG00000109423	0	0	0	0	0	0
ENSMUSG00000109421	2	0	0	0	0	0
ENSMUSG00000102661	0	0	0	0	0	0
ENSMUSG00000026832	114	143	242	72	44	69
ENSMUSG00000082459	0	0	0	0	0	0
ENSMUSG00000020545	0	0	0	0	0	0
ENSMUSG00000026835	0	0	0	0	0	0
ENSMUSG00000082451	0	0	0	0	0	0
ENSMUSG00000082450	0	0	0	0	0	0
ENSMUSG00000082453	0	0	0	0	0	0
ENSMUSG00000026834	7	0	11	14	12	4
ENSMUSG00000050996	0	0	0	0	0	0
ENSMUSG00000050994	2	6	8	0	4	4
ENSMUSG00000102667	0	0	0	0	0	0
ENSMUSG00000098875	0	0	0	0	0	0
ENSMUSG00000084118	0	0	0	0	0	0
ENSMUSG00000084119	0	0	0	0	0	0
ENSMUSG00000083329	0	0	0	0	0	0
ENSMUSG00000084112	0	0	0	0	0	0
ENSMUSG00000084110	2	1	3	2	2	1
ENSMUSG00000083320	0	0	0	0	0	0
ENSMUSG00000084117	0	0	0	0	0	0
ENSMUSG00000084114	0	0	0	0	0	0
ENSMUSG00000084115	5	4	8	10	10	6
ENSMUSG00000112800	12	12	7	31	51	35
ENSMUSG00000061959	12	10	6	47	11	28

ENSMUSG00000100369	0	0	0	0	0	0
ENSMUSG00000054523	0	0	0	0	0	0
ENSMUSG00000099059	1	1	1	1	1	1
ENSMUSG00000108669	0	0	0	0	0	0
ENSMUSG00000070203	0	0	0	0	0	0
ENSMUSG00000099054	0	0	0	0	0	0
ENSMUSG00000099057	0	0	0	0	0	0
ENSMUSG00000112807	0	0	0	0	0	0
ENSMUSG00000099051	8	6	5	2	4	3
ENSMUSG00000099053	0	0	0	0	0	0
ENSMUSG00000100363	0	0	0	0	0	0
ENSMUSG00000040907	8	8	15	0	6	8
ENSMUSG00000040901	0	0	0	0	0	4
ENSMUSG00000108666	0	0	0	0	0	0
ENSMUSG00000000617	0	0	0	0	0	0
ENSMUSG00000068431	2	0	0	0	0	0
ENSMUSG00000044021	4	4	8	0	7	0
ENSMUSG00000044026	117	128	125	162	163	219
ENSMUSG00000044024	0	4	2	4	0	10
ENSMUSG00000044025	2	0	0	0	0	0
ENSMUSG00000044029	3	5	2	4	0	0
ENSMUSG00000085248	0	0	0	0	0	0
ENSMUSG00000070258	0	0	0	0	0	0
ENSMUSG00000093974	0	0	0	0	0	0
ENSMUSG00000093976	0	0	0	0	0	0
ENSMUSG00000093977	0	1	0	0	0	0
ENSMUSG00000093971	0	0	1	0	0	0
ENSMUSG00000093973	0	0	0	0	0	0
ENSMUSG00000026668	11	6	0	0	0	0
ENSMUSG00000093979	36	29	39	9	14	12
ENSMUSG00000105321	0	0	0	0	0	0
ENSMUSG00000105320	3	0	0	0	0	3
ENSMUSG00000105325	0	0	0	0	0	0
ENSMUSG00000080142	0	0	0	0	0	0
ENSMUSG00000045378	0	0	0	0	0	0
ENSMUSG00000081891	0	0	0	0	0	0
ENSMUSG00000081892	26	39	26	39	7	20
ENSMUSG00000033900	169	163	149	116	109	197
ENSMUSG00000033906	100	103	143	26	54	83
ENSMUSG00000033904	348	327	425	234	298	229
ENSMUSG00000081898	0	0	0	0	0	0
ENSMUSG00000098096	0	0	0	0	0	0
ENSMUSG00000098094	0	0	0	0	0	0
ENSMUSG00000098095	0	0	0	0	0	0
ENSMUSG00000098092	15	2	7	6	9	9
ENSMUSG00000098091	73	84	102	72	95	132
ENSMUSG00000098099	0	0	0	2	0	0
ENSMUSG00000063522	4	0	3	6	0	6
ENSMUSG00000099941	0	1	0	0	0	0
ENSMUSG00000099940	0	0	0	0	0	0
ENSMUSG00000099946	2	0	0	0	0	0
ENSMUSG00000099945	0	0	0	0	0	0
ENSMUSG00000099944	0	0	0	0	0	0
ENSMUSG00000074436	0	0	0	0	0	0
ENSMUSG00000074437	0	0	0	0	0	0
ENSMUSG00000074434	0	0	0	0	0	0
ENSMUSG00000074435	0	0	0	0	0	0
ENSMUSG00000074433	0	0	0	0	0	0

ENSMUSG00000074439	0	0	0	0	0	0
ENSMUSG00000081234	0	0	0	0	0	0
ENSMUSG00000081235	0	0	0	0	0	0
ENSMUSG00000081237	0	0	0	0	0	0
ENSMUSG00000081231	0	0	0	0	0	0
ENSMUSG00000063297	4	17	0	10	24	25
ENSMUSG00000076212	0	0	0	0	0	0
ENSMUSG00000087468	0	0	0	0	0	0
ENSMUSG00000072874	0	0	0	0	0	3
ENSMUSG00000072878	0	1	0	0	0	0
ENSMUSG00000048469	0	0	0	0	0	0
ENSMUSG00000087462	0	0	0	0	0	0
ENSMUSG00000077316	0	0	0	0	0	2
ENSMUSG00000077317	2	0	0	0	0	0
ENSMUSG00000077314	1	0	0	3	2	0
ENSMUSG00000077315	0	0	1	0	0	0
ENSMUSG00000086487	4	0	0	0	3	0
ENSMUSG00000114631	0	0	0	0	0	0
ENSMUSG00000102940	2	3	0	4	0	0
ENSMUSG00000102941	0	0	0	0	3	0
ENSMUSG00000102942	0	0	0	0	0	0
ENSMUSG00000102943	0	0	0	0	0	0
ENSMUSG00000102946	0	0	0	0	0	3
ENSMUSG00000102948	3	0	0	6	0	3
ENSMUSG00000102949	0	0	0	0	0	0
ENSMUSG00000043020	0	0	2	0	0	0
ENSMUSG00000088353	0	0	0	0	0	0
ENSMUSG00000015665	0	0	0	0	0	0
ENSMUSG00000033122	0	0	0	0	0	0
ENSMUSG00000067998	9	6	4	2	5	0
ENSMUSG00000082155	0	0	0	0	0	0
ENSMUSG00000082156	0	0	0	0	0	0
ENSMUSG00000082157	0	0	0	0	2	2
ENSMUSG00000087306	6	2	0	0	0	0
ENSMUSG00000088175	0	0	0	0	0	1
ENSMUSG00000082152	0	0	3	3	3	5
ENSMUSG00000088177	0	0	0	0	0	0
ENSMUSG00000088179	0	0	0	0	0	0
ENSMUSG00000087308	0	0	0	0	0	0
ENSMUSG00000087309	0	0	0	0	0	0
ENSMUSG00000082158	0	0	0	0	0	0
ENSMUSG00000082159	0	0	0	0	0	0
ENSMUSG00000101419	0	0	0	0	0	0
ENSMUSG00000101418	0	0	0	0	0	0
ENSMUSG00000101417	0	0	0	0	0	0
ENSMUSG00000101411	0	0	0	0	0	0
ENSMUSG00000037636	5	6	23	4	4	8
ENSMUSG00000037638	194	33	38	185	157	171
ENSMUSG00000103308	0	0	0	0	0	0
ENSMUSG00000103305	0	0	0	0	0	0
ENSMUSG00000103306	0	0	0	0	0	0
ENSMUSG00000096621	0	0	0	0	0	0
ENSMUSG00000096620	0	0	0	0	0	0
ENSMUSG00000060176	11	13	10	13	10	14
ENSMUSG00000060177	0	0	0	0	0	0
ENSMUSG00000040046	0	0	0	0	0	0
ENSMUSG00000107819	0	0	0	0	0	0
ENSMUSG00000107818	0	0	0	0	0	0

ENSMUSG00000107810	104	128	155	99	127	221
ENSMUSG00000059816	0	0	0	0	0	0
ENSMUSG00000107815	0	0	0	0	0	0
ENSMUSG00000107817	0	0	0	0	0	0
ENSMUSG00000107816	0	0	0	0	0	0
ENSMUSG00000084650	0	0	0	0	0	0
ENSMUSG00000084653	0	2	0	0	3	0
ENSMUSG00000084652	0	0	0	0	0	0
ENSMUSG00000084654	0	0	0	0	0	0
ENSMUSG00000089229	0	0	0	0	0	0
ENSMUSG00000089228	1	0	0	0	0	0
ENSMUSG00000089227	0	0	0	0	0	0
ENSMUSG00000089226	0	0	0	0	0	0
ENSMUSG00000089220	0	0	0	0	0	0
ENSMUSG00000100458	0	0	0	0	0	0
ENSMUSG00000100459	0	0	0	0	0	0
ENSMUSG00000036949	0	13	12	0	0	0
ENSMUSG00000100450	0	0	0	0	0	0
ENSMUSG00000100451	0	0	0	0	0	0
ENSMUSG00000100456	0	0	0	0	0	0
ENSMUSG00000103694	40	44	26	24	13	16
ENSMUSG00000022376	13	22	10	34	32	49
ENSMUSG00000032657	63	50	67	37	40	46
ENSMUSG00000031847	8	6	16	0	17	6
ENSMUSG00000095910	0	0	0	0	0	0
ENSMUSG00000021720	87	88	140	47	51	51
ENSMUSG00000095912	3	5	7	0	0	0
ENSMUSG00000095916	0	0	0	0	0	0
ENSMUSG00000094831	0	0	0	0	0	0
ENSMUSG00000075427	0	2	0	2	0	2
ENSMUSG00000102163	0	0	0	0	0	0
ENSMUSG00000035560	0	0	0	0	0	0
ENSMUSG00000102166	0	0	0	0	0	0
ENSMUSG00000102164	0	0	0	0	0	0
ENSMUSG00000107106	17	11	18	26	30	33
ENSMUSG00000052909	0	0	0	0	0	0
ENSMUSG00000107108	0	0	0	3	0	0
ENSMUSG00000065450	0	0	0	0	0	0
ENSMUSG00000080890	0	0	0	0	0	0
ENSMUSG00000065456	0	0	0	0	0	0
ENSMUSG00000065457	0	0	0	0	0	0
ENSMUSG00000065454	0	0	0	0	0	0
ENSMUSG00000065458	1	0	1	1	1	1
ENSMUSG00000079544	0	0	0	0	0	0
ENSMUSG00000021553	0	0	0	0	0	0
ENSMUSG00000029788	0	0	0	0	0	0
ENSMUSG00000028789	113	112	178	41	53	74
ENSMUSG00000028784	0	0	0	0	0	0
ENSMUSG00000028782	68	63	39	46	36	60
ENSMUSG00000069171	155	79	110	55	73	89
ENSMUSG00000029260	2	2	0	0	0	0
ENSMUSG00000063887	0	0	2	0	2	0
ENSMUSG00000039809	0	0	0	2	0	0
ENSMUSG00000069280	0	0	0	0	0	0
ENSMUSG00000063881	0	0	0	0	0	0
ENSMUSG00000069289	0	0	0	0	0	0
ENSMUSG00000035387	0	0	0	0	0	0
ENSMUSG00000024008	0	0	0	4	0	8

ENSMUSG00000106478	0	0	0	0	0	0
ENSMUSG00000106474	1	1	0	1	0	0
ENSMUSG00000029269	0	0	0	0	0	0
ENSMUSG00000113757	0	0	0	0	0	0
ENSMUSG00000113755	0	0	0	0	0	0
ENSMUSG00000113754	0	0	0	0	0	0
ENSMUSG00000113759	0	0	0	0	0	0
ENSMUSG00000113758	0	0	0	0	0	0
ENSMUSG00000112077	0	0	0	0	0	0
ENSMUSG00000109628	0	0	0	0	0	0
ENSMUSG00000109629	0	0	0	0	0	0
ENSMUSG00000093767	0	0	0	0	0	0
ENSMUSG00000111445	0	0	0	0	0	4
ENSMUSG00000109625	8	4	12	16	7	8
ENSMUSG00000109626	0	0	0	0	0	0
ENSMUSG00000109627	0	0	0	0	0	0
ENSMUSG00000111441	0	0	0	0	0	0
ENSMUSG00000109621	0	0	0	0	0	0
ENSMUSG00000111443	0	0	0	0	0	0
ENSMUSG00000066101	0	0	0	0	0	0
ENSMUSG00000066100	2	0	0	0	0	0
ENSMUSG00000099917	0	0	0	0	0	0
ENSMUSG00000011350	0	0	0	0	0	0
ENSMUSG00000050875	0	0	0	0	0	0
ENSMUSG00000034384	0	0	0	0	0	0
ENSMUSG00000034382	27	15	27	0	8	9
ENSMUSG00000093258	0	0	0	0	0	0
ENSMUSG00000111200	0	0	0	0	0	0
ENSMUSG00000111057	0	0	0	0	0	0
ENSMUSG00000069824	0	0	0	0	0	0
ENSMUSG00000069823	0	0	0	0	0	0
ENSMUSG00000093254	0	0	0	0	0	0
ENSMUSG00000051596	0	0	0	0	0	0
ENSMUSG00000051590	17	11	20	28	15	12
ENSMUSG00000051591	0	0	0	0	0	0
ENSMUSG00000051592	0	0	0	0	0	0
ENSMUSG00000051593	0	0	0	0	0	0
ENSMUSG00000096393	0	0	0	0	0	0
ENSMUSG00000096392	0	0	0	0	0	0
ENSMUSG00000094204	0	0	0	0	0	0
ENSMUSG00000111050	0	0	0	0	0	0
ENSMUSG00000099918	0	0	0	0	0	0
ENSMUSG00000096399	0	0	0	0	0	0
ENSMUSG00000096398	1	0	0	0	0	0
ENSMUSG00000097668	0	0	0	0	2	0
ENSMUSG00000112934	0	0	0	0	0	2
ENSMUSG00000112935	0	0	0	0	0	0
ENSMUSG00000054418	37	52	34	4	10	14
ENSMUSG00000112937	6	0	0	0	0	0
ENSMUSG00000112930	0	0	0	0	0	0
ENSMUSG00000112931	0	0	0	0	0	0
ENSMUSG00000112932	4	2	5	4	3	3
ENSMUSG00000112933	0	0	0	0	0	0
ENSMUSG00000091620	0	0	0	0	0	0
ENSMUSG00000109996	0	0	0	0	0	0
ENSMUSG00000109994	0	0	0	0	0	0
ENSMUSG00000109995	0	0	0	0	0	0
ENSMUSG00000114198	0	0	0	0	0	0

ENSMUSG00000114199	0	0	0	0	0	0
ENSMUSG00000109990	0	0	0	0	0	0
ENSMUSG00000109991	0	0	0	0	0	0
ENSMUSG00000114194	0	0	0	0	3	0
ENSMUSG00000114195	0	0	0	0	0	0
ENSMUSG00000114197	2	0	0	0	0	0
ENSMUSG00000114191	0	0	0	0	0	0
ENSMUSG00000114192	0	0	0	0	0	0
ENSMUSG00000109999	0	0	0	0	0	0
ENSMUSG00000058743	52	70	107	19	40	27
ENSMUSG00000058741	0	0	0	0	0	0
ENSMUSG00000110473	1	0	0	0	0	0
ENSMUSG00000110470	0	0	0	0	0	0
ENSMUSG00000110479	0	0	0	0	0	0
ENSMUSG00000056812	0	0	0	0	0	0
ENSMUSG00000097958	1	0	0	0	0	3
ENSMUSG00000096537	0	0	0	0	0	0
ENSMUSG00000055994	141	202	232	128	183	172
ENSMUSG00000096535	0	1	1	1	1	1
ENSMUSG00000097959	0	0	0	2	0	0
ENSMUSG00000096533	0	0	0	0	0	0
ENSMUSG00000096532	0	0	0	0	0	0
ENSMUSG00000096530	0	0	0	0	0	0
ENSMUSG00000110984	0	0	0	0	0	0
ENSMUSG00000044594	0	0	0	0	0	0
ENSMUSG00000096538	0	0	0	0	0	0
ENSMUSG00000107508	1	5	1	1	1	1
ENSMUSG00000105394	0	0	0	0	0	0
ENSMUSG00000060523	0	0	0	0	0	0
ENSMUSG00000045179	0	0	0	0	0	0
ENSMUSG00000045178	0	0	0	0	0	0
ENSMUSG00000088333	0	0	0	0	0	0
ENSMUSG00000045174	0	0	0	0	0	0
ENSMUSG00000109837	0	0	0	0	0	0
ENSMUSG00000026312	0	0	0	0	0	0
ENSMUSG00000057149	3	3	0	0	0	0
ENSMUSG00000033187	0	0	0	0	0	0
ENSMUSG00000113393	0	2	0	1	0	0
ENSMUSG00000113391	3	2	3	2	2	2
ENSMUSG00000059595	0	0	0	0	0	0
ENSMUSG00000088331	0	0	0	0	0	0
ENSMUSG00000001819	0	0	0	0	0	0
ENSMUSG00000113394	0	0	0	0	0	0
ENSMUSG00000114076	0	0	0	0	0	0
ENSMUSG00000112978	0	0	0	0	0	0
ENSMUSG00000042331	428	316	439	176	192	278
ENSMUSG00000077156	0	0	0	0	0	0
ENSMUSG00000093570	0	0	0	0	0	0
ENSMUSG00000094700	0	0	0	0	0	0
ENSMUSG00000093573	0	0	0	0	0	0
ENSMUSG00000094706	0	0	0	0	0	0
ENSMUSG00000094707	10	9	14	0	12	0
ENSMUSG00000094704	0	0	0	0	0	0
ENSMUSG00000094709	0	0	0	1	1	1
ENSMUSG00000099638	0	0	0	0	0	0
ENSMUSG00000092629	0	0	0	0	2	0
ENSMUSG00000092627	6	7	14	3	6	3
ENSMUSG00000099637	0	0	0	0	0	0

ENSMUSG00000092624	0	0	0	0	0	0
ENSMUSG00000104625	5	0	0	5	5	5
ENSMUSG00000092621	0	0	0	0	0	0
ENSMUSG00000092620	0	0	0	0	0	0
ENSMUSG00000004948	0	0	0	0	0	0
ENSMUSG00000112169	7	3	6	0	7	9
ENSMUSG00000101476	4	0	3	0	0	0
ENSMUSG00000112162	8	4	0	0	8	5
ENSMUSG00000112161	0	0	0	0	0	0
ENSMUSG00000055730	3	0	0	0	0	0
ENSMUSG00000112166	0	0	0	0	0	0
ENSMUSG00000055732	0	0	0	0	0	0
ENSMUSG00000112164	0	0	0	0	0	0
ENSMUSG00000029999	50	48	31	20	21	37
ENSMUSG00000102576	0	0	0	0	0	0
ENSMUSG00000101472	0	0	0	0	0	0
ENSMUSG00000068348	0	0	0	0	0	0
ENSMUSG00000101950	0	0	0	0	0	0
ENSMUSG00000077484	0	0	1	1	0	0
ENSMUSG00000077480	0	0	0	0	0	0
ENSMUSG00000077488	2	0	1	0	0	0
ENSMUSG00000093394	6	0	0	0	0	0
ENSMUSG00000093397	0	0	0	0	0	0
ENSMUSG00000065111	0	0	0	0	0	0
ENSMUSG00000086072	0	0	0	0	0	0
ENSMUSG00000086071	0	0	0	0	0	0
ENSMUSG00000086070	0	0	2	0	0	0
ENSMUSG00000086077	0	0	0	0	0	0
ENSMUSG00000086074	3	0	3	1	0	0
ENSMUSG00000098949	0	0	0	0	0	0
ENSMUSG00000049202	0	0	0	2	0	0
ENSMUSG00000098944	0	0	0	0	0	2
ENSMUSG00000098946	1	1	3	0	1	3
ENSMUSG00000098947	0	0	0	1	0	1
ENSMUSG00000076355	0	0	0	0	0	0
ENSMUSG00000076357	0	0	0	0	0	0
ENSMUSG00000082791	1	1	1	1	0	0
ENSMUSG00000082796	0	0	0	0	0	0
ENSMUSG00000082797	0	0	0	0	0	0
ENSMUSG00000082794	4	2	11	0	0	0
ENSMUSG00000082795	0	0	0	0	0	0
ENSMUSG00000082798	0	0	0	0	0	0
ENSMUSG00000082799	0	0	0	0	0	0
ENSMUSG00000045952	0	0	2	0	0	0
ENSMUSG00000037437	22	43	35	8	11	5
ENSMUSG00000004231	0	0	0	0	0	0
ENSMUSG00000087678	15	13	12	59	28	54
ENSMUSG00000075141	0	0	0	0	0	0
ENSMUSG00000109175	3	3	2	0	4	3
ENSMUSG00000087674	0	0	6	0	0	6
ENSMUSG00000087675	0	0	0	0	0	0
ENSMUSG00000070904	0	0	0	0	0	0
ENSMUSG00000070902	0	0	0	0	0	0
ENSMUSG00000070908	2	1	2	4	2	2
ENSMUSG00000071361	0	0	0	0	0	0
ENSMUSG00000057805	0	0	0	0	0	0
ENSMUSG00000057801	0	0	0	0	0	0
ENSMUSG00000057802	28	22	22	19	29	19

ENSMUSG00000109178	3	4	5	4	5	5
ENSMUSG00000082139	0	0	0	0	0	0
ENSMUSG00000084320	3	0	8	0	3	3
ENSMUSG00000084325	32	29	19	23	12	39
ENSMUSG00000084324	0	0	0	0	0	0
ENSMUSG00000084327	0	0	0	0	0	0
ENSMUSG00000084326	0	0	0	0	0	0
ENSMUSG00000084791	0	0	0	0	0	0
ENSMUSG00000025504	72	63	73	67	77	122
ENSMUSG00000025507	57	22	44	20	29	37
ENSMUSG00000099233	0	0	0	0	0	1
ENSMUSG00000099392	0	0	0	0	0	0
ENSMUSG00000099393	0	0	0	0	0	0
ENSMUSG00000099390	0	0	0	0	0	0
ENSMUSG00000099391	0	0	0	0	0	0
ENSMUSG00000099396	0	0	0	0	0	0
ENSMUSG00000099394	0	0	0	0	0	1
ENSMUSG00000099395	0	0	0	0	0	0
ENSMUSG00000099399	0	0	0	0	0	0
ENSMUSG00000099239	0	0	0	0	0	0
ENSMUSG00000065613	0	0	0	0	0	0
ENSMUSG00000065616	0	0	0	0	0	0
ENSMUSG00000065617	1	0	1	1	2	1
ENSMUSG00000032269	0	0	0	0	2	0
ENSMUSG00000032268	3	5	3	0	7	6
ENSMUSG00000110335	2	4	0	0	0	0
ENSMUSG00000032262	15	8	5	6	0	14
ENSMUSG00000062042	0	0	0	0	0	0
ENSMUSG00000062044	0	0	4	0	0	0
ENSMUSG00000062046	0	0	0	0	0	0
ENSMUSG00000081914	0	0	0	0	0	0
ENSMUSG00000081917	0	0	0	0	0	0
ENSMUSG00000081916	0	0	0	0	2	0
ENSMUSG00000081910	0	0	0	0	0	0
ENSMUSG00000081913	0	0	0	0	0	0
ENSMUSG00000081912	0	0	0	0	0	0
ENSMUSG00000087034	8	1	3	3	8	7
ENSMUSG00000110280	0	0	0	0	0	0
ENSMUSG00000084918	4	4	3	4	0	3
ENSMUSG00000102632	16	23	25	17	25	22
ENSMUSG00000098119	0	1	0	0	0	0
ENSMUSG00000098118	0	0	0	0	0	0
ENSMUSG00000098763	0	0	0	0	0	0
ENSMUSG00000110284	0	0	0	0	0	0
ENSMUSG00000098116	0	0	0	0	0	0
ENSMUSG00000098115	0	0	0	0	0	0
ENSMUSG00000098114	0	0	0	0	0	0
ENSMUSG00000037034	0	0	0	0	0	0
ENSMUSG00000087036	20	12	12	4	9	14
ENSMUSG00000083666	0	0	0	0	0	0
ENSMUSG00000032841	91	94	133	38	38	64
ENSMUSG00000032842	192	166	213	170	187	124
ENSMUSG00000035818	0	0	0	7	0	5
ENSMUSG00000035811	0	0	0	0	0	0
ENSMUSG00000032092	8	0	9	3	0	5
ENSMUSG00000110135	0	0	0	0	0	0
ENSMUSG00000103977	0	0	0	0	0	0
ENSMUSG00000103971	0	0	3	0	1	0

ENSMUSG00000103978	0	0	0	0	0	0
ENSMUSG00000034145	29	24	22	7	6	2
ENSMUSG00000038997	0	0	0	0	3	0
ENSMUSG00000056035	0	0	0	0	0	0
ENSMUSG00000089859	0	1	1	0	1	1
ENSMUSG00000056031	7	4	2	0	0	2
ENSMUSG00000086494	0	0	0	0	0	0
ENSMUSG00000014813	97	181	196	74	89	103
ENSMUSG00000086497	0	0	0	0	0	0
ENSMUSG00000091422	0	0	0	0	0	0
ENSMUSG00000091425	0	0	0	0	0	0
ENSMUSG00000086491	0	0	0	0	0	0
ENSMUSG00000018126	0	0	0	0	0	2
ENSMUSG00000093731	0	0	0	0	0	0
ENSMUSG00000086490	2	0	0	0	0	0
ENSMUSG00000101369	0	0	0	0	0	0
ENSMUSG00000086498	0	0	0	0	0	0
ENSMUSG00000108803	0	0	0	0	0	0
ENSMUSG00000108800	0	0	0	0	0	0
ENSMUSG00000108805	0	0	0	0	0	0
ENSMUSG00000108804	0	0	0	0	0	0
ENSMUSG00000108809	0	0	0	0	0	0
ENSMUSG00000067561	0	0	0	0	0	0
ENSMUSG00000105988	0	0	0	0	0	0
ENSMUSG00000044912	0	0	0	0	0	0
ENSMUSG00000051228	11	27	30	30	15	18
ENSMUSG00000048062	0	0	3	0	0	0
ENSMUSG00000099812	0	0	0	5	3	8
ENSMUSG00000101608	0	0	0	0	0	0
ENSMUSG00000101606	0	0	0	0	0	0
ENSMUSG00000101607	0	0	0	0	0	0
ENSMUSG00000101605	0	0	0	0	0	0
ENSMUSG00000101602	0	0	0	0	0	0
ENSMUSG00000101600	0	0	0	0	0	0
ENSMUSG00000060030	0	0	0	0	0	0
ENSMUSG00000085806	0	0	0	0	0	0
ENSMUSG00000085805	0	0	0	0	0	0
ENSMUSG00000085804	0	0	0	0	0	0
ENSMUSG00000085803	0	0	0	0	0	0
ENSMUSG00000085801	0	0	0	0	0	0
ENSMUSG00000085808	0	0	0	0	0	0
ENSMUSG00000061928	0	0	0	0	0	0
ENSMUSG00000061923	0	0	0	0	0	0
ENSMUSG00000099653	0	0	0	0	0	0
ENSMUSG00000087531	0	0	0	0	0	0
ENSMUSG00000087534	0	0	0	0	0	0
ENSMUSG00000087537	0	0	0	0	0	0
ENSMUSG00000087536	0	0	0	0	0	0
ENSMUSG00000087539	0	0	0	0	0	0
ENSMUSG00000063380	0	0	0	0	0	0
ENSMUSG00000101247	0	0	0	0	0	0
ENSMUSG00000107609	0	0	1	0	1	0
ENSMUSG00000107601	0	0	0	0	0	0
ENSMUSG00000107602	0	0	0	0	0	0
ENSMUSG00000107605	13	16	4	26	19	15
ENSMUSG00000089358	0	0	0	0	0	0
ENSMUSG00000056648	0	0	0	0	0	0
ENSMUSG00000103110	0	0	0	0	0	0

ENSMUSG00000103111	0	0	0	0	0	0
ENSMUSG00000103112	0	0	0	0	0	0
ENSMUSG00000036172	29	24	7	4	4	4
ENSMUSG00000103114	0	6	0	4	0	9
ENSMUSG00000103115	24	16	13	12	12	12
ENSMUSG00000103116	0	0	0	0	0	2
ENSMUSG00000101245	0	0	0	0	0	0
ENSMUSG00000095895	0	2	4	0	4	0
ENSMUSG00000095894	0	1	2	2	1	1
ENSMUSG00000095896	0	0	0	0	0	0
ENSMUSG00000095890	0	0	0	0	0	0
ENSMUSG00000095893	0	0	0	0	0	0
ENSMUSG00000095899	0	0	0	0	0	0
ENSMUSG00000095898	0	0	0	0	0	0
ENSMUSG00000065555	0	1	3	0	1	2
ENSMUSG00000065551	1	0	0	0	0	0
ENSMUSG00000055609	0	0	0	0	0	0
ENSMUSG00000065559	0	0	0	0	0	0
ENSMUSG00000022501	0	0	0	0	0	0
ENSMUSG00000108138	0	0	0	0	0	0
ENSMUSG00000089078	0	0	0	0	0	0
ENSMUSG00000089079	0	0	0	0	0	0
ENSMUSG00000089077	0	0	0	0	0	0
ENSMUSG00000089075	0	0	0	0	0	0
ENSMUSG00000089073	0	0	0	0	0	0
ENSMUSG00000089071	0	0	1	0	0	0
ENSMUSG00000102360	0	0	2	0	0	0
ENSMUSG00000050577	4	0	0	0	0	0
ENSMUSG00000105948	0	0	0	0	0	0
ENSMUSG00000050578	8	24	51	14	43	7
ENSMUSG00000102365	6	3	5	3	3	0
ENSMUSG00000112361	2	6	0	0	4	0
ENSMUSG00000112360	0	0	0	0	0	0
ENSMUSG00000112363	0	2	0	0	0	0
ENSMUSG00000102367	2	0	0	0	0	0
ENSMUSG00000112365	0	0	0	0	0	0
ENSMUSG00000094277	0	0	0	0	0	0
ENSMUSG00000094274	0	0	0	0	0	0
ENSMUSG00000112366	3	0	0	0	0	0
ENSMUSG00000102646	0	2	0	0	0	2
ENSMUSG00000102643	0	0	0	0	0	0
ENSMUSG00000102641	0	0	0	0	0	0
ENSMUSG00000087940	0	0	0	0	0	0
ENSMUSG00000024901	82	90	81	56	93	55
ENSMUSG00000102648	0	0	0	0	0	0
ENSMUSG00000098258	0	0	0	0	0	0
ENSMUSG00000062528	0	0	0	0	0	0
ENSMUSG00000113098	0	0	0	0	0	0
ENSMUSG00000110212	0	0	0	0	0	0
ENSMUSG00000110211	0	0	0	0	0	0
ENSMUSG00000110210	0	0	0	0	0	0
ENSMUSG00000110217	0	0	0	0	0	0
ENSMUSG00000110216	7	0	0	0	7	0
ENSMUSG00000110215	0	0	0	0	0	0
ENSMUSG00000110214	0	0	0	0	0	0
ENSMUSG00000113090	0	0	0	0	0	0
ENSMUSG00000113091	1	0	0	0	0	0
ENSMUSG00000113092	0	0	0	0	0	0

ENSMUSG00000113093	0	0	0	0	0	0
ENSMUSG00000028590	0	0	0	0	0	0
ENSMUSG00000113095	0	0	0	0	0	0
ENSMUSG00000113096	0	0	0	0	0	0
ENSMUSG00000113097	0	0	0	0	0	0
ENSMUSG00000097670	0	0	0	0	0	0
ENSMUSG00000035735	198	126	224	77	89	98
ENSMUSG00000105516	0	0	0	0	0	0
ENSMUSG00000105515	0	0	0	0	0	0
ENSMUSG00000087575	0	0	0	0	0	0
ENSMUSG00000107084	0	0	0	0	0	0
ENSMUSG00000105511	0	0	0	0	0	0
ENSMUSG00000088846	0	0	0	0	0	0
ENSMUSG00000088840	0	0	0	0	0	0
ENSMUSG00000088842	0	0	0	0	0	0
ENSMUSG00000069324	0	0	0	1	0	0
ENSMUSG00000094858	0	0	0	0	0	0
ENSMUSG00000021749	2	0	0	0	0	0
ENSMUSG00000094856	0	0	0	0	1	1
ENSMUSG00000021747	98	115	61	100	100	163
ENSMUSG00000094854	0	0	0	0	0	0
ENSMUSG00000094855	0	0	0	0	0	0
ENSMUSG00000094852	0	2	0	0	3	2
ENSMUSG00000021743	0	0	0	0	4	0
ENSMUSG00000094850	0	0	0	0	0	0
ENSMUSG00000095213	0	0	0	0	0	0
ENSMUSG00000083455	0	0	0	0	0	0
ENSMUSG00000066319	36	33	43	27	26	35
ENSMUSG00000082802	0	0	0	0	0	0
ENSMUSG00000082807	0	0	0	0	0	0
ENSMUSG00000113542	0	4	0	0	0	0
ENSMUSG00000113543	0	0	0	0	0	0
ENSMUSG00000038760	0	0	0	3	0	0
ENSMUSG00000113541	2	0	1	0	0	0
ENSMUSG00000113546	0	0	0	0	0	0
ENSMUSG00000113547	0	0	0	0	0	0
ENSMUSG00000034467	0	0	0	0	0	0
ENSMUSG00000038765	0	0	0	0	0	0
ENSMUSG00000038768	3	0	0	0	0	0
ENSMUSG00000113549	2	0	0	1	0	0
ENSMUSG00000055972	20	22	17	24	27	9
ENSMUSG00000055971	0	0	0	0	0	0
ENSMUSG00000055976	8	6	8	6	5	2
ENSMUSG00000114011	0	0	0	0	0	0
ENSMUSG00000114010	0	0	0	0	0	0
ENSMUSG00000114013	0	0	0	0	0	0
ENSMUSG00000114012	0	0	0	0	0	2
ENSMUSG00000114015	0	0	0	0	0	0
ENSMUSG00000114017	0	0	0	0	0	0
ENSMUSG00000086462	0	0	0	0	0	0
ENSMUSG00000114018	4	0	18	0	0	3
ENSMUSG00000065619	0	0	0	0	0	0
ENSMUSG00000059626	0	0	0	0	0	0
ENSMUSG00000059625	0	0	0	0	0	0
ENSMUSG00000059623	0	0	0	0	0	0
ENSMUSG00000101407	0	0	0	0	0	0
ENSMUSG00000091987	0	0	0	0	0	0
ENSMUSG00000087330	0	0	0	0	0	0

ENSMUSG00000091983	26	6	31	5	33	9
ENSMUSG00000091980	0	0	0	0	0	0
ENSMUSG00000087337	0	0	0	0	0	0
ENSMUSG00000067047	0	0	0	0	0	0
ENSMUSG00000067049	1	0	0	0	0	0
ENSMUSG00000089114	0	0	0	0	0	0
ENSMUSG00000020598	26	35	37	7	22	12
ENSMUSG00000020599	11	0	14	3	4	0
ENSMUSG00000054594	13	0	4	14	19	16
ENSMUSG00000075485	0	0	0	0	0	0
ENSMUSG00000104397	0	0	0	0	0	2
ENSMUSG00000104396	1	0	0	0	0	2
ENSMUSG00000104395	0	0	0	0	0	0
ENSMUSG00000104393	0	0	0	0	0	0
ENSMUSG00000054598	0	2	0	0	0	3
ENSMUSG00000083160	0	0	0	0	0	0
ENSMUSG00000056995	3	3	0	0	0	0
ENSMUSG00000049410	0	0	8	7	0	0
ENSMUSG00000026295	0	0	0	0	0	0
ENSMUSG00000083651	0	0	0	0	0	0
ENSMUSG00000098333	0	0	0	0	0	0
ENSMUSG00000113857	0	0	0	0	0	0
ENSMUSG00000027748	0	0	4	6	10	19
ENSMUSG00000027744	0	6	0	0	0	0
ENSMUSG00000098336	0	0	0	0	0	0
ENSMUSG00000089111	1	0	0	0	0	0
ENSMUSG00000046300	0	0	0	0	0	0
ENSMUSG00000092988	0	0	0	0	0	1
ENSMUSG00000042817	25	13	73	0	4	0
ENSMUSG00000042816	19	20	25	20	12	31
ENSMUSG00000089113	0	0	2	0	0	1
ENSMUSG00000080511	0	0	0	0	0	0
ENSMUSG00000080519	0	0	0	0	0	0
ENSMUSG00000074802	321	400	388	160	225	206
ENSMUSG00000074804	0	0	4	3	1	2
ENSMUSG00000090166	0	0	0	0	0	0
ENSMUSG00000098358	0	0	0	0	0	0
ENSMUSG00000090162	0	0	0	0	0	0
ENSMUSG00000098351	0	0	0	0	0	0
ENSMUSG00000098354	0	0	0	0	0	0
ENSMUSG00000098357	0	1	0	0	1	1
ENSMUSG00000098356	0	1	0	0	0	0
ENSMUSG00000111965	0	0	0	0	0	0
ENSMUSG00000006179	0	0	0	0	0	0
ENSMUSG00000114498	0	3	0	0	3	4
ENSMUSG00000086917	0	0	0	0	0	0
ENSMUSG00000086910	0	0	0	0	0	0
ENSMUSG00000086911	0	0	0	0	0	0
ENSMUSG00000086912	0	0	0	0	0	0
ENSMUSG00000086913	0	0	0	0	0	0
ENSMUSG00000095662	1	2	2	2	1	2
ENSMUSG00000095663	4	6	3	1	0	0
ENSMUSG00000090292	0	0	0	0	0	1
ENSMUSG00000095661	0	1	1	0	0	0
ENSMUSG00000095666	0	0	0	0	0	0
ENSMUSG00000095667	0	0	0	0	0	0
ENSMUSG00000095664	0	0	0	0	0	0
ENSMUSG00000090298	0	0	0	0	0	0

ENSMUSG00000094482	0	0	0	0	0	0
ENSMUSG00000039578	117	127	135	116	119	138
ENSMUSG00000100567	0	0	0	0	0	0
ENSMUSG00000021538	0	0	0	0	0	0
ENSMUSG00000053909	0	0	0	0	0	0
ENSMUSG00000094131	0	0	1	2	0	0
ENSMUSG00000094133	0	0	0	0	0	0
ENSMUSG00000094132	3	5	4	0	0	7
ENSMUSG00000094135	0	0	0	0	0	0
ENSMUSG00000042102	0	0	0	0	0	0
ENSMUSG00000094137	0	0	0	0	0	0
ENSMUSG00000094488	0	0	0	0	0	0
ENSMUSG00000094139	0	0	0	0	0	0
ENSMUSG00000081641	0	0	0	0	0	0
ENSMUSG00000095310	0	0	0	0	0	0
ENSMUSG00000093451	0	0	0	0	0	0
ENSMUSG00000081647	0	0	0	0	0	0
ENSMUSG00000081646	0	0	0	0	0	0
ENSMUSG00000081649	0	0	0	0	0	0
ENSMUSG00000077728	0	0	0	0	0	0
ENSMUSG00000100797	0	0	0	0	0	0
ENSMUSG00000049902	0	0	0	0	0	0
ENSMUSG00000107162	0	0	0	0	0	0
ENSMUSG00000057654	2	1	5	4	1	2
ENSMUSG00000063251	0	0	0	0	0	0
ENSMUSG00000090839	0	0	0	0	0	0
ENSMUSG00000100792	0	0	0	0	0	0
ENSMUSG00000100791	0	0	0	0	0	0
ENSMUSG00000092476	0	0	0	0	0	0
ENSMUSG00000092477	0	0	0	0	0	0
ENSMUSG00000092473	0	0	0	0	0	0
ENSMUSG00000101644	0	0	0	0	0	0
ENSMUSG00000046486	0	0	0	0	0	0
ENSMUSG00000043472	0	0	0	0	0	0
ENSMUSG00000030752	185	152	136	200	205	207
ENSMUSG00000030757	23	5	26	19	32	14
ENSMUSG00000100799	0	0	0	0	0	0
ENSMUSG00000103626	0	0	0	0	0	0
ENSMUSG00000112749	0	0	0	0	0	0
ENSMUSG00000082344	0	0	0	0	0	0
ENSMUSG00000082340	0	0	0	0	0	0
ENSMUSG00000082342	6	2	2	7	6	12
ENSMUSG00000101773	0	0	0	0	0	0
ENSMUSG00000101770	0	0	0	0	0	0
ENSMUSG00000101771	0	0	0	0	0	0
ENSMUSG00000005883	4	3	0	3	0	7
ENSMUSG00000098484	0	0	0	0	0	0
ENSMUSG00000101853	0	0	0	0	0	0
ENSMUSG00000032446	0	0	0	0	0	0
ENSMUSG00000098481	0	0	0	0	0	0
ENSMUSG00000072774	211	191	227	123	154	137
ENSMUSG00000087737	0	0	0	0	0	1
ENSMUSG00000087736	0	0	0	0	0	0
ENSMUSG00000087732	0	0	0	0	0	0
ENSMUSG00000070732	2	0	7	7	5	2
ENSMUSG00000084428	0	0	0	0	0	0
ENSMUSG00000083438	0	0	0	0	0	0
ENSMUSG00000083439	2	4	5	2	3	2

ENSMUSG00000083434	0	0	0	0	0	0
ENSMUSG00000083436	0	0	0	0	0	0
ENSMUSG00000083437	0	0	0	0	0	0
ENSMUSG00000084426	0	0	0	0	0	0
ENSMUSG00000083431	0	0	0	0	0	0
ENSMUSG00000084425	0	0	0	0	0	0
ENSMUSG00000049015	0	5	0	0	0	0
ENSMUSG00000049011	0	0	0	0	0	0
ENSMUSG00000079516	0	0	0	0	0	0
ENSMUSG00000079513	0	0	0	0	0	0
ENSMUSG00000106530	0	0	0	0	0	0
ENSMUSG00000087880	0	0	0	0	0	0
ENSMUSG00000031936	16	16	81	0	0	3
ENSMUSG00000031937	11	14	44	4	8	4
ENSMUSG00000106533	0	0	0	0	0	0
ENSMUSG00000031932	2	0	6	0	6	0
ENSMUSG00000079519	0	0	0	0	0	0
ENSMUSG00000094698	0	0	0	0	0	0
ENSMUSG00000106532	0	0	0	0	0	0
ENSMUSG00000085770	0	0	0	0	0	0
ENSMUSG00000085773	0	0	0	0	0	0
ENSMUSG00000085772	25	43	46	7	19	5
ENSMUSG00000085775	1	0	0	0	0	0
ENSMUSG00000085774	0	0	0	0	3	0
ENSMUSG00000064044	0	0	0	0	0	0
ENSMUSG00000066756	0	0	0	0	0	0
ENSMUSG00000107447	0	0	0	0	0	3
ENSMUSG00000107445	0	0	0	0	0	0
ENSMUSG00000107443	1	4	1	2	1	2
ENSMUSG00000033470	5	4	0	0	0	0
ENSMUSG00000065795	0	0	0	0	0	0
ENSMUSG00000065794	0	0	0	0	0	0
ENSMUSG00000065797	0	0	0	0	0	0
ENSMUSG00000065791	0	0	0	0	0	0
ENSMUSG00000065792	0	0	0	0	0	0
ENSMUSG00000094151	0	0	0	0	0	0
ENSMUSG00000094150	0	0	0	0	0	0
ENSMUSG00000094156	0	0	0	0	0	0
ENSMUSG00000036045	0	0	0	0	0	0
ENSMUSG00000101760	0	0	0	0	0	0
ENSMUSG00000094155	0	0	0	0	0	0
ENSMUSG00000084289	0	0	0	0	0	0
ENSMUSG00000084286	0	0	0	0	0	0
ENSMUSG00000084287	0	0	0	0	0	0
ENSMUSG00000084285	0	0	0	0	0	0
ENSMUSG00000084282	0	0	0	0	0	0
ENSMUSG00000086937	0	0	0	0	0	0
ENSMUSG00000068011	32	42	20	33	35	31
ENSMUSG00000092866	0	0	0	0	0	0
ENSMUSG00000085483	0	5	8	6	0	0
ENSMUSG00000100421	0	0	0	0	0	0
ENSMUSG00000085489	0	0	0	0	0	0
ENSMUSG00000085488	0	0	0	0	0	0
ENSMUSG00000100423	0	0	0	0	0	0
ENSMUSG00000001763	36	41	48	53	42	45
ENSMUSG00000100422	0	0	0	0	0	0
ENSMUSG00000100429	0	0	0	0	0	0
ENSMUSG00000100428	0	0	0	0	0	0

ENSMUSG00000086939	0	0	0	0	0	0
ENSMUSG00000114424	0	0	10	0	0	3
ENSMUSG00000110539	0	0	0	0	0	0
ENSMUSG00000076376	5	3	4	1	1	3
ENSMUSG00000103424	0	2	0	0	0	0
ENSMUSG00000103399	0	2	0	1	4	2
ENSMUSG00000103425	0	0	0	0	0	0
ENSMUSG00000106410	0	0	2	1	3	0
ENSMUSG00000114426	0	0	0	0	0	0
ENSMUSG00000073413	8	9	15	10	8	13
ENSMUSG00000102250	0	0	0	0	0	0
ENSMUSG00000102251	0	0	0	0	0	0
ENSMUSG00000092517	0	4	18	5	0	5
ENSMUSG00000102255	0	0	0	0	0	0
ENSMUSG00000102256	0	0	0	0	0	0
ENSMUSG00000022849	161	129	169	135	119	144
ENSMUSG00000038560	0	0	0	0	0	0
ENSMUSG00000101768	0	6	4	0	0	0
ENSMUSG00000083839	0	0	0	0	0	0
ENSMUSG00000096743	0	0	0	0	0	0
ENSMUSG00000096744	0	0	0	0	0	0
ENSMUSG00000096748	0	0	1	0	0	2
ENSMUSG00000096749	0	0	0	0	0	0
ENSMUSG00000031169	105	125	159	86	104	129
ENSMUSG00000114503	6	9	5	9	3	5
ENSMUSG00000050702	0	2	3	0	3	0
ENSMUSG00000031162	0	0	3	0	0	0
ENSMUSG00000050700	24	11	20	0	11	7
ENSMUSG00000031160	0	0	0	0	0	0
ENSMUSG00000050704	0	0	0	0	0	0
ENSMUSG00000083835	0	0	0	0	0	0
ENSMUSG00000063106	1	0	0	0	0	0
ENSMUSG00000067684	0	0	0	0	0	0
ENSMUSG00000102870	4	2	0	0	2	3
ENSMUSG00000102872	0	0	0	0	0	0
ENSMUSG00000102878	0	0	0	0	0	0
ENSMUSG00000018776	0	0	5	0	5	6
ENSMUSG00000097740	7	8	8	28	21	35
ENSMUSG00000104158	0	0	0	0	0	0
ENSMUSG00000095960	0	0	0	0	0	0
ENSMUSG00000104155	0	0	0	0	0	0
ENSMUSG00000104157	0	0	2	2	0	0
ENSMUSG00000089768	6	6	2	4	6	0
ENSMUSG00000104152	0	0	0	0	0	0
ENSMUSG00000111716	0	0	0	0	0	0
ENSMUSG00000059708	333	385	372	277	333	344
ENSMUSG00000091212	0	0	0	0	0	0
ENSMUSG00000010505	0	0	0	0	2	0
ENSMUSG00000101185	0	0	0	0	0	0
ENSMUSG00000060480	0	0	0	0	0	0
ENSMUSG00000111438	1	0	4	0	0	3
ENSMUSG00000056468	11	5	4	8	8	21
ENSMUSG00000110821	0	0	0	0	0	0
ENSMUSG00000110823	0	0	0	0	0	0
ENSMUSG00000110827	18	20	13	25	27	22
ENSMUSG00000110829	0	0	0	0	0	0
ENSMUSG00000104849	0	0	0	0	0	0
ENSMUSG00000104843	0	0	0	0	0	0

ENSMUSG00000104841	4	2	0	0	0	2
ENSMUSG00000104840	0	0	0	0	0	0
ENSMUSG00000104845	0	0	0	0	0	0
ENSMUSG00000097604	0	2	6	0	0	5
ENSMUSG00000088264	0	0	0	0	0	0
ENSMUSG00000088266	0	0	0	0	0	0
ENSMUSG00000094211	0	0	0	0	0	0
ENSMUSG00000058078	0	0	0	0	0	0
ENSMUSG00000088261	0	0	0	0	0	0
ENSMUSG00000058071	0	0	0	0	0	0
ENSMUSG00000027400	0	0	0	0	0	0
ENSMUSG00000020722	4	5	11	0	0	8
ENSMUSG00000013367	0	0	10	4	5	0
ENSMUSG00000097602	4	1	2	1	0	1
ENSMUSG00000022696	7	20	5	27	30	26
ENSMUSG00000083205	0	0	0	0	0	0
ENSMUSG00000095462	0	0	0	0	1	0
ENSMUSG00000084256	0	0	0	0	0	0
ENSMUSG00000095465	0	0	0	3	0	0
ENSMUSG00000095466	0	0	0	0	0	0
ENSMUSG00000095467	0	0	0	0	0	0
ENSMUSG00000095469	0	0	0	0	0	0
ENSMUSG00000083201	0	0	0	0	0	0
ENSMUSG00000100161	0	0	0	0	0	0
ENSMUSG00000025219	0	0	0	0	0	0
ENSMUSG00000095939	0	0	0	0	0	0
ENSMUSG00000022715	0	0	0	0	0	0
ENSMUSG00000095806	0	0	0	0	0	0
ENSMUSG00000093108	0	0	0	0	0	0
ENSMUSG00000093105	1	0	1	0	2	1
ENSMUSG00000094336	8	5	7	7	5	10
ENSMUSG00000093101	0	0	0	0	0	0
ENSMUSG00000112269	0	0	0	0	0	0
ENSMUSG00000093102	0	0	0	0	0	0
ENSMUSG00000036322	0	0	0	0	0	0
ENSMUSG00000002055	71	64	91	2	30	67
ENSMUSG00000002057	0	0	0	0	0	2
ENSMUSG00000097823	22	28	35	32	24	27
ENSMUSG00000097821	0	0	0	0	0	0
ENSMUSG00000097824	0	0	0	0	0	0
ENSMUSG00000097825	0	0	0	0	0	1
ENSMUSG00000109235	0	0	0	0	0	0
ENSMUSG00000112539	0	0	0	0	0	0
ENSMUSG00000041247	0	0	0	0	0	0
ENSMUSG00000055341	35	43	43	45	44	65
ENSMUSG00000112531	0	0	0	0	0	0
ENSMUSG00000112532	6	7	5	2	0	5
ENSMUSG00000112533	8	20	12	0	4	25
ENSMUSG00000112534	0	0	0	0	0	0
ENSMUSG00000039315	0	2	4	0	0	0
ENSMUSG00000112536	0	0	0	0	0	2
ENSMUSG00000112537	0	0	0	0	0	0
ENSMUSG00000106148	0	0	0	0	0	0
ENSMUSG00000106144	5	6	9	4	6	10
ENSMUSG00000106146	2	0	4	0	2	4
ENSMUSG00000106140	27	34	20	44	52	49
ENSMUSG00000106141	0	0	0	0	0	0
ENSMUSG00000106143	0	0	0	0	0	0

ENSMUSG00000028417	12	16	0	7	12	17
ENSMUSG00000092275	0	0	0	0	0	0
ENSMUSG00000092276	0	0	0	0	0	0
ENSMUSG00000092270	1	2	0	1	2	1
ENSMUSG00000092271	0	0	0	0	0	0
ENSMUSG00000092272	0	0	0	0	0	0
ENSMUSG00000109649	0	0	0	0	0	0
ENSMUSG00000071952	0	0	0	0	0	0
ENSMUSG00000090667	9	4	0	0	0	0
ENSMUSG00000090225	0	0	0	0	0	0
ENSMUSG00000101347	0	0	0	0	0	0
ENSMUSG00000090227	0	0	0	0	0	0
ENSMUSG00000035678	16	20	10	16	9	6
ENSMUSG00000109643	0	0	3	0	0	0
ENSMUSG00000020627	212	149	184	24	30	36
ENSMUSG00000020624	0	0	0	0	0	3
ENSMUSG00000020622	0	0	4	0	0	0
ENSMUSG00000110002	0	0	0	0	0	0
ENSMUSG00000110003	1	0	0	0	0	0
ENSMUSG00000110000	0	0	0	0	0	0
ENSMUSG00000110006	0	0	0	0	0	0
ENSMUSG00000110004	0	0	0	0	0	0
ENSMUSG00000110005	0	0	0	0	0	0
ENSMUSG00000110008	5	8	0	6	5	2
ENSMUSG00000113667	0	0	0	0	0	0
ENSMUSG00000113666	0	0	0	0	3	0
ENSMUSG00000113664	2	1	1	3	1	1
ENSMUSG00000113663	0	2	0	0	0	0
ENSMUSG00000105147	0	0	0	0	0	0
ENSMUSG00000113661	0	0	0	0	0	0
ENSMUSG00000113669	8	18	21	2	2	0
ENSMUSG00000022085	0	0	6	0	0	0
ENSMUSG00000109138	0	0	0	0	0	0
ENSMUSG00000109139	0	0	0	0	0	0
ENSMUSG00000105493	0	0	0	0	0	0
ENSMUSG00000105492	0	0	0	0	0	0
ENSMUSG00000105490	0	0	0	0	0	0
ENSMUSG00000096883	0	11	0	0	3	0
ENSMUSG00000046974	0	0	0	0	0	0
ENSMUSG00000046975	0	0	0	0	2	0
ENSMUSG00000096885	0	0	0	1	0	0
ENSMUSG00000046971	0	0	0	0	0	0
ENSMUSG00000104971	0	0	0	0	0	0
ENSMUSG00000043453	0	0	0	0	0	0
ENSMUSG00000025396	0	0	0	0	0	0
ENSMUSG00000104976	0	0	0	0	0	0
ENSMUSG00000070533	2	0	0	0	0	0
ENSMUSG00000070532	0	0	0	0	0	0
ENSMUSG00000070531	0	0	0	0	0	0
ENSMUSG00000070530	0	0	0	0	0	0
ENSMUSG00000114249	0	0	0	0	2	0
ENSMUSG00000054128	0	0	0	0	0	0
ENSMUSG00000105725	0	0	0	0	0	0
ENSMUSG00000105727	0	0	0	0	0	0
ENSMUSG00000105726	20	21	28	26	32	37
ENSMUSG00000105720	36	37	22	38	26	45
ENSMUSG00000105723	0	0	0	0	0	0
ENSMUSG00000105722	0	0	0	0	0	0

ENSMUSG00000105729	5	0	4	0	0	0
ENSMUSG00000096225	0	0	0	0	0	0
ENSMUSG00000096223	0	0	0	0	0	0
ENSMUSG00000096222	0	1	0	0	0	0
ENSMUSG00000094575	0	0	0	0	0	0
ENSMUSG00000096229	0	0	0	0	1	0
ENSMUSG00000096228	0	0	0	0	0	0
ENSMUSG00000080566	0	0	0	0	0	0
ENSMUSG00000111753	0	0	0	0	0	0
ENSMUSG00000111752	0	0	0	0	0	0
ENSMUSG00000111755	0	0	0	0	0	0
ENSMUSG00000111754	0	0	0	0	0	0
ENSMUSG00000100278	0	0	0	0	0	0
ENSMUSG00000104454	54	27	46	39	25	32
ENSMUSG00000104456	0	0	0	0	0	0
ENSMUSG00000112476	0	0	0	0	0	0
ENSMUSG00000108770	0	0	0	0	0	0
ENSMUSG00000108773	0	0	0	0	0	0
ENSMUSG00000108779	0	0	0	1	0	0
ENSMUSG00000108778	0	0	0	0	0	4
ENSMUSG00000100274	0	0	0	0	0	0
ENSMUSG00000112953	0	0	0	0	0	0
ENSMUSG00000108512	0	0	0	0	0	0
ENSMUSG00000112956	0	0	1	0	0	0
ENSMUSG00000109436	0	6	4	0	0	7
ENSMUSG00000108517	0	0	0	0	0	0
ENSMUSG00000001566	0	0	0	0	3	0
ENSMUSG00000082270	0	0	0	0	0	0
ENSMUSG00000110587	0	0	0	0	0	0
ENSMUSG00000110585	0	3	1	2	0	2
ENSMUSG00000110583	0	0	0	0	0	0
ENSMUSG00000110582	0	0	0	0	0	0
ENSMUSG00000027530	0	0	0	0	0	0
ENSMUSG00000102110	0	0	0	0	0	0
ENSMUSG00000071489	0	0	0	0	0	0
ENSMUSG00000020913	0	0	0	0	0	0
ENSMUSG00000090315	0	0	0	0	0	0
ENSMUSG00000095146	0	0	0	0	0	0
ENSMUSG00000048978	0	0	0	0	0	0
ENSMUSG00000095141	0	0	0	0	0	0
ENSMUSG00000095140	0	0	1	0	0	0
ENSMUSG00000090318	0	0	0	0	0	1
ENSMUSG00000095149	0	0	0	0	0	0
ENSMUSG00000095148	0	0	0	0	0	0
ENSMUSG00000042604	222	164	195	211	190	172
ENSMUSG00000046119	1	0	2	0	0	0
ENSMUSG00000080495	0	0	0	0	0	0
ENSMUSG00000080499	6	6	4	2	1	6
ENSMUSG00000080498	0	0	0	0	0	0
ENSMUSG00000112340	3	7	4	10	4	14
ENSMUSG00000051198	0	0	0	0	0	0
ENSMUSG00000086892	0	0	0	0	0	0
ENSMUSG00000099253	0	0	0	0	0	3
ENSMUSG00000059256	0	0	0	0	0	0
ENSMUSG00000052151	120	89	151	36	60	51
ENSMUSG00000052157	65	56	58	52	41	75
ENSMUSG00000094431	0	1	0	0	0	0
ENSMUSG00000080720	9	6	11	7	11	8

ENSMUSG00000080722	0	0	0	0	0	0
ENSMUSG00000080725	0	0	0	0	0	0
ENSMUSG00000080726	0	0	0	0	0	0
ENSMUSG00000080729	0	0	0	0	0	0
ENSMUSG00000080728	0	0	0	0	0	0
ENSMUSG00000096084	0	0	0	0	0	0
ENSMUSG00000093664	0	0	0	0	0	0
ENSMUSG00000093665	0	0	0	0	0	0
ENSMUSG00000093666	0	0	0	0	0	0
ENSMUSG00000093660	1	1	0	0	0	0
ENSMUSG00000093662	0	0	0	0	0	0
ENSMUSG00000093663	0	0	0	0	0	0
ENSMUSG00000078840	1	0	0	0	0	0
ENSMUSG00000093669	0	0	0	0	0	0
ENSMUSG00000086765	6	0	4	13	4	15
ENSMUSG00000086764	0	0	0	0	0	0
ENSMUSG00000020890	0	0	0	0	0	0
ENSMUSG00000098678	0	2	8	0	7	4
ENSMUSG00000098679	1	2	2	1	0	2
ENSMUSG00000031364	0	0	0	0	0	0
ENSMUSG00000077397	1	0	0	0	0	0
ENSMUSG00000047643	16	17	20	7	13	14
ENSMUSG00000047642	0	0	0	0	0	3
ENSMUSG00000047641	0	0	0	0	0	0
ENSMUSG00000041583	0	0	0	0	0	0
ENSMUSG00000099257	0	0	0	0	0	0
ENSMUSG00000077398	0	0	0	0	0	0
ENSMUSG00000077399	0	0	0	0	0	0
ENSMUSG00000096558	0	0	0	0	0	0
ENSMUSG00000033213	13	25	33	4	2	6
ENSMUSG00000049737	0	0	0	0	0	0
ENSMUSG00000033210	0	0	0	0	0	0
ENSMUSG00000033214	6	0	0	0	3	0
ENSMUSG00000030091	193	215	280	220	232	271
ENSMUSG00000033219	0	0	0	0	0	0
ENSMUSG00000030093	0	0	0	0	0	0
ENSMUSG00000043165	0	0	0	0	0	0
ENSMUSG00000099255	1	2	1	2	2	2
ENSMUSG00000082336	2	2	1	7	3	0
ENSMUSG00000076448	0	0	0	0	0	0
ENSMUSG00000099781	0	0	0	0	0	0
ENSMUSG00000099780	0	0	0	0	0	0
ENSMUSG00000099783	0	0	0	0	0	0
ENSMUSG00000099782	0	0	0	0	0	0
ENSMUSG00000099785	0	0	0	0	0	0
ENSMUSG00000099784	2	0	0	0	0	0
ENSMUSG00000099787	0	0	0	0	0	0
ENSMUSG00000099786	0	0	0	0	0	0
ENSMUSG00000099789	0	0	0	0	0	0
ENSMUSG00000105680	0	0	0	0	0	0
ENSMUSG00000101487	0	0	0	0	0	0
ENSMUSG00000030543	0	0	0	0	0	0
ENSMUSG00000087388	0	0	0	0	0	0
ENSMUSG00000030544	0	0	0	0	2	0
ENSMUSG00000087382	14	7	8	10	11	8
ENSMUSG00000087383	0	0	0	0	0	0
ENSMUSG00000030549	0	0	0	0	2	0
ENSMUSG00000099258	0	0	0	0	0	0

ENSMUSG00000083603	2	5	0	0	5	3
ENSMUSG00000083602	0	0	0	0	0	0
ENSMUSG00000083601	0	0	0	0	0	0
ENSMUSG00000082333	0	0	0	0	0	0
ENSMUSG00000100726	0	0	0	0	0	0
ENSMUSG00000100727	0	0	0	0	0	0
ENSMUSG00000100724	0	0	0	0	0	0
ENSMUSG00000100722	0	0	0	0	0	0
ENSMUSG00000100723	0	0	0	0	0	0
ENSMUSG00000083611	0	0	0	0	0	0
ENSMUSG00000100721	0	0	0	0	0	0
ENSMUSG00000100728	0	0	0	0	0	0
ENSMUSG00000102470	0	0	0	0	0	0
ENSMUSG00000102473	0	0	0	3	0	0
ENSMUSG00000102478	42	28	40	6	29	29
ENSMUSG00000102479	0	0	0	0	0	0
ENSMUSG00000106480	0	0	0	0	0	0
ENSMUSG00000075139	0	0	0	0	0	0
ENSMUSG00000075136	0	0	0	0	0	0
ENSMUSG00000075137	0	0	0	0	0	0
ENSMUSG00000079495	0	0	0	0	0	6
ENSMUSG00000079494	0	0	0	0	0	0
ENSMUSG00000088970	0	0	0	0	0	0
ENSMUSG00000041399	0	0	0	0	0	0
ENSMUSG00000088971	0	0	0	0	0	0
ENSMUSG00000087967	0	0	0	0	0	0
ENSMUSG00000087964	0	0	0	0	0	0
ENSMUSG00000087960	0	0	0	0	0	0
ENSMUSG00000087961	0	0	0	0	0	0
ENSMUSG00000085528	0	0	0	0	0	0
ENSMUSG00000108234	5	5	7	2	5	6
ENSMUSG00000088973	3	0	0	1	1	1
ENSMUSG00000094069	0	0	0	0	0	0
ENSMUSG00000111984	0	0	0	0	0	0
ENSMUSG00000088975	0	0	0	0	0	0
ENSMUSG00000064588	0	0	0	0	0	0
ENSMUSG00000064585	0	0	0	0	0	0
ENSMUSG00000064584	0	0	0	0	0	0
ENSMUSG00000101405	0	0	0	0	0	0
ENSMUSG00000064581	1	0	0	0	0	0
ENSMUSG00000111985	8	6	11	7	4	10
ENSMUSG00000079722	0	0	3	0	0	0
ENSMUSG00000065185	0	0	0	0	0	0
ENSMUSG00000065187	0	1	0	0	0	0
ENSMUSG00000065181	0	0	0	0	0	0
ENSMUSG00000101929	0	0	0	0	0	0
ENSMUSG00000101928	0	0	0	0	0	0
ENSMUSG00000101927	0	0	0	0	0	0
ENSMUSG00000088654	0	0	0	0	0	0
ENSMUSG00000088657	0	0	0	0	0	0
ENSMUSG00000088656	0	0	0	0	0	0
ENSMUSG00000101923	0	0	0	0	0	0
ENSMUSG00000101922	0	0	0	0	0	0
ENSMUSG00000088653	0	0	0	0	0	0
ENSMUSG00000101920	0	0	0	0	0	0
ENSMUSG00000095394	0	0	0	0	0	0
ENSMUSG00000107189	0	0	0	0	0	0
ENSMUSG00000107184	2	0	0	4	0	4

ENSMUSG00000107183	0	0	0	2	0	0
ENSMUSG00000107181	0	0	0	0	0	0
ENSMUSG00000036658	0	0	0	1	0	0
ENSMUSG00000095931	0	1	0	0	0	0
ENSMUSG00000103406	0	0	0	0	0	0
ENSMUSG00000103407	0	0	0	0	0	0
ENSMUSG00000103404	2	0	2	5	8	9
ENSMUSG00000103402	0	0	0	0	0	0
ENSMUSG00000065238	0	0	0	0	0	0
ENSMUSG00000065239	0	0	0	0	0	0
ENSMUSG00000065237	0	0	0	0	0	0
ENSMUSG00000065235	0	0	0	0	0	0
ENSMUSG00000034656	145	140	180	61	81	100
ENSMUSG00000065233	0	0	0	0	0	0
ENSMUSG00000086066	0	0	0	0	0	0
ENSMUSG00000063804	30	26	26	22	29	34
ENSMUSG00000039555	0	0	0	0	0	0
ENSMUSG00000039552	11	6	15	9	0	10
ENSMUSG00000113429	0	0	0	0	0	0
ENSMUSG00000029700	0	0	0	0	0	0
ENSMUSG00000029706	0	0	0	0	0	0
ENSMUSG00000024552	0	0	0	0	0	0
ENSMUSG00000024553	0	0	5	0	0	0
ENSMUSG00000103612	0	0	0	0	0	0
ENSMUSG00000097376	0	0	6	0	0	0
ENSMUSG00000097377	16	20	25	10	11	19
ENSMUSG00000097374	5	18	10	14	9	12
ENSMUSG00000073207	12	16	10	8	0	12
ENSMUSG00000097372	4	0	17	8	0	0
ENSMUSG00000022860	50	2	18	0	0	0
ENSMUSG00000022861	33	29	29	18	20	51
ENSMUSG00000073208	0	0	0	0	0	0
ENSMUSG00000073209	2	0	2	5	6	10
ENSMUSG00000097378	16	18	15	2	3	0
ENSMUSG00000103298	0	0	0	0	0	0
ENSMUSG00000020704	0	0	0	5	0	2
ENSMUSG00000020701	21	16	29	2	7	10
ENSMUSG00000020703	0	0	0	0	0	0
ENSMUSG00000020702	2	0	0	0	0	0
ENSMUSG00000054320	29	27	46	17	32	32
ENSMUSG00000054321	268	361	291	326	317	447
ENSMUSG00000054325	0	0	0	0	0	0
ENSMUSG00000105123	0	0	0	0	0	0
ENSMUSG00000105122	0	0	0	0	0	0
ENSMUSG00000105121	0	0	0	0	0	0
ENSMUSG00000114269	0	0	0	0	0	0
ENSMUSG00000105126	0	0	0	0	0	0
ENSMUSG00000105125	0	0	0	0	0	0
ENSMUSG00000105124	1	0	2	0	0	0
ENSMUSG00000114262	0	0	0	0	0	0
ENSMUSG00000114263	0	0	0	0	0	0
ENSMUSG00000105129	0	0	0	0	0	0
ENSMUSG00000114261	0	0	0	0	0	0
ENSMUSG00000114265	0	0	0	0	0	0
ENSMUSG00000050138	0	0	0	0	0	3
ENSMUSG00000054498	0	0	0	0	0	0
ENSMUSG00000050132	6	10	8	4	0	9
ENSMUSG00000067276	103	52	66	0	10	6

ENSMUSG00000057674	0	0	0	0	0	0
ENSMUSG00000093421	0	0	0	0	0	0
ENSMUSG00000111364	0	0	0	0	0	0
ENSMUSG00000095154	2	0	0	0	0	1
ENSMUSG00000111365	0	0	0	0	0	0
ENSMUSG00000073811	0	0	0	0	0	0
ENSMUSG00000108570	0	0	0	0	0	0
ENSMUSG00000108577	0	0	0	0	0	0
ENSMUSG00000108576	0	0	0	0	0	0
ENSMUSG00000104294	0	0	0	0	0	0
ENSMUSG00000104295	0	2	0	1	3	1
ENSMUSG00000039224	2	0	0	0	0	0
ENSMUSG00000108579	0	0	0	0	0	0
ENSMUSG00000104299	4	0	6	2	0	0
ENSMUSG00000021070	22	13	41	9	6	6
ENSMUSG00000065319	0	0	2	0	1	0
ENSMUSG00000111862	3	0	0	0	0	3
ENSMUSG00000101216	0	0	0	0	0	0
ENSMUSG00000101118	0	0	0	0	0	0
ENSMUSG00000101119	0	0	0	0	0	0
ENSMUSG00000101116	0	0	0	0	0	0
ENSMUSG00000060726	5	3	0	0	0	0
ENSMUSG00000101114	0	0	0	0	0	0
ENSMUSG00000060724	0	0	0	0	0	0
ENSMUSG00000101112	0	0	0	0	0	0
ENSMUSG00000101113	0	0	0	0	0	0
ENSMUSG00000101110	0	0	0	0	0	0
ENSMUSG00000111342	0	2	0	0	0	0
ENSMUSG00000111343	23	8	8	17	7	8
ENSMUSG00000111344	3	0	0	0	0	0
ENSMUSG00000064915	0	2	0	0	0	0
ENSMUSG00000091006	0	0	0	0	0	0
ENSMUSG00000091004	0	2	0	0	0	0
ENSMUSG00000091005	0	0	0	0	0	0
ENSMUSG00000091002	0	0	0	0	0	2
ENSMUSG00000091003	0	0	0	0	0	0
ENSMUSG00000026931	3	0	0	0	0	0
ENSMUSG00000026936	0	0	0	0	0	0
ENSMUSG00000026937	0	0	0	0	0	0
ENSMUSG00000095785	0	0	0	0	0	0
ENSMUSG00000111611	0	0	0	0	0	0
ENSMUSG00000081038	0	0	0	0	0	0
ENSMUSG00000095786	0	0	1	0	0	0
ENSMUSG00000095780	0	0	0	0	0	0
ENSMUSG00000095782	0	0	0	0	0	0
ENSMUSG00000095789	24	39	25	29	21	39
ENSMUSG00000095788	18	9	38	0	5	3
ENSMUSG00000022034	51	54	47	3	27	45
ENSMUSG00000022033	55	57	51	0	19	30
ENSMUSG00000027048	0	14	4	0	2	0
ENSMUSG00000042448	0	0	0	0	0	0
ENSMUSG00000053773	0	0	0	0	0	0
ENSMUSG00000087407	0	0	0	0	0	0
ENSMUSG00000094789	0	0	0	0	0	0
ENSMUSG00000111159	0	0	0	0	0	0
ENSMUSG00000094782	0	0	0	0	0	0
ENSMUSG00000094784	0	0	0	0	0	0
ENSMUSG00000094785	0	0	0	0	0	0

ENSMUSG00000086569	0	0	0	0	2	0
ENSMUSG00000086563	0	0	0	0	0	0
ENSMUSG00000086564	11	12	10	16	9	6
ENSMUSG00000095935	0	0	0	0	0	0
ENSMUSG00000062814	0	0	0	0	0	0
ENSMUSG00000062818	0	2	1	0	0	0
ENSMUSG00000023345	134	119	122	125	131	201
ENSMUSG00000087401	0	0	0	0	0	0
ENSMUSG00000103394	0	0	0	2	0	3
ENSMUSG00000108156	0	0	0	0	0	0
ENSMUSG00000103051	0	0	0	0	0	0
ENSMUSG00000092168	0	0	0	0	0	0
ENSMUSG00000093311	0	0	0	0	0	0
ENSMUSG00000100068	0	0	0	0	4	0
ENSMUSG00000049532	305	224	308	129	148	167
ENSMUSG00000049530	0	0	0	0	0	0
ENSMUSG00000049538	6	3	17	0	0	0
ENSMUSG00000103699	0	0	0	0	0	0
ENSMUSG00000036510	6	2	0	0	0	0
ENSMUSG00000099453	0	0	0	0	0	0
ENSMUSG00000112706	0	1	4	0	0	3
ENSMUSG00000112705	3	4	0	0	0	0
ENSMUSG00000112704	0	0	0	0	0	0
ENSMUSG00000112703	0	0	0	0	0	0
ENSMUSG00000112702	0	0	2	1	0	1
ENSMUSG00000099458	0	0	0	0	0	0
ENSMUSG00000096921	0	0	0	0	0	0
ENSMUSG00000096922	0	0	0	0	0	0
ENSMUSG00000049281	99	94	97	93	88	108
ENSMUSG00000049280	2	0	2	0	0	0
ENSMUSG00000096927	1	0	1	1	1	1
ENSMUSG00000106339	13	9	13	0	2	2
ENSMUSG00000002578	5	13	22	7	9	0
ENSMUSG00000106330	0	0	0	0	0	0
ENSMUSG00000106336	0	0	0	0	0	0
ENSMUSG00000113252	0	0	0	0	0	0
ENSMUSG00000113253	0	0	0	0	0	0
ENSMUSG00000113251	0	0	0	0	0	0
ENSMUSG00000113254	0	0	0	0	0	0
ENSMUSG00000103055	0	0	0	0	0	0
ENSMUSG00000113259	0	0	0	0	0	0
ENSMUSG00000047253	0	0	0	0	0	0
ENSMUSG00000079392	0	1	1	1	0	0
ENSMUSG00000047257	0	0	4	0	0	0
ENSMUSG00000047259	0	0	0	0	0	0
ENSMUSG00000109309	0	0	0	0	0	0
ENSMUSG00000109308	0	0	0	0	0	0
ENSMUSG00000003227	15	23	19	0	16	20
ENSMUSG00000109301	2	2	2	2	0	0
ENSMUSG00000109305	2	9	10	2	0	9
ENSMUSG00000082712	0	0	0	2	0	0
ENSMUSG00000082713	0	0	0	0	0	0
ENSMUSG00000082710	0	0	0	0	0	0
ENSMUSG00000082717	0	0	0	0	0	0
ENSMUSG00000082714	0	0	0	0	0	0
ENSMUSG00000083049	11	23	20	10	15	17
ENSMUSG00000103056	0	5	5	0	3	0
ENSMUSG00000083041	6	7	9	3	2	1

ENSMUSG00000083042	0	0	0	0	0	0
ENSMUSG00000083044	57	55	70	59	51	64
ENSMUSG00000083047	0	0	0	0	0	0
ENSMUSG00000083046	0	0	0	0	0	0
ENSMUSG00000114701	0	0	7	0	0	0
ENSMUSG00000114700	0	0	0	0	0	0
ENSMUSG00000114703	0	0	2	3	0	3
ENSMUSG00000114702	0	0	0	0	0	0
ENSMUSG00000114707	77	64	79	86	68	78
ENSMUSG00000114706	0	0	0	0	0	0
ENSMUSG00000110633	0	0	0	0	0	2
ENSMUSG00000110632	2	0	0	0	0	1
ENSMUSG00000110634	0	0	0	0	0	0
ENSMUSG00000110636	0	0	0	2	0	0
ENSMUSG00000110639	0	0	0	0	0	0
ENSMUSG00000110638	24	0	24	10	8	14
ENSMUSG00000084459	2	1	2	1	0	1
ENSMUSG00000005107	97	102	109	65	83	124
ENSMUSG00000022015	0	5	6	0	0	0
ENSMUSG00000084456	0	0	0	0	0	0
ENSMUSG00000099586	0	0	0	0	0	0
ENSMUSG00000025588	14	20	18	26	23	24
ENSMUSG00000025582	4	7	0	0	0	0
ENSMUSG00000070099	0	0	0	0	0	0
ENSMUSG00000082688	2	0	3	2	5	0
ENSMUSG00000100169	0	0	0	0	0	0
ENSMUSG00000026582	73	101	87	116	91	91
ENSMUSG00000026587	87	100	70	63	93	104
ENSMUSG00000026589	478	408	409	118	141	176
ENSMUSG00000079120	0	0	0	0	0	0
ENSMUSG00000068263	69	37	110	21	47	51
ENSMUSG00000031481	0	0	1	0	0	2
ENSMUSG00000006488	0	0	0	0	0	0
ENSMUSG00000082687	0	0	0	1	0	0
ENSMUSG00000113790	0	0	0	0	0	2
ENSMUSG00000085051	0	0	0	4	0	2
ENSMUSG00000085053	0	0	0	0	0	0
ENSMUSG00000085057	2	2	0	0	0	0
ENSMUSG00000085059	0	0	0	0	0	0
ENSMUSG00000026058	6	0	0	0	0	0
ENSMUSG00000071311	7	4	7	4	5	7
ENSMUSG00000033826	25	76	70	26	63	41
ENSMUSG00000081994	0	0	0	0	0	0
ENSMUSG00000081991	0	0	0	0	0	0
ENSMUSG00000081993	0	0	0	0	0	0
ENSMUSG00000098193	0	0	0	0	0	0
ENSMUSG00000041849	206	154	162	132	138	173
ENSMUSG00000098190	0	0	0	0	0	0
ENSMUSG00000098197	0	0	0	0	0	0
ENSMUSG00000098195	0	0	0	0	0	0
ENSMUSG00000112773	0	0	0	0	0	0
ENSMUSG00000041842	36	26	33	4	18	18
ENSMUSG00000099860	0	0	0	0	0	0
ENSMUSG00000099861	0	0	0	0	0	0
ENSMUSG00000099862	0	0	0	0	0	0
ENSMUSG00000099863	0	0	0	0	0	0
ENSMUSG00000099864	0	0	0	0	0	0
ENSMUSG00000099866	0	0	0	0	0	0

ENSMUSG00000099867	0	0	0	0	0	0
ENSMUSG00000090643	0	0	0	0	0	0
ENSMUSG00000112774	0	0	0	0	0	0
ENSMUSG00000074575	0	0	0	0	3	0
ENSMUSG00000090642	0	0	0	0	0	0
ENSMUSG00000074579	189	213	197	180	192	255
ENSMUSG00000112775	0	0	0	0	0	0
ENSMUSG00000111986	0	0	0	0	0	0
ENSMUSG00000026405	4	0	3	3	4	0
ENSMUSG00000111981	0	0	0	0	0	0
ENSMUSG00000081370	0	2	0	0	0	4
ENSMUSG00000109047	0	0	0	0	0	0
ENSMUSG00000081377	0	0	0	0	0	0
ENSMUSG00000081379	0	0	0	0	0	0
ENSMUSG00000109040	2	0	0	0	0	0
ENSMUSG00000111983	0	0	0	0	0	0
ENSMUSG00000109041	0	0	0	0	0	0
ENSMUSG00000019942	87	96	94	31	37	72
ENSMUSG00000072934	0	0	0	0	0	0
ENSMUSG00000072931	0	0	0	0	0	0
ENSMUSG00000072930	0	0	0	0	0	0
ENSMUSG00000090505	0	0	0	0	0	0
ENSMUSG00000084825	0	0	0	0	0	0
ENSMUSG00000090509	0	0	0	0	0	0
ENSMUSG00000084829	0	0	0	0	0	0
ENSMUSG00000077218	0	0	0	0	0	0
ENSMUSG00000038917	0	0	5	0	0	0
ENSMUSG00000038916	0	0	0	0	0	0
ENSMUSG00000077215	0	0	0	0	0	0
ENSMUSG00000077214	0	0	0	0	0	0
ENSMUSG00000105494	2	1	1	2	0	1
ENSMUSG00000083153	0	0	0	0	0	0
ENSMUSG00000110688	0	0	0	0	0	0
ENSMUSG00000031535	0	0	2	2	3	0
ENSMUSG00000083554	0	13	5	0	0	16
ENSMUSG00000083156	0	0	0	0	0	0
ENSMUSG00000043144	0	0	0	2	0	0
ENSMUSG00000043145	0	0	0	0	0	0
ENSMUSG00000110687	0	0	0	0	0	0
ENSMUSG00000110680	0	0	0	0	0	0
ENSMUSG00000110681	0	0	0	0	0	0
ENSMUSG00000025043	0	0	0	0	0	0
ENSMUSG00000026560	0	0	0	0	0	0
ENSMUSG00000113422	0	0	0	0	0	0
ENSMUSG00000087884	0	0	0	0	0	0
ENSMUSG00000082051	14	12	9	15	14	14
ENSMUSG00000030666	13	10	0	0	0	0
ENSMUSG00000082053	2	0	0	0	0	0
ENSMUSG00000082052	3	7	6	4	5	4
ENSMUSG00000088039	0	0	0	0	1	0
ENSMUSG00000082054	0	0	0	0	0	0
ENSMUSG00000087029	0	0	0	0	0	0
ENSMUSG00000087028	12	12	17	22	11	34
ENSMUSG00000082059	0	0	0	0	0	1
ENSMUSG00000082058	0	0	0	0	0	0
ENSMUSG00000087024	0	0	6	2	5	8
ENSMUSG00000088031	0	0	0	0	0	0
ENSMUSG00000087022	4	6	2	2	8	6

ENSMUSG00000030669	5	0	0	0	0	0
ENSMUSG00000087020	5	8	3	3	2	6
ENSMUSG00000101354	0	0	0	0	0	0
ENSMUSG00000101357	2	2	0	0	0	0
ENSMUSG00000101350	0	0	0	0	0	0
ENSMUSG00000101351	0	0	0	0	0	0
ENSMUSG00000101352	0	0	0	0	0	0
ENSMUSG00000101353	0	0	0	0	0	0
ENSMUSG00000085889	0	0	6	0	0	0
ENSMUSG00000085880	0	0	0	0	0	2
ENSMUSG00000103610	0	0	0	0	0	0
ENSMUSG00000087886	0	0	0	0	0	0
ENSMUSG00000038300	0	0	0	0	0	0
ENSMUSG00000103267	0	0	0	0	0	0
ENSMUSG00000100089	0	0	0	0	0	0
ENSMUSG00000103265	4	2	1	3	5	2
ENSMUSG00000103262	0	0	0	0	0	0
ENSMUSG00000103263	0	0	0	0	0	0
ENSMUSG00000103260	0	0	0	4	1	3
ENSMUSG00000103261	6	12	2	10	13	20
ENSMUSG00000100083	0	0	0	0	0	0
ENSMUSG00000100082	0	0	0	0	0	0
ENSMUSG00000100080	0	0	0	0	0	0
ENSMUSG00000100087	0	0	0	0	0	0
ENSMUSG00000100086	0	0	0	0	0	0
ENSMUSG00000100085	0	0	0	0	0	0
ENSMUSG00000103269	0	0	0	0	0	0
ENSMUSG00000101686	0	0	0	0	0	0
ENSMUSG00000040724	124	95	71	77	175	261
ENSMUSG00000101684	0	0	0	0	0	0
ENSMUSG00000101685	0	0	0	0	0	0
ENSMUSG00000101683	0	0	0	0	0	0
ENSMUSG00000101681	0	0	0	0	0	0
ENSMUSG00000040729	199	271	138	216	225	263
ENSMUSG00000101688	0	0	0	0	0	0
ENSMUSG00000101689	0	0	0	0	0	0
ENSMUSG00000107681	0	0	0	0	0	0
ENSMUSG00000107683	0	0	0	0	0	0
ENSMUSG00000107684	0	0	1	0	0	1
ENSMUSG00000089349	0	0	0	0	0	0
ENSMUSG00000084598	0	0	0	0	0	0
ENSMUSG00000107687	0	0	0	0	0	0
ENSMUSG00000089344	1	0	0	0	0	0
ENSMUSG00000089341	0	0	0	0	0	0
ENSMUSG00000089342	0	0	0	0	0	0
ENSMUSG00000084591	0	2	0	0	0	0
ENSMUSG00000103190	0	0	0	0	0	0
ENSMUSG00000103193	0	0	0	0	0	0
ENSMUSG00000103196	0	0	0	0	2	0
ENSMUSG00000069248	0	0	0	0	0	0
ENSMUSG00000032750	107	73	102	45	83	107
ENSMUSG00000084041	0	0	0	0	0	0
ENSMUSG00000084043	0	0	0	0	0	0
ENSMUSG00000084044	0	0	0	0	0	0
ENSMUSG00000105638	0	0	0	0	0	0
ENSMUSG00000084046	0	0	0	0	0	0
ENSMUSG00000075302	0	0	0	0	0	0
ENSMUSG00000030283	18	42	41	14	18	17

ENSMUSG00000095813	0	0	0	0	0	0
ENSMUSG00000075304	5	0	0	0	0	0
ENSMUSG00000100555	19	13	12	0	0	12
ENSMUSG00000065884	0	0	0	0	0	0
ENSMUSG00000065881	3	0	0	0	0	0
ENSMUSG00000065882	0	0	0	0	0	0
ENSMUSG00000065889	0	0	0	0	0	0
ENSMUSG00000022589	0	0	0	0	0	0
ENSMUSG00000022580	0	0	4	4	0	2
ENSMUSG00000022583	0	0	0	0	0	0
ENSMUSG00000022582	0	7	0	0	0	0
ENSMUSG00000060967	0	0	0	0	0	0
ENSMUSG00000096356	0	0	0	0	0	0
ENSMUSG00000064428	0	0	0	0	0	0
ENSMUSG00000107027	0	0	0	0	0	0
ENSMUSG00000107022	0	0	0	0	0	0
ENSMUSG00000107028	1	0	0	0	0	2
ENSMUSG00000107029	0	0	0	2	2	2
ENSMUSG00000023185	0	0	0	0	0	0
ENSMUSG00000012282	0	0	0	0	0	0
ENSMUSG00000058953	0	0	0	0	0	0
ENSMUSG00000103618	0	0	0	0	0	0
ENSMUSG00000085316	5	8	10	0	11	3
ENSMUSG00000085314	0	0	5	0	3	7
ENSMUSG00000085312	0	0	0	0	0	0
ENSMUSG00000085318	0	0	4	0	0	3
ENSMUSG00000105633	0	0	0	2	0	0
ENSMUSG00000033581	138	134	263	103	116	110
ENSMUSG00000028661	0	0	0	4	0	0
ENSMUSG00000028664	85	62	99	14	24	27
ENSMUSG00000088596	0	0	0	0	0	0
ENSMUSG00000088597	0	0	0	0	0	0
ENSMUSG00000088594	0	0	0	0	0	0
ENSMUSG00000088595	0	0	0	0	0	0
ENSMUSG00000088598	0	0	0	0	0	0
ENSMUSG00000095447	0	0	0	0	0	0
ENSMUSG00000050463	0	0	6	0	0	6
ENSMUSG00000029134	13	25	36	9	36	40
ENSMUSG00000061614	0	0	0	0	0	0
ENSMUSG00000113011	0	0	0	0	3	0
ENSMUSG00000061616	0	0	0	0	0	0
ENSMUSG00000113013	0	0	0	0	0	0
ENSMUSG00000113016	0	0	0	0	0	0
ENSMUSG00000113017	0	0	2	0	0	0
ENSMUSG00000113018	0	0	0	0	0	0
ENSMUSG00000059334	0	0	0	0	3	5
ENSMUSG00000059336	32	32	46	26	12	30
ENSMUSG00000063615	3	0	0	0	0	0
ENSMUSG00000109548	0	2	0	0	0	0
ENSMUSG00000111528	4	0	6	0	6	3
ENSMUSG00000109541	0	0	0	0	0	3
ENSMUSG00000109540	0	0	0	0	0	0
ENSMUSG00000109543	0	0	0	0	0	0
ENSMUSG00000109542	2	0	0	0	0	0
ENSMUSG00000109545	0	0	0	0	0	2
ENSMUSG00000109544	0	0	0	0	0	0
ENSMUSG00000109547	0	0	0	0	0	0
ENSMUSG00000109546	0	0	0	0	0	0

ENSMUSG00000106642	0	0	0	0	0	0
ENSMUSG00000050818	0	0	0	0	0	0
ENSMUSG00000106640	0	0	0	0	0	0
ENSMUSG00000106647	0	0	0	0	0	0
ENSMUSG00000066060	0	0	0	0	0	0
ENSMUSG00000106644	0	0	0	0	0	0
ENSMUSG00000050813	0	0	0	0	0	0
ENSMUSG00000050810	0	0	0	0	0	0
ENSMUSG00000106648	38	37	56	56	47	72
ENSMUSG00000050815	0	0	0	0	0	0
ENSMUSG00000097504	37	59	52	40	63	44
ENSMUSG00000038496	28	22	30	19	13	20
ENSMUSG00000097502	0	0	0	0	0	0
ENSMUSG00000104645	0	0	0	0	0	0
ENSMUSG00000104644	0	0	0	0	0	0
ENSMUSG00000104647	0	0	0	0	0	0
ENSMUSG00000038498	0	0	0	0	0	0
ENSMUSG00000104640	0	0	0	0	0	0
ENSMUSG00000104643	0	0	0	0	0	0
ENSMUSG00000051499	66	62	40	47	58	62
ENSMUSG00000051498	78	72	100	31	39	47
ENSMUSG00000051490	0	0	4	4	0	2
ENSMUSG00000051493	0	0	0	2	0	0
ENSMUSG00000051497	0	0	4	0	0	0
ENSMUSG00000114549	0	0	0	0	0	0
ENSMUSG00000114548	0	0	0	0	0	0
ENSMUSG00000114546	0	0	0	0	0	0
ENSMUSG00000114544	2	0	2	1	2	0
ENSMUSG00000114543	0	0	0	0	1	0
ENSMUSG00000114542	0	0	0	0	0	0
ENSMUSG00000114541	0	0	0	0	0	0
ENSMUSG00000066392	43	34	89	0	6	12
ENSMUSG00000111484	2	0	0	0	0	2
ENSMUSG00000112878	0	0	0	0	0	0
ENSMUSG00000100936	0	0	0	0	0	0
ENSMUSG00000112871	0	0	0	0	0	0
ENSMUSG00000112870	0	0	0	0	0	0
ENSMUSG00000112873	0	0	0	0	0	0
ENSMUSG00000112872	0	0	0	0	0	0
ENSMUSG00000112876	0	0	0	0	0	0
ENSMUSG00000092222	0	0	0	0	0	0
ENSMUSG00000091908	2	0	4	2	4	2
ENSMUSG00000091903	0	0	0	0	0	0
ENSMUSG00000091902	0	0	0	0	0	0
ENSMUSG00000109892	0	0	0	0	0	0
ENSMUSG00000109891	0	0	0	0	0	0
ENSMUSG00000109890	0	0	0	0	0	3
ENSMUSG00000109897	0	0	0	0	0	0
ENSMUSG00000109896	0	0	0	0	0	0
ENSMUSG00000109895	0	0	0	0	0	0
ENSMUSG00000114091	0	0	0	0	0	0
ENSMUSG00000114090	0	0	0	0	0	2
ENSMUSG00000114092	0	0	0	0	0	0
ENSMUSG00000080684	0	0	0	0	0	0
ENSMUSG00000114097	0	0	0	0	0	0
ENSMUSG00000114096	1	1	0	1	1	0
ENSMUSG00000025747	267	161	313	87	66	134
ENSMUSG00000110734	0	0	0	0	0	0

ENSMUSG00000110736	0	0	0	0	0	0
ENSMUSG00000058172	0	0	0	0	0	0
ENSMUSG00000110739	0	0	0	0	0	0
ENSMUSG00000050366	0	1	0	0	0	0
ENSMUSG00000056910	0	0	0	0	0	0
ENSMUSG00000056912	0	0	0	0	0	0
ENSMUSG00000092227	0	0	0	0	0	0
ENSMUSG00000023964	0	0	0	0	0	3
ENSMUSG00000096676	0	0	0	0	0	0
ENSMUSG00000096677	0	0	0	1	0	0
ENSMUSG00000096673	0	0	0	0	0	0
ENSMUSG00000105777	0	0	0	0	0	0
ENSMUSG00000080689	0	0	0	0	0	0
ENSMUSG00000023968	21	4	8	6	8	16
ENSMUSG00000096679	0	0	0	0	0	0
ENSMUSG00000060404	1	0	1	0	0	0
ENSMUSG00000080222	0	0	0	0	0	0
ENSMUSG00000080223	0	0	2	0	2	0
ENSMUSG00000080227	0	0	0	1	1	0
ENSMUSG00000107301	0	0	0	0	0	0
ENSMUSG00000100876	63	53	91	48	51	43
ENSMUSG00000087224	0	0	0	0	0	0
ENSMUSG00000082614	0	0	0	0	2	0
ENSMUSG00000105775	45	55	48	66	36	61
ENSMUSG00000082613	0	0	0	0	0	0
ENSMUSG00000090050	0	0	0	0	0	0
ENSMUSG00000082612	0	0	0	0	0	0
ENSMUSG00000113832	0	0	0	0	0	0
ENSMUSG00000113833	3	0	0	0	0	0
ENSMUSG00000113830	0	0	0	0	0	0
ENSMUSG00000100877	0	0	0	0	0	0
ENSMUSG00000082611	0	0	0	0	0	0
ENSMUSG00000113834	0	0	0	0	0	0
ENSMUSG00000113835	0	0	0	3	0	0
ENSMUSG00000113838	0	0	0	0	0	0
ENSMUSG00000113839	0	0	0	0	0	0
ENSMUSG00000080592	0	0	0	0	0	0
ENSMUSG00000080590	0	0	1	0	0	0
ENSMUSG00000080596	0	0	0	0	0	0
ENSMUSG00000080597	0	0	1	0	0	0
ENSMUSG00000080594	0	0	0	0	0	0
ENSMUSG00000080595	0	0	0	0	0	0
ENSMUSG00000059493	123	126	148	72	54	83
ENSMUSG00000095371	0	0	0	0	0	0
ENSMUSG00000095372	1	0	0	0	0	0
ENSMUSG00000095374	0	0	0	0	0	0
ENSMUSG00000082619	0	0	0	0	0	0
ENSMUSG00000111069	6	0	2	2	0	2
ENSMUSG00000111068	0	0	0	0	0	0
ENSMUSG00000111066	0	0	0	0	0	0
ENSMUSG00000111064	0	0	0	0	0	1
ENSMUSG00000111062	0	0	0	0	0	0
ENSMUSG00000111061	0	0	0	0	0	0
ENSMUSG00000111060	0	0	0	0	0	0
ENSMUSG00000101512	0	0	0	0	0	0
ENSMUSG00000101513	0	0	0	0	0	0
ENSMUSG00000081175	0	0	0	0	0	0
ENSMUSG00000098225	1	3	0	4	0	0

ENSMUSG00000104567	0	0	0	0	0	0
ENSMUSG00000108020	0	0	0	0	0	4
ENSMUSG00000108544	0	0	0	0	0	0
ENSMUSG00000098226	0	1	0	0	0	0
ENSMUSG00000101514	0	0	0	0	0	0
ENSMUSG00000104563	4	3	4	4	4	5
ENSMUSG00000098223	0	2	1	4	2	2
ENSMUSG00000037771	0	0	0	0	0	0
ENSMUSG00000098222	18	7	16	13	20	10
ENSMUSG00000101518	0	0	0	0	0	0
ENSMUSG00000108545	0	0	0	0	0	0
ENSMUSG00000095594	0	0	0	0	0	0
ENSMUSG00000065074	0	0	0	0	0	0
ENSMUSG00000095591	0	0	0	0	0	0
ENSMUSG00000103629	0	0	0	0	0	0
ENSMUSG00000081037	0	0	0	0	0	0
ENSMUSG00000095599	0	0	0	0	0	0
ENSMUSG00000077059	0	1	0	0	0	0
ENSMUSG00000077058	0	0	0	0	0	0
ENSMUSG00000053980	37	32	59	48	57	37
ENSMUSG00000053985	227	256	207	204	149	236
ENSMUSG00000082073	0	0	0	0	0	0
ENSMUSG00000094429	0	0	0	0	0	0
ENSMUSG00000094428	0	0	0	0	0	0
ENSMUSG00000096766	0	0	0	0	0	0
ENSMUSG00000094422	0	0	0	0	0	0
ENSMUSG00000056004	3	11	5	5	4	8
ENSMUSG00000088767	0	0	0	0	0	0
ENSMUSG00000056008	0	0	0	0	0	0
ENSMUSG00000113278	16	27	9	35	26	33
ENSMUSG00000088766	0	0	0	0	0	0
ENSMUSG00000052581	0	0	0	0	0	0
ENSMUSG00000093804	0	2	0	0	0	0
ENSMUSG00000111152	0	0	0	0	0	0
ENSMUSG00000093806	0	0	0	0	0	0
ENSMUSG00000097991	5	9	4	9	2	6
ENSMUSG00000055679	0	0	0	0	0	0
ENSMUSG00000112001	4	4	3	3	3	2
ENSMUSG00000112002	0	0	0	0	0	0
ENSMUSG00000042184	0	0	0	0	0	0
ENSMUSG00000112005	0	0	0	0	0	0
ENSMUSG00000111156	0	0	0	0	0	0
ENSMUSG00000113270	0	0	0	0	0	0
ENSMUSG00000093802	0	0	0	0	0	0
ENSMUSG00000106358	0	0	0	0	0	0
ENSMUSG00000111154	0	0	0	0	0	0
ENSMUSG00000054988	0	0	0	0	0	0
ENSMUSG00000093501	0	0	0	0	0	0
ENSMUSG00000054986	0	0	0	0	0	0
ENSMUSG00000094736	0	0	0	0	0	0
ENSMUSG00000051802	0	0	0	0	0	0
ENSMUSG00000086339	0	0	0	0	0	0
ENSMUSG00000093292	0	0	0	0	0	0
ENSMUSG00000051804	0	0	5	0	0	0
ENSMUSG00000086335	0	0	0	0	0	0
ENSMUSG00000086336	0	0	0	0	0	0
ENSMUSG00000086337	0	0	0	0	0	0
ENSMUSG00000076010	0	0	0	0	0	0

ENSMUSG00000076014	0	0	0	0	0	0
ENSMUSG00000113356	0	0	0	0	0	0
ENSMUSG00000113354	0	0	0	0	0	0
ENSMUSG00000113352	0	0	0	0	0	0
ENSMUSG00000113351	0	0	0	0	0	0
ENSMUSG00000113350	0	0	0	0	0	0
ENSMUSG00000113359	0	0	0	0	0	0
ENSMUSG00000113358	0	0	0	0	0	0
ENSMUSG00000049098	0	0	0	0	0	0
ENSMUSG00000049094	0	0	0	0	0	0
ENSMUSG00000049097	54	60	40	22	44	70
ENSMUSG00000049093	0	0	3	0	0	0
ENSMUSG00000082528	2	0	1	0	0	0
ENSMUSG00000082523	0	0	0	0	0	0
ENSMUSG00000082524	0	0	0	0	0	0
ENSMUSG00000040121	66	54	75	35	29	44
ENSMUSG00000040127	0	0	0	0	0	0
ENSMUSG00000062170	37	56	12	14	13	10
ENSMUSG00000077597	0	2	0	1	0	0
ENSMUSG00000048697	0	0	0	0	0	0
ENSMUSG00000084206	0	0	0	0	0	0
ENSMUSG00000084204	0	0	0	0	0	0
ENSMUSG00000084205	0	0	0	0	0	0
ENSMUSG00000084202	0	0	0	0	0	4
ENSMUSG00000084203	5	3	4	0	3	3
ENSMUSG00000084200	0	0	0	0	0	0
ENSMUSG00000075543	0	0	0	0	0	0
ENSMUSG00000084208	0	0	0	0	0	0
ENSMUSG00000084209	0	0	0	0	0	0
ENSMUSG00000041293	0	3	3	0	4	0
ENSMUSG00000105807	0	0	0	0	0	0
ENSMUSG00000081553	0	2	3	2	0	2
ENSMUSG00000105800	0	0	0	0	0	0
ENSMUSG00000103531	0	0	0	0	0	2
ENSMUSG00000080962	0	0	0	0	0	4
ENSMUSG00000080961	0	0	0	0	0	0
ENSMUSG00000080960	0	0	0	0	0	0
ENSMUSG00000080965	0	0	0	0	0	0
ENSMUSG00000080964	0	0	0	0	0	0
ENSMUSG00000105208	0	0	0	0	0	0
ENSMUSG00000078640	41	32	78	5	15	18
ENSMUSG00000103538	0	0	0	0	0	0
ENSMUSG00000065719	0	0	0	0	0	0
ENSMUSG00000065718	0	0	0	0	0	0
ENSMUSG00000068547	0	0	0	0	0	0
ENSMUSG00000070111	0	0	4	0	0	2
ENSMUSG00000099128	0	0	0	0	0	0
ENSMUSG00000099120	0	0	0	0	0	0
ENSMUSG00000099124	0	0	0	0	0	0
ENSMUSG00000099126	0	0	0	0	0	0
ENSMUSG00000032343	3	5	0	6	14	3
ENSMUSG00000001496	0	0	0	0	0	0
ENSMUSG00000001497	4	0	0	0	0	0
ENSMUSG00000068099	0	0	0	0	0	0
ENSMUSG00000089105	0	0	0	2	0	0
ENSMUSG00000054450	4	2	0	0	0	0
ENSMUSG00000006724	3	4	9	0	5	0
ENSMUSG00000006720	28	47	8	25	20	28

ENSMUSG00000054951	96	97	65	44	59	44
ENSMUSG00000035187	0	0	0	0	0	0
ENSMUSG00000112649	0	0	1	0	0	0
ENSMUSG00000073494	0	0	0	0	0	0
ENSMUSG00000073497	0	0	0	0	0	0
ENSMUSG00000029630	0	0	0	0	0	0
ENSMUSG00000108982	0	0	0	0	0	0
ENSMUSG00000024207	0	0	0	0	0	0
ENSMUSG00000093681	0	0	0	0	0	0
ENSMUSG00000104798	0	0	0	0	0	0
ENSMUSG00000111268	0	0	0	0	0	0
ENSMUSG00000103833	0	0	0	1	0	0
ENSMUSG00000103832	0	0	0	0	0	0
ENSMUSG00000103831	0	0	0	0	0	5
ENSMUSG00000103837	0	2	0	4	0	2
ENSMUSG00000103836	0	0	0	0	0	0
ENSMUSG00000103839	62	52	48	29	48	48
ENSMUSG00000022439	42	73	113	32	15	25
ENSMUSG00000019756	0	0	0	0	0	0
ENSMUSG00000070138	1	0	0	1	0	2
ENSMUSG00000063188	0	0	0	0	0	0
ENSMUSG00000089952	24	35	24	4	6	12
ENSMUSG00000089957	0	0	0	0	0	0
ENSMUSG00000089956	0	0	2	0	0	0
ENSMUSG00000109822	4	3	3	2	3	6
ENSMUSG00000076977	0	0	0	0	0	0
ENSMUSG00000076976	0	1	0	0	2	0
ENSMUSG00000108921	0	1	0	0	0	0
ENSMUSG00000108922	0	0	0	0	0	0
ENSMUSG00000108924	0	0	0	0	0	0
ENSMUSG00000108926	13	8	15	25	13	18
ENSMUSG00000108928	0	0	0	0	0	0
ENSMUSG00000108929	0	0	3	0	0	4
ENSMUSG00000034959	85	134	170	96	91	87
ENSMUSG00000105869	0	0	0	0	0	0
ENSMUSG00000099667	0	0	0	0	0	0
ENSMUSG00000043333	0	2	22	0	0	4
ENSMUSG00000067604	0	0	0	0	0	0
ENSMUSG00000043331	0	0	0	0	0	0
ENSMUSG00000099664	0	0	0	0	0	0
ENSMUSG00000030257	281	156	337	27	15	62
ENSMUSG00000030254	155	233	145	92	129	124
ENSMUSG00000109826	0	0	0	0	0	0
ENSMUSG00000099660	1	0	0	0	0	0
ENSMUSG00000111260	0	0	0	0	0	0
ENSMUSG00000109700	0	0	0	0	0	0
ENSMUSG00000036855	0	0	0	0	2	0
ENSMUSG00000101528	0	0	0	0	0	0
ENSMUSG00000101522	0	0	0	0	0	0
ENSMUSG00000101521	0	0	0	0	0	0
ENSMUSG00000101520	0	0	0	0	0	0
ENSMUSG00000101527	0	0	0	0	0	0
ENSMUSG00000060371	0	3	0	0	0	0
ENSMUSG00000101525	0	0	0	0	0	0
ENSMUSG00000085925	35	30	0	0	0	17
ENSMUSG00000085926	0	0	0	0	3	0
ENSMUSG00000085921	0	0	0	0	0	0
ENSMUSG00000085922	0	0	0	0	0	0

ENSMUSG00000085928	0	0	0	0	0	0
ENSMUSG00000085929	0	7	2	4	0	0
ENSMUSG00000110614	3	0	6	0	6	3
ENSMUSG00000087278	0	0	0	0	0	0
ENSMUSG00000087279	0	0	0	0	0	0
ENSMUSG00000087276	0	0	0	0	0	0
ENSMUSG00000087277	3	4	7	2	2	10
ENSMUSG00000087273	0	0	0	0	0	0
ENSMUSG00000037705	0	0	0	0	0	0
ENSMUSG00000037708	13	18	24	10	14	16
ENSMUSG00000021953	0	0	0	0	0	0
ENSMUSG00000033633	10	31	10	62	65	63
ENSMUSG00000095072	0	0	0	0	0	0
ENSMUSG00000103015	0	0	2	0	0	0
ENSMUSG00000103017	0	0	0	0	0	0
ENSMUSG00000103010	2	0	0	0	0	0
ENSMUSG00000103012	0	0	0	0	3	0
ENSMUSG00000112777	0	0	0	0	0	0
ENSMUSG00000062224	0	0	0	0	2	0
ENSMUSG00000059964	0	0	0	0	0	0
ENSMUSG00000114425	0	0	0	0	0	0
ENSMUSG00000114428	0	0	0	0	0	0
ENSMUSG00000100947	0	0	0	0	0	0
ENSMUSG00000089138	0	0	0	0	0	0
ENSMUSG00000100940	0	0	0	0	0	0
ENSMUSG00000089132	0	0	0	0	0	0
ENSMUSG00000089130	0	0	0	0	0	0
ENSMUSG00000089137	0	0	0	0	0	0
ENSMUSG00000089136	0	0	0	0	0	0
ENSMUSG00000089134	0	0	0	0	0	0
ENSMUSG00000018634	0	0	0	0	0	0
ENSMUSG00000020335	95	120	60	87	83	98
ENSMUSG00000028148	0	0	0	0	0	0
ENSMUSG00000022798	0	0	0	0	0	0
ENSMUSG00000022790	19	16	32	15	31	30
ENSMUSG00000112246	0	0	0	0	0	0
ENSMUSG00000102765	1	3	0	0	0	0
ENSMUSG00000112244	0	0	0	0	0	0
ENSMUSG00000029819	0	0	0	0	0	0
ENSMUSG00000102761	0	0	0	0	0	0
ENSMUSG00000112240	1	0	0	0	0	0
ENSMUSG00000102763	0	0	0	0	0	0
ENSMUSG00000029813	0	0	0	3	4	4
ENSMUSG00000029811	4	0	0	0	0	0
ENSMUSG00000102768	0	0	0	0	0	0
ENSMUSG00000102769	0	0	0	0	0	0
ENSMUSG00000112248	0	0	0	0	0	0
ENSMUSG00000112249	41	77	46	33	51	55
ENSMUSG00000062444	0	3	0	0	0	0
ENSMUSG00000024795	116	162	129	50	75	125
ENSMUSG00000060816	0	0	0	0	0	0
ENSMUSG00000024798	21	44	25	31	14	42
ENSMUSG00000028492	0	0	6	3	0	0
ENSMUSG00000112210	0	2	0	0	0	0
ENSMUSG00000039391	0	0	2	0	2	0
ENSMUSG00000102735	0	3	4	0	3	0
ENSMUSG00000039396	35	23	54	0	13	29
ENSMUSG00000039032	0	0	0	0	0	0

ENSMUSG00000105411	0	0	0	0	0	0
ENSMUSG00000105410	0	0	0	0	0	0
ENSMUSG00000105416	1	1	2	3	3	0
ENSMUSG00000105419	26	22	37	36	43	64
ENSMUSG00000105418	0	0	0	0	0	0
ENSMUSG00000110089	0	3	0	0	2	0
ENSMUSG00000110082	0	0	0	0	0	0
ENSMUSG00000110080	7	0	0	0	0	7
ENSMUSG00000110081	0	0	0	2	0	0
ENSMUSG00000113194	0	0	0	0	0	0
ENSMUSG00000113193	0	0	0	0	0	0
ENSMUSG00000113192	3	0	0	0	0	0
ENSMUSG00000113199	0	0	0	0	0	0
ENSMUSG00000110809	0	0	0	0	0	0
ENSMUSG00000021175	19	9	24	0	0	6
ENSMUSG00000029369	0	0	0	0	0	0
ENSMUSG00000029361	12	17	24	6	18	22
ENSMUSG00000051758	0	0	0	2	0	0
ENSMUSG00000114358	0	0	0	0	0	0
ENSMUSG00000114356	0	0	0	0	0	0
ENSMUSG00000058052	0	0	0	0	0	0
ENSMUSG00000114352	0	0	0	7	0	0
ENSMUSG00000114353	0	0	0	0	0	0
ENSMUSG00000114350	0	0	0	0	0	0
ENSMUSG00000114351	0	0	0	0	0	0
ENSMUSG00000106853	0	0	0	0	0	0
ENSMUSG00000105271	0	0	0	0	0	0
ENSMUSG00000108262	0	0	0	0	0	0
ENSMUSG00000108261	0	0	0	0	0	0
ENSMUSG00000108265	1	0	0	0	0	0
ENSMUSG00000108264	0	0	0	0	0	0
ENSMUSG00000059106	0	0	0	0	0	0
ENSMUSG00000059105	0	0	0	0	0	0
ENSMUSG00000110018	4	0	6	0	1	6
ENSMUSG00000105279	0	0	0	0	0	0
ENSMUSG00000020491	175	139	143	121	131	175
ENSMUSG00000025468	0	3	5	7	0	0
ENSMUSG00000025461	8	7	4	0	3	0
ENSMUSG00000097714	9	8	7	0	6	2
ENSMUSG00000097717	0	2	0	0	0	0
ENSMUSG00000097710	0	0	0	0	0	0
ENSMUSG00000097711	6	5	9	7	8	12
ENSMUSG00000097712	29	20	55	13	14	18
ENSMUSG00000097713	11	7	15	15	3	6
ENSMUSG00000097719	0	0	0	0	0	0
ENSMUSG00000108487	0	0	0	0	0	0
ENSMUSG00000108485	0	0	0	0	0	0
ENSMUSG00000104455	0	0	0	0	0	0
ENSMUSG00000108483	0	0	0	0	0	0
ENSMUSG00000108482	0	0	0	0	0	0
ENSMUSG00000108481	0	2	0	0	0	0
ENSMUSG00000108480	0	0	0	0	0	0
ENSMUSG00000091712	59	57	48	65	66	115
ENSMUSG00000091713	0	0	0	0	0	0
ENSMUSG00000108488	0	0	0	0	0	0
ENSMUSG00000083940	0	0	0	0	0	0
ENSMUSG00000083941	0	0	0	0	0	0
ENSMUSG00000083942	0	0	0	0	0	0

ENSMUSG00000083944	0	0	0	0	0	0
ENSMUSG00000083945	0	0	0	0	0	0
ENSMUSG00000083946	0	0	0	0	0	0
ENSMUSG00000083947	0	0	0	0	0	0
ENSMUSG00000083948	0	0	0	0	0	0
ENSMUSG00000083949	0	0	0	0	0	0
ENSMUSG00000106426	0	0	0	0	3	0
ENSMUSG00000010066	35	39	47	57	62	47
ENSMUSG00000044359	0	0	0	0	0	0
ENSMUSG00000044352	4	6	5	11	13	26
ENSMUSG00000054702	8	8	9	0	2	0
ENSMUSG00000056706	0	0	0	0	0	0
ENSMUSG00000046262	0	0	0	0	0	0
ENSMUSG00000045083	0	2	0	0	0	0
ENSMUSG00000040726	0	0	0	0	0	0
ENSMUSG00000044681	0	0	0	0	0	2
ENSMUSG00000110507	0	0	0	0	0	0
ENSMUSG00000110506	0	0	0	0	0	0
ENSMUSG00000104451	0	0	0	0	0	0
ENSMUSG00000110503	0	0	0	0	0	0
ENSMUSG00000110502	0	0	0	0	0	0
ENSMUSG00000044689	24	5	46	8	0	6
ENSMUSG00000023433	0	3	0	0	0	0
ENSMUSG00000080419	0	1	0	0	0	0
ENSMUSG00000080415	0	0	0	0	0	0
ENSMUSG00000080413	1	1	1	1	2	1
ENSMUSG00000080412	0	0	0	0	0	0
ENSMUSG00000080411	1	0	0	0	0	0
ENSMUSG00000080410	0	0	0	0	0	0
ENSMUSG00000074928	0	0	0	0	0	0
ENSMUSG00000113974	0	0	0	0	4	0
ENSMUSG00000113973	0	1	0	0	0	3
ENSMUSG00000113972	0	0	0	0	0	0
ENSMUSG00000113971	0	1	1	0	1	0
ENSMUSG00000078972	0	0	0	0	0	0
ENSMUSG00000053287	0	0	0	0	0	0
ENSMUSG00000113979	0	0	0	0	0	0
ENSMUSG00000106428	0	0	0	0	0	0
ENSMUSG00000086810	6	0	0	0	0	9
ENSMUSG00000086817	0	0	0	0	0	0
ENSMUSG00000037469	0	0	0	0	0	0
ENSMUSG00000090396	0	0	0	0	0	0
ENSMUSG00000045559	0	0	0	0	0	0
ENSMUSG00000098541	0	0	0	0	0	0
ENSMUSG00000028804	0	2	0	3	6	3
ENSMUSG00000053868	0	0	0	0	0	0
ENSMUSG00000053863	0	0	0	0	0	0
ENSMUSG00000094678	3	0	0	0	0	2
ENSMUSG00000094679	0	0	0	0	0	0
ENSMUSG00000094670	0	0	0	0	0	0
ENSMUSG00000101788	0	0	0	0	0	0
ENSMUSG00000098741	0	0	0	0	0	0
ENSMUSG00000106622	0	0	0	1	0	0
ENSMUSG00000081458	0	0	0	0	0	0
ENSMUSG00000090975	0	0	0	0	0	0
ENSMUSG00000081450	0	0	0	0	0	0
ENSMUSG00000081452	7	2	3	5	7	4
ENSMUSG00000081455	29	23	17	19	24	30

ENSMUSG00000099709	0	0	0	0	0	0
ENSMUSG00000092538	1	0	0	0	0	0
ENSMUSG00000099701	2	0	0	0	0	0
ENSMUSG00000092532	0	0	0	0	0	0
ENSMUSG00000092530	0	0	0	0	0	0
ENSMUSG00000099704	0	0	0	0	0	0
ENSMUSG00000099707	0	0	0	0	0	0
ENSMUSG00000077572	0	0	0	0	0	0
ENSMUSG00000077576	0	0	0	0	0	0
ENSMUSG00000077579	0	0	0	0	0	0
ENSMUSG00000082228	0	0	0	0	0	0
ENSMUSG00000082229	2	0	2	5	0	0
ENSMUSG00000030017	0	0	0	0	0	0
ENSMUSG00000082222	0	0	0	0	0	0
ENSMUSG00000082227	0	0	0	0	0	0
ENSMUSG00000082225	0	0	0	0	0	0
ENSMUSG00000076190	0	0	0	0	0	0
ENSMUSG00000107985	0	0	0	0	0	0
ENSMUSG00000076199	0	0	0	0	0	0
ENSMUSG00000032528	50	38	73	46	62	66
ENSMUSG00000105899	0	0	0	0	0	0
ENSMUSG00000106626	0	0	0	0	0	0
ENSMUSG00000096480	1	0	0	1	1	1
ENSMUSG00000086105	0	10	6	0	0	0
ENSMUSG00000086104	0	0	0	0	0	0
ENSMUSG00000086103	0	0	0	0	3	4
ENSMUSG00000086109	0	2	4	0	0	5
ENSMUSG00000072437	48	91	90	49	42	66
ENSMUSG00000076222	0	0	0	0	0	0
ENSMUSG00000041649	53	36	12	16	32	27
ENSMUSG00000082489	0	0	0	0	0	0
ENSMUSG00000082486	0	0	0	0	0	0
ENSMUSG00000041644	0	0	2	2	2	0
ENSMUSG00000082485	0	0	0	0	0	0
ENSMUSG00000082482	0	0	0	0	0	0
ENSMUSG00000087473	0	0	0	0	0	0
ENSMUSG00000107988	0	0	0	0	0	0
ENSMUSG00000087649	0	0	0	0	0	1
ENSMUSG00000083570	0	0	0	0	0	0
ENSMUSG00000083573	0	0	0	0	0	0
ENSMUSG00000083572	2	0	0	0	0	0
ENSMUSG00000037542	0	0	2	0	0	0
ENSMUSG00000037541	12	18	6	5	10	12
ENSMUSG00000083576	0	0	0	0	0	0
ENSMUSG00000049112	66	41	29	19	63	41
ENSMUSG00000100806	0	0	0	0	0	0
ENSMUSG00000000394	0	0	0	0	0	0
ENSMUSG00000079414	3	8	14	6	0	4
ENSMUSG00000031870	2	0	0	4	4	2
ENSMUSG00000031872	52	33	62	22	47	34
ENSMUSG00000031876	0	0	0	0	0	4
ENSMUSG00000080889	0	0	0	0	0	0
ENSMUSG00000080880	6	3	3	5	2	4
ENSMUSG00000080884	0	0	0	0	0	0
ENSMUSG00000080886	0	0	0	0	0	0
ENSMUSG00000080887	0	0	0	0	0	0
ENSMUSG00000083681	0	2	0	0	0	0
ENSMUSG00000083680	0	0	0	0	0	0

ENSMUSG00000103079	4	0	0	4	0	0
ENSMUSG00000083685	0	0	0	0	0	0
ENSMUSG00000083684	0	0	0	1	0	0
ENSMUSG00000033730	91	78	122	12	13	12
ENSMUSG00000033737	0	2	0	0	0	0
ENSMUSG00000061336	2	3	0	2	0	0
ENSMUSG00000064675	0	0	0	0	0	0
ENSMUSG00000064677	0	0	0	0	0	0
ENSMUSG00000064671	1	0	2	0	1	0
ENSMUSG00000064670	0	0	0	0	0	0
ENSMUSG00000064673	0	0	0	0	0	0
ENSMUSG00000107279	0	0	0	0	0	2
ENSMUSG00000107278	47	46	49	36	39	61
ENSMUSG00000107270	0	0	0	0	0	0
ENSMUSG00000107272	0	0	0	0	0	2
ENSMUSG00000058030	0	0	0	0	0	0
ENSMUSG00000034402	0	0	0	0	0	0
ENSMUSG00000061489	3	0	0	0	0	0
ENSMUSG00000061486	4	3	2	8	11	19
ENSMUSG00000000938	0	0	0	0	0	0
ENSMUSG00000065102	0	0	0	0	0	0
ENSMUSG00000094319	0	0	0	0	0	0
ENSMUSG00000099089	0	0	0	0	0	0
ENSMUSG00000099086	0	0	0	0	0	0
ENSMUSG00000099087	0	0	0	0	0	0
ENSMUSG00000099085	0	0	0	0	0	0
ENSMUSG00000071089	0	6	6	0	6	0
ENSMUSG00000074261	0	4	6	0	0	6
ENSMUSG00000074268	0	0	0	0	0	0
ENSMUSG00000030830	45	45	107	21	49	52
ENSMUSG00000099808	0	0	0	0	0	0
ENSMUSG00000030834	0	0	0	0	0	0
ENSMUSG00000099806	0	0	0	0	0	0
ENSMUSG00000081869	0	0	0	0	0	0
ENSMUSG00000094313	0	0	0	0	0	0
ENSMUSG00000099802	0	0	0	0	0	0
ENSMUSG00000074486	0	0	0	0	0	0
ENSMUSG00000074483	0	0	1	0	0	0
ENSMUSG00000074480	54	25	46	18	22	24
ENSMUSG00000009248	2	0	0	0	5	0
ENSMUSG00000111153	0	0	0	0	0	0
ENSMUSG00000006574	48	9	52	30	46	74
ENSMUSG00000006570	0	0	0	0	0	0
ENSMUSG00000102358	0	0	0	0	0	0
ENSMUSG00000102355	0	0	0	0	0	0
ENSMUSG00000102354	0	0	0	0	0	0
ENSMUSG00000102350	0	0	0	0	0	0
ENSMUSG00000102353	3	4	9	4	7	7
ENSMUSG00000072884	0	0	7	7	3	4
ENSMUSG00000072888	0	0	0	0	0	0
ENSMUSG00000098027	0	0	0	0	0	0
ENSMUSG00000098025	0	0	0	0	0	0
ENSMUSG00000098023	0	0	0	0	0	0
ENSMUSG00000098022	137	143	100	123	108	139
ENSMUSG00000077903	0	0	0	0	0	0
ENSMUSG00000077902	0	0	0	0	0	0
ENSMUSG00000098029	0	0	0	0	0	0
ENSMUSG00000098028	1	0	0	0	0	1

ENSMUSG00000069755	229	218	191	137	174	232
ENSMUSG00000035923	0	0	0	0	0	0
ENSMUSG00000096029	1	0	1	1	2	0
ENSMUSG00000096028	0	0	0	0	0	0
ENSMUSG00000096024	0	0	0	0	0	0
ENSMUSG00000096027	1	0	0	1	1	0
ENSMUSG00000096021	0	0	0	0	0	0
ENSMUSG00000096023	0	0	0	0	0	0
ENSMUSG00000096022	0	1	2	0	0	0
ENSMUSG00000034031	2	1	0	1	1	1
ENSMUSG00000063225	0	0	0	0	0	0
ENSMUSG00000034039	0	0	0	0	0	0
ENSMUSG00000105284	0	0	0	0	0	1
ENSMUSG00000101492	0	0	0	0	0	0
ENSMUSG00000066767	0	0	0	0	0	0
ENSMUSG00000066760	0	0	0	0	2	2
ENSMUSG00000105283	0	3	2	0	6	3
ENSMUSG00000104219	0	0	0	0	0	0
ENSMUSG00000109701	0	0	0	0	0	0
ENSMUSG00000104211	10	10	0	4	0	2
ENSMUSG00000104216	0	0	0	0	0	0
ENSMUSG00000104215	0	0	0	0	0	0
ENSMUSG00000101196	0	0	0	0	0	0
ENSMUSG00000106889	0	0	0	0	0	0
ENSMUSG00000101194	0	0	0	0	0	0
ENSMUSG00000101195	0	0	0	0	0	0
ENSMUSG00000101192	0	0	0	0	0	0
ENSMUSG00000101193	0	0	0	0	0	0
ENSMUSG00000101190	0	0	0	0	0	0
ENSMUSG00000089486	0	0	0	0	0	0
ENSMUSG00000091556	0	0	0	0	0	0
ENSMUSG00000091557	0	0	0	0	0	0
ENSMUSG00000091550	4	3	2	3	5	2
ENSMUSG00000101198	0	0	0	0	0	0
ENSMUSG00000091553	0	0	0	0	0	0
ENSMUSG00000081358	0	0	0	0	0	0
ENSMUSG00000030351	20	35	58	26	31	50
ENSMUSG00000020961	616	525	636	387	509	579
ENSMUSG00000107536	0	0	0	0	0	0
ENSMUSG00000020963	5	1	3	0	0	2
ENSMUSG00000064992	0	0	0	0	0	0
ENSMUSG00000049018	0	0	0	0	0	0
ENSMUSG00000056544	0	0	0	0	0	0
ENSMUSG00000089489	0	0	0	0	0	0
ENSMUSG00000091080	0	0	0	0	0	0
ENSMUSG00000104964	6	10	10	8	9	8
ENSMUSG00000108251	0	0	0	0	0	0
ENSMUSG00000073947	0	0	0	0	0	0
ENSMUSG00000100245	0	0	0	0	0	0
ENSMUSG00000100244	0	0	0	0	0	0
ENSMUSG00000100247	5	0	0	0	0	0
ENSMUSG00000088182	0	0	0	0	0	0
ENSMUSG00000100241	0	0	0	0	0	0
ENSMUSG00000100240	0	0	0	0	0	0
ENSMUSG00000100243	0	0	0	0	0	0
ENSMUSG00000029875	13	16	11	26	17	34
ENSMUSG00000100249	0	0	0	0	0	0
ENSMUSG00000100248	0	0	0	0	0	0

ENSMUSG00000058244	0	2	0	0	0	0
ENSMUSG00000058248	16	34	56	27	19	30
ENSMUSG00000064333	0	0	0	0	0	0
ENSMUSG00000095705	0	0	0	0	0	0
ENSMUSG00000095704	1	2	2	0	1	0
ENSMUSG00000095706	0	0	0	0	0	0
ENSMUSG00000095703	0	0	0	0	0	0
ENSMUSG00000095702	0	0	0	0	0	0
ENSMUSG00000088459	0	0	2	0	0	0
ENSMUSG00000088458	0	0	0	0	0	0
ENSMUSG00000088450	2	0	0	0	0	0
ENSMUSG00000022613	0	0	2	0	3	0
ENSMUSG00000094074	0	0	0	0	0	0
ENSMUSG00000107734	2	0	3	0	0	2
ENSMUSG00000107735	0	0	0	0	0	0
ENSMUSG00000107737	0	0	0	0	0	0
ENSMUSG00000107730	0	0	0	0	0	0
ENSMUSG00000107731	0	0	0	0	0	0
ENSMUSG00000107732	0	0	0	0	0	0
ENSMUSG00000107738	0	0	0	0	0	0
ENSMUSG00000107739	0	0	0	0	0	2
ENSMUSG00000111927	0	0	0	0	0	0
ENSMUSG00000111924	0	0	0	0	0	0
ENSMUSG00000111925	0	0	0	0	0	0
ENSMUSG00000111922	0	3	3	0	0	3
ENSMUSG00000111923	2	0	0	0	4	0
ENSMUSG00000111920	6	0	3	0	0	6
ENSMUSG00000103603	0	0	0	0	0	2
ENSMUSG00000103608	0	0	0	0	0	0
ENSMUSG00000103609	2	3	0	2	4	8
ENSMUSG00000111928	3	3	6	3	1	3
ENSMUSG00000085640	0	0	0	0	0	0
ENSMUSG00000106295	0	0	0	0	2	1
ENSMUSG00000062892	2	0	0	0	0	0
ENSMUSG00000036598	0	0	0	2	0	2
ENSMUSG00000036596	19	32	21	0	2	7
ENSMUSG00000028758	6	0	4	0	2	0
ENSMUSG00000096297	0	0	0	0	0	0
ENSMUSG00000028753	0	0	0	1	0	0
ENSMUSG00000028751	0	4	0	0	0	7
ENSMUSG00000114300	0	0	0	0	0	0
ENSMUSG00000039215	0	0	0	0	0	0
ENSMUSG00000024619	0	0	0	0	0	0
ENSMUSG00000096290	0	0	0	0	0	0
ENSMUSG00000077270	0	0	0	0	0	0
ENSMUSG00000087053	0	0	4	0	0	0
ENSMUSG00000020102	132	130	345	471	352	573
ENSMUSG00000087051	0	2	0	0	0	0
ENSMUSG00000110329	0	0	0	0	0	0
ENSMUSG00000110328	0	0	0	0	0	0
ENSMUSG00000087056	0	0	0	0	0	0
ENSMUSG00000110324	0	0	0	0	0	0
ENSMUSG00000110323	0	0	0	0	0	0
ENSMUSG00000110322	0	0	0	0	0	0
ENSMUSG00000110321	0	0	0	0	0	0
ENSMUSG00000110320	0	2	2	0	0	0
ENSMUSG00000113784	0	0	0	0	0	0
ENSMUSG00000092042	0	0	0	0	0	0

ENSMUSG00000097037	0	0	0	0	0	0
ENSMUSG00000092040	0	0	0	0	0	0
ENSMUSG00000092047	0	0	0	0	0	0
ENSMUSG00000087054	0	0	0	0	0	0
ENSMUSG00000092045	0	0	0	0	0	1
ENSMUSG00000113783	0	0	0	0	0	0
ENSMUSG00000092048	3	0	0	0	0	0
ENSMUSG00000097039	93	99	132	28	46	73
ENSMUSG00000097038	0	0	0	0	0	0
ENSMUSG00000055150	50	54	85	43	73	69
ENSMUSG00000112786	0	0	1	0	0	0
ENSMUSG00000112783	0	2	0	2	0	0
ENSMUSG00000114837	2	0	0	0	0	0
ENSMUSG00000114833	0	8	0	0	0	0
ENSMUSG00000114832	0	0	0	0	0	0
ENSMUSG00000114831	0	0	0	0	0	0
ENSMUSG00000025058	76	99	86	16	30	33
ENSMUSG00000025056	0	0	0	0	0	0
ENSMUSG00000105141	0	0	0	0	0	0
ENSMUSG00000106957	0	0	0	0	0	0
ENSMUSG00000106955	0	0	0	0	0	0
ENSMUSG00000106954	0	0	0	0	0	0
ENSMUSG00000108380	0	0	0	0	0	0
ENSMUSG00000113437	0	0	0	0	0	0
ENSMUSG00000108382	0	0	0	0	0	0
ENSMUSG00000108383	0	0	0	0	0	0
ENSMUSG00000108384	0	0	0	0	0	0
ENSMUSG00000108385	0	0	0	0	0	0
ENSMUSG00000108387	0	0	0	0	0	0
ENSMUSG00000099507	0	0	0	0	0	0
ENSMUSG00000069258	0	0	0	0	0	0
ENSMUSG00000069259	0	0	0	0	0	0
ENSMUSG00000105602	0	0	0	0	0	0
ENSMUSG00000105603	0	0	0	0	0	0
ENSMUSG00000109381	0	0	0	0	0	0
ENSMUSG00000109385	0	0	0	0	0	0
ENSMUSG00000105605	0	0	0	0	0	0
ENSMUSG00000114781	1	0	1	0	0	0
ENSMUSG00000090427	0	5	0	0	0	0
ENSMUSG00000114783	0	0	0	0	0	0
ENSMUSG00000114784	10	8	7	0	2	0
ENSMUSG00000114787	3	0	3	2	3	3
ENSMUSG00000114786	0	0	0	0	0	0
ENSMUSG00000096344	0	0	0	0	0	0
ENSMUSG00000096345	0	0	0	0	0	0
ENSMUSG00000096347	0	0	0	0	0	0
ENSMUSG00000096340	0	0	0	0	0	0
ENSMUSG00000081948	0	0	0	0	0	0
ENSMUSG00000096342	0	0	0	0	0	0
ENSMUSG00000096348	0	0	0	0	0	0
ENSMUSG00000050478	0	2	0	0	0	0
ENSMUSG00000070368	0	0	0	0	2	10
ENSMUSG00000082975	0	0	0	0	0	0
ENSMUSG00000082973	0	0	0	0	0	0
ENSMUSG00000082970	0	0	0	0	0	0
ENSMUSG00000082971	2	0	0	0	0	0
ENSMUSG00000070360	0	0	0	0	0	0
ENSMUSG00000050473	0	0	0	2	0	3

ENSMUSG00000070366	0	0	0	0	0	0
ENSMUSG00000105142	0	0	0	0	0	0
ENSMUSG00000111498	6	2	0	3	3	7
ENSMUSG00000111499	0	0	0	0	0	0
ENSMUSG00000111494	0	0	0	0	0	0
ENSMUSG00000081765	0	0	0	0	0	0
ENSMUSG00000111493	0	0	0	0	0	0
ENSMUSG00000111490	7	11	10	3	4	3
ENSMUSG00000109924	0	0	0	0	0	0
ENSMUSG00000109922	0	0	0	0	0	0
ENSMUSG00000109921	0	0	0	0	0	0
ENSMUSG00000109920	0	0	0	0	0	0
ENSMUSG00000109929	0	0	0	0	0	0
ENSMUSG00000109928	0	3	0	0	0	0
ENSMUSG00000108032	0	0	0	0	0	0
ENSMUSG00000104553	0	0	0	0	0	0
ENSMUSG00000104555	0	0	0	0	0	0
ENSMUSG00000104556	0	5	0	3	0	3
ENSMUSG00000104558	0	0	1	0	0	0
ENSMUSG00000108038	0	0	0	0	0	0
ENSMUSG00000108039	0	0	0	0	0	3
ENSMUSG00000040866	5	0	3	0	0	0
ENSMUSG00000040867	23	5	21	4	6	6
ENSMUSG00000093839	0	0	0	0	0	0
ENSMUSG00000093831	1	0	0	0	0	3
ENSMUSG00000093830	0	0	0	0	0	0
ENSMUSG00000111144	0	0	0	0	0	0
ENSMUSG00000111146	0	0	0	0	0	0
ENSMUSG00000111147	0	0	0	2	0	0
ENSMUSG00000044526	0	0	0	0	0	0
ENSMUSG00000022144	15	7	4	12	7	5
ENSMUSG00000084505	0	0	0	0	0	0
ENSMUSG00000109909	9	8	15	14	12	13
ENSMUSG00000094506	0	0	0	0	0	1
ENSMUSG00000056162	3	0	0	0	0	0
ENSMUSG00000104801	65	75	66	51	58	53
ENSMUSG00000078212	0	0	0	0	0	2
ENSMUSG00000078213	0	0	0	0	0	0
ENSMUSG00000078210	0	0	0	0	0	0
ENSMUSG00000078214	0	0	0	0	0	0
ENSMUSG00000046016	0	0	0	0	0	0
ENSMUSG00000095064	0	0	0	0	0	0
ENSMUSG00000095065	0	0	0	0	0	0
ENSMUSG00000095066	0	0	0	0	0	0
ENSMUSG00000095060	0	0	0	0	0	0
ENSMUSG00000095063	0	0	0	0	0	0
ENSMUSG00000095069	1	1	0	0	0	1
ENSMUSG00000053129	0	0	0	0	0	0
ENSMUSG00000100074	0	0	0	0	0	0
ENSMUSG00000058662	0	0	0	0	0	0
ENSMUSG00000092876	0	0	0	0	0	0
ENSMUSG00000027368	13	19	23	11	8	6
ENSMUSG00000092879	0	0	0	0	0	0
ENSMUSG00000090588	4	0	0	0	0	0
ENSMUSG00000090585	1	2	5	5	1	3
ENSMUSG00000090581	0	0	0	0	0	0
ENSMUSG00000105227	0	0	0	0	0	0
ENSMUSG00000073043	0	0	0	0	0	0

ENSMUSG00000073045	0	0	0	0	0	0
ENSMUSG00000080609	1	0	0	0	0	0
ENSMUSG00000080607	0	0	0	0	0	0
ENSMUSG00000080600	0	0	0	0	0	0
ENSMUSG00000093581	0	0	0	0	0	0
ENSMUSG00000093580	0	0	0	0	0	0
ENSMUSG00000093582	0	0	0	0	0	0
ENSMUSG00000111858	9	6	0	0	0	6
ENSMUSG00000093587	5	5	7	11	18	17
ENSMUSG00000093589	0	0	0	0	0	0
ENSMUSG00000093588	0	0	0	0	0	0
ENSMUSG00000078962	1	0	2	0	0	0
ENSMUSG00000078964	0	0	0	0	0	0
ENSMUSG00000052749	20	28	42	13	14	43
ENSMUSG00000111857	4	0	2	2	4	5
ENSMUSG00000109116	0	0	0	0	0	0
ENSMUSG00000109117	4	0	0	0	0	0
ENSMUSG00000077293	0	0	0	0	0	0
ENSMUSG00000077291	0	0	0	0	0	0
ENSMUSG00000077290	0	0	0	0	0	0
ENSMUSG00000111850	0	0	0	0	0	0
ENSMUSG00000077295	0	0	0	0	0	0
ENSMUSG00000031241	0	0	0	0	0	0
ENSMUSG00000077299	0	0	0	0	0	0
ENSMUSG00000077298	0	0	0	0	0	0
ENSMUSG00000114919	0	0	0	0	0	0
ENSMUSG00000093059	0	0	0	0	0	0
ENSMUSG00000093056	0	0	0	0	0	0
ENSMUSG00000055010	0	0	0	0	0	0
ENSMUSG00000114915	0	0	0	0	0	0
ENSMUSG00000096241	0	0	0	0	0	0
ENSMUSG00000073658	0	7	0	0	0	0
ENSMUSG00000114912	0	0	0	0	0	0
ENSMUSG00000114913	0	0	0	0	0	0
ENSMUSG00000040413	0	0	0	0	0	0
ENSMUSG00000040412	0	0	0	0	0	0
ENSMUSG00000077435	0	0	0	0	0	0
ENSMUSG00000037979	6	11	11	0	15	10
ENSMUSG00000077433	0	0	0	0	0	0
ENSMUSG00000077430	0	0	0	0	0	0
ENSMUSG00000037973	0	0	0	0	0	0
ENSMUSG00000088546	0	0	0	0	0	0
ENSMUSG00000077439	0	0	0	0	0	0
ENSMUSG00000055210	8	18	13	2	10	13
ENSMUSG00000033080	0	0	0	0	0	0
ENSMUSG00000105308	0	0	0	0	0	0
ENSMUSG00000030196	0	0	0	0	0	0
ENSMUSG00000073959	0	2	0	0	0	0
ENSMUSG00000073950	0	0	0	0	0	0
ENSMUSG00000073951	0	0	0	0	0	0
ENSMUSG00000073952	4	0	0	0	0	0
ENSMUSG00000073953	3	1	0	0	0	0
ENSMUSG00000073954	0	0	0	0	0	0
ENSMUSG00000073955	0	0	0	0	0	0
ENSMUSG00000073956	0	0	0	0	0	0
ENSMUSG00000105307	0	3	3	0	0	0
ENSMUSG00000071553	0	0	0	0	0	0
ENSMUSG00000071550	7	7	3	0	6	0

ENSMUSG00000071551	177	163	119	125	134	110
ENSMUSG00000095914	0	0	0	0	0	0
ENSMUSG00000099518	1	1	1	0	0	0
ENSMUSG00000099511	0	0	0	0	0	0
ENSMUSG00000099513	0	0	0	0	0	0
ENSMUSG00000099514	0	0	0	0	0	0
ENSMUSG00000099516	0	0	0	0	0	0
ENSMUSG00000083729	0	0	0	0	0	2
ENSMUSG00000102019	0	0	0	0	0	0
ENSMUSG00000083720	0	0	0	0	0	0
ENSMUSG00000083721	0	0	0	0	0	0
ENSMUSG00000083722	0	0	0	0	0	0
ENSMUSG00000083725	4	0	4	0	0	8
ENSMUSG00000043850	0	0	0	0	6	0
ENSMUSG00000043857	50	32	53	2	0	10
ENSMUSG00000043855	2	0	0	0	0	0
ENSMUSG00000043859	0	0	0	0	0	0
ENSMUSG00000100003	0	0	0	0	0	0
ENSMUSG00000100001	0	0	0	0	0	0
ENSMUSG00000100000	0	0	0	0	0	0
ENSMUSG00000072601	0	0	0	0	0	0
ENSMUSG00000100004	2	0	0	0	0	0
ENSMUSG00000070601	0	8	0	0	0	0
ENSMUSG00000070606	0	0	0	0	0	0
ENSMUSG00000102538	0	3	2	0	0	0
ENSMUSG00000102535	0	0	2	0	0	0
ENSMUSG00000102534	0	0	0	0	0	0
ENSMUSG00000102536	0	0	0	0	0	0
ENSMUSG00000102531	10	25	12	9	11	4
ENSMUSG00000102530	0	0	0	0	0	0
ENSMUSG00000102533	93	61	73	79	61	55
ENSMUSG00000102532	0	0	0	0	0	0
ENSMUSG00000075079	0	0	0	0	0	0
ENSMUSG00000075078	0	3	0	0	0	0
ENSMUSG00000084515	0	0	0	0	0	0
ENSMUSG00000084512	0	2	0	0	2	4
ENSMUSG00000084513	0	0	0	0	0	0
ENSMUSG00000084511	0	1	0	0	0	0
ENSMUSG00000075073	0	0	0	0	0	0
ENSMUSG00000075072	0	0	0	0	0	0
ENSMUSG00000075075	0	0	0	2	0	0
ENSMUSG00000075074	0	0	0	0	0	0
ENSMUSG00000084518	2	0	2	0	0	0
ENSMUSG00000000215	0	0	0	0	0	0
ENSMUSG00000000214	0	0	0	0	0	0
ENSMUSG00000000216	0	0	0	0	0	0
ENSMUSG00000065807	0	0	0	0	0	0
ENSMUSG00000065803	0	0	0	0	0	0
ENSMUSG00000065802	0	0	0	0	0	0
ENSMUSG00000088621	0	0	0	0	0	0
ENSMUSG00000074388	0	0	0	0	3	0
ENSMUSG00000075383	0	0	0	0	0	0
ENSMUSG00000075382	0	0	0	2	0	0
ENSMUSG00000079600	0	0	0	0	0	0
ENSMUSG00000075380	0	0	0	0	0	0
ENSMUSG00000075387	5	0	0	0	0	0
ENSMUSG00000079604	0	0	0	0	0	3
ENSMUSG00000075384	0	0	0	0	0	0

ENSMUSG00000088518	0	0	0	0	0	0
ENSMUSG00000101848	0	0	0	0	0	0
ENSMUSG00000077829	0	0	0	0	0	0
ENSMUSG00000101844	0	0	0	0	0	0
ENSMUSG00000088510	0	0	0	0	0	0
ENSMUSG00000035020	4	5	0	0	11	6
ENSMUSG00000107803	0	0	0	0	0	0
ENSMUSG00000088517	0	0	0	0	0	0
ENSMUSG00000101842	0	0	0	0	0	0
ENSMUSG00000101843	0	1	0	0	0	0
ENSMUSG00000085399	46	40	39	24	43	36
ENSMUSG00000085394	0	0	0	0	0	0
ENSMUSG00000085393	0	4	0	0	0	0
ENSMUSG00000085390	0	0	0	0	0	0
ENSMUSG00000033502	298	281	335	200	244	284
ENSMUSG00000033501	0	0	0	0	0	0
ENSMUSG00000103723	2	4	0	3	0	0
ENSMUSG00000103727	3	10	2	0	0	3
ENSMUSG00000103725	0	0	0	0	0	0
ENSMUSG00000065373	0	0	0	0	0	0
ENSMUSG00000065372	0	0	0	3	1	0
ENSMUSG00000065371	0	0	1	0	3	2
ENSMUSG00000103728	0	0	0	0	0	0
ENSMUSG00000024459	4	0	12	0	0	0
ENSMUSG00000078554	0	0	0	0	0	0
ENSMUSG00000028593	0	0	0	0	0	0
ENSMUSG00000084611	6	8	3	7	8	9
ENSMUSG00000030976	0	0	0	0	0	0
ENSMUSG00000087504	0	0	0	0	0	0
ENSMUSG00000073565	0	4	0	0	0	3
ENSMUSG00000097236	21	33	29	33	22	52
ENSMUSG00000073568	0	0	0	0	0	2
ENSMUSG00000082076	0	0	0	3	0	0
ENSMUSG00000035226	43	59	69	60	42	73
ENSMUSG00000039760	3	0	5	4	0	0
ENSMUSG00000089642	0	0	0	0	0	0
ENSMUSG00000089645	2	7	4	2	4	6
ENSMUSG00000100826	9	3	11	7	11	3
ENSMUSG00000100821	0	0	0	0	0	0
ENSMUSG00000100820	0	0	0	0	0	0
ENSMUSG00000097584	0	0	0	0	0	0
ENSMUSG00000102092	0	0	0	0	0	0
ENSMUSG00000102090	0	0	0	0	0	0
ENSMUSG00000102096	0	0	0	0	0	0
ENSMUSG00000102095	0	0	0	11	7	7
ENSMUSG00000062342	0	0	0	0	0	0
ENSMUSG00000102098	0	11	0	5	22	17
ENSMUSG00000102099	0	0	0	0	0	0
ENSMUSG00000062345	0	2	2	0	0	0
ENSMUSG00000096182	0	0	0	0	0	0
ENSMUSG00000096183	0	0	0	0	0	0
ENSMUSG00000096181	0	0	0	0	0	0
ENSMUSG00000096186	0	0	0	0	0	0
ENSMUSG00000096184	1	0	0	0	0	0
ENSMUSG00000096185	0	0	0	0	0	0
ENSMUSG00000096189	0	0	0	0	0	0
ENSMUSG00000113946	2	2	4	1	6	0
ENSMUSG00000113947	0	4	0	0	0	0

ENSMUSG00000113944	0	0	0	0	0	0
ENSMUSG00000101315	0	0	0	0	0	0
ENSMUSG00000057228	0	0	2	0	0	0
ENSMUSG00000113943	0	0	0	0	0	0
ENSMUSG00000096109	3	0	0	0	0	0
ENSMUSG00000105372	0	0	0	0	0	0
ENSMUSG00000105373	2	3	4	2	4	3
ENSMUSG00000105370	0	1	0	0	0	2
ENSMUSG00000067331	0	0	0	0	0	0
ENSMUSG00000105377	0	2	3	1	5	0
ENSMUSG00000105375	0	0	0	0	0	0
ENSMUSG00000110748	0	0	0	0	0	0
ENSMUSG00000029576	146	133	207	63	65	71
ENSMUSG00000108630	0	0	0	2	2	4
ENSMUSG00000055811	0	0	0	0	0	0
ENSMUSG00000108634	23	12	25	23	20	30
ENSMUSG00000050671	2	5	4	2	8	0
ENSMUSG00000108638	0	0	0	0	0	0
ENSMUSG00000108639	0	0	0	0	0	0
ENSMUSG00000109092	0	0	0	0	0	0
ENSMUSG00000067591	0	5	0	0	0	0
ENSMUSG00000021240	313	312	397	237	255	319
ENSMUSG00000067594	0	0	0	0	0	0
ENSMUSG00000067597	0	2	0	0	0	0
ENSMUSG00000105959	0	0	0	0	0	0
ENSMUSG00000105955	0	0	0	0	0	0
ENSMUSG00000105324	1	0	0	0	0	0
ENSMUSG00000057021	0	0	0	0	0	0
ENSMUSG00000101850	0	0	0	0	0	0
ENSMUSG00000104029	0	0	0	0	2	2
ENSMUSG00000101019	0	0	0	0	0	0
ENSMUSG00000103949	0	1	0	1	0	0
ENSMUSG00000104021	0	0	0	0	0	0
ENSMUSG00000101012	0	0	0	0	0	0
ENSMUSG00000101011	0	0	0	0	0	0
ENSMUSG00000104022	0	0	0	0	0	5
ENSMUSG00000104025	6	0	1	0	0	2
ENSMUSG00000094599	0	0	0	0	0	0
ENSMUSG00000101015	0	0	0	0	0	0
ENSMUSG00000103947	2	0	0	0	0	0
ENSMUSG00000094598	0	0	0	0	0	0
ENSMUSG00000039714	15	9	13	0	0	4
ENSMUSG00000103944	0	0	0	0	0	0
ENSMUSG00000103943	0	0	0	0	0	0
ENSMUSG00000010435	0	0	0	0	0	0
ENSMUSG00000094593	0	1	0	0	0	1
ENSMUSG00000094590	0	5	0	0	0	0
ENSMUSG00000094597	2	0	0	0	0	0
ENSMUSG00000094595	46	35	46	14	10	21
ENSMUSG00000103245	1	0	0	0	0	0
ENSMUSG00000059411	0	2	3	1	0	0
ENSMUSG00000059410	0	0	0	0	0	0
ENSMUSG00000064818	0	0	0	0	0	0
ENSMUSG00000091325	0	0	0	0	0	0
ENSMUSG00000103240	0	0	0	0	0	0
ENSMUSG00000103242	0	2	0	0	0	0
ENSMUSG00000110958	0	0	0	0	0	0
ENSMUSG00000110956	0	0	0	0	0	0

ENSMUSG00000110957	0	0	0	0	0	1
ENSMUSG00000110951	0	0	0	0	0	0
ENSMUSG00000110952	0	0	0	0	0	0
ENSMUSG00000110953	0	0	0	0	0	0
ENSMUSG00000056089	0	0	0	0	0	0
ENSMUSG00000095518	0	0	0	0	0	0
ENSMUSG00000090742	0	0	0	0	0	0
ENSMUSG00000090740	0	0	0	0	0	0
ENSMUSG00000095510	0	0	0	0	0	0
ENSMUSG00000090747	0	0	0	0	0	0
ENSMUSG00000090744	0	0	0	0	0	0
ENSMUSG00000036760	0	0	0	0	0	0
ENSMUSG00000042279	0	0	1	0	3	0
ENSMUSG00000112086	0	2	0	0	0	0
ENSMUSG00000112087	0	0	0	0	0	0
ENSMUSG00000112089	0	6	0	2	1	1
ENSMUSG00000034783	2	0	0	0	0	0
ENSMUSG00000086445	0	0	0	0	0	0
ENSMUSG00000086443	0	0	0	0	0	0
ENSMUSG00000086441	0	0	0	0	0	0
ENSMUSG00000086449	10	0	8	6	3	0
ENSMUSG00000105487	0	0	0	0	0	0
ENSMUSG00000096818	2	0	0	3	0	0
ENSMUSG00000109180	0	0	0	0	0	0
ENSMUSG00000108798	0	0	0	0	0	0
ENSMUSG00000108796	0	0	0	0	0	0
ENSMUSG00000060057	0	0	0	0	0	0
ENSMUSG00000111316	0	0	0	0	0	0
ENSMUSG00000108794	0	0	0	0	0	0
ENSMUSG00000093211	0	0	0	0	0	0
ENSMUSG00000103754	9	8	4	7	4	10
ENSMUSG00000051885	0	0	0	0	0	0
ENSMUSG00000096810	14	16	9	11	5	6
ENSMUSG00000109188	0	0	0	0	0	0
ENSMUSG00000103752	0	0	0	0	0	0
ENSMUSG00000104471	0	0	0	0	0	0
ENSMUSG00000052504	40	21	24	8	10	9
ENSMUSG00000036251	0	0	0	0	0	0
ENSMUSG00000065309	0	0	0	0	0	1
ENSMUSG00000052508	0	0	0	0	0	0
ENSMUSG00000028386	6	7	10	11	4	15
ENSMUSG00000097914	15	21	6	10	9	12
ENSMUSG00000097911	20	19	4	9	4	10
ENSMUSG00000112624	0	0	0	0	0	0
ENSMUSG00000112625	0	0	0	0	0	0
ENSMUSG00000112626	0	0	0	0	0	0
ENSMUSG00000112620	0	0	0	0	3	0
ENSMUSG00000112622	3	5	0	3	1	4
ENSMUSG00000112623	0	0	2	0	0	0
ENSMUSG00000049362	19	12	10	12	18	5
ENSMUSG00000112180	0	0	0	0	0	0
ENSMUSG00000112628	0	0	0	0	0	0
ENSMUSG00000073973	5	2	2	2	2	6
ENSMUSG00000054905	0	0	0	0	0	0
ENSMUSG00000047356	0	0	0	0	0	0
ENSMUSG00000047352	0	2	0	0	0	0
ENSMUSG00000109229	0	0	0	0	0	0
ENSMUSG00000003309	0	0	0	0	0	0

ENSMUSG00000109223	0	0	0	0	0	0
ENSMUSG00000051262	7	8	6	3	10	15
ENSMUSG00000109226	0	0	0	0	0	0
ENSMUSG00000109227	0	0	0	0	0	0
ENSMUSG00000045394	0	0	3	0	0	0
ENSMUSG00000106566	0	0	0	0	0	0
ENSMUSG00000106564	0	0	0	0	0	0
ENSMUSG00000106569	0	0	0	0	0	0
ENSMUSG00000114668	0	0	0	0	0	0
ENSMUSG00000114669	0	0	0	0	0	0
ENSMUSG00000114666	0	0	0	0	0	0
ENSMUSG00000114667	0	0	0	0	0	0
ENSMUSG00000114664	13	3	14	14	10	14
ENSMUSG00000114665	8	8	6	0	21	17
ENSMUSG00000114663	0	0	0	0	0	0
ENSMUSG00000114660	16	39	24	20	25	28
ENSMUSG00000110178	0	0	0	0	0	0
ENSMUSG00000110179	4	6	0	10	10	15
ENSMUSG00000110176	0	0	0	0	0	0
ENSMUSG00000110174	0	0	0	0	0	0
ENSMUSG00000110172	1	0	3	0	0	0
ENSMUSG00000110171	0	0	0	0	0	0
ENSMUSG00000101093	0	0	0	0	0	0
ENSMUSG00000070448	0	0	0	0	0	0
ENSMUSG00000108177	0	0	0	0	0	0
ENSMUSG00000108174	0	2	0	0	0	0
ENSMUSG00000108172	0	0	0	0	0	0
ENSMUSG00000091601	0	0	0	0	0	0
ENSMUSG00000108178	0	0	0	0	0	0
ENSMUSG00000105032	0	0	0	0	0	0
ENSMUSG00000105035	0	0	0	2	0	1
ENSMUSG00000105034	0	0	0	0	0	0
ENSMUSG00000105037	0	0	0	0	0	0
ENSMUSG00000105036	0	0	0	0	0	0
ENSMUSG00000105039	0	0	0	0	0	0
ENSMUSG00000105038	0	0	0	0	0	0
ENSMUSG00000101094	0	0	0	0	0	0
ENSMUSG00000041219	121	183	247	67	104	97
ENSMUSG00000102203	0	0	0	0	0	0
ENSMUSG00000091453	0	0	0	0	0	0
ENSMUSG00000044199	20	18	32	24	23	30
ENSMUSG00000083250	0	0	0	1	0	0
ENSMUSG00000083251	0	0	0	0	0	0
ENSMUSG00000083252	0	0	0	0	0	0
ENSMUSG00000083253	0	0	0	0	0	0
ENSMUSG00000083254	0	0	0	0	0	0
ENSMUSG00000083255	0	0	0	0	0	0
ENSMUSG00000083256	0	0	0	0	0	1
ENSMUSG00000083257	4	0	0	2	0	0
ENSMUSG00000083258	3	2	6	4	9	5
ENSMUSG00000085158	0	0	0	0	0	0
ENSMUSG00000085155	0	0	0	0	0	0
ENSMUSG00000085157	0	0	0	0	0	0
ENSMUSG00000085150	0	0	0	0	2	0
ENSMUSG00000085152	3	5	0	2	0	0
ENSMUSG00000040138	0	0	0	0	0	0
ENSMUSG00000079378	0	0	0	0	0	0
ENSMUSG00000079379	0	0	0	0	0	0

ENSMUSG00000079374	0	0	0	0	0	0
ENSMUSG00000041961	266	352	310	314	333	396
ENSMUSG00000041966	275	630	303	544	435	286
ENSMUSG00000088779	0	0	0	0	0	0
ENSMUSG00000074639	0	0	0	0	0	0
ENSMUSG00000083871	0	0	0	0	0	0
ENSMUSG00000083872	0	0	0	0	0	0
ENSMUSG00000083873	0	0	0	0	0	0
ENSMUSG00000109723	0	0	0	0	0	0
ENSMUSG00000053375	2	0	0	0	0	0
ENSMUSG00000027444	0	0	0	0	0	0
ENSMUSG00000027443	0	0	0	0	0	0
ENSMUSG00000027442	0	0	0	0	0	3
ENSMUSG00000092931	0	0	0	0	0	0
ENSMUSG00000110076	0	0	0	0	0	0
ENSMUSG00000078025	0	0	1	0	0	0
ENSMUSG00000078024	0	0	0	0	0	0
ENSMUSG00000092939	0	1	0	0	0	1
ENSMUSG00000078026	0	0	0	0	0	0
ENSMUSG00000110072	0	0	0	0	0	0
ENSMUSG00000090265	0	0	3	0	0	0
ENSMUSG00000090268	0	0	0	0	0	0
ENSMUSG00000098476	0	0	0	0	0	0
ENSMUSG00000098471	0	0	0	0	0	0
ENSMUSG00000098472	0	0	0	0	0	0
ENSMUSG00000098478	0	0	0	0	0	0
ENSMUSG00000098479	0	0	1	0	1	0
ENSMUSG00000094623	0	0	0	0	0	0
ENSMUSG00000046607	24	22	3	37	27	11
ENSMUSG00000046160	0	0	0	0	0	0
ENSMUSG00000048559	0	0	0	0	0	0
ENSMUSG00000095839	0	0	0	2	0	0
ENSMUSG00000105464	0	0	0	0	0	0
ENSMUSG00000034829	0	0	6	0	0	0
ENSMUSG00000100109	0	0	0	0	0	0
ENSMUSG00000062082	4	0	25	0	0	0
ENSMUSG00000072100	0	0	0	0	0	0
ENSMUSG00000108853	0	0	0	0	0	0
ENSMUSG00000004894	0	0	0	0	0	0
ENSMUSG00000004892	5	0	13	3	4	9
ENSMUSG00000049605	0	3	0	0	0	0
ENSMUSG00000049604	0	0	0	0	0	0
ENSMUSG00000087148	0	0	0	0	0	0
ENSMUSG00000087149	0	0	0	0	0	0
ENSMUSG00000082398	0	0	0	2	0	0
ENSMUSG00000082396	3	9	5	9	2	8
ENSMUSG00000087145	0	0	0	0	0	0
ENSMUSG00000087146	0	2	0	0	0	0
ENSMUSG00000087147	0	0	0	0	0	3
ENSMUSG00000082392	0	0	0	0	0	0
ENSMUSG00000030789	47	63	143	22	27	18
ENSMUSG00000082390	0	0	0	0	0	0
ENSMUSG00000087143	98	114	97	51	39	62
ENSMUSG00000101259	0	0	0	0	0	0
ENSMUSG00000031789	29	29	19	22	32	43
ENSMUSG00000101251	0	0	0	0	0	0
ENSMUSG00000101250	0	0	0	0	0	0
ENSMUSG00000101253	0	0	0	0	0	0

ENSMUSG00000101252	0	0	0	0	0	0
ENSMUSG00000101255	0	0	0	0	0	0
ENSMUSG00000101254	0	0	0	0	0	0
ENSMUSG00000101257	6	6	0	10	17	32
ENSMUSG00000031780	10	2	27	0	0	0
ENSMUSG00000037833	21	30	41	14	36	46
ENSMUSG00000071454	158	254	211	133	130	134
ENSMUSG00000071452	0	0	0	0	0	0
ENSMUSG00000067860	0	0	0	0	0	0
ENSMUSG00000082649	0	0	0	0	0	0
ENSMUSG00000082644	0	0	0	0	0	0
ENSMUSG00000099770	0	0	0	0	0	0
ENSMUSG00000099771	1	0	0	0	1	0
ENSMUSG00000102596	0	0	0	0	0	0
ENSMUSG00000040601	0	0	0	0	0	0
ENSMUSG00000037780	0	0	0	0	0	0
ENSMUSG00000037784	444	274	427	253	204	264
ENSMUSG00000107965	2	0	0	0	0	0
ENSMUSG00000107964	0	0	2	0	0	2
ENSMUSG00000107967	0	0	0	0	0	0
ENSMUSG00000046130	0	0	0	0	0	0
ENSMUSG00000103099	0	0	0	0	1	0
ENSMUSG00000103095	0	0	0	0	0	0
ENSMUSG00000103097	0	0	0	0	0	0
ENSMUSG00000103096	2	0	2	0	0	0
ENSMUSG00000103091	0	0	0	0	0	0
ENSMUSG00000063240	0	0	0	0	0	0
ENSMUSG00000032496	2	0	0	0	5	0
ENSMUSG00000032494	0	0	5	0	3	0
ENSMUSG00000032493	3	0	0	0	0	0
ENSMUSG00000032491	60	65	65	39	18	25
ENSMUSG00000042854	176	129	136	139	129	195
ENSMUSG00000083116	9	12	0	15	16	20
ENSMUSG00000075220	0	0	0	0	0	0
ENSMUSG00000075221	2	2	2	0	0	4
ENSMUSG00000075222	2	1	1	6	0	0
ENSMUSG00000075223	0	0	2	0	2	6
ENSMUSG00000075224	50	46	31	37	19	63
ENSMUSG00000084728	0	0	0	0	0	0
ENSMUSG00000083119	0	0	0	0	0	0
ENSMUSG00000100610	0	0	0	0	0	0
ENSMUSG00000100611	0	0	0	0	0	0
ENSMUSG00000100616	0	0	0	0	0	0
ENSMUSG00000100617	0	0	0	0	0	0
ENSMUSG00000100614	0	0	0	0	0	0
ENSMUSG00000100618	0	0	0	0	0	0
ENSMUSG00000100619	0	0	0	0	0	0
ENSMUSG00000064748	0	0	0	0	0	0
ENSMUSG00000107341	0	0	0	0	0	0
ENSMUSG00000099049	0	0	0	0	0	0
ENSMUSG00000107344	0	0	0	0	0	0
ENSMUSG00000107346	0	0	0	0	0	0
ENSMUSG00000107349	0	0	0	0	0	0
ENSMUSG00000107348	0	0	0	0	0	0
ENSMUSG00000100818	0	0	0	0	0	0
ENSMUSG00000000402	0	0	4	0	2	0
ENSMUSG00000100819	8	9	3	2	2	7
ENSMUSG00000000409	6	13	13	0	2	0

ENSMUSG00000113114	0	0	0	0	0	0
ENSMUSG00000102080	1	1	3	7	2	0
ENSMUSG00000085450	0	0	0	0	0	0
ENSMUSG00000085451	0	0	0	0	0	0
ENSMUSG00000085453	0	0	0	0	0	3
ENSMUSG00000114532	0	0	0	0	0	0
ENSMUSG00000114533	0	1	0	0	0	0
ENSMUSG00000100812	0	0	0	0	0	0
ENSMUSG00000079031	0	0	0	0	0	0
ENSMUSG00000114531	4	8	5	0	0	2
ENSMUSG00000065533	0	0	0	0	0	0
ENSMUSG00000100814	0	0	0	0	0	0
ENSMUSG00000100816	0	0	0	0	0	0
ENSMUSG00000101633	0	0	0	0	0	0
ENSMUSG00000038676	17	15	25	28	44	49
ENSMUSG00000069044	131	1	0	0	0	102
ENSMUSG00000069041	32	17	26	32	43	46
ENSMUSG00000021464	85	72	90	19	44	40
ENSMUSG00000021469	2	5	5	0	8	23
ENSMUSG00000105752	0	0	0	0	0	0
ENSMUSG00000096193	0	0	0	0	0	0
ENSMUSG00000113115	0	0	0	0	0	0
ENSMUSG00000113116	0	0	0	0	0	0
ENSMUSG00000038331	13	23	25	17	10	16
ENSMUSG00000038330	0	0	0	0	0	0
ENSMUSG00000113112	0	0	0	0	0	0
ENSMUSG00000059279	0	3	0	0	0	0
ENSMUSG00000015443	0	0	0	0	0	0
ENSMUSG00000063713	0	0	0	0	0	0
ENSMUSG00000063715	0	0	0	0	0	0
ENSMUSG00000029674	180	180	225	186	221	261
ENSMUSG00000029673	562	414	404	499	502	565
ENSMUSG00000029671	5	0	0	0	0	0
ENSMUSG00000029678	0	0	0	0	0	0
ENSMUSG00000029679	0	0	0	0	5	0
ENSMUSG00000106762	0	0	0	0	0	0
ENSMUSG00000106763	5	2	0	12	4	5
ENSMUSG00000106764	0	3	0	3	2	0
ENSMUSG00000106768	0	0	0	0	0	0
ENSMUSG00000038591	0	0	0	0	0	0
ENSMUSG00000104722	0	0	0	1	0	0
ENSMUSG00000038598	4	0	0	0	0	0
ENSMUSG00000104726	0	0	0	0	0	0
ENSMUSG00000104727	0	0	0	0	0	0
ENSMUSG00000104725	0	0	0	0	2	1
ENSMUSG00000059187	0	0	0	0	0	0
ENSMUSG00000059189	0	0	0	0	0	0
ENSMUSG00000024173	4	0	0	0	0	0
ENSMUSG00000024171	0	0	0	0	0	0
ENSMUSG00000024176	52	34	37	6	11	14
ENSMUSG00000024174	221	231	238	122	160	157
ENSMUSG00000024175	0	0	0	5	4	5
ENSMUSG00000114464	0	0	0	0	0	0
ENSMUSG00000114465	0	0	0	0	0	0
ENSMUSG00000114466	0	0	0	0	0	0
ENSMUSG00000114460	0	0	0	0	0	0
ENSMUSG00000114461	0	0	0	0	0	0
ENSMUSG00000114462	0	0	0	0	0	0

ENSMUSG00000054258	2	2	0	0	0	0
ENSMUSG00000097794	0	0	0	0	0	0
ENSMUSG00000097795	7	6	12	4	0	9
ENSMUSG00000054256	0	0	6	0	0	5
ENSMUSG00000097797	32	17	34	2	11	13
ENSMUSG00000091827	0	0	0	0	0	0
ENSMUSG00000091821	0	1	0	0	0	2
ENSMUSG00000109750	1	5	1	2	2	2
ENSMUSG00000109752	0	0	0	0	0	0
ENSMUSG00000109753	0	0	0	0	0	0
ENSMUSG00000109756	0	0	0	0	0	0
ENSMUSG00000109757	0	0	0	0	0	0
ENSMUSG00000105237	0	0	0	0	0	0
ENSMUSG00000109759	0	0	0	0	0	0
ENSMUSG00000105235	0	0	0	0	0	0
ENSMUSG00000105234	0	0	0	0	0	0
ENSMUSG00000108933	0	0	0	0	0	0
ENSMUSG00000020419	0	0	0	0	0	0
ENSMUSG00000008153	0	9	0	4	0	0
ENSMUSG00000058631	0	0	0	0	0	0
ENSMUSG00000108409	0	0	0	0	0	0
ENSMUSG00000104186	0	0	0	0	0	0
ENSMUSG00000108406	0	0	0	0	0	0
ENSMUSG00000104185	0	0	0	0	0	0
ENSMUSG00000104182	0	0	0	0	0	0
ENSMUSG00000104183	4	2	3	2	4	5
ENSMUSG00000104180	0	0	0	0	0	0
ENSMUSG00000108400	0	0	0	0	0	0
ENSMUSG00000081296	0	0	0	0	0	1
ENSMUSG00000081297	0	0	0	0	0	1
ENSMUSG00000043673	6	20	0	10	5	10
ENSMUSG00000091794	0	0	0	0	0	0
ENSMUSG00000086135	0	0	0	0	0	0
ENSMUSG00000091792	0	0	0	0	0	0
ENSMUSG00000026009	8	9	15	8	7	5
ENSMUSG00000096447	0	0	0	0	0	0
ENSMUSG00000056782	0	0	0	0	0	0
ENSMUSG00000095239	0	0	0	0	0	0
ENSMUSG00000095238	0	0	0	0	0	0
ENSMUSG00000045005	307	293	321	245	331	358
ENSMUSG00000095234	0	0	0	0	0	0
ENSMUSG00000098825	2	6	0	0	2	2
ENSMUSG00000095232	0	0	0	0	0	0
ENSMUSG00000111236	0	0	0	0	0	0
ENSMUSG00000111234	0	0	0	0	0	0
ENSMUSG00000111235	0	0	0	0	0	0
ENSMUSG00000111232	0	0	0	0	0	0
ENSMUSG00000111233	0	0	0	0	0	0
ENSMUSG00000010601	6	4	0	0	0	0
ENSMUSG00000093942	3	3	2	3	6	5
ENSMUSG00000047535	0	0	0	0	0	0
ENSMUSG00000047534	87	68	68	22	59	56
ENSMUSG00000047531	0	0	0	0	0	0
ENSMUSG00000093948	0	0	0	0	0	0
ENSMUSG00000095491	0	0	0	0	0	0
ENSMUSG00000095490	0	0	0	0	0	0
ENSMUSG00000095499	2	1	2	2	1	1
ENSMUSG00000090466	0	0	0	0	0	0

ENSMUSG00000090465	1	0	2	0	1	1
ENSMUSG00000090462	0	0	0	0	0	0
ENSMUSG00000090461	0	0	0	0	0	0
ENSMUSG00000081724	0	0	0	0	0	0
ENSMUSG00000026873	51	53	41	59	62	92
ENSMUSG00000081721	38	47	54	51	35	45
ENSMUSG00000022101	0	4	2	0	0	0
ENSMUSG00000022103	12	4	17	17	31	36
ENSMUSG00000094548	0	0	0	0	0	0
ENSMUSG00000108876	0	0	0	0	0	0
ENSMUSG00000094545	0	0	0	0	0	0
ENSMUSG00000094540	0	0	0	0	0	0
ENSMUSG00000094542	0	0	0	0	0	0
ENSMUSG00000094543	0	0	0	0	0	0
ENSMUSG00000056128	0	0	0	0	0	0
ENSMUSG00000044847	338	331	435	283	325	353
ENSMUSG00000088310	0	0	0	0	0	0
ENSMUSG00000097899	0	0	0	0	3	0
ENSMUSG00000097898	0	0	0	0	0	0
ENSMUSG00000088311	0	0	0	0	0	0
ENSMUSG00000028264	4	4	0	0	0	0
ENSMUSG00000028262	19	6	7	0	0	0
ENSMUSG00000097896	0	0	0	0	0	0
ENSMUSG00000088317	0	0	0	0	0	0
ENSMUSG00000091970	0	0	0	0	0	0
ENSMUSG00000077640	0	0	0	0	0	0
ENSMUSG00000077641	0	0	0	0	0	0
ENSMUSG00000077642	0	0	0	0	0	0
ENSMUSG00000077643	0	0	0	0	0	0
ENSMUSG00000077644	0	0	0	0	0	0
ENSMUSG00000077645	0	0	0	0	0	0
ENSMUSG00000083501	0	0	0	0	0	0
ENSMUSG00000077648	0	1	0	0	0	0
ENSMUSG00000084283	0	0	0	1	0	0
ENSMUSG00000112171	0	0	0	0	0	0
ENSMUSG00000089182	0	0	0	0	0	0
ENSMUSG00000086189	8	0	0	0	0	0
ENSMUSG00000086188	28	21	14	19	32	24
ENSMUSG00000086187	0	0	0	3	0	7
ENSMUSG00000086185	0	0	0	0	0	0
ENSMUSG00000076118	0	0	0	0	1	0
ENSMUSG00000089183	0	0	0	0	0	0
ENSMUSG00000102456	0	0	0	0	0	0
ENSMUSG00000098695	0	0	0	0	0	0
ENSMUSG00000045824	0	0	0	0	0	0
ENSMUSG00000049191	151	142	246	81	102	179
ENSMUSG00000105943	0	0	0	0	0	0
ENSMUSG00000098691	0	1	0	0	0	0
ENSMUSG00000096854	3	10	5	1	1	1
ENSMUSG00000096856	144	150	136	161	148	167
ENSMUSG00000096850	39	34	45	6	9	13
ENSMUSG00000096851	0	0	0	1	0	0
ENSMUSG00000098876	0	0	0	0	0	0
ENSMUSG00000098877	0	1	0	0	0	0
ENSMUSG00000108083	0	0	0	0	0	0
ENSMUSG00000096858	3	0	0	0	0	0
ENSMUSG00000096859	0	0	0	0	0	0
ENSMUSG00000106245	0	0	0	0	0	0

ENSMUSG00000106243	0	2	0	1	0	0
ENSMUSG00000113077	0	0	0	0	0	0
ENSMUSG00000106248	0	0	0	0	0	2
ENSMUSG00000022097	0	0	0	0	0	0
ENSMUSG00000047094	0	0	0	0	0	0
ENSMUSG00000088824	0	0	0	0	0	0
ENSMUSG00000004341	0	0	0	0	0	0
ENSMUSG00000088825	0	0	0	0	0	0
ENSMUSG00000004344	0	0	0	0	0	0
ENSMUSG00000109417	0	0	0	0	0	0
ENSMUSG00000109415	0	0	0	0	0	0
ENSMUSG00000109414	0	0	0	0	0	0
ENSMUSG00000109413	2	1	6	0	2	8
ENSMUSG00000109412	0	0	0	0	0	0
ENSMUSG00000109411	0	0	0	0	0	0
ENSMUSG00000109410	0	0	0	0	0	0
ENSMUSG00000109419	50	58	19	74	96	154
ENSMUSG00000109418	0	0	0	0	0	0
ENSMUSG00000082406	0	0	0	0	0	0
ENSMUSG00000082407	0	0	0	0	0	0
ENSMUSG00000082404	0	0	0	1	4	0
ENSMUSG00000082405	1	0	2	0	0	2
ENSMUSG00000082403	0	0	0	0	0	0
ENSMUSG00000070870	0	0	0	0	0	0
ENSMUSG00000070877	4	0	4	6	0	0
ENSMUSG00000082408	0	0	1	0	0	0
ENSMUSG00000070875	0	0	0	0	0	0
ENSMUSG00000005696	3	8	0	0	0	3
ENSMUSG00000040373	0	0	0	0	0	0
ENSMUSG00000084123	0	0	0	0	0	0
ENSMUSG00000084120	0	0	0	0	0	0
ENSMUSG00000084124	0	0	0	0	0	0
ENSMUSG00000087037	0	0	0	0	0	0
ENSMUSG00000103161	44	15	26	4	12	6
ENSMUSG00000080800	1	0	0	0	0	0
ENSMUSG00000080802	0	0	0	0	0	0
ENSMUSG00000080803	0	0	0	0	0	0
ENSMUSG00000080805	0	0	0	0	0	0
ENSMUSG00000080806	0	0	0	0	0	0
ENSMUSG00000080807	3	0	0	0	0	0
ENSMUSG00000080808	7	7	0	0	2	0
ENSMUSG00000080809	0	1	0	0	0	0
ENSMUSG00000099006	0	0	0	0	0	0
ENSMUSG00000099000	0	0	0	0	0	0
ENSMUSG00000099009	5	8	9	0	7	15
ENSMUSG00000022099	194	166	273	113	119	114
ENSMUSG00000041020	50	37	46	23	24	39
ENSMUSG00000082778	9	2	2	9	17	5
ENSMUSG00000044014	0	0	0	0	0	0
ENSMUSG00000104014	0	0	0	0	0	0
ENSMUSG00000085292	0	0	2	0	0	0
ENSMUSG00000085293	0	0	0	0	0	0
ENSMUSG00000085290	0	0	0	0	0	0
ENSMUSG00000101065	0	0	0	0	0	0
ENSMUSG00000109569	0	0	0	0	0	0
ENSMUSG00000085295	0	5	0	0	0	0
ENSMUSG00000111508	0	0	0	0	0	0
ENSMUSG00000062608	0	0	0	0	0	0

ENSMUSG00000109566	0	0	0	0	0	0
ENSMUSG00000104013	0	0	0	0	0	0
ENSMUSG00000079235	0	0	0	0	4	4
ENSMUSG00000074404	0	0	0	0	0	0
ENSMUSG00000078182	0	0	0	0	0	0
ENSMUSG00000074402	3	0	0	0	0	0
ENSMUSG00000074401	0	0	0	0	0	0
ENSMUSG00000114087	0	0	0	0	0	0
ENSMUSG00000033952	172	152	117	47	103	161
ENSMUSG00000073759	9	0	5	0	0	4
ENSMUSG00000071150	0	0	0	0	0	0
ENSMUSG00000084192	4	0	2	2	2	4
ENSMUSG00000077981	0	0	0	0	0	0
ENSMUSG00000077980	3	1	1	1	0	0
ENSMUSG00000111018	0	0	0	0	0	0
ENSMUSG00000106624	0	0	0	0	0	0
ENSMUSG00000099914	0	0	0	0	0	0
ENSMUSG00000099915	0	0	0	0	0	0
ENSMUSG00000099916	0	0	0	0	0	0
ENSMUSG00000099910	0	0	0	0	0	0
ENSMUSG00000106627	0	0	0	0	0	0
ENSMUSG00000099912	0	0	0	0	0	0
ENSMUSG00000099951	0	0	0	1	0	0
ENSMUSG00000099955	0	0	0	0	0	0
ENSMUSG00000099957	0	0	0	0	0	0
ENSMUSG00000031085	0	0	0	0	2	0
ENSMUSG00000031089	2	4	4	0	0	0
ENSMUSG00000074446	0	0	0	0	0	0
ENSMUSG00000103487	0	0	0	0	0	0
ENSMUSG00000048456	0	0	0	0	0	0
ENSMUSG00000048455	0	0	0	0	0	0
ENSMUSG00000038860	166	150	181	161	92	182
ENSMUSG00000049815	0	0	0	0	0	0
ENSMUSG00000077327	0	0	0	0	0	0
ENSMUSG00000077325	0	0	0	0	0	0
ENSMUSG00000077320	0	0	0	0	0	0
ENSMUSG00000077328	0	0	0	0	0	0
ENSMUSG00000096019	0	0	0	0	0	0
ENSMUSG00000084913	0	0	0	0	0	0
ENSMUSG00000104668	0	4	2	2	0	4
ENSMUSG00000093851	0	0	0	0	0	0
ENSMUSG00000031621	0	0	0	0	0	0
ENSMUSG00000103482	0	1	0	0	0	0
ENSMUSG00000102933	0	0	0	0	0	0
ENSMUSG00000102932	0	0	0	0	0	0
ENSMUSG00000102934	0	2	0	0	0	0
ENSMUSG00000102937	0	1	0	0	0	0
ENSMUSG00000102936	0	0	0	0	0	0
ENSMUSG00000071860	24	26	18	5	0	4
ENSMUSG00000015653	253	182	179	49	48	55
ENSMUSG00000015652	0	0	0	0	0	6
ENSMUSG00000043569	0	0	0	0	0	0
ENSMUSG00000087339	0	0	0	0	0	0
ENSMUSG00000087333	9	10	3	0	0	0
ENSMUSG00000087332	0	0	0	0	0	0
ENSMUSG00000088103	0	0	2	0	0	0
ENSMUSG00000088102	0	0	0	0	0	0
ENSMUSG00000088105	0	0	0	0	0	0

ENSMUSG00000087335	0	0	0	0	0	0
ENSMUSG00000087334	0	3	0	0	0	0
ENSMUSG00000068818	0	0	2	0	0	2
ENSMUSG00000096598	0	0	0	0	0	0
ENSMUSG00000100795	0	0	0	0	0	0
ENSMUSG00000100790	0	0	0	0	0	0
ENSMUSG00000082243	0	0	0	0	0	0
ENSMUSG00000100798	0	0	6	0	0	0
ENSMUSG00000040035	4	3	6	0	0	0
ENSMUSG00000101777	0	0	0	0	0	0
ENSMUSG00000101774	0	0	0	0	0	0
ENSMUSG00000101775	0	0	0	0	0	0
ENSMUSG00000101779	0	0	0	0	0	0
ENSMUSG00000059845	0	0	0	0	0	0
ENSMUSG00000113884	0	0	0	0	0	0
ENSMUSG00000093859	0	0	2	0	0	0
ENSMUSG00000103688	0	0	0	0	0	0
ENSMUSG00000103689	0	0	0	0	0	0
ENSMUSG00000103684	0	0	0	0	0	0
ENSMUSG00000103685	13	16	9	9	12	11
ENSMUSG00000103686	5	3	3	8	5	3
ENSMUSG00000103681	0	0	0	0	0	0
ENSMUSG00000100420	0	0	0	0	0	0
ENSMUSG00000100425	0	0	0	0	0	0
ENSMUSG00000100424	0	0	0	0	0	0
ENSMUSG00000100426	0	0	5	2	0	0
ENSMUSG00000012443	116	145	125	35	79	143
ENSMUSG00000022347	34	34	21	39	37	35
ENSMUSG00000022346	83	95	83	53	48	35
ENSMUSG00000022342	0	0	0	0	0	0
ENSMUSG00000020279	7	5	4	0	0	0
ENSMUSG00000095968	0	0	0	0	0	0
ENSMUSG00000089760	0	0	0	0	0	0
ENSMUSG00000089761	0	0	0	0	0	0
ENSMUSG00000089767	0	0	0	0	0	0
ENSMUSG00000095961	0	0	0	0	0	0
ENSMUSG00000020270	0	0	0	0	0	0
ENSMUSG00000095963	0	0	0	2	0	0
ENSMUSG00000095962	0	0	0	0	0	0
ENSMUSG00000095965	0	0	0	0	0	0
ENSMUSG00000095964	0	0	0	0	0	0
ENSMUSG00000075416	9	10	15	0	0	0
ENSMUSG00000065469	0	0	0	0	0	0
ENSMUSG00000028068	123	84	147	24	49	79
ENSMUSG00000028064	105	151	197	90	110	92
ENSMUSG00000102481	0	0	0	0	0	0
ENSMUSG00000102480	0	0	0	0	0	0
ENSMUSG00000102483	0	0	0	0	0	0
ENSMUSG00000102482	0	0	0	0	0	0
ENSMUSG00000102485	0	0	0	0	0	0
ENSMUSG00000102484	0	0	0	0	0	4
ENSMUSG00000102487	0	0	1	0	0	0
ENSMUSG00000102486	0	0	0	0	0	1
ENSMUSG00000102489	0	0	0	0	0	0
ENSMUSG00000102488	0	0	0	0	0	0
ENSMUSG00000024694	2	0	0	0	0	0
ENSMUSG00000020182	101	110	111	118	157	96
ENSMUSG00000020185	62	74	90	36	93	88

ENSMUSG00000102159	0	0	0	0	0	0
ENSMUSG00000102158	0	0	2	0	0	1
ENSMUSG00000112479	0	0	0	0	0	0
ENSMUSG00000112478	0	21	12	12	15	9
ENSMUSG00000112475	0	0	0	0	0	0
ENSMUSG00000102152	8	8	5	0	0	1
ENSMUSG00000112477	0	0	0	0	0	0
ENSMUSG00000039296	0	0	0	0	0	0
ENSMUSG00000112470	0	0	0	0	0	0
ENSMUSG00000102155	0	0	6	0	0	0
ENSMUSG00000110940	0	0	0	0	0	0
ENSMUSG00000088688	0	0	0	0	0	0
ENSMUSG00000088686	0	0	0	0	0	0
ENSMUSG00000088687	0	0	0	0	0	0
ENSMUSG00000088684	0	0	0	2	0	2
ENSMUSG00000088680	0	0	0	0	0	0
ENSMUSG00000021587	10	0	6	5	12	5
ENSMUSG00000105688	0	0	0	0	0	0
ENSMUSG00000105689	0	0	0	0	0	0
ENSMUSG00000100144	0	0	0	0	0	0
ENSMUSG00000105682	0	0	0	0	0	0
ENSMUSG00000105683	0	0	0	0	0	0
ENSMUSG00000021638	9	22	56	20	21	43
ENSMUSG00000105687	0	1	2	0	0	2
ENSMUSG00000105684	0	0	0	0	0	0
ENSMUSG00000105685	0	0	0	0	0	0
ENSMUSG00000024034	0	0	0	0	0	0
ENSMUSG00000024033	13	24	10	12	18	12
ENSMUSG00000113997	0	0	0	0	0	0
ENSMUSG00000024039	6	15	6	0	3	9
ENSMUSG00000106485	2	0	0	0	0	0
ENSMUSG00000106487	0	0	0	0	0	0
ENSMUSG00000106486	0	0	0	0	2	0
ENSMUSG00000106481	0	0	0	0	0	0
ENSMUSG00000113709	0	0	0	0	0	0
ENSMUSG00000106483	0	0	0	0	0	0
ENSMUSG00000113704	0	0	0	2	0	0
ENSMUSG00000113705	1	0	0	0	1	0
ENSMUSG00000088972	0	0	0	0	0	0
ENSMUSG00000113707	0	0	0	0	0	0
ENSMUSG00000113700	0	0	0	0	0	0
ENSMUSG00000106488	0	0	0	0	0	0
ENSMUSG00000088976	2	0	0	0	0	0
ENSMUSG00000113703	0	0	0	0	0	0
ENSMUSG00000078776	0	0	0	2	0	0
ENSMUSG00000092167	14	12	12	0	0	3
ENSMUSG00000111416	1	0	1	0	0	0
ENSMUSG00000111417	0	0	2	0	0	0
ENSMUSG00000111413	0	0	0	0	5	0
ENSMUSG00000111411	0	0	0	0	0	0
ENSMUSG00000111418	0	0	0	0	0	0
ENSMUSG00000111419	0	0	0	2	0	0
ENSMUSG00000108300	0	0	0	0	0	0
ENSMUSG00000062651	0	4	0	0	0	0
ENSMUSG00000108302	0	0	0	0	0	0
ENSMUSG00000108304	0	0	3	0	0	0
ENSMUSG00000108306	0	0	0	0	0	0
ENSMUSG00000108307	0	0	0	0	0	0

ENSMUSG00000108308	6	6	9	0	0	5
ENSMUSG00000059022	90	117	177	84	119	66
ENSMUSG00000059023	0	3	0	0	0	0
ENSMUSG00000056078	0	0	0	0	0	0
ENSMUSG00000059027	89	95	60	110	62	103
ENSMUSG00000111288	0	0	0	0	2	0
ENSMUSG00000009900	0	0	0	0	0	0
ENSMUSG00000067285	0	0	0	0	0	0
ENSMUSG00000097612	2	7	9	10	4	8
ENSMUSG00000097615	0	0	0	0	0	0
ENSMUSG00000097618	3	0	8	0	0	0
ENSMUSG00000091618	0	0	0	0	0	0
ENSMUSG00000105850	0	0	5	0	6	8
ENSMUSG00000099412	0	0	0	0	0	0
ENSMUSG00000095508	0	0	0	0	0	0
ENSMUSG00000091617	14	17	18	15	12	22
ENSMUSG00000091615	0	0	0	0	0	0
ENSMUSG00000091614	0	0	0	0	0	0
ENSMUSG00000095507	0	0	0	0	0	0
ENSMUSG00000070890	0	0	0	0	0	0
ENSMUSG00000067750	0	0	0	0	0	0
ENSMUSG00000033981	25	9	2	2	2	3
ENSMUSG00000070891	6	19	5	4	6	12
ENSMUSG00000114164	0	0	0	0	0	0
ENSMUSG00000114167	0	0	0	0	0	0
ENSMUSG00000114166	0	0	0	0	0	0
ENSMUSG00000114161	0	0	0	3	0	0
ENSMUSG00000114163	0	0	0	0	0	0
ENSMUSG00000114162	0	0	0	0	0	0
ENSMUSG00000044254	22	26	16	16	28	22
ENSMUSG00000090750	0	0	0	0	0	0
ENSMUSG00000054422	0	0	0	0	0	2
ENSMUSG00000027670	0	0	0	0	0	0
ENSMUSG00000050215	5	7	3	5	8	3
ENSMUSG00000027674	41	25	27	25	40	57
ENSMUSG00000110759	0	0	0	0	3	2
ENSMUSG00000104086	0	0	4	0	0	0
ENSMUSG00000110429	0	0	0	0	0	0
ENSMUSG00000110425	0	0	0	0	0	0
ENSMUSG00000110426	2	0	2	0	0	0
ENSMUSG00000110427	0	0	0	2	0	0
ENSMUSG00000110420	0	0	0	0	0	0
ENSMUSG00000110421	0	0	0	0	0	0
ENSMUSG00000080358	0	1	1	0	1	0
ENSMUSG00000080359	0	0	0	0	0	0
ENSMUSG00000080356	0	2	0	0	0	0
ENSMUSG00000104080	2	3	4	2	0	0
ENSMUSG00000080350	1	1	1	0	1	1
ENSMUSG00000046095	0	0	0	0	0	0
ENSMUSG00000001864	35	46	38	9	60	84
ENSMUSG00000046093	5	8	8	5	5	5
ENSMUSG00000114908	0	0	0	0	0	0
ENSMUSG00000113414	0	0	0	0	0	0
ENSMUSG00000080686	0	0	0	0	0	0
ENSMUSG00000080687	0	2	1	0	1	0
ENSMUSG00000106331	2	0	0	0	0	0
ENSMUSG00000080680	0	0	0	0	0	0
ENSMUSG00000090059	1	0	0	0	0	0

ENSMUSG00000094810	0	0	0	0	0	0
ENSMUSG00000090051	0	0	0	0	0	0
ENSMUSG00000090057	3	2	0	0	7	0
ENSMUSG00000113578	0	0	0	0	0	0
ENSMUSG00000098229	0	0	0	0	2	0
ENSMUSG00000098228	0	0	0	0	0	0
ENSMUSG00000098227	0	0	0	0	0	0
ENSMUSG00000093509	0	0	0	0	0	0
ENSMUSG00000094739	0	1	0	0	0	0
ENSMUSG00000094738	0	0	0	0	0	0
ENSMUSG00000042345	0	0	6	2	0	0
ENSMUSG00000042349	249	176	263	40	78	86
ENSMUSG00000094732	0	0	0	0	0	0
ENSMUSG00000094730	0	0	0	0	0	0
ENSMUSG00000093504	0	0	0	0	0	0
ENSMUSG00000093507	4	5	5	3	2	9
ENSMUSG00000094734	0	0	0	0	0	0
ENSMUSG00000112095	7	15	17	5	12	10
ENSMUSG00000112094	0	0	0	0	0	0
ENSMUSG00000113576	4	0	0	0	2	0
ENSMUSG00000052217	0	0	0	0	0	0
ENSMUSG00000081089	0	0	0	0	0	0
ENSMUSG00000081088	0	0	0	0	0	0
ENSMUSG00000052212	0	5	0	0	0	0
ENSMUSG00000081083	0	0	0	0	0	0
ENSMUSG00000081082	0	0	0	0	0	0
ENSMUSG00000081081	0	0	0	0	0	0
ENSMUSG00000081080	0	0	0	0	0	0
ENSMUSG00000081084	0	0	0	0	0	0
ENSMUSG00000081555	0	0	0	0	0	0
ENSMUSG00000081557	0	0	0	0	0	0
ENSMUSG00000081551	1	2	0	1	1	1
ENSMUSG00000048693	3	0	0	0	0	0
ENSMUSG00000048699	0	0	0	0	0	0
ENSMUSG00000086684	0	0	0	0	0	0
ENSMUSG00000086685	0	0	0	0	0	0
ENSMUSG00000086687	0	1	0	0	0	0
ENSMUSG00000086689	11	6	3	0	0	3
ENSMUSG00000099666	0	0	0	0	0	0
ENSMUSG00000099665	0	0	0	0	0	0
ENSMUSG00000099661	0	0	0	0	0	0
ENSMUSG00000099668	0	0	0	0	0	0
ENSMUSG00000099669	0	0	0	0	0	0
ENSMUSG00000112134	0	0	0	0	0	0
ENSMUSG00000112135	22	16	14	21	20	21
ENSMUSG00000112136	0	0	0	0	0	0
ENSMUSG00000112137	7	10	5	11	11	12
ENSMUSG00000112130	0	0	0	0	0	0
ENSMUSG00000112131	4	0	0	0	0	0
ENSMUSG00000112132	0	0	0	0	0	0
ENSMUSG00000112133	1	0	0	0	0	0
ENSMUSG00000112139	0	0	0	0	0	0
ENSMUSG00000030114	0	0	0	0	0	0
ENSMUSG00000030117	0	10	21	0	0	0
ENSMUSG00000030111	0	25	17	8	8	5
ENSMUSG00000030110	112	156	126	225	275	364
ENSMUSG00000105529	1	0	2	1	0	1
ENSMUSG00000092362	0	0	0	0	0	0

ENSMUSG00000092363	0	0	0	0	0	0
ENSMUSG00000099592	0	0	0	0	0	3
ENSMUSG00000099593	0	0	0	0	0	0
ENSMUSG00000099594	0	0	0	0	0	0
ENSMUSG00000099595	0	0	0	0	0	0
ENSMUSG00000099596	4	0	2	1	2	2
ENSMUSG00000099598	0	0	0	0	0	0
ENSMUSG00000099599	0	0	0	0	0	0
ENSMUSG00000092369	0	0	0	0	0	0
ENSMUSG00000100034	0	0	0	0	0	0
ENSMUSG00000055298	0	0	0	0	0	2
ENSMUSG00000051977	130	122	141	85	103	109
ENSMUSG00000016179	13	17	0	9	4	12
ENSMUSG00000003411	7	14	0	5	13	19
ENSMUSG00000043760	0	0	3	2	0	0
ENSMUSG00000086025	0	0	0	0	0	0
ENSMUSG00000086026	3	0	0	0	0	0
ENSMUSG00000086020	0	0	0	0	0	0
ENSMUSG00000086021	2	0	0	0	0	0
ENSMUSG00000086022	0	0	0	6	0	0
ENSMUSG00000094868	0	0	0	0	0	0
ENSMUSG00000086028	0	0	0	0	0	0
ENSMUSG00000076309	1	1	1	1	0	1
ENSMUSG00000041722	0	0	0	0	0	0
ENSMUSG00000072686	0	0	0	0	0	0
ENSMUSG00000076305	0	0	0	0	0	0
ENSMUSG00000072683	0	0	0	0	0	0
ENSMUSG00000076303	0	0	0	0	0	0
ENSMUSG00000076300	0	0	0	0	0	0
ENSMUSG00000005718	81	83	77	63	90	91
ENSMUSG00000064616	0	0	0	0	0	0
ENSMUSG00000070933	0	0	2	0	0	0
ENSMUSG00000005716	6	4	6	0	0	0
ENSMUSG00000037466	26	26	27	14	11	26
ENSMUSG00000037463	0	2	0	0	4	0
ENSMUSG00000088726	0	0	0	0	0	0
ENSMUSG00000087643	0	0	0	0	0	0
ENSMUSG00000087641	0	0	0	0	0	0
ENSMUSG00000087647	0	0	0	0	0	0
ENSMUSG00000087646	0	0	0	0	0	0
ENSMUSG00000070683	0	0	0	0	0	0
ENSMUSG00000087648	0	0	0	0	0	0
ENSMUSG00000070687	0	0	0	0	0	0
ENSMUSG00000070686	0	0	0	0	0	0
ENSMUSG00000040231	11	5	6	3	0	7
ENSMUSG00000095745	0	0	0	0	0	0
ENSMUSG00000087909	0	0	0	0	0	0
ENSMUSG00000064137	0	0	0	0	0	0
ENSMUSG00000107533	0	0	0	0	0	0
ENSMUSG00000107530	0	0	0	0	0	0
ENSMUSG00000071332	6	0	5	0	7	0
ENSMUSG00000093227	0	0	0	1	0	0
ENSMUSG00000046790	0	0	0	0	0	1
ENSMUSG00000065660	0	0	0	0	0	0
ENSMUSG00000061561	0	2	2	0	0	0
ENSMUSG00000065665	0	0	0	0	0	0
ENSMUSG00000085649	0	0	0	0	0	0
ENSMUSG00000085648	0	0	0	0	0	0

ENSMUSG00000085645	0	0	0	0	0	0
ENSMUSG00000085646	0	0	0	0	0	0
ENSMUSG00000085641	0	0	0	0	0	0
ENSMUSG00000085642	21	28	43	34	13	30
ENSMUSG00000074300	0	0	0	0	0	0
ENSMUSG00000074302	0	0	0	0	0	0
ENSMUSG00000081946	0	0	0	0	0	0
ENSMUSG00000081945	0	0	0	0	0	0
ENSMUSG00000081942	0	0	0	2	2	0
ENSMUSG00000081943	4	0	0	3	0	0
ENSMUSG00000081940	0	0	0	0	0	0
ENSMUSG00000081941	0	0	0	0	0	0
ENSMUSG00000109797	0	0	0	0	2	0
ENSMUSG00000062037	0	0	4	0	1	0
ENSMUSG00000062036	0	0	0	2	0	0
ENSMUSG00000111591	0	0	0	0	0	0
ENSMUSG00000068167	2	0	0	0	0	0
ENSMUSG00000073656	3	0	5	0	5	9
ENSMUSG00000073650	22	17	31	9	16	20
ENSMUSG00000111625	0	0	0	0	0	0
ENSMUSG00000102013	0	0	0	0	0	0
ENSMUSG00000102010	0	0	0	0	0	0
ENSMUSG00000102011	0	0	0	0	0	0
ENSMUSG00000102016	0	0	0	0	0	0
ENSMUSG00000102017	0	0	0	0	0	0
ENSMUSG00000102015	0	0	0	0	0	0
ENSMUSG00000109840	0	0	0	0	1	0
ENSMUSG00000098011	0	0	0	0	0	0
ENSMUSG00000098145	4	5	4	2	0	4
ENSMUSG00000098147	0	0	0	0	0	0
ENSMUSG00000098141	1	0	4	2	0	2
ENSMUSG00000098143	0	0	0	0	0	0
ENSMUSG00000109843	1	0	0	3	0	5
ENSMUSG00000098149	7	8	7	10	8	10
ENSMUSG00000109848	0	0	0	0	0	0
ENSMUSG00000096107	0	0	0	0	0	0
ENSMUSG00000103942	0	0	0	0	0	0
ENSMUSG00000034171	8	4	21	5	3	0
ENSMUSG00000034177	9	11	0	0	9	0
ENSMUSG00000019992	14	12	22	8	30	17
ENSMUSG00000082169	0	0	0	0	0	0
ENSMUSG00000082168	0	0	0	0	0	0
ENSMUSG00000111085	5	10	5	0	2	2
ENSMUSG00000084891	0	0	0	0	0	0
ENSMUSG00000084890	6	0	6	0	0	0
ENSMUSG00000066640	460	409	619	316	358	501
ENSMUSG00000084898	0	0	0	0	0	0
ENSMUSG00000111081	0	0	0	0	0	0
ENSMUSG00000091459	0	0	0	0	0	0
ENSMUSG00000101092	0	0	0	0	0	0
ENSMUSG00000101091	0	0	0	0	0	0
ENSMUSG00000101090	0	0	0	0	0	0
ENSMUSG00000101097	0	0	0	0	0	0
ENSMUSG00000101096	0	0	0	0	0	0
ENSMUSG00000101095	0	0	0	0	0	0
ENSMUSG00000050534	4	0	8	0	0	0
ENSMUSG00000091450	0	0	0	0	0	0
ENSMUSG00000101099	0	0	0	0	0	0

ENSMUSG00000101098	0	0	0	0	0	0
ENSMUSG00000091455	4	0	2	9	4	17
ENSMUSG00000067519	0	0	0	0	0	0
ENSMUSG00000057337	79	80	92	94	96	122
ENSMUSG00000067513	0	0	0	0	0	0
ENSMUSG00000114260	0	3	0	0	0	0
ENSMUSG00000112635	0	0	0	0	0	0
ENSMUSG00000101404	0	0	0	0	0	0
ENSMUSG00000113817	0	0	0	0	0	0
ENSMUSG00000108859	0	0	0	0	0	0
ENSMUSG00000108854	6	10	8	17	8	17
ENSMUSG00000104692	0	0	0	0	0	0
ENSMUSG00000108856	0	4	4	0	0	0
ENSMUSG00000108857	0	0	0	0	0	1
ENSMUSG00000064890	2	0	4	2	1	1
ENSMUSG00000108852	11	7	13	9	3	7
ENSMUSG00000101403	0	0	0	0	0	0
ENSMUSG00000105540	0	3	2	0	0	0
ENSMUSG00000101901	0	0	0	0	0	0
ENSMUSG00000112638	0	0	0	0	0	0
ENSMUSG00000101637	0	0	0	0	0	0
ENSMUSG00000101634	7	17	4	0	8	4
ENSMUSG00000056379	0	0	0	0	0	0
ENSMUSG00000101632	0	0	0	0	0	0
ENSMUSG00000101631	0	0	0	0	0	0
ENSMUSG00000101906	0	1	0	0	2	1
ENSMUSG00000056370	3	0	0	0	0	0
ENSMUSG00000101639	0	0	0	0	0	0
ENSMUSG00000101638	0	0	0	0	0	0
ENSMUSG00000100322	0	0	0	0	0	0
ENSMUSG00000061911	0	2	0	4	6	5
ENSMUSG00000100321	0	0	0	0	0	0
ENSMUSG00000100326	0	0	0	0	0	0
ENSMUSG00000100324	0	0	0	0	0	0
ENSMUSG00000100325	0	0	0	0	0	0
ENSMUSG00000100328	0	0	0	0	0	0
ENSMUSG00000100329	0	0	0	0	0	0
ENSMUSG00000058360	0	0	3	0	0	0
ENSMUSG00000058368	0	0	0	0	0	0
ENSMUSG00000102285	0	0	0	0	0	0
ENSMUSG00000026875	52	83	155	37	37	40
ENSMUSG00000026874	0	0	0	0	0	0
ENSMUSG00000088318	0	0	2	0	0	0
ENSMUSG00000082427	0	0	0	0	0	0
ENSMUSG00000107671	0	0	0	0	0	0
ENSMUSG00000107673	0	0	0	0	0	0
ENSMUSG00000107672	2	0	0	0	0	0
ENSMUSG00000107675	0	0	0	0	0	0
ENSMUSG00000107677	0	0	0	0	0	0
ENSMUSG00000107676	22	19	14	7	12	7
ENSMUSG00000107679	0	0	0	0	0	0
ENSMUSG00000107678	0	0	0	0	0	0
ENSMUSG00000103160	5	11	3	7	5	9
ENSMUSG00000103167	0	0	0	0	0	0
ENSMUSG00000103168	0	0	0	0	0	0
ENSMUSG00000061000	0	0	0	0	0	0
ENSMUSG00000102409	0	0	0	0	0	0
ENSMUSG00000103311	0	0	0	0	0	0

ENSMUSG00000058981	0	0	0	0	0	0
ENSMUSG00000089047	0	0	0	0	0	0
ENSMUSG00000089046	0	0	0	0	0	0
ENSMUSG00000102280	0	0	0	0	0	0
ENSMUSG00000089043	0	2	0	0	1	0
ENSMUSG00000089041	0	0	0	0	0	0
ENSMUSG00000089040	0	0	0	0	0	0
ENSMUSG00000089049	1	0	0	0	2	0
ENSMUSG00000106070	0	0	0	0	0	0
ENSMUSG00000106071	0	5	0	0	0	2
ENSMUSG00000106072	0	0	0	0	0	0
ENSMUSG00000106074	0	0	0	0	0	0
ENSMUSG00000106076	0	0	0	0	0	0
ENSMUSG00000106077	0	0	0	0	0	0
ENSMUSG00000106078	0	1	0	0	0	0
ENSMUSG00000106079	0	0	0	0	0	0
ENSMUSG00000094243	0	0	0	0	0	0
ENSMUSG00000094240	0	0	0	1	0	0
ENSMUSG00000094247	0	0	0	0	0	0
ENSMUSG00000018924	0	4	0	0	0	6
ENSMUSG00000018925	0	2	0	5	0	0
ENSMUSG00000020020	3	8	2	4	5	0
ENSMUSG00000110249	7	6	0	0	0	2
ENSMUSG00000110246	0	0	0	0	0	0
ENSMUSG00000051276	0	0	0	0	0	0
ENSMUSG00000110241	0	3	0	0	0	0
ENSMUSG00000092162	0	0	0	0	0	0
ENSMUSG00000092164	0	0	0	0	0	0
ENSMUSG00000092166	0	0	0	0	0	0
ENSMUSG00000109232	0	0	0	0	0	0
ENSMUSG00000004609	393	497	625	165	256	290
ENSMUSG00000003657	0	0	0	0	0	0
ENSMUSG00000035704	117	134	150	67	119	147
ENSMUSG00000109234	0	0	0	0	0	0
ENSMUSG00000106694	4	2	2	2	2	0
ENSMUSG00000092587	0	0	0	0	0	0
ENSMUSG00000075153	1	0	0	0	0	0
ENSMUSG00000106690	0	0	0	0	0	0
ENSMUSG00000106692	0	0	0	0	0	0
ENSMUSG00000113573	0	0	0	0	0	0
ENSMUSG00000113572	4	0	2	0	0	0
ENSMUSG00000038751	5	5	6	9	6	5
ENSMUSG00000113570	10	6	15	2	2	6
ENSMUSG00000038756	0	0	0	0	0	0
ENSMUSG00000038754	0	0	0	0	0	0
ENSMUSG00000069355	0	0	0	0	0	0
ENSMUSG00000094867	0	1	0	0	0	0
ENSMUSG00000094866	0	0	0	0	0	0
ENSMUSG00000094865	0	0	0	0	0	0
ENSMUSG00000094864	0	0	0	0	0	0
ENSMUSG00000094861	0	0	0	0	0	0
ENSMUSG00000094860	0	0	0	0	0	0
ENSMUSG00000105527	0	0	0	0	0	0
ENSMUSG00000105525	0	0	3	0	3	1
ENSMUSG00000105524	0	0	0	1	0	0
ENSMUSG00000105523	0	3	0	2	0	3
ENSMUSG00000105522	0	1	1	1	1	0
ENSMUSG00000094869	0	0	0	0	0	0

ENSMUSG00000105520	0	2	0	0	2	0
ENSMUSG00000110309	0	0	0	0	0	0
ENSMUSG00000029219	0	0	0	0	4	6
ENSMUSG00000082833	0	0	0	1	0	2
ENSMUSG00000082832	0	0	0	0	0	0
ENSMUSG00000082831	0	0	0	0	0	0
ENSMUSG00000066364	0	0	0	0	0	0
ENSMUSG00000082835	0	0	0	0	0	0
ENSMUSG00000066366	0	0	0	0	0	0
ENSMUSG00000066368	1	3	0	0	0	3
ENSMUSG00000082839	0	0	0	0	0	0
ENSMUSG00000102403	0	0	0	0	0	0
ENSMUSG00000084646	0	0	0	0	0	0
ENSMUSG00000111598	0	0	0	0	0	0
ENSMUSG00000010803	0	6	0	0	11	0
ENSMUSG00000111590	0	0	0	2	0	0
ENSMUSG00000111597	0	0	0	0	0	0
ENSMUSG00000111596	0	0	0	0	0	0
ENSMUSG00000111594	51	51	65	79	71	100
ENSMUSG00000109844	0	0	0	0	0	0
ENSMUSG00000109845	0	0	0	0	0	0
ENSMUSG00000111627	0	5	5	3	4	3
ENSMUSG00000111621	0	5	0	2	0	0
ENSMUSG00000111620	0	9	0	3	0	4
ENSMUSG00000111622	0	0	0	0	0	0
ENSMUSG00000111629	0	0	0	0	0	5
ENSMUSG00000109849	0	0	6	0	0	0
ENSMUSG00000026468	8	0	5	0	0	0
ENSMUSG00000084643	0	0	0	0	0	0
ENSMUSG00000047938	0	0	0	0	0	0
ENSMUSG00000104698	2	0	2	0	2	0
ENSMUSG00000104695	1	3	7	3	5	0
ENSMUSG00000047935	0	0	0	2	4	2
ENSMUSG00000050538	30	25	16	14	19	16
ENSMUSG00000104691	0	0	0	0	0	0
ENSMUSG00000055942	0	0	0	0	0	0
ENSMUSG00000104473	0	0	0	0	0	0
ENSMUSG00000055944	0	0	0	0	0	0
ENSMUSG00000057540	0	0	0	0	0	0
ENSMUSG00000075188	0	0	0	0	2	0
ENSMUSG00000086975	0	0	0	0	0	0
ENSMUSG00000104340	0	0	0	0	0	0
ENSMUSG00000104341	0	0	0	0	0	0
ENSMUSG00000104342	32	25	31	10	17	34
ENSMUSG00000104343	0	3	0	5	3	10
ENSMUSG00000104345	0	0	0	0	0	0
ENSMUSG00000030851	0	0	0	0	0	0
ENSMUSG00000110264	22	18	11	6	12	0
ENSMUSG00000056632	0	0	0	0	0	0
ENSMUSG00000091504	0	0	0	0	0	0
ENSMUSG00000020226	4	7	0	0	0	0
ENSMUSG00000045797	0	0	0	0	0	0
ENSMUSG00000095380	0	0	0	0	0	0
ENSMUSG00000095383	0	0	0	0	0	0
ENSMUSG00000045794	2	0	0	0	0	0
ENSMUSG00000045792	0	0	0	0	0	0
ENSMUSG00000095386	0	0	0	0	0	0
ENSMUSG00000095389	0	0	0	1	0	0

ENSMUSG00000044772	0	0	0	0	0	0
ENSMUSG00000053044	0	4	9	0	0	5
ENSMUSG00000053046	14	60	39	19	57	60
ENSMUSG00000053040	125	119	140	117	135	136
ENSMUSG00000045421	0	2	0	0	0	3
ENSMUSG00000095659	0	0	0	0	0	0
ENSMUSG00000095658	0	0	0	0	0	0
ENSMUSG00000095653	0	0	0	0	0	0
ENSMUSG00000095650	0	0	0	0	0	0
ENSMUSG00000095657	0	0	0	0	0	0
ENSMUSG00000095655	4	4	2	2	1	2
ENSMUSG00000098382	0	0	0	0	0	0
ENSMUSG00000098383	0	0	0	0	0	0
ENSMUSG00000098386	0	0	0	0	0	0
ENSMUSG00000098388	0	0	0	0	0	0
ENSMUSG00000006143	0	0	0	0	0	0
ENSMUSG00000080529	0	0	0	0	0	0
ENSMUSG00000080522	0	0	0	0	0	0
ENSMUSG00000080526	2	0	0	0	0	0
ENSMUSG00000097315	0	0	0	0	0	0
ENSMUSG00000030361	0	3	4	0	0	0
ENSMUSG00000040489	12	17	10	17	27	17
ENSMUSG00000028976	0	0	0	0	0	4
ENSMUSG00000093719	0	0	0	0	0	0
ENSMUSG00000020916	0	0	0	0	0	0
ENSMUSG00000093711	0	0	0	0	0	0
ENSMUSG00000093714	0	0	0	3	0	0
ENSMUSG00000110169	0	0	0	0	0	0
ENSMUSG00000110168	0	0	0	0	0	0
ENSMUSG00000052025	0	0	0	0	0	0
ENSMUSG00000052026	107	98	139	0	5	0
ENSMUSG00000002240	0	0	0	0	0	0
ENSMUSG00000047150	0	0	6	0	0	0
ENSMUSG00000047155	0	0	0	0	0	0
ENSMUSG00000095928	7	2	0	0	0	0
ENSMUSG00000077776	0	0	0	0	0	0
ENSMUSG00000077771	2	0	0	0	0	0
ENSMUSG00000077772	0	0	0	2	0	2
ENSMUSG00000109383	6	3	6	0	8	4
ENSMUSG00000112063	1	3	0	0	0	1
ENSMUSG00000055555	3	0	0	0	0	0
ENSMUSG00000049685	0	0	0	0	0	0
ENSMUSG00000095927	0	0	0	1	0	0
ENSMUSG00000048080	0	0	0	2	0	0
ENSMUSG00000092447	0	0	0	0	0	0
ENSMUSG00000099416	0	0	0	0	0	0
ENSMUSG00000099411	40	45	44	72	90	84
ENSMUSG00000099410	0	0	0	0	0	0
ENSMUSG00000099413	8	3	4	8	6	10
ENSMUSG00000100464	0	0	0	0	0	0
ENSMUSG00000113033	0	0	0	0	0	0
ENSMUSG00000100467	0	0	0	0	0	0
ENSMUSG00000092448	0	0	0	0	0	0
ENSMUSG00000100466	0	0	0	0	0	0
ENSMUSG00000100461	0	0	0	0	0	0
ENSMUSG00000041301	0	0	0	0	0	0
ENSMUSG00000101497	0	0	0	0	0	0
ENSMUSG00000062152	5	1	2	2	3	5

ENSMUSG00000030701	87	89	82	84	87	98
ENSMUSG00000082316	0	0	0	0	0	0
ENSMUSG00000082312	0	0	0	0	0	2
ENSMUSG00000082310	0	0	0	0	0	0
ENSMUSG00000082311	0	0	0	0	0	0
ENSMUSG00000048337	24	38	34	20	36	27
ENSMUSG00000048334	10	14	9	9	10	13
ENSMUSG00000062151	14	6	20	3	18	11
ENSMUSG00000100469	0	0	0	0	0	0
ENSMUSG00000101490	0	0	0	0	0	0
ENSMUSG00000102162	0	0	0	1	2	2
ENSMUSG00000100120	6	0	0	0	4	0
ENSMUSG00000072726	0	0	0	2	3	0
ENSMUSG00000100123	0	0	0	0	0	0
ENSMUSG00000100124	0	0	0	0	0	0
ENSMUSG00000100125	0	0	0	0	0	0
ENSMUSG00000072722	0	0	0	0	2	0
ENSMUSG00000072723	8	8	9	2	7	3
ENSMUSG00000100129	0	0	0	0	0	0
ENSMUSG00000101498	0	0	0	0	0	0
ENSMUSG00000043531	21	21	27	14	15	33
ENSMUSG00000084413	3	1	5	4	6	2
ENSMUSG00000084412	1	0	1	0	0	3
ENSMUSG00000084417	0	0	0	0	0	0
ENSMUSG00000043537	0	0	0	0	0	0
ENSMUSG00000084415	0	0	0	0	0	0
ENSMUSG00000084414	0	0	0	0	0	0
ENSMUSG00000113779	0	0	0	0	0	0
ENSMUSG00000100698	0	0	0	0	0	0
ENSMUSG00000100699	0	0	0	0	0	1
ENSMUSG00000061633	0	0	0	0	0	0
ENSMUSG00000100692	0	0	0	0	0	0
ENSMUSG00000100693	0	0	0	0	0	0
ENSMUSG00000100690	1	0	0	0	0	0
ENSMUSG00000100696	0	0	0	0	0	2
ENSMUSG00000100694	0	0	0	0	0	0
ENSMUSG00000100695	0	0	0	0	0	0
ENSMUSG00000087852	0	0	0	0	0	0
ENSMUSG00000087853	0	0	0	0	0	0
ENSMUSG00000087850	0	0	0	0	0	0
ENSMUSG00000087855	0	0	0	0	0	0
ENSMUSG00000087858	0	0	0	0	0	0
ENSMUSG00000037167	0	0	0	0	0	0
ENSMUSG00000037161	11	11	10	0	4	9
ENSMUSG00000107479	0	0	0	0	0	0
ENSMUSG00000107477	11	17	9	6	7	6
ENSMUSG00000107475	0	0	0	0	0	0
ENSMUSG00000083191	0	0	0	0	0	2
ENSMUSG00000083190	0	0	0	0	0	0
ENSMUSG00000102197	2	4	0	0	0	0
ENSMUSG00000079528	0	0	0	0	0	0
ENSMUSG00000079525	0	0	0	0	0	0
ENSMUSG00000102195	0	0	0	0	0	0
ENSMUSG00000079521	0	0	0	0	0	0
ENSMUSG00000046687	5	6	7	2	1	5
ENSMUSG00000061246	0	0	0	0	0	0
ENSMUSG00000099970	0	0	0	0	0	0
ENSMUSG00000046683	23	5	7	12	14	29

ENSMUSG00000107078	0	0	0	0	0	0
ENSMUSG00000062914	0	0	0	0	0	3
ENSMUSG00000036412	28	17	87	16	30	18
ENSMUSG00000107110	0	0	0	0	0	0
ENSMUSG00000101439	58	60	52	30	47	45
ENSMUSG00000065031	0	0	0	0	0	0
ENSMUSG00000078698	0	0	0	0	0	0
ENSMUSG00000078691	0	0	0	0	0	0
ENSMUSG00000103579	0	0	0	0	0	0
ENSMUSG00000101234	2	31	19	21	1	20
ENSMUSG00000103571	0	0	2	0	0	0
ENSMUSG00000103575	0	0	0	0	0	0
ENSMUSG00000105105	0	0	0	0	0	0
ENSMUSG00000035148	0	0	0	0	0	0
ENSMUSG00000105106	2	0	0	0	0	0
ENSMUSG00000078346	0	0	0	0	0	0
ENSMUSG00000105102	0	0	0	0	0	0
ENSMUSG00000100900	0	0	0	0	0	0
ENSMUSG00000100904	0	0	0	0	0	0
ENSMUSG00000089458	5	0	0	5	0	3
ENSMUSG00000089459	0	0	0	0	0	0
ENSMUSG00000100908	0	0	0	0	0	0
ENSMUSG00000097468	0	0	0	0	0	9
ENSMUSG00000097463	3	17	19	5	14	6
ENSMUSG00000097466	0	0	0	0	0	0
ENSMUSG00000097465	0	0	0	0	0	0
ENSMUSG00000063796	0	0	0	0	0	0
ENSMUSG00000076030	0	0	0	0	0	0
ENSMUSG00000083243	0	0	0	0	0	0
ENSMUSG00000096042	0	0	0	0	0	0
ENSMUSG00000086452	0	0	0	0	0	0
ENSMUSG00000058508	0	0	0	0	0	1
ENSMUSG00000069620	0	0	0	0	0	0
ENSMUSG00000102229	0	0	0	0	0	0
ENSMUSG00000102221	0	0	0	0	0	0
ENSMUSG00000102223	0	0	0	0	0	0
ENSMUSG00000102224	0	0	0	0	0	0
ENSMUSG00000102227	0	0	0	0	0	0
ENSMUSG00000096738	0	0	0	0	0	0
ENSMUSG00000096731	0	0	0	0	0	0
ENSMUSG00000096730	0	0	0	0	0	0
ENSMUSG00000096733	0	0	0	0	0	0
ENSMUSG00000096732	0	0	0	0	0	0
ENSMUSG00000096735	0	0	0	1	0	1
ENSMUSG00000096734	0	0	0	0	0	0
ENSMUSG00000096737	0	0	0	0	1	0
ENSMUSG00000031130	0	0	0	0	0	0
ENSMUSG00000031131	0	0	0	0	0	0
ENSMUSG00000031132	4	0	0	0	0	0
ENSMUSG00000031138	4	2	0	0	0	0
ENSMUSG00000031139	0	0	0	0	2	0
ENSMUSG00000063137	4	10	4	0	2	3
ENSMUSG00000063133	0	0	0	0	0	0
ENSMUSG00000086558	0	0	0	0	0	0
ENSMUSG00000063130	2	0	2	0	2	10
ENSMUSG00000021362	0	0	0	0	0	0
ENSMUSG00000021363	16	15	10	10	13	10
ENSMUSG00000021364	0	0	0	0	2	4

ENSMUSG00000057706	156	130	155	57	71	94
ENSMUSG00000050089	0	0	0	0	0	0
ENSMUSG00000050085	0	0	0	0	0	0
ENSMUSG00000104105	0	0	0	0	0	0
ENSMUSG00000104102	0	0	0	0	0	0
ENSMUSG00000104103	32	32	20	23	30	27
ENSMUSG00000104100	0	0	0	0	1	0
ENSMUSG00000104101	2	2	0	2	3	0
ENSMUSG00000044186	0	0	0	0	0	0
ENSMUSG00000104109	0	4	0	6	6	0
ENSMUSG00000099762	0	0	0	0	0	0
ENSMUSG00000088465	0	0	0	0	0	0
ENSMUSG00000088468	0	0	0	0	0	0
ENSMUSG00000091174	0	0	0	0	0	0
ENSMUSG00000091175	0	0	0	0	0	0
ENSMUSG00000021986	4	4	0	0	2	2
ENSMUSG00000110858	0	0	0	0	0	0
ENSMUSG00000110851	0	0	0	0	0	0
ENSMUSG00000110853	0	0	0	0	0	0
ENSMUSG00000110855	0	0	0	0	0	0
ENSMUSG00000110854	0	0	1	1	1	0
ENSMUSG00000110857	0	0	0	0	0	0
ENSMUSG00000110856	0	0	0	0	0	0
ENSMUSG00000027202	0	0	0	0	0	0
ENSMUSG00000104817	0	0	0	0	0	0
ENSMUSG00000104811	0	0	0	0	0	0
ENSMUSG00000028972	0	0	3	0	0	0
ENSMUSG00000104819	2	0	0	0	0	0
ENSMUSG00000095412	0	0	0	0	0	0
ENSMUSG00000058084	0	0	0	0	0	0
ENSMUSG00000022180	58	71	114	49	83	70
ENSMUSG00000096593	0	0	0	0	0	0
ENSMUSG00000022187	0	0	0	0	0	0
ENSMUSG00000096595	0	0	0	0	0	0
ENSMUSG00000107895	0	0	0	0	0	0
ENSMUSG00000087758	0	0	0	0	0	0
ENSMUSG00000027973	0	0	0	0	0	0
ENSMUSG00000027971	0	0	0	0	0	3
ENSMUSG00000027977	5	11	9	6	3	23
ENSMUSG00000086278	0	0	0	0	0	0
ENSMUSG00000086275	0	0	0	0	0	0
ENSMUSG00000086273	0	0	0	0	0	0
ENSMUSG00000086272	0	0	0	0	0	0
ENSMUSG00000111810	0	0	0	0	0	0
ENSMUSG00000111811	0	0	0	0	0	0
ENSMUSG00000111816	0	0	0	0	0	0
ENSMUSG00000052625	3	0	0	0	0	0
ENSMUSG00000111818	0	0	0	0	0	0
ENSMUSG00000111819	0	0	0	0	0	3
ENSMUSG00000023274	44	44	43	23	22	24
ENSMUSG00000097814	4	0	0	0	0	0
ENSMUSG00000112541	0	0	0	0	0	0
ENSMUSG00000103389	0	0	0	0	0	0
ENSMUSG00000112543	3	8	8	9	6	3
ENSMUSG00000112542	0	0	0	0	0	0
ENSMUSG00000096196	1	0	0	0	0	1
ENSMUSG00000039364	0	0	0	2	0	0
ENSMUSG00000112549	0	0	2	0	0	0

ENSMUSG00000108559	0	0	0	0	0	0
ENSMUSG00000104279	18	24	19	25	15	17
ENSMUSG00000104275	0	0	0	0	0	0
ENSMUSG00000092285	0	0	0	0	0	0
ENSMUSG00000092287	0	0	0	0	0	0
ENSMUSG00000092286	11	15	20	6	32	19
ENSMUSG00000092281	30	31	19	25	29	24
ENSMUSG00000047013	7	12	5	4	9	0
ENSMUSG00000047014	0	3	0	0	0	0
ENSMUSG00000004031	20	6	15	11	10	2
ENSMUSG00000071613	0	0	0	7	0	0
ENSMUSG00000024727	86	57	114	14	21	17
ENSMUSG00000049420	46	22	18	0	5	0
ENSMUSG00000024728	0	0	0	0	0	0
ENSMUSG00000020617	0	0	0	0	0	0
ENSMUSG00000097103	4	8	8	4	9	15
ENSMUSG00000097107	0	0	0	0	0	0
ENSMUSG00000106448	0	3	0	0	3	0
ENSMUSG00000046942	0	0	0	0	3	0
ENSMUSG00000110031	0	0	0	0	0	2
ENSMUSG00000110035	0	0	0	0	0	5
ENSMUSG00000070568	0	0	0	0	0	0
ENSMUSG00000070564	0	0	5	0	0	0
ENSMUSG00000106445	0	0	0	0	0	0
ENSMUSG00000054117	0	0	0	0	0	0
ENSMUSG00000077049	0	1	0	0	0	0
ENSMUSG00000105778	0	0	0	0	0	0
ENSMUSG00000109497	0	0	0	0	0	0
ENSMUSG00000109496	3	0	0	0	0	0
ENSMUSG00000109495	0	2	2	2	5	1
ENSMUSG00000109494	0	0	1	0	0	0
ENSMUSG00000105773	5	2	1	2	2	2
ENSMUSG00000101772	0	0	0	0	0	0
ENSMUSG00000031478	142	130	110	104	72	141
ENSMUSG00000037106	27	37	35	46	61	41
ENSMUSG00000107768	22	8	33	0	0	2
ENSMUSG00000108728	0	0	0	0	0	0
ENSMUSG00000108726	0	0	0	0	0	0
ENSMUSG00000108727	0	0	0	0	0	0
ENSMUSG00000108724	0	0	0	0	0	0
ENSMUSG00000108725	0	0	0	0	0	0
ENSMUSG00000108722	0	0	2	0	0	0
ENSMUSG00000108723	3	2	5	0	0	2
ENSMUSG00000108721	1	2	0	0	1	0
ENSMUSG00000083359	10	7	8	9	4	12
ENSMUSG00000083358	0	0	0	0	0	0
ENSMUSG00000083355	8	0	10	0	0	5
ENSMUSG00000031471	6	0	0	0	0	6
ENSMUSG00000083356	0	0	0	4	0	0
ENSMUSG00000083351	0	0	0	0	0	0
ENSMUSG00000083350	0	0	0	0	0	0
ENSMUSG00000083352	0	0	0	0	0	0
ENSMUSG00000107761	0	0	0	0	0	0
ENSMUSG00000089029	0	0	0	0	0	0
ENSMUSG00000107767	0	0	0	0	0	0
ENSMUSG00000104919	4	0	2	5	2	7
ENSMUSG00000107765	0	0	0	0	0	0
ENSMUSG00000101776	0	0	4	0	5	3

ENSMUSG00000092743	0	0	0	0	0	0
ENSMUSG00000093905	0	0	0	0	0	0
ENSMUSG00000026614	3	0	0	0	2	4
ENSMUSG00000093907	0	0	1	0	0	1
ENSMUSG00000026616	72	81	102	52	39	47
ENSMUSG00000093901	0	0	0	0	0	0
ENSMUSG00000093900	1	0	0	0	1	0
ENSMUSG00000093903	0	0	0	0	0	0
ENSMUSG00000093902	0	0	0	0	0	0
ENSMUSG00000064468	0	0	0	0	0	0
ENSMUSG00000093909	0	0	0	0	0	0
ENSMUSG00000044633	0	0	0	0	0	0
ENSMUSG00000044639	0	0	0	0	2	0
ENSMUSG00000111976	0	0	0	0	0	0
ENSMUSG00000108266	0	0	0	0	0	0
ENSMUSG00000045322	80	50	85	12	34	32
ENSMUSG00000101170	0	0	0	0	0	0
ENSMUSG00000103655	0	0	0	0	0	0
ENSMUSG00000045328	156	153	170	23	47	106
ENSMUSG00000103654	0	0	0	0	0	0
ENSMUSG00000051618	0	0	0	0	0	0
ENSMUSG00000079773	0	3	0	0	0	0
ENSMUSG00000111453	0	0	0	0	0	0
ENSMUSG00000065381	0	0	0	0	0	0
ENSMUSG00000111450	0	0	0	0	0	0
ENSMUSG00000027564	0	0	0	0	0	0
ENSMUSG00000109612	0	0	0	0	0	0
ENSMUSG00000027560	13	12	12	0	3	6
ENSMUSG00000109615	0	0	0	0	0	0
ENSMUSG00000027568	0	0	0	0	0	0
ENSMUSG00000109614	0	0	0	0	0	0
ENSMUSG00000051611	0	0	0	0	0	0
ENSMUSG00000109616	0	3	0	5	0	5
ENSMUSG00000090346	0	0	0	0	0	3
ENSMUSG00000090342	2	4	6	0	0	0
ENSMUSG00000090341	0	0	0	0	0	0
ENSMUSG00000048981	0	0	0	0	0	0
ENSMUSG00000081240	0	0	0	0	0	0
ENSMUSG00000081243	0	0	0	0	0	0
ENSMUSG00000048982	0	3	2	0	0	0
ENSMUSG00000048988	0	14	8	0	7	6
ENSMUSG00000042671	0	0	0	0	0	0
ENSMUSG00000099999	0	0	0	0	0	0
ENSMUSG00000042677	171	185	157	114	146	192
ENSMUSG00000042678	0	0	0	0	0	2
ENSMUSG00000099996	0	0	0	0	0	7
ENSMUSG00000099990	0	0	0	0	0	0
ENSMUSG00000099991	0	0	0	0	0	0
ENSMUSG00000099992	0	0	0	0	0	0
ENSMUSG00000099993	0	0	0	0	0	0
ENSMUSG00000072188	0	0	0	0	0	0
ENSMUSG00000046167	0	0	0	0	0	2
ENSMUSG00000084999	0	0	0	0	0	0
ENSMUSG00000084992	0	4	0	0	0	0
ENSMUSG00000084990	0	0	0	0	0	0
ENSMUSG00000084996	0	0	0	0	2	3
ENSMUSG00000084995	0	0	0	0	0	0
ENSMUSG00000098649	0	0	0	0	0	0

ENSMUSG00000098642	0	0	2	0	0	0
ENSMUSG00000098640	0	0	0	0	0	0
ENSMUSG00000098647	0	0	0	0	0	0
ENSMUSG00000098644	0	0	0	0	0	0
ENSMUSG00000073130	5	0	6	0	0	0
ENSMUSG00000114415	3	6	0	0	0	0
ENSMUSG00000111427	0	0	0	0	0	0
ENSMUSG00000106996	0	0	0	0	0	0
ENSMUSG00000078815	0	0	13	56	27	16
ENSMUSG00000086736	19	46	28	34	23	44
ENSMUSG00000078817	2	4	12	5	4	6
ENSMUSG00000078810	8	14	8	11	23	5
ENSMUSG00000086732	0	0	0	0	0	0
ENSMUSG00000086733	0	0	0	0	0	0
ENSMUSG00000063354	2	8	5	0	0	2
ENSMUSG00000114414	0	0	0	0	0	0
ENSMUSG00000086738	0	0	0	0	0	0
ENSMUSG00000086739	0	0	0	0	0	0
ENSMUSG00000092506	0	0	0	0	0	0
ENSMUSG00000099415	0	0	0	0	0	0
ENSMUSG00000099414	0	0	0	0	0	0
ENSMUSG00000049761	0	0	3	0	0	0
ENSMUSG00000082291	0	0	0	0	0	0
ENSMUSG00000082297	0	0	0	0	0	0
ENSMUSG00000082295	0	0	0	0	0	0
ENSMUSG00000082294	0	0	0	0	0	0
ENSMUSG00000089731	0	0	0	0	0	0
ENSMUSG00000092441	0	0	0	0	0	0
ENSMUSG00000092440	0	0	0	0	0	0
ENSMUSG00000099418	0	0	0	0	0	0
ENSMUSG00000043692	0	0	0	0	0	0
ENSMUSG00000067941	0	0	0	6	0	5
ENSMUSG00000030577	37	38	59	5	6	16
ENSMUSG00000114413	1	0	0	0	1	2
ENSMUSG00000101440	0	0	0	0	0	0
ENSMUSG00000101441	0	0	0	0	0	0
ENSMUSG00000101442	0	0	0	0	0	0
ENSMUSG00000101443	0	0	0	0	0	0
ENSMUSG00000101444	0	0	0	0	0	0
ENSMUSG00000020286	3	0	0	2	0	0
ENSMUSG00000101446	0	0	1	0	0	0
ENSMUSG00000101447	0	0	0	0	0	0
ENSMUSG00000101448	0	0	0	0	0	0
ENSMUSG00000080829	0	0	0	2	3	1
ENSMUSG00000040525	0	0	4	0	0	4
ENSMUSG00000019489	0	0	0	0	0	0
ENSMUSG00000083830	0	0	0	0	0	0
ENSMUSG00000107848	0	0	0	0	0	0
ENSMUSG00000107849	0	0	0	0	0	0
ENSMUSG00000107842	0	0	0	0	0	0
ENSMUSG00000107840	0	0	0	0	0	0
ENSMUSG00000107841	11	14	15	3	8	20
ENSMUSG00000107847	0	0	0	0	0	0
ENSMUSG00000107844	0	0	0	0	0	0
ENSMUSG00000107845	0	0	0	0	0	0
ENSMUSG00000103358	2	10	6	0	4	4
ENSMUSG00000103352	0	0	0	0	0	0
ENSMUSG00000103353	0	0	0	0	0	0

ENSMUSG00000103350	0	0	0	0	0	0
ENSMUSG00000103351	0	0	0	0	0	0
ENSMUSG00000103357	3	0	3	0	0	0
ENSMUSG00000103354	2	2	0	0	4	2
ENSMUSG00000082125	0	0	0	0	0	0
ENSMUSG00000082126	0	0	0	0	0	0
ENSMUSG00000082122	0	0	0	0	0	0
ENSMUSG00000082129	1	1	2	0	0	1
ENSMUSG00000099205	0	0	0	0	0	0
ENSMUSG00000099201	0	0	0	0	0	0
ENSMUSG00000099203	0	0	0	0	0	0
ENSMUSG00000099208	0	0	0	0	0	0
ENSMUSG00000014164	191	192	225	326	283	409
ENSMUSG00000043485	0	0	0	0	0	0
ENSMUSG00000032623	0	0	0	0	0	0
ENSMUSG00000089270	0	0	1	2	1	1
ENSMUSG00000083655	9	37	15	30	30	27
ENSMUSG00000089272	0	0	0	0	0	0
ENSMUSG00000089273	6	4	10	14	6	10
ENSMUSG00000089274	0	0	0	0	0	0
ENSMUSG00000084607	0	2	0	2	2	1
ENSMUSG00000084604	1	2	2	0	0	0
ENSMUSG00000089277	0	0	0	0	0	0
ENSMUSG00000089278	0	0	0	0	0	0
ENSMUSG00000089279	0	0	0	0	0	0
ENSMUSG00000084608	0	0	0	0	0	0
ENSMUSG00000084609	0	0	0	0	0	0
ENSMUSG00000083659	0	0	0	0	0	0
ENSMUSG00000100150	0	0	0	0	0	0
ENSMUSG00000064939	0	0	0	0	0	0
ENSMUSG00000096386	0	0	0	1	0	0
ENSMUSG00000100718	0	0	0	0	0	0
ENSMUSG00000100717	0	0	0	0	2	0
ENSMUSG00000100716	0	0	0	0	0	0
ENSMUSG00000100715	0	0	0	0	0	0
ENSMUSG00000114218	0	0	3	0	0	0
ENSMUSG00000100713	0	3	0	0	0	0
ENSMUSG00000100712	0	0	0	0	0	0
ENSMUSG00000100711	0	0	0	0	3	0
ENSMUSG00000100710	0	0	0	0	0	0
ENSMUSG00000102408	0	0	0	0	0	0
ENSMUSG00000102405	0	0	0	0	0	0
ENSMUSG00000102407	0	0	0	0	0	0
ENSMUSG00000102406	0	0	0	0	0	0
ENSMUSG00000087946	0	0	0	0	0	0
ENSMUSG00000000365	57	40	38	31	35	35
ENSMUSG00000097677	0	0	0	0	0	0
ENSMUSG00000082314	0	0	0	0	2	0
ENSMUSG00000041347	3	4	7	4	3	3
ENSMUSG00000087957	0	0	0	0	0	0
ENSMUSG00000087954	0	0	0	0	0	0
ENSMUSG00000087958	0	0	0	0	0	0
ENSMUSG00000085551	0	0	0	0	0	0
ENSMUSG00000085553	0	0	0	0	0	0
ENSMUSG00000085552	0	1	0	0	0	0
ENSMUSG00000085555	0	0	0	2	0	0
ENSMUSG00000085556	0	0	0	0	0	0
ENSMUSG00000064550	0	0	0	0	0	0

ENSMUSG00000085558	81	54	47	67	68	74
ENSMUSG00000064554	0	0	0	0	0	0
ENSMUSG00000064555	0	0	0	0	0	0
ENSMUSG00000107159	0	0	0	0	0	0
ENSMUSG00000112408	0	0	0	0	0	0
ENSMUSG00000107153	0	0	0	0	0	0
ENSMUSG00000020798	7	10	7	9	7	10
ENSMUSG00000107157	4	2	2	5	3	5
ENSMUSG00000020799	0	0	4	0	3	2
ENSMUSG00000023073	0	0	0	0	0	0
ENSMUSG00000023070	16	0	10	18	9	14
ENSMUSG00000088603	0	0	0	0	0	0
ENSMUSG00000088601	0	0	0	0	0	0
ENSMUSG00000095768	0	0	0	0	0	0
ENSMUSG00000023083	2	3	6	0	0	0
ENSMUSG00000021506	8	0	0	3	0	0
ENSMUSG00000084725	0	0	0	0	0	0
ENSMUSG00000021509	0	0	0	0	0	0
ENSMUSG00000074720	0	0	0	0	0	0
ENSMUSG00000095764	0	0	0	0	0	0
ENSMUSG00000061457	0	0	0	0	0	0
ENSMUSG00000029736	0	0	0	0	0	5
ENSMUSG00000029735	133	143	162	121	127	163
ENSMUSG00000112250	0	0	0	0	0	0
ENSMUSG00000032415	60	58	34	52	50	69
ENSMUSG00000103438	0	0	0	0	0	0
ENSMUSG00000103437	0	0	0	0	0	0
ENSMUSG00000103434	0	0	0	0	0	0
ENSMUSG00000103433	0	1	0	0	0	0
ENSMUSG00000106400	0	0	0	0	0	0
ENSMUSG00000103431	0	0	0	0	0	0
ENSMUSG00000106402	0	0	0	0	0	0
ENSMUSG00000097349	0	0	0	0	0	0
ENSMUSG00000022857	0	0	0	0	0	0
ENSMUSG00000097346	0	12	0	0	0	0
ENSMUSG00000097344	0	0	5	0	4	0
ENSMUSG00000069785	0	0	0	0	0	0
ENSMUSG00000070136	0	0	1	0	0	0
ENSMUSG00000029086	45	25	20	54	31	44
ENSMUSG00000066158	0	0	4	5	0	0
ENSMUSG00000066153	0	0	0	0	0	0
ENSMUSG00000066154	0	0	0	0	0	0
ENSMUSG00000097691	0	0	0	0	0	0
ENSMUSG00000097695	3	1	2	3	1	4
ENSMUSG00000097694	0	0	0	0	0	0
ENSMUSG00000097697	13	15	12	5	0	0
ENSMUSG00000034087	0	0	0	0	0	0
ENSMUSG00000069873	0	0	0	0	0	0
ENSMUSG00000051652	11	8	32	17	26	22
ENSMUSG00000099104	2	3	4	0	1	5
ENSMUSG00000109659	0	0	0	0	0	0
ENSMUSG00000102389	0	2	6	0	0	2
ENSMUSG00000102386	0	0	0	0	0	0
ENSMUSG00000109654	6	20	8	9	6	8
ENSMUSG00000102385	0	0	0	0	0	3
ENSMUSG00000102382	0	0	0	0	0	0
ENSMUSG00000105171	0	0	0	1	1	0
ENSMUSG00000114250	0	1	0	1	0	0

ENSMUSG00000114257	1	1	2	1	0	2	
ENSMUSG00000114259	0	0	0	0	0	0	
ENSMUSG00000114258	0	0	0	0	0	0	
ENSMUSG00000112949	0	0	0	0	0	0	
ENSMUSG00000108529	0	0	0	0	0	0	
ENSMUSG00000108525	0	1	1	1	0	4	
ENSMUSG00000108520	12	9	14	10	13	24	
ENSMUSG00000112940	0	0	0	0	0	0	
ENSMUSG00000112943	0	0	0	0	0	0	
ENSMUSG00000067201	0	0	0	0	0	0	
ENSMUSG00000110319	0	0	0	0	0	0	
ENSMUSG00000072721	0	0	0	0	0	0	
ENSMUSG00000100126	0	0	0	0	0	0	
ENSMUSG00000100128	0	2	0	0	0	0	
ENSMUSG00000050296	275	311	200	693	550	677	451
ENSMUSG00000044217	0	7	4	0	0	0	
ENSMUSG00000091692	0	0	0	0	0	0	
ENSMUSG00000091698	0	0	0	0	0	0	
ENSMUSG00000096569	0	0	0	0	0	0	
ENSMUSG00000021087	53	57	82	34	74	69	
ENSMUSG00000100719	0	0	0	0	0	0	
ENSMUSG00000057116	2	0	0	0	0	0	
ENSMUSG00000110462	0	0	0	0	3	0	
ENSMUSG00000110463	0	0	0	0	0	0	
ENSMUSG00000110464	0	0	0	0	0	2	
ENSMUSG00000101127	0	0	0	0	0	0	
ENSMUSG00000101126	0	0	0	0	0	0	
ENSMUSG00000101125	0	0	0	0	0	0	
ENSMUSG00000101124	0	0	0	0	0	0	
ENSMUSG00000101123	0	0	0	0	0	0	
ENSMUSG00000101120	0	0	0	0	0	0	
ENSMUSG00000056863	0	0	0	0	0	0	
ENSMUSG00000101128	0	0	0	0	0	0	
ENSMUSG00000026368	0	2	0	0	0	0	
ENSMUSG00000045126	0	0	0	0	2	0	
ENSMUSG00000111373	0	0	0	0	0	0	
ENSMUSG00000064961	0	0	0	0	0	0	
ENSMUSG00000060260	178	191	204	160	176	220	
ENSMUSG00000091034	0	0	0	0	0	0	
ENSMUSG00000091031	0	0	0	0	0	0	
ENSMUSG00000045693	0	0	0	0	0	0	
ENSMUSG00000091039	0	0	0	0	0	0	
ENSMUSG00000026943	0	0	0	0	0	0	
ENSMUSG00000100714	0	0	0	0	0	0	
ENSMUSG00000056821	0	0	0	0	0	0	
ENSMUSG00000081001	0	0	0	0	0	0	
ENSMUSG00000081000	0	0	0	0	0	0	
ENSMUSG00000081007	0	0	0	0	0	0	
ENSMUSG00000081006	0	0	0	0	0	0	
ENSMUSG00000081004	7	3	3	2	5	1	
ENSMUSG00000081009	0	0	0	0	0	0	
ENSMUSG00000042477	5	4	2	0	5	14	
ENSMUSG00000027015	34	18	6	6	5	3	
ENSMUSG00000042474	2	18	10	10	6	12	
ENSMUSG00000022061	0	0	0	0	0	0	
ENSMUSG00000113630	9	19	8	0	7	7	
ENSMUSG00000111993	0	0	0	0	0	0	
ENSMUSG00000111990	0	0	0	0	0	0	

ENSMUSG00000111999	0	0	0	0	0	0
ENSMUSG00000111998	0	0	0	0	0	0
ENSMUSG00000053742	0	0	0	0	0	0
ENSMUSG00000053747	0	0	0	0	0	0
ENSMUSG00000096889	0	0	0	0	0	0
ENSMUSG00000094404	1	0	0	0	0	0
ENSMUSG00000015314	19	9	29	9	4	12
ENSMUSG00000084418	0	0	0	0	0	0
ENSMUSG00000094407	0	0	0	0	0	0
ENSMUSG00000096524	1	1	0	0	0	0
ENSMUSG00000104939	0	0	0	0	0	0
ENSMUSG00000077125	0	0	1	0	0	0
ENSMUSG00000055748	0	0	5	0	0	0
ENSMUSG00000055746	0	0	0	0	0	0
ENSMUSG00000049565	12	10	8	0	0	0
ENSMUSG00000049560	0	0	0	0	0	0
ENSMUSG00000049561	3	0	0	0	0	0
ENSMUSG00000049562	226	193	231	123	110	193
ENSMUSG00000072575	0	0	0	0	0	0
ENSMUSG00000072573	13	15	20	7	9	3
ENSMUSG00000082301	0	0	0	0	2	0
ENSMUSG00000092674	0	0	0	0	0	0
ENSMUSG00000086501	3	0	0	0	0	0
ENSMUSG00000100697	0	0	1	2	0	0
ENSMUSG00000045903	8	0	0	2	0	3
ENSMUSG00000104757	0	0	0	0	0	0
ENSMUSG00000096950	4	0	11	0	3	0
ENSMUSG00000096955	0	0	0	0	0	1
ENSMUSG00000098938	0	0	0	0	0	0
ENSMUSG00000098935	0	0	0	0	0	0
ENSMUSG00000098937	0	0	0	0	0	0
ENSMUSG00000112513	0	0	0	0	0	0
ENSMUSG00000106365	0	5	0	0	0	0
ENSMUSG00000113268	0	0	1	0	0	0
ENSMUSG00000106360	0	0	0	0	0	0
ENSMUSG00000106361	0	2	2	0	0	0
ENSMUSG00000106363	0	0	0	0	0	0
ENSMUSG00000054836	48	89	87	69	65	117
ENSMUSG00000113260	0	0	0	0	0	0
ENSMUSG00000113267	0	0	0	0	0	0
ENSMUSG00000113266	0	0	0	0	0	0
ENSMUSG00000113265	0	0	0	0	0	0
ENSMUSG00000071000	0	0	0	0	0	0
ENSMUSG00000100554	0	0	0	0	0	0
ENSMUSG00000109354	0	2	0	0	0	0
ENSMUSG00000109355	0	0	0	0	0	0
ENSMUSG00000109356	0	0	0	0	0	0
ENSMUSG00000109351	0	0	0	0	0	0
ENSMUSG00000109359	0	2	0	0	0	0
ENSMUSG00000082723	0	0	0	0	0	0
ENSMUSG00000076385	0	0	0	0	0	0
ENSMUSG00000082727	0	0	0	0	0	0
ENSMUSG00000082728	0	0	0	0	0	0
ENSMUSG00000076389	0	0	0	0	0	0
ENSMUSG00000047228	0	0	0	0	0	0
ENSMUSG00000110288	0	0	0	0	0	0
ENSMUSG00000092097	21	13	12	10	9	11
ENSMUSG00000092090	0	0	2	0	0	3

ENSMUSG00000092093	4	5	5	6	4	2
ENSMUSG00000047220	2	0	0	0	0	0
ENSMUSG00000047222	0	0	0	0	2	0
ENSMUSG00000047225	0	0	0	0	0	0
ENSMUSG00000083093	0	0	0	0	0	0
ENSMUSG00000083090	0	0	0	0	0	2
ENSMUSG00000083091	0	0	0	0	0	0
ENSMUSG00000083096	0	0	0	0	0	0
ENSMUSG00000083094	0	0	0	0	0	0
ENSMUSG00000083098	0	0	0	0	0	0
ENSMUSG00000083099	0	0	0	0	0	0
ENSMUSG00000098437	0	0	0	0	0	0
ENSMUSG00000093223	0	0	0	0	0	0
ENSMUSG00000099322	0	0	0	0	0	0
ENSMUSG00000099321	0	0	0	0	0	0
ENSMUSG00000099326	0	0	1	0	1	2
ENSMUSG00000005131	27	19	18	7	4	12
ENSMUSG00000099328	0	0	0	0	0	0
ENSMUSG00000084378	4	0	0	0	0	0
ENSMUSG00000084379	3	0	0	0	0	2
ENSMUSG00000084372	0	0	0	0	0	0
ENSMUSG00000084370	0	2	0	0	0	0
ENSMUSG00000084371	0	0	0	0	0	0
ENSMUSG00000084376	0	0	0	0	0	0
ENSMUSG00000084377	0	0	0	0	0	0
ENSMUSG00000084374	0	0	0	0	0	0
ENSMUSG00000084375	0	0	0	0	0	2
ENSMUSG00000000560	0	0	3	0	6	0
ENSMUSG00000099848	0	0	0	0	0	0
ENSMUSG00000083197	0	0	0	0	0	0
ENSMUSG00000025577	154	112	153	122	165	15
ENSMUSG00000025576	0	0	0	0	0	0
ENSMUSG00000025573	2	0	0	0	0	0
ENSMUSG00000082929	0	0	0	0	0	0
ENSMUSG00000025578	141	149	111	121	153	232
ENSMUSG00000100552	0	0	0	0	0	0
ENSMUSG00000107473	0	0	0	0	0	0
ENSMUSG00000107471	0	0	0	0	0	0
ENSMUSG00000065028	0	0	0	0	0	0
ENSMUSG00000079174	0	0	0	0	0	0
ENSMUSG00000079175	0	0	0	0	0	0
ENSMUSG00000079170	0	0	0	0	0	0
ENSMUSG00000079171	0	0	0	0	0	0
ENSMUSG00000085021	6	0	0	0	0	0
ENSMUSG00000085020	0	0	0	0	0	0
ENSMUSG00000085025	6	9	5	4	4	0
ENSMUSG00000085027	0	0	0	8	7	2
ENSMUSG00000085026	0	0	0	0	0	0
ENSMUSG00000065042	0	0	0	0	0	0
ENSMUSG00000107130	0	0	0	0	0	0
ENSMUSG00000033850	0	0	0	3	0	0
ENSMUSG00000113462	0	2	2	0	0	0
ENSMUSG00000033854	4	2	0	3	0	0
ENSMUSG00000033857	221	122	192	175	158	234
ENSMUSG00000079522	0	0	0	0	0	0
ENSMUSG00000093335	0	0	0	0	0	0
ENSMUSG00000108356	0	0	0	0	0	0
ENSMUSG00000084476	0	0	0	0	0	0

ENSMUSG00000101267	0	0	0	0	0	0
ENSMUSG00000113469	0	0	0	2	1	1
ENSMUSG00000112490	0	0	0	0	0	0
ENSMUSG00000099810	0	0	0	0	0	0
ENSMUSG00000099815	0	0	0	0	0	0
ENSMUSG00000099814	0	0	0	0	0	0
ENSMUSG00000099817	0	0	0	0	0	0
ENSMUSG00000099816	0	3	0	0	0	0
ENSMUSG00000099818	0	0	0	0	0	0
ENSMUSG00000059077	0	0	0	0	0	0
ENSMUSG00000100558	0	0	0	0	0	0
ENSMUSG00000106303	0	0	0	0	0	0
ENSMUSG00000019913	0	0	0	0	0	0
ENSMUSG00000104110	0	0	0	0	0	0
ENSMUSG00000072962	11	4	0	2	0	0
ENSMUSG00000072963	4	0	3	0	3	0
ENSMUSG00000020213	0	0	0	0	0	0
ENSMUSG00000072968	1	0	0	0	0	0
ENSMUSG00000094400	0	0	0	0	0	0
ENSMUSG00000077244	0	0	0	0	0	0
ENSMUSG00000077246	0	0	0	0	0	0
ENSMUSG00000077240	0	0	0	0	0	0
ENSMUSG00000077241	0	0	0	0	0	0
ENSMUSG00000082666	0	0	0	0	0	0
ENSMUSG00000077243	0	0	0	0	0	0
ENSMUSG00000077248	2	2	0	1	0	0
ENSMUSG00000035842	134	116	131	84	102	139
ENSMUSG00000032818	0	0	0	0	0	0
ENSMUSG00000109919	0	0	0	0	0	0
ENSMUSG00000089828	0	0	0	0	0	0
ENSMUSG00000084818	0	0	0	0	0	0
ENSMUSG00000089826	0	0	0	0	0	0
ENSMUSG00000111195	0	0	0	0	0	0
ENSMUSG00000084811	0	0	0	0	0	0
ENSMUSG00000089822	0	0	0	0	0	0
ENSMUSG00000031549	0	0	0	2	0	0
ENSMUSG00000037537	0	0	0	0	0	0
ENSMUSG00000094165	0	0	0	0	0	0
ENSMUSG00000114115	0	0	0	0	0	0
ENSMUSG00000114111	0	0	0	0	0	0
ENSMUSG00000093957	0	0	0	0	0	0
ENSMUSG00000093113	0	0	0	0	0	0
ENSMUSG00000101578	0	0	0	0	0	0
ENSMUSG00000088067	0	0	0	0	0	0
ENSMUSG00000088064	0	0	0	0	0	0
ENSMUSG00000088063	0	0	0	0	0	0
ENSMUSG00000088060	0	0	0	0	0	0
ENSMUSG00000082660	0	0	0	1	0	0
ENSMUSG00000085858	0	0	0	0	0	0
ENSMUSG00000111334	25	8	23	3	10	14
ENSMUSG00000085522	0	0	0	0	0	0
ENSMUSG00000111871	4	0	0	0	0	0
ENSMUSG00000109099	0	0	0	0	0	0
ENSMUSG00000085856	0	0	0	0	0	0
ENSMUSG00000093116	0	0	0	0	0	0
ENSMUSG00000112722	0	0	0	0	0	0
ENSMUSG00000106309	0	0	0	0	0	0
ENSMUSG00000033036	1	5	2	6	5	2

ENSMUSG00000103259	0	0	0	0	0	0
ENSMUSG00000061798	4	0	0	0	0	0
ENSMUSG00000103257	2	0	0	0	0	0
ENSMUSG00000103250	0	0	0	0	0	0
ENSMUSG00000087561	0	0	0	0	0	0
ENSMUSG00000088398	1	0	0	0	0	0
ENSMUSG00000087568	0	0	0	0	0	0
ENSMUSG00000040614	0	0	0	0	0	0
ENSMUSG00000013611	0	0	0	0	0	0
ENSMUSG00000082664	0	0	0	0	0	0
ENSMUSG00000081044	0	0	0	0	0	0
ENSMUSG00000065034	3	0	2	1	1	1
ENSMUSG00000051726	6	0	2	0	2	3
ENSMUSG00000100508	0	0	0	0	0	0
ENSMUSG00000100509	0	0	0	0	0	0
ENSMUSG00000100506	0	0	0	0	0	0
ENSMUSG00000100507	0	0	0	0	0	0
ENSMUSG00000100504	0	0	0	0	0	0
ENSMUSG00000100505	21	12	15	13	18	20
ENSMUSG00000100502	0	0	0	3	0	2
ENSMUSG00000100503	0	0	0	0	0	0
ENSMUSG00000100500	0	0	0	0	0	0
ENSMUSG00000100501	0	0	0	0	0	0
ENSMUSG00000022269	0	4	0	0	7	0
ENSMUSG00000019146	0	0	0	0	0	0
ENSMUSG00000022262	2	0	3	0	0	0
ENSMUSG00000084031	0	0	0	0	0	0
ENSMUSG00000084032	0	0	0	0	2	0
ENSMUSG00000084034	0	0	0	0	0	0
ENSMUSG00000084039	0	0	0	0	0	0
ENSMUSG00000084038	0	0	0	0	0	0
ENSMUSG00000111148	2	0	0	0	0	0
ENSMUSG00000065509	1	2	1	0	2	1
ENSMUSG00000095843	0	0	0	1	0	1
ENSMUSG00000095841	0	0	0	0	0	0
ENSMUSG00000061080	114	89	98	128	153	154
ENSMUSG00000095849	0	0	0	0	0	0
ENSMUSG00000106314	0	0	0	0	0	0
ENSMUSG00000037263	0	5	5	12	7	9
ENSMUSG00000024957	0	4	0	4	4	4
ENSMUSG00000058904	0	0	0	0	0	0
ENSMUSG00000102618	0	0	7	0	0	2
ENSMUSG00000102619	10	12	8	10	14	13
ENSMUSG00000102610	1	0	1	0	2	0
ENSMUSG00000112333	0	0	0	0	0	0
ENSMUSG00000112330	0	0	0	0	0	0
ENSMUSG00000102613	0	0	0	0	0	0
ENSMUSG00000102614	0	0	0	0	0	0
ENSMUSG00000112337	0	0	0	4	0	0
ENSMUSG00000039154	5	10	8	4	4	22
ENSMUSG00000039155	0	0	0	0	0	0
ENSMUSG00000035780	0	0	0	0	0	0
ENSMUSG00000035785	0	0	0	0	0	0
ENSMUSG00000036872	0	0	4	0	0	0
ENSMUSG00000036875	96	84	138	46	72	78
ENSMUSG00000028655	0	0	3	0	0	0
ENSMUSG00000028654	8	20	14	6	4	4
ENSMUSG00000093251	1	1	0	1	0	0

ENSMUSG00000038209	0	0	0	0	0	0
ENSMUSG00000106228	0	0	0	0	0	0
ENSMUSG00000038203	0	0	0	0	0	0
ENSMUSG00000113062	0	0	0	0	0	0
ENSMUSG00000034785	0	0	0	0	0	0
ENSMUSG00000113066	0	0	0	0	0	0
ENSMUSG00000063626	7	2	5	8	9	8
ENSMUSG00000051190	0	0	0	0	0	0
ENSMUSG00000114599	0	0	3	0	0	0
ENSMUSG00000111511	0	0	0	0	0	0
ENSMUSG00000111510	0	0	0	0	0	0
ENSMUSG00000111517	0	0	0	0	0	0
ENSMUSG00000109595	0	0	0	0	0	0
ENSMUSG00000114590	0	0	0	0	0	0
ENSMUSG00000114591	6	9	19	5	6	7
ENSMUSG00000109598	0	0	0	0	0	0
ENSMUSG00000111518	0	0	0	0	0	0
ENSMUSG00000114594	0	0	0	0	0	0
ENSMUSG00000114597	0	0	0	0	0	0
ENSMUSG00000106618	0	1	0	0	0	0
ENSMUSG00000106619	0	0	0	0	0	1
ENSMUSG00000106616	0	0	0	0	0	0
ENSMUSG00000050822	4	0	0	4	0	0
ENSMUSG00000067795	0	0	2	0	0	2
ENSMUSG00000050824	0	0	0	0	0	0
ENSMUSG00000095603	0	0	0	0	0	0
ENSMUSG00000051401	30	46	35	7	8	10
ENSMUSG00000104616	0	3	0	0	0	0
ENSMUSG00000104617	0	0	0	0	0	0
ENSMUSG00000104614	0	0	0	0	0	0
ENSMUSG00000104610	0	0	0	0	0	0
ENSMUSG00000059695	0	0	0	0	0	0
ENSMUSG00000104619	0	0	0	0	0	0
ENSMUSG00000096048	0	0	0	0	0	0
ENSMUSG00000096907	0	0	0	0	0	0
ENSMUSG00000114897	0	0	0	0	0	0
ENSMUSG00000065048	0	0	0	0	0	0
ENSMUSG00000096905	2	0	0	1	0	1
ENSMUSG00000097559	24	35	44	28	13	31
ENSMUSG00000097557	0	2	1	4	0	6
ENSMUSG00000097552	0	0	0	0	0	0
ENSMUSG00000097550	0	0	0	0	0	0
ENSMUSG00000097551	0	0	0	0	0	0
ENSMUSG00000067010	0	0	0	0	0	0
ENSMUSG00000114042	0	0	0	0	0	0
ENSMUSG00000114043	0	0	0	0	0	0
ENSMUSG00000114041	0	0	0	0	0	0
ENSMUSG00000114047	0	0	0	0	0	0
ENSMUSG00000114044	0	0	0	0	0	0
ENSMUSG00000110380	0	0	0	0	0	0
ENSMUSG00000114049	6	0	5	7	0	5
ENSMUSG00000025777	2	8	2	4	0	3
ENSMUSG00000054672	0	0	3	0	0	0
ENSMUSG00000054679	4	0	7	2	0	0
ENSMUSG00000042306	4	0	0	0	0	0
ENSMUSG00000107870	1	1	1	1	1	1
ENSMUSG00000110388	21	18	32	10	17	21
ENSMUSG00000110749	0	0	0	0	0	0

ENSMUSG00000044441	2	0	0	0	0	0
ENSMUSG00000044444	0	0	0	0	0	0
ENSMUSG00000093544	2	0	0	0	0	0
ENSMUSG00000110741	0	0	0	0	0	0
ENSMUSG00000103042	1	1	2	1	1	1
ENSMUSG00000058124	0	0	0	0	0	0
ENSMUSG00000110745	0	0	0	0	0	0
ENSMUSG00000093547	0	0	0	0	0	0
ENSMUSG00000042848	0	0	0	0	0	0
ENSMUSG00000042849	0	0	0	0	0	0
ENSMUSG00000080250	0	0	0	0	0	0
ENSMUSG00000087752	0	0	0	1	0	0
ENSMUSG00000042845	0	0	4	0	0	0
ENSMUSG00000046352	3	0	5	0	3	0
ENSMUSG00000046354	0	0	0	0	0	0
ENSMUSG00000060548	108	101	143	190	257	227
ENSMUSG00000021298	28	25	53	13	17	18
ENSMUSG00000057093	94	96	89	50	49	82
ENSMUSG00000103040	38	36	30	48	38	63
ENSMUSG00000024245	24	28	14	21	26	20
ENSMUSG00000100606	0	0	0	0	0	0
ENSMUSG00000095309	0	0	0	0	0	0
ENSMUSG00000095301	0	0	0	1	0	0
ENSMUSG00000095300	0	0	0	0	0	0
ENSMUSG00000095302	0	0	0	0	0	0
ENSMUSG00000095305	0	0	0	0	0	0
ENSMUSG00000095307	0	0	0	0	0	0
ENSMUSG00000057321	0	0	0	0	0	0
ENSMUSG00000113809	0	0	0	0	0	0
ENSMUSG00000113808	0	0	0	0	0	3
ENSMUSG00000113803	0	0	0	0	0	0
ENSMUSG00000108942	1	2	1	3	1	1
ENSMUSG00000113801	0	0	0	0	0	0
ENSMUSG00000113800	3	0	0	4	3	3
ENSMUSG00000098306	0	0	0	0	0	0
ENSMUSG00000098307	0	0	0	0	0	0
ENSMUSG00000113805	0	0	0	0	0	0
ENSMUSG00000089454	0	0	0	0	0	0
ENSMUSG00000100907	0	0	0	0	0	0
ENSMUSG00000100905	0	0	0	0	0	0
ENSMUSG00000108948	0	0	0	0	0	0
ENSMUSG00000108949	9	4	0	0	0	5
ENSMUSG00000093744	0	0	0	0	0	0
ENSMUSG00000105664	0	0	2	0	0	0
ENSMUSG00000081610	0	0	0	0	0	0
ENSMUSG00000081611	5	2	5	3	5	3
ENSMUSG00000081616	0	0	0	0	0	0
ENSMUSG00000081618	0	0	0	0	0	0
ENSMUSG00000114769	0	0	0	0	0	0
ENSMUSG00000077081	0	0	0	0	0	0
ENSMUSG00000077085	0	0	0	0	0	0
ENSMUSG00000042156	253	204	268	81	115	83
ENSMUSG00000105845	0	0	0	0	3	0
ENSMUSG00000086969	29	20	29	12	11	16
ENSMUSG00000102814	0	0	0	0	0	0
ENSMUSG00000093460	0	0	0	0	0	0
ENSMUSG00000093461	0	0	0	0	0	0
ENSMUSG00000109322	0	0	0	0	0	0

ENSMUSG00000086960	0	0	0	0	0	0
ENSMUSG00000086963	0	0	0	4	0	0
ENSMUSG00000093469	0	0	0	0	0	0
ENSMUSG00000103604	0	0	0	0	0	0
ENSMUSG00000048004	66	95	32	73	47	168
ENSMUSG00000114767	0	0	0	0	0	0
ENSMUSG00000090849	0	0	0	0	0	0
ENSMUSG00000048001	0	0	0	0	3	0
ENSMUSG00000048003	10	0	5	0	0	2
ENSMUSG00000092636	0	0	0	0	0	0
ENSMUSG00000112071	2	2	7	0	7	4
ENSMUSG00000094163	0	0	0	0	0	0
ENSMUSG00000094160	0	0	0	0	0	0
ENSMUSG00000094161	0	0	0	0	1	0
ENSMUSG00000112075	2	2	2	0	0	0
ENSMUSG00000094167	0	0	0	0	0	0
ENSMUSG00000093796	0	0	0	0	0	0
ENSMUSG00000112076	0	0	0	0	0	0
ENSMUSG00000112079	0	0	0	0	0	0
ENSMUSG00000112078	0	0	0	0	0	2
ENSMUSG00000094168	0	0	0	0	0	0
ENSMUSG00000017499	36	40	34	21	18	38
ENSMUSG00000109582	0	0	0	0	0	0
ENSMUSG00000092633	0	0	0	1	0	0
ENSMUSG00000103606	0	0	0	0	0	0
ENSMUSG00000099499	0	0	0	0	0	0
ENSMUSG00000099498	0	0	0	0	0	0
ENSMUSG00000099495	0	0	0	0	0	0
ENSMUSG00000099494	0	0	0	0	0	0
ENSMUSG00000099497	0	0	0	0	0	0
ENSMUSG00000103607	0	0	0	0	0	0
ENSMUSG00000099491	0	0	0	0	0	0
ENSMUSG00000099490	0	0	0	0	0	0
ENSMUSG00000099493	0	0	0	0	0	0
ENSMUSG00000107376	0	0	0	0	0	0
ENSMUSG00000046438	0	0	0	0	0	0
ENSMUSG00000086349	0	0	0	0	0	0
ENSMUSG00000086348	0	0	0	0	0	0
ENSMUSG00000046435	0	0	0	0	0	0
ENSMUSG00000110654	2	6	2	2	0	6
ENSMUSG00000112172	0	0	0	0	0	0
ENSMUSG00000046431	0	0	0	0	0	0
ENSMUSG00000097923	0	0	0	0	0	0
ENSMUSG00000112175	0	0	0	0	0	0
ENSMUSG00000095032	0	0	0	0	1	0
ENSMUSG00000112177	0	0	0	0	0	0
ENSMUSG00000095031	0	0	0	0	0	0
ENSMUSG00000090007	2	10	18	1	9	8
ENSMUSG00000021381	0	0	0	0	0	0
ENSMUSG00000000159	0	2	0	0	0	0
ENSMUSG00000049062	0	0	0	0	0	0
ENSMUSG00000000157	0	0	0	0	0	0
ENSMUSG00000000154	6	0	18	4	13	0
ENSMUSG00000087760	0	0	0	0	0	0
ENSMUSG00000087761	0	0	0	0	0	0
ENSMUSG00000087764	3	1	0	0	0	0
ENSMUSG00000087765	0	0	0	0	0	0
ENSMUSG00000087766	1	0	0	0	0	0

ENSMUSG00000087769	0	0	0	0	0	0
ENSMUSG00000114355	0	0	0	0	0	0
ENSMUSG00000000411	0	3	10	0	6	5
ENSMUSG00000101540	0	0	0	0	0	0
ENSMUSG00000047384	0	1	2	0	0	2
ENSMUSG00000047383	0	4	0	12	0	0
ENSMUSG00000083449	2	2	12	5	3	7
ENSMUSG00000083448	0	0	0	0	0	0
ENSMUSG00000083445	2	1	0	0	1	1
ENSMUSG00000083444	0	0	0	0	0	0
ENSMUSG00000037259	85	92	68	34	40	49
ENSMUSG00000083446	0	0	0	0	0	1
ENSMUSG00000083441	0	0	0	0	0	0
ENSMUSG00000060673	0	0	0	2	0	0
ENSMUSG00000031949	142	141	179	173	136	188
ENSMUSG00000044528	14	12	11	14	0	5
ENSMUSG00000107874	0	0	0	0	0	0
ENSMUSG00000108059	9	9	4	4	0	4
ENSMUSG00000042918	95	76	88	69	105	131
ENSMUSG00000071275	0	0	0	0	0	0
ENSMUSG00000084239	0	0	0	0	0	0
ENSMUSG00000084238	0	0	0	0	0	0
ENSMUSG00000084237	0	0	0	0	0	0
ENSMUSG00000084236	0	0	0	0	0	0
ENSMUSG00000084234	0	0	0	0	0	0
ENSMUSG00000075598	0	0	0	0	1	0
ENSMUSG00000075599	0	0	0	0	0	0
ENSMUSG00000085728	0	0	0	0	0	0
ENSMUSG00000080938	0	0	0	0	0	0
ENSMUSG00000080939	0	2	4	0	0	0
ENSMUSG00000085722	0	0	0	0	0	0
ENSMUSG00000085721	0	0	0	0	0	0
ENSMUSG00000080930	0	0	0	0	0	0
ENSMUSG00000080933	0	0	0	0	0	0
ENSMUSG00000078616	0	12	14	0	0	8
ENSMUSG00000078612	44	44	35	52	42	52
ENSMUSG00000078618	0	0	0	0	0	0
ENSMUSG00000065746	0	0	0	0	0	0
ENSMUSG00000065747	0	0	0	0	0	0
ENSMUSG00000065745	0	0	0	0	0	0
ENSMUSG00000065742	0	0	0	0	0	0
ENSMUSG00000065743	0	0	0	0	0	0
ENSMUSG00000044121	0	0	0	0	0	0
ENSMUSG00000044120	0	2	0	0	0	0
ENSMUSG00000065749	1	0	0	0	0	0
ENSMUSG00000032373	6	3	7	4	4	3
ENSMUSG00000087891	0	0	0	0	0	0
ENSMUSG00000062773	0	0	0	0	0	0
ENSMUSG00000044522	16	22	20	51	42	70
ENSMUSG00000062778	0	0	0	0	0	0
ENSMUSG00000068048	0	0	0	0	0	0
ENSMUSG00000085188	0	0	0	0	0	0
ENSMUSG00000085189	0	0	6	0	2	0
ENSMUSG00000085187	0	0	0	0	0	0
ENSMUSG00000085184	6	4	14	4	3	16
ENSMUSG00000085182	6	7	0	3	7	11
ENSMUSG00000085183	0	0	0	0	0	0
ENSMUSG00000085180	95	44	95	27	19	40

ENSMUSG00000085181	0	4	0	5	0	0
ENSMUSG00000053930	28	41	18	11	16	40
ENSMUSG00000100988	0	0	0	0	0	0
ENSMUSG00000100989	0	0	0	2	0	0
ENSMUSG00000100982	0	0	0	0	0	0
ENSMUSG00000100983	0	0	0	0	0	0
ENSMUSG00000100981	0	0	0	0	0	0
ENSMUSG00000100986	0	0	0	0	0	0
ENSMUSG00000100987	4	3	0	0	0	0
ENSMUSG00000100984	0	0	0	0	0	0
ENSMUSG00000087252	18	4	6	4	4	3
ENSMUSG00000006269	4	7	2	0	4	0
ENSMUSG00000094486	0	0	0	0	0	0
ENSMUSG00000079006	0	0	0	0	0	0
ENSMUSG00000079007	0	0	0	0	0	0
ENSMUSG00000032999	0	0	0	0	0	0
ENSMUSG00000103860	0	0	0	0	0	0
ENSMUSG00000103862	3	0	0	2	0	0
ENSMUSG00000103863	0	0	0	0	0	0
ENSMUSG00000081388	0	0	0	0	0	0
ENSMUSG00000019893	0	0	3	1	0	0
ENSMUSG00000019892	3	0	0	0	0	0
ENSMUSG00000019894	0	0	0	0	0	0
ENSMUSG00000034239	0	0	0	0	0	0
ENSMUSG00000098257	0	0	0	0	0	0
ENSMUSG00000079008	0	0	0	0	0	0
ENSMUSG00000079009	0	0	0	0	0	0
ENSMUSG00000089304	2	0	0	0	0	0
ENSMUSG00000105626	0	0	0	0	0	0
ENSMUSG00000019768	127	106	140	27	60	25
ENSMUSG00000114112	0	0	0	0	0	0
ENSMUSG00000019762	0	0	0	4	0	0
ENSMUSG00000019767	18	26	25	20	24	25
ENSMUSG00000037727	0	0	0	0	0	0
ENSMUSG00000037725	64	85	80	20	38	58
ENSMUSG00000094765	0	0	0	0	0	0
ENSMUSG00000076927	0	0	0	0	0	0
ENSMUSG00000076920	0	1	1	0	0	0
ENSMUSG00000108959	0	3	0	0	0	0
ENSMUSG00000108958	0	1	1	0	0	0
ENSMUSG00000034906	82	74	107	39	72	62
ENSMUSG00000094767	0	0	0	0	0	0
ENSMUSG00000108952	0	0	0	0	0	0
ENSMUSG00000108955	0	3	0	7	10	9
ENSMUSG00000108957	71	78	69	66	65	71
ENSMUSG00000108956	9	15	3	8	32	16
ENSMUSG00000091248	0	0	0	0	0	0
ENSMUSG00000018387	45	32	51	38	30	52
ENSMUSG00000105853	0	0	0	0	0	0
ENSMUSG00000102804	0	0	0	0	0	0
ENSMUSG00000043366	157	147	74	86	79	99
ENSMUSG00000030268	106	92	135	31	51	49
ENSMUSG00000102801	0	0	0	0	0	0
ENSMUSG00000102803	0	0	0	0	2	0
ENSMUSG00000102802	0	0	0	0	0	0
ENSMUSG00000105859	0	0	0	0	0	0
ENSMUSG00000113226	9	5	5	8	5	9
ENSMUSG00000105702	2	4	0	0	0	2

ENSMUSG00000101574	0	0	0	0	0	0
ENSMUSG00000101575	0	0	0	0	0	0
ENSMUSG00000101576	0	0	0	0	0	0
ENSMUSG00000101570	0	0	0	0	0	0
ENSMUSG00000101571	0	0	0	0	0	0
ENSMUSG00000101573	0	0	0	0	0	0
ENSMUSG00000056436	10	20	0	4	0	0
ENSMUSG00000101579	0	0	0	0	0	0
ENSMUSG00000021415	0	0	0	2	0	0
ENSMUSG00000100803	0	0	0	0	0	0
ENSMUSG00000094922	0	0	0	0	0	0
ENSMUSG00000087246	0	2	2	0	0	0
ENSMUSG00000088210	0	0	0	0	0	0
ENSMUSG00000087241	0	0	0	0	0	0
ENSMUSG00000058005	0	0	0	0	0	0
ENSMUSG00000088218	0	0	0	0	0	0
ENSMUSG00000104898	0	0	0	0	0	0
ENSMUSG00000029660	2	0	5	3	2	2
ENSMUSG00000104895	0	0	0	0	0	0
ENSMUSG00000104892	0	0	0	0	0	0
ENSMUSG00000021418	152	196	124	183	142	217
ENSMUSG00000060530	9	8	10	8	2	8
ENSMUSG00000106482	0	0	0	0	0	0
ENSMUSG00000113122	0	0	0	0	0	0
ENSMUSG00000089164	1	0	0	0	0	0
ENSMUSG00000111898	0	0	0	0	0	0
ENSMUSG00000113123	2	0	2	1	1	4
ENSMUSG00000089161	0	0	0	1	0	0
ENSMUSG00000089162	0	0	0	0	0	0
ENSMUSG00000089163	0	0	0	0	0	0
ENSMUSG00000088718	0	0	0	0	0	0
ENSMUSG00000111891	0	0	0	0	0	0
ENSMUSG00000002006	181	156	241	108	102	122
ENSMUSG00000111894	0	0	0	0	0	0
ENSMUSG00000106139	7	9	24	13	12	9
ENSMUSG00000106138	0	0	0	0	0	0
ENSMUSG00000009580	0	0	0	0	0	0
ENSMUSG00000106134	0	0	0	0	0	0
ENSMUSG00000106136	0	0	0	0	0	0
ENSMUSG00000106131	0	0	0	0	0	0
ENSMUSG00000106130	0	0	0	0	0	2
ENSMUSG00000009588	0	0	0	0	0	0
ENSMUSG00000028197	46	13	11	2	0	8
ENSMUSG00000088715	0	0	0	0	0	0
ENSMUSG00000089307	0	0	0	0	0	0
ENSMUSG00000088716	0	0	0	0	0	0
ENSMUSG00000094360	3	7	2	2	3	3
ENSMUSG00000094361	0	0	0	1	0	0
ENSMUSG00000094362	0	0	0	0	0	0
ENSMUSG00000094363	0	0	0	0	0	0
ENSMUSG00000094364	0	0	0	0	0	0
ENSMUSG00000094366	0	2	0	0	0	0
ENSMUSG00000022762	5	0	0	0	0	3
ENSMUSG00000112277	0	0	0	0	0	0
ENSMUSG00000094369	0	0	0	0	0	0
ENSMUSG00000112275	0	0	0	0	0	0
ENSMUSG00000112272	2	3	2	3	2	1
ENSMUSG00000112271	0	0	0	0	0	0

ENSMUSG00000112270	0	0	0	0	0	0
ENSMUSG00000017652	51	62	74	73	64	94
ENSMUSG00000092932	0	0	0	0	0	0
ENSMUSG00000052848	0	0	0	1	0	4
ENSMUSG00000020690	0	0	0	0	0	0
ENSMUSG00000092208	2	0	0	0	0	0
ENSMUSG00000028444	38	39	13	19	8	17
ENSMUSG00000092204	0	0	0	0	0	0
ENSMUSG00000035626	2	0	0	0	0	0
ENSMUSG00000108470	0	0	0	0	0	0
ENSMUSG00000063764	0	0	0	0	0	0
ENSMUSG00000015879	16	27	12	21	10	9
ENSMUSG00000114944	0	0	0	0	0	0
ENSMUSG00000114949	2	0	0	0	2	0
ENSMUSG00000114948	0	0	0	0	0	0
ENSMUSG00000106791	0	5	4	4	5	12
ENSMUSG00000113639	0	0	0	0	0	0
ENSMUSG00000106794	0	0	0	0	0	0
ENSMUSG00000038010	108	58	67	56	49	34
ENSMUSG00000038011	0	0	6	4	10	3
ENSMUSG00000113632	0	0	0	0	0	0
ENSMUSG00000097182	7	10	0	0	0	5
ENSMUSG00000113635	0	0	0	0	0	0
ENSMUSG00000113636	2	3	4	5	3	2
ENSMUSG00000097186	2	2	0	0	0	0
ENSMUSG00000016524	0	0	0	0	0	0
ENSMUSG00000016526	12	14	8	0	6	15
ENSMUSG00000094985	0	0	0	0	0	0
ENSMUSG00000094986	2	0	0	0	0	0
ENSMUSG00000094987	0	0	0	0	0	0
ENSMUSG00000109149	10	6	6	0	6	4
ENSMUSG00000109148	0	0	0	0	0	0
ENSMUSG00000094983	1	0	1	0	1	0
ENSMUSG00000109145	0	0	0	0	0	0
ENSMUSG00000105446	0	1	0	0	1	0
ENSMUSG00000105440	0	0	0	0	0	0
ENSMUSG00000105441	1	0	0	0	0	0
ENSMUSG00000109143	5	7	3	2	3	6
ENSMUSG00000109142	0	0	0	0	0	0
ENSMUSG00000082146	0	0	0	0	0	0
ENSMUSG00000106869	6	6	2	0	2	1
ENSMUSG00000106863	0	0	0	0	0	0
ENSMUSG00000106862	0	0	0	0	0	0
ENSMUSG00000106860	0	0	1	0	0	1
ENSMUSG00000066242	0	0	0	0	0	0
ENSMUSG00000066240	0	0	0	0	0	0
ENSMUSG00000066241	0	0	0	0	0	0
ENSMUSG00000024897	382	344	359	187	294	245
ENSMUSG00000021123	12	14	29	0	10	0
ENSMUSG00000111702	0	0	0	0	0	0
ENSMUSG00000111707	0	0	0	0	0	0
ENSMUSG00000111704	0	0	0	0	0	0
ENSMUSG00000096270	0	0	0	0	0	0
ENSMUSG00000096272	0	0	0	0	0	0
ENSMUSG00000096275	0	0	0	0	0	0
ENSMUSG00000096276	0	0	0	0	0	0
ENSMUSG00000096277	0	0	0	0	0	0
ENSMUSG00000114367	0	0	0	0	0	0

ENSMUSG00000114365	0	0	0	0	0	0
ENSMUSG00000114364	0	0	0	0	0	0
ENSMUSG00000114363	0	0	0	0	0	0
ENSMUSG00000114361	0	0	0	0	0	0
ENSMUSG00000114360	0	0	0	0	0	0
ENSMUSG00000104799	0	0	0	0	0	0
ENSMUSG00000104793	5	0	10	3	9	10
ENSMUSG00000108235	0	3	0	0	0	0
ENSMUSG00000104797	0	0	0	0	0	1
ENSMUSG00000104796	0	0	0	0	0	0
ENSMUSG00000093985	12	15	24	8	15	15
ENSMUSG00000026694	379	326	334	365	407	393
ENSMUSG00000093981	0	0	0	0	0	0
ENSMUSG00000093980	0	0	0	0	0	0
ENSMUSG00000026692	4	17	16	16	6	17
ENSMUSG00000111821	0	0	0	0	3	0
ENSMUSG00000057424	0	0	0	0	0	0
ENSMUSG00000057425	0	0	0	0	0	0
ENSMUSG00000104425	0	0	0	0	0	0
ENSMUSG00000104424	0	0	0	0	0	0
ENSMUSG00000113699	0	0	0	0	0	0
ENSMUSG00000104420	58	95	91	41	29	39
ENSMUSG00000104423	0	0	0	0	0	0
ENSMUSG00000104422	0	0	0	0	0	0
ENSMUSG00000104429	0	0	0	0	0	0
ENSMUSG00000104428	0	0	0	1	2	2
ENSMUSG00000083939	0	0	0	0	0	0
ENSMUSG00000083938	0	0	0	0	0	0
ENSMUSG00000091723	0	0	0	0	0	0
ENSMUSG00000091722	0	0	0	0	0	0
ENSMUSG00000091721	0	3	0	0	0	0
ENSMUSG00000083931	0	0	0	0	0	0
ENSMUSG00000083930	0	0	0	0	0	0
ENSMUSG00000083933	0	0	0	0	0	0
ENSMUSG00000083934	0	0	0	0	0	0
ENSMUSG00000083937	0	0	0	0	0	0
ENSMUSG00000083936	7	5	5	0	5	4
ENSMUSG00000090474	0	0	0	0	0	0
ENSMUSG00000025927	0	0	0	0	0	0
ENSMUSG00000025929	0	0	0	0	0	0
ENSMUSG00000074555	0	0	1	0	0	0
ENSMUSG00000098303	0	0	1	0	0	0
ENSMUSG00000078276	0	0	0	0	0	0
ENSMUSG00000056771	88	93	63	93	124	86
ENSMUSG00000056777	2	0	2	1	2	2
ENSMUSG00000046258	0	0	0	0	0	0
ENSMUSG00000078270	0	0	0	0	0	0
ENSMUSG00000073988	40	42	37	31	34	54
ENSMUSG00000074952	0	0	0	0	0	0
ENSMUSG00000074955	0	0	0	0	0	0
ENSMUSG00000077400	0	0	0	0	0	0
ENSMUSG00000045508	0	0	0	0	0	0
ENSMUSG00000045509	3	2	0	7	3	3
ENSMUSG00000095110	0	0	0	0	0	0
ENSMUSG00000095113	0	0	0	0	0	0
ENSMUSG00000045502	0	0	6	0	0	0
ENSMUSG00000095116	0	0	0	0	0	0
ENSMUSG00000079853	2	0	0	0	0	0

ENSMUSG00000079852	2	0	6	0	0	0
ENSMUSG00000062226	0	0	0	0	0	0
ENSMUSG00000080773	0	4	2	0	2	0
ENSMUSG00000080770	0	0	0	0	0	0
ENSMUSG00000080771	0	0	0	0	0	0
ENSMUSG00000080774	3	1	0	0	0	0
ENSMUSG00000080778	0	0	0	0	0	0
ENSMUSG00000098538	0	0	0	0	0	0
ENSMUSG00000111285	0	0	0	0	0	0
ENSMUSG00000111284	0	0	0	0	0	0
ENSMUSG00000111283	13	16	6	12	15	15
ENSMUSG00000111282	0	0	0	0	0	0
ENSMUSG00000048905	8	0	0	4	0	0
ENSMUSG00000053856	0	0	0	0	0	0
ENSMUSG00000053852	0	0	0	1	0	0
ENSMUSG00000094647	0	0	0	0	0	0
ENSMUSG00000093616	0	0	0	0	0	0
ENSMUSG00000094643	0	0	0	0	0	1
ENSMUSG00000094642	0	0	0	0	0	0
ENSMUSG00000094641	0	0	0	0	0	0
ENSMUSG00000094649	8	7	6	4	5	5
ENSMUSG00000094648	2	1	1	1	0	0
ENSMUSG00000081796	0	0	0	0	0	0
ENSMUSG00000081793	0	2	2	0	0	2
ENSMUSG00000081791	0	0	2	1	0	0
ENSMUSG00000030165	4	7	10	3	0	0
ENSMUSG00000031330	0	2	0	0	0	0
ENSMUSG00000081798	0	5	3	2	3	5
ENSMUSG00000006930	34	29	50	26	25	30
ENSMUSG00000046652	6	23	14	9	7	22
ENSMUSG00000055452	38	47	0	0	0	39
ENSMUSG00000055456	0	0	0	0	0	0
ENSMUSG00000081423	0	0	0	0	0	2
ENSMUSG00000081422	0	2	2	2	0	1
ENSMUSG00000081424	0	3	3	0	0	0
ENSMUSG00000081426	0	0	0	0	0	0
ENSMUSG00000092566	0	0	0	0	0	0
ENSMUSG00000092567	0	0	0	0	0	0
ENSMUSG00000092561	0	0	0	0	0	0
ENSMUSG00000092562	0	0	0	0	0	0
ENSMUSG00000077543	0	0	0	0	0	0
ENSMUSG00000077541	0	0	0	0	0	0
ENSMUSG00000092568	0	0	0	0	0	0
ENSMUSG00000056959	0	0	0	0	0	0
ENSMUSG00000099847	0	0	0	0	0	0
ENSMUSG00000092813	1	0	0	0	0	0
ENSMUSG00000082212	0	0	0	0	6	0
ENSMUSG00000082211	0	0	0	0	0	0
ENSMUSG00000082217	0	0	0	0	0	0
ENSMUSG00000082216	0	0	0	0	0	0
ENSMUSG00000082215	0	0	0	0	0	0
ENSMUSG00000094354	0	0	0	0	0	0
ENSMUSG00000082218	0	0	0	0	0	0
ENSMUSG00000111276	2	0	0	0	0	0
ENSMUSG00000112266	4	0	0	0	3	0
ENSMUSG00000099285	0	0	0	0	0	0
ENSMUSG00000099287	4	4	6	2	5	4
ENSMUSG00000112261	0	0	0	0	0	0

ENSMUSG00000032517	0	0	5	8	3	0
ENSMUSG00000112262	0	0	0	0	0	0
ENSMUSG00000037593	27	58	44	68	54	74
ENSMUSG00000043614	46	17	38	10	4	23
ENSMUSG00000087482	0	0	0	0	0	0
ENSMUSG00000098880	0	0	0	0	0	0
ENSMUSG00000098883	0	0	0	0	0	0
ENSMUSG00000098887	0	0	0	0	0	0
ENSMUSG00000098888	0	0	0	0	0	0
ENSMUSG00000068940	0	0	0	0	0	0
ENSMUSG00000068947	0	2	0	0	0	0
ENSMUSG00000068948	0	0	0	0	0	0
ENSMUSG00000084688	0	0	0	1	0	0
ENSMUSG00000084689	0	0	0	0	0	0
ENSMUSG00000084682	0	0	0	0	0	0
ENSMUSG00000084683	0	0	0	0	0	0
ENSMUSG00000084684	0	0	0	0	0	0
ENSMUSG00000075149	0	0	0	0	0	0
ENSMUSG00000075148	2	0	0	0	3	0
ENSMUSG00000084849	0	0	0	0	0	0
ENSMUSG00000075145	0	0	0	0	0	0
ENSMUSG00000075144	0	0	0	0	0	0
ENSMUSG00000075146	0	0	0	0	0	0
ENSMUSG00000075140	0	0	0	0	0	0
ENSMUSG00000075143	0	0	0	0	0	0
ENSMUSG00000075142	0	0	0	0	0	0
ENSMUSG00000109743	3	1	2	3	9	5
ENSMUSG00000109742	0	0	0	0	0	0
ENSMUSG00000070390	146	171	223	50	68	90
ENSMUSG00000061361	0	0	0	0	0	0
ENSMUSG00000102290	0	0	0	0	0	0
ENSMUSG00000102291	3	3	0	0	3	0
ENSMUSG00000064626	1	0	0	0	0	0
ENSMUSG00000099362	0	0	0	0	0	0
ENSMUSG00000107226	14	22	8	3	15	9
ENSMUSG00000092219	0	0	0	0	0	0
ENSMUSG00000107223	0	0	2	0	1	0
ENSMUSG00000107220	0	0	0	0	0	0
ENSMUSG00000033740	3	0	0	0	0	0
ENSMUSG00000081947	0	0	0	0	0	0
ENSMUSG00000104815	0	0	0	0	0	0
ENSMUSG00000104816	3	0	2	2	0	0
ENSMUSG00000101971	0	0	0	0	0	0
ENSMUSG00000101974	0	0	0	0	0	0
ENSMUSG00000101975	0	0	0	0	0	0
ENSMUSG00000101977	0	0	0	0	0	0
ENSMUSG00000101978	0	0	0	0	0	0
ENSMUSG00000092210	0	0	0	2	0	0
ENSMUSG00000078799	0	0	0	0	0	0
ENSMUSG00000092211	0	0	0	0	0	0
ENSMUSG00000104812	0	0	0	0	0	0
ENSMUSG00000078793	0	0	0	0	0	0
ENSMUSG00000092216	0	0	0	0	0	0
ENSMUSG00000074236	0	0	0	0	0	0
ENSMUSG00000074237	0	0	0	0	0	0
ENSMUSG00000078796	0	0	0	0	0	0
ENSMUSG00000078795	4	4	5	0	3	0
ENSMUSG00000092217	0	0	0	0	0	0

ENSMUSG00000092214	0	0	0	0	0	0
ENSMUSG00000020428	0	0	0	0	0	0
ENSMUSG00000078597	0	0	0	0	0	0
ENSMUSG00000020429	0	0	0	0	0	0
ENSMUSG00000078591	3	6	3	6	2	0
ENSMUSG00000065283	0	0	0	0	0	0
ENSMUSG00000081863	0	0	0	0	0	0
ENSMUSG00000050087	6	3	0	0	0	0
ENSMUSG00000078420	0	0	0	0	0	3
ENSMUSG00000062105	0	0	0	0	0	0
ENSMUSG00000089558	0	0	0	0	0	0
ENSMUSG00000062103	0	0	0	0	0	0
ENSMUSG00000089556	0	0	0	0	0	0
ENSMUSG00000089551	0	0	0	0	0	0
ENSMUSG00000089550	0	0	0	0	0	0
ENSMUSG00000089553	0	0	0	0	0	0
ENSMUSG00000089552	0	0	0	0	0	0
ENSMUSG00000095413	4	1	2	4	3	2
ENSMUSG00000063851	0	3	2	0	3	6
ENSMUSG00000095414	0	0	2	1	0	0
ENSMUSG00000035355	0	4	11	0	2	2
ENSMUSG00000102307	2	3	5	4	7	4
ENSMUSG00000102300	0	0	2	0	0	0
ENSMUSG00000102308	0	0	0	0	0	0
ENSMUSG00000102309	2	2	0	0	0	2
ENSMUSG00000038656	0	0	0	0	0	0
ENSMUSG00000038651	0	0	0	0	0	0
ENSMUSG00000069706	2	0	0	0	0	0
ENSMUSG00000069707	0	0	0	0	0	0
ENSMUSG00000069708	0	0	0	0	0	0
ENSMUSG00000096076	0	0	0	0	0	0
ENSMUSG00000096075	3	1	4	0	1	2
ENSMUSG00000096072	0	0	0	0	0	0
ENSMUSG00000096073	0	0	0	0	0	0
ENSMUSG00000096070	0	0	1	0	0	0
ENSMUSG00000096071	0	0	0	0	0	0
ENSMUSG00000031073	0	0	0	0	0	0
ENSMUSG00000034009	15	3	22	12	3	4
ENSMUSG00000034000	0	0	0	0	0	0
ENSMUSG00000063277	0	10	12	0	0	0
ENSMUSG00000042532	12	14	10	12	26	40
ENSMUSG00000104223	0	0	0	0	0	0
ENSMUSG00000104220	1	0	2	2	10	0
ENSMUSG00000104229	0	0	0	0	0	0
ENSMUSG00000104228	0	0	0	0	0	0
ENSMUSG00000106784	0	0	0	0	0	0
ENSMUSG00000060771	408	537	362	312	267	407
ENSMUSG00000094256	1	3	1	4	1	6
ENSMUSG00000059562	8	3	7	0	4	6
ENSMUSG00000100032	0	0	0	0	0	0
ENSMUSG00000088783	0	0	0	0	0	0
ENSMUSG00000061829	0	0	0	0	0	0
ENSMUSG00000100219	0	0	0	0	0	0
ENSMUSG00000100216	0	0	0	0	0	0
ENSMUSG00000100217	0	1	1	0	0	0
ENSMUSG00000061825	0	1	0	0	0	0
ENSMUSG00000104467	42	33	29	25	19	37
ENSMUSG00000100212	6	5	11	1	4	3

ENSMUSG00000109158	0	0	0	0	0	2
ENSMUSG00000100211	0	0	0	0	0	0
ENSMUSG00000043029	32	34	26	17	25	28
ENSMUSG00000105470	42	22	21	54	44	42
ENSMUSG00000068809	0	0	0	0	0	0
ENSMUSG00000095757	0	0	0	0	0	0
ENSMUSG00000095754	0	0	0	0	0	0
ENSMUSG00000095755	0	0	9	0	0	7
ENSMUSG00000095752	1	4	1	0	0	0
ENSMUSG00000095758	0	0	0	0	0	0
ENSMUSG00000095759	0	0	0	0	1	0
ENSMUSG00000088405	0	0	0	0	0	0
ENSMUSG00000088407	0	0	0	0	0	0
ENSMUSG00000088402	0	2	0	0	0	0
ENSMUSG00000088403	0	0	0	0	0	0
ENSMUSG00000058216	0	0	0	0	0	0
ENSMUSG00000105476	0	0	0	0	0	0
ENSMUSG00000088408	0	0	0	0	0	0
ENSMUSG00000088409	0	0	0	0	0	0
ENSMUSG00000049894	0	0	0	0	0	0
ENSMUSG00000105782	0	0	0	0	0	0
ENSMUSG00000065956	0	0	0	0	0	0
ENSMUSG00000094085	0	0	0	0	0	0
ENSMUSG00000094084	0	0	0	0	0	0
ENSMUSG00000064926	0	0	0	0	0	0
ENSMUSG00000094086	0	0	0	0	0	0
ENSMUSG00000094081	0	0	0	1	0	0
ENSMUSG00000094080	0	0	0	0	0	0
ENSMUSG00000094083	0	0	0	0	0	0
ENSMUSG00000094082	0	0	0	0	0	0
ENSMUSG00000086530	0	0	0	0	0	0
ENSMUSG00000086531	0	0	0	0	0	0
ENSMUSG00000062844	0	0	0	0	0	0
ENSMUSG00000111919	0	0	0	0	0	0
ENSMUSG00000111917	6	0	2	2	0	1
ENSMUSG00000111915	0	0	0	0	0	0
ENSMUSG00000036095	91	88	89	95	78	78
ENSMUSG00000111911	0	0	0	0	0	0
ENSMUSG00000111910	0	0	0	0	0	0
ENSMUSG00000023333	0	0	0	0	0	0
ENSMUSG00000106367	0	0	0	0	0	0
ENSMUSG00000093368	0	0	0	0	0	0
ENSMUSG00000039246	71	72	48	26	26	57
ENSMUSG00000093362	0	0	0	0	0	0
ENSMUSG00000009628	28	13	23	4	16	10
ENSMUSG00000022763	0	0	0	0	3	0
ENSMUSG00000002633	0	0	0	0	0	0
ENSMUSG00000052471	3	0	3	0	0	1
ENSMUSG00000113942	0	0	0	3	0	0
ENSMUSG00000045989	0	0	0	0	0	0
ENSMUSG00000112750	0	0	0	0	0	0
ENSMUSG00000112752	0	0	0	0	0	0
ENSMUSG00000112753	0	0	0	0	0	0
ENSMUSG00000055125	8	13	10	3	11	36
ENSMUSG00000112757	15	9	5	8	16	13
ENSMUSG00000024647	0	0	0	0	0	0
ENSMUSG00000020159	0	0	0	0	0	0
ENSMUSG00000031512	4	10	5	6	8	2

ENSMUSG00000092019	0	0	0	0	0	0
ENSMUSG00000097068	47	41	40	27	43	48
ENSMUSG00000097066	0	0	0	0	0	0
ENSMUSG00000096439	0	0	0	0	0	0
ENSMUSG00000097063	0	0	2	0	0	3
ENSMUSG00000096436	0	0	0	0	0	0
ENSMUSG00000096434	0	0	0	0	0	0
ENSMUSG00000038020	42	41	34	21	19	59
ENSMUSG00000110663	0	0	0	0	0	0
ENSMUSG00000110666	0	0	0	0	0	0
ENSMUSG00000110664	2	2	1	1	2	7
ENSMUSG00000110665	0	0	0	0	0	0
ENSMUSG00000113406	0	0	0	0	0	0
ENSMUSG00000113404	10	0	0	4	4	6
ENSMUSG00000113403	1	2	2	7	4	4
ENSMUSG00000113402	0	0	0	0	0	0
ENSMUSG00000113400	0	0	0	0	0	0
ENSMUSG00000113409	3	0	0	3	3	0
ENSMUSG00000113408	0	0	1	1	0	0
ENSMUSG00000003273	164	153	159	108	107	131
ENSMUSG00000105639	0	0	0	0	1	3
ENSMUSG00000003271	0	0	2	0	0	0
ENSMUSG00000051354	5	0	6	0	0	0
ENSMUSG00000003279	6	6	8	2	6	7
ENSMUSG00000105635	8	3	3	3	3	4
ENSMUSG00000114753	0	0	0	0	0	0
ENSMUSG00000114750	0	0	0	0	0	0
ENSMUSG00000093158	0	0	0	0	0	0
ENSMUSG00000114754	0	0	0	0	0	0
ENSMUSG00000095431	0	0	0	0	0	0
ENSMUSG00000082949	0	0	0	0	0	0
ENSMUSG00000023279	10	4	22	0	0	0
ENSMUSG00000082947	0	0	0	0	0	0
ENSMUSG00000082945	0	0	0	0	0	0
ENSMUSG00000082943	0	0	0	0	0	0
ENSMUSG00000082940	0	0	0	0	0	0
ENSMUSG00000108009	0	0	0	0	0	0
ENSMUSG00000113868	0	2	5	0	0	0
ENSMUSG00000108003	0	0	0	0	0	0
ENSMUSG00000108002	2	0	0	0	0	0
ENSMUSG00000108000	0	0	0	0	0	0
ENSMUSG00000108007	0	0	0	0	0	0
ENSMUSG00000108005	0	0	0	0	0	0
ENSMUSG00000108004	44	50	38	22	27	37
ENSMUSG00000083018	0	0	0	0	0	0
ENSMUSG00000036357	0	0	0	0	0	0
ENSMUSG00000083010	0	0	0	0	0	0
ENSMUSG00000083016	2	0	0	0	0	0
ENSMUSG00000083014	0	0	4	2	0	3
ENSMUSG00000083015	6	9	0	9	6	5
ENSMUSG00000085638	0	0	0	0	0	0
ENSMUSG00000052629	0	4	0	0	0	0
ENSMUSG00000098105	1	7	5	5	4	3
ENSMUSG00000085635	0	0	0	0	0	0
ENSMUSG00000040856	0	0	0	0	0	0
ENSMUSG00000111135	0	1	0	0	2	0
ENSMUSG00000111137	0	0	0	0	0	0
ENSMUSG00000108672	0	0	0	0	0	0

ENSMUSG00000097819	6	4	5	6	5	5
ENSMUSG00000096245	0	0	0	0	0	0
ENSMUSG00000001656	0	0	0	0	0	0
ENSMUSG00000001657	0	0	0	0	0	0
ENSMUSG00000001655	0	0	0	0	0	0
ENSMUSG00000026085	0	0	0	0	0	0
ENSMUSG00000026080	31	27	43	14	25	22
ENSMUSG00000095202	0	0	0	0	0	0
ENSMUSG00000096248	0	0	0	0	0	0
ENSMUSG00000045075	12	10	6	3	3	2
ENSMUSG00000109987	0	0	0	0	0	0
ENSMUSG00000108209	0	0	0	0	0	0
ENSMUSG00000104789	0	0	0	0	0	0
ENSMUSG00000081323	0	0	0	0	0	0
ENSMUSG00000081321	0	0	0	0	0	2
ENSMUSG00000081327	0	0	0	0	0	0
ENSMUSG00000081324	0	0	0	0	0	0
ENSMUSG00000095209	0	0	0	1	0	0
ENSMUSG00000099899	0	0	0	0	0	0
ENSMUSG00000099898	0	0	0	0	0	0
ENSMUSG00000092849	0	0	0	0	0	0
ENSMUSG00000092848	0	0	0	0	0	0
ENSMUSG00000112540	0	0	0	0	0	0
ENSMUSG00000092843	1	0	1	0	1	0
ENSMUSG00000099890	0	0	0	0	0	2
ENSMUSG00000092840	0	0	0	0	0	0
ENSMUSG00000092847	0	0	0	0	0	0
ENSMUSG00000027356	0	0	0	0	0	0
ENSMUSG00000027355	0	0	0	0	0	0
ENSMUSG00000099896	0	0	0	0	0	0
ENSMUSG00000104780	2	0	2	0	2	0
ENSMUSG00000104782	0	0	0	0	0	0
ENSMUSG00000046041	0	0	0	0	0	0
ENSMUSG00000104783	0	0	0	0	0	0
ENSMUSG00000104785	0	0	0	0	0	0
ENSMUSG00000098768	0	0	0	0	0	0
ENSMUSG00000090574	0	1	0	0	0	0
ENSMUSG00000090577	0	0	0	0	0	0
ENSMUSG00000035805	0	0	0	0	0	0
ENSMUSG00000098760	0	0	0	0	0	0
ENSMUSG00000090578	1	0	1	1	1	1
ENSMUSG00000053461	0	4	6	0	2	0
ENSMUSG00000073077	8	0	3	5	13	5
ENSMUSG00000053465	0	0	1	0	0	0
ENSMUSG00000032852	0	0	0	0	0	0
ENSMUSG00000080635	0	0	0	0	0	0
ENSMUSG00000032899	2	6	6	2	0	2
ENSMUSG00000080631	0	0	0	0	0	0
ENSMUSG00000032890	2	0	8	4	0	4
ENSMUSG00000032894	0	0	0	0	0	0
ENSMUSG00000078912	0	0	0	0	0	0
ENSMUSG00000078917	3	1	3	1	3	2
ENSMUSG00000052736	10	4	15	6	4	7
ENSMUSG00000065095	0	0	0	0	0	0
ENSMUSG00000037910	0	0	0	0	0	0
ENSMUSG00000031271	0	0	0	0	0	0
ENSMUSG00000031270	0	0	0	0	0	0
ENSMUSG00000101378	0	0	0	0	0	0

ENSMUSG00000093000	0	0	0	0	0	0
ENSMUSG00000093999	0	0	0	0	0	0
ENSMUSG00000026683	35	45	44	7	15	41
ENSMUSG00000037942	0	0	2	0	0	0
ENSMUSG00000037946	183	137	267	54	108	86
ENSMUSG00000037944	16	12	35	6	0	6
ENSMUSG00000030638	13	20	12	12	0	13
ENSMUSG00000030636	0	2	0	0	0	0
ENSMUSG00000073920	0	0	0	0	0	0
ENSMUSG00000073923	0	0	0	0	0	3
ENSMUSG00000073922	0	0	0	0	0	0
ENSMUSG00000073925	0	0	0	0	0	0
ENSMUSG00000101328	0	0	2	0	0	0
ENSMUSG00000073927	0	0	0	0	0	0
ENSMUSG00000101325	0	0	0	0	0	0
ENSMUSG00000073928	0	0	0	0	0	0
ENSMUSG00000101321	0	0	0	0	0	0
ENSMUSG00000101323	0	0	0	0	0	0
ENSMUSG00000071562	0	0	0	0	0	0
ENSMUSG00000071561	0	0	0	0	2	0
ENSMUSG00000033177	0	0	0	0	0	0
ENSMUSG00000087071	0	0	0	0	0	0
ENSMUSG00000087074	0	0	0	0	0	0
ENSMUSG00000087075	4	0	0	0	0	6
ENSMUSG00000087076	0	1	1	0	3	3
ENSMUSG00000087077	0	0	0	0	0	0
ENSMUSG00000082009	0	0	0	0	0	0
ENSMUSG00000099560	0	0	0	0	0	0
ENSMUSG00000099562	0	0	0	0	0	0
ENSMUSG00000099565	0	0	0	0	0	0
ENSMUSG00000099564	0	0	0	1	2	0
ENSMUSG00000099566	0	0	0	0	0	0
ENSMUSG00000019230	0	4	0	3	0	0
ENSMUSG00000019232	0	0	4	2	0	0
ENSMUSG00000083751	0	0	0	0	0	0
ENSMUSG00000089338	0	0	0	0	0	0
ENSMUSG00000089335	0	0	0	0	0	0
ENSMUSG00000089334	0	0	0	0	0	0
ENSMUSG00000084524	0	0	0	0	0	0
ENSMUSG00000089331	0	0	0	0	0	0
ENSMUSG00000089333	0	0	0	0	0	0
ENSMUSG00000084520	1	1	0	0	1	1
ENSMUSG00000100058	0	0	0	0	0	0
ENSMUSG00000100059	0	0	0	0	0	0
ENSMUSG00000100054	0	0	0	0	0	0
ENSMUSG00000100055	0	0	0	0	0	0
ENSMUSG00000041791	0	0	0	0	0	0
ENSMUSG00000100057	0	0	0	0	0	0
ENSMUSG00000100050	0	0	0	0	0	0
ENSMUSG00000100051	0	0	0	0	0	0
ENSMUSG00000100052	0	0	0	0	0	0
ENSMUSG00000102566	1	0	3	1	2	5
ENSMUSG00000102561	0	3	0	0	0	0
ENSMUSG00000107582	0	0	0	0	0	0
ENSMUSG00000107581	0	0	0	0	0	0
ENSMUSG00000107580	0	0	0	0	0	1
ENSMUSG00000107587	0	0	0	0	0	0
ENSMUSG00000107585	0	3	0	0	0	0

ENSMUSG00000107584	0	0	0	0	0	0
ENSMUSG00000000247	0	3	0	0	0	0
ENSMUSG00000000244	27	44	56	14	18	32
ENSMUSG00000087695	0	0	0	0	0	0
ENSMUSG00000100584	0	0	0	0	0	0
ENSMUSG00000100582	0	0	0	0	0	0
ENSMUSG00000065853	1	0	0	0	2	1
ENSMUSG00000100580	0	0	0	0	0	0
ENSMUSG00000087693	0	0	0	0	0	0
ENSMUSG00000100588	0	0	0	0	0	0
ENSMUSG00000100589	0	0	0	0	0	0
ENSMUSG00000112091	0	0	0	3	0	0
ENSMUSG00000085696	2	3	3	2	6	16
ENSMUSG00000085693	0	0	0	0	0	0
ENSMUSG00000085690	0	0	0	0	0	0
ENSMUSG00000085691	0	0	0	0	0	0
ENSMUSG00000102690	0	0	0	2	0	0
ENSMUSG00000102692	425	248	478	27	44	46
ENSMUSG00000102693	0	2	0	0	0	0
ENSMUSG00000102694	2	0	2	0	2	0
ENSMUSG00000102696	0	0	0	0	0	0
ENSMUSG00000102699	0	0	3	0	0	0
ENSMUSG00000033634	3	0	0	0	2	0
ENSMUSG00000079639	0	0	0	0	0	0
ENSMUSG00000079638	0	0	0	0	0	0
ENSMUSG00000079633	0	0	0	0	0	0
ENSMUSG00000079630	0	0	0	0	0	0
ENSMUSG00000079637	0	3	0	0	0	0
ENSMUSG00000079636	0	0	0	0	0	0
ENSMUSG00000079635	0	0	0	0	0	0
ENSMUSG00000065582	0	1	0	0	0	0
ENSMUSG00000065341	0	2	0	0	0	0
ENSMUSG00000065585	0	0	0	0	0	0
ENSMUSG00000065586	0	0	0	0	0	0
ENSMUSG00000068758	84	72	92	29	29	53
ENSMUSG00000085348	0	0	0	0	0	0
ENSMUSG00000088521	0	0	0	0	0	0
ENSMUSG00000088526	0	0	0	0	0	0
ENSMUSG00000088525	0	0	0	0	0	0
ENSMUSG00000088528	0	0	0	0	0	0
ENSMUSG00000074003	2	3	0	4	3	2
ENSMUSG00000074006	13	5	7	0	9	5
ENSMUSG00000074004	0	0	0	2	0	6
ENSMUSG00000077851	0	0	0	0	0	0
ENSMUSG00000065320	0	0	0	0	2	0
ENSMUSG00000065321	0	0	0	0	0	0
ENSMUSG00000034706	4	0	4	3	0	0
ENSMUSG00000022132	16	6	8	0	3	5
ENSMUSG00000024485	4	0	0	0	0	0
ENSMUSG00000113363	0	0	0	0	0	0
ENSMUSG00000089364	0	0	0	0	0	0
ENSMUSG00000103775	2	0	1	0	0	0
ENSMUSG00000103773	0	0	0	0	0	0
ENSMUSG00000097268	2	0	0	0	5	0
ENSMUSG00000097266	0	0	0	0	0	0
ENSMUSG00000097260	0	0	2	0	0	0
ENSMUSG00000097262	0	0	0	0	0	0
ENSMUSG00000097104	0	0	0	0	0	0

ENSMUSG00000029168	3	5	0	3	4	2
ENSMUSG00000097106	2	0	0	4	9	0
ENSMUSG00000089696	0	0	0	0	0	0
ENSMUSG00000089694	7	3	4	1	2	3
ENSMUSG00000109518	0	0	0	0	0	0
ENSMUSG00000109519	0	0	0	0	0	0
ENSMUSG00000109513	0	0	0	0	0	0
ENSMUSG00000109516	0	0	0	1	0	0
ENSMUSG00000062314	0	0	0	0	0	0
ENSMUSG00000114512	0	0	0	0	0	0
ENSMUSG00000062310	12	10	25	10	17	14
ENSMUSG00000100878	0	0	0	0	0	0
ENSMUSG00000114517	0	0	0	0	0	0
ENSMUSG00000114518	0	0	0	0	0	0
ENSMUSG00000114519	0	0	0	0	0	0
ENSMUSG00000100874	0	0	0	0	0	0
ENSMUSG00000100875	0	0	0	0	0	0
ENSMUSG00000100872	5	0	0	0	0	0
ENSMUSG00000100873	0	0	0	0	0	0
ENSMUSG00000100870	0	0	0	0	0	0
ENSMUSG00000058435	0	0	0	0	0	0
ENSMUSG00000020562	0	0	0	0	0	0
ENSMUSG00000108649	2	2	0	0	0	0
ENSMUSG00000091415	2	0	3	6	0	5
ENSMUSG00000108641	0	0	0	0	0	0
ENSMUSG00000112823	45	50	58	49	41	65
ENSMUSG00000050645	0	0	0	0	0	0
ENSMUSG00000112821	0	0	0	0	0	0
ENSMUSG00000112826	0	0	0	0	0	0
ENSMUSG00000112827	0	1	6	4	2	1
ENSMUSG00000108647	0	0	0	0	0	0
ENSMUSG00000108646	0	0	0	0	0	0
ENSMUSG00000081514	0	0	0	0	0	0
ENSMUSG00000105343	0	0	0	0	0	0
ENSMUSG00000054727	0	0	0	0	0	0
ENSMUSG00000105346	0	0	0	0	0	0
ENSMUSG00000067360	0	0	0	0	0	0
ENSMUSG00000105344	10	21	8	5	4	1
ENSMUSG00000096605	0	0	0	0	0	0
ENSMUSG00000023914	0	0	0	0	0	0
ENSMUSG00000096607	0	0	0	0	0	0
ENSMUSG00000096606	37	109	56	51	53	91
ENSMUSG00000023911	12	5	21	9	8	8
ENSMUSG00000023918	28	20	18	43	18	34
ENSMUSG00000050394	75	44	49	27	39	26
ENSMUSG00000091950	3	1	1	4	0	5
ENSMUSG00000091956	6	5	0	10	17	0
ENSMUSG00000064928	0	0	0	0	0	0
ENSMUSG00000105967	1	0	1	0	0	0
ENSMUSG00000105964	0	0	0	0	0	0
ENSMUSG00000105963	0	0	0	0	0	0
ENSMUSG00000105961	0	0	0	0	0	0
ENSMUSG00000105968	0	0	0	0	0	0
ENSMUSG00000097202	0	0	0	0	0	0
ENSMUSG00000048709	0	0	3	0	0	0
ENSMUSG00000101044	0	0	0	0	0	0
ENSMUSG00000101045	0	0	0	0	0	0
ENSMUSG00000101046	0	0	0	0	0	0

ENSMUSG00000101040	0	0	0	0	0	0
ENSMUSG00000101041	0	0	0	0	0	0
ENSMUSG00000101042	0	0	0	0	0	0
ENSMUSG00000073514	7	0	2	0	0	5
ENSMUSG00000056946	0	0	0	0	0	0
ENSMUSG00000110039	0	0	0	0	0	0
ENSMUSG00000021214	0	0	0	0	2	0
ENSMUSG00000021216	0	0	0	0	0	0
ENSMUSG00000021217	376	356	380	145	191	183
ENSMUSG00000021210	0	0	0	0	0	0
ENSMUSG00000026247	0	0	0	0	0	0
ENSMUSG00000024125	0	0	0	0	0	0
ENSMUSG00000021213	26	42	54	39	11	44
ENSMUSG00000111033	0	0	0	0	0	0
ENSMUSG00000111035	0	0	0	0	0	0
ENSMUSG00000113883	4	0	0	0	0	0
ENSMUSG00000110033	0	0	0	0	0	0
ENSMUSG00000113881	2	0	4	0	0	0
ENSMUSG00000113880	3	8	1	3	2	4
ENSMUSG00000113887	0	0	0	0	0	0
ENSMUSG00000110032	4	0	0	0	0	0
ENSMUSG00000113888	0	0	0	0	0	0
ENSMUSG00000024124	5	0	4	0	0	0
ENSMUSG00000089090	0	0	0	0	0	0
ENSMUSG00000063975	0	1	0	0	0	0
ENSMUSG00000089091	0	0	0	0	0	0
ENSMUSG00000110037	0	0	0	0	0	0
ENSMUSG00000064842	0	0	0	0	0	0
ENSMUSG00000064840	0	0	0	0	0	0
ENSMUSG00000110036	0	0	0	0	0	0
ENSMUSG00000091375	0	0	4	0	0	0
ENSMUSG00000091376	0	0	0	0	0	0
ENSMUSG00000084061	0	0	0	0	0	0
ENSMUSG00000110034	0	0	0	0	0	0
ENSMUSG00000089096	6	1	1	5	3	2
ENSMUSG00000081122	0	0	0	0	0	0
ENSMUSG00000081124	0	0	0	0	0	0
ENSMUSG00000081127	0	0	0	0	0	0
ENSMUSG00000081129	0	0	0	0	0	0
ENSMUSG00000095220	0	0	0	0	0	0
ENSMUSG00000105210	0	0	0	0	0	0
ENSMUSG00000035274	79	51	83	8	2	18
ENSMUSG00000090773	0	0	0	0	0	0
ENSMUSG00000090772	0	0	0	0	0	0
ENSMUSG00000081698	0	0	0	0	0	0
ENSMUSG00000081699	0	0	2	0	0	0
ENSMUSG00000090777	0	0	0	0	0	0
ENSMUSG00000090774	0	0	0	0	0	0
ENSMUSG00000081693	0	0	0	0	0	0
ENSMUSG00000081690	5	2	2	3	0	1
ENSMUSG00000081696	0	0	0	0	0	0
ENSMUSG00000081697	0	0	0	0	0	0
ENSMUSG00000081694	0	0	0	0	0	0
ENSMUSG00000058399	0	0	0	0	0	0
ENSMUSG00000058398	0	0	0	0	0	0
ENSMUSG00000094471	0	0	0	0	0	0
ENSMUSG00000094472	0	0	0	0	0	0
ENSMUSG00000094473	0	0	0	0	0	0

ENSMUSG00000094474	0	0	0	0	0	0
ENSMUSG00000094476	0	0	0	0	0	0
ENSMUSG00000094477	0	1	0	0	0	1
ENSMUSG00000056055	21	22	25	21	16	23
ENSMUSG00000086478	0	0	0	0	0	0
ENSMUSG00000086474	0	0	0	0	0	0
ENSMUSG00000088721	0	0	0	0	0	0
ENSMUSG00000105215	0	0	0	0	0	0
ENSMUSG00000046755	0	0	0	0	0	0
ENSMUSG00000044976	17	14	20	18	14	57
ENSMUSG00000052537	0	0	0	0	0	0
ENSMUSG00000077001	2	1	1	0	0	0
ENSMUSG00000105214	0	0	0	0	0	0
ENSMUSG00000055629	0	0	0	6	2	0
ENSMUSG00000055621	0	0	0	0	0	0
ENSMUSG00000094294	0	0	0	0	0	0
ENSMUSG00000094295	0	1	0	0	0	0
ENSMUSG00000094296	0	0	0	0	0	0
ENSMUSG00000094297	0	0	0	2	0	0
ENSMUSG00000094290	0	0	0	0	0	0
ENSMUSG00000094291	0	0	0	0	0	0
ENSMUSG00000027801	0	0	0	0	0	0
ENSMUSG00000094293	0	0	0	0	0	0
ENSMUSG00000093411	0	0	0	0	0	0
ENSMUSG00000094298	0	0	0	0	1	0
ENSMUSG00000028351	6	6	8	6	0	16
ENSMUSG00000028356	0	0	0	0	0	0
ENSMUSG00000028357	0	0	0	0	0	0
ENSMUSG00000097929	0	0	0	0	0	0
ENSMUSG00000028358	73	50	93	26	31	32
ENSMUSG00000097924	8	14	0	3	7	14
ENSMUSG00000097921	12	16	7	18	14	7
ENSMUSG00000067555	0	0	0	0	2	0
ENSMUSG00000016239	107	114	99	102	85	81
ENSMUSG00000105993	0	0	0	3	0	0
ENSMUSG00000055088	0	1	0	1	2	0
ENSMUSG00000105996	0	0	0	0	0	0
ENSMUSG00000049353	0	6	5	0	2	4
ENSMUSG00000049350	0	0	0	0	0	0
ENSMUSG00000105994	0	0	0	0	0	0
ENSMUSG00000029409	0	0	0	0	0	0
ENSMUSG00000109490	0	0	0	0	0	0
ENSMUSG00000113308	0	0	0	0	0	0
ENSMUSG00000113309	0	0	0	0	0	0
ENSMUSG00000106083	0	0	0	0	0	0
ENSMUSG00000106085	0	0	0	2	0	0
ENSMUSG00000106084	0	3	0	0	0	0
ENSMUSG00000113300	0	0	0	0	0	0
ENSMUSG00000106088	0	0	0	0	0	0
ENSMUSG00000113302	0	0	0	0	0	0
ENSMUSG00000113303	0	2	4	0	0	0
ENSMUSG00000113304	0	2	0	0	0	0
ENSMUSG00000113305	0	0	0	0	0	0
ENSMUSG00000113306	0	0	0	0	0	0
ENSMUSG00000054976	0	0	0	2	0	0
ENSMUSG00000035420	0	0	0	0	0	0
ENSMUSG00000071769	0	0	0	0	0	0
ENSMUSG00000071763	0	0	0	0	0	0

ENSMUSG00000071764	0	0	0	0	0	0
ENSMUSG00000071767	0	0	0	0	0	0
ENSMUSG00000071766	0	0	0	0	0	0
ENSMUSG00000109251	10	5	9	2	0	5
ENSMUSG00000109250	0	0	0	0	0	0
ENSMUSG00000109254	0	0	0	0	0	0
ENSMUSG00000051251	0	0	0	0	0	0
ENSMUSG00000109258	0	0	0	0	0	0
ENSMUSG00000106551	3	2	2	3	8	4
ENSMUSG00000106557	27	25	17	24	29	22
ENSMUSG00000106554	0	0	0	0	0	3
ENSMUSG00000092191	0	0	0	0	0	0
ENSMUSG00000092193	0	0	0	0	0	0
ENSMUSG00000092195	3	0	0	0	0	0
ENSMUSG00000092197	0	0	0	0	0	0
ENSMUSG00000092196	0	0	0	0	0	0
ENSMUSG00000092199	0	0	0	0	0	0
ENSMUSG00000088691	0	0	0	0	0	0
ENSMUSG00000105316	2	0	0	0	0	0
ENSMUSG00000105159	0	0	0	0	0	0
ENSMUSG00000025188	193	117	191	57	83	97
ENSMUSG00000025189	0	0	4	0	3	0
ENSMUSG00000082578	0	0	0	0	0	0
ENSMUSG00000082579	1	1	1	3	2	1
ENSMUSG00000114653	3	1	5	3	1	0
ENSMUSG00000114652	5	0	0	2	0	0
ENSMUSG00000114651	0	0	0	0	0	0
ENSMUSG00000082573	0	0	0	0	0	0
ENSMUSG00000105317	0	0	0	0	0	0
ENSMUSG00000082577	1	0	0	0	0	0
ENSMUSG00000082574	0	0	0	0	0	0
ENSMUSG00000114658	0	0	0	0	0	0
ENSMUSG00000110147	0	0	0	2	0	0
ENSMUSG00000110145	0	0	0	0	0	0
ENSMUSG00000110143	0	0	0	0	0	0
ENSMUSG00000110140	0	0	0	0	0	0
ENSMUSG00000110149	0	0	0	0	0	0
ENSMUSG00000083229	0	0	0	0	0	0
ENSMUSG00000083228	0	0	0	0	0	0
ENSMUSG00000083221	0	0	0	0	0	0
ENSMUSG00000083223	0	0	2	0	0	3
ENSMUSG00000083222	0	0	2	0	0	0
ENSMUSG00000083225	0	0	0	0	0	0
ENSMUSG00000083224	0	0	0	0	0	0
ENSMUSG00000083227	0	0	0	0	0	0
ENSMUSG00000083226	0	2	0	2	0	0
ENSMUSG00000070498	3	0	5	0	0	5
ENSMUSG00000025231	168	309	319	93	142	195
ENSMUSG00000070495	14	3	17	6	24	14
ENSMUSG00000099178	0	0	0	0	0	0
ENSMUSG00000070141	1	1	0	0	0	0
ENSMUSG00000035896	5	8	5	6	6	10
ENSMUSG00000099173	0	0	0	0	0	0
ENSMUSG00000099170	0	0	3	4	0	0
ENSMUSG00000099176	0	0	0	0	0	0
ENSMUSG00000101673	0	0	0	0	0	0
ENSMUSG00000101672	0	0	0	0	0	0
ENSMUSG00000035057	0	0	0	0	0	0

ENSMUSG00000105313	0	0	0	0	0	0
ENSMUSG00000075512	0	0	0	0	0	0
ENSMUSG00000075511	25	30	30	23	13	22
ENSMUSG00000075517	0	0	0	0	0	0
ENSMUSG00000101676	0	0	0	0	0	0
ENSMUSG00000000730	4	10	0	11	2	15
ENSMUSG00000000731	0	0	0	0	2	0
ENSMUSG00000006777	0	0	0	0	0	0
ENSMUSG00000085107	0	0	0	0	0	0
ENSMUSG00000085104	0	0	0	0	0	0
ENSMUSG00000085817	0	0	0	0	0	0
ENSMUSG00000085101	0	0	0	0	0	0
ENSMUSG00000085108	0	0	0	0	0	0
ENSMUSG00000026765	0	0	3	0	0	5
ENSMUSG00000026764	150	146	243	93	100	123
ENSMUSG00000079342	0	0	0	0	0	0
ENSMUSG00000079346	0	0	0	0	0	0
ENSMUSG00000079349	0	0	0	0	0	0
ENSMUSG00000020297	5	0	9	2	6	0
ENSMUSG00000045217	0	0	0	0	0	0
ENSMUSG00000045215	54	41	58	26	18	46
ENSMUSG00000079966	0	0	0	0	0	0
ENSMUSG00000079963	0	0	0	0	0	0
ENSMUSG00000019817	152	149	191	72	107	91
ENSMUSG00000007827	469	424	419	321	335	468
ENSMUSG00000086838	0	0	0	0	0	0
ENSMUSG00000032915	31	45	55	19	45	28
ENSMUSG00000089905	2	0	0	0	0	0
ENSMUSG00000048562	0	0	0	0	0	0
ENSMUSG00000107935	0	0	0	0	0	0
ENSMUSG00000069998	0	0	0	0	0	0
ENSMUSG00000095128	0	0	0	0	0	0
ENSMUSG00000031758	127	149	175	95	111	140
ENSMUSG00000107933	0	0	0	0	0	0
ENSMUSG00000031756	47	48	25	31	27	35
ENSMUSG00000031757	0	0	0	0	0	0
ENSMUSG00000061584	0	0	0	0	0	0
ENSMUSG00000004842	0	5	0	0	0	0
ENSMUSG00000102889	0	0	0	0	0	0
ENSMUSG00000102885	0	0	0	0	0	0
ENSMUSG00000102884	0	0	0	0	0	0
ENSMUSG00000102887	0	0	0	0	0	0
ENSMUSG00000102886	2	0	0	0	5	4
ENSMUSG00000102880	3	3	3	0	0	3
ENSMUSG00000102883	0	0	0	0	0	0
ENSMUSG00000101282	0	0	0	0	0	0
ENSMUSG00000034987	53	49	53	37	58	55
ENSMUSG00000101280	0	0	0	0	0	0
ENSMUSG00000101281	2	0	0	0	0	2
ENSMUSG00000101286	0	0	0	0	0	0
ENSMUSG00000101287	0	0	0	0	0	0
ENSMUSG00000101284	0	0	0	0	0	0
ENSMUSG00000101285	0	0	0	0	0	0
ENSMUSG00000101288	0	0	0	0	0	3
ENSMUSG00000101289	0	0	0	0	0	0
ENSMUSG00000098861	1	0	0	0	0	0
ENSMUSG00000085951	0	0	0	0	0	0
ENSMUSG00000085950	0	0	0	0	0	0

ENSMUSG00000085953	3	0	0	0	0	0
ENSMUSG00000085952	0	0	0	0	0	0
ENSMUSG00000030406	17	20	31	6	9	19
ENSMUSG00000082635	0	0	0	0	0	0
ENSMUSG00000030402	0	3	5	0	3	0
ENSMUSG00000082637	0	0	0	0	0	0
ENSMUSG00000082636	0	0	0	0	0	0
ENSMUSG00000082639	0	0	0	0	0	0
ENSMUSG00000082638	0	0	0	0	0	0
ENSMUSG00000088296	0	0	0	0	0	0
ENSMUSG00000088291	0	0	0	0	0	0
ENSMUSG00000040632	0	0	0	0	0	0
ENSMUSG00000060303	0	0	0	0	2	0
ENSMUSG00000037759	23	17	9	12	4	4
ENSMUSG00000107938	0	0	0	0	0	0
ENSMUSG00000100628	0	2	0	0	0	0
ENSMUSG00000100627	4	0	0	0	0	3
ENSMUSG00000100626	7	5	4	3	4	6
ENSMUSG00000100625	0	2	0	0	0	0
ENSMUSG00000100624	0	0	0	0	0	0
ENSMUSG00000094654	0	0	0	0	0	0
ENSMUSG00000086788	0	0	0	0	0	2
ENSMUSG00000093908	25	30	39	21	28	31
ENSMUSG00000050625	0	2	0	0	0	0
ENSMUSG00000059939	202	175	213	119	131	197
ENSMUSG00000103294	0	0	3	0	0	0
ENSMUSG00000059934	0	0	0	0	0	0
ENSMUSG00000093607	0	0	0	0	0	0
ENSMUSG00000084776	0	0	0	0	0	0
ENSMUSG00000084774	0	0	0	0	0	0
ENSMUSG00000084775	6	7	0	0	4	6
ENSMUSG00000084772	0	0	0	0	0	0
ENSMUSG00000084773	0	5	4	0	0	0
ENSMUSG00000050623	5	11	8	11	11	15
ENSMUSG00000086785	9	5	5	3	2	0
ENSMUSG00000032131	0	12	10	2	0	2
ENSMUSG00000022456	22	30	42	8	4	22
ENSMUSG00000022455	0	0	0	0	0	0
ENSMUSG00000022454	0	0	0	0	0	0
ENSMUSG00000095282	0	0	0	0	0	0
ENSMUSG00000102715	0	0	0	0	0	0
ENSMUSG00000102716	0	0	0	0	0	0
ENSMUSG00000102710	0	0	0	0	0	0
ENSMUSG00000102712	2	2	1	6	2	6
ENSMUSG00000024857	0	0	0	0	0	0
ENSMUSG00000102718	0	2	0	0	0	0
ENSMUSG00000012350	0	2	0	0	0	0
ENSMUSG00000060878	7	7	10	3	6	6
ENSMUSG00000062434	0	0	0	0	0	0
ENSMUSG00000062433	0	0	0	0	0	0
ENSMUSG00000062432	10	7	22	26	3	9
ENSMUSG00000113038	0	0	0	0	2	0
ENSMUSG00000038259	2	0	0	0	0	0
ENSMUSG00000021439	0	0	0	0	0	0
ENSMUSG00000094904	1	1	1	1	1	1
ENSMUSG00000094905	0	0	0	0	0	0
ENSMUSG00000094901	0	0	0	0	0	0
ENSMUSG00000094903	0	0	0	0	0	0

ENSMUSG00000095283	0	0	0	0	0	0
ENSMUSG00000038253	8	3	10	8	12	26
ENSMUSG00000029646	0	0	0	0	0	0
ENSMUSG00000029644	0	0	0	0	0	0
ENSMUSG00000036931	14	10	51	19	16	22
ENSMUSG00000036938	0	0	0	0	0	0
ENSMUSG00000034774	5	5	0	0	0	0
ENSMUSG00000113146	2	0	0	1	0	0
ENSMUSG00000113144	0	0	0	0	0	0
ENSMUSG00000113145	0	0	0	0	0	0
ENSMUSG00000113034	0	0	0	0	0	0
ENSMUSG00000001510	0	0	2	0	0	0
ENSMUSG00000113035	6	4	10	0	4	4
ENSMUSG00000113148	0	0	0	0	0	0
ENSMUSG00000034482	0	0	3	0	0	0
ENSMUSG00000051095	4	3	3	2	2	5
ENSMUSG00000097291	0	0	2	0	1	3
ENSMUSG00000106718	0	0	0	0	0	0
ENSMUSG00000106710	7	15	8	3	6	10
ENSMUSG00000106713	0	0	2	0	0	0
ENSMUSG00000106714	10	12	11	7	9	9
ENSMUSG00000038094	4	0	3	0	0	3
ENSMUSG00000104712	0	0	0	0	0	0
ENSMUSG00000104711	0	0	0	0	0	0
ENSMUSG00000104710	0	0	6	0	0	4
ENSMUSG00000104717	0	0	0	0	0	0
ENSMUSG00000104716	0	0	0	0	0	0
ENSMUSG00000104715	0	0	0	0	0	0
ENSMUSG00000104714	0	0	0	0	0	0
ENSMUSG00000097299	0	0	0	0	0	0
ENSMUSG00000104718	0	0	0	0	0	0
ENSMUSG00000020897	42	32	25	0	15	31
ENSMUSG00000111788	0	0	0	0	0	0
ENSMUSG00000111782	0	2	0	0	0	2
ENSMUSG00000109720	0	0	0	0	0	0
ENSMUSG00000111780	0	0	0	0	0	0
ENSMUSG00000111781	0	0	0	0	0	0
ENSMUSG00000111786	0	0	0	0	0	0
ENSMUSG00000111787	0	1	0	0	0	0
ENSMUSG00000109726	0	0	0	0	0	0
ENSMUSG00000114495	0	0	0	0	0	0
ENSMUSG00000114494	0	0	0	0	0	0
ENSMUSG00000114497	0	0	0	0	0	0
ENSMUSG00000114491	0	0	0	0	0	0
ENSMUSG00000114490	0	0	0	0	0	0
ENSMUSG00000114499	0	0	0	0	0	0
ENSMUSG00000095193	27	27	15	12	19	18
ENSMUSG00000097767	0	0	2	0	0	4
ENSMUSG00000095883	0	0	0	0	0	0
ENSMUSG00000095880	0	0	0	0	0	0
ENSMUSG00000104050	0	0	0	0	0	0
ENSMUSG00000109796	0	0	0	0	0	0
ENSMUSG00000101023	0	0	0	1	0	0
ENSMUSG00000025431	0	0	0	0	0	0
ENSMUSG00000104054	0	0	0	0	0	0
ENSMUSG00000104055	0	0	0	0	0	0
ENSMUSG00000050058	0	0	0	0	0	0
ENSMUSG00000044320	0	0	17	0	0	0

ENSMUSG00000044322	0	0	0	0	0	0
ENSMUSG00000110550	0	2	0	0	0	0
ENSMUSG00000110552	0	0	0	0	0	0
ENSMUSG00000110554	0	0	0	2	0	0
ENSMUSG00000110557	0	0	0	0	0	2
ENSMUSG00000110559	2	0	0	0	0	0
ENSMUSG00000059463	0	0	0	0	0	0
ENSMUSG00000060469	0	0	0	0	0	0
ENSMUSG00000050933	0	0	0	0	0	0
ENSMUSG00000026167	0	0	0	0	0	0
ENSMUSG00000026166	0	0	0	0	0	0
ENSMUSG00000061048	61	26	43	0	0	0
ENSMUSG00000096414	0	0	0	0	0	0
ENSMUSG00000096412	0	1	0	0	0	1
ENSMUSG00000096413	0	0	0	0	0	0
ENSMUSG00000090951	2	0	0	2	0	5
ENSMUSG00000080443	0	0	0	0	0	0
ENSMUSG00000013562	0	0	0	0	0	0
ENSMUSG00000045052	0	0	0	0	0	0
ENSMUSG00000045053	0	0	0	0	0	2
ENSMUSG00000113924	2	1	1	1	1	1
ENSMUSG00000113925	0	0	0	0	0	0
ENSMUSG00000113927	0	0	0	0	0	0
ENSMUSG00000099728	1	1	0	0	0	0
ENSMUSG00000095192	0	0	0	0	0	0
ENSMUSG00000095195	0	0	0	9	3	16
ENSMUSG00000095196	3	0	1	3	1	2
ENSMUSG00000111207	2	4	0	3	0	0
ENSMUSG00000111205	0	3	4	0	4	7
ENSMUSG00000111204	6	4	5	5	9	8
ENSMUSG00000111203	0	0	0	0	0	0
ENSMUSG00000111202	12	10	14	6	12	12
ENSMUSG00000111201	0	0	0	0	0	0
ENSMUSG00000045589	6	0	5	5	8	8
ENSMUSG00000077553	0	0	0	0	0	1
ENSMUSG00000081719	0	0	0	0	0	0
ENSMUSG00000081718	0	4	0	2	0	0
ENSMUSG00000081717	0	0	0	0	0	0
ENSMUSG00000099723	0	0	0	0	0	0
ENSMUSG00000081714	0	0	0	0	0	0
ENSMUSG00000081713	0	0	0	0	0	0
ENSMUSG00000081711	0	0	0	0	1	1
ENSMUSG00000077555	0	0	0	0	0	0
ENSMUSG00000102707	0	0	0	0	0	0
ENSMUSG00000028938	0	0	0	0	0	0
ENSMUSG00000086848	0	0	0	0	0	0
ENSMUSG00000086842	0	3	2	0	0	0
ENSMUSG00000018470	14	4	10	7	6	20
ENSMUSG00000086840	0	0	0	0	0	0
ENSMUSG00000086847	0	0	0	0	0	0
ENSMUSG00000086844	9	0	7	10	7	10
ENSMUSG00000086845	2	0	0	0	0	0
ENSMUSG00000052182	0	0	0	0	0	0
ENSMUSG00000052180	0	0	4	0	0	0
ENSMUSG00000052187	0	0	0	0	0	0
ENSMUSG00000090927	0	0	0	0	0	0
ENSMUSG00000090925	0	0	3	0	5	2
ENSMUSG00000048763	38	31	44	14	44	28

ENSMUSG00000048764	0	2	0	0	0	0
ENSMUSG00000048766	0	0	0	0	0	0
ENSMUSG00000077633	2	0	0	0	0	0
ENSMUSG00000103142	0	0	0	0	0	0
ENSMUSG00000093699	0	0	0	0	0	0
ENSMUSG00000042050	359	239	310	208	235	287
ENSMUSG00000093696	0	0	0	0	0	0
ENSMUSG00000093692	0	0	0	0	0	0
ENSMUSG00000101022	0	0	0	0	0	0
ENSMUSG00000109522	0	0	0	0	0	0
ENSMUSG00000099758	0	0	0	0	0	0
ENSMUSG00000099759	32	19	19	14	18	3
ENSMUSG00000099752	0	0	0	0	0	0
ENSMUSG00000049797	0	0	0	0	0	0
ENSMUSG00000099750	0	2	0	0	0	0
ENSMUSG00000099751	0	0	0	0	0	0
ENSMUSG00000099756	0	0	0	0	0	0
ENSMUSG00000108881	0	0	0	0	0	0
ENSMUSG00000099754	0	0	0	0	0	0
ENSMUSG00000099755	0	0	0	0	0	0
ENSMUSG00000108887	0	0	0	0	0	0
ENSMUSG00000109524	0	0	0	0	0	0
ENSMUSG00000108885	0	0	0	0	0	0
ENSMUSG00000046774	0	0	0	4	0	0
ENSMUSG00000108884	0	0	0	0	0	3
ENSMUSG00000100847	0	0	0	0	0	0
ENSMUSG00000100845	0	0	0	0	0	0
ENSMUSG00000049498	0	0	0	0	0	0
ENSMUSG00000086159	0	0	0	2	0	0
ENSMUSG00000100844	0	0	0	0	0	0
ENSMUSG00000086150	13	17	19	11	2	13
ENSMUSG00000049491	0	0	0	0	0	1
ENSMUSG00000086152	0	0	0	0	0	0
ENSMUSG00000049493	3	5	10	12	3	0
ENSMUSG00000086154	0	3	3	0	5	0
ENSMUSG00000100841	0	0	0	0	0	0
ENSMUSG00000049148	11	28	16	12	21	27
ENSMUSG00000049149	0	0	1	0	3	2
ENSMUSG00000098807	2	1	1	1	0	0
ENSMUSG00000076272	4	0	0	0	0	3
ENSMUSG00000087409	0	0	0	0	0	0
ENSMUSG00000076274	0	0	0	0	0	0
ENSMUSG00000082437	0	0	0	0	0	0
ENSMUSG00000082435	0	0	0	0	0	0
ENSMUSG00000077205	0	0	0	0	0	0
ENSMUSG00000082432	0	0	0	0	0	0
ENSMUSG00000087403	99	130	101	90	83	48
ENSMUSG00000083528	2	0	0	0	2	0
ENSMUSG00000083522	0	0	1	0	0	0
ENSMUSG00000045877	0	3	10	6	4	5
ENSMUSG00000083520	4	0	0	0	0	3
ENSMUSG00000083527	0	3	0	0	0	0
ENSMUSG00000083524	0	0	0	0	0	0
ENSMUSG00000045871	66	37	14	28	54	53
ENSMUSG00000004317	484	483	561	168	334	335
ENSMUSG00000111366	0	0	0	0	0	0
ENSMUSG00000112376	30	29	47	22	27	27
ENSMUSG00000070841	0	0	0	0	0	0

ENSMUSG00000070847	0	0	0	0	0	0
ENSMUSG00000070844	0	0	0	0	0	0
ENSMUSG00000071138	0	0	0	0	0	0
ENSMUSG00000040340	0	0	0	0	0	0
ENSMUSG00000021850	14	14	17	33	29	29
ENSMUSG00000102650	0	0	0	12	4	2
ENSMUSG00000084179	0	0	0	0	0	0
ENSMUSG00000083388	0	0	0	0	0	0
ENSMUSG00000083389	0	0	0	0	0	0
ENSMUSG00000083386	0	0	3	2	0	0
ENSMUSG00000083387	2	0	0	0	0	2
ENSMUSG00000083384	0	0	0	0	0	0
ENSMUSG00000083385	0	3	0	0	0	0
ENSMUSG00000083382	14	19	18	25	23	31
ENSMUSG00000084171	0	0	0	0	0	0
ENSMUSG00000084172	0	0	0	0	0	0
ENSMUSG00000084173	0	0	0	0	0	0
ENSMUSG00000091929	0	0	0	0	0	0
ENSMUSG00000025375	168	163	231	170	201	193
ENSMUSG00000112812	0	0	0	0	0	0
ENSMUSG00000080871	0	0	0	0	0	0
ENSMUSG00000080870	11	11	17	8	16	16
ENSMUSG00000080872	0	0	0	0	0	0
ENSMUSG00000080874	0	0	0	0	0	0
ENSMUSG00000080876	0	0	0	0	0	0
ENSMUSG00000108691	0	0	0	0	0	0
ENSMUSG00000100378	0	0	0	0	0	0
ENSMUSG00000108696	5	3	3	3	5	3
ENSMUSG00000112379	4	0	0	0	0	0
ENSMUSG00000040966	0	0	0	0	0	0
ENSMUSG00000040963	0	14	10	0	3	6
ENSMUSG00000001095	0	0	0	0	0	0
ENSMUSG00000040969	0	0	0	0	0	0
ENSMUSG00000112819	0	0	0	0	0	0
ENSMUSG00000075726	0	0	0	0	0	0
ENSMUSG00000044040	0	0	0	0	0	0
ENSMUSG00000044041	0	0	0	0	0	0
ENSMUSG00000044042	206	170	215	24	69	70
ENSMUSG00000070240	0	0	0	0	0	0
ENSMUSG00000090089	0	0	0	0	0	0
ENSMUSG00000070263	0	0	0	0	0	0
ENSMUSG00000105291	0	0	0	0	0	0
ENSMUSG00000099034	41	43	69	27	85	34
ENSMUSG00000099031	0	0	1	0	0	0
ENSMUSG00000032033	0	0	0	0	0	0
ENSMUSG00000070249	0	0	0	0	0	0
ENSMUSG00000030889	229	299	225	246	245	354
ENSMUSG00000086326	0	0	0	0	0	0
ENSMUSG00000099013	0	0	0	0	0	0
ENSMUSG00000034339	0	0	0	0	0	0
ENSMUSG00000000942	0	0	0	4	3	4
ENSMUSG00000065173	0	2	0	0	0	0
ENSMUSG00000099014	0	0	0	0	0	0
ENSMUSG00000085285	0	0	0	0	0	0
ENSMUSG00000105310	0	0	0	0	0	0
ENSMUSG00000067352	0	0	0	0	0	0
ENSMUSG00000110209	0	0	0	0	0	0
ENSMUSG00000062184	102	60	95	17	18	10

ENSMUSG00000062186	0	0	0	0	0	0
ENSMUSG00000062181	0	0	3	0	0	0
ENSMUSG00000081832	0	0	0	0	0	0
ENSMUSG00000081831	0	0	0	0	0	0
ENSMUSG00000081836	0	0	0	0	0	0
ENSMUSG00000081834	0	0	0	0	0	0
ENSMUSG00000081835	2	4	2	1	3	3
ENSMUSG00000073761	0	3	2	0	0	0
ENSMUSG00000073765	0	0	0	0	0	0
ENSMUSG00000073764	0	0	0	0	0	0
ENSMUSG00000073769	0	0	0	0	2	0
ENSMUSG00000073768	0	0	3	0	0	0
ENSMUSG00000063582	0	0	0	0	0	0
ENSMUSG00000063583	0	0	0	3	0	0
ENSMUSG00000098078	0	0	0	0	0	0
ENSMUSG00000098079	0	0	0	0	0	0
ENSMUSG00000098070	0	0	0	0	0	0
ENSMUSG00000098071	0	0	0	0	0	0
ENSMUSG00000098072	0	0	0	0	0	0
ENSMUSG00000098073	0	0	0	0	0	0
ENSMUSG00000098074	0	0	0	0	0	0
ENSMUSG00000098075	0	0	0	0	0	0
ENSMUSG00000098077	1	0	0	0	2	2
ENSMUSG00000110718	0	0	0	0	0	0
ENSMUSG00000087491	0	0	0	0	0	0
ENSMUSG00000102633	11	10	8	9	0	6
ENSMUSG00000110710	48	18	34	18	44	50
ENSMUSG00000066912	3	0	0	0	0	0
ENSMUSG00000110717	0	0	0	0	0	0
ENSMUSG00000084923	0	0	0	2	0	0
ENSMUSG00000084921	0	0	0	0	0	0
ENSMUSG00000084920	0	0	0	0	0	0
ENSMUSG00000084927	0	0	0	0	0	3
ENSMUSG00000084924	0	0	0	0	0	0
ENSMUSG00000043073	0	0	0	0	0	0
ENSMUSG00000091528	0	0	0	0	0	0
ENSMUSG00000086730	0	0	0	2	0	0
ENSMUSG00000091526	0	0	0	3	0	0
ENSMUSG00000071855	37	48	65	19	33	38
ENSMUSG00000043087	0	0	0	0	0	0
ENSMUSG00000043089	0	0	0	0	0	0
ENSMUSG00000043088	28	29	32	8	16	23
ENSMUSG00000030302	8	14	15	17	22	31
ENSMUSG00000030307	0	0	0	0	0	2
ENSMUSG00000038527	105	92	118	45	45	95
ENSMUSG00000100296	0	2	0	0	0	0
ENSMUSG00000100297	0	0	0	0	0	0
ENSMUSG00000100294	0	0	0	0	0	0
ENSMUSG00000100295	0	0	0	0	0	0
ENSMUSG00000087360	7	1	4	5	3	3
ENSMUSG00000100290	0	0	0	0	0	0
ENSMUSG00000087363	0	0	2	0	0	0
ENSMUSG00000087369	0	0	0	0	0	0
ENSMUSG00000100298	0	0	0	0	0	0
ENSMUSG00000100299	0	0	0	0	0	0
ENSMUSG00000104998	0	0	0	0	0	0
ENSMUSG00000101748	0	0	0	0	0	0
ENSMUSG00000101743	0	0	0	0	0	0

ENSMUSG00000101742	0	0	0	0	0	0
ENSMUSG00000101741	0	0	3	0	4	3
ENSMUSG00000101740	0	0	0	0	0	0
ENSMUSG00000101747	0	0	0	0	0	0
ENSMUSG00000104994	45	50	40	50	50	54
ENSMUSG00000101745	0	0	0	0	0	0
ENSMUSG00000101744	0	0	0	0	0	0
ENSMUSG00000037653	34	30	19	21	26	32
ENSMUSG00000093499	0	0	0	0	2	0
ENSMUSG00000066097	0	3	0	0	0	0
ENSMUSG00000033147	280	354	368	242	291	320
ENSMUSG00000008932	42	25	30	50	19	68
ENSMUSG00000096999	0	0	0	0	0	0
ENSMUSG00000088484	0	0	2	0	0	0
ENSMUSG00000088485	8	2	7	2	5	4
ENSMUSG00000088480	0	0	0	0	0	0
ENSMUSG00000088481	0	0	0	0	0	0
ENSMUSG00000088482	0	0	0	0	0	0
ENSMUSG00000088483	0	0	0	0	0	0
ENSMUSG00000059877	0	0	0	0	0	0
ENSMUSG00000059873	0	5	6	8	2	8
ENSMUSG00000103675	0	0	0	0	0	0
ENSMUSG00000036013	0	0	0	0	0	0
ENSMUSG00000103673	0	0	0	0	0	0
ENSMUSG00000103678	0	0	2	0	0	0
ENSMUSG00000089280	0	0	0	0	0	0
ENSMUSG00000089282	0	0	0	0	0	0
ENSMUSG00000089285	0	0	0	0	0	0
ENSMUSG00000107740	0	0	0	0	0	0
ENSMUSG00000089287	0	0	0	0	0	0
ENSMUSG00000089289	0	0	0	0	0	0
ENSMUSG00000089288	0	0	0	0	0	0
ENSMUSG00000107748	0	0	0	0	0	0
ENSMUSG00000065435	0	0	0	0	0	0
ENSMUSG00000094822	2	0	0	0	0	0
ENSMUSG00000065438	0	1	1	0	0	0
ENSMUSG00000021730	18	78	417	27	192	142
ENSMUSG00000094009	0	0	1	1	0	0
ENSMUSG00000094008	0	0	0	0	0	0
ENSMUSG00000094005	1	1	0	0	0	1
ENSMUSG00000094004	0	0	0	0	0	0
ENSMUSG00000094007	1	1	1	0	1	1
ENSMUSG00000094001	0	1	0	0	0	0
ENSMUSG00000094002	0	0	0	0	2	2
ENSMUSG00000089759	26	23	17	26	17	22
ENSMUSG00000089753	9	8	13	4	7	11
ENSMUSG00000089754	0	0	0	0	0	0
ENSMUSG00000102104	0	0	0	0	0	0
ENSMUSG00000102105	0	0	0	0	0	0
ENSMUSG00000102106	0	0	0	0	0	0
ENSMUSG00000112425	4	4	2	0	0	0
ENSMUSG00000102100	0	0	0	0	0	0
ENSMUSG00000112420	5	8	8	0	3	14
ENSMUSG00000102103	0	0	0	0	0	0
ENSMUSG00000102108	0	0	0	0	0	0
ENSMUSG00000102109	0	0	0	0	0	0
ENSMUSG00000112428	0	0	0	0	0	0
ENSMUSG00000114860	0	0	0	0	0	0

ENSMUSG00000114861	0	0	0	0	0	0
ENSMUSG00000114862	5	0	0	4	3	0
ENSMUSG00000114864	0	0	0	0	0	0
ENSMUSG00000114865	0	9	0	11	15	14
ENSMUSG00000114866	0	0	0	0	0	0
ENSMUSG00000114867	0	0	0	0	0	0
ENSMUSG00000114868	0	0	0	0	0	0
ENSMUSG00000114869	4	11	3	0	2	0
ENSMUSG00000111552	0	0	0	0	0	0
ENSMUSG00000110370	0	0	2	2	0	0
ENSMUSG00000110371	17	25	25	24	20	18
ENSMUSG00000110375	0	0	0	0	0	0
ENSMUSG00000110377	21	15	24	25	15	44
ENSMUSG00000109028	12	20	16	21	13	16
ENSMUSG00000109029	0	5	6	3	8	6
ENSMUSG00000109022	0	0	0	0	0	0
ENSMUSG00000109023	0	0	0	0	0	0
ENSMUSG00000109020	0	0	0	0	0	0
ENSMUSG00000109021	6	6	12	14	15	16
ENSMUSG00000109026	0	0	0	0	0	0
ENSMUSG00000109027	0	0	0	0	0	0
ENSMUSG00000109024	0	0	0	0	0	0
ENSMUSG00000109025	0	0	0	0	0	0
ENSMUSG00000029054	2	5	11	0	6	5
ENSMUSG00000029055	0	5	5	0	3	0
ENSMUSG00000106909	0	0	0	0	0	0
ENSMUSG00000106900	0	0	0	0	0	0
ENSMUSG00000106905	0	0	0	0	0	0
ENSMUSG00000106906	0	0	0	0	0	0
ENSMUSG00000038115	2	2	0	0	2	0
ENSMUSG00000079479	0	0	0	0	0	0
ENSMUSG00000088903	0	0	0	0	0	0
ENSMUSG00000088905	0	0	0	0	0	0
ENSMUSG00000088904	0	0	0	0	0	0
ENSMUSG00000088907	0	1	0	0	0	0
ENSMUSG00000038112	26	32	32	13	13	18
ENSMUSG00000088909	0	0	0	0	0	0
ENSMUSG00000088908	0	0	0	0	0	0
ENSMUSG00000069227	8	12	6	6	8	9
ENSMUSG00000021600	0	0	0	0	0	0
ENSMUSG00000051687	0	0	0	0	0	0
ENSMUSG00000051680	0	0	0	0	0	0
ENSMUSG00000111429	2	0	0	0	0	0
ENSMUSG00000111428	2	0	1	1	0	2
ENSMUSG00000109686	0	0	0	0	0	0
ENSMUSG00000111426	0	0	0	1	0	0
ENSMUSG00000109682	0	0	0	0	0	0
ENSMUSG00000111422	0	0	0	0	0	0
ENSMUSG00000111421	24	31	29	24	30	28
ENSMUSG00000111420	0	0	0	0	0	0
ENSMUSG00000079471	3	0	0	0	0	0
ENSMUSG00000096337	34	18	25	23	15	31
ENSMUSG00000114287	0	0	0	0	0	0
ENSMUSG00000114280	5	1	0	0	0	2
ENSMUSG00000096330	2	2	0	0	5	3
ENSMUSG00000096332	0	0	0	0	0	0
ENSMUSG00000096339	0	0	0	0	0	0
ENSMUSG00000096338	0	0	0	0	0	0

ENSMUSG00000108379	0	0	0	0	0	0
ENSMUSG00000103149	29	48	40	28	35	70
ENSMUSG00000113489	0	0	0	0	0	0
ENSMUSG00000108370	0	0	0	0	0	0
ENSMUSG00000034324	6	8	1	5	4	3
ENSMUSG00000108372	4	4	2	0	2	0
ENSMUSG00000108375	2	1	0	0	0	0
ENSMUSG00000113482	0	0	0	0	0	0
ENSMUSG00000113481	0	0	0	0	0	0
ENSMUSG00000113480	0	0	0	0	0	0
ENSMUSG00000109978	0	0	0	0	0	0
ENSMUSG00000109979	0	0	0	0	0	0
ENSMUSG00000093868	0	0	0	0	0	0
ENSMUSG00000093869	0	0	0	0	0	0
ENSMUSG00000109971	0	0	0	0	0	0
ENSMUSG00000109972	0	0	0	0	0	0
ENSMUSG00000026556	66	77	79	47	67	59
ENSMUSG00000093867	0	4	0	1	0	0
ENSMUSG00000093865	0	0	0	0	7	8
ENSMUSG00000114134	0	0	0	0	0	0
ENSMUSG00000114132	2	0	0	0	0	0
ENSMUSG00000114130	0	0	0	0	0	0
ENSMUSG00000066477	0	0	0	0	0	0
ENSMUSG00000104502	0	4	2	2	0	2
ENSMUSG00000108082	0	0	0	0	0	0
ENSMUSG00000104500	0	0	0	0	0	0
ENSMUSG00000047889	0	0	0	0	0	0
ENSMUSG00000108086	0	0	0	0	0	0
ENSMUSG00000108085	0	0	0	0	0	0
ENSMUSG00000108084	0	0	0	0	0	0
ENSMUSG00000108089	4	4	3	6	1	1
ENSMUSG00000108088	0	2	0	0	0	0
ENSMUSG00000091648	5	8	3	6	15	21
ENSMUSG00000069825	0	0	0	0	0	0
ENSMUSG00000067704	0	0	0	0	0	0
ENSMUSG00000067700	0	0	0	0	0	0
ENSMUSG00000067702	0	0	0	0	0	0
ENSMUSG00000061356	0	0	0	0	3	0
ENSMUSG00000044286	0	2	0	2	0	2
ENSMUSG00000044288	4	0	0	0	0	4
ENSMUSG00000096102	0	0	0	0	0	0
ENSMUSG00000111174	2	0	0	0	0	0
ENSMUSG00000112999	8	5	2	0	0	2
ENSMUSG00000112994	0	0	0	0	0	0
ENSMUSG00000112992	0	0	0	0	0	0
ENSMUSG00000112993	0	0	0	0	0	0
ENSMUSG00000112991	0	9	4	4	7	6
ENSMUSG00000109808	0	0	0	0	0	0
ENSMUSG00000109218	2	8	0	0	0	8
ENSMUSG00000097948	2	2	0	0	4	0
ENSMUSG00000048904	0	0	0	0	0	0
ENSMUSG00000045620	0	0	0	2	4	4
ENSMUSG00000098059	0	0	0	0	0	0
ENSMUSG00000090063	13	5	0	0	0	4
ENSMUSG00000090062	0	0	2	0	0	0
ENSMUSG00000090061	4	8	11	2	10	5
ENSMUSG00000095011	0	0	0	0	0	0
ENSMUSG00000095010	0	0	0	0	0	0

ENSMUSG00000095019	1	0	0	0	0	0
ENSMUSG00000098275	35	28	32	25	27	25
ENSMUSG00000098272	0	0	0	0	0	0
ENSMUSG00000098273	1	3	4	1	0	1
ENSMUSG00000098271	0	0	0	3	0	0
ENSMUSG00000067949	0	0	2	0	2	2
ENSMUSG00000096105	0	0	0	0	0	0
ENSMUSG00000096741	0	0	0	0	0	0
ENSMUSG00000047716	0	0	0	0	0	0
ENSMUSG00000053790	0	0	0	0	0	0
ENSMUSG00000094763	0	0	0	0	0	0
ENSMUSG00000094768	0	0	0	0	0	0
ENSMUSG00000111045	1	1	1	0	0	1
ENSMUSG00000101030	0	0	0	0	0	0
ENSMUSG00000052221	6	2	2	2	5	7
ENSMUSG00000093089	1	0	1	0	0	2
ENSMUSG00000093085	3	2	3	0	0	2
ENSMUSG00000055026	2	0	3	2	5	4
ENSMUSG00000093082	0	0	0	0	0	0
ENSMUSG00000093083	0	0	0	0	0	0
ENSMUSG00000026890	173	141	238	125	130	187
ENSMUSG00000090685	0	0	2	3	4	0
ENSMUSG00000090684	0	0	0	0	0	1
ENSMUSG00000112675	0	0	0	0	0	0
ENSMUSG00000081508	0	0	0	0	0	0
ENSMUSG00000081506	0	0	0	0	0	0
ENSMUSG00000081507	0	0	0	0	0	0
ENSMUSG00000081505	0	0	0	2	0	0
ENSMUSG00000081502	0	0	0	0	0	0
ENSMUSG00000081500	0	0	0	0	0	0
ENSMUSG00000090688	0	0	0	0	0	0
ENSMUSG00000077468	0	0	0	0	0	1
ENSMUSG00000092685	0	1	0	0	1	0
ENSMUSG00000099659	0	0	0	0	0	0
ENSMUSG00000099657	0	0	0	0	0	0
ENSMUSG00000099656	0	0	0	0	0	0
ENSMUSG00000077462	0	0	0	0	0	0
ENSMUSG00000099654	0	0	0	0	0	0
ENSMUSG00000077464	0	0	0	0	0	0
ENSMUSG00000099651	0	0	0	0	0	0
ENSMUSG00000099650	0	0	0	0	0	0
ENSMUSG00000101449	0	1	0	3	0	1
ENSMUSG00000049843	0	0	0	0	0	0
ENSMUSG00000049848	0	0	0	0	0	0
ENSMUSG00000048138	0	0	0	0	0	0
ENSMUSG00000086612	3	0	0	2	3	3
ENSMUSG00000030145	151	152	151	97	125	149
ENSMUSG00000030142	8	46	31	8	0	7
ENSMUSG00000030143	0	0	0	0	0	0
ENSMUSG00000030149	21	21	42	12	8	14
ENSMUSG00000092359	0	0	0	0	0	0
ENSMUSG00000092353	11	16	27	19	19	15
ENSMUSG00000092352	0	0	0	0	0	0
ENSMUSG00000092351	0	0	0	0	0	0
ENSMUSG00000092350	0	0	0	0	0	0
ENSMUSG00000092357	0	0	0	0	0	0
ENSMUSG00000087137	0	0	0	0	0	0
ENSMUSG00000051940	0	0	0	0	0	0

ENSMUSG00000096742	0	0	0	0	0	0
ENSMUSG00000043715	0	0	0	0	0	0
ENSMUSG00000082088	24	0	23	14	11	25
ENSMUSG00000082089	0	0	3	1	0	1
ENSMUSG00000082082	0	0	0	0	0	0
ENSMUSG00000082083	0	0	0	0	0	0
ENSMUSG00000082080	0	0	0	0	0	0
ENSMUSG00000082081	0	0	0	0	0	0
ENSMUSG00000082084	0	0	0	0	0	0
ENSMUSG00000082085	0	0	0	0	0	0
ENSMUSG00000076337	0	0	0	0	0	0
ENSMUSG00000076335	0	0	0	0	0	0
ENSMUSG00000041710	4	0	0	0	0	0
ENSMUSG00000076338	0	0	0	0	0	0
ENSMUSG00000093846	11	3	3	0	6	0
ENSMUSG00000032783	7	11	8	6	4	6
ENSMUSG00000037492	7	29	4	8	9	12
ENSMUSG00000113605	0	0	1	0	0	0
ENSMUSG00000093847	0	1	0	0	0	2
ENSMUSG00000072653	149	105	112	42	45	57
ENSMUSG00000087618	0	0	0	0	0	0
ENSMUSG00000087617	0	0	0	0	3	0
ENSMUSG00000087613	0	0	0	0	0	0
ENSMUSG00000075023	0	0	0	0	0	0
ENSMUSG00000075020	6	15	14	10	6	19
ENSMUSG00000097682	0	0	0	0	0	0
ENSMUSG00000075024	0	0	0	0	0	0
ENSMUSG00000110330	0	0	0	0	0	0
ENSMUSG00000107503	0	0	0	0	0	0
ENSMUSG00000107501	0	0	0	0	0	0
ENSMUSG00000107500	0	0	0	0	0	0
ENSMUSG00000107506	0	0	0	0	0	0
ENSMUSG00000101839	0	0	0	0	0	0
ENSMUSG00000101835	0	0	0	0	0	0
ENSMUSG00000101837	0	0	0	0	0	0
ENSMUSG00000101836	0	0	0	0	0	0
ENSMUSG00000101831	0	0	0	0	0	0
ENSMUSG00000068289	0	0	0	0	0	0
ENSMUSG00000101833	0	0	0	0	0	0
ENSMUSG00000085616	0	0	0	0	0	0
ENSMUSG00000074378	0	0	0	0	0	0
ENSMUSG00000085610	0	0	0	0	0	0
ENSMUSG00000074373	0	0	0	0	0	0
ENSMUSG00000074375	0	0	0	0	0	0
ENSMUSG00000074377	0	0	0	0	0	0
ENSMUSG00000085619	0	0	0	0	0	0
ENSMUSG00000032598	262	279	311	175	218	308
ENSMUSG00000090727	0	0	0	0	0	0
ENSMUSG00000075973	0	0	0	0	0	0
ENSMUSG00000081976	0	0	0	0	0	0
ENSMUSG00000081973	0	0	0	0	0	0
ENSMUSG00000081972	8	2	3	1	3	2
ENSMUSG00000081971	0	0	0	0	0	0
ENSMUSG00000078503	0	0	0	0	0	0
ENSMUSG00000024409	0	0	2	0	0	0
ENSMUSG00000078506	0	0	0	0	0	0
ENSMUSG00000078507	0	0	0	0	0	0
ENSMUSG00000078504	0	0	0	0	0	0

ENSMUSG00000078505	0	0	0	0	0	0
ENSMUSG00000024402	0	0	3	0	0	0
ENSMUSG00000078508	0	0	0	0	0	0
ENSMUSG00000024401	33	26	58	16	12	46
ENSMUSG00000024406	0	0	0	0	0	0
ENSMUSG00000089618	0	0	0	0	0	0
ENSMUSG00000089619	0	0	0	0	0	0
ENSMUSG00000089610	0	0	0	0	0	0
ENSMUSG00000089616	0	0	0	0	0	0
ENSMUSG00000089614	0	0	0	0	0	0
ENSMUSG00000089615	0	0	0	0	0	0
ENSMUSG00000084907	0	0	0	0	0	0
ENSMUSG00000084906	0	0	0	0	0	0
ENSMUSG00000032204	0	4	10	0	0	0
ENSMUSG00000102023	0	0	0	0	0	0
ENSMUSG00000102022	0	0	0	0	0	0
ENSMUSG00000102020	0	0	0	0	0	0
ENSMUSG00000102027	0	8	11	0	0	0
ENSMUSG00000102026	0	0	0	0	0	0
ENSMUSG00000102025	0	0	0	0	0	0
ENSMUSG00000102028	0	0	0	0	0	0
ENSMUSG00000062391	21	22	22	21	31	49
ENSMUSG00000062393	0	0	0	0	0	2
ENSMUSG00000103998	35	0	0	0	31	0
ENSMUSG00000103990	0	0	0	0	0	0
ENSMUSG00000103991	19	16	19	27	18	16
ENSMUSG00000103992	3	0	0	3	1	3
ENSMUSG00000103993	0	0	0	0	0	0
ENSMUSG00000103994	0	0	0	0	0	0
ENSMUSG00000103995	0	2	4	2	0	0
ENSMUSG00000103997	0	0	0	0	0	0
ENSMUSG00000073532	3	0	0	0	3	3
ENSMUSG00000083654	0	0	0	0	0	0
ENSMUSG00000073530	30	10	51	0	3	0
ENSMUSG00000110290	0	4	0	0	0	0
ENSMUSG00000077857	0	0	0	0	0	0
ENSMUSG00000077858	0	0	0	1	0	0
ENSMUSG00000084600	0	0	0	0	0	0
ENSMUSG00000069421	0	0	0	0	2	0
ENSMUSG00000083657	0	0	0	1	0	0
ENSMUSG00000042216	67	47	53	17	9	13
ENSMUSG00000089275	0	0	0	0	0	0
ENSMUSG00000094402	0	0	0	0	0	0
ENSMUSG00000057402	0	0	0	0	0	0
ENSMUSG00000084605	0	0	0	0	0	0
ENSMUSG00000042212	0	0	0	0	0	0
ENSMUSG00000096133	0	0	0	0	0	0
ENSMUSG00000096131	0	0	0	0	0	0
ENSMUSG00000025165	0	0	0	0	0	0
ENSMUSG00000096137	0	0	0	0	0	0
ENSMUSG00000096136	0	0	0	0	0	0
ENSMUSG00000096135	0	0	0	0	0	0
ENSMUSG00000096134	1	0	0	0	1	0
ENSMUSG00000096139	0	0	0	0	0	0
ENSMUSG00000094406	0	0	0	0	0	0
ENSMUSG00000078430	0	0	0	0	0	0
ENSMUSG00000034122	0	0	0	0	0	0
ENSMUSG00000032080	0	0	0	0	0	0

ENSMUSG00000103236	0	0	0	0	0	0
ENSMUSG00000024532	0	0	0	0	0	0
ENSMUSG00000023999	78	80	85	154	90	157
ENSMUSG00000096688	0	0	0	0	0	0
ENSMUSG00000096685	0	0	0	0	0	0
ENSMUSG00000096686	0	0	0	0	0	0
ENSMUSG00000096680	0	0	0	0	0	0
ENSMUSG00000096683	0	0	0	0	0	0
ENSMUSG00000096682	0	0	0	0	0	0
ENSMUSG00000066671	0	0	0	0	0	0
ENSMUSG00000041698	2	1	0	0	0	0
ENSMUSG00000024534	177	243	258	131	208	197
ENSMUSG00000071073	7	5	4	0	2	7
ENSMUSG00000091483	0	0	0	0	0	0
ENSMUSG00000091481	0	0	0	0	0	0
ENSMUSG00000091486	0	0	0	0	0	0
ENSMUSG00000020875	0	0	1	2	0	0
ENSMUSG00000020872	11	22	32	15	20	25
ENSMUSG00000020871	0	0	0	0	0	0
ENSMUSG00000105844	0	0	0	0	0	0
ENSMUSG00000104072	0	0	0	0	0	0
ENSMUSG00000104073	0	0	0	0	0	0
ENSMUSG00000104070	0	0	0	0	0	0
ENSMUSG00000104071	0	0	0	0	0	0
ENSMUSG00000104076	0	0	0	0	0	0
ENSMUSG00000060630	4	0	0	0	0	0
ENSMUSG00000104074	7	8	10	9	9	7
ENSMUSG00000104075	0	0	0	0	0	3
ENSMUSG00000099873	0	0	0	0	0	0
ENSMUSG00000015476	24	15	29	16	11	20
ENSMUSG00000048077	0	0	0	0	0	0
ENSMUSG00000056328	0	0	0	0	2	0
ENSMUSG00000056329	0	0	0	5	0	0
ENSMUSG00000100318	0	2	0	0	0	0
ENSMUSG00000114634	0	0	0	0	0	0
ENSMUSG00000100312	0	0	0	0	0	0
ENSMUSG00000100311	0	0	0	0	0	0
ENSMUSG00000061947	0	0	0	0	0	0
ENSMUSG00000100317	0	0	0	0	0	0
ENSMUSG00000100316	0	0	0	0	0	0
ENSMUSG00000100315	0	0	0	0	0	0
ENSMUSG00000100314	0	0	0	0	0	0
ENSMUSG00000110929	0	0	0	0	0	0
ENSMUSG00000102400	0	0	0	0	0	0
ENSMUSG00000110925	0	0	0	0	0	0
ENSMUSG00000110924	0	0	0	0	0	0
ENSMUSG00000110927	0	0	0	0	0	0
ENSMUSG00000110920	0	0	0	0	0	0
ENSMUSG00000110923	0	0	0	0	0	0
ENSMUSG00000110922	18	27	0	32	35	35
ENSMUSG00000102402	0	0	0	0	0	0
ENSMUSG00000114632	0	0	0	0	0	0
ENSMUSG00000102404	0	0	0	0	0	0
ENSMUSG00000112260	0	0	0	0	0	0
ENSMUSG00000026826	76	103	72	35	41	56
ENSMUSG00000026828	0	4	0	4	4	16
ENSMUSG00000088361	0	0	0	0	0	0
ENSMUSG00000088360	0	0	0	0	0	0

ENSMUSG00000088363	1	1	1	1	0	1
ENSMUSG00000088365	0	0	1	0	0	0
ENSMUSG00000088367	0	0	0	0	0	0
ENSMUSG00000088369	0	0	0	0	0	0
ENSMUSG00000077038	0	0	1	0	0	2
ENSMUSG00000036198	3	0	0	0	0	0
ENSMUSG00000095569	0	0	0	0	0	0
ENSMUSG00000036192	91	89	76	82	63	124
ENSMUSG00000095561	0	0	0	0	0	0
ENSMUSG00000101895	6	5	0	0	0	6
ENSMUSG00000095563	0	0	0	0	0	0
ENSMUSG00000112025	0	0	2	2	7	0
ENSMUSG00000112389	1	1	0	0	0	0
ENSMUSG00000094218	0	0	0	0	0	0
ENSMUSG00000094219	0	0	0	0	0	0
ENSMUSG00000027889	314	266	334	167	170	192
ENSMUSG00000094214	0	1	0	0	0	0
ENSMUSG00000027886	0	0	0	0	0	0
ENSMUSG00000093240	1	0	0	0	0	0
ENSMUSG00000112380	0	0	0	0	0	0
ENSMUSG00000112387	0	0	0	0	0	0
ENSMUSG00000112386	0	0	0	6	2	0
ENSMUSG00000112385	0	0	0	0	0	0
ENSMUSG00000027880	4	0	0	0	0	0
ENSMUSG00000112029	0	0	0	0	0	0
ENSMUSG00000036223	8	0	13	0	4	6
ENSMUSG00000113382	0	0	0	0	0	0
ENSMUSG00000113383	0	0	0	0	0	0
ENSMUSG00000113384	0	0	0	0	0	0
ENSMUSG00000028681	57	59	120	53	46	55
ENSMUSG00000113387	2	0	0	0	0	0
ENSMUSG00000113388	1	0	0	0	2	0
ENSMUSG00000108149	0	0	0	0	0	0
ENSMUSG00000102377	0	0	0	0	0	0
ENSMUSG00000018919	12	0	0	0	0	0
ENSMUSG00000087956	0	0	0	0	0	0
ENSMUSG00000017723	5	0	0	0	0	0
ENSMUSG00000018916	0	0	0	0	0	0
ENSMUSG00000018914	0	0	0	0	0	0
ENSMUSG00000020051	5	8	0	6	6	11
ENSMUSG00000106008	0	0	0	0	0	0
ENSMUSG00000009471	0	0	0	0	0	5
ENSMUSG00000106001	0	0	0	0	2	0
ENSMUSG00000106000	0	0	0	0	0	0
ENSMUSG00000106003	0	0	0	0	0	0
ENSMUSG00000106002	0	0	0	0	0	0
ENSMUSG00000106005	16	28	22	17	18	14
ENSMUSG00000106004	0	0	0	0	0	0
ENSMUSG00000106007	0	0	0	0	0	0
ENSMUSG00000028539	0	8	12	0	4	4
ENSMUSG00000028536	0	0	0	0	0	0
ENSMUSG00000028533	0	0	0	0	0	0
ENSMUSG00000092116	0	4	6	0	0	0
ENSMUSG00000039476	21	8	44	0	2	0
ENSMUSG00000112653	0	0	0	0	0	0
ENSMUSG00000112659	0	0	0	7	0	1
ENSMUSG00000112658	0	0	0	0	0	0
ENSMUSG00000025105	99	80	22	0	2	0

ENSMUSG00000113524	0	0	0	0	0	0
ENSMUSG00000113526	0	0	0	0	0	0
ENSMUSG00000113527	2	1	4	6	5	3
ENSMUSG00000038700	0	3	0	0	5	5
ENSMUSG00000113522	0	0	0	0	0	0
ENSMUSG00000113523	0	2	0	0	0	0
ENSMUSG00000038709	0	0	0	0	0	0
ENSMUSG00000085559	0	0	0	0	0	0
ENSMUSG00000064551	0	0	0	0	0	0
ENSMUSG00000082868	0	0	0	0	0	0
ENSMUSG00000082869	0	0	0	0	0	0
ENSMUSG00000082864	0	0	0	0	0	0
ENSMUSG00000082865	0	0	0	0	3	3
ENSMUSG00000082861	14	15	4	1	7	6
ENSMUSG00000082862	2	0	0	0	1	0
ENSMUSG00000082863	0	0	0	0	0	0
ENSMUSG00000108128	0	0	0	0	0	0
ENSMUSG00000108129	0	4	2	0	2	2
ENSMUSG00000086146	0	0	0	0	0	5
ENSMUSG00000070417	0	0	4	0	0	0
ENSMUSG00000070419	0	0	0	0	0	0
ENSMUSG00000108127	4	0	0	0	0	0
ENSMUSG00000105067	0	0	0	0	0	0
ENSMUSG00000105064	0	0	0	0	0	0
ENSMUSG00000105065	9	3	3	2	7	11
ENSMUSG00000109133	1	4	0	2	3	4
ENSMUSG00000111678	0	0	0	0	0	0
ENSMUSG00000026458	136	154	245	81	114	127
ENSMUSG00000111674	0	2	0	0	0	0
ENSMUSG00000111675	4	0	0	0	0	0
ENSMUSG00000111672	0	0	0	0	0	0
ENSMUSG00000088136	0	0	0	0	0	0
ENSMUSG00000111670	0	0	0	0	0	0
ENSMUSG00000026450	0	0	0	0	0	0
ENSMUSG00000079998	0	0	0	0	0	0
ENSMUSG00000036960	23	0	31	0	6	9
ENSMUSG00000088132	0	0	2	0	0	0
ENSMUSG00000104331	2	1	1	1	1	1
ENSMUSG00000104330	32	17	17	7	13	33
ENSMUSG00000104333	2	4	1	3	0	7
ENSMUSG00000104335	0	0	0	0	0	0
ENSMUSG00000104334	0	0	0	0	0	0
ENSMUSG00000104337	0	0	0	0	0	0
ENSMUSG00000104338	0	0	0	0	1	0
ENSMUSG00000036961	15	16	22	27	34	22
ENSMUSG00000079774	0	0	0	0	0	0
ENSMUSG00000083803	0	1	0	0	0	0
ENSMUSG00000045291	0	0	0	0	0	0
ENSMUSG00000083809	0	0	0	0	0	0
ENSMUSG00000044748	0	0	0	0	0	0
ENSMUSG00000044743	0	0	0	0	0	0
ENSMUSG00000098347	0	0	0	0	0	0
ENSMUSG00000113844	0	0	2	0	2	3
ENSMUSG00000098343	0	0	0	0	0	0
ENSMUSG00000090180	0	0	0	0	0	0
ENSMUSG00000027419	0	0	0	0	0	0
ENSMUSG00000092961	0	0	0	0	0	0
ENSMUSG00000092965	0	0	0	0	0	0

ENSMUSG00000027416	0	0	0	0	0	0
ENSMUSG00000113849	0	0	0	0	0	0
ENSMUSG00000100310	0	0	0	0	0	0
ENSMUSG00000088010	0	0	0	0	0	0
ENSMUSG00000046678	3	0	0	0	0	0
ENSMUSG00000095604	0	0	0	0	0	0
ENSMUSG00000095605	0	1	0	0	0	1
ENSMUSG00000095606	0	0	0	0	0	0
ENSMUSG00000095600	0	0	0	0	0	3
ENSMUSG00000095601	0	0	0	0	0	0
ENSMUSG00000095602	0	0	0	0	0	0
ENSMUSG00000090235	9	11	13	0	4	8
ENSMUSG00000095608	0	1	0	0	0	0
ENSMUSG00000088017	0	0	0	0	0	0
ENSMUSG00000080577	0	0	0	0	1	0
ENSMUSG00000113371	0	0	0	0	0	0
ENSMUSG00000092493	0	0	0	0	0	0
ENSMUSG00000092494	0	0	0	0	5	0
ENSMUSG00000052075	0	0	0	0	0	0
ENSMUSG00000098405	0	0	0	0	0	0
ENSMUSG00000028946	6	2	0	5	0	0
ENSMUSG00000098407	0	0	0	0	0	0
ENSMUSG00000098406	0	0	0	2	0	0
ENSMUSG00000098401	0	0	2	0	0	0
ENSMUSG00000098400	0	0	0	0	0	0
ENSMUSG00000028940	5	0	0	0	0	0
ENSMUSG00000098409	0	0	0	0	0	0
ENSMUSG00000113378	0	0	3	0	0	0
ENSMUSG00000093761	0	0	0	0	0	0
ENSMUSG00000093763	0	0	0	0	0	0
ENSMUSG00000093762	0	1	0	0	0	0
ENSMUSG00000093765	0	3	3	0	4	0
ENSMUSG00000093764	0	0	0	0	0	0
ENSMUSG00000093766	0	0	0	0	0	0
ENSMUSG00000110381	0	0	0	0	0	0
ENSMUSG00000047104	10	8	26	5	3	0
ENSMUSG00000031424	0	0	0	0	0	0
ENSMUSG00000047109	0	0	0	0	0	0
ENSMUSG00000031428	22	14	16	14	6	14
ENSMUSG00000037868	134	100	293	24	28	19
ENSMUSG00000049653	4	6	0	0	0	0
ENSMUSG00000112355	0	0	0	0	0	0
ENSMUSG00000105550	13	14	5	0	12	4
ENSMUSG00000112011	0	0	0	0	0	0
ENSMUSG00000095699	1	0	0	0	0	0
ENSMUSG00000095698	0	0	0	0	0	0
ENSMUSG00000101202	0	0	0	0	0	0
ENSMUSG00000101203	0	0	0	0	0	0
ENSMUSG00000101200	0	0	0	0	0	0
ENSMUSG00000110804	0	0	0	0	0	0
ENSMUSG00000101206	0	0	0	0	0	0
ENSMUSG00000101207	0	0	0	0	0	0
ENSMUSG00000101204	0	0	0	0	0	0
ENSMUSG00000095693	0	0	0	0	0	0
ENSMUSG00000087139	0	0	0	0	0	0
ENSMUSG00000082325	0	0	0	0	0	0
ENSMUSG00000082323	0	0	0	0	1	0
ENSMUSG00000048304	0	0	0	0	0	0

ENSMUSG00000099446	0	0	0	0	0	0
ENSMUSG00000099444	0	0	0	0	0	0
ENSMUSG00000099445	0	0	0	0	0	0
ENSMUSG00000099443	3	2	1	3	1	3
ENSMUSG00000050520	0	3	0	0	0	0
ENSMUSG00000037280	32	18	24	8	0	7
ENSMUSG00000084449	0	0	0	0	0	0
ENSMUSG00000083499	0	0	0	0	0	0
ENSMUSG00000084444	0	0	0	0	0	0
ENSMUSG00000084445	0	0	0	0	0	0
ENSMUSG00000084446	0	3	1	2	1	0
ENSMUSG00000083495	0	2	0	0	0	0
ENSMUSG00000083492	0	0	0	0	0	0
ENSMUSG00000083493	0	0	0	0	0	0
ENSMUSG00000084442	0	0	1	1	0	0
ENSMUSG00000083491	0	0	0	0	0	0
ENSMUSG00000100159	0	0	0	0	0	0
ENSMUSG00000110490	2	0	0	2	0	8
ENSMUSG00000100151	0	0	0	0	0	0
ENSMUSG00000030486	117	150	135	88	112	163
ENSMUSG00000101762	0	0	0	0	0	0
ENSMUSG00000030483	15	15	2	21	14	39
ENSMUSG00000100157	0	0	0	0	0	0
ENSMUSG00000034660	0	0	0	0	0	0
ENSMUSG00000037139	115	137	181	154	175	198
ENSMUSG00000083140	0	0	2	0	0	0
ENSMUSG00000065203	0	0	0	0	0	0
ENSMUSG00000083142	0	0	0	0	0	0
ENSMUSG00000083144	0	0	0	0	0	0
ENSMUSG00000037130	0	0	0	0	0	0
ENSMUSG00000087043	0	0	0	0	0	0
ENSMUSG00000065201	0	0	0	0	0	0
ENSMUSG00000102296	0	0	0	0	0	0
ENSMUSG00000087792	0	0	0	0	0	0
ENSMUSG00000041505	0	0	0	0	0	0
ENSMUSG00000087799	0	0	0	0	0	0
ENSMUSG00000057612	0	0	0	0	0	0
ENSMUSG00000106504	0	0	0	0	0	0
ENSMUSG00000085798	0	0	0	0	0	0
ENSMUSG00000085797	0	0	0	0	0	0
ENSMUSG00000106506	0	0	0	0	0	0
ENSMUSG00000085794	0	0	0	0	0	0
ENSMUSG00000106507	1	0	2	0	0	3
ENSMUSG00000102799	0	0	0	0	0	0
ENSMUSG00000064717	0	0	0	0	0	0
ENSMUSG00000064714	0	0	0	0	0	0
ENSMUSG00000102795	0	0	0	0	0	0
ENSMUSG00000064718	0	0	0	0	0	0
ENSMUSG00000102796	5	0	3	0	0	4
ENSMUSG00000102791	0	0	0	0	0	0
ENSMUSG00000102790	0	0	0	0	2	0
ENSMUSG00000102793	0	0	0	0	0	0
ENSMUSG00000102792	0	0	0	0	0	0
ENSMUSG00000087869	0	0	0	0	0	0
ENSMUSG00000031998	0	0	0	0	0	5
ENSMUSG00000087863	0	0	0	0	0	0
ENSMUSG00000087862	0	0	0	0	0	0
ENSMUSG00000031995	78	63	84	6	2	4

ENSMUSG00000087867	0	0	0	0	0	0
ENSMUSG00000031991	0	0	0	0	0	0
ENSMUSG00000066748	0	0	0	2	0	0
ENSMUSG00000005824	8	5	6	4	3	7
ENSMUSG00000074128	0	0	0	0	0	0
ENSMUSG00000085424	0	0	0	0	0	0
ENSMUSG00000085427	25	21	29	31	26	33
ENSMUSG00000085426	0	0	0	0	0	0
ENSMUSG00000085421	0	0	0	0	0	0
ENSMUSG00000085420	0	0	0	0	0	0
ENSMUSG00000074121	4	8	0	0	4	6
ENSMUSG00000074123	0	0	0	0	0	0
ENSMUSG00000085429	4	0	0	0	4	0
ENSMUSG00000085428	2	0	0	0	0	0
ENSMUSG00000074127	0	0	0	0	0	0
ENSMUSG00000107312	7	6	2	2	6	2
ENSMUSG00000107310	38	33	36	37	40	31
ENSMUSG00000107315	0	0	0	0	0	0
ENSMUSG00000036422	0	0	0	0	2	1
ENSMUSG00000079061	0	0	0	0	0	0
ENSMUSG00000079064	0	0	0	0	0	0
ENSMUSG00000101361	2	0	0	0	0	0
ENSMUSG00000103692	0	0	0	0	0	3
ENSMUSG00000100411	0	0	0	0	0	0
ENSMUSG00000103542	0	0	0	0	0	0
ENSMUSG00000103541	0	0	0	0	0	0
ENSMUSG00000103540	0	2	0	0	0	0
ENSMUSG00000103545	0	0	0	0	0	0
ENSMUSG00000103549	0	0	0	0	0	0
ENSMUSG00000059229	0	1	2	1	0	0
ENSMUSG00000039672	3	0	0	0	0	0
ENSMUSG00000035191	0	0	4	4	0	0
ENSMUSG00000039676	0	0	6	0	0	0
ENSMUSG00000097453	0	0	0	0	0	0
ENSMUSG00000097452	0	0	0	0	0	0
ENSMUSG00000097450	1	1	1	2	1	1
ENSMUSG00000097455	8	13	20	4	10	5
ENSMUSG00000097458	0	0	0	0	0	0
ENSMUSG00000077635	0	0	0	0	0	0
ENSMUSG00000106401	0	0	0	0	0	0
ENSMUSG00000081653	0	0	0	0	0	0
ENSMUSG00000100938	0	0	0	0	0	0
ENSMUSG00000100932	0	0	0	0	0	0
ENSMUSG00000089423	0	0	0	0	0	0
ENSMUSG00000100930	0	0	0	0	0	0
ENSMUSG00000114411	0	0	0	0	0	0
ENSMUSG00000114410	0	0	0	0	0	0
ENSMUSG00000089427	0	0	0	0	0	0
ENSMUSG00000077738	0	0	0	0	0	0
ENSMUSG00000109159	0	0	0	0	0	0
ENSMUSG00000020447	4	13	2	0	5	0
ENSMUSG00000050765	1	3	0	2	0	0
ENSMUSG00000050766	0	0	0	2	3	0
ENSMUSG00000050762	2	0	0	0	0	3
ENSMUSG00000050763	0	0	0	0	0	0
ENSMUSG00000105260	0	4	0	0	0	4
ENSMUSG00000105261	5	0	8	0	3	0
ENSMUSG00000105262	0	0	0	0	0	0

ENSMUSG00000105263	60	57	55	43	62	68
ENSMUSG00000105264	0	5	2	2	0	2
ENSMUSG00000105265	6	2	4	11	1	4
ENSMUSG00000105266	0	0	0	0	0	0
ENSMUSG00000105267	0	0	0	0	0	0
ENSMUSG00000105269	0	0	0	0	0	0
ENSMUSG00000073894	0	0	0	0	0	0
ENSMUSG00000066820	0	0	0	0	0	0
ENSMUSG00000077736	0	0	0	0	0	0
ENSMUSG00000058685	0	0	0	0	0	0
ENSMUSG00000066828	0	0	0	0	0	0
ENSMUSG00000081720	0	0	2	0	0	4
ENSMUSG00000091859	0	0	0	0	0	0
ENSMUSG00000105885	0	0	0	0	0	0
ENSMUSG00000105887	0	0	0	0	0	0
ENSMUSG00000105880	0	0	0	0	2	0
ENSMUSG00000105882	0	0	0	0	0	0
ENSMUSG00000057754	0	0	0	0	0	0
ENSMUSG00000096496	0	0	0	0	0	0
ENSMUSG00000096497	0	0	0	0	0	0
ENSMUSG00000105888	0	0	0	0	0	0
ENSMUSG00000105889	0	0	0	0	0	0
ENSMUSG00000096492	1	0	1	1	0	1
ENSMUSG00000020940	0	0	0	0	0	0
ENSMUSG00000108450	0	0	0	0	0	0
ENSMUSG00000108451	0	0	0	0	0	0
ENSMUSG00000108454	0	0	0	0	0	0
ENSMUSG00000108456	14	12	10	14	4	6
ENSMUSG00000108457	0	0	0	0	0	0
ENSMUSG00000104137	0	0	0	0	0	0
ENSMUSG00000108459	0	0	0	0	0	0
ENSMUSG00000104135	0	0	0	0	0	0
ENSMUSG00000104134	0	0	0	0	0	0
ENSMUSG00000104132	0	0	0	0	0	0
ENSMUSG00000104131	0	0	0	0	0	0
ENSMUSG00000021314	65	55	37	68	42	103
ENSMUSG00000057280	39	29	37	5	10	19
ENSMUSG00000059768	0	0	0	0	0	0
ENSMUSG00000059763	0	0	0	0	0	0
ENSMUSG00000059762	0	0	0	0	0	0
ENSMUSG00000102518	0	0	0	0	0	0
ENSMUSG00000102417	0	0	0	0	0	0
ENSMUSG00000110882	7	4	8	7	2	3
ENSMUSG00000110880	0	0	0	0	0	0
ENSMUSG00000110881	0	0	0	3	0	0
ENSMUSG00000110886	0	0	0	0	0	0
ENSMUSG00000110884	4	0	0	0	0	0
ENSMUSG00000110888	6	4	5	9	2	4
ENSMUSG00000110889	0	0	0	0	0	0
ENSMUSG00000091148	0	0	2	0	0	0
ENSMUSG00000065410	0	0	0	0	0	0
ENSMUSG00000091142	0	0	0	0	0	0
ENSMUSG00000091140	0	0	0	0	0	0
ENSMUSG00000091146	0	0	0	0	0	0
ENSMUSG00000056155	10	11	3	0	6	0
ENSMUSG00000056158	5	0	3	0	0	0
ENSMUSG00000111565	0	3	3	0	0	0
ENSMUSG00000053508	0	0	0	0	0	0

ENSMUSG00000042501	0	0	0	0	0	0
ENSMUSG00000094539	0	0	0	0	0	0
ENSMUSG00000094538	0	0	0	0	0	0
ENSMUSG00000022155	0	0	0	0	0	0
ENSMUSG00000094535	0	0	0	0	0	0
ENSMUSG00000094534	4	0	0	0	0	2
ENSMUSG00000111823	0	0	0	0	0	0
ENSMUSG00000111822	0	0	0	0	0	0
ENSMUSG00000111825	2	0	1	0	2	0
ENSMUSG00000065906	0	0	0	0	0	0
ENSMUSG00000065907	0	0	0	0	0	0
ENSMUSG00000065903	0	0	0	0	0	0
ENSMUSG00000094391	0	0	0	1	0	0
ENSMUSG00000027925	0	0	0	0	0	0
ENSMUSG00000027927	0	0	0	0	0	0
ENSMUSG00000093169	0	0	1	0	0	0
ENSMUSG00000027923	0	0	0	0	0	0
ENSMUSG00000094399	0	0	0	0	0	0
ENSMUSG00000093164	0	0	0	0	0	0
ENSMUSG00000093163	1	0	0	0	0	1
ENSMUSG00000093161	0	0	0	0	0	0
ENSMUSG00000093160	0	0	0	0	0	0
ENSMUSG00000086226	0	0	0	0	0	0
ENSMUSG00000086227	0	0	0	0	0	0
ENSMUSG00000086224	0	0	0	0	0	0
ENSMUSG00000017688	0	0	0	0	0	0
ENSMUSG00000086228	17	11	8	11	24	18
ENSMUSG00000097848	0	0	0	0	0	0
ENSMUSG00000097840	6	0	4	0	0	2
ENSMUSG00000097841	3	0	0	0	0	0
ENSMUSG00000090219	3	3	0	0	0	0
ENSMUSG00000017978	121	103	96	67	107	116
ENSMUSG00000049411	215	234	256	278	212	303
ENSMUSG00000029564	0	0	0	0	0	0
ENSMUSG00000047021	0	0	0	0	0	0
ENSMUSG00000047025	0	0	0	0	0	0
ENSMUSG00000084802	0	0	0	0	0	0
ENSMUSG00000003070	10	7	14	18	18	24
ENSMUSG00000071640	0	0	0	0	0	0
ENSMUSG00000046991	0	4	1	0	0	0
ENSMUSG00000109195	5	3	4	4	3	3
ENSMUSG00000109192	0	0	0	0	0	0
ENSMUSG00000046995	0	0	0	0	0	0
ENSMUSG00000109190	0	0	0	0	0	0
ENSMUSG00000076140	0	1	0	0	0	0
ENSMUSG00000106219	6	0	8	0	0	0
ENSMUSG00000076142	1	2	1	0	1	1
ENSMUSG00000102388	0	0	0	0	0	0
ENSMUSG00000106215	0	0	0	0	0	0
ENSMUSG00000109657	0	0	0	0	0	0
ENSMUSG00000005952	4	7	6	0	0	4
ENSMUSG00000109651	0	0	0	0	0	0
ENSMUSG00000107263	8	4	4	4	3	0
ENSMUSG00000109650	0	0	0	0	0	0
ENSMUSG00000109443	0	0	0	0	0	0
ENSMUSG00000109444	0	0	0	0	0	0
ENSMUSG00000109446	0	0	0	0	0	0
ENSMUSG00000109447	0	0	0	0	0	0

ENSMUSG00000096821	0	0	0	0	0	0
ENSMUSG00000096823	0	0	0	0	0	0
ENSMUSG00000096822	0	0	0	0	0	0
ENSMUSG00000104909	0	0	0	0	3	0
ENSMUSG00000096829	0	0	0	0	0	0
ENSMUSG00000030742	12	4	8	2	0	11
ENSMUSG00000110064	0	0	0	0	0	0
ENSMUSG00000110066	0	0	0	0	0	0
ENSMUSG00000110061	1	11	1	13	17	7
ENSMUSG00000110063	0	0	0	0	0	0
ENSMUSG00000104904	11	6	0	9	13	4
ENSMUSG00000054146	0	0	0	0	0	0
ENSMUSG00000054141	0	0	0	0	0	0
ENSMUSG00000054142	2	0	0	0	0	0
ENSMUSG00000104902	0	0	0	0	0	0
ENSMUSG00000110311	1	2	1	1	1	3
ENSMUSG00000058755	9	6	9	5	6	9
ENSMUSG00000105746	6	8	0	0	8	0
ENSMUSG00000105745	23	24	17	8	4	28
ENSMUSG00000105744	0	0	1	0	0	2
ENSMUSG00000105749	0	0	0	0	0	0
ENSMUSG00000082354	0	0	0	0	0	0
ENSMUSG00000083805	0	0	0	0	0	0
ENSMUSG00000005045	0	13	19	16	4	36
ENSMUSG00000108716	0	0	0	0	0	0
ENSMUSG00000108713	0	0	0	0	0	0
ENSMUSG00000108712	0	2	0	0	0	0
ENSMUSG00000108711	0	0	0	0	0	0
ENSMUSG00000108719	0	0	0	0	0	0
ENSMUSG00000108718	0	0	0	0	0	0
ENSMUSG00000083804	3	0	0	0	0	0
ENSMUSG00000111560	0	0	0	0	0	0
ENSMUSG00000083303	0	0	0	0	0	0
ENSMUSG00000083300	0	0	0	2	0	0
ENSMUSG00000099738	0	0	0	0	0	0
ENSMUSG00000083309	0	0	0	0	0	0
ENSMUSG00000000673	11	0	24	0	0	0
ENSMUSG00000041078	4	0	1	2	3	0
ENSMUSG00000083807	0	0	0	0	2	0
ENSMUSG00000112446	1	12	4	11	6	5
ENSMUSG00000112447	0	0	0	0	0	0
ENSMUSG00000085223	8	4	0	3	1	5
ENSMUSG00000102126	0	0	0	0	0	0
ENSMUSG00000085221	0	0	0	0	0	0
ENSMUSG00000100268	0	0	0	0	0	0
ENSMUSG00000085226	5	4	0	3	1	2
ENSMUSG00000085225	0	0	0	0	0	0
ENSMUSG00000085224	0	0	0	0	0	0
ENSMUSG00000026648	431	367	580	256	381	313
ENSMUSG00000026646	117	151	157	75	120	101
ENSMUSG00000102122	0	0	0	0	0	0
ENSMUSG00000026644	10	19	4	15	5	8
ENSMUSG00000026645	0	0	0	0	0	0
ENSMUSG00000093952	0	0	0	0	0	0
ENSMUSG00000093950	0	0	0	0	0	0
ENSMUSG00000079263	0	0	0	0	0	0
ENSMUSG00000079265	0	0	0	0	0	0
ENSMUSG00000079266	0	0	0	0	0	2

ENSMUSG00000100262	0	0	0	0	0	0
ENSMUSG00000079269	0	0	0	0	0	0
ENSMUSG00000083801	0	0	0	0	0	0
ENSMUSG00000100260	0	0	0	0	0	0
ENSMUSG00000100266	0	1	0	0	0	0
ENSMUSG00000100265	0	0	0	0	0	0
ENSMUSG00000100264	0	0	0	0	0	0
ENSMUSG00000045318	9	3	12	8	16	8
ENSMUSG00000112941	13	16	15	0	12	14
ENSMUSG00000045314	0	3	0	1	6	0
ENSMUSG00000113760	0	0	0	0	0	0
ENSMUSG00000070178	0	0	0	0	0	0
ENSMUSG00000114843	0	0	0	0	0	0
ENSMUSG00000114840	0	0	0	0	0	0
ENSMUSG00000071493	0	0	0	0	0	0
ENSMUSG00000114846	0	0	0	0	0	0
ENSMUSG00000102196	0	0	0	0	0	0
ENSMUSG00000114845	0	0	0	0	0	0
ENSMUSG00000063506	126	145	321	68	98	89
ENSMUSG00000063507	0	0	0	0	0	0
ENSMUSG00000074454	0	0	0	0	0	0
ENSMUSG00000078137	14	14	17	36	62	53
ENSMUSG00000078132	0	0	0	0	0	0
ENSMUSG00000078131	0	0	0	0	0	0
ENSMUSG00000078130	0	0	0	0	0	0
ENSMUSG00000107674	0	0	0	0	0	0
ENSMUSG00000081218	0	0	0	0	0	0
ENSMUSG00000081216	0	0	0	0	0	0
ENSMUSG00000081215	0	0	0	0	0	0
ENSMUSG00000081211	0	0	0	0	0	0
ENSMUSG00000110352	0	1	2	0	3	4
ENSMUSG00000073103	4	0	0	0	0	0
ENSMUSG00000073102	0	2	0	0	2	0
ENSMUSG00000099965	0	0	0	0	0	0
ENSMUSG00000099964	0	0	0	0	0	0
ENSMUSG00000099967	0	0	0	0	0	0
ENSMUSG00000099961	0	0	0	0	0	0
ENSMUSG00000099960	0	0	0	0	0	0
ENSMUSG00000099963	0	0	0	0	0	0
ENSMUSG00000099962	0	0	0	0	0	0
ENSMUSG00000099969	0	0	1	0	0	0
ENSMUSG00000099968	0	0	0	0	0	0
ENSMUSG00000101303	0	0	0	0	0	0
ENSMUSG00000048400	0	0	0	2	0	0
ENSMUSG00000048402	200	88	161	28	39	37
ENSMUSG00000077378	0	0	0	0	0	0
ENSMUSG00000077379	0	0	0	0	0	0
ENSMUSG00000113688	14	11	10	6	12	8
ENSMUSG00000098611	0	0	0	0	0	0
ENSMUSG00000098612	0	0	0	0	0	0
ENSMUSG00000031383	0	0	0	0	0	0
ENSMUSG00000031381	65	184	117	151	128	194
ENSMUSG00000098619	0	0	0	0	0	0
ENSMUSG00000031385	82	92	60	145	119	173
ENSMUSG00000031384	0	0	0	0	0	0
ENSMUSG00000030382	0	2	0	2	3	3
ENSMUSG00000102968	0	0	0	0	0	0
ENSMUSG00000102963	45	29	42	38	44	38

ENSMUSG00000043003	33	46	16	100	43	44
ENSMUSG00000102966	0	0	0	0	0	0
ENSMUSG00000102965	0	0	0	0	0	0
ENSMUSG00000112149	1	6	2	2	1	3
ENSMUSG00000049758	0	5	0	0	0	0
ENSMUSG00000041592	96	54	80	21	30	30
ENSMUSG00000031654	5	4	0	4	0	6
ENSMUSG00000031651	0	0	0	0	0	0
ENSMUSG00000101305	0	0	0	0	0	0
ENSMUSG00000093745	0	0	0	0	0	0
ENSMUSG00000067971	0	0	0	0	0	0
ENSMUSG00000030525	0	8	1	0	5	3
ENSMUSG00000088159	0	0	0	0	0	0
ENSMUSG00000082178	0	0	0	0	0	0
ENSMUSG00000030523	0	2	2	3	0	5
ENSMUSG00000088152	0	0	0	0	0	0
ENSMUSG00000082177	0	0	0	1	0	0
ENSMUSG00000088150	0	0	0	0	2	0
ENSMUSG00000082175	0	0	0	0	0	0
ENSMUSG00000082172	0	0	0	0	0	0
ENSMUSG00000088154	0	0	0	0	0	0
ENSMUSG00000088155	0	0	0	0	0	0
ENSMUSG00000101433	0	0	0	0	0	0
ENSMUSG00000101435	0	0	0	0	0	0
ENSMUSG00000101434	0	0	0	0	0	0
ENSMUSG00000101436	0	0	0	0	0	0
ENSMUSG00000021208	15	0	3	0	0	2
ENSMUSG00000107873	0	3	0	3	3	0
ENSMUSG00000107876	4	3	4	0	0	2
ENSMUSG00000107879	112	137	157	100	184	250
ENSMUSG00000103362	0	0	0	0	0	0
ENSMUSG00000103361	0	0	0	0	0	0
ENSMUSG00000103365	0	0	0	0	0	0
ENSMUSG00000103369	0	0	0	0	0	0
ENSMUSG00000103368	0	3	0	1	3	0
ENSMUSG00000040061	91	109	143	86	97	128
ENSMUSG00000040065	0	0	0	0	0	0
ENSMUSG00000089208	0	0	1	0	2	0
ENSMUSG00000084639	0	0	0	0	0	0
ENSMUSG00000084633	0	0	0	0	0	0
ENSMUSG00000084631	0	0	0	0	0	0
ENSMUSG00000084637	0	0	0	0	0	0
ENSMUSG00000084636	0	0	0	0	0	0
ENSMUSG00000084635	0	0	0	0	0	0
ENSMUSG00000089206	0	0	0	0	0	0
ENSMUSG00000075198	0	0	0	0	0	0
ENSMUSG00000075199	0	0	0	0	0	0
ENSMUSG00000075196	0	0	0	0	0	0
ENSMUSG00000075197	0	0	0	0	0	0
ENSMUSG00000075194	0	0	0	0	0	0
ENSMUSG00000075195	0	0	0	0	0	0
ENSMUSG00000075192	0	0	0	0	0	0
ENSMUSG00000075193	0	0	0	0	0	0
ENSMUSG00000075190	0	0	0	0	0	0
ENSMUSG00000022311	0	0	0	0	0	2
ENSMUSG00000022315	0	0	0	0	0	0
ENSMUSG00000021081	0	0	0	0	0	0
ENSMUSG00000064697	0	0	0	0	0	0

ENSMUSG00000064691	0	0	0	0	0	0
ENSMUSG00000064698	0	0	0	0	0	0
ENSMUSG00000095938	0	0	1	0	0	1
ENSMUSG00000020229	0	0	0	0	0	0
ENSMUSG00000049028	0	0	2	2	0	0
ENSMUSG00000095936	0	0	0	0	0	0
ENSMUSG00000100472	0	0	0	0	0	0
ENSMUSG00000100473	0	0	0	0	0	0
ENSMUSG00000041333	0	0	0	0	0	0
ENSMUSG00000100471	0	0	0	0	0	0
ENSMUSG00000100476	0	0	0	0	0	0
ENSMUSG00000100477	0	0	0	0	0	0
ENSMUSG00000100474	0	0	0	0	0	0
ENSMUSG00000100475	0	0	0	0	0	0
ENSMUSG00000096564	0	0	0	0	0	0
ENSMUSG00000100478	0	0	0	0	0	0
ENSMUSG00000100479	0	0	0	0	0	0
ENSMUSG00000068999	0	0	0	0	0	0
ENSMUSG00000096565	0	0	0	0	0	0
ENSMUSG00000096566	0	0	0	0	0	0
ENSMUSG00000113716	0	0	0	0	0	0
ENSMUSG00000113714	0	0	0	0	0	0
ENSMUSG00000102188	0	0	0	0	0	2
ENSMUSG00000102189	0	0	0	0	0	0
ENSMUSG00000064527	0	0	0	0	0	0
ENSMUSG00000064526	0	0	0	0	0	0
ENSMUSG00000064529	0	0	0	0	0	0
ENSMUSG00000102185	0	0	0	0	0	0
ENSMUSG00000035547	209	173	264	75	81	111
ENSMUSG00000102187	0	0	0	0	0	0
ENSMUSG00000102180	0	0	2	4	3	2
ENSMUSG00000035540	0	0	0	0	0	0
ENSMUSG00000102182	0	0	0	0	0	0
ENSMUSG00000088966	0	0	0	0	0	0
ENSMUSG00000107122	0	0	0	0	0	0
ENSMUSG00000107125	0	0	0	0	0	3
ENSMUSG00000113711	0	0	0	0	0	0
ENSMUSG00000107129	0	0	0	0	0	0
ENSMUSG00000113710	0	0	0	0	0	1
ENSMUSG00000079741	0	0	0	0	0	0
ENSMUSG00000079740	0	0	0	0	0	0
ENSMUSG00000023047	7	2	4	0	0	2
ENSMUSG00000023045	9	0	10	0	0	0
ENSMUSG00000023041	0	0	0	0	0	0
ENSMUSG00000028012	2	2	2	14	10	12
ENSMUSG00000078481	0	0	0	0	0	0
ENSMUSG00000069117	12	0	10	0	5	15
ENSMUSG00000096180	0	0	0	0	0	0
ENSMUSG00000069118	0	0	0	0	0	0
ENSMUSG00000074715	5	34	20	20	16	21
ENSMUSG00000029769	132	76	195	87	34	52
ENSMUSG00000029766	0	0	0	0	0	0
ENSMUSG00000101981	0	0	0	0	0	0
ENSMUSG00000101980	0	0	0	0	0	0
ENSMUSG00000101983	0	0	0	0	0	0
ENSMUSG00000101982	0	0	0	0	0	0
ENSMUSG00000101985	0	0	0	0	0	0
ENSMUSG00000101984	0	0	0	0	0	0

ENSMUSG00000101987	0	0	0	0	0	0
ENSMUSG00000088638	0	0	0	0	0	0
ENSMUSG00000101989	0	0	0	0	0	0
ENSMUSG00000101988	0	0	0	0	0	0
ENSMUSG00000088635	0	0	0	0	0	0
ENSMUSG00000088633	0	0	0	0	0	0
ENSMUSG00000061469	0	0	0	0	0	0
ENSMUSG00000088630	0	0	0	0	0	0
ENSMUSG00000021680	0	0	0	0	0	0
ENSMUSG00000021685	0	0	0	0	0	0
ENSMUSG00000063535	24	26	24	20	13	14
ENSMUSG00000024064	0	0	0	0	0	0
ENSMUSG00000088988	0	0	0	0	0	0
ENSMUSG00000106454	0	0	0	0	0	0
ENSMUSG00000106455	4	4	11	6	7	6
ENSMUSG00000038199	0	0	0	0	0	0
ENSMUSG00000113778	0	0	1	2	0	0
ENSMUSG00000106450	31	43	30	61	46	52
ENSMUSG00000111400	0	0	0	0	0	0
ENSMUSG00000088981	1	0	0	0	0	0
ENSMUSG00000088980	0	0	0	1	0	0
ENSMUSG00000113777	0	0	0	0	0	0
ENSMUSG00000088985	0	0	0	0	0	0
ENSMUSG00000113770	0	0	0	0	0	0
ENSMUSG00000113773	0	0	0	0	0	0
ENSMUSG00000113772	0	0	0	0	0	0
ENSMUSG00000049674	0	0	0	0	0	0
ENSMUSG00000089589	0	0	0	0	0	0
ENSMUSG00000089587	0	0	0	0	0	0
ENSMUSG00000089584	0	0	0	0	0	0
ENSMUSG00000089585	0	0	0	0	0	0
ENSMUSG00000089583	0	0	0	0	0	0
ENSMUSG00000089580	0	0	1	0	0	0
ENSMUSG00000089581	1	2	2	2	3	1
ENSMUSG00000097398	0	0	0	0	0	0
ENSMUSG00000097399	0	6	9	4	7	6
ENSMUSG00000106982	2	0	0	0	0	0
ENSMUSG00000106986	1	1	1	1	1	1
ENSMUSG00000106987	2	0	0	0	0	0
ENSMUSG00000097391	71	71	97	34	27	35
ENSMUSG00000022886	0	0	0	0	0	0
ENSMUSG00000097642	1	1	1	0	1	0
ENSMUSG00000054383	16	33	37	19	31	30
ENSMUSG00000104588	0	6	0	0	0	2
ENSMUSG00000097646	0	0	0	0	0	0
ENSMUSG00000097645	3	0	15	3	0	0
ENSMUSG00000104582	0	0	0	0	0	0
ENSMUSG00000097648	0	0	0	0	0	0
ENSMUSG00000104586	0	0	5	0	3	0
ENSMUSG00000104587	0	0	0	0	0	0
ENSMUSG00000104584	1	0	0	0	0	0
ENSMUSG00000101129	0	0	0	0	0	0
ENSMUSG00000069806	64	21	67	22	34	35
ENSMUSG00000105185	0	0	0	0	0	0
ENSMUSG00000105184	22	7	9	8	7	6
ENSMUSG00000105186	2	3	2	2	1	1
ENSMUSG00000114208	0	0	0	0	0	0
ENSMUSG00000109603	0	0	4	0	0	2

ENSMUSG00000105183	0	0	0	0	0	0
ENSMUSG00000107432	0	0	0	1	7	0
ENSMUSG00000114204	0	0	0	0	0	0
ENSMUSG00000114206	0	0	0	0	0	0
ENSMUSG00000114207	0	0	2	3	0	0
ENSMUSG00000105189	1	1	0	2	0	1
ENSMUSG00000114201	0	0	0	0	0	0
ENSMUSG00000114203	0	0	0	0	0	0
ENSMUSG00000107434	0	0	0	0	0	6
ENSMUSG00000112917	1	2	0	2	2	1
ENSMUSG00000112914	0	5	11	6	0	7
ENSMUSG00000112915	0	0	0	0	0	0
ENSMUSG00000112912	0	0	0	0	0	0
ENSMUSG00000112913	0	0	0	0	0	0
ENSMUSG00000112910	0	0	0	0	0	0
ENSMUSG00000112919	18	16	17	22	14	24
ENSMUSG00000107438	0	0	0	0	0	0
ENSMUSG00000058728	0	0	0	0	0	0
ENSMUSG00000058729	178	208	202	223	180	240
ENSMUSG00000044201	8	0	0	0	4	4
ENSMUSG00000058725	0	0	0	0	0	0
ENSMUSG00000110455	0	0	0	0	0	0
ENSMUSG00000110454	0	0	0	0	0	0
ENSMUSG00000110450	0	0	0	0	0	0
ENSMUSG00000060780	0	0	0	0	0	0
ENSMUSG00000050266	5	3	2	10	2	1
ENSMUSG00000060787	0	0	0	0	0	0
ENSMUSG00000098508	0	0	0	0	0	0
ENSMUSG00000096510	0	1	1	1	0	1
ENSMUSG00000096513	0	0	0	0	0	0
ENSMUSG00000096512	1	0	0	1	0	0
ENSMUSG00000096514	0	0	0	0	0	0
ENSMUSG00000096516	2	0	0	0	0	0
ENSMUSG00000023868	197	192	206	178	266	211
ENSMUSG00000111378	2	0	0	2	0	0
ENSMUSG00000101228	0	0	0	0	0	0
ENSMUSG00000111568	0	0	0	0	0	0
ENSMUSG00000060257	0	0	0	0	5	0
ENSMUSG00000060256	0	0	0	0	0	0
ENSMUSG00000060254	0	0	0	0	0	0
ENSMUSG00000059030	3	0	0	0	0	0
ENSMUSG00000056832	152	142	189	109	136	167
ENSMUSG00000062873	0	0	0	0	0	0
ENSMUSG00000045150	3	0	0	0	0	0
ENSMUSG00000026336	1	0	0	0	0	0
ENSMUSG00000009356	0	0	0	0	0	2
ENSMUSG00000101789	0	0	0	0	0	0
ENSMUSG00000026331	3	0	0	0	0	0
ENSMUSG00000103940	13	4	7	9	9	8
ENSMUSG00000065789	2	1	1	1	0	1
ENSMUSG00000095095	0	0	0	0	0	0
ENSMUSG00000095094	0	0	0	0	0	0
ENSMUSG00000111326	1	0	0	0	0	0
ENSMUSG00000111327	0	5	1	2	3	1
ENSMUSG00000111320	0	0	0	0	0	0
ENSMUSG00000095093	0	0	0	0	0	0
ENSMUSG00000095092	2	0	0	1	0	0
ENSMUSG00000081054	0	0	0	0	0	0

ENSMUSG00000081055	0	0	0	0	0	0
ENSMUSG00000081057	0	0	0	0	0	0
ENSMUSG00000047799	0	0	0	0	0	0
ENSMUSG00000047798	15	10	28	7	4	11
ENSMUSG00000047794	0	0	0	0	3	0
ENSMUSG00000047797	0	0	0	0	0	0
ENSMUSG00000085763	0	4	4	0	0	9
ENSMUSG00000022057	0	0	0	0	0	0
ENSMUSG00000109952	0	0	0	0	0	0
ENSMUSG00000022054	3	5	4	0	5	0
ENSMUSG00000090607	0	0	0	0	0	0
ENSMUSG00000043969	0	0	0	0	0	0
ENSMUSG00000090603	0	0	0	0	0	0
ENSMUSG00000081586	0	0	0	0	0	0
ENSMUSG00000081587	0	0	0	0	0	0
ENSMUSG00000081584	0	0	0	0	0	0
ENSMUSG00000048643	0	0	0	0	0	0
ENSMUSG00000081580	0	0	0	0	0	0
ENSMUSG00000114151	0	0	1	0	0	0
ENSMUSG00000093552	0	0	0	0	0	0
ENSMUSG00000093551	0	0	0	0	0	0
ENSMUSG00000093557	0	0	0	0	0	0
ENSMUSG00000114402	0	0	0	0	0	0
ENSMUSG00000063478	0	0	0	0	0	0
ENSMUSG00000092608	0	0	0	0	0	0
ENSMUSG00000092601	0	0	0	0	0	0
ENSMUSG00000092600	2	0	0	0	0	0
ENSMUSG00000092603	1	0	0	0	0	0
ENSMUSG00000092602	0	0	5	0	0	0
ENSMUSG00000092606	0	0	0	0	0	0
ENSMUSG00000077176	0	0	0	0	0	0
ENSMUSG00000077177	0	0	0	0	0	0
ENSMUSG00000077174	0	0	0	0	0	0
ENSMUSG00000077172	0	0	0	0	0	0
ENSMUSG00000055717	0	0	5	0	0	0
ENSMUSG00000086588	0	0	0	0	0	0
ENSMUSG00000112141	15	12	19	6	5	12
ENSMUSG00000112143	0	0	0	0	0	0
ENSMUSG00000112142	4	12	5	10	4	10
ENSMUSG00000086580	0	1	0	1	0	0
ENSMUSG00000086587	19	27	24	15	23	26
ENSMUSG00000086055	0	0	0	0	0	0
ENSMUSG00000086057	0	0	0	0	0	0
ENSMUSG00000086056	0	0	0	0	0	0
ENSMUSG00000049593	0	0	0	0	0	0
ENSMUSG00000086052	0	0	0	0	0	0
ENSMUSG00000049598	3	0	0	0	0	0
ENSMUSG00000111437	0	0	0	0	0	0
ENSMUSG00000057037	0	0	0	0	0	0
ENSMUSG00000099466	0	0	0	0	0	0
ENSMUSG00000111430	0	0	0	0	0	0
ENSMUSG00000098966	0	1	0	0	1	1
ENSMUSG00000098964	0	0	0	0	0	0
ENSMUSG00000098965	0	1	0	0	0	0
ENSMUSG00000098962	0	0	0	0	0	0
ENSMUSG00000098963	0	0	0	0	0	0
ENSMUSG00000098960	0	0	1	0	0	0
ENSMUSG00000098961	0	0	0	0	0	0

ENSMUSG00000082775	0	0	0	0	0	0
ENSMUSG00000082776	5	6	4	11	10	6
ENSMUSG00000082777	0	0	0	0	0	0
ENSMUSG00000082770	0	0	0	0	0	0
ENSMUSG00000082771	0	0	0	0	5	9
ENSMUSG00000098968	2	2	0	0	3	2
ENSMUSG00000082773	0	0	0	0	0	0
ENSMUSG00000113235	0	0	0	0	0	0
ENSMUSG00000113236	0	0	0	0	0	0
ENSMUSG00000113230	0	0	0	0	0	0
ENSMUSG00000113231	0	0	2	0	0	0
ENSMUSG00000084536	0	0	0	0	0	0
ENSMUSG00000113233	0	0	0	0	0	0
ENSMUSG00000101596	0	0	0	2	0	0
ENSMUSG00000113238	22	19	8	29	32	21
ENSMUSG00000114404	0	0	0	0	0	0
ENSMUSG00000101594	2	0	0	0	0	2
ENSMUSG00000079380	3	2	4	0	0	0
ENSMUSG00000070960	32	22	42	21	34	30
ENSMUSG00000040264	0	2	0	0	0	0
ENSMUSG00000092433	0	0	0	0	0	0
ENSMUSG00000114890	0	0	0	0	0	0
ENSMUSG00000084532	0	0	0	0	0	0
ENSMUSG00000085497	0	0	0	0	3	0
ENSMUSG00000048534	14	20	31	8	6	4
ENSMUSG00000084341	0	2	0	0	0	0
ENSMUSG00000084346	0	0	0	0	0	0
ENSMUSG00000084344	0	0	0	0	0	0
ENSMUSG00000084348	5	3	4	4	3	1
ENSMUSG00000025528	0	0	0	0	0	0
ENSMUSG00000025529	39	37	27	40	37	74
ENSMUSG00000025527	5	5	5	0	4	0
ENSMUSG00000070385	0	0	1	0	0	3
ENSMUSG00000070380	0	0	0	0	0	0
ENSMUSG00000099379	0	0	0	0	0	0
ENSMUSG00000070382	0	0	0	0	0	0
ENSMUSG00000070383	0	0	0	0	0	0
ENSMUSG00000099374	0	0	0	0	0	0
ENSMUSG00000099375	38	41	63	25	39	32
ENSMUSG00000099376	0	0	0	0	0	0
ENSMUSG00000070388	0	4	0	0	8	6
ENSMUSG00000099371	0	0	0	0	0	0
ENSMUSG00000099372	0	0	0	0	0	0
ENSMUSG00000078586	0	0	0	0	0	0
ENSMUSG00000075604	0	0	0	0	0	0
ENSMUSG00000079186	0	5	0	0	0	0
ENSMUSG00000111633	0	0	0	0	0	6
ENSMUSG00000030463	0	0	0	0	0	0
ENSMUSG00000075600	141	136	141	107	119	119
ENSMUSG00000065630	1	0	0	0	0	0
ENSMUSG00000100178	0	0	0	2	0	0
ENSMUSG00000065636	0	0	0	0	0	0
ENSMUSG00000065635	0	0	0	0	0	0
ENSMUSG00000075067	0	0	0	0	0	0
ENSMUSG00000080046	0	0	0	0	0	0
ENSMUSG00000100172	1	0	0	0	0	0
ENSMUSG00000062061	0	0	0	0	0	0
ENSMUSG00000062064	0	0	0	0	0	0

ENSMUSG00000033882	0	6	0	7	0	13
ENSMUSG00000073601	0	0	0	0	0	0
ENSMUSG00000073602	0	0	0	0	0	0
ENSMUSG00000112765	4	7	8	0	0	0
ENSMUSG00000095985	0	0	0	0	0	0
ENSMUSG00000055134	32	35	23	32	55	69
ENSMUSG00000054457	0	0	0	0	0	0
ENSMUSG00000111997	20	31	24	13	4	22
ENSMUSG00000111996	0	0	0	0	0	0
ENSMUSG00000109058	0	0	0	0	0	0
ENSMUSG00000090634	4	0	0	0	0	0
ENSMUSG00000054453	12	10	24	6	8	10
ENSMUSG00000098132	103	107	104	111	131	144
ENSMUSG00000092374	0	0	0	0	0	0
ENSMUSG00000098139	0	0	0	0	0	0
ENSMUSG00000109054	33	34	43	44	31	25
ENSMUSG00000084731	0	0	0	0	0	0
ENSMUSG00000111991	0	0	0	0	0	0
ENSMUSG00000112769	0	0	0	0	0	0
ENSMUSG00000035831	0	0	0	0	0	0
ENSMUSG00000109050	0	0	0	0	0	0
ENSMUSG00000048616	10	4	14	2	0	7
ENSMUSG00000072955	2	4	11	7	2	4
ENSMUSG00000072952	0	0	0	0	5	4
ENSMUSG00000072950	0	0	0	0	0	0
ENSMUSG00000103911	2	0	0	0	0	0
ENSMUSG00000103912	0	0	0	0	0	0
ENSMUSG00000103913	0	0	0	0	0	0
ENSMUSG00000103914	3	0	0	0	0	0
ENSMUSG00000103915	0	0	0	0	0	0
ENSMUSG00000103917	0	0	0	0	0	0
ENSMUSG00000103918	0	0	0	0	0	0
ENSMUSG00000103919	0	0	0	0	0	0
ENSMUSG00000100142	0	0	0	0	0	0
ENSMUSG00000058295	0	0	0	0	0	0
ENSMUSG00000038973	0	0	0	0	0	0
ENSMUSG00000032191	0	0	0	0	0	0
ENSMUSG00000084734	0	0	0	0	0	0
ENSMUSG00000057370	0	0	0	0	0	4
ENSMUSG00000076994	1	0	0	0	0	0
ENSMUSG00000089876	21	12	6	22	17	26
ENSMUSG00000089874	0	0	0	0	0	0
ENSMUSG00000089189	0	0	0	0	0	0
ENSMUSG00000089871	0	0	0	0	0	0
ENSMUSG00000111175	0	0	0	0	0	0
ENSMUSG00000089879	0	0	0	0	0	2
ENSMUSG00000091402	0	0	0	0	0	0
ENSMUSG00000091400	0	0	0	0	0	0
ENSMUSG00000098662	1	2	2	1	1	1
ENSMUSG00000110697	25	23	13	8	0	9
ENSMUSG00000089184	3	2	1	0	0	1
ENSMUSG00000110696	24	53	52	32	43	26
ENSMUSG00000111177	0	0	0	0	0	0
ENSMUSG00000110694	0	0	0	0	0	0
ENSMUSG00000059663	0	0	0	0	0	0
ENSMUSG00000000182	0	0	0	0	3	0
ENSMUSG00000108828	0	0	0	0	0	0
ENSMUSG00000108824	0	0	0	0	0	0

ENSMUSG00000108827	0	0	0	0	0	0
ENSMUSG00000108821	0	0	0	0	0	0
ENSMUSG00000108820	10	11	7	8	7	15
ENSMUSG00000108822	0	0	0	0	0	0
ENSMUSG00000043219	4	0	2	2	3	4
ENSMUSG00000067543	0	0	0	0	0	0
ENSMUSG00000067541	0	0	0	0	0	0
ENSMUSG00000067545	0	0	0	0	0	0
ENSMUSG00000100392	0	2	6	0	0	0
ENSMUSG00000087083	0	0	0	0	0	0
ENSMUSG00000100390	0	0	0	0	0	0
ENSMUSG00000087085	0	0	0	0	0	0
ENSMUSG00000100396	2	11	4	2	2	0
ENSMUSG00000100395	0	0	0	0	0	0
ENSMUSG00000087089	0	0	0	0	0	0
ENSMUSG00000100399	0	0	0	0	0	0
ENSMUSG00000100398	0	0	0	0	0	0
ENSMUSG00000088052	0	0	0	0	0	0
ENSMUSG00000088051	0	0	0	0	0	0
ENSMUSG00000088050	0	0	0	0	0	0
ENSMUSG00000101668	0	0	0	0	0	0
ENSMUSG00000101669	0	0	0	0	0	0
ENSMUSG00000101661	0	0	0	0	0	0
ENSMUSG00000101664	0	0	0	0	0	0
ENSMUSG00000101665	0	0	0	0	0	0
ENSMUSG00000060017	0	0	0	0	0	0
ENSMUSG00000101667	0	0	0	0	0	0
ENSMUSG00000037994	23	31	14	0	3	3
ENSMUSG00000085820	0	0	0	0	0	0
ENSMUSG00000085822	0	0	0	0	0	0
ENSMUSG00000037991	9	12	13	10	28	28
ENSMUSG00000085827	0	0	0	0	0	0
ENSMUSG00000108907	0	0	0	0	0	0
ENSMUSG00000033061	0	0	0	0	0	0
ENSMUSG00000033063	0	0	0	0	0	0
ENSMUSG00000084076	0	0	0	0	0	0
ENSMUSG00000087559	0	0	0	0	0	0
ENSMUSG00000110439	0	0	0	0	0	0
ENSMUSG00000087553	0	0	0	0	0	0
ENSMUSG00000114656	0	0	0	0	0	0
ENSMUSG00000087550	0	0	0	0	0	0
ENSMUSG00000013643	0	0	0	0	0	0
ENSMUSG00000040749	84	91	67	90	95	114
ENSMUSG00000103288	0	0	0	0	0	0
ENSMUSG00000103289	0	0	0	0	0	0
ENSMUSG00000103280	16	12	12	21	10	6
ENSMUSG00000103282	0	0	0	0	0	0
ENSMUSG00000103287	0	0	0	0	0	0
ENSMUSG00000036110	0	0	0	0	0	0
ENSMUSG00000103136	2	0	0	0	0	0
ENSMUSG00000105624	74	79	52	62	70	66
ENSMUSG00000105625	0	0	0	0	0	0
ENSMUSG00000100537	0	0	0	0	0	0
ENSMUSG00000100535	0	0	0	0	0	0
ENSMUSG00000100534	0	0	0	0	0	0
ENSMUSG00000100533	0	0	0	0	0	0
ENSMUSG00000100532	0	0	0	0	0	0
ENSMUSG00000100531	0	0	0	0	0	0

ENSMUSG00000100530	0	0	0	0	0	0
ENSMUSG00000105627	0	0	0	0	0	0
ENSMUSG00000100539	0	0	0	0	0	0
ENSMUSG00000100538	0	0	0	0	0	0
ENSMUSG00000064223	0	0	0	0	0	0
ENSMUSG00000064224	0	0	0	0	0	0
ENSMUSG00000107622	4	0	0	0	0	0
ENSMUSG00000107623	5	0	0	0	0	0
ENSMUSG00000107620	3	1	3	1	1	2
ENSMUSG00000084072	0	0	0	0	0	0
ENSMUSG00000107625	0	0	0	0	0	0
ENSMUSG00000095879	0	0	0	0	1	0
ENSMUSG00000095878	0	0	0	0	0	0
ENSMUSG00000095874	0	0	0	0	0	0
ENSMUSG00000095873	0	2	0	0	0	0
ENSMUSG00000095872	0	0	0	0	0	0
ENSMUSG00000095870	0	0	0	0	0	0
ENSMUSG00000065577	0	1	0	0	1	0
ENSMUSG00000111179	0	0	0	0	0	0
ENSMUSG00000089018	0	0	0	0	0	0
ENSMUSG00000089019	0	2	0	0	0	0
ENSMUSG00000100135	0	0	0	0	0	0
ENSMUSG00000077127	0	0	0	0	0	0
ENSMUSG00000089016	0	0	0	0	0	0
ENSMUSG00000089017	0	0	0	0	0	0
ENSMUSG00000028602	0	8	4	6	2	0
ENSMUSG00000112303	21	11	16	16	21	13
ENSMUSG00000102663	0	0	0	0	0	0
ENSMUSG00000112300	21	24	24	12	18	17
ENSMUSG00000112307	51	58	96	65	85	70
ENSMUSG00000112306	0	0	0	0	0	0
ENSMUSG00000112305	0	0	0	0	0	0
ENSMUSG00000102668	0	0	0	0	0	0
ENSMUSG00000112309	23	7	14	10	10	18
ENSMUSG00000036800	0	0	0	0	0	2
ENSMUSG00000036805	0	0	0	0	0	0
ENSMUSG00000110235	59	42	88	5	12	25
ENSMUSG00000110236	0	0	0	0	0	0
ENSMUSG00000110231	0	0	0	0	0	0
ENSMUSG00000110232	0	0	0	0	0	0
ENSMUSG00000114080	0	0	0	0	0	0
ENSMUSG00000084078	0	0	0	0	0	0
ENSMUSG00000043102	13	16	16	7	7	10
ENSMUSG00000105570	0	0	0	0	0	0
ENSMUSG00000105571	0	0	0	0	3	0
ENSMUSG00000039785	0	0	0	0	0	0
ENSMUSG00000105573	0	0	0	0	0	0
ENSMUSG00000105575	0	0	0	0	0	0
ENSMUSG00000105576	0	0	0	0	0	0
ENSMUSG00000105577	0	0	0	0	0	1
ENSMUSG00000105578	3	0	1	0	1	2
ENSMUSG00000105579	0	0	0	0	0	0
ENSMUSG00000101381	1	1	0	0	0	0
ENSMUSG00000088868	0	0	0	0	0	0
ENSMUSG00000088867	0	0	0	0	0	0
ENSMUSG00000088864	0	0	0	2	0	0
ENSMUSG00000088865	0	0	0	0	0	0
ENSMUSG00000088862	0	0	0	0	0	0

ENSMUSG00000088863	0	0	0	0	0	0
ENSMUSG00000031340	98	78	101	36	33	38
ENSMUSG00000088861	0	0	0	0	0	0
ENSMUSG00000094838	0	0	0	0	0	0
ENSMUSG00000108016	0	0	0	0	0	0
ENSMUSG00000016458	175	164	157	97	132	201
ENSMUSG00000021721	0	6	0	0	0	0
ENSMUSG00000094833	0	0	0	0	0	0
ENSMUSG00000094834	0	0	0	0	0	0
ENSMUSG00000094836	0	0	0	0	0	0
ENSMUSG00000094837	0	0	0	0	0	0
ENSMUSG00000100542	0	0	0	0	0	0
ENSMUSG00000111549	0	0	0	0	0	0
ENSMUSG00000111544	0	0	0	0	0	0
ENSMUSG00000111546	0	0	0	0	0	0
ENSMUSG00000111541	0	0	0	0	0	0
ENSMUSG00000111543	0	0	0	0	0	0
ENSMUSG00000101387	0	0	0	0	0	0
ENSMUSG00000029268	0	0	0	0	0	0
ENSMUSG00000005503	0	0	0	0	0	0
ENSMUSG00000050876	0	0	0	0	0	0
ENSMUSG00000050870	0	0	0	0	0	0
ENSMUSG00000038782	0	9	9	2	12	15
ENSMUSG00000058935	8	0	0	2	0	6
ENSMUSG00000059397	0	0	0	0	0	0
ENSMUSG00000059395	17	18	14	20	16	16
ENSMUSG00000109835	0	0	0	0	0	0
ENSMUSG00000109834	0	0	0	0	0	0
ENSMUSG00000114079	0	0	0	0	0	0
ENSMUSG00000114078	0	0	0	0	0	0
ENSMUSG00000105390	0	0	0	0	0	0
ENSMUSG00000109830	0	0	0	0	0	0
ENSMUSG00000109833	0	0	0	0	0	0
ENSMUSG00000109832	0	0	0	0	0	0
ENSMUSG00000114073	13	18	3	15	56	51
ENSMUSG00000114071	0	0	0	0	0	0
ENSMUSG00000109839	0	0	0	0	0	0
ENSMUSG00000109838	0	0	0	0	0	0
ENSMUSG00000114075	0	0	0	0	0	0
ENSMUSG00000114074	0	0	0	0	0	0
ENSMUSG00000059648	0	0	0	0	0	0
ENSMUSG00000047969	0	0	0	0	0	0
ENSMUSG00000097563	4	3	0	3	3	0
ENSMUSG00000097562	0	0	0	0	0	0
ENSMUSG00000104628	0	0	0	0	0	0
ENSMUSG00000104626	21	17	14	0	4	3
ENSMUSG00000047960	3	0	0	0	0	0
ENSMUSG00000059645	0	0	0	0	0	0
ENSMUSG00000097569	8	8	13	0	6	0
ENSMUSG00000067064	0	0	0	0	0	0
ENSMUSG00000112899	0	0	0	0	0	0
ENSMUSG00000112898	0	0	0	0	0	0
ENSMUSG00000112893	0	0	0	0	0	0
ENSMUSG00000112892	5	9	0	6	0	0
ENSMUSG00000112891	16	26	21	24	19	26
ENSMUSG00000112890	0	0	0	0	0	0
ENSMUSG00000112896	0	0	0	0	0	0
ENSMUSG00000027761	0	0	0	0	0	0

ENSMUSG00000086677	3	0	11	2	6	10
ENSMUSG00000083889	0	0	0	0	0	0
ENSMUSG00000083888	0	0	0	0	0	0
ENSMUSG00000083884	0	0	0	0	0	0
ENSMUSG00000083886	0	1	0	0	0	0
ENSMUSG00000083882	0	0	0	0	0	0
ENSMUSG00000057592	0	0	0	0	0	0
ENSMUSG00000113565	3	2	3	2	2	3
ENSMUSG00000110792	2	0	0	0	0	0
ENSMUSG00000110793	0	0	0	0	0	0
ENSMUSG00000058114	0	0	0	0	0	0
ENSMUSG00000025892	9	7	21	3	2	0
ENSMUSG00000110794	4	9	12	20	21	13
ENSMUSG00000110795	0	2	2	0	0	4
ENSMUSG00000110799	0	0	0	0	0	0
ENSMUSG00000058119	0	0	0	0	0	0
ENSMUSG00000069668	0	0	0	0	0	0
ENSMUSG00000027490	32	44	39	0	40	58
ENSMUSG00000080283	0	3	0	0	0	0
ENSMUSG00000046367	0	0	0	0	0	0
ENSMUSG00000056643	0	0	0	0	0	0
ENSMUSG00000082313	0	0	4	0	7	0
ENSMUSG00000053030	14	12	0	0	0	0
ENSMUSG00000048652	33	37	49	66	34	64
ENSMUSG00000111381	0	0	0	0	0	0
ENSMUSG00000090102	0	0	0	0	0	0
ENSMUSG00000090109	0	0	0	0	0	0
ENSMUSG00000098212	0	0	0	0	0	0
ENSMUSG00000113854	3	2	0	2	1	1
ENSMUSG00000113855	0	0	0	0	0	0
ENSMUSG00000113856	4	5	5	1	8	2
ENSMUSG00000098330	0	0	0	0	0	0
ENSMUSG00000113850	0	1	0	0	0	0
ENSMUSG00000098335	0	0	0	0	0	0
ENSMUSG00000113858	0	0	0	0	0	0
ENSMUSG00000113859	4	4	4	0	0	0
ENSMUSG00000053695	0	0	0	0	0	0
ENSMUSG00000093929	0	0	0	0	0	0
ENSMUSG00000094481	0	0	0	0	0	0
ENSMUSG00000094485	0	0	0	0	0	0
ENSMUSG00000094484	0	0	0	1	0	0
ENSMUSG00000093458	0	0	0	0	0	0
ENSMUSG00000094489	0	0	0	0	0	0
ENSMUSG00000093453	0	3	2	3	4	2
ENSMUSG00000045471	0	0	0	0	0	0
ENSMUSG00000045474	0	0	0	0	0	0
ENSMUSG00000045475	0	0	0	0	0	0
ENSMUSG00000095684	0	0	0	1	0	0
ENSMUSG00000095685	0	0	0	0	0	0
ENSMUSG00000095686	0	0	2	0	0	0
ENSMUSG00000045479	0	0	0	0	0	0
ENSMUSG00000095681	25	18	36	13	28	28
ENSMUSG00000095683	0	0	0	1	0	0
ENSMUSG00000098485	0	0	0	0	0	0
ENSMUSG00000047189	0	0	0	0	0	0
ENSMUSG00000053961	0	0	0	0	0	0
ENSMUSG00000100460	0	0	0	0	0	0
ENSMUSG00000094159	1	0	0	1	0	0

ENSMUSG00000088753	0	0	0	0	0	0
ENSMUSG00000042124	0	0	0	0	0	0
ENSMUSG00000094152	0	0	0	0	0	0
ENSMUSG00000094157	0	0	0	0	0	0
ENSMUSG00000086936	0	0	0	0	0	0
ENSMUSG00000017195	0	2	0	0	0	0
ENSMUSG00000100463	0	0	0	0	0	0
ENSMUSG00000081628	0	0	0	0	0	0
ENSMUSG00000081623	0	0	0	0	0	0
ENSMUSG00000081622	16	31	17	21	16	22
ENSMUSG00000081626	0	0	0	0	0	0
ENSMUSG00000081625	1	0	0	0	0	0
ENSMUSG00000081624	0	0	0	0	0	0
ENSMUSG00000100915	0	0	0	0	0	0
ENSMUSG00000100462	0	0	0	0	0	0
ENSMUSG00000093817	0	0	0	0	0	0
ENSMUSG00000077707	0	0	0	0	0	0
ENSMUSG00000077706	0	0	0	0	0	0
ENSMUSG00000077701	0	0	0	0	0	0
ENSMUSG00000077700	0	0	0	1	0	0
ENSMUSG00000077703	0	0	0	0	0	0
ENSMUSG00000100914	0	0	0	0	0	0
ENSMUSG00000093814	2	0	0	0	0	0
ENSMUSG00000044985	0	0	0	0	0	0
ENSMUSG00000090816	0	0	0	0	0	0
ENSMUSG00000044988	0	0	0	0	0	0
ENSMUSG00000100917	0	0	0	0	0	0
ENSMUSG00000113261	6	6	3	5	6	3
ENSMUSG00000092419	2	0	0	0	0	0
ENSMUSG00000111840	0	0	0	0	0	4
ENSMUSG00000094748	0	0	0	0	0	0
ENSMUSG00000048388	219	217	112	57	76	71
ENSMUSG00000092410	0	0	0	0	0	0
ENSMUSG00000092411	0	0	0	0	0	0
ENSMUSG00000048385	0	0	0	0	0	0
ENSMUSG00000092413	0	0	0	0	0	0
ENSMUSG00000092414	0	0	0	0	0	0
ENSMUSG00000094197	0	0	0	0	0	0
ENSMUSG00000094747	5	3	0	0	0	0
ENSMUSG00000113264	2	0	0	0	0	0
ENSMUSG00000093536	0	2	4	4	2	3
ENSMUSG00000003545	5	24	23	15	7	3
ENSMUSG00000051860	1	0	0	0	0	2
ENSMUSG00000055523	3	0	3	0	0	0
ENSMUSG00000093530	0	0	0	0	0	0
ENSMUSG00000043456	261	309	283	322	348	480
ENSMUSG00000030775	0	0	0	0	0	0
ENSMUSG00000093533	0	0	0	0	0	0
ENSMUSG00000112767	0	0	0	0	0	0
ENSMUSG00000072754	0	0	0	0	0	0
ENSMUSG00000072753	0	0	0	0	0	0
ENSMUSG00000062496	0	8	5	0	2	0
ENSMUSG00000071481	0	0	0	0	0	0
ENSMUSG00000043982	0	0	0	0	0	0
ENSMUSG00000049387	0	8	0	0	5	6
ENSMUSG00000100913	0	0	0	0	0	0
ENSMUSG00000043986	0	0	0	0	0	0
ENSMUSG00000112761	2	0	3	3	0	0

ENSMUSG00000111844	5	6	13	3	6	0
ENSMUSG00000087718	0	0	0	0	0	0
ENSMUSG00000085864	0	0	0	0	0	0
ENSMUSG00000087710	0	0	0	0	0	0
ENSMUSG00000087712	0	1	0	0	0	0
ENSMUSG00000087715	0	0	0	0	0	0
ENSMUSG00000087717	0	0	0	0	0	0
ENSMUSG00000087716	0	0	0	0	0	0
ENSMUSG00000040187	185	197	224	179	176	191
ENSMUSG00000040183	179	184	168	181	188	212
ENSMUSG00000037202	17	39	23	15	15	24
ENSMUSG00000083417	0	0	0	0	0	0
ENSMUSG00000083414	0	2	0	0	0	0
ENSMUSG00000049036	7	3	9	5	0	0
ENSMUSG00000083419	0	0	0	0	0	0
ENSMUSG00000079577	0	0	0	0	0	0
ENSMUSG00000079578	0	0	0	0	0	0
ENSMUSG00000079579	0	0	0	0	0	0
ENSMUSG00000031919	2	0	0	0	5	0
ENSMUSG00000107421	0	0	0	0	0	0
ENSMUSG00000107423	0	0	0	0	0	0
ENSMUSG00000107424	0	0	0	0	0	0
ENSMUSG00000107425	0	0	0	0	0	0
ENSMUSG00000071229	0	0	0	0	0	3
ENSMUSG00000089678	0	0	0	0	0	0
ENSMUSG00000061210	2	2	0	5	0	0
ENSMUSG00000105817	0	0	0	0	0	0
ENSMUSG00000063434	0	0	0	0	0	0
ENSMUSG00000102848	0	0	0	0	0	0
ENSMUSG00000065771	0	2	1	0	0	0
ENSMUSG00000065770	0	0	0	0	0	0
ENSMUSG00000085712	0	0	0	0	0	0
ENSMUSG00000085717	0	0	0	0	0	0
ENSMUSG00000085715	22	5	24	16	24	13
ENSMUSG00000064793	0	0	0	0	0	0
ENSMUSG00000087316	0	0	0	0	0	0
ENSMUSG00000109055	1	0	0	3	0	0
ENSMUSG00000107398	0	0	0	0	0	0
ENSMUSG00000107399	0	0	0	0	0	0
ENSMUSG00000107392	0	0	0	0	0	0
ENSMUSG00000107390	31	21	38	5	23	15
ENSMUSG00000107396	0	0	0	0	0	0
ENSMUSG00000107397	0	0	2	0	2	0
ENSMUSG00000107394	0	0	0	0	0	0
ENSMUSG00000082722	0	0	0	0	0	0
ENSMUSG00000082721	0	0	0	0	0	0
ENSMUSG00000079395	0	0	0	0	0	0
ENSMUSG00000079396	7	17	7	9	10	14
ENSMUSG00000068078	0	0	0	0	0	0
ENSMUSG00000079391	17	9	23	11	11	19
ENSMUSG00000075833	0	1	0	0	0	0
ENSMUSG00000075832	0	0	0	0	0	0
ENSMUSG00000068073	0	0	0	0	0	0
ENSMUSG00000068071	0	0	0	0	0	0
ENSMUSG00000068075	0	0	0	0	0	0
ENSMUSG00000068074	0	0	0	0	0	0
ENSMUSG00000093243	0	0	0	0	0	0
ENSMUSG00000107904	0	0	0	0	0	0

ENSMUSG00000093934	0	0	0	0	0	0
ENSMUSG00000082725	0	0	0	0	0	0
ENSMUSG00000078627	10	7	4	0	0	0
ENSMUSG00000078626	0	0	0	0	0	0
ENSMUSG00000078625	0	0	0	0	0	0
ENSMUSG00000078624	0	1	0	0	1	0
ENSMUSG00000078621	0	0	0	0	0	0
ENSMUSG00000059205	0	0	0	0	0	0
ENSMUSG00000089117	0	0	0	0	0	0
ENSMUSG00000006784	14	12	7	20	32	26
ENSMUSG00000056219	2	2	3	0	2	2
ENSMUSG00000032327	21	5	26	0	2	0
ENSMUSG00000032323	0	2	4	0	2	3
ENSMUSG00000062296	0	4	4	0	0	6
ENSMUSG00000086699	0	0	0	0	0	0
ENSMUSG00000102278	0	3	0	4	3	2
ENSMUSG00000102279	0	0	0	0	0	0
ENSMUSG00000102272	0	3	0	3	4	0
ENSMUSG00000102273	0	0	0	0	0	0
ENSMUSG00000102271	4	0	1	4	0	2
ENSMUSG00000102276	0	0	3	0	0	0
ENSMUSG00000102277	48	64	38	19	22	18
ENSMUSG00000102274	0	0	0	0	0	0
ENSMUSG00000103895	4	0	2	1	0	0
ENSMUSG00000103894	0	0	0	0	0	0
ENSMUSG00000103896	0	0	1	0	0	0
ENSMUSG00000103890	0	0	0	0	0	0
ENSMUSG00000103893	0	0	0	0	0	0
ENSMUSG00000103892	0	0	0	0	0	0
ENSMUSG00000038541	0	0	0	0	0	7
ENSMUSG00000069670	25	13	10	6	10	12
ENSMUSG00000096769	0	0	0	0	0	0
ENSMUSG00000096763	3	2	3	3	2	2
ENSMUSG00000096760	0	0	0	0	0	0
ENSMUSG00000096761	0	0	0	0	0	0
ENSMUSG00000019737	0	12	12	9	15	15
ENSMUSG00000031142	0	0	5	0	0	4
ENSMUSG00000063163	0	0	0	0	0	0
ENSMUSG00000063166	0	0	0	0	0	0
ENSMUSG00000094980	0	0	0	0	0	0
ENSMUSG00000105809	0	0	0	0	0	0
ENSMUSG00000102858	2	1	1	0	0	1
ENSMUSG00000102859	2	0	0	0	0	2
ENSMUSG00000102857	0	0	0	0	0	0
ENSMUSG00000105806	0	0	0	0	0	0
ENSMUSG00000102855	0	0	0	0	0	0
ENSMUSG00000102852	0	0	0	0	0	0
ENSMUSG00000102853	0	0	0	0	0	0
ENSMUSG00000102851	0	3	2	0	0	0
ENSMUSG00000076915	0	0	1	0	0	0
ENSMUSG00000078235	0	0	0	0	0	3
ENSMUSG00000091239	0	0	0	0	0	0
ENSMUSG00000108983	0	2	0	0	0	0
ENSMUSG00000108980	0	0	0	0	0	0
ENSMUSG00000108981	0	2	0	0	0	0
ENSMUSG00000108987	0	0	0	0	0	0
ENSMUSG00000091230	0	0	0	0	0	0
ENSMUSG00000108988	0	0	0	0	0	0

ENSMUSG00000103031	0	0	0	0	0	0
ENSMUSG00000051537	14	20	7	15	9	4
ENSMUSG00000030235	14	13	8	15	20	18
ENSMUSG00000030236	0	0	0	0	0	0
ENSMUSG00000030237	29	57	33	24	35	36
ENSMUSG00000030230	0	0	0	0	0	1
ENSMUSG00000077445	0	0	0	0	0	0
ENSMUSG00000097870	3	0	0	4	0	2
ENSMUSG00000064728	0	0	0	0	0	0
ENSMUSG00000110808	0	0	0	0	0	0
ENSMUSG00000110802	0	0	0	0	0	0
ENSMUSG00000104865	0	0	0	0	0	0
ENSMUSG00000104861	0	0	0	0	0	0
ENSMUSG00000104860	0	0	1	3	0	0
ENSMUSG00000104869	24	24	36	57	48	36
ENSMUSG00000104868	0	0	0	0	0	0
ENSMUSG00000088248	0	2	1	1	0	0
ENSMUSG00000088244	3	0	1	2	0	1
ENSMUSG00000088245	0	0	0	0	0	0
ENSMUSG00000088243	0	0	0	0	0	0
ENSMUSG00000088240	0	0	0	0	0	0
ENSMUSG00000088241	0	0	0	0	0	0
ENSMUSG00000108073	0	0	0	0	0	0
ENSMUSG00000022484	0	0	0	0	0	0
ENSMUSG00000022485	0	4	2	0	0	0
ENSMUSG00000022487	0	0	0	0	0	0
ENSMUSG00000022483	0	0	15	0	0	0
ENSMUSG00000107987	0	0	0	0	0	0
ENSMUSG00000107986	0	0	0	0	0	0
ENSMUSG00000095448	0	0	0	0	3	0
ENSMUSG00000107984	0	0	0	0	0	0
ENSMUSG00000107983	0	0	0	0	0	0
ENSMUSG00000107982	0	0	0	0	0	0
ENSMUSG00000107981	0	0	0	0	0	0
ENSMUSG00000107980	0	0	0	0	0	0
ENSMUSG00000095440	129	118	130	83	90	127
ENSMUSG00000095446	0	0	0	0	0	0
ENSMUSG00000062235	5	6	3	6	6	4
ENSMUSG00000095445	5	3	1	5	2	3
ENSMUSG00000103077	0	0	0	0	0	0
ENSMUSG00000114430	0	0	0	0	0	0
ENSMUSG00000103073	0	0	0	0	0	0
ENSMUSG00000103070	4	2	1	1	1	1
ENSMUSG00000065987	0	0	0	0	0	0
ENSMUSG00000103078	0	0	0	0	0	0
ENSMUSG00000089409	0	0	0	0	0	0
ENSMUSG00000061150	0	0	0	0	0	0
ENSMUSG00000094312	0	0	0	0	0	0
ENSMUSG00000094314	0	0	1	0	0	0
ENSMUSG00000094317	0	0	0	0	0	0
ENSMUSG00000094316	0	0	0	0	0	0
ENSMUSG00000089404	2	0	1	0	0	0
ENSMUSG00000113046	0	0	0	0	0	0
ENSMUSG00000023289	0	0	0	0	0	0
ENSMUSG00000036306	161	166	209	227	282	306
ENSMUSG00000036304	0	4	16	0	2	6
ENSMUSG00000106164	29	32	27	44	54	54
ENSMUSG00000106165	0	0	0	0	0	0

ENSMUSG00000106162	0	3	0	0	0	0
ENSMUSG00000106160	0	0	0	0	0	0
ENSMUSG00000106168	0	0	0	0	0	0
ENSMUSG00000092855	0	0	0	0	0	0
ENSMUSG00000029878	0	3	3	0	0	0
ENSMUSG00000024883	72	83	120	57	73	103
ENSMUSG00000017607	12	14	35	12	13	30
ENSMUSG00000060827	0	0	0	0	0	0
ENSMUSG00000020396	0	0	7	0	0	7
ENSMUSG00000106292	3	4	4	4	3	3
ENSMUSG00000106293	0	0	0	0	0	0
ENSMUSG00000097935	14	12	8	4	7	6
ENSMUSG00000106297	0	0	0	0	0	0
ENSMUSG00000106298	0	0	0	0	0	0
ENSMUSG00000028475	334	388	417	422	446	508
ENSMUSG00000092256	0	0	0	0	0	0
ENSMUSG00000102746	0	0	0	0	0	0
ENSMUSG00000092255	0	0	0	0	0	0
ENSMUSG00000092250	0	0	0	0	0	0
ENSMUSG00000092251	0	0	0	0	0	0
ENSMUSG00000092259	0	0	0	0	0	0
ENSMUSG00000035651	0	0	0	0	0	0
ENSMUSG00000092375	0	0	0	0	0	0
ENSMUSG00000112511	0	0	0	0	0	0
ENSMUSG00000112516	1	4	1	1	0	2
ENSMUSG00000039337	0	0	0	0	0	0
ENSMUSG00000112514	0	2	2	0	0	0
ENSMUSG00000039335	0	0	0	0	0	0
ENSMUSG00000024770	0	0	0	0	0	0
ENSMUSG00000024771	0	0	0	0	0	0
ENSMUSG00000112518	0	0	0	0	0	0
ENSMUSG00000112519	0	0	7	0	0	0
ENSMUSG00000024774	0	0	0	0	0	0
ENSMUSG00000099581	0	0	0	0	0	0
ENSMUSG00000092376	1	0	0	0	0	0
ENSMUSG00000092370	0	0	0	0	0	0
ENSMUSG00000113641	0	0	0	0	0	0
ENSMUSG00000097156	2	0	0	0	0	2
ENSMUSG00000113644	0	0	0	0	0	0
ENSMUSG00000038067	3	2	0	0	3	0
ENSMUSG00000097155	0	2	0	0	0	4
ENSMUSG00000114918	0	0	5	0	0	0
ENSMUSG00000114916	0	0	0	0	0	0
ENSMUSG00000114910	0	0	0	0	0	0
ENSMUSG00000114911	0	0	0	0	0	0
ENSMUSG00000104027	0	0	0	0	0	0
ENSMUSG00000005339	0	0	0	0	0	0
ENSMUSG00000066272	10	6	4	5	10	10
ENSMUSG00000106832	6	3	7	7	3	3
ENSMUSG00000106833	0	0	0	0	0	0
ENSMUSG00000070511	0	0	0	0	0	0
ENSMUSG00000110817	0	4	3	0	0	7
ENSMUSG00000021118	8	11	10	7	18	10
ENSMUSG00000096200	0	0	0	0	0	0
ENSMUSG00000096202	0	0	0	0	0	0
ENSMUSG00000096209	0	0	0	0	0	0
ENSMUSG00000096208	0	0	0	0	0	0
ENSMUSG00000100008	0	0	0	0	0	0

ENSMUSG00000111739	0	0	0	0	0	0
ENSMUSG00000111738	20	21	17	16	15	19
ENSMUSG00000111733	2	0	0	0	0	0
ENSMUSG00000111732	0	0	0	0	0	0
ENSMUSG00000111730	0	2	0	0	0	0
ENSMUSG00000104852	137	157	139	95	91	140
ENSMUSG00000111734	0	0	0	0	0	0
ENSMUSG00000057417	0	0	0	0	0	3
ENSMUSG00000104855	0	0	0	0	0	0
ENSMUSG00000025912	151	194	208	74	74	95
ENSMUSG00000025916	7	8	0	0	7	11
ENSMUSG00000094592	0	0	0	0	0	0
ENSMUSG00000104478	8	3	3	6	4	4
ENSMUSG00000104479	27	26	23	28	21	42
ENSMUSG00000108797	0	0	0	0	0	0
ENSMUSG00000104477	0	0	0	0	0	0
ENSMUSG00000108795	0	0	0	0	0	0
ENSMUSG00000104472	0	0	0	0	0	0
ENSMUSG00000108792	0	0	0	0	0	0
ENSMUSG00000104470	0	0	0	0	0	0
ENSMUSG00000108790	8	8	8	10	12	22
ENSMUSG00000027220	0	0	4	0	0	0
ENSMUSG00000045395	0	0	0	2	0	0
ENSMUSG00000095714	0	0	0	0	0	0
ENSMUSG00000044664	0	0	0	0	0	0
ENSMUSG00000044667	2	9	8	0	0	0
ENSMUSG00000083430	0	0	0	0	0	0
ENSMUSG00000114009	3	0	0	0	0	0
ENSMUSG00000073834	0	0	0	0	0	0
ENSMUSG00000079806	0	0	0	0	0	0
ENSMUSG00000079800	2	0	0	0	0	0
ENSMUSG00000112591	0	0	0	0	3	3
ENSMUSG00000027517	0	0	0	0	0	0
ENSMUSG00000026542	0	0	0	0	0	3
ENSMUSG00000046282	0	0	0	0	5	0
ENSMUSG00000027518	0	0	0	0	0	0
ENSMUSG00000046287	0	1	0	0	0	0
ENSMUSG00000046133	0	0	0	0	0	0
ENSMUSG00000074987	8	3	4	12	5	4
ENSMUSG00000095163	0	0	0	0	0	0
ENSMUSG00000095168	0	1	0	2	0	1
ENSMUSG00000045539	0	0	0	0	0	0
ENSMUSG00000086666	0	0	0	0	0	0
ENSMUSG00000052131	0	0	0	0	2	0
ENSMUSG00000052135	26	27	16	32	44	65
ENSMUSG00000028864	114	136	186	31	55	95
ENSMUSG00000028865	0	0	0	0	0	0
ENSMUSG00000081295	0	0	0	0	0	0
ENSMUSG00000081292	0	0	0	0	0	0
ENSMUSG00000081293	2	0	0	0	0	0
ENSMUSG00000081290	0	0	0	0	0	0
ENSMUSG00000091874	0	0	0	0	0	0
ENSMUSG00000081298	0	0	0	0	0	0
ENSMUSG00000080749	0	0	0	0	0	0
ENSMUSG00000080748	0	0	0	0	2	0
ENSMUSG00000038327	0	0	0	0	0	0
ENSMUSG00000080743	0	0	1	0	2	0
ENSMUSG00000080742	2	0	0	0	0	0

ENSMUSG00000094496	0	0	0	1	0	0
ENSMUSG00000114000	0	0	0	0	0	0
ENSMUSG00000093648	0	0	1	0	0	0
ENSMUSG00000093649	0	0	0	0	0	0
ENSMUSG00000093642	0	0	0	0	0	0
ENSMUSG00000086743	5	6	10	10	3	0
ENSMUSG00000048480	0	0	0	0	0	3
ENSMUSG00000048483	4	8	0	0	7	8
ENSMUSG00000048484	0	0	6	4	0	2
ENSMUSG00000048485	0	0	7	0	0	0
ENSMUSG00000098697	0	0	0	0	0	0
ENSMUSG00000047661	0	0	0	0	0	0
ENSMUSG00000079048	3	0	3	0	6	0
ENSMUSG00000047667	4	0	0	0	0	0
ENSMUSG00000098756	0	0	0	0	0	0
ENSMUSG00000033233	135	189	184	152	174	212
ENSMUSG00000098983	1	0	0	0	0	0
ENSMUSG00000073686	0	0	0	0	0	0
ENSMUSG00000022496	8	10	0	0	5	8
ENSMUSG00000113177	0	0	0	0	0	0
ENSMUSG00000112650	0	0	0	0	0	0
ENSMUSG00000077514	0	0	0	0	0	0
ENSMUSG00000077517	0	0	0	0	0	0
ENSMUSG00000077511	0	0	0	0	0	0
ENSMUSG00000040576	0	0	0	0	0	0
ENSMUSG00000077518	0	0	0	0	0	0
ENSMUSG00000077519	0	0	0	0	0	0
ENSMUSG00000113172	0	0	0	6	1	0
ENSMUSG00000082249	0	0	0	0	0	0
ENSMUSG00000082244	0	1	0	0	0	0
ENSMUSG00000082247	7	9	7	8	8	5
ENSMUSG00000082240	0	0	0	0	0	0
ENSMUSG00000082242	0	0	0	0	0	0
ENSMUSG00000048226	0	0	0	0	0	0
ENSMUSG00000099274	0	0	0	0	0	0
ENSMUSG00000099270	0	0	0	0	0	0
ENSMUSG00000099273	0	0	0	0	1	0
ENSMUSG00000099279	0	0	0	0	0	0
ENSMUSG00000099278	0	0	0	0	0	0
ENSMUSG00000083599	0	0	0	0	0	0
ENSMUSG00000083598	0	0	0	0	0	0
ENSMUSG00000083592	0	0	0	0	0	0
ENSMUSG00000083590	0	0	0	0	0	0
ENSMUSG00000083597	0	0	0	0	0	0
ENSMUSG00000083594	1	3	4	5	2	1
ENSMUSG00000093032	0	0	1	0	0	0
ENSMUSG00000062791	0	0	0	0	0	0
ENSMUSG00000100740	0	0	0	0	0	0
ENSMUSG00000100741	0	0	0	0	0	0
ENSMUSG00000100742	0	0	0	0	0	0
ENSMUSG00000100743	0	0	0	0	0	0
ENSMUSG00000100744	0	0	0	0	0	0
ENSMUSG00000100745	0	0	0	0	0	0
ENSMUSG00000100746	0	0	0	0	0	0
ENSMUSG00000100749	0	0	0	0	0	0
ENSMUSG00000041620	0	0	0	0	0	0
ENSMUSG00000112488	0	0	0	0	0	0
ENSMUSG00000102458	0	0	0	0	0	0

ENSMUSG00000064619	0	0	0	0	0	0
ENSMUSG00000064618	0	0	0	0	0	0
ENSMUSG00000064617	0	0	0	0	0	0
ENSMUSG00000102453	0	0	0	0	0	0
ENSMUSG00000102450	0	0	0	0	0	0
ENSMUSG00000064613	0	0	0	0	0	0
ENSMUSG00000083625	0	0	0	0	0	0
ENSMUSG00000083620	0	0	0	0	0	0
ENSMUSG00000083623	8	0	0	0	0	7
ENSMUSG00000083629	0	0	0	0	0	0
ENSMUSG00000083628	0	0	0	0	0	0
ENSMUSG00000087908	0	0	0	0	0	0
ENSMUSG00000075117	0	0	0	0	0	0
ENSMUSG00000075114	0	0	0	0	0	0
ENSMUSG00000075115	0	0	0	0	0	0
ENSMUSG00000075112	0	0	0	0	0	0
ENSMUSG00000075113	0	3	0	0	0	0
ENSMUSG00000075110	0	0	0	0	0	0
ENSMUSG00000075111	0	0	0	0	0	0
ENSMUSG00000031893	3	3	0	0	0	4
ENSMUSG00000087902	0	0	0	0	0	0
ENSMUSG00000087904	0	0	0	0	0	0
ENSMUSG00000087905	0	0	0	0	0	0
ENSMUSG00000075119	0	0	0	0	0	0
ENSMUSG00000061397	0	0	6	0	0	0
ENSMUSG00000061393	101	74	96	155	216	189
ENSMUSG00000061392	0	0	0	0	0	0
ENSMUSG00000085502	0	0	0	0	0	0
ENSMUSG00000085500	0	0	0	0	0	0
ENSMUSG00000085507	0	0	0	0	0	0
ENSMUSG00000085504	0	0	0	0	0	0
ENSMUSG00000085508	0	0	3	0	0	0
ENSMUSG00000029348	57	42	47	22	14	31
ENSMUSG00000107219	0	0	0	0	0	0
ENSMUSG00000107218	0	0	0	0	0	0
ENSMUSG00000107217	0	0	0	0	0	0
ENSMUSG00000107214	0	0	0	0	0	0
ENSMUSG00000107213	0	0	0	0	0	0
ENSMUSG00000107212	0	0	0	0	0	0
ENSMUSG00000033794	0	0	0	0	0	2
ENSMUSG00000101909	0	0	0	0	0	0
ENSMUSG00000101908	0	0	0	0	0	0
ENSMUSG00000101900	0	0	0	0	0	0
ENSMUSG00000101903	0	4	0	3	4	4
ENSMUSG00000101902	0	0	0	0	0	0
ENSMUSG00000101907	0	0	0	0	0	0
ENSMUSG00000110298	51	67	69	43	46	84
ENSMUSG00000083171	0	0	0	0	0	0
ENSMUSG00000036672	136	111	196	85	84	104
ENSMUSG00000103460	0	6	0	4	11	2
ENSMUSG00000103461	0	0	0	0	0	0
ENSMUSG00000103462	0	0	0	0	0	0
ENSMUSG00000103463	0	0	0	0	0	0
ENSMUSG00000103464	3	1	3	2	1	2
ENSMUSG00000103465	0	0	0	0	0	0
ENSMUSG00000103466	0	2	2	0	0	2
ENSMUSG00000103468	0	0	0	0	0	0
ENSMUSG00000065254	2	0	1	1	0	0

ENSMUSG00000065255	0	0	0	0	0	0
ENSMUSG00000065256	0	0	0	0	0	0
ENSMUSG00000093112	0	0	0	0	0	0
ENSMUSG00000094325	0	0	0	0	0	0
ENSMUSG00000063821	11	10	13	0	3	6
ENSMUSG00000063823	0	0	0	0	0	0
ENSMUSG00000063827	0	0	0	0	0	0
ENSMUSG00000079698	0	0	0	0	0	0
ENSMUSG00000030854	2	0	3	0	0	2
ENSMUSG00000093115	0	0	0	0	0	0
ENSMUSG00000024578	0	0	0	0	0	0
ENSMUSG00000024579	42	55	75	26	24	63
ENSMUSG00000089506	0	0	0	0	0	0
ENSMUSG00000089507	0	0	0	0	0	0
ENSMUSG00000097310	5	3	5	0	3	0
ENSMUSG00000097311	0	0	0	0	0	0
ENSMUSG00000097313	0	4	0	0	4	0
ENSMUSG00000022805	3	3	0	2	0	0
ENSMUSG00000073226	0	0	0	0	0	0
ENSMUSG00000022809	2	0	0	0	0	0
ENSMUSG00000038665	2	5	2	3	5	16
ENSMUSG00000062142	0	0	0	0	0	0
ENSMUSG00000062157	177	124	183	195	198	171
ENSMUSG00000062154	0	0	0	0	0	0
ENSMUSG00000069733	0	0	0	0	0	6
ENSMUSG00000102339	0	0	0	0	0	0
ENSMUSG00000102338	0	0	0	0	0	0
ENSMUSG00000102337	0	0	0	0	0	2
ENSMUSG00000102336	167	161	187	89	114	173
ENSMUSG00000102335	0	0	0	0	0	0
ENSMUSG00000102334	0	0	0	0	0	0
ENSMUSG00000105101	0	0	0	0	0	0
ENSMUSG00000102332	0	0	0	0	0	0
ENSMUSG00000102331	45	17	22	4	8	8
ENSMUSG00000102330	1	1	0	3	1	3
ENSMUSG00000060402	7	14	7	4	0	4
ENSMUSG00000096049	1	1	1	0	1	0
ENSMUSG00000096046	0	0	0	0	0	0
ENSMUSG00000096045	0	0	0	0	0	0
ENSMUSG00000096044	0	0	0	0	0	0
ENSMUSG00000096043	0	0	0	0	0	0
ENSMUSG00000096041	0	0	0	0	0	0
ENSMUSG00000094116	0	0	0	0	0	0
ENSMUSG00000066704	0	0	0	0	0	0
ENSMUSG00000060407	0	0	0	0	0	0
ENSMUSG00000096289	4	2	0	0	0	0
ENSMUSG00000096288	0	0	0	0	0	0
ENSMUSG00000094115	0	0	0	0	0	0
ENSMUSG00000105292	0	0	0	0	0	0
ENSMUSG00000105294	0	0	0	0	0	0
ENSMUSG00000021056	0	0	0	0	0	0
ENSMUSG00000021057	331	319	267	375	449	256
ENSMUSG00000057650	0	0	0	0	0	0
ENSMUSG00000105299	0	0	0	0	0	0
ENSMUSG00000022657	0	0	0	0	0	0
ENSMUSG00000114331	0	0	0	0	0	0
ENSMUSG00000102753	0	0	0	0	0	0
ENSMUSG00000103594	0	0	0	0	0	0

ENSMUSG00000114332	0	0	0	5	0	0
ENSMUSG00000091297	11	8	8	6	14	10
ENSMUSG00000050150	16	12	8	9	9	13
ENSMUSG00000104278	0	0	0	0	0	0
ENSMUSG00000108558	0	0	0	0	0	0
ENSMUSG00000108554	0	2	0	0	0	0
ENSMUSG00000104276	0	0	0	0	0	0
ENSMUSG00000050158	0	5	0	0	0	3
ENSMUSG00000104270	0	0	0	0	0	0
ENSMUSG00000108550	0	0	0	0	0	0
ENSMUSG00000108553	0	0	0	0	0	0
ENSMUSG00000108552	0	0	0	0	0	0
ENSMUSG00000071322	0	0	9	0	0	0
ENSMUSG00000096287	0	0	0	0	0	0
ENSMUSG00000101178	0	0	0	0	0	0
ENSMUSG00000101179	0	0	0	0	0	0
ENSMUSG00000107526	0	0	0	0	0	0
ENSMUSG00000060701	0	0	0	0	0	0
ENSMUSG00000101171	0	0	0	3	0	0
ENSMUSG00000101173	0	0	0	0	0	0
ENSMUSG00000101177	0	0	0	0	0	0
ENSMUSG00000010751	29	35	89	16	55	54
ENSMUSG00000084269	0	0	0	0	0	0
ENSMUSG00000101338	0	0	0	0	0	0
ENSMUSG00000064932	0	0	0	0	0	0
ENSMUSG00000101339	0	0	0	0	0	0
ENSMUSG00000091061	0	0	0	0	0	0
ENSMUSG00000073938	1	3	0	0	0	0
ENSMUSG00000056215	5	0	6	0	0	0
ENSMUSG00000101332	1	0	0	0	0	0
ENSMUSG00000095769	0	0	0	0	1	0
ENSMUSG00000101333	0	0	0	0	0	0
ENSMUSG00000082616	0	0	0	0	0	0
ENSMUSG00000095765	0	0	3	3	0	0
ENSMUSG00000026919	0	0	0	0	0	0
ENSMUSG00000095763	0	0	0	0	0	0
ENSMUSG00000104746	0	0	0	0	0	0
ENSMUSG00000100227	0	0	0	0	0	0
ENSMUSG00000100226	0	0	0	0	0	0
ENSMUSG00000108246	4	5	3	0	1	6
ENSMUSG00000100223	0	0	0	0	0	0
ENSMUSG00000100221	0	0	0	0	0	0
ENSMUSG00000101336	0	0	0	0	0	0
ENSMUSG00000100229	0	0	1	0	0	0
ENSMUSG00000027068	18	33	41	6	4	12
ENSMUSG00000042428	151	152	195	132	146	165
ENSMUSG00000061577	5	0	15	6	0	0
ENSMUSG00000102655	0	0	0	0	0	0
ENSMUSG00000113729	0	0	2	0	0	0
ENSMUSG00000086503	69	61	47	69	96	29
ENSMUSG00000062833	0	0	0	0	0	0
ENSMUSG00000086500	7	9	4	3	4	0
ENSMUSG00000086504	0	0	0	0	0	0
ENSMUSG00000086509	0	0	0	0	0	0
ENSMUSG00000102657	38	36	44	17	14	17
ENSMUSG00000114171	0	0	0	0	0	0
ENSMUSG00000092248	0	0	0	0	0	0
ENSMUSG00000112497	0	0	0	0	0	0

ENSMUSG00000112496	0	0	11	0	0	0
ENSMUSG00000112495	12	12	10	15	9	15
ENSMUSG00000112494	0	0	2	0	0	0
ENSMUSG00000112492	0	6	0	7	4	2
ENSMUSG00000112491	0	0	0	0	0	3
ENSMUSG00000093331	0	0	0	0	0	0
ENSMUSG00000049515	7	7	4	6	0	6
ENSMUSG00000049511	0	0	7	0	0	0
ENSMUSG00000067144	0	0	0	0	0	0
ENSMUSG00000093338	0	0	0	0	0	0
ENSMUSG00000112498	0	0	0	0	0	0
ENSMUSG00000009670	0	0	3	0	3	12
ENSMUSG00000112721	2	0	0	0	0	1
ENSMUSG00000112720	0	0	0	0	0	0
ENSMUSG00000112723	0	0	0	0	3	1
ENSMUSG00000109098	0	0	0	0	0	0
ENSMUSG00000112725	0	0	0	0	0	0
ENSMUSG00000112724	0	0	0	0	0	0
ENSMUSG00000112727	25	26	38	21	36	33
ENSMUSG00000112726	0	0	0	0	0	0
ENSMUSG00000109093	0	0	0	0	0	0
ENSMUSG00000112728	4	0	0	0	0	0
ENSMUSG00000109091	0	0	0	0	0	0
ENSMUSG00000109097	0	0	8	0	10	0
ENSMUSG00000109096	6	5	4	4	0	2
ENSMUSG00000109095	0	0	0	0	0	0
ENSMUSG00000017832	9	6	5	13	9	11
ENSMUSG00000106315	0	0	0	0	0	0
ENSMUSG00000087045	0	0	0	0	0	0
ENSMUSG00000106316	0	0	0	0	0	0
ENSMUSG00000106311	0	0	0	0	0	0
ENSMUSG00000106310	0	3	0	0	0	0
ENSMUSG00000106313	0	0	0	0	0	1
ENSMUSG00000106319	3	0	0	0	1	0
ENSMUSG00000106318	0	0	0	0	0	0
ENSMUSG00000097055	21	15	13	7	4	13
ENSMUSG00000097054	0	0	0	0	0	0
ENSMUSG00000097050	0	0	0	0	0	0
ENSMUSG00000110097	0	0	0	0	0	0
ENSMUSG00000051362	0	0	0	0	0	0
ENSMUSG00000051361	0	0	0	0	0	0
ENSMUSG00000087042	0	0	0	0	0	0
ENSMUSG00000051367	62	72	110	50	63	50
ENSMUSG00000004296	0	0	3	0	0	0
ENSMUSG00000096902	0	0	0	0	0	0
ENSMUSG00000096903	0	0	0	0	0	0
ENSMUSG00000114893	10	0	7	0	0	0
ENSMUSG00000096901	1	2	2	1	2	1
ENSMUSG00000096906	0	2	1	0	0	0
ENSMUSG00000114894	0	0	0	2	1	0
ENSMUSG00000096904	11	19	16	14	4	0
ENSMUSG00000114896	0	0	0	0	0	0
ENSMUSG00000114899	0	0	0	0	0	0
ENSMUSG00000046899	0	0	0	0	0	0
ENSMUSG00000110383	0	0	0	0	0	0
ENSMUSG00000110382	0	18	4	10	4	0
ENSMUSG00000110385	0	0	0	0	0	0
ENSMUSG00000110384	0	0	0	0	0	0

ENSMUSG00000110386	35	36	57	29	26	31
ENSMUSG00000110389	0	0	0	0	0	0
ENSMUSG00000099578	0	0	0	0	2	0
ENSMUSG00000099577	0	0	0	0	0	0
ENSMUSG00000039321	36	40	22	17	10	13
ENSMUSG00000109325	0	0	0	1	0	0
ENSMUSG00000105665	0	0	0	0	0	0
ENSMUSG00000105666	2	0	0	0	0	0
ENSMUSG00000099575	0	0	0	0	0	0
ENSMUSG00000105660	0	3	2	0	0	3
ENSMUSG00000039323	0	0	20	0	0	0
ENSMUSG00000105662	0	0	0	0	0	0
ENSMUSG00000081950	0	0	0	0	0	0
ENSMUSG00000114762	0	0	0	0	0	0
ENSMUSG00000102116	0	0	0	0	0	0
ENSMUSG00000109329	0	0	0	0	0	0
ENSMUSG00000114766	0	0	2	0	0	0
ENSMUSG00000114765	0	0	0	0	0	0
ENSMUSG00000114764	0	0	0	0	0	0
ENSMUSG00000110659	0	0	0	0	0	0
ENSMUSG00000025038	0	12	11	4	11	0
ENSMUSG00000099570	0	0	0	0	0	0
ENSMUSG00000110653	0	0	0	0	0	2
ENSMUSG00000081955	0	0	0	0	0	0
ENSMUSG00000091779	0	0	0	0	0	0
ENSMUSG00000070304	16	10	9	26	12	27
ENSMUSG00000070305	99	96	107	104	106	121
ENSMUSG00000070306	0	3	3	0	0	0
ENSMUSG00000108054	0	0	0	0	0	0
ENSMUSG00000108056	0	0	0	0	0	0
ENSMUSG00000108057	0	0	0	0	0	0
ENSMUSG00000108050	0	0	0	0	0	0
ENSMUSG00000108052	0	0	0	0	0	0
ENSMUSG00000108058	0	0	2	0	0	0
ENSMUSG00000083023	0	0	0	0	0	0
ENSMUSG00000083020	0	0	0	0	0	0
ENSMUSG00000083026	0	0	0	0	0	0
ENSMUSG00000083025	0	0	0	0	0	0
ENSMUSG00000083024	0	0	0	0	0	0
ENSMUSG00000083029	0	0	0	0	0	0
ENSMUSG00000092377	2	0	0	0	0	0
ENSMUSG00000082991	0	0	0	0	0	0
ENSMUSG00000082993	0	0	0	0	0	0
ENSMUSG00000082994	1	0	0	0	0	0
ENSMUSG00000082995	0	0	0	0	0	0
ENSMUSG00000082997	0	0	0	0	0	0
ENSMUSG00000102113	0	0	0	1	0	0
ENSMUSG00000050069	0	0	0	0	0	0
ENSMUSG00000107528	0	0	0	0	0	0
ENSMUSG00000079103	6	0	0	5	0	0
ENSMUSG00000083748	0	0	0	0	0	0
ENSMUSG00000083749	0	0	0	0	0	0
ENSMUSG00000089322	0	0	0	0	0	0
ENSMUSG00000085079	0	0	0	0	0	0
ENSMUSG00000022176	0	0	0	0	0	0
ENSMUSG00000085073	0	0	0	0	0	0
ENSMUSG00000085070	0	0	0	0	0	0
ENSMUSG00000085077	0	0	0	0	0	0

ENSMUSG00000085074	0	0	0	0	0	0
ENSMUSG00000085075	0	0	0	0	0	2
ENSMUSG00000084530	0	0	0	0	0	1
ENSMUSG00000001661	0	1	2	3	0	2
ENSMUSG00000084531	0	0	0	1	0	0
ENSMUSG00000026070	19	66	26	7	4	3
ENSMUSG00000026073	5	5	5	4	3	5
ENSMUSG00000033805	2	5	0	0	0	2
ENSMUSG00000041789	3	0	3	0	0	0
ENSMUSG00000053185	0	0	0	0	0	0
ENSMUSG00000053184	0	0	0	0	0	0
ENSMUSG00000081318	0	0	0	0	0	0
ENSMUSG00000081313	0	0	0	0	0	0
ENSMUSG00000081312	0	0	0	0	0	0
ENSMUSG00000081310	1	0	0	0	0	0
ENSMUSG00000081317	0	0	0	2	0	0
ENSMUSG00000081316	2	1	1	0	2	0
ENSMUSG00000081315	0	0	0	0	0	0
ENSMUSG00000081314	4	3	5	6	9	3
ENSMUSG00000073028	0	0	0	0	0	0
ENSMUSG00000073027	0	0	0	0	0	0
ENSMUSG00000099842	0	0	0	0	0	0
ENSMUSG00000099840	0	0	0	0	0	0
ENSMUSG00000099841	0	0	0	0	0	0
ENSMUSG00000099846	0	0	0	2	3	0
ENSMUSG00000027301	0	0	0	0	0	0
ENSMUSG00000099845	0	0	0	0	0	0
ENSMUSG00000099849	0	0	0	0	0	0
ENSMUSG00000025991	0	0	0	4	0	0
ENSMUSG00000084840	0	0	0	0	0	0
ENSMUSG00000084842	0	0	0	1	2	4
ENSMUSG00000084844	43	9	37	15	18	37
ENSMUSG00000084847	0	0	0	0	0	0
ENSMUSG00000090527	0	0	0	0	0	0
ENSMUSG00000098713	7	8	13	16	15	16
ENSMUSG00000099474	0	0	0	0	0	0
ENSMUSG00000098717	0	0	0	0	0	0
ENSMUSG00000088941	0	0	0	0	0	0
ENSMUSG00000090981	0	0	0	0	0	0
ENSMUSG00000109101	21	28	28	35	32	23
ENSMUSG00000111845	0	0	0	0	0	0
ENSMUSG00000044816	0	0	0	0	0	0
ENSMUSG00000043164	0	5	4	0	3	0
ENSMUSG00000105483	0	0	0	0	0	0
ENSMUSG00000109105	14	11	22	10	12	18
ENSMUSG00000043168	4	0	0	0	0	0
ENSMUSG00000105486	0	0	0	0	0	0
ENSMUSG00000077235	0	0	0	0	0	2
ENSMUSG00000096890	2	0	0	0	0	6
ENSMUSG00000077237	0	0	0	0	0	0
ENSMUSG00000077231	0	0	0	0	0	0
ENSMUSG00000077230	0	0	0	0	0	0
ENSMUSG00000096891	0	0	0	0	0	0
ENSMUSG00000094078	0	0	0	0	0	0
ENSMUSG00000096893	0	0	0	0	0	0
ENSMUSG00000096894	0	0	0	0	0	0
ENSMUSG00000096896	0	0	0	0	0	0
ENSMUSG00000081877	0	0	0	0	0	0

ENSMUSG00000114906	0	2	0	0	0	0
ENSMUSG00000072049	0	0	0	0	0	0
ENSMUSG00000104532	0	0	0	0	0	0
ENSMUSG00000107598	0	0	0	0	0	0
ENSMUSG00000046908	14	16	19	18	8	10
ENSMUSG00000082039	0	0	0	0	0	0
ENSMUSG00000082038	0	0	0	0	0	0
ENSMUSG00000082033	0	0	0	0	0	0
ENSMUSG00000082032	2	0	2	1	0	0
ENSMUSG00000087003	0	0	0	0	0	0
ENSMUSG00000082030	0	0	0	0	0	0
ENSMUSG00000082037	0	0	0	0	0	0
ENSMUSG00000082036	10	15	12	13	7	8
ENSMUSG00000087007	0	0	0	0	0	0
ENSMUSG00000101376	0	0	0	0	0	0
ENSMUSG00000101377	0	0	0	0	0	0
ENSMUSG00000101374	0	0	0	0	0	0
ENSMUSG00000101375	0	0	0	0	0	0
ENSMUSG00000101372	0	0	0	0	0	0
ENSMUSG00000101373	0	0	0	0	0	0
ENSMUSG00000101371	0	0	0	0	3	2
ENSMUSG00000037913	10	5	20	3	2	2
ENSMUSG00000071531	4	0	14	4	0	2
ENSMUSG00000103201	7	13	8	0	0	2
ENSMUSG00000103202	0	0	0	0	0	0
ENSMUSG00000103204	0	0	0	0	0	4
ENSMUSG00000043833	46	42	44	40	31	33
ENSMUSG00000103209	0	0	0	0	0	0
ENSMUSG00000095741	0	0	0	0	0	0
ENSMUSG00000055643	0	0	0	0	0	0
ENSMUSG00000113938	0	0	0	0	0	0
ENSMUSG00000070526	28	42	36	44	27	38
ENSMUSG00000093184	0	0	0	0	0	0
ENSMUSG00000089366	0	0	0	0	0	0
ENSMUSG00000075099	0	0	0	0	0	0
ENSMUSG00000089363	0	0	0	0	0	0
ENSMUSG00000075093	0	0	0	0	0	0
ENSMUSG00000075092	0	0	0	0	0	0
ENSMUSG00000075091	0	0	0	0	0	0
ENSMUSG00000075090	0	0	0	0	0	0
ENSMUSG00000075097	0	0	0	0	0	0
ENSMUSG00000075095	0	0	0	0	0	0
ENSMUSG00000075094	0	0	0	0	0	0
ENSMUSG00000022218	7	9	19	21	16	20
ENSMUSG00000022211	44	77	47	128	85	106
ENSMUSG00000022212	0	2	0	0	0	0
ENSMUSG00000102552	0	0	0	0	0	0
ENSMUSG00000102551	0	0	0	0	0	0
ENSMUSG00000102550	0	0	2	0	0	0
ENSMUSG00000084066	0	0	0	0	0	0
ENSMUSG00000089095	0	0	0	0	0	0
ENSMUSG00000089097	0	0	0	0	0	0
ENSMUSG00000089098	0	0	0	0	0	0
ENSMUSG00000084068	0	0	0	0	0	0
ENSMUSG00000084069	0	0	0	0	2	0
ENSMUSG00000068890	0	0	0	0	0	0
ENSMUSG00000065866	0	0	0	0	0	0
ENSMUSG00000065865	0	0	0	0	0	0

ENSMUSG00000099022	0	0	0	0	0	0
ENSMUSG00000095744	0	0	0	0	0	0
ENSMUSG00000035429	0	0	2	0	0	0
ENSMUSG00000064408	0	0	0	0	0	0
ENSMUSG00000060988	2	0	0	0	0	0
ENSMUSG00000064407	0	0	0	1	0	0
ENSMUSG00000060985	0	0	0	0	0	0
ENSMUSG00000035427	0	0	0	0	0	0
ENSMUSG00000064400	0	0	1	0	1	1
ENSMUSG00000107008	2	0	0	0	3	0
ENSMUSG00000107006	0	0	0	0	0	0
ENSMUSG00000107004	0	0	0	0	0	0
ENSMUSG00000107005	0	0	0	0	0	3
ENSMUSG00000079666	0	0	0	0	0	0
ENSMUSG00000023169	590	491	600	394	438	569
ENSMUSG00000023165	0	0	0	0	0	0
ENSMUSG00000113932	0	0	0	0	0	0
ENSMUSG00000104474	3	0	0	3	0	0
ENSMUSG00000058975	19	36	22	34	21	14
ENSMUSG00000058976	0	0	0	0	0	0
ENSMUSG00000085330	0	0	0	0	0	0
ENSMUSG00000085333	0	0	0	0	0	0
ENSMUSG00000097181	0	0	0	0	0	0
ENSMUSG00000062585	48	39	78	31	36	50
ENSMUSG00000074059	2	0	0	0	0	0
ENSMUSG00000113930	6	23	12	33	12	33
ENSMUSG00000088578	0	0	0	0	0	0
ENSMUSG00000088579	0	0	0	0	0	0
ENSMUSG00000088574	0	0	0	0	0	0
ENSMUSG00000088575	0	0	0	0	0	0
ENSMUSG00000088570	1	0	0	0	2	0
ENSMUSG00000088571	0	0	0	0	0	0
ENSMUSG00000088572	0	0	0	0	0	0
ENSMUSG00000113937	0	0	0	0	0	0
ENSMUSG00000069385	0	0	0	0	0	0
ENSMUSG00000077834	0	0	0	0	0	0
ENSMUSG00000036882	7	3	4	5	11	4
ENSMUSG00000103785	0	2	0	0	0	0
ENSMUSG00000103787	0	0	0	0	0	0
ENSMUSG00000113039	0	0	0	0	0	0
ENSMUSG00000103781	0	0	0	0	0	0
ENSMUSG00000103782	0	0	0	0	0	0
ENSMUSG00000061637	2	0	0	0	0	0
ENSMUSG00000034773	7	3	9	0	5	3
ENSMUSG00000113031	0	0	0	0	0	0
ENSMUSG00000103788	0	0	0	0	0	0
ENSMUSG00000034777	2	0	3	0	1	0
ENSMUSG00000038255	0	0	2	0	0	0
ENSMUSG00000034486	3	0	8	3	5	0
ENSMUSG00000063672	0	0	0	0	0	0
ENSMUSG00000034488	42	58	32	18	14	33
ENSMUSG00000016982	0	2	2	0	0	0
ENSMUSG00000051160	3	0	0	0	0	0
ENSMUSG00000059319	3	0	0	0	0	0
ENSMUSG00000029151	13	0	0	0	0	0
ENSMUSG00000029155	5	13	13	6	5	12
ENSMUSG00000029154	0	0	0	0	0	0
ENSMUSG00000106669	0	0	0	0	0	0

ENSMUSG00000106667	0	0	0	0	0	0
ENSMUSG00000106660	0	0	0	0	0	0
ENSMUSG00000114569	0	0	0	0	0	0
ENSMUSG00000109521	0	0	0	0	0	0
ENSMUSG00000109520	0	0	0	0	0	0
ENSMUSG00000109527	0	0	0	0	0	0
ENSMUSG00000109526	0	0	0	0	0	0
ENSMUSG00000109525	0	0	0	0	0	0
ENSMUSG00000113950	0	0	0	0	0	0
ENSMUSG00000114561	0	0	0	0	2	0
ENSMUSG00000114560	0	0	0	0	0	0
ENSMUSG00000109529	5	0	0	0	0	0
ENSMUSG00000109528	0	0	0	0	0	0
ENSMUSG00000114565	0	0	0	0	0	0
ENSMUSG00000114564	0	0	0	0	0	0
ENSMUSG00000114567	0	0	0	0	0	0
ENSMUSG00000114566	0	0	0	0	0	0
ENSMUSG00000100846	0	0	0	0	0	0
ENSMUSG00000100843	0	0	2	0	0	0
ENSMUSG00000100842	0	2	0	0	0	0
ENSMUSG00000100849	0	0	0	0	0	0
ENSMUSG00000100848	0	0	0	0	0	0
ENSMUSG00000108692	0	0	0	0	0	0
ENSMUSG00000108693	28	33	54	28	17	15
ENSMUSG00000112811	0	0	0	0	1	2
ENSMUSG00000112810	0	0	2	0	0	0
ENSMUSG00000112817	0	0	0	0	0	0
ENSMUSG00000108697	0	0	0	0	0	0
ENSMUSG00000112815	1	2	0	0	0	0
ENSMUSG00000108695	0	0	0	0	0	0
ENSMUSG00000091923	0	0	0	0	0	0
ENSMUSG00000091924	0	0	0	0	0	0
ENSMUSG00000105318	0	0	0	0	0	0
ENSMUSG00000105319	0	0	0	0	0	0
ENSMUSG00000105314	0	0	0	0	0	0
ENSMUSG00000105315	0	0	0	0	0	0
ENSMUSG00000110712	0	5	0	0	0	0
ENSMUSG00000110713	0	0	0	0	0	0
ENSMUSG00000066914	0	0	0	0	0	0
ENSMUSG00000058194	0	0	0	0	0	0
ENSMUSG00000058192	453	448	465	404	444	479
ENSMUSG00000110714	0	0	0	0	0	0
ENSMUSG00000050343	0	0	2	0	0	0
ENSMUSG00000096114	0	0	0	0	0	0
ENSMUSG00000095509	0	0	0	0	0	0
ENSMUSG00000096117	0	0	0	0	0	0
ENSMUSG00000096658	0	0	0	0	0	0
ENSMUSG00000023949	0	0	0	0	0	0
ENSMUSG00000096654	0	0	0	0	0	0
ENSMUSG00000023945	0	0	5	5	0	0
ENSMUSG00000096652	0	0	0	0	0	0
ENSMUSG00000023943	0	0	0	0	0	0
ENSMUSG00000096650	0	0	0	0	0	0
ENSMUSG00000096651	0	0	1	0	0	1
ENSMUSG00000111748	36	47	44	22	36	23
ENSMUSG00000111749	0	0	0	0	0	0
ENSMUSG00000111747	3	0	0	0	2	0
ENSMUSG00000026272	0	0	0	0	0	0

ENSMUSG00000026271	25	26	81	28	16	16
ENSMUSG00000113870	0	0	0	0	0	0
ENSMUSG00000095505	0	0	0	0	0	0
ENSMUSG00000064876	0	0	0	0	0	0
ENSMUSG00000064872	0	0	0	0	0	0
ENSMUSG00000083779	0	0	0	0	0	0
ENSMUSG00000111049	0	0	0	0	0	0
ENSMUSG00000111048	0	0	0	0	0	0
ENSMUSG00000095358	0	0	0	0	0	0
ENSMUSG00000097036	0	0	0	0	0	0
ENSMUSG00000111396	0	0	0	0	0	0
ENSMUSG00000103256	2	0	0	0	0	3
ENSMUSG00000111041	0	0	0	0	0	0
ENSMUSG00000095353	2	0	0	0	0	2
ENSMUSG00000095356	0	0	0	0	0	0
ENSMUSG00000095357	0	0	0	0	0	0
ENSMUSG00000111047	2	0	0	0	0	0
ENSMUSG00000111046	0	0	0	0	0	0
ENSMUSG00000081151	2	0	0	3	1	0
ENSMUSG00000081150	1	0	0	0	0	1
ENSMUSG00000081155	0	0	0	0	0	0
ENSMUSG00000081154	0	0	0	0	0	0
ENSMUSG00000107044	8	8	7	0	4	0
ENSMUSG00000087563	0	0	0	0	0	0
ENSMUSG00000088394	0	0	0	0	0	2
ENSMUSG00000056399	0	0	0	0	0	0
ENSMUSG00000088395	0	0	0	0	0	0
ENSMUSG00000107049	0	2	0	0	0	0
ENSMUSG00000090728	0	0	0	0	0	0
ENSMUSG00000088397	0	0	0	0	0	0
ENSMUSG00000090722	56	49	85	25	30	25
ENSMUSG00000094409	2	0	0	0	0	0
ENSMUSG00000094408	0	0	0	0	0	0
ENSMUSG00000042219	0	0	0	0	0	0
ENSMUSG00000094401	0	0	0	0	0	0
ENSMUSG00000056023	2	0	0	0	0	0
ENSMUSG00000044903	3	0	0	0	0	0
ENSMUSG00000108760	0	0	0	0	0	0
ENSMUSG00000090897	0	0	0	0	0	0
ENSMUSG00000090894	0	0	0	0	0	0
ENSMUSG00000028362	13	12	14	0	0	0
ENSMUSG00000108767	2	4	2	0	2	2
ENSMUSG00000077039	0	0	0	0	0	0
ENSMUSG00000055612	63	51	91	57	49	83
ENSMUSG00000112020	34	12	16	0	0	0
ENSMUSG00000055610	5	0	0	0	0	0
ENSMUSG00000112027	12	9	11	15	11	14
ENSMUSG00000112024	0	0	0	0	0	0
ENSMUSG00000112028	0	0	0	0	0	0
ENSMUSG00000046463	33	40	46	8	9	27
ENSMUSG00000086315	3	0	0	3	9	3
ENSMUSG00000038165	0	0	0	0	0	0
ENSMUSG00000086319	0	0	0	0	0	0
ENSMUSG00000092767	0	0	0	0	0	0
ENSMUSG00000092762	0	0	0	0	0	0
ENSMUSG00000092763	0	0	0	0	0	0
ENSMUSG00000092761	0	0	0	0	1	0
ENSMUSG00000077784	0	0	0	0	0	0

ENSMUSG00000077786	0	0	0	0	0	0
ENSMUSG00000077783	0	0	0	0	0	0
ENSMUSG00000096806	0	0	0	0	0	0
ENSMUSG00000049307	63	74	110	83	44	62
ENSMUSG00000034730	1	1	5	1	4	4
ENSMUSG00000034732	9	16	5	0	9	3
ENSMUSG00000092490	13	26	11	0	0	0
ENSMUSG00000113373	0	0	0	0	0	0
ENSMUSG00000113372	0	0	0	0	0	0
ENSMUSG00000113375	0	0	0	0	0	0
ENSMUSG00000113374	0	0	0	0	0	0
ENSMUSG00000113377	1	0	0	0	0	0
ENSMUSG00000113376	0	0	0	0	0	0
ENSMUSG00000092499	0	0	0	0	0	0
ENSMUSG00000109602	0	2	6	0	0	0
ENSMUSG00000076129	0	0	0	0	0	0
ENSMUSG00000106505	0	0	0	0	0	0
ENSMUSG00000106501	0	0	0	0	0	0
ENSMUSG00000106502	0	0	0	0	0	0
ENSMUSG00000106503	0	0	0	0	0	0
ENSMUSG00000106509	0	0	0	0	2	0
ENSMUSG00000100386	0	0	0	0	0	0
ENSMUSG00000082543	5	10	5	5	5	4
ENSMUSG00000082540	0	0	0	0	0	0
ENSMUSG00000082547	0	0	0	0	0	0
ENSMUSG00000082546	0	0	0	0	0	0
ENSMUSG00000082545	0	0	0	2	0	0
ENSMUSG00000082544	0	0	0	0	0	0
ENSMUSG00000082548	0	0	0	0	0	0
ENSMUSG00000108404	0	0	0	0	0	0
ENSMUSG00000084262	0	2	0	0	0	0
ENSMUSG00000084267	0	0	0	0	0	0
ENSMUSG00000091468	0	0	0	0	0	0
ENSMUSG00000100389	5	11	3	46	32	22
ENSMUSG00000025202	18	0	10	3	7	17
ENSMUSG00000041272	15	30	9	9	32	9
ENSMUSG00000080949	0	0	0	0	0	0
ENSMUSG00000080948	2	0	1	0	1	3
ENSMUSG00000080944	0	0	0	0	0	0
ENSMUSG00000080947	0	0	0	0	0	0
ENSMUSG00000080943	0	0	0	0	0	0
ENSMUSG00000099146	0	3	0	0	0	0
ENSMUSG00000099149	0	0	0	0	0	0
ENSMUSG00000099148	0	0	0	0	0	0
ENSMUSG00000057179	0	1	0	0	0	0
ENSMUSG00000112082	0	4	0	0	0	0
ENSMUSG00000075524	0	0	0	0	0	0
ENSMUSG00000044170	5	0	0	0	0	0
ENSMUSG00000000706	0	0	0	0	0	0
ENSMUSG00000044172	37	18	30	0	5	2
ENSMUSG00000044176	0	0	0	0	0	0
ENSMUSG00000070173	0	0	0	0	0	0
ENSMUSG00000070175	0	0	0	0	0	0
ENSMUSG00000085139	14	24	4	0	3	0
ENSMUSG00000085135	4	8	2	0	0	0
ENSMUSG00000085130	0	0	0	0	0	0
ENSMUSG00000074695	0	0	0	0	0	0
ENSMUSG00000109425	0	0	0	0	0	0

ENSMUSG00000067522	0	0	0	0	0	2
ENSMUSG00000039329	0	0	0	0	0	0
ENSMUSG00000102304	0	0	0	0	0	0
ENSMUSG00000082458	0	0	0	0	0	0
ENSMUSG00000045225	1	1	1	1	1	3
ENSMUSG00000084030	0	0	0	0	0	0
ENSMUSG00000102303	0	0	0	0	0	0
ENSMUSG00000103815	0	0	0	0	0	0
ENSMUSG00000103814	0	0	0	0	0	0
ENSMUSG00000103817	0	0	0	0	0	0
ENSMUSG00000103816	0	0	0	0	0	0
ENSMUSG00000103811	7	5	5	5	2	11
ENSMUSG00000103810	0	0	0	0	0	0
ENSMUSG00000103813	0	0	0	0	0	0
ENSMUSG00000103812	0	0	0	0	0	0
ENSMUSG00000034057	33	37	34	29	52	42
ENSMUSG00000099652	0	0	0	0	0	0
ENSMUSG00000095845	0	0	0	6	0	0
ENSMUSG00000089934	0	0	0	1	0	0
ENSMUSG00000031497	108	130	85	95	84	77
ENSMUSG00000031492	0	0	0	0	0	0
ENSMUSG00000031491	0	0	0	0	0	0
ENSMUSG00000020912	0	0	0	0	0	0
ENSMUSG00000061082	0	0	0	0	0	0
ENSMUSG00000110406	0	0	0	0	0	0
ENSMUSG00000065505	1	1	0	0	0	0
ENSMUSG00000108843	1	0	0	0	0	0
ENSMUSG00000089667	0	0	0	0	0	0
ENSMUSG00000108908	0	0	0	0	0	0
ENSMUSG00000108909	3	2	0	5	3	2
ENSMUSG00000031727	0	0	0	0	0	0
ENSMUSG00000108902	0	0	0	3	3	4
ENSMUSG00000108903	0	0	0	0	0	0
ENSMUSG00000108900	0	0	0	0	0	0
ENSMUSG00000108906	0	0	0	0	0	0
ENSMUSG00000108904	3	0	0	0	0	0
ENSMUSG00000108905	0	1	1	1	1	1
ENSMUSG00000093860	0	0	0	0	0	0
ENSMUSG00000089664	0	0	0	0	0	3
ENSMUSG00000089663	17	42	25	30	30	25
ENSMUSG00000043314	1	0	0	0	0	0
ENSMUSG00000043310	2	0	0	0	0	0
ENSMUSG00000043312	0	4	0	0	0	0
ENSMUSG00000102384	0	0	0	0	0	0
ENSMUSG00000043319	0	0	0	0	0	0
ENSMUSG00000083324	0	0	0	0	0	0
ENSMUSG00000085909	0	0	0	0	0	0
ENSMUSG00000085905	0	0	0	0	0	0
ENSMUSG00000033383	0	0	0	0	0	0
ENSMUSG00000033386	218	200	257	163	161	173
ENSMUSG00000110403	13	14	12	9	13	9
ENSMUSG00000087219	0	0	0	0	0	0
ENSMUSG00000087210	0	0	0	0	0	0
ENSMUSG00000087211	0	0	0	0	0	0
ENSMUSG00000087214	0	0	0	0	0	0
ENSMUSG00000087215	0	0	0	0	0	0
ENSMUSG00000087216	0	0	0	0	0	0
ENSMUSG00000087217	0	0	0	0	0	0

ENSMUSG00000101505	2	0	0	0	0	0
ENSMUSG00000101504	0	0	0	0	0	0
ENSMUSG00000101507	0	0	0	0	0	0
ENSMUSG00000101506	0	0	0	0	0	0
ENSMUSG00000101501	14	7	7	0	0	3
ENSMUSG00000101500	0	0	0	0	0	0
ENSMUSG00000040663	52	36	32	0	1	29
ENSMUSG00000037761	233	247	240	208	214	283
ENSMUSG00000101508	0	0	0	0	0	0
ENSMUSG00000099765	0	0	0	0	0	0
ENSMUSG00000100674	0	0	0	0	0	0
ENSMUSG00000100675	0	0	0	0	0	0
ENSMUSG00000100676	0	0	0	0	0	0
ENSMUSG00000100677	0	0	0	0	0	0
ENSMUSG00000100670	0	0	0	0	0	0
ENSMUSG00000100671	0	0	0	0	0	0
ENSMUSG00000100673	0	0	0	0	0	0
ENSMUSG00000100678	0	0	0	0	0	0
ENSMUSG00000059901	28	40	15	32	27	36
ENSMUSG00000112473	0	0	0	0	0	0
ENSMUSG00000107908	0	0	0	0	0	0
ENSMUSG00000107907	0	0	0	0	0	3
ENSMUSG00000107906	3	2	4	6	2	4
ENSMUSG00000107905	0	0	0	0	0	0
ENSMUSG00000059908	0	3	0	0	0	0
ENSMUSG00000107903	0	0	0	0	0	0
ENSMUSG00000107902	0	2	0	0	0	0
ENSMUSG00000107901	0	0	0	0	0	0
ENSMUSG00000107900	0	0	0	0	0	0
ENSMUSG00000084747	0	0	0	0	0	0
ENSMUSG00000084746	0	0	0	0	0	0
ENSMUSG00000089116	0	0	0	0	0	0
ENSMUSG00000084740	0	0	0	0	0	0
ENSMUSG00000089119	0	0	0	0	0	0
ENSMUSG00000089118	0	0	0	2	0	0
ENSMUSG00000084748	0	0	2	0	0	0
ENSMUSG00000016942	13	4	14	2	7	10
ENSMUSG00000022408	5	8	7	9	0	4
ENSMUSG00000095155	0	0	0	1	0	0
ENSMUSG00000018654	67	160	281	63	53	126
ENSMUSG00000018656	2	0	5	2	0	0
ENSMUSG00000020317	0	0	0	0	0	0
ENSMUSG00000020310	0	0	0	3	0	0
ENSMUSG00000090308	0	0	0	0	0	0
ENSMUSG00000081282	2	3	0	1	0	4
ENSMUSG00000112221	0	0	0	0	0	0
ENSMUSG00000112222	0	0	0	0	0	0
ENSMUSG00000112223	0	0	0	0	0	0
ENSMUSG00000112224	0	0	0	0	0	0
ENSMUSG00000112225	0	0	0	0	0	0
ENSMUSG00000112226	2	7	5	0	7	0
ENSMUSG00000112227	0	0	0	0	0	0
ENSMUSG00000112228	0	0	0	0	0	0
ENSMUSG00000102747	0	0	0	0	0	0
ENSMUSG00000102745	3	0	4	6	7	3
ENSMUSG00000102743	0	0	0	0	0	0
ENSMUSG00000102740	0	5	1	3	3	5
ENSMUSG00000102741	0	0	0	0	0	2

ENSMUSG00000099056	0	0	0	0	0	0
ENSMUSG00000062464	10	0	6	0	4	0
ENSMUSG00000052818	0	0	0	0	0	0
ENSMUSG00000052819	0	0	0	2	0	0
ENSMUSG00000036962	0	0	0	0	0	0
ENSMUSG00000015829	14	36	22	14	24	30
ENSMUSG00000112598	0	0	0	0	0	0
ENSMUSG00000114523	0	0	0	0	0	0
ENSMUSG00000112590	0	0	0	0	0	0
ENSMUSG00000114522	0	0	0	0	0	0
ENSMUSG00000112596	53	42	57	25	20	43
ENSMUSG00000112597	1	0	0	0	0	0
ENSMUSG00000029699	23	12	13	20	14	14
ENSMUSG00000105439	0	0	0	0	0	0
ENSMUSG00000105438	0	0	0	0	0	2
ENSMUSG00000105435	0	0	0	0	0	0
ENSMUSG00000105437	0	0	2	0	0	0
ENSMUSG00000105436	0	0	0	0	0	0
ENSMUSG00000029695	15	27	52	35	20	31
ENSMUSG00000029697	0	0	0	0	0	0
ENSMUSG00000113179	5	3	0	0	0	0
ENSMUSG00000038357	0	0	0	0	0	0
ENSMUSG00000113175	0	0	0	0	0	0
ENSMUSG00000038354	169	180	197	103	140	165
ENSMUSG00000113173	0	0	0	0	0	0
ENSMUSG00000038352	214	300	241	15	94	118
ENSMUSG00000113171	0	0	0	0	0	0
ENSMUSG00000020573	365	340	474	118	139	201
ENSMUSG00000042631	0	0	0	0	0	0
ENSMUSG00000016552	67	39	43	34	57	58
ENSMUSG00000021194	0	0	0	0	0	0
ENSMUSG00000039913	10	0	8	23	11	18
ENSMUSG00000024114	0	0	0	0	0	0
ENSMUSG00000024116	0	0	0	0	0	0
ENSMUSG00000034528	0	0	0	0	0	0
ENSMUSG00000103595	0	0	0	0	0	0
ENSMUSG00000103597	3	2	1	0	0	1
ENSMUSG00000103592	0	3	0	0	2	0
ENSMUSG00000103598	0	0	0	0	0	0
ENSMUSG00000102616	2	0	0	0	0	0
ENSMUSG00000112335	0	0	0	2	2	2
ENSMUSG00000094327	1	0	0	0	0	0
ENSMUSG00000055891	0	0	0	0	0	0
ENSMUSG00000051732	0	0	0	0	0	0
ENSMUSG00000055895	0	0	0	0	0	0
ENSMUSG00000114338	0	0	0	0	0	0
ENSMUSG00000114339	0	0	0	0	0	0
ENSMUSG00000105295	0	0	0	0	0	0
ENSMUSG00000105297	0	0	0	0	0	0
ENSMUSG00000105296	3	0	4	0	12	0
ENSMUSG00000096281	0	0	0	0	0	0
ENSMUSG00000105298	0	1	0	0	0	0
ENSMUSG00000096283	0	0	0	0	0	0
ENSMUSG00000114334	0	0	0	0	0	0
ENSMUSG00000114335	0	0	0	0	0	0
ENSMUSG00000114336	0	0	0	0	0	0
ENSMUSG00000096286	1	0	1	2	1	0
ENSMUSG00000081639	0	0	0	0	0	0

ENSMUSG00000041052	0	10	0	0	0	0
ENSMUSG00000097484	0	4	0	0	0	5
ENSMUSG00000097486	0	0	0	0	0	0
ENSMUSG00000108249	0	0	0	0	2	0
ENSMUSG00000097481	6	8	5	0	0	0
ENSMUSG00000097482	14	15	10	11	5	2
ENSMUSG00000097483	0	0	0	0	0	0
ENSMUSG00000104744	0	0	0	0	0	0
ENSMUSG00000108244	0	0	0	0	0	0
ENSMUSG00000108247	0	0	0	0	0	0
ENSMUSG00000104747	0	0	0	0	0	0
ENSMUSG00000108241	0	0	0	0	0	0
ENSMUSG00000108240	0	0	0	3	0	0
ENSMUSG00000104742	0	0	0	0	0	0
ENSMUSG00000108242	0	0	0	0	0	0
ENSMUSG00000091888	0	0	0	0	0	0
ENSMUSG00000091887	0	0	0	0	0	0
ENSMUSG00000100332	0	0	0	0	0	0
ENSMUSG00000083330	0	1	0	0	0	2
ENSMUSG00000002897	351	374	451	144	163	178
ENSMUSG00000097738	3	0	0	0	0	0
ENSMUSG00000097739	0	0	0	0	0	0
ENSMUSG00000097736	0	0	0	0	0	0
ENSMUSG00000097735	0	0	0	0	0	0
ENSMUSG00000050066	0	0	2	0	0	0
ENSMUSG00000050063	0	0	0	0	0	0
ENSMUSG00000091771	0	0	0	0	0	0
ENSMUSG00000091775	0	0	0	0	2	0
ENSMUSG00000091776	1	0	1	1	1	0
ENSMUSG00000083969	0	0	0	0	0	2
ENSMUSG00000083963	0	0	0	0	0	0
ENSMUSG00000083960	0	0	0	0	0	0
ENSMUSG00000083961	2	2	2	3	4	2
ENSMUSG00000083966	11	18	14	6	7	7
ENSMUSG00000083967	0	0	0	0	0	0
ENSMUSG00000114592	0	0	0	0	0	0
ENSMUSG00000057499	3	10	1	5	2	3
ENSMUSG00000058656	66	58	47	38	47	74
ENSMUSG00000044375	6	0	3	0	3	4
ENSMUSG00000054763	11	0	22	6	7	15
ENSMUSG00000054764	0	0	0	0	0	0
ENSMUSG00000058659	0	0	0	0	0	0
ENSMUSG00000044378	0	0	0	0	0	0
ENSMUSG00000043668	82	89	83	41	60	37
ENSMUSG00000046203	0	0	0	0	0	0
ENSMUSG00000046204	0	6	5	0	4	8
ENSMUSG00000086128	0	0	0	0	0	0
ENSMUSG00000056728	0	0	0	0	0	0
ENSMUSG00000010044	0	0	0	0	0	0
ENSMUSG00000110521	4	21	12	9	0	16
ENSMUSG00000110520	0	0	0	0	0	0
ENSMUSG00000110522	0	0	0	0	0	0
ENSMUSG00000110524	0	5	0	2	6	4
ENSMUSG00000110527	0	0	0	0	0	0
ENSMUSG00000110526	0	0	0	0	0	0
ENSMUSG00000080430	0	0	0	0	0	0
ENSMUSG00000080434	0	0	0	0	0	0
ENSMUSG00000080437	0	0	0	0	0	0

ENSMUSG00000095259	0	0	0	1	0	0
ENSMUSG00000095257	0	0	0	0	0	0
ENSMUSG00000095256	0	0	0	0	0	0
ENSMUSG00000095255	0	0	0	0	0	0
ENSMUSG00000095251	0	0	0	0	0	0
ENSMUSG00000095250	3	2	0	2	0	2
ENSMUSG00000113951	0	0	0	0	0	0
ENSMUSG00000113953	0	0	0	0	0	0
ENSMUSG00000113952	0	0	0	0	2	0
ENSMUSG00000113955	0	0	0	0	0	0
ENSMUSG00000113954	0	0	0	0	0	0
ENSMUSG00000113957	0	0	0	0	0	0
ENSMUSG00000113956	0	0	0	0	0	0
ENSMUSG00000113959	50	44	49	16	15	39
ENSMUSG00000026840	46	31	50	11	16	21
ENSMUSG00000080732	0	0	0	0	0	0
ENSMUSG00000098561	0	0	0	0	0	0
ENSMUSG00000098565	0	0	0	1	0	0
ENSMUSG00000026669	40	26	25	12	21	31
ENSMUSG00000094618	2	10	13	12	5	15
ENSMUSG00000094610	0	0	0	0	0	0
ENSMUSG00000094612	0	0	0	0	0	0
ENSMUSG00000094613	0	0	0	10	0	0
ENSMUSG00000042002	0	0	0	0	4	0
ENSMUSG00000094616	0	0	0	0	0	0
ENSMUSG00000086870	0	0	0	0	0	0
ENSMUSG00000086875	0	0	0	0	0	0
ENSMUSG00000084806	11	8	15	0	0	0
ENSMUSG00000081470	4	0	0	3	3	0
ENSMUSG00000081477	0	0	0	0	0	0
ENSMUSG00000081474	0	0	0	0	0	0
ENSMUSG00000081475	0	0	0	0	0	0
ENSMUSG00000081478	0	0	0	0	0	0
ENSMUSG00000088307	0	0	0	0	0	0
ENSMUSG00000090919	67	66	50	38	29	25
ENSMUSG00000092515	0	0	0	0	0	0
ENSMUSG00000092514	0	0	0	0	0	0
ENSMUSG00000099761	0	0	0	0	0	0
ENSMUSG00000077598	0	0	0	0	0	0
ENSMUSG00000077599	0	0	0	0	0	0
ENSMUSG00000092513	0	0	0	0	0	0
ENSMUSG00000092512	1	0	0	0	1	0
ENSMUSG00000077594	0	0	0	0	0	0
ENSMUSG00000077595	1	2	0	2	4	0
ENSMUSG00000099768	0	0	3	0	0	0
ENSMUSG00000092519	0	0	2	2	0	0
ENSMUSG00000092518	0	0	0	0	0	0
ENSMUSG00000055403	0	0	0	0	0	0
ENSMUSG00000055408	4	0	0	0	2	0
ENSMUSG00000055409	1	0	0	2	0	1
ENSMUSG00000086296	10	13	5	16	11	23
ENSMUSG00000086291	39	10	50	5	12	9
ENSMUSG00000030031	129	186	100	96	121	189
ENSMUSG00000072419	0	0	0	0	0	0
ENSMUSG00000094323	0	0	0	0	0	0
ENSMUSG00000022542	0	0	0	0	0	0
ENSMUSG00000086124	0	0	0	0	0	0
ENSMUSG00000086127	0	0	0	0	0	0

ENSMUSG00000086126	0	0	0	0	0	0
ENSMUSG00000043664	8	8	20	0	8	4
ENSMUSG00000043661	18	18	15	10	11	20
ENSMUSG00000098685	0	0	0	0	0	0
ENSMUSG00000000003	0	0	0	0	0	0
ENSMUSG00000098683	0	0	0	0	0	0
ENSMUSG00000076241	0	0	1	0	0	1
ENSMUSG00000084184	0	0	0	0	0	0
ENSMUSG00000113064	0	0	0	0	0	0
ENSMUSG00000087457	0	3	0	0	0	0
ENSMUSG00000087454	0	0	0	0	0	0
ENSMUSG00000087455	0	0	0	0	0	0
ENSMUSG00000087452	0	0	0	0	0	0
ENSMUSG00000087450	0	0	0	0	0	0
ENSMUSG00000070815	0	0	0	0	0	0
ENSMUSG00000070816	0	0	0	0	0	0
ENSMUSG00000070817	0	0	0	0	0	0
ENSMUSG00000070810	0	0	0	0	0	0
ENSMUSG00000070811	0	0	0	0	0	0
ENSMUSG00000087459	0	0	0	0	0	0
ENSMUSG00000037568	43	36	23	33	20	25
ENSMUSG00000083513	0	0	0	0	0	0
ENSMUSG00000083510	0	0	0	0	0	0
ENSMUSG00000083517	0	0	0	0	0	0
ENSMUSG00000045842	4	7	12	7	7	9
ENSMUSG00000083515	0	0	0	0	0	0
ENSMUSG00000083514	0	0	0	0	0	0
ENSMUSG00000049173	0	0	0	0	0	0
ENSMUSG00000083519	0	0	2	0	0	0
ENSMUSG00000083518	0	0	0	0	3	4
ENSMUSG00000049176	0	0	0	2	0	0
ENSMUSG00000088830	0	0	0	0	0	0
ENSMUSG00000079439	0	0	0	0	0	0
ENSMUSG00000079438	0	0	0	0	0	0
ENSMUSG00000105978	0	0	0	0	0	0
ENSMUSG00000079433	0	0	0	0	0	0
ENSMUSG00000079432	0	0	0	0	0	0
ENSMUSG00000087981	0	0	0	0	0	0
ENSMUSG00000087982	1	1	1	1	1	1
ENSMUSG00000087983	2	0	0	2	2	0
ENSMUSG00000087985	0	0	0	0	0	0
ENSMUSG00000040314	0	0	0	0	0	0
ENSMUSG00000088839	0	0	0	0	0	0
ENSMUSG00000040312	151	165	168	78	77	137
ENSMUSG00000071149	0	0	0	0	0	0
ENSMUSG00000084185	0	0	0	0	0	0
ENSMUSG00000040456	0	2	2	0	0	0
ENSMUSG00000084189	0	0	0	0	0	0
ENSMUSG00000033717	0	0	3	3	0	1
ENSMUSG00000103154	0	0	0	0	0	0
ENSMUSG00000103155	1	1	0	1	0	0
ENSMUSG00000103156	0	6	4	4	7	6
ENSMUSG00000081893	0	0	0	0	0	0
ENSMUSG00000103150	0	0	0	0	0	0
ENSMUSG00000101070	0	0	0	0	0	0
ENSMUSG00000112358	22	32	22	19	34	32
ENSMUSG00000103153	0	0	0	0	0	0
ENSMUSG00000079299	0	0	0	0	0	0

ENSMUSG00000079298	15	5	28	5	15	0
ENSMUSG00000101074	0	0	0	0	0	0
ENSMUSG00000085588	0	0	0	0	0	0
ENSMUSG00000109593	0	0	0	0	0	0
ENSMUSG00000085582	0	0	0	0	0	0
ENSMUSG00000085580	0	0	0	0	0	0
ENSMUSG00000085584	13	20	8	8	4	14
ENSMUSG00000085585	0	0	0	0	0	0
ENSMUSG00000074248	0	0	0	0	0	0
ENSMUSG00000104006	0	0	0	0	0	0
ENSMUSG00000078746	20	0	0	0	3	11
ENSMUSG00000074240	15	33	17	46	31	35
ENSMUSG00000078742	0	0	0	0	0	0
ENSMUSG00000029299	44	25	42	32	21	44
ENSMUSG00000081807	0	0	0	0	0	0
ENSMUSG00000081804	0	0	0	0	0	0
ENSMUSG00000065122	0	0	0	0	0	0
ENSMUSG00000032048	0	0	0	0	0	0
ENSMUSG00000071147	0	0	0	0	0	0
ENSMUSG00000073739	0	0	0	0	0	0
ENSMUSG00000073735	0	0	0	0	0	0
ENSMUSG00000066058	8	0	0	6	2	0
ENSMUSG00000073730	2	0	0	2	0	2
ENSMUSG00000099949	0	0	0	0	0	0
ENSMUSG00000073733	9	28	20	17	14	28
ENSMUSG00000106614	0	0	0	0	0	0
ENSMUSG00000099948	0	0	0	0	0	0
ENSMUSG00000106615	0	0	0	0	0	0
ENSMUSG00000106610	3	2	2	2	1	2
ENSMUSG00000106611	2	0	0	0	0	0
ENSMUSG00000099943	0	0	0	0	0	0
ENSMUSG00000099942	0	0	0	0	0	0
ENSMUSG00000106613	0	0	0	0	0	0
ENSMUSG00000111008	0	0	0	0	0	0
ENSMUSG00000098001	20	12	8	11	4	16
ENSMUSG00000098003	0	0	0	0	0	0
ENSMUSG00000098002	4	3	2	0	2	3
ENSMUSG00000098005	0	0	2	0	0	0
ENSMUSG00000098007	0	0	0	0	0	0
ENSMUSG00000098006	8	8	7	4	2	11
ENSMUSG00000098008	0	0	0	0	0	0
ENSMUSG00000084182	0	0	0	0	0	0
ENSMUSG00000077925	3	1	0	0	0	0
ENSMUSG00000063206	0	0	0	0	0	0
ENSMUSG00000034435	10	5	5	11	8	15
ENSMUSG00000084188	0	0	0	0	0	0
ENSMUSG00000109687	0	0	0	0	0	0
ENSMUSG00000113893	0	0	0	0	0	0
ENSMUSG00000031026	2	17	20	4	30	21
ENSMUSG00000031443	6	15	9	10	12	17
ENSMUSG00000104613	0	0	0	0	0	0
ENSMUSG00000084971	0	0	0	0	0	0
ENSMUSG00000038883	0	0	0	0	0	0
ENSMUSG00000081034	3	5	6	2	5	3
ENSMUSG00000091573	0	0	0	0	0	0
ENSMUSG00000030373	0	0	0	0	0	0
ENSMUSG00000030376	27	28	28	18	26	14
ENSMUSG00000109680	0	0	6	0	4	5

ENSMUSG00000030378	0	0	0	0	0	0
ENSMUSG00000020901	76	121	236	17	56	57
ENSMUSG00000020905	73	72	78	71	96	110
ENSMUSG00000020904	19	10	12	8	17	19
ENSMUSG00000063905	0	0	0	0	0	0
ENSMUSG00000067438	0	0	0	0	0	0
ENSMUSG00000104942	0	0	0	0	0	0
ENSMUSG00000101715	0	0	0	0	0	0
ENSMUSG00000104941	0	0	0	0	0	0
ENSMUSG00000101710	0	0	0	0	0	0
ENSMUSG00000104944	0	0	0	0	0	0
ENSMUSG00000101713	0	0	0	0	0	0
ENSMUSG00000056293	0	0	0	0	0	0
ENSMUSG00000104948	0	0	0	0	0	0
ENSMUSG00000104949	0	0	0	0	0	0
ENSMUSG00000056296	0	0	0	0	0	0
ENSMUSG00000101719	0	0	0	0	0	0
ENSMUSG00000104243	0	0	0	0	0	0
ENSMUSG00000061875	0	0	0	0	0	0
ENSMUSG00000061877	0	0	0	0	0	2
ENSMUSG00000061878	76	75	141	33	25	33
ENSMUSG00000088474	0	5	3	2	2	0
ENSMUSG00000088477	0	0	0	0	0	0
ENSMUSG00000088476	0	0	0	0	0	0
ENSMUSG00000088471	0	0	0	0	0	0
ENSMUSG00000088470	0	0	0	0	0	0
ENSMUSG00000088473	0	0	0	0	0	0
ENSMUSG00000088472	0	0	0	0	0	0
ENSMUSG00000058260	1	9	10	3	1	16
ENSMUSG00000082252	0	0	0	0	0	0
ENSMUSG00000082251	0	0	0	0	0	0
ENSMUSG00000064310	0	0	0	0	0	4
ENSMUSG00000091151	0	0	0	0	0	0
ENSMUSG00000026994	14	5	12	3	0	6
ENSMUSG00000099798	0	0	2	2	0	0
ENSMUSG00000078253	0	0	0	0	3	0
ENSMUSG00000107716	0	0	0	0	0	0
ENSMUSG00000107710	0	0	0	0	0	0
ENSMUSG00000107711	0	0	0	0	0	0
ENSMUSG00000111940	7	4	4	5	7	0
ENSMUSG00000111941	0	0	0	0	0	0
ENSMUSG00000111943	9	11	12	7	14	5
ENSMUSG00000111945	0	0	0	0	3	0
ENSMUSG00000036027	0	0	4	0	0	6
ENSMUSG00000111949	0	0	0	0	0	0
ENSMUSG00000103625	7	15	6	6	11	3
ENSMUSG00000036022	134	81	139	67	122	91
ENSMUSG00000094052	0	0	0	0	0	0
ENSMUSG00000094053	0	0	0	0	0	0
ENSMUSG00000064862	4	0	0	0	0	0
ENSMUSG00000094059	0	0	0	0	0	0
ENSMUSG00000095989	1	0	1	1	1	0
ENSMUSG00000095988	0	0	0	0	0	0
ENSMUSG00000089700	0	0	0	0	0	0
ENSMUSG00000089703	5	0	0	0	2	0
ENSMUSG00000095982	0	4	8	9	2	3
ENSMUSG00000095980	0	0	0	0	0	0
ENSMUSG00000095987	0	0	0	0	0	3

ENSMUSG00000020295	0	0	6	0	0	0
ENSMUSG00000095984	0	0	0	0	0	0
ENSMUSG00000020125	0	0	0	0	0	0
ENSMUSG00000020121	207	193	242	87	149	144
ENSMUSG00000078117	0	8	0	3	0	4
ENSMUSG00000020123	36	42	20	54	59	64
ENSMUSG00000028730	0	0	0	0	0	0
ENSMUSG00000106394	0	0	0	0	0	0
ENSMUSG00000106397	0	0	0	0	0	0
ENSMUSG00000106396	0	7	0	3	6	5
ENSMUSG00000028736	0	0	0	0	0	0
ENSMUSG00000101642	0	0	0	0	0	0
ENSMUSG00000112419	6	4	4	6	6	6
ENSMUSG00000112418	4	3	0	0	0	0
ENSMUSG00000112416	0	0	0	0	0	0
ENSMUSG00000035594	0	0	0	0	0	0
ENSMUSG00000112414	0	0	0	0	0	0
ENSMUSG00000035592	0	0	0	0	0	0
ENSMUSG00000112412	0	0	0	0	0	0
ENSMUSG00000112410	6	12	10	8	6	6
ENSMUSG00000087860	0	0	0	0	0	0
ENSMUSG00000110301	0	0	0	0	0	0
ENSMUSG00000110300	0	0	0	0	0	0
ENSMUSG00000110305	0	0	0	0	0	0
ENSMUSG00000110304	0	0	2	0	2	7
ENSMUSG00000110307	0	0	0	0	0	0
ENSMUSG00000110306	0	0	0	0	0	0
ENSMUSG00000092060	317	260	385	170	143	175
ENSMUSG00000035041	3	0	7	3	3	5
ENSMUSG00000055177	0	0	0	0	0	0
ENSMUSG00000114818	0	0	0	0	0	0
ENSMUSG00000109011	0	0	0	0	0	0
ENSMUSG00000114811	0	0	0	0	0	0
ENSMUSG00000114810	0	0	0	0	0	0
ENSMUSG00000109019	0	0	0	0	0	0
ENSMUSG00000109018	3	6	3	3	3	6
ENSMUSG00000114817	0	5	7	0	0	5
ENSMUSG00000114816	0	0	0	0	0	0
ENSMUSG00000106971	0	0	0	0	0	0
ENSMUSG00000106970	0	0	0	0	0	0
ENSMUSG00000106975	0	0	0	1	0	0
ENSMUSG00000106974	0	0	0	0	0	0
ENSMUSG00000113452	0	0	0	0	0	0
ENSMUSG00000113453	0	0	0	0	0	0
ENSMUSG00000113455	0	0	0	0	0	0
ENSMUSG00000113457	0	0	0	0	0	0
ENSMUSG00000099025	0	8	8	0	0	8
ENSMUSG00000085869	0	0	0	0	0	0
ENSMUSG00000096365	0	0	0	0	0	0
ENSMUSG00000096362	1	2	1	0	2	0
ENSMUSG00000096360	0	0	0	0	0	0
ENSMUSG00000082915	0	0	0	0	0	0
ENSMUSG00000082918	0	0	0	0	0	0
ENSMUSG00000082919	0	0	0	0	0	0
ENSMUSG00000005474	0	0	0	0	0	0
ENSMUSG00000005470	61	49	45	36	31	74
ENSMUSG00000077312	0	0	0	0	0	0
ENSMUSG00000111162	0	0	4	0	6	5

ENSMUSG00000109900	0	0	0	0	0	0
ENSMUSG00000111161	2	2	1	0	0	1
ENSMUSG00000026567	8	9	2	12	7	8
ENSMUSG00000111167	0	0	5	0	0	0
ENSMUSG00000109907	0	0	0	0	0	0
ENSMUSG00000093854	0	0	0	0	0	0
ENSMUSG00000114105	0	0	0	0	0	0
ENSMUSG00000114103	2	6	4	6	4	8
ENSMUSG00000114101	0	0	0	0	0	0
ENSMUSG00000089528	0	0	0	0	0	0
ENSMUSG00000104539	0	0	0	0	0	0
ENSMUSG00000104538	0	0	0	0	0	0
ENSMUSG00000104534	0	0	0	0	0	0
ENSMUSG00000089529	0	0	0	0	0	0
ENSMUSG00000039492	4	4	0	0	0	0
ENSMUSG00000004613	0	0	0	0	0	0
ENSMUSG00000081806	0	0	0	0	0	0
ENSMUSG00000056885	0	0	0	0	0	0
ENSMUSG00000056883	0	0	0	0	0	0
ENSMUSG00000026387	0	0	0	0	0	0
ENSMUSG00000089524	0	0	0	0	0	0
ENSMUSG00000026383	369	508	376	567	634	699
ENSMUSG00000026380	82	92	30	57	51	73
ENSMUSG00000110488	0	0	0	0	0	0
ENSMUSG00000055072	0	1	3	0	3	2
ENSMUSG00000110483	0	0	0	0	0	0
ENSMUSG00000110481	24	53	52	0	43	26
ENSMUSG00000045672	686	412	472	233	195	238
ENSMUSG00000095049	0	0	0	0	0	0
ENSMUSG00000095047	0	0	0	0	0	0
ENSMUSG00000095044	0	0	0	0	0	0
ENSMUSG00000095045	0	1	0	0	0	0
ENSMUSG00000090030	0	0	0	0	0	0
ENSMUSG00000095040	5	15	6	3	0	13
ENSMUSG00000053101	26	27	27	5	4	7
ENSMUSG00000109992	0	0	0	0	0	0
ENSMUSG00000023802	0	0	0	0	0	0
ENSMUSG00000042909	0	0	0	0	0	0
ENSMUSG00000092894	0	0	0	0	0	0
ENSMUSG00000092896	1	0	0	0	0	0
ENSMUSG00000027380	0	0	0	0	3	3
ENSMUSG00000092891	0	0	0	0	0	0
ENSMUSG00000089520	0	0	0	0	0	0
ENSMUSG00000094444	0	0	0	0	0	0
ENSMUSG00000081392	0	0	0	0	0	0
ENSMUSG00000081391	0	0	0	0	0	0
ENSMUSG00000081390	0	0	0	0	0	2
ENSMUSG00000081397	0	2	0	2	0	0
ENSMUSG00000081396	0	0	3	0	9	4
ENSMUSG00000098249	0	0	0	0	0	0
ENSMUSG00000098246	0	0	0	0	0	0
ENSMUSG00000098245	0	0	0	0	0	0
ENSMUSG00000098244	0	0	0	0	0	0
ENSMUSG00000098243	36	39	23	22	19	36
ENSMUSG00000098242	0	0	2	0	0	0
ENSMUSG00000098241	0	0	0	0	0	0
ENSMUSG00000094440	0	0	0	0	0	0
ENSMUSG00000080668	0	0	0	0	0	0

ENSMUSG00000099103	0	0	0	0	0	0
ENSMUSG00000080663	0	0	0	0	0	0
ENSMUSG00000095817	0	0	0	0	0	0
ENSMUSG00000080666	0	0	0	0	0	0
ENSMUSG00000089522	0	0	0	0	0	0
ENSMUSG00000078942	136	135	225	79	83	122
ENSMUSG00000086620	0	0	0	0	0	0
ENSMUSG00000086621	0	0	0	0	0	0
ENSMUSG00000086624	0	0	0	0	0	0
ENSMUSG00000086628	0	0	0	0	0	2
ENSMUSG00000078949	0	0	0	0	0	0
ENSMUSG00000095911	0	0	0	0	0	0
ENSMUSG00000098797	2	0	0	0	0	0
ENSMUSG00000098796	0	0	1	3	0	0
ENSMUSG00000031220	4	4	3	3	3	4
ENSMUSG00000031224	0	0	0	0	0	2
ENSMUSG00000106689	0	0	0	0	0	0
ENSMUSG00000047749	207	158	212	84	93	120
ENSMUSG00000113501	2	1	0	2	2	1
ENSMUSG00000093070	0	1	0	1	0	0
ENSMUSG00000093073	0	0	0	0	0	0
ENSMUSG00000093079	0	0	0	0	0	0
ENSMUSG00000049811	127	125	92	62	50	57
ENSMUSG00000052760	105	80	159	42	72	77
ENSMUSG00000081537	0	0	0	0	0	0
ENSMUSG00000081536	0	0	0	0	0	0
ENSMUSG00000081533	0	0	0	0	0	0
ENSMUSG00000081531	0	0	0	0	0	0
ENSMUSG00000039533	0	0	0	0	0	4
ENSMUSG00000031594	0	9	21	18	18	13
ENSMUSG00000081538	0	0	0	0	0	0
ENSMUSG00000077411	0	0	0	0	0	0
ENSMUSG00000077410	0	0	0	0	0	0
ENSMUSG00000077413	0	0	0	0	0	0
ENSMUSG00000073971	2	0	0	0	0	0
ENSMUSG00000073976	0	0	0	0	0	0
ENSMUSG00000073977	0	3	0	0	0	0
ENSMUSG00000077417	0	0	0	0	0	0
ENSMUSG00000073975	8	4	11	2	4	5
ENSMUSG00000077418	0	0	0	0	0	0
ENSMUSG00000073978	0	2	0	0	0	0
ENSMUSG00000073979	0	0	0	0	0	0
ENSMUSG00000040432	5	5	8	6	5	8
ENSMUSG00000105285	0	0	0	0	0	0
ENSMUSG00000099378	0	0	0	0	0	0
ENSMUSG00000089406	1	0	0	0	0	0
ENSMUSG00000030173	0	0	0	0	0	0
ENSMUSG00000099688	0	0	0	0	0	0
ENSMUSG00000048142	36	50	58	41	50	98
ENSMUSG00000048148	23	18	14	20	29	28
ENSMUSG00000062556	0	0	0	0	0	0
ENSMUSG00000099683	0	0	0	0	0	0
ENSMUSG00000099684	0	0	0	0	0	0
ENSMUSG00000099685	0	0	0	0	0	0
ENSMUSG00000099686	3	0	0	6	5	7
ENSMUSG00000099687	0	0	0	0	0	0
ENSMUSG00000099532	0	0	0	0	0	0
ENSMUSG00000092305	8	4	7	3	7	6

ENSMUSG00000092306	0	0	0	0	0	0
ENSMUSG00000092307	0	0	0	0	0	0
ENSMUSG00000099536	0	0	0	0	0	0
ENSMUSG00000099537	0	0	0	0	0	0
ENSMUSG00000099534	1	1	2	0	4	0
ENSMUSG00000092303	0	0	0	0	0	0
ENSMUSG00000099539	0	0	0	0	0	0
ENSMUSG00000092308	0	0	0	0	0	0
ENSMUSG00000092309	23	19	14	11	9	10
ENSMUSG00000101643	0	2	0	0	0	0
ENSMUSG00000046591	28	21	19	4	9	14
ENSMUSG00000046593	4	0	10	0	0	0
ENSMUSG00000072595	0	0	0	0	0	0
ENSMUSG00000072591	0	0	0	0	0	0
ENSMUSG00000072599	0	0	0	0	0	0
ENSMUSG00000072598	0	0	0	0	0	0
ENSMUSG00000070990	0	0	0	0	0	3
ENSMUSG00000073737	63	83	83	67	67	99
ENSMUSG00000070999	2	0	4	0	2	4
ENSMUSG00000037334	0	0	0	0	0	0
ENSMUSG00000037335	0	0	0	0	0	0
ENSMUSG00000037337	28	39	60	15	15	27
ENSMUSG00000102446	0	0	0	0	0	0
ENSMUSG00000113436	0	0	0	0	0	0
ENSMUSG00000100024	0	0	0	0	0	0
ENSMUSG00000100027	0	0	0	0	0	0
ENSMUSG00000100021	0	0	0	0	0	0
ENSMUSG00000089401	0	0	0	0	0	0
ENSMUSG00000100023	0	0	0	0	0	0
ENSMUSG00000100022	0	0	0	0	4	0
ENSMUSG00000070661	0	2	0	0	0	0
ENSMUSG00000064156	0	0	0	0	2	0
ENSMUSG00000102137	0	0	0	0	0	0
ENSMUSG00000083701	0	0	0	0	0	0
ENSMUSG00000083706	0	0	0	0	0	0
ENSMUSG00000083707	0	0	0	0	0	2
ENSMUSG00000084576	0	0	0	0	0	0
ENSMUSG00000084579	0	0	0	0	0	0
ENSMUSG00000083709	0	1	0	0	3	0
ENSMUSG00000068819	4	0	0	0	2	0
ENSMUSG00000075012	30	26	38	22	12	34
ENSMUSG00000075100	0	0	0	0	0	0
ENSMUSG00000068814	0	0	0	0	0	0
ENSMUSG00000068817	2	0	0	2	2	0
ENSMUSG00000068816	0	0	0	0	0	0
ENSMUSG00000085620	0	0	0	0	0	0
ENSMUSG00000054051	760	758	790	569	680	793
ENSMUSG00000107555	0	0	0	0	0	0
ENSMUSG00000107557	0	0	0	0	0	0
ENSMUSG00000107550	0	0	0	0	0	0
ENSMUSG00000107551	0	0	0	0	0	0
ENSMUSG00000107552	0	0	0	0	0	0
ENSMUSG00000050505	18	17	7	10	22	16
ENSMUSG00000065689	0	0	0	0	0	0
ENSMUSG00000065685	0	0	0	0	0	0
ENSMUSG00000065684	0	0	0	0	0	0
ENSMUSG00000101866	0	0	0	0	0	0
ENSMUSG00000101864	0	0	0	0	0	0

ENSMUSG00000101862	0	0	2	0	0	0
ENSMUSG00000101863	0	0	0	0	0	0
ENSMUSG00000101860	4	9	0	10	6	4
ENSMUSG00000101861	0	0	0	0	0	0
ENSMUSG00000101868	0	0	0	0	0	0
ENSMUSG00000101869	0	0	0	0	0	0
ENSMUSG00000064486	0	0	0	0	0	0
ENSMUSG00000064487	1	1	0	0	0	0
ENSMUSG00000064485	0	0	0	0	0	0
ENSMUSG00000087917	0	0	0	0	0	0
ENSMUSG00000064489	0	5	2	2	0	1
ENSMUSG00000084394	0	0	0	0	0	0
ENSMUSG00000107087	0	0	0	0	0	0
ENSMUSG00000084396	0	0	0	0	0	0
ENSMUSG00000084397	0	0	0	0	0	0
ENSMUSG00000084391	0	0	0	0	0	0
ENSMUSG00000084393	0	0	0	0	0	0
ENSMUSG00000033520	6	7	6	0	0	7
ENSMUSG00000107089	3	5	0	7	5	2
ENSMUSG00000103709	0	1	0	0	0	3
ENSMUSG00000103708	0	0	0	0	0	0
ENSMUSG00000075906	0	0	0	0	0	0
ENSMUSG00000103705	0	0	0	0	0	0
ENSMUSG00000103704	0	0	0	0	0	0
ENSMUSG00000103700	0	0	0	0	0	0
ENSMUSG00000103703	63	74	41	67	68	129
ENSMUSG00000103702	0	0	0	0	0	0
ENSMUSG00000068105	0	4	6	0	6	4
ENSMUSG00000068101	16	9	23	11	10	9
ENSMUSG00000078531	0	0	3	0	0	0
ENSMUSG00000078537	0	0	0	0	0	0
ENSMUSG00000030954	0	0	0	0	0	0
ENSMUSG00000089625	0	0	0	0	0	0
ENSMUSG00000097215	0	0	0	0	0	0
ENSMUSG00000097214	5	0	4	0	0	0
ENSMUSG00000063900	0	0	0	0	0	0
ENSMUSG00000035200	0	0	0	0	0	0
ENSMUSG00000035201	0	0	0	0	0	0
ENSMUSG00000024386	0	0	0	0	0	0
ENSMUSG00000062365	2	0	2	7	2	0
ENSMUSG00000062369	0	0	0	0	0	0
ENSMUSG00000048806	0	0	0	0	0	0
ENSMUSG00000097932	0	0	0	0	0	0
ENSMUSG00000097933	0	0	2	4	5	0
ENSMUSG00000069475	1	0	0	0	0	0
ENSMUSG00000069476	0	0	0	0	0	0
ENSMUSG00000102079	0	0	0	0	0	0
ENSMUSG00000102074	0	0	0	0	0	0
ENSMUSG00000102075	1	3	0	6	0	5
ENSMUSG00000102077	0	0	0	0	0	0
ENSMUSG00000102071	0	0	0	0	0	0
ENSMUSG00000102072	0	0	0	0	0	0
ENSMUSG00000102073	0	0	5	0	0	0
ENSMUSG00000096164	0	0	0	0	0	0
ENSMUSG00000096166	0	3	0	2	2	1
ENSMUSG00000096167	2	0	0	0	2	0
ENSMUSG00000096161	0	0	0	0	0	0
ENSMUSG00000058447	4	1	0	0	20	0

ENSMUSG00000108610	0	0	0	0	0	0
ENSMUSG00000108616	0	0	0	0	0	2
ENSMUSG00000098309	0	0	0	0	0	0
ENSMUSG00000105657	0	0	0	0	2	0
ENSMUSG00000063018	7	8	12	14	0	0
ENSMUSG00000108102	3	0	3	0	0	0
ENSMUSG00000105930	0	0	0	0	0	0
ENSMUSG00000105931	0	0	0	0	0	0
ENSMUSG00000105934	0	0	0	0	0	0
ENSMUSG00000104683	0	0	0	0	0	0
ENSMUSG00000105937	0	0	0	0	0	0
ENSMUSG00000023032	59	70	166	76	124	104
ENSMUSG00000023033	2	4	0	4	0	0
ENSMUSG00000108106	0	0	0	0	0	0
ENSMUSG00000098300	0	0	0	0	0	0
ENSMUSG00000066990	0	0	0	0	0	0
ENSMUSG00000113807	0	0	0	0	0	0
ENSMUSG00000101035	0	0	0	0	0	0
ENSMUSG00000101036	0	0	0	0	0	0
ENSMUSG00000101031	0	0	0	0	0	0
ENSMUSG00000104046	1	3	8	3	0	0
ENSMUSG00000104045	0	0	0	0	0	0
ENSMUSG00000101032	0	0	0	0	0	0
ENSMUSG00000104048	0	0	0	0	0	0
ENSMUSG00000101039	0	0	0	0	0	0
ENSMUSG00000101415	0	0	0	0	0	0
ENSMUSG00000059473	0	0	0	0	0	0
ENSMUSG00000091304	0	0	0	0	0	0
ENSMUSG00000091307	0	0	0	0	0	0
ENSMUSG00000101412	0	0	0	0	0	0
ENSMUSG00000111618	0	0	0	0	0	0
ENSMUSG00000111619	0	0	0	0	1	0
ENSMUSG00000101935	1	0	0	1	0	0
ENSMUSG00000056316	0	0	0	0	0	0
ENSMUSG00000111610	6	4	4	0	8	8
ENSMUSG00000055937	0	0	0	0	0	0
ENSMUSG00000021848	0	0	0	0	0	0
ENSMUSG00000100346	0	0	0	0	0	0
ENSMUSG00000100340	0	0	0	0	0	0
ENSMUSG00000100341	0	0	0	0	0	0
ENSMUSG00000100343	0	0	0	0	0	0
ENSMUSG00000111615	0	5	0	0	0	3
ENSMUSG00000110978	0	0	0	1	3	0
ENSMUSG00000113335	8	13	13	4	4	9
ENSMUSG00000054967	43	44	25	26	15	21
ENSMUSG00000113336	0	0	0	0	0	0
ENSMUSG00000103300	0	0	0	0	0	0
ENSMUSG00000031027	8	0	5	0	0	0
ENSMUSG00000103302	0	0	0	0	0	0
ENSMUSG00000113333	0	0	0	0	0	0
ENSMUSG00000113332	0	0	0	0	0	0
ENSMUSG00000103304	0	0	0	0	0	0
ENSMUSG00000086422	0	0	0	0	0	0
ENSMUSG00000086425	0	0	0	5	4	0
ENSMUSG00000086426	0	0	0	0	0	0
ENSMUSG00000086427	0	0	0	0	0	0
ENSMUSG00000086428	0	0	0	0	0	0
ENSMUSG00000095536	0	0	0	0	0	0

ENSMUSG00000095537	0	0	0	0	0	0
ENSMUSG00000095534	2	0	0	1	0	0
ENSMUSG00000095532	0	0	0	0	0	0
ENSMUSG00000095533	0	0	0	0	0	0
ENSMUSG00000095531	0	0	0	0	0	1
ENSMUSG00000113339	0	0	0	0	0	0
ENSMUSG00000077483	0	0	0	0	0	0
ENSMUSG00000111208	0	0	0	0	0	0
ENSMUSG00000027855	0	0	0	0	0	0
ENSMUSG00000093271	0	0	0	0	0	0
ENSMUSG00000093270	0	1	1	1	1	0
ENSMUSG00000103786	0	0	0	0	0	0
ENSMUSG00000093274	1	0	0	0	0	0
ENSMUSG00000086391	0	4	0	0	5	0
ENSMUSG00000086396	0	0	0	0	0	0
ENSMUSG00000052562	0	0	0	0	0	0
ENSMUSG00000052566	184	216	197	118	138	135
ENSMUSG00000097974	9	28	50	18	39	43
ENSMUSG00000097977	1	4	2	2	0	11
ENSMUSG00000097979	7	21	30	18	30	26
ENSMUSG00000004668	0	0	0	0	0	0
ENSMUSG00000112608	0	0	0	0	0	0
ENSMUSG00000112609	0	0	0	0	0	0
ENSMUSG00000112607	0	0	0	0	0	0
ENSMUSG00000112604	0	0	0	0	0	2
ENSMUSG00000055033	2	4	2	4	2	3
ENSMUSG00000004661	121	128	161	131	146	162
ENSMUSG00000112600	0	0	0	0	0	0
ENSMUSG00000055030	0	0	0	0	0	0
ENSMUSG00000096030	0	0	0	0	0	0
ENSMUSG00000081612	0	0	0	0	0	0
ENSMUSG00000081613	0	0	0	0	0	0
ENSMUSG00000032854	0	0	0	0	0	0
ENSMUSG00000051209	0	0	0	4	0	0
ENSMUSG00000071738	0	0	0	0	0	0
ENSMUSG00000065248	0	0	0	0	0	0
ENSMUSG00000051200	0	0	0	0	0	0
ENSMUSG00000051207	0	0	0	0	0	0
ENSMUSG00000093879	0	0	0	0	0	0
ENSMUSG00000027073	0	0	0	0	0	0
ENSMUSG00000109240	0	0	0	0	0	0
ENSMUSG00000106582	0	0	0	0	0	0
ENSMUSG00000109202	0	0	0	0	0	0
ENSMUSG00000114609	0	0	0	0	0	0
ENSMUSG00000105583	0	0	0	0	0	0
ENSMUSG00000105582	0	0	0	0	0	0
ENSMUSG00000109207	0	0	0	0	0	0
ENSMUSG00000109204	0	0	0	0	0	0
ENSMUSG00000105589	0	0	0	0	0	0
ENSMUSG00000105588	0	0	0	0	0	0
ENSMUSG00000109209	8	8	8	8	5	3
ENSMUSG00000114604	0	0	0	0	0	0
ENSMUSG00000114606	2	0	0	0	0	0
ENSMUSG00000114607	5	4	3	6	7	3
ENSMUSG00000077086	0	0	0	0	0	0
ENSMUSG00000110112	0	0	0	0	0	0
ENSMUSG00000110113	0	0	0	1	0	0
ENSMUSG00000110114	0	0	0	0	3	0

ENSMUSG00000110115	0	0	0	0	0	0
ENSMUSG00000110116	0	0	0	0	0	0
ENSMUSG00000110117	0	0	0	0	0	0
ENSMUSG00000082894	0	0	0	0	0	0
ENSMUSG00000082897	0	0	0	0	0	0
ENSMUSG00000082891	0	0	0	0	0	0
ENSMUSG00000082890	0	0	0	0	0	0
ENSMUSG00000082892	0	0	0	0	0	0
ENSMUSG00000070460	0	0	0	0	0	0
ENSMUSG00000025288	0	0	0	0	0	0
ENSMUSG00000054074	249	214	71	116	261	199
ENSMUSG00000070464	0	0	0	0	0	0
ENSMUSG00000108151	0	0	0	0	0	0
ENSMUSG00000108150	2	1	1	0	0	0
ENSMUSG00000108153	0	0	0	0	0	0
ENSMUSG00000050553	0	0	0	3	0	0
ENSMUSG00000108157	0	0	0	0	0	0
ENSMUSG00000005202	0	0	0	0	0	0
ENSMUSG00000050558	10	5	15	0	7	6
ENSMUSG00000105059	0	0	0	0	0	0
ENSMUSG00000093875	0	0	0	0	0	0
ENSMUSG00000105052	0	0	0	0	0	0
ENSMUSG00000105051	0	2	0	2	0	0
ENSMUSG00000105056	0	0	0	0	0	0
ENSMUSG00000105054	0	0	0	0	0	0
ENSMUSG00000097934	5	0	0	0	0	0
ENSMUSG00000083176	5	2	0	2	7	0
ENSMUSG00000099440	0	0	0	0	0	0
ENSMUSG00000093877	0	1	0	0	0	0
ENSMUSG00000083278	0	0	0	0	0	0
ENSMUSG00000083279	2	0	0	0	0	0
ENSMUSG00000074764	0	0	0	0	0	0
ENSMUSG00000083272	0	0	0	0	0	0
ENSMUSG00000083270	0	1	2	0	2	5
ENSMUSG00000083271	0	0	0	0	0	0
ENSMUSG00000083276	0	0	0	0	0	0
ENSMUSG00000083275	0	0	0	0	0	0
ENSMUSG00000111646	0	0	0	0	0	0
ENSMUSG00000111645	0	0	0	0	0	0
ENSMUSG00000111643	0	0	0	0	0	0
ENSMUSG00000111641	4	4	12	4	8	4
ENSMUSG00000111649	0	0	0	0	0	4
ENSMUSG00000100452	0	0	0	0	0	0
ENSMUSG00000100453	1	0	0	0	0	0
ENSMUSG00000026734	4	0	0	1	0	2
ENSMUSG00000026735	0	0	0	0	0	0
ENSMUSG00000041945	70	52	127	33	80	92
ENSMUSG00000044719	4	8	15	4	7	10
ENSMUSG00000083858	0	0	0	0	0	0
ENSMUSG00000086760	0	0	0	0	0	0
ENSMUSG00000083855	0	0	0	0	0	0
ENSMUSG00000083851	0	0	0	0	0	0
ENSMUSG00000027460	32	11	43	6	0	0
ENSMUSG00000027463	59	73	118	49	46	56
ENSMUSG00000092912	0	0	0	0	0	0
ENSMUSG00000092914	2	0	0	0	0	0
ENSMUSG00000092917	0	0	0	0	1	1
ENSMUSG00000092919	0	0	0	0	0	0

ENSMUSG00000078042	0	0	0	0	0	0
ENSMUSG00000078041	0	0	0	0	0	0
ENSMUSG00000022375	0	0	0	0	0	0
ENSMUSG00000090202	0	11	10	0	2	9
ENSMUSG00000098459	2	3	3	0	4	1
ENSMUSG00000098455	0	0	0	0	0	0
ENSMUSG00000098452	0	0	0	0	0	0
ENSMUSG00000097936	0	0	0	0	0	0
ENSMUSG00000097302	0	0	0	0	0	0
ENSMUSG00000032940	0	6	0	0	0	4
ENSMUSG00000032948	0	0	0	0	0	0
ENSMUSG00000046623	0	5	6	0	0	0
ENSMUSG00000046620	1	0	0	0	0	0
ENSMUSG00000086987	3	4	10	4	4	4
ENSMUSG00000086981	0	0	0	0	0	0
ENSMUSG00000086980	0	0	0	0	0	0
ENSMUSG00000086989	0	0	0	0	0	0
ENSMUSG00000022818	18	6	11	4	6	0
ENSMUSG00000110155	1	0	0	0	0	0
ENSMUSG00000043398	34	38	47	34	33	33
ENSMUSG00000112070	1	0	2	0	0	0
ENSMUSG00000112072	0	0	0	0	0	0
ENSMUSG00000095902	0	0	0	0	0	0
ENSMUSG00000020908	1	0	0	0	2	0
ENSMUSG00000031411	0	0	0	0	0	0
ENSMUSG00000031410	0	0	0	0	0	0
ENSMUSG00000085985	0	0	0	0	0	0
ENSMUSG00000085980	0	0	0	0	0	0
ENSMUSG00000095917	0	0	0	0	0	0
ENSMUSG00000085989	0	0	0	0	0	0
ENSMUSG00000095918	0	0	0	0	0	0
ENSMUSG00000114928	1	0	0	0	0	0
ENSMUSG00000101233	0	0	0	0	0	0
ENSMUSG00000101231	0	0	0	0	0	0
ENSMUSG00000101230	0	0	0	0	0	0
ENSMUSG00000101235	0	0	0	0	0	0
ENSMUSG00000073830	0	0	0	0	0	0
ENSMUSG00000071471	0	0	0	0	0	0
ENSMUSG00000071470	0	0	0	4	0	0
ENSMUSG00000071475	2	0	0	0	0	0
ENSMUSG00000043972	0	0	0	0	2	0
ENSMUSG00000030450	0	0	0	0	0	0
ENSMUSG00000087166	0	0	0	0	3	0
ENSMUSG00000087164	0	0	0	0	0	0
ENSMUSG00000087162	0	0	0	0	0	3
ENSMUSG00000089514	0	0	0	0	0	0
ENSMUSG00000099477	0	0	0	0	0	0
ENSMUSG00000099476	0	0	0	0	0	0
ENSMUSG00000101589	4	8	5	7	5	5
ENSMUSG00000099472	0	0	0	2	0	0
ENSMUSG00000099470	6	6	20	4	3	8
ENSMUSG00000101584	0	0	0	0	0	0
ENSMUSG00000101587	46	71	57	38	34	58
ENSMUSG00000089516	0	0	0	0	0	0
ENSMUSG00000101581	4	0	4	0	0	0
ENSMUSG00000101580	0	0	0	0	0	0
ENSMUSG00000099479	0	0	0	0	0	0
ENSMUSG00000112012	0	0	0	0	0	0

ENSMUSG00000100182	12	4	0	4	23	29
ENSMUSG00000082663	0	0	0	2	0	0
ENSMUSG00000100180	0	0	0	0	0	0
ENSMUSG00000082661	0	0	0	0	0	0
ENSMUSG00000100186	0	0	0	0	0	0
ENSMUSG00000082667	0	0	0	0	0	0
ENSMUSG00000100184	1	0	0	0	0	0
ENSMUSG00000100185	0	0	0	0	0	0
ENSMUSG00000100188	0	5	3	5	8	7
ENSMUSG00000082669	0	0	0	0	0	0
ENSMUSG00000007457	0	3	0	0	0	0
ENSMUSG00000094933	0	0	0	0	0	0
ENSMUSG00000083130	0	0	0	0	0	0
ENSMUSG00000037101	0	0	0	0	0	0
ENSMUSG00000089194	0	0	0	0	0	0
ENSMUSG00000089191	0	0	0	0	0	0
ENSMUSG00000092948	0	0	0	0	0	0
ENSMUSG00000041538	70	61	83	30	22	26
ENSMUSG00000041536	0	0	0	0	0	0
ENSMUSG00000041534	0	0	0	0	2	0
ENSMUSG00000064722	0	0	0	0	0	0
ENSMUSG00000064720	1	0	2	0	0	0
ENSMUSG00000064726	0	0	1	0	0	0
ENSMUSG00000107322	0	0	0	0	0	0
ENSMUSG00000107327	0	1	1	0	0	0
ENSMUSG00000107326	0	0	0	0	0	0
ENSMUSG00000075202	0	1	1	0	0	0
ENSMUSG00000075200	0	0	0	0	0	0
ENSMUSG00000075201	0	0	0	0	0	0
ENSMUSG00000075206	0	0	0	0	0	0
ENSMUSG00000079584	0	0	0	0	0	0
ENSMUSG00000075204	0	0	0	0	0	0
ENSMUSG00000075205	0	0	0	2	0	0
ENSMUSG00000068600	0	0	0	0	0	0
ENSMUSG00000075208	0	0	0	0	0	0
ENSMUSG00000075209	0	2	0	0	0	0
ENSMUSG00000087835	0	0	0	0	0	0
ENSMUSG00000087836	0	0	0	0	0	0
ENSMUSG00000087830	0	0	0	0	0	0
ENSMUSG00000087831	2	0	1	2	5	3
ENSMUSG00000087832	0	0	0	0	0	0
ENSMUSG00000087833	0	0	0	0	0	0
ENSMUSG00000102168	0	0	0	0	0	0
ENSMUSG00000102169	0	0	0	0	0	0
ENSMUSG00000085478	0	0	0	0	0	0
ENSMUSG00000085476	0	0	0	0	0	0
ENSMUSG00000082976	0	0	0	0	0	0
ENSMUSG00000085472	0	0	0	0	0	0
ENSMUSG00000085473	6	4	3	2	11	1
ENSMUSG00000085470	0	0	0	0	0	0
ENSMUSG00000074155	0	0	0	0	0	0
ENSMUSG00000074156	0	0	0	0	0	0
ENSMUSG00000064508	0	0	0	0	0	0
ENSMUSG00000079058	0	0	0	0	0	0
ENSMUSG00000082977	0	0	0	0	0	0
ENSMUSG00000079056	42	101	57	73	74	69
ENSMUSG00000079055	15	4	10	4	3	10
ENSMUSG00000096220	0	0	0	0	2	0

ENSMUSG00000023755	102	137	96	69	86	90
ENSMUSG00000088747	0	0	0	0	0	0
ENSMUSG00000088746	0	0	0	0	0	0
ENSMUSG00000088749	0	0	0	0	0	0
ENSMUSG00000088748	0	0	0	0	0	0
ENSMUSG00000094979	0	0	0	0	0	0
ENSMUSG00000021448	44	41	66	59	60	69
ENSMUSG00000094975	0	0	0	0	0	0
ENSMUSG00000094977	0	0	0	0	0	0
ENSMUSG00000094976	0	0	0	0	0	0
ENSMUSG00000039620	75	104	78	41	68	83
ENSMUSG00000021440	0	0	0	0	0	0
ENSMUSG00000094973	3	1	1	2	0	0
ENSMUSG00000039623	326	324	389	210	249	338
ENSMUSG00000095623	0	0	0	0	0	0
ENSMUSG00000112010	0	0	0	0	0	0
ENSMUSG00000086346	0	0	0	0	0	0
ENSMUSG00000028031	8	5	7	3	0	5
ENSMUSG00000101714	0	0	0	0	0	0
ENSMUSG00000029618	0	0	0	0	0	0
ENSMUSG00000101717	0	0	0	0	0	0
ENSMUSG00000103518	0	0	0	0	0	0
ENSMUSG00000103519	0	3	0	0	0	0
ENSMUSG00000103514	0	0	0	0	0	0
ENSMUSG00000106743	3	3	5	1	1	1
ENSMUSG00000106740	0	0	0	0	0	0
ENSMUSG00000106741	0	0	1	0	0	0
ENSMUSG00000103510	0	0	0	0	0	0
ENSMUSG00000103511	0	0	0	0	0	0
ENSMUSG00000103512	0	0	0	0	0	0
ENSMUSG00000103513	0	0	5	3	2	1
ENSMUSG00000097408	1	1	0	0	0	0
ENSMUSG00000022935	0	0	0	0	0	0
ENSMUSG00000097406	6	0	1	0	5	0
ENSMUSG00000022931	0	0	0	0	0	3
ENSMUSG00000076028	3	0	1	0	1	0
ENSMUSG00000056290	44	39	48	11	21	24
ENSMUSG00000051046	0	0	0	0	2	0
ENSMUSG00000101718	0	0	0	0	0	0
ENSMUSG00000051048	64	38	77	0	0	0
ENSMUSG00000100964	0	0	0	0	0	0
ENSMUSG00000100965	0	0	0	0	0	0
ENSMUSG00000100966	0	0	0	0	0	0
ENSMUSG00000114445	0	0	0	0	0	0
ENSMUSG00000114442	0	0	0	7	0	0
ENSMUSG00000100961	0	0	0	0	0	0
ENSMUSG00000100962	0	0	0	2	0	0
ENSMUSG00000114441	0	0	0	0	0	0
ENSMUSG00000100968	0	0	0	0	0	0
ENSMUSG00000114448	2	0	0	0	0	0
ENSMUSG00000114449	0	0	0	0	0	0
ENSMUSG00000082285	0	0	0	0	0	0
ENSMUSG00000020474	90	93	85	69	76	64
ENSMUSG00000054272	0	0	0	0	0	0
ENSMUSG00000099572	0	0	0	0	0	0
ENSMUSG00000105218	0	0	0	0	0	0
ENSMUSG00000109772	0	0	0	0	0	0
ENSMUSG00000109773	0	0	4	0	0	0

ENSMUSG00000109771	6	6	18	5	6	10
ENSMUSG00000066810	1	0	0	0	0	0
ENSMUSG00000108429	0	0	0	0	0	0
ENSMUSG00000108428	0	0	0	3	0	0
ENSMUSG00000108421	0	0	0	0	0	0
ENSMUSG00000108420	3	7	14	7	18	26
ENSMUSG00000108422	4	2	2	0	0	0
ENSMUSG00000108424	0	0	0	0	0	0
ENSMUSG00000108427	0	0	0	0	0	0
ENSMUSG00000108426	0	0	0	0	0	0
ENSMUSG00000091806	0	0	0	0	0	0
ENSMUSG00000091805	0	0	0	0	0	0
ENSMUSG00000024088	0	0	0	0	0	0
ENSMUSG00000091808	0	0	0	0	0	0
ENSMUSG00000091809	0	0	0	0	0	0
ENSMUSG00000096465	0	4	0	0	0	0
ENSMUSG00000096467	0	0	0	0	0	0
ENSMUSG00000096463	3	0	1	2	1	4
ENSMUSG00000096462	0	0	1	0	0	0
ENSMUSG00000096468	0	0	0	0	0	0
ENSMUSG00000114333	0	0	0	0	0	0
ENSMUSG00000010592	5	8	0	14	12	8
ENSMUSG00000088000	0	0	1	0	0	0
ENSMUSG00000045022	2	0	0	0	0	0
ENSMUSG00000111252	0	0	0	0	0	0
ENSMUSG00000111258	0	0	0	0	0	0
ENSMUSG00000111259	0	0	0	0	0	0
ENSMUSG00000091418	19	14	11	5	13	15
ENSMUSG00000027261	0	0	0	0	0	0
ENSMUSG00000091114	0	0	0	0	0	0
ENSMUSG00000091116	0	0	0	0	0	0
ENSMUSG00000091110	0	0	0	0	0	0
ENSMUSG00000090449	0	0	0	0	0	0
ENSMUSG00000090448	0	0	0	0	0	0
ENSMUSG00000090440	0	0	0	0	0	0
ENSMUSG00000081741	0	0	0	0	0	0
ENSMUSG00000081742	0	0	0	0	0	0
ENSMUSG00000081743	0	0	0	0	0	0
ENSMUSG00000081744	0	0	0	0	0	0
ENSMUSG00000081746	0	0	0	0	0	0
ENSMUSG00000081748	2	0	0	1	3	4
ENSMUSG00000081749	0	0	0	0	0	0
ENSMUSG00000053552	11	11	36	21	19	27
ENSMUSG00000053550	0	0	0	0	4	0
ENSMUSG00000042554	0	0	0	0	2	0
ENSMUSG00000088004	3	4	6	1	3	4
ENSMUSG00000094567	0	0	0	0	0	0
ENSMUSG00000094564	0	0	0	0	0	0
ENSMUSG00000094563	1	1	0	0	0	0
ENSMUSG00000094560	0	0	0	0	0	0
ENSMUSG00000111878	0	0	0	0	0	0
ENSMUSG00000094690	12	0	6	4	6	6
ENSMUSG00000094691	0	0	0	0	0	0
ENSMUSG00000094692	0	0	0	0	0	0
ENSMUSG00000094693	0	0	0	0	0	0
ENSMUSG00000094696	0	0	0	0	0	0
ENSMUSG00000094699	0	0	0	0	0	0
ENSMUSG00000109802	0	0	0	0	0	0

ENSMUSG00000086214	0	0	0	0	2	0
ENSMUSG00000086211	4	0	0	0	0	0
ENSMUSG00000072324	1	0	1	0	0	0
ENSMUSG00000077668	0	0	0	0	0	0
ENSMUSG00000077669	0	0	0	0	0	0
ENSMUSG00000077663	0	0	0	0	0	0
ENSMUSG00000077660	0	0	0	0	0	0
ENSMUSG00000077661	0	0	0	0	0	0
ENSMUSG00000077666	0	0	0	0	0	0
ENSMUSG00000077664	0	0	0	0	0	0
ENSMUSG00000055480	257	168	146	142	178	209
ENSMUSG00000049460	0	0	0	0	0	0
ENSMUSG00000029553	31	58	32	17	18	19
ENSMUSG00000072492	0	0	2	0	0	0
ENSMUSG00000092599	0	0	0	0	0	0
ENSMUSG00000108566	0	0	0	0	0	0
ENSMUSG00000049972	0	0	0	0	0	0
ENSMUSG00000092597	0	0	0	0	0	0
ENSMUSG00000050121	0	0	0	0	0	0
ENSMUSG00000092591	0	0	0	0	2	0
ENSMUSG00000071679	0	0	0	0	0	0
ENSMUSG00000104261	0	0	0	0	0	0
ENSMUSG00000096878	0	0	9	0	0	0
ENSMUSG00000096879	0	0	0	0	0	0
ENSMUSG00000098852	0	0	0	0	0	0
ENSMUSG00000098853	0	0	0	0	0	0
ENSMUSG00000096874	0	0	0	0	0	0
ENSMUSG00000096875	0	0	0	0	0	0
ENSMUSG00000096872	0	2	0	2	0	0
ENSMUSG00000098854	0	0	0	0	0	0
ENSMUSG00000096871	0	0	0	0	0	0
ENSMUSG00000106263	2	0	0	0	0	0
ENSMUSG00000106267	0	0	0	0	0	0
ENSMUSG00000106266	0	0	0	0	0	0
ENSMUSG00000106268	0	0	1	0	0	0
ENSMUSG00000112004	0	0	0	0	0	0
ENSMUSG00000047079	0	0	0	0	0	0
ENSMUSG00000109431	0	0	0	0	0	0
ENSMUSG00000109433	0	0	0	0	0	0
ENSMUSG00000109432	0	0	0	0	0	0
ENSMUSG00000004360	168	124	53	25	37	54
ENSMUSG00000109437	0	0	0	0	0	0
ENSMUSG00000109439	0	0	0	0	0	0
ENSMUSG00000102387	0	0	0	0	0	0
ENSMUSG00000083447	0	0	0	0	0	0
ENSMUSG00000104659	0	0	0	0	0	0
ENSMUSG00000082461	2	0	6	2	0	2
ENSMUSG00000082462	0	0	0	0	0	0
ENSMUSG00000103488	0	0	0	0	0	0
ENSMUSG00000082468	0	0	0	0	4	0
ENSMUSG00000103489	0	0	0	0	0	0
ENSMUSG00000112006	0	0	0	0	0	0
ENSMUSG00000050961	0	0	0	0	0	0
ENSMUSG00000050966	0	0	0	0	0	0
ENSMUSG00000021047	96	123	64	40	98	121
ENSMUSG00000106477	0	0	0	0	1	0
ENSMUSG00000084108	0	0	0	0	0	0
ENSMUSG00000083338	0	0	0	0	2	0

ENSMUSG00000083336	0	0	0	0	0	0
ENSMUSG00000083335	0	2	0	0	0	0
ENSMUSG00000106473	0	0	0	0	0	0
ENSMUSG00000084101	0	0	0	0	0	0
ENSMUSG00000084103	0	0	0	0	0	0
ENSMUSG00000025328	0	0	0	0	0	0
ENSMUSG00000025329	0	0	0	0	0	0
ENSMUSG00000080828	0	0	0	0	0	0
ENSMUSG00000080822	0	0	0	0	0	0
ENSMUSG00000080820	0	0	0	0	0	0
ENSMUSG00000080826	0	0	0	0	0	0
ENSMUSG00000080827	0	0	0	0	0	0
ENSMUSG00000080825	0	0	0	0	0	0
ENSMUSG00000040938	27	29	17	22	37	38
ENSMUSG00000081012	0	0	0	0	0	0
ENSMUSG00000099068	1	1	3	2	2	1
ENSMUSG00000040935	0	0	0	0	0	0
ENSMUSG00000107718	0	0	0	0	0	0
ENSMUSG00000000627	17	8	4	11	11	25
ENSMUSG00000102380	17	18	20	8	6	14
ENSMUSG00000041044	0	0	0	0	0	0
ENSMUSG00000044039	0	0	0	0	0	0
ENSMUSG00000070235	0	0	0	0	0	0
ENSMUSG00000070236	0	0	0	0	0	0
ENSMUSG00000107715	0	4	2	2	0	0
ENSMUSG00000085275	0	0	0	0	0	0
ENSMUSG00000085271	4	2	4	0	0	0
ENSMUSG00000102381	0	0	0	0	0	0
ENSMUSG00000093967	0	1	0	4	2	1
ENSMUSG00000093965	0	0	0	1	0	0
ENSMUSG00000093964	0	0	0	0	0	0
ENSMUSG00000093963	0	0	0	0	0	0
ENSMUSG00000093961	0	0	0	0	0	0
ENSMUSG00000111942	51	51	61	72	40	59
ENSMUSG00000026679	23	21	35	6	13	28
ENSMUSG00000093968	0	0	0	0	0	0
ENSMUSG00000111944	0	0	0	0	0	0
ENSMUSG00000111946	1	0	0	0	1	1
ENSMUSG00000111947	29	31	18	22	30	20
ENSMUSG00000080175	0	0	0	0	0	0
ENSMUSG00000111948	0	0	0	0	0	0
ENSMUSG00000074420	0	3	0	0	0	0
ENSMUSG00000074424	0	3	0	0	0	0
ENSMUSG00000045340	0	0	0	0	0	0
ENSMUSG00000045341	0	0	0	0	0	0
ENSMUSG00000045348	43	40	74	24	21	29
ENSMUSG00000045349	0	8	12	6	10	6
ENSMUSG00000081881	0	0	0	0	2	0
ENSMUSG00000081880	0	0	0	0	0	0
ENSMUSG00000081886	0	0	0	0	0	0
ENSMUSG00000081884	0	3	1	4	5	4
ENSMUSG00000111449	0	0	0	0	0	0
ENSMUSG00000101161	0	0	0	1	0	0
ENSMUSG00000111448	0	0	0	0	0	0
ENSMUSG00000101160	0	0	0	0	0	0
ENSMUSG00000109624	0	0	0	0	0	0
ENSMUSG00000063531	23	15	10	18	16	33
ENSMUSG00000099939	0	0	0	0	0	0

ENSMUSG00000099936	20	37	22	29	37	37
ENSMUSG00000099934	2	0	1	0	0	6
ENSMUSG00000101164	0	0	0	0	0	0
ENSMUSG00000109620	0	0	0	0	0	0
ENSMUSG00000111440	0	0	0	0	0	0
ENSMUSG00000098089	0	0	0	0	0	0
ENSMUSG00000109622	0	0	0	0	0	0
ENSMUSG00000098081	0	0	0	0	0	0
ENSMUSG00000098080	2	0	0	0	0	4
ENSMUSG00000098083	0	0	0	0	0	0
ENSMUSG00000098082	0	3	3	0	0	0
ENSMUSG00000098085	0	0	0	0	0	0
ENSMUSG00000098084	0	0	0	0	0	0
ENSMUSG00000098087	0	0	0	0	0	0
ENSMUSG00000098086	0	0	0	0	0	0
ENSMUSG00000081225	0	0	0	0	0	2
ENSMUSG00000081224	0	0	0	0	0	0
ENSMUSG00000081222	2	0	0	0	0	0
ENSMUSG00000066108	16	10	23	8	23	14
ENSMUSG00000081229	0	2	2	0	0	0
ENSMUSG00000105521	0	0	0	0	0	0
ENSMUSG00000073386	0	0	0	0	0	0
ENSMUSG00000019803	0	0	0	0	0	0
ENSMUSG00000048473	3	0	0	4	5	3
ENSMUSG00000077303	0	0	0	0	0	0
ENSMUSG00000077302	0	0	0	0	0	0
ENSMUSG00000077305	0	0	0	0	0	0
ENSMUSG00000077304	0	0	0	0	0	0
ENSMUSG00000038805	0	0	0	0	0	0
ENSMUSG00000077306	2	0	0	0	0	0
ENSMUSG00000077309	3	5	7	0	1	0
ENSMUSG00000035984	74	65	60	41	43	56
ENSMUSG00000048824	0	0	0	0	0	0
ENSMUSG00000102952	0	0	0	0	0	0
ENSMUSG00000102957	0	0	0	0	0	0
ENSMUSG00000102955	0	0	0	0	0	0
ENSMUSG00000081165	4	0	0	3	0	0
ENSMUSG00000020299	2	0	0	0	0	0
ENSMUSG00000089707	0	0	2	0	0	2
ENSMUSG00000043583	3	0	0	0	0	0
ENSMUSG00000113331	0	0	0	0	0	0
ENSMUSG00000082147	0	0	0	0	0	0
ENSMUSG00000088162	0	0	0	0	0	0
ENSMUSG00000088161	0	0	0	0	0	0
ENSMUSG00000093153	0	0	0	0	0	0
ENSMUSG00000088167	0	0	0	0	0	0
ENSMUSG00000088166	0	0	0	0	0	0
ENSMUSG00000088165	0	2	0	1	0	5
ENSMUSG00000088164	0	0	0	0	0	0
ENSMUSG00000087318	0	0	0	0	0	0
ENSMUSG00000082149	0	0	0	0	0	1
ENSMUSG00000037603	13	11	9	12	15	15
ENSMUSG00000037605	177	210	152	169	197	225
ENSMUSG00000080936	0	0	0	0	0	0
ENSMUSG00000103339	0	0	0	0	0	0
ENSMUSG00000103336	10	10	4	4	3	5
ENSMUSG00000106399	0	0	0	0	0	0
ENSMUSG00000106398	0	0	0	0	0	0

ENSMUSG00000019359	0	0	0	0	5	0
ENSMUSG00000014104	0	0	0	0	1	0
ENSMUSG00000101796	0	0	0	0	0	0
ENSMUSG00000101797	0	0	0	0	0	0
ENSMUSG00000101790	0	0	0	0	0	0
ENSMUSG00000101791	0	0	0	5	0	0
ENSMUSG00000040055	0	2	0	0	2	0
ENSMUSG00000059821	0	0	0	0	0	0
ENSMUSG00000107828	0	4	0	0	0	0
ENSMUSG00000107826	0	0	0	0	0	2
ENSMUSG00000107821	0	0	0	3	0	0
ENSMUSG00000107822	0	0	0	0	0	0
ENSMUSG00000084664	0	0	0	0	0	0
ENSMUSG00000107797	0	0	0	0	0	0
ENSMUSG00000009108	26	18	14	15	15	16
ENSMUSG00000084660	0	0	0	0	0	0
ENSMUSG00000084661	0	0	0	0	0	0
ENSMUSG00000107790	0	0	0	0	0	0
ENSMUSG00000107791	0	0	0	0	0	0
ENSMUSG00000084668	0	0	0	0	0	0
ENSMUSG00000100449	0	0	0	0	0	0
ENSMUSG00000100448	2	1	0	1	2	0
ENSMUSG00000100443	0	0	0	0	0	0
ENSMUSG00000100442	0	0	0	0	0	0
ENSMUSG00000100440	0	0	0	0	0	0
ENSMUSG00000100447	0	0	0	0	0	0
ENSMUSG00000100446	0	0	0	0	0	3
ENSMUSG00000100445	0	0	0	0	0	0
ENSMUSG00000100444	0	0	0	0	0	0
ENSMUSG00000022364	299	256	274	224	214	309
ENSMUSG00000022367	66	98	98	37	45	52
ENSMUSG00000022366	0	0	0	0	0	0
ENSMUSG00000064397	0	0	0	0	0	0
ENSMUSG00000064394	0	0	0	0	0	0
ENSMUSG00000064395	0	0	0	2	0	0
ENSMUSG00000064392	0	0	0	0	0	0
ENSMUSG00000064393	0	0	0	0	0	0
ENSMUSG00000032641	105	125	96	100	93	116
ENSMUSG00000064391	0	0	0	0	0	0
ENSMUSG00000064398	7	3	0	0	0	5
ENSMUSG00000064399	0	0	0	0	0	0
ENSMUSG00000082302	0	0	0	0	0	0
ENSMUSG00000089789	11	9	12	6	8	12
ENSMUSG00000089780	0	0	0	2	0	0
ENSMUSG00000089783	0	0	0	0	0	0
ENSMUSG00000095903	0	0	0	0	0	0
ENSMUSG00000095901	0	0	0	0	0	0
ENSMUSG00000095900	0	0	0	0	0	0
ENSMUSG00000095907	0	0	0	0	0	0
ENSMUSG00000020216	32	29	22	12	5	19
ENSMUSG00000075437	0	0	0	0	0	0
ENSMUSG00000020214	0	0	0	0	0	0
ENSMUSG00000095909	0	0	0	0	0	0
ENSMUSG00000102175	10	5	2	0	0	2
ENSMUSG00000102174	0	0	0	0	0	0
ENSMUSG00000102177	0	0	0	0	0	0
ENSMUSG00000102176	0	0	0	0	0	4
ENSMUSG00000102170	0	0	0	0	0	0

ENSMUSG00000102173	0	0	0	0	0	0
ENSMUSG00000102178	0	0	0	0	0	0
ENSMUSG00000099421	0	0	0	0	0	0
ENSMUSG00000065441	0	0	0	0	0	0
ENSMUSG00000065443	0	0	0	0	0	0
ENSMUSG00000026904	5	7	5	0	0	0
ENSMUSG00000065448	0	0	0	0	2	0
ENSMUSG00000097666	32	52	28	47	53	79
ENSMUSG00000112415	0	0	0	0	0	0
ENSMUSG00000109283	0	0	0	0	0	0
ENSMUSG00000029797	39	40	38	22	14	47
ENSMUSG00000029790	107	93	126	78	71	137
ENSMUSG00000112938	0	0	0	0	0	0
ENSMUSG00000095798	2	0	0	0	0	0
ENSMUSG00000054417	0	0	0	0	0	0
ENSMUSG00000109281	0	0	0	4	0	3
ENSMUSG00000039813	82	71	100	81	104	140
ENSMUSG00000039814	0	7	4	0	0	14
ENSMUSG00000090047	0	2	0	0	0	0
ENSMUSG00000103491	0	0	0	0	0	2
ENSMUSG00000103494	0	0	0	0	0	0
ENSMUSG00000103499	0	0	0	0	0	0
ENSMUSG00000113726	0	2	0	0	0	0
ENSMUSG00000113727	3	8	0	6	3	7
ENSMUSG00000113724	5	2	7	0	1	5
ENSMUSG00000113725	0	1	0	0	1	0
ENSMUSG00000088957	0	0	0	0	0	0
ENSMUSG00000113720	0	0	0	0	0	0
ENSMUSG00000113721	0	0	0	0	0	0
ENSMUSG00000088959	0	0	0	0	0	0
ENSMUSG00000091627	0	0	0	0	0	0
ENSMUSG00000059003	9	12	14	3	0	0
ENSMUSG00000110308	1	0	0	0	1	0
ENSMUSG00000111470	0	0	0	0	0	0
ENSMUSG00000111473	0	0	0	0	2	0
ENSMUSG00000111474	0	0	0	0	0	0
ENSMUSG00000109639	0	0	0	0	0	0
ENSMUSG00000111477	0	0	0	0	0	0
ENSMUSG00000109637	0	0	0	0	0	0
ENSMUSG00000109636	12	1	11	6	7	10
ENSMUSG00000109634	2	4	0	4	0	0
ENSMUSG00000109633	0	0	0	0	0	0
ENSMUSG00000109632	4	0	7	0	0	4
ENSMUSG00000109631	0	0	0	0	0	0
ENSMUSG00000109630	0	0	0	0	0	0
ENSMUSG00000011349	0	0	0	0	0	0
ENSMUSG00000042433	0	0	0	0	0	0
ENSMUSG00000066137	0	0	0	0	4	0
ENSMUSG00000108328	0	0	0	0	0	0
ENSMUSG00000108329	0	0	0	0	0	0
ENSMUSG00000108323	0	0	0	0	0	0
ENSMUSG00000034394	16	11	42	0	6	8
ENSMUSG00000108326	0	0	0	0	0	0
ENSMUSG00000108327	0	0	0	0	0	0
ENSMUSG00000108325	2	0	0	0	0	0
ENSMUSG00000110302	3	4	3	3	0	7
ENSMUSG00000048574	3	2	0	0	1	1
ENSMUSG00000109988	0	0	0	0	0	0

ENSMUSG00000109993	0	0	0	0	0	0
ENSMUSG00000097676	0	0	0	0	0	0
ENSMUSG00000054406	0	0	0	0	0	0
ENSMUSG00000038148	0	0	0	0	0	0
ENSMUSG00000112929	0	0	0	0	0	0
ENSMUSG00000112928	0	0	0	0	0	0
ENSMUSG00000108586	0	0	0	0	0	0
ENSMUSG00000108587	0	0	0	0	0	0
ENSMUSG00000112924	0	0	0	0	0	0
ENSMUSG00000112923	0	0	0	0	0	0
ENSMUSG00000108583	0	0	0	0	0	0
ENSMUSG00000108580	0	0	0	0	0	0
ENSMUSG00000112920	0	0	0	0	0	0
ENSMUSG00000091635	0	0	0	0	0	0
ENSMUSG00000091636	4	0	0	0	4	2
ENSMUSG00000091631	0	0	0	0	0	0
ENSMUSG00000091638	0	0	0	0	0	0
ENSMUSG00000100133	0	0	0	0	0	0
ENSMUSG00000092066	0	0	0	0	0	0
ENSMUSG00000114189	0	0	0	0	0	0
ENSMUSG00000114187	0	0	0	0	0	0
ENSMUSG00000114186	0	0	0	0	0	0
ENSMUSG00000114185	0	0	8	0	0	0
ENSMUSG00000114183	0	0	0	0	0	0
ENSMUSG00000114182	0	0	0	0	0	0
ENSMUSG00000114181	0	0	0	0	0	0
ENSMUSG00000114180	0	0	0	0	0	0
ENSMUSG00000025650	60	46	74	28	6	36
ENSMUSG00000110404	4	7	3	1	4	2
ENSMUSG00000110402	0	0	0	0	0	0
ENSMUSG00000050239	0	0	0	0	0	0
ENSMUSG00000025665	6	0	0	8	9	7
ENSMUSG00000097519	0	4	0	0	0	0
ENSMUSG00000100138	0	0	0	0	0	0
ENSMUSG00000067773	0	0	0	0	0	0
ENSMUSG00000072739	0	0	0	0	3	5
ENSMUSG00000044581	0	0	0	1	0	0
ENSMUSG00000099894	0	0	0	0	0	0
ENSMUSG00000052861	4	37	36	0	5	7
ENSMUSG00000060204	0	0	0	0	0	0
ENSMUSG00000060205	0	0	0	0	0	0
ENSMUSG00000060206	673	458	742	285	235	513
ENSMUSG00000060201	0	0	0	0	0	0
ENSMUSG00000110477	0	0	0	0	0	0
ENSMUSG00000060208	0	0	0	0	0	0
ENSMUSG00000109671	0	0	0	0	0	0
ENSMUSG00000080378	0	0	0	0	0	0
ENSMUSG00000110476	0	0	0	0	0	0
ENSMUSG00000114819	1	0	0	0	0	0
ENSMUSG00000109012	0	0	0	0	0	0
ENSMUSG00000026304	42	35	24	54	62	83
ENSMUSG00000026303	0	16	39	0	14	17
ENSMUSG00000026301	0	0	0	0	0	0
ENSMUSG00000057174	0	0	0	0	0	0
ENSMUSG00000057170	0	0	0	0	0	0
ENSMUSG00000057173	0	7	3	7	13	19
ENSMUSG00000109017	0	0	0	0	0	0
ENSMUSG00000046818	51	36	23	45	29	60

ENSMUSG00000110478	0	0	0	0	0	0
ENSMUSG00000099700	0	0	0	0	0	0
ENSMUSG00000096982	0	0	0	0	0	0
ENSMUSG00000097650	4	0	3	0	0	0
ENSMUSG00000114813	0	0	0	0	0	0
ENSMUSG00000001804	2	0	0	0	0	0
ENSMUSG00000114815	0	0	0	0	0	0
ENSMUSG00000096987	0	17	0	0	0	0
ENSMUSG00000056815	0	0	0	0	0	0
ENSMUSG00000048677	286	336	336	164	210	239
ENSMUSG00000065141	0	0	0	0	0	0
ENSMUSG00000077149	0	0	0	0	0	0
ENSMUSG00000077144	0	0	0	0	0	0
ENSMUSG00000077143	0	0	0	0	0	0
ENSMUSG00000077142	0	0	0	0	0	0
ENSMUSG00000067767	0	0	0	0	0	0
ENSMUSG00000093563	0	0	0	0	0	0
ENSMUSG00000094714	0	0	0	0	0	0
ENSMUSG00000094717	0	1	0	0	0	1
ENSMUSG00000093560	0	0	0	0	0	0
ENSMUSG00000093567	0	0	0	0	0	1
ENSMUSG00000094710	1	1	1	0	0	1
ENSMUSG00000093564	0	0	0	0	0	0
ENSMUSG00000093568	0	0	0	0	0	0
ENSMUSG00000094719	0	0	0	0	0	0
ENSMUSG00000094718	1	0	0	0	0	0
ENSMUSG00000017204	10	13	23	0	4	7
ENSMUSG00000107667	2	0	9	0	0	0
ENSMUSG00000099605	0	0	0	0	0	0
ENSMUSG00000099606	0	0	0	0	0	0
ENSMUSG00000099608	1	0	0	0	1	1
ENSMUSG00000077492	0	0	0	0	0	0
ENSMUSG00000077495	0	0	0	0	0	0
ENSMUSG00000112118	0	0	0	0	0	0
ENSMUSG00000112119	0	0	0	0	0	0
ENSMUSG00000112117	59	93	46	125	77	143
ENSMUSG00000112114	0	0	0	0	0	0
ENSMUSG00000112115	0	0	0	0	0	0
ENSMUSG00000112112	2	0	0	0	3	2
ENSMUSG00000112113	0	0	0	0	0	0
ENSMUSG00000112111	0	0	2	0	0	2
ENSMUSG00000096534	0	0	0	0	0	0
ENSMUSG00000101314	0	0	0	0	0	0
ENSMUSG00000104774	0	0	0	1	1	0
ENSMUSG00000092388	0	0	0	0	0	0
ENSMUSG00000092385	0	0	0	0	0	0
ENSMUSG00000092387	0	0	0	0	0	0
ENSMUSG00000054892	18	17	17	18	6	14
ENSMUSG00000054893	207	226	197	207	189	256
ENSMUSG00000054890	5	3	8	2	0	0
ENSMUSG00000092383	0	0	0	0	0	0
ENSMUSG00000051917	0	0	0	0	0	0
ENSMUSG00000093385	0	0	0	0	0	0
ENSMUSG00000046518	0	0	0	0	0	0
ENSMUSG00000096539	0	0	0	0	0	0
ENSMUSG00000086008	2	0	0	0	0	0
ENSMUSG00000086009	0	0	0	0	0	0
ENSMUSG00000086006	0	4	0	0	0	0

ENSMUSG00000086007	0	0	0	0	0	0
ENSMUSG00000043740	33	29	37	7	8	11
ENSMUSG00000043747	0	0	2	2	5	9
ENSMUSG00000086000	0	0	0	0	3	0
ENSMUSG00000086001	0	0	0	0	0	0
ENSMUSG00000098957	0	0	1	0	0	0
ENSMUSG00000076361	0	0	0	0	0	0
ENSMUSG00000098952	0	0	0	0	0	0
ENSMUSG00000076366	0	0	0	0	0	0
ENSMUSG00000082785	0	0	0	0	0	0
ENSMUSG00000082784	0	0	0	0	0	0
ENSMUSG00000082786	0	0	0	0	0	0
ENSMUSG00000082781	0	0	0	0	0	0
ENSMUSG00000082783	0	0	0	0	0	0
ENSMUSG00000082782	0	4	0	0	0	0
ENSMUSG00000082789	0	2	2	0	0	0
ENSMUSG00000082788	0	0	0	0	0	0
ENSMUSG00000037446	11	20	19	0	3	2
ENSMUSG00000089205	0	0	0	0	0	0
ENSMUSG00000037440	31	28	18	0	0	0
ENSMUSG00000049230	38	49	51	29	42	36
ENSMUSG00000101318	0	0	0	0	0	0
ENSMUSG00000099500	0	0	0	0	0	0
ENSMUSG00000087668	0	0	4	3	4	0
ENSMUSG00000087663	0	0	0	0	0	0
ENSMUSG00000087662	0	0	0	0	0	0
ENSMUSG00000102891	0	0	0	0	0	0
ENSMUSG00000040217	0	0	0	0	0	0
ENSMUSG00000083789	0	0	0	1	0	0
ENSMUSG00000083782	0	0	0	0	0	0
ENSMUSG00000083781	0	0	0	0	0	0
ENSMUSG00000083786	1	1	1	1	4	0
ENSMUSG00000083787	0	0	0	0	0	0
ENSMUSG00000083784	0	0	0	0	0	0
ENSMUSG00000083785	0	0	0	0	0	0
ENSMUSG00000057817	3	0	2	0	0	0
ENSMUSG00000086239	0	0	0	0	0	0
ENSMUSG00000071356	0	0	0	0	0	0
ENSMUSG00000084318	0	0	0	0	0	0
ENSMUSG00000107483	0	0	0	0	0	3
ENSMUSG00000084315	0	0	0	0	0	0
ENSMUSG00000084310	0	0	0	0	0	0
ENSMUSG00000084311	0	0	0	0	0	0
ENSMUSG00000084312	0	0	0	0	1	0
ENSMUSG00000000817	0	0	0	0	0	0
ENSMUSG00000083167	0	0	0	0	0	0
ENSMUSG00000082910	0	0	0	0	0	0
ENSMUSG00000025515	0	2	8	0	0	0
ENSMUSG00000084797	7	6	6	10	11	7
ENSMUSG00000082912	0	0	0	0	0	0
ENSMUSG00000025519	0	0	0	0	0	0
ENSMUSG00000099385	0	0	0	0	0	0
ENSMUSG00000099387	0	0	0	0	0	0
ENSMUSG00000099386	0	0	0	0	0	0
ENSMUSG00000099381	4	7	1	2	2	3
ENSMUSG00000099380	0	0	0	0	0	0
ENSMUSG00000099383	0	0	0	0	0	0
ENSMUSG00000074322	0	0	0	0	0	0

ENSMUSG00000099388	0	0	0	0	0	0
ENSMUSG00000021211	2	0	0	0	0	0
ENSMUSG00000081928	0	0	2	0	2	0
ENSMUSG00000081921	0	0	0	0	0	0
ENSMUSG00000075983	0	0	0	0	0	0
ENSMUSG00000081923	0	0	2	0	0	0
ENSMUSG00000081924	0	0	0	0	0	0
ENSMUSG00000081925	0	0	0	0	0	0
ENSMUSG00000065603	2	1	2	0	2	0
ENSMUSG00000065600	1	1	2	1	2	2
ENSMUSG00000065609	0	0	1	0	0	0
ENSMUSG00000070000	15	7	24	4	4	8
ENSMUSG00000032254	136	102	99	38	76	110
ENSMUSG00000074589	0	0	0	0	0	0
ENSMUSG00000032259	21	20	29	56	33	34
ENSMUSG00000073678	672	799	643	731	837	933
ENSMUSG00000068182	0	0	0	0	0	0
ENSMUSG00000068181	0	0	0	0	0	0
ENSMUSG00000068180	0	0	0	0	0	0
ENSMUSG00000006411	29	22	48	12	21	14
ENSMUSG00000092999	0	0	0	0	0	0
ENSMUSG00000111953	1	0	0	0	0	0
ENSMUSG00000072982	0	0	0	0	0	0
ENSMUSG00000072983	0	0	0	0	0	0
ENSMUSG00000098169	0	0	0	0	0	0
ENSMUSG00000098167	0	0	0	0	0	0
ENSMUSG00000098165	0	0	0	0	0	2
ENSMUSG00000098162	0	0	0	0	0	0
ENSMUSG00000098163	0	0	0	0	0	0
ENSMUSG00000098160	0	0	0	0	0	0
ENSMUSG00000098161	0	0	0	0	0	0
ENSMUSG00000103963	0	0	0	0	0	0
ENSMUSG00000103962	0	0	0	0	0	0
ENSMUSG00000103965	0	0	0	0	0	0
ENSMUSG00000103967	25	37	11	14	28	25
ENSMUSG00000103966	14	14	24	6	15	12
ENSMUSG00000034159	0	0	0	0	0	0
ENSMUSG00000034156	12	5	4	0	0	8
ENSMUSG00000034151	0	0	0	0	0	0
ENSMUSG00000093853	0	0	0	0	0	0
ENSMUSG00000025774	0	0	0	0	0	0
ENSMUSG00000109903	0	0	0	0	0	0
ENSMUSG00000051504	0	5	25	9	6	10
ENSMUSG00000093857	0	0	0	0	0	0
ENSMUSG00000109906	0	0	0	0	0	0
ENSMUSG00000089840	0	0	0	0	0	0
ENSMUSG00000111168	0	0	0	0	0	0
ENSMUSG00000091430	0	0	0	0	0	0
ENSMUSG00000051509	0	0	0	0	0	0
ENSMUSG00000091435	0	0	0	0	0	0
ENSMUSG00000001815	0	0	0	0	0	0
ENSMUSG00000114100	0	0	0	0	0	0
ENSMUSG00000091387	82	69	123	10	8	12
ENSMUSG00000091386	0	0	0	0	0	0
ENSMUSG00000091381	0	5	0	0	0	0
ENSMUSG00000088280	0	0	0	0	0	0
ENSMUSG00000067571	0	0	0	0	0	0
ENSMUSG00000067578	0	0	0	0	0	0

ENSMUSG00000114109	0	0	0	0	0	0
ENSMUSG00000114108	0	0	0	0	0	0
ENSMUSG00000040629	0	0	0	0	0	0
ENSMUSG00000101611	0	0	0	0	0	0
ENSMUSG00000101613	0	0	0	0	0	0
ENSMUSG00000101615	6	0	0	0	0	0
ENSMUSG00000101614	0	0	0	0	0	0
ENSMUSG00000101617	0	0	0	0	0	0
ENSMUSG00000101616	0	0	0	0	0	0
ENSMUSG00000101619	0	0	0	0	0	0
ENSMUSG00000104533	0	0	0	0	0	0
ENSMUSG00000113738	0	0	0	0	0	0
ENSMUSG00000061937	0	0	0	0	0	0
ENSMUSG00000088083	0	0	0	0	0	0
ENSMUSG00000088084	0	0	0	0	0	0
ENSMUSG00000104531	0	0	0	0	0	0
ENSMUSG00000088332	2	1	2	1	3	2
ENSMUSG00000087505	0	0	0	0	0	2
ENSMUSG00000104530	0	1	0	0	0	3
ENSMUSG00000088337	0	0	0	0	0	0
ENSMUSG00000088334	0	0	0	0	0	0
ENSMUSG00000104537	0	0	0	0	0	0
ENSMUSG00000008461	0	0	0	0	0	0
ENSMUSG00000104536	0	0	0	0	0	0
ENSMUSG00000064213	0	0	0	0	0	0
ENSMUSG00000101794	0	0	0	0	0	0
ENSMUSG00000065027	0	0	0	0	0	0
ENSMUSG00000022534	9	6	17	9	6	3
ENSMUSG00000022537	509	541	520	332	471	516
ENSMUSG00000107619	0	12	11	4	0	6
ENSMUSG00000107618	0	0	0	0	0	0
ENSMUSG00000108245	0	0	0	0	0	0
ENSMUSG00000107612	0	0	0	0	0	0
ENSMUSG00000084508	0	0	0	0	0	0
ENSMUSG00000107616	3	0	2	0	0	0
ENSMUSG00000107615	2	0	0	0	0	0
ENSMUSG00000107614	0	0	0	0	0	0
ENSMUSG00000103108	0	0	0	0	0	0
ENSMUSG00000103102	0	0	0	0	0	0
ENSMUSG00000103107	0	0	0	0	0	0
ENSMUSG00000103106	0	0	0	0	0	0
ENSMUSG00000103105	0	0	0	0	3	0
ENSMUSG00000106323	0	1	1	1	0	0
ENSMUSG00000106324	0	0	0	0	0	0
ENSMUSG00000061065	0	0	0	0	0	0
ENSMUSG00000107947	3	0	0	0	0	0
ENSMUSG00000106327	0	0	0	0	0	0
ENSMUSG00000009739	374	410	459	323	377	483
ENSMUSG00000089060	0	0	0	0	0	0
ENSMUSG00000089062	0	0	0	0	0	0
ENSMUSG00000089065	0	0	0	0	0	0
ENSMUSG00000089066	0	0	0	0	0	0
ENSMUSG00000009731	34	20	33	6	22	24
ENSMUSG00000089068	0	0	0	0	0	0
ENSMUSG00000009734	0	0	2	0	0	2
ENSMUSG00000020000	13	12	30	22	15	27
ENSMUSG00000106059	0	0	2	0	0	0
ENSMUSG00000020007	0	0	0	0	0	0

ENSMUSG00000106052	0	0	0	0	0	0
ENSMUSG00000106053	0	0	0	0	0	0
ENSMUSG00000106050	0	0	0	0	0	0
ENSMUSG00000106051	0	0	0	0	0	0
ENSMUSG00000106056	0	0	0	0	0	0
ENSMUSG00000098534	0	0	0	0	0	0
ENSMUSG00000035498	13	10	32	14	9	12
ENSMUSG00000094269	0	0	0	0	0	0
ENSMUSG00000112354	0	0	0	0	0	0
ENSMUSG00000094264	0	0	0	0	0	0
ENSMUSG00000035491	2	0	0	0	0	0
ENSMUSG00000094266	0	2	0	0	0	0
ENSMUSG00000094261	1	1	1	0	0	0
ENSMUSG00000112351	0	0	1	0	0	0
ENSMUSG00000112353	0	0	0	0	0	0
ENSMUSG00000098536	1	3	1	6	1	8
ENSMUSG00000110266	0	0	2	0	0	0
ENSMUSG00000110267	0	0	0	0	0	0
ENSMUSG00000113089	0	0	0	0	0	0
ENSMUSG00000110265	0	0	2	0	0	0
ENSMUSG00000110262	2	0	0	0	0	0
ENSMUSG00000110263	3	0	0	0	0	0
ENSMUSG00000028589	0	0	0	0	0	0
ENSMUSG00000110261	0	0	2	2	0	0
ENSMUSG00000092142	0	0	0	0	0	0
ENSMUSG00000113082	0	0	0	0	0	0
ENSMUSG00000113081	0	0	0	3	0	0
ENSMUSG00000028584	0	0	0	0	0	0
ENSMUSG00000113084	0	2	0	2	0	0
ENSMUSG00000112686	0	0	0	0	0	0
ENSMUSG00000112687	0	0	0	0	0	0
ENSMUSG00000112684	3	5	0	0	4	0
ENSMUSG00000112685	0	0	0	0	0	0
ENSMUSG00000112682	0	4	9	0	2	0
ENSMUSG00000112683	0	0	0	0	0	0
ENSMUSG00000112681	0	0	0	2	0	2
ENSMUSG00000112688	0	0	0	0	0	0
ENSMUSG00000112689	3	2	3	6	1	3
ENSMUSG00000114680	0	0	0	0	0	0
ENSMUSG00000114682	0	0	0	0	0	0
ENSMUSG00000114684	0	0	0	0	0	0
ENSMUSG00000114685	0	0	0	0	0	0
ENSMUSG00000029182	0	0	0	0	0	0
ENSMUSG00000114689	1	4	8	5	0	3
ENSMUSG00000029188	0	0	0	0	0	0
ENSMUSG00000029189	53	39	12	42	37	66
ENSMUSG00000110190	0	0	0	0	0	0
ENSMUSG00000110196	0	0	0	0	0	0
ENSMUSG00000110198	0	0	0	0	0	0
ENSMUSG00000110199	0	0	0	0	0	0
ENSMUSG00000088857	2	0	1	0	0	0
ENSMUSG00000109318	0	0	0	0	0	0
ENSMUSG00000088854	0	0	0	0	0	0
ENSMUSG00000088852	0	0	0	0	0	0
ENSMUSG00000088851	0	0	0	0	0	0
ENSMUSG00000088850	0	1	2	0	2	2
ENSMUSG00000088858	0	0	0	0	0	0
ENSMUSG00000094841	0	0	0	0	0	0

ENSMUSG00000105508	0	0	0	0	0	0
ENSMUSG00000109289	0	0	0	0	0	0
ENSMUSG00000094847	0	0	0	0	0	0
ENSMUSG00000094846	0	0	0	0	0	0
ENSMUSG00000105501	0	0	0	0	0	0
ENSMUSG00000105500	11	10	5	11	7	10
ENSMUSG00000109280	0	0	0	0	0	0
ENSMUSG00000105502	22	8	6	2	5	4
ENSMUSG00000105505	0	0	0	0	0	0
ENSMUSG00000109287	0	0	0	0	0	0
ENSMUSG00000105507	0	0	0	0	0	0
ENSMUSG00000109285	0	0	0	0	0	0
ENSMUSG00000029236	0	0	0	0	0	0
ENSMUSG00000071047	0	4	0	0	1	0
ENSMUSG00000098278	0	0	0	0	0	0
ENSMUSG00000082819	0	0	0	0	0	0
ENSMUSG00000082818	0	0	0	0	0	0
ENSMUSG00000082815	0	0	0	0	0	0
ENSMUSG00000082814	0	0	0	0	0	0
ENSMUSG00000082817	0	0	0	0	0	0
ENSMUSG00000109312	0	0	0	0	0	0
ENSMUSG00000082812	8	4	11	5	6	12
ENSMUSG00000038777	139	81	76	148	96	137
ENSMUSG00000113551	0	0	0	1	0	2
ENSMUSG00000113553	0	0	0	0	0	0
ENSMUSG00000113559	0	0	0	0	0	0
ENSMUSG00000113558	0	0	0	3	0	5
ENSMUSG00000051313	0	2	0	0	0	0
ENSMUSG00000051424	0	0	0	0	0	0
ENSMUSG00000055961	2	0	0	0	0	0
ENSMUSG00000055960	0	0	0	0	0	0
ENSMUSG00000109869	0	0	0	3	0	0
ENSMUSG00000109867	0	0	0	0	0	0
ENSMUSG00000109860	0	0	0	0	0	0
ENSMUSG00000114026	0	0	0	0	0	0
ENSMUSG00000114027	0	0	0	0	0	0
ENSMUSG00000114020	0	0	0	0	0	0
ENSMUSG00000114021	0	0	0	0	0	0
ENSMUSG00000114022	0	0	0	0	0	0
ENSMUSG00000114028	0	0	0	0	0	0
ENSMUSG00000114029	0	0	0	0	0	0
ENSMUSG00000096913	0	0	0	0	0	0
ENSMUSG00000059639	0	0	6	0	0	0
ENSMUSG00000077158	0	0	0	0	0	0
ENSMUSG00000047959	26	5	30	10	10	15
ENSMUSG00000020583	28	26	10	0	0	0
ENSMUSG00000104362	0	0	0	0	0	0
ENSMUSG00000104361	0	1	2	2	4	3
ENSMUSG00000104366	0	0	0	0	0	0
ENSMUSG00000077152	0	0	0	0	0	0
ENSMUSG00000104364	0	0	0	0	0	0
ENSMUSG00000104365	2	0	0	3	0	3
ENSMUSG00000077153	0	0	0	0	0	0
ENSMUSG00000094702	0	1	0	1	0	0
ENSMUSG00000093572	0	0	0	0	0	0
ENSMUSG00000094701	0	0	0	0	0	0
ENSMUSG00000054618	6	15	17	6	2	0
ENSMUSG00000056617	0	0	0	0	1	0

ENSMUSG00000090179	3	1	0	2	4	0
ENSMUSG00000039653	4	2	8	0	0	0
ENSMUSG00000090176	0	0	0	0	0	0
ENSMUSG00000044798	0	0	0	0	0	0
ENSMUSG00000092998	0	0	0	0	0	0
ENSMUSG00000114886	0	2	0	6	4	0
ENSMUSG00000092997	0	0	0	0	0	0
ENSMUSG00000024256	0	0	0	0	0	0
ENSMUSG00000024254	8	0	6	0	0	0
ENSMUSG00000080508	0	0	0	0	0	0
ENSMUSG00000074876	55	40	57	38	32	70
ENSMUSG00000074877	0	1	1	1	1	1
ENSMUSG00000074872	0	0	0	0	0	0
ENSMUSG00000074870	0	0	0	0	0	0
ENSMUSG00000074871	0	0	0	0	0	0
ENSMUSG00000045441	166	90	96	44	40	64
ENSMUSG00000045440	10	12	15	12	17	20
ENSMUSG00000098368	0	0	0	0	0	0
ENSMUSG00000098365	0	0	0	0	2	0
ENSMUSG00000098366	0	0	0	0	0	0
ENSMUSG00000098360	0	0	0	0	0	0
ENSMUSG00000053391	0	0	0	0	0	0
ENSMUSG00000100132	0	0	0	0	0	0
ENSMUSG00000053395	0	0	0	0	1	0
ENSMUSG00000110487	0	0	0	0	0	0
ENSMUSG00000053399	0	0	0	0	0	0
ENSMUSG00000108893	0	0	0	0	0	0
ENSMUSG00000093484	9	0	0	13	0	0
ENSMUSG00000089441	0	0	0	0	0	0
ENSMUSG00000093487	0	0	0	0	0	0
ENSMUSG00000086906	0	0	0	0	0	0
ENSMUSG00000086903	0	0	0	0	0	0
ENSMUSG00000086902	0	0	0	0	0	0
ENSMUSG00000086900	3	0	0	0	4	0
ENSMUSG00000095674	1	0	0	0	0	0
ENSMUSG00000095671	0	0	0	0	0	0
ENSMUSG00000089445	0	0	0	0	0	0
ENSMUSG00000095673	0	0	1	0	0	0
ENSMUSG00000090284	0	0	0	0	0	0
ENSMUSG00000089444	0	0	0	0	0	0
ENSMUSG00000095678	0	0	0	0	0	0
ENSMUSG00000047171	22	31	12	27	31	71
ENSMUSG00000097752	0	0	0	0	0	0
ENSMUSG00000094107	0	0	0	0	0	0
ENSMUSG00000093738	0	0	0	0	0	0
ENSMUSG00000049916	0	0	2	0	0	0
ENSMUSG00000097753	0	0	0	0	0	0
ENSMUSG00000002266	58	91	94	76	82	118
ENSMUSG00000114719	0	0	0	0	0	0
ENSMUSG00000081674	0	2	0	0	2	2
ENSMUSG00000081675	14	16	12	18	21	24
ENSMUSG00000081676	0	0	0	0	0	0
ENSMUSG00000081670	9	22	16	25	20	14
ENSMUSG00000081672	1	0	1	4	3	2
ENSMUSG00000064795	0	0	0	0	0	0
ENSMUSG00000081679	2	3	4	1	3	4
ENSMUSG00000077757	0	0	0	0	0	0
ENSMUSG00000077755	0	0	0	0	0	0

ENSMUSG00000077752	0	0	0	0	0	0
ENSMUSG00000077750	0	0	0	0	0	0
ENSMUSG00000114881	0	0	0	0	0	0
ENSMUSG00000022945	49	31	46	22	32	46
ENSMUSG00000114711	1	1	0	0	0	0
ENSMUSG00000055571	0	0	0	0	0	0
ENSMUSG00000099636	0	0	0	0	0	0
ENSMUSG00000102704	75	59	65	38	35	63
ENSMUSG00000111163	28	8	14	12	20	0
ENSMUSG00000090827	7	13	16	7	15	13
ENSMUSG00000090824	0	0	0	0	0	0
ENSMUSG00000099633	0	0	0	0	0	0
ENSMUSG00000090821	0	0	0	0	0	0
ENSMUSG00000092461	0	0	0	0	0	0
ENSMUSG00000092460	0	0	0	0	0	0
ENSMUSG00000092462	0	0	0	0	0	0
ENSMUSG00000092464	0	0	0	0	0	0
ENSMUSG00000092467	0	0	0	0	0	0
ENSMUSG00000092466	0	0	0	0	0	0
ENSMUSG00000092469	0	0	0	0	0	0
ENSMUSG00000092468	0	0	0	0	0	0
ENSMUSG00000112168	0	0	0	0	0	0
ENSMUSG00000043468	3	0	8	14	7	3
ENSMUSG00000043461	0	0	0	0	0	0
ENSMUSG00000043460	13	16	4	17	29	25
ENSMUSG00000082370	0	0	0	0	0	0
ENSMUSG00000082371	0	0	0	0	0	0
ENSMUSG00000030724	12	11	46	22	14	24
ENSMUSG00000082373	0	0	0	0	0	0
ENSMUSG00000030722	115	125	140	113	102	116
ENSMUSG00000082378	0	0	0	0	0	0
ENSMUSG00000110621	0	0	0	0	0	0
ENSMUSG00000048356	2	0	0	0	0	0
ENSMUSG00000095048	0	0	0	0	0	0
ENSMUSG00000111166	0	0	0	0	0	0
ENSMUSG00000112167	1	0	0	0	0	0
ENSMUSG00000084063	0	0	0	0	0	0
ENSMUSG00000032473	0	0	0	0	0	0
ENSMUSG00000112165	0	0	0	0	0	0
ENSMUSG00000090036	7	15	5	6	10	6
ENSMUSG00000045678	3	0	0	1	0	0
ENSMUSG00000072708	0	0	0	0	0	0
ENSMUSG00000072709	0	0	0	0	0	0
ENSMUSG00000072707	0	0	0	0	0	1
ENSMUSG00000100106	0	0	0	0	0	0
ENSMUSG00000100107	0	0	0	0	0	0
ENSMUSG00000082594	0	0	0	0	0	3
ENSMUSG00000082595	0	0	0	0	0	0
ENSMUSG00000082590	0	0	0	0	0	0
ENSMUSG00000082591	0	0	0	0	0	0
ENSMUSG00000082593	0	1	0	1	1	1
ENSMUSG00000070702	0	0	0	0	0	0
ENSMUSG00000070704	0	0	0	0	0	0
ENSMUSG00000064052	0	0	0	0	0	0
ENSMUSG00000108047	2	0	0	0	0	0
ENSMUSG00000084439	0	0	0	0	0	0
ENSMUSG00000083428	0	0	0	0	0	0
ENSMUSG00000083427	0	0	0	0	0	0

ENSMUSG00000083423	0	0	0	0	0	0
ENSMUSG00000083422	0	0	0	0	0	0
ENSMUSG00000083420	0	0	0	0	0	0
ENSMUSG00000047842	20	26	10	3	5	8
ENSMUSG00000049008	0	3	7	0	6	5
ENSMUSG00000031927	0	0	0	0	0	0
ENSMUSG00000085748	0	5	4	3	0	3
ENSMUSG00000080998	2	2	0	1	0	2
ENSMUSG00000085744	0	0	0	0	0	0
ENSMUSG00000085745	0	2	2	0	0	0
ENSMUSG00000080994	19	27	27	27	15	30
ENSMUSG00000080995	0	0	0	0	0	0
ENSMUSG00000085740	0	0	0	0	0	0
ENSMUSG00000093610	0	0	0	0	0	0
ENSMUSG00000085742	8	6	8	8	0	8
ENSMUSG00000085743	12	0	5	8	7	6
ENSMUSG00000107459	0	0	0	0	0	0
ENSMUSG00000037188	0	6	13	0	3	0
ENSMUSG00000107450	0	0	0	0	0	0
ENSMUSG00000037185	49	49	81	8	9	29
ENSMUSG00000107452	0	0	0	0	0	0
ENSMUSG00000107457	0	0	0	0	0	0
ENSMUSG00000112234	0	0	0	0	0	0
ENSMUSG00000114839	0	0	0	0	0	0
ENSMUSG00000075286	0	0	0	0	0	0
ENSMUSG00000084065	0	0	0	0	0	0
ENSMUSG00000111169	0	0	0	0	0	0
ENSMUSG00000057381	0	0	0	0	0	0
ENSMUSG00000083053	0	0	0	0	0	0
ENSMUSG00000084293	0	0	0	0	0	0
ENSMUSG00000084295	0	0	0	0	0	0
ENSMUSG00000084294	0	0	0	0	0	0
ENSMUSG00000084297	0	0	0	0	0	0
ENSMUSG00000084296	0	0	0	0	0	0
ENSMUSG00000084298	0	0	0	0	0	0
ENSMUSG00000099819	0	0	0	0	0	0
ENSMUSG00000065875	0	0	0	0	2	0
ENSMUSG00000075864	0	0	0	0	0	0
ENSMUSG00000065012	0	0	0	0	0	0
ENSMUSG00000065011	0	0	0	0	0	0
ENSMUSG00000065018	0	0	2	0	0	0
ENSMUSG00000099190	0	0	0	0	0	0
ENSMUSG00000099191	0	0	0	0	0	0
ENSMUSG00000099198	0	0	0	0	0	0
ENSMUSG00000082060	0	0	0	0	0	0
ENSMUSG00000001773	4	6	0	0	0	0
ENSMUSG00000077487	0	0	0	0	0	0
ENSMUSG00000025128	16	30	15	18	17	17
ENSMUSG00000077485	0	0	0	0	0	0
ENSMUSG00000104810	0	3	0	0	0	0
ENSMUSG00000073420	0	0	0	0	0	0
ENSMUSG00000073424	0	0	0	0	2	5
ENSMUSG00000112431	3	10	4	4	1	5
ENSMUSG00000073427	119	256	161	120	79	126
ENSMUSG00000041141	0	0	0	0	0	3
ENSMUSG00000086163	0	0	0	0	0	0
ENSMUSG00000041147	247	334	340	218	241	332
ENSMUSG00000035121	92	89	82	78	54	115

ENSMUSG00000089479	0	0	0	0	0	0
ENSMUSG00000059690	0	0	0	0	0	0
ENSMUSG00000062245	3	0	0	0	0	0
ENSMUSG00000089472	0	0	0	0	0	0
ENSMUSG00000089473	0	0	2	0	0	0
ENSMUSG00000089470	0	0	0	0	0	0
ENSMUSG00000089471	0	0	0	0	0	0
ENSMUSG00000087245	0	0	0	0	0	0
ENSMUSG00000087244	0	0	0	0	0	0
ENSMUSG00000079476	0	0	0	0	0	0
ENSMUSG00000088212	3	0	2	4	3	3
ENSMUSG00000051920	0	0	0	0	0	0
ENSMUSG00000102242	0	0	0	0	0	0
ENSMUSG00000102240	0	0	0	0	0	0
ENSMUSG00000102247	0	0	0	0	0	0
ENSMUSG00000102246	0	3	0	0	0	8
ENSMUSG00000102244	0	0	0	0	0	0
ENSMUSG00000102214	0	0	0	0	0	0
ENSMUSG00000102215	0	0	0	0	0	0
ENSMUSG00000099911	0	0	0	0	0	0
ENSMUSG00000058523	0	0	0	0	0	0
ENSMUSG00000102216	0	0	0	0	0	0
ENSMUSG00000038572	0	1	0	0	2	0
ENSMUSG00000102217	0	0	0	0	0	0
ENSMUSG00000038576	4	6	8	9	3	3
ENSMUSG00000093398	0	0	0	0	0	0
ENSMUSG00000038578	35	48	87	39	35	63
ENSMUSG00000081399	1	0	0	0	0	0
ENSMUSG00000046828	2	0	0	0	0	0
ENSMUSG00000096753	16	10	8	10	13	6
ENSMUSG00000096750	0	0	0	0	0	0
ENSMUSG00000096757	0	0	0	0	0	0
ENSMUSG00000096759	0	0	0	0	0	1
ENSMUSG00000088391	0	0	0	0	0	0
ENSMUSG00000031118	0	0	0	0	0	0
ENSMUSG00000050711	61	52	118	0	0	0
ENSMUSG00000063116	4	0	0	1	0	0
ENSMUSG00000104791	0	0	0	0	0	0
ENSMUSG00000091012	0	0	0	0	0	0
ENSMUSG00000021342	0	0	0	0	0	0
ENSMUSG00000021346	0	0	0	0	0	0
ENSMUSG00000021347	0	0	0	0	0	0
ENSMUSG00000102867	0	0	0	0	0	0
ENSMUSG00000102866	0	0	0	0	0	0
ENSMUSG00000102865	0	2	1	3	1	1
ENSMUSG00000102860	0	0	0	0	0	0
ENSMUSG00000057726	0	0	0	0	0	0
ENSMUSG00000057723	0	0	0	0	0	0
ENSMUSG00000057722	139	186	196	329	269	399
ENSMUSG00000066897	1	0	0	0	0	0
ENSMUSG00000066896	2	0	5	2	1	0
ENSMUSG00000066899	0	0	0	0	0	0
ENSMUSG00000113458	0	0	0	0	0	0
ENSMUSG00000104162	0	0	0	0	0	0
ENSMUSG00000104163	0	0	0	0	0	0
ENSMUSG00000104167	3	3	0	0	0	2
ENSMUSG00000104169	0	0	0	0	0	0
ENSMUSG00000044835	123	128	97	63	64	137

ENSMUSG00000021907	0	0	0	0	0	0
ENSMUSG00000055170	0	3	0	0	0	0
ENSMUSG00000113459	0	0	0	2	7	3
ENSMUSG00000082362	0	0	3	0	3	1
ENSMUSG00000060499	0	0	0	0	0	0
ENSMUSG00000060491	0	0	0	0	0	0
ENSMUSG00000060490	0	0	0	0	0	0
ENSMUSG00000091195	0	0	0	0	0	0
ENSMUSG00000094934	0	0	0	0	0	0
ENSMUSG00000078940	0	0	0	0	0	0
ENSMUSG00000104839	0	0	0	0	0	0
ENSMUSG00000104837	0	0	0	0	0	0
ENSMUSG00000104835	4	11	11	0	0	0
ENSMUSG00000081197	0	0	0	0	0	0
ENSMUSG00000086629	176	194	171	146	160	171
ENSMUSG00000088276	0	0	0	0	0	0
ENSMUSG00000088275	0	0	0	0	0	0
ENSMUSG00000095029	4	1	2	0	0	0
ENSMUSG00000058063	0	0	0	0	0	0
ENSMUSG00000088279	0	0	0	0	0	0
ENSMUSG00000013353	0	0	0	0	0	0
ENSMUSG00000081191	0	0	3	0	0	0
ENSMUSG00000095470	0	0	0	0	0	0
ENSMUSG00000095477	3	1	1	2	0	4
ENSMUSG00000095476	0	0	0	0	0	0
ENSMUSG00000095475	0	0	0	0	0	0
ENSMUSG00000095474	0	0	0	6	0	0
ENSMUSG00000095479	0	1	0	0	0	0
ENSMUSG00000098795	0	0	0	0	0	0
ENSMUSG00000016327	4	11	0	6	10	5
ENSMUSG00000022705	3	0	0	0	0	0
ENSMUSG00000093134	0	0	0	0	0	0
ENSMUSG00000002068	44	45	57	25	63	41
ENSMUSG00000106462	0	0	0	0	0	0
ENSMUSG00000028280	12	10	5	27	11	12
ENSMUSG00000023257	0	0	0	0	0	0
ENSMUSG00000028287	0	0	0	0	0	0
ENSMUSG00000112529	0	0	0	0	0	0
ENSMUSG00000112523	4	4	5	2	4	2
ENSMUSG00000112522	3	1	3	2	4	3
ENSMUSG00000112520	0	0	0	0	0	0
ENSMUSG00000112527	0	0	2	0	0	0
ENSMUSG00000112526	0	0	0	0	0	0
ENSMUSG00000112525	0	0	2	0	0	0
ENSMUSG00000112524	0	0	0	0	0	0
ENSMUSG00000017639	23	22	31	18	18	25
ENSMUSG00000029882	0	0	4	0	0	0
ENSMUSG00000100903	0	0	0	0	0	0
ENSMUSG00000106159	0	0	0	0	0	0
ENSMUSG00000106157	32	49	46	18	54	73
ENSMUSG00000106156	0	0	2	0	0	0
ENSMUSG00000106155	0	0	0	0	0	0
ENSMUSG00000106154	0	0	0	0	0	0
ENSMUSG00000048644	6	8	16	10	26	28
ENSMUSG00000106150	0	0	0	0	0	0
ENSMUSG00000092269	0	0	0	0	0	0
ENSMUSG00000092265	0	0	0	0	0	0
ENSMUSG00000092264	0	0	0	0	0	0

ENSMUSG00000092263	0	0	0	0	0	0
ENSMUSG00000063777	0	0	0	0	0	0
ENSMUSG00000094184	0	0	0	0	0	0
ENSMUSG00000077180	0	0	0	0	1	0
ENSMUSG00000087677	0	0	0	0	0	2
ENSMUSG00000020633	0	0	0	0	0	0
ENSMUSG00000020636	0	0	0	0	0	0
ENSMUSG00000020639	47	83	46	47	66	69
ENSMUSG00000110015	0	0	0	0	0	0
ENSMUSG00000110014	1	1	0	1	1	1
ENSMUSG00000097169	0	0	0	0	0	0
ENSMUSG00000110016	0	0	0	0	0	0
ENSMUSG00000110011	0	0	0	0	0	0
ENSMUSG00000110013	0	0	0	0	0	0
ENSMUSG00000110012	0	0	0	0	0	2
ENSMUSG00000097163	0	0	3	0	0	3
ENSMUSG00000100906	0	0	0	0	0	0
ENSMUSG00000110019	0	0	0	0	0	0
ENSMUSG00000108479	0	0	0	0	0	0
ENSMUSG00000059251	0	0	0	0	0	0
ENSMUSG00000042157	0	0	0	0	0	0
ENSMUSG00000103045	2	2	4	2	1	2
ENSMUSG00000109127	0	0	0	0	0	0
ENSMUSG00000109125	49	93	14	49	75	96
ENSMUSG00000109124	0	0	0	0	0	0
ENSMUSG00000109123	0	0	0	3	0	0
ENSMUSG00000109122	16	7	9	2	8	10
ENSMUSG00000109121	3	0	0	0	0	0
ENSMUSG00000103043	0	2	0	0	0	0
ENSMUSG00000095024	0	0	0	0	0	0
ENSMUSG00000104112	0	0	0	0	0	0
ENSMUSG00000104115	0	0	0	0	0	0
ENSMUSG00000093072	0	0	0	0	0	0
ENSMUSG00000112776	0	0	0	0	0	0
ENSMUSG00000054135	117	87	102	93	109	97
ENSMUSG00000054134	0	2	2	0	0	2
ENSMUSG00000054136	0	0	0	0	0	0
ENSMUSG00000108296	0	0	0	0	0	0
ENSMUSG00000108297	2	2	0	2	0	0
ENSMUSG00000108295	0	0	0	0	2	0
ENSMUSG00000108293	0	0	3	0	0	1
ENSMUSG00000108290	0	0	0	0	0	0
ENSMUSG00000105718	0	0	0	0	0	0
ENSMUSG00000105712	0	0	0	0	0	0
ENSMUSG00000109049	0	0	0	0	0	0
ENSMUSG00000105715	0	0	0	0	0	0
ENSMUSG00000098939	0	0	0	0	0	0
ENSMUSG00000061165	0	0	0	0	0	0
ENSMUSG00000111859	0	0	0	0	0	0
ENSMUSG00000108748	0	0	0	2	0	0
ENSMUSG00000108742	0	0	0	0	0	0
ENSMUSG00000108743	0	0	0	0	0	0
ENSMUSG00000108744	0	0	0	0	0	0
ENSMUSG00000108745	0	0	0	0	0	0
ENSMUSG00000108746	0	0	0	0	0	0
ENSMUSG00000111764	0	0	0	0	0	0
ENSMUSG00000111765	25	44	16	108	67	140
ENSMUSG00000111766	0	0	0	0	0	0

ENSMUSG00000111767	0	0	2	9	0	2
ENSMUSG00000111760	0	0	0	0	0	0
ENSMUSG00000111762	0	0	0	0	0	0
ENSMUSG00000111763	0	0	0	0	0	0
ENSMUSG00000094183	0	0	0	0	0	0
ENSMUSG00000057444	3	0	0	0	0	0
ENSMUSG00000057446	0	0	0	0	0	0
ENSMUSG00000057447	3	0	0	0	0	0
ENSMUSG00000057443	0	0	0	0	0	0
ENSMUSG00000025945	0	0	0	0	0	0
ENSMUSG00000025946	0	0	0	1	0	0
ENSMUSG00000104446	0	0	0	0	0	0
ENSMUSG00000104442	0	0	0	0	0	0
ENSMUSG00000104441	0	0	0	0	0	0
ENSMUSG00000104440	0	0	0	0	0	0
ENSMUSG00000101618	0	0	0	0	0	0
ENSMUSG00000077741	0	0	0	0	0	0
ENSMUSG00000111852	0	0	0	0	0	0
ENSMUSG00000097609	0	0	0	0	0	0
ENSMUSG00000081539	0	0	0	0	0	0
ENSMUSG00000073972	6	7	6	2	0	0
ENSMUSG00000088833	0	0	0	0	0	0
ENSMUSG00000073970	0	0	0	2	0	0
ENSMUSG00000078262	0	0	0	0	0	0
ENSMUSG00000077743	0	0	0	0	0	0
ENSMUSG00000104703	2	0	0	0	0	0
ENSMUSG00000078261	0	0	0	0	0	0
ENSMUSG00000077415	0	0	0	0	0	0
ENSMUSG00000078260	0	0	0	0	0	0
ENSMUSG00000113981	0	0	0	0	2	0
ENSMUSG00000073974	20	2	13	7	7	15
ENSMUSG00000113986	0	0	0	0	0	0
ENSMUSG00000104707	0	0	0	0	0	0
ENSMUSG00000078161	39	34	42	128	111	113
ENSMUSG00000103028	0	0	0	0	0	0
ENSMUSG00000109042	0	0	0	0	0	0
ENSMUSG00000090328	0	0	0	1	0	1
ENSMUSG00000112290	0	0	0	0	0	0
ENSMUSG00000082681	0	0	0	0	0	0
ENSMUSG00000090323	1	2	1	1	1	1
ENSMUSG00000090326	0	0	0	0	4	0
ENSMUSG00000109043	0	0	0	0	0	0
ENSMUSG00000048965	53	53	59	39	50	72
ENSMUSG00000053217	0	0	0	0	0	0
ENSMUSG00000053216	0	0	0	0	0	0
ENSMUSG00000053211	0	0	0	0	0	0
ENSMUSG00000111896	0	0	0	0	0	0
ENSMUSG00000046109	0	0	0	0	0	0
ENSMUSG00000080536	0	0	0	0	0	0
ENSMUSG00000101712	0	0	0	0	0	0
ENSMUSG00000075132	0	1	1	0	0	1
ENSMUSG00000073158	35	33	38	9	24	29
ENSMUSG00000080714	0	0	0	0	0	0
ENSMUSG00000080716	0	0	0	0	0	0
ENSMUSG00000080710	0	3	0	0	0	0
ENSMUSG00000080713	0	0	0	0	0	0
ENSMUSG00000075133	0	0	0	0	0	0
ENSMUSG00000080719	0	0	0	0	0	0

ENSMUSG00000114482	0	0	0	0	0	0
ENSMUSG00000093675	6	2	0	0	0	0
ENSMUSG00000081365	0	0	0	0	0	0
ENSMUSG00000093673	3	3	2	3	4	0
ENSMUSG00000086719	0	0	0	0	0	0
ENSMUSG00000043050	0	0	0	0	0	0
ENSMUSG00000086712	10	26	10	24	19	37
ENSMUSG00000086713	2	0	1	0	0	0
ENSMUSG00000086710	0	0	0	0	0	0
ENSMUSG00000086711	0	0	0	0	0	0
ENSMUSG00000106132	0	0	0	0	0	0
ENSMUSG00000095629	0	0	0	0	0	0
ENSMUSG00000098665	0	0	0	0	0	0
ENSMUSG00000098667	0	0	0	0	2	0
ENSMUSG00000077381	0	0	0	2	0	0
ENSMUSG00000077383	0	0	0	0	0	2
ENSMUSG00000098669	0	0	0	1	0	0
ENSMUSG00000077384	0	0	0	0	0	0
ENSMUSG00000077387	0	4	4	1	4	4
ENSMUSG00000049708	0	0	0	0	0	0
ENSMUSG00000114489	0	0	0	0	0	0
ENSMUSG00000033268	30	21	47	25	35	38
ENSMUSG00000031682	0	0	0	0	4	3
ENSMUSG00000109200	0	0	0	0	0	0
ENSMUSG00000037683	0	0	0	6	0	0
ENSMUSG00000037686	17	25	9	2	12	1
ENSMUSG00000099854	0	0	0	0	0	0
ENSMUSG00000037689	0	0	0	0	0	0
ENSMUSG00000031202	45	28	34	10	15	20
ENSMUSG00000099682	0	0	0	0	0	0
ENSMUSG00000099850	0	0	0	0	3	0
ENSMUSG00000039059	0	0	0	3	0	0
ENSMUSG00000077427	0	0	0	0	0	0
ENSMUSG00000094367	7	7	4	1	3	3
ENSMUSG00000101468	0	0	0	0	0	0
ENSMUSG00000101469	0	0	0	0	0	0
ENSMUSG00000101462	24	17	13	11	9	39
ENSMUSG00000101463	0	0	0	0	0	0
ENSMUSG00000040502	103	113	145	108	96	128
ENSMUSG00000101466	0	0	0	0	0	0
ENSMUSG00000101467	0	0	0	0	0	0
ENSMUSG00000101464	0	0	0	0	0	0
ENSMUSG00000101465	0	0	0	0	0	0
ENSMUSG00000099530	0	0	0	0	0	0
ENSMUSG00000099531	0	0	0	0	0	0
ENSMUSG00000054069	0	0	0	0	0	0
ENSMUSG00000030553	13	6	10	4	0	4
ENSMUSG00000087394	4	0	0	0	0	4
ENSMUSG00000099535	0	0	0	0	0	0
ENSMUSG00000030559	2	0	0	7	5	0
ENSMUSG00000087390	19	24	24	29	26	37
ENSMUSG00000087393	0	0	0	0	0	0
ENSMUSG00000099223	1	0	2	0	0	1
ENSMUSG00000037025	0	0	0	0	0	0
ENSMUSG00000083674	43	47	49	39	56	54
ENSMUSG00000089251	0	0	0	0	0	0
ENSMUSG00000037028	0	0	0	0	0	0
ENSMUSG00000083673	0	0	0	0	0	0

ENSMUSG00000083671	0	0	0	0	0	0
ENSMUSG00000100739	0	0	0	0	0	0
ENSMUSG00000100731	0	0	0	0	0	0
ENSMUSG00000100730	0	0	0	0	0	0
ENSMUSG00000100733	0	0	0	0	0	0
ENSMUSG00000100732	0	0	0	0	0	0
ENSMUSG00000100734	0	0	0	0	0	0
ENSMUSG00000100737	0	0	0	0	0	0
ENSMUSG00000100736	1	2	0	2	0	2
ENSMUSG00000096430	1	0	0	0	0	0
ENSMUSG00000026424	14	0	0	20	17	43
ENSMUSG00000102462	0	0	0	0	0	0
ENSMUSG00000102467	0	0	0	0	0	1
ENSMUSG00000102465	0	3	0	0	0	0
ENSMUSG00000102464	0	0	0	0	0	0
ENSMUSG00000096431	0	0	0	0	0	0
ENSMUSG00000102469	1	0	0	0	0	0
ENSMUSG00000084856	0	0	0	0	0	0
ENSMUSG00000000308	29	17	47	0	0	3
ENSMUSG00000075128	0	0	0	0	0	0
ENSMUSG00000000303	7	0	2	0	0	0
ENSMUSG00000075126	0	0	0	0	0	0
ENSMUSG00000000301	33	38	43	14	29	29
ENSMUSG00000075121	0	0	0	0	0	0
ENSMUSG00000075120	0	0	0	0	0	0
ENSMUSG00000087973	0	0	0	0	0	0
ENSMUSG00000087975	0	0	0	0	0	0
ENSMUSG00000087979	0	0	0	0	0	0
ENSMUSG00000102287	0	5	0	0	0	4
ENSMUSG00000102286	0	0	0	0	0	0
ENSMUSG00000085533	0	0	0	0	0	0
ENSMUSG00000085532	0	0	0	3	2	0
ENSMUSG00000085531	0	0	0	0	0	0
ENSMUSG00000085530	0	0	0	0	2	0
ENSMUSG00000085536	0	0	0	0	0	0
ENSMUSG00000085534	0	0	0	0	0	0
ENSMUSG00000064572	0	0	0	0	0	0
ENSMUSG00000064573	0	0	0	0	0	0
ENSMUSG00000064570	0	0	0	0	0	0
ENSMUSG00000064577	0	0	0	0	0	0
ENSMUSG00000064574	0	0	0	0	0	0
ENSMUSG00000064575	0	0	0	0	0	0
ENSMUSG00000046590	0	0	0	0	0	0
ENSMUSG00000064579	0	0	0	0	0	0
ENSMUSG00000074292	4	0	0	0	0	0
ENSMUSG00000074291	0	0	0	0	0	0
ENSMUSG00000109730	0	0	0	0	0	0
ENSMUSG00000098725	1	1	1	1	1	1
ENSMUSG00000023011	43	16	41	0	0	4
ENSMUSG00000023015	129	138	152	35	81	116
ENSMUSG00000067882	0	0	7	0	2	0
ENSMUSG00000088660	0	0	0	0	0	0
ENSMUSG00000104807	0	0	0	0	0	0
ENSMUSG00000065195	0	0	0	0	0	0
ENSMUSG00000088663	0	0	0	0	0	0
ENSMUSG00000065193	0	0	2	0	0	0
ENSMUSG00000065192	0	0	0	0	0	0
ENSMUSG00000088666	0	0	0	0	0	0

ENSMUSG00000104806	3	3	2	3	5	3
ENSMUSG00000088669	1	0	0	0	0	0
ENSMUSG00000109738	0	0	0	0	0	0
ENSMUSG00000109326	0	0	0	7	6	0
ENSMUSG00000039540	0	0	0	0	0	0
ENSMUSG00000039543	2	0	0	0	2	0
ENSMUSG00000039546	0	3	0	0	0	6
ENSMUSG00000107173	9	8	11	6	6	6
ENSMUSG00000107177	0	0	0	0	0	0
ENSMUSG00000107178	21	18	10	15	11	14
ENSMUSG00000065221	0	0	0	0	0	0
ENSMUSG00000065220	0	0	0	0	0	0
ENSMUSG00000065223	0	0	0	0	0	0
ENSMUSG00000034648	40	44	42	11	28	55
ENSMUSG00000034645	0	2	0	0	0	2
ENSMUSG00000104809	0	0	0	0	0	0
ENSMUSG00000078442	0	0	0	0	0	0
ENSMUSG00000024546	0	0	0	0	0	0
ENSMUSG00000029716	10	7	22	0	0	0
ENSMUSG00000029712	1	0	2	0	0	0
ENSMUSG00000103411	4	3	6	7	11	14
ENSMUSG00000103413	0	0	0	0	0	0
ENSMUSG00000103415	2	2	2	0	0	4
ENSMUSG00000103417	0	0	0	0	0	0
ENSMUSG00000103416	0	0	0	0	0	0
ENSMUSG00000097369	0	8	0	0	5	5
ENSMUSG00000094589	0	0	0	0	0	0
ENSMUSG00000022871	44	15	41	4	29	20
ENSMUSG00000097363	0	0	0	0	0	0
ENSMUSG00000097362	0	0	0	0	3	0
ENSMUSG00000022875	0	0	0	0	0	0
ENSMUSG00000022877	0	0	0	0	0	0
ENSMUSG00000022876	54	67	84	18	22	28
ENSMUSG00000070995	0	0	0	0	0	0
ENSMUSG00000059087	0	0	0	0	0	0
ENSMUSG00000090411	0	0	0	0	0	0
ENSMUSG00000114330	0	0	0	0	0	0
ENSMUSG00000095420	0	0	0	0	0	0
ENSMUSG00000066850	0	0	0	0	0	0
ENSMUSG00000062165	0	0	0	2	0	0
ENSMUSG00000062162	0	0	0	0	0	0
ENSMUSG00000062168	0	4	0	0	0	4
ENSMUSG00000020732	47	49	50	34	33	43
ENSMUSG00000020734	26	33	17	36	55	73
ENSMUSG00000103628	0	0	0	0	0	0
ENSMUSG00000105157	0	0	0	0	0	0
ENSMUSG00000105154	0	0	1	0	0	0
ENSMUSG00000105155	5	0	0	1	3	1
ENSMUSG00000105152	0	0	0	0	0	0
ENSMUSG00000105153	0	0	0	0	0	0
ENSMUSG00000105150	0	0	0	0	0	0
ENSMUSG00000105151	0	0	0	0	0	0
ENSMUSG00000114275	0	0	0	0	0	0
ENSMUSG00000114274	0	0	0	0	0	0
ENSMUSG00000114271	0	0	0	0	0	0
ENSMUSG00000114270	0	0	3	0	0	0
ENSMUSG00000114273	2	0	0	0	0	0
ENSMUSG00000114272	0	0	0	0	0	1

ENSMUSG00000114941	0	0	0	0	0	0
ENSMUSG00000050108	0	0	0	0	0	0
ENSMUSG00000054488	2	0	0	2	4	0
ENSMUSG00000114947	0	0	0	0	0	0
ENSMUSG00000050107	28	40	37	13	35	22
ENSMUSG00000050102	0	0	0	0	0	0
ENSMUSG00000114946	2	1	0	0	0	0
ENSMUSG00000069855	0	0	0	0	0	0
ENSMUSG00000096099	0	0	0	0	0	0
ENSMUSG00000067261	7	6	0	5	2	5
ENSMUSG00000113249	0	0	0	0	0	0
ENSMUSG00000096090	0	0	0	0	0	0
ENSMUSG00000096091	0	0	0	0	0	0
ENSMUSG00000096094	0	0	2	3	0	0
ENSMUSG00000096095	0	0	0	1	0	0
ENSMUSG00000096097	1	1	1	1	1	2
ENSMUSG00000109328	0	0	0	0	0	0
ENSMUSG00000096546	3	9	0	0	0	0
ENSMUSG00000054850	7	10	13	7	15	12
ENSMUSG00000104285	0	0	0	0	3	0
ENSMUSG00000113638	0	0	0	0	0	0
ENSMUSG00000108504	0	0	0	0	0	0
ENSMUSG00000104286	0	0	0	0	0	0
ENSMUSG00000104281	0	2	0	0	0	0
ENSMUSG00000104283	20	38	29	15	41	29
ENSMUSG00000104282	4	0	0	3	0	6
ENSMUSG00000104289	0	0	0	0	0	0
ENSMUSG00000104288	0	0	0	0	0	0
ENSMUSG00000108508	0	0	0	0	0	0
ENSMUSG00000108509	0	0	0	0	0	0
ENSMUSG00000114337	0	0	0	0	0	0
ENSMUSG00000101292	0	0	0	0	0	0
ENSMUSG00000094582	0	0	0	2	0	0
ENSMUSG00000106799	0	0	0	3	0	2
ENSMUSG00000097180	31	32	39	24	36	30
ENSMUSG00000101101	0	0	0	0	0	0
ENSMUSG00000101100	0	0	0	0	0	0
ENSMUSG00000101103	0	0	0	1	0	0
ENSMUSG00000113633	2	0	0	0	0	0
ENSMUSG00000101105	0	0	0	0	0	0
ENSMUSG00000101107	0	0	0	0	0	0
ENSMUSG00000101106	1	0	0	0	0	0
ENSMUSG00000101109	0	0	0	0	0	0
ENSMUSG00000101108	0	0	0	0	0	0
ENSMUSG00000038015	0	0	0	0	0	0
ENSMUSG00000045109	0	0	0	0	0	0
ENSMUSG00000072625	5	0	0	3	0	0
ENSMUSG00000100028	0	0	0	0	0	0
ENSMUSG00000111359	0	0	0	0	0	0
ENSMUSG00000111355	0	3	0	0	0	0
ENSMUSG00000111354	0	0	0	0	0	0
ENSMUSG00000111357	0	0	0	0	0	0
ENSMUSG00000111351	3	0	9	0	3	3
ENSMUSG00000111350	0	0	0	0	0	0
ENSMUSG00000111353	0	2	0	0	0	0
ENSMUSG00000016529	0	0	0	0	5	4
ENSMUSG00000100026	0	0	0	0	0	0
ENSMUSG00000060284	5	0	5	0	2	0

ENSMUSG00000107056	0	0	0	0	0	0
ENSMUSG00000064900	0	0	0	0	0	0
ENSMUSG00000064905	0	0	0	0	0	0
ENSMUSG00000064904	0	0	0	0	0	0
ENSMUSG00000091011	0	0	0	0	0	0
ENSMUSG00000091010	0	0	0	0	0	1
ENSMUSG00000091013	0	0	0	0	0	0
ENSMUSG00000045584	0	0	0	0	0	0
ENSMUSG00000091014	0	0	0	2	0	2
ENSMUSG00000091017	0	2	0	0	0	0
ENSMUSG00000091016	0	0	0	0	0	0
ENSMUSG00000059504	0	0	0	0	0	0
ENSMUSG00000059503	0	0	0	0	0	0
ENSMUSG00000087004	0	0	7	0	0	2
ENSMUSG00000081024	0	4	0	0	0	0
ENSMUSG00000081026	0	0	0	0	0	0
ENSMUSG00000081020	0	0	0	0	0	0
ENSMUSG00000081023	0	2	0	0	2	2
ENSMUSG00000081022	0	0	0	0	0	0
ENSMUSG00000022002	0	0	0	0	0	0
ENSMUSG00000083494	0	0	0	0	0	0
ENSMUSG00000056223	0	0	0	0	0	0
ENSMUSG00000056222	2	0	0	0	0	0
ENSMUSG00000094981	0	0	0	0	1	0
ENSMUSG00000110561	0	0	0	0	0	0
ENSMUSG00000097832	12	5	9	0	10	9
ENSMUSG00000110560	0	0	0	0	0	0
ENSMUSG00000090658	0	0	0	0	0	0
ENSMUSG00000110562	0	0	0	0	0	2
ENSMUSG00000053765	0	0	0	0	0	0
ENSMUSG00000109141	0	0	0	0	0	0
ENSMUSG00000109140	0	0	0	0	0	0
ENSMUSG00000094799	0	0	0	1	0	0
ENSMUSG00000094798	0	1	1	1	1	1
ENSMUSG00000094794	0	0	1	0	0	0
ENSMUSG00000036117	0	13	6	4	11	7
ENSMUSG00000105443	0	0	0	0	0	0
ENSMUSG00000094791	0	0	0	0	0	0
ENSMUSG00000094793	0	0	0	0	0	0
ENSMUSG00000112195	0	0	4	2	0	1
ENSMUSG00000112192	0	0	0	0	0	0
ENSMUSG00000112190	0	0	0	0	0	0
ENSMUSG00000112191	0	0	0	0	0	0
ENSMUSG00000086557	0	0	0	0	0	0
ENSMUSG00000086550	0	0	0	0	0	0
ENSMUSG00000112198	0	0	0	0	0	0
ENSMUSG00000112199	0	0	0	0	0	0
ENSMUSG00000052417	0	0	0	0	0	0
ENSMUSG00000083568	0	0	0	0	0	0
ENSMUSG00000105528	5	0	0	0	0	2
ENSMUSG00000023350	0	0	0	2	0	0
ENSMUSG00000051998	31	13	27	13	7	11
ENSMUSG00000093306	2	1	2	2	0	3
ENSMUSG00000093303	0	0	0	0	0	0
ENSMUSG00000093301	0	0	0	1	1	0
ENSMUSG00000093300	0	0	0	0	0	0
ENSMUSG00000086087	0	0	0	0	0	0
ENSMUSG00000084573	0	0	0	0	0	0

ENSMUSG00000086080	0	0	0	0	0	0
ENSMUSG00000086081	0	0	0	0	1	0
ENSMUSG00000084574	0	0	0	0	0	0
ENSMUSG00000049548	0	0	0	0	0	0
ENSMUSG00000017861	27	23	25	8	13	10
ENSMUSG00000092652	1	1	1	1	1	1
ENSMUSG00000092653	2	1	0	0	0	0
ENSMUSG00000092656	0	0	0	0	0	0
ENSMUSG00000092657	1	0	0	0	0	0
ENSMUSG00000096939	0	0	0	0	0	0
ENSMUSG00000096930	4	0	0	2	4	2
ENSMUSG00000096937	23	24	44	14	17	29
ENSMUSG00000096936	8	8	0	8	4	8
ENSMUSG00000096934	0	0	0	0	0	0
ENSMUSG00000106349	0	0	0	0	0	0
ENSMUSG00000106346	0	0	0	0	0	0
ENSMUSG00000106345	0	0	0	0	0	0
ENSMUSG00000106342	0	0	0	0	0	0
ENSMUSG00000106343	0	0	0	0	0	0
ENSMUSG00000106340	0	0	0	0	0	0
ENSMUSG00000113244	0	0	0	0	0	0
ENSMUSG00000106865	0	0	0	0	0	0
ENSMUSG00000068810	0	0	0	0	0	0
ENSMUSG00000109372	119	144	82	77	79	124
ENSMUSG00000109373	2	0	0	0	0	0
ENSMUSG00000082709	0	0	0	0	0	0
ENSMUSG00000082708	0	0	3	0	1	0
ENSMUSG00000108364	0	0	0	0	0	0
ENSMUSG00000068815	0	0	0	0	0	0
ENSMUSG00000082707	0	0	0	0	0	0
ENSMUSG00000082701	0	0	0	0	0	0
ENSMUSG00000082700	0	0	0	0	0	0
ENSMUSG00000082703	0	0	0	3	0	0
ENSMUSG00000086243	0	0	0	0	0	0
ENSMUSG00000060715	2	4	0	2	3	4
ENSMUSG00000083078	0	0	0	0	0	0
ENSMUSG00000083079	0	0	0	0	0	0
ENSMUSG00000083074	0	0	1	0	0	0
ENSMUSG00000083075	0	0	0	2	2	0
ENSMUSG00000083072	2	0	1	0	5	3
ENSMUSG00000083073	0	0	0	0	0	0
ENSMUSG00000114734	9	6	6	0	0	0
ENSMUSG00000114735	0	0	0	0	0	0
ENSMUSG00000114736	0	0	0	0	0	0
ENSMUSG00000114730	0	0	0	0	0	0
ENSMUSG00000114731	0	0	0	0	0	0
ENSMUSG00000114732	0	0	0	0	0	0
ENSMUSG00000082600	0	2	3	0	0	0
ENSMUSG00000114739	1	0	1	1	0	1
ENSMUSG00000110604	0	4	0	0	0	0
ENSMUSG00000110605	66	51	85	24	23	28
ENSMUSG00000110606	0	0	0	0	0	0
ENSMUSG00000110607	0	0	0	0	0	0
ENSMUSG00000110600	0	0	0	0	0	0
ENSMUSG00000110602	0	0	0	0	0	0
ENSMUSG00000110608	3	1	6	0	2	0
ENSMUSG00000110609	0	0	0	0	0	0
ENSMUSG00000099308	0	0	0	0	0	0

ENSMUSG00000099304	0	0	0	0	1	0
ENSMUSG00000099303	0	0	0	1	0	0
ENSMUSG00000047861	2	0	0	0	0	0
ENSMUSG00000111800	0	0	0	0	0	0
ENSMUSG00000111803	0	0	0	0	0	0
ENSMUSG00000057899	39	39	14	35	43	37
ENSMUSG00000000544	0	0	0	0	0	0
ENSMUSG00000057890	0	0	0	0	0	0
ENSMUSG00000041165	0	0	0	0	0	0
ENSMUSG00000092213	0	0	0	0	0	0
ENSMUSG00000025592	0	0	0	0	0	0
ENSMUSG00000096278	0	0	0	0	0	0
ENSMUSG00000111703	0	0	0	0	0	0
ENSMUSG00000111700	0	0	0	0	0	0
ENSMUSG00000070080	0	0	0	0	0	0
ENSMUSG00000093884	10	20	18	19	35	55
ENSMUSG00000093886	2	0	1	1	1	2
ENSMUSG00000093887	4	2	8	2	4	7
ENSMUSG00000093883	0	0	0	0	1	0
ENSMUSG00000093889	0	0	0	0	0	0
ENSMUSG00000079156	0	0	0	0	0	0
ENSMUSG00000079157	0	0	0	4	0	0
ENSMUSG00000087283	0	0	0	0	0	0
ENSMUSG00000105608	0	0	0	0	0	0
ENSMUSG00000111708	0	0	0	0	0	0
ENSMUSG00000096273	0	0	0	0	0	0
ENSMUSG00000094663	0	0	0	0	0	0
ENSMUSG00000096274	1	0	1	1	0	0
ENSMUSG00000097861	0	0	0	0	0	0
ENSMUSG00000006490	0	0	0	0	0	0
ENSMUSG00000085043	0	7	2	0	0	0
ENSMUSG00000085041	2	0	0	0	0	0
ENSMUSG00000085047	0	0	0	0	0	0
ENSMUSG00000085046	0	0	0	0	0	0
ENSMUSG00000085044	0	0	0	0	0	0
ENSMUSG00000107558	0	2	0	0	0	0
ENSMUSG00000094743	0	0	0	0	0	0
ENSMUSG00000091355	0	0	0	0	0	0
ENSMUSG00000026023	12	0	4	0	0	0
ENSMUSG00000033831	0	0	0	0	0	0
ENSMUSG00000105170	0	0	0	0	0	0
ENSMUSG00000033837	0	0	3	0	0	4
ENSMUSG00000033834	0	0	0	0	0	0
ENSMUSG00000087038	0	0	0	0	0	0
ENSMUSG00000087039	0	0	0	1	0	0
ENSMUSG00000073908	0	0	2	0	0	0
ENSMUSG00000077881	0	0	0	0	0	0
ENSMUSG00000077886	0	0	0	0	0	0
ENSMUSG00000099878	10	8	0	0	0	0
ENSMUSG00000099871	14	4	14	5	0	0
ENSMUSG00000099870	0	2	0	0	0	0
ENSMUSG00000099877	0	0	0	0	0	0
ENSMUSG00000099876	20	17	25	25	14	31
ENSMUSG00000074507	0	0	0	0	0	0
ENSMUSG00000074505	167	144	66	160	143	216
ENSMUSG00000074502	1	1	0	0	0	0
ENSMUSG00000078280	0	0	0	0	0	0
ENSMUSG00000074500	47	40	62	32	28	33

ENSMUSG00000082042	0	0	0	0	0	0
ENSMUSG00000074508	2	8	2	0	0	0
ENSMUSG00000087031	0	0	0	0	0	0
ENSMUSG00000082040	0	1	0	0	0	0
ENSMUSG00000114368	0	0	0	0	0	0
ENSMUSG00000081346	0	0	0	0	0	0
ENSMUSG00000081341	2	0	0	0	0	0
ENSMUSG00000081349	0	0	0	0	0	0
ENSMUSG00000019936	52	24	0	0	0	0
ENSMUSG00000019935	3	0	0	0	0	2
ENSMUSG00000019932	0	0	0	0	0	0
ENSMUSG00000095218	0	0	0	0	0	0
ENSMUSG00000063661	0	0	0	0	0	0
ENSMUSG00000072900	1	0	1	0	0	0
ENSMUSG00000072905	0	0	0	0	0	0
ENSMUSG00000104792	0	0	0	0	0	0
ENSMUSG00000094669	0	0	0	0	0	0
ENSMUSG00000090515	0	0	0	0	0	0
ENSMUSG00000090512	0	0	0	0	0	0
ENSMUSG00000090519	0	0	0	0	0	0
ENSMUSG00000038925	0	0	0	0	0	0
ENSMUSG00000077268	0	0	0	0	0	0
ENSMUSG00000077269	0	0	0	0	0	0
ENSMUSG00000104794	3	1	4	2	3	2
ENSMUSG00000077262	0	0	0	0	0	0
ENSMUSG00000077263	0	0	0	0	0	0
ENSMUSG00000077260	0	0	0	0	0	0
ENSMUSG00000083168	0	0	0	0	0	0
ENSMUSG00000112578	0	0	0	0	0	0
ENSMUSG00000035860	15	0	5	25	26	19
ENSMUSG00000035861	0	0	0	0	0	0
ENSMUSG00000035864	0	0	0	0	1	0
ENSMUSG00000038192	0	0	0	0	0	0
ENSMUSG00000084831	0	0	0	0	0	0
ENSMUSG00000084833	0	0	0	0	0	0
ENSMUSG00000084837	0	0	0	0	0	0
ENSMUSG00000110283	0	0	0	0	0	0
ENSMUSG00000043157	74	52	77	21	25	39
ENSMUSG00000043155	69	105	72	78	77	127
ENSMUSG00000095816	0	0	0	0	0	0
ENSMUSG00000106187	0	0	0	0	0	0
ENSMUSG00000093988	0	0	0	0	0	0
ENSMUSG00000065659	1	0	0	0	0	0
ENSMUSG00000093984	0	0	0	0	0	0
ENSMUSG00000093987	0	0	0	0	0	0
ENSMUSG00000033015	0	0	0	0	0	2
ENSMUSG00000093986	0	0	0	0	0	0
ENSMUSG00000026691	3	7	8	4	0	0
ENSMUSG00000088009	0	0	0	0	0	0
ENSMUSG00000043827	0	0	0	0	0	0
ENSMUSG00000101349	0	0	0	0	0	0
ENSMUSG00000101341	0	0	0	0	0	0
ENSMUSG00000085872	0	0	0	0	0	0
ENSMUSG00000085873	6	22	4	5	3	0
ENSMUSG00000085870	0	0	0	0	0	0
ENSMUSG00000085871	3	8	7	5	0	12
ENSMUSG00000085876	11	29	10	14	8	22
ENSMUSG00000085877	0	0	0	0	1	0

ENSMUSG00000103279	0	0	0	0	0	0
ENSMUSG00000103270	0	0	0	44	0	0
ENSMUSG00000103275	0	0	0	0	0	0
ENSMUSG00000087584	0	0	0	0	0	0
ENSMUSG00000087585	0	0	0	0	0	0
ENSMUSG00000087586	0	0	0	0	0	0
ENSMUSG00000087587	0	0	0	0	0	0
ENSMUSG00000087581	0	0	0	0	0	0
ENSMUSG00000101699	0	0	0	0	0	0
ENSMUSG00000101698	10	12	8	10	14	13
ENSMUSG00000060049	0	0	0	0	0	0
ENSMUSG00000084395	3	0	0	0	0	0
ENSMUSG00000101691	0	0	0	0	0	0
ENSMUSG00000101690	0	0	0	0	0	0
ENSMUSG00000101692	0	0	0	0	0	0
ENSMUSG00000101694	0	0	0	0	0	0
ENSMUSG00000060044	17	10	15	3	7	5
ENSMUSG00000094928	0	0	0	0	0	0
ENSMUSG00000084390	8	16	20	4	10	21
ENSMUSG00000107693	0	0	0	0	0	0
ENSMUSG00000107692	0	0	0	0	0	0
ENSMUSG00000107691	0	0	0	0	0	0
ENSMUSG00000107697	0	0	0	0	0	0
ENSMUSG00000107696	3	0	9	3	7	8
ENSMUSG00000089359	0	0	0	1	0	0
ENSMUSG00000107694	0	0	0	0	0	0
ENSMUSG00000084589	0	0	0	0	0	0
ENSMUSG00000084588	0	0	0	0	0	0
ENSMUSG00000107699	2	0	0	0	0	0
ENSMUSG00000089353	0	0	0	0	0	0
ENSMUSG00000089352	0	0	0	0	0	0
ENSMUSG00000089351	0	0	0	0	0	0
ENSMUSG00000089350	0	0	0	0	0	0
ENSMUSG00000100568	0	0	0	0	0	0
ENSMUSG00000103182	0	1	0	1	1	0
ENSMUSG00000103181	0	0	1	0	0	0
ENSMUSG00000103185	0	0	0	0	0	0
ENSMUSG00000103184	0	0	0	0	0	0
ENSMUSG00000100560	0	0	0	0	0	0
ENSMUSG00000100562	0	0	0	0	0	0
ENSMUSG00000103188	0	0	0	0	0	0
ENSMUSG00000100564	0	0	0	0	0	0
ENSMUSG00000100565	0	0	0	0	0	0
ENSMUSG00000100566	0	0	0	0	0	0
ENSMUSG00000024261	0	0	0	0	0	0
ENSMUSG00000064293	0	0	0	0	0	0
ENSMUSG00000084584	1	0	5	3	5	2
ENSMUSG00000022245	0	0	0	3	0	0
ENSMUSG00000022243	0	0	0	0	0	0
ENSMUSG00000103053	4	3	2	1	5	2
ENSMUSG00000064299	0	0	0	0	0	0
ENSMUSG00000032769	0	5	5	0	6	0
ENSMUSG00000022249	0	0	0	0	0	0
ENSMUSG00000084059	0	0	0	0	0	0
ENSMUSG00000082606	0	0	0	0	0	0
ENSMUSG00000084057	0	0	0	3	0	0
ENSMUSG00000084056	0	0	0	0	0	0
ENSMUSG00000084055	0	0	0	0	0	0

ENSMUSG00000084054	0	0	0	0	0	0
ENSMUSG00000095820	0	0	0	0	0	0
ENSMUSG00000095821	0	0	0	0	0	0
ENSMUSG00000095822	0	0	0	0	0	0
ENSMUSG00000095825	0	0	0	0	0	0
ENSMUSG00000095826	0	0	0	0	0	0
ENSMUSG00000075379	4	0	0	0	0	0
ENSMUSG00000095828	0	0	0	0	0	0
ENSMUSG00000087571	0	4	3	0	0	0
ENSMUSG00000041449	0	10	23	6	0	5
ENSMUSG00000102588	3	1	0	3	9	3
ENSMUSG00000102589	0	0	0	0	0	0
ENSMUSG00000104426	0	0	0	0	0	0
ENSMUSG00000102580	0	0	0	0	0	0
ENSMUSG00000102581	4	1	3	0	0	6
ENSMUSG00000102582	0	0	0	0	0	0
ENSMUSG00000102583	0	0	0	0	0	0
ENSMUSG00000102585	0	0	0	0	0	0
ENSMUSG00000102586	0	0	0	0	0	0
ENSMUSG00000102587	0	0	0	0	0	0
ENSMUSG00000024979	2	6	5	8	23	14
ENSMUSG00000064433	0	0	0	0	0	0
ENSMUSG00000064432	0	0	0	0	0	0
ENSMUSG00000024973	0	0	7	0	6	0
ENSMUSG00000107037	7	13	5	6	3	2
ENSMUSG00000107036	0	0	2	0	0	0
ENSMUSG00000107034	0	0	0	0	0	0
ENSMUSG00000107032	0	0	0	0	0	0
ENSMUSG00000107031	0	0	0	0	0	0
ENSMUSG00000020080	0	0	0	0	0	2
ENSMUSG00000020081	0	0	0	0	0	0
ENSMUSG00000065523	1	1	1	0	1	0
ENSMUSG00000082601	0	0	0	0	0	0
ENSMUSG00000065521	0	0	0	0	0	0
ENSMUSG00000065526	0	1	0	0	0	1
ENSMUSG00000065524	0	0	1	0	0	0
ENSMUSG00000023192	0	0	0	0	0	0
ENSMUSG00000058923	0	0	0	0	0	0
ENSMUSG00000060956	0	0	0	0	0	0
ENSMUSG00000082602	0	0	0	0	0	0
ENSMUSG00000062553	1	3	0	0	0	0
ENSMUSG00000062551	0	0	0	0	0	0
ENSMUSG00000028678	29	26	39	0	10	6
ENSMUSG00000103074	0	0	0	1	0	0
ENSMUSG00000110048	0	0	0	0	0	0
ENSMUSG00000088585	0	0	0	0	0	0
ENSMUSG00000088587	0	0	0	0	0	0
ENSMUSG00000106231	0	0	0	2	0	0
ENSMUSG00000088581	0	0	0	0	0	0
ENSMUSG00000088583	1	2	2	0	0	2
ENSMUSG00000088582	0	0	0	0	0	0
ENSMUSG00000088589	0	0	0	0	0	0
ENSMUSG00000084666	0	0	0	0	0	0
ENSMUSG00000029102	12	10	13	9	16	7
ENSMUSG00000036853	0	0	5	0	0	0
ENSMUSG00000106239	14	8	7	9	10	5
ENSMUSG00000113002	0	0	0	0	0	0
ENSMUSG00000113001	0	0	0	0	0	0

ENSMUSG00000113000	0	0	0	0	0	0
ENSMUSG00000113007	0	0	0	0	0	0
ENSMUSG00000113006	0	0	0	0	0	0
ENSMUSG00000061601	66	51	40	31	59	37
ENSMUSG00000113004	0	0	0	0	0	0
ENSMUSG00000113009	0	0	0	0	0	0
ENSMUSG00000113008	0	0	0	1	0	0
ENSMUSG00000063600	0	0	0	0	2	10
ENSMUSG00000059327	234	186	243	87	123	83
ENSMUSG00000109578	0	0	0	0	0	0
ENSMUSG00000109579	0	0	0	0	0	0
ENSMUSG00000111535	6	5	11	3	0	0
ENSMUSG00000109575	0	0	0	0	0	4
ENSMUSG00000111537	3	11	10	4	5	9
ENSMUSG00000109577	0	0	0	0	0	0
ENSMUSG00000109571	0	0	0	0	0	0
ENSMUSG00000109572	0	0	0	0	0	0
ENSMUSG00000111532	0	0	0	0	0	0
ENSMUSG00000066071	0	0	0	0	0	0
ENSMUSG00000066072	0	0	0	0	0	0
ENSMUSG00000106637	0	1	0	0	0	0
ENSMUSG00000106634	0	0	0	10	0	0
ENSMUSG00000106635	0	0	0	0	0	0
ENSMUSG00000082608	0	0	0	0	0	0
ENSMUSG00000106633	0	0	0	2	0	0
ENSMUSG00000109321	22	17	22	6	6	4
ENSMUSG00000050803	0	0	0	0	0	0
ENSMUSG00000106639	2	0	0	0	0	0
ENSMUSG00000038486	132	129	115	198	214	216
ENSMUSG00000104672	0	0	0	0	0	0
ENSMUSG00000104673	0	0	0	0	0	0
ENSMUSG00000104674	0	0	0	0	0	0
ENSMUSG00000104678	0	0	0	0	0	0
ENSMUSG00000110163	0	0	0	0	0	0
ENSMUSG00000097538	0	0	0	0	0	0
ENSMUSG00000066382	0	0	0	0	0	0
ENSMUSG00000011154	0	0	0	0	0	0
ENSMUSG00000097532	27	20	33	23	20	23
ENSMUSG00000112848	0	0	0	0	0	0
ENSMUSG00000112844	5	2	4	3	2	5
ENSMUSG00000112845	0	0	0	0	0	0
ENSMUSG00000112846	0	0	0	0	0	0
ENSMUSG00000112847	59	63	76	74	64	88
ENSMUSG00000112840	0	0	0	0	0	0
ENSMUSG00000112841	4	0	0	0	0	0
ENSMUSG00000112842	0	0	0	0	0	0
ENSMUSG00000112843	12	19	23	7	4	3
ENSMUSG00000110723	0	0	0	0	0	0
ENSMUSG00000110721	0	0	0	0	0	0
ENSMUSG00000110720	16	14	17	9	15	14
ENSMUSG00000110727	0	0	6	0	4	3
ENSMUSG00000110725	0	0	0	0	0	0
ENSMUSG00000110724	0	0	0	0	2	0
ENSMUSG00000104095	0	0	0	0	0	0
ENSMUSG00000104096	0	0	0	0	0	0
ENSMUSG00000104091	3	0	0	0	0	0
ENSMUSG00000104092	2	0	1	2	5	0
ENSMUSG00000104093	9	25	15	6	11	7

ENSMUSG00000056926	0	0	0	0	0	0
ENSMUSG00000104098	0	0	0	0	0	0
ENSMUSG00000056921	0	0	0	0	0	0
ENSMUSG00000028244	0	0	0	0	0	0
ENSMUSG00000028246	101	38	49	17	31	46
ENSMUSG00000096667	0	2	0	1	0	0
ENSMUSG00000096666	0	0	0	0	0	0
ENSMUSG00000096664	0	0	0	0	0	0
ENSMUSG00000096663	0	0	0	0	0	0
ENSMUSG00000096661	0	0	0	0	0	0
ENSMUSG00000096660	0	0	0	0	0	0
ENSMUSG00000044461	24	15	18	16	13	37
ENSMUSG00000096668	3	0	0	0	0	0
ENSMUSG00000060560	0	0	0	0	0	0
ENSMUSG00000060565	0	0	0	0	0	0
ENSMUSG00000060566	2	8	2	0	2	2
ENSMUSG00000060568	131	150	153	63	81	90
ENSMUSG00000083742	0	0	0	0	0	0
ENSMUSG00000046259	0	0	0	0	0	0
ENSMUSG00000026224	5	3	0	0	0	0
ENSMUSG00000026226	0	0	0	0	2	0
ENSMUSG00000026227	0	0	0	0	0	0
ENSMUSG00000026220	5	17	14	6	6	8
ENSMUSG00000090662	0	0	0	0	0	0
ENSMUSG00000113825	0	0	0	0	0	0
ENSMUSG00000113827	0	0	0	0	0	0
ENSMUSG00000113826	0	0	0	0	0	0
ENSMUSG00000084743	0	0	0	0	0	0
ENSMUSG00000113820	0	0	0	0	0	0
ENSMUSG00000113823	2	0	0	0	0	0
ENSMUSG00000113822	0	0	0	0	0	0
ENSMUSG00000110049	0	0	0	0	0	0
ENSMUSG00000113829	0	0	0	0	0	0
ENSMUSG00000113828	0	0	0	0	0	0
ENSMUSG00000093689	0	0	0	2	0	0
ENSMUSG00000080586	0	0	0	0	0	0
ENSMUSG00000080580	0	0	0	0	0	0
ENSMUSG00000056696	2	0	0	0	0	0
ENSMUSG00000109323	0	0	0	0	4	0
ENSMUSG00000045731	0	0	0	0	0	0
ENSMUSG00000110042	0	0	0	0	0	0
ENSMUSG00000084077	0	0	0	0	0	0
ENSMUSG00000111092	0	18	8	9	7	10
ENSMUSG00000111093	0	0	0	0	0	0
ENSMUSG00000057349	0	0	0	0	0	0
ENSMUSG00000095360	0	0	3	2	0	0
ENSMUSG00000111096	0	0	0	0	0	0
ENSMUSG00000111097	0	0	0	0	5	5
ENSMUSG00000095365	0	0	0	0	0	0
ENSMUSG00000093683	0	0	0	0	0	0
ENSMUSG00000089087	0	0	0	0	0	0
ENSMUSG00000111098	0	0	0	0	0	0
ENSMUSG00000084070	0	0	0	0	0	0
ENSMUSG00000084741	0	0	0	0	0	0
ENSMUSG00000081180	0	0	0	0	0	0
ENSMUSG00000081181	0	0	0	0	0	0
ENSMUSG00000081186	0	0	0	0	0	0
ENSMUSG00000081184	0	0	0	0	0	0

ENSMUSG00000081188	0	0	0	0	0	0
ENSMUSG00000047462	6	6	16	0	4	0
ENSMUSG00000093685	0	0	0	0	0	0
ENSMUSG00000089088	0	0	0	0	0	0
ENSMUSG00000075314	0	0	0	0	0	0
ENSMUSG00000095807	0	0	0	0	0	0
ENSMUSG00000080925	0	0	0	0	0	0
ENSMUSG00000052334	0	0	0	0	0	0
ENSMUSG00000095586	0	0	0	0	0	0
ENSMUSG00000095584	0	0	0	0	0	0
ENSMUSG00000095581	0	0	0	0	0	0
ENSMUSG00000096561	0	0	0	0	1	0
ENSMUSG00000077065	0	0	0	0	0	0
ENSMUSG00000100544	0	0	0	0	0	0
ENSMUSG00000093406	0	0	0	0	0	0
ENSMUSG00000107295	6	9	5	5	0	2
ENSMUSG00000108807	3	0	5	4	9	6
ENSMUSG00000052595	0	0	2	1	0	0
ENSMUSG00000097981	2	4	4	4	4	4
ENSMUSG00000097980	0	0	0	0	0	0
ENSMUSG00000097982	0	0	0	0	0	0
ENSMUSG00000048022	0	4	7	0	4	2
ENSMUSG00000097984	0	0	0	0	0	0
ENSMUSG00000028332	3	0	0	2	0	0
ENSMUSG00000097986	0	0	0	0	0	0
ENSMUSG00000097989	32	25	31	23	33	28
ENSMUSG00000097988	0	4	0	0	0	0
ENSMUSG00000048029	0	9	16	5	4	4
ENSMUSG00000112019	0	2	0	0	1	0
ENSMUSG00000112018	0	0	0	2	0	0
ENSMUSG00000094188	0	0	0	0	0	0
ENSMUSG00000094189	0	0	0	0	0	0
ENSMUSG00000112013	0	2	1	1	1	0
ENSMUSG00000094185	14	14	14	11	12	12
ENSMUSG00000094186	0	0	0	0	0	0
ENSMUSG00000042179	0	0	0	0	0	0
ENSMUSG00000094180	0	0	0	0	0	0
ENSMUSG00000094181	0	0	0	0	0	0
ENSMUSG00000094182	0	0	0	0	0	0
ENSMUSG00000112014	0	4	8	0	5	0
ENSMUSG00000067562	0	0	0	0	0	0
ENSMUSG00000097985	0	0	0	0	2	0
ENSMUSG00000104520	3	0	2	0	0	0
ENSMUSG00000109466	0	0	0	0	0	0
ENSMUSG00000105764	0	0	0	0	0	0
ENSMUSG00000104521	0	0	0	0	0	0
ENSMUSG00000051811	6	9	17	11	20	17
ENSMUSG00000105984	0	0	0	0	0	0
ENSMUSG00000076005	0	0	0	0	0	0
ENSMUSG00000076006	0	1	1	0	0	0
ENSMUSG00000045246	6	0	4	10	0	0
ENSMUSG00000110128	0	0	0	0	0	0
ENSMUSG00000082519	0	0	1	0	0	0
ENSMUSG00000087742	0	2	0	0	0	1
ENSMUSG00000087741	2	0	0	0	0	0
ENSMUSG00000087746	0	0	0	0	0	0
ENSMUSG00000082511	0	0	0	0	0	0
ENSMUSG00000082512	2	0	6	0	0	1

ENSMUSG00000037279	0	0	0	0	0	0
ENSMUSG00000070780	76	95	127	43	19	56
ENSMUSG00000113793	0	0	0	0	0	0
ENSMUSG00000094273	0	0	0	0	0	0
ENSMUSG00000084219	0	0	0	0	0	0
ENSMUSG00000001260	0	0	0	0	0	0
ENSMUSG00000084210	0	0	0	0	0	0
ENSMUSG00000084214	0	0	0	0	0	0
ENSMUSG00000084217	0	0	0	0	0	0
ENSMUSG00000084216	1	0	0	0	0	2
ENSMUSG00000107299	0	0	0	0	0	0
ENSMUSG00000022429	7	0	4	10	2	7
ENSMUSG00000075574	0	0	0	0	0	0
ENSMUSG00000075570	0	0	0	0	0	0
ENSMUSG00000075571	0	0	0	0	0	3
ENSMUSG00000075572	0	0	0	0	0	0
ENSMUSG00000075573	0	0	0	0	0	0
ENSMUSG00000025255	138	184	117	37	63	35
ENSMUSG00000094271	1	1	1	1	1	1
ENSMUSG00000080916	2	1	0	0	1	4
ENSMUSG00000080917	0	2	0	0	4	0
ENSMUSG00000080914	13	3	3	4	5	18
ENSMUSG00000080915	0	0	0	0	0	0
ENSMUSG00000080912	1	0	0	0	0	0
ENSMUSG00000080913	0	0	0	0	0	0
ENSMUSG00000080910	0	0	0	0	0	0
ENSMUSG00000080911	0	0	0	0	0	0
ENSMUSG00000080918	0	0	0	0	0	0
ENSMUSG00000078672	0	0	0	0	0	0
ENSMUSG00000078673	0	0	0	0	0	0
ENSMUSG00000078674	0	0	0	0	0	0
ENSMUSG00000078675	0	0	0	0	0	0
ENSMUSG00000086164	0	0	0	0	0	0
ENSMUSG00000094276	0	0	0	0	0	0
ENSMUSG00000030484	0	0	0	0	0	0
ENSMUSG00000065721	0	0	0	0	0	0
ENSMUSG00000065722	0	0	0	0	0	0
ENSMUSG00000113920	0	0	0	0	0	0
ENSMUSG00000065726	0	0	0	0	0	0
ENSMUSG00000044106	0	0	0	0	0	0
ENSMUSG00000065729	0	0	0	0	0	0
ENSMUSG00000113921	0	0	0	1	0	0
ENSMUSG00000032357	0	0	0	0	2	0
ENSMUSG00000032356	3	0	5	0	3	7
ENSMUSG00000062712	0	2	0	0	0	0
ENSMUSG00000062713	0	3	0	0	0	0
ENSMUSG00000065090	0	2	0	0	1	0
ENSMUSG00000065091	0	0	0	0	0	0
ENSMUSG00000065096	0	0	0	0	0	0
ENSMUSG00000078097	3	1	4	1	2	2
ENSMUSG00000078091	19	21	16	24	27	35
ENSMUSG00000095411	0	0	0	0	0	0
ENSMUSG00000095147	1	0	0	0	0	2
ENSMUSG00000063446	2	0	0	0	3	0
ENSMUSG00000095086	0	2	0	0	0	0
ENSMUSG00000104404	0	0	0	0	0	0
ENSMUSG00000103844	0	0	0	0	0	0
ENSMUSG00000103845	0	0	0	9	12	0

ENSMUSG00000103842	0	0	0	0	0	0
ENSMUSG00000082571	0	0	0	0	0	0
ENSMUSG00000103840	0	0	0	0	0	0
ENSMUSG00000103841	0	0	0	0	0	0
ENSMUSG00000103849	0	0	0	0	0	0
ENSMUSG00000031194	0	0	0	0	0	0
ENSMUSG00000063193	100	101	120	20	29	30
ENSMUSG00000089968	26	13	20	18	10	17
ENSMUSG00000089964	2	0	0	0	0	0
ENSMUSG00000089965	4	0	0	2	0	0
ENSMUSG00000089963	0	0	0	0	0	0
ENSMUSG00000084245	0	0	0	0	0	0
ENSMUSG00000012520	0	0	3	0	0	0
ENSMUSG00000111286	0	0	0	0	0	0
ENSMUSG00000050114	0	0	0	0	0	0
ENSMUSG00000076948	0	0	0	0	0	1
ENSMUSG00000111281	0	0	0	0	2	0
ENSMUSG00000083763	0	0	0	0	0	0
ENSMUSG00000076941	1	3	3	1	2	2
ENSMUSG00000076944	0	0	0	0	0	0
ENSMUSG00000098533	0	0	0	0	0	0
ENSMUSG00000034968	38	23	24	13	21	27
ENSMUSG00000039138	0	0	0	0	0	0
ENSMUSG00000028836	0	5	7	10	9	33
ENSMUSG00000108931	0	0	0	0	0	0
ENSMUSG00000108930	0	0	0	0	0	0
ENSMUSG00000091260	0	0	0	0	0	0
ENSMUSG00000108938	0	0	0	0	0	0
ENSMUSG00000105874	0	0	0	0	0	0
ENSMUSG00000043342	5	4	5	5	0	5
ENSMUSG00000105873	13	14	10	2	9	16
ENSMUSG00000105872	0	0	0	0	0	0
ENSMUSG00000030244	0	0	0	0	0	0
ENSMUSG00000085688	0	0	0	0	0	0
ENSMUSG00000085202	0	0	0	0	0	0
ENSMUSG00000101558	0	0	0	0	0	0
ENSMUSG00000101557	0	0	0	0	0	0
ENSMUSG00000101554	0	0	0	0	0	0
ENSMUSG00000101552	0	0	0	0	0	0
ENSMUSG00000101553	0	0	0	0	0	0
ENSMUSG00000101550	0	0	2	0	2	5
ENSMUSG00000101551	0	0	0	0	0	0
ENSMUSG00000085936	133	129	180	92	121	118
ENSMUSG00000085933	0	0	0	0	0	0
ENSMUSG00000088562	0	0	0	0	0	0
ENSMUSG00000083768	1	0	0	0	0	4
ENSMUSG00000087265	0	0	0	2	0	4
ENSMUSG00000087264	0	0	0	0	0	0
ENSMUSG00000087267	0	0	0	0	0	0
ENSMUSG00000087268	4	10	3	0	3	0
ENSMUSG00000014030	20	28	24	16	7	6
ENSMUSG00000037735	16	4	15	0	4	6
ENSMUSG00000087417	0	0	0	0	0	0
ENSMUSG00000098818	0	0	0	0	1	0
ENSMUSG00000087415	0	0	0	0	0	0
ENSMUSG00000021928	232	247	249	174	172	194
ENSMUSG00000050612	5	4	6	0	0	5
ENSMUSG00000050613	0	0	0	0	0	0

ENSMUSG00000059956	0	0	0	0	0	0
ENSMUSG00000103029	0	0	0	0	0	0
ENSMUSG00000108613	0	0	0	0	0	0
ENSMUSG00000103020	0	0	0	0	0	0
ENSMUSG00000103021	0	0	0	0	0	0
ENSMUSG00000095511	0	0	0	0	0	0
ENSMUSG00000103025	13	0	15	0	3	1
ENSMUSG00000009070	0	0	0	0	0	0
ENSMUSG00000101510	0	0	0	0	0	0
ENSMUSG00000089149	0	0	0	0	0	0
ENSMUSG00000089146	0	0	0	0	0	0
ENSMUSG00000089147	1	2	1	0	0	0
ENSMUSG00000089144	0	2	0	0	2	1
ENSMUSG00000089143	0	0	0	0	0	0
ENSMUSG00000089141	0	0	0	0	0	0
ENSMUSG00000023781	10	11	7	0	0	10
ENSMUSG00000112259	0	2	0	2	0	0
ENSMUSG00000112258	0	0	0	0	0	0
ENSMUSG00000094349	0	0	0	0	0	0
ENSMUSG00000094342	0	0	0	0	0	0
ENSMUSG00000039070	0	0	0	0	0	0
ENSMUSG00000112253	0	0	0	0	0	0
ENSMUSG00000112252	15	15	8	20	10	16
ENSMUSG00000094346	0	0	0	0	0	0
ENSMUSG00000094347	4	2	2	1	1	1
ENSMUSG00000112257	0	0	2	1	2	0
ENSMUSG00000022780	0	0	0	0	0	0
ENSMUSG00000102777	0	0	0	0	0	0
ENSMUSG00000102774	0	0	0	0	0	0
ENSMUSG00000102773	0	0	0	0	0	0
ENSMUSG00000102772	0	0	0	0	0	0
ENSMUSG00000102771	0	0	0	0	2	3
ENSMUSG00000024837	0	0	0	0	3	0
ENSMUSG00000018620	0	0	0	0	0	0
ENSMUSG00000018623	0	0	0	0	0	0
ENSMUSG00000102778	0	0	0	0	0	0
ENSMUSG00000105938	0	0	0	0	0	0
ENSMUSG00000036957	30	12	22	11	14	17
ENSMUSG00000036951	0	0	0	0	0	0
ENSMUSG00000036958	0	0	0	0	0	0
ENSMUSG00000038013	326	342	363	279	348	449
ENSMUSG00000015854	0	9	13	0	0	0
ENSMUSG00000039385	6	5	3	22	3	0
ENSMUSG00000105467	0	0	0	0	0	0
ENSMUSG00000005465	165	147	197	78	104	119
ENSMUSG00000105462	0	0	0	0	0	0
ENSMUSG00000114965	0	3	0	3	0	0
ENSMUSG00000114964	0	2	0	0	0	2
ENSMUSG00000114960	0	0	0	3	0	0
ENSMUSG00000114962	1	2	0	4	0	2
ENSMUSG00000110098	0	0	0	0	0	0
ENSMUSG00000110096	0	0	0	0	0	0
ENSMUSG00000110092	0	0	0	0	0	0
ENSMUSG00000038246	0	0	0	0	0	0
ENSMUSG00000088790	2	0	0	0	0	2
ENSMUSG00000088791	0	0	0	0	0	0
ENSMUSG00000047868	0	0	0	0	0	0
ENSMUSG00000088798	0	0	0	0	0	0

ENSMUSG00000088799	0	0	0	0	0	0
ENSMUSG00000113022	5	3	0	0	5	4
ENSMUSG00000021492	0	0	0	0	0	0
ENSMUSG00000021490	0	0	0	0	0	0
ENSMUSG00000105790	0	0	0	0	0	0
ENSMUSG00000105792	0	0	0	2	3	0
ENSMUSG00000029378	0	0	0	0	0	0
ENSMUSG00000105794	0	0	0	0	0	0
ENSMUSG00000029370	0	3	0	0	0	0
ENSMUSG00000029377	66	60	156	2	4	0
ENSMUSG00000029375	0	0	0	0	0	0
ENSMUSG00000034551	71	67	75	43	49	63
ENSMUSG00000113613	0	0	0	0	0	0
ENSMUSG00000034552	0	0	0	0	0	0
ENSMUSG00000113616	0	0	0	0	0	0
ENSMUSG00000113617	0	0	0	0	0	0
ENSMUSG00000113614	0	0	0	0	0	0
ENSMUSG00000113615	0	0	0	0	0	0
ENSMUSG00000113618	0	0	0	0	0	0
ENSMUSG00000083986	7	1	3	6	3	0
ENSMUSG00000021148	0	0	0	0	0	0
ENSMUSG00000045336	0	3	0	0	0	0
ENSMUSG00000114341	0	0	0	0	0	0
ENSMUSG00000114343	0	0	0	0	0	0
ENSMUSG00000114342	0	0	0	0	0	0
ENSMUSG00000114345	0	0	0	0	0	3
ENSMUSG00000057997	0	0	0	0	0	0
ENSMUSG00000096254	0	0	0	0	0	0
ENSMUSG00000114346	0	0	0	0	0	0
ENSMUSG00000114348	0	0	0	0	0	0
ENSMUSG00000096258	0	0	0	0	0	0
ENSMUSG00000096259	0	0	0	0	0	0
ENSMUSG00000106845	0	0	5	0	0	0
ENSMUSG00000106846	0	0	0	0	0	0
ENSMUSG00000106843	1	0	0	0	0	0
ENSMUSG00000050926	2	2	2	2	2	5
ENSMUSG00000108215	0	2	0	0	0	0
ENSMUSG00000108213	0	0	0	0	0	0
ENSMUSG00000110067	0	0	0	0	0	0
ENSMUSG00000067173	3	3	0	0	0	0
ENSMUSG00000083281	0	0	0	0	0	0
ENSMUSG00000025496	0	6	4	0	0	0
ENSMUSG00000025497	0	0	0	0	0	0
ENSMUSG00000104041	14	6	17	5	15	2
ENSMUSG00000097701	0	4	0	0	0	4
ENSMUSG00000097709	10	18	13	8	5	5
ENSMUSG00000060663	7	5	12	6	0	4
ENSMUSG00000091700	0	0	0	0	0	0
ENSMUSG00000091703	0	0	0	0	0	0
ENSMUSG00000101033	0	0	0	0	0	0
ENSMUSG00000091709	0	0	0	0	0	0
ENSMUSG00000044349	14	10	38	8	28	4
ENSMUSG00000044348	144	156	109	76	91	124
ENSMUSG00000101038	0	0	0	0	0	0
ENSMUSG00000046272	2	0	0	0	2	2
ENSMUSG00000056716	0	3	0	5	0	0
ENSMUSG00000095289	0	0	0	0	0	0
ENSMUSG00000110731	0	0	0	0	0	0

ENSMUSG00000095286	0	0	0	0	0	0
ENSMUSG00000095287	0	0	0	0	0	0
ENSMUSG00000044694	76	61	48	56	82	82
ENSMUSG00000110732	0	0	0	0	0	0
ENSMUSG00000039339	0	0	0	5	0	2
ENSMUSG00000045569	0	0	0	0	0	0
ENSMUSG00000046180	0	0	0	0	0	0
ENSMUSG00000074934	4	0	0	0	0	0
ENSMUSG00000074939	5	0	0	3	0	0
ENSMUSG00000045566	0	0	0	0	0	0
ENSMUSG00000079875	0	0	0	0	0	0
ENSMUSG00000053297	55	62	64	19	32	36
ENSMUSG00000080798	0	0	0	0	0	0
ENSMUSG00000080799	0	0	0	0	0	0
ENSMUSG00000080794	0	0	0	0	2	0
ENSMUSG00000080795	1	0	1	0	0	0
ENSMUSG00000080796	29	17	11	11	5	14
ENSMUSG00000080792	0	0	0	0	0	0
ENSMUSG00000086828	0	0	0	0	0	0
ENSMUSG00000086827	0	0	0	0	0	0
ENSMUSG00000086821	0	0	0	0	0	0
ENSMUSG00000086822	0	0	1	9	6	7
ENSMUSG00000086823	0	0	0	0	0	0
ENSMUSG00000095136	0	1	0	0	0	1
ENSMUSG00000095137	0	0	0	0	0	0
ENSMUSG00000095135	0	0	0	0	0	0
ENSMUSG00000095138	0	0	0	0	0	0
ENSMUSG00000095139	0	0	2	2	0	4
ENSMUSG00000098553	1	1	1	0	1	0
ENSMUSG00000098552	0	0	0	0	0	0
ENSMUSG00000098555	0	0	0	0	0	0
ENSMUSG00000053873	0	0	0	0	0	0
ENSMUSG00000047631	3	0	0	0	0	0
ENSMUSG00000047636	0	0	0	0	0	0
ENSMUSG00000077547	0	0	0	0	0	0
ENSMUSG00000047638	26	20	29	18	29	38
ENSMUSG00000110737	5	0	0	3	0	4
ENSMUSG00000094660	0	0	0	0	0	0
ENSMUSG00000094667	0	0	0	0	0	0
ENSMUSG00000006958	196	130	169	127	158	198
ENSMUSG00000086795	0	0	0	0	0	2
ENSMUSG00000086792	0	0	4	0	0	0
ENSMUSG00000086790	0	0	0	0	0	0
ENSMUSG00000035472	4	0	5	0	0	0
ENSMUSG00000086798	0	0	0	0	0	0
ENSMUSG00000081449	0	0	0	0	0	0
ENSMUSG00000090947	0	0	0	0	0	0
ENSMUSG00000090358	0	0	0	0	0	2
ENSMUSG00000081443	0	0	0	0	0	0
ENSMUSG00000048782	22	12	7	6	4	16
ENSMUSG00000081440	3	0	0	0	0	0
ENSMUSG00000081447	1	0	0	0	0	0
ENSMUSG00000090949	0	0	0	0	0	0
ENSMUSG00000081445	0	2	0	0	0	2
ENSMUSG00000081444	0	0	0	0	0	0
ENSMUSG00000030069	0	0	0	0	0	0
ENSMUSG00000048251	41	54	37	84	80	104
ENSMUSG00000099739	0	0	0	0	0	0

ENSMUSG00000092548	0	0	0	0	0	0
ENSMUSG00000099734	0	0	0	0	0	0
ENSMUSG00000092547	0	0	0	0	0	0
ENSMUSG00000099736	0	0	0	0	0	0
ENSMUSG00000099737	0	0	0	0	0	0
ENSMUSG00000092542	0	0	0	0	0	0
ENSMUSG00000099731	0	0	0	0	0	0
ENSMUSG00000099732	0	0	0	0	0	0
ENSMUSG00000099733	0	0	0	0	0	0
ENSMUSG00000077567	2	2	3	7	1	3
ENSMUSG00000077562	0	0	0	0	0	0
ENSMUSG00000098585	0	0	0	0	0	0
ENSMUSG00000030001	0	0	0	0	0	0
ENSMUSG00000030000	71	24	93	9	11	8
ENSMUSG00000082239	0	0	0	0	0	0
ENSMUSG00000082238	0	0	0	0	0	0
ENSMUSG00000030004	0	0	3	0	0	0
ENSMUSG00000082235	0	0	0	0	0	0
ENSMUSG00000082234	4	0	0	2	0	0
ENSMUSG00000082237	0	0	0	0	0	0
ENSMUSG00000082236	0	0	0	0	0	0
ENSMUSG00000022382	7	0	0	0	3	0
ENSMUSG00000032537	105	159	122	161	151	217
ENSMUSG00000032530	5	2	7	5	4	5
ENSMUSG00000032532	0	0	4	0	2	0
ENSMUSG00000004552	6	0	16	0	0	4
ENSMUSG00000040972	0	0	0	5	0	0
ENSMUSG00000072423	0	0	0	0	0	0
ENSMUSG00000041670	4	0	3	0	0	2
ENSMUSG00000082490	0	0	0	0	2	0
ENSMUSG00000082492	0	0	0	0	1	0
ENSMUSG00000082495	0	0	0	0	0	0
ENSMUSG00000082497	0	0	0	0	0	0
ENSMUSG00000082496	0	0	0	0	0	0
ENSMUSG00000082499	0	0	0	0	0	0
ENSMUSG00000087465	0	0	0	0	0	0
ENSMUSG00000087464	0	0	0	0	0	0
ENSMUSG00000076219	0	0	1	0	0	0
ENSMUSG00000087461	0	0	0	0	0	0
ENSMUSG00000087460	0	0	0	0	0	0
ENSMUSG00000083548	0	0	0	0	0	0
ENSMUSG00000083549	0	0	0	0	0	0
ENSMUSG00000083544	0	0	0	0	0	0
ENSMUSG00000083546	0	0	0	0	0	0
ENSMUSG00000083547	0	0	0	0	0	0
ENSMUSG00000083542	0	0	0	0	0	0
ENSMUSG00000083543	0	0	0	0	0	0
ENSMUSG00000049122	11	3	10	4	5	6
ENSMUSG00000049123	0	0	0	0	0	0
ENSMUSG00000049128	0	0	0	0	0	0
ENSMUSG00000079466	0	0	0	0	0	0
ENSMUSG00000000381	0	4	0	2	4	0
ENSMUSG00000112362	0	0	1	3	0	2
ENSMUSG00000031844	0	0	0	0	0	0
ENSMUSG00000031845	3	3	1	0	0	3
ENSMUSG00000068966	302	274	285	245	234	279
ENSMUSG00000031842	124	189	132	143	193	252
ENSMUSG00000068962	24	11	10	0	0	12

ENSMUSG00000080899	2	0	0	0	0	0
ENSMUSG00000080898	0	0	0	0	0	0
ENSMUSG00000112367	8	5	4	2	7	9
ENSMUSG00000080892	0	0	0	0	0	0
ENSMUSG00000080891	2	0	0	0	0	0
ENSMUSG00000094275	0	0	0	0	0	0
ENSMUSG00000080897	0	0	0	0	0	0
ENSMUSG00000026815	0	0	0	0	0	0
ENSMUSG00000080895	0	0	0	0	0	0
ENSMUSG00000080894	0	0	0	0	0	0
ENSMUSG00000059610	0	0	0	0	0	2
ENSMUSG00000102645	0	0	0	0	0	0
ENSMUSG00000033720	238	230	232	286	329	293
ENSMUSG00000079794	0	0	0	0	0	0
ENSMUSG00000033726	0	0	0	0	0	0
ENSMUSG00000100345	0	0	0	0	0	0
ENSMUSG00000061342	0	0	0	0	0	0
ENSMUSG00000094079	0	0	0	0	0	0
ENSMUSG00000108685	0	0	0	0	0	0
ENSMUSG00000064646	0	0	0	0	0	0
ENSMUSG00000024903	0	0	0	0	0	0
ENSMUSG00000108687	0	2	0	2	0	0
ENSMUSG00000110536	2	2	0	0	0	2
ENSMUSG00000107240	3	0	3	0	0	0
ENSMUSG00000107241	0	0	0	0	0	0
ENSMUSG00000107243	0	0	0	0	0	0
ENSMUSG00000107244	0	0	0	0	3	0
ENSMUSG00000107247	0	0	0	0	0	0
ENSMUSG00000107248	0	0	0	0	0	0
ENSMUSG00000062527	0	1	0	0	0	0
ENSMUSG00000112861	0	0	0	0	0	0
ENSMUSG00000100349	2	5	0	0	1	0
ENSMUSG00000068341	0	0	0	0	0	0
ENSMUSG00000101958	0	0	0	0	0	0
ENSMUSG00000062524	4	0	16	0	4	0
ENSMUSG00000068349	0	0	0	0	0	0
ENSMUSG00000101953	0	0	0	0	0	0
ENSMUSG00000065115	0	0	0	0	0	0
ENSMUSG00000101951	15	10	6	11	7	8
ENSMUSG00000101956	1	0	0	0	0	0
ENSMUSG00000101957	0	0	0	0	0	0
ENSMUSG00000101954	0	0	0	0	0	0
ENSMUSG00000101955	0	0	0	0	0	0
ENSMUSG00000099091	0	0	0	0	0	0
ENSMUSG00000099094	7	4	8	0	0	0
ENSMUSG00000099097	0	0	0	0	0	0
ENSMUSG00000110977	0	0	0	0	0	0
ENSMUSG00000099099	0	0	0	0	0	0
ENSMUSG00000110973	0	1	0	0	0	0
ENSMUSG00000109880	0	0	0	0	0	0
ENSMUSG00000030827	3	0	4	0	0	0
ENSMUSG00000110970	0	0	0	0	0	0
ENSMUSG00000081856	0	0	0	0	0	0
ENSMUSG00000081851	0	0	0	0	0	0
ENSMUSG00000081852	1	0	0	0	0	2
ENSMUSG00000081853	0	1	0	0	0	0
ENSMUSG00000081858	0	0	0	0	0	0
ENSMUSG00000081859	0	0	0	0	0	0

ENSMUSG00000105309	0	0	0	0	0	0
ENSMUSG00000113099	0	0	0	0	0	0
ENSMUSG00000099020	0	0	0	0	0	0
ENSMUSG00000109887	0	0	0	0	0	0
ENSMUSG00000032091	0	0	0	0	0	0
ENSMUSG00000032094	0	8	6	2	0	0
ENSMUSG00000105303	0	0	0	2	0	0
ENSMUSG00000094077	0	0	0	0	0	0
ENSMUSG00000105301	0	0	0	0	0	0
ENSMUSG00000105300	0	0	0	0	0	0
ENSMUSG00000006567	0	0	0	0	5	0
ENSMUSG00000035371	0	0	0	0	0	0
ENSMUSG00000102366	0	0	0	0	0	0
ENSMUSG00000097091	3	0	0	0	0	0
ENSMUSG00000038630	3	5	0	11	14	0
ENSMUSG00000094072	0	0	0	0	0	1
ENSMUSG00000073293	23	43	36	58	37	61
ENSMUSG00000073290	0	0	0	0	0	0
ENSMUSG00000096011	0	0	0	0	0	0
ENSMUSG00000096014	0	0	0	0	0	0
ENSMUSG00000096016	0	0	0	0	0	0
ENSMUSG00000096017	0	0	0	0	0	0
ENSMUSG00000096018	0	0	0	0	0	0
ENSMUSG00000109666	0	0	0	0	0	0
ENSMUSG00000110708	0	0	0	0	0	0
ENSMUSG00000063252	0	0	0	0	0	0
ENSMUSG00000102985	0	0	0	0	3	0
ENSMUSG00000102986	0	0	0	0	1	0
ENSMUSG00000102987	0	0	0	0	0	0
ENSMUSG00000110703	0	0	0	0	0	0
ENSMUSG00000110702	26	42	61	34	37	38
ENSMUSG00000102988	0	0	0	0	2	0
ENSMUSG00000066750	0	0	0	0	0	0
ENSMUSG00000066755	14	10	12	0	0	0
ENSMUSG00000104209	0	0	0	0	0	0
ENSMUSG00000104205	0	0	0	2	0	0
ENSMUSG00000104207	0	0	0	0	0	0
ENSMUSG00000104201	0	0	0	0	0	0
ENSMUSG00000104203	0	3	0	0	0	0
ENSMUSG00000104202	1	1	1	1	0	0
ENSMUSG00000091541	0	0	3	0	0	0
ENSMUSG00000086724	0	0	0	0	0	2
ENSMUSG00000020950	0	0	0	0	0	0
ENSMUSG00000101189	0	0	0	0	0	0
ENSMUSG00000060759	0	0	0	0	0	0
ENSMUSG00000101181	0	0	0	0	0	0
ENSMUSG00000101180	0	0	0	0	0	0
ENSMUSG00000101183	0	0	0	0	0	0
ENSMUSG00000101182	0	0	0	0	0	0
ENSMUSG00000060756	0	0	0	0	0	0
ENSMUSG00000101184	0	0	0	0	0	0
ENSMUSG00000101186	0	0	0	0	0	0
ENSMUSG00000091099	0	0	0	0	0	0
ENSMUSG00000064982	0	0	0	0	0	0
ENSMUSG00000064987	3	0	3	2	0	0
ENSMUSG00000091091	7	6	10	5	6	19
ENSMUSG00000061809	0	0	0	0	0	0
ENSMUSG00000100279	0	0	0	0	0	0

ENSMUSG00000100270	0	0	0	0	0	0
ENSMUSG00000100271	0	0	0	0	0	0
ENSMUSG00000100272	0	0	0	0	2	0
ENSMUSG00000061802	0	0	0	0	0	0
ENSMUSG00000100275	0	0	0	0	0	0
ENSMUSG00000100276	0	0	0	0	0	0
ENSMUSG00000100277	0	0	0	0	0	0
ENSMUSG00000096642	0	0	0	0	0	0
ENSMUSG00000064325	0	0	0	0	0	0
ENSMUSG00000064329	0	0	0	2	0	0
ENSMUSG00000095730	20	46	53	32	26	38
ENSMUSG00000095734	0	0	0	0	0	0
ENSMUSG00000095739	0	0	0	0	0	0
ENSMUSG00000088429	0	0	0	0	0	0
ENSMUSG00000088426	0	0	0	0	0	0
ENSMUSG00000088425	0	0	2	0	0	0
ENSMUSG00000088422	0	0	0	0	0	0
ENSMUSG00000088421	0	0	0	0	0	0
ENSMUSG00000088848	0	0	0	0	0	0
ENSMUSG00000022622	6	9	21	25	16	18
ENSMUSG00000094068	0	0	0	0	0	0
ENSMUSG00000094067	0	0	0	0	0	1
ENSMUSG00000094064	0	0	0	0	0	0
ENSMUSG00000094063	0	0	0	0	0	0
ENSMUSG00000094060	0	0	0	0	0	0
ENSMUSG00000111939	0	4	1	0	0	0
ENSMUSG00000111931	0	0	0	0	0	0
ENSMUSG00000111930	0	0	0	0	0	0
ENSMUSG00000111933	21	22	30	24	31	36
ENSMUSG00000111935	4	8	10	8	8	7
ENSMUSG00000111934	0	0	0	0	0	0
ENSMUSG00000111937	0	1	1	0	0	2
ENSMUSG00000111936	0	0	0	0	0	0
ENSMUSG00000103617	0	0	0	0	0	0
ENSMUSG00000103616	3	0	5	0	0	0
ENSMUSG00000103615	0	0	0	0	0	0
ENSMUSG00000062861	159	184	146	155	161	159
ENSMUSG00000062868	0	0	0	0	0	0
ENSMUSG00000028747	0	0	0	0	0	0
ENSMUSG00000028749	8	5	0	0	8	4
ENSMUSG00000024669	7	3	14	0	0	0
ENSMUSG00000020177	0	0	0	0	0	0
ENSMUSG00000020175	46	31	64	17	34	40
ENSMUSG00000097009	6	8	3	2	10	4
ENSMUSG00000043274	1	0	0	0	0	0
ENSMUSG00000097001	0	0	0	0	0	0
ENSMUSG00000097005	0	0	0	0	0	0
ENSMUSG00000092036	0	3	0	0	0	0
ENSMUSG00000113796	0	0	3	0	0	0
ENSMUSG00000113794	0	0	0	0	0	0
ENSMUSG00000092032	0	0	0	0	0	0
ENSMUSG00000113791	0	0	0	0	0	0
ENSMUSG00000092031	0	0	0	0	0	0
ENSMUSG00000038179	65	86	130	32	38	20
ENSMUSG00000092039	2	0	0	1	0	0
ENSMUSG00000035033	0	0	0	0	0	0
ENSMUSG00000112778	0	0	0	1	0	0
ENSMUSG00000046846	0	0	0	0	0	0

ENSMUSG00000046845	0	0	0	0	0	0
ENSMUSG00000046840	0	0	0	0	0	0
ENSMUSG00000110685	0	0	0	0	0	4
ENSMUSG00000113421	0	0	0	0	0	0
ENSMUSG00000113420	0	0	0	0	0	0
ENSMUSG00000108398	0	4	3	0	0	0
ENSMUSG00000113425	18	26	14	12	12	8
ENSMUSG00000113427	33	40	40	39	21	27
ENSMUSG00000113426	0	0	0	0	0	0
ENSMUSG00000108393	0	0	0	0	0	0
ENSMUSG00000108392	0	0	0	0	0	0
ENSMUSG00000108390	0	0	0	0	0	0
ENSMUSG00000108397	0	2	0	0	0	0
ENSMUSG00000108395	0	0	0	0	0	0
ENSMUSG00000100945	0	0	0	0	0	0
ENSMUSG00000105615	0	0	0	0	0	0
ENSMUSG00000105614	0	0	0	0	0	0
ENSMUSG00000105616	0	0	0	0	0	0
ENSMUSG00000082804	0	0	0	0	0	0
ENSMUSG00000105612	0	0	0	0	0	0
ENSMUSG00000105619	0	0	0	0	0	0
ENSMUSG00000105618	0	0	0	0	0	0
ENSMUSG00000082968	1	0	0	0	0	0
ENSMUSG00000096353	0	0	0	0	0	0
ENSMUSG00000096352	0	1	0	0	1	0
ENSMUSG00000082961	0	5	2	0	2	1
ENSMUSG00000082960	0	0	0	0	2	0
ENSMUSG00000082963	0	0	0	0	0	0
ENSMUSG00000082962	3	1	2	0	1	2
ENSMUSG00000082965	0	0	0	0	0	0
ENSMUSG00000096359	0	0	0	0	0	0
ENSMUSG00000082966	0	0	0	0	0	0
ENSMUSG00000050468	0	0	0	0	0	0
ENSMUSG00000070379	0	0	0	0	0	0
ENSMUSG00000070378	0	0	0	0	0	0
ENSMUSG00000079445	13	8	21	0	3	3
ENSMUSG00000070375	0	0	0	0	0	0
ENSMUSG00000070374	0	0	0	0	0	0
ENSMUSG00000070377	0	0	0	0	0	0
ENSMUSG00000113544	0	0	2	0	0	0
ENSMUSG00000079440	0	0	0	0	0	0
ENSMUSG00000113545	0	1	0	0	0	0
ENSMUSG00000111481	30	39	33	34	34	49
ENSMUSG00000111480	0	0	0	0	0	0
ENSMUSG00000111483	0	0	0	2	0	0
ENSMUSG00000111482	0	0	0	0	0	0
ENSMUSG00000111485	1	3	3	0	2	4
ENSMUSG00000111487	0	0	2	0	2	0
ENSMUSG00000111486	0	0	0	0	0	0
ENSMUSG00000111489	0	0	0	0	0	0
ENSMUSG00000111488	0	5	6	0	0	0
ENSMUSG00000113548	0	0	0	0	0	0
ENSMUSG00000093276	0	0	0	0	0	0
ENSMUSG00000108025	0	0	0	0	0	0
ENSMUSG00000108027	0	0	0	0	0	0
ENSMUSG00000108026	7	3	0	0	0	0
ENSMUSG00000104560	0	0	0	0	0	0
ENSMUSG00000104561	0	0	0	0	0	0

ENSMUSG00000108023	0	0	0	0	0	0
ENSMUSG00000108022	0	0	0	0	0	0
ENSMUSG00000104568	0	0	0	0	0	0
ENSMUSG00000108028	0	0	0	0	0	0
ENSMUSG00000111157	0	0	0	0	0	0
ENSMUSG00000026531	0	0	0	0	0	0
ENSMUSG00000061367	0	0	0	0	0	0
ENSMUSG00000044556	15	6	18	8	5	11
ENSMUSG00000044551	50	33	53	16	31	50
ENSMUSG00000109819	0	0	0	0	0	0
ENSMUSG00000051414	0	0	0	0	0	0
ENSMUSG00000097972	0	0	0	0	0	0
ENSMUSG00000109813	0	0	0	0	0	0
ENSMUSG00000097973	8	6	15	6	7	10
ENSMUSG00000078208	0	0	0	0	0	0
ENSMUSG00000078206	0	0	0	0	0	0
ENSMUSG00000055978	17	24	35	70	97	126
ENSMUSG00000097976	0	0	0	0	0	0
ENSMUSG00000095076	0	0	0	0	0	0
ENSMUSG00000095075	0	0	0	0	0	0
ENSMUSG00000095074	0	0	0	0	0	0
ENSMUSG00000109815	0	0	0	0	0	0
ENSMUSG00000095071	0	0	0	0	0	0
ENSMUSG00000095070	0	0	0	0	0	0
ENSMUSG00000098290	0	0	0	0	0	0
ENSMUSG00000026432	0	0	0	0	0	0
ENSMUSG00000098294	0	0	0	0	0	0
ENSMUSG00000098295	0	0	0	0	0	0
ENSMUSG00000027376	0	0	0	12	10	2
ENSMUSG00000027379	66	39	47	0	6	26
ENSMUSG00000052281	0	0	0	0	2	4
ENSMUSG00000090598	2	0	1	2	1	2
ENSMUSG00000090594	0	0	0	0	0	0
ENSMUSG00000082114	3	3	0	0	3	0
ENSMUSG00000073051	0	0	0	0	0	0
ENSMUSG00000032671	0	4	3	0	4	0
ENSMUSG00000073052	0	0	0	0	0	0
ENSMUSG00000080616	0	0	0	0	0	0
ENSMUSG00000111078	0	0	3	0	0	0
ENSMUSG00000082117	0	0	0	0	0	0
ENSMUSG00000113390	0	0	2	0	0	2
ENSMUSG00000086618	0	0	0	0	0	0
ENSMUSG00000086613	0	0	0	0	0	0
ENSMUSG00000086614	0	0	0	0	0	0
ENSMUSG00000111074	0	0	0	0	0	0
ENSMUSG00000095344	0	0	0	0	0	0
ENSMUSG00000111679	0	0	0	0	0	0
ENSMUSG00000007107	0	0	4	0	0	0
ENSMUSG00000098742	0	0	0	2	0	1
ENSMUSG00000098743	1	0	1	0	1	2
ENSMUSG00000098740	0	0	0	0	0	0
ENSMUSG00000095346	0	0	0	0	0	0
ENSMUSG00000098747	0	0	0	0	0	0
ENSMUSG00000098744	1	0	0	0	0	0
ENSMUSG00000098745	0	0	0	0	0	0
ENSMUSG00000111070	0	0	0	0	0	0
ENSMUSG00000098748	0	0	0	0	0	0
ENSMUSG00000098749	2	2	1	3	4	3

ENSMUSG00000111071	0	0	0	0	0	0
ENSMUSG00000047773	0	0	0	2	0	0
ENSMUSG00000095342	0	0	0	0	1	0
ENSMUSG00000093029	0	0	0	0	0	0
ENSMUSG00000093024	0	0	0	0	0	0
ENSMUSG00000093025	0	0	0	0	0	0
ENSMUSG00000093021	1	0	0	0	0	0
ENSMUSG00000075589	5	3	0	0	8	0
ENSMUSG00000112602	0	0	0	0	0	0
ENSMUSG00000112603	0	0	0	0	0	0
ENSMUSG00000109062	0	0	0	0	0	0
ENSMUSG00000081167	0	5	0	2	0	5
ENSMUSG00000076530	0	0	0	0	0	0
ENSMUSG00000073949	0	0	0	0	0	0
ENSMUSG00000073948	0	0	0	0	0	0
ENSMUSG00000073943	0	0	0	0	0	0
ENSMUSG00000073946	0	0	0	1	0	0
ENSMUSG00000073945	0	0	0	0	0	0
ENSMUSG00000073944	0	0	0	0	0	0
ENSMUSG00000040797	90	158	81	108	130	74
ENSMUSG00000111051	0	0	0	0	0	0
ENSMUSG00000039981	29	40	74	12	13	10
ENSMUSG00000030616	88	96	131	37	47	63
ENSMUSG00000030617	0	20	18	1	6	6
ENSMUSG00000099509	0	0	0	0	0	0
ENSMUSG00000099508	0	0	0	0	0	0
ENSMUSG00000099502	0	0	0	0	3	0
ENSMUSG00000099504	0	0	0	0	0	0
ENSMUSG00000037346	0	0	0	0	0	0
ENSMUSG00000084501	0	0	0	0	0	0
ENSMUSG00000083732	11	10	3	2	13	7
ENSMUSG00000083731	0	0	1	0	0	0
ENSMUSG00000084502	0	0	0	0	0	0
ENSMUSG00000083735	0	0	0	0	0	0
ENSMUSG00000043843	2	0	0	0	0	0
ENSMUSG00000100076	0	0	0	0	0	0
ENSMUSG00000100077	0	0	0	0	0	0
ENSMUSG00000100072	0	0	0	0	0	0
ENSMUSG00000100073	0	0	0	0	0	0
ENSMUSG00000100070	0	0	0	0	0	0
ENSMUSG00000100071	4	8	5	4	4	5
ENSMUSG00000100079	0	0	0	2	0	0
ENSMUSG00000102500	0	0	0	0	0	0
ENSMUSG00000102501	48	37	35	24	24	62
ENSMUSG00000102502	0	0	0	0	0	0
ENSMUSG00000102504	162	152	122	78	55	80
ENSMUSG00000102505	0	0	0	0	0	6
ENSMUSG00000102506	0	0	0	0	0	0
ENSMUSG00000102507	0	0	0	0	0	0
ENSMUSG00000102508	0	0	4	0	3	1
ENSMUSG00000075044	0	0	0	0	0	0
ENSMUSG00000075045	0	0	0	1	0	0
ENSMUSG00000075046	0	0	0	0	0	0
ENSMUSG00000000223	33	33	47	10	12	22
ENSMUSG00000075042	0	0	2	0	0	0
ENSMUSG00000065836	2	2	2	0	0	0
ENSMUSG00000065837	0	0	0	0	0	0
ENSMUSG00000026459	0	0	3	0	0	0

ENSMUSG00000074397	0	0	0	0	0	0
ENSMUSG00000079619	0	0	0	0	0	0
ENSMUSG00000088509	0	0	0	0	0	0
ENSMUSG00000101858	0	0	0	0	0	0
ENSMUSG00000088505	0	0	0	0	0	0
ENSMUSG00000088504	0	0	1	0	2	1
ENSMUSG00000101855	2	0	1	2	0	2
ENSMUSG00000101854	2	10	5	5	4	4
ENSMUSG00000088501	0	0	0	0	0	0
ENSMUSG00000101852	0	0	0	0	0	0
ENSMUSG00000101851	1	0	0	0	0	0
ENSMUSG00000088502	0	0	0	0	0	0
ENSMUSG00000039278	0	0	0	0	0	0
ENSMUSG00000085368	0	0	0	0	0	0
ENSMUSG00000085369	0	0	0	0	0	0
ENSMUSG00000036766	0	5	2	0	0	0
ENSMUSG00000103757	0	0	0	0	0	0
ENSMUSG00000103755	4	0	0	3	0	3
ENSMUSG00000065307	0	0	0	0	1	0
ENSMUSG00000065303	0	0	0	0	0	0
ENSMUSG00000103758	0	0	0	0	0	0
ENSMUSG00000029451	0	0	0	0	0	0
ENSMUSG00000063935	2	0	0	1	0	0
ENSMUSG00000109874	0	0	0	0	2	0
ENSMUSG00000063932	0	0	0	0	0	0
ENSMUSG00000074026	0	0	1	0	0	0
ENSMUSG00000074029	2	2	0	0	0	0
ENSMUSG00000040490	6	32	10	16	11	12
ENSMUSG00000104390	0	0	0	0	0	0
ENSMUSG00000073554	0	0	0	0	0	0
ENSMUSG00000073551	0	0	0	0	0	0
ENSMUSG00000097241	0	0	0	0	0	0
ENSMUSG00000063681	22	33	6	55	78	76
ENSMUSG00000063683	0	0	0	0	0	0
ENSMUSG00000097248	0	0	0	0	3	4
ENSMUSG00000035238	0	0	0	0	3	0
ENSMUSG00000104113	5	6	20	10	11	19
ENSMUSG00000106584	2	0	0	0	2	2
ENSMUSG00000093429	6	10	1	7	2	8
ENSMUSG00000038408	0	0	0	0	0	0
ENSMUSG00000034185	0	0	0	0	0	0
ENSMUSG00000038402	7	8	0	0	4	0
ENSMUSG00000051136	0	0	0	0	0	0
ENSMUSG00000102085	0	0	0	0	0	0
ENSMUSG00000102084	0	6	10	0	0	0
ENSMUSG00000114538	0	3	0	2	0	0
ENSMUSG00000084616	0	13	2	2	4	7
ENSMUSG00000102081	0	0	0	0	0	0
ENSMUSG00000102083	4	1	4	5	1	1
ENSMUSG00000102082	0	0	0	0	0	0
ENSMUSG00000100810	0	0	0	0	0	0
ENSMUSG00000083643	0	0	0	2	0	0
ENSMUSG00000114530	0	0	0	0	0	0
ENSMUSG00000100813	9	3	13	8	20	10
ENSMUSG00000114536	4	0	0	4	0	0
ENSMUSG00000102088	0	0	0	0	0	0
ENSMUSG00000114534	0	0	0	0	0	0
ENSMUSG00000100817	0	0	0	0	0	0

ENSMUSG00000096194	0	0	0	0	0	0
ENSMUSG00000096197	0	0	0	2	0	6
ENSMUSG00000096190	0	0	0	0	0	1
ENSMUSG00000094430	0	0	0	0	0	0
ENSMUSG00000084612	0	0	0	0	0	0
ENSMUSG00000096198	0	0	0	0	0	0
ENSMUSG00000093427	0	0	0	0	0	0
ENSMUSG00000054568	0	0	0	0	3	0
ENSMUSG00000094432	0	0	0	0	0	0
ENSMUSG00000050663	74	74	13	47	43	55
ENSMUSG00000105365	1	0	0	0	1	0
ENSMUSG00000105364	0	0	1	0	1	0
ENSMUSG00000105366	17	16	6	6	3	13
ENSMUSG00000105360	0	0	0	0	0	0
ENSMUSG00000108623	0	0	0	0	0	0
ENSMUSG00000108622	4	0	0	1	3	0
ENSMUSG00000108620	0	0	0	0	0	0
ENSMUSG00000108627	0	0	0	0	0	0
ENSMUSG00000091972	0	0	0	0	0	0
ENSMUSG00000024521	15	25	37	6	13	14
ENSMUSG00000108629	0	1	0	0	0	0
ENSMUSG00000105942	19	12	7	16	10	10
ENSMUSG00000105949	0	0	0	0	0	0
ENSMUSG00000021239	0	0	0	0	0	0
ENSMUSG00000101068	0	0	0	0	0	0
ENSMUSG00000101069	0	0	0	0	0	0
ENSMUSG00000101066	1	1	0	0	0	0
ENSMUSG00000104015	0	0	0	0	0	0
ENSMUSG00000101064	0	0	0	0	0	0
ENSMUSG00000104017	23	16	6	8	0	5
ENSMUSG00000101063	0	0	0	0	0	0
ENSMUSG00000101060	0	0	0	0	0	0
ENSMUSG00000101061	0	4	6	10	0	0
ENSMUSG00000090881	12	5	24	3	4	13
ENSMUSG00000086644	0	3	9	1	4	0
ENSMUSG00000095809	0	0	0	0	0	0
ENSMUSG00000111019	1	0	0	4	5	0
ENSMUSG00000111013	0	0	0	0	0	0
ENSMUSG00000111010	0	0	0	0	0	0
ENSMUSG00000111014	0	0	0	0	0	0
ENSMUSG00000022330	0	0	0	0	0	0
ENSMUSG00000064822	0	0	0	0	0	0
ENSMUSG00000064824	0	0	0	0	0	0
ENSMUSG00000114600	0	0	0	0	2	0
ENSMUSG00000059429	2	1	0	0	1	0
ENSMUSG00000091358	0	0	0	0	0	0
ENSMUSG00000018211	0	0	0	0	0	0
ENSMUSG00000022335	281	297	269	222	269	273
ENSMUSG00000091350	0	0	0	0	0	0
ENSMUSG00000106688	9	12	11	3	17	5
ENSMUSG00000081108	0	0	0	0	0	0
ENSMUSG00000099114	1	1	5	2	0	1
ENSMUSG00000081101	0	0	0	0	0	0
ENSMUSG00000110118	0	0	0	0	0	0
ENSMUSG00000110119	0	0	0	0	0	0
ENSMUSG00000110947	0	0	0	2	0	2
ENSMUSG00000110946	0	0	0	0	0	0
ENSMUSG00000110943	0	0	0	0	0	0

ENSMUSG00000110942	0	0	0	0	0	0
ENSMUSG00000110941	0	0	0	0	0	0
ENSMUSG00000092774	0	0	0	0	0	0
ENSMUSG00000027173	63	82	55	51	50	88
ENSMUSG00000102434	0	5	0	0	0	0
ENSMUSG00000028310	0	0	0	0	0	0
ENSMUSG00000056073	58	57	41	81	79	88
ENSMUSG00000006546	0	0	0	0	0	0
ENSMUSG00000110110	0	0	0	0	0	0
ENSMUSG00000095802	0	0	0	0	0	0
ENSMUSG00000090751	0	0	0	0	0	0
ENSMUSG00000095501	0	0	0	0	0	0
ENSMUSG00000025172	8	19	11	0	17	18
ENSMUSG00000053640	0	0	0	0	0	0
ENSMUSG00000042240	0	0	0	0	0	0
ENSMUSG00000094458	0	0	0	0	0	0
ENSMUSG00000042243	0	0	0	0	0	0
ENSMUSG00000042248	0	0	0	0	0	0
ENSMUSG00000025176	39	28	33	15	16	18
ENSMUSG00000094457	0	0	0	0	0	1
ENSMUSG00000094454	0	0	0	0	0	0
ENSMUSG00000112092	0	0	0	0	0	0
ENSMUSG00000112097	0	0	0	0	0	0
ENSMUSG00000112098	0	0	0	0	0	0
ENSMUSG00000086455	0	0	0	0	0	0
ENSMUSG00000002190	0	0	0	0	0	0
ENSMUSG00000052516	89	129	35	105	118	139
ENSMUSG00000089418	0	0	0	0	0	0
ENSMUSG00000097013	13	18	5	12	9	6
ENSMUSG00000093228	0	0	0	0	0	0
ENSMUSG00000027827	91	134	145	84	83	128
ENSMUSG00000093224	1	0	0	0	0	0
ENSMUSG00000070465	4	3	12	0	0	0
ENSMUSG00000093226	0	0	0	0	0	0
ENSMUSG00000051896	0	0	0	0	0	0
ENSMUSG00000093221	0	0	0	0	0	0
ENSMUSG00000082898	0	0	0	0	0	0
ENSMUSG00000097908	30	16	38	20	22	42
ENSMUSG00000036244	0	11	5	0	3	0
ENSMUSG00000097901	12	11	9	3	8	5
ENSMUSG00000097907	0	0	0	0	0	0
ENSMUSG00000110832	1	2	0	0	0	0
ENSMUSG00000112636	0	0	0	0	0	0
ENSMUSG00000095929	0	0	0	0	0	0
ENSMUSG00000086306	0	0	0	0	0	0
ENSMUSG00000112633	2	0	1	1	0	0
ENSMUSG00000112631	0	0	0	0	0	0
ENSMUSG00000112630	0	0	0	0	0	0
ENSMUSG00000089414	0	0	0	0	0	0
ENSMUSG00000049372	0	0	0	0	0	0
ENSMUSG00000113322	0	2	4	3	3	2
ENSMUSG00000113323	0	1	0	0	0	0
ENSMUSG00000113320	0	0	0	0	0	0
ENSMUSG00000113327	0	0	0	0	0	0
ENSMUSG00000113324	0	4	0	0	0	4
ENSMUSG00000113325	0	0	0	0	0	0
ENSMUSG00000089415	0	0	0	0	0	0
ENSMUSG00000113329	0	0	0	0	0	0

ENSMUSG00000047363	23	9	24	3	25	7
ENSMUSG00000102181	0	0	0	0	0	0
ENSMUSG00000109238	0	5	3	0	0	0
ENSMUSG00000089416	0	0	0	0	0	0
ENSMUSG00000109230	0	0	0	0	0	0
ENSMUSG00000109237	4	4	2	0	2	2
ENSMUSG00000106577	0	0	0	0	0	0
ENSMUSG00000106571	0	0	0	0	0	0
ENSMUSG00000106570	65	52	37	38	53	68
ENSMUSG00000106573	0	0	0	0	0	0
ENSMUSG00000106578	0	0	0	0	0	0
ENSMUSG00000080517	0	0	0	0	0	0
ENSMUSG00000114671	0	0	0	0	0	0
ENSMUSG00000114673	0	0	0	0	0	0
ENSMUSG00000110051	3	3	4	3	2	0
ENSMUSG00000114675	6	1	0	0	0	0
ENSMUSG00000114674	0	0	0	0	0	0
ENSMUSG00000114677	0	0	0	0	0	0
ENSMUSG00000114676	0	0	0	0	1	0
ENSMUSG00000114679	0	0	0	0	0	0
ENSMUSG00000114678	2	2	0	4	5	2
ENSMUSG00000110160	0	0	0	0	0	0
ENSMUSG00000110162	2	0	0	0	0	0
ENSMUSG00000077954	0	0	0	0	0	0
ENSMUSG00000054003	0	0	0	0	0	0
ENSMUSG00000054005	0	0	0	0	0	0
ENSMUSG00000005251	28	20	19	16	12	12
ENSMUSG00000047986	15	14	20	11	9	39
ENSMUSG00000047980	0	0	0	0	0	0
ENSMUSG00000105005	0	0	0	0	0	0
ENSMUSG00000105006	10	9	12	6	20	15
ENSMUSG00000105000	0	0	0	0	0	0
ENSMUSG00000105002	0	0	0	0	0	0
ENSMUSG00000105008	0	5	0	0	2	4
ENSMUSG00000089413	0	0	0	0	0	0
ENSMUSG00000101232	0	0	0	0	0	0
ENSMUSG00000083242	0	0	0	0	0	0
ENSMUSG00000083241	4	0	2	0	0	2
ENSMUSG00000083245	6	26	18	14	11	13
ENSMUSG00000083244	4	1	0	2	3	0
ENSMUSG00000083249	0	0	0	0	0	0
ENSMUSG00000083248	0	0	0	0	0	0
ENSMUSG00000088126	0	0	0	0	0	0
ENSMUSG00000068536	0	0	0	0	0	0
ENSMUSG00000068537	0	0	0	2	0	0
ENSMUSG00000068535	0	0	0	0	0	0
ENSMUSG00000085160	4	0	4	4	0	0
ENSMUSG00000085165	0	0	0	0	0	0
ENSMUSG00000085167	0	0	0	0	2	0
ENSMUSG00000095498	0	0	0	0	0	0
ENSMUSG00000114936	3	0	4	3	0	0
ENSMUSG00000026700	0	0	0	0	0	0
ENSMUSG00000041975	167	214	204	163	170	209
ENSMUSG00000101750	1	2	5	2	3	2
ENSMUSG00000090168	0	0	0	0	3	0
ENSMUSG00000029636	48	52	63	45	32	57
ENSMUSG00000101753	0	0	0	0	0	0
ENSMUSG00000074625	0	0	0	0	0	0

ENSMUSG00000101754	0	0	0	0	0	0
ENSMUSG00000074628	3	0	0	0	0	0
ENSMUSG00000101755	0	0	0	0	0	0
ENSMUSG00000083867	0	0	0	0	0	0
ENSMUSG00000083866	5	5	7	2	8	10
ENSMUSG00000083864	0	0	0	0	0	0
ENSMUSG00000101756	0	0	0	0	0	0
ENSMUSG00000083861	0	0	0	0	0	0
ENSMUSG00000083860	0	0	0	0	0	3
ENSMUSG00000101757	0	0	0	0	0	0
ENSMUSG00000083869	0	0	0	0	0	0
ENSMUSG00000083868	0	0	0	0	0	0
ENSMUSG00000053360	0	0	3	0	0	0
ENSMUSG00000073396	0	0	0	0	0	0
ENSMUSG00000019876	37	87	83	48	30	17
ENSMUSG00000053367	0	6	1	0	3	0
ENSMUSG00000019874	9	0	0	0	5	0
ENSMUSG00000053368	2	3	0	0	0	3
ENSMUSG00000073399	0	4	0	4	4	8
ENSMUSG00000078017	0	0	0	0	0	0
ENSMUSG00000090254	0	0	0	0	0	0
ENSMUSG00000090255	0	0	7	7	7	7
ENSMUSG00000090256	3	13	0	11	0	7
ENSMUSG00000090259	0	2	2	0	0	0
ENSMUSG00000098467	0	0	0	0	0	0
ENSMUSG00000076458	0	0	0	0	0	0
ENSMUSG00000098469	0	0	0	0	0	0
ENSMUSG00000042724	20	20	32	5	2	10
ENSMUSG00000032937	0	0	0	0	0	0
ENSMUSG00000032936	0	0	0	0	0	0
ENSMUSG00000046615	0	0	0	0	0	0
ENSMUSG00000046610	0	0	4	5	0	6
ENSMUSG00000096079	0	0	0	0	0	0
ENSMUSG00000048544	15	14	15	18	22	9
ENSMUSG00000048540	0	0	0	0	0	0
ENSMUSG00000092487	0	0	0	0	0	0
ENSMUSG00000031074	0	0	0	0	0	0
ENSMUSG00000024598	220	133	528	27	45	50
ENSMUSG00000113342	0	0	0	0	0	0
ENSMUSG00000031079	82	87	90	58	53	58
ENSMUSG00000086919	0	0	0	0	0	0
ENSMUSG00000004864	0	0	8	0	0	0
ENSMUSG00000090291	8	11	11	8	15	10
ENSMUSG00000082383	0	18	13	14	14	17
ENSMUSG00000030792	0	0	0	0	0	0
ENSMUSG00000082389	0	0	0	0	0	0
ENSMUSG00000082388	0	0	0	0	0	0
ENSMUSG00000087152	0	0	0	0	0	4
ENSMUSG00000031772	0	0	0	2	0	0
ENSMUSG00000031775	26	10	8	0	12	8
ENSMUSG00000101264	0	0	0	0	0	0
ENSMUSG00000101265	0	0	0	0	0	0
ENSMUSG00000101266	2	0	0	0	0	0
ENSMUSG00000031779	0	6	0	4	0	0
ENSMUSG00000101260	0	0	0	0	0	0
ENSMUSG00000101261	0	0	0	0	0	0
ENSMUSG00000101263	0	0	0	0	0	0
ENSMUSG00000030424	150	210	157	152	133	166

ENSMUSG00000030427	9	24	16	5	8	14
ENSMUSG00000082652	0	0	0	0	0	0
ENSMUSG00000082650	0	0	0	0	0	0
ENSMUSG00000082657	0	0	0	0	0	0
ENSMUSG00000082656	0	0	0	0	0	0
ENSMUSG00000014747	12	14	21	6	17	17
ENSMUSG00000040610	0	0	0	0	0	0
ENSMUSG00000104986	8	7	6	3	0	10
ENSMUSG00000107953	0	2	0	0	2	0
ENSMUSG00000107956	0	0	0	0	0	0
ENSMUSG00000107957	0	0	0	0	0	0
ENSMUSG00000107959	0	0	0	0	0	0
ENSMUSG00000108471	2	3	0	0	0	0
ENSMUSG00000104982	0	0	0	0	0	0
ENSMUSG00000104980	0	0	0	0	0	0
ENSMUSG00000066684	24	20	32	13	10	25
ENSMUSG00000032482	20	20	19	12	23	39
ENSMUSG00000032484	0	0	0	0	0	0
ENSMUSG00000084718	0	0	0	0	0	0
ENSMUSG00000084719	0	0	0	0	0	0
ENSMUSG00000059867	0	0	0	0	0	0
ENSMUSG00000084710	0	0	0	0	0	0
ENSMUSG00000084713	0	0	0	0	0	0
ENSMUSG00000084714	0	0	0	0	0	0
ENSMUSG00000047161	0	0	0	0	0	0
ENSMUSG00000100605	0	0	0	0	0	0
ENSMUSG00000100604	0	0	0	0	0	0
ENSMUSG00000100607	0	0	0	0	0	0
ENSMUSG00000041567	6	3	0	5	14	8
ENSMUSG00000100600	2	0	0	0	2	0
ENSMUSG00000100603	0	4	0	6	3	4
ENSMUSG00000100602	9	7	5	9	4	9
ENSMUSG00000100609	0	0	0	0	0	0
ENSMUSG00000100608	0	0	0	0	0	0
ENSMUSG00000022479	21	35	36	21	10	14
ENSMUSG00000064775	0	0	0	0	0	0
ENSMUSG00000064776	0	0	0	0	0	0
ENSMUSG00000064777	0	0	0	0	0	0
ENSMUSG00000064772	0	2	0	0	0	0
ENSMUSG00000064779	0	0	0	0	0	0
ENSMUSG00000107375	2	5	3	2	2	2
ENSMUSG00000062987	0	0	0	0	0	0
ENSMUSG00000107377	0	1	0	0	0	0
ENSMUSG00000107370	27	33	37	26	19	44
ENSMUSG00000107371	0	0	0	0	0	0
ENSMUSG00000107373	16	10	9	18	13	14
ENSMUSG00000000416	72	81	72	82	92	87
ENSMUSG00000061184	0	0	0	0	2	0
ENSMUSG00000061186	23	8	18	31	19	30
ENSMUSG00000066680	0	0	0	0	0	0
ENSMUSG00000085447	0	0	0	0	0	0
ENSMUSG00000085445	15	0	10	22	12	10
ENSMUSG00000085444	0	0	0	1	4	0
ENSMUSG00000085441	0	0	0	0	0	0
ENSMUSG00000035683	45	20	25	4	12	13
ENSMUSG00000035681	18	40	33	24	33	31
ENSMUSG00000103660	0	0	0	0	0	0
ENSMUSG00000085449	0	0	0	0	0	0

ENSMUSG00000085448	4	5	7	0	0	0
ENSMUSG00000079005	0	0	0	0	0	0
ENSMUSG00000079000	0	0	0	0	0	0
ENSMUSG00000036480	0	0	0	0	0	0
ENSMUSG00000028132	111	120	66	203	172	182
ENSMUSG00000028133	56	54	54	65	66	75
ENSMUSG00000104227	0	0	0	0	0	0
ENSMUSG00000104226	0	0	0	0	0	0
ENSMUSG00000069072	4	5	0	0	0	0
ENSMUSG00000103796	0	1	0	0	1	0
ENSMUSG00000104224	0	0	0	0	0	0
ENSMUSG00000021416	0	0	0	0	0	0
ENSMUSG00000094927	0	1	0	0	0	1
ENSMUSG00000021414	26	12	18	4	6	11
ENSMUSG00000094925	0	0	0	0	0	0
ENSMUSG00000094920	0	0	8	0	8	0
ENSMUSG00000094921	0	0	0	0	2	0
ENSMUSG00000029663	0	0	0	0	0	0
ENSMUSG00000029664	57	62	93	30	19	52
ENSMUSG00000088714	0	0	0	1	0	0
ENSMUSG00000113129	0	0	0	0	0	0
ENSMUSG00000088710	0	0	0	0	0	0
ENSMUSG00000100401	0	0	0	0	0	0
ENSMUSG00000059244	6	6	9	4	6	14
ENSMUSG00000059246	0	0	0	0	0	0
ENSMUSG00000100400	0	0	0	0	0	0
ENSMUSG00000063762	0	0	0	0	0	0
ENSMUSG00000051079	10	14	8	40	29	38
ENSMUSG00000051076	13	8	5	0	0	0
ENSMUSG00000113698	29	8	6	4	20	22
ENSMUSG00000106776	0	0	6	0	0	2
ENSMUSG00000106775	0	0	0	0	0	0
ENSMUSG00000106779	0	0	0	0	0	0
ENSMUSG00000100408	0	0	0	0	0	0
ENSMUSG00000113696	0	0	0	0	0	0
ENSMUSG00000113697	0	0	0	0	0	0
ENSMUSG00000113695	2	2	0	1	2	4
ENSMUSG00000104739	0	0	0	0	0	0
ENSMUSG00000104738	0	0	0	0	0	0
ENSMUSG00000104734	1	0	0	0	0	0
ENSMUSG00000104731	0	0	0	2	0	0
ENSMUSG00000104730	2	3	2	1	0	1
ENSMUSG00000104732	0	0	0	0	0	0
ENSMUSG00000114478	0	0	0	0	0	0
ENSMUSG00000114476	0	0	0	0	0	0
ENSMUSG00000114475	0	0	0	0	0	0
ENSMUSG00000114474	4	5	0	0	5	0
ENSMUSG00000114473	0	0	0	0	0	0
ENSMUSG00000114472	0	0	0	0	0	0
ENSMUSG00000114471	0	0	0	0	0	0
ENSMUSG00000108173	1	1	0	0	0	1
ENSMUSG00000097789	22	11	15	39	25	28
ENSMUSG00000097780	1	0	0	0	0	0
ENSMUSG00000091834	0	0	0	0	1	0
ENSMUSG00000091832	0	0	0	0	0	0
ENSMUSG00000102294	0	0	0	0	0	0
ENSMUSG00000102295	0	0	0	0	0	0
ENSMUSG00000109741	0	0	0	0	0	0

ENSMUSG00000102297	0	0	0	0	0	0
ENSMUSG00000109747	0	0	0	0	0	0
ENSMUSG00000109746	0	0	0	0	0	0
ENSMUSG00000109745	0	0	0	0	0	0
ENSMUSG00000109744	0	0	0	0	0	0
ENSMUSG00000057913	6	7	0	8	6	9
ENSMUSG00000109749	0	0	0	0	0	0
ENSMUSG00000109748	0	0	0	0	0	0
ENSMUSG00000102298	0	1	0	3	3	4
ENSMUSG00000058590	0	0	0	0	0	0
ENSMUSG00000058626	0	4	37	45	28	57
ENSMUSG00000058620	61	40	25	47	63	76
ENSMUSG00000050030	0	0	0	0	0	0
ENSMUSG00000058628	0	0	0	0	0	0
ENSMUSG00000077726	0	0	0	0	0	0
ENSMUSG00000104191	0	0	0	0	0	0
ENSMUSG00000104190	3	0	5	2	3	1
ENSMUSG00000104192	0	0	2	0	2	0
ENSMUSG00000093863	0	0	0	0	0	0
ENSMUSG00000104194	0	0	0	0	0	0
ENSMUSG00000104197	18	9	10	7	8	2
ENSMUSG00000104199	0	0	0	0	0	0
ENSMUSG00000104198	0	0	0	0	0	0
ENSMUSG00000101154	0	0	0	0	0	0
ENSMUSG00000096784	0	1	0	0	1	0
ENSMUSG00000096786	0	1	0	0	0	1
ENSMUSG00000096782	0	0	0	0	0	1
ENSMUSG00000096788	0	0	0	0	0	0
ENSMUSG00000110572	0	0	0	0	0	0
ENSMUSG00000110573	0	0	0	0	0	0
ENSMUSG00000110570	1	2	0	0	2	0
ENSMUSG00000110571	0	0	0	0	0	0
ENSMUSG00000060445	33	48	43	53	36	63
ENSMUSG00000095948	0	0	0	0	0	0
ENSMUSG00000026147	8	0	0	0	0	0
ENSMUSG00000020256	240	206	202	521	370	410
ENSMUSG00000096437	2	1	2	1	1	1
ENSMUSG00000096432	0	0	0	0	1	0
ENSMUSG00000113903	12	2	7	6	8	10
ENSMUSG00000113901	0	0	0	0	0	0
ENSMUSG00000113906	0	0	0	0	0	0
ENSMUSG00000113904	0	0	0	0	0	0
ENSMUSG00000113908	2	1	4	3	1	1
ENSMUSG00000113909	11	14	34	0	0	0
ENSMUSG00000108303	0	0	0	0	0	0
ENSMUSG00000056797	0	0	0	0	0	0
ENSMUSG00000095201	0	0	0	0	0	0
ENSMUSG00000095205	1	2	0	2	0	1
ENSMUSG00000095207	4	6	6	2	2	4
ENSMUSG00000047501	0	0	6	0	2	0
ENSMUSG00000047502	0	7	7	4	4	3
ENSMUSG00000047508	0	11	4	4	2	6
ENSMUSG00000111229	0	0	0	0	0	0
ENSMUSG00000111221	0	0	0	0	0	0
ENSMUSG00000111222	0	0	0	0	0	0
ENSMUSG00000111225	0	0	0	0	0	0
ENSMUSG00000111224	0	0	1	2	0	2
ENSMUSG00000081739	15	15	14	28	24	14

ENSMUSG00000090497	0	0	0	0	0	2
ENSMUSG00000090494	1	1	1	1	0	0
ENSMUSG00000064201	0	0	0	0	0	0
ENSMUSG00000081734	0	0	0	0	0	0
ENSMUSG00000081737	0	0	0	0	0	0
ENSMUSG00000081736	0	0	0	0	0	0
ENSMUSG00000094559	0	0	0	0	0	0
ENSMUSG00000094558	1	1	0	0	0	0
ENSMUSG00000094556	0	0	0	0	0	0
ENSMUSG00000094555	0	0	0	0	0	1
ENSMUSG00000094554	0	0	0	0	0	0
ENSMUSG00000094553	0	0	0	0	0	0
ENSMUSG00000094550	1	0	0	0	0	0
ENSMUSG00000027469	124	121	91	25	63	90
ENSMUSG00000056133	0	0	0	0	0	0
ENSMUSG00000017049	0	0	0	0	0	0
ENSMUSG00000027468	0	0	0	0	0	0
ENSMUSG00000048706	127	152	140	124	111	168
ENSMUSG00000046750	0	0	0	0	0	0
ENSMUSG00000077653	0	0	0	0	0	0
ENSMUSG00000077650	0	0	0	0	0	0
ENSMUSG00000077655	0	0	0	0	0	0
ENSMUSG00000077659	0	0	0	1	0	3
ENSMUSG00000086172	0	0	0	0	0	0
ENSMUSG00000086173	0	0	0	0	0	0
ENSMUSG00000086170	0	0	0	0	0	0
ENSMUSG00000086171	2	0	0	0	2	0
ENSMUSG00000086176	0	0	0	0	0	0
ENSMUSG00000086177	0	0	0	0	0	0
ENSMUSG00000086175	0	0	0	0	0	0
ENSMUSG00000086179	0	0	0	0	0	0
ENSMUSG00000098135	2	0	0	0	0	0
ENSMUSG00000082821	0	0	0	0	0	0
ENSMUSG00000102390	0	0	2	0	0	2
ENSMUSG00000102393	0	0	0	0	0	0
ENSMUSG00000096847	0	0	0	0	0	0
ENSMUSG00000096845	0	0	1	0	0	0
ENSMUSG00000098860	0	0	0	0	0	0
ENSMUSG00000098867	0	0	0	0	0	0
ENSMUSG00000096840	2	0	0	0	0	0
ENSMUSG00000082411	0	0	0	0	3	0
ENSMUSG00000082410	0	0	0	0	0	0
ENSMUSG00000098869	0	0	0	0	0	0
ENSMUSG00000098868	0	0	1	1	1	0
ENSMUSG00000082415	0	0	0	0	0	0
ENSMUSG00000082414	7	8	4	4	3	4
ENSMUSG00000096849	0	0	0	0	0	0
ENSMUSG00000096848	0	0	0	0	0	0
ENSMUSG00000102397	0	0	0	0	0	0
ENSMUSG00000102396	0	0	0	0	0	0
ENSMUSG00000105166	4	6	2	0	0	0
ENSMUSG00000045812	0	0	0	0	0	0
ENSMUSG00000045813	54	25	68	24	15	8
ENSMUSG00000045815	0	0	0	0	0	0
ENSMUSG00000071112	0	11	13	12	4	0
ENSMUSG00000071113	0	0	0	0	0	2
ENSMUSG00000070868	17	10	16	6	6	8
ENSMUSG00000070866	0	0	0	3	0	0

ENSMUSG00000050957	12	18	14	0	4	9
ENSMUSG00000104918	8	6	10	4	0	0
ENSMUSG00000040364	15	15	15	9	12	18
ENSMUSG00000084155	0	0	0	0	0	3
ENSMUSG00000084152	0	0	0	0	0	0
ENSMUSG00000084150	0	0	0	0	0	0
ENSMUSG00000084151	0	4	3	3	0	0
ENSMUSG00000084158	0	0	0	0	0	0
ENSMUSG00000082346	0	0	0	0	0	0
ENSMUSG00000080813	10	6	6	4	3	8
ENSMUSG00000080812	0	0	0	0	0	0
ENSMUSG00000080817	0	0	2	0	0	2
ENSMUSG00000080816	6	5	6	7	5	7
ENSMUSG00000080815	2	0	0	0	0	0
ENSMUSG00000080814	0	0	4	0	0	3
ENSMUSG00000080819	0	0	0	0	0	0
ENSMUSG00000080818	0	0	0	0	1	0
ENSMUSG00000078737	0	0	0	0	0	0
ENSMUSG00000102131	0	0	0	0	0	0
ENSMUSG00000110905	0	0	0	0	0	0
ENSMUSG00000044065	0	0	0	0	0	0
ENSMUSG00000044062	2	0	0	0	0	3
ENSMUSG00000044061	0	0	0	0	0	0
ENSMUSG00000099019	0	0	0	0	0	0
ENSMUSG00000099011	0	0	0	0	0	0
ENSMUSG00000099010	0	0	0	0	0	0
ENSMUSG00000099015	0	0	0	0	0	0
ENSMUSG00000099017	0	1	1	0	0	0
ENSMUSG00000102134	0	0	0	0	0	0
ENSMUSG00000032012	54	60	104	32	40	38
ENSMUSG00000032013	0	0	7	0	0	0
ENSMUSG00000032017	7	3	1	3	5	0
ENSMUSG00000032015	0	0	7	0	0	0
ENSMUSG00000065709	0	0	0	0	0	0
ENSMUSG00000100218	0	0	0	2	0	0
ENSMUSG00000112971	0	3	0	0	0	0
ENSMUSG00000112973	0	0	0	0	0	0
ENSMUSG00000006651	73	48	48	12	51	44
ENSMUSG00000108539	0	0	0	0	0	0
ENSMUSG00000112977	0	0	0	0	0	0
ENSMUSG00000094926	0	0	0	0	0	0
ENSMUSG00000105989	0	0	0	0	0	0
ENSMUSG00000112979	0	0	0	0	0	0
ENSMUSG00000078198	0	0	0	0	0	0
ENSMUSG00000044164	4	0	0	0	0	0
ENSMUSG00000033949	74	56	62	55	79	103
ENSMUSG00000070108	0	0	0	0	0	0
ENSMUSG00000101359	0	0	0	0	0	0
ENSMUSG00000099906	0	0	0	0	0	0
ENSMUSG00000099905	0	0	0	0	0	0
ENSMUSG00000099904	0	0	0	0	0	0
ENSMUSG00000099903	0	0	0	0	0	0
ENSMUSG00000099901	0	0	0	1	0	1
ENSMUSG00000099900	0	0	0	0	0	0
ENSMUSG00000070105	0	0	0	0	0	0
ENSMUSG00000098052	3	0	2	0	0	0
ENSMUSG00000098053	0	0	0	0	0	0
ENSMUSG00000098051	0	0	3	2	0	3

ENSMUSG00000098056	9	5	5	3	7	5
ENSMUSG00000098054	0	0	0	0	0	0
ENSMUSG00000098055	63	58	45	50	40	55
ENSMUSG00000098058	0	2	0	0	0	2
ENSMUSG00000031097	20	19	20	31	34	42
ENSMUSG00000105985	264	226	133	356	283	335
ENSMUSG00000031099	161	184	147	110	100	118
ENSMUSG00000031098	3	0	16	0	0	0
ENSMUSG00000110345	8	4	0	0	7	0
ENSMUSG00000094923	0	0	0	1	0	0
ENSMUSG00000110341	12	11	19	29	22	37
ENSMUSG00000110340	0	0	0	0	0	0
ENSMUSG00000072778	0	0	0	0	0	0
ENSMUSG00000025370	0	0	0	0	0	0
ENSMUSG00000105986	2	13	10	0	5	5
ENSMUSG00000084905	0	0	0	0	0	0
ENSMUSG00000084904	40	42	31	39	60	82
ENSMUSG00000084901	0	0	0	0	0	0
ENSMUSG00000084902	0	0	0	0	0	0
ENSMUSG00000031635	0	0	0	2	0	0
ENSMUSG00000102908	0	0	0	0	0	0
ENSMUSG00000102904	0	0	0	0	0	0
ENSMUSG00000102906	0	0	0	0	0	0
ENSMUSG00000102907	0	0	2	0	0	2
ENSMUSG00000102901	0	0	0	0	0	0
ENSMUSG00000102902	0	0	0	0	0	0
ENSMUSG00000062737	0	0	0	0	0	0
ENSMUSG00000030324	8	6	24	5	24	0
ENSMUSG00000030325	6	6	8	0	6	2
ENSMUSG00000030329	18	15	16	0	13	3
ENSMUSG00000015627	20	12	4	0	0	0
ENSMUSG00000088138	0	0	0	0	0	0
ENSMUSG00000087346	0	2	0	1	0	0
ENSMUSG00000087344	4	3	0	9	7	6
ENSMUSG00000087345	0	0	0	0	0	0
ENSMUSG00000087343	0	0	0	0	0	0
ENSMUSG00000087340	4	0	0	0	0	8
ENSMUSG00000088133	0	0	0	0	0	0
ENSMUSG00000037674	882	886	869	604	685	703
ENSMUSG00000068083	0	0	0	0	0	0
ENSMUSG00000089530	1	0	0	0	1	2
ENSMUSG00000094929	0	0	0	0	0	0
ENSMUSG00000101765	0	0	0	0	0	0
ENSMUSG00000101764	0	0	0	2	0	0
ENSMUSG00000101766	0	0	0	0	0	0
ENSMUSG00000101761	0	0	0	0	0	0
ENSMUSG00000101763	0	0	0	2	0	2
ENSMUSG00000060131	133	151	188	39	29	41
ENSMUSG00000040009	25	32	20	12	28	16
ENSMUSG00000059857	1	4	3	5	14	3
ENSMUSG00000019368	0	0	0	0	0	0
ENSMUSG00000103697	193	226	225	166	187	187
ENSMUSG00000103695	0	0	0	0	0	0
ENSMUSG00000103690	0	2	0	0	0	0
ENSMUSG00000100415	0	0	0	0	0	0
ENSMUSG00000100416	0	0	0	0	0	0
ENSMUSG00000100417	0	0	0	0	0	0
ENSMUSG00000100412	0	0	0	0	0	0

ENSMUSG00000100413	0	0	0	1	0	0
ENSMUSG00000100418	0	0	0	0	0	0
ENSMUSG00000073964	0	0	0	0	0	0
ENSMUSG00000049010	0	0	0	2	0	0
ENSMUSG00000095959	0	0	0	0	0	0
ENSMUSG00000089773	0	0	0	0	0	0
ENSMUSG00000095954	0	0	0	0	0	0
ENSMUSG00000095956	0	0	0	2	2	0
ENSMUSG00000095957	3	0	2	0	0	0
ENSMUSG00000095950	0	0	0	0	1	0
ENSMUSG00000095951	0	0	0	0	0	0
ENSMUSG00000095952	0	0	0	0	0	0
ENSMUSG00000095953	0	0	0	0	0	0
ENSMUSG00000028078	135	123	148	67	136	123
ENSMUSG00000065414	1	0	0	0	0	0
ENSMUSG00000065415	0	0	0	0	0	0
ENSMUSG00000000103	0	0	0	0	0	0
ENSMUSG00000028071	8	16	7	0	10	4
ENSMUSG00000034687	116	105	128	35	26	53
ENSMUSG00000096576	0	0	0	0	0	0
ENSMUSG00000096574	15	0	15	11	0	0
ENSMUSG00000052942	117	126	90	35	53	53
ENSMUSG00000023577	0	0	0	0	0	0
ENSMUSG00000112440	0	1	0	0	0	0
ENSMUSG00000112444	0	0	0	0	0	0
ENSMUSG00000102128	0	0	0	0	0	0
ENSMUSG00000102129	0	0	0	0	0	0
ENSMUSG00000112448	2	2	0	0	0	0
ENSMUSG00000102127	0	0	0	0	0	0
ENSMUSG00000102124	0	0	0	0	0	0
ENSMUSG00000102125	0	0	0	0	0	0
ENSMUSG00000035523	0	0	0	0	0	0
ENSMUSG00000102123	0	0	0	0	0	0
ENSMUSG00000102120	0	0	0	0	0	2
ENSMUSG00000102121	0	0	0	0	0	0
ENSMUSG00000114848	0	0	0	0	0	0
ENSMUSG00000114849	0	0	0	0	0	0
ENSMUSG00000114841	0	0	0	0	0	0
ENSMUSG00000114847	0	0	0	0	0	0
ENSMUSG00000110358	0	0	0	0	0	0
ENSMUSG00000110359	0	0	0	0	0	0
ENSMUSG00000031933	0	0	0	0	0	0
ENSMUSG00000110356	0	0	0	0	0	0
ENSMUSG00000110354	0	0	0	0	0	0
ENSMUSG00000091407	0	0	0	0	0	0
ENSMUSG00000088693	0	0	0	0	0	0
ENSMUSG00000088692	0	0	0	0	0	0
ENSMUSG00000088697	0	0	0	0	0	0
ENSMUSG00000111918	14	20	6	20	13	28
ENSMUSG00000016626	0	0	0	0	3	0
ENSMUSG00000016624	120	99	112	98	104	126
ENSMUSG00000091404	0	0	1	0	0	1
ENSMUSG00000111914	0	0	0	0	0	3
ENSMUSG00000105699	6	2	5	3	2	0
ENSMUSG00000085987	0	0	5	0	0	4
ENSMUSG00000105695	21	12	7	6	4	8
ENSMUSG00000029032	30	6	19	11	19	14
ENSMUSG00000105693	0	0	0	0	0	0

ENSMUSG00000111912	2	0	0	0	0	0
ENSMUSG00000111439	2	0	2	0	0	0
ENSMUSG00000097082	16	20	18	27	12	14
ENSMUSG00000097086	0	0	0	0	0	0
ENSMUSG00000113718	0	0	0	0	0	0
ENSMUSG00000113717	0	0	0	0	0	8
ENSMUSG00000113715	0	0	0	0	0	0
ENSMUSG00000088960	0	0	0	0	0	0
ENSMUSG00000113713	0	0	0	0	0	0
ENSMUSG00000113712	0	0	0	0	0	0
ENSMUSG00000034690	0	0	0	0	0	0
ENSMUSG00000088964	1	0	0	0	0	0
ENSMUSG00000111436	2	7	5	3	1	1
ENSMUSG00000111403	0	0	0	0	0	0
ENSMUSG00000111405	0	0	0	0	0	0
ENSMUSG00000111404	0	0	0	0	0	0
ENSMUSG00000111406	0	0	0	0	0	0
ENSMUSG00000100273	0	0	0	0	0	0
ENSMUSG00000064043	177	124	197	69	79	73
ENSMUSG00000106924	3	3	3	4	5	6
ENSMUSG00000106925	0	0	0	1	0	0
ENSMUSG00000106928	21	28	44	36	48	35
ENSMUSG00000096320	0	0	0	0	0	0
ENSMUSG00000108313	0	0	0	0	0	0
ENSMUSG00000108311	9	0	0	0	0	0
ENSMUSG00000108315	0	0	0	0	0	0
ENSMUSG00000108314	94	64	106	61	81	96
ENSMUSG00000108318	0	4	0	0	0	0
ENSMUSG00000114158	0	0	0	0	0	0
ENSMUSG00000109950	0	0	0	0	0	0
ENSMUSG00000109951	0	0	0	0	0	0
ENSMUSG00000109956	31	44	30	21	16	35
ENSMUSG00000109955	0	0	0	0	3	0
ENSMUSG00000114152	7	6	5	5	5	3
ENSMUSG00000114153	3	4	19	0	0	0
ENSMUSG00000114154	11	0	0	0	0	2
ENSMUSG00000114156	0	0	0	0	0	0
ENSMUSG00000114157	7	2	7	20	15	6
ENSMUSG00000107446	0	0	0	0	0	0
ENSMUSG00000045132	0	0	0	0	0	0
ENSMUSG00000097624	4	0	0	0	0	0
ENSMUSG00000097627	0	0	0	0	0	0
ENSMUSG00000097621	5	0	0	0	0	0
ENSMUSG00000097622	0	3	0	0	0	0
ENSMUSG00000097623	0	0	0	0	0	0
ENSMUSG00000097628	3	7	3	1	1	5
ENSMUSG00000098137	0	0	0	0	0	0
ENSMUSG00000107440	10	7	6	17	6	6
ENSMUSG00000091668	0	0	0	0	0	0
ENSMUSG00000081106	5	2	6	0	0	0
ENSMUSG00000107441	0	0	0	0	0	0
ENSMUSG00000091662	0	0	0	0	0	0
ENSMUSG00000067724	22	15	18	11	14	14
ENSMUSG00000081107	0	0	0	0	0	0
ENSMUSG00000044265	0	0	0	0	0	0
ENSMUSG00000054459	0	0	0	0	0	3
ENSMUSG00000027663	598	432	542	265	368	427
ENSMUSG00000111306	0	0	0	0	0	0

ENSMUSG00000050201	0	0	0	0	0	0
ENSMUSG00000111300	0	0	0	0	0	0
ENSMUSG00000108367	0	7	4	0	0	0
ENSMUSG00000073835	0	0	0	0	0	0
ENSMUSG00000110437	0	0	0	2	0	0
ENSMUSG00000110435	0	0	1	1	0	0
ENSMUSG00000110434	3	9	7	11	5	14
ENSMUSG00000110431	0	0	0	0	0	0
ENSMUSG00000110430	0	0	0	0	0	0
ENSMUSG00000108360	0	0	0	0	0	0
ENSMUSG00000080349	0	0	0	0	0	0
ENSMUSG00000080348	0	0	0	0	0	0
ENSMUSG00000080347	0	0	0	0	0	0
ENSMUSG00000080346	1	1	0	0	0	0
ENSMUSG00000080345	0	0	0	0	0	0
ENSMUSG00000059049	342	199	338	11	9	18
ENSMUSG00000052479	0	0	0	0	0	0
ENSMUSG00000101239	0	0	0	0	0	0
ENSMUSG00000101238	0	0	0	0	0	0
ENSMUSG00000080698	0	0	0	0	0	0
ENSMUSG00000080695	0	0	0	0	0	0
ENSMUSG00000080694	0	0	0	0	0	0
ENSMUSG00000080697	0	0	0	0	0	0
ENSMUSG00000080696	0	0	0	0	0	0
ENSMUSG00000111388	2	7	4	2	9	8
ENSMUSG00000111389	56	54	27	30	26	43
ENSMUSG00000111386	0	0	0	0	0	0
ENSMUSG00000111387	0	0	0	0	0	0
ENSMUSG00000111384	3	0	0	0	0	0
ENSMUSG00000045609	2	2	2	1	3	3
ENSMUSG00000045608	0	0	0	3	0	0
ENSMUSG00000090042	0	0	0	0	0	0
ENSMUSG00000098214	0	0	0	0	0	0
ENSMUSG00000098216	0	0	0	0	0	0
ENSMUSG00000064954	0	0	0	1	0	0
ENSMUSG00000042372	0	0	0	0	0	0
ENSMUSG00000094746	0	0	0	0	0	0
ENSMUSG00000094745	0	0	0	0	2	0
ENSMUSG00000093531	0	0	0	0	0	0
ENSMUSG00000094741	0	0	0	0	0	0
ENSMUSG00000077199	0	0	2	0	0	0
ENSMUSG00000077190	0	0	0	0	0	0
ENSMUSG00000077194	0	0	0	2	1	0
ENSMUSG00000077196	0	0	0	0	0	0
ENSMUSG00000077197	0	0	0	0	0	0
ENSMUSG00000086696	0	0	0	0	0	0
ENSMUSG00000065568	1	2	1	0	0	0
ENSMUSG00000086698	0	0	0	0	0	0
ENSMUSG00000081569	0	0	0	0	0	0
ENSMUSG00000081560	0	0	0	0	0	0
ENSMUSG00000081561	1	1	0	0	0	2
ENSMUSG00000081563	0	0	0	0	0	0
ENSMUSG00000081566	0	0	0	0	0	0
ENSMUSG00000081567	0	0	0	0	0	0
ENSMUSG00000099679	0	0	0	0	0	0
ENSMUSG00000099678	0	0	0	0	0	0
ENSMUSG00000087167	0	0	0	0	0	0
ENSMUSG00000099671	0	0	0	0	0	0

ENSMUSG00000099670	0	0	1	0	0	0
ENSMUSG00000099673	0	0	0	0	0	0
ENSMUSG00000099672	0	0	0	0	0	0
ENSMUSG00000099675	0	0	0	0	0	0
ENSMUSG00000099674	0	0	0	0	0	0
ENSMUSG00000099677	0	0	0	0	0	0
ENSMUSG00000099676	0	0	0	0	0	0
ENSMUSG00000077442	0	0	0	0	0	0
ENSMUSG00000077441	0	0	0	0	0	0
ENSMUSG00000045684	0	0	0	0	0	0
ENSMUSG00000112127	2	5	0	0	0	0
ENSMUSG00000112126	0	0	0	0	0	0
ENSMUSG00000112125	0	0	0	0	0	0
ENSMUSG00000112123	0	0	0	0	0	0
ENSMUSG00000112122	0	1	1	1	3	1
ENSMUSG00000112121	27	41	22	50	40	55
ENSMUSG00000112120	9	2	18	0	0	0
ENSMUSG00000049864	0	0	0	0	1	0
ENSMUSG00000004988	16	15	9	10	12	8
ENSMUSG00000112128	0	0	0	0	0	0
ENSMUSG00000092372	0	0	0	0	0	0
ENSMUSG00000099589	0	0	0	0	0	1
ENSMUSG00000099588	0	0	0	0	0	0
ENSMUSG00000051965	5	3	0	3	2	4
ENSMUSG00000108091	0	0	0	0	0	0
ENSMUSG00000051969	6	18	24	3	4	3
ENSMUSG00000112754	0	0	0	0	0	0
ENSMUSG00000108092	1	2	1	2	1	0
ENSMUSG00000086037	0	0	0	0	0	0
ENSMUSG00000086036	0	0	0	0	0	0
ENSMUSG00000086035	2	3	6	0	0	1
ENSMUSG00000086034	6	0	0	2	7	0
ENSMUSG00000086031	0	4	0	0	0	0
ENSMUSG00000086030	0	0	0	0	0	0
ENSMUSG00000099473	0	0	0	0	0	0
ENSMUSG00000101588	0	0	0	0	0	0
ENSMUSG00000072672	0	0	0	5	0	0
ENSMUSG00000076313	0	0	0	0	1	0
ENSMUSG00000076316	0	1	0	0	0	1
ENSMUSG00000041735	0	0	0	0	0	0
ENSMUSG00000076318	0	0	0	0	0	0
ENSMUSG00000072679	0	0	3	0	0	0
ENSMUSG00000041730	6	7	6	9	4	6
ENSMUSG00000037477	4	4	0	9	0	5
ENSMUSG00000092986	0	0	0	0	0	0
ENSMUSG00000047894	0	2	0	0	0	0
ENSMUSG00000049249	0	4	0	5	2	5
ENSMUSG00000098982	0	0	0	0	0	0
ENSMUSG00000101582	0	0	0	0	0	0
ENSMUSG00000098984	0	0	0	0	0	0
ENSMUSG00000091670	0	0	0	0	0	0
ENSMUSG00000087636	0	0	0	0	1	0
ENSMUSG00000087637	0	0	0	0	0	0
ENSMUSG00000087638	5	3	0	0	0	0
ENSMUSG00000087639	31	9	20	27	18	5
ENSMUSG00000047295	0	9	2	0	3	2
ENSMUSG00000047293	0	0	0	2	0	2
ENSMUSG00000091676	0	0	0	0	0	0

ENSMUSG00000065561	0	0	1	0	0	2
ENSMUSG00000079699	3	0	6	0	2	0
ENSMUSG00000079697	11	3	2	3	1	0
ENSMUSG00000079693	0	0	0	0	0	3
ENSMUSG00000064129	0	0	0	0	0	0
ENSMUSG00000085484	0	0	0	0	0	0
ENSMUSG00000064121	0	0	0	0	0	0
ENSMUSG00000107525	0	0	0	0	0	0
ENSMUSG00000107527	0	0	0	0	0	0
ENSMUSG00000107521	0	0	0	0	0	0
ENSMUSG00000107520	0	0	0	0	0	0
ENSMUSG00000107523	1	0	0	0	0	0
ENSMUSG00000107522	0	0	0	0	0	0
ENSMUSG00000033579	0	0	0	0	2	0
ENSMUSG00000065658	0	2	0	0	1	0
ENSMUSG00000061578	11	2	0	3	1	4
ENSMUSG00000065656	0	0	0	0	0	0
ENSMUSG00000085675	0	0	0	2	0	0
ENSMUSG00000085676	0	0	0	0	0	0
ENSMUSG00000085677	0	0	0	0	0	0
ENSMUSG00000085679	0	0	0	0	0	0
ENSMUSG00000074311	0	0	0	0	0	0
ENSMUSG00000108644	0	0	0	0	0	0
ENSMUSG00000030909	0	0	0	0	0	0
ENSMUSG00000087292	0	0	0	0	0	0
ENSMUSG00000081954	0	0	0	0	0	0
ENSMUSG00000081959	0	0	0	1	0	0
ENSMUSG00000068151	0	0	0	0	0	0
ENSMUSG00000075958	0	0	0	0	0	0
ENSMUSG00000087295	0	0	0	0	0	0
ENSMUSG00000087296	0	0	0	0	0	0
ENSMUSG00000082665	8	4	2	4	4	4
ENSMUSG00000062007	0	8	0	0	0	0
ENSMUSG00000065384	0	0	0	0	0	0
ENSMUSG00000065385	0	0	0	0	0	0
ENSMUSG00000065382	0	0	0	0	0	0
ENSMUSG00000065380	0	0	0	0	0	0
ENSMUSG00000026546	29	32	27	14	9	16
ENSMUSG00000065388	0	0	0	0	2	2
ENSMUSG00000085094	0	0	0	0	0	0
ENSMUSG00000085090	0	0	0	0	0	0
ENSMUSG00000032221	112	53	69	10	15	61
ENSMUSG00000088624	0	0	0	0	0	0
ENSMUSG00000102008	0	0	0	0	0	0
ENSMUSG00000102005	0	0	0	0	0	0
ENSMUSG00000102004	0	0	0	0	0	0
ENSMUSG00000102006	0	0	0	0	0	0
ENSMUSG00000102001	0	0	0	0	0	0
ENSMUSG00000102000	0	0	0	0	0	0
ENSMUSG00000102003	0	0	0	0	0	0
ENSMUSG00000102002	0	0	0	0	0	0
ENSMUSG00000100891	0	0	0	0	0	0
ENSMUSG00000100892	0	0	0	0	0	0
ENSMUSG00000100893	0	0	0	0	0	0
ENSMUSG00000100894	0	0	0	0	0	0
ENSMUSG00000100895	0	0	0	0	0	0
ENSMUSG00000100896	0	0	0	0	0	0
ENSMUSG00000100897	2	0	0	0	0	0

ENSMUSG00000100898	0	0	0	0	0	0
ENSMUSG00000100899	1	0	0	0	0	0
ENSMUSG00000098157	0	0	0	0	0	2
ENSMUSG00000098156	5	5	3	0	4	8
ENSMUSG00000098154	5	4	3	1	2	6
ENSMUSG00000098153	4	2	0	3	0	6
ENSMUSG00000098152	0	0	0	0	0	0
ENSMUSG00000098150	0	0	0	0	0	0
ENSMUSG00000077832	0	0	0	0	0	0
ENSMUSG00000077830	0	0	0	0	0	0
ENSMUSG00000077836	0	0	0	0	0	0
ENSMUSG00000098159	0	0	0	0	0	0
ENSMUSG00000098158	47	59	67	44	43	68
ENSMUSG00000089898	0	0	0	0	0	0
ENSMUSG00000096119	0	0	0	0	0	0
ENSMUSG00000089891	0	0	0	0	0	0
ENSMUSG00000089892	0	0	0	0	0	0
ENSMUSG00000089893	0	0	0	0	0	0
ENSMUSG00000089894	0	0	0	0	0	0
ENSMUSG00000096110	0	0	0	0	0	0
ENSMUSG00000096113	0	1	1	0	0	0
ENSMUSG00000048875	0	0	0	4	0	3
ENSMUSG00000034107	5	0	5	6	9	0
ENSMUSG00000019982	2	3	5	0	6	5
ENSMUSG00000058287	0	0	0	0	0	0
ENSMUSG00000063063	0	5	1	0	0	2
ENSMUSG00000083135	0	0	0	0	0	0
ENSMUSG00000014852	0	0	0	0	0	0
ENSMUSG00000083137	0	0	0	0	0	0
ENSMUSG00000083136	0	0	0	0	0	0
ENSMUSG00000110660	20	12	11	11	8	18
ENSMUSG00000110661	4	6	0	0	0	4
ENSMUSG00000067528	0	3	1	0	0	0
ENSMUSG00000067529	5	4	4	3	0	0
ENSMUSG00000083138	0	0	0	0	0	0
ENSMUSG00000067524	0	0	0	0	0	0
ENSMUSG00000067525	0	3	0	0	0	0
ENSMUSG00000067526	0	0	1	0	1	0
ENSMUSG00000018168	18	39	56	17	24	14
ENSMUSG00000035131	0	2	0	0	0	0
ENSMUSG00000089190	0	0	0	0	0	0
ENSMUSG00000108848	0	0	0	0	0	0
ENSMUSG00000108847	0	0	0	0	0	0
ENSMUSG00000108846	1	4	2	4	2	2
ENSMUSG00000108845	0	0	0	0	1	0
ENSMUSG00000034818	12	0	0	0	4	0
ENSMUSG00000108842	20	39	40	32	18	16
ENSMUSG00000108841	4	3	0	0	2	0
ENSMUSG00000108840	2	0	7	0	0	0
ENSMUSG00000031710	0	0	0	0	0	0
ENSMUSG00000108916	2	0	0	0	0	0
ENSMUSG00000077590	0	0	0	0	0	0
ENSMUSG00000100335	0	0	0	0	0	0
ENSMUSG00000100334	0	0	0	0	0	0
ENSMUSG00000100337	1	0	0	10	9	4
ENSMUSG00000100336	0	0	0	0	0	0
ENSMUSG00000100331	0	0	0	0	0	0
ENSMUSG00000100330	0	0	0	0	0	0

ENSMUSG00000100333	0	0	0	0	0	0
ENSMUSG00000061961	0	0	0	0	0	0
ENSMUSG00000100339	0	0	0	0	0	0
ENSMUSG00000100338	0	0	0	0	0	0
ENSMUSG00000061969	0	0	0	0	0	0
ENSMUSG00000108301	0	0	0	0	0	0
ENSMUSG00000088301	0	0	0	0	0	0
ENSMUSG00000088304	0	0	0	0	0	0
ENSMUSG00000107644	0	0	0	0	3	0
ENSMUSG00000107645	0	0	0	0	0	0
ENSMUSG00000107646	0	0	0	0	0	0
ENSMUSG00000107641	0	0	0	0	0	0
ENSMUSG00000107642	0	0	0	1	3	0
ENSMUSG00000107648	0	3	0	0	0	0
ENSMUSG00000107649	0	0	0	0	0	0
ENSMUSG00000103152	2	0	0	0	0	0
ENSMUSG00000103158	0	0	0	0	0	0
ENSMUSG00000114757	0	0	0	0	0	0
ENSMUSG00000064727	0	0	0	0	0	0
ENSMUSG00000106060	0	0	0	0	0	0
ENSMUSG00000106067	21	27	24	17	17	18
ENSMUSG00000106066	0	0	0	0	0	0
ENSMUSG00000106065	0	0	0	0	0	0
ENSMUSG00000106064	0	0	0	0	0	0
ENSMUSG00000106069	0	0	0	0	0	0
ENSMUSG00000088620	0	0	0	0	0	0
ENSMUSG00000094236	0	0	0	0	0	0
ENSMUSG00000094237	0	0	0	0	0	0
ENSMUSG00000089312	0	0	0	0	0	0
ENSMUSG00000094235	0	0	0	0	0	0
ENSMUSG00000094232	0	0	0	0	0	0
ENSMUSG00000094233	0	0	0	0	0	0
ENSMUSG00000094230	0	0	0	0	0	0
ENSMUSG00000094238	2	1	1	1	1	2
ENSMUSG00000094239	0	0	0	0	0	0
ENSMUSG00000020033	0	2	0	0	0	2
ENSMUSG00000020037	0	0	0	0	0	0
ENSMUSG00000110259	0	0	0	0	0	0
ENSMUSG00000075203	0	0	0	0	0	0
ENSMUSG00000110257	0	0	0	0	0	0
ENSMUSG00000110255	0	0	0	0	0	0
ENSMUSG00000110254	0	0	0	0	0	0
ENSMUSG00000110253	0	0	0	0	0	0
ENSMUSG00000110252	0	0	0	0	0	0
ENSMUSG00000110251	2	4	0	1	2	0
ENSMUSG00000110250	0	0	0	0	0	0
ENSMUSG00000092173	0	0	0	0	0	0
ENSMUSG00000092172	0	0	0	0	0	0
ENSMUSG00000092171	18	9	17	3	21	23
ENSMUSG00000092175	0	0	0	0	0	0
ENSMUSG00000092174	0	0	0	0	0	2
ENSMUSG00000092179	0	0	0	0	0	0
ENSMUSG00000092178	24	38	21	23	0	0
ENSMUSG00000101329	0	0	0	0	0	0
ENSMUSG00000055067	306	344	369	375	337	452
ENSMUSG00000025127	17	18	59	0	8	0
ENSMUSG00000106683	8	5	10	8	16	14
ENSMUSG00000113508	0	0	0	0	0	0

ENSMUSG00000113509	0	0	0	0	0	0
ENSMUSG00000113506	4	5	0	0	0	1
ENSMUSG00000113507	0	0	0	7	0	0
ENSMUSG00000113505	10	0	2	2	0	1
ENSMUSG00000113502	12	3	13	6	14	8
ENSMUSG00000113503	0	0	0	0	0	0
ENSMUSG00000113500	0	0	0	0	0	0
ENSMUSG00000038721	4	0	0	0	0	6
ENSMUSG00000029206	51	49	50	23	32	56
ENSMUSG00000029205	0	0	0	0	0	0
ENSMUSG00000029204	96	70	149	64	54	50
ENSMUSG00000049573	0	0	0	0	0	0
ENSMUSG00000104989	0	0	0	0	0	0
ENSMUSG00000082846	0	0	0	1	0	0
ENSMUSG00000082847	0	0	0	0	0	0
ENSMUSG00000070438	0	0	0	0	0	0
ENSMUSG00000082845	0	0	0	0	0	0
ENSMUSG00000082842	0	0	0	0	0	0
ENSMUSG00000082843	0	0	0	0	0	0
ENSMUSG00000082840	2	1	3	2	0	2
ENSMUSG00000082841	0	0	0	0	0	2
ENSMUSG00000100029	0	0	0	0	0	0
ENSMUSG00000066359	0	0	0	0	0	0
ENSMUSG00000054087	0	0	0	0	0	0
ENSMUSG00000082849	0	0	0	0	0	0
ENSMUSG00000053146	0	0	0	0	0	0
ENSMUSG00000105088	0	0	0	0	0	0
ENSMUSG00000105089	0	0	0	0	0	0
ENSMUSG00000105086	22	9	4	8	2	2
ENSMUSG00000105087	3	9	3	32	24	29
ENSMUSG00000105082	0	8	12	6	7	0
ENSMUSG00000109856	0	0	0	0	0	0
ENSMUSG00000109855	0	0	0	0	0	0
ENSMUSG00000109853	0	0	0	0	0	0
ENSMUSG00000109851	0	0	0	0	0	0
ENSMUSG00000109858	0	0	0	0	0	0
ENSMUSG00000025789	18	0	9	6	4	6
ENSMUSG00000025783	0	0	0	2	0	0
ENSMUSG00000104681	0	0	0	0	0	0
ENSMUSG00000108103	0	0	0	0	0	0
ENSMUSG00000108100	0	0	0	0	0	0
ENSMUSG00000104685	2	0	0	0	0	0
ENSMUSG00000104684	0	5	2	3	0	3
ENSMUSG00000108104	0	0	0	0	0	0
ENSMUSG00000108105	0	1	0	0	0	0
ENSMUSG00000104688	0	0	0	0	0	0
ENSMUSG00000062783	0	14	29	9	12	12
ENSMUSG00000111612	2	0	0	0	0	0
ENSMUSG00000055933	0	0	0	0	0	0
ENSMUSG00000111616	0	0	0	0	0	0
ENSMUSG00000002930	0	0	0	0	0	0
ENSMUSG00000026787	2	0	2	0	0	0
ENSMUSG00000026786	242	276	332	36	102	123
ENSMUSG00000025876	47	52	55	35	57	62
ENSMUSG00000025877	60	110	142	20	44	67
ENSMUSG00000104352	0	0	0	0	0	0
ENSMUSG00000104351	12	9	12	9	6	15
ENSMUSG00000104350	5	7	18	3	12	0

ENSMUSG00000104356	0	0	0	0	0	0
ENSMUSG00000104355	0	0	0	0	0	0
ENSMUSG00000107714	0	0	0	0	0	0
ENSMUSG00000027788	0	0	0	0	0	0
ENSMUSG00000095003	0	0	0	0	0	0
ENSMUSG00000111395	0	0	0	2	2	0
ENSMUSG00000053054	0	0	0	0	0	0
ENSMUSG00000027431	2	0	0	0	0	0
ENSMUSG00000027434	0	0	0	0	0	0
ENSMUSG00000046699	293	210	388	133	101	135
ENSMUSG00000046697	0	0	0	0	0	0
ENSMUSG00000045417	0	0	0	0	0	0
ENSMUSG00000095628	0	0	0	0	0	0
ENSMUSG00000095626	0	0	0	0	0	0
ENSMUSG00000095627	0	0	0	0	0	1
ENSMUSG00000095624	0	0	0	0	0	0
ENSMUSG00000095625	0	0	0	0	0	0
ENSMUSG00000095620	0	0	0	0	0	0
ENSMUSG00000095621	0	0	0	0	0	0
ENSMUSG00000098395	0	0	2	0	0	0
ENSMUSG00000098394	4	4	1	1	7	3
ENSMUSG00000098391	0	0	0	0	0	0
ENSMUSG00000098390	0	0	0	0	0	0
ENSMUSG00000098393	0	0	0	0	0	0
ENSMUSG00000098392	0	1	2	2	1	1
ENSMUSG00000055193	0	0	0	0	0	0
ENSMUSG00000080558	0	2	0	0	0	0
ENSMUSG00000080555	0	0	0	0	0	0
ENSMUSG00000113216	26	29	47	55	20	24
ENSMUSG00000098204	0	0	0	0	0	0
ENSMUSG00000052012	0	0	0	0	0	0
ENSMUSG00000052013	81	89	103	34	38	48
ENSMUSG00000052014	0	0	0	0	0	0
ENSMUSG00000096965	4	0	14	0	0	0
ENSMUSG00000088743	0	0	0	0	0	0
ENSMUSG00000093708	0	0	0	0	0	0
ENSMUSG00000093703	0	0	0	0	0	0
ENSMUSG00000093702	0	0	0	0	0	0
ENSMUSG00000096747	1	0	0	0	0	0
ENSMUSG00000093707	0	0	0	0	0	0
ENSMUSG00000093705	0	0	0	0	0	0
ENSMUSG00000021940	0	0	0	0	0	0
ENSMUSG00000031444	0	0	10	8	0	4
ENSMUSG00000047129	7	24	3	21	22	17
ENSMUSG00000031449	0	0	0	0	0	0
ENSMUSG00000077767	0	0	0	0	0	0
ENSMUSG00000077761	0	0	0	0	0	0
ENSMUSG00000037843	0	0	0	4	0	6
ENSMUSG00000037846	28	26	28	0	10	12
ENSMUSG00000077769	0	0	0	0	0	0
ENSMUSG00000077768	0	0	0	0	0	0
ENSMUSG00000055540	0	0	0	5	6	10
ENSMUSG00000033350	112	88	112	65	82	87
ENSMUSG00000031163	0	0	0	0	0	0
ENSMUSG00000094752	0	0	0	0	0	0
ENSMUSG00000111295	0	0	0	0	0	0
ENSMUSG00000073860	0	0	0	0	0	0
ENSMUSG00000039628	0	0	0	0	0	0

ENSMUSG00000073862	0	0	0	0	0	0
ENSMUSG00000094978	0	0	0	0	0	0
ENSMUSG00000040693	0	0	0	0	0	0
ENSMUSG00000103960	0	0	0	0	0	0
ENSMUSG00000071424	5	3	2	4	0	3
ENSMUSG00000071428	0	0	0	0	0	0
ENSMUSG00000106378	0	0	0	0	0	0
ENSMUSG00000021441	0	0	0	2	0	0
ENSMUSG00000082303	0	0	0	0	0	0
ENSMUSG00000030713	0	0	0	2	4	7
ENSMUSG00000082307	0	0	0	0	0	0
ENSMUSG00000082306	0	0	0	0	0	0
ENSMUSG00000099429	0	0	0	0	0	0
ENSMUSG00000048329	14	33	22	21	20	15
ENSMUSG00000094972	0	0	0	0	0	0
ENSMUSG00000099424	0	0	0	0	0	0
ENSMUSG00000099425	0	0	0	0	0	0
ENSMUSG00000099426	0	0	0	0	0	0
ENSMUSG00000099427	0	0	0	0	0	0
ENSMUSG00000043924	14	3	10	4	6	0
ENSMUSG00000043925	8	0	3	0	7	0
ENSMUSG00000100137	0	0	0	0	0	0
ENSMUSG00000100136	0	0	0	0	0	0
ENSMUSG00000072731	10	4	0	0	3	7
ENSMUSG00000100134	0	0	1	0	0	0
ENSMUSG00000070777	0	0	0	0	0	0
ENSMUSG00000103964	0	5	0	0	0	4
ENSMUSG00000070778	0	0	0	0	0	0
ENSMUSG00000037159	0	0	0	0	0	0
ENSMUSG00000064084	0	0	0	1	0	0
ENSMUSG00000037157	17	0	5	6	4	14
ENSMUSG00000084467	0	0	0	0	0	0
ENSMUSG00000106114	0	0	0	0	0	0
ENSMUSG00000084463	0	0	0	0	0	0
ENSMUSG00000084460	0	0	0	0	0	0
ENSMUSG00000084461	0	0	2	0	0	0
ENSMUSG00000044249	0	0	0	0	0	0
ENSMUSG00000084468	0	0	0	0	0	0
ENSMUSG00000084469	0	0	0	0	0	0
ENSMUSG00000100689	0	0	0	1	0	0
ENSMUSG00000100684	0	0	0	0	0	0
ENSMUSG00000100687	0	0	0	0	0	2
ENSMUSG00000100681	0	0	0	0	0	0
ENSMUSG00000100683	0	0	0	0	0	0
ENSMUSG00000100682	0	0	0	0	0	0
ENSMUSG00000087845	0	0	0	0	0	0
ENSMUSG00000087847	0	0	0	0	0	0
ENSMUSG00000087846	0	0	0	0	0	0
ENSMUSG00000087841	0	0	0	0	0	0
ENSMUSG00000087843	0	0	0	0	0	0
ENSMUSG00000087842	0	0	0	0	0	0
ENSMUSG00000087848	0	0	0	0	0	0
ENSMUSG00000095191	0	0	0	1	0	0
ENSMUSG00000051793	0	0	0	0	0	0
ENSMUSG00000083162	0	0	0	0	0	0
ENSMUSG00000083163	0	0	0	0	0	0
ENSMUSG00000107480	2	0	0	0	0	0
ENSMUSG00000084793	0	0	0	0	0	0

ENSMUSG00000084795	0	0	0	0	0	0
ENSMUSG00000107484	0	0	0	0	0	0
ENSMUSG00000083165	3	1	5	0	2	4
ENSMUSG00000084798	11	5	5	1	0	7
ENSMUSG00000107488	0	0	0	3	0	0
ENSMUSG00000062905	0	4	0	0	0	2
ENSMUSG00000095194	0	0	0	0	0	0
ENSMUSG00000033436	572	457	586	284	316	429
ENSMUSG00000057735	0	0	0	0	0	0
ENSMUSG00000036401	0	0	0	0	0	0
ENSMUSG00000065029	0	0	0	0	0	0
ENSMUSG00000065022	1	0	1	0	0	0
ENSMUSG00000093434	0	0	0	0	0	0
ENSMUSG00000078680	0	0	0	0	0	0
ENSMUSG00000078683	0	0	0	0	0	0
ENSMUSG00000078685	0	0	0	0	0	0
ENSMUSG00000078687	0	0	0	0	0	0
ENSMUSG00000078686	0	0	0	0	0	0
ENSMUSG00000078689	0	0	0	0	0	0
ENSMUSG00000078688	0	0	0	0	0	0
ENSMUSG00000074109	0	6	0	0	0	0
ENSMUSG00000088565	0	0	0	0	0	0
ENSMUSG00000108237	0	0	0	0	0	0
ENSMUSG00000103568	0	0	0	0	0	0
ENSMUSG00000103564	0	0	0	0	2	0
ENSMUSG00000103566	0	0	0	0	0	0
ENSMUSG00000103561	0	0	0	0	0	0
ENSMUSG00000103563	0	0	0	0	0	0
ENSMUSG00000073457	0	0	0	0	0	0
ENSMUSG00000073458	0	0	0	0	0	0
ENSMUSG00000000701	0	0	0	0	0	0
ENSMUSG00000059218	0	0	0	0	0	0
ENSMUSG00000035179	0	0	2	3	0	0
ENSMUSG00000095198	0	0	0	0	0	0
ENSMUSG00000035177	0	0	0	0	0	0
ENSMUSG00000078355	0	0	0	0	0	0
ENSMUSG00000078354	0	0	0	0	0	0
ENSMUSG00000089443	0	0	0	0	0	0
ENSMUSG00000089442	0	0	0	0	0	0
ENSMUSG00000100912	0	0	0	0	0	0
ENSMUSG00000100919	0	0	0	0	0	0
ENSMUSG00000100918	0	0	0	0	0	0
ENSMUSG00000022949	5	0	5	0	0	6
ENSMUSG00000022948	111	77	90	94	91	126
ENSMUSG00000038524	180	135	176	93	113	102
ENSMUSG00000097476	9	11	17	6	14	26
ENSMUSG00000097473	0	0	0	0	0	0
ENSMUSG00000038523	0	15	5	0	4	0
ENSMUSG00000111514	4	0	14	0	0	8
ENSMUSG00000062272	0	1	3	0	2	3
ENSMUSG00000040258	0	2	4	4	4	6
ENSMUSG00000062275	0	0	0	0	0	0
ENSMUSG00000109241	2	2	1	0	2	1
ENSMUSG00000058513	0	0	0	0	0	0
ENSMUSG00000058515	0	0	0	0	2	0
ENSMUSG00000105248	0	0	0	0	0	0
ENSMUSG00000102219	0	0	0	0	0	0
ENSMUSG00000105242	0	0	0	0	0	0

ENSMUSG00000090690	0	0	0	0	0	0
ENSMUSG00000105240	0	0	0	0	0	0
ENSMUSG00000105241	0	0	0	0	0	0
ENSMUSG00000105246	0	0	0	0	0	0
ENSMUSG00000105247	40	41	65	24	26	33
ENSMUSG00000105244	3	9	10	5	8	3
ENSMUSG00000102213	0	0	0	0	0	0
ENSMUSG00000096708	0	0	0	0	0	0
ENSMUSG00000096709	0	0	1	1	0	1
ENSMUSG00000096704	0	0	0	0	0	0
ENSMUSG00000096705	0	2	0	0	0	0
ENSMUSG00000096706	0	0	0	0	0	0
ENSMUSG00000096707	0	0	0	0	0	0
ENSMUSG00000096700	0	0	0	0	0	0
ENSMUSG00000096703	0	0	0	0	0	0
ENSMUSG00000106742	0	0	0	0	0	0
ENSMUSG00000103516	0	0	0	0	0	0
ENSMUSG00000103517	0	0	0	0	0	0
ENSMUSG00000050742	5	6	7	4	0	2
ENSMUSG00000106746	0	0	0	0	3	0
ENSMUSG00000106747	0	0	0	0	4	3
ENSMUSG00000106745	0	0	0	0	0	0
ENSMUSG00000021379	54	26	74	13	15	43
ENSMUSG00000057770	0	0	0	0	0	0
ENSMUSG00000048101	0	0	0	0	0	0
ENSMUSG00000097409	0	0	0	0	0	0
ENSMUSG00000104118	257	249	173	132	133	160
ENSMUSG00000108478	43	34	31	11	9	10
ENSMUSG00000108473	5	6	1	1	3	3
ENSMUSG00000108474	0	0	0	0	0	0
ENSMUSG00000092697	0	0	0	0	0	0
ENSMUSG00000048108	24	0	27	0	20	23
ENSMUSG00000096601	0	0	0	1	0	0
ENSMUSG00000113989	0	0	0	0	0	0
ENSMUSG00000113982	0	0	0	0	0	0
ENSMUSG00000113985	0	0	0	0	0	0
ENSMUSG00000042821	50	29	49	19	18	26
ENSMUSG00000091163	0	7	6	3	0	0
ENSMUSG00000021997	4	0	0	0	0	0
ENSMUSG00000110059	0	0	0	0	0	0
ENSMUSG00000110864	0	0	0	0	3	0
ENSMUSG00000110865	0	0	0	0	0	0
ENSMUSG00000110866	0	0	0	0	3	0
ENSMUSG00000110862	0	0	4	0	0	2
ENSMUSG00000110863	13	14	7	11	9	9
ENSMUSG00000027230	30	31	43	10	28	21
ENSMUSG00000104805	0	0	0	0	0	0
ENSMUSG00000104804	0	0	0	0	0	0
ENSMUSG00000095776	0	0	0	1	0	0
ENSMUSG00000104800	0	0	0	0	0	0
ENSMUSG00000104808	3	0	0	0	0	3
ENSMUSG00000095428	0	0	0	0	0	0
ENSMUSG00000095424	0	0	0	0	0	0
ENSMUSG00000095425	0	0	0	0	0	0
ENSMUSG00000090417	0	0	0	0	0	0
ENSMUSG00000095422	0	1	0	0	0	0
ENSMUSG00000095423	0	0	0	0	0	0
ENSMUSG00000042525	0	0	0	0	0	0

ENSMUSG00000042523	383	415	449	37	81	490
ENSMUSG00000114446	0	4	0	0	0	0
ENSMUSG00000100122	0	0	0	0	0	0
ENSMUSG00000114447	0	0	0	1	0	2
ENSMUSG00000065924	0	0	0	0	0	0
ENSMUSG00000065922	0	0	0	0	0	0
ENSMUSG00000114444	0	0	0	4	0	0
ENSMUSG00000109377	0	0	0	0	0	0
ENSMUSG00000100960	2	1	0	0	0	0
ENSMUSG00000114443	0	0	0	0	0	0
ENSMUSG00000027908	0	0	0	0	0	0
ENSMUSG00000093142	0	0	0	0	0	0
ENSMUSG00000027902	0	0	0	0	0	0
ENSMUSG00000027359	0	0	0	0	0	0
ENSMUSG00000086240	1	3	1	1	3	1
ENSMUSG00000086241	8	0	0	8	5	3
ENSMUSG00000027358	55	45	61	26	12	30
ENSMUSG00000086244	0	0	0	0	0	0
ENSMUSG00000086246	0	0	0	0	0	0
ENSMUSG00000086249	5	7	5	4	7	1
ENSMUSG00000099359	0	0	0	0	0	0
ENSMUSG00000111806	0	0	3	0	5	9
ENSMUSG00000111801	0	0	0	0	0	3
ENSMUSG00000111802	0	2	0	0	0	0
ENSMUSG00000023263	0	0	0	0	0	0
ENSMUSG00000023267	26	29	11	53	59	90
ENSMUSG00000052658	18	31	20	20	28	28
ENSMUSG00000092842	0	0	0	0	0	0
ENSMUSG00000099892	0	0	0	0	0	0
ENSMUSG00000055385	0	0	0	0	0	0
ENSMUSG00000112575	0	0	0	0	0	0
ENSMUSG00000112576	12	1	2	2	6	10
ENSMUSG00000112570	0	0	0	0	0	0
ENSMUSG00000099897	0	0	0	0	0	0
ENSMUSG00000112572	0	0	0	0	0	0
ENSMUSG00000112573	0	0	0	0	0	0
ENSMUSG00000029503	3	0	0	0	6	0
ENSMUSG00000039358	0	0	0	0	0	2
ENSMUSG00000112579	0	0	0	0	0	0
ENSMUSG00000106188	9	10	11	28	16	12
ENSMUSG00000112233	0	0	0	0	0	0
ENSMUSG00000106185	0	0	0	0	0	0
ENSMUSG00000112230	0	0	0	0	0	0
ENSMUSG00000112236	0	0	0	0	0	0
ENSMUSG00000112235	1	0	0	0	0	0
ENSMUSG00000046049	0	0	0	3	0	0
ENSMUSG00000047003	161	183	162	130	123	189
ENSMUSG00000047002	0	1	0	0	0	0
ENSMUSG00000071629	0	0	0	2	0	0
ENSMUSG00000003053	0	0	0	0	0	0
ENSMUSG00000017950	0	0	0	0	0	0
ENSMUSG00000024815	2	0	2	0	0	0
ENSMUSG00000106232	0	0	0	0	0	0
ENSMUSG00000106233	0	0	0	0	0	0
ENSMUSG00000106235	0	0	0	0	0	0
ENSMUSG00000106238	2	0	0	0	0	0
ENSMUSG00000097134	4	0	3	0	0	3
ENSMUSG00000097137	0	0	0	0	0	0

ENSMUSG00000097130	0	0	0	0	0	0
ENSMUSG00000071195	0	0	0	0	0	0
ENSMUSG00000110822	0	0	0	0	0	0
ENSMUSG00000110043	2	1	4	0	0	0
ENSMUSG00000110826	0	0	0	0	0	0
ENSMUSG00000054169	0	0	0	0	0	0
ENSMUSG00000098684	0	0	0	0	0	0
ENSMUSG00000054162	10	2	2	0	2	0
ENSMUSG00000109778	0	0	0	0	0	0
ENSMUSG00000054160	0	0	0	0	0	0
ENSMUSG00000054161	0	15	0	0	24	30
ENSMUSG00000110828	0	0	0	0	0	0
ENSMUSG00000109779	0	0	0	0	0	0
ENSMUSG00000105760	0	0	0	0	0	0
ENSMUSG00000105763	0	0	0	0	0	0
ENSMUSG00000109461	1	4	0	0	0	0
ENSMUSG00000105765	0	0	0	0	0	0
ENSMUSG00000109467	0	0	0	0	0	0
ENSMUSG00000105767	0	0	0	0	0	0
ENSMUSG00000109465	0	0	0	2	0	0
ENSMUSG00000105768	0	0	0	0	0	0
ENSMUSG00000109468	0	0	0	0	0	1
ENSMUSG00000109469	0	0	0	0	0	0
ENSMUSG00000104908	3	1	3	3	1	3
ENSMUSG00000036972	0	0	0	0	0	0
ENSMUSG00000105213	0	0	0	0	0	0
ENSMUSG00000109776	0	0	0	0	0	0
ENSMUSG00000090572	0	0	0	0	0	0
ENSMUSG00000109777	0	0	0	0	0	0
ENSMUSG00000108731	0	0	0	0	0	0
ENSMUSG00000108730	0	0	0	0	0	0
ENSMUSG00000108735	0	0	0	0	0	0
ENSMUSG00000109774	0	0	0	0	0	0
ENSMUSG00000108736	15	7	16	5	6	0
ENSMUSG00000108739	0	0	0	0	0	0
ENSMUSG00000109775	3	0	0	0	0	0
ENSMUSG00000083361	0	0	0	0	0	0
ENSMUSG00000083363	0	0	0	0	0	0
ENSMUSG00000083366	0	0	0	0	0	0
ENSMUSG00000083368	0	0	0	0	0	0
ENSMUSG00000083369	0	0	0	0	0	0
ENSMUSG00000098765	0	0	0	0	0	0
ENSMUSG00000111196	0	0	0	0	0	0
ENSMUSG00000104842	0	0	0	0	0	0
ENSMUSG00000104847	0	0	0	0	0	0
ENSMUSG00000104846	0	0	0	0	3	0
ENSMUSG00000085209	0	0	0	0	0	0
ENSMUSG00000104844	0	0	0	0	0	0
ENSMUSG00000085203	0	0	0	0	0	0
ENSMUSG00000093931	0	0	0	0	0	0
ENSMUSG00000026622	15	22	22	0	2	38
ENSMUSG00000111194	0	0	0	0	0	0
ENSMUSG00000093935	0	0	0	0	0	0
ENSMUSG00000093937	0	0	0	0	0	0
ENSMUSG00000079247	3	2	1	1	1	1
ENSMUSG00000079244	0	0	0	0	0	0
ENSMUSG00000104905	0	0	0	0	0	0
ENSMUSG00000078798	0	1	0	0	0	0

ENSMUSG00000080186	0	0	0	0	0	1
ENSMUSG00000080636	0	0	0	0	0	0
ENSMUSG00000111192	0	0	0	0	0	0
ENSMUSG00000041707	60	51	134	31	13	28
ENSMUSG00000045337	0	0	0	0	0	0
ENSMUSG00000083987	0	0	0	0	0	0
ENSMUSG00000045331	0	0	0	0	0	0
ENSMUSG00000045330	0	0	0	0	0	0
ENSMUSG00000045333	243	208	330	71	56	94
ENSMUSG00000083983	0	0	0	0	0	0
ENSMUSG00000045339	0	0	0	0	0	0
ENSMUSG00000111191	0	0	0	0	0	0
ENSMUSG00000027577	0	2	4	3	0	0
ENSMUSG00000078118	0	0	0	0	0	0
ENSMUSG00000103486	10	0	13	1	10	11
ENSMUSG00000027570	25	16	19	9	12	20
ENSMUSG00000074472	0	0	0	0	0	0
ENSMUSG00000074473	0	0	0	2	0	0
ENSMUSG00000027579	20	10	23	48	17	23
ENSMUSG00000078116	0	0	0	0	0	0
ENSMUSG00000104901	0	0	0	2	3	2
ENSMUSG00000090351	0	0	0	0	0	0
ENSMUSG00000081279	0	0	0	0	0	0
ENSMUSG00000090353	0	0	0	0	0	0
ENSMUSG00000090352	2	4	6	0	0	0
ENSMUSG00000090355	0	0	0	0	0	0
ENSMUSG00000090356	0	0	0	0	1	0
ENSMUSG00000081271	0	0	0	0	0	0
ENSMUSG00000081273	0	0	0	0	0	0
ENSMUSG00000081275	0	0	0	0	0	0
ENSMUSG00000081276	0	0	6	0	0	2
ENSMUSG00000098583	0	0	0	0	0	0
ENSMUSG00000042641	6	5	0	3	10	3
ENSMUSG00000099988	0	0	0	0	0	0
ENSMUSG00000099986	0	0	0	0	0	0
ENSMUSG00000099985	0	0	0	0	0	0
ENSMUSG00000099983	0	0	0	0	0	0
ENSMUSG00000099982	0	0	0	0	0	0
ENSMUSG00000099981	0	0	0	0	0	0
ENSMUSG00000046150	5	0	0	0	0	0
ENSMUSG00000029682	0	0	0	0	0	0
ENSMUSG00000088260	0	0	0	0	0	0
ENSMUSG00000046159	46	45	37	52	19	76
ENSMUSG00000084989	4	8	0	6	6	8
ENSMUSG00000084986	0	0	0	0	0	0
ENSMUSG00000084981	0	0	0	0	0	0
ENSMUSG00000084980	0	0	0	0	0	0
ENSMUSG00000084983	0	0	0	0	0	0
ENSMUSG00000084982	0	0	0	0	0	0
ENSMUSG00000048424	0	0	0	0	0	0
ENSMUSG00000048425	0	0	0	0	0	0
ENSMUSG00000098636	0	0	0	0	0	0
ENSMUSG00000098634	1	0	3	4	4	5
ENSMUSG00000098635	0	0	1	1	0	1
ENSMUSG00000098630	0	0	0	0	0	0
ENSMUSG00000098631	0	0	0	0	0	0
ENSMUSG00000086726	0	2	0	0	0	0
ENSMUSG00000043060	0	0	2	0	0	0

ENSMUSG00000043066	0	0	0	0	0	0
ENSMUSG00000086721	8	0	4	4	0	0
ENSMUSG00000078800	0	0	0	0	0	0
ENSMUSG00000043068	42	65	41	48	42	28
ENSMUSG00000078808	0	0	0	0	0	0
ENSMUSG00000007034	0	0	0	0	0	0
ENSMUSG00000077351	0	0	0	0	0	0
ENSMUSG00000077356	0	0	0	0	0	0
ENSMUSG00000080065	0	0	0	0	2	0
ENSMUSG00000099929	0	0	0	0	0	0
ENSMUSG00000033256	74	72	85	60	66	88
ENSMUSG00000033255	0	0	5	0	2	0
ENSMUSG00000076407	0	0	0	0	0	0
ENSMUSG00000044597	0	0	0	0	0	0
ENSMUSG00000113189	0	0	0	0	0	0
ENSMUSG00000067951	0	0	0	0	0	0
ENSMUSG00000082119	0	0	0	0	0	0
ENSMUSG00000046811	8	4	6	0	0	2
ENSMUSG00000043681	0	0	0	0	0	0
ENSMUSG00000030509	515	648	535	543	580	651
ENSMUSG00000030507	0	0	0	0	0	0
ENSMUSG00000082113	0	1	0	0	0	0
ENSMUSG00000082115	0	0	0	0	0	0
ENSMUSG00000030500	0	0	0	0	0	2
ENSMUSG00000101453	0	0	0	0	0	0
ENSMUSG00000101451	2	2	4	3	4	2
ENSMUSG00000101457	0	0	0	0	0	0
ENSMUSG00000101456	0	0	0	0	0	0
ENSMUSG00000101455	0	0	0	0	0	0
ENSMUSG00000101454	0	0	0	0	0	0
ENSMUSG00000101459	0	0	0	0	0	0
ENSMUSG00000046913	0	0	0	2	0	0
ENSMUSG00000107859	0	0	0	0	0	4
ENSMUSG00000107855	0	0	0	0	0	0
ENSMUSG00000107854	0	0	0	0	0	0
ENSMUSG00000107857	0	0	0	0	0	0
ENSMUSG00000107856	0	0	0	0	0	0
ENSMUSG00000107851	0	2	0	0	0	0
ENSMUSG00000107850	0	0	0	0	0	0
ENSMUSG00000107852	0	0	0	0	0	0
ENSMUSG00000103349	5	0	5	0	6	8
ENSMUSG00000103347	0	0	0	0	0	0
ENSMUSG00000103346	3	2	0	0	0	0
ENSMUSG00000103340	0	0	0	0	0	0
ENSMUSG00000099216	0	0	0	0	0	0
ENSMUSG00000099215	0	0	0	0	0	0
ENSMUSG00000099214	0	0	1	0	0	0
ENSMUSG00000099213	0	1	0	0	1	1
ENSMUSG00000099218	0	0	0	0	0	0
ENSMUSG00000089263	0	0	0	0	0	0
ENSMUSG00000083646	3	1	1	0	2	1
ENSMUSG00000083644	0	0	0	0	0	0
ENSMUSG00000089267	2	1	2	5	1	2
ENSMUSG00000084610	0	0	0	0	0	0
ENSMUSG00000083641	0	0	0	0	0	0
ENSMUSG00000083640	0	0	0	0	0	0
ENSMUSG00000084619	0	0	0	0	0	0
ENSMUSG00000083648	0	0	0	0	0	0

ENSMUSG00000041681	0	0	0	0	0	0
ENSMUSG00000032611	23	26	18	27	24	29
ENSMUSG00000102438	0	0	0	0	0	0
ENSMUSG00000102437	15	12	5	0	7	0
ENSMUSG00000102430	0	0	0	0	0	0
ENSMUSG00000102432	0	0	0	0	0	0
ENSMUSG00000041359	0	0	0	0	0	0
ENSMUSG00000041358	0	0	0	0	0	0
ENSMUSG00000100499	0	0	0	0	0	0
ENSMUSG00000100494	0	0	0	0	0	0
ENSMUSG00000100495	0	0	0	0	0	0
ENSMUSG00000100496	0	0	0	0	0	0
ENSMUSG00000100497	0	0	0	0	0	2
ENSMUSG00000100490	4	0	0	4	0	2
ENSMUSG00000100491	0	0	0	0	0	0
ENSMUSG00000100492	0	0	0	0	0	0
ENSMUSG00000051056	0	0	0	2	0	1
ENSMUSG00000085565	2	0	0	0	0	0
ENSMUSG00000064549	0	0	0	0	0	0
ENSMUSG00000085561	0	0	0	0	0	0
ENSMUSG00000085562	0	0	0	0	0	0
ENSMUSG00000081187	25	28	35	23	38	53
ENSMUSG00000064541	0	0	0	0	0	0
ENSMUSG00000064547	0	0	0	0	0	0
ENSMUSG00000085569	10	0	5	0	0	0
ENSMUSG00000064545	1	0	0	0	0	0
ENSMUSG00000107148	22	38	34	25	36	30
ENSMUSG00000107141	0	0	0	0	0	0
ENSMUSG00000107144	0	0	0	0	0	0
ENSMUSG00000079764	13	11	7	8	7	6
ENSMUSG00000075465	0	0	0	0	0	0
ENSMUSG00000023066	610	763	651	600	654	780
ENSMUSG00000065497	0	0	1	1	0	0
ENSMUSG00000065493	1	0	1	1	0	0
ENSMUSG00000065491	0	0	0	0	0	0
ENSMUSG00000058897	26	31	32	64	34	55
ENSMUSG00000065498	1	0	0	0	0	0
ENSMUSG00000065499	0	0	0	0	0	0
ENSMUSG00000069132	0	3	0	0	0	0
ENSMUSG00000074731	0	0	0	0	0	2
ENSMUSG00000074735	31	33	22	18	19	25
ENSMUSG00000074737	3	0	0	0	0	0
ENSMUSG00000074736	21	16	9	0	0	3
ENSMUSG00000074739	0	0	0	0	0	0
ENSMUSG00000074738	76	44	76	60	50	58
ENSMUSG00000024593	235	120	255	24	53	55
ENSMUSG00000099824	0	0	0	0	0	0
ENSMUSG00000088611	0	0	0	0	0	0
ENSMUSG00000058281	0	2	0	0	0	3
ENSMUSG00000088614	0	0	0	0	0	0
ENSMUSG00000088617	0	0	0	0	0	0
ENSMUSG00000094337	0	0	0	0	0	0
ENSMUSG00000099828	0	0	0	0	0	0
ENSMUSG00000106434	1	0	0	0	0	0
ENSMUSG00000106437	0	0	0	0	0	0
ENSMUSG00000094330	0	1	0	0	0	0
ENSMUSG00000109078	0	0	0	0	0	0
ENSMUSG00000112939	0	0	0	0	0	0

ENSMUSG00000069796	0	0	0	0	0	0
ENSMUSG00000024043	220	216	189	140	198	268
ENSMUSG00000024041	0	0	0	0	0	0
ENSMUSG00000066141	0	0	0	0	0	0
ENSMUSG00000101526	0	0	0	0	0	0
ENSMUSG00000052616	5	3	5	0	3	0
ENSMUSG00000054360	0	0	0	0	0	0
ENSMUSG00000051729	0	0	0	2	0	0
ENSMUSG00000054362	0	0	0	0	0	0
ENSMUSG00000109661	1	0	0	0	0	0
ENSMUSG00000102392	0	0	0	0	0	0
ENSMUSG00000109664	9	0	5	9	8	7
ENSMUSG00000102394	0	0	0	0	0	0
ENSMUSG00000105169	0	0	0	0	0	0
ENSMUSG00000114655	0	1	0	0	0	0
ENSMUSG00000102399	0	0	0	2	0	0
ENSMUSG00000102398	3	6	0	2	4	0
ENSMUSG00000105164	1	0	0	0	0	0
ENSMUSG00000105162	0	0	0	0	0	0
ENSMUSG00000114226	0	0	0	0	0	0
ENSMUSG00000114227	0	0	0	0	0	0
ENSMUSG00000114224	0	0	0	0	0	0
ENSMUSG00000114225	0	0	0	0	0	0
ENSMUSG00000114222	0	0	0	0	0	0
ENSMUSG00000114223	0	0	0	0	0	0
ENSMUSG00000114221	0	0	0	0	0	0
ENSMUSG00000114228	2	1	1	1	1	1
ENSMUSG00000051727	8	8	0	0	0	0
ENSMUSG00000008028	0	0	0	0	0	2
ENSMUSG00000112970	0	0	0	1	0	1
ENSMUSG00000097822	0	1	2	1	0	1
ENSMUSG00000112972	2	4	1	6	4	2
ENSMUSG00000112974	0	0	0	0	0	0
ENSMUSG00000112975	1	0	0	0	0	2
ENSMUSG00000112976	0	1	0	0	0	0
ENSMUSG00000108538	0	0	0	0	0	0
ENSMUSG00000108537	0	0	0	0	0	0
ENSMUSG00000108535	2	0	0	0	0	0
ENSMUSG00000108534	0	0	0	0	0	0
ENSMUSG00000108532	0	0	0	0	0	0
ENSMUSG00000112936	0	0	0	0	0	0
ENSMUSG00000067231	0	0	0	0	0	0
ENSMUSG00000112788	1	5	1	1	2	3
ENSMUSG00000102806	0	0	0	0	0	0
ENSMUSG00000085634	0	0	0	0	0	0
ENSMUSG00000109071	0	0	0	0	0	0
ENSMUSG00000050281	0	0	0	0	0	0
ENSMUSG00000114320	0	0	0	0	0	0
ENSMUSG00000019909	0	0	0	0	0	0
ENSMUSG00000096573	2	2	7	2	0	0
ENSMUSG00000073085	0	0	0	0	0	0
ENSMUSG00000094452	0	0	0	0	0	0
ENSMUSG00000112785	0	0	0	0	0	0
ENSMUSG00000100830	0	0	0	0	0	0
ENSMUSG00000045027	0	0	0	0	0	0
ENSMUSG00000112538	0	0	0	0	0	0
ENSMUSG00000056853	1	0	0	0	0	0
ENSMUSG00000056854	2	0	0	0	0	0

ENSMUSG00000056856	0	3	4	0	0	0
ENSMUSG00000056858	0	0	0	0	0	0
ENSMUSG00000026358	71	61	64	231	245	200
ENSMUSG00000111304	0	0	0	0	0	0
ENSMUSG00000111305	0	0	0	0	0	0
ENSMUSG00000111302	0	0	0	0	0	0
ENSMUSG00000111303	0	0	0	0	0	0
ENSMUSG00000111251	0	0	0	0	0	0
ENSMUSG00000060275	43	48	24	101	65	102
ENSMUSG00000096560	0	0	0	0	0	0
ENSMUSG00000039313	4	0	8	2	0	3
ENSMUSG00000064958	0	0	0	0	0	0
ENSMUSG00000108272	0	0	0	0	0	0
ENSMUSG00000112535	0	0	0	0	3	0
ENSMUSG00000026976	0	0	0	0	0	0
ENSMUSG00000073926	2	0	0	0	0	0
ENSMUSG00000026979	96	149	170	55	50	63
ENSMUSG00000100834	0	0	0	0	0	0
ENSMUSG00000073929	0	0	0	0	0	0
ENSMUSG00000051716	6	3	6	2	0	3
ENSMUSG00000081072	0	0	0	0	0	0
ENSMUSG00000081073	0	0	0	0	0	0
ENSMUSG00000101327	0	0	0	0	0	0
ENSMUSG00000081079	0	0	0	0	0	0
ENSMUSG00000101326	0	0	0	0	0	0
ENSMUSG00000022076	0	0	0	0	0	0
ENSMUSG00000056270	0	0	0	0	0	0
ENSMUSG00000101601	0	0	0	0	0	0
ENSMUSG00000090629	0	0	0	0	0	1
ENSMUSG00000090623	0	0	0	0	0	0
ENSMUSG00000090624	0	3	0	0	0	0
ENSMUSG00000090627	0	0	0	0	0	0
ENSMUSG00000090626	100	144	103	130	121	73
ENSMUSG00000048620	1	3	4	27	6	4
ENSMUSG00000048626	0	0	0	0	0	0
ENSMUSG00000048628	0	0	0	0	3	0
ENSMUSG00000024292	0	0	0	12	0	17
ENSMUSG00000106271	1	0	2	0	3	4
ENSMUSG00000048191	22	21	33	17	16	44
ENSMUSG00000048197	0	0	0	0	0	0
ENSMUSG00000077110	0	0	0	0	0	0
ENSMUSG00000077111	0	0	0	0	0	0
ENSMUSG00000077113	0	0	1	0	0	0
ENSMUSG00000077119	0	0	0	0	0	0
ENSMUSG00000109135	0	0	0	0	0	0
ENSMUSG00000096567	0	0	0	0	0	0
ENSMUSG00000055775	0	0	5	0	0	0
ENSMUSG00000049576	31	39	30	41	31	32
ENSMUSG00000046561	97	76	73	32	32	51
ENSMUSG00000072541	0	0	0	0	0	0
ENSMUSG00000092666	0	0	0	0	0	0
ENSMUSG00000092664	0	0	0	0	0	0
ENSMUSG00000092668	0	0	0	0	0	0
ENSMUSG00000043865	0	0	0	2	0	0
ENSMUSG00000055194	2	2	0	0	0	0
ENSMUSG00000055197	0	0	0	0	0	0
ENSMUSG00000045912	0	0	4	3	0	5
ENSMUSG00000055198	0	0	0	0	0	0

ENSMUSG00000096964	5	9	4	3	0	4
ENSMUSG00000098901	0	0	0	0	0	0
ENSMUSG00000096966	0	0	0	0	0	0
ENSMUSG00000098903	0	0	0	0	0	0
ENSMUSG00000096960	10	0	6	0	7	5
ENSMUSG00000098909	0	0	0	0	0	0
ENSMUSG00000076395	0	0	0	0	0	0
ENSMUSG00000076396	1	0	0	0	0	0
ENSMUSG00000106377	0	1	1	2	0	0
ENSMUSG00000106373	0	0	0	0	0	0
ENSMUSG00000106372	0	0	0	0	0	0
ENSMUSG00000113218	0	0	0	0	0	0
ENSMUSG00000106370	0	0	0	0	0	0
ENSMUSG00000054827	0	0	0	0	0	0
ENSMUSG00000113217	0	0	0	0	0	0
ENSMUSG00000113214	0	0	0	0	0	0
ENSMUSG00000113215	0	0	0	0	0	0
ENSMUSG00000113213	0	0	0	0	0	0
ENSMUSG00000113210	0	7	0	0	0	0
ENSMUSG00000113211	0	0	0	0	0	0
ENSMUSG00000096442	0	0	0	0	0	0
ENSMUSG00000113069	1	1	1	3	1	0
ENSMUSG00000071019	0	0	0	0	0	0
ENSMUSG00000071015	0	0	0	0	0	0
ENSMUSG00000109347	0	0	0	0	0	0
ENSMUSG00000109346	0	0	0	0	0	0
ENSMUSG00000109345	1	0	0	0	0	0
ENSMUSG00000109344	0	0	0	0	0	0
ENSMUSG00000109343	0	0	0	0	0	0
ENSMUSG00000103119	2	2	4	2	6	4
ENSMUSG00000109348	0	0	0	0	0	0
ENSMUSG00000082757	0	0	0	0	0	0
ENSMUSG00000082754	0	0	0	0	0	0
ENSMUSG00000082752	0	0	0	0	0	0
ENSMUSG00000082750	0	0	0	2	0	0
ENSMUSG00000070942	228	244	261	100	78	191
ENSMUSG00000070943	0	0	0	0	0	0
ENSMUSG00000082758	0	2	0	0	0	0
ENSMUSG00000082759	0	0	0	0	0	1
ENSMUSG00000082014	0	0	0	0	0	0
ENSMUSG00000092083	5	1	0	0	0	0
ENSMUSG00000092080	0	0	0	0	0	0
ENSMUSG00000106593	0	0	0	0	0	0
ENSMUSG00000035852	10	8	13	4	3	10
ENSMUSG00000083085	0	0	0	0	0	0
ENSMUSG00000083084	0	0	0	0	0	0
ENSMUSG00000083081	0	3	0	0	0	0
ENSMUSG00000083083	9	22	16	17	10	14
ENSMUSG00000083089	0	0	0	0	0	0
ENSMUSG00000083088	0	0	0	0	0	0
ENSMUSG00000081965	6	2	2	2	4	8
ENSMUSG00000068149	0	0	0	0	0	0
ENSMUSG00000025090	0	0	0	0	0	0
ENSMUSG00000025091	0	0	0	0	0	0
ENSMUSG00000077416	1	3	4	0	0	0
ENSMUSG00000099357	0	0	0	0	0	0
ENSMUSG00000099354	5	3	5	7	6	7
ENSMUSG00000099355	0	0	0	0	0	0

ENSMUSG00000099352	0	0	0	0	0	0
ENSMUSG00000099353	0	0	0	0	0	0
ENSMUSG00000099350	0	0	0	0	0	0
ENSMUSG00000099351	0	0	0	0	0	0
ENSMUSG00000096445	0	0	0	0	0	0
ENSMUSG00000032808	2	0	0	3	0	0
ENSMUSG00000084369	0	0	0	0	0	0
ENSMUSG00000084367	0	0	0	0	0	0
ENSMUSG00000084366	0	0	0	0	0	0
ENSMUSG00000084361	0	0	0	0	0	0
ENSMUSG00000084363	0	0	0	0	0	0
ENSMUSG00000041119	79	99	193	119	77	123
ENSMUSG00000089811	0	0	0	0	0	0
ENSMUSG00000065395	0	1	1	0	0	0
ENSMUSG00000065397	1	0	1	1	1	1
ENSMUSG00000089819	0	0	0	0	0	0
ENSMUSG00000030981	14	13	11	7	14	4
ENSMUSG00000079169	0	0	0	0	0	0
ENSMUSG00000083750	0	0	0	4	0	7
ENSMUSG00000079164	82	51	73	26	26	36
ENSMUSG00000079163	0	2	0	0	0	0
ENSMUSG00000079162	0	0	0	0	0	10
ENSMUSG00000068226	6	8	5	8	13	9
ENSMUSG00000068227	23	33	37	20	14	9
ENSMUSG00000083755	0	0	0	0	0	0
ENSMUSG00000080069	0	0	0	0	0	0
ENSMUSG00000085014	0	0	0	0	0	0
ENSMUSG00000085017	0	0	0	0	0	0
ENSMUSG00000110001	0	0	0	0	0	0
ENSMUSG00000110007	0	0	0	2	0	0
ENSMUSG00000026012	71	99	95	150	146	189
ENSMUSG00000033860	0	0	0	0	0	0
ENSMUSG00000113063	0	0	0	0	0	0
ENSMUSG00000094565	0	0	0	0	0	0
ENSMUSG00000084521	0	0	0	0	0	0
ENSMUSG00000097171	2	0	0	0	0	0
ENSMUSG00000041805	0	0	0	0	0	0
ENSMUSG00000110009	0	0	0	0	0	0
ENSMUSG00000103113	0	0	0	0	0	0
ENSMUSG00000041809	82	71	84	23	36	26
ENSMUSG00000099826	0	0	0	0	0	0
ENSMUSG00000099827	0	0	0	0	0	0
ENSMUSG00000099820	14	3	5	4	0	10
ENSMUSG00000099821	0	0	0	0	0	0
ENSMUSG00000099822	0	0	0	0	0	0
ENSMUSG00000099823	0	0	0	0	0	0
ENSMUSG00000099829	0	0	0	0	0	0
ENSMUSG00000107449	0	0	0	0	0	0
ENSMUSG00000038044	16	15	14	5	15	27
ENSMUSG00000107350	0	7	7	0	0	0
ENSMUSG00000100056	0	0	0	0	0	0
ENSMUSG00000094848	0	0	0	0	0	0
ENSMUSG00000019905	0	0	0	0	0	2
ENSMUSG00000019900	0	0	0	0	2	0
ENSMUSG00000063350	0	0	0	0	0	0
ENSMUSG00000101075	0	0	0	0	0	0
ENSMUSG00000103938	0	4	0	0	2	0
ENSMUSG00000113668	0	0	0	0	0	0

ENSMUSG00000103936	0	0	0	0	0	0
ENSMUSG00000103934	0	0	0	0	0	0
ENSMUSG00000029885	0	0	0	0	0	0
ENSMUSG00000105499	0	4	0	0	0	0
ENSMUSG00000077256	0	0	0	0	0	0
ENSMUSG00000077255	0	0	0	0	0	0
ENSMUSG00000105498	0	1	0	0	0	0
ENSMUSG00000077253	0	0	0	0	0	0
ENSMUSG00000077252	0	0	0	0	0	0
ENSMUSG00000077251	0	0	0	0	0	0
ENSMUSG00000077259	0	0	0	0	0	0
ENSMUSG00000111874	0	3	0	0	0	0
ENSMUSG00000032807	0	0	0	0	0	0
ENSMUSG00000111876	0	0	0	0	0	0
ENSMUSG00000109134	0	0	0	0	3	0
ENSMUSG00000089815	0	0	0	0	4	0
ENSMUSG00000089816	0	0	0	0	0	0
ENSMUSG00000111870	0	0	0	0	2	0
ENSMUSG00000109338	0	0	0	0	0	4
ENSMUSG00000044863	0	0	0	0	0	0
ENSMUSG00000031576	0	0	0	0	0	0
ENSMUSG00000096881	0	0	0	0	0	0
ENSMUSG00000096880	0	0	1	2	1	0
ENSMUSG00000114435	0	0	0	0	0	0
ENSMUSG00000101398	0	6	2	10	3	6
ENSMUSG00000101399	0	0	0	0	0	0
ENSMUSG00000101390	0	0	0	0	0	0
ENSMUSG00000101391	0	0	0	0	0	0
ENSMUSG00000101393	0	0	0	0	0	0
ENSMUSG00000101394	0	0	0	0	0	0
ENSMUSG00000034891	0	0	0	0	0	0
ENSMUSG00000101396	0	0	0	0	0	0
ENSMUSG00000046213	0	0	0	0	0	0
ENSMUSG00000043230	10	14	54	4	8	12
ENSMUSG00000109471	0	0	0	0	0	0
ENSMUSG00000046215	0	0	0	0	0	2
ENSMUSG00000088070	0	0	0	0	0	0
ENSMUSG00000088073	0	0	0	0	0	0
ENSMUSG00000088077	0	0	0	0	0	0
ENSMUSG00000088076	0	0	0	0	0	0
ENSMUSG00000085843	10	7	9	0	0	5
ENSMUSG00000045094	144	160	268	138	215	198
ENSMUSG00000085841	0	0	0	0	0	0
ENSMUSG00000085840	0	0	0	3	0	2
ENSMUSG00000085844	0	0	0	0	0	0
ENSMUSG00000065645	0	0	0	0	0	0
ENSMUSG00000102568	0	0	0	0	0	0
ENSMUSG00000102569	3	0	0	0	0	0
ENSMUSG00000088389	0	0	0	0	0	0
ENSMUSG00000101641	5	5	3	7	7	12
ENSMUSG00000101647	0	0	0	0	0	0
ENSMUSG00000101648	2	0	2	3	5	0
ENSMUSG00000036139	0	0	0	0	1	0
ENSMUSG00000036136	16	7	5	0	3	6
ENSMUSG00000036131	35	29	50	7	11	9
ENSMUSG00000100511	0	0	0	0	0	0
ENSMUSG00000100513	0	0	0	0	0	0
ENSMUSG00000100512	0	3	4	0	0	8

ENSMUSG00000100515	0	0	0	0	3	0
ENSMUSG00000100517	0	0	0	0	0	0
ENSMUSG00000100519	0	0	0	0	0	0
ENSMUSG00000100518	0	0	0	0	0	0
ENSMUSG00000100587	0	0	0	0	0	0
ENSMUSG00000101395	0	0	0	0	0	0
ENSMUSG00000105474	0	0	0	0	0	0
ENSMUSG00000089380	0	0	0	0	0	0
ENSMUSG00000089381	1	0	0	0	0	0
ENSMUSG00000089382	0	0	0	0	0	0
ENSMUSG00000089383	0	0	0	0	0	0
ENSMUSG00000089384	0	0	0	0	0	0
ENSMUSG00000089385	0	0	0	0	0	0
ENSMUSG00000089387	0	0	0	0	0	0
ENSMUSG00000089033	0	0	0	0	0	0
ENSMUSG00000084006	0	0	3	2	0	0
ENSMUSG00000112383	0	0	5	0	0	0
ENSMUSG00000084001	0	0	0	0	0	0
ENSMUSG00000089034	0	0	0	0	0	0
ENSMUSG00000089035	2	0	0	2	2	0
ENSMUSG00000095851	0	0	0	0	0	0
ENSMUSG00000095850	0	0	0	0	0	0
ENSMUSG00000095853	0	1	1	0	0	1
ENSMUSG00000100581	0	0	0	0	0	0
ENSMUSG00000084009	0	0	0	0	0	0
ENSMUSG00000095857	0	1	0	0	1	2
ENSMUSG00000095856	1	1	2	0	1	1
ENSMUSG00000065519	2	0	2	0	2	0
ENSMUSG00000065512	0	0	0	0	0	1
ENSMUSG00000065511	0	0	0	0	0	0
ENSMUSG00000112382	0	0	0	0	0	0
ENSMUSG00000089036	0	0	0	0	0	0
ENSMUSG00000107045	2	3	2	0	1	3
ENSMUSG00000065858	0	0	0	0	0	0
ENSMUSG00000114065	0	0	0	0	0	0
ENSMUSG00000021977	0	0	0	0	0	0
ENSMUSG00000102609	2	0	0	0	0	0
ENSMUSG00000102608	0	0	0	0	0	0
ENSMUSG00000035448	8	0	7	0	5	0
ENSMUSG00000112328	0	0	0	0	0	0
ENSMUSG00000102603	0	0	0	0	2	0
ENSMUSG00000112324	0	0	0	0	0	9
ENSMUSG00000102601	0	0	0	0	0	0
ENSMUSG00000102600	2	5	4	0	0	7
ENSMUSG00000112321	3	7	1	4	4	5
ENSMUSG00000102606	0	1	1	1	0	0
ENSMUSG00000112322	0	0	0	0	0	0
ENSMUSG00000036106	20	62	46	13	39	61
ENSMUSG00000062564	2	0	0	0	0	0
ENSMUSG00000083413	0	0	0	0	0	0
ENSMUSG00000028626	70	39	67	5	2	6
ENSMUSG00000064455	0	0	0	0	0	0
ENSMUSG00000013523	0	0	4	0	6	0
ENSMUSG00000094813	0	3	0	0	1	0
ENSMUSG00000021700	0	0	0	7	0	4
ENSMUSG00000094811	0	0	0	0	0	0
ENSMUSG00000089037	0	0	0	0	0	0
ENSMUSG00000094814	0	0	0	0	0	0

ENSMUSG00000094818	0	0	0	0	0	0
ENSMUSG00000094819	0	0	0	0	0	0
ENSMUSG00000105554	4	0	0	0	0	0
ENSMUSG00000105555	0	0	0	0	0	1
ENSMUSG00000088808	0	0	0	0	0	0
ENSMUSG00000088809	0	0	0	0	0	0
ENSMUSG00000113054	0	0	0	0	4	0
ENSMUSG00000113055	1	4	5	0	0	2
ENSMUSG00000038236	0	0	0	4	0	6
ENSMUSG00000113057	0	0	0	0	0	0
ENSMUSG00000113050	0	0	0	0	0	0
ENSMUSG00000113051	0	0	0	0	0	0
ENSMUSG00000059874	2	1	0	0	0	0
ENSMUSG00000051182	2	0	2	2	0	0
ENSMUSG00000051180	0	0	0	0	3	0
ENSMUSG00000111567	0	0	0	0	0	0
ENSMUSG00000109583	0	0	0	0	0	0
ENSMUSG00000029288	0	0	0	0	0	0
ENSMUSG00000114583	2	0	0	0	0	0
ENSMUSG00000029286	4	2	0	0	0	0
ENSMUSG00000114580	0	0	0	0	0	0
ENSMUSG00000029283	85	69	98	47	66	114
ENSMUSG00000114586	0	0	0	0	0	2
ENSMUSG00000029281	0	0	0	0	0	0
ENSMUSG00000029280	0	0	0	0	0	0
ENSMUSG00000081194	0	4	0	5	0	0
ENSMUSG00000106608	1	1	1	0	1	0
ENSMUSG00000106606	0	0	0	0	0	0
ENSMUSG00000106604	0	0	0	0	0	0
ENSMUSG00000106602	0	0	0	0	0	0
ENSMUSG00000106601	0	0	0	0	0	0
ENSMUSG00000113587	0	0	0	0	0	0
ENSMUSG00000050854	0	0	2	0	0	0
ENSMUSG00000113582	0	0	0	0	0	0
ENSMUSG00000113583	0	0	0	0	0	0
ENSMUSG00000113581	35	20	57	4	0	4
ENSMUSG00000059668	0	0	0	0	0	0
ENSMUSG00000111690	0	0	0	0	0	0
ENSMUSG00000111691	0	0	0	0	0	0
ENSMUSG00000111694	0	0	0	0	0	0
ENSMUSG00000111695	0	0	0	2	0	0
ENSMUSG00000111696	1	2	2	5	5	3
ENSMUSG00000111697	0	0	0	0	0	0
ENSMUSG00000111698	0	0	0	0	0	1
ENSMUSG00000111699	0	0	0	0	0	0
ENSMUSG00000114659	0	0	0	0	2	0
ENSMUSG00000104609	0	0	0	0	0	0
ENSMUSG00000097540	0	0	0	0	0	0
ENSMUSG00000108188	0	0	0	0	0	0
ENSMUSG00000097545	0	0	0	0	0	0
ENSMUSG00000050587	47	48	44	43	50	77
ENSMUSG00000104601	0	0	0	0	0	0
ENSMUSG00000097548	3	0	0	0	0	0
ENSMUSG00000108180	8	6	12	6	3	2
ENSMUSG00000104602	0	0	3	0	0	0
ENSMUSG00000108186	0	0	0	0	0	1
ENSMUSG00000104604	0	0	0	0	0	0
ENSMUSG00000104607	0	0	0	0	0	0

ENSMUSG00000018822	3	8	9	28	21	18
ENSMUSG00000094733	0	0	0	0	0	0
ENSMUSG00000067001	0	0	0	0	0	0
ENSMUSG00000067006	0	0	0	0	1	0
ENSMUSG00000114059	1	1	0	1	1	1
ENSMUSG00000114058	0	0	0	0	0	0
ENSMUSG00000088523	0	0	0	0	0	0
ENSMUSG00000027702	4	10	10	6	8	13
ENSMUSG00000054666	1	0	1	3	1	0
ENSMUSG00000050321	0	0	0	0	0	0
ENSMUSG00000054662	1	3	1	1	0	3
ENSMUSG00000050328	0	0	0	0	0	0
ENSMUSG00000111751	0	0	0	0	0	0
ENSMUSG00000111750	0	0	0	0	0	0
ENSMUSG00000044453	2	0	7	0	4	4
ENSMUSG00000058132	2	0	0	0	0	0
ENSMUSG00000044457	0	0	0	0	0	0
ENSMUSG00000110779	2	0	1	0	1	3
ENSMUSG00000044454	0	0	0	0	0	0
ENSMUSG00000110776	0	0	0	0	0	0
ENSMUSG00000110770	0	0	0	0	0	0
ENSMUSG00000110771	0	0	0	0	0	0
ENSMUSG00000008590	0	0	0	0	0	0
ENSMUSG00000080266	0	0	0	0	0	0
ENSMUSG00000111759	0	0	0	0	3	0
ENSMUSG00000111758	14	7	8	6	17	17
ENSMUSG00000099855	0	0	0	0	0	0
ENSMUSG00000055838	0	0	2	0	0	0
ENSMUSG00000094735	0	0	0	0	0	0
ENSMUSG00000090129	0	0	0	0	0	1
ENSMUSG00000095339	0	0	0	0	0	0
ENSMUSG00000090122	5	6	14	6	2	3
ENSMUSG00000090120	3	0	0	4	4	0
ENSMUSG00000090121	0	0	0	0	0	0
ENSMUSG00000090126	0	0	0	0	0	0
ENSMUSG00000095331	0	0	0	0	0	0
ENSMUSG00000090125	19	12	25	2	2	4
ENSMUSG00000098318	31	39	70	9	18	18
ENSMUSG00000113879	0	0	0	0	0	0
ENSMUSG00000098315	0	0	0	0	0	0
ENSMUSG00000113875	0	0	0	0	0	0
ENSMUSG00000113873	0	0	0	0	0	0
ENSMUSG00000098313	0	0	0	0	0	0
ENSMUSG00000088383	0	0	0	0	0	0
ENSMUSG00000052364	39	44	55	26	19	32
ENSMUSG00000088380	0	0	0	0	0	0
ENSMUSG00000090383	0	0	0	0	0	0
ENSMUSG00000052368	4	3	4	4	2	2
ENSMUSG00000087570	0	0	0	0	0	1
ENSMUSG00000081607	0	0	4	0	2	1
ENSMUSG00000081606	0	0	0	0	0	0
ENSMUSG00000081601	0	1	0	0	0	0
ENSMUSG00000081600	36	58	43	35	54	48
ENSMUSG00000107059	0	0	0	0	0	0
ENSMUSG00000081609	0	0	0	0	0	0
ENSMUSG00000107058	2	6	4	0	5	4
ENSMUSG00000077095	0	0	0	0	1	0
ENSMUSG00000077096	0	0	0	0	0	0

ENSMUSG00000077090	0	0	0	0	0	0
ENSMUSG00000086951	0	0	0	0	0	0
ENSMUSG00000086952	0	0	0	0	0	0
ENSMUSG00000086955	0	0	0	0	0	0
ENSMUSG00000086957	0	0	0	0	0	0
ENSMUSG00000086959	0	0	0	0	0	0
ENSMUSG00000090788	0	0	0	0	0	0
ENSMUSG00000083195	0	5	2	3	8	5
ENSMUSG00000052099	0	1	0	5	2	12
ENSMUSG00000090785	6	4	0	5	5	0
ENSMUSG00000090782	0	0	0	0	0	0
ENSMUSG00000090781	0	0	0	0	0	0
ENSMUSG00000048015	0	0	0	0	0	0
ENSMUSG00000048013	0	0	0	0	0	0
ENSMUSG00000101646	0	0	0	0	0	0
ENSMUSG00000094175	1	0	1	0	0	1
ENSMUSG00000112045	0	0	0	0	0	0
ENSMUSG00000093781	1	0	0	0	0	0
ENSMUSG00000112040	0	0	0	0	0	0
ENSMUSG00000094170	0	0	0	0	0	0
ENSMUSG00000093785	0	0	0	0	0	0
ENSMUSG00000112043	1	0	1	3	1	1
ENSMUSG00000093788	0	0	0	0	0	0
ENSMUSG00000049946	0	0	0	0	0	0
ENSMUSG00000112049	1	1	0	1	1	1
ENSMUSG00000098573	0	0	0	0	0	0
ENSMUSG00000090872	0	8	20	0	0	0
ENSMUSG00000098572	0	0	0	0	0	0
ENSMUSG00000090874	0	0	0	0	0	0
ENSMUSG00000101649	0	0	0	0	0	0
ENSMUSG00000096876	0	0	0	0	0	0
ENSMUSG00000092437	0	0	0	0	0	0
ENSMUSG00000092435	0	0	0	0	0	0
ENSMUSG00000092439	0	0	0	0	0	0
ENSMUSG00000096691	0	0	0	4	0	0
ENSMUSG00000094673	0	0	0	0	0	0
ENSMUSG00000065322	0	0	0	0	0	0
ENSMUSG00000071937	0	5	0	0	0	0
ENSMUSG00000096697	0	0	0	0	0	0
ENSMUSG00000086375	0	0	0	0	0	0
ENSMUSG00000086377	5	5	6	4	5	6
ENSMUSG00000086370	296	267	272	225	170	228
ENSMUSG00000086371	0	0	0	0	0	0
ENSMUSG00000043439	4	6	6	22	12	17
ENSMUSG00000098855	0	0	0	0	0	0
ENSMUSG00000086379	0	4	0	9	7	5
ENSMUSG00000106265	12	26	14	16	26	26
ENSMUSG00000000142	143	183	141	121	121	141
ENSMUSG00000087772	0	0	0	0	0	0
ENSMUSG00000087777	0	0	0	0	0	0
ENSMUSG00000087775	0	0	0	0	0	0
ENSMUSG00000047390	0	0	0	0	0	0
ENSMUSG00000047394	4	14	9	9	15	16
ENSMUSG00000114420	0	0	0	0	0	0
ENSMUSG00000094136	1	7	1	6	2	9
ENSMUSG00000040165	4	0	10	0	7	4
ENSMUSG00000040164	8	0	6	0	3	5
ENSMUSG00000083470	0	0	0	0	0	0

ENSMUSG00000037224	104	83	190	37	48	46
ENSMUSG00000083475	0	0	0	0	0	0
ENSMUSG00000083478	0	0	0	0	0	0
ENSMUSG00000049052	0	0	0	0	0	0
ENSMUSG00000084496	0	0	0	0	0	2
ENSMUSG00000049057	0	1	0	0	0	0
ENSMUSG00000079559	43	22	56	25	36	36
ENSMUSG00000079553	34	47	53	23	23	21
ENSMUSG00000079554	0	0	0	0	0	0
ENSMUSG00000064006	0	0	0	1	0	0
ENSMUSG00000097480	0	0	0	0	0	0
ENSMUSG00000107408	0	0	0	0	0	0
ENSMUSG00000071207	0	0	0	0	0	0
ENSMUSG00000107400	0	0	0	0	0	0
ENSMUSG00000107401	0	0	0	0	0	0
ENSMUSG00000107407	0	0	0	0	0	2
ENSMUSG00000107405	0	0	0	0	0	0
ENSMUSG00000083294	0	0	3	2	0	0
ENSMUSG00000083296	0	0	0	0	0	0
ENSMUSG00000083297	0	0	0	0	0	0
ENSMUSG00000083290	0	0	0	0	0	0
ENSMUSG00000083291	0	0	0	0	0	0
ENSMUSG00000083292	0	0	0	0	0	0
ENSMUSG00000083293	0	0	0	0	0	0
ENSMUSG00000083298	0	0	0	0	0	0
ENSMUSG00000083299	0	0	0	0	0	0
ENSMUSG00000065758	0	0	0	0	0	0
ENSMUSG00000075587	2	0	0	0	1	0
ENSMUSG00000075586	0	0	0	0	0	0
ENSMUSG00000065751	0	0	0	0	0	0
ENSMUSG00000068587	10	16	51	6	20	12
ENSMUSG00000075588	15	41	9	38	34	27
ENSMUSG00000065757	1	1	1	1	1	0
ENSMUSG00000080929	1	1	1	0	0	0
ENSMUSG00000080927	0	0	0	0	0	0
ENSMUSG00000085734	0	0	0	0	0	0
ENSMUSG00000085737	0	0	0	0	0	0
ENSMUSG00000080924	0	0	0	0	0	0
ENSMUSG00000082173	0	0	0	0	0	0
ENSMUSG00000062278	0	0	0	0	0	0
ENSMUSG00000084599	0	0	0	0	0	0
ENSMUSG00000102730	0	0	0	0	0	0
ENSMUSG00000004366	0	0	0	0	0	0
ENSMUSG00000078601	0	0	0	0	0	0
ENSMUSG00000078600	0	0	0	0	0	0
ENSMUSG00000078605	0	0	0	0	0	0
ENSMUSG00000078607	22	21	16	39	55	79
ENSMUSG00000085193	0	0	0	0	0	0
ENSMUSG00000081501	3	3	0	2	5	3
ENSMUSG00000085198	0	0	0	2	0	0
ENSMUSG00000054431	0	0	0	0	0	0
ENSMUSG00000102734	0	0	0	0	0	0
ENSMUSG00000100999	0	0	0	0	0	0
ENSMUSG00000100998	0	0	0	0	1	0
ENSMUSG00000100995	0	0	0	0	0	0
ENSMUSG00000100994	0	0	0	0	0	0
ENSMUSG00000100996	0	0	0	0	0	0
ENSMUSG00000100991	0	0	0	0	0	0

ENSMUSG00000100990	0	0	0	0	0	0
ENSMUSG00000100993	0	0	0	0	0	0
ENSMUSG00000006270	0	0	0	0	0	0
ENSMUSG00000095858	0	0	0	0	0	0
ENSMUSG00000089030	1	5	4	10	8	10
ENSMUSG00000083981	0	0	0	0	0	0
ENSMUSG00000103878	0	0	0	0	0	0
ENSMUSG00000103877	0	0	0	0	0	0
ENSMUSG00000103876	0	0	0	0	0	0
ENSMUSG00000103875	0	0	1	0	2	0
ENSMUSG00000103874	0	0	0	0	0	0
ENSMUSG00000103873	0	0	0	0	0	0
ENSMUSG00000103872	0	0	0	0	0	0
ENSMUSG00000103871	0	0	0	0	0	0
ENSMUSG00000034227	0	4	0	0	0	0
ENSMUSG00000034226	0	0	0	0	0	0
ENSMUSG00000034224	0	0	0	0	0	0
ENSMUSG00000089038	0	0	0	0	0	2
ENSMUSG00000050967	18	12	23	0	0	0
ENSMUSG00000093382	0	0	0	0	0	0
ENSMUSG00000095852	0	1	0	1	0	0
ENSMUSG00000110030	8	10	9	0	0	6
ENSMUSG00000105551	0	0	0	0	0	0
ENSMUSG00000089997	0	0	0	0	0	0
ENSMUSG00000089996	0	0	2	3	0	0
ENSMUSG00000089990	0	0	0	0	0	0
ENSMUSG00000089992	0	0	0	0	0	0
ENSMUSG00000066512	0	0	0	0	0	0
ENSMUSG00000066513	0	0	0	0	0	0
ENSMUSG00000066516	0	0	0	0	0	0
ENSMUSG00000066515	0	0	0	0	0	0
ENSMUSG00000067649	0	0	0	0	0	0
ENSMUSG00000102839	0	0	0	0	0	0
ENSMUSG00000102834	0	0	4	0	0	0
ENSMUSG00000102835	0	0	0	0	0	0
ENSMUSG00000089690	0	0	0	0	0	0
ENSMUSG00000034913	0	0	0	0	0	0
ENSMUSG00000034912	52	75	30	89	74	91
ENSMUSG00000108968	0	0	0	0	0	0
ENSMUSG00000108969	0	0	0	0	0	0
ENSMUSG00000034919	0	0	0	0	0	0
ENSMUSG00000108965	0	0	0	0	0	0
ENSMUSG00000108966	0	0	0	0	0	0
ENSMUSG00000108967	0	4	0	0	0	0
ENSMUSG00000108960	0	0	0	0	0	0
ENSMUSG00000108961	0	0	0	0	0	0
ENSMUSG00000108962	0	0	0	0	0	0
ENSMUSG00000108963	0	0	0	0	0	0
ENSMUSG00000091255	0	0	0	0	0	0
ENSMUSG00000105826	5	0	10	2	6	3
ENSMUSG00000105823	0	0	0	0	0	0
ENSMUSG00000105821	0	0	0	0	0	0
ENSMUSG00000092317	0	0	0	0	0	0
ENSMUSG00000101566	0	0	0	0	0	0
ENSMUSG00000101565	0	0	0	0	0	0
ENSMUSG00000101564	0	0	0	0	0	0
ENSMUSG00000101563	3	0	0	0	0	0
ENSMUSG00000101562	0	0	0	0	0	0

ENSMUSG00000101560	0	0	0	0	0	0
ENSMUSG00000056426	0	0	0	0	0	0
ENSMUSG00000084107	0	0	0	0	0	0
ENSMUSG00000101569	0	0	0	0	0	0
ENSMUSG00000092520	0	0	0	0	0	0
ENSMUSG00000084102	0	0	0	0	0	0
ENSMUSG00000008318	95	113	173	85	104	139
ENSMUSG00000104889	0	0	0	0	0	0
ENSMUSG00000104887	0	0	0	0	0	0
ENSMUSG00000104883	0	0	0	0	0	0
ENSMUSG00000104881	0	3	0	1	0	0
ENSMUSG00000021919	0	0	0	0	0	0
ENSMUSG00000088228	0	0	0	0	0	0
ENSMUSG00000088229	0	0	0	0	3	0
ENSMUSG00000088220	0	2	1	0	0	0
ENSMUSG00000088222	0	0	0	0	0	0
ENSMUSG00000088223	0	0	0	0	0	0
ENSMUSG00000088225	0	0	0	0	0	0
ENSMUSG00000088227	0	0	0	0	0	0
ENSMUSG00000077588	0	0	0	0	0	0
ENSMUSG00000104759	0	2	0	0	0	0
ENSMUSG00000022755	0	0	2	0	0	0
ENSMUSG00000022756	61	29	30	79	92	114
ENSMUSG00000109512	0	0	0	0	0	0
ENSMUSG00000089175	0	0	0	0	0	0
ENSMUSG00000089174	0	0	0	0	0	0
ENSMUSG00000089171	0	0	0	0	0	0
ENSMUSG00000111885	0	0	0	0	0	0
ENSMUSG00000111884	0	0	0	0	0	0
ENSMUSG00000111881	0	0	0	0	0	0
ENSMUSG00000111880	3	3	0	0	0	0
ENSMUSG00000089179	2	0	0	0	0	0
ENSMUSG00000089178	0	0	0	0	0	0
ENSMUSG00000106100	0	0	0	0	0	0
ENSMUSG00000106101	0	0	0	0	0	0
ENSMUSG00000106104	0	0	0	0	0	0
ENSMUSG00000106109	3	0	0	0	0	0
ENSMUSG00000028186	2	0	2	0	0	2
ENSMUSG00000028185	0	0	0	0	0	0
ENSMUSG00000109514	0	0	0	0	0	0
ENSMUSG00000097897	0	0	0	0	0	0
ENSMUSG00000109515	0	0	0	0	0	0
ENSMUSG00000112282	0	0	0	0	0	0
ENSMUSG00000114510	0	0	0	0	0	0
ENSMUSG00000112280	268	236	252	246	182	320
ENSMUSG00000112286	0	0	0	0	0	0
ENSMUSG00000112284	0	0	0	0	0	0
ENSMUSG00000094374	3	3	5	2	2	2
ENSMUSG00000094379	0	0	0	0	0	0
ENSMUSG00000094378	0	0	0	0	0	0
ENSMUSG00000114513	0	0	0	0	0	0
ENSMUSG00000114514	0	0	0	0	0	0
ENSMUSG00000009596	0	0	0	0	0	0
ENSMUSG00000020660	9	5	2	4	8	11
ENSMUSG00000086748	0	0	0	0	0	0
ENSMUSG00000100879	0	0	0	0	0	0
ENSMUSG00000092231	0	0	0	0	0	0
ENSMUSG00000023403	0	0	0	0	0	0

ENSMUSG00000028453	137	102	166	75	96	90
ENSMUSG00000092237	0	0	0	0	0	0
ENSMUSG00000092239	0	0	0	0	0	0
ENSMUSG00000035638	3	0	0	0	0	0
ENSMUSG00000055301	0	5	3	0	3	3
ENSMUSG00000107282	0	0	0	0	0	0
ENSMUSG00000024757	2	2	0	0	0	0
ENSMUSG00000029586	0	0	0	0	0	4
ENSMUSG00000100871	0	0	0	0	0	0
ENSMUSG00000114932	1	0	0	1	0	0
ENSMUSG00000114930	0	0	0	0	0	0
ENSMUSG00000099459	0	0	0	0	0	0
ENSMUSG00000038009	0	0	8	0	2	0
ENSMUSG00000113628	0	0	0	0	0	0
ENSMUSG00000028950	8	2	8	0	8	8
ENSMUSG00000113620	0	0	0	0	0	0
ENSMUSG00000113626	94	117	113	49	47	66
ENSMUSG00000113624	0	0	0	0	0	0
ENSMUSG00000094997	0	0	0	0	0	0
ENSMUSG00000109171	0	4	3	0	0	0
ENSMUSG00000109172	0	1	0	0	0	0
ENSMUSG00000094994	0	0	0	1	0	0
ENSMUSG00000109174	0	0	0	0	0	0
ENSMUSG00000105459	0	0	0	0	0	0
ENSMUSG00000109177	0	0	0	0	0	0
ENSMUSG00000105457	77	123	76	63	58	73
ENSMUSG00000109179	92	93	125	74	70	112
ENSMUSG00000105455	0	0	0	0	0	0
ENSMUSG00000094999	0	0	0	0	0	0
ENSMUSG00000094998	0	0	0	0	0	0
ENSMUSG00000029320	0	0	0	0	0	0
ENSMUSG00000112325	0	0	0	0	0	0
ENSMUSG00000066257	0	0	0	0	0	0
ENSMUSG00000106819	1	0	1	3	0	0
ENSMUSG00000106817	0	0	0	0	0	0
ENSMUSG00000106814	0	0	0	0	0	0
ENSMUSG00000106812	0	0	0	0	0	0
ENSMUSG00000039146	0	0	0	0	0	0
ENSMUSG00000113785	0	0	0	0	0	0
ENSMUSG00000069554	3	1	2	4	3	3
ENSMUSG00000100301	3	4	1	0	5	7
ENSMUSG00000111715	0	0	0	0	0	0
ENSMUSG00000111717	0	0	0	0	0	0
ENSMUSG00000111711	0	0	0	0	0	0
ENSMUSG00000109791	0	0	0	0	0	0
ENSMUSG00000109792	0	0	0	0	0	0
ENSMUSG00000109798	0	0	0	0	0	0
ENSMUSG00000096264	0	0	1	0	0	0
ENSMUSG00000100307	0	0	0	0	0	0
ENSMUSG00000108642	0	0	0	0	0	0
ENSMUSG00000005357	4	0	16	0	0	2
ENSMUSG00000005355	0	0	0	0	0	0
ENSMUSG00000108228	47	55	30	15	19	28
ENSMUSG00000108645	0	0	0	0	0	0
ENSMUSG00000108225	0	0	0	0	0	0
ENSMUSG00000108224	0	0	0	0	0	0
ENSMUSG00000108223	0	0	0	0	0	0
ENSMUSG00000108222	0	0	0	0	0	0

ENSMUSG00000108221	0	0	0	0	0	0
ENSMUSG00000108220	0	0	3	3	3	3
ENSMUSG00000059146	33	41	34	20	43	44
ENSMUSG00000050641	0	0	0	0	0	0
ENSMUSG00000113780	31	30	22	21	25	30
ENSMUSG00000055874	0	0	0	0	0	0
ENSMUSG00000097992	0	0	0	0	0	0
ENSMUSG00000044390	20	12	25	26	16	19
ENSMUSG00000057439	0	0	0	0	0	0
ENSMUSG00000104410	0	0	0	0	0	0
ENSMUSG00000104413	0	0	0	0	0	0
ENSMUSG00000100911	0	0	0	0	0	0
ENSMUSG00000083908	0	0	0	0	0	0
ENSMUSG00000083907	22	29	45	41	39	53
ENSMUSG00000083900	4	0	4	2	1	5
ENSMUSG00000083901	0	0	0	0	0	0
ENSMUSG00000083903	0	0	0	0	0	0
ENSMUSG00000010021	2	5	14	10	3	12
ENSMUSG00000028039	0	7	4	0	3	3
ENSMUSG00000025936	15	100	12	77	79	127
ENSMUSG00000025931	174	178	224	117	128	195
ENSMUSG00000025932	105	85	60	89	94	128
ENSMUSG00000044641	128	105	133	66	68	87
ENSMUSG00000044646	179	120	170	75	83	89
ENSMUSG00000044647	4	8	6	23	16	34
ENSMUSG00000044649	0	0	0	0	0	0
ENSMUSG00000074968	33	17	19	0	2	8
ENSMUSG00000096604	0	0	0	0	0	0
ENSMUSG00000095108	0	0	0	0	0	0
ENSMUSG00000045515	6	1	0	11	0	2
ENSMUSG00000045514	7	3	2	0	3	0
ENSMUSG00000095103	0	0	0	0	0	0
ENSMUSG00000095102	0	0	0	0	0	0
ENSMUSG00000095101	2	3	1	1	1	1
ENSMUSG00000095100	0	0	0	0	0	0
ENSMUSG00000095107	0	0	0	0	0	0
ENSMUSG00000113819	0	2	3	1	6	3
ENSMUSG00000095105	14	7	26	6	5	0
ENSMUSG00000095104	0	0	0	0	0	0
ENSMUSG00000092898	0	0	0	0	0	0
ENSMUSG00000060685	0	0	0	0	0	0
ENSMUSG00000101893	0	0	0	0	0	0
ENSMUSG00000080765	0	0	0	0	0	0
ENSMUSG00000080764	0	0	0	0	0	0
ENSMUSG00000080767	0	0	0	0	0	0
ENSMUSG00000080766	0	0	0	0	0	0
ENSMUSG00000080760	0	4	0	5	6	4
ENSMUSG00000080762	0	0	0	0	0	0
ENSMUSG00000048933	0	0	0	0	0	0
ENSMUSG00000083753	0	0	0	0	0	0
ENSMUSG00000098509	4	4	4	8	2	17
ENSMUSG00000098504	0	0	0	0	0	0
ENSMUSG00000098505	0	0	0	0	2	0
ENSMUSG00000098506	15	13	12	6	14	16
ENSMUSG00000098507	0	0	0	0	0	0
ENSMUSG00000098501	0	0	0	0	0	0
ENSMUSG00000098502	0	0	0	0	0	0
ENSMUSG00000093621	0	0	0	0	0	0

ENSMUSG00000093622	4	0	5	0	5	0
ENSMUSG00000093623	0	0	0	0	0	0
ENSMUSG00000093625	0	0	0	0	0	0
ENSMUSG00000093626	0	0	0	0	0	0
ENSMUSG00000093627	0	0	0	0	0	0
ENSMUSG00000105559	0	0	0	0	0	0
ENSMUSG00000031329	0	0	0	0	0	0
ENSMUSG00000050397	0	2	0	0	0	0
ENSMUSG00000031325	1	0	0	0	0	0
ENSMUSG00000031327	327	310	287	215	225	239
ENSMUSG00000031326	0	0	0	0	0	0
ENSMUSG00000047606	0	0	0	0	0	0
ENSMUSG00000031323	0	0	0	2	0	0
ENSMUSG00000047604	65	57	42	42	65	79
ENSMUSG00000077685	0	0	0	0	0	0
ENSMUSG00000077687	0	1	0	0	0	0
ENSMUSG00000077680	0	0	0	0	0	0
ENSMUSG00000077681	0	0	0	0	1	0
ENSMUSG00000077682	0	0	0	0	0	0
ENSMUSG00000077683	0	0	0	0	0	0
ENSMUSG00000081414	0	2	1	4	0	0
ENSMUSG00000081415	0	1	0	0	2	0
ENSMUSG00000081417	0	0	0	0	0	0
ENSMUSG00000081410	0	0	0	0	0	0
ENSMUSG00000081412	0	0	0	0	0	0
ENSMUSG00000092576	0	0	0	0	0	0
ENSMUSG00000092575	0	0	0	0	0	0
ENSMUSG00000092574	11	21	11	0	0	0
ENSMUSG00000092573	0	0	0	0	0	0
ENSMUSG00000092572	0	0	0	0	0	0
ENSMUSG00000092571	3	0	0	0	4	3
ENSMUSG00000092570	0	0	0	0	0	0
ENSMUSG00000077535	0	0	0	0	0	0
ENSMUSG00000040557	58	19	40	20	25	41
ENSMUSG00000040554	0	0	0	3	0	0
ENSMUSG00000092578	0	0	0	0	0	0
ENSMUSG00000030050	0	0	0	0	0	0
ENSMUSG00000030051	189	184	154	133	136	138
ENSMUSG00000082267	0	0	0	0	0	0
ENSMUSG00000082262	2	0	0	0	1	0
ENSMUSG00000082263	0	0	0	0	0	0
ENSMUSG00000082261	0	0	0	0	0	0
ENSMUSG00000048206	0	0	0	0	0	0
ENSMUSG00000088506	0	0	0	0	0	0
ENSMUSG00000082268	0	0	0	0	0	0
ENSMUSG00000082269	0	0	0	0	0	0
ENSMUSG00000099299	0	0	0	0	0	0
ENSMUSG00000099298	0	0	0	0	0	0
ENSMUSG00000099295	1	1	1	1	1	1
ENSMUSG00000049109	13	9	11	8	6	4
ENSMUSG00000099293	0	0	0	0	0	0
ENSMUSG00000032566	0	0	0	0	0	0
ENSMUSG00000032564	3	6	3	10	5	8
ENSMUSG00000032561	4	0	4	8	2	4
ENSMUSG00000098676	1	1	1	1	1	1
ENSMUSG00000098677	1	0	0	0	0	0
ENSMUSG00000043605	0	3	0	0	0	0
ENSMUSG00000088800	0	0	0	0	0	0

ENSMUSG00000030584	11	14	8	2	4	4
ENSMUSG00000072477	0	0	0	0	0	0
ENSMUSG00000072473	0	0	0	0	0	0
ENSMUSG00000113056	0	0	0	0	0	0
ENSMUSG00000032691	19	55	56	15	19	16
ENSMUSG00000041608	0	0	0	0	0	0
ENSMUSG00000100769	0	0	0	0	0	0
ENSMUSG00000100762	0	0	0	0	0	0
ENSMUSG00000100760	2	0	0	0	0	0
ENSMUSG00000100761	0	0	0	0	0	0
ENSMUSG00000041605	5	0	0	0	0	0
ENSMUSG00000100767	0	0	0	0	0	0
ENSMUSG00000100765	0	0	0	0	0	0
ENSMUSG00000087922	0	0	0	0	0	0
ENSMUSG00000087923	0	0	0	0	0	0
ENSMUSG00000087921	0	0	0	0	0	0
ENSMUSG00000087929	0	0	0	0	0	0
ENSMUSG00000064631	0	0	0	0	0	0
ENSMUSG00000064630	0	0	0	0	0	0
ENSMUSG00000037053	0	0	0	0	0	0
ENSMUSG00000084696	0	2	2	3	0	2
ENSMUSG00000084690	0	0	0	0	0	0
ENSMUSG00000075170	0	0	0	0	0	0
ENSMUSG00000075171	0	0	0	0	0	0
ENSMUSG00000075172	0	0	0	0	0	0
ENSMUSG00000075174	0	0	0	0	0	0
ENSMUSG00000075175	0	0	0	0	0	0
ENSMUSG00000075176	0	0	0	0	0	0
ENSMUSG00000087995	0	0	0	0	0	1
ENSMUSG00000061376	0	0	0	0	0	0
ENSMUSG00000087994	0	0	0	0	0	0
ENSMUSG00000028314	0	0	0	0	0	0
ENSMUSG00000081882	0	0	0	0	0	0
ENSMUSG00000107233	0	0	0	0	0	0
ENSMUSG00000107232	0	0	0	0	0	0
ENSMUSG00000107234	10	8	2	2	0	2
ENSMUSG00000107237	0	0	0	0	0	0
ENSMUSG00000033774	0	0	0	0	0	0
ENSMUSG00000103143	23	36	24	40	19	25
ENSMUSG00000033770	0	0	0	0	3	0
ENSMUSG00000103141	0	0	0	0	0	0
ENSMUSG00000101963	4	0	1	1	1	3
ENSMUSG00000101962	0	0	0	0	0	0
ENSMUSG00000101961	0	0	0	0	0	0
ENSMUSG00000101960	0	0	0	0	0	0
ENSMUSG00000101967	0	0	0	0	0	0
ENSMUSG00000101966	0	0	0	0	0	0
ENSMUSG00000101965	0	0	0	0	0	0
ENSMUSG00000101969	9	8	8	0	8	8
ENSMUSG00000101968	4	17	9	3	8	5
ENSMUSG00000111566	1	0	0	0	0	0
ENSMUSG00000039106	0	0	0	0	0	0
ENSMUSG00000074224	3	0	0	0	0	0
ENSMUSG00000114589	0	0	0	0	0	0
ENSMUSG00000074220	162	148	130	70	91	130
ENSMUSG00000109586	0	0	0	0	0	0
ENSMUSG00000111562	0	0	0	0	0	0
ENSMUSG00000111563	0	0	0	0	0	0

ENSMUSG00000103448	0	0	0	0	0	0
ENSMUSG00000103449	0	0	0	0	0	0
ENSMUSG00000103440	2	2	2	0	0	0
ENSMUSG00000111561	0	0	0	0	0	0
ENSMUSG00000103446	0	0	0	0	0	0
ENSMUSG00000103444	0	0	0	0	0	0
ENSMUSG00000103445	5	7	2	2	0	6
ENSMUSG00000065274	0	0	0	0	0	0
ENSMUSG00000065275	0	0	0	0	0	0
ENSMUSG00000083117	0	0	0	0	0	0
ENSMUSG00000073797	0	0	0	0	0	0
ENSMUSG00000073794	0	0	0	0	0	0
ENSMUSG00000065279	0	2	0	0	0	2
ENSMUSG00000030092	0	0	0	0	0	0
ENSMUSG00000061022	0	0	0	0	0	0
ENSMUSG00000037086	0	0	0	0	0	0
ENSMUSG00000039518	6	2	13	5	6	5
ENSMUSG00000114587	0	0	0	0	0	0
ENSMUSG00000014542	5	16	0	0	14	8
ENSMUSG00000029282	0	0	0	0	0	0
ENSMUSG00000024510	0	0	0	0	0	0
ENSMUSG00000024512	0	0	0	0	0	0
ENSMUSG00000057047	0	0	0	0	0	0
ENSMUSG00000024517	0	0	0	0	0	0
ENSMUSG00000024518	0	0	0	0	0	0
ENSMUSG00000024519	0	0	0	0	0	0
ENSMUSG00000111569	2	0	0	0	0	0
ENSMUSG00000088740	0	0	0	0	0	0
ENSMUSG00000083695	0	2	2	0	2	6
ENSMUSG00000089560	0	0	0	0	0	0
ENSMUSG00000089561	0	0	0	0	0	0
ENSMUSG00000089562	0	0	0	0	0	0
ENSMUSG00000089563	0	0	0	0	0	0
ENSMUSG00000089564	0	0	0	0	0	0
ENSMUSG00000014543	0	3	0	0	0	0
ENSMUSG00000097338	0	0	0	0	0	0
ENSMUSG00000097339	0	0	0	0	0	5
ENSMUSG00000038641	3	0	0	0	0	0
ENSMUSG00000022821	0	0	0	0	0	6
ENSMUSG00000073245	0	0	0	0	0	0
ENSMUSG00000073247	0	0	0	0	0	0
ENSMUSG00000063842	0	0	0	0	0	0
ENSMUSG00000026241	0	0	0	0	0	0
ENSMUSG00000063846	0	0	0	0	0	0
ENSMUSG00000063844	0	0	0	2	0	0
ENSMUSG00000071178	5	5	3	5	4	4
ENSMUSG00000062132	0	0	0	0	0	0
ENSMUSG00000106989	0	0	0	0	0	0
ENSMUSG00000027894	93	96	84	126	75	87
ENSMUSG00000069718	0	0	0	0	0	0
ENSMUSG00000099935	0	0	0	1	0	0
ENSMUSG00000077941	0	0	0	0	0	0
ENSMUSG00000099932	0	0	0	0	0	0
ENSMUSG00000069712	2	1	1	1	1	1
ENSMUSG00000113584	0	0	0	0	0	0
ENSMUSG00000069717	0	0	0	0	0	0
ENSMUSG00000099933	0	0	0	0	0	0
ENSMUSG00000102318	0	0	0	0	0	0

ENSMUSG00000099930	1	1	2	0	1	3
ENSMUSG00000102311	0	0	0	0	0	0
ENSMUSG00000102310	0	0	0	0	0	0
ENSMUSG00000094226	5	5	2	7	5	5
ENSMUSG00000102315	0	0	0	0	0	0
ENSMUSG00000102314	0	0	0	0	0	0
ENSMUSG00000102317	0	0	0	0	0	0
ENSMUSG00000050853	0	0	0	2	0	1
ENSMUSG00000096069	0	0	0	0	0	0
ENSMUSG00000096068	0	0	0	0	0	0
ENSMUSG00000035964	0	0	16	0	0	0
ENSMUSG00000035963	0	0	0	0	0	0
ENSMUSG00000096061	0	0	0	0	0	1
ENSMUSG00000096067	0	0	0	0	0	0
ENSMUSG00000096066	0	0	0	0	0	0
ENSMUSG00000066720	0	0	0	0	0	0
ENSMUSG00000113882	0	0	0	2	0	3
ENSMUSG00000021032	2	4	0	0	3	8
ENSMUSG00000023882	48	130	38	77	29	94
ENSMUSG00000050179	0	0	0	0	0	0
ENSMUSG00000104256	0	0	0	0	0	0
ENSMUSG00000104257	0	0	0	0	0	0
ENSMUSG00000104254	0	0	0	0	0	0
ENSMUSG00000104255	0	0	0	0	0	0
ENSMUSG00000104251	0	0	0	0	0	0
ENSMUSG00000104258	0	0	0	0	0	0
ENSMUSG00000104259	0	0	0	0	0	0
ENSMUSG00000057182	238	279	225	200	197	251
ENSMUSG00000051455	0	6	7	7	5	10
ENSMUSG00000101153	0	0	4	0	2	0
ENSMUSG00000101150	0	0	0	0	0	0
ENSMUSG00000101151	0	0	0	0	0	0
ENSMUSG00000101157	0	0	0	0	0	0
ENSMUSG00000060765	0	0	0	0	0	0
ENSMUSG00000101155	0	0	0	0	0	0
ENSMUSG00000101158	0	0	0	0	0	0
ENSMUSG00000101159	0	0	0	0	0	0
ENSMUSG00000091595	0	0	0	0	0	0
ENSMUSG00000091043	0	0	0	0	0	0
ENSMUSG00000091040	0	1	1	0	1	0
ENSMUSG00000056586	0	0	0	0	0	0
ENSMUSG00000018589	0	0	0	0	0	0
ENSMUSG00000091045	0	0	0	0	0	0
ENSMUSG00000091048	0	0	0	0	0	0
ENSMUSG00000091049	6	5	7	6	6	3
ENSMUSG00000018581	2	2	3	0	4	0
ENSMUSG00000064843	0	0	0	0	0	0
ENSMUSG00000057215	0	0	0	0	0	0
ENSMUSG00000081220	0	0	0	0	0	0
ENSMUSG00000104921	0	0	0	0	0	0
ENSMUSG00000060180	2	0	2	2	0	0
ENSMUSG00000104928	0	0	0	0	0	0
ENSMUSG00000109740	2	5	2	6	1	2
ENSMUSG00000100203	0	0	0	0	0	0
ENSMUSG00000100202	0	0	0	0	0	0
ENSMUSG00000100205	0	0	0	0	0	0
ENSMUSG00000100204	3	0	2	3	0	3
ENSMUSG00000100207	0	0	0	0	0	0

ENSMUSG00000100206	0	0	0	0	0	0
ENSMUSG00000100209	0	0	0	0	0	0
ENSMUSG00000042401	0	0	4	0	0	0
ENSMUSG00000110709	0	0	0	0	0	0
ENSMUSG00000097543	0	0	0	0	0	0
ENSMUSG00000088417	0	0	0	0	0	0
ENSMUSG00000088415	1	0	0	0	0	0
ENSMUSG00000088410	0	0	0	0	1	0
ENSMUSG00000088419	0	0	0	0	0	0
ENSMUSG00000088418	0	0	0	0	0	0
ENSMUSG00000086525	0	0	0	0	0	2
ENSMUSG00000086524	0	0	0	1	2	3
ENSMUSG00000086521	0	0	0	0	0	0
ENSMUSG00000086523	0	0	0	0	0	0
ENSMUSG00000081962	0	0	0	0	0	0
ENSMUSG00000093351	1	0	0	0	0	0
ENSMUSG00000093352	0	0	3	0	2	1
ENSMUSG00000039257	0	0	0	0	0	0
ENSMUSG00000093358	0	0	0	0	0	0
ENSMUSG00000093359	1	1	0	0	0	0
ENSMUSG00000065132	0	0	0	0	0	0
ENSMUSG00000052469	0	0	0	0	0	0
ENSMUSG00000009654	0	0	0	0	0	0
ENSMUSG00000036557	0	0	4	0	6	0
ENSMUSG00000017588	0	0	0	0	0	0
ENSMUSG00000036555	332	281	316	208	257	277
ENSMUSG00000113293	0	0	0	0	0	1
ENSMUSG00000087723	0	0	0	0	0	0
ENSMUSG00000112748	2	1	0	0	0	0
ENSMUSG00000112742	0	0	0	3	0	0
ENSMUSG00000112747	0	0	0	0	0	0
ENSMUSG00000020140	11	6	5	5	4	3
ENSMUSG00000097072	0	0	0	0	0	0
ENSMUSG00000097074	0	0	0	0	0	0
ENSMUSG00000092009	0	0	0	0	0	0
ENSMUSG00000097076	0	0	0	0	0	0
ENSMUSG00000097079	0	3	0	0	0	2
ENSMUSG00000035775	0	0	2	0	0	0
ENSMUSG00000110675	0	0	0	0	0	0
ENSMUSG00000110674	0	0	0	0	0	0
ENSMUSG00000025014	0	0	0	0	0	0
ENSMUSG00000110676	26	15	28	19	37	29
ENSMUSG00000110671	0	0	0	0	0	0
ENSMUSG00000110670	2	0	0	0	0	0
ENSMUSG00000025010	244	265	269	295	354	374
ENSMUSG00000110672	0	0	0	2	0	0
ENSMUSG00000005493	4	9	12	9	18	8
ENSMUSG00000110678	0	0	0	0	0	0
ENSMUSG00000087389	0	0	0	0	0	0
ENSMUSG00000110465	2	0	0	0	0	0
ENSMUSG00000051340	1	0	0	0	0	0
ENSMUSG00000105649	0	0	0	0	0	0
ENSMUSG00000105642	1	0	0	0	0	0
ENSMUSG00000105640	0	0	0	0	0	0
ENSMUSG00000114744	0	0	0	0	0	0
ENSMUSG00000114747	0	0	0	0	0	0
ENSMUSG00000114741	0	0	0	0	3	0
ENSMUSG00000114740	0	0	0	0	0	0

ENSMUSG00000104506	0	0	0	0	0	0
ENSMUSG00000050435	0	0	0	0	0	0
ENSMUSG00000070324	0	0	0	0	0	0
ENSMUSG00000070323	28	26	11	4	0	6
ENSMUSG00000108078	0	0	0	0	0	0
ENSMUSG00000108079	0	0	5	0	0	0
ENSMUSG00000108077	0	0	0	0	0	4
ENSMUSG00000005148	5	0	8	3	3	4
ENSMUSG00000108072	0	0	0	0	0	0
ENSMUSG00000050439	0	0	0	0	0	0
ENSMUSG00000108071	0	0	4	0	2	0
ENSMUSG00000083008	0	0	0	0	0	0
ENSMUSG00000083001	0	0	0	0	0	0
ENSMUSG00000112644	0	0	0	0	0	0
ENSMUSG00000112645	0	0	0	0	0	0
ENSMUSG00000040828	0	0	6	0	6	0
ENSMUSG00000099256	0	0	0	0	0	0
ENSMUSG00000111104	9	17	12	12	12	19
ENSMUSG00000111105	0	0	0	0	0	0
ENSMUSG00000111106	0	0	0	0	0	0
ENSMUSG00000111100	0	0	0	0	0	0
ENSMUSG00000111102	0	0	0	0	0	0
ENSMUSG00000081879	0	0	0	0	0	0
ENSMUSG00000054667	0	0	0	0	0	0
ENSMUSG00000081876	0	0	0	0	0	0
ENSMUSG00000027703	0	4	0	0	0	0
ENSMUSG00000044560	0	0	0	0	0	0
ENSMUSG00000026090	28	27	13	26	37	46
ENSMUSG00000083118	0	0	2	3	3	2
ENSMUSG00000041886	0	4	2	0	0	0
ENSMUSG00000041885	0	0	0	0	0	0
ENSMUSG00000078256	0	0	0	0	0	0
ENSMUSG00000078257	0	2	0	0	0	0
ENSMUSG00000078254	0	0	0	0	0	0
ENSMUSG00000083608	0	1	0	0	0	0
ENSMUSG00000078252	0	0	0	2	0	0
ENSMUSG00000078258	0	0	0	0	0	0
ENSMUSG00000078259	0	0	0	0	0	0
ENSMUSG00000090098	22	0	0	11	7	24
ENSMUSG00000090099	0	0	0	0	0	0
ENSMUSG00000090097	0	0	0	0	0	0
ENSMUSG00000090090	0	0	0	0	0	0
ENSMUSG00000081335	0	0	0	0	0	0
ENSMUSG00000081334	0	0	0	0	0	0
ENSMUSG00000081330	0	0	0	0	1	2
ENSMUSG00000081333	0	0	0	0	0	0
ENSMUSG00000081332	3	0	0	0	0	0
ENSMUSG00000094470	0	0	0	0	0	0
ENSMUSG00000027326	115	103	137	29	49	67
ENSMUSG00000083607	0	0	0	0	0	0
ENSMUSG00000092834	0	0	0	0	0	0
ENSMUSG00000092835	0	0	1	2	0	4
ENSMUSG00000092832	0	0	0	0	0	0
ENSMUSG00000092830	0	0	0	0	0	0
ENSMUSG00000046057	2	1	1	0	0	0
ENSMUSG00000079580	0	0	0	0	0	0
ENSMUSG00000090549	0	2	1	4	0	0
ENSMUSG00000084861	0	0	0	0	0	3

ENSMUSG00000084866	16	19	19	22	16	13
ENSMUSG00000090546	5	0	0	0	0	0
ENSMUSG00000084865	0	2	0	0	0	3
ENSMUSG00000098779	0	0	0	0	0	0
ENSMUSG00000098778	0	0	0	0	0	0
ENSMUSG00000094044	0	0	0	0	0	0
ENSMUSG00000098772	0	0	0	0	0	0
ENSMUSG00000098777	0	0	0	0	0	0
ENSMUSG00000098776	0	0	0	0	0	0
ENSMUSG00000098775	0	0	0	0	0	0
ENSMUSG00000073006	0	0	0	0	0	0
ENSMUSG00000073000	25	44	40	52	45	31
ENSMUSG00000073001	0	0	0	0	0	0
ENSMUSG00000073008	12	22	33	6	12	8
ENSMUSG00000078924	0	0	0	0	0	0
ENSMUSG00000038738	60	54	52	29	28	44
ENSMUSG00000031284	206	109	169	14	27	14
ENSMUSG00000031285	0	0	0	0	0	0
ENSMUSG00000086475	0	0	0	0	0	0
ENSMUSG00000093015	1	0	0	1	1	1
ENSMUSG00000093014	0	0	0	0	0	0
ENSMUSG00000104505	0	0	0	0	0	0
ENSMUSG00000093010	0	0	0	0	0	0
ENSMUSG00000093019	0	0	0	0	0	0
ENSMUSG00000086470	0	6	3	0	4	3
ENSMUSG00000013338	4	2	0	0	2	0
ENSMUSG00000023093	0	0	0	0	0	0
ENSMUSG00000101310	0	0	0	0	0	0
ENSMUSG00000073915	2	0	0	0	0	0
ENSMUSG00000101312	0	0	0	0	0	0
ENSMUSG00000101313	0	0	0	0	0	0
ENSMUSG00000073910	46	46	54	57	50	55
ENSMUSG00000094326	17	14	12	14	9	18
ENSMUSG00000073913	0	0	0	0	0	0
ENSMUSG00000040452	0	0	0	0	3	0
ENSMUSG00000069080	0	0	0	0	0	0
ENSMUSG00000071519	0	0	0	0	0	0
ENSMUSG00000071517	0	0	0	0	0	0
ENSMUSG00000071510	17	8	3	6	8	2
ENSMUSG00000103221	0	0	0	0	0	0
ENSMUSG00000103226	0	0	0	0	0	0
ENSMUSG00000103224	2	6	1	4	1	0
ENSMUSG00000043811	0	58	0	35	62	94
ENSMUSG00000082015	0	0	0	0	0	0
ENSMUSG00000087062	0	0	0	0	0	0
ENSMUSG00000087061	0	0	0	0	0	0
ENSMUSG00000087060	13	20	8	9	13	22
ENSMUSG00000087067	0	10	20	3	17	0
ENSMUSG00000082010	0	0	0	0	0	0
ENSMUSG00000082012	0	0	0	1	0	0
ENSMUSG00000087069	0	0	0	0	0	0
ENSMUSG00000082019	0	0	0	0	0	0
ENSMUSG00000082018	0	0	0	0	2	0
ENSMUSG00000099554	0	0	0	0	0	0
ENSMUSG00000099555	0	0	0	0	0	0
ENSMUSG00000099556	0	0	0	0	0	0
ENSMUSG00000099550	0	0	0	0	0	0
ENSMUSG00000099551	0	0	0	0	0	0

ENSMUSG00000099552	0	7	4	0	0	0
ENSMUSG00000099553	0	0	0	0	0	0
ENSMUSG00000099558	0	0	0	2	0	0
ENSMUSG00000099559	0	0	0	0	0	0
ENSMUSG00000032717	51	39	72	23	0	16
ENSMUSG00000089301	0	0	0	0	0	0
ENSMUSG00000083760	0	0	2	0	0	0
ENSMUSG00000069085	0	0	0	0	0	0
ENSMUSG00000100049	0	0	0	0	0	0
ENSMUSG00000100048	0	0	0	1	0	0
ENSMUSG00000100047	0	0	0	0	0	0
ENSMUSG00000100046	1	4	2	0	0	0
ENSMUSG00000100045	0	0	0	0	0	0
ENSMUSG00000100044	0	0	2	0	0	0
ENSMUSG00000100043	0	0	0	0	0	0
ENSMUSG00000100042	0	0	0	0	0	0
ENSMUSG00000100041	2	5	0	2	12	9
ENSMUSG00000100040	0	0	0	0	0	0
ENSMUSG00000102579	0	5	0	0	0	0
ENSMUSG00000102578	0	0	0	1	0	0
ENSMUSG00000102572	0	0	0	0	0	0
ENSMUSG00000102574	0	2	2	0	0	0
ENSMUSG00000084089	0	0	0	0	0	0
ENSMUSG00000084086	0	1	0	0	0	0
ENSMUSG00000084080	0	0	0	0	0	0
ENSMUSG00000084082	0	0	0	0	0	0
ENSMUSG00000084083	0	0	0	0	0	0
ENSMUSG00000100598	0	0	0	0	0	0
ENSMUSG00000100591	0	0	0	0	0	0
ENSMUSG00000065840	2	0	0	0	0	0
ENSMUSG00000065843	0	0	0	0	0	0
ENSMUSG00000100592	0	0	0	0	0	0
ENSMUSG00000100595	0	0	0	0	0	3
ENSMUSG00000100594	52	52	52	69	68	98
ENSMUSG00000100597	0	0	0	0	0	0
ENSMUSG00000087685	0	3	0	0	4	0
ENSMUSG00000087684	4	12	20	4	6	8
ENSMUSG00000087682	0	0	0	0	0	0
ENSMUSG00000087689	0	0	0	0	0	1
ENSMUSG00000087688	0	0	0	0	0	0
ENSMUSG00000085681	0	0	0	0	0	0
ENSMUSG00000085680	0	0	0	0	0	0
ENSMUSG00000085683	0	0	0	4	0	0
ENSMUSG00000085682	4	7	6	11	25	13
ENSMUSG00000085686	0	0	0	0	0	0
ENSMUSG00000064460	0	0	0	0	0	0
ENSMUSG00000064463	0	0	0	0	0	0
ENSMUSG00000064464	0	0	0	0	0	0
ENSMUSG00000064467	0	0	0	0	0	0
ENSMUSG00000102683	0	0	0	0	0	0
ENSMUSG00000102682	0	0	0	0	0	0
ENSMUSG00000102681	0	0	0	0	0	0
ENSMUSG00000102680	0	0	0	0	0	0
ENSMUSG00000102687	8	3	4	3	8	3
ENSMUSG00000102686	0	0	0	0	0	0
ENSMUSG00000102685	1	3	0	3	4	2
ENSMUSG00000102684	8	3	7	0	3	3
ENSMUSG00000107060	5	8	5	0	0	0

ENSMUSG00000107061	9	9	9	5	6	11
ENSMUSG00000102689	2	0	0	0	0	0
ENSMUSG00000107063	0	0	0	0	0	0
ENSMUSG00000107064	0	0	0	0	0	0
ENSMUSG00000107065	0	0	0	0	0	0
ENSMUSG00000107067	3	0	3	0	0	0
ENSMUSG00000079649	0	0	0	0	0	0
ENSMUSG00000079644	0	0	0	0	0	0
ENSMUSG00000079642	0	0	0	0	0	0
ENSMUSG00000065597	0	0	0	0	0	0
ENSMUSG00000065596	0	0	0	0	0	0
ENSMUSG00000023140	0	0	0	0	0	0
ENSMUSG00000068748	26	17	52	33	13	36
ENSMUSG00000082426	3	0	0	0	0	0
ENSMUSG00000085359	0	0	0	0	0	0
ENSMUSG00000085358	0	0	0	0	0	0
ENSMUSG00000088553	0	0	0	0	0	0
ENSMUSG00000085353	0	0	0	0	0	0
ENSMUSG00000085352	0	0	0	0	0	0
ENSMUSG00000085351	0	0	0	2	0	4
ENSMUSG00000085350	0	0	0	0	0	0
ENSMUSG00000085357	0	0	0	0	0	0
ENSMUSG00000038227	0	0	0	0	0	0
ENSMUSG00000074072	0	0	0	0	0	0
ENSMUSG00000074071	8	6	29	6	0	6
ENSMUSG00000036718	93	114	119	62	80	101
ENSMUSG00000087965	0	0	0	0	0	0
ENSMUSG00000094205	0	0	0	0	0	0
ENSMUSG00000101880	0	0	2	0	0	0
ENSMUSG00000101881	0	0	0	0	0	0
ENSMUSG00000101882	1	0	0	0	0	0
ENSMUSG00000101883	0	0	0	0	0	0
ENSMUSG00000101885	0	0	0	0	0	0
ENSMUSG00000101887	0	0	0	0	0	0
ENSMUSG00000088884	0	0	0	0	0	0
ENSMUSG00000039728	0	0	0	0	0	0
ENSMUSG00000094898	0	0	0	0	0	0
ENSMUSG00000021789	0	0	0	0	0	0
ENSMUSG00000094892	0	0	0	0	0	0
ENSMUSG00000039720	0	0	0	0	0	0
ENSMUSG00000094890	0	0	0	0	0	0
ENSMUSG00000094891	18	18	13	27	30	28
ENSMUSG00000094897	0	0	0	0	0	0
ENSMUSG00000094894	0	0	0	0	0	0
ENSMUSG00000094895	0	0	0	0	0	0
ENSMUSG00000024497	0	0	0	0	0	0
ENSMUSG00000103769	1	1	0	0	0	0
ENSMUSG00000103767	11	12	19	2	4	4
ENSMUSG00000061653	0	0	0	0	0	0
ENSMUSG00000061654	0	0	0	0	0	0
ENSMUSG00000103762	8	0	3	3	3	3
ENSMUSG00000103761	2	0	4	0	2	0
ENSMUSG00000097278	4	6	8	1	4	6
ENSMUSG00000098036	0	0	0	0	2	0
ENSMUSG00000097271	41	30	29	54	58	105
ENSMUSG00000059371	0	0	0	0	0	0
ENSMUSG00000063651	0	0	0	0	0	0
ENSMUSG00000059375	0	0	0	0	0	0

ENSMUSG00000028354	3	10	0	6	6	9
ENSMUSG00000089685	0	0	0	0	0	0
ENSMUSG00000089684	0	0	0	0	0	0
ENSMUSG00000089689	0	0	0	0	0	0
ENSMUSG00000066027	0	0	0	0	0	0
ENSMUSG00000092731	0	0	0	0	0	0
ENSMUSG00000097920	0	0	0	0	0	0
ENSMUSG00000109508	0	0	0	0	0	0
ENSMUSG00000109504	0	0	0	0	0	0
ENSMUSG00000109501	0	0	0	0	0	0
ENSMUSG00000100861	0	0	0	0	0	0
ENSMUSG00000114502	0	0	0	0	0	0
ENSMUSG00000114507	0	0	2	0	0	0
ENSMUSG00000114506	0	0	0	0	0	0
ENSMUSG00000114505	0	0	0	0	0	0
ENSMUSG00000100866	0	0	0	0	0	0
ENSMUSG00000100869	0	0	0	0	0	0
ENSMUSG00000114509	0	0	0	0	0	0
ENSMUSG00000114508	0	0	0	0	0	0
ENSMUSG00000062309	47	30	32	20	45	24
ENSMUSG00000066688	0	0	0	0	0	0
ENSMUSG00000058427	11	19	5	0	2	3
ENSMUSG00000066682	47	34	22	14	18	17
ENSMUSG00000112839	0	0	0	0	0	0
ENSMUSG00000108679	0	0	0	0	0	0
ENSMUSG00000054555	144	110	161	32	35	56
ENSMUSG00000112835	10	13	3	18	9	16
ENSMUSG00000108675	0	0	0	0	0	0
ENSMUSG00000112837	2	0	0	4	0	0
ENSMUSG00000108670	0	3	0	0	0	0
ENSMUSG00000108671	13	11	14	0	6	2
ENSMUSG00000112833	5	0	0	0	3	0
ENSMUSG00000112832	0	0	0	0	0	0
ENSMUSG00000071788	0	0	0	0	0	0
ENSMUSG00000105336	0	0	0	0	0	0
ENSMUSG00000105337	0	4	2	0	0	0
ENSMUSG00000105334	0	0	0	0	0	0
ENSMUSG00000088160	0	0	0	0	0	0
ENSMUSG00000105330	1	0	1	0	0	0
ENSMUSG00000105331	0	0	1	0	0	0
ENSMUSG00000082143	2	0	0	0	0	0
ENSMUSG00000095336	0	0	0	0	0	1
ENSMUSG00000082142	0	0	0	0	0	0
ENSMUSG00000058488	0	10	0	0	4	4
ENSMUSG00000095330	0	0	0	0	0	0
ENSMUSG00000082140	0	0	0	1	0	0
ENSMUSG00000060688	0	0	0	0	0	0
ENSMUSG00000082504	0	0	0	0	0	0
ENSMUSG00000015619	6	0	4	0	8	8
ENSMUSG00000091942	4	4	6	4	0	4
ENSMUSG00000091945	0	0	0	0	0	0
ENSMUSG00000015222	433	425	465	326	348	278
ENSMUSG00000096639	0	0	0	0	0	0
ENSMUSG00000077377	0	0	0	0	0	0
ENSMUSG00000096282	0	0	0	0	0	0
ENSMUSG00000043529	0	0	0	0	0	0
ENSMUSG00000096630	0	0	0	0	0	0
ENSMUSG00000096634	0	0	0	0	0	0

ENSMUSG00000096635	2	1	0	1	0	0
ENSMUSG00000023926	0	0	3	3	0	0
ENSMUSG00000108505	0	0	0	0	0	0
ENSMUSG00000098314	0	0	0	0	0	1
ENSMUSG00000113874	0	0	0	0	0	0
ENSMUSG00000101057	0	0	0	0	0	0
ENSMUSG00000101056	0	0	0	0	0	0
ENSMUSG00000101055	0	0	0	0	0	0
ENSMUSG00000101054	0	0	0	0	0	0
ENSMUSG00000101053	0	0	0	0	0	0
ENSMUSG00000101052	0	0	0	0	0	0
ENSMUSG00000101050	0	0	0	0	0	0
ENSMUSG00000113872	0	0	2	0	0	0
ENSMUSG00000101058	0	0	0	0	0	0
ENSMUSG00000037600	5	5	7	3	9	11
ENSMUSG00000021209	28	32	26	33	33	24
ENSMUSG00000026253	10	5	0	0	5	0
ENSMUSG00000111022	0	0	0	2	0	0
ENSMUSG00000111020	0	0	0	0	0	0
ENSMUSG00000111029	0	0	0	0	0	0
ENSMUSG00000110998	0	0	0	0	0	0
ENSMUSG00000110999	0	0	0	0	0	0
ENSMUSG00000096033	0	0	0	0	0	0
ENSMUSG00000110991	0	0	0	0	0	0
ENSMUSG00000110992	0	0	0	0	0	0
ENSMUSG00000110995	0	0	0	0	0	0
ENSMUSG00000110996	0	0	0	0	0	0
ENSMUSG00000107686	0	0	0	0	0	0
ENSMUSG00000060534	0	0	0	0	0	0
ENSMUSG00000111609	0	0	0	0	0	0
ENSMUSG00000091361	0	0	0	0	0	0
ENSMUSG00000096035	4	0	0	0	0	8
ENSMUSG00000091368	1	0	1	0	1	1
ENSMUSG00000096285	0	0	0	0	0	0
ENSMUSG00000081133	0	0	0	0	0	0
ENSMUSG00000081132	2	0	0	1	0	0
ENSMUSG00000081131	0	0	0	0	0	0
ENSMUSG00000081130	0	0	0	0	0	0
ENSMUSG00000081136	4	3	3	2	3	4
ENSMUSG00000081135	0	1	0	0	0	0
ENSMUSG00000081134	0	0	0	0	0	0
ENSMUSG00000081139	18	16	21	13	13	21
ENSMUSG00000081138	0	2	0	0	0	0
ENSMUSG00000111601	0	0	0	0	2	1
ENSMUSG00000033114	166	137	162	83	100	105
ENSMUSG00000027120	0	0	0	0	0	0
ENSMUSG00000113301	0	0	0	0	0	0
ENSMUSG00000109800	0	0	0	0	0	0
ENSMUSG00000111604	0	0	0	0	0	0
ENSMUSG00000090704	0	0	0	0	0	0
ENSMUSG00000081689	0	0	0	0	0	0
ENSMUSG00000081688	0	0	0	0	0	0
ENSMUSG00000090700	0	0	0	0	0	0
ENSMUSG00000081685	0	0	0	0	0	0
ENSMUSG00000103338	0	0	0	0	0	0
ENSMUSG00000081687	0	0	0	0	0	0
ENSMUSG00000113307	0	0	0	0	0	0
ENSMUSG00000081681	0	0	0	0	0	0

ENSMUSG00000081680	0	0	0	0	0	0
ENSMUSG00000081683	0	0	0	0	3	0
ENSMUSG00000038022	352	290	320	191	133	273
ENSMUSG00000053675	0	0	3	0	0	0
ENSMUSG00000053678	0	0	0	0	0	0
ENSMUSG00000008489	7	4	3	4	3	11
ENSMUSG00000103334	0	0	0	0	0	0
ENSMUSG00000094461	0	0	0	0	0	0
ENSMUSG00000094460	0	0	0	0	0	0
ENSMUSG00000094467	0	0	2	1	1	3
ENSMUSG00000094466	0	0	0	0	0	0
ENSMUSG00000094465	0	0	1	0	0	0
ENSMUSG00000094464	0	0	0	0	0	0
ENSMUSG00000056043	0	3	4	0	1	6
ENSMUSG00000086483	0	0	0	0	0	0
ENSMUSG00000086480	2	0	0	0	0	0
ENSMUSG00000086481	2	0	3	0	0	0
ENSMUSG00000086485	0	0	4	5	7	0
ENSMUSG00000103330	0	0	0	0	0	0
ENSMUSG00000103331	7	15	0	3	6	7
ENSMUSG00000063230	0	0	0	0	0	0
ENSMUSG00000044966	28	12	29	6	16	16
ENSMUSG00000088312	0	0	0	0	0	0
ENSMUSG00000103333	0	0	0	0	0	0
ENSMUSG00000077019	1	1	1	0	1	1
ENSMUSG00000077015	0	0	1	0	0	0
ENSMUSG00000077010	0	0	0	0	0	0
ENSMUSG00000107187	0	0	0	0	0	0
ENSMUSG00000107185	1	1	4	1	0	2
ENSMUSG00000094287	0	0	0	0	0	0
ENSMUSG00000094286	3	4	4	3	2	1
ENSMUSG00000094285	0	0	0	0	0	0
ENSMUSG00000046449	42	34	46	22	27	30
ENSMUSG00000094283	0	0	0	0	0	0
ENSMUSG00000094280	0	0	0	0	0	0
ENSMUSG00000109581	0	2	0	0	3	0
ENSMUSG00000094288	0	0	0	0	0	0
ENSMUSG00000088987	0	0	0	0	0	0
ENSMUSG00000045493	3	0	0	0	0	0
ENSMUSG00000092702	13	26	21	4	4	6
ENSMUSG00000092705	0	0	0	0	0	0
ENSMUSG00000109580	0	1	0	0	0	0
ENSMUSG00000100902	0	0	0	0	0	0
ENSMUSG00000052363	0	0	0	0	0	0
ENSMUSG00000055095	0	0	0	0	0	0
ENSMUSG00000029436	81	57	79	58	50	80
ENSMUSG00000049321	202	303	159	297	277	275
ENSMUSG00000051257	0	0	0	0	0	0
ENSMUSG00000113319	0	0	0	0	0	0
ENSMUSG00000054944	23	9	22	0	4	2
ENSMUSG00000113312	0	0	0	0	0	0
ENSMUSG00000054940	0	0	0	0	0	0
ENSMUSG00000113316	0	0	0	0	0	0
ENSMUSG00000113314	0	0	0	0	0	0
ENSMUSG00000111025	0	0	0	0	1	0
ENSMUSG00000060161	0	0	0	0	0	0
ENSMUSG00000071750	0	0	0	0	0	0
ENSMUSG00000071753	3	0	0	3	0	0

ENSMUSG00000109264	0	0	0	0	0	0
ENSMUSG00000109259	0	0	0	0	0	0
ENSMUSG00000109266	0	0	0	0	0	0
ENSMUSG00000109267	0	0	0	0	0	0
ENSMUSG00000109260	0	0	0	4	5	0
ENSMUSG00000101792	8	0	4	8	0	0
ENSMUSG00000109262	0	0	0	0	0	0
ENSMUSG00000109263	0	0	0	0	0	0
ENSMUSG00000101793	0	0	0	0	0	0
ENSMUSG00000109269	0	0	0	0	0	0
ENSMUSG00000106529	0	0	0	0	0	0
ENSMUSG00000106526	0	0	0	0	0	0
ENSMUSG00000106527	0	0	0	0	0	0
ENSMUSG00000106524	0	0	0	0	0	0
ENSMUSG00000106525	0	0	0	0	0	3
ENSMUSG00000106520	0	0	0	0	0	0
ENSMUSG00000106521	0	0	0	0	0	0
ENSMUSG00000051258	0	0	0	0	0	2
ENSMUSG00000089266	0	0	0	0	0	0
ENSMUSG00000082569	0	0	0	0	0	0
ENSMUSG00000082565	0	0	0	0	0	0
ENSMUSG00000082564	0	0	0	0	0	0
ENSMUSG00000082567	0	0	0	0	0	0
ENSMUSG00000082566	0	0	0	0	0	0
ENSMUSG00000025197	0	0	0	0	0	0
ENSMUSG00000025196	4	8	0	0	0	0
ENSMUSG00000082563	0	0	0	0	0	0
ENSMUSG00000025194	0	3	3	0	0	0
ENSMUSG00000106552	0	0	0	0	0	0
ENSMUSG00000109589	0	0	0	0	0	0
ENSMUSG00000005267	281	286	301	190	219	213
ENSMUSG00000084665	0	0	0	0	0	0
ENSMUSG00000077092	0	0	0	0	0	0
ENSMUSG00000083810	0	0	0	0	0	0
ENSMUSG00000106556	0	0	0	0	0	0
ENSMUSG00000084248	0	0	0	0	0	0
ENSMUSG00000083218	0	1	3	4	1	2
ENSMUSG00000109588	0	0	0	0	0	0
ENSMUSG00000083214	0	0	0	0	0	0
ENSMUSG00000083215	0	0	0	0	0	0
ENSMUSG00000084240	4	4	4	4	4	4
ENSMUSG00000084246	0	0	0	0	0	0
ENSMUSG00000083211	0	0	0	0	0	0
ENSMUSG00000083212	0	0	0	0	0	0
ENSMUSG00000083213	1	0	0	2	0	0
ENSMUSG00000083815	2	0	2	6	2	1
ENSMUSG00000070489	0	0	0	0	0	1
ENSMUSG00000041255	0	0	0	0	0	0
ENSMUSG00000025229	0	10	4	2	0	0
ENSMUSG00000083819	0	2	1	3	1	1
ENSMUSG00000099169	0	0	0	0	0	0
ENSMUSG00000099168	0	0	0	0	0	0
ENSMUSG00000099165	1	1	1	1	0	0
ENSMUSG00000099166	2	2	0	0	2	0
ENSMUSG00000099162	0	0	0	0	0	0
ENSMUSG00000074776	0	0	0	0	0	0
ENSMUSG00000097085	0	4	5	5	0	5
ENSMUSG00000075502	37	34	41	50	47	55

ENSMUSG00000068506	0	0	0	0	0	0
ENSMUSG00000006764	0	0	6	0	0	0
ENSMUSG00000085113	0	0	6	0	0	3
ENSMUSG00000085112	19	18	11	11	5	15
ENSMUSG00000085115	0	0	0	0	0	0
ENSMUSG00000085114	0	0	0	0	0	0
ENSMUSG00000074678	0	3	0	0	0	4
ENSMUSG00000074679	0	0	0	0	0	0
ENSMUSG00000074677	11	21	27	1	7	11
ENSMUSG00000074671	112	94	82	44	47	62
ENSMUSG00000074673	17	6	16	6	10	22
ENSMUSG00000026751	0	0	0	0	0	0
ENSMUSG00000079330	0	0	0	3	0	0
ENSMUSG00000041923	0	0	2	0	0	0
ENSMUSG00000045202	0	0	0	0	0	0
ENSMUSG00000045204	0	0	0	0	0	0
ENSMUSG00000104221	2	0	0	0	0	0
ENSMUSG00000071818	0	0	0	0	0	0
ENSMUSG00000114657	0	0	0	0	0	0
ENSMUSG00000114654	0	3	2	0	0	0
ENSMUSG00000089914	0	0	0	0	0	0
ENSMUSG00000048516	0	0	0	0	0	0
ENSMUSG00000111308	0	0	2	2	2	0
ENSMUSG00000099129	0	0	0	0	0	0
ENSMUSG00000109862	1	0	0	0	0	0
ENSMUSG00000112044	0	0	0	0	0	0
ENSMUSG00000030292	0	4	0	0	4	8
ENSMUSG00000088992	0	0	1	0	0	0
ENSMUSG00000034990	18	18	32	22	12	17
ENSMUSG00000034997	61	83	27	74	43	101
ENSMUSG00000112041	7	4	5	14	7	15
ENSMUSG00000075431	0	0	0	0	0	0
ENSMUSG00000094173	0	0	0	0	0	0
ENSMUSG00000020212	200	178	178	176	183	252
ENSMUSG00000094172	0	0	0	0	0	0
ENSMUSG00000109806	0	0	0	0	2	0
ENSMUSG00000095906	0	0	0	0	0	0
ENSMUSG00000095905	0	1	1	1	0	1
ENSMUSG00000087180	0	0	0	0	0	0
ENSMUSG00000112048	0	0	0	1	6	2
ENSMUSG00000087184	0	0	0	0	0	0
ENSMUSG00000087185	0	0	0	0	0	0
ENSMUSG00000087187	7	3	6	3	2	0
ENSMUSG00000109861	0	0	0	0	0	0
ENSMUSG00000085968	0	0	0	0	0	0
ENSMUSG00000101294	1	1	0	0	0	0
ENSMUSG00000101297	0	0	0	0	0	0
ENSMUSG00000101296	0	0	0	0	0	0
ENSMUSG00000101291	0	0	0	0	0	0
ENSMUSG00000101290	0	0	0	0	0	0
ENSMUSG00000055312	0	2	0	4	4	0
ENSMUSG00000085961	0	0	0	0	0	0
ENSMUSG00000101299	0	0	0	0	0	0
ENSMUSG00000101298	0	0	0	0	0	0
ENSMUSG00000085966	0	0	0	0	0	0
ENSMUSG00000085967	4	0	15	8	20	4
ENSMUSG00000077470	1	0	0	1	0	0
ENSMUSG00000033676	35	31	54	9	4	10

ENSMUSG00000087230	0	0	0	0	0	0
ENSMUSG00000104439	0	0	0	0	0	0
ENSMUSG00000087238	0	0	0	0	0	0
ENSMUSG00000105193	0	0	0	0	0	0
ENSMUSG00000060332	0	2	0	0	0	0
ENSMUSG00000111002	2	0	0	0	0	0
ENSMUSG00000060335	0	0	0	0	0	0
ENSMUSG00000040640	75	83	110	77	72	37
ENSMUSG00000104023	0	0	0	0	0	0
ENSMUSG00000100658	62	75	233	79	90	90
ENSMUSG00000100659	0	0	0	0	0	0
ENSMUSG00000100656	0	0	0	0	0	0
ENSMUSG00000100657	0	0	0	0	0	0
ENSMUSG00000100654	0	0	0	0	0	0
ENSMUSG00000100655	0	0	0	0	0	0
ENSMUSG00000100652	0	0	0	0	0	0
ENSMUSG00000100653	0	0	0	0	0	0
ENSMUSG00000100650	0	0	0	0	0	0
ENSMUSG00000100651	0	0	0	0	0	0
ENSMUSG00000107929	0	0	0	0	0	0
ENSMUSG00000107923	0	0	0	0	0	0
ENSMUSG00000059921	122	75	94	25	17	10
ENSMUSG00000107924	0	0	1	0	0	2
ENSMUSG00000107927	4	2	4	0	0	0
ENSMUSG00000107926	0	0	0	0	0	0
ENSMUSG00000084760	4	0	3	0	0	2
ENSMUSG00000084762	0	0	0	0	0	2
ENSMUSG00000084766	0	0	0	2	0	0
ENSMUSG00000084768	0	0	0	0	0	0
ENSMUSG00000092431	0	0	0	0	0	0
ENSMUSG00000022422	0	10	4	7	6	11
ENSMUSG00000090636	0	0	0	0	0	1
ENSMUSG00000032108	0	0	0	0	0	0
ENSMUSG00000024863	0	0	0	0	0	0
ENSMUSG00000024868	0	0	0	0	0	0
ENSMUSG00000020374	7	9	0	26	0	30
ENSMUSG00000065045	0	0	0	0	0	0
ENSMUSG00000102728	4	8	1	2	3	4
ENSMUSG00000102729	0	0	0	0	0	0
ENSMUSG00000112208	0	0	0	0	0	0
ENSMUSG00000112209	3	0	0	0	2	0
ENSMUSG00000112202	0	0	0	0	0	0
ENSMUSG00000060807	0	0	0	0	0	0
ENSMUSG00000112200	0	2	0	1	0	0
ENSMUSG00000112206	0	0	0	0	0	0
ENSMUSG00000112207	0	0	0	0	0	0
ENSMUSG00000102726	0	0	0	0	0	0
ENSMUSG00000112205	0	0	0	0	0	0
ENSMUSG00000028109	0	0	0	0	0	0
ENSMUSG00000095632	0	0	0	0	0	0
ENSMUSG00000094919	1	0	0	0	0	0
ENSMUSG00000094917	0	0	0	0	0	0
ENSMUSG00000094915	4	6	10	6	4	7
ENSMUSG00000094913	0	0	0	0	0	0
ENSMUSG00000094912	0	0	0	0	0	0
ENSMUSG00000094911	0	0	0	0	0	0
ENSMUSG00000094910	53	40	77	25	29	59
ENSMUSG00000038379	24	28	8	6	6	7

ENSMUSG00000113158	0	0	0	0	0	0
ENSMUSG00000113153	0	0	0	0	0	0
ENSMUSG00000023484	18	5	13	0	0	0
ENSMUSG00000113155	0	0	0	0	0	0
ENSMUSG00000114268	1	0	0	0	0	1
ENSMUSG00000113157	50	44	43	32	42	33
ENSMUSG00000113156	0	0	0	0	0	0
ENSMUSG00000052912	27	37	39	46	48	34
ENSMUSG00000024134	0	0	3	0	0	0
ENSMUSG00000104955	0	0	0	0	0	0
ENSMUSG00000106729	10	12	12	6	13	5
ENSMUSG00000104954	3	2	0	0	0	0
ENSMUSG00000106725	5	0	0	0	0	0
ENSMUSG00000106726	0	0	0	0	0	2
ENSMUSG00000106466	0	0	0	0	0	0
ENSMUSG00000106721	0	0	0	0	0	0
ENSMUSG00000101704	0	0	0	0	0	1
ENSMUSG00000101702	0	3	0	3	0	0
ENSMUSG00000104763	0	0	0	0	0	0
ENSMUSG00000104760	0	0	0	0	0	0
ENSMUSG00000089422	0	0	0	0	0	0
ENSMUSG00000101700	0	0	0	0	0	0
ENSMUSG00000108698	0	0	0	0	0	0
ENSMUSG00000109718	0	0	0	0	0	0
ENSMUSG00000109719	5	1	5	1	2	2
ENSMUSG00000109714	19	23	15	9	16	18
ENSMUSG00000065078	1	0	0	0	1	0
ENSMUSG00000111796	0	0	0	0	0	0
ENSMUSG00000095932	0	0	0	0	0	0
ENSMUSG00000109711	0	0	0	0	0	0
ENSMUSG00000089490	0	0	0	0	0	0
ENSMUSG00000056281	0	0	0	0	0	0
ENSMUSG00000089499	0	0	0	0	0	0
ENSMUSG00000106897	0	0	0	0	0	0
ENSMUSG00000106893	0	0	1	0	0	0
ENSMUSG00000106891	40	29	45	21	17	20
ENSMUSG00000022996	18	6	33	4	6	4
ENSMUSG00000022997	0	0	0	0	0	0
ENSMUSG00000011008	13	24	12	19	2	18
ENSMUSG00000106899	0	0	0	0	0	0
ENSMUSG00000104490	0	0	0	0	0	0
ENSMUSG00000104494	0	0	0	0	0	0
ENSMUSG00000104498	3	3	4	3	4	4
ENSMUSG00000104499	0	0	0	0	0	0
ENSMUSG00000076050	0	0	0	2	0	0
ENSMUSG00000114313	0	0	0	0	1	0
ENSMUSG00000114311	0	0	0	0	0	0
ENSMUSG00000114317	1	0	0	0	0	0
ENSMUSG00000114314	0	0	0	0	0	0
ENSMUSG00000114318	0	0	0	0	0	0
ENSMUSG00000114319	0	0	0	0	0	0
ENSMUSG00000025420	3	4	0	0	4	2
ENSMUSG00000025427	16	17	14	10	9	5
ENSMUSG00000025425	3	0	8	8	8	0
ENSMUSG00000054293	76	69	66	43	25	44
ENSMUSG00000097754	0	0	0	0	0	0
ENSMUSG00000054746	0	0	0	0	0	0
ENSMUSG00000091752	0	0	0	0	0	0

ENSMUSG00000091756	4	2	6	3	5	3
ENSMUSG00000091757	0	0	0	0	0	0
ENSMUSG00000091754	45	38	38	16	28	31
ENSMUSG00000111431	3	0	0	0	0	0
ENSMUSG00000050134	0	0	0	2	0	0
ENSMUSG00000044313	5	3	4	0	0	0
ENSMUSG00000055159	4	4	0	0	0	0
ENSMUSG00000058670	0	0	0	0	0	0
ENSMUSG00000110542	2	5	5	0	0	0
ENSMUSG00000110541	2	0	0	0	0	0
ENSMUSG00000110544	0	0	0	0	0	0
ENSMUSG00000095934	0	0	0	0	0	0
ENSMUSG00000060470	72	79	134	63	43	53
ENSMUSG00000100258	0	0	0	0	0	0
ENSMUSG00000026196	63	76	76	40	54	93
ENSMUSG00000096405	0	0	0	0	0	0
ENSMUSG00000096402	0	0	0	0	0	0
ENSMUSG00000114943	9	10	24	5	9	9
ENSMUSG00000096409	0	0	0	0	0	0
ENSMUSG00000080451	3	2	2	0	0	0
ENSMUSG00000080459	1	0	0	0	0	0
ENSMUSG00000095279	0	0	0	0	0	0
ENSMUSG00000045048	7	7	7	2	2	6
ENSMUSG00000095270	0	0	0	0	0	0
ENSMUSG00000095273	0	0	0	0	0	0
ENSMUSG00000095272	3	9	4	1	2	2
ENSMUSG00000095275	0	0	0	0	0	0
ENSMUSG00000095274	0	0	0	0	0	0
ENSMUSG00000095277	0	2	0	0	1	0
ENSMUSG00000095276	0	0	0	0	0	0
ENSMUSG00000098412	6	8	4	8	8	5
ENSMUSG00000094588	0	0	0	0	0	0
ENSMUSG00000094581	0	0	0	0	0	0
ENSMUSG00000094584	0	0	0	0	0	0
ENSMUSG00000094585	0	0	0	0	0	0
ENSMUSG00000094586	0	0	0	0	0	0
ENSMUSG00000094587	0	0	0	0	0	0
ENSMUSG00000098414	0	1	0	0	0	2
ENSMUSG00000095483	0	0	0	0	0	0
ENSMUSG00000045591	0	0	0	0	0	0
ENSMUSG00000095187	0	0	0	0	0	2
ENSMUSG00000095185	0	0	0	0	0	0
ENSMUSG00000095189	0	0	0	0	0	0
ENSMUSG00000095188	0	0	0	0	0	0
ENSMUSG00000047686	40	16	19	22	36	32
ENSMUSG00000042064	2	0	0	0	3	0
ENSMUSG00000086858	0	0	0	0	0	0
ENSMUSG00000086854	0	0	0	0	0	0
ENSMUSG00000086856	0	0	0	0	0	0
ENSMUSG00000081498	0	0	0	0	0	0
ENSMUSG00000081494	19	14	16	18	20	17
ENSMUSG00000081495	0	0	0	0	0	0
ENSMUSG00000081490	0	0	0	0	0	0
ENSMUSG00000048752	0	0	0	0	0	0
ENSMUSG00000077608	0	0	0	0	0	0
ENSMUSG00000077609	0	0	0	0	0	0
ENSMUSG00000077601	0	0	0	0	0	0
ENSMUSG00000077603	0	0	0	0	0	0

ENSMUSG00000094632	0	1	0	0	0	0
ENSMUSG00000094630	0	0	0	0	0	0
ENSMUSG00000094631	0	0	0	0	0	0
ENSMUSG00000094636	0	0	0	0	0	0
ENSMUSG00000094637	0	0	0	0	0	2
ENSMUSG00000094635	0	0	0	0	0	0
ENSMUSG00000094639	0	0	0	0	0	0
ENSMUSG00000095713	0	0	0	0	0	0
ENSMUSG00000026981	6	6	9	2	6	8
ENSMUSG00000095717	0	0	0	0	0	0
ENSMUSG00000048284	7	15	20	4	12	6
ENSMUSG00000099749	0	0	0	0	0	0
ENSMUSG00000099748	0	0	0	0	0	0
ENSMUSG00000099745	0	0	0	0	0	0
ENSMUSG00000099744	0	0	0	0	0	0
ENSMUSG00000099747	0	0	0	0	0	0
ENSMUSG00000099740	0	0	0	0	0	0
ENSMUSG00000099742	0	0	0	0	0	0
ENSMUSG00000046764	0	0	0	0	0	0
ENSMUSG00000007653	0	2	7	0	8	15
ENSMUSG00000104296	0	0	0	0	0	0
ENSMUSG00000104297	0	0	0	0	0	0
ENSMUSG00000108575	0	0	0	0	0	0
ENSMUSG00000111269	0	0	0	0	0	0
ENSMUSG00000108589	0	0	0	0	0	0
ENSMUSG00000086142	0	0	0	0	0	0
ENSMUSG00000086141	0	0	0	0	0	0
ENSMUSG00000088447	0	0	0	0	0	0
ENSMUSG00000104298	0	0	1	0	2	0
ENSMUSG00000086144	0	0	0	0	0	0
ENSMUSG00000049154	0	0	0	0	0	0
ENSMUSG00000098838	0	0	2	1	0	0
ENSMUSG00000027350	6	0	8	14	0	4
ENSMUSG00000049152	0	0	0	0	0	0
ENSMUSG00000098834	0	0	0	0	0	0
ENSMUSG00000098832	1	0	0	0	0	0
ENSMUSG00000098833	0	0	0	0	0	0
ENSMUSG00000087431	4	4	0	3	0	6
ENSMUSG00000087432	0	0	0	0	0	0
ENSMUSG00000087434	0	0	0	0	0	0
ENSMUSG00000076269	0	1	4	1	2	3
ENSMUSG00000083472	0	0	0	0	0	0
ENSMUSG00000083539	0	4	0	1	0	0
ENSMUSG00000083535	0	0	0	0	0	0
ENSMUSG00000083534	6	6	6	0	8	0
ENSMUSG00000083537	0	0	0	0	0	0
ENSMUSG00000083530	0	0	0	0	0	0
ENSMUSG00000083533	0	2	0	0	0	0
ENSMUSG00000103492	17	31	40	24	26	30
ENSMUSG00000031837	0	0	0	0	0	0
ENSMUSG00000031833	219	189	264	117	119	168
ENSMUSG00000094049	0	0	0	0	0	0
ENSMUSG00000020713	0	0	0	0	0	0
ENSMUSG00000050901	0	0	0	0	0	0
ENSMUSG00000106465	0	0	0	0	0	0
ENSMUSG00000083390	2	0	0	0	0	1
ENSMUSG00000083395	0	0	0	0	0	0
ENSMUSG00000084169	0	0	0	0	0	0

ENSMUSG00000083399	0	0	0	0	0	0
ENSMUSG00000083398	0	0	0	0	0	0
ENSMUSG00000108582	0	0	0	0	0	0
ENSMUSG00000084164	1	1	0	1	0	1
ENSMUSG00000084163	0	0	0	0	0	0
ENSMUSG00000084162	0	0	0	0	0	0
ENSMUSG00000084161	0	0	0	0	0	0
ENSMUSG00000084160	0	0	0	0	0	0
ENSMUSG00000106461	0	5	0	0	0	0
ENSMUSG00000106460	0	0	0	0	0	0
ENSMUSG00000022604	639	646	535	586	585	689
ENSMUSG00000080849	0	0	0	6	0	0
ENSMUSG00000080846	0	0	0	0	0	0
ENSMUSG00000080847	0	0	0	0	0	0
ENSMUSG00000080840	4	4	7	2	3	7
ENSMUSG00000080841	0	1	0	0	0	0
ENSMUSG00000080842	0	0	0	0	1	0
ENSMUSG00000018868	0	0	0	0	0	0
ENSMUSG00000113723	0	0	0	0	0	0
ENSMUSG00000088954	0	0	0	0	0	0
ENSMUSG00000078768	50	72	62	56	67	77
ENSMUSG00000113241	0	0	0	0	0	0
ENSMUSG00000044055	0	7	0	0	0	0
ENSMUSG00000044058	0	0	0	0	0	0
ENSMUSG00000078490	60	64	30	49	42	62
ENSMUSG00000032021	4	0	3	0	0	2
ENSMUSG00000078494	0	2	0	2	0	0
ENSMUSG00000033998	9	11	0	2	10	10
ENSMUSG00000062649	0	0	0	0	0	0
ENSMUSG00000103631	0	0	0	0	0	0
ENSMUSG00000065143	0	0	0	0	0	0
ENSMUSG00000103630	15	23	9	20	18	24
ENSMUSG00000111951	12	10	21	24	17	14
ENSMUSG00000103635	0	0	6	2	2	0
ENSMUSG00000081827	0	0	0	0	0	0
ENSMUSG00000081821	0	0	0	0	0	0
ENSMUSG00000081820	2	0	0	0	0	0
ENSMUSG00000081823	0	0	0	0	0	0
ENSMUSG00000013974	0	9	7	0	0	0
ENSMUSG00000101117	0	0	0	0	0	0
ENSMUSG00000006538	0	4	0	4	0	4
ENSMUSG00000063594	121	61	88	1	12	4
ENSMUSG00000101115	0	0	0	0	0	0
ENSMUSG00000114429	0	0	0	0	0	0
ENSMUSG00000032257	0	0	0	0	0	0
ENSMUSG00000111478	0	0	1	0	0	0
ENSMUSG00000111479	0	0	0	0	0	0
ENSMUSG00000098069	0	0	2	0	0	0
ENSMUSG00000098060	0	0	0	0	0	0
ENSMUSG00000098065	0	1	0	0	3	2
ENSMUSG00000098064	0	0	0	0	0	0
ENSMUSG00000107409	0	0	0	0	0	0
ENSMUSG00000033342	0	0	0	0	0	0
ENSMUSG00000107403	0	0	0	0	0	0
ENSMUSG00000071204	0	0	0	0	0	0
ENSMUSG00000098213	0	0	0	0	0	0
ENSMUSG00000071203	139	174	203	50	87	142
ENSMUSG00000095369	0	0	0	0	0	0

ENSMUSG00000107404	0	0	0	0	0	0
ENSMUSG00000086909	0	0	0	0	0	0
ENSMUSG00000077558	0	0	0	0	0	0
ENSMUSG00000086908	0	0	0	0	0	0
ENSMUSG00000084959	0	0	0	0	0	0
ENSMUSG00000084956	0	0	0	0	0	0
ENSMUSG00000084955	0	0	0	0	0	0
ENSMUSG00000086907	0	0	0	0	0	0
ENSMUSG00000082318	2	0	0	1	2	0
ENSMUSG00000111346	0	0	0	2	0	0
ENSMUSG00000107632	1	2	2	2	2	3
ENSMUSG00000091511	0	0	0	0	0	0
ENSMUSG00000091519	0	0	0	0	0	0
ENSMUSG00000095660	0	0	0	0	0	0
ENSMUSG00000030310	0	0	0	0	0	3
ENSMUSG00000096857	0	0	0	1	0	0
ENSMUSG00000025418	0	0	0	0	0	0
ENSMUSG00000101482	0	1	3	2	0	1
ENSMUSG00000101483	0	0	0	0	0	0
ENSMUSG00000087374	0	0	0	0	0	0
ENSMUSG00000087373	0	0	0	0	0	0
ENSMUSG00000087371	7	0	0	5	5	12
ENSMUSG00000087378	0	0	0	0	0	0
ENSMUSG00000101739	0	0	0	0	0	0
ENSMUSG00000101736	0	0	0	0	0	0
ENSMUSG00000101737	0	0	0	0	0	0
ENSMUSG00000101734	0	0	0	0	0	0
ENSMUSG00000101735	0	0	0	0	0	0
ENSMUSG00000101732	0	0	0	0	0	0
ENSMUSG00000101733	0	0	0	0	0	0
ENSMUSG00000101731	13	9	12	27	12	12
ENSMUSG00000100289	0	0	0	0	0	0
ENSMUSG00000100288	0	0	0	0	0	0
ENSMUSG00000100283	0	0	0	1	0	0
ENSMUSG00000100282	0	0	0	0	0	0
ENSMUSG00000100285	1	0	0	0	0	0
ENSMUSG00000100284	0	0	0	0	0	0
ENSMUSG00000100286	0	0	0	0	0	0
ENSMUSG00000088499	0	0	0	0	1	0
ENSMUSG00000088498	0	0	0	0	0	0
ENSMUSG00000088497	0	0	0	0	0	0
ENSMUSG00000088495	0	0	0	0	0	0
ENSMUSG00000088491	0	2	1	2	0	0
ENSMUSG00000109981	0	0	0	0	0	0
ENSMUSG00000109980	2	0	0	0	0	0
ENSMUSG00000109983	0	0	0	0	0	0
ENSMUSG00000064377	0	0	0	1	0	0
ENSMUSG00000059887	0	0	0	0	0	3
ENSMUSG00000107888	0	0	0	0	0	0
ENSMUSG00000107889	0	0	0	0	0	0
ENSMUSG00000109985	0	0	0	0	0	0
ENSMUSG00000107885	0	0	0	0	0	0
ENSMUSG00000107882	0	0	0	0	0	0
ENSMUSG00000107883	0	2	0	0	0	2
ENSMUSG00000107880	0	0	0	0	0	0
ENSMUSG00000051582	0	2	0	0	0	0
ENSMUSG00000023387	0	0	0	0	0	0
ENSMUSG00000051297	0	0	0	0	0	0

ENSMUSG00000107771	0	0	0	0	0	0
ENSMUSG00000107772	0	0	0	0	0	0
ENSMUSG00000107773	0	0	0	0	0	0
ENSMUSG00000107774	0	0	0	0	0	0
ENSMUSG00000107776	0	0	0	0	0	0
ENSMUSG00000107777	0	0	0	0	0	0
ENSMUSG00000103648	0	0	0	0	0	0
ENSMUSG00000111968	2	0	0	0	0	0
ENSMUSG00000111969	2	0	0	4	14	5
ENSMUSG00000087123	0	0	0	0	0	0
ENSMUSG00000103640	2	0	0	0	0	0
ENSMUSG00000103641	0	0	0	0	0	0
ENSMUSG00000111960	0	0	0	0	0	0
ENSMUSG00000111961	0	0	0	0	0	0
ENSMUSG00000103644	0	0	0	0	0	0
ENSMUSG00000111967	0	0	0	0	1	0
ENSMUSG00000103647	0	0	0	0	0	0
ENSMUSG00000087121	4	2	5	0	0	0
ENSMUSG00000050368	0	5	0	0	2	0
ENSMUSG00000065427	0	0	0	0	0	1
ENSMUSG00000065426	1	0	1	1	0	0
ENSMUSG00000065425	0	0	0	0	1	0
ENSMUSG00000082332	0	0	0	0	0	0
ENSMUSG00000058820	0	0	0	0	0	0
ENSMUSG00000094030	1	1	2	3	1	1
ENSMUSG00000094032	0	0	0	0	0	0
ENSMUSG00000094033	0	1	0	0	0	1
ENSMUSG00000094034	0	0	0	0	0	0
ENSMUSG00000022650	0	0	0	0	0	0
ENSMUSG00000094036	0	0	0	0	0	0
ENSMUSG00000022652	0	0	0	0	0	0
ENSMUSG00000094038	0	0	0	0	0	0
ENSMUSG00000094039	0	0	0	0	0	1
ENSMUSG00000022659	6	15	22	0	4	6
ENSMUSG00000089727	0	0	3	0	0	0
ENSMUSG00000089724	0	0	0	0	0	0
ENSMUSG00000052974	0	0	0	0	0	0
ENSMUSG00000039201	134	121	153	125	116	151
ENSMUSG00000028716	8	0	3	0	0	5
ENSMUSG00000028715	0	0	0	0	5	0
ENSMUSG00000028718	36	34	43	10	15	22
ENSMUSG00000097671	0	0	0	0	0	0
ENSMUSG00000112438	0	0	0	0	0	0
ENSMUSG00000112433	29	51	72	27	36	25
ENSMUSG00000112432	0	0	0	0	0	0
ENSMUSG00000112435	0	2	0	0	0	0
ENSMUSG00000112434	0	0	0	0	0	0
ENSMUSG00000112436	0	0	0	0	0	0
ENSMUSG00000102115	0	0	0	0	0	0
ENSMUSG00000102119	0	0	0	0	0	0
ENSMUSG00000102118	0	0	0	0	0	0
ENSMUSG00000114873	0	0	0	0	0	0
ENSMUSG00000114872	0	0	0	0	0	0
ENSMUSG00000114870	0	0	0	0	0	0
ENSMUSG00000114877	0	0	0	0	0	0
ENSMUSG00000114876	0	0	0	0	0	0
ENSMUSG00000114875	9	6	6	0	0	7
ENSMUSG00000114874	0	0	0	0	0	0

ENSMUSG00000114879	0	0	0	0	0	0
ENSMUSG00000114878	0	0	0	2	2	0
ENSMUSG00000038128	115	84	112	38	54	67
ENSMUSG00000110362	0	0	0	0	0	0
ENSMUSG00000110361	3	0	0	0	1	0
ENSMUSG00000110367	0	0	0	0	0	0
ENSMUSG00000110366	0	2	0	0	0	2
ENSMUSG00000110365	0	0	0	0	0	0
ENSMUSG00000110364	0	0	0	0	0	0
ENSMUSG00000110368	0	0	0	0	0	0
ENSMUSG00000109039	0	0	0	0	0	0
ENSMUSG00000035067	149	178	101	28	58	34
ENSMUSG00000109034	0	0	0	0	0	0
ENSMUSG00000109030	0	2	0	1	0	0
ENSMUSG00000109033	0	0	0	0	0	0
ENSMUSG00000109032	4	7	15	2	5	2
ENSMUSG00000029044	0	0	0	0	0	0
ENSMUSG00000054409	5	0	0	0	0	2
ENSMUSG00000112922	144	136	128	139	141	182
ENSMUSG00000112921	0	0	0	0	0	0
ENSMUSG00000106913	3	0	0	0	0	0
ENSMUSG00000066197	0	0	0	0	0	0
ENSMUSG00000108581	0	0	0	2	2	0
ENSMUSG00000106916	0	0	0	0	0	0
ENSMUSG00000106915	0	3	3	0	3	0
ENSMUSG00000066191	180	134	196	96	85	126
ENSMUSG00000095970	0	0	0	0	0	0
ENSMUSG00000088935	0	0	0	0	0	0
ENSMUSG00000088937	0	0	0	0	0	0
ENSMUSG00000088930	0	0	0	0	0	0
ENSMUSG00000088933	0	0	0	0	0	0
ENSMUSG00000077465	0	0	0	0	0	0
ENSMUSG00000088939	0	0	0	0	0	0
ENSMUSG00000021678	60	51	117	18	36	34
ENSMUSG00000021679	0	0	0	0	0	0
ENSMUSG00000096308	0	0	0	0	0	0
ENSMUSG00000109690	0	0	0	0	0	0
ENSMUSG00000109692	0	0	0	0	0	0
ENSMUSG00000109694	0	0	0	0	0	0
ENSMUSG00000109696	60	62	44	65	68	98
ENSMUSG00000109699	0	0	0	0	0	0
ENSMUSG00000096301	0	0	0	0	0	0
ENSMUSG00000096302	0	0	0	0	0	0
ENSMUSG00000096303	0	0	0	0	0	0
ENSMUSG00000096304	0	0	0	0	0	0
ENSMUSG00000096305	0	0	0	0	0	0
ENSMUSG00000096306	0	0	0	0	0	0
ENSMUSG00000114297	0	1	0	0	0	0
ENSMUSG00000114296	0	0	0	0	0	0
ENSMUSG00000114295	0	0	0	0	0	0
ENSMUSG00000114294	0	2	0	0	0	0
ENSMUSG00000114290	0	0	0	0	0	0
ENSMUSG00000082932	0	0	0	0	0	2
ENSMUSG00000082933	0	0	0	0	0	0
ENSMUSG00000082937	0	0	0	0	0	0
ENSMUSG00000114299	0	0	3	0	0	0
ENSMUSG00000110339	0	0	0	0	0	0
ENSMUSG00000005410	163	167	214	136	124	233

ENSMUSG00000113473	12	8	9	6	10	5
ENSMUSG00000113470	0	0	0	0	0	0
ENSMUSG00000108348	5	0	3	0	3	3
ENSMUSG00000113477	0	0	0	0	0	0
ENSMUSG00000113474	0	0	0	0	0	0
ENSMUSG00000108344	0	0	0	0	0	0
ENSMUSG00000108345	0	0	0	0	0	0
ENSMUSG00000108346	0	0	0	0	1	0
ENSMUSG00000113479	0	0	0	0	0	0
ENSMUSG00000108340	0	0	0	0	0	0
ENSMUSG00000108342	0	0	0	0	0	0
ENSMUSG00000108343	0	0	0	0	0	0
ENSMUSG00000110334	0	0	0	0	0	0
ENSMUSG00000059060	31	41	22	36	10	31
ENSMUSG00000109969	0	0	0	0	0	0
ENSMUSG00000109968	0	0	0	0	0	0
ENSMUSG00000109962	0	0	0	0	0	0
ENSMUSG00000109961	0	0	0	0	0	0
ENSMUSG00000093870	0	0	0	0	0	0
ENSMUSG00000111183	0	0	0	0	0	0
ENSMUSG00000114121	0	0	0	0	0	0
ENSMUSG00000114120	0	0	0	0	0	0
ENSMUSG00000114123	3	1	3	2	2	1
ENSMUSG00000114122	0	0	0	0	0	0
ENSMUSG00000110332	0	5	0	0	0	0
ENSMUSG00000114127	3	1	2	3	0	1
ENSMUSG00000114126	0	0	0	0	0	0
ENSMUSG00000114129	1	2	2	1	1	1
ENSMUSG00000114128	0	0	0	0	0	0
ENSMUSG00000110333	0	0	0	0	0	0
ENSMUSG00000038151	304	271	206	94	81	98
ENSMUSG00000035528	0	4	0	0	0	0
ENSMUSG00000104514	16	11	10	10	14	15
ENSMUSG00000104516	0	0	0	0	0	0
ENSMUSG00000104511	0	0	0	0	0	0
ENSMUSG00000104510	0	0	0	0	0	0
ENSMUSG00000104513	0	0	0	0	0	0
ENSMUSG00000104518	0	0	0	0	0	0
ENSMUSG00000025652	1	0	1	0	0	1
ENSMUSG00000091657	0	0	0	0	0	0
ENSMUSG00000091656	0	0	0	0	0	0
ENSMUSG00000091652	0	0	0	0	0	0
ENSMUSG00000091651	0	0	0	0	0	0
ENSMUSG00000091650	0	0	0	0	0	0
ENSMUSG00000091659	0	0	0	0	0	0
ENSMUSG00000096846	0	0	0	0	0	0
ENSMUSG00000067716	0	0	0	0	0	0
ENSMUSG00000067714	12	0	4	4	8	0
ENSMUSG00000010142	65	51	59	10	6	25
ENSMUSG00000032303	0	0	0	0	0	0
ENSMUSG00000044294	0	0	0	0	0	0
ENSMUSG00000044296	78	102	49	57	75	92
ENSMUSG00000044293	0	0	0	0	0	0
ENSMUSG00000112983	0	0	0	0	0	5
ENSMUSG00000112982	0	0	0	0	0	0
ENSMUSG00000112984	0	0	0	0	0	0
ENSMUSG00000112987	0	0	0	0	0	0
ENSMUSG00000112986	2	2	0	2	0	3

ENSMUSG00000112989	0	0	0	0	0	0
ENSMUSG00000112988	0	0	0	0	0	0
ENSMUSG00000100140	0	0	0	0	0	0
ENSMUSG00000098219	0	0	2	4	0	4
ENSMUSG00000112794	6	8	2	15	7	7
ENSMUSG00000027635	95	70	118	49	55	95
ENSMUSG00000003974	0	0	0	0	0	0
ENSMUSG00000112790	65	83	81	75	76	80
ENSMUSG00000112791	0	0	0	0	1	0
ENSMUSG00000095028	10	5	21	0	4	13
ENSMUSG00000090665	6	3	4	0	3	3
ENSMUSG00000112792	0	3	1	0	0	0
ENSMUSG00000095021	0	0	0	0	0	0
ENSMUSG00000095022	0	0	0	0	0	0
ENSMUSG00000045655	0	0	0	0	0	0
ENSMUSG00000095025	0	1	0	0	0	0
ENSMUSG00000095026	0	0	0	0	0	0
ENSMUSG00000095027	0	0	0	0	0	0
ENSMUSG00000110401	0	0	0	0	0	0
ENSMUSG00000090660	0	0	0	0	0	0
ENSMUSG00000053161	0	0	0	0	0	0
ENSMUSG00000053166	21	30	17	64	75	80
ENSMUSG00000053168	0	0	0	0	5	6
ENSMUSG00000080394	0	0	0	0	0	0
ENSMUSG00000046826	3	0	16	2	4	2
ENSMUSG00000082913	0	0	2	0	0	0
ENSMUSG00000050232	22	10	25	8	4	4
ENSMUSG00000114824	0	0	0	0	0	0
ENSMUSG00000110408	0	0	6	0	0	1
ENSMUSG00000114825	0	0	0	0	0	0
ENSMUSG00000096518	0	0	0	0	0	0
ENSMUSG00000110409	5	1	3	0	0	0
ENSMUSG00000096997	0	0	0	0	0	0
ENSMUSG00000090016	0	0	0	0	0	0
ENSMUSG00000098268	0	0	1	0	1	1
ENSMUSG00000090012	0	0	0	0	0	0
ENSMUSG00000090013	0	0	0	0	0	0
ENSMUSG00000098261	0	0	0	0	0	0
ENSMUSG00000098262	0	0	0	0	0	0
ENSMUSG00000098264	0	0	0	0	0	0
ENSMUSG00000098266	0	0	0	0	0	0
ENSMUSG00000114823	0	0	0	0	6	0
ENSMUSG00000053783	0	0	0	0	0	0
ENSMUSG00000114794	0	0	0	0	0	0
ENSMUSG00000098878	0	0	0	0	0	0
ENSMUSG00000094775	0	0	0	0	0	0
ENSMUSG00000094773	0	0	0	0	0	0
ENSMUSG00000094771	0	0	0	0	0	0
ENSMUSG00000094778	1	0	0	0	0	0
ENSMUSG00000047728	0	0	0	0	0	0
ENSMUSG00000047720	0	0	0	0	0	0
ENSMUSG00000055780	12	0	0	0	0	0
ENSMUSG00000055789	0	0	0	0	0	0
ENSMUSG00000093096	0	0	0	0	0	0
ENSMUSG00000114799	0	0	0	0	0	0
ENSMUSG00000093093	0	0	0	0	0	0
ENSMUSG00000093091	3	0	2	0	2	3
ENSMUSG00000093090	0	0	0	0	0	0

ENSMUSG00000076874	0	1	0	0	0	0
ENSMUSG00000052785	2	0	0	0	0	0
ENSMUSG00000094954	0	0	0	0	0	0
ENSMUSG00000081510	0	0	0	0	0	0
ENSMUSG00000081513	0	0	0	0	0	0
ENSMUSG00000081515	0	0	0	0	0	0
ENSMUSG00000081517	0	0	0	0	0	0
ENSMUSG00000073998	2	0	0	0	0	0
ENSMUSG00000077473	0	0	0	0	0	0
ENSMUSG00000077472	0	0	0	0	0	0
ENSMUSG00000077471	0	0	0	0	0	0
ENSMUSG00000073997	3	2	0	2	4	2
ENSMUSG00000077477	0	0	0	0	0	0
ENSMUSG00000077474	0	0	0	0	0	0
ENSMUSG00000055254	27	22	34	12	28	27
ENSMUSG00000113447	0	0	0	0	0	0
ENSMUSG00000094284	0	0	0	0	0	0
ENSMUSG00000030156	32	6	22	2	8	3
ENSMUSG00000092328	0	0	0	0	0	0
ENSMUSG00000092326	0	0	0	0	0	0
ENSMUSG00000092322	0	0	0	3	0	0
ENSMUSG00000043727	0	0	9	2	8	10
ENSMUSG00000082098	0	0	0	0	0	0
ENSMUSG00000082095	1	1	0	0	4	0
ENSMUSG00000082091	0	0	0	0	0	0
ENSMUSG00000082090	0	0	0	2	0	0
ENSMUSG00000082093	6	1	4	0	0	0
ENSMUSG00000082092	0	0	0	0	0	0
ENSMUSG00000032796	0	0	0	0	5	5
ENSMUSG00000071591	0	8	0	0	0	0
ENSMUSG00000043892	0	0	0	0	0	0
ENSMUSG00000109394	12	15	20	19	11	27
ENSMUSG00000068876	23	8	5	4	0	4
ENSMUSG00000087609	0	0	0	0	0	0
ENSMUSG00000087608	0	0	0	0	0	0
ENSMUSG00000068879	0	0	0	0	0	0
ENSMUSG00000087604	0	0	6	0	0	6
ENSMUSG00000087603	0	0	0	0	0	0
ENSMUSG00000087602	0	0	0	0	0	0
ENSMUSG00000070645	0	0	0	0	3	0
ENSMUSG00000082587	1	3	0	1	0	2
ENSMUSG00000084558	1	1	0	0	0	0
ENSMUSG00000084559	1	0	1	1	1	1
ENSMUSG00000037315	186	148	116	112	137	210
ENSMUSG00000084550	0	0	0	0	0	0
ENSMUSG00000084551	0	0	0	0	0	0
ENSMUSG00000084555	0	0	0	0	0	0
ENSMUSG00000023940	38	60	50	31	23	39
ENSMUSG00000085608	0	4	1	2	1	5
ENSMUSG00000085600	0	0	0	0	0	0
ENSMUSG00000107574	0	0	0	0	0	0
ENSMUSG00000107575	0	0	0	0	0	0
ENSMUSG00000107572	0	0	0	0	0	0
ENSMUSG00000107573	0	0	0	0	0	0
ENSMUSG00000084782	4	0	3	6	4	8
ENSMUSG00000109297	0	0	1	0	0	0
ENSMUSG00000101801	0	2	0	0	0	0
ENSMUSG00000061524	0	0	0	0	0	0

ENSMUSG00000101803	0	0	0	0	0	0
ENSMUSG00000101804	0	0	0	0	0	0
ENSMUSG00000101805	0	0	0	0	0	0
ENSMUSG00000061520	2	2	0	1	0	0
ENSMUSG00000101807	0	0	0	0	0	0
ENSMUSG00000101808	0	0	0	0	0	0
ENSMUSG00000101809	0	0	0	0	0	0
ENSMUSG00000107490	0	0	0	0	0	0
ENSMUSG00000067596	0	0	0	0	0	0
ENSMUSG00000083173	0	0	2	0	0	0
ENSMUSG00000001120	77	48	91	50	69	64
ENSMUSG00000074345	11	22	21	8	12	10
ENSMUSG00000107492	0	0	0	0	0	0
ENSMUSG00000074342	46	49	61	19	25	52
ENSMUSG00000074343	0	0	0	0	0	0
ENSMUSG00000009941	0	0	0	0	0	3
ENSMUSG00000067599	0	10	10	0	0	0
ENSMUSG00000075968	0	0	0	0	0	0
ENSMUSG00000073699	0	0	0	0	0	0
ENSMUSG00000068122	0	0	0	0	0	2
ENSMUSG00000068129	4	15	9	12	15	15
ENSMUSG00000076876	0	0	0	0	0	0
ENSMUSG00000100192	0	0	0	0	0	0
ENSMUSG00000078511	0	0	0	0	0	0
ENSMUSG00000078510	0	0	0	0	0	0
ENSMUSG00000078513	0	0	0	0	0	0
ENSMUSG00000078512	0	0	0	0	0	0
ENSMUSG00000078519	12	12	15	9	0	13
ENSMUSG00000078518	0	0	0	0	0	0
ENSMUSG00000089608	0	0	0	0	0	0
ENSMUSG00000101283	0	0	0	0	0	0
ENSMUSG00000089605	0	0	0	0	2	0
ENSMUSG00000089607	0	0	0	0	0	0
ENSMUSG00000089606	0	0	0	0	0	0
ENSMUSG00000089601	1	0	0	0	0	0
ENSMUSG00000089603	0	0	0	0	0	0
ENSMUSG00000035262	6	4	0	8	11	20
ENSMUSG00000091539	0	1	1	2	0	1
ENSMUSG00000102056	0	0	0	0	0	0
ENSMUSG00000102057	0	0	0	0	0	0
ENSMUSG00000102054	0	0	0	0	0	0
ENSMUSG00000102055	9	8	6	3	0	5
ENSMUSG00000102052	0	0	0	0	0	0
ENSMUSG00000102053	0	0	0	0	0	0
ENSMUSG00000102050	0	0	0	0	0	0
ENSMUSG00000102051	0	0	0	0	0	0
ENSMUSG00000102058	0	0	0	0	0	0
ENSMUSG00000103989	0	0	0	0	0	0
ENSMUSG00000048826	64	37	54	24	46	38
ENSMUSG00000103982	0	0	0	0	0	0
ENSMUSG00000103981	2	2	1	3	3	2
ENSMUSG00000103980	0	0	0	0	0	0
ENSMUSG00000103987	0	0	0	0	0	0
ENSMUSG00000088021	0	0	0	0	0	0
ENSMUSG00000073528	2	0	0	1	0	0
ENSMUSG00000069456	0	0	0	0	0	0
ENSMUSG00000096147	0	1	2	0	0	0
ENSMUSG00000096144	0	0	0	0	7	0

ENSMUSG00000096142	1	0	1	1	0	0
ENSMUSG00000102087	0	0	0	0	0	0
ENSMUSG00000096140	0	0	3	0	0	0
ENSMUSG00000096141	10	8	4	9	9	19
ENSMUSG00000096148	0	0	0	0	0	0
ENSMUSG00000038453	14	10	4	6	18	4
ENSMUSG00000034139	0	0	0	0	0	0
ENSMUSG00000063386	0	0	0	0	0	0
ENSMUSG00000069986	4	1	6	3	2	0
ENSMUSG00000063383	84	102	114	69	101	109
ENSMUSG00000109936	15	9	9	8	6	13
ENSMUSG00000093825	0	0	0	0	0	0
ENSMUSG00000088025	0	0	0	0	0	0
ENSMUSG00000093823	0	0	0	0	0	0
ENSMUSG00000111173	0	0	0	0	0	0
ENSMUSG00000091494	0	0	0	0	0	0
ENSMUSG00000105912	0	0	4	0	0	0
ENSMUSG00000057068	0	0	0	0	0	0
ENSMUSG00000105916	0	0	0	0	0	0
ENSMUSG00000105917	0	0	0	0	0	0
ENSMUSG00000105914	0	0	0	0	0	0
ENSMUSG00000057067	3	0	0	0	0	0
ENSMUSG00000020886	175	107	106	33	73	69
ENSMUSG00000020884	0	0	0	0	0	0
ENSMUSG00000082630	0	4	0	0	5	2
ENSMUSG00000104069	2	1	0	1	4	4
ENSMUSG00000104068	0	0	2	2	2	0
ENSMUSG00000104065	0	0	0	0	0	0
ENSMUSG00000104067	0	0	0	0	0	0
ENSMUSG00000104062	0	0	0	0	0	0
ENSMUSG00000059459	0	0	0	0	0	2
ENSMUSG00000059455	0	0	0	0	0	0
ENSMUSG00000088295	0	2	0	0	0	0
ENSMUSG00000107553	0	0	0	0	0	0
ENSMUSG00000015405	75	71	101	48	69	79
ENSMUSG00000015401	0	4	0	0	0	0
ENSMUSG00000108899	2	0	1	0	2	4
ENSMUSG00000108890	0	0	0	0	0	0
ENSMUSG00000108891	0	0	0	0	0	0
ENSMUSG00000108895	2	9	2	0	0	2
ENSMUSG00000108896	0	0	0	0	0	0
ENSMUSG00000056332	3	0	0	0	0	0
ENSMUSG00000100246	0	0	0	1	0	0
ENSMUSG00000026831	0	0	0	0	0	0
ENSMUSG00000100368	0	0	0	0	0	0
ENSMUSG00000061958	0	0	0	0	0	0
ENSMUSG00000100366	0	0	0	0	0	0
ENSMUSG00000100367	0	0	2	0	0	0
ENSMUSG00000100364	0	0	0	0	0	0
ENSMUSG00000100365	0	0	0	0	0	0
ENSMUSG00000100362	0	0	0	0	0	0
ENSMUSG00000061952	0	0	0	0	0	0
ENSMUSG00000100360	0	0	0	0	0	0
ENSMUSG00000100361	0	0	0	0	0	0
ENSMUSG00000110911	0	0	0	0	0	0
ENSMUSG00000110912	0	0	0	0	0	0
ENSMUSG00000110916	0	0	0	0	0	0
ENSMUSG00000110917	0	0	0	0	0	0

ENSMUSG00000110918	0	0	0	0	0	0
ENSMUSG00000112700	4	8	5	2	11	11
ENSMUSG00000104541	0	0	0	0	0	0
ENSMUSG00000088354	1	0	0	0	1	0
ENSMUSG00000088350	0	0	0	0	0	0
ENSMUSG00000081033	0	0	0	0	0	0
ENSMUSG00000112708	0	0	0	0	0	0
ENSMUSG00000088358	0	0	0	0	0	0
ENSMUSG00000086408	0	2	0	0	0	0
ENSMUSG00000106338	0	0	0	0	0	0
ENSMUSG00000086402	0	0	0	0	0	6
ENSMUSG00000086404	0	0	0	0	0	7
ENSMUSG00000086405	0	0	0	0	0	0
ENSMUSG00000095550	0	0	0	3	0	0
ENSMUSG00000095551	0	0	0	0	0	0
ENSMUSG00000095552	7	9	5	4	4	3
ENSMUSG00000095553	0	0	0	0	0	0
ENSMUSG00000095555	0	1	1	0	1	0
ENSMUSG00000095557	0	0	0	0	0	0
ENSMUSG00000095558	1	1	0	0	0	0
ENSMUSG00000106333	0	0	0	0	0	0
ENSMUSG00000107934	0	0	0	0	0	0
ENSMUSG00000027870	0	0	2	0	0	0
ENSMUSG00000027871	0	3	0	0	5	0
ENSMUSG00000027876	0	0	0	0	0	0
ENSMUSG00000106335	0	0	0	0	0	0
ENSMUSG00000039198	0	12	0	20	32	17
ENSMUSG00000094208	0	0	0	0	0	0
ENSMUSG00000094206	0	0	0	0	0	0
ENSMUSG00000039194	0	0	0	0	0	2
ENSMUSG00000106334	10	9	0	0	0	0
ENSMUSG00000094203	0	0	0	0	0	0
ENSMUSG00000094202	0	0	0	0	0	0
ENSMUSG00000094201	0	0	1	0	0	2
ENSMUSG00000094200	0	2	0	0	0	0
ENSMUSG00000036218	37	24	64	67	89	55
ENSMUSG00000036211	4	2	2	0	0	8
ENSMUSG00000107931	0	0	0	0	0	0
ENSMUSG00000052544	129	194	207	102	139	176
ENSMUSG00000097952	0	0	0	0	0	0
ENSMUSG00000097953	0	6	0	0	0	6
ENSMUSG00000097951	0	0	0	0	0	0
ENSMUSG00000097957	0	0	0	0	0	0
ENSMUSG00000097955	0	0	0	0	0	0
ENSMUSG00000113397	2	0	0	0	0	0
ENSMUSG00000113395	4	1	3	1	1	1
ENSMUSG00000113399	0	0	0	0	0	0
ENSMUSG00000113398	0	4	0	0	0	0
ENSMUSG00000112668	0	0	0	0	0	0
ENSMUSG00000088797	0	0	0	0	0	0
ENSMUSG00000112660	2	4	2	6	0	0
ENSMUSG00000112661	0	0	0	0	0	0
ENSMUSG00000112662	0	0	0	0	0	2
ENSMUSG00000112663	0	0	0	0	0	0
ENSMUSG00000112665	0	0	0	0	0	0
ENSMUSG00000112666	0	0	0	0	0	10
ENSMUSG00000112667	6	4	5	3	4	5
ENSMUSG00000024992	4	0	0	0	5	0

ENSMUSG00000106038	3	0	0	0	0	0
ENSMUSG00000020061	5	0	0	0	6	0
ENSMUSG00000020062	0	0	0	0	0	0
ENSMUSG00000106034	0	0	0	0	0	0
ENSMUSG00000106036	2	0	2	0	0	0
ENSMUSG00000106033	0	0	0	0	0	0
ENSMUSG00000028520	6	0	0	0	0	0
ENSMUSG00000028523	0	0	0	0	0	2
ENSMUSG00000028524	137	108	117	73	74	103
ENSMUSG00000028528	34	31	25	12	16	21
ENSMUSG00000092120	0	0	0	0	0	0
ENSMUSG00000092123	0	0	0	0	0	0
ENSMUSG00000099804	3	4	3	6	7	6
ENSMUSG00000110281	0	0	0	0	0	0
ENSMUSG00000110286	0	0	0	0	0	0
ENSMUSG00000110132	3	3	0	0	0	0
ENSMUSG00000110133	0	0	0	0	0	0
ENSMUSG00000110130	0	0	0	0	0	0
ENSMUSG00000110137	0	0	0	0	0	0
ENSMUSG00000110134	0	0	0	0	0	0
ENSMUSG00000110139	0	1	3	0	1	0
ENSMUSG00000113537	0	0	0	0	0	0
ENSMUSG00000113535	0	6	18	2	6	10
ENSMUSG00000113534	0	0	0	0	0	0
ENSMUSG00000113533	0	0	0	0	0	0
ENSMUSG00000113531	0	0	0	0	0	0
ENSMUSG00000113530	0	0	0	0	0	0
ENSMUSG00000113539	0	0	0	0	0	0
ENSMUSG00000113538	0	0	0	0	0	0
ENSMUSG00000097237	0	0	0	0	0	0
ENSMUSG00000051220	88	76	74	28	57	67
ENSMUSG00000003341	2	0	0	0	0	2
ENSMUSG00000051225	0	0	6	4	10	5
ENSMUSG00000114623	0	4	3	7	0	0
ENSMUSG00000114620	0	0	0	0	0	0
ENSMUSG00000114621	3	0	0	0	6	0
ENSMUSG00000114625	0	0	0	0	0	0
ENSMUSG00000114628	0	0	0	0	0	0
ENSMUSG00000114629	0	0	0	0	0	0
ENSMUSG00000088387	0	0	0	0	0	0
ENSMUSG00000082879	0	0	1	0	0	2
ENSMUSG00000082877	0	0	0	0	0	0
ENSMUSG00000082873	12	4	8	3	12	18
ENSMUSG00000082872	1	0	0	2	0	2
ENSMUSG00000082871	0	2	2	0	0	0
ENSMUSG00000106441	0	0	0	0	0	0
ENSMUSG00000054052	0	0	0	0	0	0
ENSMUSG00000108139	0	2	0	0	0	0
ENSMUSG00000103553	97	100	89	158	77	110
ENSMUSG00000070407	76	80	87	64	70	82
ENSMUSG00000054054	0	0	0	0	0	0
ENSMUSG00000108132	18	10	28	4	19	18
ENSMUSG00000108131	0	0	0	0	0	0
ENSMUSG00000108130	0	0	0	0	0	0
ENSMUSG00000108135	0	0	0	0	2	0
ENSMUSG00000108134	0	0	0	0	0	0
ENSMUSG00000056679	53	36	48	14	10	8
ENSMUSG00000109300	0	0	0	0	0	0

ENSMUSG00000101857	0	0	0	0	0	0
ENSMUSG00000109306	0	0	0	0	0	0
ENSMUSG00000105075	0	0	0	0	0	0
ENSMUSG00000105074	0	0	0	0	0	0
ENSMUSG00000105070	0	0	0	0	0	0
ENSMUSG00000105073	0	0	0	0	0	0
ENSMUSG00000103724	0	0	0	0	0	0
ENSMUSG00000105078	4	8	3	0	6	3
ENSMUSG00000110229	0	0	0	0	0	0
ENSMUSG00000088384	0	0	0	0	0	0
ENSMUSG00000111660	0	0	0	0	0	0
ENSMUSG00000111663	0	0	0	0	0	0
ENSMUSG00000111665	2	0	0	0	0	2
ENSMUSG00000111666	3	0	0	0	0	0
ENSMUSG00000111669	0	0	0	0	0	0
ENSMUSG00000111668	0	0	0	0	0	0
ENSMUSG00000057503	0	0	0	0	0	0
ENSMUSG00000044485	0	0	0	0	0	0
ENSMUSG00000044487	0	0	0	0	0	0
ENSMUSG00000104304	0	0	0	0	0	0
ENSMUSG00000104306	4	0	0	0	0	3
ENSMUSG00000104301	4	0	0	0	0	0
ENSMUSG00000104303	7	8	8	5	8	8
ENSMUSG00000104308	0	0	0	0	0	0
ENSMUSG00000094712	0	0	0	0	0	0
ENSMUSG00000045288	0	0	4	0	6	0
ENSMUSG00000045281	14	19	16	23	24	29
ENSMUSG00000083831	0	0	0	0	0	0
ENSMUSG00000045284	119	226	95	227	223	235
ENSMUSG00000083837	7	10	7	8	1	7
ENSMUSG00000090191	0	0	0	0	0	0
ENSMUSG00000027409	0	5	6	12	11	6
ENSMUSG00000042888	0	0	0	0	0	0
ENSMUSG00000027403	0	0	2	0	0	0
ENSMUSG00000092972	0	0	0	0	0	0
ENSMUSG00000027401	4	0	2	0	0	0
ENSMUSG00000092974	0	0	0	0	0	0
ENSMUSG00000034918	0	0	0	0	0	0
ENSMUSG00000046396	0	0	0	0	0	1
ENSMUSG00000078061	0	0	0	0	0	0
ENSMUSG00000078060	0	0	0	0	0	1
ENSMUSG00000024223	0	0	0	0	0	0
ENSMUSG00000098438	0	0	0	0	0	0
ENSMUSG00000095615	4	11	8	4	0	0
ENSMUSG00000095614	3	0	0	0	0	0
ENSMUSG00000097370	0	0	0	0	0	0
ENSMUSG00000090223	7	24	4	16	0	10
ENSMUSG00000095610	0	0	0	0	0	0
ENSMUSG00000098432	0	0	0	0	0	0
ENSMUSG00000098434	1	2	0	3	0	0
ENSMUSG00000090228	0	0	0	0	0	0
ENSMUSG00000095619	0	0	0	0	0	0
ENSMUSG00000095618	0	0	0	0	0	0
ENSMUSG00000053338	0	0	0	0	2	0
ENSMUSG00000042774	0	0	0	0	0	1
ENSMUSG00000074899	84	135	176	95	94	135
ENSMUSG00000097371	7	9	7	10	9	3
ENSMUSG00000080562	0	0	1	0	0	0

ENSMUSG00000080561	0	0	0	0	0	0
ENSMUSG00000074892	4	2	0	0	0	0
ENSMUSG00000046643	0	2	0	0	0	0
ENSMUSG00000074895	0	0	0	0	0	0
ENSMUSG00000089434	0	0	0	0	0	0
ENSMUSG00000091259	0	0	0	0	0	0
ENSMUSG00000081343	3	0	2	0	0	0
ENSMUSG00000089431	2	0	0	0	0	0
ENSMUSG00000097993	57	5	62	4	0	11
ENSMUSG00000093755	0	0	0	0	0	0
ENSMUSG00000093756	0	0	0	0	0	0
ENSMUSG00000093753	1	0	0	0	1	0
ENSMUSG00000093759	0	0	0	0	0	0
ENSMUSG00000105825	0	0	0	0	0	0
ENSMUSG00000099600	0	0	0	0	0	1
ENSMUSG00000031430	0	0	0	0	0	0
ENSMUSG00000031434	230	186	170	157	178	199
ENSMUSG00000047115	31	27	24	16	9	14
ENSMUSG00000099603	0	0	0	0	0	0
ENSMUSG00000099604	0	0	0	0	0	0
ENSMUSG00000099607	0	0	0	0	0	0
ENSMUSG00000038537	0	0	0	0	0	0
ENSMUSG00000049641	0	0	0	0	0	4
ENSMUSG00000049649	7	11	13	5	8	7
ENSMUSG00000049648	2	0	0	0	0	0
ENSMUSG00000101218	0	0	0	0	0	0
ENSMUSG00000101215	0	0	0	0	0	0
ENSMUSG00000101211	0	0	0	0	0	0
ENSMUSG00000101210	19	14	24	9	6	7
ENSMUSG00000101213	1	0	0	0	0	0
ENSMUSG00000101212	0	0	0	0	0	0
ENSMUSG00000043913	0	0	0	0	0	0
ENSMUSG00000043911	2	2	0	3	0	0
ENSMUSG00000087100	0	0	0	0	0	0
ENSMUSG00000087102	0	0	0	0	0	0
ENSMUSG00000087103	0	0	4	0	0	0
ENSMUSG00000087104	0	0	0	0	0	0
ENSMUSG00000087106	1	0	4	2	1	3
ENSMUSG00000099889	2	2	2	0	0	2
ENSMUSG00000099451	0	0	0	0	0	0
ENSMUSG00000099450	0	0	0	0	0	0
ENSMUSG00000099455	1	0	0	0	0	0
ENSMUSG00000099454	0	0	0	0	0	0
ENSMUSG00000099457	0	0	0	0	0	0
ENSMUSG00000099456	0	0	0	0	0	0
ENSMUSG00000019564	175	147	169	112	113	183
ENSMUSG00000083485	0	0	0	0	0	0
ENSMUSG00000083484	0	0	0	0	0	0
ENSMUSG00000083487	0	0	0	0	0	0
ENSMUSG00000083486	0	0	0	0	0	0
ENSMUSG00000083489	3	2	2	0	5	5
ENSMUSG00000083488	0	0	0	0	0	0
ENSMUSG00000084450	0	0	0	0	0	0
ENSMUSG00000030472	0	0	4	0	0	0
ENSMUSG00000100168	0	0	0	0	0	0
ENSMUSG00000082689	0	0	0	0	0	0
ENSMUSG00000030474	53	68	60	28	40	35
ENSMUSG00000100929	0	0	0	0	0	0

ENSMUSG00000100165	0	0	0	0	0	0
ENSMUSG00000100166	0	0	0	0	0	0
ENSMUSG00000100167	0	0	0	0	0	0
ENSMUSG00000082680	0	0	0	0	0	0
ENSMUSG00000082683	2	0	0	0	0	0
ENSMUSG00000099312	0	0	1	1	1	1
ENSMUSG00000099310	12	11	7	8	10	11
ENSMUSG00000032181	4	11	7	3	6	12
ENSMUSG00000099317	0	0	0	0	0	0
ENSMUSG00000083159	3	0	0	2	0	0
ENSMUSG00000083158	0	0	0	0	0	0
ENSMUSG00000037124	0	0	0	0	0	0
ENSMUSG00000037129	19	16	32	40	52	73
ENSMUSG00000083151	0	0	0	0	0	0
ENSMUSG00000083155	0	0	0	0	0	0
ENSMUSG00000083154	0	0	0	0	0	0
ENSMUSG00000114406	0	0	0	0	0	0
ENSMUSG00000100926	0	0	0	0	0	0
ENSMUSG00000041512	0	0	0	0	0	0
ENSMUSG00000114405	0	0	0	0	0	0
ENSMUSG00000064707	0	0	0	0	0	0
ENSMUSG00000064706	0	0	0	0	0	0
ENSMUSG00000032338	129	170	150	148	140	225
ENSMUSG00000060888	0	0	0	0	0	0
ENSMUSG00000031981	0	0	0	0	0	0
ENSMUSG00000105819	0	0	0	0	0	0
ENSMUSG00000087817	0	0	0	0	0	0
ENSMUSG00000087815	0	0	0	0	0	0
ENSMUSG00000087812	0	0	0	0	0	0
ENSMUSG00000087813	0	0	0	0	0	0
ENSMUSG00000087810	0	0	0	0	0	0
ENSMUSG00000085411	0	0	6	4	5	11
ENSMUSG00000085412	9	12	15	13	20	20
ENSMUSG00000085413	0	0	0	0	0	0
ENSMUSG00000085415	18	9	15	16	12	7
ENSMUSG00000085416	0	0	0	0	0	0
ENSMUSG00000074139	0	0	0	0	0	0
ENSMUSG00000015882	1068	1224	1009	922	911	1011
ENSMUSG00000015880	67	95	55	2	19	44
ENSMUSG00000107305	0	0	0	0	0	0
ENSMUSG00000107304	21	29	29	3	11	23
ENSMUSG00000107306	15	17	24	17	18	13
ENSMUSG00000062952	0	0	0	0	0	0
ENSMUSG00000107300	4	3	4	0	4	4
ENSMUSG00000107303	0	0	0	0	0	0
ENSMUSG00000107309	0	0	0	0	0	0
ENSMUSG00000107308	0	0	0	0	0	0
ENSMUSG00000079070	0	0	0	0	0	0
ENSMUSG00000065072	0	0	0	0	0	0
ENSMUSG00000088764	0	0	0	0	0	0
ENSMUSG00000088760	0	0	0	0	0	0
ENSMUSG00000062483	0	0	0	0	0	0
ENSMUSG00000092382	0	0	0	0	0	0
ENSMUSG00000103536	0	0	0	0	0	0
ENSMUSG00000103537	0	0	0	0	0	0
ENSMUSG00000103534	0	0	0	0	0	0
ENSMUSG00000103535	0	0	0	0	0	0
ENSMUSG00000103533	2	5	0	4	7	1

ENSMUSG00000103530	0	0	0	0	0	0
ENSMUSG00000016150	2	2	2	13	2	10
ENSMUSG00000034583	0	0	0	0	0	0
ENSMUSG00000034586	76	101	137	55	66	78
ENSMUSG00000034584	6	8	12	2	4	0
ENSMUSG00000103539	0	0	2	0	0	0
ENSMUSG00000059230	0	0	0	0	0	0
ENSMUSG00000078307	42	41	172	36	67	71
ENSMUSG00000102688	0	0	0	0	0	0
ENSMUSG00000024209	5	11	7	0	2	5
ENSMUSG00000078302	0	0	0	5	5	0
ENSMUSG00000024206	104	92	88	59	64	56
ENSMUSG00000078308	41	31	0	30	0	0
ENSMUSG00000039608	0	0	0	0	0	0
ENSMUSG00000097426	0	6	0	0	6	0
ENSMUSG00000097427	0	0	0	0	0	0
ENSMUSG00000097425	0	3	0	0	0	0
ENSMUSG00000097420	0	0	0	0	0	0
ENSMUSG00000097421	0	0	0	0	0	0
ENSMUSG00000097428	3	0	0	0	3	0
ENSMUSG00000097429	4	0	9	0	8	9
ENSMUSG00000051029	0	0	0	0	0	0
ENSMUSG00000114814	0	0	0	0	0	0
ENSMUSG00000114421	0	0	0	0	0	0
ENSMUSG00000114423	0	0	0	0	0	0
ENSMUSG00000114427	0	0	0	0	0	0
ENSMUSG00000100946	0	0	0	0	0	0
ENSMUSG00000089410	0	0	0	0	0	0
ENSMUSG00000100943	0	0	0	0	0	0
ENSMUSG00000093381	0	0	0	0	0	0
ENSMUSG00000089306	1	1	0	0	0	0
ENSMUSG00000054215	0	0	0	0	0	0
ENSMUSG00000050776	0	0	0	0	0	0
ENSMUSG00000099766	0	0	0	0	0	0
ENSMUSG00000050772	1	0	1	0	1	0
ENSMUSG00000074849	0	0	0	0	0	0
ENSMUSG00000086003	11	8	9	9	5	8
ENSMUSG00000105273	0	3	0	0	0	0
ENSMUSG00000114398	0	0	0	0	0	0
ENSMUSG00000105270	0	2	1	0	2	1
ENSMUSG00000105277	3	2	0	0	0	2
ENSMUSG00000105274	0	0	0	0	0	0
ENSMUSG00000114392	0	0	0	0	0	0
ENSMUSG00000114393	0	0	0	0	0	0
ENSMUSG00000114390	0	0	0	0	0	0
ENSMUSG00000105278	0	0	0	0	3	4
ENSMUSG00000114396	0	0	0	0	0	0
ENSMUSG00000114394	0	0	0	0	0	0
ENSMUSG00000114395	0	0	2	0	0	0
ENSMUSG00000008193	8	27	19	8	5	12
ENSMUSG00000074847	0	0	0	0	6	4
ENSMUSG00000067813	0	0	0	0	0	0
ENSMUSG00000091863	0	0	0	0	0	0
ENSMUSG00000091864	9	12	11	9	6	9
ENSMUSG00000091867	0	0	0	0	0	0
ENSMUSG00000105897	0	0	0	0	0	0
ENSMUSG00000043698	0	0	3	0	0	0
ENSMUSG00000105892	7	10	9	11	14	14

ENSMUSG00000096488	5	0	0	0	0	3
ENSMUSG00000096485	0	0	0	0	0	0
ENSMUSG00000096484	10	7	8	7	5	10
ENSMUSG00000096482	0	0	0	0	0	0
ENSMUSG00000096481	0	0	0	0	0	0
ENSMUSG00000105898	0	0	0	0	0	0
ENSMUSG00000108441	0	0	0	0	0	0
ENSMUSG00000108440	0	0	0	0	0	0
ENSMUSG00000108445	0	0	0	0	0	0
ENSMUSG00000108444	0	0	0	0	0	0
ENSMUSG00000108449	0	0	0	0	0	0
ENSMUSG00000108448	4	5	2	10	1	0
ENSMUSG00000089262	0	0	0	0	0	0
ENSMUSG00000026117	4	0	14	0	0	0
ENSMUSG00000026114	99	95	63	101	175	243
ENSMUSG00000105919	0	0	0	0	0	0
ENSMUSG00000101334	0	0	0	0	0	0
ENSMUSG00000091286	0	0	0	0	0	0
ENSMUSG00000091280	0	0	0	0	0	0
ENSMUSG00000091281	0	0	0	0	0	0
ENSMUSG00000091288	0	0	0	0	0	0
ENSMUSG00000091289	0	0	0	0	0	0
ENSMUSG00000111272	1	3	2	4	2	4
ENSMUSG00000111273	0	0	0	0	0	0
ENSMUSG00000111270	0	0	0	0	0	0
ENSMUSG00000111274	0	0	0	0	0	0
ENSMUSG00000111275	10	13	10	5	6	0
ENSMUSG00000027249	0	0	2	0	2	2
ENSMUSG00000110897	0	0	0	0	0	0
ENSMUSG00000110896	2	4	1	1	1	1
ENSMUSG00000110891	0	0	0	0	0	0
ENSMUSG00000110893	0	0	0	0	0	0
ENSMUSG00000110892	0	0	0	2	0	0
ENSMUSG00000110899	11	35	17	7	4	10
ENSMUSG00000110898	0	0	0	0	0	0
ENSMUSG00000086636	0	0	0	0	0	0
ENSMUSG00000091131	0	0	0	0	0	0
ENSMUSG00000078953	0	0	0	0	0	0
ENSMUSG00000081769	78	67	61	57	76	83
ENSMUSG00000081762	0	0	0	0	0	0
ENSMUSG00000081763	0	0	0	0	0	0
ENSMUSG00000081761	0	0	0	0	0	0
ENSMUSG00000081767	0	0	0	0	0	0
ENSMUSG00000089264	0	0	0	0	0	0
ENSMUSG00000099591	0	0	0	0	0	0
ENSMUSG00000112279	0	0	0	0	0	3
ENSMUSG00000094501	0	0	0	0	0	0
ENSMUSG00000045967	6	0	6	12	12	40
ENSMUSG00000094504	0	0	0	0	0	0
ENSMUSG00000094507	1	0	0	0	0	0
ENSMUSG00000094508	0	0	0	0	0	0
ENSMUSG00000022149	0	0	0	0	0	0
ENSMUSG00000044801	0	0	0	0	0	0
ENSMUSG00000044807	406	377	424	145	169	224
ENSMUSG00000111853	0	0	0	0	0	0
ENSMUSG00000065916	0	0	0	0	0	0
ENSMUSG00000093192	0	0	0	0	0	0
ENSMUSG00000086238	0	0	0	0	0	0

ENSMUSG00000093190	0	0	0	0	0	0
ENSMUSG00000093191	0	0	0	0	0	0
ENSMUSG00000086232	0	0	0	0	0	0
ENSMUSG00000077280	0	0	0	0	0	0
ENSMUSG00000023236	0	2	0	0	0	0
ENSMUSG00000028226	197	121	117	32	70	72
ENSMUSG00000023232	20	29	41	0	10	6
ENSMUSG00000097853	3	1	3	0	0	0
ENSMUSG00000097851	0	0	0	0	0	0
ENSMUSG00000097850	0	0	0	0	0	0
ENSMUSG00000016346	0	0	9	6	3	6
ENSMUSG00000077288	0	0	0	0	0	0
ENSMUSG00000086518	0	0	0	0	0	0
ENSMUSG00000049404	26	29	39	16	20	29
ENSMUSG00000049409	20	11	17	17	24	9
ENSMUSG00000057766	81	143	98	141	139	216
ENSMUSG00000047050	3	0	0	0	0	0
ENSMUSG00000109181	0	2	0	1	1	0
ENSMUSG00000096819	0	0	0	0	1	0
ENSMUSG00000109183	0	0	0	0	0	0
ENSMUSG00000109182	0	0	0	0	0	0
ENSMUSG00000109185	0	0	0	0	0	0
ENSMUSG00000109184	0	0	0	0	0	0
ENSMUSG00000109189	0	0	0	0	0	0
ENSMUSG00000096811	0	0	0	0	0	0
ENSMUSG00000096812	0	0	0	0	0	0
ENSMUSG00000096813	0	0	0	0	0	0
ENSMUSG00000096814	0	0	0	0	0	0
ENSMUSG00000096817	0	0	0	0	0	0
ENSMUSG00000106201	0	0	0	0	0	0
ENSMUSG00000106207	0	0	0	0	0	0
ENSMUSG00000103059	0	0	0	0	0	0
ENSMUSG00000109458	0	0	0	0	0	0
ENSMUSG00000103058	0	0	0	0	0	0
ENSMUSG00000109453	0	0	0	2	2	0
ENSMUSG00000109452	88	103	83	46	64	89
ENSMUSG00000109451	0	0	0	0	0	0
ENSMUSG00000109450	0	0	0	0	0	0
ENSMUSG00000109457	1	0	0	0	0	0
ENSMUSG00000108468	16	20	12	14	14	8
ENSMUSG00000082448	0	0	0	0	0	0
ENSMUSG00000082449	0	0	0	0	0	0
ENSMUSG00000082442	0	0	0	0	0	0
ENSMUSG00000082443	0	0	0	0	0	0
ENSMUSG00000082440	0	0	0	0	2	0
ENSMUSG00000082447	0	0	0	0	0	2
ENSMUSG00000082444	0	0	0	0	0	0
ENSMUSG00000006542	106	105	116	96	107	137
ENSMUSG00000110075	0	0	0	0	0	0
ENSMUSG00000110074	0	0	0	0	0	0
ENSMUSG00000110073	0	0	0	0	0	0
ENSMUSG00000110071	39	34	39	10	23	9
ENSMUSG00000050982	0	0	0	3	0	0
ENSMUSG00000110078	0	3	0	0	0	0
ENSMUSG00000054156	0	0	0	0	0	0
ENSMUSG00000049809	0	0	0	1	0	0
ENSMUSG00000083319	0	3	0	0	0	0
ENSMUSG00000083318	0	0	0	0	0	0

ENSMUSG00000083310	0	0	0	0	0	0
ENSMUSG00000108462	0	0	0	0	0	0
ENSMUSG00000083315	0	0	0	0	0	0
ENSMUSG00000083316	0	2	0	0	0	0
ENSMUSG00000049804	447	405	651	296	383	486
ENSMUSG00000098316	0	0	0	0	0	0
ENSMUSG00000098194	0	0	0	0	2	1
ENSMUSG00000073179	8	10	20	10	9	21
ENSMUSG00000098311	0	0	0	0	0	0
ENSMUSG00000099042	0	0	0	0	0	0
ENSMUSG00000070219	0	0	0	0	0	0
ENSMUSG00000040919	8	0	0	0	0	0
ENSMUSG00000022758	60	64	71	65	123	88
ENSMUSG00000022759	0	0	0	0	0	0
ENSMUSG00000101043	0	0	0	0	0	0
ENSMUSG00000000606	0	0	0	0	0	0
ENSMUSG00000041068	0	0	0	0	0	0
ENSMUSG00000068426	0	0	0	0	0	0
ENSMUSG00000068428	0	0	0	0	0	0
ENSMUSG00000041064	12	4	16	0	7	4
ENSMUSG00000041062	0	0	0	0	4	0
ENSMUSG00000106712	0	0	0	0	0	0
ENSMUSG00000085256	0	0	3	0	3	0
ENSMUSG00000085257	2	0	0	0	0	0
ENSMUSG00000085254	0	0	0	0	0	0
ENSMUSG00000085252	6	0	0	17	13	20
ENSMUSG00000085251	0	0	0	0	0	0
ENSMUSG00000098312	5	2	1	1	2	2
ENSMUSG00000093941	0	0	0	0	0	0
ENSMUSG00000093940	0	0	0	0	0	0
ENSMUSG00000093943	0	0	0	0	0	0
ENSMUSG00000022753	5	0	0	4	0	4
ENSMUSG00000093945	0	0	0	0	0	0
ENSMUSG00000093944	0	0	0	0	0	0
ENSMUSG00000093947	0	0	0	0	0	0
ENSMUSG00000093946	0	0	0	0	0	0
ENSMUSG00000093949	0	0	0	0	0	0
ENSMUSG00000099865	0	0	0	0	0	0
ENSMUSG00000079273	0	0	0	0	0	0
ENSMUSG00000079272	0	0	0	0	0	0
ENSMUSG00000079271	0	0	0	0	0	0
ENSMUSG00000068399	0	0	0	0	0	0
ENSMUSG00000079276	0	0	0	0	0	0
ENSMUSG00000068392	0	0	0	0	0	0
ENSMUSG00000079278	0	0	0	0	0	0
ENSMUSG00000068394	249	284	312	255	280	389
ENSMUSG00000080155	0	0	0	0	0	0
ENSMUSG00000080158	2	0	0	0	0	0
ENSMUSG00000077647	0	0	0	0	0	0
ENSMUSG00000045364	0	0	0	0	0	0
ENSMUSG00000045362	53	58	57	15	14	40
ENSMUSG00000079893	0	0	0	0	0	0
ENSMUSG00000079892	0	0	0	0	0	0
ENSMUSG00000079890	0	0	0	0	0	0
ENSMUSG00000099950	0	0	0	0	0	0
ENSMUSG00000063514	0	0	0	0	0	0
ENSMUSG00000099952	0	0	0	0	0	0
ENSMUSG00000099953	2	0	0	0	0	0

ENSMUSG00000099956	0	0	0	0	0	0
ENSMUSG00000073960	0	3	0	0	0	0
ENSMUSG00000074443	0	0	0	0	0	0
ENSMUSG00000074442	0	0	0	0	0	0
ENSMUSG00000074441	0	0	0	0	0	0
ENSMUSG00000113996	0	0	0	0	0	0
ENSMUSG00000074447	0	0	0	0	0	0
ENSMUSG00000077422	0	0	1	0	0	2
ENSMUSG00000074445	3	3	2	0	0	0
ENSMUSG00000074444	0	0	0	0	0	0
ENSMUSG00000074448	0	0	0	0	0	0
ENSMUSG00000113990	0	0	0	0	0	0
ENSMUSG00000113993	6	0	0	0	2	2
ENSMUSG00000113992	0	0	0	0	0	0
ENSMUSG00000093426	0	0	0	0	0	0
ENSMUSG00000081202	0	0	0	0	0	0
ENSMUSG00000081207	0	0	0	0	0	0
ENSMUSG00000077428	0	0	0	0	0	0
ENSMUSG00000009047	0	0	0	2	0	0
ENSMUSG00000072849	4	9	0	14	11	11
ENSMUSG00000111883	0	0	0	0	0	0
ENSMUSG00000072845	0	0	0	2	4	3
ENSMUSG00000111882	0	0	0	0	0	0
ENSMUSG00000048411	0	0	0	0	0	0
ENSMUSG00000077369	0	0	0	0	0	0
ENSMUSG00000077368	0	0	0	0	0	0
ENSMUSG00000098603	0	0	0	0	0	0
ENSMUSG00000098601	0	0	0	0	0	0
ENSMUSG00000111783	0	0	0	0	0	0
ENSMUSG00000077361	0	0	0	0	0	0
ENSMUSG00000077366	0	0	0	0	0	0
ENSMUSG00000111785	12	16	7	2	0	5
ENSMUSG00000030393	172	125	152	128	146	163
ENSMUSG00000082586	0	0	0	0	0	0
ENSMUSG00000031669	70	92	84	60	67	86
ENSMUSG00000031661	65	44	57	13	25	32
ENSMUSG00000031665	22	23	28	12	26	32
ENSMUSG00000101921	0	0	3	2	2	0
ENSMUSG00000086096	4	0	3	0	4	9
ENSMUSG00000102979	0	0	0	0	0	0
ENSMUSG00000102975	3	2	3	3	0	4
ENSMUSG00000102977	0	0	0	0	0	0
ENSMUSG00000102971	2	0	0	0	0	0
ENSMUSG00000033282	409	418	473	287	326	362
ENSMUSG00000112283	0	0	0	0	0	0
ENSMUSG00000094371	0	0	0	0	0	0
ENSMUSG00000082161	0	0	0	0	0	0
ENSMUSG00000082160	11	25	9	21	25	25
ENSMUSG00000082163	6	14	11	4	9	13
ENSMUSG00000082162	0	0	0	0	0	0
ENSMUSG00000088149	0	0	0	0	0	0
ENSMUSG00000088148	0	0	6	0	0	0
ENSMUSG00000082167	0	0	0	0	0	0
ENSMUSG00000088145	0	0	0	0	0	0
ENSMUSG00000088144	0	0	0	0	0	0
ENSMUSG00000088147	0	0	0	0	0	0
ENSMUSG00000088143	0	0	0	0	0	0
ENSMUSG00000101401	0	0	0	0	0	0

ENSMUSG00000101402	0	0	0	0	0	0
ENSMUSG00000049618	0	0	0	0	0	0
ENSMUSG00000093541	0	0	0	0	0	0
ENSMUSG00000107805	0	0	0	0	0	0
ENSMUSG00000107800	0	0	0	0	0	0
ENSMUSG00000107808	0	0	0	0	0	0
ENSMUSG00000107809	0	0	0	0	0	0
ENSMUSG00000103314	0	0	0	1	1	1
ENSMUSG00000103315	0	0	0	0	0	0
ENSMUSG00000103312	0	0	0	0	0	0
ENSMUSG00000093540	6	7	0	14	11	8
ENSMUSG00000099521	0	0	0	0	0	0
ENSMUSG00000103318	0	0	0	0	0	0
ENSMUSG00000099520	0	0	0	0	0	0
ENSMUSG00000092311	0	0	0	0	0	0
ENSMUSG00000096284	0	2	0	0	0	0
ENSMUSG00000107474	0	0	0	0	0	0
ENSMUSG00000089234	0	0	0	0	0	0
ENSMUSG00000084647	0	0	0	0	0	0
ENSMUSG00000089237	2	2	2	1	1	1
ENSMUSG00000089230	0	0	0	0	0	0
ENSMUSG00000089231	0	0	0	0	2	0
ENSMUSG00000084640	0	0	0	0	0	0
ENSMUSG00000089233	0	0	0	0	0	0
ENSMUSG00000075181	2	0	0	0	0	0
ENSMUSG00000075185	0	0	0	0	0	0
ENSMUSG00000075184	0	2	0	3	0	0
ENSMUSG00000075187	1	0	0	0	0	0
ENSMUSG00000075186	0	0	0	0	0	0
ENSMUSG00000075189	0	0	0	0	0	0
ENSMUSG00000022303	0	0	0	0	0	0
ENSMUSG00000103246	0	0	0	0	0	0
ENSMUSG00000022304	0	3	0	0	9	0
ENSMUSG00000101998	0	0	0	0	0	0
ENSMUSG00000095925	0	0	0	0	0	0
ENSMUSG00000095924	0	0	0	0	0	0
ENSMUSG00000095926	0	0	0	0	0	0
ENSMUSG00000095922	0	0	0	0	0	0
ENSMUSG00000100465	6	12	14	8	16	3
ENSMUSG00000100468	0	0	0	0	0	0
ENSMUSG00000113958	0	0	0	0	0	0
ENSMUSG00000096554	0	0	0	0	0	0
ENSMUSG00000064515	0	0	0	0	0	0
ENSMUSG00000064510	0	0	0	0	0	0
ENSMUSG00000064511	0	0	0	0	0	0
ENSMUSG00000102199	0	0	0	0	0	0
ENSMUSG00000102198	0	3	0	2	0	3
ENSMUSG00000046585	2	0	0	7	0	0
ENSMUSG00000107114	0	0	0	0	0	4
ENSMUSG00000107117	0	0	0	0	0	0
ENSMUSG00000107112	4	0	0	2	0	4
ENSMUSG00000107113	0	0	0	0	0	0
ENSMUSG00000028020	4	13	5	9	2	14
ENSMUSG00000028023	3	3	7	4	7	0
ENSMUSG00000100119	0	0	0	0	0	0
ENSMUSG00000021545	0	0	0	0	0	0
ENSMUSG00000098698	0	0	0	0	0	0
ENSMUSG00000021541	0	0	0	0	0	0

ENSMUSG00000100118	0	0	0	0	0	0
ENSMUSG00000029778	357	449	522	255	70	153
ENSMUSG00000019834	0	0	0	0	0	0
ENSMUSG00000025433	0	3	0	0	0	0
ENSMUSG00000092316	0	0	0	0	0	0
ENSMUSG00000069292	0	0	0	0	0	0
ENSMUSG00000069294	0	0	0	0	0	0
ENSMUSG00000069295	0	0	0	0	0	0
ENSMUSG00000069296	0	0	0	0	0	0
ENSMUSG00000069297	0	0	0	0	0	0
ENSMUSG00000069299	0	0	0	0	0	0
ENSMUSG00000090142	0	1	0	0	0	0
ENSMUSG00000035395	0	0	0	0	0	0
ENSMUSG00000035390	105	59	75	18	19	27
ENSMUSG00000039830	0	0	0	0	0	4
ENSMUSG00000039838	9	0	2	4	9	3
ENSMUSG00000106444	0	0	0	0	0	0
ENSMUSG00000113740	0	0	0	1	0	0
ENSMUSG00000113744	0	0	0	0	0	0
ENSMUSG00000113745	0	0	0	0	0	0
ENSMUSG00000113747	0	0	0	0	0	0
ENSMUSG00000113748	0	0	0	0	0	0
ENSMUSG00000034623	0	15	4	7	22	13
ENSMUSG00000109619	0	0	0	0	0	0
ENSMUSG00000111458	0	0	0	0	0	0
ENSMUSG00000095436	0	0	0	0	0	0
ENSMUSG00000051617	0	0	0	0	0	0
ENSMUSG00000051616	0	0	0	0	0	0
ENSMUSG00000109613	0	0	0	0	0	0
ENSMUSG00000111451	0	0	0	0	0	0
ENSMUSG00000111456	0	0	0	0	0	0
ENSMUSG00000111457	0	0	0	0	0	0
ENSMUSG00000109617	0	2	4	0	0	0
ENSMUSG00000111455	0	0	0	0	0	0
ENSMUSG00000089591	0	0	0	0	0	0
ENSMUSG00000089593	0	0	0	0	0	0
ENSMUSG00000104978	0	0	0	0	0	2
ENSMUSG00000111188	0	2	2	0	0	0
ENSMUSG00000066113	216	254	352	83	72	88
ENSMUSG00000106995	2	2	0	0	0	0
ENSMUSG00000106994	20	13	20	9	26	28
ENSMUSG00000097382	0	0	0	0	0	0
ENSMUSG00000106999	4	0	3	2	1	2
ENSMUSG00000097387	4	7	0	7	4	12
ENSMUSG00000097386	4	18	16	0	0	10
ENSMUSG00000098696	0	0	0	0	0	0
ENSMUSG00000097655	0	0	0	0	0	0
ENSMUSG00000097651	0	0	0	0	0	0
ENSMUSG00000104598	0	0	0	0	0	0
ENSMUSG00000097653	0	0	0	0	0	0
ENSMUSG00000104595	0	0	0	0	0	0
ENSMUSG00000104596	0	0	0	0	0	0
ENSMUSG00000097659	0	0	0	0	0	0
ENSMUSG00000104590	0	0	0	0	0	0
ENSMUSG00000104593	0	0	0	0	0	0
ENSMUSG00000104592	5	9	9	5	9	5
ENSMUSG00000093784	0	1	1	0	0	0
ENSMUSG00000069830	14	15	41	6	2	12

ENSMUSG00000050050	0	4	9	8	7	2
ENSMUSG00000096380	0	0	0	0	0	0
ENSMUSG00000096381	0	0	0	0	0	0
ENSMUSG00000096384	0	0	0	0	0	0
ENSMUSG00000114219	0	0	0	0	0	0
ENSMUSG00000096387	0	0	0	0	0	0
ENSMUSG00000114216	0	0	0	0	1	0
ENSMUSG00000114215	0	0	0	0	0	0
ENSMUSG00000114214	0	0	0	0	0	0
ENSMUSG00000114211	0	0	0	1	0	0
ENSMUSG00000112901	0	1	4	0	0	6
ENSMUSG00000112903	0	0	0	0	0	0
ENSMUSG00000112902	0	0	2	0	0	0
ENSMUSG00000112905	0	0	0	0	0	0
ENSMUSG00000112904	0	0	0	0	0	0
ENSMUSG00000112907	8	6	3	0	4	0
ENSMUSG00000112906	3	3	0	3	0	3
ENSMUSG00000114933	0	0	0	0	0	0
ENSMUSG00000082899	0	0	0	0	0	0
ENSMUSG00000058731	0	0	0	0	0	0
ENSMUSG00000044216	5	10	0	18	10	22
ENSMUSG00000044213	0	0	1	0	0	0
ENSMUSG00000110461	0	0	0	0	0	0
ENSMUSG00000110466	0	0	0	2	0	0
ENSMUSG00000110468	0	0	0	0	0	0
ENSMUSG00000050251	0	0	0	0	0	0
ENSMUSG00000056829	0	0	0	0	0	0
ENSMUSG00000114938	0	0	0	0	0	0
ENSMUSG00000104972	0	2	0	0	0	0
ENSMUSG00000056822	0	0	0	0	0	0
ENSMUSG00000056824	0	0	0	0	0	0
ENSMUSG00000111186	3	2	2	0	0	0
ENSMUSG00000094521	0	0	0	0	0	0
ENSMUSG00000096525	0	0	0	0	0	0
ENSMUSG00000096527	0	0	0	0	0	0
ENSMUSG00000096520	0	0	0	0	0	0
ENSMUSG00000094524	0	0	0	0	0	0
ENSMUSG00000096522	2	0	0	1	0	0
ENSMUSG00000096523	0	0	0	0	0	0
ENSMUSG00000032093	15	0	6	2	3	0
ENSMUSG00000096528	0	0	0	0	0	0
ENSMUSG00000098969	0	0	0	0	0	0
ENSMUSG00000013418	2	4	0	4	0	3
ENSMUSG00000090306	0	0	0	0	0	0
ENSMUSG00000080317	0	0	0	0	0	0
ENSMUSG00000060224	119	53	98	33	37	52
ENSMUSG00000111180	0	0	0	0	0	0
ENSMUSG00000013415	3	0	0	0	0	0
ENSMUSG00000026322	0	0	0	0	0	0
ENSMUSG00000072616	0	0	0	0	0	0
ENSMUSG00000026327	0	0	0	0	0	0
ENSMUSG00000100014	0	0	0	0	0	0
ENSMUSG00000099872	0	0	0	3	0	0
ENSMUSG00000100019	0	0	0	0	0	0
ENSMUSG00000111182	0	0	0	0	0	0
ENSMUSG00000072618	8	0	10	0	0	0
ENSMUSG00000001823	0	0	0	0	0	0
ENSMUSG00000111338	0	0	0	0	0	0

ENSMUSG00000111335	0	0	0	0	0	0
ENSMUSG00000111330	0	0	0	0	0	0
ENSMUSG00000081048	0	0	0	0	0	3
ENSMUSG00000081045	0	2	0	0	0	1
ENSMUSG00000081042	2	0	0	5	3	1
ENSMUSG00000081041	0	0	0	0	0	0
ENSMUSG00000111831	0	0	0	0	0	0
ENSMUSG00000110555	0	0	0	0	0	0
ENSMUSG00000109170	0	0	0	0	0	0
ENSMUSG00000044827	138	120	163	29	38	61
ENSMUSG00000112314	0	0	0	0	0	0
ENSMUSG00000109173	0	1	1	0	0	0
ENSMUSG00000044824	2	0	3	3	0	0
ENSMUSG00000099938	0	0	0	0	0	0
ENSMUSG00000094992	0	0	0	0	0	0
ENSMUSG00000081598	0	2	0	0	0	0
ENSMUSG00000107930	0	0	0	0	0	2
ENSMUSG00000094991	0	0	0	0	0	0
ENSMUSG00000081591	0	0	0	0	0	0
ENSMUSG00000081592	2	1	1	1	0	0
ENSMUSG00000048655	7	9	17	5	0	2
ENSMUSG00000081596	0	0	0	0	0	0
ENSMUSG00000077169	0	0	0	0	0	0
ENSMUSG00000046932	0	0	0	0	0	2
ENSMUSG00000077161	0	0	0	0	0	0
ENSMUSG00000077160	0	0	0	0	0	0
ENSMUSG00000077163	0	0	0	0	0	0
ENSMUSG00000077166	0	0	0	0	0	0
ENSMUSG00000094527	0	0	0	0	0	0
ENSMUSG00000093549	0	0	0	0	0	0
ENSMUSG00000093543	3	0	0	0	0	0
ENSMUSG00000105451	0	0	0	0	0	0
ENSMUSG00000099628	0	0	0	0	0	0
ENSMUSG00000099629	0	0	0	0	0	0
ENSMUSG00000092638	0	0	0	0	0	0
ENSMUSG00000092639	0	0	0	0	0	0
ENSMUSG00000099622	8	20	23	6	6	16
ENSMUSG00000099623	0	0	0	1	0	0
ENSMUSG00000099620	0	0	0	0	0	0
ENSMUSG00000099621	0	0	0	0	0	0
ENSMUSG00000099626	0	0	0	0	0	0
ENSMUSG00000099627	0	0	0	0	0	0
ENSMUSG00000099625	0	0	0	0	2	0
ENSMUSG00000112178	0	0	0	0	2	0
ENSMUSG00000112179	0	0	0	0	0	0
ENSMUSG00000112170	0	0	0	0	0	0
ENSMUSG00000055700	0	0	0	0	0	0
ENSMUSG00000112174	0	0	0	0	0	0
ENSMUSG00000055704	4	0	0	0	2	0
ENSMUSG00000064141	49	59	62	66	61	85
ENSMUSG00000112319	1	1	1	1	1	1
ENSMUSG00000021396	7	0	9	0	2	5
ENSMUSG00000071719	0	0	0	0	0	0
ENSMUSG00000051936	0	0	0	0	0	0
ENSMUSG00000086060	0	0	0	0	0	0
ENSMUSG00000086063	0	0	0	0	0	0
ENSMUSG00000086067	0	3	0	0	0	0
ENSMUSG00000098979	0	1	2	3	1	2

ENSMUSG00000098978	0	0	0	0	0	0
ENSMUSG00000049217	1	0	0	0	0	0
ENSMUSG00000049214	0	0	0	0	0	0
ENSMUSG00000104833	0	4	0	0	0	2
ENSMUSG00000098970	0	0	0	0	0	0
ENSMUSG00000026162	1	5	2	1	0	3
ENSMUSG00000076343	0	0	0	0	0	0
ENSMUSG00000062794	47	42	99	48	63	78
ENSMUSG00000082767	0	0	0	0	0	0
ENSMUSG00000113227	0	0	0	0	0	0
ENSMUSG00000000202	0	0	0	0	0	0
ENSMUSG00000113225	0	0	0	0	0	0
ENSMUSG00000113221	1	0	0	0	0	0
ENSMUSG00000113220	0	0	0	0	0	0
ENSMUSG00000113229	0	0	3	0	0	0
ENSMUSG00000113228	0	0	0	0	0	0
ENSMUSG00000094386	0	0	0	0	0	0
ENSMUSG00000065819	0	0	1	0	0	0
ENSMUSG00000037424	0	0	0	0	0	0
ENSMUSG00000037428	0	0	0	0	0	0
ENSMUSG00000112450	8	0	2	2	6	8
ENSMUSG00000065812	0	0	0	0	0	0
ENSMUSG00000093173	0	0	0	0	0	0
ENSMUSG00000070977	0	0	0	0	0	0
ENSMUSG00000090547	0	0	0	0	0	0
ENSMUSG00000040272	246	268	285	194	317	355
ENSMUSG00000040270	155	301	344	73	200	181
ENSMUSG00000040276	25	22	93	7	23	61
ENSMUSG00000037393	0	0	0	0	0	0
ENSMUSG00000037390	0	0	0	0	0	0
ENSMUSG00000065815	0	0	0	0	0	0
ENSMUSG00000100590	0	0	0	0	0	0
ENSMUSG00000055373	0	0	2	0	0	2
ENSMUSG00000057836	9	7	37	31	16	50
ENSMUSG00000094309	0	0	0	0	0	0
ENSMUSG00000040891	0	0	0	5	3	0
ENSMUSG00000040899	0	0	6	0	0	4
ENSMUSG00000084336	0	0	0	0	0	0
ENSMUSG00000084337	0	0	0	0	0	0
ENSMUSG00000084334	0	0	0	0	0	0
ENSMUSG00000084335	0	0	0	0	0	0
ENSMUSG00000084332	0	0	2	0	0	0
ENSMUSG00000094442	0	0	0	0	0	0
ENSMUSG00000084330	0	0	0	0	0	0
ENSMUSG00000075611	0	0	0	0	0	0
ENSMUSG00000075610	0	0	0	0	0	0
ENSMUSG00000084338	0	0	0	0	0	0
ENSMUSG00000103038	40	38	44	19	20	17
ENSMUSG00000046493	0	0	0	0	0	0
ENSMUSG00000099369	0	0	0	0	0	0
ENSMUSG00000099368	0	0	1	0	0	1
ENSMUSG00000099367	0	0	0	0	0	0
ENSMUSG00000099366	0	0	0	0	0	0
ENSMUSG00000099364	0	8	0	2	3	3
ENSMUSG00000099363	0	0	0	0	0	0
ENSMUSG00000097873	0	9	0	9	0	8
ENSMUSG00000099361	0	0	0	0	0	0
ENSMUSG00000109794	0	0	0	0	0	0

ENSMUSG00000078599	0	0	0	0	0	0
ENSMUSG00000078598	0	0	0	0	0	0
ENSMUSG00000096268	0	0	0	0	0	0
ENSMUSG00000023210	0	0	0	0	0	0
ENSMUSG00000064490	0	0	0	0	0	0
ENSMUSG00000111710	0	0	0	0	0	0
ENSMUSG00000065629	0	0	0	0	0	0
ENSMUSG00000068219	0	0	0	0	0	0
ENSMUSG00000068218	0	0	0	0	0	0
ENSMUSG00000065625	0	0	0	0	0	0
ENSMUSG00000065620	0	0	0	0	0	0
ENSMUSG00000065622	0	0	0	0	0	0
ENSMUSG00000070023	0	0	0	0	0	0
ENSMUSG00000064497	0	0	0	0	0	0
ENSMUSG00000080057	0	0	0	0	2	0
ENSMUSG00000032278	60	79	80	40	68	50
ENSMUSG00000080051	0	0	0	0	0	0
ENSMUSG00000080050	0	0	0	0	0	0
ENSMUSG00000111719	0	0	0	0	0	0
ENSMUSG00000032274	1	2	0	0	0	0
ENSMUSG00000058290	93	57	129	11	40	69
ENSMUSG00000081903	0	0	0	0	0	0
ENSMUSG00000081900	0	0	0	0	0	0
ENSMUSG00000081901	0	0	0	0	0	0
ENSMUSG00000081904	0	0	0	0	0	0
ENSMUSG00000081905	0	0	0	0	0	0
ENSMUSG00000081908	1	0	0	0	0	0
ENSMUSG00000081909	0	0	0	0	0	0
ENSMUSG00000082056	0	0	0	0	0	0
ENSMUSG00000098108	0	0	0	0	0	0
ENSMUSG00000113928	0	0	0	0	0	0
ENSMUSG00000088036	2	0	1	0	0	0
ENSMUSG00000113929	0	0	0	0	0	0
ENSMUSG00000098100	0	1	2	0	0	2
ENSMUSG00000098102	2	0	7	0	3	4
ENSMUSG00000098103	0	3	0	4	0	0
ENSMUSG00000098106	0	0	0	0	0	0
ENSMUSG00000087021	0	0	0	0	0	0
ENSMUSG00000095263	0	0	0	0	0	0
ENSMUSG00000063305	0	0	0	0	0	0
ENSMUSG00000095261	0	0	0	0	0	1
ENSMUSG00000081174	0	0	0	0	0	0
ENSMUSG00000040498	62	34	65	39	103	82
ENSMUSG00000057207	0	0	1	1	0	0
ENSMUSG00000101358	0	0	0	0	0	0
ENSMUSG00000113923	0	0	0	1	0	0
ENSMUSG00000108229	0	0	0	0	0	0
ENSMUSG00000081173	0	0	0	0	0	0
ENSMUSG00000057203	0	0	0	0	0	0
ENSMUSG00000103903	0	0	0	0	0	0
ENSMUSG00000103902	0	0	0	0	0	0
ENSMUSG00000103905	0	0	0	0	0	0
ENSMUSG00000103909	0	0	0	0	0	0
ENSMUSG00000095269	1	1	0	0	0	1
ENSMUSG00000101356	0	0	0	0	0	0
ENSMUSG00000112562	0	0	0	0	0	0
ENSMUSG00000038980	0	0	0	0	0	2
ENSMUSG00000081172	0	0	0	0	0	0

ENSMUSG00000040495	3	3	5	0	3	0
ENSMUSG00000081171	0	0	0	0	0	0
ENSMUSG00000070563	2	0	0	0	0	0
ENSMUSG00000089866	0	0	0	0	0	0
ENSMUSG00000101871	0	0	0	0	0	0
ENSMUSG00000101870	0	0	0	0	0	0
ENSMUSG00000103969	0	0	0	0	0	0
ENSMUSG00000103968	2	0	0	0	0	0
ENSMUSG00000091416	0	0	0	0	0	0
ENSMUSG00000091411	0	0	0	0	0	0
ENSMUSG00000091410	3	0	0	1	1	1
ENSMUSG00000020808	52	43	53	17	33	35
ENSMUSG00000020804	8	14	14	11	14	10
ENSMUSG00000020805	0	0	0	0	0	0
ENSMUSG00000106196	0	0	0	0	0	0
ENSMUSG00000108810	0	0	0	0	0	0
ENSMUSG00000108811	0	0	0	0	0	0
ENSMUSG00000108814	0	0	0	0	0	0
ENSMUSG00000108816	0	0	0	0	0	0
ENSMUSG00000108818	0	2	0	0	0	0
ENSMUSG00000093534	0	0	0	0	0	0
ENSMUSG00000105991	0	0	0	0	0	0
ENSMUSG00000105997	0	0	0	0	0	0
ENSMUSG00000107098	0	0	0	0	0	0
ENSMUSG00000101679	0	0	0	0	0	0
ENSMUSG00000060024	0	0	0	0	0	0
ENSMUSG00000001155	4	0	0	0	0	7
ENSMUSG00000013653	0	0	0	0	0	0
ENSMUSG00000101670	0	0	0	0	0	0
ENSMUSG00000014686	0	0	4	0	0	0
ENSMUSG00000101675	0	0	0	0	0	0
ENSMUSG00000085816	0	0	4	0	0	6
ENSMUSG00000037984	0	0	0	0	0	0
ENSMUSG00000085810	8	13	10	8	0	10
ENSMUSG00000085813	0	0	0	0	0	0
ENSMUSG00000103264	0	0	0	0	0	0
ENSMUSG00000093812	0	0	0	0	0	0
ENSMUSG00000015484	0	0	0	0	0	0
ENSMUSG00000100088	0	0	0	0	0	0
ENSMUSG00000109463	0	0	0	0	0	0
ENSMUSG00000047031	0	0	0	0	0	0
ENSMUSG00000087527	0	2	0	0	2	0
ENSMUSG00000087524	0	0	0	0	0	0
ENSMUSG00000087522	2	0	0	3	2	3
ENSMUSG00000087521	0	0	0	0	0	0
ENSMUSG00000095190	0	0	0	0	0	0
ENSMUSG00000045587	0	0	0	0	0	0
ENSMUSG00000103292	5	2	12	0	2	0
ENSMUSG00000103297	0	0	0	2	0	0
ENSMUSG00000103296	0	0	0	0	0	0
ENSMUSG00000103295	0	0	0	0	0	0
ENSMUSG00000103127	0	0	0	0	0	0
ENSMUSG00000103126	5	4	5	0	0	2
ENSMUSG00000103121	0	0	0	0	0	0
ENSMUSG00000104419	3	0	2	0	0	0
ENSMUSG00000045581	0	0	0	0	0	0
ENSMUSG00000036169	5	0	0	0	0	5
ENSMUSG00000071633	0	0	0	0	0	0

ENSMUSG00000014351	0	0	0	0	0	0
ENSMUSG00000064232	0	0	0	0	0	0
ENSMUSG00000107635	0	0	0	0	0	0
ENSMUSG00000107637	0	0	0	0	0	0
ENSMUSG00000107631	0	0	2	0	0	0
ENSMUSG00000107630	0	0	0	0	0	0
ENSMUSG00000104411	0	0	0	0	0	0
ENSMUSG00000101408	0	0	0	0	0	0
ENSMUSG00000104412	0	0	0	0	0	0
ENSMUSG00000075934	0	0	0	0	0	0
ENSMUSG00000036292	142	82	139	55	53	43
ENSMUSG00000095886	0	0	0	0	0	0
ENSMUSG00000095887	0	0	0	0	0	0
ENSMUSG00000095884	0	0	0	1	1	1
ENSMUSG00000095885	0	0	0	0	0	0
ENSMUSG00000036298	188	121	126	45	45	50
ENSMUSG00000104414	0	0	0	0	0	0
ENSMUSG00000110045	0	0	0	0	0	2
ENSMUSG00000065546	0	0	0	0	0	0
ENSMUSG00000103733	2	3	3	2	4	3
ENSMUSG00000065542	0	0	2	0	0	0
ENSMUSG00000104417	0	0	0	0	0	0
ENSMUSG00000065549	0	0	0	0	0	0
ENSMUSG00000022512	4	3	10	2	5	0
ENSMUSG00000022510	16	24	26	23	26	39
ENSMUSG00000101687	0	0	0	0	0	0
ENSMUSG00000103737	0	0	0	0	0	0
ENSMUSG00000089008	0	0	0	0	0	0
ENSMUSG00000018479	0	0	0	0	0	0
ENSMUSG00000081716	0	0	0	0	0	0
ENSMUSG00000089001	0	0	0	0	0	0
ENSMUSG00000089000	0	0	0	0	0	0
ENSMUSG00000089007	0	0	0	0	0	0
ENSMUSG00000089005	0	1	0	0	0	0
ENSMUSG00000089004	0	0	0	0	0	0
ENSMUSG00000084567	0	0	0	0	0	0
ENSMUSG00000028613	86	74	134	6	24	24
ENSMUSG00000028612	0	3	0	0	0	0
ENSMUSG00000028610	0	0	0	0	0	0
ENSMUSG00000102654	0	5	0	0	0	0
ENSMUSG00000112377	0	0	0	0	0	0
ENSMUSG00000102656	0	0	0	0	0	0
ENSMUSG00000109065	0	0	0	0	0	0
ENSMUSG00000112372	0	0	0	0	0	0
ENSMUSG00000102652	3	8	7	4	4	0
ENSMUSG00000106589	0	0	0	0	0	0
ENSMUSG00000102658	3	0	0	0	0	0
ENSMUSG00000102659	3	0	0	3	0	0
ENSMUSG00000112378	0	0	0	0	0	0
ENSMUSG00000109100	0	0	0	0	0	0
ENSMUSG00000114780	4	4	2	6	0	2
ENSMUSG00000036834	0	0	0	2	0	0
ENSMUSG00000110201	0	0	0	2	0	0
ENSMUSG00000110203	0	0	0	0	0	0
ENSMUSG00000110204	0	0	0	0	0	0
ENSMUSG00000110205	0	0	0	0	0	0
ENSMUSG00000036832	19	4	9	0	9	3
ENSMUSG00000110207	0	0	0	0	0	0

ENSMUSG00000089348	0	0	0	0	0	0
ENSMUSG00000109103	1	0	0	0	0	0
ENSMUSG00000107685	0	0	0	0	0	0
ENSMUSG00000084596	0	0	0	0	0	0
ENSMUSG00000105563	0	0	0	0	0	0
ENSMUSG00000105560	0	0	0	0	0	5
ENSMUSG00000105567	0	0	0	0	0	0
ENSMUSG00000105566	0	0	0	0	0	0
ENSMUSG00000105564	0	0	0	0	0	0
ENSMUSG00000066090	0	0	0	0	0	2
ENSMUSG00000088870	0	0	0	0	0	0
ENSMUSG00000088873	1	1	1	2	0	1
ENSMUSG00000088872	2	0	0	0	0	0
ENSMUSG00000088874	0	0	0	0	0	0
ENSMUSG00000088879	0	0	0	0	0	0
ENSMUSG00000088878	0	0	0	0	0	0
ENSMUSG00000107431	2	0	2	0	0	0
ENSMUSG00000069315	0	0	0	0	0	0
ENSMUSG00000094829	0	0	0	0	0	0
ENSMUSG00000094823	0	0	0	0	0	0
ENSMUSG00000021732	4	4	0	7	5	14
ENSMUSG00000094821	0	0	0	0	0	0
ENSMUSG00000094820	0	0	0	2	0	0
ENSMUSG00000094827	0	0	0	0	0	0
ENSMUSG00000094825	0	0	0	0	0	0
ENSMUSG00000111558	0	0	0	0	0	0
ENSMUSG00000111557	0	1	0	0	0	0
ENSMUSG00000096451	0	0	0	0	0	0
ENSMUSG00000111555	0	0	0	0	0	0
ENSMUSG00000111554	2	6	4	8	6	4
ENSMUSG00000111553	0	0	0	0	0	0
ENSMUSG00000029255	0	0	0	2	0	0
ENSMUSG00000111551	12	13	11	6	5	10
ENSMUSG00000111550	0	0	0	0	0	0
ENSMUSG00000038797	58	168	151	105	66	163
ENSMUSG00000050866	0	0	0	0	0	0
ENSMUSG00000050865	0	0	0	0	0	0
ENSMUSG00000038791	0	0	0	0	0	0
ENSMUSG00000059383	0	0	0	0	0	0
ENSMUSG00000059382	0	0	0	0	0	0
ENSMUSG00000035804	0	0	0	0	0	0
ENSMUSG00000026420	0	0	0	0	0	0
ENSMUSG00000109805	0	0	0	0	0	0
ENSMUSG00000105387	0	0	0	0	0	0
ENSMUSG00000105386	0	0	0	0	0	0
ENSMUSG00000105384	0	0	0	0	0	0
ENSMUSG00000114008	0	0	0	0	0	0
ENSMUSG00000105380	0	0	0	0	0	0
ENSMUSG00000114006	0	0	0	0	0	0
ENSMUSG00000112782	0	0	3	0	0	0
ENSMUSG00000114002	0	0	0	0	0	0
ENSMUSG00000105389	0	0	0	0	0	0
ENSMUSG00000114001	0	0	0	0	0	0
ENSMUSG00000059654	0	0	0	0	0	0
ENSMUSG00000091995	0	0	0	0	0	0
ENSMUSG00000091996	0	10	6	6	9	9
ENSMUSG00000091990	0	0	0	0	0	0
ENSMUSG00000025739	11	6	13	0	0	3

ENSMUSG00000114782	0	0	0	0	0	2
ENSMUSG00000054582	16	9	19	0	0	0
ENSMUSG00000104384	0	0	0	0	0	0
ENSMUSG00000104385	0	0	0	0	0	0
ENSMUSG00000104386	0	0	0	0	0	0
ENSMUSG00000104381	0	1	0	0	1	1
ENSMUSG00000104382	0	0	0	0	0	0
ENSMUSG00000056987	9	2	0	6	9	6
ENSMUSG00000109069	0	0	0	0	0	0
ENSMUSG00000044400	0	0	0	0	0	0
ENSMUSG00000028255	0	0	0	0	0	0
ENSMUSG00000110784	0	0	0	0	0	0
ENSMUSG00000110787	0	0	0	0	0	0
ENSMUSG00000110786	0	3	3	0	0	0
ENSMUSG00000110781	0	0	0	0	0	0
ENSMUSG00000110780	0	0	0	0	0	0
ENSMUSG00000110783	0	0	0	0	0	0
ENSMUSG00000110782	0	0	0	0	0	0
ENSMUSG00000110789	0	0	0	0	0	0
ENSMUSG00000110788	0	0	0	0	0	0
ENSMUSG00000054630	0	0	0	0	0	0
ENSMUSG00000027776	10	0	0	6	5	13
ENSMUSG00000027483	0	0	0	0	0	0
ENSMUSG00000027482	0	0	0	0	0	0
ENSMUSG00000027481	0	0	0	0	0	0
ENSMUSG00000027480	0	0	0	0	0	0
ENSMUSG00000027485	0	0	0	3	5	0
ENSMUSG00000027484	0	0	0	0	0	0
ENSMUSG00000027489	16	9	13	9	17	17
ENSMUSG00000080299	0	0	0	0	0	0
ENSMUSG00000080298	0	0	0	0	0	0
ENSMUSG00000001986	449	299	273	65	73	87
ENSMUSG00000053004	12	22	36	13	17	16
ENSMUSG00000053007	739	830	463	371	458	526
ENSMUSG00000084045	0	0	0	0	0	0
ENSMUSG00000074812	0	0	0	0	0	0
ENSMUSG00000074817	40	56	56	16	26	27
ENSMUSG00000090111	0	0	0	0	0	0
ENSMUSG00000090116	0	0	0	0	0	0
ENSMUSG00000110041	0	0	0	0	0	0
ENSMUSG00000113847	0	0	0	0	0	0
ENSMUSG00000113846	0	3	0	3	0	0
ENSMUSG00000113845	2	0	6	0	3	0
ENSMUSG00000098345	0	0	0	0	0	0
ENSMUSG00000113843	7	6	6	8	6	8
ENSMUSG00000098348	0	0	0	0	0	0
ENSMUSG00000095818	0	0	0	0	0	0
ENSMUSG00000095814	0	0	0	0	0	0
ENSMUSG00000086923	0	0	0	0	0	0
ENSMUSG00000086927	2	0	0	0	0	0
ENSMUSG00000086926	10	8	12	7	6	9
ENSMUSG00000045467	12	19	11	7	12	18
ENSMUSG00000045466	115	162	163	158	141	147
ENSMUSG00000045463	0	0	0	0	0	0
ENSMUSG00000095697	0	0	0	0	0	0
ENSMUSG00000095696	0	0	0	0	0	0
ENSMUSG00000052353	86	31	79	0	0	0
ENSMUSG00000095691	0	0	0	0	0	0

ENSMUSG00000028931	69	73	119	53	64	87
ENSMUSG00000095812	14	18	10	23	15	19
ENSMUSG00000047197	0	0	8	0	5	2
ENSMUSG00000100557	0	0	0	0	0	0
ENSMUSG00000100556	0	0	0	0	0	0
ENSMUSG00000094128	1	1	1	0	1	0
ENSMUSG00000094129	0	0	0	0	0	0
ENSMUSG00000094125	9	5	8	7	5	10
ENSMUSG00000042118	0	0	0	3	0	0
ENSMUSG00000094123	0	0	0	0	0	0
ENSMUSG00000094120	0	0	0	0	0	0
ENSMUSG00000100553	0	0	0	2	0	0
ENSMUSG00000108877	0	0	0	0	0	0
ENSMUSG00000108875	0	0	0	0	0	0
ENSMUSG00000081658	0	0	0	0	0	0
ENSMUSG00000081659	0	2	0	0	0	0
ENSMUSG00000081654	0	0	7	0	0	0
ENSMUSG00000081652	0	0	0	0	0	0
ENSMUSG00000073899	2	0	0	0	0	0
ENSMUSG00000073898	0	0	0	0	0	0
ENSMUSG00000077730	0	0	0	0	0	0
ENSMUSG00000100559	0	0	0	0	0	0
ENSMUSG00000073893	3	0	0	3	0	0
ENSMUSG00000077735	0	0	0	0	0	0
ENSMUSG00000073897	0	0	0	0	0	0
ENSMUSG00000073896	0	1	0	0	0	0
ENSMUSG00000100831	0	0	0	0	0	0
ENSMUSG00000100836	0	0	0	0	0	0
ENSMUSG00000055515	0	0	0	0	0	0
ENSMUSG00000017453	18	8	12	9	14	20
ENSMUSG00000100835	0	0	0	0	0	0
ENSMUSG00000090801	0	0	0	0	0	0
ENSMUSG00000090806	0	0	0	0	0	0
ENSMUSG00000090808	0	0	0	0	0	0
ENSMUSG00000108879	0	0	0	0	0	0
ENSMUSG00000048373	4	0	0	0	2	0
ENSMUSG00000092409	1	0	0	1	0	0
ENSMUSG00000092408	0	0	0	0	0	0
ENSMUSG00000048377	0	0	0	0	0	0
ENSMUSG00000092402	0	2	0	0	0	0
ENSMUSG00000092401	0	0	0	0	0	0
ENSMUSG00000092404	0	0	0	0	0	0
ENSMUSG00000105757	0	0	0	0	0	0
ENSMUSG00000067577	0	0	0	10	8	6
ENSMUSG00000051877	0	0	0	0	0	0
ENSMUSG00000003555	4	0	0	0	4	0
ENSMUSG00000043267	0	0	0	0	0	2
ENSMUSG00000043441	0	0	0	0	0	0
ENSMUSG00000092496	0	0	0	0	0	0
ENSMUSG00000051879	0	0	0	0	0	0
ENSMUSG00000082352	2	0	0	0	0	0
ENSMUSG00000082353	0	0	0	0	0	0
ENSMUSG00000082350	4	0	0	0	0	0
ENSMUSG00000082351	0	0	0	0	0	0
ENSMUSG00000076067	1	0	0	0	0	0
ENSMUSG00000076063	0	0	0	0	0	0
ENSMUSG00000005891	0	0	0	0	0	0
ENSMUSG00000005892	0	0	0	0	0	0

ENSMUSG00000099753	0	0	0	0	0	0
ENSMUSG00000032452	32	41	34	54	63	50
ENSMUSG00000032451	0	0	0	0	0	0
ENSMUSG00000032454	0	3	0	0	0	0
ENSMUSG00000071491	0	0	0	0	0	0
ENSMUSG00000071490	0	0	0	0	0	0
ENSMUSG00000071494	8	8	4	7	6	3
ENSMUSG00000100093	0	0	0	0	0	0
ENSMUSG00000097596	0	0	0	0	0	0
ENSMUSG00000035407	100	50	119	54	43	46
ENSMUSG00000043993	125	110	109	119	100	118
ENSMUSG00000074965	0	0	0	0	0	0
ENSMUSG00000093864	0	0	0	0	0	0
ENSMUSG00000072763	56	48	52	27	21	29
ENSMUSG00000074966	0	0	0	0	0	0
ENSMUSG00000087724	0	0	0	0	0	0
ENSMUSG00000087726	0	0	0	0	0	0
ENSMUSG00000087720	0	0	0	0	0	0
ENSMUSG00000109464	0	0	0	0	0	0
ENSMUSG00000064429	0	0	0	0	0	0
ENSMUSG00000083409	0	0	0	0	0	0
ENSMUSG00000083401	0	0	0	0	0	0
ENSMUSG00000083403	0	0	0	0	0	0
ENSMUSG00000083402	0	0	0	0	0	0
ENSMUSG00000083404	0	0	0	0	0	0
ENSMUSG00000083407	0	0	0	0	0	0
ENSMUSG00000083406	2	0	0	0	0	0
ENSMUSG00000079563	11	6	0	0	4	0
ENSMUSG00000079560	9	0	0	4	14	15
ENSMUSG00000064983	0	0	0	0	0	0
ENSMUSG00000083508	0	1	0	0	0	0
ENSMUSG00000087897	0	0	0	0	0	0
ENSMUSG00000087895	0	0	0	0	0	0
ENSMUSG00000087892	0	0	1	0	1	0
ENSMUSG00000087893	0	0	0	0	0	0
ENSMUSG00000087890	0	0	0	0	0	0
ENSMUSG00000045518	0	0	3	3	0	5
ENSMUSG00000087898	2	0	0	0	0	3
ENSMUSG00000064036	0	0	0	0	0	0
ENSMUSG00000064039	3	7	3	0	0	0
ENSMUSG00000071237	2	3	1	2	3	3
ENSMUSG00000071235	0	0	0	0	0	0
ENSMUSG00000071234	0	0	0	3	0	0
ENSMUSG00000107435	0	6	7	0	4	2
ENSMUSG00000071230	0	0	0	0	0	0
ENSMUSG00000033460	141	179	177	76	90	125
ENSMUSG00000065787	0	0	0	0	0	0
ENSMUSG00000112390	0	0	0	0	0	0
ENSMUSG00000065788	0	0	0	0	0	0
ENSMUSG00000061208	0	0	0	0	0	0
ENSMUSG00000111728	0	0	0	0	0	0
ENSMUSG00000064788	0	0	0	0	0	0
ENSMUSG00000085761	12	12	14	4	0	11
ENSMUSG00000064781	0	0	0	0	0	0
ENSMUSG00000064783	0	0	0	0	0	0
ENSMUSG00000064782	0	0	0	0	0	0
ENSMUSG00000107385	0	0	0	0	0	0
ENSMUSG00000107384	0	0	0	0	0	0

ENSMUSG00000107386	0	0	0	0	0	0
ENSMUSG00000107380	0	0	0	0	0	0
ENSMUSG00000079387	0	0	0	0	0	0
ENSMUSG00000079386	0	5	5	0	6	4
ENSMUSG00000079383	4	23	8	0	7	10
ENSMUSG00000079382	0	0	0	0	0	0
ENSMUSG00000068008	0	0	0	0	0	0
ENSMUSG00000068009	0	0	0	0	0	0
ENSMUSG00000079389	0	0	0	0	0	0
ENSMUSG00000079388	8	2	12	8	6	0
ENSMUSG00000085491	0	0	0	0	0	0
ENSMUSG00000058952	4	4	30	0	4	5
ENSMUSG00000021966	0	2	0	0	0	3
ENSMUSG00000073400	11	0	6	3	0	6
ENSMUSG00000073408	0	0	0	0	0	0
ENSMUSG00000073803	0	3	0	0	0	0
ENSMUSG00000102269	9	11	22	16	10	24
ENSMUSG00000102268	0	0	4	0	0	4
ENSMUSG00000102265	0	0	0	0	0	0
ENSMUSG00000102264	0	0	0	0	0	0
ENSMUSG00000102266	8	16	15	7	2	10
ENSMUSG00000102261	3	3	5	5	0	3
ENSMUSG00000102263	0	0	0	0	0	0
ENSMUSG00000102262	0	0	0	0	0	0
ENSMUSG00000038555	18	10	14	7	19	34
ENSMUSG00000034275	708	623	789	549	708	769
ENSMUSG00000088085	0	0	0	0	0	0
ENSMUSG00000073924	0	0	0	3	0	0
ENSMUSG00000058349	2	0	0	0	0	0
ENSMUSG00000096775	15	13	24	0	8	20
ENSMUSG00000096774	0	0	0	0	0	0
ENSMUSG00000096777	0	0	0	0	0	0
ENSMUSG00000096776	0	0	0	0	0	0
ENSMUSG00000096771	0	0	2	1	0	0
ENSMUSG00000096770	0	0	0	0	0	0
ENSMUSG00000096773	0	0	0	0	0	0
ENSMUSG00000096772	0	0	0	0	0	0
ENSMUSG00000031179	0	0	0	0	0	0
ENSMUSG00000031170	2	3	0	0	0	0
ENSMUSG00000031173	0	0	0	0	0	0
ENSMUSG00000059862	0	0	0	2	0	0
ENSMUSG00000063173	0	0	0	0	0	0
ENSMUSG00000063177	0	0	0	0	0	0
ENSMUSG00000063176	0	0	0	1	0	0
ENSMUSG00000088339	0	0	0	0	0	0
ENSMUSG00000102840	0	0	0	0	0	0
ENSMUSG00000102842	0	0	0	0	0	0
ENSMUSG00000102847	0	0	0	0	0	0
ENSMUSG00000086151	0	0	0	0	0	0
ENSMUSG00000102849	0	0	0	0	0	0
ENSMUSG00000105816	0	0	0	0	0	0
ENSMUSG00000067698	2	0	0	6	0	5
ENSMUSG00000105812	0	0	0	0	0	0
ENSMUSG00000105811	0	0	0	0	0	0
ENSMUSG00000086153	0	0	0	0	0	0
ENSMUSG00000110692	0	0	0	0	0	0
ENSMUSG00000111893	0	0	0	0	0	0
ENSMUSG00000048938	0	0	0	0	0	0

ENSMUSG00000104149	3	1	0	1	0	0
ENSMUSG00000048939	4	0	0	0	3	4
ENSMUSG00000104142	0	0	2	0	0	0
ENSMUSG00000105634	0	0	0	0	0	0
ENSMUSG00000104146	0	0	0	0	0	0
ENSMUSG00000104147	0	0	0	0	0	0
ENSMUSG00000104144	0	0	0	0	0	0
ENSMUSG00000104145	119	93	88	87	100	75
ENSMUSG00000108995	0	0	0	0	0	0
ENSMUSG00000108997	0	0	0	0	0	0
ENSMUSG00000028845	9	6	13	14	9	6
ENSMUSG00000108993	0	2	0	0	2	0
ENSMUSG00000108992	0	0	0	0	0	0
ENSMUSG00000091204	0	0	0	0	0	0
ENSMUSG00000091205	0	0	0	0	0	0
ENSMUSG00000091206	0	0	0	0	0	0
ENSMUSG00000108998	0	0	0	0	0	0
ENSMUSG00000097599	0	0	0	0	0	0
ENSMUSG00000015721	1	3	2	1	2	2
ENSMUSG00000114904	0	0	0	0	0	0
ENSMUSG00000030226	0	0	0	0	0	0
ENSMUSG00000030228	2	2	0	9	2	9
ENSMUSG00000110819	0	0	0	0	0	0
ENSMUSG00000110815	11	6	8	2	8	6
ENSMUSG00000110814	0	0	0	0	0	0
ENSMUSG00000110811	0	0	0	0	2	0
ENSMUSG00000110810	0	0	0	0	0	0
ENSMUSG00000110813	0	0	0	0	0	0
ENSMUSG00000110812	1	0	0	0	0	0
ENSMUSG00000104858	0	0	0	0	0	0
ENSMUSG00000104859	0	3	0	0	0	0
ENSMUSG00000093920	0	0	0	0	0	0
ENSMUSG00000112559	0	0	0	0	0	0
ENSMUSG00000082433	5	8	10	2	4	5
ENSMUSG00000088255	0	0	0	0	0	0
ENSMUSG00000088257	0	0	0	0	0	0
ENSMUSG00000088258	0	0	0	0	0	0
ENSMUSG00000058046	0	0	0	0	0	0
ENSMUSG00000021536	69	37	84	8	10	10
ENSMUSG00000022494	7	0	2	2	10	4
ENSMUSG00000022491	0	0	0	0	0	0
ENSMUSG00000095458	0	0	1	0	0	0
ENSMUSG00000095455	0	0	0	0	0	0
ENSMUSG00000095456	86	0	0	0	0	0
ENSMUSG00000095450	0	1	0	1	1	0
ENSMUSG00000095452	0	0	0	0	0	0
ENSMUSG00000012396	0	0	0	0	0	0
ENSMUSG00000091272	10	0	34	4	0	2
ENSMUSG00000039098	0	0	8	0	0	0
ENSMUSG00000094328	0	0	0	0	0	0
ENSMUSG00000039097	0	0	2	0	0	4
ENSMUSG00000094321	0	0	0	0	0	0
ENSMUSG00000039095	0	0	0	0	4	0
ENSMUSG00000009092	29	18	36	19	14	30
ENSMUSG00000009093	0	0	0	0	0	0
ENSMUSG00000009097	8	15	8	14	6	0
ENSMUSG00000029869	178	112	159	47	38	65
ENSMUSG00000029868	6	1	0	0	0	0

ENSMUSG00000029867	0	0	0	0	0	0
ENSMUSG00000029866	0	0	0	6	0	0
ENSMUSG00000029865	0	0	0	0	0	0
ENSMUSG00000020383	0	0	0	0	2	0
ENSMUSG00000106170	0	0	0	0	0	0
ENSMUSG00000106173	0	0	0	0	0	0
ENSMUSG00000106172	0	0	0	0	0	0
ENSMUSG00000106174	0	0	0	0	0	0
ENSMUSG00000106176	0	0	0	0	0	0
ENSMUSG00000106283	0	0	0	0	0	0
ENSMUSG00000106282	0	0	0	0	0	0
ENSMUSG00000106280	0	0	0	0	0	0
ENSMUSG00000106286	0	0	0	0	0	0
ENSMUSG00000106285	2	0	0	0	0	0
ENSMUSG00000106284	0	0	0	0	0	0
ENSMUSG00000092240	0	0	0	0	0	0
ENSMUSG00000092242	0	0	0	0	0	0
ENSMUSG00000092245	0	0	0	3	0	0
ENSMUSG00000092244	0	0	0	0	0	0
ENSMUSG00000092247	2	4	4	3	2	6
ENSMUSG00000102413	0	0	0	0	0	0
ENSMUSG00000112505	0	0	2	2	3	2
ENSMUSG00000112504	0	0	0	0	0	0
ENSMUSG00000024768	0	0	0	0	0	0
ENSMUSG00000112501	0	2	0	0	0	0
ENSMUSG00000112500	0	0	0	0	4	2
ENSMUSG00000112503	2	1	0	1	1	0
ENSMUSG00000112502	3	5	5	6	4	7
ENSMUSG00000112509	0	0	0	0	0	0
ENSMUSG00000113012	0	0	2	0	0	0
ENSMUSG00000020659	216	275	164	138	225	228
ENSMUSG00000020651	0	4	0	0	0	0
ENSMUSG00000105921	0	0	0	0	0	0
ENSMUSG00000105920	0	0	0	0	0	0
ENSMUSG00000105926	0	5	5	0	7	2
ENSMUSG00000113674	0	0	0	0	0	0
ENSMUSG00000113675	0	0	0	0	0	1
ENSMUSG00000113676	0	0	0	0	0	0
ENSMUSG00000113677	0	0	0	0	0	0
ENSMUSG00000113671	12	2	7	6	0	12
ENSMUSG00000113672	0	0	0	0	0	0
ENSMUSG00000113673	3	0	0	0	0	0
ENSMUSG00000095125	0	0	0	0	0	0
ENSMUSG00000113678	0	0	0	0	0	0
ENSMUSG00000097148	16	21	18	20	21	26
ENSMUSG00000105488	0	0	0	0	0	0
ENSMUSG00000109106	0	0	0	0	0	0
ENSMUSG00000096897	0	0	0	0	0	0
ENSMUSG00000096898	0	0	0	0	0	0
ENSMUSG00000096899	0	0	0	0	0	1
ENSMUSG00000114903	0	0	0	0	0	0
ENSMUSG00000114902	5	2	8	17	6	12
ENSMUSG00000114901	1	0	0	0	0	0
ENSMUSG00000114900	0	0	0	0	0	0
ENSMUSG00000025380	8	11	12	5	13	12
ENSMUSG00000106824	11	18	11	2	10	9
ENSMUSG00000070524	0	3	0	0	0	0
ENSMUSG00000106821	0	0	0	0	0	0

ENSMUSG00000106820	11	15	10	0	4	2
ENSMUSG00000070529	0	0	0	0	0	0
ENSMUSG00000106829	0	0	0	0	0	0
ENSMUSG00000082103	0	0	0	0	0	0
ENSMUSG00000105732	0	0	0	0	0	0
ENSMUSG00000105730	0	0	0	0	0	0
ENSMUSG00000105731	0	0	0	0	0	0
ENSMUSG00000105737	0	0	0	0	0	0
ENSMUSG00000105734	0	0	0	0	0	0
ENSMUSG00000105735	0	0	0	0	2	0
ENSMUSG00000105739	5	0	0	0	0	0
ENSMUSG00000096235	0	0	0	0	0	0
ENSMUSG00000096236	0	0	0	0	0	0
ENSMUSG00000096237	0	0	0	61	0	0
ENSMUSG00000096230	0	0	0	0	0	0
ENSMUSG00000096231	0	0	0	0	0	0
ENSMUSG00000096239	1	1	1	0	0	0
ENSMUSG00000095121	0	0	0	0	0	0
ENSMUSG00000064740	0	0	0	0	0	0
ENSMUSG00000113821	0	0	0	0	0	0
ENSMUSG00000055826	0	0	2	2	2	0
ENSMUSG00000055827	0	0	0	0	0	0
ENSMUSG00000055820	0	0	0	0	0	0
ENSMUSG00000111741	0	0	0	0	0	0
ENSMUSG00000057464	0	0	0	0	0	0
ENSMUSG00000057465	2	0	0	2	0	0
ENSMUSG00000057461	0	0	0	0	0	0
ENSMUSG00000088420	0	0	0	0	0	0
ENSMUSG00000025964	129	78	94	133	139	232
ENSMUSG00000110028	0	0	0	0	0	0
ENSMUSG00000025963	0	0	0	0	0	0
ENSMUSG00000025961	6	15	13	10	5	12
ENSMUSG00000104461	0	0	0	0	0	0
ENSMUSG00000104460	0	0	0	0	0	0
ENSMUSG00000040998	48	12	26	0	14	20
ENSMUSG00000104462	0	0	3	3	2	0
ENSMUSG00000108766	1	8	6	4	4	4
ENSMUSG00000104464	0	0	0	0	0	0
ENSMUSG00000108764	0	0	0	0	0	0
ENSMUSG00000108765	0	0	0	0	0	0
ENSMUSG00000104468	0	0	0	0	0	0
ENSMUSG00000108768	0	0	0	0	0	0
ENSMUSG00000044678	0	9	23	0	0	0
ENSMUSG00000050241	4	6	10	5	0	5
ENSMUSG00000022208	8	4	15	5	4	8
ENSMUSG00000081576	0	0	0	0	0	0
ENSMUSG00000022209	0	0	0	3	0	0
ENSMUSG00000110594	0	2	0	0	0	0
ENSMUSG00000110596	0	0	0	0	0	0
ENSMUSG00000110597	0	0	0	0	0	0
ENSMUSG00000110590	0	0	0	0	0	0
ENSMUSG00000110592	8	19	13	8	10	5
ENSMUSG00000027520	53	68	24	18	16	10
ENSMUSG00000027525	15	7	18	18	9	23
ENSMUSG00000084175	0	0	0	0	0	0
ENSMUSG00000076323	0	0	0	0	0	0
ENSMUSG00000046295	21	11	19	8	17	16
ENSMUSG00000074995	0	0	0	0	0	0

ENSMUSG00000074997	0	0	0	0	0	0
ENSMUSG00000074996	0	0	0	0	0	0
ENSMUSG00000074991	0	0	0	0	0	0
ENSMUSG00000106645	0	0	0	0	0	0
ENSMUSG00000066061	0	0	0	0	0	0
ENSMUSG00000074999	0	7	7	4	0	7
ENSMUSG00000083383	0	0	0	0	0	0
ENSMUSG00000090302	0	0	0	0	0	0
ENSMUSG00000081288	0	0	0	0	0	0
ENSMUSG00000095156	0	0	0	0	0	3
ENSMUSG00000095150	0	0	0	0	0	0
ENSMUSG00000095151	0	0	1	1	0	0
ENSMUSG00000090304	0	0	0	0	0	0
ENSMUSG00000095153	0	0	0	0	0	0
ENSMUSG00000081280	0	0	0	0	0	0
ENSMUSG00000081283	0	0	0	0	0	0
ENSMUSG00000083381	0	0	0	0	0	0
ENSMUSG00000081285	5	4	3	1	7	2
ENSMUSG00000081287	3	0	0	0	0	0
ENSMUSG00000081286	0	0	0	0	0	0
ENSMUSG00000015437	8	8	9	0	0	0
ENSMUSG00000088733	0	0	0	0	0	0
ENSMUSG00000080487	0	0	0	0	1	0
ENSMUSG00000042632	246	278	300	285	283	354
ENSMUSG00000086887	0	0	0	0	0	0
ENSMUSG00000086884	0	0	0	0	0	0
ENSMUSG00000086882	0	0	0	0	0	0
ENSMUSG00000052142	59	54	73	39	25	22
ENSMUSG00000028876	0	0	0	0	0	0
ENSMUSG00000028874	9	12	77	13	5	12
ENSMUSG00000073173	0	0	0	0	0	0
ENSMUSG00000077539	0	0	0	0	0	0
ENSMUSG00000073177	0	0	0	0	0	0
ENSMUSG00000080738	0	0	0	0	0	0
ENSMUSG00000080737	0	0	0	0	0	0
ENSMUSG00000080734	0	0	0	0	0	0
ENSMUSG00000080735	0	0	0	0	0	0
ENSMUSG00000090436	4	0	0	0	0	0
ENSMUSG00000080733	0	0	0	0	0	0
ENSMUSG00000080730	0	0	0	0	0	0
ENSMUSG00000093651	0	0	2	2	0	3
ENSMUSG00000093652	0	0	0	0	0	0
ENSMUSG00000110022	0	0	0	0	0	0
ENSMUSG00000078850	2	2	0	0	0	1
ENSMUSG00000086771	0	0	0	0	0	0
ENSMUSG00000086775	0	4	0	0	0	0
ENSMUSG00000092579	0	0	0	0	0	0
ENSMUSG00000082622	0	3	0	0	0	0
ENSMUSG00000110023	0	0	0	0	0	0
ENSMUSG00000098688	0	0	0	0	0	0
ENSMUSG00000098687	0	0	0	0	0	0
ENSMUSG00000031376	0	5	0	6	30	12
ENSMUSG00000047655	0	0	0	0	0	0
ENSMUSG00000031374	5	0	0	0	0	0
ENSMUSG00000031372	0	0	0	0	0	2
ENSMUSG00000047652	0	0	0	0	0	0
ENSMUSG00000073255	0	0	0	0	0	0
ENSMUSG00000092811	0	0	0	0	0	0

ENSMUSG00000110109	0	0	0	0	0	2
ENSMUSG00000049723	43	46	56	13	11	20
ENSMUSG00000049721	7	0	3	7	0	0
ENSMUSG00000060583	2	0	0	0	0	0
ENSMUSG00000077507	0	0	0	0	0	0
ENSMUSG00000076489	0	0	0	1	0	0
ENSMUSG00000097888	12	13	17	11	15	17
ENSMUSG00000077504	0	0	0	0	0	0
ENSMUSG00000077503	0	0	0	0	0	0
ENSMUSG00000077502	0	0	0	0	0	0
ENSMUSG00000077501	0	0	0	0	0	0
ENSMUSG00000040569	37	36	31	24	26	63
ENSMUSG00000067909	0	0	0	0	0	0
ENSMUSG00000076453	0	0	0	0	0	0
ENSMUSG00000076450	0	2	1	0	0	0
ENSMUSG00000076457	0	0	0	0	0	0
ENSMUSG00000082259	0	0	0	0	0	0
ENSMUSG00000082256	0	0	0	0	0	0
ENSMUSG00000082255	0	0	0	0	0	0
ENSMUSG00000082254	0	0	0	0	0	0
ENSMUSG00000082250	0	0	0	0	0	0
ENSMUSG00000048236	0	0	0	0	0	0
ENSMUSG00000048231	0	0	0	0	0	0
ENSMUSG00000099799	0	0	0	0	0	0
ENSMUSG00000099794	0	0	0	0	0	0
ENSMUSG00000099795	0	0	0	0	0	0
ENSMUSG00000099792	0	0	0	0	0	0
ENSMUSG00000099793	0	0	0	0	0	0
ENSMUSG00000099790	0	0	0	0	0	0
ENSMUSG00000099791	0	0	0	0	0	0
ENSMUSG00000076485	0	0	0	0	0	0
ENSMUSG00000083877	0	0	0	0	0	0
ENSMUSG00000064507	0	0	0	0	0	0
ENSMUSG00000099248	0	0	0	0	0	0
ENSMUSG00000099243	4	5	3	3	5	3
ENSMUSG00000099247	0	0	0	0	0	0
ENSMUSG00000030745	66	67	138	37	16	34
ENSMUSG00000070880	3	0	0	0	0	0
ENSMUSG00000083618	0	0	14	14	0	12
ENSMUSG00000040380	5	0	0	0	0	0
ENSMUSG00000037007	437	468	427	526	495	610
ENSMUSG00000083613	0	0	0	0	0	0
ENSMUSG00000083615	0	0	0	0	0	0
ENSMUSG00000083617	0	0	0	0	0	0
ENSMUSG00000000094	0	0	0	0	0	0
ENSMUSG00000100753	0	0	0	0	0	0
ENSMUSG00000100752	0	0	0	0	0	0
ENSMUSG00000100751	0	0	0	0	0	0
ENSMUSG00000100757	0	0	0	0	0	0
ENSMUSG00000100756	0	0	0	0	0	0
ENSMUSG00000100754	0	0	0	0	0	0
ENSMUSG00000100758	0	0	0	0	0	0
ENSMUSG00000094267	0	1	1	1	1	0
ENSMUSG00000112357	4	16	4	10	13	20
ENSMUSG00000112350	0	0	0	0	0	0
ENSMUSG00000104749	0	0	0	0	0	0
ENSMUSG00000102445	0	0	0	0	0	0
ENSMUSG00000102447	0	1	0	0	0	0

ENSMUSG00000039133	29	8	14	10	1	6
ENSMUSG00000102443	0	1	0	0	0	0
ENSMUSG00000102442	0	0	0	0	0	0
ENSMUSG00000075101	0	0	0	0	0	0
ENSMUSG00000079481	624	560	853	296	414	542
ENSMUSG00000075102	0	0	0	0	0	0
ENSMUSG00000075105	0	0	0	0	0	0
ENSMUSG00000075104	0	0	0	0	0	0
ENSMUSG00000075107	0	0	0	0	0	0
ENSMUSG00000087919	0	0	0	0	0	0
ENSMUSG00000087911	0	0	0	0	0	0
ENSMUSG00000087910	0	0	0	0	0	0
ENSMUSG00000041380	0	0	0	0	0	0
ENSMUSG00000087916	0	0	0	0	0	0
ENSMUSG00000087914	2	0	0	0	0	0
ENSMUSG00000099294	0	0	0	0	0	0
ENSMUSG00000110968	0	0	2	2	0	0
ENSMUSG00000097630	0	0	0	0	0	0
ENSMUSG00000097083	0	0	0	0	0	0
ENSMUSG00000085515	0	0	0	0	0	0
ENSMUSG00000085516	0	0	4	3	5	2
ENSMUSG00000085510	0	0	0	0	0	0
ENSMUSG00000085512	0	0	8	12	9	5
ENSMUSG00000064596	0	0	0	0	0	0
ENSMUSG00000054510	0	9	0	2	0	0
ENSMUSG00000064591	0	0	0	0	0	0
ENSMUSG00000100141	10	13	8	29	23	30
ENSMUSG00000023039	50	33	11	0	0	2
ENSMUSG00000101938	0	0	0	0	0	0
ENSMUSG00000061414	209	212	178	201	229	310
ENSMUSG00000088643	1	2	0	0	1	2
ENSMUSG00000088640	0	0	0	0	0	0
ENSMUSG00000088646	0	0	0	0	0	0
ENSMUSG00000101931	0	0	0	0	0	0
ENSMUSG00000088644	0	0	0	0	0	0
ENSMUSG00000101933	0	0	0	0	0	0
ENSMUSG00000100359	0	0	0	0	0	0
ENSMUSG00000090637	0	0	0	0	0	2
ENSMUSG00000107198	0	0	0	0	0	0
ENSMUSG00000107199	0	0	0	0	0	0
ENSMUSG00000107192	0	0	0	0	0	0
ENSMUSG00000107193	0	0	0	0	0	0
ENSMUSG00000103471	0	0	0	0	0	0
ENSMUSG00000103470	0	0	0	0	0	0
ENSMUSG00000103477	0	0	0	0	0	2
ENSMUSG00000103475	6	4	4	5	7	7
ENSMUSG00000103474	0	0	0	0	0	0
ENSMUSG00000027654	30	23	26	6	14	14
ENSMUSG00000065242	0	0	0	0	0	0
ENSMUSG00000103479	0	0	0	0	0	0
ENSMUSG00000103478	0	0	0	0	0	0
ENSMUSG00000065244	0	0	0	0	0	0
ENSMUSG00000090635	0	0	0	0	0	0
ENSMUSG00000063810	879	1039	736	600	662	820
ENSMUSG00000027157	0	0	0	0	0	0
ENSMUSG00000031398	152	96	119	50	70	59
ENSMUSG00000097886	0	0	0	0	0	0
ENSMUSG00000074768	0	0	0	0	0	2

ENSMUSG00000112296	0	0	0	0	0	0
ENSMUSG00000024565	0	0	0	0	0	0
ENSMUSG00000092148	0	0	0	0	0	0
ENSMUSG00000099032	58	51	55	13	14	25
ENSMUSG00000114093	0	0	0	0	0	0
ENSMUSG00000109898	0	0	0	0	0	0
ENSMUSG00000110260	0	0	0	0	0	0
ENSMUSG00000038692	49	18	32	27	38	51
ENSMUSG00000113083	0	0	0	0	0	0
ENSMUSG00000097301	0	2	0	0	0	0
ENSMUSG00000038691	0	0	0	0	0	0
ENSMUSG00000097307	0	0	2	0	0	0
ENSMUSG00000073234	0	0	0	0	0	0
ENSMUSG00000097308	0	0	0	0	0	6
ENSMUSG00000084000	0	0	0	0	0	0
ENSMUSG00000062148	0	0	0	0	0	0
ENSMUSG00000091382	0	0	0	0	0	0
ENSMUSG00000020712	0	5	0	0	0	0
ENSMUSG00000090631	7	4	7	8	5	4
ENSMUSG00000082198	0	0	0	0	0	0
ENSMUSG00000044248	0	0	0	0	0	0
ENSMUSG00000054310	1	0	0	0	0	0
ENSMUSG00000103573	0	0	0	0	0	0
ENSMUSG00000074115	0	0	6	0	0	0
ENSMUSG00000025746	40	40	74	5	5	0
ENSMUSG00000105131	0	0	0	0	0	0
ENSMUSG00000105132	0	0	0	0	0	0
ENSMUSG00000105133	0	0	0	0	0	0
ENSMUSG00000105139	45	46	43	10	14	26
ENSMUSG00000110730	0	7	6	0	0	4
ENSMUSG00000066936	0	0	0	0	0	0
ENSMUSG00000058176	1	1	0	2	1	0
ENSMUSG00000099442	0	0	0	0	0	0
ENSMUSG00000062638	0	0	0	0	0	0
ENSMUSG00000086591	16	14	29	13	16	14
ENSMUSG00000067242	104	189	117	305	284	351
ENSMUSG00000108561	0	0	0	0	0	3
ENSMUSG00000007480	0	0	0	0	0	2
ENSMUSG00000050122	8	7	0	0	0	0
ENSMUSG00000108565	0	0	0	0	0	0
ENSMUSG00000104269	0	0	0	0	0	0
ENSMUSG00000108567	0	1	0	0	0	0
ENSMUSG00000104267	0	0	0	0	0	0
ENSMUSG00000108569	12	15	12	8	6	5
ENSMUSG00000104265	0	0	0	0	0	0
ENSMUSG00000050128	0	0	0	0	0	0
ENSMUSG00000104260	0	0	0	0	0	0
ENSMUSG00000090638	0	0	0	0	0	0
ENSMUSG00000060738	0	0	3	0	0	0
ENSMUSG00000101163	1	1	1	1	0	1
ENSMUSG00000060735	5	2	0	0	0	2
ENSMUSG00000101167	24	5	27	6	4	13
ENSMUSG00000010760	0	0	0	0	0	0
ENSMUSG00000110192	0	0	0	0	0	0
ENSMUSG00000110193	0	2	0	3	0	0
ENSMUSG00000064921	0	1	0	2	0	0
ENSMUSG00000091071	22	10	22	12	10	12
ENSMUSG00000091077	0	0	0	0	0	0

ENSMUSG00000091076	0	0	0	0	0	0
ENSMUSG00000106180	0	3	2	0	3	3
ENSMUSG00000056203	7	17	2	4	12	12
ENSMUSG00000095792	0	1	0	0	0	0
ENSMUSG00000095793	0	0	0	0	0	0
ENSMUSG00000095791	0	1	1	0	1	0
ENSMUSG00000095796	0	0	0	0	0	0
ENSMUSG00000095797	0	0	0	0	0	0
ENSMUSG00000095799	2	1	0	0	0	0
ENSMUSG00000096674	0	0	0	0	0	0
ENSMUSG00000022021	117	103	93	21	25	65
ENSMUSG00000022026	0	0	0	0	0	0
ENSMUSG00000050395	0	8	4	0	0	0
ENSMUSG00000090671	0	1	3	0	2	0
ENSMUSG00000106183	0	0	0	0	0	0
ENSMUSG00000090675	2	3	2	0	4	1
ENSMUSG00000042388	0	9	0	4	7	0
ENSMUSG00000099207	0	0	0	0	0	0
ENSMUSG00000042386	0	0	0	0	0	0
ENSMUSG00000113802	0	0	0	0	0	0
ENSMUSG00000086576	0	0	0	0	0	0
ENSMUSG00000086577	0	0	0	0	0	0
ENSMUSG00000086573	4	2	8	4	0	0
ENSMUSG00000021751	27	32	41	13	9	6
ENSMUSG00000094840	6	0	9	9	0	0
ENSMUSG00000093329	1	0	0	1	1	1
ENSMUSG00000093322	0	0	1	0	0	0
ENSMUSG00000021758	16	10	18	21	18	23
ENSMUSG00000049528	0	0	0	0	0	0
ENSMUSG00000081837	0	0	0	0	0	0
ENSMUSG00000052435	0	0	0	2	0	0
ENSMUSG00000036500	0	0	0	0	0	0
ENSMUSG00000052430	27	28	20	12	16	10
ENSMUSG00000109286	0	0	0	0	0	0
ENSMUSG00000007209	0	0	0	0	0	0
ENSMUSG00000081839	0	0	0	0	0	0
ENSMUSG00000112715	0	0	0	0	0	0
ENSMUSG00000112716	0	0	0	0	0	0
ENSMUSG00000112717	0	0	0	0	0	0
ENSMUSG00000112711	0	0	0	0	0	0
ENSMUSG00000112712	0	0	0	0	0	0
ENSMUSG00000049291	0	0	0	0	0	0
ENSMUSG00000112718	0	0	0	0	0	0
ENSMUSG00000112719	6	6	3	3	11	9
ENSMUSG00000106329	38	30	26	25	18	13
ENSMUSG00000106320	153	187	144	223	205	231
ENSMUSG00000106321	1	3	0	4	3	0
ENSMUSG00000106325	0	0	0	0	0	0
ENSMUSG00000029235	0	0	0	0	0	0
ENSMUSG00000083747	0	0	0	0	0	0
ENSMUSG00000047261	12	7	7	0	3	0
ENSMUSG00000088511	0	0	0	0	0	0
ENSMUSG00000109319	0	0	0	0	0	0
ENSMUSG00000109311	0	0	2	0	0	0
ENSMUSG00000051314	7	7	7	0	7	13
ENSMUSG00000109313	0	0	0	0	0	0
ENSMUSG00000039013	0	3	0	0	0	0
ENSMUSG00000109316	2	0	0	0	0	0

ENSMUSG00000031182	0	2	0	0	0	0
ENSMUSG00000096915	1	0	0	0	0	0
ENSMUSG00000096914	0	0	0	0	0	0
ENSMUSG00000096911	0	0	0	0	0	0
ENSMUSG00000083744	0	0	0	0	2	0
ENSMUSG00000096918	0	2	5	0	0	0
ENSMUSG00000081036	0	0	0	0	0	0
ENSMUSG00000105675	0	0	0	0	0	0
ENSMUSG00000105673	0	0	0	0	0	0
ENSMUSG00000105672	0	0	0	0	0	0
ENSMUSG00000114718	4	5	9	5	2	0
ENSMUSG00000105670	0	0	0	0	0	0
ENSMUSG00000114716	0	0	0	0	0	0
ENSMUSG00000114717	0	0	0	0	0	0
ENSMUSG00000114715	25	10	17	5	4	13
ENSMUSG00000114712	0	0	0	0	0	0
ENSMUSG00000114713	0	0	0	0	0	0
ENSMUSG00000114710	0	0	0	0	0	0
ENSMUSG00000105678	0	0	0	0	0	0
ENSMUSG00000110628	0	0	0	0	0	0
ENSMUSG00000110629	0	0	0	2	0	4
ENSMUSG00000110626	6	12	3	20	30	31
ENSMUSG00000110624	0	0	0	0	0	0
ENSMUSG00000110625	0	0	0	0	0	0
ENSMUSG00000110622	0	0	0	0	0	0
ENSMUSG00000110623	0	0	0	0	0	0
ENSMUSG00000025020	18	27	18	8	14	19
ENSMUSG00000070313	0	0	0	0	0	0
ENSMUSG00000034456	0	4	0	0	0	0
ENSMUSG00000070311	0	0	0	0	0	0
ENSMUSG00000047841	0	4	5	0	0	4
ENSMUSG00000108045	2	6	2	3	1	3
ENSMUSG00000108044	0	0	0	0	0	0
ENSMUSG00000085217	0	0	0	0	0	0
ENSMUSG00000108041	0	0	0	0	0	0
ENSMUSG00000108040	0	0	0	0	0	0
ENSMUSG00000034450	0	0	0	0	0	0
ENSMUSG00000108048	3	5	4	0	0	5
ENSMUSG00000083057	0	0	0	0	0	0
ENSMUSG00000083055	0	0	0	2	2	0
ENSMUSG00000083052	0	0	0	0	0	0
ENSMUSG00000034452	31	28	21	7	8	18
ENSMUSG00000111149	0	0	0	0	0	0
ENSMUSG00000083058	0	0	0	0	0	0
ENSMUSG00000083059	0	0	0	0	0	0
ENSMUSG00000065182	0	0	0	0	0	0
ENSMUSG00000079451	0	0	0	0	0	0
ENSMUSG00000082983	0	0	0	0	0	0
ENSMUSG00000082982	0	0	0	0	0	0
ENSMUSG00000079450	71	116	100	84	79	83
ENSMUSG00000082987	4	4	1	0	0	2
ENSMUSG00000082986	0	0	0	0	0	0
ENSMUSG00000082985	6	12	4	11	3	3
ENSMUSG00000105212	8	22	20	4	9	8
ENSMUSG00000095787	1	1	1	1	1	0
ENSMUSG00000113837	0	0	0	0	0	0
ENSMUSG00000026592	0	0	0	0	1	0
ENSMUSG00000096797	0	0	0	0	0	0

ENSMUSG00000090314	0	0	0	0	0	0
ENSMUSG00000085067	0	0	0	0	0	0
ENSMUSG00000085066	0	0	0	0	0	0
ENSMUSG00000085060	0	0	0	0	0	0
ENSMUSG00000081982	0	0	0	0	0	0
ENSMUSG00000081980	0	0	0	0	0	0
ENSMUSG00000081986	0	0	0	0	0	0
ENSMUSG00000081987	0	0	0	0	0	0
ENSMUSG00000081985	0	0	0	0	0	0
ENSMUSG00000098180	5	11	3	5	9	6
ENSMUSG00000098185	0	0	0	0	0	2
ENSMUSG00000098186	0	0	0	0	0	0
ENSMUSG00000098187	2	1	1	0	3	8
ENSMUSG00000098189	0	0	0	0	0	0
ENSMUSG00000041857	0	0	0	0	0	0
ENSMUSG00000078269	0	0	0	0	0	0
ENSMUSG00000078263	0	0	0	0	0	0
ENSMUSG00000093218	0	0	1	0	0	0
ENSMUSG00000097966	0	0	0	0	0	0
ENSMUSG00000081369	0	0	0	0	0	0
ENSMUSG00000083105	0	0	0	0	0	0
ENSMUSG00000081364	0	0	0	0	0	0
ENSMUSG00000097964	0	0	0	0	0	0
ENSMUSG00000081362	0	0	0	0	0	0
ENSMUSG00000081363	0	0	0	0	0	0
ENSMUSG00000081360	0	0	0	0	0	0
ENSMUSG00000053420	0	0	0	0	0	0
ENSMUSG00000027317	0	0	0	0	0	2
ENSMUSG00000092806	1	0	0	0	0	0
ENSMUSG00000027315	7	0	11	4	0	0
ENSMUSG00000099856	0	0	0	0	0	0
ENSMUSG00000099851	0	0	0	0	0	0
ENSMUSG00000092802	0	0	0	0	0	0
ENSMUSG00000099853	1	2	0	0	0	1
ENSMUSG00000099852	0	0	0	0	0	0
ENSMUSG00000027318	62	57	102	34	46	46
ENSMUSG00000072929	0	0	0	0	0	0
ENSMUSG00000051682	6	0	12	4	0	0
ENSMUSG00000072923	0	0	0	0	0	0
ENSMUSG00000084853	0	0	0	0	0	0
ENSMUSG00000090533	0	0	0	0	0	0
ENSMUSG00000090535	0	0	0	0	0	0
ENSMUSG00000090534	0	0	0	0	0	0
ENSMUSG00000084855	0	1	0	0	0	0
ENSMUSG00000084854	0	0	0	0	0	0
ENSMUSG00000090539	0	0	2	0	0	3
ENSMUSG00000090538	0	0	0	0	0	0
ENSMUSG00000077208	0	0	0	0	0	1
ENSMUSG00000077209	0	0	0	0	0	0
ENSMUSG00000098726	3	0	1	2	1	3
ENSMUSG00000098727	0	0	0	0	0	0
ENSMUSG00000098720	0	0	0	0	0	0
ENSMUSG00000098721	0	0	1	0	0	0
ENSMUSG00000098722	0	0	0	0	0	0
ENSMUSG00000077201	0	0	0	0	0	0
ENSMUSG00000077204	0	0	0	0	0	0
ENSMUSG00000077207	0	0	0	0	0	0
ENSMUSG00000088756	0	0	0	0	0	0

ENSMUSG00000046178	9	0	2	5	0	5
ENSMUSG00000095377	0	4	0	3	0	0
ENSMUSG00000043289	0	0	0	0	0	0
ENSMUSG00000076557	0	0	0	0	0	0
ENSMUSG00000031506	39	64	82	13	33	59
ENSMUSG00000059632	0	0	0	0	0	0
ENSMUSG00000109655	3	0	0	2	7	7
ENSMUSG00000030674	4	0	7	0	4	0
ENSMUSG00000088028	0	0	0	0	3	0
ENSMUSG00000030670	54	50	31	15	18	39
ENSMUSG00000082048	0	0	0	0	0	0
ENSMUSG00000082049	5	0	2	0	0	3
ENSMUSG00000082046	3	0	0	0	0	0
ENSMUSG00000087035	0	0	0	0	0	0
ENSMUSG00000088020	0	0	0	0	0	0
ENSMUSG00000082045	1	3	1	4	2	3
ENSMUSG00000088026	0	0	0	0	0	0
ENSMUSG00000082043	0	0	0	0	0	0
ENSMUSG00000087032	0	0	0	0	0	0
ENSMUSG00000087033	0	0	0	0	0	0
ENSMUSG00000101368	0	0	0	0	0	0
ENSMUSG00000101362	0	0	0	0	0	0
ENSMUSG00000101364	0	0	0	0	0	0
ENSMUSG00000101367	0	0	0	0	0	0
ENSMUSG00000101366	0	0	0	0	0	0
ENSMUSG00000047473	329	384	356	449	475	548
ENSMUSG00000085898	0	2	0	0	0	2
ENSMUSG00000085899	3	0	2	0	0	0
ENSMUSG00000085895	0	2	0	0	0	0
ENSMUSG00000085896	0	0	0	0	0	0
ENSMUSG00000085892	0	0	0	0	0	0
ENSMUSG00000071522	0	0	0	2	0	0
ENSMUSG00000071521	0	0	0	0	0	0
ENSMUSG00000100098	0	0	0	0	0	0
ENSMUSG00000100099	0	0	0	0	0	0
ENSMUSG00000103211	0	0	0	0	0	0
ENSMUSG00000103210	0	0	0	0	0	0
ENSMUSG00000103215	4	3	3	1	4	7
ENSMUSG00000103214	0	0	0	0	0	0
ENSMUSG00000100091	0	0	0	0	0	0
ENSMUSG00000100092	0	0	0	0	0	0
ENSMUSG00000103218	0	0	0	0	0	0
ENSMUSG00000100094	10	3	11	3	3	8
ENSMUSG00000100095	0	0	0	0	0	0
ENSMUSG00000100096	0	0	0	2	4	0
ENSMUSG00000100097	0	0	0	0	0	0
ENSMUSG00000088897	0	0	0	0	0	0
ENSMUSG00000083109	0	0	0	0	0	0
ENSMUSG00000014602	224	105	349	21	44	52
ENSMUSG00000065068	0	0	0	0	0	0
ENSMUSG00000107869	8	7	7	5	5	11
ENSMUSG00000014609	0	0	4	0	0	0
ENSMUSG00000089373	2	0	0	0	2	0
ENSMUSG00000089372	0	0	0	0	0	0
ENSMUSG00000089376	12	5	13	11	6	5
ENSMUSG00000109653	0	0	0	0	0	0
ENSMUSG00000022226	0	0	0	0	0	0
ENSMUSG00000022227	0	0	0	0	0	0

ENSMUSG00000022229	0	0	0	0	0	0
ENSMUSG00000110719	0	0	0	0	0	0
ENSMUSG00000075318	115	99	116	41	69	38
ENSMUSG00000084074	0	0	0	0	0	0
ENSMUSG00000084073	0	0	0	0	0	0
ENSMUSG00000095804	1	0	0	0	0	0
ENSMUSG00000065892	0	0	0	0	0	0
ENSMUSG00000100543	0	0	0	0	0	0
ENSMUSG00000100540	0	0	0	0	0	0
ENSMUSG00000100541	1	0	0	0	0	0
ENSMUSG00000100546	0	0	4	8	2	0
ENSMUSG00000100547	0	0	0	0	0	0
ENSMUSG00000065894	0	0	0	0	0	0
ENSMUSG00000041468	3	3	0	6	8	4
ENSMUSG00000065898	0	0	0	0	0	0
ENSMUSG00000100549	0	0	0	0	0	0
ENSMUSG00000022598	0	7	6	0	0	3
ENSMUSG00000064412	0	1	1	0	0	0
ENSMUSG00000060974	0	0	0	0	0	0
ENSMUSG00000035435	0	0	0	0	0	0
ENSMUSG00000035431	5	0	0	9	9	7
ENSMUSG00000107013	0	0	0	0	0	0
ENSMUSG00000107015	3	5	2	0	1	0
ENSMUSG00000107014	0	1	0	0	0	0
ENSMUSG00000107016	0	0	0	0	0	0
ENSMUSG00000079679	0	0	0	0	0	0
ENSMUSG00000109758	0	0	0	0	0	0
ENSMUSG00000085305	0	0	0	0	0	0
ENSMUSG00000085301	0	0	0	0	0	0
ENSMUSG00000085302	0	0	0	0	0	0
ENSMUSG00000051769	0	0	0	0	0	0
ENSMUSG00000033595	100	65	59	141	113	159
ENSMUSG00000095593	0	0	0	0	0	0
ENSMUSG00000071723	55	43	49	33	33	24
ENSMUSG00000088564	3	0	0	0	0	1
ENSMUSG00000088563	1	2	0	0	0	0
ENSMUSG00000063994	0	0	0	0	0	0
ENSMUSG00000063998	0	0	0	0	0	0
ENSMUSG00000069390	2	0	0	0	0	0
ENSMUSG00000024334	8	9	6	0	5	0
ENSMUSG00000034748	145	135	210	109	125	172
ENSMUSG00000113028	2	0	0	0	0	0
ENSMUSG00000061626	3	0	0	0	0	0
ENSMUSG00000113025	0	0	0	0	0	0
ENSMUSG00000113027	0	5	0	0	0	0
ENSMUSG00000113026	0	0	0	0	0	0
ENSMUSG00000113021	0	0	0	0	0	0
ENSMUSG00000113023	0	0	0	0	0	0
ENSMUSG00000038242	0	0	0	0	1	0
ENSMUSG00000063663	898	1132	944	815	813	1087
ENSMUSG00000059303	0	0	0	0	0	0
ENSMUSG00000063660	0	0	0	0	0	0
ENSMUSG00000051159	8	3	11	8	9	5
ENSMUSG00000051156	0	0	0	0	0	0
ENSMUSG00000016998	0	0	0	0	0	0
ENSMUSG00000051153	0	0	0	0	0	0
ENSMUSG00000029123	15	5	17	14	5	5
ENSMUSG00000106650	0	0	0	0	0	0

ENSMUSG00000106652	0	0	0	0	0	0
ENSMUSG00000106653	0	2	2	0	0	0
ENSMUSG00000106655	0	0	0	0	0	0
ENSMUSG00000088991	0	0	0	0	0	0
ENSMUSG00000106657	1	0	0	0	0	0
ENSMUSG00000106658	0	0	0	0	0	0
ENSMUSG00000097513	6	4	6	2	6	2
ENSMUSG00000104658	0	0	0	0	0	0
ENSMUSG00000097511	0	0	5	0	0	0
ENSMUSG00000097516	0	0	0	0	0	0
ENSMUSG00000097514	5	0	0	0	0	0
ENSMUSG00000104652	1	0	1	0	0	0
ENSMUSG00000104650	0	0	0	0	0	0
ENSMUSG00000104651	0	3	4	0	3	3
ENSMUSG00000104656	0	1	2	1	0	0
ENSMUSG00000104654	11	10	9	18	3	20
ENSMUSG00000069971	0	0	0	0	0	0
ENSMUSG00000110459	0	0	0	0	0	0
ENSMUSG00000109556	0	0	0	0	0	0
ENSMUSG00000109557	0	0	0	0	0	0
ENSMUSG00000109554	2	0	4	3	1	2
ENSMUSG00000106594	0	0	0	0	0	0
ENSMUSG00000109552	12	2	12	5	13	6
ENSMUSG00000109553	0	0	0	0	0	0
ENSMUSG00000109551	0	0	0	0	2	0
ENSMUSG00000114554	0	0	2	0	0	0
ENSMUSG00000114556	0	0	0	0	0	0
ENSMUSG00000114557	0	0	0	0	0	0
ENSMUSG00000114550	0	0	0	0	0	0
ENSMUSG00000114553	2	0	3	0	0	0
ENSMUSG00000094427	0	0	0	0	0	0
ENSMUSG00000094426	0	0	0	0	0	0
ENSMUSG00000011171	34	57	38	24	25	56
ENSMUSG00000093436	0	0	0	0	0	0
ENSMUSG00000094423	0	0	0	0	0	0
ENSMUSG00000112781	7	9	7	11	6	10
ENSMUSG00000071169	0	0	0	0	0	0
ENSMUSG00000050685	0	0	0	0	2	0
ENSMUSG00000112866	3	5	0	3	0	9
ENSMUSG00000112867	0	0	0	0	0	0
ENSMUSG00000112864	0	0	0	0	0	0
ENSMUSG00000112865	0	8	0	2	0	0
ENSMUSG00000112862	0	0	0	0	0	0
ENSMUSG00000093433	0	0	0	0	0	0
ENSMUSG00000108683	0	0	0	0	0	0
ENSMUSG00000108682	0	0	0	0	0	0
ENSMUSG00000091911	0	0	0	0	0	0
ENSMUSG00000112868	0	0	2	0	0	0
ENSMUSG00000112869	0	0	0	0	0	0
ENSMUSG00000109882	19	8	10	18	17	28
ENSMUSG00000109883	0	0	0	0	0	0
ENSMUSG00000109884	0	0	0	0	0	0
ENSMUSG00000109885	0	0	0	0	0	0
ENSMUSG00000109886	0	0	0	0	0	0
ENSMUSG00000051481	0	0	0	0	0	0
ENSMUSG00000105305	0	0	0	0	0	0
ENSMUSG00000114084	0	0	0	0	0	0
ENSMUSG00000114085	0	0	0	0	0	0

ENSMUSG00000114082	0	0	0	0	0	0
ENSMUSG00000114083	0	1	3	3	3	1
ENSMUSG00000020524	5	16	4	4	7	6
ENSMUSG00000066905	0	0	0	0	0	0
ENSMUSG00000110705	1	0	0	0	0	0
ENSMUSG00000110707	0	0	0	0	0	0
ENSMUSG00000110701	5	5	8	0	0	2
ENSMUSG00000110700	0	0	0	0	0	0
ENSMUSG00000096641	0	0	0	0	0	0
ENSMUSG00000096640	0	0	1	0	0	0
ENSMUSG00000071858	0	0	0	0	0	0
ENSMUSG00000096645	0	0	0	0	0	0
ENSMUSG00000096644	0	0	0	0	0	0
ENSMUSG00000096646	13	14	9	7	5	8
ENSMUSG00000096648	0	0	0	0	0	0
ENSMUSG00000060508	0	0	0	0	0	0
ENSMUSG00000060503	0	0	0	0	0	0
ENSMUSG00000111141	0	0	0	0	0	0
ENSMUSG00000112780	0	0	0	0	0	0
ENSMUSG00000089455	0	0	0	0	0	0
ENSMUSG00000056901	0	0	0	0	0	0
ENSMUSG00000110551	2	1	2	2	2	2
ENSMUSG00000026205	25	17	8	4	0	2
ENSMUSG00000052584	0	9	4	5	10	0
ENSMUSG00000044923	0	2	0	0	0	0
ENSMUSG00000059488	0	0	0	0	0	0
ENSMUSG00000059483	0	2	0	0	0	0
ENSMUSG00000059481	0	0	0	2	0	0
ENSMUSG00000045751	101	91	121	53	43	80
ENSMUSG00000022829	3	2	0	2	0	0
ENSMUSG00000095349	0	0	0	0	0	0
ENSMUSG00000095348	1	17	14	9	1	1
ENSMUSG00000095345	0	0	0	0	0	0
ENSMUSG00000111075	0	0	0	0	0	0
ENSMUSG00000095347	1	0	0	0	0	0
ENSMUSG00000111077	8	6	16	18	8	11
ENSMUSG00000095341	0	0	0	0	0	0
ENSMUSG00000095340	0	0	2	0	0	0
ENSMUSG00000111072	0	0	0	0	0	0
ENSMUSG00000111073	0	0	0	0	0	0
ENSMUSG00000081168	0	0	0	0	0	0
ENSMUSG00000081169	0	0	2	0	0	0
ENSMUSG00000081160	0	1	0	0	0	0
ENSMUSG00000064687	0	0	0	0	0	0
ENSMUSG00000053624	0	0	0	2	0	0
ENSMUSG00000047444	0	0	0	1	0	0
ENSMUSG00000053626	81	113	92	23	15	36
ENSMUSG00000110108	0	0	0	0	0	0
ENSMUSG00000090738	0	0	0	0	0	0
ENSMUSG00000090736	0	0	0	0	0	0
ENSMUSG00000090731	0	0	0	0	0	0
ENSMUSG00000107284	0	0	0	0	0	0
ENSMUSG00000094438	0	0	0	0	0	0
ENSMUSG00000042189	0	0	0	0	0	0
ENSMUSG00000107285	0	0	0	0	0	0
ENSMUSG00000094436	0	0	0	0	0	0
ENSMUSG00000107287	0	0	0	0	0	0
ENSMUSG00000107280	11	14	8	19	6	15

ENSMUSG00000056018	5	5	3	0	5	7
ENSMUSG00000112003	0	2	2	0	2	0
ENSMUSG00000070459	0	0	0	0	0	0
ENSMUSG00000077040	0	0	0	0	0	0
ENSMUSG00000042195	3	0	0	0	0	5
ENSMUSG00000107289	0	0	0	0	0	0
ENSMUSG00000112035	0	0	0	0	0	0
ENSMUSG00000112034	0	0	0	0	0	0
ENSMUSG00000112037	0	0	0	0	0	0
ENSMUSG00000112036	5	0	2	0	2	8
ENSMUSG00000112030	0	0	0	0	0	0
ENSMUSG00000112039	0	0	0	0	0	0
ENSMUSG00000112038	0	3	4	0	0	0
ENSMUSG00000086302	0	0	0	0	0	0
ENSMUSG00000107180	0	0	0	0	0	0
ENSMUSG00000055594	0	0	0	0	0	0
ENSMUSG00000093282	0	0	1	0	0	0
ENSMUSG00000093283	0	0	0	0	0	0
ENSMUSG00000093281	0	0	0	0	0	0
ENSMUSG00000105581	0	0	0	0	0	0
ENSMUSG00000049315	0	0	0	0	0	0
ENSMUSG00000049314	0	0	0	0	0	4
ENSMUSG00000094311	0	0	0	0	0	0
ENSMUSG00000113344	2	0	2	2	2	1
ENSMUSG00000113345	0	0	0	0	0	0
ENSMUSG00000113346	16	24	13	13	32	27
ENSMUSG00000092480	0	0	0	0	0	0
ENSMUSG00000092485	0	0	0	0	0	0
ENSMUSG00000113343	0	0	0	0	0	0
ENSMUSG00000092489	0	0	0	0	0	0
ENSMUSG00000113348	0	1	0	0	1	0
ENSMUSG00000016763	70	73	129	43	61	25
ENSMUSG00000082418	0	0	0	0	1	0
ENSMUSG00000101780	0	0	0	0	0	0
ENSMUSG00000106516	22	15	9	23	25	21
ENSMUSG00000106513	0	0	2	0	0	0
ENSMUSG00000106512	4	6	3	7	5	6
ENSMUSG00000106511	6	5	10	7	6	5
ENSMUSG00000106510	0	0	0	0	1	0
ENSMUSG00000106519	0	0	0	0	0	0
ENSMUSG00000106518	4	0	0	1	0	6
ENSMUSG00000040132	0	0	0	0	0	0
ENSMUSG00000040133	153	2	203	13	12	8
ENSMUSG00000040134	0	0	0	0	0	0
ENSMUSG00000042796	2	3	2	2	2	2
ENSMUSG00000080504	0	0	0	0	0	0
ENSMUSG00000080506	1	2	1	1	1	2
ENSMUSG00000105584	20	27	13	33	25	38
ENSMUSG00000080500	0	0	0	0	0	0
ENSMUSG00000082537	0	0	0	0	0	0
ENSMUSG00000082534	8	0	0	6	0	4
ENSMUSG00000082535	15	22	21	37	24	34
ENSMUSG00000082532	7	5	5	3	7	8
ENSMUSG00000082533	0	0	0	0	0	0
ENSMUSG00000082531	0	0	0	0	0	0
ENSMUSG00000082538	0	0	0	0	0	0
ENSMUSG00000082539	0	0	0	0	0	2
ENSMUSG00000035551	0	6	14	8	11	6

ENSMUSG00000001249	45	81	77	33	60	51
ENSMUSG00000084273	0	0	0	0	0	0
ENSMUSG00000084272	0	0	0	0	0	0
ENSMUSG00000084270	3	0	0	0	0	0
ENSMUSG00000085579	0	0	0	0	0	0
ENSMUSG00000084275	0	0	0	0	0	0
ENSMUSG00000084278	0	0	0	0	0	0
ENSMUSG00000025279	2	0	4	3	4	0
ENSMUSG00000080970	0	0	0	0	0	0
ENSMUSG00000080978	0	0	0	0	0	2
ENSMUSG00000080979	6	12	7	7	7	7
ENSMUSG00000099136	1	1	1	1	2	1
ENSMUSG00000087364	0	0	0	0	0	5
ENSMUSG00000099135	0	0	0	0	0	0
ENSMUSG00000099133	0	0	0	0	0	0
ENSMUSG00000078650	0	0	0	0	0	0
ENSMUSG00000078657	0	0	0	0	0	0
ENSMUSG00000099138	26	21	18	50	27	29
ENSMUSG00000099139	0	0	0	0	0	0
ENSMUSG00000105252	2	0	3	0	0	0
ENSMUSG00000100292	0	0	0	0	0	0
ENSMUSG00000077948	0	0	0	0	0	0
ENSMUSG00000062874	0	2	1	1	3	3
ENSMUSG00000093291	0	0	0	0	0	0
ENSMUSG00000065708	0	0	0	0	0	0
ENSMUSG00000075555	10	4	9	5	5	8
ENSMUSG00000075552	0	0	0	0	0	0
ENSMUSG00000075551	0	0	0	0	0	0
ENSMUSG00000065702	0	0	0	0	0	0
ENSMUSG00000044162	54	79	59	198	158	208
ENSMUSG00000065700	0	0	0	0	0	0
ENSMUSG00000100291	8	24	23	50	38	35
ENSMUSG00000065706	0	0	0	0	0	0
ENSMUSG00000065705	0	0	1	0	0	1
ENSMUSG00000070106	0	0	0	0	0	0
ENSMUSG00000070107	0	0	0	0	0	0
ENSMUSG00000070104	0	0	0	0	0	0
ENSMUSG00000107131	0	0	0	0	0	0
ENSMUSG00000070102	0	1	2	1	1	1
ENSMUSG00000070103	0	0	0	0	0	0
ENSMUSG00000070100	0	0	0	0	0	0
ENSMUSG00000093294	0	0	0	0	0	1
ENSMUSG00000074681	0	0	0	0	0	0
ENSMUSG00000074680	0	0	0	0	0	0
ENSMUSG00000062732	0	0	0	0	0	0
ENSMUSG00000086332	0	0	0	0	0	0
ENSMUSG00000094215	0	0	0	0	0	0
ENSMUSG00000068086	3	4	9	3	1	2
ENSMUSG00000068085	11	0	10	2	2	0
ENSMUSG00000068082	0	0	0	0	0	0
ENSMUSG00000028003	10	14	8	0	35	151
ENSMUSG00000060114	0	0	0	0	0	0
ENSMUSG00000105667	0	0	0	0	0	0
ENSMUSG00000104993	0	0	0	0	0	0
ENSMUSG00000104995	0	0	0	0	0	0
ENSMUSG00000101746	0	0	0	0	0	0
ENSMUSG00000060112	0	0	2	0	0	0
ENSMUSG00000013766	0	0	0	0	0	0

ENSMUSG00000094210	0	0	0	0	0	0
ENSMUSG00000039099	6	10	14	4	0	6
ENSMUSG00000105661	0	0	0	0	0	0
ENSMUSG00000082416	0	0	0	0	0	3
ENSMUSG00000103825	2	2	0	0	0	0
ENSMUSG00000103829	0	0	0	0	0	0
ENSMUSG00000093489	0	0	0	0	0	0
ENSMUSG00000035095	26	24	30	6	9	3
ENSMUSG00000105663	5	9	12	16	5	13
ENSMUSG00000112499	0	0	0	0	1	0
ENSMUSG00000094213	1	0	0	0	0	0
ENSMUSG00000083262	0	0	3	0	4	0
ENSMUSG00000066723	0	0	0	0	0	0
ENSMUSG00000089942	15	53	42	0	9	0
ENSMUSG00000089949	0	0	0	0	3	2
ENSMUSG00000082514	0	0	0	0	0	0
ENSMUSG00000015441	0	0	0	0	0	0
ENSMUSG00000100963	13	16	26	20	15	29
ENSMUSG00000074704	0	0	0	0	0	0
ENSMUSG00000076966	1	1	1	1	0	0
ENSMUSG00000076964	0	0	0	0	0	0
ENSMUSG00000062878	0	0	0	0	0	0
ENSMUSG00000108919	0	0	0	3	0	0
ENSMUSG00000108918	0	0	0	0	0	0
ENSMUSG00000108915	1	0	0	0	0	1
ENSMUSG00000108914	0	0	0	0	0	0
ENSMUSG00000108917	0	0	0	0	0	0
ENSMUSG00000034949	197	136	170	127	124	142
ENSMUSG00000086905	19	20	17	10	10	17
ENSMUSG00000108913	0	0	0	0	0	0
ENSMUSG00000086904	2	8	0	0	0	2
ENSMUSG00000108572	8	6	15	0	7	8
ENSMUSG00000067613	0	0	0	0	0	0
ENSMUSG00000067610	0	0	5	0	0	2
ENSMUSG00000067614	0	0	0	0	0	0
ENSMUSG00000067615	0	0	0	0	0	0
ENSMUSG00000089534	0	0	0	0	0	0
ENSMUSG00000105669	1	2	0	0	0	0
ENSMUSG00000063260	0	0	0	0	0	0
ENSMUSG00000101538	0	0	0	0	0	0
ENSMUSG00000101539	0	0	0	0	0	0
ENSMUSG00000101530	0	0	0	0	0	0
ENSMUSG00000101531	0	0	0	0	0	0
ENSMUSG00000101532	0	0	0	0	0	0
ENSMUSG00000101533	0	0	0	0	0	0
ENSMUSG00000101534	0	0	0	0	0	0
ENSMUSG00000101536	0	0	0	0	0	0
ENSMUSG00000101537	0	0	0	0	0	0
ENSMUSG00000085911	0	0	0	0	0	0
ENSMUSG00000085912	14	12	31	12	14	23
ENSMUSG00000085915	0	0	0	0	0	0
ENSMUSG00000085919	0	0	0	0	0	0
ENSMUSG00000085918	0	0	0	0	0	0
ENSMUSG00000062252	0	0	2	6	3	0
ENSMUSG00000087208	1	0	0	1	0	0
ENSMUSG00000107975	0	0	0	0	0	0
ENSMUSG00000087200	0	2	5	0	0	0
ENSMUSG00000040675	273	218	191	260	244	296

ENSMUSG00000037716	0	0	0	0	0	3
ENSMUSG00000103003	0	0	0	0	0	0
ENSMUSG00000074440	0	0	0	0	0	0
ENSMUSG00000103004	2	0	0	0	0	0
ENSMUSG00000103005	0	0	0	0	0	0
ENSMUSG00000103008	0	0	0	0	0	0
ENSMUSG00000100667	0	0	0	0	0	0
ENSMUSG00000100666	0	0	2	0	0	0
ENSMUSG00000100665	0	0	0	0	0	0
ENSMUSG00000100664	0	0	0	0	0	0
ENSMUSG00000100663	0	0	0	0	0	0
ENSMUSG00000100661	0	0	0	0	0	0
ENSMUSG00000100660	0	0	0	0	0	0
ENSMUSG00000100669	0	0	0	0	0	0
ENSMUSG00000100668	0	0	0	0	0	0
ENSMUSG00000059974	33	32	56	23	46	29
ENSMUSG00000059972	0	0	0	0	0	0
ENSMUSG00000102958	0	0	0	0	0	0
ENSMUSG00000088995	0	0	0	0	0	0
ENSMUSG00000061126	0	0	0	0	0	0
ENSMUSG00000022416	0	8	9	2	2	0
ENSMUSG00000089128	0	0	0	0	0	0
ENSMUSG00000089129	48	40	31	29	25	42
ENSMUSG00000089120	0	0	0	0	0	0
ENSMUSG00000089123	0	0	0	0	0	0
ENSMUSG00000089127	0	0	1	0	1	0
ENSMUSG00000018648	32	26	34	7	18	14
ENSMUSG00000093732	0	0	0	0	0	0
ENSMUSG00000094105	0	0	0	0	0	0
ENSMUSG00000102751	0	0	0	0	0	0
ENSMUSG00000112232	0	0	0	0	0	0
ENSMUSG00000112231	0	0	0	0	0	1
ENSMUSG00000102754	0	0	0	0	0	0
ENSMUSG00000102757	0	0	0	0	0	0
ENSMUSG00000102756	2	0	0	0	0	0
ENSMUSG00000102759	5	5	16	0	5	0
ENSMUSG00000093736	0	0	0	0	0	2
ENSMUSG00000111031	0	0	0	0	0	0
ENSMUSG00000112238	0	0	0	0	0	0
ENSMUSG00000109591	0	0	0	0	0	0
ENSMUSG00000094101	0	0	1	0	0	0
ENSMUSG00000062472	0	0	0	0	0	0
ENSMUSG00000062477	0	0	0	0	0	2
ENSMUSG00000111032	0	0	0	0	0	0
ENSMUSG00000088940	0	0	0	0	0	0
ENSMUSG00000094109	0	0	0	0	0	0
ENSMUSG00000112588	0	0	0	0	0	0
ENSMUSG00000112585	0	0	0	0	0	0
ENSMUSG00000112584	0	0	0	0	0	0
ENSMUSG00000112587	0	0	0	0	0	0
ENSMUSG00000112586	0	0	0	0	2	0
ENSMUSG00000112580	0	0	0	0	0	0
ENSMUSG00000112583	1	0	1	0	0	0
ENSMUSG00000112582	0	0	0	0	0	0
ENSMUSG00000105402	0	0	4	0	4	6
ENSMUSG00000105405	0	0	0	0	0	0
ENSMUSG00000105406	0	0	4	3	1	2
ENSMUSG00000105407	0	0	0	0	0	0

ENSMUSG00000105409	0	0	0	0	0	1
ENSMUSG00000029680	0	0	0	3	0	0
ENSMUSG00000113182	0	0	0	0	0	0
ENSMUSG00000113180	0	0	0	0	0	0
ENSMUSG00000113186	2	0	0	0	0	0
ENSMUSG00000113187	0	0	0	0	0	0
ENSMUSG00000113184	8	7	6	4	0	4
ENSMUSG00000113185	22	29	27	35	18	28
ENSMUSG00000028488	6	16	15	4	33	58
ENSMUSG00000039903	26	17	38	31	26	24
ENSMUSG00000039904	0	0	0	0	0	0
ENSMUSG00000024107	0	0	0	0	0	7
ENSMUSG00000024105	0	0	0	0	0	0
ENSMUSG00000103587	3	0	0	0	0	0
ENSMUSG00000103586	4	12	0	4	10	10
ENSMUSG00000034538	85	103	97	78	98	129
ENSMUSG00000103583	0	0	0	0	0	0
ENSMUSG00000103581	19	15	17	20	17	13
ENSMUSG00000103589	0	0	0	0	0	0
ENSMUSG00000069588	6	4	3	3	0	0
ENSMUSG00000069584	0	0	0	0	0	0
ENSMUSG00000069581	0	0	0	0	0	0
ENSMUSG00000069582	0	0	0	0	0	0
ENSMUSG00000069583	0	0	0	0	0	0
ENSMUSG00000051728	3	1	6	6	4	3
ENSMUSG00000114328	0	0	0	0	0	0
ENSMUSG00000084606	0	0	0	0	0	0
ENSMUSG00000114323	0	0	0	0	0	0
ENSMUSG00000114322	0	0	0	0	0	0
ENSMUSG00000057977	0	0	0	0	0	0
ENSMUSG00000114327	0	0	0	0	0	0
ENSMUSG00000114326	8	5	2	4	0	3
ENSMUSG00000114325	0	0	0	0	0	0
ENSMUSG00000114324	0	0	0	0	0	0
ENSMUSG00000011034	18	30	6	32	23	48
ENSMUSG00000097497	2	8	5	0	0	12
ENSMUSG00000097496	0	0	0	0	0	0
ENSMUSG00000097495	4	5	0	0	5	7
ENSMUSG00000108274	0	0	0	0	0	0
ENSMUSG00000108276	0	0	3	0	0	0
ENSMUSG00000104758	0	0	0	0	1	1
ENSMUSG00000108278	4	10	5	12	4	2
ENSMUSG00000108279	0	2	0	0	0	0
ENSMUSG00000059112	2	0	0	0	0	0
ENSMUSG00000059113	0	0	0	0	0	0
ENSMUSG00000059114	0	0	0	0	0	0
ENSMUSG00000104752	0	0	0	0	0	0
ENSMUSG00000104751	0	0	0	0	0	1
ENSMUSG00000091894	0	0	0	0	0	0
ENSMUSG00000091897	0	0	0	0	0	0
ENSMUSG00000067156	0	0	0	0	0	0
ENSMUSG00000107745	0	8	7	0	2	0
ENSMUSG00000025478	203	200	150	268	269	309
ENSMUSG00000107744	0	0	0	0	0	0
ENSMUSG00000025475	14	14	38	0	6	0
ENSMUSG00000097720	0	0	0	0	0	0
ENSMUSG00000107746	0	0	0	0	0	0
ENSMUSG00000097725	1	0	0	0	0	6

ENSMUSG00000097727	13	22	8	4	10	22
ENSMUSG00000097726	0	0	0	0	0	0
ENSMUSG00000089284	0	0	0	0	0	0
ENSMUSG00000108495	0	0	0	0	0	0
ENSMUSG00000108496	0	0	0	0	0	0
ENSMUSG00000108497	0	0	0	0	0	0
ENSMUSG00000108490	0	0	0	0	0	0
ENSMUSG00000108491	0	0	0	0	0	0
ENSMUSG00000108492	0	0	0	0	0	0
ENSMUSG00000108493	0	0	0	0	0	2
ENSMUSG00000091762	0	0	0	0	0	0
ENSMUSG00000108498	0	0	0	0	0	0
ENSMUSG00000091764	241	208	194	153	155	196
ENSMUSG00000083979	0	0	0	0	2	0
ENSMUSG00000083978	0	0	0	0	0	0
ENSMUSG00000083975	0	0	1	0	0	0
ENSMUSG00000083977	0	0	0	0	0	0
ENSMUSG00000083970	0	0	0	0	0	0
ENSMUSG00000083973	0	0	0	0	0	0
ENSMUSG00000083972	0	0	0	0	0	0
ENSMUSG00000044361	0	0	3	0	2	0
ENSMUSG00000044362	15	9	16	8	7	8
ENSMUSG00000044365	174	171	118	162	160	192
ENSMUSG00000044367	124	139	140	114	177	178
ENSMUSG00000058643	0	0	0	0	0	0
ENSMUSG00000111377	0	0	0	0	0	0
ENSMUSG00000046210	0	0	0	0	0	3
ENSMUSG00000045091	0	0	0	0	0	0
ENSMUSG00000089408	0	0	0	0	0	0
ENSMUSG00000110518	0	0	0	0	0	0
ENSMUSG00000110519	3	0	0	0	7	5
ENSMUSG00000110510	9	9	6	9	6	3
ENSMUSG00000110511	0	0	0	0	0	0
ENSMUSG00000110512	0	0	0	0	0	0
ENSMUSG00000110513	0	0	0	0	0	0
ENSMUSG00000109818	0	0	0	0	0	0
ENSMUSG00000080409	0	0	0	0	0	0
ENSMUSG00000080405	0	0	0	0	2	0
ENSMUSG00000080401	0	0	0	0	0	0
ENSMUSG00000080402	0	0	0	0	0	0
ENSMUSG00000074919	0	0	0	0	0	0
ENSMUSG00000020656	69	73	120	33	45	43
ENSMUSG00000074913	0	0	0	0	0	0
ENSMUSG00000111374	0	0	0	0	0	0
ENSMUSG00000113964	2	1	2	2	2	5
ENSMUSG00000113967	0	0	0	0	0	0
ENSMUSG00000113960	0	0	0	0	0	0
ENSMUSG00000113961	1	2	2	1	3	1
ENSMUSG00000113962	0	0	0	0	0	0
ENSMUSG00000113963	0	0	0	0	0	0
ENSMUSG00000113968	0	0	0	0	0	0
ENSMUSG00000113969	0	0	0	0	0	0
ENSMUSG00000045540	0	0	0	0	0	0
ENSMUSG00000073377	0	0	0	0	0	0
ENSMUSG00000090385	0	0	0	0	0	0
ENSMUSG00000098578	0	0	0	0	0	0
ENSMUSG00000111379	0	4	0	7	0	0
ENSMUSG00000093523	0	0	0	0	0	0

ENSMUSG00000053815	0	0	0	0	0	0
ENSMUSG00000094609	0	0	0	0	0	0
ENSMUSG00000094608	2	3	4	2	2	5
ENSMUSG00000094603	0	1	0	0	0	0
ENSMUSG00000094602	0	0	0	0	0	0
ENSMUSG00000094601	0	0	0	0	0	0
ENSMUSG00000094600	0	0	0	0	0	0
ENSMUSG00000094606	0	0	0	0	0	0
ENSMUSG00000094604	1	0	0	0	0	0
ENSMUSG00000086807	0	2	0	0	0	0
ENSMUSG00000086800	0	0	0	0	0	0
ENSMUSG00000086808	0	0	0	0	0	0
ENSMUSG00000081465	0	0	0	0	0	0
ENSMUSG00000081464	0	0	0	0	0	0
ENSMUSG00000081467	0	2	0	0	0	0
ENSMUSG00000081466	0	0	0	0	0	0
ENSMUSG00000081469	0	0	0	0	0	0
ENSMUSG00000109811	0	0	0	0	0	0
ENSMUSG00000017310	0	0	0	0	0	0
ENSMUSG00000017311	0	0	0	0	0	0
ENSMUSG00000017314	108	125	153	105	128	164
ENSMUSG00000017316	0	0	0	0	0	0
ENSMUSG00000090968	0	0	0	0	0	0
ENSMUSG00000090966	0	0	0	0	0	0
ENSMUSG00000090967	0	0	0	0	0	0
ENSMUSG00000090963	66	85	76	56	43	34
ENSMUSG00000090961	0	0	0	0	0	0
ENSMUSG00000092528	132	143	277	32	62	64
ENSMUSG00000092529	0	0	0	0	0	0
ENSMUSG00000007594	0	0	0	0	0	0
ENSMUSG00000092521	0	0	0	0	0	0
ENSMUSG00000092522	0	7	4	12	13	10
ENSMUSG00000092523	0	0	0	0	0	0
ENSMUSG00000092524	8	4	0	2	4	9
ENSMUSG00000099716	0	0	0	0	0	0
ENSMUSG00000099717	0	0	0	0	0	0
ENSMUSG00000099714	0	0	2	0	0	0
ENSMUSG00000099715	0	0	0	0	0	0
ENSMUSG00000099712	0	0	0	0	0	0
ENSMUSG00000099713	0	0	0	0	0	0
ENSMUSG00000099711	0	0	0	0	0	0
ENSMUSG00000077587	0	0	0	0	0	0
ENSMUSG00000077586	0	0	0	0	0	2
ENSMUSG00000077585	0	0	0	0	0	0
ENSMUSG00000077582	0	0	0	0	0	0
ENSMUSG00000055415	0	0	0	0	0	0
ENSMUSG00000055416	0	0	4	1	8	2
ENSMUSG00000089757	0	0	0	0	0	0
ENSMUSG00000109816	0	0	0	0	0	0
ENSMUSG00000064834	0	0	0	0	0	0
ENSMUSG00000111525	0	0	0	0	0	0
ENSMUSG00000005994	0	0	0	0	0	0
ENSMUSG00000086114	0	0	0	0	0	0
ENSMUSG00000086115	0	0	0	0	0	0
ENSMUSG00000086117	0	0	0	0	0	0
ENSMUSG00000086111	9	0	13	5	7	9
ENSMUSG00000043659	0	0	0	0	0	0
ENSMUSG00000086118	4	4	0	10	6	0

ENSMUSG00000114239	0	0	0	0	0	0
ENSMUSG00000076238	0	0	0	0	0	0
ENSMUSG00000046490	11	5	4	10	9	18
ENSMUSG00000087441	0	0	0	0	0	0
ENSMUSG00000076237	0	0	0	0	0	0
ENSMUSG00000076234	0	0	0	0	0	0
ENSMUSG00000076232	0	0	0	0	0	0
ENSMUSG00000076233	0	0	0	0	0	0
ENSMUSG00000109670	0	0	0	0	0	0
ENSMUSG00000109918	0	0	0	0	0	0
ENSMUSG00000070806	0	0	2	0	0	0
ENSMUSG00000070802	114	115	102	137	137	177
ENSMUSG00000105197	0	0	0	0	0	0
ENSMUSG00000083566	0	0	0	0	0	0
ENSMUSG00000083564	0	0	1	0	1	0
ENSMUSG00000083565	5	2	4	8	3	5
ENSMUSG00000083560	0	0	0	0	0	4
ENSMUSG00000083561	0	0	0	0	0	0
ENSMUSG00000049100	3	2	0	3	0	14
ENSMUSG00000079402	5	0	0	0	0	0
ENSMUSG00000079407	0	0	0	0	0	1
ENSMUSG00000043463	20	5	6	14	28	18
ENSMUSG00000109916	0	0	0	0	0	0
ENSMUSG00000087990	0	0	0	0	0	0
ENSMUSG00000087997	0	0	0	0	0	0
ENSMUSG00000031860	0	0	0	0	0	0
ENSMUSG00000031861	17	23	14	6	10	5
ENSMUSG00000087999	0	0	0	0	0	0
ENSMUSG00000114236	0	0	0	0	0	0
ENSMUSG00000104922	0	0	0	0	0	0
ENSMUSG00000082372	0	0	0	0	0	0
ENSMUSG00000109917	0	0	0	0	0	0
ENSMUSG00000083691	0	0	0	0	0	0
ENSMUSG00000071177	0	3	3	3	4	0
ENSMUSG00000083697	0	0	0	0	0	1
ENSMUSG00000083698	0	0	0	0	0	0
ENSMUSG00000083699	0	0	0	0	0	0
ENSMUSG00000109915	0	0	2	0	0	0
ENSMUSG00000064664	0	0	0	0	0	0
ENSMUSG00000111238	0	0	0	0	0	0
ENSMUSG00000107266	0	0	0	0	0	0
ENSMUSG00000109912	0	0	0	0	0	0
ENSMUSG00000111239	0	0	0	0	0	0
ENSMUSG00000000903	5	0	0	0	0	0
ENSMUSG00000066797	0	0	0	0	0	0
ENSMUSG00000068323	6	0	4	0	0	0
ENSMUSG00000061490	3	0	0	0	0	0
ENSMUSG00000068327	0	0	0	0	0	0
ENSMUSG00000095743	0	0	0	0	0	0
ENSMUSG00000085599	0	0	0	0	0	0
ENSMUSG00000085598	0	0	0	0	0	0
ENSMUSG00000109913	2	5	5	0	5	3
ENSMUSG00000112424	0	0	0	0	0	3
ENSMUSG00000109085	0	0	0	0	0	0
ENSMUSG00000102107	0	0	0	0	0	0
ENSMUSG00000074274	0	0	0	0	0	0
ENSMUSG00000078759	0	0	0	0	0	0
ENSMUSG00000074277	14	11	13	0	10	10

ENSMUSG00000078757	0	0	0	0	0	0
ENSMUSG00000078754	0	0	0	0	0	0
ENSMUSG00000078753	0	0	0	0	0	0
ENSMUSG00000078752	4	0	0	0	0	0
ENSMUSG00000046634	2	3	7	2	0	1
ENSMUSG00000102102	0	0	0	0	0	0
ENSMUSG00000020830	3	10	6	0	2	0
ENSMUSG00000081874	0	0	0	0	0	0
ENSMUSG00000081875	0	0	0	0	0	0
ENSMUSG00000081870	0	0	0	0	0	0
ENSMUSG00000109911	0	0	0	0	0	0
ENSMUSG00000096294	0	0	0	0	0	0
ENSMUSG00000032079	0	0	0	0	0	0
ENSMUSG00000074497	8	14	28	11	14	16
ENSMUSG00000074491	7	10	21	10	0	17
ENSMUSG00000073724	0	0	0	0	0	0
ENSMUSG00000073723	0	0	0	0	0	0
ENSMUSG00000073722	0	0	0	0	0	0
ENSMUSG00000073721	0	0	0	0	0	0
ENSMUSG00000102348	0	0	0	0	0	0
ENSMUSG00000102342	0	0	0	0	0	0
ENSMUSG00000102343	0	0	0	0	0	0
ENSMUSG00000102341	0	0	0	0	0	0
ENSMUSG00000102346	0	0	0	0	0	0
ENSMUSG00000102347	0	0	0	0	0	0
ENSMUSG00000102345	0	0	0	0	0	0
ENSMUSG00000029184	0	2	0	0	3	0
ENSMUSG00000099123	1	0	3	0	0	0
ENSMUSG00000098035	0	0	0	0	0	0
ENSMUSG00000038618	21	11	39	11	25	42
ENSMUSG00000098037	1	8	4	7	8	3
ENSMUSG00000098030	0	0	0	0	0	0
ENSMUSG00000098031	1	0	0	0	1	1
ENSMUSG00000098033	0	0	0	0	0	0
ENSMUSG00000098038	0	0	0	0	0	0
ENSMUSG00000110372	0	0	0	0	0	0
ENSMUSG00000069743	145	170	165	77	96	100
ENSMUSG00000067225	0	0	0	0	0	0
ENSMUSG00000035930	34	51	8	0	0	0
ENSMUSG00000035932	0	0	3	0	0	0
ENSMUSG00000110379	0	0	0	0	0	0
ENSMUSG00000096031	1	0	1	0	1	0
ENSMUSG00000096036	0	0	0	0	0	1
ENSMUSG00000082751	0	0	0	0	0	0
ENSMUSG00000083334	0	0	0	0	0	0
ENSMUSG00000100108	0	0	0	0	0	0
ENSMUSG00000063239	0	0	0	0	0	0
ENSMUSG00000034040	203	142	167	190	126	165
ENSMUSG00000063232	0	0	0	0	0	0
ENSMUSG00000063234	4	0	7	0	0	0
ENSMUSG00000083333	0	0	0	0	0	0
ENSMUSG00000039110	23	25	34	13	18	30
ENSMUSG00000100103	0	0	0	0	0	0
ENSMUSG00000084967	0	0	0	0	13	8
ENSMUSG00000084966	0	0	0	0	0	0
ENSMUSG00000084965	0	0	0	0	0	0
ENSMUSG00000084963	0	0	4	0	0	0
ENSMUSG00000066772	2	0	0	0	0	0

ENSMUSG00000084969	0	0	0	0	0	0
ENSMUSG00000100105	0	0	0	0	0	0
ENSMUSG00000070708	2	0	0	0	0	0
ENSMUSG00000070709	545	604	565	493	487	636
ENSMUSG00000091563	0	8	0	0	0	10
ENSMUSG00000071816	0	2	0	0	2	0
ENSMUSG00000071815	0	0	0	0	0	0
ENSMUSG00000030344	5	0	0	3	0	0
ENSMUSG00000030345	0	0	0	0	0	0
ENSMUSG00000030346	52	42	64	30	38	65
ENSMUSG00000030340	10	3	9	0	14	26
ENSMUSG00000044220	0	0	0	0	0	0
ENSMUSG00000064940	0	0	0	0	0	0
ENSMUSG00000075893	0	0	0	0	0	0
ENSMUSG00000104790	0	0	0	0	0	0
ENSMUSG00000097172	0	0	0	0	0	0
ENSMUSG00000056531	27	16	20	20	4	17
ENSMUSG00000101707	0	0	0	0	0	0
ENSMUSG00000101706	0	0	0	0	0	0
ENSMUSG00000104957	1	2	4	2	8	2
ENSMUSG00000104956	0	0	0	0	0	0
ENSMUSG00000101703	0	0	0	2	3	0
ENSMUSG00000056288	7	5	5	6	0	3
ENSMUSG00000104952	0	0	0	0	0	0
ENSMUSG00000104959	0	4	0	3	5	3
ENSMUSG00000101709	0	0	0	0	0	0
ENSMUSG00000101708	1	8	7	6	8	4
ENSMUSG00000100252	0	0	2	0	0	0
ENSMUSG00000100253	0	0	0	0	0	0
ENSMUSG00000100250	0	0	0	0	0	0
ENSMUSG00000100251	0	0	0	0	0	0
ENSMUSG00000100256	0	0	0	0	0	0
ENSMUSG00000088198	0	0	0	1	0	1
ENSMUSG00000100255	0	7	4	0	0	4
ENSMUSG00000088196	0	0	0	0	0	0
ENSMUSG00000088197	0	0	0	0	0	0
ENSMUSG00000088194	0	0	0	0	0	0
ENSMUSG00000088195	0	0	0	0	0	0
ENSMUSG00000088192	0	0	0	0	0	0
ENSMUSG00000088193	0	0	0	2	0	0
ENSMUSG00000088191	0	0	0	0	0	0
ENSMUSG00000058250	0	0	0	0	0	0
ENSMUSG00000058251	0	0	0	0	0	0
ENSMUSG00000058252	0	0	0	0	0	0
ENSMUSG00000064308	8	12	10	10	33	29
ENSMUSG00000095718	0	0	0	0	0	0
ENSMUSG00000026989	0	0	4	0	3	0
ENSMUSG00000026985	0	0	0	0	0	0
ENSMUSG00000026984	0	0	0	0	0	0
ENSMUSG00000095710	0	0	0	0	0	0
ENSMUSG00000095716	1	1	0	0	0	0
ENSMUSG00000026980	71	72	47	80	118	151
ENSMUSG00000026983	1	0	0	0	0	0
ENSMUSG00000088448	0	0	0	0	0	0
ENSMUSG00000084435	0	0	0	0	0	0
ENSMUSG00000088440	0	0	0	0	0	0
ENSMUSG00000088441	0	0	0	0	0	0
ENSMUSG00000088443	0	0	0	0	0	0

ENSMUSG00000088444	0	0	0	0	0	0
ENSMUSG00000088446	0	0	0	0	0	0
ENSMUSG00000083421	10	4	3	0	0	0
ENSMUSG00000022602	99	142	84	56	77	88
ENSMUSG00000022603	0	0	0	0	0	0
ENSMUSG00000107700	0	0	0	0	0	0
ENSMUSG00000107703	2	0	0	0	0	0
ENSMUSG00000107704	0	0	0	0	0	0
ENSMUSG00000107707	0	0	0	0	0	0
ENSMUSG00000107709	31	19	24	32	28	27
ENSMUSG00000107708	0	0	0	0	0	0
ENSMUSG00000111952	0	0	0	0	0	0
ENSMUSG00000103633	0	0	1	0	0	2
ENSMUSG00000111950	0	0	0	0	0	0
ENSMUSG00000111956	0	0	0	0	0	0
ENSMUSG00000111954	3	0	2	20	5	9
ENSMUSG00000103639	0	0	0	0	0	0
ENSMUSG00000111959	0	0	0	0	0	0
ENSMUSG00000103588	5	0	0	3	0	5
ENSMUSG00000096506	0	0	5	0	0	0
ENSMUSG00000096504	0	0	0	0	0	0
ENSMUSG00000096502	0	0	0	0	0	0
ENSMUSG00000088906	0	0	0	0	1	0
ENSMUSG00000023873	12	8	0	0	5	6
ENSMUSG00000096500	0	0	0	0	0	0
ENSMUSG00000036586	0	0	0	0	0	0
ENSMUSG00000036587	0	7	6	0	6	0
ENSMUSG00000089718	5	12	7	18	9	9
ENSMUSG00000035580	0	0	0	0	0	6
ENSMUSG00000039200	2	0	0	0	0	0
ENSMUSG00000035582	5	0	0	0	0	0
ENSMUSG00000024600	37	20	16	0	10	9
ENSMUSG00000039209	7	6	4	0	0	0
ENSMUSG00000021609	0	0	0	0	0	0
ENSMUSG00000110338	0	0	4	0	0	2
ENSMUSG00000110336	0	0	0	0	0	0
ENSMUSG00000011486	0	0	0	0	0	0
ENSMUSG00000036083	0	0	0	2	0	0
ENSMUSG00000092054	0	3	0	0	0	0
ENSMUSG00000092055	0	0	0	0	0	0
ENSMUSG00000092056	0	0	0	0	0	0
ENSMUSG00000004885	10	14	16	0	0	0
ENSMUSG00000112798	0	0	0	0	0	0
ENSMUSG00000112799	0	0	2	0	0	0
ENSMUSG00000112796	0	0	0	0	0	0
ENSMUSG00000112797	0	0	0	0	0	0
ENSMUSG00000109067	0	0	0	0	0	0
ENSMUSG00000109064	0	0	0	0	0	0
ENSMUSG00000114828	4	3	0	4	5	5
ENSMUSG00000109063	0	0	0	0	0	0
ENSMUSG00000109060	0	0	0	0	0	0
ENSMUSG00000114826	0	0	0	0	0	0
ENSMUSG00000114820	0	0	0	0	0	0
ENSMUSG00000114821	0	0	0	0	0	0
ENSMUSG00000114795	0	0	0	0	0	0
ENSMUSG00000114791	0	0	0	0	0	0
ENSMUSG00000114798	0	0	0	0	0	0
ENSMUSG00000106944	12	11	3	4	2	4

ENSMUSG00000106945	0	0	1	0	0	0
ENSMUSG00000113449	0	0	0	0	0	0
ENSMUSG00000080997	0	0	0	0	3	0
ENSMUSG00000113443	1	0	1	0	0	0
ENSMUSG00000113442	0	0	0	0	0	0
ENSMUSG00000113441	0	0	0	0	0	0
ENSMUSG00000113440	72	68	66	62	36	61
ENSMUSG00000106948	3	6	0	1	1	1
ENSMUSG00000113446	0	0	0	0	0	0
ENSMUSG00000113445	0	0	0	0	0	0
ENSMUSG00000113444	0	0	0	0	0	0
ENSMUSG00000085747	0	0	0	0	0	0
ENSMUSG00000109688	0	0	0	0	0	0
ENSMUSG00000080992	0	0	0	0	0	0
ENSMUSG00000109689	0	0	0	0	0	0
ENSMUSG00000021647	0	0	0	0	0	2
ENSMUSG00000109391	0	0	0	0	0	0
ENSMUSG00000109392	0	0	0	4	7	9
ENSMUSG00000051392	0	2	0	0	2	0
ENSMUSG00000109395	2	0	2	0	4	2
ENSMUSG00000109396	0	0	0	0	0	0
ENSMUSG00000109397	0	0	0	5	0	0
ENSMUSG00000109398	23	14	34	23	25	27
ENSMUSG00000114697	0	0	0	0	0	0
ENSMUSG00000096371	0	0	0	0	0	0
ENSMUSG00000096373	0	0	0	0	0	0
ENSMUSG00000096372	0	0	0	0	0	0
ENSMUSG00000096375	0	0	0	0	0	0
ENSMUSG00000096374	0	0	0	0	0	0
ENSMUSG00000096376	0	0	0	0	0	0
ENSMUSG00000096379	0	0	0	0	0	0
ENSMUSG00000109683	0	0	0	0	0	0
ENSMUSG00000082903	0	0	0	0	0	0
ENSMUSG00000082900	9	8	15	8	11	26
ENSMUSG00000082907	4	6	8	9	6	3
ENSMUSG00000082906	0	0	0	0	0	0
ENSMUSG00000082905	0	0	0	0	0	0
ENSMUSG00000066438	25	11	13	0	0	8
ENSMUSG00000109681	0	0	0	0	0	0
ENSMUSG00000096331	0	0	0	0	0	0
ENSMUSG00000109934	0	0	0	0	0	0
ENSMUSG00000109935	0	0	0	0	0	0
ENSMUSG00000026514	1	2	0	0	2	0
ENSMUSG00000109937	0	0	0	0	0	0
ENSMUSG00000093822	0	0	0	0	0	0
ENSMUSG00000109931	8	9	3	6	6	9
ENSMUSG00000093820	0	0	0	0	0	0
ENSMUSG00000109938	0	0	0	0	0	2
ENSMUSG00000109939	0	0	0	0	0	0
ENSMUSG00000093828	0	0	5	0	5	0
ENSMUSG00000050447	20	28	18	13	13	16
ENSMUSG00000104549	2	3	1	1	3	0
ENSMUSG00000114288	0	0	0	0	0	0
ENSMUSG00000104544	0	0	0	0	0	0
ENSMUSG00000104542	0	0	0	0	0	0
ENSMUSG00000104543	0	0	0	0	0	0
ENSMUSG00000114289	0	0	0	0	0	0
ENSMUSG00000044538	0	0	0	0	0	0

ENSMUSG00000044537	0	0	0	0	0	0
ENSMUSG00000111297	0	0	0	0	0	0
ENSMUSG00000108373	0	0	0	0	0	0
ENSMUSG00000113484	0	0	0	0	0	0
ENSMUSG00000093356	0	0	0	0	0	0
ENSMUSG00000113483	0	0	0	0	0	0
ENSMUSG00000108374	0	0	0	0	0	0
ENSMUSG00000108377	0	0	0	0	0	0
ENSMUSG00000110491	0	0	0	0	0	0
ENSMUSG00000101246	0	0	0	0	0	0
ENSMUSG00000110493	0	0	0	0	0	0
ENSMUSG00000110492	0	0	0	2	0	0
ENSMUSG00000110495	0	0	0	0	0	0
ENSMUSG00000110494	0	0	0	2	0	0
ENSMUSG00000110497	0	0	0	2	0	0
ENSMUSG00000106656	0	0	0	0	0	0
ENSMUSG00000110498	16	11	18	6	3	8
ENSMUSG00000101244	0	0	0	0	0	0
ENSMUSG00000101242	0	0	0	0	0	0
ENSMUSG00000101243	0	0	0	0	0	0
ENSMUSG00000046000	5	0	0	0	0	0
ENSMUSG00000046006	8	12	8	0	6	8
ENSMUSG00000101240	0	0	0	0	0	0
ENSMUSG00000046005	4	0	0	0	4	0
ENSMUSG00000046008	0	0	0	0	0	0
ENSMUSG00000095051	0	0	0	0	0	0
ENSMUSG00000085991	0	0	0	0	0	0
ENSMUSG00000095053	0	0	0	0	0	0
ENSMUSG00000095052	0	0	0	0	0	0
ENSMUSG00000045662	0	8	0	0	0	3
ENSMUSG00000095057	0	0	0	0	0	0
ENSMUSG00000095056	0	0	0	3	0	0
ENSMUSG00000095059	0	0	0	0	0	0
ENSMUSG00000090021	0	0	6	7	11	11
ENSMUSG00000085992	4	0	2	0	3	0
ENSMUSG00000006342	113	106	177	59	65	74
ENSMUSG00000006345	0	0	0	0	0	0
ENSMUSG00000092888	0	0	0	2	3	0
ENSMUSG00000092880	0	0	0	0	0	0
ENSMUSG00000114793	0	0	0	0	0	0
ENSMUSG00000114790	0	0	0	0	2	0
ENSMUSG00000093862	2	1	10	2	0	3
ENSMUSG00000080678	0	0	0	0	0	0
ENSMUSG00000080673	0	0	0	0	0	0
ENSMUSG00000080671	0	0	0	0	0	0
ENSMUSG00000109973	18	11	13	0	0	6
ENSMUSG00000093866	0	0	0	0	0	0
ENSMUSG00000093595	0	0	0	0	0	0
ENSMUSG00000093592	0	0	0	0	0	0
ENSMUSG00000078955	0	0	0	0	0	0
ENSMUSG00000078954	0	18	18	11	9	17
ENSMUSG00000086637	0	2	7	0	0	5
ENSMUSG00000078956	3	4	9	8	4	0
ENSMUSG00000086631	4	0	2	4	0	0
ENSMUSG00000086630	0	0	0	0	0	0
ENSMUSG00000086633	0	0	0	0	0	0
ENSMUSG00000109977	0	0	0	0	3	3
ENSMUSG00000078958	12	14	6	16	8	16

ENSMUSG00000031233	0	0	0	0	0	0
ENSMUSG00000077281	0	0	0	0	0	0
ENSMUSG00000047759	32	39	41	11	24	23
ENSMUSG00000077289	0	0	0	0	0	0
ENSMUSG00000113296	0	0	0	0	0	0
ENSMUSG00000113297	0	0	0	2	0	0
ENSMUSG00000093041	0	0	0	0	0	0
ENSMUSG00000049806	0	0	0	0	0	0
ENSMUSG00000076880	0	0	0	0	0	0
ENSMUSG00000076883	0	0	0	0	0	0
ENSMUSG00000076882	0	0	0	0	0	0
ENSMUSG00000113291	0	0	0	0	0	0
ENSMUSG00000052779	0	0	0	0	0	0
ENSMUSG00000073965	0	0	0	0	0	0
ENSMUSG00000077425	0	0	0	0	0	0
ENSMUSG00000073967	0	0	0	0	2	0
ENSMUSG00000073966	4	3	0	3	0	0
ENSMUSG00000073961	3	1	0	3	0	0
ENSMUSG00000040405	0	0	0	0	0	0
ENSMUSG00000073963	0	0	0	0	0	0
ENSMUSG00000073962	0	0	0	0	0	0
ENSMUSG00000073969	2	3	2	0	0	2
ENSMUSG00000055202	9	21	20	7	16	10
ENSMUSG00000113299	0	0	2	0	0	0
ENSMUSG00000048174	75	106	80	90	84	121
ENSMUSG00000099699	0	0	0	0	0	0
ENSMUSG00000099698	0	0	0	0	0	0
ENSMUSG00000048173	3	0	0	0	0	0
ENSMUSG00000099692	0	0	0	0	0	0
ENSMUSG00000099691	0	0	0	0	0	0
ENSMUSG00000099690	0	0	0	0	0	0
ENSMUSG00000099696	18	22	15	28	45	31
ENSMUSG00000099695	0	0	0	0	0	0
ENSMUSG00000099525	0	0	0	0	0	0
ENSMUSG00000099524	0	0	0	0	0	0
ENSMUSG00000099527	0	0	0	0	0	0
ENSMUSG00000099526	0	0	0	0	0	0
ENSMUSG00000092313	0	0	0	0	0	0
ENSMUSG00000092312	0	0	0	0	0	0
ENSMUSG00000099523	0	0	0	0	0	0
ENSMUSG00000099522	0	0	0	0	0	0
ENSMUSG00000099529	0	0	0	0	0	0
ENSMUSG00000099528	0	0	0	0	0	0
ENSMUSG00000092318	0	0	0	0	0	0
ENSMUSG00000045991	0	8	0	5	6	0
ENSMUSG00000108087	0	0	0	0	0	0
ENSMUSG00000065333	0	0	0	0	0	0
ENSMUSG00000024653	0	2	0	0	0	0
ENSMUSG00000104509	0	0	0	0	0	0
ENSMUSG00000024650	0	0	0	0	0	0
ENSMUSG00000100010	0	0	0	0	0	0
ENSMUSG00000100011	0	0	0	0	0	0
ENSMUSG00000100012	0	0	0	0	0	0
ENSMUSG00000100013	0	0	0	0	0	0
ENSMUSG00000100015	0	0	0	0	0	0
ENSMUSG00000100016	0	0	0	0	0	0
ENSMUSG00000089950	0	0	0	0	0	0
ENSMUSG00000041754	0	2	5	0	0	2

ENSMUSG00000041750	6	0	0	8	0	0
ENSMUSG00000070618	0	0	0	0	0	0
ENSMUSG00000070619	0	0	0	0	0	0
ENSMUSG00000070616	0	0	0	0	0	0
ENSMUSG00000070617	0	0	0	0	0	0
ENSMUSG00000102528	0	0	0	0	0	0
ENSMUSG00000102529	0	0	0	0	0	0
ENSMUSG00000102522	0	0	0	0	0	0
ENSMUSG00000102520	0	0	0	0	0	0
ENSMUSG00000102521	0	0	0	0	0	0
ENSMUSG00000102524	2	0	0	0	0	1
ENSMUSG00000064140	0	6	4	0	3	0
ENSMUSG00000083715	0	0	0	0	0	0
ENSMUSG00000083714	0	0	0	0	0	0
ENSMUSG00000083717	0	0	0	0	0	0
ENSMUSG00000084560	0	0	0	0	0	0
ENSMUSG00000083711	0	0	0	0	0	0
ENSMUSG00000084566	0	0	0	0	0	0
ENSMUSG00000084564	0	1	0	0	0	0
ENSMUSG00000084569	0	0	0	0	0	0
ENSMUSG00000084568	0	0	0	0	0	0
ENSMUSG00000083719	0	0	0	0	0	0
ENSMUSG00000075066	0	1	0	0	0	0
ENSMUSG00000107972	0	0	0	0	0	0
ENSMUSG00000075064	0	0	0	0	0	0
ENSMUSG00000075065	0	0	0	0	0	0
ENSMUSG00000075062	0	0	0	0	0	0
ENSMUSG00000075063	0	0	0	0	0	0
ENSMUSG00000075061	0	0	0	0	0	0
ENSMUSG00000068824	0	0	0	0	0	0
ENSMUSG00000065813	0	0	0	0	0	0
ENSMUSG00000065811	0	0	0	0	0	0
ENSMUSG00000075068	2	0	0	0	0	0
ENSMUSG00000075069	0	0	0	0	0	0
ENSMUSG00000068794	85	71	126	81	61	89
ENSMUSG00000084484	0	0	0	0	0	0
ENSMUSG00000107977	0	0	1	0	1	0
ENSMUSG00000107548	4	0	0	0	0	0
ENSMUSG00000107546	0	0	0	0	0	0
ENSMUSG00000107545	17	3	15	0	4	10
ENSMUSG00000107544	0	0	0	0	0	0
ENSMUSG00000107543	0	0	0	0	0	0
ENSMUSG00000107542	0	3	2	0	1	0
ENSMUSG00000107541	0	0	0	0	0	0
ENSMUSG00000107540	0	0	0	0	0	0
ENSMUSG00000075394	0	0	0	0	0	0
ENSMUSG00000075395	0	0	0	0	0	0
ENSMUSG00000101879	0	0	0	0	0	0
ENSMUSG00000061517	0	0	0	0	0	3
ENSMUSG00000042499	0	0	0	0	0	0
ENSMUSG00000101873	0	0	0	0	0	0
ENSMUSG00000101874	0	0	2	0	2	0
ENSMUSG00000101877	0	5	0	0	0	0
ENSMUSG00000103082	0	0	0	0	0	0
ENSMUSG00000085388	0	0	0	0	0	0
ENSMUSG00000085389	0	2	0	0	2	0
ENSMUSG00000041550	0	0	8	0	0	0
ENSMUSG00000085386	0	0	0	0	0	0

ENSMUSG00000085387	0	0	0	0	0	0
ENSMUSG00000085380	0	0	0	0	0	0
ENSMUSG00000085383	0	0	0	0	0	0
ENSMUSG00000107093	0	0	0	0	0	0
ENSMUSG00000092008	0	0	0	0	0	0
ENSMUSG00000084387	0	0	0	0	0	0
ENSMUSG00000084386	0	0	0	0	0	0
ENSMUSG00000084385	0	0	0	0	0	0
ENSMUSG00000036749	0	0	0	0	0	0
ENSMUSG00000036743	33	25	55	40	33	26
ENSMUSG00000036744	0	0	0	0	0	0
ENSMUSG00000065361	0	2	0	1	2	1
ENSMUSG00000065367	0	0	0	0	0	0
ENSMUSG00000041552	38	19	30	12	7	0
ENSMUSG00000100191	0	0	0	2	0	0
ENSMUSG00000100190	0	0	0	0	0	0
ENSMUSG00000114582	13	14	32	6	8	13
ENSMUSG00000051956	2	3	5	0	3	3
ENSMUSG00000078546	163	107	98	54	58	52
ENSMUSG00000103430	0	0	0	0	0	0
ENSMUSG00000030963	0	0	0	0	0	0
ENSMUSG00000024448	0	0	0	0	0	0
ENSMUSG00000089654	0	0	0	0	0	0
ENSMUSG00000089655	0	0	0	0	0	0
ENSMUSG00000089653	0	0	0	0	0	0
ENSMUSG00000097228	0	0	0	0	0	0
ENSMUSG00000073574	0	0	0	0	0	0
ENSMUSG00000073572	0	0	0	0	0	0
ENSMUSG00000073573	0	0	0	0	0	0
ENSMUSG00000039774	31	53	42	21	20	22
ENSMUSG00000039775	0	0	0	0	0	0
ENSMUSG00000100837	0	0	0	0	0	0
ENSMUSG00000100839	0	1	0	1	1	1
ENSMUSG00000097598	0	0	0	0	0	0
ENSMUSG00000048814	345	328	370	408	405	518
ENSMUSG00000048810	0	0	0	0	0	0
ENSMUSG00000038425	147	108	132	72	71	92
ENSMUSG00000097594	1	0	1	1	1	1
ENSMUSG00000112995	0	0	0	0	0	0
ENSMUSG00000102069	0	0	0	0	0	0
ENSMUSG00000102068	0	0	0	0	0	0
ENSMUSG00000102067	0	0	0	0	0	0
ENSMUSG00000102065	0	0	0	0	0	0
ENSMUSG00000102064	0	0	0	0	0	0
ENSMUSG00000102063	0	0	0	0	0	0
ENSMUSG00000102061	0	0	0	0	0	0
ENSMUSG00000102060	7	0	0	11	6	8
ENSMUSG00000105191	0	0	0	0	0	0
ENSMUSG00000096179	0	0	0	0	0	0
ENSMUSG00000096178	0	0	0	0	0	0
ENSMUSG00000096177	6	3	5	4	8	11
ENSMUSG00000096175	0	0	0	0	1	0
ENSMUSG00000096172	1	1	1	1	0	1
ENSMUSG00000058470	32	77	90	57	55	57
ENSMUSG00000109221	3	2	2	5	14	7
ENSMUSG00000050603	0	0	0	0	0	0
ENSMUSG00000050600	11	17	29	7	7	6
ENSMUSG00000050605	156	116	159	108	109	123

ENSMUSG00000084733	0	0	0	0	0	0
ENSMUSG00000108605	0	0	0	0	0	0
ENSMUSG00000108606	0	0	0	0	0	0
ENSMUSG00000108600	0	0	0	0	0	0
ENSMUSG00000108602	0	0	0	0	0	0
ENSMUSG00000105928	13	11	13	15	5	16
ENSMUSG00000021257	137	130	138	149	156	196
ENSMUSG00000105923	0	0	0	0	0	0
ENSMUSG00000021259	15	13	2	5	13	21
ENSMUSG00000083103	0	0	0	0	0	0
ENSMUSG00000105927	0	0	0	0	0	2
ENSMUSG00000057050	0	0	0	0	0	0
ENSMUSG00000057058	6	0	13	12	13	2
ENSMUSG00000025013	17	37	13	69	136	101
ENSMUSG00000101009	3	5	3	7	10	12
ENSMUSG00000101000	0	0	0	0	0	0
ENSMUSG00000101001	0	0	0	0	0	0
ENSMUSG00000101002	0	0	0	0	0	0
ENSMUSG00000101003	0	0	0	0	0	0
ENSMUSG00000101004	0	0	0	0	0	0
ENSMUSG00000101005	0	0	0	0	0	0
ENSMUSG00000101006	0	0	0	0	0	0
ENSMUSG00000101007	0	6	0	4	4	2
ENSMUSG00000104036	0	0	0	0	0	0
ENSMUSG00000104037	52	65	69	35	52	88
ENSMUSG00000104035	0	0	0	0	0	1
ENSMUSG00000104032	0	0	0	0	0	0
ENSMUSG00000104033	0	0	0	0	0	0
ENSMUSG00000104031	6	19	16	17	17	12
ENSMUSG00000104038	0	0	0	0	0	0
ENSMUSG00000059401	271	247	284	172	217	206
ENSMUSG00000018238	6	9	12	10	5	16
ENSMUSG00000078365	0	2	0	0	5	0
ENSMUSG00000061984	0	0	0	0	0	0
ENSMUSG00000062793	0	0	0	0	0	0
ENSMUSG00000024266	0	0	0	0	0	0
ENSMUSG00000064802	1	0	4	0	0	1
ENSMUSG00000064800	0	0	0	0	0	0
ENSMUSG00000064806	0	0	0	0	0	0
ENSMUSG00000064805	0	0	0	0	0	0
ENSMUSG00000021872	5	0	14	10	8	6
ENSMUSG00000021879	4	15	0	11	6	10
ENSMUSG00000100357	0	0	0	0	0	0
ENSMUSG00000100356	0	0	0	0	0	0
ENSMUSG00000100355	0	0	0	0	0	0
ENSMUSG00000100354	6	21	21	8	34	32
ENSMUSG00000100353	0	0	0	0	0	0
ENSMUSG00000100352	0	0	0	0	0	0
ENSMUSG00000100350	0	0	0	0	0	0
ENSMUSG00000055368	0	0	2	6	2	0
ENSMUSG00000110962	4	3	0	0	0	0
ENSMUSG00000110964	0	0	0	0	0	0
ENSMUSG00000110967	0	0	0	0	0	0
ENSMUSG00000100358	0	0	0	0	0	0
ENSMUSG00000089477	0	0	0	0	0	0
ENSMUSG00000095529	0	0	0	0	0	0
ENSMUSG00000095528	0	0	0	0	0	0
ENSMUSG00000095521	0	0	0	0	0	0

ENSMUSG00000095520	0	0	0	0	0	0
ENSMUSG00000095523	0	0	0	0	0	0
ENSMUSG00000095522	0	0	0	0	0	0
ENSMUSG00000095525	0	0	0	0	0	0
ENSMUSG00000095527	0	0	0	0	0	0
ENSMUSG00000105862	0	0	0	0	0	0
ENSMUSG00000042268	11	2	4	4	4	0
ENSMUSG00000109284	0	0	0	0	0	0
ENSMUSG00000042262	0	0	0	3	0	0
ENSMUSG00000094396	0	0	0	0	0	0
ENSMUSG00000042265	0	5	8	3	0	7
ENSMUSG00000086432	0	0	0	0	0	0
ENSMUSG00000086431	0	0	0	0	0	0
ENSMUSG00000086437	0	0	0	0	0	0
ENSMUSG00000086439	0	0	0	0	0	0
ENSMUSG00000086438	0	0	0	0	0	0
ENSMUSG00000114745	0	0	0	0	0	0
ENSMUSG00000064759	0	0	0	0	0	0
ENSMUSG00000008789	0	0	0	0	0	0
ENSMUSG00000040660	0	0	0	0	0	0
ENSMUSG00000114743	0	0	0	0	0	0
ENSMUSG00000094945	0	0	0	0	0	0
ENSMUSG00000027843	40	51	84	26	24	20
ENSMUSG00000114742	0	0	0	0	0	0
ENSMUSG00000093206	1	1	1	1	1	1
ENSMUSG00000093207	1	1	0	0	1	1
ENSMUSG00000086389	6	5	9	8	8	5
ENSMUSG00000093203	0	0	0	2	0	0
ENSMUSG00000006200	0	0	0	0	0	0
ENSMUSG00000086386	0	0	0	0	0	0
ENSMUSG00000086381	0	0	0	0	0	0
ENSMUSG00000086380	0	0	0	0	0	0
ENSMUSG00000086382	16	26	21	6	14	18
ENSMUSG00000036264	172	166	180	62	114	115
ENSMUSG00000114748	0	0	0	0	0	0
ENSMUSG00000097963	0	0	0	0	0	0
ENSMUSG00000097962	0	0	0	0	0	0
ENSMUSG00000097961	46	26	49	12	16	24
ENSMUSG00000097965	0	0	0	0	0	0
ENSMUSG00000028391	11	25	5	0	6	3
ENSMUSG00000112611	0	0	0	0	0	0
ENSMUSG00000112610	0	0	0	0	0	0
ENSMUSG00000112615	0	0	0	0	0	0
ENSMUSG00000112614	19	14	23	25	24	15
ENSMUSG00000112617	0	0	0	0	0	0
ENSMUSG00000112616	0	3	0	2	0	0
ENSMUSG00000112619	0	0	0	0	0	0
ENSMUSG00000112618	0	0	0	0	0	0
ENSMUSG00000094948	0	0	0	0	0	0
ENSMUSG00000055763	14	15	21	18	14	28
ENSMUSG00000112151	0	0	0	0	0	0
ENSMUSG00000054938	3	5	2	11	4	8
ENSMUSG00000054934	0	0	4	0	2	6
ENSMUSG00000054931	77	57	95	74	63	70
ENSMUSG00000054932	0	0	0	0	0	0
ENSMUSG00000047347	14	19	20	20	13	18
ENSMUSG00000051219	0	4	0	0	0	1
ENSMUSG00000000435	0	0	0	0	0	0

ENSMUSG00000071726	0	0	0	0	0	1
ENSMUSG00000016498	7	6	4	0	0	0
ENSMUSG00000112158	0	0	0	0	0	0
ENSMUSG00000101529	0	0	0	0	0	0
ENSMUSG00000106599	0	0	0	0	0	0
ENSMUSG00000106598	2	0	0	0	0	0
ENSMUSG00000086042	0	0	0	0	0	0
ENSMUSG00000068617	2	2	0	0	0	0
ENSMUSG00000060375	0	0	0	0	0	0
ENSMUSG00000108074	0	0	0	0	0	0
ENSMUSG00000109215	0	0	0	0	0	0
ENSMUSG00000109214	0	0	0	0	0	0
ENSMUSG00000114619	1	0	0	0	0	0
ENSMUSG00000114618	10	12	0	6	10	9
ENSMUSG00000109211	0	0	0	0	0	0
ENSMUSG00000109210	0	0	0	0	0	0
ENSMUSG00000109213	0	0	0	0	0	0
ENSMUSG00000109212	0	0	0	0	0	0
ENSMUSG00000114613	0	0	0	0	0	0
ENSMUSG00000114611	0	0	0	0	0	0
ENSMUSG00000114610	0	0	0	2	0	0
ENSMUSG00000109219	0	0	0	0	0	0
ENSMUSG00000114616	0	0	0	0	0	0
ENSMUSG00000114615	0	0	0	0	0	0
ENSMUSG00000114614	0	0	0	0	0	0
ENSMUSG00000110103	0	0	0	0	0	0
ENSMUSG00000110102	0	1	1	3	5	1
ENSMUSG00000110101	11	31	11	28	24	26
ENSMUSG00000110100	0	0	0	0	0	0
ENSMUSG00000110105	72	80	124	62	64	99
ENSMUSG00000005232	0	0	0	0	0	2
ENSMUSG00000005233	71	64	75	30	25	19
ENSMUSG00000005237	11	23	22	19	1	28
ENSMUSG00000108164	0	0	0	0	0	0
ENSMUSG00000108167	0	0	0	0	0	0
ENSMUSG00000108160	0	0	0	0	0	0
ENSMUSG00000108161	2	2	2	4	5	1
ENSMUSG00000108162	1	0	0	0	0	0
ENSMUSG00000108163	0	0	0	0	0	0
ENSMUSG00000083009	0	0	4	0	0	0
ENSMUSG00000105028	0	0	0	0	0	0
ENSMUSG00000105026	0	0	0	0	0	0
ENSMUSG00000105022	0	0	3	0	0	0
ENSMUSG00000105023	10	16	11	9	18	13
ENSMUSG00000105020	0	2	0	0	0	0
ENSMUSG00000108187	0	0	0	0	0	0
ENSMUSG00000070182	0	0	0	0	0	0
ENSMUSG00000064850	0	0	0	0	0	0
ENSMUSG00000083269	0	0	0	0	0	0
ENSMUSG00000083264	0	0	0	0	0	0
ENSMUSG00000083267	0	0	0	0	0	0
ENSMUSG00000026497	0	0	4	0	0	6
ENSMUSG00000083263	0	0	0	0	0	0
ENSMUSG00000085142	0	0	0	0	0	0
ENSMUSG00000085141	0	0	8	9	0	0
ENSMUSG00000085146	0	0	0	0	0	0
ENSMUSG00000085147	6	0	2	0	3	0
ENSMUSG00000026725	7	4	13	3	12	0

ENSMUSG00000044707	15	14	26	13	35	12
ENSMUSG00000044705	2	0	0	0	0	0
ENSMUSG00000092619	0	0	0	0	0	0
ENSMUSG00000044708	9	11	30	5	4	10
ENSMUSG00000074607	11	4	9	0	16	5
ENSMUSG00000083841	2	2	2	2	3	1
ENSMUSG00000083843	0	0	0	0	0	0
ENSMUSG00000045251	110	77	88	95	69	97
ENSMUSG00000083845	0	0	0	0	0	0
ENSMUSG00000083847	0	0	0	0	0	0
ENSMUSG00000083846	0	0	0	0	1	0
ENSMUSG00000083848	0	0	0	0	0	0
ENSMUSG00000095015	0	0	0	0	0	0
ENSMUSG00000072738	2	0	0	0	0	0
ENSMUSG00000095017	1	1	2	1	1	1
ENSMUSG00000053303	0	0	0	0	0	0
ENSMUSG00000073375	5	1	7	6	5	14
ENSMUSG00000019856	54	37	59	39	48	54
ENSMUSG00000073371	0	0	0	0	0	0
ENSMUSG00000019853	82	84	101	18	11	18
ENSMUSG00000019852	26	22	40	0	15	8
ENSMUSG00000027456	39	56	27	40	38	71
ENSMUSG00000027457	0	12	0	0	0	0
ENSMUSG00000092920	0	2	0	0	1	0
ENSMUSG00000092921	0	0	0	0	0	0
ENSMUSG00000092923	2	3	4	0	4	1
ENSMUSG00000078034	0	0	0	0	0	0
ENSMUSG00000078035	0	0	0	0	0	0
ENSMUSG00000078032	0	0	0	0	0	0
ENSMUSG00000078031	0	0	0	0	0	0
ENSMUSG00000078038	0	0	0	0	0	0
ENSMUSG00000090270	0	0	0	2	0	0
ENSMUSG00000098441	1	1	1	1	1	1
ENSMUSG00000098444	0	0	0	0	0	0
ENSMUSG00000098446	0	0	0	0	0	0
ENSMUSG00000098448	0	2	0	0	0	0
ENSMUSG00000098276	0	0	0	0	0	0
ENSMUSG00000088810	0	0	0	0	0	0
ENSMUSG00000082598	0	0	0	0	0	0
ENSMUSG00000082749	0	0	0	0	0	3
ENSMUSG00000096756	0	0	0	0	0	0
ENSMUSG00000088737	0	0	0	0	0	0
ENSMUSG00000088734	0	0	0	0	0	0
ENSMUSG00000053797	0	0	0	0	0	0
ENSMUSG00000088732	0	0	0	0	0	0
ENSMUSG00000088814	0	0	0	0	0	0
ENSMUSG00000096758	0	0	0	0	0	0
ENSMUSG00000088730	0	0	0	0	0	0
ENSMUSG00000088918	0	0	0	0	0	0
ENSMUSG00000087171	0	0	0	0	0	0
ENSMUSG00000087170	0	0	0	0	0	0
ENSMUSG00000087173	0	0	0	0	0	0
ENSMUSG00000087172	16	18	24	28	17	35
ENSMUSG00000094764	0	0	0	0	0	0
ENSMUSG00000078998	0	0	0	0	0	0
ENSMUSG00000111109	0	0	0	0	0	0
ENSMUSG00000085998	0	4	5	2	1	3
ENSMUSG00000101241	0	0	0	0	0	0

ENSMUSG00000085993	0	0	0	0	0	0
ENSMUSG00000101248	0	0	0	0	0	0
ENSMUSG00000037797	0	0	0	0	0	0
ENSMUSG00000094762	0	0	0	0	0	0
ENSMUSG00000037790	0	0	0	0	0	0
ENSMUSG00000037798	0	0	0	0	0	0
ENSMUSG00000043945	0	0	0	1	0	0
ENSMUSG00000067878	35	38	52	8	20	20
ENSMUSG00000078994	92	153	128	65	71	85
ENSMUSG00000014725	0	0	0	2	0	0
ENSMUSG00000107978	0	0	0	0	0	0
ENSMUSG00000107979	0	0	0	0	0	0
ENSMUSG00000084480	0	0	0	0	0	0
ENSMUSG00000107973	0	0	0	0	0	0
ENSMUSG00000107976	22	24	30	14	9	12
ENSMUSG00000084485	0	0	0	0	0	0
ENSMUSG00000084487	2	1	1	0	0	0
ENSMUSG00000103080	0	0	2	0	0	0
ENSMUSG00000103086	0	0	0	0	0	0
ENSMUSG00000103084	0	0	0	0	0	0
ENSMUSG00000100195	0	0	0	0	0	0
ENSMUSG00000100194	0	0	0	0	0	0
ENSMUSG00000100197	0	0	0	0	0	0
ENSMUSG00000100196	0	0	0	0	0	0
ENSMUSG00000082671	0	0	0	0	0	0
ENSMUSG00000082670	0	0	0	0	0	0
ENSMUSG00000100193	0	0	0	0	0	0
ENSMUSG00000082672	0	0	0	0	0	0
ENSMUSG00000087289	0	0	13	0	0	0
ENSMUSG00000087288	0	0	0	0	0	0
ENSMUSG00000082679	0	0	0	0	0	0
ENSMUSG00000100198	0	0	0	0	0	0
ENSMUSG00000032179	32	57	51	38	35	39
ENSMUSG00000021348	0	0	0	0	0	0
ENSMUSG00000084732	0	0	0	0	0	0
ENSMUSG00000083106	0	0	0	0	0	0
ENSMUSG00000084738	0	0	0	0	2	1
ENSMUSG00000041544	0	0	0	0	4	0
ENSMUSG00000113108	0	0	0	0	0	0
ENSMUSG00000064758	4	6	3	3	2	3
ENSMUSG00000064755	0	0	0	0	0	0
ENSMUSG00000107356	0	0	0	0	0	0
ENSMUSG00000107357	0	0	0	0	0	0
ENSMUSG00000021345	0	0	0	0	0	0
ENSMUSG00000107353	0	2	0	0	0	2
ENSMUSG00000062961	4	8	0	0	0	5
ENSMUSG00000075215	0	0	0	0	0	0
ENSMUSG00000075214	0	0	0	0	0	0
ENSMUSG00000075217	0	0	0	0	0	0
ENSMUSG00000075211	0	0	0	0	0	0
ENSMUSG00000075210	0	0	0	0	0	0
ENSMUSG00000075212	0	0	0	0	0	0
ENSMUSG00000075219	0	0	2	0	0	3
ENSMUSG00000075218	0	0	0	0	0	0
ENSMUSG00000113107	0	0	0	0	0	0
ENSMUSG00000087823	0	0	0	0	0	0
ENSMUSG00000087820	0	0	0	0	0	0
ENSMUSG00000087829	3	0	0	0	0	2

ENSMUSG00000085463	0	0	0	0	0	0
ENSMUSG00000085466	0	1	0	1	1	1
ENSMUSG00000085469	0	0	0	0	0	0
ENSMUSG00000085468	0	0	0	0	0	0
ENSMUSG00000074164	0	0	0	0	0	0
ENSMUSG00000033491	10	4	24	4	0	8
ENSMUSG00000079029	0	0	0	0	0	0
ENSMUSG00000033498	0	7	0	0	0	0
ENSMUSG00000079024	0	0	0	0	0	0
ENSMUSG00000079025	0	0	0	0	0	0
ENSMUSG00000036463	0	0	0	5	0	4
ENSMUSG00000088736	0	0	0	0	0	0
ENSMUSG00000088731	0	0	0	0	0	0
ENSMUSG00000069053	0	0	0	0	0	0
ENSMUSG00000088738	0	0	0	0	0	0
ENSMUSG00000088739	0	0	0	0	0	0
ENSMUSG00000094941	0	0	0	0	0	0
ENSMUSG00000094943	0	0	0	0	0	0
ENSMUSG00000094946	0	0	1	0	0	0
ENSMUSG00000021478	20	12	23	6	0	8
ENSMUSG00000011463	0	0	0	0	0	0
ENSMUSG00000113109	4	3	0	1	1	1
ENSMUSG00000113103	1	0	0	0	0	0
ENSMUSG00000061701	0	0	0	0	0	0
ENSMUSG00000063704	2	4	8	5	0	9
ENSMUSG00000051051	0	0	0	0	0	0
ENSMUSG00000051050	0	0	0	0	0	0
ENSMUSG00000051054	3	2	2	2	2	2
ENSMUSG00000029603	40	48	61	14	19	29
ENSMUSG00000029602	0	0	2	0	0	0
ENSMUSG00000029608	0	0	0	0	0	0
ENSMUSG00000106759	0	2	0	0	2	0
ENSMUSG00000106754	10	21	6	0	0	10
ENSMUSG00000103504	0	0	0	0	0	0
ENSMUSG00000103503	0	0	0	0	0	0
ENSMUSG00000103501	0	0	0	0	0	0
ENSMUSG00000106752	3	2	1	3	2	4
ENSMUSG00000097418	0	3	1	1	4	1
ENSMUSG00000097416	9	5	1	2	3	4
ENSMUSG00000038580	0	0	0	0	0	0
ENSMUSG00000088392	0	0	0	0	0	0
ENSMUSG00000114451	0	0	0	0	0	0
ENSMUSG00000024186	57	33	97	20	10	11
ENSMUSG00000114452	9	10	6	8	11	20
ENSMUSG00000114455	0	0	0	0	0	0
ENSMUSG00000114457	0	0	0	0	0	0
ENSMUSG00000100977	0	0	0	0	0	0
ENSMUSG00000100976	0	0	0	0	0	0
ENSMUSG00000100974	0	0	0	0	0	0
ENSMUSG00000100973	0	0	0	0	0	0
ENSMUSG00000100972	0	0	0	0	0	0
ENSMUSG00000100971	0	0	0	0	0	0
ENSMUSG00000100970	0	0	0	0	0	0
ENSMUSG00000100979	0	0	0	0	0	0
ENSMUSG00000100978	0	0	0	0	0	0
ENSMUSG00000020405	0	0	0	0	0	0
ENSMUSG00000020401	2	0	0	0	0	0
ENSMUSG00000054266	0	0	0	0	0	0

ENSMUSG00000091813	0	0	0	0	0	0
ENSMUSG00000091812	0	0	0	0	0	0
ENSMUSG00000091815	0	0	0	0	0	0
ENSMUSG00000091814	2	3	5	0	0	0
ENSMUSG00000091817	0	0	0	0	0	0
ENSMUSG00000091816	0	0	0	0	0	0
ENSMUSG00000109769	0	0	0	0	0	0
ENSMUSG00000109768	0	0	1	0	0	0
ENSMUSG00000109765	0	0	0	0	0	0
ENSMUSG00000105225	0	0	0	0	0	0
ENSMUSG00000109767	0	0	0	0	0	0
ENSMUSG00000109766	3	1	0	0	0	0
ENSMUSG00000109761	7	0	3	0	0	0
ENSMUSG00000105221	0	0	0	0	0	0
ENSMUSG00000105222	0	0	0	0	0	0
ENSMUSG00000109762	0	0	0	0	0	0
ENSMUSG00000090681	0	0	0	0	0	0
ENSMUSG00000108418	4	0	3	0	5	3
ENSMUSG00000108419	0	0	0	0	0	0
ENSMUSG00000050015	0	2	0	0	0	0
ENSMUSG00000108415	0	0	0	0	0	0
ENSMUSG00000108417	0	0	0	0	0	0
ENSMUSG00000108410	0	0	0	3	0	0
ENSMUSG00000108411	0	0	0	0	0	0
ENSMUSG00000108413	0	0	0	0	0	0
ENSMUSG00000106756	0	0	0	0	0	0
ENSMUSG00000106753	6	9	10	7	0	7
ENSMUSG00000103500	0	0	0	0	0	0
ENSMUSG00000060424	0	0	0	0	0	3
ENSMUSG00000097419	0	0	0	0	0	0
ENSMUSG00000057799	0	0	0	0	0	0
ENSMUSG00000078255	0	0	0	0	0	0
ENSMUSG00000026125	0	0	0	0	0	0
ENSMUSG00000026124	0	0	0	0	0	0
ENSMUSG00000096450	2	0	0	1	0	1
ENSMUSG00000096454	0	0	0	0	0	0
ENSMUSG00000096457	0	0	0	0	0	0
ENSMUSG00000028971	0	0	0	0	0	0
ENSMUSG00000057240	0	0	0	0	0	0
ENSMUSG00000057246	2	0	0	0	5	0
ENSMUSG00000077467	0	0	0	0	0	0
ENSMUSG00000109315	0	0	0	0	0	0
ENSMUSG00000045010	0	0	7	6	0	2
ENSMUSG00000095228	0	0	0	0	0	0
ENSMUSG00000045013	0	0	0	0	0	0
ENSMUSG00000045019	0	0	0	0	0	0
ENSMUSG00000111242	0	0	0	0	0	0
ENSMUSG00000111245	0	0	0	0	0	0
ENSMUSG00000053545	0	0	0	0	0	0
ENSMUSG00000027296	4	0	0	11	2	0
ENSMUSG00000027297	0	0	0	0	0	0
ENSMUSG00000027298	221	186	178	100	83	86
ENSMUSG00000091101	0	0	0	0	0	0
ENSMUSG00000017064	0	0	0	0	0	0
ENSMUSG00000090478	0	0	0	0	0	0
ENSMUSG00000095489	0	0	0	0	0	0
ENSMUSG00000095486	0	0	0	0	0	0
ENSMUSG00000095487	0	0	0	0	0	0

ENSMUSG00000095484	0	0	0	0	0	0
ENSMUSG00000095482	0	0	0	0	0	0
ENSMUSG00000090471	2	2	0	0	5	2
ENSMUSG00000090472	0	0	0	0	0	0
ENSMUSG00000081755	0	0	0	0	0	0
ENSMUSG00000094579	0	0	0	0	0	0
ENSMUSG00000094578	0	0	0	0	0	0
ENSMUSG00000022116	0	0	0	0	0	0
ENSMUSG00000094576	15	17	11	12	10	10
ENSMUSG00000081336	0	0	0	0	0	0
ENSMUSG00000056115	0	0	0	0	0	0
ENSMUSG00000114450	0	0	0	0	0	0
ENSMUSG00000017390	0	0	7	8	0	0
ENSMUSG00000111866	0	0	0	0	0	0
ENSMUSG00000111864	20	26	29	10	8	9
ENSMUSG00000097881	0	0	0	0	0	0
ENSMUSG00000097884	4	0	3	0	2	8
ENSMUSG00000111869	0	0	0	0	0	0
ENSMUSG00000053896	0	0	0	0	0	0
ENSMUSG00000042092	0	0	0	0	0	0
ENSMUSG00000042097	22	18	34	10	13	13
ENSMUSG00000042096	0	0	0	0	0	0
ENSMUSG00000094682	0	0	0	0	0	0
ENSMUSG00000094681	0	0	0	0	0	0
ENSMUSG00000094680	0	0	0	0	0	0
ENSMUSG00000094687	0	0	0	0	0	0
ENSMUSG00000094684	0	0	0	0	0	0
ENSMUSG00000094688	1	0	1	1	0	1
ENSMUSG00000046733	20	4	15	10	4	4
ENSMUSG00000091193	0	0	0	0	2	0
ENSMUSG00000077678	0	0	0	0	0	0
ENSMUSG00000077675	0	0	0	0	0	0
ENSMUSG00000077674	0	0	0	0	0	0
ENSMUSG00000077671	0	0	0	0	0	0
ENSMUSG00000077673	0	0	0	0	0	0
ENSMUSG00000077672	0	0	0	0	0	0
ENSMUSG00000086194	0	0	0	0	0	0
ENSMUSG00000049456	2	0	4	0	0	0
ENSMUSG00000086197	0	0	0	0	0	0
ENSMUSG00000112245	0	0	0	0	0	0
ENSMUSG00000071680	0	0	0	0	0	0
ENSMUSG00000071686	0	0	0	0	0	0
ENSMUSG00000045835	0	0	0	0	0	0
ENSMUSG00000096861	0	0	0	0	0	0
ENSMUSG00000096863	0	0	0	0	0	0
ENSMUSG00000098849	0	0	0	0	0	0
ENSMUSG00000096864	0	0	0	0	0	1
ENSMUSG00000096867	0	0	0	0	0	0
ENSMUSG00000096866	0	0	0	0	0	0
ENSMUSG00000098845	0	0	0	0	0	0
ENSMUSG00000096868	0	0	0	0	0	0
ENSMUSG00000098847	0	0	0	0	0	0
ENSMUSG00000098846	0	0	0	0	0	0
ENSMUSG00000106255	0	4	0	0	0	0
ENSMUSG00000106256	0	0	0	0	0	0
ENSMUSG00000106257	0	0	0	0	0	0
ENSMUSG00000106250	0	0	0	0	0	0
ENSMUSG00000106253	0	0	0	0	0	0

ENSMUSG00000106258	4	8	9	15	10	8
ENSMUSG00000106259	0	0	0	0	0	0
ENSMUSG00000005917	5	0	0	0	0	0
ENSMUSG00000004359	7	6	11	8	5	11
ENSMUSG00000110351	0	0	0	0	0	0
ENSMUSG00000109404	0	0	2	0	0	0
ENSMUSG00000109405	0	0	0	0	0	0
ENSMUSG00000109406	0	0	0	0	0	0
ENSMUSG00000109407	0	0	0	0	0	0
ENSMUSG00000109400	0	0	0	0	0	0
ENSMUSG00000109401	21	39	16	44	42	54
ENSMUSG00000109402	0	0	0	0	0	0
ENSMUSG00000109403	0	0	0	0	0	0
ENSMUSG00000109409	0	0	1	0	0	0
ENSMUSG00000082473	0	0	0	0	1	0
ENSMUSG00000082479	0	0	0	0	0	0
ENSMUSG00000110831	0	0	0	0	0	2
ENSMUSG00000110837	0	0	0	0	0	0
ENSMUSG00000050978	0	0	0	0	2	0
ENSMUSG00000110836	0	0	0	0	0	0
ENSMUSG00000110835	0	0	0	0	3	0
ENSMUSG00000084130	0	0	0	0	0	0
ENSMUSG00000084133	1	3	2	0	0	0
ENSMUSG00000084134	0	0	0	0	0	0
ENSMUSG00000084136	0	0	0	0	0	0
ENSMUSG00000084138	2	5	0	7	2	4
ENSMUSG00000110839	0	0	0	0	0	0
ENSMUSG00000110838	0	0	0	0	0	0
ENSMUSG00000025333	0	0	0	0	2	0
ENSMUSG00000025330	54	24	64	13	11	18
ENSMUSG00000080835	0	0	0	0	0	0
ENSMUSG00000080834	0	0	0	0	0	0
ENSMUSG00000105226	6	6	9	1	2	5
ENSMUSG00000080833	0	0	0	0	0	0
ENSMUSG00000099071	1	2	0	0	0	0
ENSMUSG00000099070	0	0	1	0	0	0
ENSMUSG00000099074	3	2	3	0	0	0
ENSMUSG00000043789	0	0	0	0	0	0
ENSMUSG00000099078	0	0	0	0	0	0
ENSMUSG00000109760	0	0	0	0	3	3
ENSMUSG00000023443	0	0	0	0	0	0
ENSMUSG00000109763	0	0	0	0	0	0
ENSMUSG00000105223	2	0	0	1	0	2
ENSMUSG00000104838	0	0	0	0	0	0
ENSMUSG00000068452	25	17	14	12	0	8
ENSMUSG00000000632	14	11	31	14	7	4
ENSMUSG00000085267	0	0	0	0	4	0
ENSMUSG00000104832	0	0	0	0	0	0
ENSMUSG00000085265	0	0	0	0	0	0
ENSMUSG00000085263	0	0	0	0	0	0
ENSMUSG00000085261	6	0	15	6	5	10
ENSMUSG00000042801	0	0	0	0	0	0
ENSMUSG00000104831	0	0	0	0	0	0
ENSMUSG00000000983	8	0	12	0	0	0
ENSMUSG00000053472	12	10	6	12	17	10
ENSMUSG00000056184	0	0	0	0	0	0
ENSMUSG00000079222	0	0	0	0	0	0
ENSMUSG00000006586	419	279	314	325	293	214

ENSMUSG00000110094	0	0	0	0	0	0
ENSMUSG00000099635	0	0	0	0	0	0
ENSMUSG00000074418	2	0	0	0	0	0
ENSMUSG00000074419	15	0	15	8	0	6
ENSMUSG00000074417	17	26	18	18	27	0
ENSMUSG00000076332	0	0	0	0	0	0
ENSMUSG00000033961	206	193	131	154	204	247
ENSMUSG00000054497	0	0	0	0	0	0
ENSMUSG00000033967	13	15	24	11	11	0
ENSMUSG00000077991	0	0	0	0	0	0
ENSMUSG00000077996	0	0	0	0	0	0
ENSMUSG00000077998	0	0	0	0	0	1
ENSMUSG00000099921	0	0	0	0	0	0
ENSMUSG00000099920	0	0	0	0	0	0
ENSMUSG00000099922	0	0	0	0	0	0
ENSMUSG00000099925	0	0	0	0	0	0
ENSMUSG00000099924	6	3	0	4	0	4
ENSMUSG00000099926	0	0	0	0	0	0
ENSMUSG00000063548	0	0	0	0	0	0
ENSMUSG00000007989	204	288	266	242	233	268
ENSMUSG00000107778	0	0	0	0	0	0
ENSMUSG00000095945	0	0	0	0	0	0
ENSMUSG00000078925	0	0	0	2	2	0
ENSMUSG00000086646	0	0	0	0	0	0
ENSMUSG00000086643	0	0	0	0	0	0
ENSMUSG00000072814	0	0	0	0	0	0
ENSMUSG00000072813	0	0	0	0	0	0
ENSMUSG00000048445	83	86	77	75	53	97
ENSMUSG00000048440	188	177	196	183	160	198
ENSMUSG00000077334	0	0	0	0	0	0
ENSMUSG00000077336	0	0	0	0	0	0
ENSMUSG00000077332	0	0	0	0	0	0
ENSMUSG00000077333	0	0	0	0	0	0
ENSMUSG00000077338	0	1	0	0	0	1
ENSMUSG00000038879	23	30	22	16	11	17
ENSMUSG00000031610	0	0	6	0	0	0
ENSMUSG00000110083	0	0	0	0	0	0
ENSMUSG00000110086	2	0	4	0	0	2
ENSMUSG00000102926	0	0	0	0	0	0
ENSMUSG00000102920	0	0	0	0	0	0
ENSMUSG00000085006	0	4	6	0	0	4
ENSMUSG00000008843	0	0	0	0	0	0
ENSMUSG00000102928	0	0	0	0	0	0
ENSMUSG00000071890	4	0	0	0	0	0
ENSMUSG00000043592	0	0	0	0	0	0
ENSMUSG00000087329	0	0	0	0	0	0
ENSMUSG00000031289	0	0	0	0	0	0
ENSMUSG00000087323	0	0	0	0	0	0
ENSMUSG00000087325	0	0	0	0	0	0
ENSMUSG00000087326	14	14	17	0	10	9
ENSMUSG00000088116	0	0	0	0	0	0
ENSMUSG00000088112	0	0	0	0	0	0
ENSMUSG00000088111	0	0	0	0	0	0
ENSMUSG00000037613	97	93	139	22	89	101
ENSMUSG00000037610	0	0	2	0	0	0
ENSMUSG00000033105	231	164	222	214	201	209
ENSMUSG00000103329	0	0	0	0	0	0
ENSMUSG00000103328	0	0	0	0	0	0

ENSMUSG00000103327	0	0	0	0	0	0
ENSMUSG00000103325	6	5	16	4	8	16
ENSMUSG00000103323	0	0	0	0	0	0
ENSMUSG00000103322	1	1	0	0	0	1
ENSMUSG00000103321	0	0	2	0	0	0
ENSMUSG00000100785	0	0	0	0	0	0
ENSMUSG00000100780	0	0	0	0	0	0
ENSMUSG00000100783	9	12	9	7	18	33
ENSMUSG00000100788	0	0	0	0	0	0
ENSMUSG00000100789	0	0	2	0	5	0
ENSMUSG00000101786	0	0	0	0	0	0
ENSMUSG00000101783	0	0	0	0	0	0
ENSMUSG00000059832	0	0	0	0	0	0
ENSMUSG00000107839	1	0	0	0	0	0
ENSMUSG00000107837	0	0	0	0	0	0
ENSMUSG00000107836	0	0	0	0	0	0
ENSMUSG00000107834	0	0	0	0	0	0
ENSMUSG00000107832	0	0	0	0	0	0
ENSMUSG00000107831	0	0	0	0	0	0
ENSMUSG00000107830	0	0	0	0	0	0
ENSMUSG00000029368	0	0	3	0	0	0
ENSMUSG00000084675	0	0	0	0	0	0
ENSMUSG00000084671	0	0	0	0	0	0
ENSMUSG00000009115	0	0	0	0	0	0
ENSMUSG00000100439	0	0	0	0	0	0
ENSMUSG00000100437	0	5	3	0	0	1
ENSMUSG00000100434	0	3	0	0	0	0
ENSMUSG00000100432	0	0	0	0	0	0
ENSMUSG00000100433	0	0	0	0	0	0
ENSMUSG00000100431	0	0	0	0	0	0
ENSMUSG00000064386	0	0	0	0	0	0
ENSMUSG00000107788	18	23	18	28	25	23
ENSMUSG00000113334	11	0	9	0	0	0
ENSMUSG00000107781	0	0	0	0	0	0
ENSMUSG00000107783	0	0	0	0	0	0
ENSMUSG00000107785	0	0	0	0	0	0
ENSMUSG00000107784	0	0	0	0	0	0
ENSMUSG00000107787	0	0	0	0	0	0
ENSMUSG00000089794	0	11	6	8	4	15
ENSMUSG00000089792	0	0	0	0	0	0
ENSMUSG00000089791	10	2	5	9	7	5
ENSMUSG00000095976	0	0	0	1	0	0
ENSMUSG00000095973	0	0	0	0	0	0
ENSMUSG00000075402	0	0	0	0	0	0
ENSMUSG00000095971	0	0	0	0	0	0
ENSMUSG00000065477	1	0	0	0	0	0
ENSMUSG00000022686	29	51	55	19	10	35
ENSMUSG00000022683	8	0	5	7	7	3
ENSMUSG00000102498	6	0	6	5	6	5
ENSMUSG00000102499	0	0	0	0	0	0
ENSMUSG00000102497	0	0	0	0	0	0
ENSMUSG00000082326	0	0	0	0	0	0
ENSMUSG00000102492	0	1	0	0	1	0
ENSMUSG00000102491	0	0	0	0	0	0
ENSMUSG00000024680	0	0	0	5	0	0
ENSMUSG00000024681	0	0	0	0	0	0
ENSMUSG00000024682	0	2	0	0	0	0
ENSMUSG00000043948	0	6	8	1	5	7

ENSMUSG00000052922	0	0	3	0	0	0
ENSMUSG00000098308	4	0	4	3	0	2
ENSMUSG00000035509	25	42	33	26	18	21
ENSMUSG00000102149	0	0	0	0	0	0
ENSMUSG00000112469	0	0	0	0	0	0
ENSMUSG00000102140	5	10	0	11	14	2
ENSMUSG00000112460	0	0	0	0	0	0
ENSMUSG00000112466	0	0	0	0	0	0
ENSMUSG00000112467	0	0	0	0	0	0
ENSMUSG00000112464	0	0	0	0	0	2
ENSMUSG00000112465	0	2	0	0	0	0
ENSMUSG00000096453	0	0	0	0	0	1
ENSMUSG00000048349	8	4	3	7	0	0
ENSMUSG00000096455	0	0	0	0	0	0
ENSMUSG00000097927	0	0	0	0	0	0
ENSMUSG00000039860	0	0	0	0	0	0
ENSMUSG00000024027	0	0	0	0	0	0
ENSMUSG00000029015	0	0	0	0	0	0
ENSMUSG00000024028	0	0	0	0	0	0
ENSMUSG00000024029	0	0	0	0	0	0
ENSMUSG00000106492	2	0	4	2	0	0
ENSMUSG00000106493	0	0	0	0	0	0
ENSMUSG00000106491	2	5	0	5	5	2
ENSMUSG00000106496	0	0	0	0	0	0
ENSMUSG00000106494	0	0	0	0	0	0
ENSMUSG00000106495	0	0	0	0	1	0
ENSMUSG00000113730	0	0	0	0	3	0
ENSMUSG00000106498	69	101	48	64	78	93
ENSMUSG00000113734	0	0	0	0	0	0
ENSMUSG00000113737	8	17	6	23	23	30
ENSMUSG00000113736	0	0	0	0	0	0
ENSMUSG00000099838	0	0	0	0	0	0
ENSMUSG00000059014	0	0	0	0	0	0
ENSMUSG00000106540	0	0	0	0	0	0
ENSMUSG00000111463	0	0	0	0	0	0
ENSMUSG00000111460	0	1	0	0	0	0
ENSMUSG00000111467	0	0	0	0	0	0
ENSMUSG00000111465	0	0	0	2	4	0
ENSMUSG00000111464	0	0	0	0	0	0
ENSMUSG00000066129	2	0	3	0	2	0
ENSMUSG00000066122	0	0	0	0	0	0
ENSMUSG00000034362	8	8	2	16	16	10
ENSMUSG00000108339	0	0	0	0	0	0
ENSMUSG00000108335	0	0	0	0	0	0
ENSMUSG00000108334	0	0	0	0	0	0
ENSMUSG00000108337	1	0	1	0	0	0
ENSMUSG00000108336	0	0	0	0	0	0
ENSMUSG00000108331	2	0	0	0	0	0
ENSMUSG00000108330	0	5	0	0	2	0
ENSMUSG00000086896	0	0	0	0	0	0
ENSMUSG00000048391	0	1	0	0	0	0
ENSMUSG00000067299	0	0	0	0	0	0
ENSMUSG00000067292	0	0	0	0	0	0
ENSMUSG00000098123	10	19	15	9	14	26
ENSMUSG00000097608	0	2	0	0	0	0
ENSMUSG00000050197	0	0	0	0	0	0
ENSMUSG00000108590	0	0	0	0	0	0
ENSMUSG00000108593	0	0	0	0	0	0

ENSMUSG00000108592	0	0	0	5	3	2
ENSMUSG00000108597	0	0	0	2	0	0
ENSMUSG00000050190	0	0	0	0	0	0
ENSMUSG00000108598	0	0	0	0	0	0
ENSMUSG00000098125	0	0	0	0	0	0
ENSMUSG00000091605	0	0	0	0	0	0
ENSMUSG00000091606	0	0	0	0	0	0
ENSMUSG00000050198	0	0	0	0	0	0
ENSMUSG00000098128	2	0	0	0	0	0
ENSMUSG00000114173	0	0	0	0	0	0
ENSMUSG00000114170	0	0	0	0	0	0
ENSMUSG00000097836	40	27	0	0	0	0
ENSMUSG00000114176	4	0	0	0	0	0
ENSMUSG00000114174	0	0	0	0	0	0
ENSMUSG00000114175	0	0	0	0	0	0
ENSMUSG00000097831	0	0	0	3	0	0
ENSMUSG00000114178	0	0	0	0	0	0
ENSMUSG00000101018	0	0	0	0	0	0
ENSMUSG00000044244	60	52	60	38	54	58
ENSMUSG00000025648	276	264	258	171	174	197
ENSMUSG00000050224	0	0	0	0	0	0
ENSMUSG00000027643	0	0	0	0	0	0
ENSMUSG00000098304	0	0	0	0	0	0
ENSMUSG00000010136	0	2	4	0	4	0
ENSMUSG00000095229	0	0	0	0	0	0
ENSMUSG00000110413	0	0	0	0	0	0
ENSMUSG00000110412	0	0	0	0	0	0
ENSMUSG00000110415	0	0	0	0	0	0
ENSMUSG00000110417	0	0	0	0	0	0
ENSMUSG00000110416	0	0	0	0	0	0
ENSMUSG00000080368	0	0	0	0	0	0
ENSMUSG00000080360	0	0	0	0	0	0
ENSMUSG00000080363	0	0	0	0	0	0
ENSMUSG00000042976	12	20	35	13	10	7
ENSMUSG00000080367	0	1	0	0	0	0
ENSMUSG00000095222	0	0	1	1	0	1
ENSMUSG00000046088	0	0	0	1	0	2
ENSMUSG00000106851	0	0	0	0	0	0
ENSMUSG00000060212	28	43	26	15	17	37
ENSMUSG00000046082	0	0	0	0	0	0
ENSMUSG00000009214	0	0	0	0	0	0
ENSMUSG00000095221	0	0	0	0	0	0
ENSMUSG00000106854	0	0	0	0	0	0
ENSMUSG00000095226	0	0	0	0	0	0
ENSMUSG00000076399	0	0	0	0	0	1
ENSMUSG00000029917	0	0	0	0	0	0
ENSMUSG00000057163	0	0	0	0	0	0
ENSMUSG00000095224	16	9	32	13	0	18
ENSMUSG00000057161	0	0	0	0	0	0
ENSMUSG00000057160	0	0	0	0	3	2
ENSMUSG00000111243	0	5	4	2	16	5
ENSMUSG00000076398	0	0	0	0	0	0
ENSMUSG00000111240	0	0	0	0	0	0
ENSMUSG00000073914	0	0	0	0	0	0
ENSMUSG00000101311	0	0	0	0	0	0
ENSMUSG00000111244	1	0	0	2	0	0
ENSMUSG00000073916	2	2	0	0	2	0
ENSMUSG00000073917	0	0	0	0	0	0

ENSMUSG00000111248	0	0	0	0	0	0
ENSMUSG00000101317	0	0	0	0	0	0
ENSMUSG00000101973	0	0	0	0	0	0
ENSMUSG00000094729	0	0	0	0	0	0
ENSMUSG00000101319	0	0	0	0	0	0
ENSMUSG00000094721	0	0	0	2	0	0
ENSMUSG00000094722	0	0	0	0	0	0
ENSMUSG00000094723	0	0	0	0	0	0
ENSMUSG00000094725	0	0	0	1	0	0
ENSMUSG00000094726	0	0	0	0	0	0
ENSMUSG00000094727	0	0	0	0	0	0
ENSMUSG00000029883	0	0	0	0	0	0
ENSMUSG00000081099	0	1	1	0	0	1
ENSMUSG00000081092	0	0	0	0	0	0
ENSMUSG00000081095	0	2	0	0	0	0
ENSMUSG00000081096	14	15	11	10	11	21
ENSMUSG00000081097	0	0	0	0	0	0
ENSMUSG00000081542	0	0	0	0	0	0
ENSMUSG00000081543	0	0	0	0	0	0
ENSMUSG00000048686	0	0	0	0	0	0
ENSMUSG00000081547	0	0	0	0	0	0
ENSMUSG00000081548	0	0	0	0	0	0
ENSMUSG00000093516	0	0	0	0	0	0
ENSMUSG00000093513	0	0	0	0	0	0
ENSMUSG00000093511	12	0	0	0	13	0
ENSMUSG00000093518	0	0	0	0	0	0
ENSMUSG00000101830	0	0	0	0	0	0
ENSMUSG00000017211	0	0	0	0	0	0
ENSMUSG00000057609	0	0	0	0	0	0
ENSMUSG00000052482	2	5	0	2	4	2
ENSMUSG00000099612	0	0	0	0	0	0
ENSMUSG00000099611	0	0	0	0	0	0
ENSMUSG00000099617	0	0	0	0	0	0
ENSMUSG00000099615	0	0	0	0	0	0
ENSMUSG00000099614	0	0	0	0	0	0
ENSMUSG00000099619	0	0	0	0	0	0
ENSMUSG00000112101	0	0	0	0	0	0
ENSMUSG00000112100	0	0	0	0	0	0
ENSMUSG00000112103	0	0	0	2	2	2
ENSMUSG00000112102	0	0	0	0	0	0
ENSMUSG00000112105	7	16	10	7	14	9
ENSMUSG00000112104	0	0	0	0	0	0
ENSMUSG00000112107	0	0	0	0	0	0
ENSMUSG00000112109	0	0	0	0	0	0
ENSMUSG00000112108	0	0	0	0	0	0
ENSMUSG00000103228	0	3	0	0	0	1
ENSMUSG00000004961	14	18	38	6	4	16
ENSMUSG00000030108	0	0	0	2	5	0
ENSMUSG00000030109	0	0	0	0	4	0
ENSMUSG00000085618	0	0	0	0	0	0
ENSMUSG00000103223	0	0	6	0	0	0
ENSMUSG00000092396	0	0	0	0	0	0
ENSMUSG00000092393	0	0	0	0	0	0
ENSMUSG00000092392	0	5	5	0	0	0
ENSMUSG00000092390	0	0	0	0	0	0
ENSMUSG00000051900	2	0	0	0	0	0
ENSMUSG00000086010	0	0	0	0	0	0
ENSMUSG00000086013	22	37	24	15	18	35

ENSMUSG00000086017	0	0	0	0	0	0
ENSMUSG00000086019	0	0	0	0	0	0
ENSMUSG00000103225	2	0	4	3	1	4
ENSMUSG00000072476	17	15	32	11	9	17
ENSMUSG00000043753	0	0	4	0	0	0
ENSMUSG00000046500	0	0	0	3	0	3
ENSMUSG00000020490	0	0	0	0	0	4
ENSMUSG00000076371	0	0	0	0	0	0
ENSMUSG00000076370	0	0	0	0	0	0
ENSMUSG00000020493	36	16	20	5	22	20
ENSMUSG00000085982	14	25	7	22	10	16
ENSMUSG00000082011	0	0	0	0	0	0
ENSMUSG00000085999	0	0	0	0	0	0
ENSMUSG00000025469	0	0	0	0	0	0
ENSMUSG00000037451	0	0	0	0	0	0
ENSMUSG00000111533	6	5	10	0	0	3
ENSMUSG00000049229	0	0	0	0	0	0
ENSMUSG00000087658	5	1	4	4	11	11
ENSMUSG00000087659	0	0	0	0	0	0
ENSMUSG00000083791	0	2	0	0	0	0
ENSMUSG00000090476	0	0	0	0	0	0
ENSMUSG00000083792	0	0	0	2	0	0
ENSMUSG00000090470	0	0	0	0	0	0
ENSMUSG00000103271	2	1	0	0	0	0
ENSMUSG00000070695	0	0	0	0	0	0
ENSMUSG00000070690	0	0	0	0	0	0
ENSMUSG00000071341	0	0	0	0	0	0
ENSMUSG00000084309	0	0	0	0	0	0
ENSMUSG00000084308	0	0	0	0	0	1
ENSMUSG00000084307	2	0	0	0	0	0
ENSMUSG00000084305	0	0	0	0	0	0
ENSMUSG00000084303	1	0	0	0	0	0
ENSMUSG00000084301	7	2	2	4	0	0
ENSMUSG00000000861	84	142	74	34	112	115
ENSMUSG00000085658	33	28	20	26	24	20
ENSMUSG00000085659	0	0	0	0	0	0
ENSMUSG00000091718	0	0	0	0	0	0
ENSMUSG00000085652	0	0	0	0	0	0
ENSMUSG00000085651	0	0	0	0	0	0
ENSMUSG00000085656	0	0	0	5	0	2
ENSMUSG00000089800	4	1	2	0	0	7
ENSMUSG00000081932	2	1	1	1	0	2
ENSMUSG00000081931	0	0	0	0	0	0
ENSMUSG00000081934	0	0	0	0	0	0
ENSMUSG00000083764	0	0	0	0	0	0
ENSMUSG00000065673	1	2	0	0	4	2
ENSMUSG00000089302	0	0	0	0	0	0
ENSMUSG00000035486	12	11	7	38	47	65
ENSMUSG00000094571	0	0	1	2	0	0
ENSMUSG00000094570	0	0	0	0	0	0
ENSMUSG00000094573	0	0	0	0	0	1
ENSMUSG00000089305	0	0	0	0	0	0
ENSMUSG00000074591	0	0	0	0	0	0
ENSMUSG00000074593	0	0	0	0	0	0
ENSMUSG00000094572	0	0	1	0	0	0
ENSMUSG00000074595	0	0	0	0	0	4
ENSMUSG00000074594	0	0	0	0	0	0
ENSMUSG00000074596	0	0	0	0	0	0

ENSMUSG00000022112	0	0	0	0	0	0
ENSMUSG00000022113	0	0	0	0	0	0
ENSMUSG00000094577	0	0	0	0	0	0
ENSMUSG00000042540	0	0	0	0	0	0
ENSMUSG00000103276	0	0	0	0	0	0
ENSMUSG00000062028	0	3	0	0	4	0
ENSMUSG00000068173	0	0	0	0	0	0
ENSMUSG00000103166	2	0	2	0	0	0
ENSMUSG00000072995	0	0	0	0	0	0
ENSMUSG00000098171	0	0	0	0	0	0
ENSMUSG00000098170	0	0	0	0	0	0
ENSMUSG00000086089	0	0	0	0	0	0
ENSMUSG00000078509	0	0	0	0	0	0
ENSMUSG00000098175	0	0	1	0	0	0
ENSMUSG00000098174	0	0	0	0	0	0
ENSMUSG00000098177	0	0	0	0	0	0
ENSMUSG00000098176	62	60	60	72	30	71
ENSMUSG00000098179	1	0	0	0	0	0
ENSMUSG00000077815	0	0	0	0	0	0
ENSMUSG00000084247	0	0	0	0	0	0
ENSMUSG00000103958	0	0	0	2	0	0
ENSMUSG00000048899	22	28	14	46	42	25
ENSMUSG00000103954	0	0	0	0	0	0
ENSMUSG00000048895	91	98	94	118	107	98
ENSMUSG00000103950	0	0	0	0	0	0
ENSMUSG00000103951	0	0	0	0	0	0
ENSMUSG00000109641	0	0	0	0	0	0
ENSMUSG00000063089	0	0	0	0	0	0
ENSMUSG00000084885	27	5	14	2	3	7
ENSMUSG00000084887	0	0	0	0	0	0
ENSMUSG00000111865	4	3	0	3	2	1
ENSMUSG00000109126	0	0	0	0	0	0
ENSMUSG00000111863	0	0	0	0	0	0
ENSMUSG00000048728	35	50	34	38	37	26
ENSMUSG00000101080	0	0	0	0	0	0
ENSMUSG00000101081	0	0	0	0	0	0
ENSMUSG00000101083	0	0	0	0	0	0
ENSMUSG00000101084	0	0	0	0	0	0
ENSMUSG00000101085	0	0	0	0	0	0
ENSMUSG00000101087	0	0	0	0	0	0
ENSMUSG00000101088	0	0	0	0	0	0
ENSMUSG00000101089	0	0	0	0	0	0
ENSMUSG00000077971	0	0	0	0	0	0
ENSMUSG00000091442	0	0	0	0	0	0
ENSMUSG00000091441	0	0	0	0	0	0
ENSMUSG00000097883	0	0	0	0	0	0
ENSMUSG00000019906	60	80	77	76	105	102
ENSMUSG00000028217	0	0	0	0	0	0
ENSMUSG00000020839	15	16	20	0	4	10
ENSMUSG00000108868	0	0	0	0	0	0
ENSMUSG00000064889	0	0	0	0	0	0
ENSMUSG00000064882	0	0	0	0	0	0
ENSMUSG00000034837	0	0	6	0	3	0
ENSMUSG00000108863	0	0	0	0	0	0
ENSMUSG00000108865	0	0	0	0	0	0
ENSMUSG00000108864	0	0	0	0	0	0
ENSMUSG00000108867	0	0	0	0	0	0
ENSMUSG00000108866	0	0	0	0	0	0

ENSMUSG00000006462	90	93	62	99	152	156
ENSMUSG00000006463	145	133	145	137	152	184
ENSMUSG00000101624	0	0	0	0	0	0
ENSMUSG00000101626	0	0	0	0	0	0
ENSMUSG00000101627	0	0	0	0	0	0
ENSMUSG00000101622	0	0	0	0	0	0
ENSMUSG00000101623	0	0	0	0	0	0
ENSMUSG00000101629	0	0	0	0	0	0
ENSMUSG00000088099	0	0	0	0	0	0
ENSMUSG00000088098	0	0	0	0	0	0
ENSMUSG00000061906	0	0	0	0	0	0
ENSMUSG00000088093	0	0	0	0	0	0
ENSMUSG00000088091	0	0	0	0	0	0
ENSMUSG00000046269	61	43	29	32	39	18
ENSMUSG00000088097	0	0	1	0	0	0
ENSMUSG00000088096	0	0	0	2	0	0
ENSMUSG00000088095	0	0	0	0	0	0
ENSMUSG00000088094	0	0	0	0	0	0
ENSMUSG00000087517	0	0	0	0	0	0
ENSMUSG00000088324	2	0	0	0	0	0
ENSMUSG00000088327	0	0	0	0	0	0
ENSMUSG00000088326	0	0	0	0	1	0
ENSMUSG00000087512	0	0	0	0	0	0
ENSMUSG00000087510	0	0	0	0	0	0
ENSMUSG00000058354	0	0	0	0	0	0
ENSMUSG00000088329	0	0	0	0	0	0
ENSMUSG00000064263	0	0	0	0	0	0
ENSMUSG00000070547	5	0	0	0	3	13
ENSMUSG00000026866	2	0	0	0	0	0
ENSMUSG00000065845	5	4	0	0	0	2
ENSMUSG00000108299	0	0	0	0	0	0
ENSMUSG00000108832	3	3	6	9	0	7
ENSMUSG00000101382	0	0	0	0	0	0
ENSMUSG00000108831	0	0	0	0	0	3
ENSMUSG00000107669	0	0	0	0	0	0
ENSMUSG00000107666	0	0	0	0	0	0
ENSMUSG00000034883	0	4	0	0	0	4
ENSMUSG00000107664	2	0	0	0	0	0
ENSMUSG00000107662	0	0	0	0	0	0
ENSMUSG00000107663	0	0	0	0	0	0
ENSMUSG00000107660	0	0	0	0	0	0
ENSMUSG00000107661	0	0	0	0	0	0
ENSMUSG00000101385	0	0	0	0	0	0
ENSMUSG00000103179	2	0	0	2	2	0
ENSMUSG00000103176	0	0	0	0	0	0
ENSMUSG00000103177	0	0	0	0	0	0
ENSMUSG00000103174	0	0	0	0	0	0
ENSMUSG00000103175	4	2	4	2	0	2
ENSMUSG00000103172	0	0	0	0	0	0
ENSMUSG00000036151	22	37	33	19	31	49
ENSMUSG00000100020	0	0	0	0	0	0
ENSMUSG00000110508	0	0	0	0	0	0
ENSMUSG00000061013	4	2	0	10	7	3
ENSMUSG00000103131	13	8	17	11	8	8
ENSMUSG00000089055	0	0	0	0	0	0
ENSMUSG00000018486	22	17	34	14	12	4
ENSMUSG00000089052	0	0	0	0	0	0
ENSMUSG00000089053	0	0	0	0	0	0

ENSMUSG00000110500	0	0	0	0	0	0
ENSMUSG00000020014	11	20	14	11	21	28
ENSMUSG00000020017	0	0	2	8	0	5
ENSMUSG00000105713	0	0	0	0	0	0
ENSMUSG00000106049	101	135	111	117	125	154
ENSMUSG00000106048	3	3	8	0	0	3
ENSMUSG00000106045	24	31	25	43	44	55
ENSMUSG00000106044	3	2	3	2	2	7
ENSMUSG00000106041	0	0	0	0	0	0
ENSMUSG00000106040	0	0	0	0	0	0
ENSMUSG00000106043	1	0	2	0	0	0
ENSMUSG00000106042	0	0	0	0	0	0
ENSMUSG00000094252	0	0	0	0	0	0
ENSMUSG00000094253	1	1	0	0	0	0
ENSMUSG00000094254	0	0	0	0	0	0
ENSMUSG00000094255	0	0	0	0	0	0
ENSMUSG00000110278	0	0	0	2	3	0
ENSMUSG00000110271	0	0	0	0	0	0
ENSMUSG00000110273	3	3	7	0	6	0
ENSMUSG00000110274	0	0	0	0	0	0
ENSMUSG00000110276	0	0	0	0	0	0
ENSMUSG00000112699	0	0	0	0	0	0
ENSMUSG00000055048	0	0	0	0	0	0
ENSMUSG00000107069	0	0	0	0	0	0
ENSMUSG00000112691	0	0	0	0	0	0
ENSMUSG00000112696	0	0	0	0	2	3
ENSMUSG00000114693	0	0	0	0	0	0
ENSMUSG00000114690	0	2	0	0	0	4
ENSMUSG00000029193	0	0	0	0	0	0
ENSMUSG00000114694	4	4	10	3	2	6
ENSMUSG00000114699	0	0	0	0	0	0
ENSMUSG00000114698	0	0	0	0	0	0
ENSMUSG00000110182	0	0	0	0	0	0
ENSMUSG00000110180	0	0	0	0	0	0
ENSMUSG00000110187	16	10	19	3	0	12
ENSMUSG00000110186	0	0	0	0	0	0
ENSMUSG00000110184	0	0	0	0	0	0
ENSMUSG00000113560	0	0	0	0	0	0
ENSMUSG00000100384	0	0	0	0	0	0
ENSMUSG00000113562	0	0	0	0	0	0
ENSMUSG00000113563	0	0	0	0	0	1
ENSMUSG00000113564	0	0	0	0	0	0
ENSMUSG00000038745	0	2	0	0	0	0
ENSMUSG00000113567	0	0	0	0	0	0
ENSMUSG00000100387	0	0	0	0	0	0
ENSMUSG00000094874	2	0	0	1	0	0
ENSMUSG00000094875	0	0	0	0	0	0
ENSMUSG00000094876	0	0	0	0	0	0
ENSMUSG00000109299	0	0	0	0	0	0
ENSMUSG00000109298	0	0	0	0	0	0
ENSMUSG00000051295	8	10	23	16	11	11
ENSMUSG00000021763	196	184	186	232	261	244
ENSMUSG00000109295	0	0	0	0	0	0
ENSMUSG00000109294	0	0	0	0	0	0
ENSMUSG00000105536	2	0	0	0	3	0
ENSMUSG00000094878	2	0	0	0	0	0
ENSMUSG00000105531	0	0	0	0	0	0
ENSMUSG00000105532	3	3	0	0	0	0

ENSMUSG00000109292	0	0	0	0	0	0
ENSMUSG00000087095	0	0	0	0	0	0
ENSMUSG00000087093	0	0	2	0	0	0
ENSMUSG00000112320	0	0	0	0	0	2
ENSMUSG00000082822	0	0	0	0	0	0
ENSMUSG00000082823	2	0	0	0	0	2
ENSMUSG00000082824	0	0	0	0	0	0
ENSMUSG00000082825	0	0	0	0	0	1
ENSMUSG00000066378	0	0	0	0	0	0
ENSMUSG00000082827	0	0	0	0	0	0
ENSMUSG00000066372	0	0	0	0	0	0
ENSMUSG00000005547	0	0	0	0	0	0
ENSMUSG00000005540	22	15	40	21	13	34
ENSMUSG00000082358	0	0	0	0	0	0
ENSMUSG00000111589	0	0	0	0	0	0
ENSMUSG00000111580	0	0	0	0	0	0
ENSMUSG00000111581	1	0	0	0	1	0
ENSMUSG00000111582	0	0	0	0	0	0
ENSMUSG00000111585	1	1	2	0	1	2
ENSMUSG00000111587	0	0	0	0	0	0
ENSMUSG00000109871	30	26	22	17	11	33
ENSMUSG00000109870	0	2	0	0	0	0
ENSMUSG00000051435	25	34	58	3	0	7
ENSMUSG00000026417	0	0	0	0	0	0
ENSMUSG00000026416	0	0	0	0	0	0
ENSMUSG00000026415	0	0	3	0	0	0
ENSMUSG00000082359	0	0	0	0	0	0
ENSMUSG00000109878	0	0	0	0	0	0
ENSMUSG00000026418	0	0	0	0	0	0
ENSMUSG00000114037	0	0	0	0	0	0
ENSMUSG00000108740	2	0	0	0	0	0
ENSMUSG00000114035	0	0	0	0	0	0
ENSMUSG00000114033	0	0	0	0	0	0
ENSMUSG00000114032	0	0	0	0	0	0
ENSMUSG00000114031	0	0	0	0	0	0
ENSMUSG00000114039	0	0	0	0	0	0
ENSMUSG00000114038	3	6	2	2	5	0
ENSMUSG00000050526	0	4	4	0	0	2
ENSMUSG00000059606	0	0	0	2	0	0
ENSMUSG00000086196	0	0	0	0	0	0
ENSMUSG00000068740	136	177	96	108	150	210
ENSMUSG00000113970	0	0	0	0	0	0
ENSMUSG00000065138	0	1	1	0	2	0
ENSMUSG00000083577	0	0	2	0	0	0
ENSMUSG00000104375	0	0	0	0	0	0
ENSMUSG00000104377	0	2	1	3	0	4
ENSMUSG00000104376	0	0	0	0	0	0
ENSMUSG00000104370	4	0	0	0	0	0
ENSMUSG00000104373	0	0	0	0	0	0
ENSMUSG00000088554	0	0	0	0	0	0
ENSMUSG00000056600	0	0	0	2	0	0
ENSMUSG00000056605	0	0	0	0	0	0
ENSMUSG00000046323	0	0	6	0	0	0
ENSMUSG00000046321	0	0	0	0	0	0
ENSMUSG00000045780	3	2	1	2	1	2
ENSMUSG00000095398	0	0	0	0	0	0
ENSMUSG00000095399	0	0	0	0	0	0
ENSMUSG00000095392	0	0	0	0	0	0

ENSMUSG00000095390	0	0	0	0	0	0
ENSMUSG00000086819	0	0	0	0	0	0
ENSMUSG00000044787	0	0	0	0	0	0
ENSMUSG00000053070	41	45	36	27	36	39
ENSMUSG00000034127	34	18	10	6	10	0
ENSMUSG00000034121	117	101	127	107	109	152
ENSMUSG00000095640	0	0	0	0	0	0
ENSMUSG00000095641	0	0	0	0	0	0
ENSMUSG00000052382	0	0	0	0	0	0
ENSMUSG00000095647	0	0	0	0	0	0
ENSMUSG00000039539	0	0	0	0	0	0
ENSMUSG00000098379	0	0	0	0	0	0
ENSMUSG00000098378	0	0	0	0	0	0
ENSMUSG00000098373	0	0	0	0	0	0
ENSMUSG00000098370	0	0	2	0	0	0
ENSMUSG00000053389	0	0	0	0	0	0
ENSMUSG00000053388	0	0	0	0	0	0
ENSMUSG00000042784	20	25	19	6	22	18
ENSMUSG00000042787	176	96	197	116	156	215
ENSMUSG00000081166	0	0	0	0	0	0
ENSMUSG00000024530	10	11	14	12	20	23
ENSMUSG00000093493	0	0	0	0	0	0
ENSMUSG00000110844	0	0	0	0	0	0
ENSMUSG00000093497	0	0	0	0	0	0
ENSMUSG00000087547	0	0	0	0	4	0
ENSMUSG00000028909	181	148	192	110	122	134
ENSMUSG00000112813	0	0	0	0	0	0
ENSMUSG00000093725	0	0	0	0	0	0
ENSMUSG00000093724	0	0	0	0	0	0
ENSMUSG00000093727	0	0	0	0	0	0
ENSMUSG00000094113	0	0	0	0	0	0
ENSMUSG00000049929	198	155	151	80	129	154
ENSMUSG00000049926	0	0	4	0	2	0
ENSMUSG00000081161	3	0	0	0	0	0
ENSMUSG00000093728	0	0	0	0	0	0
ENSMUSG00000094119	2	0	0	0	0	0
ENSMUSG00000094118	0	0	0	0	0	0
ENSMUSG00000101888	0	0	0	0	0	0
ENSMUSG00000070458	0	0	0	0	0	0
ENSMUSG00000081667	0	0	0	0	0	0
ENSMUSG00000047149	0	0	0	0	0	0
ENSMUSG00000081665	31	43	33	18	0	13
ENSMUSG00000081664	3	0	0	0	0	0
ENSMUSG00000081663	1	3	5	0	0	0
ENSMUSG00000081662	0	0	0	0	0	0
ENSMUSG00000081660	69	72	73	54	58	60
ENSMUSG00000081162	0	0	0	0	0	0
ENSMUSG00000047143	0	3	4	0	0	0
ENSMUSG00000047146	750	752	718	705	795	780
ENSMUSG00000081668	3	4	7	7	2	3
ENSMUSG00000073842	0	0	0	0	0	0
ENSMUSG00000073840	0	0	0	0	0	0
ENSMUSG00000077745	0	0	0	0	0	0
ENSMUSG00000073844	0	0	0	0	0	0
ENSMUSG00000077749	0	0	0	0	0	0
ENSMUSG00000055561	5	0	0	0	2	0
ENSMUSG00000055560	57	74	63	75	54	64
ENSMUSG00000055567	3	3	4	4	5	6

ENSMUSG00000081163	0	0	0	0	0	0
ENSMUSG00000017400	0	0	0	0	0	0
ENSMUSG00000101884	0	0	0	0	0	0
ENSMUSG00000096689	0	0	0	0	0	0
ENSMUSG00000098848	0	0	0	0	0	0
ENSMUSG00000088880	0	0	0	0	0	0
ENSMUSG00000099400	2	0	0	0	0	0
ENSMUSG00000092457	0	0	0	0	0	0
ENSMUSG00000092450	0	0	0	0	0	0
ENSMUSG00000092451	0	0	0	0	0	0
ENSMUSG00000092452	0	0	0	0	0	2
ENSMUSG00000099405	1	1	0	0	0	0
ENSMUSG00000099408	0	0	0	0	0	0
ENSMUSG00000099409	0	0	0	0	0	0
ENSMUSG00000092458	0	0	0	0	0	0
ENSMUSG00000101658	0	0	0	0	0	0
ENSMUSG00000023995	4	0	2	0	0	0
ENSMUSG00000088883	0	0	0	0	0	0
ENSMUSG00000088885	0	0	0	0	0	1
ENSMUSG00000049691	0	0	0	0	0	0
ENSMUSG00000030731	52	51	59	52	91	88
ENSMUSG00000043498	7	1	0	2	0	0
ENSMUSG00000030732	3	3	0	0	0	0
ENSMUSG00000082363	0	0	0	0	0	0
ENSMUSG00000088888	0	0	0	0	0	0
ENSMUSG00000082361	107	100	96	109	119	132
ENSMUSG00000082360	0	0	0	0	0	0
ENSMUSG00000082365	0	0	0	0	0	0
ENSMUSG00000082364	8	6	7	4	6	6
ENSMUSG00000082369	0	2	0	2	0	1
ENSMUSG00000082191	0	0	0	0	0	0
ENSMUSG00000081047	0	0	0	0	0	0
ENSMUSG00000093807	0	1	1	1	0	1
ENSMUSG00000066672	0	3	0	0	0	0
ENSMUSG00000112814	6	5	5	7	6	7
ENSMUSG00000102903	0	0	0	0	0	2
ENSMUSG00000093800	25	32	33	26	27	19
ENSMUSG00000092732	0	0	0	0	0	0
ENSMUSG00000100115	0	0	0	0	0	0
ENSMUSG00000072718	11	7	0	0	0	0
ENSMUSG00000100117	0	0	0	0	0	0
ENSMUSG00000100116	0	0	0	0	0	0
ENSMUSG00000100111	0	0	0	0	0	0
ENSMUSG00000100110	0	0	0	0	0	0
ENSMUSG00000100112	0	0	0	0	0	0
ENSMUSG00000082581	0	0	0	0	0	0
ENSMUSG00000094893	0	0	0	0	0	0
ENSMUSG00000064063	0	0	0	0	0	0
ENSMUSG00000084401	0	0	0	0	0	0
ENSMUSG00000094675	0	0	0	0	0	0
ENSMUSG00000084405	0	0	0	1	0	0
ENSMUSG00000094672	0	0	0	0	0	0
ENSMUSG00000042029	307	235	256	128	191	317
ENSMUSG00000000125	0	0	0	0	0	0
ENSMUSG00000068697	9	11	16	0	8	13
ENSMUSG00000068696	14	12	10	12	4	3
ENSMUSG00000080981	12	3	8	5	2	6
ENSMUSG00000080980	0	0	0	0	0	0

ENSMUSG00000080983	0	0	0	0	0	0
ENSMUSG00000080982	0	0	0	0	0	0
ENSMUSG00000080984	0	0	0	0	0	0
ENSMUSG00000085759	0	0	0	0	0	0
ENSMUSG00000080986	0	0	0	0	0	0
ENSMUSG00000085757	0	0	0	0	0	0
ENSMUSG00000085756	0	0	0	0	0	0
ENSMUSG00000085753	0	0	0	0	0	0
ENSMUSG00000037172	457	488	511	505	487	602
ENSMUSG00000037171	12	5	2	9	0	8
ENSMUSG00000083188	0	0	0	0	0	0
ENSMUSG00000107469	0	0	0	0	0	0
ENSMUSG00000100525	0	0	0	0	0	0
ENSMUSG00000107464	0	0	0	0	0	0
ENSMUSG00000107465	0	0	0	0	0	0
ENSMUSG00000107466	0	0	0	0	0	0
ENSMUSG00000107467	0	0	0	0	0	0
ENSMUSG00000083180	0	0	0	0	0	0
ENSMUSG00000083181	0	0	0	0	0	0
ENSMUSG00000083182	0	0	0	0	0	0
ENSMUSG00000107463	0	0	0	0	0	0
ENSMUSG00000079539	0	0	0	0	0	0
ENSMUSG00000079534	0	0	0	0	0	9
ENSMUSG00000079536	0	0	0	0	0	0
ENSMUSG00000075296	0	0	0	0	0	0
ENSMUSG00000054351	0	0	0	0	0	0
ENSMUSG00000079531	0	0	0	0	0	4
ENSMUSG00000079532	0	0	0	0	0	0
ENSMUSG00000061259	1	0	0	0	0	0
ENSMUSG00000075006	0	0	0	0	0	1
ENSMUSG00000111309	0	0	0	0	0	0
ENSMUSG00000104834	0	0	0	0	0	0
ENSMUSG00000109013	0	0	0	0	0	0
ENSMUSG00000082421	0	0	0	0	0	0
ENSMUSG00000075874	0	1	0	0	0	0
ENSMUSG00000065005	0	0	0	0	0	0
ENSMUSG00000065004	0	0	0	0	0	0
ENSMUSG00000065007	0	0	0	0	0	0
ENSMUSG00000065006	0	0	0	0	0	1
ENSMUSG00000099186	0	0	0	0	0	0
ENSMUSG00000099184	0	0	0	0	0	0
ENSMUSG00000099182	2	0	2	0	0	0
ENSMUSG00000099189	1	1	1	1	1	1
ENSMUSG00000099188	0	0	0	0	0	0
ENSMUSG00000073433	23	21	38	5	2	9
ENSMUSG00000073430	0	0	0	0	0	0
ENSMUSG00000026188	4	0	8	4	0	0
ENSMUSG00000062782	0	0	0	0	0	0
ENSMUSG00000062785	20	22	6	12	5	34
ENSMUSG00000062257	72	26	24	96	50	51
ENSMUSG00000089469	0	0	0	0	0	0
ENSMUSG00000089468	0	0	0	0	0	0
ENSMUSG00000082472	0	0	0	0	0	0
ENSMUSG00000089464	0	0	0	0	0	0
ENSMUSG00000089467	0	0	0	0	0	0
ENSMUSG00000089461	0	0	0	0	0	0
ENSMUSG00000089460	0	0	0	0	0	0
ENSMUSG00000089462	0	0	0	0	0	0

ENSMUSG00000063780	0	2	0	0	0	0
ENSMUSG00000102236	0	2	2	3	2	0
ENSMUSG00000084017	2	0	7	5	0	2
ENSMUSG00000102235	3	0	3	0	0	0
ENSMUSG00000102233	0	0	0	0	1	0
ENSMUSG00000102230	0	0	0	0	0	0
ENSMUSG00000102231	0	0	0	0	0	0
ENSMUSG00000102239	0	0	0	0	0	0
ENSMUSG00000059379	0	0	0	0	0	0
ENSMUSG00000058537	0	0	0	0	0	2
ENSMUSG00000089023	0	0	0	0	0	0
ENSMUSG00000069633	215	194	224	188	195	282
ENSMUSG00000093454	0	0	0	0	0	0
ENSMUSG00000096724	0	0	0	0	0	0
ENSMUSG00000096722	12	16	11	11	12	16
ENSMUSG00000096721	0	0	0	0	0	0
ENSMUSG00000084018	0	0	0	0	0	0
ENSMUSG00000096728	0	0	0	0	0	0
ENSMUSG00000096729	6	4	5	5	3	6
ENSMUSG00000031105	99	110	119	89	69	113
ENSMUSG00000031104	12	22	14	0	3	4
ENSMUSG00000063120	0	0	0	0	0	0
ENSMUSG00000021356	76	67	92	45	55	81
ENSMUSG00000057710	0	0	0	0	0	0
ENSMUSG00000057715	0	3	0	3	0	0
ENSMUSG00000057716	30	39	11	24	54	25
ENSMUSG00000109114	0	0	0	0	0	0
ENSMUSG00000050092	0	0	0	0	0	0
ENSMUSG00000104173	0	0	0	0	0	0
ENSMUSG00000104172	0	0	0	0	0	0
ENSMUSG00000104177	0	0	0	0	0	0
ENSMUSG00000104176	0	0	0	0	0	0
ENSMUSG00000104175	0	0	0	0	0	0
ENSMUSG00000104178	0	0	0	0	0	0
ENSMUSG00000059729	1	3	1	2	2	1
ENSMUSG00000113120	0	0	0	0	0	0
ENSMUSG00000113121	2	0	0	0	0	0
ENSMUSG00000084135	0	0	0	0	0	0
ENSMUSG00000091189	0	0	0	0	0	0
ENSMUSG00000056445	23	1	7	22	20	10
ENSMUSG00000091183	196	181	178	87	75	110
ENSMUSG00000095418	0	0	0	0	0	0
ENSMUSG00000110846	0	3	0	0	0	0
ENSMUSG00000110849	0	0	0	0	0	0
ENSMUSG00000099702	0	1	0	0	0	1
ENSMUSG00000104825	0	0	0	0	0	0
ENSMUSG00000104827	5	12	5	0	0	0
ENSMUSG00000056197	0	0	0	0	0	0
ENSMUSG00000092536	2	0	0	0	0	0
ENSMUSG00000092535	0	0	0	0	0	0
ENSMUSG00000070150	2	0	0	0	0	0
ENSMUSG00000077574	0	0	0	0	0	0
ENSMUSG00000077578	0	1	1	0	1	1
ENSMUSG00000113127	0	0	0	0	0	0
ENSMUSG00000095402	0	0	0	0	0	0
ENSMUSG00000095401	0	0	0	0	0	0
ENSMUSG00000065940	0	0	0	0	0	0
ENSMUSG00000065943	2	2	1	0	0	2

ENSMUSG00000065945	0	0	0	0	0	0
ENSMUSG00000095408	0	0	0	1	0	1
ENSMUSG00000095409	0	0	0	0	0	0
ENSMUSG00000027965	0	0	0	0	0	5
ENSMUSG00000027966	614	142	225	8	5	4
ENSMUSG00000027967	0	0	0	0	0	0
ENSMUSG00000093122	0	0	0	0	0	0
ENSMUSG00000086268	0	4	0	0	0	5
ENSMUSG00000093120	2	2	1	1	1	1
ENSMUSG00000093125	0	0	0	0	0	0
ENSMUSG00000086262	0	0	0	0	0	0
ENSMUSG00000086263	0	0	0	0	0	0
ENSMUSG00000093129	0	0	0	0	0	0
ENSMUSG00000086261	0	0	0	0	0	0
ENSMUSG00000086267	0	0	0	0	0	0
ENSMUSG00000086264	0	0	0	0	0	0
ENSMUSG00000086265	2	2	1	1	1	1
ENSMUSG00000052631	0	0	2	0	5	0
ENSMUSG00000028294	0	0	0	0	2	0
ENSMUSG00000028298	0	0	0	0	0	0
ENSMUSG00000109506	4	0	0	0	0	0
ENSMUSG00000040929	369	401	366	240	263	350
ENSMUSG00000109500	0	0	0	0	0	0
ENSMUSG00000109503	0	0	0	0	0	0
ENSMUSG00000112558	2	0	0	0	0	3
ENSMUSG00000112557	5	6	1	4	5	4
ENSMUSG00000112554	0	0	0	0	0	2
ENSMUSG00000109502	1	1	1	1	1	0
ENSMUSG00000112552	0	0	0	0	0	0
ENSMUSG00000112553	0	0	0	0	0	0
ENSMUSG00000055360	0	0	0	0	0	0
ENSMUSG00000018698	0	0	0	0	0	0
ENSMUSG00000098537	1	2	2	3	1	2
ENSMUSG00000100864	0	0	0	0	0	0
ENSMUSG00000092292	0	0	0	0	0	0
ENSMUSG00000092293	0	0	0	0	0	0
ENSMUSG00000028435	0	0	0	0	0	0
ENSMUSG00000028434	58	56	45	111	98	146
ENSMUSG00000092296	0	0	0	0	0	0
ENSMUSG00000092297	0	0	0	0	0	0
ENSMUSG00000092294	0	0	0	0	0	0
ENSMUSG00000092295	0	0	0	0	0	0
ENSMUSG00000092298	0	0	0	0	0	0
ENSMUSG00000093058	0	0	0	0	0	0
ENSMUSG00000029522	0	0	0	0	0	0
ENSMUSG00000029526	0	0	0	0	5	0
ENSMUSG00000024731	0	0	0	0	0	0
ENSMUSG00000024738	0	0	0	0	0	0
ENSMUSG00000020607	39	35	13	33	48	55
ENSMUSG00000020609	0	2	0	0	0	0
ENSMUSG00000097118	0	0	0	0	0	0
ENSMUSG00000097116	0	0	0	0	0	0
ENSMUSG00000097115	4	2	9	0	4	9
ENSMUSG00000097112	0	0	0	0	0	0
ENSMUSG00000097113	13	9	11	0	0	9
ENSMUSG00000097110	0	0	0	0	0	0
ENSMUSG00000046958	5	0	0	0	0	0
ENSMUSG00000046957	0	0	0	3	0	0

ENSMUSG00000046952	11	9	16	8	11	7
ENSMUSG00000110024	0	0	0	0	0	0
ENSMUSG00000110025	0	0	0	0	0	0
ENSMUSG00000110027	0	0	0	0	0	0
ENSMUSG00000102678	0	0	0	0	0	0
ENSMUSG00000070551	0	0	0	0	0	0
ENSMUSG00000070550	0	0	0	0	0	0
ENSMUSG00000054102	0	0	0	0	0	0
ENSMUSG00000070552	0	0	0	0	0	0
ENSMUSG00000054106	0	0	0	0	0	0
ENSMUSG00000104743	0	0	0	0	0	0
ENSMUSG00000108280	0	0	5	0	0	2
ENSMUSG00000108285	0	0	0	0	0	0
ENSMUSG00000108284	0	0	0	2	0	1
ENSMUSG00000108287	0	0	0	0	0	0
ENSMUSG00000039179	0	0	0	0	0	0
ENSMUSG00000108289	0	0	0	0	0	0
ENSMUSG00000108288	0	0	0	0	0	0
ENSMUSG00000058420	103	96	133	65	47	45
ENSMUSG00000102674	2	0	0	0	0	0
ENSMUSG00000105709	3	3	0	6	0	0
ENSMUSG00000105708	6	7	4	15	11	0
ENSMUSG00000109488	0	0	0	0	0	0
ENSMUSG00000109489	5	9	8	9	10	11
ENSMUSG00000105703	134	112	157	246	281	234
ENSMUSG00000060918	1	1	0	0	0	1
ENSMUSG00000109487	0	0	0	0	0	0
ENSMUSG00000109480	0	0	0	0	0	0
ENSMUSG00000105705	0	0	0	0	0	0
ENSMUSG00000097975	2	0	0	0	0	0
ENSMUSG00000044083	2	0	2	0	0	0
ENSMUSG00000044084	0	0	4	4	0	0
ENSMUSG00000108674	0	0	0	0	3	0
ENSMUSG00000108759	0	0	0	0	0	0
ENSMUSG00000108758	0	0	0	0	0	2
ENSMUSG00000005089	39	61	75	16	11	21
ENSMUSG00000112834	0	0	0	0	0	0
ENSMUSG00000108751	0	0	0	0	0	0
ENSMUSG00000108757	0	0	0	0	0	0
ENSMUSG00000108756	0	0	0	0	2	0
ENSMUSG00000108754	1	0	0	0	0	0
ENSMUSG00000083348	0	0	0	0	0	0
ENSMUSG00000083343	0	0	0	0	0	0
ENSMUSG00000083346	0	0	0	0	0	0
ENSMUSG00000083347	0	0	0	0	0	0
ENSMUSG00000083345	0	0	0	0	0	0
ENSMUSG00000111776	6	10	7	11	8	16
ENSMUSG00000111774	11	0	3	0	6	0
ENSMUSG00000111772	0	0	0	0	0	0
ENSMUSG00000111771	0	0	0	0	0	0
ENSMUSG00000111770	0	0	0	0	0	0
ENSMUSG00000036521	0	0	0	0	0	0
ENSMUSG00000108673	0	0	0	0	0	0
ENSMUSG00000111778	0	0	0	0	0	0
ENSMUSG00000057457	4	15	9	3	4	7
ENSMUSG00000057455	0	0	0	0	0	0
ENSMUSG00000057454	0	0	0	0	0	0
ENSMUSG00000025955	0	0	0	3	0	0

ENSMUSG00000025952	0	0	0	0	0	0
ENSMUSG00000099767	0	0	0	0	0	0
ENSMUSG00000093912	0	1	3	0	0	1
ENSMUSG00000093913	0	0	0	0	0	0
ENSMUSG00000093910	21	11	20	5	0	9
ENSMUSG00000093916	0	0	0	2	0	0
ENSMUSG00000093917	0	0	0	0	0	0
ENSMUSG00000093914	2	1	1	0	0	1
ENSMUSG00000093918	0	0	0	0	0	0
ENSMUSG00000026609	7	6	12	17	12	25
ENSMUSG00000044624	0	0	0	0	2	0
ENSMUSG00000036816	12	14	6	21	13	9
ENSMUSG00000044626	0	5	0	3	0	7
ENSMUSG00000110223	0	0	0	0	0	0
ENSMUSG00000109596	0	0	0	0	0	0
ENSMUSG00000110227	0	0	0	0	0	0
ENSMUSG00000001504	67	103	87	140	64	94
ENSMUSG00000105335	0	0	0	0	0	0
ENSMUSG00000007907	0	0	0	0	0	0
ENSMUSG00000007908	9	9	3	2	1	5
ENSMUSG00000053228	0	0	0	0	0	0
ENSMUSG00000023247	0	0	0	2	0	0
ENSMUSG00000101348	0	0	0	0	0	2
ENSMUSG00000078177	3	2	4	0	3	3
ENSMUSG00000078179	3	0	2	0	1	2
ENSMUSG00000090333	0	0	0	0	0	0
ENSMUSG00000090332	0	0	0	0	0	0
ENSMUSG00000090336	0	0	6	0	2	0
ENSMUSG00000048992	0	0	0	0	0	0
ENSMUSG00000081253	0	0	0	0	0	0
ENSMUSG00000081250	0	3	0	3	3	8
ENSMUSG00000048996	0	3	0	3	0	0
ENSMUSG00000048997	74	78	42	60	74	95
ENSMUSG00000048994	0	2	0	0	0	0
ENSMUSG00000081255	0	0	2	0	0	0
ENSMUSG00000101346	0	0	0	0	0	0
ENSMUSG00000042662	10	6	5	0	0	0
ENSMUSG00000076229	0	0	0	0	0	0
ENSMUSG00000109597	1	0	0	0	0	0
ENSMUSG00000046173	0	0	0	0	0	0
ENSMUSG00000087470	0	0	0	0	0	0
ENSMUSG00000098658	3	0	5	3	2	2
ENSMUSG00000098659	0	0	0	0	0	0
ENSMUSG00000041642	227	175	315	82	110	122
ENSMUSG00000098655	0	1	0	0	0	0
ENSMUSG00000073141	0	0	0	0	0	0
ENSMUSG00000101344	0	0	0	1	1	0
ENSMUSG00000073145	0	4	12	8	7	5
ENSMUSG00000080707	0	0	0	0	0	0
ENSMUSG00000080706	0	0	0	0	0	0
ENSMUSG00000080705	0	0	0	0	0	0
ENSMUSG00000080704	0	0	0	0	0	0
ENSMUSG00000086701	0	0	3	0	0	0
ENSMUSG00000086703	0	0	0	0	0	0
ENSMUSG00000086702	0	0	0	0	0	0
ENSMUSG00000086707	0	0	0	0	0	0
ENSMUSG00000086706	0	0	2	0	0	0
ENSMUSG00000086709	0	0	0	0	0	0

ENSMUSG00000075760	0	0	0	0	0	0
ENSMUSG00000059854	5	4	12	4	10	3
ENSMUSG00000008438	18	26	29	34	28	37
ENSMUSG00000031344	6	8	10	0	5	0
ENSMUSG00000049719	0	2	0	0	0	0
ENSMUSG00000094808	0	0	0	0	0	0
ENSMUSG00000082288	0	0	0	0	0	0
ENSMUSG00000093679	0	0	0	0	0	0
ENSMUSG00000088368	0	0	0	0	0	0
ENSMUSG00000082280	2	0	0	0	0	0
ENSMUSG00000082281	0	0	0	0	0	0
ENSMUSG00000037544	52	50	34	19	14	28
ENSMUSG00000101479	0	0	0	0	0	0
ENSMUSG00000101478	0	0	0	0	0	0
ENSMUSG00000101475	0	0	0	0	0	0
ENSMUSG00000101474	0	0	0	0	0	0
ENSMUSG00000040536	66	93	83	47	86	59
ENSMUSG00000040537	1053	1257	1188	1487	1582	1717
ENSMUSG00000101471	0	0	0	0	0	0
ENSMUSG00000101470	0	0	0	0	0	0
ENSMUSG00000101473	0	0	0	0	0	0
ENSMUSG00000040533	0	0	0	0	0	0
ENSMUSG00000098664	3	0	0	4	0	0
ENSMUSG00000082136	0	0	0	0	0	0
ENSMUSG00000082134	2	0	0	1	0	0
ENSMUSG00000082135	0	0	0	0	0	0
ENSMUSG00000082138	0	0	0	0	0	0
ENSMUSG00000113048	0	0	0	0	0	0
ENSMUSG00000032589	8	13	10	2	0	10
ENSMUSG00000099235	0	0	0	0	0	0
ENSMUSG00000099237	0	0	0	0	0	0
ENSMUSG00000099236	0	1	1	1	1	0
ENSMUSG00000032584	217	212	225	41	88	106
ENSMUSG00000032586	25	15	18	21	27	32
ENSMUSG00000038224	0	0	0	0	7	0
ENSMUSG00000088817	0	0	0	0	0	0
ENSMUSG00000088816	0	0	1	0	0	0
ENSMUSG00000083661	2	0	0	0	0	0
ENSMUSG00000083665	0	0	0	0	0	0
ENSMUSG00000083664	0	0	0	0	0	0
ENSMUSG00000089244	0	0	0	0	0	0
ENSMUSG00000089247	0	0	0	0	0	0
ENSMUSG00000100708	0	0	0	0	0	0
ENSMUSG00000100709	0	0	0	0	0	0
ENSMUSG00000100704	0	0	0	0	0	0
ENSMUSG00000100705	0	0	0	0	0	0
ENSMUSG00000100700	0	0	0	0	0	0
ENSMUSG00000100702	1	0	2	2	3	1
ENSMUSG00000102414	0	3	0	0	0	2
ENSMUSG00000102418	25	7	52	0	6	3
ENSMUSG00000087945	0	0	0	0	0	0
ENSMUSG00000041377	4	0	0	0	0	0
ENSMUSG00000087947	0	0	0	0	0	0
ENSMUSG00000087941	0	0	0	0	0	0
ENSMUSG00000087942	0	0	0	0	0	0
ENSMUSG00000041372	123	60	28	0	0	0
ENSMUSG00000085549	0	0	0	0	0	0
ENSMUSG00000085545	0	0	0	0	0	0

ENSMUSG00000085542	0	0	0	0	0	0
ENSMUSG00000085540	0	0	2	0	0	6
ENSMUSG00000088313	0	0	0	0	0	0
ENSMUSG00000074283	84	79	89	72	78	98
ENSMUSG00000074282	84	86	100	56	50	83
ENSMUSG00000074289	0	0	0	0	0	0
ENSMUSG00000064569	0	0	0	0	0	0
ENSMUSG00000079703	0	0	0	0	0	0
ENSMUSG00000079702	0	0	0	0	0	0
ENSMUSG00000079701	0	0	0	0	0	0
ENSMUSG00000079705	0	0	0	0	0	0
ENSMUSG00000079704	0	0	0	0	0	0
ENSMUSG00000113485	0	0	0	2	2	4
ENSMUSG00000023000	26	39	54	14	20	27
ENSMUSG00000023009	182	210	231	97	136	192
ENSMUSG00000095333	0	0	0	0	0	0
ENSMUSG00000088672	0	0	0	0	0	0
ENSMUSG00000088671	0	0	0	0	0	0
ENSMUSG00000088677	0	3	3	2	4	0
ENSMUSG00000088674	0	0	0	0	1	0
ENSMUSG00000088679	0	0	0	0	0	0
ENSMUSG00000088678	0	0	0	0	0	0
ENSMUSG00000021539	0	0	3	0	0	0
ENSMUSG00000039579	54	63	45	46	46	51
ENSMUSG00000021534	0	2	0	2	0	0
ENSMUSG00000039577	13	21	25	0	8	5
ENSMUSG00000107164	3	0	0	0	0	0
ENSMUSG00000107167	0	0	0	0	0	0
ENSMUSG00000029273	0	0	0	0	0	0
ENSMUSG00000036634	8	0	0	3	0	2
ENSMUSG00000065215	0	0	0	0	0	0
ENSMUSG00000065212	0	0	0	0	0	0
ENSMUSG00000034677	0	0	0	0	0	0
ENSMUSG00000083683	0	0	0	0	0	0
ENSMUSG00000083682	0	0	0	0	0	0
ENSMUSG00000071165	0	0	0	0	0	0
ENSMUSG00000047564	0	0	0	0	0	0
ENSMUSG00000106412	0	0	0	0	0	0
ENSMUSG00000106413	0	0	0	0	0	0
ENSMUSG00000103426	0	0	0	0	0	0
ENSMUSG00000106411	3	4	3	1	3	1
ENSMUSG00000106416	10	14	28	19	12	10
ENSMUSG00000103421	0	0	0	0	0	0
ENSMUSG00000103423	12	15	11	25	10	14
ENSMUSG00000022847	0	7	8	10	0	7
ENSMUSG00000111807	0	0	0	0	0	0
ENSMUSG00000047565	7	4	2	0	3	4
ENSMUSG00000029092	35	22	31	18	17	14
ENSMUSG00000026251	0	0	0	0	0	0
ENSMUSG00000029097	167	224	223	203	176	194
ENSMUSG00000047562	0	0	0	0	0	0
ENSMUSG00000050840	61	53	52	67	64	115
ENSMUSG00000020723	0	0	0	0	0	0
ENSMUSG00000111023	0	0	0	0	0	0
ENSMUSG00000111021	2	0	0	0	0	0
ENSMUSG00000113594	0	0	0	0	0	0
ENSMUSG00000097686	0	0	0	0	0	0
ENSMUSG00000027887	22	22	15	10	14	18

ENSMUSG00000054342	30	31	52	2	3	12
ENSMUSG00000034416	2	0	4	0	5	7
ENSMUSG00000099928	0	0	0	0	0	0
ENSMUSG00000034098	2	0	0	0	3	3
ENSMUSG00000112381	8	7	14	3	8	13
ENSMUSG00000097689	0	0	0	0	0	0
ENSMUSG00000109648	0	0	0	0	0	0
ENSMUSG00000094217	0	0	0	0	0	0
ENSMUSG00000051648	6	5	2	18	3	13
ENSMUSG00000105146	0	0	0	0	0	0
ENSMUSG00000114240	0	0	0	0	0	0
ENSMUSG00000114242	0	0	0	0	0	0
ENSMUSG00000114245	0	0	0	0	0	0
ENSMUSG00000114247	0	0	0	0	0	0
ENSMUSG00000114248	0	0	0	0	0	0
ENSMUSG00000102981	0	0	0	0	0	0
ENSMUSG00000063549	0	0	0	0	0	0
ENSMUSG00000108519	0	2	0	0	4	2
ENSMUSG00000112958	0	0	0	0	0	0
ENSMUSG00000112952	8	6	7	20	16	10
ENSMUSG00000108510	0	0	0	0	0	0
ENSMUSG00000108513	7	0	0	0	0	0
ENSMUSG00000112951	0	0	0	0	0	0
ENSMUSG00000108515	2	2	0	0	0	0
ENSMUSG00000112957	0	0	0	0	0	0
ENSMUSG00000112954	0	0	0	0	0	0
ENSMUSG00000108516	0	0	0	0	0	0
ENSMUSG00000108236	0	0	0	0	0	0
ENSMUSG00000067215	0	0	0	0	0	0
ENSMUSG00000031688	0	0	0	0	0	0
ENSMUSG00000096082	0	0	0	0	0	0
ENSMUSG00000096080	0	0	0	0	0	0
ENSMUSG00000096087	0	0	0	0	0	0
ENSMUSG00000067219	3	0	0	0	4	0
ENSMUSG00000096555	0	0	0	0	0	0
ENSMUSG00000057697	2	0	3	0	0	0
ENSMUSG00000096550	0	0	0	0	0	0
ENSMUSG00000057699	0	0	0	0	0	0
ENSMUSG00000091680	0	0	0	0	1	0
ENSMUSG00000091682	0	0	4	0	0	0
ENSMUSG00000108230	0	0	0	0	0	0
ENSMUSG00000091686	0	0	4	0	0	8
ENSMUSG00000076052	0	0	1	1	0	0
ENSMUSG00000021090	0	6	9	3	8	6
ENSMUSG00000021097	82	120	102	101	92	109
ENSMUSG00000021095	0	0	0	0	0	0
ENSMUSG00000021098	6	16	4	0	8	3
ENSMUSG00000021099	0	0	0	0	0	0
ENSMUSG00000064855	0	0	0	0	0	0
ENSMUSG00000064854	0	0	0	0	0	0
ENSMUSG00000101134	0	0	0	0	0	0
ENSMUSG00000114068	16	20	7	9	7	7
ENSMUSG00000101137	0	0	0	0	0	0
ENSMUSG00000101130	0	0	0	2	0	0
ENSMUSG00000101131	0	0	0	0	0	0
ENSMUSG00000111682	0	0	0	0	0	0
ENSMUSG00000101138	0	0	0	0	0	0
ENSMUSG00000109820	0	0	0	0	0	0

ENSMUSG00000109821	0	0	0	0	0	0
ENSMUSG00000077791	0	0	0	0	0	0
ENSMUSG00000045111	0	0	0	0	0	0
ENSMUSG00000109827	0	0	0	0	0	0
ENSMUSG00000111360	2	4	6	6	3	0
ENSMUSG00000113381	0	0	0	1	0	0
ENSMUSG00000114062	0	0	0	0	0	0
ENSMUSG00000114063	0	0	0	0	0	0
ENSMUSG00000064977	0	0	0	0	0	0
ENSMUSG00000068437	0	1	0	0	0	0
ENSMUSG00000064972	0	0	0	0	0	0
ENSMUSG00000064971	0	0	0	0	0	0
ENSMUSG00000092533	0	0	0	0	0	0
ENSMUSG00000091027	1	0	0	0	0	1
ENSMUSG00000091022	0	0	0	0	0	0
ENSMUSG00000094154	0	0	1	0	0	0
ENSMUSG00000026959	0	0	0	0	5	0
ENSMUSG00000026955	20	8	3	0	10	8
ENSMUSG00000081013	1	0	0	0	0	0
ENSMUSG00000081014	2	0	0	0	0	0
ENSMUSG00000081017	0	0	0	0	0	0
ENSMUSG00000081018	0	0	0	0	0	0
ENSMUSG00000084909	0	0	0	0	0	0
ENSMUSG00000056258	8	0	10	0	0	0
ENSMUSG00000114768	0	0	0	2	0	0
ENSMUSG00000111982	0	0	0	0	0	0
ENSMUSG00000048602	22	19	20	41	39	70
ENSMUSG00000111988	0	0	0	0	0	0
ENSMUSG00000109846	0	0	0	2	2	0
ENSMUSG00000073243	0	0	0	0	0	0
ENSMUSG00000095670	0	0	0	0	0	0
ENSMUSG00000108198	0	0	0	0	0	0
ENSMUSG00000086549	4	2	0	0	0	0
ENSMUSG00000086548	0	0	0	0	0	0
ENSMUSG00000086547	6	5	6	3	2	0
ENSMUSG00000086545	0	0	0	0	0	0
ENSMUSG00000086542	4	0	4	0	4	0
ENSMUSG00000086541	26	37	27	6	4	9
ENSMUSG00000086540	0	0	0	0	0	0
ENSMUSG00000104635	0	0	0	0	0	0
ENSMUSG00000108197	0	2	0	5	0	0
ENSMUSG00000079606	0	0	0	0	0	0
ENSMUSG00000077138	0	0	0	0	0	0
ENSMUSG00000040505	0	0	0	0	0	0
ENSMUSG00000077131	0	0	0	0	0	0
ENSMUSG00000112189	0	0	0	0	0	0
ENSMUSG00000112188	7	14	0	8	6	4
ENSMUSG00000112181	0	0	0	0	0	0
ENSMUSG00000112183	0	0	0	0	0	0
ENSMUSG00000112185	0	0	0	0	0	0
ENSMUSG00000112184	0	0	0	0	0	0
ENSMUSG00000112187	0	0	0	0	0	0
ENSMUSG00000051980	0	0	0	1	0	4
ENSMUSG00000051986	0	0	0	0	0	0
ENSMUSG00000051985	0	0	2	0	3	0
ENSMUSG00000049551	33	44	32	26	20	22
ENSMUSG00000086093	0	0	0	0	0	0
ENSMUSG00000086092	0	0	0	0	0	0

ENSMUSG00000086095	18	18	23	21	34	29
ENSMUSG00000086097	0	0	0	0	0	0
ENSMUSG00000049556	55	20	21	5	23	15
ENSMUSG00000084503	0	0	0	0	0	0
ENSMUSG00000106009	1	0	0	0	0	0
ENSMUSG00000065107	0	0	0	0	0	0
ENSMUSG00000114562	0	0	0	0	0	0
ENSMUSG00000092647	0	0	0	0	0	0
ENSMUSG00000092643	0	0	0	0	0	0
ENSMUSG00000020052	0	0	0	0	0	0
ENSMUSG00000028591	34	3	32	0	0	0
ENSMUSG00000096949	0	0	0	0	0	0
ENSMUSG00000098920	0	0	0	0	0	0
ENSMUSG00000096943	0	0	0	0	0	3
ENSMUSG00000098924	0	0	0	0	0	0
ENSMUSG00000106351	0	0	0	0	0	0
ENSMUSG00000106350	0	0	0	0	1	0
ENSMUSG00000106353	2	2	0	0	0	2
ENSMUSG00000106352	0	0	0	0	0	0
ENSMUSG00000106355	2	0	0	0	0	0
ENSMUSG00000106357	9	2	7	0	0	2
ENSMUSG00000106356	33	36	17	38	19	42
ENSMUSG00000106359	0	0	0	0	0	0
ENSMUSG00000113271	0	0	0	0	0	0
ENSMUSG00000113272	0	0	0	0	0	0
ENSMUSG00000113273	0	0	0	0	0	0
ENSMUSG00000113276	0	0	0	0	0	0
ENSMUSG00000082074	0	0	0	0	0	0
ENSMUSG00000047237	0	0	0	0	0	0
ENSMUSG00000047230	0	0	0	0	0	0
ENSMUSG00000071037	43	64	42	42	44	48
ENSMUSG00000109360	0	0	0	0	0	0
ENSMUSG00000109365	0	0	0	0	0	0
ENSMUSG00000109366	9	11	1	7	8	12
ENSMUSG00000109368	0	0	0	0	0	0
ENSMUSG00000082732	0	0	0	0	0	0
ENSMUSG00000082733	0	0	0	0	0	0
ENSMUSG00000082734	0	0	0	0	0	0
ENSMUSG00000082735	0	0	0	0	0	0
ENSMUSG00000082736	0	0	0	0	0	0
ENSMUSG00000082739	0	0	0	0	0	0
ENSMUSG00000114209	0	0	2	0	0	0
ENSMUSG00000092111	0	0	0	0	0	0
ENSMUSG00000083069	0	0	0	0	0	0
ENSMUSG00000083068	0	0	0	0	0	3
ENSMUSG00000083067	0	0	0	0	0	0
ENSMUSG00000083066	0	0	0	0	0	0
ENSMUSG00000083065	0	0	0	0	0	0
ENSMUSG00000083063	0	0	0	0	0	0
ENSMUSG00000083062	0	0	0	0	0	0
ENSMUSG00000083060	0	0	0	0	0	0
ENSMUSG00000114727	0	0	0	0	0	0
ENSMUSG00000114726	0	0	3	4	11	4
ENSMUSG00000114725	0	0	0	0	0	2
ENSMUSG00000114724	4	0	0	0	0	0
ENSMUSG00000108775	0	0	0	0	0	0
ENSMUSG00000114721	0	0	0	0	0	0
ENSMUSG00000114720	0	0	0	0	0	0

ENSMUSG00000114729	0	1	0	0	0	0
ENSMUSG00000110617	3	6	5	4	6	8
ENSMUSG00000110616	0	0	0	0	0	0
ENSMUSG00000110111	0	0	0	0	0	0
ENSMUSG00000067745	0	0	0	0	0	0
ENSMUSG00000110610	2	3	3	0	0	3
ENSMUSG00000110619	0	0	2	1	1	0
ENSMUSG00000110618	25	14	41	13	6	19
ENSMUSG00000099331	0	0	0	0	0	0
ENSMUSG00000099332	0	0	0	0	0	0
ENSMUSG00000108774	0	0	0	0	0	0
ENSMUSG00000099334	0	0	0	0	0	0
ENSMUSG00000099335	0	0	0	0	0	0
ENSMUSG00000099336	0	0	0	0	0	0
ENSMUSG00000099339	0	0	0	1	0	0
ENSMUSG00000062687	3	0	0	0	3	0
ENSMUSG00000109252	3	2	6	6	9	12
ENSMUSG00000100187	0	0	0	0	0	0
ENSMUSG00000108776	0	0	0	0	0	0
ENSMUSG00000070072	0	0	0	0	0	0
ENSMUSG00000070073	0	0	0	0	0	0
ENSMUSG00000070071	0	0	0	0	0	0
ENSMUSG00000070076	0	0	0	0	0	0
ENSMUSG00000099228	1	0	1	1	1	0
ENSMUSG00000070078	0	0	0	0	0	0
ENSMUSG00000099229	0	0	0	0	0	0
ENSMUSG00000112656	0	0	0	0	0	0
ENSMUSG00000112651	0	0	0	0	0	0
ENSMUSG00000099224	0	0	2	0	0	1
ENSMUSG00000102150	0	0	0	0	0	0
ENSMUSG00000093895	0	0	0	0	0	0
ENSMUSG00000112883	0	0	0	0	0	0
ENSMUSG00000093892	0	0	0	0	0	0
ENSMUSG00000093891	0	0	1	0	0	1
ENSMUSG00000093890	2	0	0	0	0	0
ENSMUSG00000112884	0	0	0	0	0	0
ENSMUSG00000113277	0	0	0	0	0	0
ENSMUSG00000093899	0	0	0	0	0	0
ENSMUSG00000093898	6	10	6	7	5	13
ENSMUSG00000064512	0	1	0	0	0	0
ENSMUSG00000081862	0	0	0	0	0	0
ENSMUSG00000099220	0	0	0	0	0	0
ENSMUSG00000081864	0	0	0	0	0	0
ENSMUSG00000096986	0	0	4	0	0	0
ENSMUSG00000090701	0	0	0	0	0	0
ENSMUSG00000085034	0	0	0	0	0	0
ENSMUSG00000085035	0	0	0	0	0	0
ENSMUSG00000085032	0	0	0	0	0	0
ENSMUSG00000074517	18	18	20	16	6	20
ENSMUSG00000090709	0	0	0	0	0	0
ENSMUSG00000077896	0	0	0	0	0	0
ENSMUSG00000077892	0	0	0	0	0	0
ENSMUSG00000099807	0	0	0	0	0	0
ENSMUSG00000099805	0	0	0	0	0	0
ENSMUSG00000099800	0	0	0	0	0	0
ENSMUSG00000099801	0	0	0	0	0	0
ENSMUSG00000089258	0	0	0	0	0	0
ENSMUSG00000112281	0	0	0	0	0	0

ENSMUSG00000081357	0	0	0	0	0	0
ENSMUSG00000081355	0	0	0	0	0	0
ENSMUSG00000081354	0	0	1	2	0	0
ENSMUSG00000072714	1	1	2	1	0	1
ENSMUSG00000089252	0	0	0	0	0	0
ENSMUSG00000053414	25	37	31	41	37	33
ENSMUSG00000083677	6	4	2	3	3	2
ENSMUSG00000063374	0	0	0	0	0	0
ENSMUSG00000063376	0	0	0	0	0	0
ENSMUSG00000089256	0	0	0	2	0	0
ENSMUSG00000050164	0	0	2	0	0	0
ENSMUSG00000110651	0	0	0	0	0	0
ENSMUSG00000099432	0	0	0	0	0	0
ENSMUSG00000038932	3	0	0	7	0	0
ENSMUSG00000077273	0	0	0	0	0	0
ENSMUSG00000077272	0	0	0	0	0	0
ENSMUSG00000077275	0	0	0	0	0	0
ENSMUSG00000077277	0	0	0	0	0	0
ENSMUSG00000077279	0	0	0	0	0	0
ENSMUSG00000035878	147	119	162	125	168	168
ENSMUSG00000035875	6	3	8	3	16	0
ENSMUSG00000084805	0	0	0	0	0	0
ENSMUSG00000089830	0	0	0	0	0	0
ENSMUSG00000084807	2	0	6	0	0	3
ENSMUSG00000089836	0	0	0	0	0	0
ENSMUSG00000089834	0	0	0	0	0	0
ENSMUSG00000084803	46	77	88	42	48	89
ENSMUSG00000099430	0	0	0	0	0	0
ENSMUSG00000031558	1134	849	796	348	418	464
ENSMUSG00000031559	0	0	0	0	0	0
ENSMUSG00000090374	0	0	0	0	0	0
ENSMUSG00000031554	28	32	20	0	0	5
ENSMUSG00000031552	0	0	0	0	0	0
ENSMUSG00000031553	0	0	6	0	0	0
ENSMUSG00000099436	0	0	0	0	0	0
ENSMUSG00000112289	0	0	0	0	4	3
ENSMUSG00000110760	0	0	0	0	0	0
ENSMUSG00000007379	232	208	244	166	198	308
ENSMUSG00000082077	0	0	0	0	0	0
ENSMUSG00000082071	0	0	0	0	0	0
ENSMUSG00000088019	0	1	0	1	0	0
ENSMUSG00000082079	0	0	2	0	2	0
ENSMUSG00000085865	0	0	0	0	0	0
ENSMUSG00000085861	0	0	0	0	4	3
ENSMUSG00000085860	0	0	0	0	0	0
ENSMUSG00000085863	0	0	0	0	0	0
ENSMUSG00000033024	70	144	47	56	51	64
ENSMUSG00000085868	0	0	0	0	0	0
ENSMUSG00000033027	0	0	0	0	0	0
ENSMUSG00000103248	0	0	0	0	0	0
ENSMUSG00000103249	0	0	0	0	0	0
ENSMUSG00000103241	0	0	0	0	0	0
ENSMUSG00000103243	0	0	0	0	0	0
ENSMUSG00000087598	331	327	373	233	248	231
ENSMUSG00000087592	0	0	0	0	0	0
ENSMUSG00000087591	0	0	0	0	0	0
ENSMUSG00000090882	0	0	0	0	0	0
ENSMUSG00000040703	70	48	27	0	0	0

ENSMUSG00000040706	10	6	0	10	10	9
ENSMUSG00000100579	0	0	0	0	0	0
ENSMUSG00000100578	0	0	0	0	0	0
ENSMUSG00000100573	0	0	0	0	0	0
ENSMUSG00000100571	0	0	0	0	0	0
ENSMUSG00000100570	0	0	0	0	0	0
ENSMUSG00000100577	0	0	0	0	0	0
ENSMUSG00000100576	0	0	0	0	0	0
ENSMUSG00000100575	0	0	0	0	0	0
ENSMUSG00000100574	0	0	0	0	0	0
ENSMUSG00000032776	19	29	25	4	6	4
ENSMUSG00000032773	5	4	14	0	8	5
ENSMUSG00000084028	2	2	0	0	0	0
ENSMUSG00000084029	1	4	2	0	0	0
ENSMUSG00000084026	0	0	0	0	0	0
ENSMUSG00000084027	0	0	0	0	0	0
ENSMUSG00000084022	0	0	0	0	0	3
ENSMUSG00000084021	0	1	0	1	0	3
ENSMUSG00000095831	0	0	0	0	2	0
ENSMUSG00000095837	0	0	0	0	0	0
ENSMUSG00000095836	10	6	0	0	3	4
ENSMUSG00000095834	0	0	0	0	0	0
ENSMUSG00000075127	0	0	0	0	0	0
ENSMUSG00000102599	2	0	2	1	2	7
ENSMUSG00000102598	0	0	0	0	0	0
ENSMUSG00000102592	3	0	0	0	0	0
ENSMUSG00000102591	3	0	0	0	2	3
ENSMUSG00000102590	10	5	12	0	7	14
ENSMUSG00000102597	0	0	0	0	0	0
ENSMUSG00000075125	2	0	0	0	0	0
ENSMUSG00000102595	0	0	0	0	0	2
ENSMUSG00000099573	0	0	0	0	0	0
ENSMUSG00000102624	0	0	0	0	0	0
ENSMUSG00000102623	3	0	5	4	6	8
ENSMUSG00000102622	0	0	0	0	0	0
ENSMUSG00000102629	0	0	0	0	0	0
ENSMUSG00000102628	7	0	2	2	0	2
ENSMUSG00000020096	0	0	0	0	0	0
ENSMUSG00000020090	5	8	6	4	10	14
ENSMUSG00000083738	0	0	0	0	0	0
ENSMUSG00000065532	0	0	0	0	0	0
ENSMUSG00000058933	0	0	0	0	0	0
ENSMUSG00000065539	0	0	0	0	0	0
ENSMUSG00000105381	0	0	0	0	0	0
ENSMUSG00000099571	0	0	0	0	0	0
ENSMUSG00000062545	7	8	9	5	0	7
ENSMUSG00000062546	0	0	0	0	0	0
ENSMUSG00000062543	0	0	0	0	0	0
ENSMUSG00000062542	0	0	0	0	0	2
ENSMUSG00000028640	0	0	0	0	0	0
ENSMUSG00000028644	3	8	9	4	0	10
ENSMUSG00000094715	0	0	0	0	0	0
ENSMUSG00000113646	0	0	0	0	0	0
ENSMUSG00000024340	4	0	0	0	0	4
ENSMUSG00000024347	8	0	7	5	0	5
ENSMUSG00000029112	0	0	0	0	0	0
ENSMUSG00000088823	0	0	0	0	0	0
ENSMUSG00000088820	2	0	2	2	0	0

ENSMUSG00000113075	0	0	0	0	0	0
ENSMUSG00000038210	0	0	0	0	0	0
ENSMUSG00000113071	0	0	0	0	0	0
ENSMUSG00000088829	0	0	0	0	0	0
ENSMUSG00000038218	0	0	0	0	0	0
ENSMUSG00000113079	0	0	0	0	0	0
ENSMUSG00000063632	28	11	4	2	6	2
ENSMUSG00000104437	0	0	0	0	0	0
ENSMUSG00000111500	0	0	0	0	0	0
ENSMUSG00000111503	0	0	0	0	0	0
ENSMUSG00000111504	0	0	0	0	0	4
ENSMUSG00000111506	0	0	1	1	1	0
ENSMUSG00000111507	0	0	0	0	0	0
ENSMUSG00000109567	0	0	0	0	0	0
ENSMUSG00000111509	0	0	3	0	0	0
ENSMUSG00000109565	0	0	2	0	0	0
ENSMUSG00000109564	1083	1012	264	0	12	4
ENSMUSG00000109562	4	10	0	5	16	12
ENSMUSG00000109561	0	0	0	0	0	0
ENSMUSG00000109560	3	0	3	0	0	0
ENSMUSG00000106628	2	0	0	0	0	0
ENSMUSG00000106621	0	4	2	0	2	0
ENSMUSG00000050830	11	17	16	11	16	31
ENSMUSG00000104663	11	5	13	12	19	14
ENSMUSG00000104660	1	1	1	1	0	1
ENSMUSG00000104667	0	0	4	0	0	0
ENSMUSG00000104666	0	0	0	0	0	0
ENSMUSG00000104665	0	3	5	2	1	0
ENSMUSG00000104664	0	0	0	0	0	0
ENSMUSG00000059687	0	0	0	0	0	0
ENSMUSG00000038602	31	46	27	51	68	65
ENSMUSG00000088649	0	0	0	0	0	0
ENSMUSG00000097529	0	0	0	0	0	0
ENSMUSG00000083737	0	0	0	0	0	0
ENSMUSG00000097522	0	0	0	0	0	0
ENSMUSG00000097524	45	71	37	35	64	62
ENSMUSG00000112857	6	9	6	6	12	15
ENSMUSG00000112856	0	0	0	0	0	0
ENSMUSG00000112854	0	4	8	0	0	4
ENSMUSG00000112853	6	6	4	2	0	2
ENSMUSG00000112851	0	0	0	0	0	0
ENSMUSG00000113520	0	0	0	0	0	0
ENSMUSG00000048498	0	0	0	0	2	0
ENSMUSG00000097807	0	0	0	0	0	0
ENSMUSG00000110756	0	0	0	0	0	0
ENSMUSG00000110757	0	2	0	0	0	0
ENSMUSG00000110755	0	0	0	2	0	0
ENSMUSG00000110752	0	0	0	0	3	0
ENSMUSG00000095323	0	0	1	0	0	1
ENSMUSG00000104087	18	8	5	20	13	13
ENSMUSG00000104085	0	0	0	0	0	0
ENSMUSG00000104084	0	0	0	0	0	0
ENSMUSG00000104082	0	0	0	0	0	0
ENSMUSG00000090132	0	0	0	0	0	0
ENSMUSG00000104089	0	0	0	0	0	0
ENSMUSG00000104088	8	1	6	4	6	2
ENSMUSG00000015242	0	0	0	0	0	0
ENSMUSG00000113863	0	0	0	0	0	0

ENSMUSG00000082017	0	0	0	0	0	0
ENSMUSG00000058153	6	6	0	0	0	0
ENSMUSG00000058152	50	40	48	7	19	21
ENSMUSG00000060556	1	3	3	0	0	0
ENSMUSG00000080241	0	0	0	0	0	0
ENSMUSG00000107100	0	0	0	0	0	0
ENSMUSG00000026237	0	0	0	0	0	0
ENSMUSG00000027186	14	12	14	4	11	13
ENSMUSG00000045708	0	0	2	0	0	0
ENSMUSG00000045709	8	5	6	5	4	6
ENSMUSG00000113818	0	0	0	0	0	0
ENSMUSG00000111084	0	0	0	0	0	0
ENSMUSG00000095314	0	0	0	0	0	0
ENSMUSG00000095312	0	2	0	0	0	0
ENSMUSG00000111083	11	14	13	8	11	10
ENSMUSG00000113813	0	0	0	0	0	0
ENSMUSG00000113814	0	0	0	0	0	3
ENSMUSG00000113815	0	1	0	0	0	0
ENSMUSG00000095318	0	0	0	0	0	0
ENSMUSG00000095319	0	0	0	0	0	0
ENSMUSG00000081195	0	0	0	0	0	0
ENSMUSG00000081196	0	0	0	0	5	0
ENSMUSG00000081199	0	0	0	0	0	0
ENSMUSG00000110563	4	6	2	4	5	1
ENSMUSG00000052302	74	110	125	89	91	164
ENSMUSG00000052303	0	0	0	0	0	0
ENSMUSG00000054945	29	35	29	26	21	50
ENSMUSG00000042282	0	0	0	0	0	0
ENSMUSG00000113317	0	0	0	0	0	0
ENSMUSG00000094499	0	0	0	0	0	0
ENSMUSG00000094494	1	0	0	0	0	0
ENSMUSG00000086978	0	0	0	0	0	0
ENSMUSG00000099584	0	0	0	4	0	0
ENSMUSG00000093414	2	0	0	0	0	0
ENSMUSG00000086970	0	0	0	0	0	0
ENSMUSG00000086971	0	0	0	0	0	0
ENSMUSG00000086974	0	0	0	0	0	0
ENSMUSG00000094493	0	0	0	0	0	0
ENSMUSG00000093443	0	1	1	0	0	0
ENSMUSG00000048038	3	4	7	0	5	0
ENSMUSG00000090854	0	0	0	0	0	0
ENSMUSG00000048031	0	0	0	0	0	0
ENSMUSG00000057513	0	0	0	0	0	0
ENSMUSG00000028327	10	0	0	0	0	0
ENSMUSG00000063221	0	0	0	0	0	0
ENSMUSG00000100784	0	0	0	0	0	0
ENSMUSG00000086122	0	0	0	0	0	0
ENSMUSG00000112068	0	0	0	0	1	2
ENSMUSG00000112069	0	0	0	0	0	0
ENSMUSG00000112066	0	0	0	0	0	0
ENSMUSG00000094196	0	0	0	0	0	0
ENSMUSG00000094195	0	0	0	0	0	0
ENSMUSG00000112065	0	0	0	0	0	0
ENSMUSG00000112062	2	0	0	0	0	0
ENSMUSG00000094192	0	0	0	0	0	0
ENSMUSG00000094190	0	0	0	2	0	1
ENSMUSG00000100787	0	0	0	0	0	0
ENSMUSG00000100782	12	24	20	24	20	35

ENSMUSG00000072249	0	0	0	0	0	0
ENSMUSG00000099489	0	0	0	0	0	0
ENSMUSG00000099482	0	0	0	0	0	0
ENSMUSG00000099483	0	0	0	0	0	0
ENSMUSG00000099480	0	0	0	0	0	0
ENSMUSG00000099486	0	0	0	0	0	0
ENSMUSG00000099487	0	0	0	0	0	0
ENSMUSG00000099485	0	0	0	0	0	0
ENSMUSG00000016283	0	0	0	0	0	0
ENSMUSG00000003505	0	0	0	0	0	0
ENSMUSG00000051827	0	0	0	0	0	0
ENSMUSG00000043418	0	0	0	0	0	0
ENSMUSG00000043419	34	33	22	22	16	17
ENSMUSG00000086356	0	0	7	0	0	0
ENSMUSG00000036647	0	0	0	0	0	0
ENSMUSG00000043410	1	1	3	0	2	2
ENSMUSG00000086355	0	0	0	0	0	0
ENSMUSG00000086352	0	0	0	0	0	0
ENSMUSG00000086353	157	133	146	39	93	93
ENSMUSG00000065337	0	0	0	0	0	0
ENSMUSG00000076039	0	1	1	3	1	3
ENSMUSG00000072791	0	0	0	0	0	0
ENSMUSG00000080480	0	0	0	0	0	0
ENSMUSG00000016756	359	409	629	229	212	286
ENSMUSG00000088761	0	0	0	0	0	0
ENSMUSG00000101787	0	0	0	0	0	0
ENSMUSG00000101785	0	0	0	0	0	0
ENSMUSG00000109261	0	4	5	13	13	8
ENSMUSG00000049073	0	0	0	0	0	0
ENSMUSG00000101784	0	0	1	3	0	0
ENSMUSG00000101782	0	0	0	0	0	0
ENSMUSG00000087759	0	0	0	0	0	0
ENSMUSG00000101781	0	0	0	0	0	0
ENSMUSG00000087754	0	0	0	0	1	0
ENSMUSG00000082505	2	0	0	2	3	4
ENSMUSG00000087756	0	0	0	0	0	0
ENSMUSG00000082502	0	0	0	0	0	0
ENSMUSG00000082501	0	0	0	0	0	0
ENSMUSG00000082500	0	0	0	0	0	0
ENSMUSG00000040140	5	8	8	8	6	4
ENSMUSG00000040146	180	170	182	134	164	226
ENSMUSG00000083452	0	0	0	0	0	0
ENSMUSG00000083453	0	0	0	0	0	0
ENSMUSG00000083450	0	0	0	0	0	0
ENSMUSG00000083451	0	0	0	0	0	2
ENSMUSG00000083456	0	0	0	0	0	0
ENSMUSG00000031952	0	0	0	0	0	0
ENSMUSG00000031953	181	225	233	181	218	250
ENSMUSG00000031951	137	125	140	79	90	144
ENSMUSG00000031957	0	0	0	0	0	0
ENSMUSG00000070797	0	0	0	0	0	0
ENSMUSG00000070796	0	0	0	0	0	0
ENSMUSG00000070794	0	0	0	0	0	0
ENSMUSG00000070799	0	0	0	0	0	0
ENSMUSG00000070798	0	0	0	0	0	0
ENSMUSG00000111736	0	0	0	0	0	0
ENSMUSG00000071262	5	6	0	7	4	7
ENSMUSG00000071265	0	0	0	0	0	0

ENSMUSG00000084228	0	0	0	0	0	0
ENSMUSG00000106522	0	0	0	0	0	0
ENSMUSG00000084226	0	0	0	0	0	0
ENSMUSG00000084220	0	0	0	0	0	0
ENSMUSG00000084222	0	0	0	0	0	0
ENSMUSG00000106523	0	0	0	0	0	0
ENSMUSG00000065330	0	0	0	0	0	0
ENSMUSG00000094140	0	0	0	0	0	0
ENSMUSG00000083806	0	0	0	0	0	0
ENSMUSG00000075567	0	0	0	0	0	0
ENSMUSG00000075566	0	0	0	0	0	0
ENSMUSG00000093128	0	0	0	0	0	0
ENSMUSG00000023013	46	16	45	8	7	8
ENSMUSG00000094145	0	0	0	0	0	0
ENSMUSG00000094146	0	0	0	0	0	0
ENSMUSG00000051437	0	0	0	3	0	0
ENSMUSG00000078668	0	0	0	0	0	0
ENSMUSG00000088889	0	2	0	0	0	0
ENSMUSG00000086940	0	0	0	0	0	0
ENSMUSG00000065737	0	0	0	0	0	0
ENSMUSG00000065739	0	0	0	0	0	0
ENSMUSG00000096628	0	0	0	0	0	0
ENSMUSG00000070133	0	0	0	0	0	0
ENSMUSG00000099102	0	0	0	0	0	0
ENSMUSG00000070139	1	0	0	0	0	0
ENSMUSG00000032360	0	0	0	0	0	2
ENSMUSG00000101197	0	0	0	0	0	0
ENSMUSG00000032368	0	0	0	0	0	3
ENSMUSG00000106538	0	0	0	0	0	0
ENSMUSG00000062704	12	9	12	17	13	17
ENSMUSG00000065088	0	0	0	0	0	0
ENSMUSG00000100430	0	0	3	0	0	0
ENSMUSG00000090795	6	7	5	8	7	16
ENSMUSG00000091552	0	0	0	0	0	0
ENSMUSG00000078080	0	0	0	0	0	0
ENSMUSG00000101199	0	0	0	0	0	0
ENSMUSG00000032985	0	0	0	6	0	0
ENSMUSG00000032987	2	0	0	0	0	0
ENSMUSG00000103859	0	0	0	0	0	0
ENSMUSG00000103858	0	0	0	0	0	0
ENSMUSG00000103850	0	0	0	0	0	0
ENSMUSG00000103852	0	0	0	0	0	0
ENSMUSG00000103856	0	0	0	0	0	0
ENSMUSG00000097361	0	0	0	0	0	0
ENSMUSG00000019888	5	7	0	1	0	0
ENSMUSG00000019880	6	10	6	2	2	6
ENSMUSG00000092790	0	0	0	0	0	0
ENSMUSG00000030353	65	56	95	94	67	81
ENSMUSG00000097366	22	34	35	28	31	39
ENSMUSG00000030350	0	0	0	0	0	0
ENSMUSG00000089973	0	0	0	0	0	0
ENSMUSG00000095152	0	0	0	0	0	0
ENSMUSG00000031181	0	0	0	0	0	0
ENSMUSG00000077716	0	0	0	0	0	0
ENSMUSG00000019772	2	0	3	0	0	0
ENSMUSG00000019773	60	87	78	35	44	61
ENSMUSG00000019775	93	127	82	95	72	137
ENSMUSG00000077717	0	0	0	0	0	0

ENSMUSG00000071179	0	0	0	0	0	0
ENSMUSG00000082560	1	0	0	0	0	0
ENSMUSG00000082562	0	0	0	0	0	0
ENSMUSG00000077718	0	0	0	0	0	0
ENSMUSG00000110474	4	0	0	0	0	0
ENSMUSG00000076959	0	0	0	0	0	0
ENSMUSG00000076958	0	0	0	0	0	0
ENSMUSG00000076952	0	0	0	0	0	0
ENSMUSG00000076951	1	1	0	0	0	1
ENSMUSG00000076950	0	0	0	0	0	0
ENSMUSG00000076956	0	0	0	0	0	0
ENSMUSG00000111139	0	0	0	0	0	0
ENSMUSG00000108946	0	1	0	0	0	0
ENSMUSG00000108947	0	0	4	0	0	0
ENSMUSG00000108944	1	0	0	0	0	0
ENSMUSG00000108945	0	0	0	0	0	0
ENSMUSG00000108941	0	0	0	0	0	0
ENSMUSG00000109889	0	0	0	0	0	0
ENSMUSG00000091273	0	0	0	0	0	0
ENSMUSG00000105840	0	0	0	0	0	0
ENSMUSG00000102813	27	77	80	63	76	66
ENSMUSG00000102810	0	0	0	0	0	0
ENSMUSG00000105843	6	15	24	14	14	14
ENSMUSG00000067627	0	0	0	0	0	0
ENSMUSG00000043354	0	0	0	0	0	5
ENSMUSG00000043357	0	0	0	0	0	0
ENSMUSG00000102815	2	0	0	0	0	1
ENSMUSG00000105849	1	0	1	1	0	2
ENSMUSG00000102818	0	0	0	0	0	0
ENSMUSG00000095974	0	0	0	0	0	0
ENSMUSG00000101705	0	0	0	0	0	0
ENSMUSG00000101541	0	0	0	0	0	0
ENSMUSG00000028051	0	4	0	0	3	8
ENSMUSG00000101543	0	0	0	0	0	0
ENSMUSG00000101542	0	0	0	0	0	0
ENSMUSG00000101545	0	0	0	0	0	0
ENSMUSG00000101544	0	0	0	0	0	0
ENSMUSG00000101547	0	0	0	0	0	0
ENSMUSG00000101546	0	0	0	0	0	0
ENSMUSG00000101549	0	0	0	0	0	0
ENSMUSG00000095978	0	0	0	0	0	0
ENSMUSG00000095979	0	0	0	0	0	0
ENSMUSG00000087255	0	0	0	0	0	0
ENSMUSG00000058019	3	4	11	0	0	4
ENSMUSG00000089539	0	0	0	0	0	0
ENSMUSG00000088204	0	0	0	0	0	0
ENSMUSG00000088205	2	2	2	0	0	1
ENSMUSG00000088209	0	0	0	0	0	0
ENSMUSG00000058017	0	0	0	0	0	0
ENSMUSG00000058014	0	3	0	0	0	0
ENSMUSG00000005268	524	638	615	676	805	999
ENSMUSG00000091087	0	0	0	0	0	0
ENSMUSG00000089533	0	0	0	0	0	0
ENSMUSG00000021933	4	3	0	0	5	2
ENSMUSG00000033615	0	0	6	0	6	4
ENSMUSG00000045713	0	0	0	0	0	1
ENSMUSG00000083216	0	0	0	0	0	0
ENSMUSG00000110568	0	3	0	0	0	0

ENSMUSG00000103035	0	0	0	0	0	0
ENSMUSG00000061115	0	0	0	0	0	0
ENSMUSG00000089151	0	0	0	0	0	0
ENSMUSG00000089153	0	1	0	0	0	0
ENSMUSG00000089152	0	0	0	0	0	0
ENSMUSG00000089157	0	0	0	0	0	0
ENSMUSG00000089156	0	0	0	0	0	0
ENSMUSG00000089158	0	0	0	0	0	0
ENSMUSG00000106128	2	1	3	1	2	2
ENSMUSG00000106122	0	0	0	0	0	0
ENSMUSG00000106123	3	0	0	4	4	3
ENSMUSG00000106126	0	0	0	0	0	0
ENSMUSG00000106124	0	0	0	0	0	0
ENSMUSG00000046676	0	0	0	0	0	0
ENSMUSG00000097031	0	0	0	0	0	0
ENSMUSG00000112268	0	0	0	0	0	0
ENSMUSG00000094358	0	0	0	0	0	0
ENSMUSG00000112264	0	0	0	0	0	0
ENSMUSG00000112265	0	0	0	0	0	0
ENSMUSG00000094357	0	0	0	0	0	0
ENSMUSG00000112267	10	11	9	0	0	5
ENSMUSG00000094351	0	0	0	0	0	0
ENSMUSG00000094350	12	22	18	14	5	10
ENSMUSG00000094353	0	0	0	0	0	0
ENSMUSG00000094352	1	1	0	0	0	0
ENSMUSG00000029830	54	44	81	62	35	51
ENSMUSG00000029831	0	0	0	0	0	0
ENSMUSG00000029837	3	0	0	0	0	0
ENSMUSG00000052854	39	54	14	41	25	50
ENSMUSG00000020686	7	6	4	10	3	15
ENSMUSG00000035615	0	4	0	0	0	3
ENSMUSG00000114950	2	0	0	0	0	0
ENSMUSG00000114954	0	0	0	0	0	0
ENSMUSG00000114955	0	0	0	1	0	1
ENSMUSG00000114956	0	0	0	0	0	0
ENSMUSG00000114957	0	0	0	0	0	0
ENSMUSG00000114958	0	0	0	0	0	0
ENSMUSG00000114959	0	0	0	0	0	0
ENSMUSG00000105491	0	0	0	0	0	0
ENSMUSG00000052928	415	378	492	196	259	285
ENSMUSG00000106788	0	0	0	0	0	0
ENSMUSG00000106786	0	0	0	0	0	0
ENSMUSG00000106780	0	0	0	0	0	0
ENSMUSG00000088784	2	0	0	0	2	1
ENSMUSG00000088782	0	0	0	0	0	0
ENSMUSG00000105479	0	0	0	0	0	0
ENSMUSG00000105478	8	12	23	18	16	17
ENSMUSG00000021483	93	90	119	97	65	109
ENSMUSG00000086364	0	0	0	0	0	0
ENSMUSG00000104961	0	1	0	0	0	0
ENSMUSG00000109152	0	0	0	0	0	0
ENSMUSG00000109153	0	0	0	0	0	0
ENSMUSG00000105473	0	0	0	0	0	0
ENSMUSG00000109151	0	0	0	0	0	0
ENSMUSG00000105475	0	0	0	0	0	0
ENSMUSG00000109155	0	0	0	0	0	0
ENSMUSG00000105783	1	0	0	0	0	0
ENSMUSG00000105781	0	0	0	0	0	0

ENSMUSG00000105787	0	0	0	2	0	0
ENSMUSG00000105786	0	0	0	0	0	0
ENSMUSG00000012187	0	0	0	0	0	4
ENSMUSG00000029306	0	0	0	0	0	0
ENSMUSG00000029307	0	0	5	0	0	4
ENSMUSG00000099300	0	0	0	0	0	0
ENSMUSG00000104965	13	21	14	12	17	22
ENSMUSG00000106878	2	3	0	0	0	5
ENSMUSG00000106879	0	0	0	0	0	0
ENSMUSG00000066239	0	0	0	0	0	0
ENSMUSG00000106872	0	0	0	2	2	0
ENSMUSG00000106873	4	3	2	3	3	0
ENSMUSG00000106875	0	0	0	0	0	0
ENSMUSG00000106876	0	0	0	0	0	2
ENSMUSG00000097196	0	0	0	0	0	0
ENSMUSG00000113604	0	0	0	0	0	0
ENSMUSG00000113607	0	0	0	0	0	0
ENSMUSG00000038026	0	7	6	0	2	5
ENSMUSG00000058588	0	2	0	2	0	0
ENSMUSG00000097190	0	0	0	0	0	0
ENSMUSG00000113602	0	0	0	0	0	0
ENSMUSG00000113609	0	0	0	0	0	0
ENSMUSG00000113608	1	0	0	0	0	0
ENSMUSG00000097199	0	0	0	0	0	0
ENSMUSG00000080990	0	0	0	2	0	0
ENSMUSG00000105158	0	0	0	0	0	0
ENSMUSG00000076041	0	0	0	0	0	0
ENSMUSG00000105495	24	31	32	29	37	36
ENSMUSG00000114374	0	0	0	0	0	0
ENSMUSG00000096244	0	0	0	0	0	0
ENSMUSG00000096247	0	0	0	0	0	0
ENSMUSG00000114377	0	0	0	0	0	0
ENSMUSG00000057981	0	0	0	0	0	0
ENSMUSG00000096240	0	0	0	0	0	0
ENSMUSG00000114372	0	0	0	3	0	2
ENSMUSG00000096249	0	0	0	0	0	0
ENSMUSG00000114379	0	0	0	0	0	0
ENSMUSG00000104788	0	0	0	0	0	0
ENSMUSG00000108208	0	0	0	0	0	0
ENSMUSG00000108201	2	0	0	0	0	0
ENSMUSG00000104781	15	8	22	9	11	11
ENSMUSG00000108206	0	0	0	0	0	0
ENSMUSG00000076049	0	0	0	0	0	0
ENSMUSG00000059169	0	0	0	0	0	0
ENSMUSG00000093998	1	0	0	0	0	0
ENSMUSG00000093993	0	0	0	0	0	0
ENSMUSG00000093990	2	1	2	1	1	1
ENSMUSG00000026686	0	0	0	0	0	0
ENSMUSG00000093997	0	0	0	0	1	0
ENSMUSG00000093994	0	0	0	0	0	0
ENSMUSG00000093995	0	0	0	0	0	0
ENSMUSG00000102142	53	20	40	22	29	33
ENSMUSG00000025480	2	0	0	0	0	2
ENSMUSG00000104433	2	0	0	0	0	0
ENSMUSG00000104430	0	0	0	0	0	0
ENSMUSG00000104431	0	0	0	0	0	0
ENSMUSG00000102146	0	0	0	0	0	0
ENSMUSG00000104438	0	0	0	0	0	0

ENSMUSG00000091734	0	0	0	0	0	0
ENSMUSG00000091731	0	0	0	0	0	0
ENSMUSG00000091733	0	0	0	0	0	0
ENSMUSG00000083926	0	0	0	0	0	0
ENSMUSG00000083927	0	0	0	0	0	0
ENSMUSG00000083925	0	0	0	0	0	0
ENSMUSG00000083923	0	0	0	0	0	0
ENSMUSG00000083920	0	0	0	0	0	0
ENSMUSG00000083921	0	0	0	0	0	0
ENSMUSG00000083928	0	0	0	0	0	0
ENSMUSG00000088181	1	0	0	0	0	0
ENSMUSG00000088180	0	0	0	0	0	0
ENSMUSG00000061815	20	25	34	21	13	22
ENSMUSG00000046248	0	2	0	0	0	4
ENSMUSG00000088187	0	0	0	0	0	0
ENSMUSG00000100242	0	0	0	0	0	0
ENSMUSG00000046240	4	0	4	8	0	0
ENSMUSG00000046242	6	0	0	7	2	6
ENSMUSG00000095298	0	0	5	0	0	0
ENSMUSG00000095293	0	0	0	0	0	0
ENSMUSG00000095292	0	0	0	0	0	0
ENSMUSG00000095290	1	0	3	1	1	3
ENSMUSG00000095296	0	0	0	0	0	0
ENSMUSG00000095295	10	8	15	0	6	9
ENSMUSG00000074946	0	5	0	0	0	0
ENSMUSG00000074947	0	0	0	0	0	0
ENSMUSG00000074945	0	0	0	0	0	0
ENSMUSG00000045575	0	0	0	0	0	0
ENSMUSG00000079842	0	0	0	0	0	0
ENSMUSG00000079845	0	21	40	9	3	50
ENSMUSG00000067262	0	0	0	0	0	0
ENSMUSG00000100950	2	0	0	0	0	0
ENSMUSG00000006056	0	0	0	0	0	0
ENSMUSG00000080789	1	0	4	0	2	0
ENSMUSG00000080788	0	0	1	1	0	0
ENSMUSG00000080785	0	0	0	0	0	2
ENSMUSG00000080782	0	0	0	0	3	0
ENSMUSG00000080781	0	0	0	0	0	0
ENSMUSG00000086835	0	0	0	0	0	0
ENSMUSG00000086833	0	0	0	0	0	0
ENSMUSG00000086831	0	0	0	0	0	0
ENSMUSG00000086830	0	0	0	0	0	0
ENSMUSG00000111294	0	0	0	0	0	0
ENSMUSG00000048919	0	2	2	0	0	0
ENSMUSG00000111290	0	0	0	0	0	0
ENSMUSG00000095122	0	0	0	0	0	0
ENSMUSG00000098402	0	0	0	0	0	0
ENSMUSG00000111298	0	0	0	0	0	0
ENSMUSG00000111299	0	0	0	0	0	0
ENSMUSG00000098526	1	1	1	1	1	1
ENSMUSG00000098522	0	0	0	0	0	0
ENSMUSG00000098521	0	0	0	0	1	1
ENSMUSG00000047592	67	53	85	16	16	28
ENSMUSG00000047591	0	1	0	5	2	4
ENSMUSG00000098528	0	2	2	0	4	2
ENSMUSG00000047594	1	0	0	0	0	1
ENSMUSG00000053846	2	0	0	2	4	6
ENSMUSG00000047626	0	2	0	0	0	0

ENSMUSG00000094227	0	0	0	1	0	0
ENSMUSG00000086789	0	0	0	0	0	0
ENSMUSG00000100955	0	0	0	0	0	0
ENSMUSG00000094656	0	0	0	0	0	0
ENSMUSG00000093601	0	0	0	0	0	0
ENSMUSG00000094651	51	43	68	26	0	37
ENSMUSG00000086783	0	0	0	0	0	0
ENSMUSG00000094658	0	0	0	0	0	0
ENSMUSG00000094659	0	0	0	0	0	0
ENSMUSG00000081784	0	0	0	0	0	0
ENSMUSG00000081786	0	0	0	0	0	0
ENSMUSG00000081781	0	0	0	0	0	0
ENSMUSG00000006948	0	0	0	0	0	0
ENSMUSG00000022738	0	0	0	0	0	0
ENSMUSG00000055446	2	0	0	0	0	0
ENSMUSG00000109828	16	12	8	6	12	19
ENSMUSG00000105658	0	0	0	0	0	0
ENSMUSG00000100956	0	0	0	0	0	0
ENSMUSG00000090955	10	3	8	2	5	4
ENSMUSG00000081438	0	0	0	0	0	0
ENSMUSG00000081439	0	0	0	0	0	0
ENSMUSG00000081436	2	2	2	3	2	1
ENSMUSG00000081437	0	5	2	0	0	0
ENSMUSG00000081435	0	0	0	0	0	0
ENSMUSG00000081432	0	0	0	0	0	3
ENSMUSG00000081433	0	0	0	0	0	0
ENSMUSG00000092551	0	0	0	0	0	0
ENSMUSG00000077559	0	0	1	0	2	0
ENSMUSG00000092552	0	0	0	0	0	0
ENSMUSG00000092555	0	0	0	0	0	0
ENSMUSG00000099729	0	0	0	0	0	0
ENSMUSG00000092556	4	4	6	2	0	5
ENSMUSG00000099727	0	0	0	0	0	0
ENSMUSG00000099726	0	0	0	0	0	0
ENSMUSG00000099725	0	0	0	0	0	0
ENSMUSG00000099724	0	0	0	0	0	0
ENSMUSG00000077554	0	0	0	0	0	0
ENSMUSG00000099722	26	19	7	5	5	21
ENSMUSG00000099721	0	0	0	0	0	0
ENSMUSG00000049796	5	7	0	16	13	5
ENSMUSG00000039081	82	43	55	69	31	21
ENSMUSG00000082200	0	0	0	0	0	0
ENSMUSG00000082202	0	0	0	0	0	0
ENSMUSG00000030075	6	1	2	5	5	7
ENSMUSG00000082204	0	0	0	0	0	0
ENSMUSG00000082205	0	0	0	0	0	3
ENSMUSG00000082208	6	3	5	5	3	10
ENSMUSG00000048261	3	1	1	0	8	2
ENSMUSG00000105756	0	0	0	0	0	0
ENSMUSG00000108507	0	0	0	2	2	6
ENSMUSG00000032502	17	6	20	9	12	0
ENSMUSG00000032503	34	76	56	46	103	75
ENSMUSG00000089446	0	0	0	2	0	0
ENSMUSG00000108502	0	2	0	1	0	0
ENSMUSG00000040197	0	0	0	0	0	0
ENSMUSG00000004540	0	0	6	0	0	0
ENSMUSG00000004542	0	3	2	0	0	0
ENSMUSG00000088454	0	0	0	0	0	0

ENSMUSG00000041660	20	6	14	0	4	4
ENSMUSG00000087495	0	0	0	0	0	0
ENSMUSG00000041669	0	0	0	0	0	0
ENSMUSG00000100856	0	0	0	0	0	0
ENSMUSG00000083556	0	0	0	0	0	0
ENSMUSG00000100857	0	2	0	0	0	0
ENSMUSG00000083550	0	0	0	0	0	0
ENSMUSG00000082526	0	0	0	0	0	0
ENSMUSG00000049133	0	0	0	0	0	0
ENSMUSG00000098899	0	0	0	0	0	0
ENSMUSG00000098894	0	0	2	0	0	0
ENSMUSG00000098895	11	11	0	0	0	0
ENSMUSG00000098891	4	4	1	7	5	2
ENSMUSG00000068950	1	2	0	0	0	0
ENSMUSG00000113739	3	0	0	12	4	6
ENSMUSG00000068957	0	0	3	0	0	0
ENSMUSG00000068959	341	320	322	250	309	341
ENSMUSG00000031853	100	121	92	145	147	186
ENSMUSG00000049041	0	0	0	0	0	0
ENSMUSG00000075158	0	0	0	0	0	0
ENSMUSG00000075159	0	0	0	0	0	0
ENSMUSG00000075150	0	0	0	0	0	0
ENSMUSG00000075151	0	0	2	0	0	0
ENSMUSG00000075156	5	3	0	0	2	2
ENSMUSG00000113733	0	0	0	0	0	0
ENSMUSG00000075155	0	0	0	0	1	0
ENSMUSG00000022619	0	0	0	0	0	0
ENSMUSG00000067482	0	0	0	0	0	0
ENSMUSG00000064659	0	0	0	0	0	0
ENSMUSG00000064658	2	0	0	0	1	0
ENSMUSG00000094071	0	0	0	0	0	0
ENSMUSG00000107253	10	12	2	13	10	6
ENSMUSG00000107252	8	2	9	3	11	6
ENSMUSG00000107251	18	20	14	16	18	14
ENSMUSG00000107256	0	2	0	1	0	3
ENSMUSG00000107254	0	0	0	0	1	0
ENSMUSG00000107259	0	0	0	5	0	2
ENSMUSG00000107258	0	0	0	0	0	0
ENSMUSG00000047102	0	0	0	0	0	0
ENSMUSG00000101949	0	1	0	0	0	0
ENSMUSG00000101948	0	0	0	1	0	0
ENSMUSG00000068373	14	12	7	7	16	13
ENSMUSG00000101944	0	0	0	0	0	0
ENSMUSG00000101947	0	0	0	0	0	0
ENSMUSG00000101940	0	0	0	6	0	12
ENSMUSG00000101943	0	0	0	0	0	0
ENSMUSG00000078780	0	8	4	0	0	3
ENSMUSG00000078783	0	5	3	0	0	0
ENSMUSG00000074206	3	22	14	5	7	10
ENSMUSG00000026535	4	9	20	4	12	11
ENSMUSG00000001023	0	0	0	0	0	0
ENSMUSG00000103060	2	3	3	2	4	0
ENSMUSG00000103120	0	0	0	0	0	0
ENSMUSG00000081847	0	0	0	0	0	0
ENSMUSG00000081844	0	0	0	0	0	0
ENSMUSG00000081843	0	0	0	0	0	0
ENSMUSG00000081842	2	0	0	1	0	0
ENSMUSG00000081841	0	0	0	0	1	0

ENSMUSG00000065291	0	0	0	0	0	0
ENSMUSG00000081849	3	0	3	4	0	3
ENSMUSG00000065293	0	0	0	0	0	0
ENSMUSG00000111926	0	0	0	0	0	2
ENSMUSG00000032087	0	0	3	0	0	0
ENSMUSG00000032081	0	0	0	0	0	0
ENSMUSG00000032083	0	0	0	4	0	0
ENSMUSG00000089548	0	0	0	0	0	0
ENSMUSG00000089543	0	0	0	0	0	0
ENSMUSG00000089541	0	0	0	0	0	0
ENSMUSG00000089546	0	0	0	0	0	0
ENSMUSG00000089544	0	0	0	1	1	0
ENSMUSG00000083663	0	0	0	0	0	0
ENSMUSG00000063867	0	0	0	1	0	0
ENSMUSG00000102373	0	0	0	0	0	0
ENSMUSG00000102372	0	0	0	0	0	2
ENSMUSG00000102371	0	0	0	0	0	3
ENSMUSG00000102379	2	0	0	0	0	0
ENSMUSG00000102378	0	0	0	0	0	0
ENSMUSG00000066652	0	0	0	0	0	0
ENSMUSG00000077962	0	3	1	1	1	1
ENSMUSG00000073267	0	0	0	0	0	0
ENSMUSG00000038624	25	60	90	32	46	75
ENSMUSG00000030178	0	6	2	0	0	2
ENSMUSG00000107419	0	0	0	0	0	0
ENSMUSG00000093114	0	0	0	0	0	0
ENSMUSG00000035946	0	0	0	0	0	0
ENSMUSG00000096003	7	0	15	0	5	0
ENSMUSG00000096002	0	0	0	0	0	0
ENSMUSG00000096001	5	0	4	0	0	0
ENSMUSG00000096000	0	0	0	0	0	0
ENSMUSG00000096005	0	0	0	0	0	0
ENSMUSG00000096009	0	0	0	0	0	0
ENSMUSG00000096008	0	0	0	0	0	0
ENSMUSG00000083662	0	0	0	0	0	0
ENSMUSG00000021010	14	14	19	0	0	4
ENSMUSG00000084254	0	0	0	0	0	0
ENSMUSG00000102995	0	0	0	0	0	2
ENSMUSG00000083284	3	3	5	7	3	5
ENSMUSG00000102993	0	0	0	0	0	0
ENSMUSG00000102991	0	0	0	0	0	0
ENSMUSG00000057615	0	4	3	4	3	5
ENSMUSG00000102999	0	0	0	0	0	0
ENSMUSG00000102998	0	0	0	0	0	0
ENSMUSG00000066747	0	2	0	0	0	0
ENSMUSG00000066749	2	3	0	0	0	0
ENSMUSG00000101208	0	0	0	0	0	0
ENSMUSG00000104230	0	0	0	0	0	0
ENSMUSG00000104232	0	0	0	0	0	0
ENSMUSG00000104233	0	0	0	0	0	0
ENSMUSG00000104236	0	0	0	0	0	0
ENSMUSG00000073809	1	3	0	2	2	2
ENSMUSG00000073807	2	1	1	1	3	2
ENSMUSG00000111352	0	0	0	0	0	0
ENSMUSG00000088386	0	0	0	0	0	0
ENSMUSG00000111362	0	0	0	0	0	0
ENSMUSG00000101201	0	0	0	0	0	0
ENSMUSG00000020945	0	0	0	0	0	0

ENSMUSG00000101205	0	0	0	0	0	0
ENSMUSG00000060742	0	0	0	0	0	0
ENSMUSG00000111363	0	0	0	0	0	0
ENSMUSG00000056564	0	0	0	0	0	0
ENSMUSG00000109809	0	0	0	0	0	0
ENSMUSG00000010797	15	3	29	0	8	0
ENSMUSG00000010796	0	0	0	6	0	0
ENSMUSG00000100263	0	0	0	0	0	0
ENSMUSG00000100261	0	0	0	0	0	0
ENSMUSG00000100267	0	0	0	0	0	0
ENSMUSG00000095722	0	0	0	0	0	0
ENSMUSG00000095721	0	0	0	0	0	0
ENSMUSG00000095720	0	0	0	0	0	0
ENSMUSG00000095727	1	0	0	0	0	0
ENSMUSG00000095725	0	0	0	0	0	0
ENSMUSG00000095724	0	0	0	0	0	0
ENSMUSG00000095729	0	0	0	0	0	0
ENSMUSG00000095728	0	0	0	0	0	0
ENSMUSG00000088433	0	0	0	0	0	0
ENSMUSG00000088432	0	0	0	0	0	0
ENSMUSG00000088435	0	0	0	0	0	0
ENSMUSG00000088436	0	2	0	0	1	0
ENSMUSG00000088439	0	0	0	0	0	0
ENSMUSG00000088438	0	0	0	0	0	0
ENSMUSG00000094093	0	0	0	0	0	0
ENSMUSG00000094095	0	1	0	0	0	0
ENSMUSG00000094098	0	0	0	0	2	0
ENSMUSG00000094099	0	0	0	0	0	0
ENSMUSG00000111908	1	2	1	1	2	5
ENSMUSG00000111906	0	0	0	0	0	0
ENSMUSG00000111900	0	0	0	0	0	0
ENSMUSG00000111901	4	0	0	0	4	0
ENSMUSG00000111902	0	0	0	0	0	0
ENSMUSG00000111903	0	0	0	0	0	4
ENSMUSG00000082324	0	0	0	0	0	0
ENSMUSG00000109801	0	0	0	0	0	0
ENSMUSG00000082322	2	2	2	0	2	6
ENSMUSG00000093379	2	0	0	0	0	0
ENSMUSG00000087133	0	0	0	0	0	0
ENSMUSG00000093373	0	0	0	0	0	0
ENSMUSG00000093376	0	0	0	0	0	0
ENSMUSG00000036574	0	0	0	2	0	0
ENSMUSG00000091788	0	0	0	0	0	0
ENSMUSG00000050963	0	0	0	0	0	0
ENSMUSG00000028777	0	34	11	5	2	8
ENSMUSG00000099448	4	2	1	4	0	0
ENSMUSG00000109803	4	0	0	0	0	0
ENSMUSG00000099449	0	0	0	0	0	0
ENSMUSG00000028778	0	0	0	0	0	5
ENSMUSG00000024670	0	7	6	0	0	0
ENSMUSG00000024673	22	43	42	18	13	19
ENSMUSG00000018862	0	0	0	0	0	0
ENSMUSG00000018865	6	6	0	0	0	0
ENSMUSG00000097603	0	2	1	0	6	10
ENSMUSG00000111246	21	26	11	24	28	21
ENSMUSG00000020164	0	0	0	0	0	0
ENSMUSG00000020168	0	0	0	0	0	0
ENSMUSG00000099441	0	0	0	0	0	0

ENSMUSG00000092029	3	4	1	3	1	5
ENSMUSG00000097018	0	0	0	0	0	0
ENSMUSG00000092021	0	22	7	11	6	0
ENSMUSG00000097012	0	4	0	3	0	0
ENSMUSG00000097010	2	0	0	0	0	0
ENSMUSG00000097017	5	0	0	0	0	0
ENSMUSG00000097016	0	0	4	0	0	0
ENSMUSG00000092027	0	0	0	0	0	0
ENSMUSG00000097014	0	2	0	0	0	2
ENSMUSG00000004707	67	95	142	21	28	18
ENSMUSG00000112763	0	0	0	0	0	0
ENSMUSG00000112762	0	0	0	0	0	0
ENSMUSG00000109057	0	0	0	0	0	2
ENSMUSG00000109056	6	0	13	8	0	0
ENSMUSG00000004709	28	32	62	16	4	15
ENSMUSG00000109053	0	0	0	0	0	0
ENSMUSG00000112768	0	0	0	0	0	0
ENSMUSG00000055138	0	0	0	0	0	0
ENSMUSG00000046856	23	4	12	0	9	14
ENSMUSG00000042459	0	0	0	0	0	0
ENSMUSG00000110699	5	0	6	6	7	7
ENSMUSG00000110698	0	0	0	0	0	0
ENSMUSG00000110695	0	0	0	0	0	0
ENSMUSG00000110693	0	0	0	0	0	0
ENSMUSG00000110691	0	0	0	3	0	0
ENSMUSG00000113415	0	0	0	0	0	0
ENSMUSG00000113417	0	0	0	0	0	0
ENSMUSG00000113410	0	0	0	0	0	0
ENSMUSG00000113411	2	1	0	0	0	0
ENSMUSG00000113412	2	2	3	3	2	3
ENSMUSG00000113413	0	0	0	0	0	2
ENSMUSG00000113418	0	0	0	0	0	0
ENSMUSG00000113419	0	1	0	1	1	1
ENSMUSG00000096383	0	1	1	1	0	0
ENSMUSG00000084448	0	0	0	0	0	0
ENSMUSG00000082958	1	0	0	0	0	0
ENSMUSG00000082955	0	0	0	0	0	0
ENSMUSG00000082956	0	0	0	0	0	4
ENSMUSG00000082957	0	0	0	0	1	0
ENSMUSG00000082950	2	0	0	0	0	0
ENSMUSG00000082952	2	3	14	0	0	0
ENSMUSG00000108018	0	0	0	0	0	0
ENSMUSG00000070345	0	0	3	2	0	8
ENSMUSG00000108014	0	0	2	0	0	1
ENSMUSG00000108015	12	16	5	12	13	19
ENSMUSG00000050410	106	130	118	69	69	131
ENSMUSG00000108017	0	0	0	0	0	0
ENSMUSG00000109963	0	0	0	0	0	0
ENSMUSG00000037014	36	43	30	49	45	54
ENSMUSG00000084441	0	0	0	0	0	0
ENSMUSG00000104165	9	1	2	0	9	6
ENSMUSG00000083490	0	0	0	0	0	0
ENSMUSG00000084443	0	0	0	0	0	0
ENSMUSG00000104577	2	0	0	0	0	0
ENSMUSG00000092043	0	0	0	0	0	0
ENSMUSG00000047810	107	133	222	66	48	78
ENSMUSG00000104573	0	0	0	0	0	0
ENSMUSG00000104572	0	0	0	0	0	0

ENSMUSG00000104570	0	0	0	0	0	0
ENSMUSG00000104578	0	0	0	0	0	0
ENSMUSG00000113787	0	0	0	0	0	0
ENSMUSG00000111129	0	3	3	0	4	0
ENSMUSG00000026527	46	46	6	48	74	83
ENSMUSG00000111127	0	0	0	0	0	0
ENSMUSG00000026523	0	4	2	0	0	0
ENSMUSG00000111120	0	0	0	0	0	0
ENSMUSG00000111121	0	0	0	0	0	0
ENSMUSG00000100152	0	0	0	0	0	0
ENSMUSG00000113782	0	0	0	0	0	3
ENSMUSG00000100155	0	0	0	0	0	0
ENSMUSG00000044542	2	0	0	0	0	0
ENSMUSG00000113788	5	14	0	12	6	10
ENSMUSG00000001622	0	0	0	0	0	0
ENSMUSG00000109966	0	0	0	0	0	0
ENSMUSG00000044243	0	0	0	0	0	2
ENSMUSG00000040621	54	57	48	64	45	36
ENSMUSG00000027699	95	77	75	16	57	71
ENSMUSG00000083668	0	0	2	0	1	0
ENSMUSG00000112787	0	0	0	0	0	0
ENSMUSG00000109964	0	0	0	0	0	0
ENSMUSG00000109070	0	0	0	0	0	0
ENSMUSG00000090655	0	0	0	0	0	0
ENSMUSG00000090657	0	0	0	0	0	0
ENSMUSG00000098289	0	0	0	0	1	0
ENSMUSG00000108611	0	0	0	0	0	0
ENSMUSG00000090659	61	74	77	35	50	64
ENSMUSG00000098287	0	0	0	0	0	0
ENSMUSG00000103157	0	0	0	0	0	0
ENSMUSG00000092850	1	3	0	2	0	0
ENSMUSG00000092852	1	1	1	1	1	1
ENSMUSG00000092853	0	0	0	0	0	0
ENSMUSG00000027344	2	0	4	0	0	0
ENSMUSG00000092856	0	0	0	0	0	0
ENSMUSG00000027347	40	44	79	10	14	22
ENSMUSG00000099886	0	5	0	0	0	0
ENSMUSG00000099884	0	0	0	0	0	0
ENSMUSG00000099882	0	0	0	0	0	0
ENSMUSG00000099883	0	0	0	0	0	0
ENSMUSG00000099880	0	0	0	0	0	0
ENSMUSG00000082720	0	0	0	0	0	0
ENSMUSG00000099888	0	0	0	0	0	0
ENSMUSG00000046834	0	0	0	0	0	0
ENSMUSG00000053499	0	0	0	0	0	0
ENSMUSG00000073069	0	0	0	0	0	0
ENSMUSG00000094305	0	0	0	0	0	0
ENSMUSG00000053490	0	0	0	0	0	0
ENSMUSG00000080624	1	0	0	0	0	0
ENSMUSG00000080625	0	0	0	0	0	0
ENSMUSG00000080627	0	0	0	0	0	0
ENSMUSG00000082726	0	3	0	0	0	0
ENSMUSG00000096869	0	0	0	0	0	0
ENSMUSG00000086668	0	0	0	1	0	0
ENSMUSG00000078906	19	16	10	7	23	16
ENSMUSG00000078907	3	0	8	0	5	4
ENSMUSG00000086665	21	10	9	8	7	17
ENSMUSG00000086662	0	0	0	0	0	0

ENSMUSG00000086660	0	0	0	0	0	0
ENSMUSG00000083141	0	0	0	0	0	0
ENSMUSG00000098757	0	0	0	0	0	0
ENSMUSG00000083143	0	0	0	0	0	0
ENSMUSG00000098758	0	0	0	0	0	0
ENSMUSG00000031262	42	23	43	8	13	24
ENSMUSG00000109976	0	0	0	0	0	0
ENSMUSG00000083147	0	0	0	0	0	0
ENSMUSG00000093031	6	0	2	1	3	0
ENSMUSG00000093033	0	0	0	0	0	0
ENSMUSG00000025051	0	0	0	0	0	0
ENSMUSG00000104575	0	0	0	0	0	0
ENSMUSG00000093036	0	0	0	0	0	0
ENSMUSG00000093039	0	0	0	0	0	0
ENSMUSG00000106442	0	0	0	0	0	0
ENSMUSG00000072082	45	35	56	19	20	35
ENSMUSG00000097023	0	0	0	0	0	0
ENSMUSG00000037953	0	0	0	0	0	0
ENSMUSG00000113439	0	0	0	0	0	0
ENSMUSG00000112194	0	0	0	0	0	0
ENSMUSG00000043787	0	0	0	0	0	0
ENSMUSG00000113435	0	0	0	0	0	0
ENSMUSG00000113433	0	0	0	0	0	0
ENSMUSG00000040478	0	0	0	0	0	0
ENSMUSG00000040471	0	0	0	0	0	0
ENSMUSG00000073937	0	0	0	0	0	0
ENSMUSG00000040473	103	80	89	39	37	67
ENSMUSG00000101331	0	0	0	0	0	0
ENSMUSG00000073932	0	0	0	0	0	0
ENSMUSG00000073931	0	0	0	1	0	0
ENSMUSG00000087796	0	0	0	0	0	0
ENSMUSG00000086552	0	0	3	0	0	0
ENSMUSG00000087798	0	0	0	0	0	0
ENSMUSG00000043870	7	3	3	3	1	4
ENSMUSG00000043873	1	12	18	7	2	9
ENSMUSG00000030607	152	23	65	5	2	6
ENSMUSG00000030606	16	11	16	7	15	16
ENSMUSG00000087049	8	15	12	11	10	13
ENSMUSG00000087044	0	0	0	0	0	0
ENSMUSG00000099574	0	0	0	0	0	0
ENSMUSG00000097024	0	0	0	1	0	0
ENSMUSG00000037375	222	240	212	219	239	290
ENSMUSG00000089323	0	0	0	0	0	0
ENSMUSG00000089320	1	0	0	0	0	0
ENSMUSG00000089326	0	0	0	0	0	0
ENSMUSG00000089327	0	0	0	0	0	0
ENSMUSG00000083740	0	0	0	0	0	0
ENSMUSG00000083741	0	0	0	0	0	0
ENSMUSG00000100069	0	0	0	0	0	0
ENSMUSG00000114789	6	0	0	2	8	6
ENSMUSG00000100061	0	0	0	0	0	0
ENSMUSG00000100060	0	3	10	4	2	2
ENSMUSG00000100063	0	0	0	0	0	0
ENSMUSG00000100062	0	0	0	0	0	0
ENSMUSG00000100065	1	0	0	0	0	0
ENSMUSG00000100064	0	0	0	0	0	0
ENSMUSG00000100067	0	0	0	0	0	0
ENSMUSG00000100066	0	0	0	1	0	0

ENSMUSG00000109380	0	0	0	0	0	0
ENSMUSG00000070621	0	0	0	0	0	0
ENSMUSG00000109384	0	0	0	2	0	0
ENSMUSG00000064194	0	0	0	0	0	0
ENSMUSG00000085796	0	0	0	0	0	0
ENSMUSG00000102513	0	0	0	0	0	0
ENSMUSG00000102512	0	0	0	0	0	0
ENSMUSG00000102511	0	0	0	0	0	0
ENSMUSG00000102510	0	0	0	0	0	0
ENSMUSG00000102516	140	168	157	65	72	127
ENSMUSG00000102515	0	0	0	0	0	0
ENSMUSG00000102514	0	0	0	0	0	0
ENSMUSG00000107590	0	0	2	0	0	0
ENSMUSG00000107592	0	0	0	0	0	0
ENSMUSG00000080366	1	3	1	1	2	1
ENSMUSG00000107594	0	0	0	0	0	0
ENSMUSG00000109389	0	0	0	0	0	0
ENSMUSG00000107596	2	0	1	6	2	2
ENSMUSG00000107597	0	0	3	0	0	0
ENSMUSG00000109388	0	0	0	0	0	0
ENSMUSG00000065823	0	0	0	0	0	0
ENSMUSG00000041431	72	61	57	4	35	35
ENSMUSG00000065825	0	0	0	0	0	0
ENSMUSG00000065828	1	1	1	0	0	0
ENSMUSG00000098563	0	0	0	0	0	0
ENSMUSG00000114844	0	0	0	0	0	0
ENSMUSG00000064446	0	0	0	0	0	0
ENSMUSG00000064444	0	0	0	1	0	0
ENSMUSG00000102794	0	0	0	0	0	0
ENSMUSG00000096341	0	0	0	0	0	0
ENSMUSG00000079628	0	0	0	0	0	0
ENSMUSG00000079629	0	0	0	0	0	0
ENSMUSG00000097029	0	0	0	0	0	0
ENSMUSG00000079620	9	4	2	12	15	26
ENSMUSG00000079625	0	6	0	3	0	0
ENSMUSG00000079626	0	0	0	0	0	0
ENSMUSG00000079627	6	0	4	0	0	4
ENSMUSG00000023120	0	0	0	0	0	0
ENSMUSG00000023122	0	0	0	0	0	0
ENSMUSG00000088530	0	0	0	0	0	0
ENSMUSG00000088533	0	0	0	0	0	0
ENSMUSG00000088535	0	0	0	0	0	0
ENSMUSG00000088536	0	0	0	0	0	0
ENSMUSG00000088538	0	0	0	0	0	0
ENSMUSG00000088539	0	0	0	0	0	0
ENSMUSG00000085375	0	0	0	0	0	0
ENSMUSG00000085371	1	4	0	1	0	1
ENSMUSG00000074011	0	0	0	0	0	0
ENSMUSG00000085378	0	0	0	0	0	0
ENSMUSG00000107042	0	0	0	1	0	0
ENSMUSG00000107043	0	0	0	0	0	0
ENSMUSG00000107046	0	0	0	0	0	0
ENSMUSG00000107047	0	0	0	0	0	0
ENSMUSG00000100832	0	0	0	0	0	0
ENSMUSG00000036770	0	0	0	0	0	0
ENSMUSG00000036777	234	191	231	62	84	161
ENSMUSG00000111630	0	0	0	0	0	0
ENSMUSG00000065311	0	0	0	0	0	0

ENSMUSG00000065313	0	0	0	0	0	0
ENSMUSG00000065312	0	0	0	0	0	0
ENSMUSG00000078577	0	0	0	0	0	0
ENSMUSG00000078576	0	0	0	0	0	0
ENSMUSG00000078575	0	0	0	0	0	0
ENSMUSG00000024479	15	5	6	0	6	5
ENSMUSG00000103740	0	0	0	0	0	0
ENSMUSG00000103745	0	0	0	0	2	0
ENSMUSG00000103744	0	0	0	0	0	0
ENSMUSG00000085969	0	0	0	0	0	0
ENSMUSG00000097254	0	0	10	3	5	0
ENSMUSG00000097256	0	0	0	0	0	0
ENSMUSG00000059352	0	0	2	0	0	0
ENSMUSG00000039742	0	0	0	0	0	0
ENSMUSG00000032297	0	0	0	0	0	0
ENSMUSG00000039747	141	131	326	58	47	104
ENSMUSG00000032291	0	0	0	0	0	0
ENSMUSG00000032292	0	0	0	0	0	0
ENSMUSG00000039748	28	19	21	0	12	24
ENSMUSG00000017868	0	0	0	0	0	0
ENSMUSG00000085965	0	4	1	1	3	4
ENSMUSG00000100809	0	0	0	0	0	0
ENSMUSG00000100808	0	0	0	0	0	0
ENSMUSG00000114529	8	5	10	11	7	11
ENSMUSG00000114528	0	0	0	0	0	0
ENSMUSG00000062327	0	0	0	0	0	0
ENSMUSG00000114525	0	0	0	0	0	0
ENSMUSG00000100802	0	0	0	0	0	0
ENSMUSG00000114527	0	0	0	0	0	0
ENSMUSG00000100800	0	0	0	0	0	0
ENSMUSG00000100807	0	0	0	0	2	0
ENSMUSG00000114520	0	1	0	0	0	0
ENSMUSG00000100805	0	0	0	0	0	0
ENSMUSG00000100804	0	0	0	0	0	0
ENSMUSG00000058400	0	8	0	0	0	4
ENSMUSG00000058402	154	135	196	129	196	116
ENSMUSG00000050654	4	0	0	0	3	3
ENSMUSG00000050650	0	0	0	0	0	0
ENSMUSG00000069922	0	0	0	0	0	0
ENSMUSG00000069925	0	0	0	0	0	0
ENSMUSG00000105351	0	0	0	0	0	0
ENSMUSG00000105352	0	0	0	0	0	0
ENSMUSG00000105353	1	1	1	1	1	1
ENSMUSG00000105354	0	0	0	0	0	0
ENSMUSG00000023902	0	2	0	0	0	2
ENSMUSG00000023903	6	0	9	0	4	0
ENSMUSG00000023906	0	0	0	0	0	0
ENSMUSG00000096614	0	0	1	1	0	1
ENSMUSG00000096619	0	2	0	0	0	0
ENSMUSG00000108657	0	0	0	0	0	0
ENSMUSG00000108654	0	0	0	0	0	0
ENSMUSG00000108652	0	0	0	0	0	0
ENSMUSG00000108651	0	0	0	0	0	0
ENSMUSG00000091961	0	0	0	0	0	0
ENSMUSG00000091960	0	0	0	0	0	0
ENSMUSG00000091962	0	0	0	0	0	0
ENSMUSG00000105977	2	2	3	3	3	4
ENSMUSG00000057000	4	0	0	0	0	0

ENSMUSG00000057003	0	0	0	0	0	0
ENSMUSG00000101071	0	0	0	0	0	0
ENSMUSG00000101073	0	0	0	0	0	0
ENSMUSG00000101072	0	0	0	1	0	0
ENSMUSG00000050382	117	76	132	70	88	128
ENSMUSG00000050383	0	0	0	0	0	0
ENSMUSG00000104009	2	0	0	0	0	0
ENSMUSG00000101076	0	0	0	0	0	0
ENSMUSG00000104007	0	0	0	0	0	0
ENSMUSG00000101078	0	0	0	0	0	0
ENSMUSG00000104004	0	0	0	2	0	0
ENSMUSG00000104001	0	1	0	2	0	0
ENSMUSG00000104000	0	0	4	0	0	8
ENSMUSG00000111005	0	0	0	0	0	0
ENSMUSG00000113890	1	0	0	0	0	1
ENSMUSG00000113891	0	0	0	0	0	0
ENSMUSG00000113896	0	0	0	0	0	0
ENSMUSG00000113897	0	0	0	0	0	4
ENSMUSG00000064831	0	0	0	0	0	0
ENSMUSG00000064830	0	0	0	0	0	0
ENSMUSG00000064836	0	0	0	0	0	0
ENSMUSG00000084060	0	0	0	0	0	0
ENSMUSG00000091347	0	0	0	0	0	0
ENSMUSG00000059434	0	0	3	0	0	0
ENSMUSG00000098232	8	5	13	7	15	12
ENSMUSG00000081119	0	0	0	0	0	0
ENSMUSG00000098233	0	0	0	0	0	0
ENSMUSG00000081115	0	0	0	0	0	0
ENSMUSG00000081114	0	0	0	0	0	0
ENSMUSG00000081117	0	0	0	0	0	0
ENSMUSG00000081116	0	0	0	0	0	0
ENSMUSG00000061292	0	0	0	0	0	0
ENSMUSG00000081112	0	0	0	0	0	0
ENSMUSG00000098231	0	0	0	0	0	0
ENSMUSG00000061295	0	0	0	0	0	0
ENSMUSG00000027107	9	6	9	3	4	5
ENSMUSG00000061296	0	0	0	0	0	0
ENSMUSG00000056061	0	0	0	0	0	0
ENSMUSG00000090762	0	0	0	0	0	0
ENSMUSG00000090764	0	0	0	0	0	0
ENSMUSG00000053654	0	0	0	0	0	0
ENSMUSG00000088772	0	0	0	0	0	0
ENSMUSG00000042251	400	426	404	582	580	730
ENSMUSG00000042250	0	0	0	0	0	0
ENSMUSG00000094449	0	0	0	0	0	0
ENSMUSG00000042256	4	4	0	0	3	0
ENSMUSG00000088773	0	0	0	0	0	0
ENSMUSG00000094445	0	0	0	0	0	0
ENSMUSG00000042258	0	0	0	0	0	0
ENSMUSG00000094443	0	0	0	0	4	0
ENSMUSG00000096446	0	0	0	0	0	0
ENSMUSG00000086460	0	0	0	0	0	0
ENSMUSG00000088774	0	0	0	0	0	0
ENSMUSG00000106841	0	0	0	0	0	0
ENSMUSG00000052520	0	0	0	0	0	0
ENSMUSG00000111142	2	0	0	0	0	0
ENSMUSG00000094728	0	1	1	1	1	0
ENSMUSG00000111143	0	0	0	0	0	0

ENSMUSG00000093835	0	0	0	0	0	0
ENSMUSG00000098564	0	0	0	0	0	0
ENSMUSG00000003585	75	52	81	21	41	67
ENSMUSG00000093238	0	0	0	0	0	0
ENSMUSG00000093236	0	0	0	0	0	0
ENSMUSG00000093234	0	0	0	0	0	0
ENSMUSG00000093231	0	0	0	0	0	0
ENSMUSG00000093230	0	0	0	0	0	0
ENSMUSG00000042357	20	13	30	0	7	0
ENSMUSG00000097938	0	0	0	0	0	0
ENSMUSG00000097939	0	0	0	0	0	0
ENSMUSG00000092723	0	0	0	0	0	0
ENSMUSG00000092720	2	0	0	0	0	0
ENSMUSG00000097937	0	0	2	0	1	1
ENSMUSG00000097931	0	0	0	0	0	0
ENSMUSG00000113246	0	0	0	0	0	0
ENSMUSG00000016200	7	6	5	0	0	9
ENSMUSG00000042351	24	8	30	4	4	10
ENSMUSG00000113243	0	0	0	0	0	0
ENSMUSG00000029414	67	54	90	5	24	77
ENSMUSG00000029410	0	8	16	2	3	6
ENSMUSG00000106098	0	0	0	0	0	0
ENSMUSG00000106097	0	0	0	0	3	0
ENSMUSG00000106094	0	0	0	0	0	0
ENSMUSG00000106093	2	13	8	0	10	0
ENSMUSG00000106090	0	0	0	0	0	0
ENSMUSG00000113248	0	2	2	2	4	0
ENSMUSG00000113337	0	0	0	0	0	0
ENSMUSG00000004113	3	6	3	2	11	9
ENSMUSG00000004110	9	18	19	0	14	16
ENSMUSG00000071772	0	0	0	0	0	0
ENSMUSG00000071773	0	0	0	0	0	0
ENSMUSG00000071770	0	0	0	0	0	0
ENSMUSG00000071771	0	0	0	0	0	0
ENSMUSG00000109247	0	0	0	0	0	0
ENSMUSG00000106548	0	0	0	0	0	0
ENSMUSG00000106549	5	14	5	8	5	6
ENSMUSG00000067220	0	0	0	0	0	0
ENSMUSG00000106544	0	0	0	0	0	0
ENSMUSG00000106545	0	0	0	0	0	0
ENSMUSG00000106547	37	46	48	60	47	84
ENSMUSG00000092186	0	0	0	0	0	0
ENSMUSG00000005864	9	5	7	4	0	0
ENSMUSG00000092182	14	8	18	11	10	10
ENSMUSG00000113862	0	0	0	0	0	0
ENSMUSG00000094035	10	4	8	7	12	2
ENSMUSG00000046474	0	0	0	0	0	0
ENSMUSG00000114645	0	0	0	0	0	0
ENSMUSG00000114646	0	0	0	0	0	0
ENSMUSG00000114647	0	0	0	0	0	0
ENSMUSG00000114640	0	0	0	0	0	0
ENSMUSG00000114641	0	0	0	0	0	0
ENSMUSG00000114643	0	0	0	0	0	0
ENSMUSG00000114648	0	0	0	0	0	0
ENSMUSG00000103600	0	0	0	0	0	0
ENSMUSG00000011263	80	87	104	38	26	40
ENSMUSG00000110157	0	0	0	0	0	0
ENSMUSG00000110151	0	0	0	0	0	0

ENSMUSG00000110152	0	0	0	0	0	0
ENSMUSG00000110153	0	0	0	0	0	0
ENSMUSG00000110158	0	0	0	0	0	0
ENSMUSG00000054034	9	4	3	0	0	0
ENSMUSG00000054036	0	0	0	2	0	0
ENSMUSG00000054031	0	0	0	0	0	0
ENSMUSG00000054033	4	0	0	0	0	0
ENSMUSG00000109376	0	0	0	0	0	0
ENSMUSG00000047995	0	0	3	0	0	0
ENSMUSG00000047992	3	0	0	0	0	4
ENSMUSG00000047990	0	0	0	0	0	0
ENSMUSG00000105016	0	0	0	0	0	0
ENSMUSG00000109375	0	0	0	0	0	0
ENSMUSG00000105013	0	0	0	0	0	0
ENSMUSG00000105011	3	4	0	4	6	4
ENSMUSG00000105010	0	0	0	0	0	0
ENSMUSG00000105018	0	0	0	0	0	0
ENSMUSG00000041237	0	0	0	0	0	0
ENSMUSG00000109371	0	0	0	0	0	2
ENSMUSG00000109072	0	0	0	0	0	0
ENSMUSG00000083236	0	0	0	0	0	2
ENSMUSG00000083237	0	0	2	0	0	0
ENSMUSG00000083234	0	0	0	0	0	0
ENSMUSG00000107922	0	0	0	0	0	0
ENSMUSG00000083230	2	0	0	0	0	0
ENSMUSG00000083231	0	0	0	2	0	0
ENSMUSG00000107925	0	0	0	0	0	0
ENSMUSG00000093420	0	0	0	0	0	0
ENSMUSG00000083238	0	0	0	0	0	3
ENSMUSG00000083239	0	0	0	0	0	0
ENSMUSG00000082704	0	0	0	0	0	0
ENSMUSG00000068522	4	7	0	0	0	0
ENSMUSG00000103427	256	263	243	150	136	178
ENSMUSG00000085173	0	2	0	0	1	0
ENSMUSG00000085179	0	0	0	0	0	0
ENSMUSG00000026770	50	48	45	51	42	57
ENSMUSG00000079350	0	0	0	0	0	0
ENSMUSG00000018849	32	13	11	0	0	0
ENSMUSG00000079359	0	0	0	0	0	0
ENSMUSG00000000120	15	3	46	0	18	10
ENSMUSG00000070857	0	0	0	0	0	0
ENSMUSG00000041907	8	2	2	6	0	6
ENSMUSG00000074655	0	0	0	0	0	0
ENSMUSG00000074653	2	2	0	2	0	0
ENSMUSG00000074651	0	0	0	0	0	0
ENSMUSG00000084492	0	0	1	1	0	0
ENSMUSG00000045267	0	0	2	0	0	0
ENSMUSG00000075266	22	56	21	27	25	34
ENSMUSG00000075267	0	0	0	0	0	0
ENSMUSG00000024233	0	0	0	0	0	0
ENSMUSG00000063430	68	31	160	4	6	5
ENSMUSG00000093519	0	0	0	0	0	0
ENSMUSG00000103602	0	0	0	0	0	0
ENSMUSG00000111638	0	0	0	0	0	0
ENSMUSG00000078008	0	0	0	0	0	0
ENSMUSG00000034923	0	0	0	0	0	0
ENSMUSG00000078002	0	0	0	0	0	0
ENSMUSG00000075269	4	0	3	3	0	0

ENSMUSG00000092559	12	12	4	1	2	5
ENSMUSG00000090249	0	0	0	0	0	0
ENSMUSG00000091227	0	0	0	0	0	0
ENSMUSG00000109074	5	8	7	7	5	7
ENSMUSG00000048573	0	0	0	0	0	0
ENSMUSG00000083077	4	0	4	0	0	4
ENSMUSG00000078218	0	0	0	0	0	0
ENSMUSG00000032101	0	2	0	0	0	0
ENSMUSG00000099613	0	0	0	0	0	0
ENSMUSG00000024032	0	0	0	0	0	0
ENSMUSG00000004872	4	0	0	0	0	0
ENSMUSG00000102890	0	0	0	0	0	0
ENSMUSG00000015001	0	5	11	8	0	0
ENSMUSG00000102896	0	0	0	0	0	0
ENSMUSG00000102897	2	0	0	0	0	0
ENSMUSG00000102894	0	0	4	0	0	0
ENSMUSG00000102895	0	0	0	0	3	2
ENSMUSG00000049625	100	95	202	53	70	56
ENSMUSG00000049620	0	0	0	0	0	0
ENSMUSG00000031766	7	18	11	18	7	36
ENSMUSG00000101278	0	0	0	0	0	0
ENSMUSG00000101277	0	0	0	0	0	0
ENSMUSG00000101276	0	0	0	0	0	0
ENSMUSG00000101275	0	0	0	0	0	0
ENSMUSG00000101273	0	0	0	0	0	0
ENSMUSG00000101272	0	0	0	0	0	0
ENSMUSG00000101271	0	0	0	0	0	0
ENSMUSG00000101270	0	0	0	0	0	0
ENSMUSG00000085940	0	0	0	0	0	0
ENSMUSG00000082111	1	0	3	0	0	0
ENSMUSG00000085946	0	0	0	0	0	0
ENSMUSG00000085949	0	0	0	0	0	0
ENSMUSG00000067848	0	0	0	0	0	0
ENSMUSG00000100958	0	0	0	0	0	0
ENSMUSG00000088288	0	0	0	0	0	0
ENSMUSG00000030411	127	153	144	96	175	205
ENSMUSG00000082628	0	0	0	0	0	0
ENSMUSG00000082629	0	3	0	0	0	0
ENSMUSG00000088282	0	0	0	0	0	0
ENSMUSG00000088283	0	0	0	0	0	0
ENSMUSG00000082624	0	0	0	0	0	0
ENSMUSG00000088281	0	0	0	0	0	0
ENSMUSG00000088286	0	0	0	0	0	0
ENSMUSG00000082623	0	3	0	0	0	0
ENSMUSG00000082620	0	0	0	0	0	0
ENSMUSG00000082621	0	0	0	0	0	0
ENSMUSG00000060311	0	0	0	0	0	0
ENSMUSG00000060317	0	0	0	0	0	0
ENSMUSG00000107943	0	0	0	0	0	0
ENSMUSG00000107941	0	0	0	0	0	0
ENSMUSG00000107940	0	0	0	0	0	0
ENSMUSG00000107946	7	4	4	4	0	0
ENSMUSG00000107945	0	0	0	1	0	0
ENSMUSG00000107948	0	0	0	0	0	3
ENSMUSG00000100639	0	0	0	0	0	0
ENSMUSG00000027345	0	0	0	0	0	0
ENSMUSG00000100630	0	0	0	0	0	0
ENSMUSG00000100634	0	0	0	0	0	0

ENSMUSG00000100636	3	0	0	0	0	0
ENSMUSG00000100637	0	0	0	0	0	0
ENSMUSG00000109076	0	11	0	7	8	6
ENSMUSG00000100939	0	0	0	0	0	0
ENSMUSG00000099307	1	1	1	1	2	1
ENSMUSG00000084703	0	0	0	0	0	0
ENSMUSG00000084702	0	0	0	0	0	0
ENSMUSG00000084700	0	0	0	0	0	0
ENSMUSG00000084705	0	0	0	0	0	0
ENSMUSG00000084704	0	0	1	0	0	0
ENSMUSG00000100933	0	0	0	0	0	0
ENSMUSG00000075249	0	0	0	0	0	2
ENSMUSG00000114416	0	0	0	0	0	0
ENSMUSG00000089425	0	0	0	0	0	0
ENSMUSG00000041578	0	0	0	0	0	0
ENSMUSG00000022441	0	0	0	0	0	0
ENSMUSG00000100935	0	0	0	0	0	0
ENSMUSG00000022449	46	55	77	16	12	14
ENSMUSG00000102702	8	9	5	10	11	8
ENSMUSG00000102701	0	0	0	0	0	0
ENSMUSG00000064762	0	0	0	0	0	0
ENSMUSG00000064761	0	0	0	0	0	0
ENSMUSG00000102705	1	1	1	2	4	1
ENSMUSG00000024842	0	0	11	8	0	6
ENSMUSG00000102709	7	7	13	11	6	12
ENSMUSG00000064769	0	0	0	0	0	0
ENSMUSG00000107365	0	3	0	0	0	0
ENSMUSG00000107363	3	10	7	5	11	10
ENSMUSG00000107360	0	0	0	0	0	0
ENSMUSG00000068647	2	0	0	2	0	0
ENSMUSG00000061195	0	0	0	0	0	0
ENSMUSG00000092871	0	0	0	0	0	0
ENSMUSG00000060862	575	561	710	451	580	767
ENSMUSG00000060863	0	0	0	0	0	0
ENSMUSG00000035694	0	0	0	0	0	0
ENSMUSG00000062426	0	0	0	0	0	0
ENSMUSG00000092878	0	0	0	0	0	0
ENSMUSG00000079016	0	0	0	0	0	0
ENSMUSG00000079015	0	0	0	0	0	0
ENSMUSG00000079014	46	13	104	3	0	0
ENSMUSG00000079013	0	0	0	0	0	0
ENSMUSG00000079011	0	0	0	0	0	0
ENSMUSG00000079010	0	0	0	0	0	0
ENSMUSG00000028125	70	113	92	130	116	113
ENSMUSG00000112204	0	0	0	0	0	0
ENSMUSG00000054191	0	4	6	0	0	9
ENSMUSG00000094939	0	0	0	0	0	0
ENSMUSG00000094931	3	0	0	0	0	0
ENSMUSG00000021403	5	15	23	0	0	4
ENSMUSG00000092398	0	0	0	0	0	0
ENSMUSG00000021404	0	0	0	0	0	0
ENSMUSG00000094937	0	0	0	0	0	0
ENSMUSG00000029658	0	8	0	0	0	0
ENSMUSG00000029656	0	0	0	0	0	0
ENSMUSG00000036923	48	17	70	44	22	13
ENSMUSG00000036921	0	0	0	0	0	0
ENSMUSG00000036925	0	0	0	0	0	0
ENSMUSG00000036928	16	13	5	8	6	25

ENSMUSG00000113133	0	0	0	0	0	0
ENSMUSG00000062400	0	0	0	0	0	0
ENSMUSG00000113131	0	0	0	0	0	0
ENSMUSG00000113130	0	0	0	3	0	3
ENSMUSG00000088709	0	0	0	0	0	0
ENSMUSG00000088707	0	0	0	0	0	0
ENSMUSG00000088706	0	0	0	0	0	0
ENSMUSG00000113139	0	0	0	0	0	0
ENSMUSG00000088704	0	0	0	0	0	0
ENSMUSG00000088703	0	0	0	1	0	2
ENSMUSG00000088701	0	0	0	0	0	0
ENSMUSG00000088700	0	0	0	0	0	0
ENSMUSG00000063779	0	0	0	0	5	0
ENSMUSG00000051081	0	0	0	0	0	0
ENSMUSG00000024154	0	0	0	0	0	0
ENSMUSG00000024155	5	13	9	0	8	3
ENSMUSG00000106704	0	0	0	0	0	0
ENSMUSG00000106705	0	0	0	0	0	0
ENSMUSG00000113689	0	4	6	2	0	4
ENSMUSG00000106703	0	0	0	2	0	0
ENSMUSG00000106701	0	0	0	0	0	0
ENSMUSG00000113685	0	0	0	0	0	0
ENSMUSG00000113684	0	2	1	0	0	1
ENSMUSG00000113686	0	0	0	0	0	0
ENSMUSG00000113680	12	22	14	32	12	13
ENSMUSG00000106708	0	0	0	0	0	0
ENSMUSG00000090589	0	0	0	0	0	0
ENSMUSG00000104700	0	0	0	0	0	0
ENSMUSG00000104702	0	0	0	0	0	0
ENSMUSG00000104705	2	2	0	2	2	2
ENSMUSG00000058022	0	0	0	0	0	0
ENSMUSG00000104708	2	6	4	6	8	6
ENSMUSG00000036902	96	48	81	79	79	106
ENSMUSG00000029386	151	124	157	66	119	119
ENSMUSG00000114480	0	0	0	0	0	0
ENSMUSG00000052295	0	0	0	0	0	5
ENSMUSG00000114486	0	0	0	0	0	0
ENSMUSG00000114484	0	0	0	0	0	0
ENSMUSG00000114485	0	0	0	1	0	0
ENSMUSG00000094971	1	1	1	1	0	1
ENSMUSG00000112369	0	0	0	0	2	0
ENSMUSG00000036907	3	0	0	0	0	0
ENSMUSG00000097779	3	0	11	5	4	4
ENSMUSG00000109736	3	0	0	0	0	0
ENSMUSG00000109737	5	0	5	0	0	0
ENSMUSG00000109734	0	0	0	0	0	0
ENSMUSG00000102284	0	0	0	0	0	0
ENSMUSG00000102283	0	0	0	0	0	0
ENSMUSG00000109733	0	0	0	0	0	0
ENSMUSG00000102281	0	0	0	0	0	0
ENSMUSG00000109731	0	6	4	2	5	10
ENSMUSG00000057903	0	0	0	0	0	0
ENSMUSG00000102288	0	0	0	0	0	0
ENSMUSG00000020436	1	0	0	0	0	0
ENSMUSG00000025401	7	0	0	0	0	0
ENSMUSG00000025400	0	0	0	0	0	0
ENSMUSG00000020431	198	173	198	448	371	374
ENSMUSG00000050028	0	0	0	0	0	0

ENSMUSG00000050023	0	0	0	0	0	0
ENSMUSG00000067103	0	0	0	0	2	0
ENSMUSG00000058618	0	0	0	0	0	0
ENSMUSG00000096796	0	0	0	0	0	0
ENSMUSG00000096794	0	1	0	0	0	0
ENSMUSG00000096793	15	13	14	7	7	12
ENSMUSG00000096792	0	0	0	0	0	0
ENSMUSG00000096790	0	0	0	0	0	0
ENSMUSG00000058613	0	0	0	0	0	0
ENSMUSG00000096799	0	0	0	0	0	0
ENSMUSG00000096798	0	0	0	0	0	0
ENSMUSG00000110565	0	0	0	0	0	0
ENSMUSG00000110564	0	0	0	0	0	0
ENSMUSG00000110566	0	0	0	0	0	0
ENSMUSG00000060459	0	0	0	0	0	0
ENSMUSG00000010086	0	5	0	0	0	0
ENSMUSG00000096421	0	0	0	0	0	0
ENSMUSG00000096420	0	0	0	0	0	0
ENSMUSG00000096423	0	0	0	0	0	0
ENSMUSG00000096425	1	0	0	0	0	0
ENSMUSG00000096424	0	0	0	0	0	0
ENSMUSG00000096427	0	0	0	0	0	0
ENSMUSG00000096426	1	1	0	0	0	0
ENSMUSG00000096429	0	0	0	0	0	0
ENSMUSG00000113915	0	0	1	0	0	0
ENSMUSG00000113914	0	0	0	3	0	0
ENSMUSG00000113916	0	0	0	0	0	0
ENSMUSG00000113911	0	0	0	0	0	0
ENSMUSG00000113912	0	0	0	0	0	0
ENSMUSG00000113919	0	0	0	0	0	0
ENSMUSG00000087124	0	0	0	0	0	0
ENSMUSG00000080476	0	2	0	0	0	0
ENSMUSG00000093584	0	0	0	0	0	0
ENSMUSG00000094918	0	0	0	0	0	0
ENSMUSG00000045065	0	3	0	3	0	10
ENSMUSG00000088239	0	0	0	0	0	0
ENSMUSG00000095212	0	0	0	0	0	0
ENSMUSG00000095214	0	0	0	0	0	0
ENSMUSG00000057270	0	0	0	0	0	0
ENSMUSG00000039683	87	72	88	14	29	42
ENSMUSG00000053593	0	0	6	0	0	0
ENSMUSG00000047518	13	4	3	4	5	2
ENSMUSG00000111219	0	0	0	0	0	0
ENSMUSG00000111215	0	0	0	0	0	0
ENSMUSG00000111216	0	0	0	0	0	0
ENSMUSG00000111217	0	0	0	0	1	2
ENSMUSG00000111210	0	0	0	0	0	0
ENSMUSG00000111211	15	5	14	3	22	3
ENSMUSG00000111213	5	4	6	5	5	9
ENSMUSG00000090485	0	0	0	0	0	0
ENSMUSG00000090487	0	0	0	0	0	0
ENSMUSG00000090486	0	0	0	0	0	0
ENSMUSG00000090481	0	0	0	0	0	0
ENSMUSG00000081709	0	0	0	0	0	0
ENSMUSG00000090482	0	0	0	0	0	0
ENSMUSG00000081704	0	0	0	0	0	0
ENSMUSG00000081706	0	0	0	0	0	0
ENSMUSG00000088802	0	0	0	0	0	0

ENSMUSG00000022126	6	4	4	0	0	0
ENSMUSG00000022123	13	6	52	0	0	0
ENSMUSG00000088803	0	0	0	0	0	0
ENSMUSG00000114558	28	37	32	45	32	23
ENSMUSG00000048776	30	33	59	0	4	11
ENSMUSG00000048775	0	0	0	0	0	0
ENSMUSG00000028240	0	0	0	0	0	0
ENSMUSG00000088804	0	0	0	2	2	0
ENSMUSG00000093688	0	0	0	0	0	0
ENSMUSG00000042041	0	0	0	0	0	0
ENSMUSG00000113150	3	1	0	0	0	1
ENSMUSG00000113152	0	0	0	0	0	5
ENSMUSG00000113154	9	8	10	15	8	10
ENSMUSG00000077627	0	0	0	0	0	0
ENSMUSG00000077624	0	0	2	0	2	2
ENSMUSG00000077622	0	0	0	0	0	0
ENSMUSG00000077292	0	0	0	0	0	0
ENSMUSG00000077628	0	0	0	0	0	0
ENSMUSG00000077629	0	0	0	0	0	0
ENSMUSG00000046748	0	4	0	0	0	0
ENSMUSG00000086166	0	0	0	0	0	0
ENSMUSG00000086161	0	0	0	0	0	0
ENSMUSG00000086160	0	4	0	0	0	0
ENSMUSG00000086162	0	0	0	0	0	0
ENSMUSG00000077294	0	0	0	0	0	0
ENSMUSG00000086168	0	0	0	0	0	0
ENSMUSG00000057751	242	196	245	119	132	166
ENSMUSG00000087652	0	0	0	0	0	0
ENSMUSG00000098817	0	0	0	0	0	0
ENSMUSG00000098815	1	1	1	1	1	0
ENSMUSG00000082428	1	0	0	2	0	0
ENSMUSG00000076288	0	0	0	0	0	0
ENSMUSG00000076286	0	0	1	0	0	0
ENSMUSG00000087410	0	0	0	0	0	0
ENSMUSG00000076284	0	0	0	0	0	0
ENSMUSG00000076282	0	0	0	0	0	0
ENSMUSG00000082422	0	0	0	0	0	0
ENSMUSG00000098819	0	0	0	0	0	0
ENSMUSG00000103066	0	1	0	1	1	0
ENSMUSG00000106681	0	0	0	0	5	0
ENSMUSG00000103067	0	0	0	0	0	0
ENSMUSG00000037529	0	0	0	0	0	0
ENSMUSG00000071104	15	0	0	0	0	0
ENSMUSG00000071102	5	7	7	5	8	7
ENSMUSG00000004328	26	16	12	44	46	35
ENSMUSG00000103063	0	0	0	0	0	0
ENSMUSG00000039977	46	33	50	37	27	35
ENSMUSG00000070852	0	3	0	0	0	0
ENSMUSG00000070853	0	0	0	0	0	0
ENSMUSG00000070855	0	5	0	0	3	0
ENSMUSG00000070856	0	0	0	0	0	0
ENSMUSG00000050921	39	35	55	7	18	12
ENSMUSG00000104136	11	14	17	14	14	20
ENSMUSG00000084148	6	5	2	6	0	4
ENSMUSG00000084142	0	0	0	0	0	0
ENSMUSG00000084147	0	0	0	0	0	0
ENSMUSG00000080863	0	0	0	0	0	0
ENSMUSG00000080861	0	0	0	0	0	0

ENSMUSG00000080868	0	0	0	0	0	0
ENSMUSG00000080869	0	0	0	0	0	0
ENSMUSG00000078706	0	0	0	0	0	0
ENSMUSG00000044071	16	6	3	0	10	11
ENSMUSG00000068480	1	4	1	1	3	1
ENSMUSG00000041009	0	0	0	0	0	0
ENSMUSG00000099879	22	27	26	27	25	22
ENSMUSG00000041000	36	41	62	9	22	46
ENSMUSG00000062621	0	0	0	0	0	0
ENSMUSG00000062624	0	0	0	0	0	0
ENSMUSG00000030897	0	0	13	0	0	0
ENSMUSG00000030898	3	0	5	0	0	0
ENSMUSG00000062629	0	2	0	0	0	0
ENSMUSG00000065164	0	0	0	0	0	0
ENSMUSG00000065165	0	0	0	0	0	0
ENSMUSG00000108253	0	0	0	0	0	0
ENSMUSG00000102676	2	2	1	2	8	6
ENSMUSG00000062199	0	0	0	2	0	0
ENSMUSG00000073774	0	0	0	0	0	0
ENSMUSG00000073779	9	0	6	0	0	0
ENSMUSG00000098045	3	0	5	5	0	3
ENSMUSG00000098044	1	0	0	0	0	0
ENSMUSG00000098047	0	0	0	0	0	0
ENSMUSG00000098046	0	0	0	0	0	0
ENSMUSG00000098040	28	51	22	60	48	46
ENSMUSG00000098043	4	0	2	0	2	0
ENSMUSG00000098042	0	0	0	0	0	0
ENSMUSG00000073197	26	18	31	10	14	13
ENSMUSG00000037974	0	0	0	0	0	0
ENSMUSG00000102677	0	0	0	0	0	0
ENSMUSG00000072825	323	283	383	231	323	274
ENSMUSG00000070691	12	19	57	9	17	16
ENSMUSG00000072822	0	0	0	0	0	0
ENSMUSG00000072821	0	0	0	0	0	0
ENSMUSG00000111799	0	0	0	0	0	0
ENSMUSG00000081345	0	0	0	1	0	0
ENSMUSG00000038840	4	0	0	0	0	0
ENSMUSG00000088284	0	0	0	0	1	1
ENSMUSG00000106116	0	0	0	0	2	0
ENSMUSG00000084938	1	0	3	0	0	0
ENSMUSG00000084939	0	0	0	0	0	0
ENSMUSG00000097218	1	1	1	1	2	1
ENSMUSG00000113867	0	0	0	0	0	0
ENSMUSG00000081342	0	0	0	0	0	0
ENSMUSG00000109710	0	0	0	0	0	0
ENSMUSG00000084932	0	1	0	0	0	0
ENSMUSG00000084933	0	0	0	0	0	0
ENSMUSG00000084934	6	0	6	4	2	0
ENSMUSG00000084935	0	0	0	0	0	0
ENSMUSG00000084937	0	0	0	0	0	0
ENSMUSG00000088285	0	0	0	0	0	0
ENSMUSG00000106118	0	0	0	0	0	0
ENSMUSG00000030194	0	0	0	0	0	0
ENSMUSG00000081348	2	0	0	0	4	0
ENSMUSG00000091531	0	0	1	0	0	2
ENSMUSG00000102917	9	5	7	8	8	7
ENSMUSG00000102915	1	2	0	0	0	0
ENSMUSG00000102913	0	0	0	0	0	0

ENSMUSG00000102911	0	0	0	0	0	0
ENSMUSG00000102910	0	0	0	0	0	0
ENSMUSG00000026258	3	0	0	0	0	0
ENSMUSG00000091396	0	0	0	0	0	0
ENSMUSG00000043549	0	0	0	0	0	0
ENSMUSG00000094307	0	0	0	1	0	0
ENSMUSG00000087358	0	0	0	0	0	0
ENSMUSG00000088124	0	0	0	0	0	0
ENSMUSG00000088123	0	0	0	0	0	0
ENSMUSG00000088122	0	0	0	0	0	0
ENSMUSG00000088121	0	0	0	0	0	0
ENSMUSG00000101758	0	0	0	0	0	0
ENSMUSG00000101759	0	0	0	0	0	0
ENSMUSG00000101752	0	0	0	0	0	0
ENSMUSG00000094301	0	0	0	0	0	0
ENSMUSG00000033156	0	0	0	8	3	0
ENSMUSG00000106898	2	0	0	0	2	0
ENSMUSG00000022991	0	0	0	0	0	0
ENSMUSG00000112298	8	9	11	12	5	13
ENSMUSG00000094308	0	0	0	0	0	0
ENSMUSG00000040017	0	0	0	0	0	0
ENSMUSG00000104987	5	6	6	5	7	6
ENSMUSG00000040013	0	0	0	0	0	0
ENSMUSG00000104981	0	0	0	0	0	0
ENSMUSG00000059864	8	8	6	14	9	12
ENSMUSG00000104988	2	3	2	1	3	3
ENSMUSG00000009145	62	69	57	86	41	71
ENSMUSG00000103666	0	0	0	0	0	0
ENSMUSG00000103667	0	0	0	0	0	0
ENSMUSG00000103665	2	0	0	0	0	0
ENSMUSG00000100407	0	0	0	0	0	0
ENSMUSG00000100406	0	0	0	0	0	0
ENSMUSG00000100404	0	0	0	0	0	0
ENSMUSG00000100403	0	0	0	0	0	0
ENSMUSG00000107752	0	2	0	1	3	0
ENSMUSG00000107757	0	0	0	2	2	0
ENSMUSG00000107754	0	0	0	0	0	0
ENSMUSG00000089297	0	0	0	0	0	0
ENSMUSG00000107758	0	0	0	0	0	0
ENSMUSG00000089293	0	0	0	0	0	0
ENSMUSG00000089291	0	0	0	0	0	0
ENSMUSG00000020258	65	34	61	73	83	81
ENSMUSG00000095947	0	0	2	1	0	1
ENSMUSG00000095946	0	0	0	0	0	0
ENSMUSG00000020255	2561	2879	2748	2648	2586	3295
ENSMUSG00000095944	0	0	0	0	0	0
ENSMUSG00000095942	0	0	0	0	0	0
ENSMUSG00000095940	0	0	0	4	1	2
ENSMUSG00000065400	0	0	1	0	0	2
ENSMUSG00000065402	0	0	0	0	0	0
ENSMUSG00000065404	0	0	0	0	0	0
ENSMUSG00000065407	0	0	0	0	0	0
ENSMUSG00000065408	0	1	0	1	1	0
ENSMUSG00000094012	0	0	0	0	0	0
ENSMUSG00000094010	0	0	0	0	0	0
ENSMUSG00000094011	0	0	0	0	0	0
ENSMUSG00000094015	1	2	1	0	1	0
ENSMUSG00000089748	0	0	0	0	0	0

ENSMUSG00000089749	0	0	0	0	0	0
ENSMUSG00000052951	0	0	0	0	6	2
ENSMUSG00000052955	0	0	0	0	0	0
ENSMUSG00000076459	1	1	3	0	2	0
ENSMUSG00000058807	0	0	0	0	0	0
ENSMUSG00000058802	2	0	0	0	0	0
ENSMUSG00000112453	0	0	0	0	0	0
ENSMUSG00000112452	0	0	0	0	0	0
ENSMUSG00000112451	0	0	0	0	0	0
ENSMUSG00000102132	0	0	0	0	0	0
ENSMUSG00000112457	0	0	0	0	0	0
ENSMUSG00000112456	0	0	0	0	0	0
ENSMUSG00000112455	0	0	0	0	0	0
ENSMUSG00000112454	0	4	0	0	0	0
ENSMUSG00000102138	0	0	0	0	0	0
ENSMUSG00000112458	0	0	0	0	0	0
ENSMUSG00000081367	0	0	0	0	0	0
ENSMUSG00000114855	0	0	0	0	0	0
ENSMUSG00000114854	0	0	0	0	0	2
ENSMUSG00000114856	0	0	0	0	0	0
ENSMUSG00000114850	0	0	0	0	0	0
ENSMUSG00000114852	0	0	0	0	0	0
ENSMUSG00000110349	0	0	0	0	0	0
ENSMUSG00000110348	0	0	0	1	0	0
ENSMUSG00000110347	0	0	0	0	0	0
ENSMUSG00000110346	0	0	0	0	0	0
ENSMUSG00000110343	0	0	0	0	0	0
ENSMUSG00000069189	0	0	0	0	0	0
ENSMUSG00000092228	0	0	0	0	0	0
ENSMUSG00000081200	0	0	2	0	0	0
ENSMUSG00000029026	8	0	6	0	0	0
ENSMUSG00000097093	39	29	22	32	24	40
ENSMUSG00000097092	0	0	0	0	0	4
ENSMUSG00000034683	2	7	0	0	0	0
ENSMUSG00000097095	0	2	0	0	0	0
ENSMUSG00000034689	0	0	0	0	0	0
ENSMUSG00000088914	0	0	0	0	0	0
ENSMUSG00000081939	0	0	0	0	0	0
ENSMUSG00000088910	0	0	0	1	0	0
ENSMUSG00000088911	0	0	0	0	0	0
ENSMUSG00000081938	0	0	0	0	0	0
ENSMUSG00000021611	15	2	20	0	5	2
ENSMUSG00000021612	0	0	0	0	0	0
ENSMUSG00000111435	0	0	0	0	0	0
ENSMUSG00000111433	0	0	0	0	0	0
ENSMUSG00000096322	0	0	0	0	0	0
ENSMUSG00000096323	0	0	0	0	0	0
ENSMUSG00000042873	10	12	9	19	25	13
ENSMUSG00000096324	0	0	0	0	0	0
ENSMUSG00000096328	0	0	0	0	0	0
ENSMUSG00000106935	0	0	0	0	0	0
ENSMUSG00000034359	0	0	0	0	0	0
ENSMUSG00000106936	0	0	0	0	1	0
ENSMUSG00000113498	0	0	0	0	0	0
ENSMUSG00000113499	0	0	0	0	0	0
ENSMUSG00000106932	0	0	2	0	0	0
ENSMUSG00000113495	4	0	0	0	0	2
ENSMUSG00000113496	0	0	0	0	0	0

ENSMUSG00000106939	0	0	0	0	0	0
ENSMUSG00000106938	0	0	0	0	0	0
ENSMUSG00000113492	6	0	0	0	0	2
ENSMUSG00000113493	0	0	0	0	0	0
ENSMUSG00000108362	0	2	0	0	0	0
ENSMUSG00000059042	0	0	0	0	0	0
ENSMUSG00000059043	6	0	0	4	4	6
ENSMUSG00000109945	0	4	4	0	0	4
ENSMUSG00000109944	0	0	0	0	0	0
ENSMUSG00000114149	0	0	0	0	0	0
ENSMUSG00000114148	39	49	30	73	81	102
ENSMUSG00000109943	0	0	0	0	0	0
ENSMUSG00000114143	0	3	5	0	5	4
ENSMUSG00000114142	0	0	0	0	0	0
ENSMUSG00000114141	0	0	0	0	0	0
ENSMUSG00000114140	0	0	0	0	0	0
ENSMUSG00000109949	0	0	0	0	0	0
ENSMUSG00000109948	0	0	0	0	0	0
ENSMUSG00000114145	0	0	0	0	0	0
ENSMUSG00000108090	0	3	0	1	0	0
ENSMUSG00000097636	3	20	5	0	0	4
ENSMUSG00000047898	0	0	0	0	2	0
ENSMUSG00000108094	0	0	0	0	0	0
ENSMUSG00000097632	38	20	18	21	45	56
ENSMUSG00000108097	0	0	0	0	0	3
ENSMUSG00000108098	0	0	0	0	0	0
ENSMUSG00000108099	0	0	1	1	0	0
ENSMUSG00000047897	0	0	0	0	0	0
ENSMUSG00000097639	0	0	0	0	0	0
ENSMUSG00000097638	0	0	0	1	0	0
ENSMUSG00000091671	0	0	0	0	0	0
ENSMUSG00000091672	4	7	15	2	7	7
ENSMUSG00000091679	0	0	0	0	0	0
ENSMUSG00000025610	29	22	38	10	30	24
ENSMUSG00000054446	0	0	0	0	0	0
ENSMUSG00000022156	0	0	0	0	0	0
ENSMUSG00000022157	0	0	0	1	0	0
ENSMUSG00000112364	0	0	0	0	0	0
ENSMUSG00000083950	0	1	0	0	0	0
ENSMUSG00000101769	0	0	0	0	0	0
ENSMUSG00000094531	0	0	0	0	0	0
ENSMUSG00000094532	0	0	0	0	0	0
ENSMUSG00000042940	0	0	0	0	0	0
ENSMUSG00000038077	30	33	31	95	44	110
ENSMUSG00000045631	0	0	0	0	0	0
ENSMUSG00000083956	0	0	0	0	0	0
ENSMUSG00000110843	0	0	0	0	0	0
ENSMUSG00000113652	0	0	0	0	0	0
ENSMUSG00000072605	0	0	0	0	0	0
ENSMUSG00000083287	24	29	46	23	24	27
ENSMUSG00000006313	12	9	3	6	10	17
ENSMUSG00000006310	29	24	14	14	35	37
ENSMUSG00000006311	0	0	0	0	0	0
ENSMUSG00000053141	16	4	12	4	0	0
ENSMUSG00000072600	0	0	0	0	0	0
ENSMUSG00000100009	17	35	18	24	21	29
ENSMUSG00000095002	3	3	0	0	0	0
ENSMUSG00000090071	0	0	1	0	0	0

ENSMUSG00000095000	1	2	1	0	1	0
ENSMUSG00000090073	0	0	0	0	0	0
ENSMUSG00000095006	0	0	0	0	0	0
ENSMUSG00000111397	0	0	0	0	2	0
ENSMUSG00000095005	0	0	0	0	0	0
ENSMUSG00000111399	0	0	0	0	0	0
ENSMUSG00000111398	14	5	10	5	1	6
ENSMUSG00000095008	0	0	0	0	0	0
ENSMUSG00000095009	0	0	0	0	0	0
ENSMUSG00000111829	0	0	0	0	0	0
ENSMUSG00000098203	0	0	0	0	0	0
ENSMUSG00000098202	25	8	19	6	4	6
ENSMUSG00000098200	0	0	0	0	0	0
ENSMUSG00000098207	0	0	0	0	0	0
ENSMUSG00000098206	57	42	62	19	32	35
ENSMUSG00000098205	0	0	0	0	0	0
ENSMUSG00000098209	0	0	0	0	0	0
ENSMUSG00000098208	9	8	20	12	18	8
ENSMUSG00000047702	3	2	2	3	2	4
ENSMUSG00000042367	0	2	4	0	5	0
ENSMUSG00000094750	0	0	0	0	0	0
ENSMUSG00000094753	0	0	0	0	0	0
ENSMUSG00000094755	0	0	0	0	0	0
ENSMUSG00000094757	0	0	0	0	0	0
ENSMUSG00000042360	0	0	0	0	0	0
ENSMUSG00000094759	0	0	0	0	0	0
ENSMUSG00000093522	0	0	0	0	0	0
ENSMUSG00000109163	1	2	2	0	4	0
ENSMUSG00000111820	0	0	0	0	0	0
ENSMUSG00000052234	0	0	0	0	0	0
ENSMUSG00000052236	0	0	0	0	0	0
ENSMUSG00000070609	0	0	0	0	0	0
ENSMUSG00000109167	14	15	20	13	7	17
ENSMUSG00000110549	0	0	0	0	0	0
ENSMUSG00000077188	0	0	0	0	0	0
ENSMUSG00000077181	0	0	0	0	0	0
ENSMUSG00000109164	0	0	0	0	0	0
ENSMUSG00000077184	0	0	0	0	0	0
ENSMUSG00000110085	0	3	0	0	0	0
ENSMUSG00000046924	0	0	0	0	0	0
ENSMUSG00000078984	0	0	2	0	2	2
ENSMUSG00000114925	0	0	0	0	0	0
ENSMUSG00000114924	0	0	0	0	0	0
ENSMUSG00000081579	0	0	0	0	0	0
ENSMUSG00000081578	0	2	0	0	0	0
ENSMUSG00000002341	0	0	0	0	0	0
ENSMUSG00000090691	18	15	23	11	11	4
ENSMUSG00000090692	0	0	0	0	0	0
ENSMUSG00000081573	0	0	0	0	0	0
ENSMUSG00000081571	0	0	0	0	0	0
ENSMUSG00000114926	8	0	4	0	0	0
ENSMUSG00000081577	0	0	0	0	0	0
ENSMUSG00000090699	0	0	0	0	0	0
ENSMUSG00000099648	0	0	0	0	0	0
ENSMUSG00000099649	0	0	0	0	0	0
ENSMUSG00000099644	0	0	0	0	0	0
ENSMUSG00000099645	0	0	0	0	0	0
ENSMUSG00000099646	0	0	0	0	0	0

ENSMUSG00000099640	0	0	0	0	0	0
ENSMUSG00000099641	0	0	0	0	0	0
ENSMUSG00000099642	0	0	0	0	0	0
ENSMUSG00000099643	0	0	0	0	0	0
ENSMUSG00000077455	0	0	0	0	0	0
ENSMUSG00000077456	0	0	0	0	0	0
ENSMUSG00000077451	0	0	0	0	0	0
ENSMUSG00000077453	0	0	0	0	0	0
ENSMUSG00000077459	0	0	0	0	0	0
ENSMUSG00000029923	13	14	16	6	6	24
ENSMUSG00000030131	0	0	0	0	0	0
ENSMUSG00000030134	64	50	69	14	17	26
ENSMUSG00000092349	0	0	3	0	0	0
ENSMUSG00000084517	0	0	0	0	0	0
ENSMUSG00000092340	0	0	0	0	0	0
ENSMUSG00000092342	0	4	0	0	0	0
ENSMUSG00000092343	0	0	0	0	0	0
ENSMUSG00000092346	0	0	0	0	0	0
ENSMUSG00000003436	0	0	0	0	0	0
ENSMUSG00000051951	83	37	70	16	19	22
ENSMUSG00000051952	0	0	0	0	0	0
ENSMUSG00000043705	0	0	0	0	0	0
ENSMUSG00000041700	0	0	4	0	0	0
ENSMUSG00000072664	0	0	0	0	0	0
ENSMUSG00000041703	0	0	0	0	0	0
ENSMUSG00000072663	0	2	0	2	2	2
ENSMUSG00000094390	1	0	1	1	1	0
ENSMUSG00000041708	0	0	0	0	0	0
ENSMUSG00000106806	0	0	0	0	0	0
ENSMUSG00000037482	0	0	3	0	2	0
ENSMUSG00000098999	0	0	2	0	0	0
ENSMUSG00000098998	1	6	1	3	1	1
ENSMUSG00000098993	0	0	0	0	0	0
ENSMUSG00000098991	0	0	0	0	0	0
ENSMUSG00000098996	1	1	1	1	1	0
ENSMUSG00000098994	0	0	0	0	0	0
ENSMUSG00000087628	0	0	0	0	0	0
ENSMUSG00000087620	0	0	3	0	0	2
ENSMUSG00000087622	0	0	0	0	0	0
ENSMUSG00000087625	1	0	1	0	0	0
ENSMUSG00000087624	0	0	3	0	0	0
ENSMUSG00000087627	19	12	4	9	0	4
ENSMUSG00000087626	0	4	1	4	0	0
ENSMUSG00000083289	0	0	0	0	0	0
ENSMUSG00000047286	0	0	0	0	0	0
ENSMUSG00000064110	0	0	0	0	0	0
ENSMUSG00000064115	18	17	3	53	47	55
ENSMUSG00000107510	0	0	0	0	0	0
ENSMUSG00000107512	0	0	0	0	0	0
ENSMUSG00000107513	0	0	0	0	0	0
ENSMUSG00000107516	0	0	0	0	0	0
ENSMUSG00000107517	0	0	0	0	0	0
ENSMUSG00000107518	0	0	0	0	0	0
ENSMUSG00000107519	0	0	0	0	0	0
ENSMUSG00000033569	2	0	2	4	3	8
ENSMUSG00000098173	0	0	0	0	0	0
ENSMUSG00000101829	0	0	0	0	0	0
ENSMUSG00000065648	0	0	0	0	0	0

ENSMUSG00000101822	0	0	0	0	0	0
ENSMUSG00000061549	0	0	0	0	0	0
ENSMUSG00000101820	0	0	0	0	0	0
ENSMUSG00000101821	0	0	0	0	0	0
ENSMUSG00000101826	0	0	0	0	0	0
ENSMUSG00000101824	0	0	0	0	0	0
ENSMUSG00000101825	0	0	0	0	0	0
ENSMUSG00000085662	0	0	0	0	0	0
ENSMUSG00000085660	0	0	0	0	0	0
ENSMUSG00000085666	11	19	20	17	14	31
ENSMUSG00000085665	17	20	16	12	20	28
ENSMUSG00000085669	2	2	6	0	0	5
ENSMUSG00000085668	0	0	0	0	0	0
ENSMUSG00000074366	0	0	0	0	0	0
ENSMUSG00000001103	9	5	0	0	6	6
ENSMUSG00000074365	0	0	0	0	0	0
ENSMUSG00000074361	4	7	72	56	3	50
ENSMUSG00000074369	0	0	0	0	0	0
ENSMUSG00000030911	10	7	11	11	20	18
ENSMUSG00000081964	0	0	0	3	0	0
ENSMUSG00000065398	2	0	0	1	0	0
ENSMUSG00000081966	0	0	0	0	0	0
ENSMUSG00000081960	0	0	0	0	0	0
ENSMUSG00000081961	0	0	0	0	0	0
ENSMUSG00000081969	2	2	1	2	2	1
ENSMUSG00000109780	0	0	0	0	0	0
ENSMUSG00000074385	0	0	0	0	0	0
ENSMUSG00000112430	5	11	4	2	16	16
ENSMUSG00000062017	0	0	4	0	0	0
ENSMUSG00000082065	0	0	0	0	0	0
ENSMUSG00000085085	0	4	0	0	0	0
ENSMUSG00000085082	0	0	0	0	0	0
ENSMUSG00000099582	15	7	3	0	7	14
ENSMUSG00000035246	21	13	16	8	4	10
ENSMUSG00000102030	0	0	6	6	5	4
ENSMUSG00000102031	0	0	0	0	0	0
ENSMUSG00000102032	0	0	0	0	0	0
ENSMUSG00000102033	0	0	0	0	0	0
ENSMUSG00000102035	0	0	0	0	0	0
ENSMUSG00000102036	0	0	0	0	0	0
ENSMUSG00000102037	14	11	35	6	0	0
ENSMUSG00000102039	0	0	0	0	0	0
ENSMUSG00000100883	0	0	0	0	0	0
ENSMUSG00000100882	0	0	0	0	0	0
ENSMUSG00000100881	0	0	0	0	0	0
ENSMUSG00000100887	0	0	0	0	0	0
ENSMUSG00000100886	0	0	0	0	0	0
ENSMUSG00000100885	0	0	0	0	0	0
ENSMUSG00000100884	0	0	0	0	0	0
ENSMUSG00000100889	0	0	0	0	0	0
ENSMUSG00000100888	0	0	0	0	0	0
ENSMUSG00000079602	0	0	0	0	0	0
ENSMUSG00000079603	0	0	0	0	0	0
ENSMUSG00000113939	0	0	0	0	0	0
ENSMUSG00000082069	0	0	0	0	0	0
ENSMUSG00000069430	0	0	0	0	0	0
ENSMUSG00000057216	0	0	0	0	0	0
ENSMUSG00000096120	0	0	0	0	1	0

ENSMUSG00000113933	0	0	0	0	0	0
ENSMUSG00000096122	0	0	0	0	0	0
ENSMUSG00000089888	0	0	0	0	0	0
ENSMUSG00000096124	0	0	0	0	0	0
ENSMUSG00000096126	0	0	1	0	0	0
ENSMUSG00000096127	0	0	0	0	0	0
ENSMUSG00000089887	4	0	0	0	0	0
ENSMUSG00000089885	0	0	0	0	0	0
ENSMUSG00000034110	183	192	193	116	159	231
ENSMUSG00000034112	5	3	2	0	2	0
ENSMUSG00000034115	0	0	0	0	0	0
ENSMUSG00000113934	0	0	0	0	0	0
ENSMUSG00000101345	0	0	0	0	0	0
ENSMUSG00000101343	0	0	0	0	0	0
ENSMUSG00000099585	0	0	0	0	0	0
ENSMUSG00000101342	0	0	0	0	0	0
ENSMUSG00000096698	0	0	0	0	0	0
ENSMUSG00000096692	0	0	0	0	0	1
ENSMUSG00000096690	0	0	0	0	0	0
ENSMUSG00000101340	0	0	0	0	0	0
ENSMUSG00000023987	0	0	0	0	0	0
ENSMUSG00000096694	2	0	2	0	0	0
ENSMUSG00000096695	0	0	0	0	0	0
ENSMUSG00000063058	0	0	0	0	0	0
ENSMUSG00000088512	0	0	0	0	0	0
ENSMUSG00000101846	0	0	0	0	0	0
ENSMUSG00000101847	0	1	0	0	0	0
ENSMUSG00000091477	0	0	0	0	0	0
ENSMUSG00000091475	43	38	59	16	27	15
ENSMUSG00000091472	17	35	19	17	24	38
ENSMUSG00000091470	0	0	0	0	0	0
ENSMUSG00000111720	3	0	4	0	0	0
ENSMUSG00000088515	0	0	0	0	0	0
ENSMUSG00000020867	8	0	6	0	8	8
ENSMUSG00000060621	0	6	0	0	0	0
ENSMUSG00000096130	0	0	0	0	0	0
ENSMUSG00000056350	0	0	0	0	0	0
ENSMUSG00000100308	0	0	0	0	0	0
ENSMUSG00000100300	0	0	0	0	0	0
ENSMUSG00000061972	17	19	19	14	4	9
ENSMUSG00000100304	0	0	0	0	0	0
ENSMUSG00000100305	0	0	0	0	0	0
ENSMUSG00000061977	0	0	0	0	0	0
ENSMUSG00000110938	0	0	0	0	0	0
ENSMUSG00000110930	9	4	0	2	2	2
ENSMUSG00000110936	0	0	0	2	0	0
ENSMUSG00000110937	0	0	0	0	0	0
ENSMUSG00000064252	2	0	0	0	0	0
ENSMUSG00000064259	0	0	0	0	0	0
ENSMUSG00000021804	0	0	0	2	0	0
ENSMUSG00000021803	3	0	12	0	0	0
ENSMUSG00000095182	0	0	0	0	0	0
ENSMUSG00000089084	0	0	0	0	0	0
ENSMUSG00000088374	0	0	0	0	0	0
ENSMUSG00000088370	0	0	0	0	0	0
ENSMUSG00000033508	7	12	11	8	5	2
ENSMUSG00000022577	0	0	0	0	0	0
ENSMUSG00000107657	0	0	3	1	1	2

ENSMUSG00000107656	0	0	0	0	0	0
ENSMUSG00000095578	0	0	0	0	0	0
ENSMUSG00000107652	0	0	0	0	0	0
ENSMUSG00000107651	2	3	2	0	0	0
ENSMUSG00000046999	16	18	70	10	2	18
ENSMUSG00000095573	0	0	0	0	0	0
ENSMUSG00000095570	0	0	0	0	0	0
ENSMUSG00000095576	0	0	3	2	0	4
ENSMUSG00000095577	0	0	0	0	0	0
ENSMUSG00000103147	8	5	1	4	1	0
ENSMUSG00000103145	6	0	7	6	4	2
ENSMUSG00000103140	0	0	0	0	0	0
ENSMUSG00000104401	0	0	0	0	0	0
ENSMUSG00000103148	0	1	0	0	0	0
ENSMUSG00000112398	0	0	0	0	0	0
ENSMUSG00000112399	0	0	0	0	0	0
ENSMUSG00000109197	0	0	0	0	0	0
ENSMUSG00000094221	0	0	0	0	0	0
ENSMUSG00000027895	0	0	9	4	7	0
ENSMUSG00000112392	0	0	0	0	2	0
ENSMUSG00000094222	0	0	0	0	0	0
ENSMUSG00000112394	0	0	0	0	0	0
ENSMUSG00000112395	0	0	0	0	0	0
ENSMUSG00000112396	0	0	0	0	0	0
ENSMUSG00000103726	3	0	5	0	11	15
ENSMUSG00000109193	0	0	0	4	0	0
ENSMUSG00000036231	0	0	0	0	0	0
ENSMUSG00000098237	0	0	0	0	0	0
ENSMUSG00000106017	0	0	0	0	0	2
ENSMUSG00000106015	0	3	5	4	4	0
ENSMUSG00000106013	0	0	0	0	0	0
ENSMUSG00000106010	9	20	9	10	5	15
ENSMUSG00000106011	0	0	0	0	0	0
ENSMUSG00000076145	3	1	2	0	0	0
ENSMUSG00000111523	3	0	0	0	2	0
ENSMUSG00000053820	0	0	0	0	0	0
ENSMUSG00000018907	0	0	0	0	0	0
ENSMUSG00000106210	0	0	0	0	0	0
ENSMUSG00000017733	0	0	0	0	0	0
ENSMUSG00000009487	0	2	0	0	13	0
ENSMUSG00000020042	110	67	107	148	143	211
ENSMUSG00000111520	0	0	0	2	0	2
ENSMUSG00000106214	60	46	39	22	20	14
ENSMUSG00000028546	0	4	0	4	0	0
ENSMUSG00000028544	0	0	0	0	0	0
ENSMUSG00000092107	0	0	0	0	0	0
ENSMUSG00000106217	0	0	0	0	0	0
ENSMUSG00000092109	0	0	0	0	0	0
ENSMUSG00000112647	6	4	0	0	6	6
ENSMUSG00000004626	101	122	125	66	93	87
ENSMUSG00000112648	0	0	0	0	0	0
ENSMUSG00000055078	0	0	0	0	0	0
ENSMUSG00000008999	35	53	33	54	54	81
ENSMUSG00000113513	0	0	0	0	0	0
ENSMUSG00000113512	0	0	0	0	0	0
ENSMUSG00000113515	9	13	18	12	18	29
ENSMUSG00000113514	3	0	8	3	3	4
ENSMUSG00000113517	0	3	7	6	6	12

ENSMUSG00000113518	0	0	0	0	0	0
ENSMUSG00000069376	181	227	239	195	180	232
ENSMUSG00000100569	0	0	0	0	0	0
ENSMUSG00000069378	2	0	0	5	0	0
ENSMUSG00000103180	0	0	0	0	0	0
ENSMUSG00000111524	1	0	0	0	0	0
ENSMUSG00000082859	0	0	0	0	0	0
ENSMUSG00000082858	0	0	0	0	0	0
ENSMUSG00000082851	6	6	1	10	6	3
ENSMUSG00000082850	0	0	0	0	0	0
ENSMUSG00000082853	0	0	0	0	0	0
ENSMUSG00000082852	0	0	0	0	0	0
ENSMUSG00000082856	0	0	0	0	0	0
ENSMUSG00000050511	13	10	5	30	21	28
ENSMUSG00000070421	0	3	0	0	0	0
ENSMUSG00000103189	0	3	0	1	0	2
ENSMUSG00000100563	0	0	0	0	0	0
ENSMUSG00000105099	0	0	0	0	0	1
ENSMUSG00000105098	0	0	0	0	0	0
ENSMUSG00000105097	0	0	0	0	0	0
ENSMUSG00000105094	0	0	0	0	0	0
ENSMUSG00000105092	0	0	0	0	0	0
ENSMUSG00000105091	0	1	0	1	0	0
ENSMUSG00000105090	0	0	0	0	0	0
ENSMUSG00000108114	2	0	0	0	0	0
ENSMUSG00000108117	0	0	0	0	0	0
ENSMUSG00000108110	0	0	0	0	0	0
ENSMUSG00000108113	0	0	0	0	0	0
ENSMUSG00000109449	0	0	0	0	0	0
ENSMUSG00000108119	0	2	0	0	0	0
ENSMUSG00000108118	0	0	0	0	0	0
ENSMUSG00000047911	2	3	9	0	7	0
ENSMUSG00000111600	0	2	0	0	0	0
ENSMUSG00000111606	0	0	3	0	0	3
ENSMUSG00000026442	5	0	12	0	0	12
ENSMUSG00000026443	75	61	102	19	41	46
ENSMUSG00000057564	0	0	0	0	0	0
ENSMUSG00000099064	0	0	0	0	0	0
ENSMUSG00000109440	0	0	0	0	0	0
ENSMUSG00000109441	0	0	0	0	0	0
ENSMUSG00000025867	114	110	174	69	121	156
ENSMUSG00000111444	0	0	0	0	0	0
ENSMUSG00000104328	4	0	4	8	0	7
ENSMUSG00000104329	4	2	0	4	3	3
ENSMUSG00000104326	0	0	0	0	0	0
ENSMUSG00000104327	2	0	0	0	0	0
ENSMUSG00000104323	0	0	0	0	0	0
ENSMUSG00000104320	0	0	0	0	0	0
ENSMUSG00000104321	0	0	0	0	0	0
ENSMUSG00000077782	0	0	0	0	0	0
ENSMUSG00000022240	16	20	15	10	14	7
ENSMUSG00000083814	0	0	0	0	0	0
ENSMUSG00000083818	0	0	0	0	0	0
ENSMUSG00000064294	33	32	12	19	25	30
ENSMUSG00000096820	0	0	0	0	0	0
ENSMUSG00000027793	0	0	0	0	0	0
ENSMUSG00000027790	0	2	0	0	0	0
ENSMUSG00000098900	55	105	72	67	90	86

ENSMUSG00000027796	151	107	130	121	128	160
ENSMUSG00000027794	0	0	0	0	0	0
ENSMUSG00000096828	0	0	0	0	0	0
ENSMUSG00000041986	0	0	0	2	0	4
ENSMUSG00000110065	1	3	4	2	1	2
ENSMUSG00000087027	0	0	0	0	0	0
ENSMUSG00000092956	0	0	0	0	0	0
ENSMUSG00000027420	35	34	11	14	10	13
ENSMUSG00000092953	0	0	0	0	0	0
ENSMUSG00000092952	0	0	0	0	0	1
ENSMUSG00000095631	0	0	0	0	0	0
ENSMUSG00000045404	56	74	53	30	9	60
ENSMUSG00000095634	0	0	0	0	0	0
ENSMUSG00000095637	0	0	0	0	0	0
ENSMUSG00000095636	0	0	0	0	0	0
ENSMUSG00000095639	0	0	0	0	0	0
ENSMUSG00000095638	0	0	0	0	0	0
ENSMUSG00000042750	0	0	0	0	0	0
ENSMUSG00000042751	19	20	11	8	16	9
ENSMUSG00000080545	0	0	0	0	0	0
ENSMUSG00000095824	25	16	21	18	13	27
ENSMUSG00000075378	0	0	0	0	0	0
ENSMUSG00000095827	0	0	0	0	0	0
ENSMUSG00000095829	0	0	0	0	0	0
ENSMUSG00000052005	0	4	0	0	0	0
ENSMUSG00000098417	0	0	0	0	0	0
ENSMUSG00000036790	25	10	21	8	10	19
ENSMUSG00000108861	0	0	0	0	0	0
ENSMUSG00000075370	2	0	0	0	0	0
ENSMUSG00000064883	0	0	0	0	0	0
ENSMUSG00000108862	0	0	0	0	0	0
ENSMUSG00000093776	0	0	0	0	0	0
ENSMUSG00000093774	0	0	0	0	0	0
ENSMUSG00000093775	0	3	0	0	0	0
ENSMUSG00000093770	0	0	0	0	0	0
ENSMUSG00000034833	15	34	22	12	16	8
ENSMUSG00000031452	5	0	5	0	0	0
ENSMUSG00000031450	19	36	31	10	15	27
ENSMUSG00000100827	0	0	0	0	0	0
ENSMUSG00000100038	0	0	0	0	0	0
ENSMUSG00000033343	0	0	0	0	0	1
ENSMUSG00000100823	0	0	0	0	0	0
ENSMUSG00000100822	0	0	0	0	0	0
ENSMUSG00000098862	0	0	0	0	0	0
ENSMUSG00000061527	0	0	0	0	0	0
ENSMUSG00000071434	0	0	0	0	0	0
ENSMUSG00000082338	0	0	0	0	0	0
ENSMUSG00000082339	0	0	0	0	0	0
ENSMUSG00000087127	0	0	0	0	0	0
ENSMUSG00000099439	0	0	0	0	0	0
ENSMUSG00000099438	0	0	0	0	0	0
ENSMUSG00000048312	0	0	0	0	0	0
ENSMUSG00000099433	0	0	0	0	0	0
ENSMUSG00000099431	0	0	0	0	0	0
ENSMUSG00000099437	0	0	0	0	0	0
ENSMUSG00000099435	0	0	0	0	0	0
ENSMUSG00000099434	0	0	0	0	0	0
ENSMUSG00000064436	0	0	0	0	0	0

ENSMUSG00000084479	0	0	0	0	0	0
ENSMUSG00000038415	4	5	6	6	2	0
ENSMUSG00000102636	0	0	0	0	0	0
ENSMUSG00000084473	0	0	0	0	0	0
ENSMUSG00000084475	0	0	0	2	0	0
ENSMUSG00000102637	0	0	0	0	0	0
ENSMUSG00000043932	0	4	0	0	3	0
ENSMUSG00000043939	8	12	11	0	0	0
ENSMUSG00000030491	3	0	5	8	0	6
ENSMUSG00000030492	0	0	0	0	0	0
ENSMUSG00000100146	0	0	0	0	0	0
ENSMUSG00000100147	13	10	19	12	23	30
ENSMUSG00000100145	1	1	0	0	0	0
ENSMUSG00000100148	0	0	0	0	0	0
ENSMUSG00000100149	0	0	0	0	0	0
ENSMUSG00000091326	1	0	0	0	0	0
ENSMUSG00000087789	0	0	0	0	0	0
ENSMUSG00000087786	0	0	0	0	0	0
ENSMUSG00000087781	0	0	0	0	0	0
ENSMUSG00000102789	0	0	0	0	0	0
ENSMUSG00000102787	0	0	0	0	0	0
ENSMUSG00000102785	3	0	0	2	0	0
ENSMUSG00000107495	3	2	3	3	5	5
ENSMUSG00000107494	0	0	0	0	0	0
ENSMUSG00000083177	0	0	0	0	0	0
ENSMUSG00000107496	0	0	0	0	0	0
ENSMUSG00000084787	0	0	0	0	0	0
ENSMUSG00000107493	5	15	4	8	18	11
ENSMUSG00000083172	0	0	0	0	0	0
ENSMUSG00000095172	0	0	0	0	0	0
ENSMUSG00000084789	0	0	2	0	0	0
ENSMUSG00000084788	2	5	2	0	0	0
ENSMUSG00000107498	0	0	0	0	0	0
ENSMUSG00000087878	0	0	0	0	0	0
ENSMUSG00000087879	0	0	0	0	0	0
ENSMUSG00000087871	0	0	0	0	0	0
ENSMUSG00000087873	0	0	0	0	0	0
ENSMUSG00000087875	0	0	0	1	0	1
ENSMUSG00000087877	0	0	0	0	0	0
ENSMUSG00000084053	0	0	0	0	0	0
ENSMUSG00000085431	0	0	0	0	0	0
ENSMUSG00000108717	0	0	0	0	0	0
ENSMUSG00000037246	0	0	0	0	0	0
ENSMUSG00000036437	20	32	14	32	37	52
ENSMUSG00000079093	0	0	0	0	0	0
ENSMUSG00000079091	0	0	0	0	0	0
ENSMUSG00000065057	0	1	0	0	0	0
ENSMUSG00000065053	0	0	0	0	0	0
ENSMUSG00000065050	0	0	0	0	0	0
ENSMUSG00000100035	0	0	0	0	0	0
ENSMUSG00000074111	0	0	0	0	0	0
ENSMUSG00000103554	0	0	0	0	0	0
ENSMUSG00000103555	0	0	0	0	0	0
ENSMUSG00000103556	6	2	2	7	6	10
ENSMUSG00000103557	0	0	0	0	0	0
ENSMUSG00000103559	2	2	0	0	0	0
ENSMUSG00000083304	0	0	0	0	0	0
ENSMUSG00000073460	32	18	10	4	11	14

ENSMUSG00000039661	4	7	4	0	0	6
ENSMUSG00000032394	0	3	0	0	0	0
ENSMUSG00000078324	1	1	0	0	0	0
ENSMUSG00000078320	0	0	0	0	0	0
ENSMUSG00000104853	0	0	0	0	0	0
ENSMUSG00000024227	0	0	0	0	0	0
ENSMUSG00000024224	0	0	0	0	0	0
ENSMUSG00000024225	0	0	0	0	0	0
ENSMUSG00000089436	0	0	0	0	0	0
ENSMUSG00000089438	0	0	0	0	0	0
ENSMUSG00000097441	0	0	0	0	0	0
ENSMUSG00000097446	1	0	0	0	2	1
ENSMUSG00000097447	1	3	5	4	3	3
ENSMUSG00000097448	0	6	5	0	0	0
ENSMUSG00000112331	0	0	0	0	0	0
ENSMUSG00000051002	0	0	0	0	0	0
ENSMUSG00000051003	0	2	0	0	0	0
ENSMUSG00000100928	0	0	0	0	0	0
ENSMUSG00000114408	0	0	0	0	0	0
ENSMUSG00000100920	0	0	0	0	0	0
ENSMUSG00000114403	0	0	2	0	2	2
ENSMUSG00000114400	0	0	0	0	0	0
ENSMUSG00000100923	0	0	0	4	1	3
ENSMUSG00000100925	0	0	0	0	0	0
ENSMUSG00000062200	0	0	0	0	0	0
ENSMUSG00000062201	0	0	0	0	0	0
ENSMUSG00000036699	8	0	4	4	0	7
ENSMUSG00000103662	30	10	13	2	5	9
ENSMUSG00000066584	0	0	0	0	0	0
ENSMUSG00000066586	0	0	0	0	0	0
ENSMUSG00000066583	0	0	0	0	0	0
ENSMUSG00000050751	34	18	29	11	14	19
ENSMUSG00000054236	1	0	0	0	0	0
ENSMUSG00000058568	0	0	0	0	0	0
ENSMUSG00000050756	0	0	0	0	0	0
ENSMUSG00000103663	0	0	0	0	0	0
ENSMUSG00000105258	0	0	5	6	0	9
ENSMUSG00000102208	0	0	0	0	0	0
ENSMUSG00000102207	5	6	0	4	3	3
ENSMUSG00000105254	0	0	0	0	0	0
ENSMUSG00000105257	2	0	1	0	0	0
ENSMUSG00000102204	6	6	7	6	5	8
ENSMUSG00000105251	0	0	0	1	0	0
ENSMUSG00000105250	0	0	0	0	0	0
ENSMUSG00000102200	0	3	0	0	0	0
ENSMUSG00000112334	0	0	0	0	0	0
ENSMUSG00000096719	0	0	5	0	0	0
ENSMUSG00000096717	0	0	0	0	0	0
ENSMUSG00000096716	0	0	0	0	0	0
ENSMUSG00000096714	0	0	0	0	0	0
ENSMUSG00000096711	0	0	0	0	0	0
ENSMUSG00000058692	0	0	0	0	0	0
ENSMUSG00000110640	0	0	0	0	0	0
ENSMUSG00000088320	0	0	0	0	0	0
ENSMUSG00000113135	0	0	0	0	0	0
ENSMUSG00000088049	0	0	0	0	0	0
ENSMUSG00000088322	0	0	0	0	0	1
ENSMUSG00000091847	0	0	0	0	0	0

ENSMUSG00000091844	2	5	4	2	0	2
ENSMUSG00000067186	0	0	0	0	0	0
ENSMUSG00000067338	0	6	3	6	10	0
ENSMUSG00000021303	8	14	7	3	6	3
ENSMUSG00000021301	18	10	5	5	11	3
ENSMUSG00000057761	0	0	0	0	0	0
ENSMUSG00000056509	0	0	0	0	0	0
ENSMUSG00000105401	18	11	12	8	8	7
ENSMUSG00000105371	0	0	0	0	0	0
ENSMUSG00000108469	0	0	0	0	0	0
ENSMUSG00000104129	0	0	0	0	0	0
ENSMUSG00000085222	2	0	0	0	0	0
ENSMUSG00000108464	0	0	0	2	0	0
ENSMUSG00000108467	0	0	0	0	0	0
ENSMUSG00000104127	0	0	0	0	0	0
ENSMUSG00000104120	6	2	0	0	1	0
ENSMUSG00000104123	3	2	0	0	4	0
ENSMUSG00000103664	0	0	0	0	0	0
ENSMUSG00000113998	42	30	48	38	37	38
ENSMUSG00000113991	1	1	0	2	1	1
ENSMUSG00000088046	0	0	0	0	0	0
ENSMUSG00000093958	0	0	0	0	0	0
ENSMUSG00000110879	0	0	0	0	0	0
ENSMUSG00000110878	0	0	0	0	2	0
ENSMUSG00000110876	0	0	0	0	0	0
ENSMUSG00000110874	2	4	0	8	8	6
ENSMUSG00000110873	0	0	0	3	0	0
ENSMUSG00000110872	0	0	0	1	0	0
ENSMUSG00000110870	0	0	0	0	0	0
ENSMUSG00000027225	0	0	0	0	0	0
ENSMUSG00000027224	6	10	11	7	4	0
ENSMUSG00000027229	0	0	0	0	0	0
ENSMUSG00000056145	108	86	111	27	29	48
ENSMUSG00000056148	0	0	3	8	4	0
ENSMUSG00000095437	0	1	0	0	0	0
ENSMUSG00000090404	2	2	0	0	2	0
ENSMUSG00000095434	0	0	0	1	0	0
ENSMUSG00000095433	0	0	0	0	0	0
ENSMUSG00000036959	295	253	320	143	208	232
ENSMUSG00000095430	0	0	0	0	0	0
ENSMUSG00000110833	0	0	0	0	0	0
ENSMUSG00000042510	60	44	52	45	59	48
ENSMUSG00000094528	0	0	0	0	0	0
ENSMUSG00000094529	0	0	0	0	0	0
ENSMUSG00000042514	0	0	2	2	0	0
ENSMUSG00000094522	0	0	0	0	0	0
ENSMUSG00000094520	2	2	2	2	1	1
ENSMUSG00000050677	18	23	24	7	19	33
ENSMUSG00000111830	0	0	0	0	0	0
ENSMUSG00000052642	0	0	0	0	0	0
ENSMUSG00000111834	0	0	0	0	0	0
ENSMUSG00000111836	2	0	0	3	1	2
ENSMUSG00000111837	0	0	0	0	0	0
ENSMUSG00000065935	0	2	1	0	3	1
ENSMUSG00000065937	0	0	0	0	0	0
ENSMUSG00000097999	23	17	28	16	17	23
ENSMUSG00000108637	0	0	0	0	0	0
ENSMUSG00000027913	0	0	0	0	0	0

ENSMUSG00000027912	0	0	0	0	0	0
ENSMUSG00000094389	0	0	0	0	0	0
ENSMUSG00000027919	0	0	0	0	0	0
ENSMUSG00000094384	0	0	0	0	0	0
ENSMUSG00000094385	0	0	0	0	0	0
ENSMUSG00000093170	0	0	0	0	1	0
ENSMUSG00000094383	0	0	0	0	0	0
ENSMUSG00000094380	2	0	0	0	0	0
ENSMUSG00000094381	0	0	0	0	0	0
ENSMUSG00000086253	0	0	0	0	0	0
ENSMUSG00000086250	0	0	0	0	0	0
ENSMUSG00000086257	0	0	0	0	0	0
ENSMUSG00000086256	5	0	8	0	0	0
ENSMUSG00000086255	0	0	0	0	0	0
ENSMUSG00000086254	0	0	0	0	0	0
ENSMUSG00000086758	0	0	0	0	0	0
ENSMUSG00000097878	0	0	0	0	0	0
ENSMUSG00000023216	15	10	0	0	4	6
ENSMUSG00000112565	0	0	0	0	0	0
ENSMUSG00000112564	0	0	0	0	0	0
ENSMUSG00000112563	3	2	0	1	0	0
ENSMUSG00000039342	0	0	0	0	2	0
ENSMUSG00000112561	0	0	0	0	0	0
ENSMUSG00000112560	0	0	0	0	0	0
ENSMUSG00000049427	0	0	0	0	0	1
ENSMUSG00000029517	0	0	0	3	0	0
ENSMUSG00000029516	116	148	152	58	87	97
ENSMUSG00000112569	0	0	0	0	0	0
ENSMUSG00000112568	0	0	0	0	0	0
ENSMUSG00000106199	0	0	0	0	0	0
ENSMUSG00000101379	0	0	0	0	0	0
ENSMUSG00000047034	0	0	0	0	0	0
ENSMUSG00000047039	0	3	0	0	0	0
ENSMUSG00000045883	0	0	0	0	0	0
ENSMUSG00000071636	4	8	6	16	16	10
ENSMUSG00000088203	0	0	0	0	0	0
ENSMUSG00000017943	4	3	0	6	0	5
ENSMUSG00000105956	0	0	0	0	0	0
ENSMUSG00000106227	3	0	0	0	0	0
ENSMUSG00000106226	0	0	0	0	0	0
ENSMUSG00000106223	0	0	0	0	0	2
ENSMUSG00000093833	0	0	0	0	0	0
ENSMUSG00000005947	20	7	57	10	8	12
ENSMUSG00000061602	0	0	0	0	0	0
ENSMUSG00000097127	3	1	6	1	1	2
ENSMUSG00000097120	5	4	0	0	4	0
ENSMUSG00000105952	0	0	0	0	0	0
ENSMUSG00000071185	0	0	0	3	0	0
ENSMUSG00000071181	33	25	45	21	17	48
ENSMUSG00000096830	0	0	0	0	0	0
ENSMUSG00000096831	0	0	0	0	0	0
ENSMUSG00000096834	0	0	0	0	0	0
ENSMUSG00000096835	2	1	1	0	0	1
ENSMUSG00000096839	0	0	2	0	0	0
ENSMUSG00000110050	0	0	0	0	0	0
ENSMUSG00000110052	0	2	3	0	0	0
ENSMUSG00000110054	0	0	0	0	0	0
ENSMUSG00000110056	0	0	0	0	0	0

ENSMUSG00000109475	0	0	0	0	0	0
ENSMUSG00000109477	0	0	0	0	0	0
ENSMUSG00000109476	0	1	1	0	0	0
ENSMUSG00000105750	22	36	28	7	13	11
ENSMUSG00000105751	0	0	0	0	0	0
ENSMUSG00000109473	0	0	0	4	3	0
ENSMUSG00000109478	0	0	0	0	0	0
ENSMUSG00000109253	1	0	0	0	0	0
ENSMUSG00000070837	6	0	0	0	5	6
ENSMUSG00000091312	0	0	0	0	0	0
ENSMUSG00000005057	47	42	92	22	34	14
ENSMUSG00000108705	0	0	0	0	0	0
ENSMUSG00000108707	0	0	0	0	0	0
ENSMUSG00000108708	0	0	0	0	0	0
ENSMUSG00000083377	0	0	2	0	0	0
ENSMUSG00000083376	0	0	0	0	0	0
ENSMUSG00000083375	10	23	13	18	13	13
ENSMUSG00000083379	0	0	0	0	0	0
ENSMUSG00000103170	4	4	0	0	0	0
ENSMUSG00000068407	0	0	0	0	0	0
ENSMUSG00000101010	0	0	0	0	0	0
ENSMUSG00000101017	0	0	0	0	0	0
ENSMUSG00000104024	0	0	0	0	0	0
ENSMUSG00000085230	0	0	0	0	0	0
ENSMUSG00000085232	4	0	10	3	0	3
ENSMUSG00000085235	0	0	0	0	0	0
ENSMUSG00000111534	0	0	0	0	0	0
ENSMUSG00000085239	0	0	0	0	0	0
ENSMUSG00000077146	0	0	0	0	1	0
ENSMUSG00000081053	0	0	0	0	0	0
ENSMUSG00000093928	0	0	0	0	0	0
ENSMUSG00000093923	0	0	0	0	0	0
ENSMUSG00000093922	0	0	0	0	0	0
ENSMUSG00000026630	19	17	23	5	3	6
ENSMUSG00000093927	0	0	0	0	0	0
ENSMUSG00000093926	0	0	0	0	0	0
ENSMUSG00000111530	4	0	2	0	0	0
ENSMUSG00000079259	4	0	0	0	0	0
ENSMUSG00000079258	0	0	6	0	0	0
ENSMUSG00000109573	0	0	0	0	0	0
ENSMUSG00000062760	29	14	27	18	8	10
ENSMUSG00000045306	0	0	0	0	0	0
ENSMUSG00000083997	0	0	0	0	0	0
ENSMUSG00000083994	0	0	0	0	0	0
ENSMUSG00000083993	1	3	1	5	2	3
ENSMUSG00000083991	0	0	0	0	0	0
ENSMUSG00000083999	0	0	0	0	0	0
ENSMUSG00000053251	0	0	0	0	0	0
ENSMUSG00000074469	0	0	0	0	0	0
ENSMUSG00000078129	8	11	7	0	8	13
ENSMUSG00000053268	0	0	0	0	0	0
ENSMUSG00000078127	5	0	5	0	6	6
ENSMUSG00000078123	0	0	0	0	0	0
ENSMUSG00000084167	0	0	0	0	0	0
ENSMUSG00000075036	4	1	2	1	4	3
ENSMUSG00000106632	0	0	0	0	0	0
ENSMUSG00000090152	0	0	0	0	3	0
ENSMUSG00000050808	18	10	27	0	0	0

ENSMUSG00000090364	0	0	0	0	0	4
ENSMUSG00000090365	0	0	0	0	0	0
ENSMUSG00000081269	0	0	0	0	0	0
ENSMUSG00000081268	0	0	0	0	0	0
ENSMUSG00000090362	0	0	0	0	0	0
ENSMUSG00000090363	0	10	15	15	27	20
ENSMUSG00000081263	2	0	0	0	0	0
ENSMUSG00000081262	6	9	7	11	8	6
ENSMUSG00000090369	22	17	20	3	7	9
ENSMUSG00000081265	8	2	4	0	5	1
ENSMUSG00000098595	1	2	1	1	1	1
ENSMUSG00000098594	0	0	0	0	0	0
ENSMUSG00000073119	0	0	0	4	0	0
ENSMUSG00000098591	0	0	0	0	0	0
ENSMUSG00000098590	0	0	0	0	0	0
ENSMUSG00000073116	3	0	0	0	0	0
ENSMUSG00000073117	4	0	0	0	0	0
ENSMUSG00000073110	0	0	0	0	0	0
ENSMUSG00000073111	0	0	0	0	0	0
ENSMUSG00000098599	0	0	0	0	0	0
ENSMUSG00000099972	0	0	0	0	0	0
ENSMUSG00000099973	0	0	0	0	0	0
ENSMUSG00000042655	0	0	0	0	0	0
ENSMUSG00000099971	0	0	0	0	0	0
ENSMUSG00000099976	0	0	0	0	0	0
ENSMUSG00000099977	0	0	0	0	0	0
ENSMUSG00000099975	0	0	0	0	0	0
ENSMUSG00000099978	0	0	0	0	0	0
ENSMUSG00000099979	1	0	0	0	0	0
ENSMUSG00000098621	0	0	0	0	1	0
ENSMUSG00000098620	13	15	18	9	8	0
ENSMUSG00000098622	0	0	0	0	0	0
ENSMUSG00000098627	0	0	0	0	0	0
ENSMUSG00000031397	6	7	4	0	0	0
ENSMUSG00000089057	0	0	0	0	0	0
ENSMUSG00000093446	0	0	0	2	0	2
ENSMUSG00000019429	0	0	0	0	0	0
ENSMUSG00000043015	150	145	236	185	107	193
ENSMUSG00000043013	0	0	0	0	0	0
ENSMUSG00000007021	5	5	6	9	0	6
ENSMUSG00000092483	2	0	0	0	0	0
ENSMUSG00000077344	0	0	0	0	0	0
ENSMUSG00000077342	0	0	0	0	0	0
ENSMUSG00000077348	0	0	0	0	0	0
ENSMUSG00000049741	14	22	7	5	11	8
ENSMUSG00000076436	0	0	3	0	0	0
ENSMUSG00000109162	0	3	0	0	0	0
ENSMUSG00000080843	0	0	0	0	0	0
ENSMUSG00000076439	4	5	0	6	2	5
ENSMUSG00000031645	0	0	0	0	0	0
ENSMUSG00000075179	0	0	0	0	0	0
ENSMUSG00000030510	4	7	10	0	5	11
ENSMUSG00000030519	47	28	21	47	34	31
ENSMUSG00000082101	0	0	0	6	0	0
ENSMUSG00000082106	0	0	0	0	0	0
ENSMUSG00000082104	0	0	0	0	0	0
ENSMUSG00000101426	0	0	0	0	0	0
ENSMUSG00000101427	0	0	0	0	0	0

ENSMUSG00000101424	0	0	0	0	0	0
ENSMUSG00000101425	0	0	0	0	0	0
ENSMUSG00000101422	0	0	0	0	0	0
ENSMUSG00000101423	0	0	0	0	0	0
ENSMUSG00000101420	0	0	0	0	0	0
ENSMUSG00000101421	0	0	0	0	0	0
ENSMUSG00000110247	2	0	0	0	2	0
ENSMUSG00000101428	0	0	0	0	0	0
ENSMUSG00000101429	0	0	0	0	0	0
ENSMUSG00000107862	0	0	0	0	0	0
ENSMUSG00000107864	0	0	0	0	0	0
ENSMUSG00000107866	0	0	0	0	0	0
ENSMUSG00000103370	0	0	0	0	0	0
ENSMUSG00000103371	0	0	0	0	0	0
ENSMUSG00000103372	0	0	0	0	0	0
ENSMUSG00000103375	2	0	0	0	3	0
ENSMUSG00000103376	0	0	0	0	0	0
ENSMUSG00000103377	15	10	14	0	0	12
ENSMUSG00000103378	0	0	0	0	2	4
ENSMUSG00000103379	0	0	0	0	0	0
ENSMUSG00000066383	0	0	0	0	0	0
ENSMUSG00000097539	0	0	0	0	0	0
ENSMUSG00000019312	0	0	0	0	0	0
ENSMUSG00000084628	1	4	4	1	5	1
ENSMUSG00000084629	0	0	0	0	0	0
ENSMUSG00000084620	1	0	0	0	0	0
ENSMUSG00000084621	0	1	0	0	0	0
ENSMUSG00000084627	0	0	0	0	0	0
ENSMUSG00000089217	0	0	0	0	0	0
ENSMUSG00000089210	0	0	0	0	0	0
ENSMUSG00000089211	0	0	0	0	0	0
ENSMUSG00000098435	0	0	0	0	0	0
ENSMUSG00000089218	0	0	0	0	0	0
ENSMUSG00000089219	0	0	0	0	0	0
ENSMUSG00000081556	0	0	0	0	0	0
ENSMUSG00000112349	1	3	1	0	4	3
ENSMUSG00000112348	0	0	0	0	0	0
ENSMUSG00000094258	0	0	0	0	6	0
ENSMUSG00000113118	0	0	0	1	0	0
ENSMUSG00000098436	0	0	0	0	0	0
ENSMUSG00000093602	0	0	0	0	0	0
ENSMUSG00000022321	14	5	0	4	0	0
ENSMUSG00000064685	0	0	0	0	0	0
ENSMUSG00000064686	0	0	0	0	0	0
ENSMUSG00000112344	0	0	0	0	0	0
ENSMUSG00000102429	0	0	0	0	0	0
ENSMUSG00000107514	0	3	0	0	0	0
ENSMUSG00000064688	0	0	0	0	0	0
ENSMUSG00000064689	0	0	0	0	0	0
ENSMUSG00000102421	0	0	0	0	0	0
ENSMUSG00000112342	1	0	0	2	0	0
ENSMUSG00000102420	3	0	0	0	0	0
ENSMUSG00000100489	0	0	0	0	0	0
ENSMUSG00000100488	0	0	0	0	0	0
ENSMUSG00000100487	0	0	0	0	0	0
ENSMUSG00000100485	0	0	0	0	0	0
ENSMUSG00000100484	0	0	0	0	0	0
ENSMUSG00000100483	0	0	0	0	0	0

ENSMUSG00000041323	0	5	3	2	0	0
ENSMUSG00000100480	7	19	9	4	10	12
ENSMUSG00000085577	0	0	0	0	0	0
ENSMUSG00000085576	4	25	13	0	0	6
ENSMUSG00000032572	2	0	0	0	0	0
ENSMUSG00000064536	0	0	1	1	1	0
ENSMUSG00000064532	0	0	0	0	0	0
ENSMUSG00000035557	0	0	0	0	0	0
ENSMUSG00000064530	0	0	0	0	2	0
ENSMUSG00000107139	0	0	0	0	0	0
ENSMUSG00000107135	0	0	0	0	0	0
ENSMUSG00000107132	0	0	0	0	0	0
ENSMUSG00000065489	1	1	0	1	0	1
ENSMUSG00000065488	0	0	0	1	0	0
ENSMUSG00000028008	0	0	0	0	0	0
ENSMUSG00000028009	0	0	0	0	0	0
ENSMUSG00000065481	0	0	0	0	0	0
ENSMUSG00000065480	0	0	0	0	0	0
ENSMUSG00000065483	0	0	0	0	0	0
ENSMUSG00000028001	0	0	0	0	0	0
ENSMUSG00000065485	2	0	3	0	0	3
ENSMUSG00000075478	0	0	0	0	1	0
ENSMUSG00000028004	0	0	0	0	0	0
ENSMUSG00000065486	0	0	0	0	0	0
ENSMUSG00000058884	1	0	0	0	0	0
ENSMUSG00000077708	0	0	0	0	0	0
ENSMUSG00000093520	3	0	0	0	0	0
ENSMUSG00000021566	0	0	0	0	0	0
ENSMUSG00000021565	4	8	0	0	0	0
ENSMUSG00000029755	0	0	0	0	0	0
ENSMUSG00000029754	2	0	0	0	0	2
ENSMUSG00000029757	0	6	8	0	0	5
ENSMUSG00000022445	0	0	0	0	2	0
ENSMUSG00000036602	0	0	0	0	0	0
ENSMUSG00000088628	0	1	0	0	2	0
ENSMUSG00000101996	0	0	0	0	0	0
ENSMUSG00000101997	0	0	0	0	0	0
ENSMUSG00000101994	0	0	0	0	0	0
ENSMUSG00000101992	0	0	0	0	0	0
ENSMUSG00000101993	0	0	0	0	0	0
ENSMUSG00000101990	0	0	0	0	0	0
ENSMUSG00000101991	0	0	0	0	0	0
ENSMUSG00000088996	0	0	0	0	0	0
ENSMUSG00000088999	0	0	0	0	0	0
ENSMUSG00000021696	14	6	4	6	10	9
ENSMUSG00000021697	7	8	9	0	13	14
ENSMUSG00000106423	0	1	0	0	0	0
ENSMUSG00000113768	0	0	0	0	0	0
ENSMUSG00000106420	0	0	0	1	0	0
ENSMUSG00000106425	0	0	0	0	0	0
ENSMUSG00000113763	1	0	0	0	1	0
ENSMUSG00000038180	36	36	22	31	14	16
ENSMUSG00000113761	0	0	0	0	0	0
ENSMUSG00000113767	0	0	0	5	0	0
ENSMUSG00000034607	4	0	4	0	6	0
ENSMUSG00000113765	0	0	0	0	0	0
ENSMUSG00000032023	0	0	0	0	0	0
ENSMUSG00000028575	8	0	9	8	0	7

ENSMUSG00000051638	0	0	0	0	0	0
ENSMUSG00000024056	43	56	47	4	23	47
ENSMUSG00000024050	380	358	369	308	296	405
ENSMUSG00000038292	0	0	0	0	0	0
ENSMUSG00000066170	30	25	48	20	20	26
ENSMUSG00000077376	0	0	0	0	0	0
ENSMUSG00000066176	0	0	0	0	0	0
ENSMUSG00000092152	0	0	0	0	0	0
ENSMUSG00000054379	0	0	0	0	0	0
ENSMUSG00000058499	0	0	0	0	0	0
ENSMUSG00000069818	0	3	0	0	0	0
ENSMUSG00000069816	0	0	0	0	0	0
ENSMUSG00000069814	12	9	11	12	10	20
ENSMUSG00000105192	0	0	0	0	0	0
ENSMUSG00000109672	0	0	0	0	0	0
ENSMUSG00000105190	0	0	0	0	0	0
ENSMUSG00000082188	0	0	0	0	0	0
ENSMUSG00000109677	0	0	0	0	0	0
ENSMUSG00000109676	0	0	0	0	0	0
ENSMUSG00000109675	4	10	12	5	0	0
ENSMUSG00000114231	0	0	0	0	0	0
ENSMUSG00000114233	0	0	0	0	0	0
ENSMUSG00000109678	0	0	0	0	0	0
ENSMUSG00000114234	0	0	0	0	0	1
ENSMUSG00000114237	0	0	0	0	0	0
ENSMUSG00000058491	0	0	0	0	0	0
ENSMUSG00000058717	0	0	0	0	0	0
ENSMUSG00000112963	0	0	0	0	0	0
ENSMUSG00000112962	0	0	0	0	0	0
ENSMUSG00000112961	0	0	0	0	0	1
ENSMUSG00000112960	3	3	6	0	2	1
ENSMUSG00000112967	17	22	4	4	8	1
ENSMUSG00000112966	0	0	0	0	2	0
ENSMUSG00000112965	0	0	0	0	0	0
ENSMUSG00000112969	5	1	3	4	2	5
ENSMUSG00000112968	0	0	0	0	0	0
ENSMUSG00000067229	0	0	0	0	0	0
ENSMUSG00000110448	0	4	0	0	0	0
ENSMUSG00000110449	0	0	0	0	0	0
ENSMUSG00000110443	2	0	1	0	0	0
ENSMUSG00000110447	0	0	0	0	0	0
ENSMUSG00000110445	0	0	0	0	0	0
ENSMUSG00000050272	0	0	0	0	0	0
ENSMUSG00000050276	0	0	0	0	0	0
ENSMUSG00000060794	0	0	0	0	0	0
ENSMUSG00000096508	0	0	0	0	0	0
ENSMUSG00000096507	0	0	0	1	0	0
ENSMUSG00000114692	0	0	0	0	0	0
ENSMUSG00000029195	13	27	9	14	9	13
ENSMUSG00000060240	50	53	43	33	48	55
ENSMUSG00000080330	0	0	0	0	0	0
ENSMUSG00000080331	0	0	0	0	0	0
ENSMUSG00000080336	0	0	2	0	0	0
ENSMUSG00000060245	0	0	0	0	0	0
ENSMUSG00000114695	0	0	0	0	0	0
ENSMUSG00000056849	1	1	3	0	1	1
ENSMUSG00000104099	0	0	0	0	0	0
ENSMUSG00000087225	0	0	0	0	0	0

ENSMUSG00000045148	0	0	0	0	0	0
ENSMUSG00000064943	2	0	0	0	0	0
ENSMUSG00000113569	19	12	19	12	11	6
ENSMUSG00000095089	0	0	0	0	0	0
ENSMUSG00000095082	0	0	0	0	0	0
ENSMUSG00000111310	1	0	0	0	0	0
ENSMUSG00000095081	0	0	0	0	0	0
ENSMUSG00000111315	0	0	0	0	0	0
ENSMUSG00000111317	0	0	0	0	0	0
ENSMUSG00000095085	0	0	0	0	0	0
ENSMUSG00000081069	0	0	0	0	0	0
ENSMUSG00000026963	0	0	0	0	0	0
ENSMUSG00000026961	0	0	0	0	0	0
ENSMUSG00000112081	0	3	0	0	0	0
ENSMUSG00000081061	0	0	0	0	0	0
ENSMUSG00000081060	0	0	0	0	0	0
ENSMUSG00000081063	0	0	0	0	0	0
ENSMUSG00000081062	0	2	0	0	0	2
ENSMUSG00000081065	0	0	0	0	0	0
ENSMUSG00000081064	0	0	0	0	0	0
ENSMUSG00000081066	0	0	0	0	0	0
ENSMUSG00000053729	0	0	0	0	0	0
ENSMUSG00000112083	0	0	0	0	0	0
ENSMUSG00000087226	0	0	0	0	0	0
ENSMUSG00000113561	0	4	0	0	0	0
ENSMUSG00000053720	0	0	0	0	0	0
ENSMUSG00000112085	0	0	0	0	0	0
ENSMUSG00000022044	2	6	7	0	0	7
ENSMUSG00000042496	387	404	478	320	447	450
ENSMUSG00000042498	0	8	12	2	4	3
ENSMUSG00000112088	0	0	0	0	0	0
ENSMUSG00000090618	1	0	1	1	0	1
ENSMUSG00000087220	0	0	0	0	0	0
ENSMUSG00000023978	4	0	6	7	13	9
ENSMUSG00000048186	190	261	152	283	165	339
ENSMUSG00000077107	0	1	0	0	2	0
ENSMUSG00000077104	0	0	0	0	0	0
ENSMUSG00000112152	0	0	0	0	0	0
ENSMUSG00000055761	10	7	13	0	0	0
ENSMUSG00000004933	39	50	58	42	36	48
ENSMUSG00000112156	0	0	0	0	0	0
ENSMUSG00000112157	0	0	0	0	0	0
ENSMUSG00000112154	0	0	0	0	0	0
ENSMUSG00000112155	0	0	0	0	0	0
ENSMUSG00000105538	0	0	0	0	0	0
ENSMUSG00000086592	0	0	0	0	0	0
ENSMUSG00000112159	0	0	0	0	0	0
ENSMUSG00000086595	0	0	0	0	0	0
ENSMUSG00000086596	42	9	10	20	22	16
ENSMUSG00000105539	0	0	0	0	0	0
ENSMUSG00000046550	8	4	10	0	0	4
ENSMUSG00000049583	2	0	3	2	3	0
ENSMUSG00000086048	0	0	0	0	0	0
ENSMUSG00000072553	0	0	0	0	0	0
ENSMUSG00000092616	0	0	0	0	0	0
ENSMUSG00000092617	2	5	4	2	3	4
ENSMUSG00000092614	0	0	0	0	0	0
ENSMUSG00000092615	0	0	0	0	0	0

ENSMUSG00000092612	0	0	0	0	0	0
ENSMUSG00000092610	0	0	0	0	0	0
ENSMUSG00000092611	0	0	0	0	0	0
ENSMUSG00000109291	0	0	5	0	0	0
ENSMUSG00000092618	0	0	0	0	0	0
ENSMUSG00000094879	0	0	0	0	0	0
ENSMUSG00000096977	5	9	9	4	5	5
ENSMUSG00000098910	0	0	0	0	0	0
ENSMUSG00000096973	0	0	0	0	0	0
ENSMUSG00000098916	0	0	0	0	0	0
ENSMUSG00000082740	4	2	2	8	0	4
ENSMUSG00000113201	0	0	0	0	0	0
ENSMUSG00000113205	0	0	0	0	0	0
ENSMUSG00000113207	3	2	3	2	2	3
ENSMUSG00000113206	0	0	0	0	0	0
ENSMUSG00000113208	0	0	0	0	0	0
ENSMUSG00000045928	0	0	0	0	0	0
ENSMUSG00000045929	7	0	5	0	5	6
ENSMUSG00000095266	0	0	0	0	0	0
ENSMUSG00000095267	0	0	0	0	0	0
ENSMUSG00000083637	0	0	0	0	0	0
ENSMUSG00000101416	0	0	0	0	0	0
ENSMUSG00000099107	0	0	0	0	0	0
ENSMUSG00000047207	0	0	0	0	0	0
ENSMUSG00000047203	0	0	0	0	0	0
ENSMUSG00000071398	18	4	14	8	14	8
ENSMUSG00000059842	46	58	66	52	72	67
ENSMUSG00000071392	0	5	0	10	3	3
ENSMUSG00000057855	0	0	0	0	0	0
ENSMUSG00000063590	0	0	0	0	0	0
ENSMUSG00000025081	0	0	0	0	0	0
ENSMUSG00000025086	331	370	309	351	356	351
ENSMUSG00000075160	0	0	0	0	0	0
ENSMUSG00000065473	0	0	0	0	0	0
ENSMUSG00000093214	0	0	0	0	0	0
ENSMUSG00000084351	0	0	0	0	0	0
ENSMUSG00000084352	0	0	0	0	0	0
ENSMUSG00000084355	0	0	0	0	0	0
ENSMUSG00000084356	0	0	0	0	0	0
ENSMUSG00000084358	0	0	0	0	0	0
ENSMUSG00000084359	0	0	0	0	0	0
ENSMUSG00000093216	0	0	0	0	0	0
ENSMUSG00000075168	0	0	0	0	0	2
ENSMUSG00000025557	0	0	0	0	0	0
ENSMUSG00000099349	2	0	0	1	0	0
ENSMUSG00000099348	0	0	0	0	0	0
ENSMUSG00000101511	3	1	2	5	0	5
ENSMUSG00000099341	0	0	0	0	0	0
ENSMUSG00000099340	0	0	0	0	0	0
ENSMUSG00000099344	0	0	0	0	0	0
ENSMUSG00000099347	0	0	0	0	0	0
ENSMUSG00000099346	0	0	0	0	0	0
ENSMUSG00000030994	92	90	79	239	80	93
ENSMUSG00000079192	5	5	3	0	0	1
ENSMUSG00000068231	0	0	0	0	0	0
ENSMUSG00000068232	0	0	0	3	2	0
ENSMUSG00000068234	0	0	0	0	0	0
ENSMUSG00000080071	0	0	0	0	0	0

ENSMUSG00000080073	0	0	0	0	0	0
ENSMUSG00000080072	0	0	0	0	1	0
ENSMUSG00000085008	2	0	0	5	0	0
ENSMUSG00000085007	0	0	0	0	0	2
ENSMUSG00000080078	153	165	172	157	157	223
ENSMUSG00000085002	0	0	0	1	0	0
ENSMUSG00000085000	0	0	0	0	0	0
ENSMUSG00000113401	0	0	0	0	0	0
ENSMUSG00000113490	0	0	0	0	0	0
ENSMUSG00000096390	1	0	0	0	0	0
ENSMUSG00000026413	0	0	2	0	0	4
ENSMUSG00000099836	0	0	0	0	0	0
ENSMUSG00000099834	0	0	0	0	0	0
ENSMUSG00000099833	0	0	0	0	0	0
ENSMUSG00000099832	0	0	0	0	0	0
ENSMUSG00000099831	0	0	0	0	0	0
ENSMUSG00000099839	0	0	0	0	0	0
ENSMUSG00000111637	0	0	0	0	0	0
ENSMUSG00000111635	0	0	0	0	0	0
ENSMUSG00000098122	0	0	0	0	0	0
ENSMUSG00000098126	0	0	0	0	0	0
ENSMUSG00000098129	0	0	0	0	0	0
ENSMUSG00000073094	0	0	0	0	0	0
ENSMUSG00000111913	0	2	2	0	0	0
ENSMUSG00000072944	0	0	0	0	2	0
ENSMUSG00000072945	0	0	0	0	0	0
ENSMUSG00000103929	0	0	0	0	0	0
ENSMUSG00000097918	0	0	0	0	0	0
ENSMUSG00000103924	0	0	0	0	0	0
ENSMUSG00000103927	0	0	0	0	0	0
ENSMUSG00000103921	36	30	21	20	18	32
ENSMUSG00000103920	0	0	0	0	0	3
ENSMUSG00000103923	0	2	0	0	0	0
ENSMUSG00000038968	0	0	0	0	0	0
ENSMUSG00000114030	0	0	0	0	0	0
ENSMUSG00000038963	0	0	0	0	0	2
ENSMUSG00000100438	0	0	0	0	0	0
ENSMUSG00000056699	0	0	0	2	0	4
ENSMUSG00000095363	0	0	0	0	0	0
ENSMUSG00000088156	0	0	0	0	0	0
ENSMUSG00000035829	106	122	131	9	159	130
ENSMUSG00000111091	0	0	0	0	0	0
ENSMUSG00000082170	0	2	0	0	1	2
ENSMUSG00000089804	2	0	0	0	7	0
ENSMUSG00000089808	0	0	2	1	0	0
ENSMUSG00000111094	23	19	26	25	16	24
ENSMUSG00000031562	37	44	42	43	48	46
ENSMUSG00000111814	0	0	0	0	0	0
ENSMUSG00000095368	0	0	0	0	0	0
ENSMUSG00000108203	0	0	0	0	0	0
ENSMUSG00000081183	0	0	0	0	0	0
ENSMUSG00000108363	0	0	0	0	0	0
ENSMUSG00000108838	0	3	0	0	0	3
ENSMUSG00000101388	0	0	0	0	0	0
ENSMUSG00000101383	0	0	0	0	0	0
ENSMUSG00000108202	3	0	0	0	0	0
ENSMUSG00000108830	24	27	22	11	23	31
ENSMUSG00000101380	0	0	0	0	0	0

ENSMUSG00000108836	2	0	0	0	0	0
ENSMUSG00000108837	0	0	0	0	0	0
ENSMUSG00000108834	0	0	0	0	0	0
ENSMUSG00000108835	0	0	0	0	0	0
ENSMUSG00000112629	0	0	0	0	0	0
ENSMUSG00000043226	0	0	0	0	0	0
ENSMUSG00000112676	0	0	0	0	0	0
ENSMUSG00000100380	0	0	0	0	0	0
ENSMUSG00000100381	0	0	0	0	0	0
ENSMUSG00000081189	2	4	0	2	6	6
ENSMUSG00000088048	0	0	0	0	0	0
ENSMUSG00000100385	0	0	0	0	0	0
ENSMUSG00000087098	0	0	0	0	0	0
ENSMUSG00000087099	0	0	0	0	0	0
ENSMUSG00000100388	0	0	0	0	0	0
ENSMUSG00000108361	0	0	0	0	0	0
ENSMUSG00000087094	0	0	0	0	0	4
ENSMUSG00000088047	0	0	0	0	0	0
ENSMUSG00000088040	0	0	0	0	0	0
ENSMUSG00000088041	0	0	0	0	0	0
ENSMUSG00000087090	4	4	0	8	8	2
ENSMUSG00000088043	0	0	0	0	0	0
ENSMUSG00000101917	0	0	0	0	0	0
ENSMUSG00000085836	0	0	0	0	0	0
ENSMUSG00000085837	0	0	0	0	0	4
ENSMUSG00000085834	0	0	0	0	0	0
ENSMUSG00000085835	0	0	0	0	0	0
ENSMUSG00000085833	0	0	0	0	0	0
ENSMUSG00000085830	8	8	5	0	4	6
ENSMUSG00000085831	0	0	0	0	0	0
ENSMUSG00000021795	0	0	0	0	0	0
ENSMUSG00000107872	0	3	1	0	0	0
ENSMUSG00000087540	0	0	0	0	5	0
ENSMUSG00000087542	4	2	2	1	0	0
ENSMUSG00000087544	0	5	0	0	0	0
ENSMUSG00000087545	0	0	0	0	0	4
ENSMUSG00000101654	0	0	0	0	0	0
ENSMUSG00000101657	0	0	0	0	0	0
ENSMUSG00000101650	0	0	0	0	0	0
ENSMUSG00000101653	0	0	0	0	0	0
ENSMUSG00000101652	0	0	0	0	0	0
ENSMUSG00000101659	0	0	0	0	0	0
ENSMUSG00000021797	0	0	0	0	0	0
ENSMUSG00000036109	94	119	102	53	74	64
ENSMUSG00000100524	0	0	0	0	0	0
ENSMUSG00000084919	0	0	0	0	0	0
ENSMUSG00000100526	0	0	0	0	0	0
ENSMUSG00000100527	0	0	0	0	0	0
ENSMUSG00000100520	0	0	0	0	0	0
ENSMUSG00000100521	0	0	0	0	0	0
ENSMUSG00000100523	0	0	0	0	0	0
ENSMUSG00000100528	0	0	0	0	0	0
ENSMUSG00000022286	100	91	4	197	189	174
ENSMUSG00000001076	0	0	0	0	0	0
ENSMUSG00000089398	0	0	0	0	0	0
ENSMUSG00000089393	0	0	0	0	0	0
ENSMUSG00000089391	0	0	0	0	0	0
ENSMUSG00000089390	0	1	1	0	0	1

ENSMUSG00000089397	0	0	0	0	0	0
ENSMUSG00000089395	0	0	0	0	0	0
ENSMUSG00000089394	0	0	0	0	0	0
ENSMUSG00000089025	0	0	0	0	0	0
ENSMUSG00000084015	0	0	0	0	0	0
ENSMUSG00000084014	0	0	0	0	0	0
ENSMUSG00000089021	0	0	0	0	0	0
ENSMUSG00000084012	0	0	0	0	0	0
ENSMUSG00000084011	6	3	14	3	7	0
ENSMUSG00000018451	14	19	21	7	4	5
ENSMUSG00000095864	0	0	0	0	0	0
ENSMUSG00000095867	0	0	0	0	0	0
ENSMUSG00000095860	0	0	0	0	0	0
ENSMUSG00000084019	0	0	0	0	0	0
ENSMUSG00000018459	30	8	32	7	12	14
ENSMUSG00000065569	0	0	0	0	0	0
ENSMUSG00000065565	0	0	0	0	1	0
ENSMUSG00000112735	0	0	0	0	0	0
ENSMUSG00000018983	44	53	69	18	27	27
ENSMUSG00000028635	0	0	0	0	0	0
ENSMUSG00000109228	0	0	0	0	2	0
ENSMUSG00000070546	0	0	0	0	0	2
ENSMUSG00000112310	0	0	0	0	1	0
ENSMUSG00000112312	0	0	0	0	0	0
ENSMUSG00000071713	133	125	289	61	67	74
ENSMUSG00000039174	0	0	0	0	0	0
ENSMUSG00000112315	0	0	0	0	0	0
ENSMUSG00000112316	0	0	0	0	0	0
ENSMUSG00000102679	0	0	0	0	0	0
ENSMUSG00000112318	0	0	0	0	0	0
ENSMUSG00000035456	0	0	0	0	15	5
ENSMUSG00000035455	81	57	9	8	31	59
ENSMUSG00000035454	0	0	0	0	0	0
ENSMUSG00000102672	62	50	95	51	71	55
ENSMUSG00000035451	0	0	0	0	0	0
ENSMUSG00000095587	0	0	0	0	0	0
ENSMUSG00000062511	0	0	0	0	0	0
ENSMUSG00000109220	0	0	0	0	0	0
ENSMUSG00000110228	0	0	0	0	0	0
ENSMUSG00000110222	0	0	0	0	0	0
ENSMUSG00000110220	0	0	0	0	0	0
ENSMUSG00000036815	1	0	4	2	3	0
ENSMUSG00000110226	0	0	0	0	0	0
ENSMUSG00000036813	0	0	0	0	0	3
ENSMUSG00000110224	0	2	0	2	0	2
ENSMUSG00000110225	1	0	0	1	0	0
ENSMUSG00000114417	0	0	0	0	0	0
ENSMUSG00000094805	0	0	0	0	0	0
ENSMUSG00000094804	0	0	0	0	0	0
ENSMUSG00000094807	0	1	0	0	1	1
ENSMUSG00000094806	0	0	2	0	0	2
ENSMUSG00000105549	4	4	4	4	2	0
ENSMUSG00000105548	0	0	0	0	0	0
ENSMUSG00000094803	0	0	0	0	0	0
ENSMUSG00000094802	0	0	0	0	0	0
ENSMUSG00000105546	1	0	0	0	0	1
ENSMUSG00000021718	0	0	0	0	0	0
ENSMUSG00000088819	0	0	0	0	0	0

ENSMUSG00000088818	0	0	0	0	0	0
ENSMUSG00000113049	0	0	0	0	0	0
ENSMUSG00000088813	0	0	0	0	0	0
ENSMUSG00000088812	0	0	0	0	0	0
ENSMUSG00000088811	0	0	0	0	0	0
ENSMUSG00000113044	0	0	0	0	0	0
ENSMUSG00000113043	0	0	0	0	0	0
ENSMUSG00000113042	0	0	0	0	2	0
ENSMUSG00000113040	0	0	0	0	0	0
ENSMUSG00000065261	0	0	0	0	0	0
ENSMUSG00000111578	0	1	0	0	0	0
ENSMUSG00000111570	0	0	0	0	0	0
ENSMUSG00000111573	0	0	0	0	0	0
ENSMUSG00000111572	0	0	0	0	0	0
ENSMUSG00000111575	0	0	0	0	0	0
ENSMUSG00000111574	0	0	0	0	0	0
ENSMUSG00000111577	0	2	0	0	0	0
ENSMUSG00000111576	0	0	0	0	0	0
ENSMUSG00000029275	0	0	0	0	0	0
ENSMUSG00000029279	72	60	48	41	28	35
ENSMUSG00000113599	0	0	0	0	0	0
ENSMUSG00000113598	12	8	0	0	6	14
ENSMUSG00000113590	0	0	0	0	0	0
ENSMUSG00000113592	0	0	0	0	0	0
ENSMUSG00000113595	0	0	0	0	0	0
ENSMUSG00000050844	0	0	0	0	0	0
ENSMUSG00000113597	0	0	0	0	0	1
ENSMUSG00000089203	0	0	0	0	0	0
ENSMUSG00000111683	0	0	0	0	0	0
ENSMUSG00000109823	0	0	0	0	0	0
ENSMUSG00000111681	43	41	57	29	34	30
ENSMUSG00000111680	0	0	0	0	0	0
ENSMUSG00000111687	0	0	0	0	0	0
ENSMUSG00000089207	0	0	0	0	0	0
ENSMUSG00000109824	0	0	0	1	0	0
ENSMUSG00000109825	0	0	0	0	0	0
ENSMUSG00000114060	0	0	0	0	0	0
ENSMUSG00000111689	0	0	0	0	0	0
ENSMUSG00000109829	33	30	37	30	20	66
ENSMUSG00000114064	0	0	0	0	0	0
ENSMUSG00000106568	5	0	8	3	2	1
ENSMUSG00000114066	0	0	0	0	0	0
ENSMUSG00000011118	2	2	4	0	0	0
ENSMUSG00000097575	0	0	0	0	4	0
ENSMUSG00000097571	95	144	107	77	81	114
ENSMUSG00000097572	17	10	12	8	10	12
ENSMUSG00000104634	0	2	5	2	4	4
ENSMUSG00000108194	0	0	0	0	0	0
ENSMUSG00000104636	0	0	0	0	0	0
ENSMUSG00000104637	2	0	0	0	0	0
ENSMUSG00000108191	0	0	0	4	5	0
ENSMUSG00000097579	0	0	7	0	5	0
ENSMUSG00000108193	0	0	0	0	0	0
ENSMUSG00000108192	0	0	0	0	0	0
ENSMUSG00000044222	0	0	0	0	0	0
ENSMUSG00000114575	3	9	7	3	10	8
ENSMUSG00000110648	1	0	0	1	1	1
ENSMUSG00000112888	0	0	0	0	0	0

ENSMUSG00000074796	10	16	4	0	3	4
ENSMUSG00000112885	0	0	0	0	0	0
ENSMUSG00000112886	0	0	0	0	0	0
ENSMUSG00000027718	0	0	0	0	2	0
ENSMUSG00000083898	0	0	0	0	0	0
ENSMUSG00000083892	0	0	0	3	7	0
ENSMUSG00000083893	0	0	0	0	0	0
ENSMUSG00000083890	0	0	0	0	0	0
ENSMUSG00000083896	0	0	0	0	0	0
ENSMUSG00000083895	0	0	0	0	0	0
ENSMUSG00000100179	0	0	0	0	0	0
ENSMUSG00000089510	0	0	0	0	0	0
ENSMUSG00000110769	0	0	0	0	0	0
ENSMUSG00000110767	0	0	0	0	0	0
ENSMUSG00000110766	0	0	0	0	0	0
ENSMUSG00000110762	0	0	0	0	0	0
ENSMUSG00000110761	0	0	0	2	0	3
ENSMUSG00000044429	0	0	0	0	0	0
ENSMUSG00000042861	0	0	0	0	0	0
ENSMUSG00000042863	0	0	0	0	0	0
ENSMUSG00000107593	0	2	2	3	8	3
ENSMUSG00000042869	0	0	0	0	0	0
ENSMUSG00000109248	0	0	0	0	0	0
ENSMUSG00000046372	0	0	2	4	7	0
ENSMUSG00000056656	6	0	3	0	0	0
ENSMUSG00000044227	0	0	0	0	0	0
ENSMUSG00000109249	3	0	0	0	2	0
ENSMUSG00000053024	112	86	124	76	86	94
ENSMUSG00000109246	0	0	0	0	0	0
ENSMUSG00000103498	0	0	0	0	0	0
ENSMUSG00000074832	0	1	0	1	0	0
ENSMUSG00000074830	0	0	0	0	0	0
ENSMUSG00000074837	0	0	0	0	0	0
ENSMUSG00000090138	0	0	0	0	0	0
ENSMUSG00000095328	0	0	0	0	0	0
ENSMUSG00000095327	0	0	0	0	0	0
ENSMUSG00000095324	0	0	0	0	0	0
ENSMUSG00000090131	0	0	0	0	0	0
ENSMUSG00000095322	0	0	0	0	0	0
ENSMUSG00000095321	0	0	0	0	0	0
ENSMUSG00000113861	4	3	1	2	6	3
ENSMUSG00000113860	0	0	0	0	0	0
ENSMUSG00000098323	0	0	0	0	0	0
ENSMUSG00000098326	0	0	0	0	0	0
ENSMUSG00000113866	5	4	2	4	2	8
ENSMUSG00000092759	0	0	0	0	0	0
ENSMUSG00000047428	8	4	4	0	6	10
ENSMUSG00000053684	45	67	55	14	27	43
ENSMUSG00000093449	0	0	0	0	0	0
ENSMUSG00000094498	0	0	0	0	0	0
ENSMUSG00000093444	0	0	0	0	0	0
ENSMUSG00000092753	0	0	0	0	0	0
ENSMUSG00000052372	13	12	9	0	2	0
ENSMUSG00000022833	149	185	151	178	201	267
ENSMUSG00000098493	0	0	0	0	0	0
ENSMUSG00000098494	0	0	0	0	0	0
ENSMUSG00000053979	0	0	0	0	0	0
ENSMUSG00000109242	0	0	2	0	0	0

ENSMUSG00000099420	1	1	1	1	0	1
ENSMUSG00000075377	0	0	0	0	0	0
ENSMUSG00000053977	14	6	15	14	11	8
ENSMUSG00000094148	0	0	0	0	0	0
ENSMUSG00000094149	0	0	0	0	0	0
ENSMUSG00000094144	0	0	0	0	0	0
ENSMUSG00000094147	0	0	0	0	0	0
ENSMUSG00000025154	277	63	381	127	218	231
ENSMUSG00000086947	6	11	4	7	5	6
ENSMUSG00000086948	0	0	0	0	0	0
ENSMUSG00000100170	0	0	0	0	0	0
ENSMUSG00000081638	0	0	0	0	0	0
ENSMUSG00000090790	0	0	0	0	0	0
ENSMUSG00000090793	0	0	0	0	0	0
ENSMUSG00000052087	59	51	61	19	36	37
ENSMUSG00000090794	0	0	0	0	0	0
ENSMUSG00000090796	0	0	0	0	0	0
ENSMUSG00000081630	0	0	0	0	0	0
ENSMUSG00000090798	0	0	0	0	0	0
ENSMUSG00000081632	0	0	0	0	0	0
ENSMUSG00000081635	2	0	0	0	0	0
ENSMUSG00000094187	0	0	0	0	0	0
ENSMUSG00000092793	0	0	0	0	0	0
ENSMUSG00000092792	0	0	0	0	0	1
ENSMUSG00000092796	1	1	0	0	1	1
ENSMUSG00000112016	0	0	0	0	0	0
ENSMUSG00000077713	0	0	0	0	0	0
ENSMUSG00000077710	0	0	0	0	0	0
ENSMUSG00000095933	0	0	0	0	0	0
ENSMUSG00000077719	0	0	0	0	0	0
ENSMUSG00000112057	0	0	0	0	0	0
ENSMUSG00000112056	1	0	2	0	2	0
ENSMUSG00000112055	0	0	0	0	0	0
ENSMUSG00000112054	0	0	0	0	0	0
ENSMUSG00000112053	2	6	0	4	4	0
ENSMUSG00000112051	4	4	3	3	3	2
ENSMUSG00000112050	0	0	0	0	0	0
ENSMUSG00000090863	2	0	2	2	0	0
ENSMUSG00000044994	0	0	0	0	4	0
ENSMUSG00000090865	0	0	1	0	0	0
ENSMUSG00000090864	0	0	0	0	0	0
ENSMUSG00000048398	0	0	0	0	0	0
ENSMUSG00000048399	0	0	0	0	0	0
ENSMUSG00000100470	0	0	0	0	0	0
ENSMUSG00000092425	0	0	0	0	0	0
ENSMUSG00000092427	0	0	0	0	0	0
ENSMUSG00000092426	0	0	0	0	0	0
ENSMUSG00000092422	0	0	0	0	0	0
ENSMUSG00000092429	0	0	0	0	0	0
ENSMUSG00000092428	2	0	3	0	0	1
ENSMUSG00000101484	0	0	0	0	0	0
ENSMUSG00000101486	0	0	0	0	0	0
ENSMUSG00000086366	0	0	0	0	0	0
ENSMUSG00000030768	419	424	557	237	353	360
ENSMUSG00000030769	0	0	0	0	7	0
ENSMUSG00000086363	41	32	41	11	31	25
ENSMUSG00000101480	0	0	0	0	0	0
ENSMUSG00000043429	0	0	0	0	0	0

ENSMUSG00000072295	0	0	0	0	0	0
ENSMUSG00000101481	0	3	0	6	0	0
ENSMUSG00000076042	0	0	0	0	0	0
ENSMUSG00000072259	0	1	0	3	0	0
ENSMUSG00000101489	0	0	0	0	0	0
ENSMUSG00000108179	2	0	4	0	0	0
ENSMUSG00000087702	0	0	0	0	0	0
ENSMUSG00000072624	0	0	0	0	0	0
ENSMUSG00000087708	0	0	0	0	0	0
ENSMUSG00000083462	0	1	1	1	1	0
ENSMUSG00000083460	1	3	4	2	2	5
ENSMUSG00000083467	0	0	0	0	0	0
ENSMUSG00000083466	0	0	0	0	0	0
ENSMUSG00000083469	0	1	0	0	0	2
ENSMUSG00000099503	0	0	0	0	0	0
ENSMUSG00000043522	0	0	0	0	0	0
ENSMUSG00000079542	0	0	0	0	0	0
ENSMUSG00000031966	0	0	0	3	0	0
ENSMUSG00000064016	0	0	0	0	0	0
ENSMUSG00000064010	0	0	0	0	0	0
ENSMUSG00000102184	0	0	4	0	0	3
ENSMUSG00000107418	0	0	0	2	0	0
ENSMUSG00000107415	0	0	0	0	0	1
ENSMUSG00000107417	0	2	5	4	2	1
ENSMUSG00000107416	0	0	0	0	0	0
ENSMUSG00000107410	0	0	0	0	0	0
ENSMUSG00000107413	1	0	0	1	0	1
ENSMUSG00000107412	0	0	0	0	0	0
ENSMUSG00000000794	156	202	309	27	9	94
ENSMUSG00000065768	0	0	0	0	0	0
ENSMUSG00000000791	0	0	0	0	0	0
ENSMUSG00000000792	0	0	6	0	5	6
ENSMUSG00000065760	0	0	0	0	0	0
ENSMUSG00000065763	0	0	0	0	0	0
ENSMUSG00000085700	0	0	0	0	0	0
ENSMUSG00000085701	0	0	0	0	0	0
ENSMUSG00000085706	0	0	0	0	0	0
ENSMUSG00000085707	0	0	0	0	0	0
ENSMUSG00000107123	0	0	0	0	2	0
ENSMUSG00000035576	3	0	0	0	0	4
ENSMUSG00000001228	114	122	123	39	66	168
ENSMUSG00000001225	34	32	6	0	2	3
ENSMUSG00000107127	0	0	0	0	0	0
ENSMUSG00000107126	0	0	0	0	0	0
ENSMUSG00000069721	0	0	0	2	0	0
ENSMUSG00000068068	0	0	0	0	0	0
ENSMUSG00000094048	0	0	0	0	0	0
ENSMUSG00000068067	0	0	0	0	0	0
ENSMUSG00000086338	0	0	0	0	0	0
ENSMUSG00000099505	0	0	0	0	0	0
ENSMUSG00000084181	0	0	0	0	0	0
ENSMUSG00000074199	0	0	3	0	0	0
ENSMUSG00000078639	0	0	0	0	0	0
ENSMUSG00000074195	0	0	0	0	0	0
ENSMUSG00000074196	0	0	0	0	0	0
ENSMUSG00000078632	0	0	0	0	0	0
ENSMUSG00000062757	0	0	0	0	0	0
ENSMUSG00000062751	0	0	0	0	0	0

ENSMUSG00000105113	0	0	0	0	0	0
ENSMUSG00000086321	0	0	0	0	0	0
ENSMUSG00000060924	68	57	93	51	43	95
ENSMUSG00000105111	2	0	0	2	0	0
ENSMUSG00000102321	0	0	0	0	2	0
ENSMUSG00000032318	0	0	0	0	0	0
ENSMUSG00000032310	0	0	0	0	0	0
ENSMUSG00000090892	0	0	0	0	0	0
ENSMUSG00000094424	0	0	0	0	0	0
ENSMUSG00000062282	0	0	0	0	0	0
ENSMUSG00000060105	0	0	0	0	0	2
ENSMUSG00000113657	0	0	0	0	0	0
ENSMUSG00000084180	0	0	0	0	0	0
ENSMUSG00000090891	0	6	4	0	0	6
ENSMUSG00000103882	50	51	49	21	39	60
ENSMUSG00000103880	3	3	8	0	0	1
ENSMUSG00000103881	0	3	0	0	0	0
ENSMUSG00000103886	0	0	0	1	0	0
ENSMUSG00000103889	3	0	0	0	0	0
ENSMUSG00000063157	0	0	0	0	2	0
ENSMUSG00000072780	0	0	0	0	0	0
ENSMUSG00000066500	0	0	0	0	0	0
ENSMUSG00000031150	21	21	21	0	21	23
ENSMUSG00000031155	47	46	66	52	41	54
ENSMUSG00000082327	0	0	0	0	0	0
ENSMUSG00000021572	53	38	52	90	74	63
ENSMUSG00000047828	0	0	0	0	0	0
ENSMUSG00000105839	0	1	0	0	0	2
ENSMUSG00000105838	0	0	0	0	0	0
ENSMUSG00000067679	0	0	0	0	0	0
ENSMUSG00000105831	0	0	0	0	0	0
ENSMUSG00000102822	1	0	0	0	0	0
ENSMUSG00000102821	0	0	2	0	0	0
ENSMUSG00000102826	0	0	0	0	0	0
ENSMUSG00000102825	0	0	0	2	0	0
ENSMUSG00000105836	5	7	6	9	7	10
ENSMUSG00000113651	5	2	0	1	3	1
ENSMUSG00000108978	0	0	0	0	1	0
ENSMUSG00000108977	0	0	0	0	0	0
ENSMUSG00000108976	0	0	0	5	3	3
ENSMUSG00000108975	0	0	0	0	0	0
ENSMUSG00000108974	0	0	0	0	0	0
ENSMUSG00000108972	3	1	0	3	0	0
ENSMUSG00000108971	15	11	21	26	16	14
ENSMUSG00000108970	3	0	0	0	0	0
ENSMUSG00000091221	0	2	4	0	1	3
ENSMUSG00000030200	3	0	0	0	0	3
ENSMUSG00000030205	0	0	0	0	0	0
ENSMUSG00000030209	4	0	0	0	0	0
ENSMUSG00000101494	0	0	0	0	0	0
ENSMUSG00000056411	6	15	13	8	9	11
ENSMUSG00000022388	0	0	0	0	0	0
ENSMUSG00000082006	0	0	0	0	0	0
ENSMUSG00000058028	0	0	0	0	0	0
ENSMUSG00000104367	0	5	4	0	1	1
ENSMUSG00000104870	0	0	0	0	0	0
ENSMUSG00000104875	0	0	0	0	0	0
ENSMUSG00000059991	0	0	0	0	0	0

ENSMUSG00000104878	0	0	0	0	0	0
ENSMUSG00000104879	0	0	0	0	0	0
ENSMUSG00000059994	17	17	24	8	2	4
ENSMUSG00000101491	0	0	0	0	0	0
ENSMUSG00000087565	0	0	0	0	0	0
ENSMUSG00000095384	0	0	0	0	0	0
ENSMUSG00000065040	0	0	0	0	0	0
ENSMUSG00000088232	0	0	0	0	0	0
ENSMUSG00000088237	0	0	0	0	0	0
ENSMUSG00000088236	0	0	0	0	0	0
ENSMUSG00000088234	0	0	0	0	0	0
ENSMUSG00000101986	0	0	0	0	3	0
ENSMUSG00000088637	2	0	0	0	1	1
ENSMUSG00000047497	281	194	351	70	98	153
ENSMUSG00000107995	0	0	0	0	0	0
ENSMUSG00000107996	0	0	0	3	0	0
ENSMUSG00000047495	1	0	3	0	0	0
ENSMUSG00000107993	0	0	0	0	0	0
ENSMUSG00000107998	0	0	0	0	0	0
ENSMUSG00000103064	0	0	0	0	0	0
ENSMUSG00000103069	0	0	0	0	0	0
ENSMUSG00000061144	0	0	0	0	0	0
ENSMUSG00000111729	0	0	0	0	0	0
ENSMUSG00000087747	0	0	0	0	0	0
ENSMUSG00000111995	0	0	0	0	0	0
ENSMUSG00000106113	0	0	0	0	0	0
ENSMUSG00000106112	0	0	2	0	0	0
ENSMUSG00000106111	0	0	0	0	0	0
ENSMUSG00000106117	0	0	0	0	0	0
ENSMUSG00000104248	0	0	0	0	0	0
ENSMUSG00000012042	0	0	0	0	0	0
ENSMUSG00000106119	0	0	0	0	0	0
ENSMUSG00000108548	0	0	0	0	3	0
ENSMUSG00000103396	0	0	0	0	0	0
ENSMUSG00000111994	0	0	0	0	0	2
ENSMUSG00000103397	0	0	0	0	0	0
ENSMUSG00000112295	2	5	2	0	4	2
ENSMUSG00000029848	0	0	0	0	0	0
ENSMUSG00000094304	0	2	0	0	0	0
ENSMUSG00000103395	0	0	0	0	0	0
ENSMUSG00000094303	0	0	0	0	0	0
ENSMUSG00000112293	3	0	0	0	0	0
ENSMUSG00000112292	0	0	0	0	0	1
ENSMUSG00000112299	0	0	0	0	0	0
ENSMUSG00000029844	9	0	0	5	7	10
ENSMUSG00000029847	10	2	7	0	8	5
ENSMUSG00000093726	2	12	10	0	5	2
ENSMUSG00000108540	0	0	0	0	0	0
ENSMUSG00000094112	0	0	0	0	0	0
ENSMUSG00000020673	0	0	4	0	0	0
ENSMUSG00000020672	101	71	105	0	0	0
ENSMUSG00000028460	2	7	0	0	0	0
ENSMUSG00000020679	0	0	0	0	0	0
ENSMUSG00000011632	0	0	0	0	0	1
ENSMUSG00000092223	0	0	3	0	0	0
ENSMUSG00000092221	0	0	0	0	0	0
ENSMUSG00000101499	2	0	0	0	0	0
ENSMUSG00000104247	0	0	0	0	0	1

ENSMUSG00000055333	31	34	18	31	7	47
ENSMUSG00000093729	0	0	0	0	0	0
ENSMUSG00000040154	0	0	0	0	0	0
ENSMUSG00000026285	0	2	2	2	0	0
ENSMUSG00000029595	0	0	0	0	0	0
ENSMUSG00000024749	0	0	0	0	0	2
ENSMUSG00000113658	0	0	0	0	0	0
ENSMUSG00000113659	0	0	0	0	0	0
ENSMUSG00000113654	0	0	0	0	0	0
ENSMUSG00000113655	0	0	0	0	0	0
ENSMUSG00000083127	0	0	0	0	0	0
ENSMUSG00000113653	0	0	0	0	0	0
ENSMUSG00000038071	0	0	0	0	0	0
ENSMUSG00000109161	0	0	0	0	0	0
ENSMUSG00000109166	0	0	0	0	0	0
ENSMUSG00000114921	2	0	0	0	0	0
ENSMUSG00000114920	0	0	0	0	0	0
ENSMUSG00000109169	0	0	0	0	0	0
ENSMUSG00000114927	0	0	0	0	0	0
ENSMUSG00000046922	0	0	0	0	0	0
ENSMUSG00000029337	0	8	6	0	0	0
ENSMUSG00000029335	15	7	15	0	0	6
ENSMUSG00000106800	3	0	0	0	0	4
ENSMUSG00000106803	1	0	0	0	1	0
ENSMUSG00000106802	6	6	0	2	2	2
ENSMUSG00000106805	0	0	0	0	0	0
ENSMUSG00000066262	0	0	0	0	2	0
ENSMUSG00000066263	0	5	0	0	0	2
ENSMUSG00000106808	0	0	0	0	0	0
ENSMUSG00000070501	7	8	7	5	5	7
ENSMUSG00000066268	0	0	0	0	0	0
ENSMUSG00000066269	0	0	0	0	0	0
ENSMUSG00000070504	0	0	0	0	0	0
ENSMUSG00000081666	6	3	7	2	9	5
ENSMUSG00000021101	0	0	10	0	4	4
ENSMUSG00000106452	0	0	0	0	0	0
ENSMUSG00000109786	0	0	0	0	0	0
ENSMUSG00000096218	2	3	1	2	3	1
ENSMUSG00000096219	0	0	0	0	0	0
ENSMUSG00000096215	0	0	0	0	0	0
ENSMUSG00000096212	0	0	0	0	0	0
ENSMUSG00000096213	0	0	0	0	0	0
ENSMUSG00000109789	1	2	0	0	0	0
ENSMUSG00000109788	0	0	0	0	0	0
ENSMUSG00000083123	0	0	0	0	0	0
ENSMUSG00000088983	0	0	0	0	0	0
ENSMUSG00000005320	2	0	0	0	2	0
ENSMUSG00000113771	0	0	0	0	0	0
ENSMUSG00000074637	2	0	0	2	0	6
ENSMUSG00000111722	4	8	5	10	0	0
ENSMUSG00000088986	0	0	0	0	0	0
ENSMUSG00000111726	3	0	0	0	0	0
ENSMUSG00000057400	0	0	0	0	0	0
ENSMUSG00000107775	0	0	0	0	0	0
ENSMUSG00000025900	34	48	31	99	130	114
ENSMUSG00000025905	0	0	0	0	0	4
ENSMUSG00000104409	0	0	0	0	0	0
ENSMUSG00000104408	0	0	0	0	0	0

ENSMUSG00000108788	0	0	0	0	0	0
ENSMUSG00000104403	0	0	0	0	0	0
ENSMUSG00000108785	0	0	0	0	0	0
ENSMUSG00000108786	0	0	0	0	0	3
ENSMUSG00000108787	0	0	0	0	0	0
ENSMUSG00000104407	0	0	0	0	0	0
ENSMUSG00000108781	0	0	0	0	0	0
ENSMUSG00000108782	0	0	0	0	0	0
ENSMUSG00000108783	2	0	0	0	0	0
ENSMUSG00000045381	0	5	8	0	1	3
ENSMUSG00000083919	0	0	0	0	0	0
ENSMUSG00000083918	0	0	0	0	0	0
ENSMUSG00000083917	0	7	0	0	0	0
ENSMUSG00000083916	0	0	0	0	0	0
ENSMUSG00000083915	0	0	0	0	0	3
ENSMUSG00000083913	0	0	0	0	0	1
ENSMUSG00000083912	0	0	0	0	0	0
ENSMUSG00000111962	4	0	4	6	3	0
ENSMUSG00000111963	0	0	0	15	18	9
ENSMUSG00000103643	0	0	0	0	0	0
ENSMUSG00000103383	0	0	0	0	0	0
ENSMUSG00000088044	0	0	0	0	1	0
ENSMUSG00000095506	0	0	0	0	0	0
ENSMUSG00000056755	63	58	72	15	51	42
ENSMUSG00000056752	9	0	12	16	6	7
ENSMUSG00000056758	0	0	0	0	2	7
ENSMUSG00000103385	3	4	5	2	0	0
ENSMUSG00000027505	8	0	10	5	4	3
ENSMUSG00000027508	439	403	501	302	315	410
ENSMUSG00000092314	0	0	0	0	0	0
ENSMUSG00000074970	0	0	0	0	0	0
ENSMUSG00000045521	14	16	21	8	14	8
ENSMUSG00000065329	0	0	4	0	0	0
ENSMUSG00000095179	0	0	0	0	0	0
ENSMUSG00000095176	0	0	0	0	0	0
ENSMUSG00000095175	0	0	0	0	0	0
ENSMUSG00000045528	0	0	0	0	0	0
ENSMUSG00000037247	0	0	0	0	5	0
ENSMUSG00000095171	0	0	0	0	0	0
ENSMUSG00000091586	126	123	146	78	101	121
ENSMUSG00000088045	0	0	0	0	0	0
ENSMUSG00000098519	0	0	0	0	0	0
ENSMUSG00000098518	2	0	1	2	0	2
ENSMUSG00000098515	0	0	0	0	0	0
ENSMUSG00000098514	0	1	0	0	0	0
ENSMUSG00000080758	0	0	0	0	0	0
ENSMUSG00000080750	0	0	0	0	0	0
ENSMUSG00000080751	0	0	0	0	0	0
ENSMUSG00000080752	0	0	0	0	0	0
ENSMUSG00000065428	1	1	1	1	1	0
ENSMUSG00000080755	0	0	0	0	0	0
ENSMUSG00000080756	0	0	0	0	0	0
ENSMUSG00000106985	0	0	0	0	0	0
ENSMUSG00000093639	0	0	0	0	0	0
ENSMUSG00000093632	0	1	0	0	0	0
ENSMUSG00000093635	0	0	0	0	0	0
ENSMUSG00000086752	20	8	18	0	5	0
ENSMUSG00000086750	0	5	2	0	0	0

ENSMUSG00000092934	1	0	1	1	0	0
ENSMUSG00000086757	0	0	0	0	0	0
ENSMUSG00000097393	0	0	0	0	0	2
ENSMUSG00000094037	0	0	0	0	0	0
ENSMUSG00000047674	0	0	0	0	0	0
ENSMUSG00000077696	0	1	0	0	0	0
ENSMUSG00000077693	0	0	0	0	0	0
ENSMUSG00000077691	0	0	0	0	0	0
ENSMUSG00000099403	0	0	0	0	0	0
ENSMUSG00000092456	2	2	0	0	0	0
ENSMUSG00000055471	0	0	0	0	0	0
ENSMUSG00000033227	0	0	8	0	6	3
ENSMUSG00000099407	0	0	0	0	0	0
ENSMUSG00000081407	0	0	0	0	0	0
ENSMUSG00000081402	13	10	8	9	12	10
ENSMUSG00000081409	0	0	0	0	0	0
ENSMUSG00000081408	0	0	0	0	0	0
ENSMUSG00000077521	0	0	0	0	0	0
ENSMUSG00000077522	0	0	0	0	0	0
ENSMUSG00000077528	0	2	0	0	0	0
ENSMUSG00000040541	0	0	0	0	0	0
ENSMUSG00000040543	97	83	67	136	130	211
ENSMUSG00000111126	0	0	0	0	0	0
ENSMUSG00000082278	9	10	11	5	6	4
ENSMUSG00000030049	0	0	0	0	0	0
ENSMUSG00000030048	6	5	6	12	23	25
ENSMUSG00000030043	0	0	0	0	0	0
ENSMUSG00000048215	2	0	2	2	2	1
ENSMUSG00000048217	3	0	0	0	0	0
ENSMUSG00000048216	29	21	10	4	20	11
ENSMUSG00000099263	0	0	0	0	0	0
ENSMUSG00000099266	0	0	0	0	0	0
ENSMUSG00000099264	0	0	0	0	0	0
ENSMUSG00000099269	0	0	0	0	0	0
ENSMUSG00000111124	0	0	0	0	0	0
ENSMUSG00000083580	0	0	0	0	0	0
ENSMUSG00000083581	0	0	0	0	0	0
ENSMUSG00000083584	0	0	0	0	0	0
ENSMUSG00000083586	0	0	0	0	0	0
ENSMUSG00000083587	0	0	0	0	0	0
ENSMUSG00000030598	53	65	58	24	26	34
ENSMUSG00000030592	8	22	19	3	3	3
ENSMUSG00000082181	0	0	0	0	0	0
ENSMUSG00000082187	4	0	0	0	0	0
ENSMUSG00000082189	0	0	0	0	0	0
ENSMUSG00000095503	0	0	0	0	0	0
ENSMUSG00000109607	0	0	0	0	0	2
ENSMUSG00000049694	0	0	0	0	0	0
ENSMUSG00000111122	0	0	0	0	0	0
ENSMUSG00000109605	0	0	0	0	0	0
ENSMUSG00000049692	0	0	0	0	0	0
ENSMUSG00000037064	0	0	0	0	0	0
ENSMUSG00000105180	0	0	0	0	0	0
ENSMUSG00000109601	0	0	2	0	0	0
ENSMUSG00000110578	0	0	0	0	0	0
ENSMUSG00000100779	0	4	0	0	0	0
ENSMUSG00000100778	0	0	0	0	0	0
ENSMUSG00000100777	0	0	0	0	0	0

ENSMUSG00000100776	0	0	0	0	0	0
ENSMUSG00000100771	0	0	0	0	0	0
ENSMUSG00000100773	0	0	0	0	0	0
ENSMUSG00000100772	0	0	0	0	0	0
ENSMUSG00000087937	0	0	0	0	0	0
ENSMUSG00000087936	0	0	0	0	0	0
ENSMUSG00000087932	0	0	0	0	0	0
ENSMUSG00000114200	0	3	0	0	0	3
ENSMUSG00000087939	0	5	0	0	0	3
ENSMUSG00000105188	0	0	0	0	3	0
ENSMUSG00000064605	0	0	0	0	0	0
ENSMUSG00000049699	0	0	6	0	0	0
ENSMUSG00000064601	0	0	0	0	0	0
ENSMUSG00000083632	2	1	3	1	2	3
ENSMUSG00000083630	0	0	0	0	0	3
ENSMUSG00000083631	0	0	0	0	0	0
ENSMUSG00000083636	0	2	0	0	0	1
ENSMUSG00000083635	0	0	0	0	0	0
ENSMUSG00000075163	0	0	0	0	0	0
ENSMUSG00000075161	0	0	0	0	0	0
ENSMUSG00000075167	0	0	0	0	0	0
ENSMUSG00000075166	0	0	0	0	0	0
ENSMUSG00000075165	0	0	0	0	0	0
ENSMUSG00000075164	0	0	0	0	0	0
ENSMUSG00000075169	0	0	0	0	0	0
ENSMUSG00000031884	0	0	0	0	0	0
ENSMUSG00000031409	0	0	0	0	0	0
ENSMUSG00000112439	2	0	0	0	0	0
ENSMUSG00000061387	0	0	0	0	0	0
ENSMUSG00000061388	0	0	0	0	0	0
ENSMUSG00000028893	139	147	127	64	75	113
ENSMUSG00000107208	2	5	2	2	6	2
ENSMUSG00000107204	0	0	0	0	0	0
ENSMUSG00000107207	4	2	4	0	0	3
ENSMUSG00000107203	4	0	2	5	2	8
ENSMUSG00000033765	0	2	0	0	0	0
ENSMUSG00000101916	0	0	0	0	0	0
ENSMUSG00000095774	0	0	0	0	0	0
ENSMUSG00000101914	0	0	0	0	0	0
ENSMUSG00000101915	0	0	0	0	0	0
ENSMUSG00000101912	0	0	0	0	0	0
ENSMUSG00000101913	0	0	0	0	0	0
ENSMUSG00000101910	0	0	0	0	0	0
ENSMUSG00000101911	0	0	0	0	0	0
ENSMUSG00000101918	0	0	0	0	0	0
ENSMUSG00000101919	1	0	0	0	0	0
ENSMUSG00000095770	0	0	0	1	0	0
ENSMUSG00000095773	0	0	0	0	0	0
ENSMUSG00000103459	0	0	0	2	0	0
ENSMUSG00000102111	0	0	0	0	0	0
ENSMUSG00000103454	0	0	1	2	2	3
ENSMUSG00000032921	0	0	0	0	0	5
ENSMUSG00000103451	0	0	0	0	0	0
ENSMUSG00000044103	0	8	6	0	0	6
ENSMUSG00000073781	0	0	0	0	0	0
ENSMUSG00000065266	0	0	0	0	0	0
ENSMUSG00000073785	0	0	0	3	0	0
ENSMUSG00000065260	0	0	0	0	0	0

ENSMUSG00000073786	0	0	0	0	0	0
ENSMUSG00000065269	0	0	0	0	0	0
ENSMUSG00000105480	0	0	0	0	0	0
ENSMUSG00000065727	0	0	0	0	0	0
ENSMUSG00000074783	11	3	0	15	16	18
ENSMUSG00000024503	0	0	0	0	0	0
ENSMUSG00000024502	0	0	0	0	0	0
ENSMUSG00000024500	0	0	0	0	0	0
ENSMUSG00000024505	135	152	106	91	125	149
ENSMUSG00000030866	0	0	0	0	0	0
ENSMUSG00000105481	6	6	3	0	3	5
ENSMUSG00000089570	0	0	0	0	0	0
ENSMUSG00000089576	0	0	3	3	0	0
ENSMUSG00000089575	0	0	0	0	0	0
ENSMUSG00000097327	29	17	12	12	13	21
ENSMUSG00000097326	4	0	0	0	0	0
ENSMUSG00000073257	0	0	0	0	0	0
ENSMUSG00000110576	0	0	0	0	0	0
ENSMUSG00000022832	0	6	0	0	0	0
ENSMUSG00000062128	0	0	0	0	0	0
ENSMUSG00000062121	0	0	0	0	0	0
ENSMUSG00000110577	0	0	0	0	0	0
ENSMUSG00000062124	0	0	0	0	0	0
ENSMUSG00000027072	0	0	0	0	0	0
ENSMUSG00000105484	0	0	0	0	0	3
ENSMUSG00000042417	0	0	5	10	10	5
ENSMUSG00000110363	0	0	0	0	0	0
ENSMUSG00000070128	1	0	1	1	1	1
ENSMUSG00000069720	0	0	0	0	0	0
ENSMUSG00000069722	0	0	0	0	0	0
ENSMUSG00000070129	0	1	0	0	0	1
ENSMUSG00000102328	0	0	0	0	0	0
ENSMUSG00000105485	0	4	2	2	2	2
ENSMUSG00000102325	0	0	0	0	0	0
ENSMUSG00000102327	0	0	0	0	0	0
ENSMUSG00000102320	0	0	0	0	0	0
ENSMUSG00000105117	0	0	0	0	0	0
ENSMUSG00000095500	0	0	0	0	0	0
ENSMUSG00000096057	0	0	0	0	0	0
ENSMUSG00000096051	0	0	0	0	0	0
ENSMUSG00000096053	1	1	1	1	0	0
ENSMUSG00000110369	0	0	0	0	0	0
ENSMUSG00000032358	8	0	2	0	0	0
ENSMUSG00000069890	2	4	4	0	0	7
ENSMUSG00000096865	0	0	0	0	0	0
ENSMUSG00000072717	0	0	0	0	0	0
ENSMUSG00000096587	0	0	0	0	0	0
ENSMUSG00000096584	1	0	0	0	0	0
ENSMUSG00000096585	0	0	0	0	0	0
ENSMUSG00000096582	1	1	1	1	0	1
ENSMUSG00000096583	1	0	1	0	0	0
ENSMUSG00000096589	0	0	0	0	0	0
ENSMUSG00000104249	0	0	0	0	0	0
ENSMUSG00000050141	16	33	23	17	18	9
ENSMUSG00000050147	60	37	52	53	47	28
ENSMUSG00000104242	0	0	0	0	0	0
ENSMUSG00000044205	0	0	0	0	0	0
ENSMUSG00000070719	0	0	0	0	0	0

ENSMUSG00000082584	0	0	0	0	0	0
ENSMUSG00000082583	0	0	0	0	0	2
ENSMUSG00000101145	0	0	0	0	0	0
ENSMUSG00000101147	0	0	0	0	0	0
ENSMUSG00000101146	0	0	0	0	0	0
ENSMUSG00000101141	2	1	0	0	0	0
ENSMUSG00000101140	0	0	0	0	0	0
ENSMUSG00000101143	0	0	0	0	0	0
ENSMUSG00000101142	0	0	0	0	0	0
ENSMUSG00000091583	0	0	0	0	0	0
ENSMUSG00000101149	0	0	0	1	0	0
ENSMUSG00000101148	0	0	0	0	0	0
ENSMUSG00000091585	0	0	0	0	0	0
ENSMUSG00000091584	0	0	0	0	0	0
ENSMUSG00000091055	0	0	0	0	0	0
ENSMUSG00000059547	0	0	0	0	0	0
ENSMUSG00000056592	165	153	187	74	82	111
ENSMUSG00000091059	0	0	0	0	0	0
ENSMUSG00000082589	0	0	0	0	0	2
ENSMUSG00000110452	0	0	0	0	0	0
ENSMUSG00000015519	0	0	0	0	0	0
ENSMUSG00000112882	0	0	0	0	0	0
ENSMUSG00000104936	40	34	27	64	38	77
ENSMUSG00000104935	3	0	0	0	0	0
ENSMUSG00000104934	0	0	0	0	0	0
ENSMUSG00000104933	1	1	1	0	0	0
ENSMUSG00000104932	0	0	0	0	0	0
ENSMUSG00000104931	0	0	0	0	0	0
ENSMUSG00000104930	0	0	3	0	0	0
ENSMUSG00000095778	0	0	0	0	0	0
ENSMUSG00000030443	77	77	71	46	32	63
ENSMUSG00000100234	1	1	1	1	0	1
ENSMUSG00000100236	0	0	0	4	1	0
ENSMUSG00000100230	0	0	0	0	0	0
ENSMUSG00000100231	0	0	0	0	0	0
ENSMUSG00000100232	0	0	0	0	0	0
ENSMUSG00000100233	0	0	0	0	0	0
ENSMUSG00000100238	0	0	0	0	0	0
ENSMUSG00000100239	0	0	0	0	0	0
ENSMUSG00000042414	0	0	0	0	0	0
ENSMUSG00000027070	92	93	21	0	3	3
ENSMUSG00000027077	0	0	0	0	6	0
ENSMUSG00000027075	182	189	162	120	73	98
ENSMUSG00000108619	0	0	0	0	0	0
ENSMUSG00000097173	3	0	0	2	0	0
ENSMUSG00000099052	0	0	0	0	0	0

8m.gene.count.matrix

gene_id	C1	C2	C3	C4	C5	M1	M2	M3	M4	M5
ENSMUSG00000045915	0	0	3	0	0	0	6	0	0	3
ENSMUSG00000086512	0	0	0	0	0	0	0	0	0	0
ENSMUSG00000086515	0	0	6	0	3	0	3	0	0	3
ENSMUSG00000086517	0	0	0	0	0	0	0	0	0	0
ENSMUSG00000062826	0	0	0	0	0	0	3	0	3	0
ENSMUSG00000086519	0	0	0	0	0	0	0	0	0	6
ENSMUSG00000101049	0	0	0	0	0	0	0	0	0	0
ENSMUSG00000112484	0	0	0	0	0	0	5	0	3	0
ENSMUSG00000112485	0	0	0	0	0	0	0	0	0	0
ENSMUSG00000089450	0	0	0	0	0	0	0	0	0	0
ENSMUSG00000112487	0	0	0	0	0	0	0	0	0	0
ENSMUSG00000112480	0	0	0	0	0	0	0	0	0	0
ENSMUSG00000093346	0	1	2	1	1	1	0	1	3	1
ENSMUSG00000112482	0	0	0	0	0	0	0	0	0	5
ENSMUSG00000093344	0	0	0	0	0	0	0	0	0	0
ENSMUSG00000049506	0	0	0	0	0	0	0	0	0	0
ENSMUSG00000039269	0	0	0	0	0	0	0	0	0	0
ENSMUSG00000100948	0	0	0	0	0	0	0	0	0	0
ENSMUSG00000108612	9	5	2	10	2	5	5	4	3	3
ENSMUSG00000009646	0	0	0	0	3	0	3	0	0	0
ENSMUSG00000036523	28	43	38	29	36	89	130	68	108	55
ENSMUSG00000089451	0	0	0	0	0	0	0	0	0	0
ENSMUSG00000100949	0	0	0	0	0	0	0	0	0	0
ENSMUSG00000094310	0	0	0	0	0	0	0	0	0	0
ENSMUSG00000089452	0	0	0	0	0	0	0	0	0	0
ENSMUSG00000109088	0	0	0	0	0	0	0	0	0	0
ENSMUSG00000109089	28	27	28	31	14	22	46	26	17	10
ENSMUSG00000055109	0	0	0	0	0	0	0	0	0	0
ENSMUSG00000112739	6	15	12	6	0	24	6	6	9	11
ENSMUSG00000109080	0	6	6	3	0	6	9	3	6	0
ENSMUSG00000109081	0	0	0	0	0	0	0	0	0	0
ENSMUSG00000109083	0	0	0	0	0	0	0	0	0	6
ENSMUSG00000112732	0	0	0	0	0	0	0	0	0	0
ENSMUSG00000055102	0	0	0	0	0	0	0	0	0	0
ENSMUSG00000109086	5	2	0	3	0	2	1	0	0	0
ENSMUSG00000113288	0	0	0	0	0	3	3	0	3	0
ENSMUSG00000106300	0	0	0	0	0	0	0	0	3	0
ENSMUSG00000106301	0	0	0	0	0	0	0	0	0	0
ENSMUSG00000106304	3	13	10	3	11	4	12	14	17	16
ENSMUSG00000091292	0	0	0	0	0	0	0	0	0	0
ENSMUSG00000113281	0	0	0	0	0	0	0	0	0	0
ENSMUSG00000113280	0	0	0	0	0	0	0	0	0	0
ENSMUSG00000113283	0	0	0	0	0	0	0	0	0	0
ENSMUSG00000113282	0	0	0	0	2	0	0	0	0	0
ENSMUSG00000113285	0	0	0	0	0	0	0	0	0	2
ENSMUSG00000113284	0	0	3	0	0	0	0	0	0	3
ENSMUSG00000113287	0	0	0	0	0	0	0	3	0	0
ENSMUSG00000097044	0	0	0	0	0	0	0	3	0	0
ENSMUSG00000097040	0	5	0	0	1	3	0	0	0	0
ENSMUSG00000097041	0	0	0	0	0	0	0	0	0	0
ENSMUSG00000097043	0	0	0	0	0	0	0	0	0	0
ENSMUSG00000097049	0	0	0	3	0	4	0	0	0	3
ENSMUSG00000100944	0	3	0	0	2	0	0	0	0	2
ENSMUSG00000071065	0	0	0	0	0	0	0	0	0	0
ENSMUSG00000046881	2	0	3	0	3	3	0	3	3	0
ENSMUSG00000046886	0	0	0	0	0	0	6	0	0	0

ENSMUSG00000114889	0	0	0	0	0	0	0	0	0	0
ENSMUSG00000114885	0	0	0	0	0	0	0	0	0	0
ENSMUSG00000114883	0	0	0	0	0	0	0	0	2	0
ENSMUSG00000110398	0	0	0	0	0	0	0	0	0	0
ENSMUSG00000110391	0	0	0	0	2	0	0	0	0	0
ENSMUSG00000109691	0	0	0	0	0	0	0	0	0	0
ENSMUSG00000080987	0	0	0	0	0	0	0	0	0	0
ENSMUSG00000096309	0	0	0	0	0	0	0	0	0	0
ENSMUSG00000060416	0	0	0	0	0	0	0	0	0	0
ENSMUSG00000089257	0	0	0	0	0	0	0	0	0	0
ENSMUSG00000100942	0	0	0	0	0	0	0	0	0	0
ENSMUSG00000105651	33	35	28	32	20	35	12	23	20	17
ENSMUSG00000114779	0	0	0	0	0	0	0	0	0	0
ENSMUSG00000109330	0	0	0	0	0	0	0	0	0	0
ENSMUSG00000109331	0	0	0	0	0	0	0	0	0	0
ENSMUSG00000105654	0	0	0	0	0	0	0	0	0	0
ENSMUSG00000109334	0	0	0	0	0	0	0	0	0	0
ENSMUSG00000109335	0	0	0	0	0	0	0	0	0	0
ENSMUSG00000114770	0	0	0	0	0	0	0	0	0	0
ENSMUSG00000080321	0	0	0	0	0	0	0	0	0	0
ENSMUSG00000114772	0	0	0	0	0	0	0	0	0	0
ENSMUSG00000109339	0	0	0	0	0	0	0	0	0	0
ENSMUSG00000114774	0	0	0	0	0	0	0	0	0	0
ENSMUSG00000114775	0	0	5	3	0	3	0	0	0	0
ENSMUSG00000114776	0	0	0	0	0	0	0	0	0	0
ENSMUSG00000114777	0	0	0	0	0	0	0	0	0	0
ENSMUSG00000049608	6	9	20	21	6	6	63	3	39	24
ENSMUSG00000025001	166	108	234	169	96	98	216	173	214	165
ENSMUSG00000110641	0	0	0	0	0	0	0	0	0	0
ENSMUSG00000025003	0	0	0	0	0	0	0	0	0	0
ENSMUSG00000110643	0	0	0	0	0	0	0	0	0	0
ENSMUSG00000025004	0	0	0	0	0	0	0	0	0	0
ENSMUSG00000110646	0	0	0	0	0	0	0	0	0	0
ENSMUSG00000110647	41	41	51	41	26	52	43	24	36	38
ENSMUSG00000070330	0	0	2	0	0	0	0	0	0	3
ENSMUSG00000070332	3	0	0	6	6	0	1	3	7	0
ENSMUSG00000070335	0	0	0	0	0	0	0	0	0	0
ENSMUSG00000070334	0	0	0	0	0	0	0	0	0	0
ENSMUSG00000070337	17	6	6	9	8	6	11	6	6	12
ENSMUSG00000070336	34	53	32	61	54	72	72	60	59	28
ENSMUSG00000108060	0	0	0	7	0	0	0	0	0	0
ENSMUSG00000108063	0	0	0	0	0	0	0	0	0	0
ENSMUSG00000050424	0	0	0	6	0	0	0	3	0	0
ENSMUSG00000108065	0	0	0	0	0	0	0	0	0	0
ENSMUSG00000108066	0	0	0	0	0	0	0	0	0	0
ENSMUSG00000108069	0	0	0	0	0	0	0	0	0	0
ENSMUSG00000108068	0	0	0	0	0	0	0	0	0	0
ENSMUSG00000040836	371	409	455	350	368	487	886	578	629	517
ENSMUSG00000083030	0	0	0	0	0	0	0	0	0	0
ENSMUSG00000083031	0	0	0	0	0	0	3	0	0	0
ENSMUSG00000083034	0	0	0	0	0	0	0	0	6	0
ENSMUSG00000083039	0	0	0	0	0	0	0	0	0	0
ENSMUSG00000100941	0	0	0	0	0	0	0	0	0	0
ENSMUSG00000082939	0	0	0	0	0	0	0	0	0	0
ENSMUSG00000083185	0	0	0	0	0	0	0	0	0	0
ENSMUSG00000060412	0	0	0	0	0	0	0	0	0	0
ENSMUSG00000107460	0	0	0	0	0	0	0	0	0	0
ENSMUSG00000087144	0	0	0	0	0	0	0	0	0	0

ENSMUSG00000079034	0	0	0	0	0	0	0	0	0	0
ENSMUSG00000082397	0	0	0	0	0	0	0	0	0	0
ENSMUSG00000087140	0	0	0	0	0	0	0	0	0	0
ENSMUSG00000111117	0	0	0	0	0	0	0	0	0	0
ENSMUSG00000111116	58	35	105	45	50	54	67	39	80	51
ENSMUSG00000111115	0	0	0	0	0	0	0	0	0	0
ENSMUSG00000111114	0	0	0	0	0	0	0	0	0	0
ENSMUSG00000111112	0	0	0	0	0	0	0	0	0	0
ENSMUSG00000111111	0	0	0	0	0	0	0	0	0	0
ENSMUSG00000111110	0	0	0	0	0	0	0	0	0	0
ENSMUSG00000079112	0	0	0	0	0	0	0	0	0	0
ENSMUSG00000079113	0	0	0	0	0	0	0	0	0	0
ENSMUSG00000114298	0	0	0	0	0	0	0	0	0	0
ENSMUSG00000079116	0	0	0	0	0	0	0	0	0	0
ENSMUSG00000111119	0	0	0	0	0	0	0	0	0	0
ENSMUSG00000009350	0	0	0	0	0	0	0	0	0	0
ENSMUSG00000108349	0	0	0	0	0	0	0	0	0	0
ENSMUSG00000093342	0	0	0	0	0	2	0	0	0	0
ENSMUSG00000001670	15	42	6	21	21	69	20	3	9	15
ENSMUSG00000112486	0	0	0	0	0	0	0	0	0	0
ENSMUSG00000001672	9	5	0	9	0	5	16	6	0	3
ENSMUSG00000026063	0	0	0	0	0	0	0	0	0	0
ENSMUSG00000093340	0	0	0	0	0	0	0	0	0	0
ENSMUSG00000108556	0	0	0	0	0	0	0	0	0	0
ENSMUSG00000031786	0	0	0	0	0	0	3	0	0	0
ENSMUSG00000026068	65	40	66	242	63	85	122	98	192	101
ENSMUSG00000026069	251	217	173	325	209	256	518	214	229	351
ENSMUSG00000112481	5	3	3	0	0	3	9	3	3	0
ENSMUSG00000112483	26	14	22	43	15	49	30	37	42	26
ENSMUSG00000041872	0	4	0	0	0	0	2	0	0	0
ENSMUSG00000053198	102	114	116	121	100	117	288	142	148	148
ENSMUSG00000053194	0	0	0	0	0	0	8	0	0	0
ENSMUSG00000101256	0	0	0	0	0	0	0	0	0	0
ENSMUSG00000078240	2	0	3	0	0	3	3	2	3	3
ENSMUSG00000081308	0	0	0	0	0	0	0	0	0	0
ENSMUSG00000090087	0	0	0	0	0	0	0	0	0	0
ENSMUSG00000081300	0	0	0	0	0	0	0	0	0	0
ENSMUSG00000081304	5	1	2	0	0	1	2	0	0	7
ENSMUSG00000081305	0	0	0	0	0	0	0	0	0	0
ENSMUSG00000081306	0	0	0	0	0	0	0	0	0	0
ENSMUSG00000053441	118	153	67	83	113	86	260	141	61	52
ENSMUSG00000073012	0	0	0	0	0	0	3	0	0	0
ENSMUSG00000092821	0	0	0	0	0	0	0	0	0	0
ENSMUSG00000092822	0	0	0	0	0	0	0	0	0	0
ENSMUSG00000027335	160	104	115	99	116	124	84	86	99	92
ENSMUSG00000042993	5	0	5	3	4	6	3	2	5	4
ENSMUSG00000059069	0	0	0	0	0	0	0	0	0	0
ENSMUSG00000036526	15	12	35	0	12	13	77	40	50	66
ENSMUSG00000090558	0	0	0	4	3	0	0	2	0	0
ENSMUSG00000084875	0	0	0	0	0	0	0	0	0	0
ENSMUSG00000090552	0	0	0	0	0	0	0	2	0	0
ENSMUSG00000090550	0	0	0	0	6	0	0	0	0	0
ENSMUSG00000084871	0	0	0	0	0	0	0	0	0	0
ENSMUSG00000090556	8	3	6	16	6	0	6	0	3	0
ENSMUSG00000090555	0	0	5	0	0	0	0	0	0	0
ENSMUSG00000098709	0	0	0	0	0	0	0	0	0	0
ENSMUSG00000098707	0	0	0	0	0	0	0	2	0	0
ENSMUSG00000098705	0	0	0	0	0	0	0	0	0	0

ENSMUSG00000030657	284	367	346	298	267	500	754	495	691	555
ENSMUSG00000098703	0	0	0	0	0	0	0	0	0	0
ENSMUSG00000098700	0	0	0	0	0	0	0	0	0	0
ENSMUSG00000093878	0	0	1	3	2	0	3	2	2	2
ENSMUSG00000034219	0	0	0	0	0	0	0	0	0	0
ENSMUSG00000078935	0	0	0	0	0	0	0	0	0	0
ENSMUSG00000078934	0	0	0	0	0	0	0	0	0	0
ENSMUSG00000086653	0	0	0	0	0	0	0	0	0	0
ENSMUSG00000086652	7	0	5	14	3	9	0	15	11	5
ENSMUSG00000086650	0	0	0	0	0	0	0	0	0	0
ENSMUSG00000093871	0	0	0	0	0	0	0	0	0	0
ENSMUSG00000043119	0	0	0	0	0	0	0	0	0	0
ENSMUSG00000086659	0	0	0	0	0	0	0	0	0	0
ENSMUSG00000086658	0	0	0	0	0	1	1	0	0	2
ENSMUSG00000109965	0	4	0	2	5	3	7	1	10	0
ENSMUSG00000077223	0	0	0	0	0	0	0	0	0	0
ENSMUSG00000031297	6	8	24	6	6	9	29	8	4	17
ENSMUSG00000077224	0	0	0	0	0	0	0	0	0	0
ENSMUSG00000077228	0	0	0	0	0	0	0	0	0	0
ENSMUSG00000031298	93	221	146	106	144	176	531	200	283	221
ENSMUSG00000114124	0	0	0	0	0	0	0	0	0	0
ENSMUSG00000082641	0	0	2	0	0	0	2	0	0	0
ENSMUSG00000111324	0	0	15	0	0	3	12	5	13	6
ENSMUSG00000082020	0	0	0	0	0	0	0	0	0	0
ENSMUSG00000082025	0	0	0	0	0	0	0	0	0	0
ENSMUSG00000030650	51	68	45	48	54	65	48	69	99	88
ENSMUSG00000111016	0	0	0	0	0	0	0	0	0	0
ENSMUSG00000095091	0	0	0	0	0	0	0	0	0	0
ENSMUSG00000073907	0	0	0	0	0	3	0	0	0	3
ENSMUSG00000101302	0	0	0	0	0	0	0	0	0	0
ENSMUSG00000101301	0	0	0	0	0	0	0	0	0	0
ENSMUSG00000101300	0	0	0	0	0	0	0	0	0	0
ENSMUSG00000101307	0	5	0	0	0	0	0	3	0	0
ENSMUSG00000111322	0	0	0	0	0	0	0	0	0	0
ENSMUSG00000073901	0	0	0	0	0	0	0	0	0	0
ENSMUSG00000073900	0	0	0	0	0	0	0	0	0	0
ENSMUSG00000037922	278	262	307	477	157	174	549	271	316	388
ENSMUSG00000037924	3	0	0	0	0	0	0	0	3	0
ENSMUSG00000071506	0	3	0	8	0	0	0	3	3	3
ENSMUSG00000043800	0	0	0	0	0	0	0	0	0	0
ENSMUSG00000103239	0	0	0	0	0	0	0	0	0	0
ENSMUSG00000043807	17	9	28	14	10	49	36	24	9	41
ENSMUSG00000112737	0	0	0	0	0	0	0	0	0	0
ENSMUSG00000103234	1	5	2	3	4	7	3	7	4	4
ENSMUSG00000103237	0	0	0	0	0	0	0	0	2	0
ENSMUSG00000103231	0	0	0	0	0	0	0	0	0	0
ENSMUSG00000103230	11	2	10	10	3	11	25	22	2	8
ENSMUSG00000087015	0	0	0	0	0	0	0	0	0	0
ENSMUSG00000087010	0	0	0	0	0	0	0	0	0	0
ENSMUSG00000109084	0	0	0	0	0	0	0	0	0	0
ENSMUSG00000112733	3	0	0	0	0	3	2	0	4	0
ENSMUSG00000099547	0	0	0	0	0	0	0	0	0	0
ENSMUSG00000099546	0	0	0	0	0	0	0	0	0	0
ENSMUSG00000099545	1	1	1	2	1	1	1	1	1	1
ENSMUSG00000099544	0	0	0	0	0	0	0	0	0	0
ENSMUSG00000099542	0	0	0	0	0	0	0	0	0	0
ENSMUSG00000099541	0	0	0	0	0	0	0	0	0	0
ENSMUSG00000019214	98	96	100	98	98	108	127	106	118	105

ENSMUSG00000099549	0	0	0	0	0	0	0	0	0	0
ENSMUSG00000099548	0	0	0	0	0	0	0	0	0	0
ENSMUSG00000081050	0	0	0	0	0	0	0	0	0	0
ENSMUSG00000083775	0	0	0	0	0	0	0	0	0	0
ENSMUSG00000089316	0	0	0	0	0	0	0	0	0	0
ENSMUSG00000089314	0	0	0	0	0	0	0	0	0	0
ENSMUSG00000075081	0	0	0	0	0	0	0	0	0	0
ENSMUSG00000075084	0	0	0	0	0	0	0	0	0	0
ENSMUSG00000075086	0	0	0	0	0	0	0	0	0	0
ENSMUSG00000026882	0	0	0	0	0	0	0	0	0	0
ENSMUSG00000102548	11	9	5	11	9	0	18	11	3	10
ENSMUSG00000102549	5	8	11	13	7	8	0	25	22	12
ENSMUSG00000102547	0	0	0	0	0	0	0	0	0	0
ENSMUSG00000102541	0	0	0	0	0	0	0	0	0	0
ENSMUSG00000084097	0	0	0	0	0	0	0	0	0	0
ENSMUSG00000084096	0	0	0	0	0	0	0	0	5	0
ENSMUSG00000084095	0	0	0	0	0	0	0	0	0	0
ENSMUSG00000084094	2	0	5	0	6	2	3	6	3	2
ENSMUSG00000084092	0	0	0	0	0	0	0	0	0	0
ENSMUSG00000068888	0	0	0	0	0	0	0	0	0	0
ENSMUSG00000000263	0	0	0	6	0	0	0	0	0	0
ENSMUSG00000068887	0	0	0	0	0	0	0	0	0	0
ENSMUSG00000068885	0	0	0	0	0	0	0	0	0	0
ENSMUSG00000065872	0	0	0	0	0	0	0	0	0	0
ENSMUSG00000022056	0	0	0	0	0	0	0	0	0	0
ENSMUSG00000064471	0	0	0	0	0	0	0	0	0	0
ENSMUSG00000106305	0	0	0	0	0	0	0	0	0	0
ENSMUSG00000064477	0	0	0	0	0	0	0	0	0	0
ENSMUSG00000022055	0	27	14	9	6	6	29	33	36	9
ENSMUSG00000107960	0	0	0	0	0	0	0	0	0	0
ENSMUSG00000107073	0	0	0	0	0	0	0	0	0	0
ENSMUSG00000107072	0	0	0	0	0	0	0	0	0	6
ENSMUSG00000107070	0	0	0	0	0	0	3	0	0	0
ENSMUSG00000106308	0	0	0	0	0	0	0	0	0	0
ENSMUSG00000107074	0	0	0	0	0	0	0	0	0	0
ENSMUSG00000023159	0	0	0	0	0	0	0	0	0	0
ENSMUSG00000075330	0	7	0	5	3	0	0	0	2	6
ENSMUSG00000079652	3	0	0	0	5	6	24	0	4	5
ENSMUSG00000079655	0	0	0	0	0	0	0	0	0	0
ENSMUSG00000079654	117	120	169	150	112	155	261	202	222	169
ENSMUSG00000075334	15	9	12	9	9	9	14	17	3	21
ENSMUSG00000075335	0	0	0	0	0	0	0	0	0	0
ENSMUSG00000023153	42	70	42	80	48	52	34	34	46	63
ENSMUSG00000068776	0	0	0	0	0	0	0	0	0	0
ENSMUSG00000111250	18	0	6	0	17	6	0	3	9	6
ENSMUSG00000113286	0	0	0	0	0	0	0	0	0	0
ENSMUSG00000037033	0	0	0	0	0	0	0	0	0	0
ENSMUSG00000085329	0	0	0	0	0	0	0	0	0	0
ENSMUSG00000085324	0	0	0	0	0	0	0	0	0	0
ENSMUSG00000085325	0	0	0	0	0	0	0	0	0	0
ENSMUSG00000085323	0	0	0	0	0	2	0	0	0	0
ENSMUSG00000085320	0	0	0	0	0	0	0	0	0	0
ENSMUSG00000085321	0	0	0	0	0	0	0	0	0	0
ENSMUSG00000074061	0	0	0	0	0	0	0	0	0	0
ENSMUSG00000074060	0	0	3	0	0	0	0	0	0	0
ENSMUSG00000074063	158	137	172	194	249	91	254	101	156	134
ENSMUSG00000074062	0	0	0	0	0	0	0	0	0	0
ENSMUSG00000074067	56	34	29	29	33	19	21	32	16	23

ENSMUSG00000103090	0	0	0	0	0	0	0	0	0	0
ENSMUSG00000088541	0	0	0	0	0	0	0	0	0	0
ENSMUSG00000101891	0	0	0	0	0	0	0	0	0	0
ENSMUSG00000088542	0	0	0	0	0	0	0	0	0	0
ENSMUSG00000101897	2	0	0	0	0	0	0	0	0	0
ENSMUSG00000101896	0	0	0	0	0	0	0	0	0	0
ENSMUSG00000088547	0	0	0	0	0	0	0	1	0	0
ENSMUSG00000101894	0	0	0	0	0	0	0	0	0	0
ENSMUSG00000034764	2	0	0	6	3	3	7	0	0	3
ENSMUSG00000034762	3	3	0	0	6	2	3	3	21	0
ENSMUSG00000111253	0	0	0	0	0	0	0	0	0	0
ENSMUSG00000088898	0	0	0	0	0	0	0	0	0	0
ENSMUSG00000034768	52	57	79	48	72	69	27	69	39	54
ENSMUSG00000021799	89	157	76	112	110	94	119	139	40	106
ENSMUSG00000094888	0	0	0	0	0	0	0	0	0	0
ENSMUSG00000094885	0	0	0	0	0	0	0	0	0	0
ENSMUSG00000094884	0	0	0	0	0	0	0	0	0	0
ENSMUSG00000094887	0	0	0	0	0	0	0	0	0	0
ENSMUSG00000039717	0	0	0	0	0	0	0	0	0	0
ENSMUSG00000039710	3	0	0	8	0	0	0	0	0	0
ENSMUSG00000021790	0	0	0	0	0	0	0	0	0	0
ENSMUSG00000094883	0	0	0	0	0	0	0	0	0	0
ENSMUSG00000062591	48	61	54	53	55	60	51	42	32	58
ENSMUSG00000044292	0	0	0	0	0	0	0	0	0	0
ENSMUSG00000062598	0	0	0	0	0	0	0	0	0	0
ENSMUSG00000103792	0	0	0	0	0	0	0	0	0	1
ENSMUSG00000103790	0	0	0	0	0	0	0	0	0	0
ENSMUSG00000036892	0	3	0	7	0	0	0	0	0	3
ENSMUSG00000103797	0	0	0	0	0	0	0	0	0	0
ENSMUSG00000103794	0	0	0	0	0	0	0	0	0	0
ENSMUSG00000103795	0	0	0	0	0	0	0	0	0	0
ENSMUSG00000103798	0	0	0	0	0	0	0	0	0	0
ENSMUSG00000103799	0	0	0	0	3	0	0	0	0	0
ENSMUSG00000036899	0	0	0	3	0	0	0	0	0	0
ENSMUSG00000097282	16	15	15	12	8	21	18	10	10	23
ENSMUSG00000097283	0	0	0	0	0	0	0	0	3	0
ENSMUSG00000097280	0	0	3	0	0	0	0	0	0	0
ENSMUSG00000097281	0	0	0	0	2	0	6	0	0	8
ENSMUSG00000097286	3	0	0	6	0	0	3	11	0	6
ENSMUSG00000097287	87	127	141	156	134	150	216	174	169	172
ENSMUSG00000097288	3	0	0	5	3	6	0	3	6	0
ENSMUSG00000097289	0	0	3	0	0	0	6	6	3	3
ENSMUSG00000063646	10	28	18	27	14	32	67	32	43	33
ENSMUSG00000059366	0	0	0	0	0	0	0	0	0	0
ENSMUSG00000112980	68	53	34	66	39	50	84	79	70	55
ENSMUSG00000106679	0	0	0	0	0	0	0	0	0	0
ENSMUSG00000106673	3	6	7	9	2	0	2	3	5	2
ENSMUSG00000106670	0	0	0	0	0	0	0	0	0	0
ENSMUSG00000066031	0	0	0	0	0	0	0	0	0	0
ENSMUSG00000066030	0	0	0	0	0	0	0	0	0	0
ENSMUSG00000106674	0	0	0	4	0	0	0	0	0	0
ENSMUSG00000106675	0	0	0	0	0	0	0	0	0	0
ENSMUSG00000111257	0	0	0	0	0	0	0	0	0	2
ENSMUSG00000069910	62	80	99	73	63	51	119	99	76	79
ENSMUSG00000109530	0	0	0	0	0	0	0	0	0	0
ENSMUSG00000109533	0	0	0	0	0	0	0	0	0	0
ENSMUSG00000109534	0	0	0	3	0	0	0	0	0	0
ENSMUSG00000109535	0	0	0	0	0	0	0	0	0	0

ENSMUSG00000109537	0	0	0	0	0	0	0	0	0	0
ENSMUSG00000100854	0	0	0	0	0	0	0	0	0	0
ENSMUSG00000114577	0	0	0	0	0	0	0	0	0	0
ENSMUSG00000114572	0	1	0	0	0	0	0	0	0	0
ENSMUSG00000100851	0	0	0	0	0	0	0	0	0	0
ENSMUSG00000100852	0	0	0	0	0	0	0	0	0	0
ENSMUSG00000100853	0	0	0	0	0	0	0	0	0	0
ENSMUSG00000100858	0	0	0	2	0	0	0	0	0	0
ENSMUSG00000100859	0	0	0	0	0	0	0	0	0	0
ENSMUSG00000114578	0	0	0	0	0	0	0	0	0	0
ENSMUSG00000101139	0	0	0	0	0	0	0	0	0	0
ENSMUSG00000066697	0	0	0	0	0	0	0	0	0	0
ENSMUSG00000054526	0	0	0	0	0	0	0	0	0	0
ENSMUSG00000112801	0	2	0	0	0	0	0	0	0	0
ENSMUSG00000112802	0	0	3	3	0	9	4	9	2	3
ENSMUSG00000112803	0	0	0	1	0	0	3	0	4	6
ENSMUSG00000112805	0	14	5	3	0	0	16	10	3	3
ENSMUSG00000054520	457	357	434	638	348	510	707	364	605	330
ENSMUSG00000108668	3	3	8	0	7	5	11	7	16	8
ENSMUSG00000108667	0	0	0	0	0	0	0	0	0	0
ENSMUSG00000112809	0	0	0	0	0	0	0	0	0	0
ENSMUSG00000108665	0	0	0	0	0	0	0	0	0	0
ENSMUSG00000108663	0	0	0	0	0	0	0	0	0	0
ENSMUSG00000108662	0	0	0	0	0	0	0	0	0	0
ENSMUSG00000108661	0	0	0	0	0	0	0	0	0	0
ENSMUSG00000105329	0	0	0	0	0	0	0	0	0	0
ENSMUSG00000067341	3	0	9	0	0	6	35	9	14	17
ENSMUSG00000081589	0	0	0	0	0	0	0	0	0	0
ENSMUSG00000058188	0	0	0	0	0	0	0	0	0	0
ENSMUSG00000066964	0	0	0	0	0	0	0	0	0	0
ENSMUSG00000110396	0	0	0	0	0	0	0	0	0	0
ENSMUSG00000060699	0	0	0	0	0	0	0	0	0	0
ENSMUSG00000060691	0	0	0	0	0	0	0	0	0	0
ENSMUSG00000091936	0	0	0	0	0	0	0	0	0	0
ENSMUSG00000075228	0	0	0	0	0	0	0	0	0	0
ENSMUSG00000110395	0	0	0	0	0	0	0	0	0	0
ENSMUSG00000091933	0	0	0	0	0	0	0	0	0	0
ENSMUSG00000091930	0	0	0	0	0	0	0	0	0	0
ENSMUSG00000110392	0	0	0	0	0	0	0	0	0	0
ENSMUSG00000083115	0	0	0	0	0	0	0	0	0	0
ENSMUSG00000091938	51	55	49	94	43	95	73	0	81	50
ENSMUSG00000110393	28	56	39	23	19	42	67	52	56	67
ENSMUSG00000096629	7	3	0	14	13	29	15	36	14	33
ENSMUSG00000083114	0	0	0	0	0	0	0	0	0	0
ENSMUSG00000110390	9	6	3	0	3	3	5	3	0	3
ENSMUSG00000083113	0	0	0	0	0	0	0	0	0	0
ENSMUSG00000023931	24	39	19	20	20	39	27	61	29	35
ENSMUSG00000023930	0	0	0	0	0	0	0	0	0	0
ENSMUSG00000096627	0	0	0	0	0	0	0	0	0	0
ENSMUSG00000096626	0	0	0	0	0	0	0	0	0	0
ENSMUSG00000096624	1	0	0	0	1	1	0	0	0	1
ENSMUSG00000104680	0	0	0	0	0	0	0	0	0	0
ENSMUSG00000083110	0	0	0	0	0	0	0	0	0	0
ENSMUSG00000093554	0	0	0	0	0	0	0	0	0	0
ENSMUSG00000056961	0	0	0	0	0	0	0	0	0	0
ENSMUSG00000056966	39	26	42	42	23	49	32	65	27	61
ENSMUSG00000109974	0	0	0	0	0	0	0	0	0	0
ENSMUSG00000111054	0	0	0	0	0	0	0	0	0	0

ENSMUSG00000111055	107	91	90	79	60	75	50	119	103	98
ENSMUSG00000111052	11	0	0	8	5	0	3	6	0	0
ENSMUSG00000010461	72	100	126	44	110	181	406	169	334	199
ENSMUSG00000100613	0	0	0	0	0	0	0	0	0	0
ENSMUSG00000110989	0	0	0	0	0	0	0	0	0	0
ENSMUSG00000110988	0	0	0	0	0	0	0	0	0	0
ENSMUSG00000110983	0	0	0	0	0	0	0	0	0	0
ENSMUSG00000110982	5	3	6	0	3	0	3	3	0	0
ENSMUSG00000110981	0	0	0	3	5	6	3	3	0	0
ENSMUSG00000110987	0	0	0	0	0	0	0	0	0	0
ENSMUSG00000110986	3	3	0	9	9	5	6	3	3	3
ENSMUSG00000110985	0	0	0	0	0	0	0	0	0	0
ENSMUSG00000024184	0	0	0	0	0	14	0	0	12	3
ENSMUSG00000064860	0	0	0	0	0	0	0	0	0	0
ENSMUSG00000108932	0	0	0	0	0	0	0	0	0	0
ENSMUSG00000091268	0	0	0	0	0	0	0	0	0	0
ENSMUSG00000045775	35	33	42	34	27	30	43	39	63	43
ENSMUSG00000018259	0	0	0	0	0	0	0	0	0	0
ENSMUSG00000108936	0	0	0	0	0	0	0	0	0	0
ENSMUSG00000026885	154	147	195	301	219	209	169	158	170	176
ENSMUSG00000081146	0	0	0	0	0	0	0	0	0	0
ENSMUSG00000081147	0	0	0	0	0	0	0	0	0	0
ENSMUSG00000081144	0	0	0	0	0	0	0	0	0	0
ENSMUSG00000081145	0	0	0	0	0	0	0	0	0	0
ENSMUSG00000081141	0	0	0	0	0	0	0	0	0	0
ENSMUSG00000108939	0	0	0	0	0	0	0	0	0	0
ENSMUSG00000081148	0	0	0	0	0	0	0	0	0	0
ENSMUSG00000027134	100	118	64	108	143	120	196	165	169	154
ENSMUSG00000056380	5	3	0	0	0	9	0	0	0	0
ENSMUSG00000090718	0	0	0	0	0	0	0	0	0	0
ENSMUSG00000017165	0	0	0	0	0	0	0	0	0	0
ENSMUSG00000109333	0	0	0	0	0	0	0	0	0	0
ENSMUSG00000090710	0	0	0	0	0	0	0	0	0	0
ENSMUSG00000090713	0	0	3	0	0	0	0	0	0	0
ENSMUSG00000090715	0	0	0	0	0	0	0	0	0	0
ENSMUSG00000090714	0	0	0	0	0	0	3	0	0	0
ENSMUSG00000105652	0	0	0	0	0	0	0	3	0	0
ENSMUSG00000008496	123	132	82	108	107	297	117	51	212	195
ENSMUSG00000042200	5	6	0	0	0	8	11	6	10	16
ENSMUSG00000094416	0	0	0	0	0	0	0	0	0	0
ENSMUSG00000094415	0	0	0	0	0	0	0	0	0	0
ENSMUSG00000094412	0	0	0	0	0	0	0	0	0	0
ENSMUSG00000114771	7	4	0	5	11	8	14	8	7	17
ENSMUSG00000056032	3	6	6	12	3	6	21	6	3	6
ENSMUSG00000094370	0	0	0	0	0	0	19	0	0	34
ENSMUSG00000094419	0	0	0	0	0	0	0	0	0	0
ENSMUSG00000086496	0	0	0	0	0	0	0	0	0	0
ENSMUSG00000105871	1	0	4	5	3	1	9	2	1	4
ENSMUSG00000064743	0	0	0	0	0	0	0	0	0	0
ENSMUSG00000028370	347	396	313	389	327	343	981	616	498	451
ENSMUSG00000054994	0	0	0	0	0	0	0	0	0	0
ENSMUSG00000048067	0	0	0	0	0	0	0	0	0	0
ENSMUSG00000077021	0	0	0	0	0	0	0	0	1	0
ENSMUSG00000077022	0	0	0	0	0	0	0	0	0	0
ENSMUSG00000077025	0	0	1	0	0	0	0	0	0	1
ENSMUSG00000064746	0	0	0	0	0	0	0	0	0	0
ENSMUSG00000031480	636	649	686	617	622	563	671	578	675	700
ENSMUSG00000055602	0	0	8	1	3	13	19	6	0	7

ENSMUSG00000104057	7	4	3	2	3	0	0	0	7	5
ENSMUSG00000046456	30	19	33	17	24	29	48	44	50	43
ENSMUSG00000083312	0	0	0	0	0	0	0	0	0	0
ENSMUSG00000046450	0	0	0	0	0	0	0	0	0	0
ENSMUSG00000110642	0	0	2	0	0	3	0	0	0	0
ENSMUSG00000025002	0	3	0	0	3	0	0	0	0	0
ENSMUSG00000092715	0	0	0	0	0	0	0	0	0	0
ENSMUSG00000092714	0	0	0	0	0	0	0	0	0	0
ENSMUSG00000077792	0	0	0	0	0	0	0	0	0	0
ENSMUSG00000077793	0	0	0	0	0	0	0	0	0	0
ENSMUSG00000092719	1	0	0	0	0	0	3	0	0	0
ENSMUSG00000092718	0	0	0	0	0	0	0	0	0	0
ENSMUSG00000077796	0	0	0	0	0	0	0	0	0	0
ENSMUSG00000077794	0	0	0	0	0	0	0	0	2	0
ENSMUSG00000110645	0	0	0	0	0	0	0	0	0	0
ENSMUSG00000049334	0	0	0	0	0	0	0	0	0	0
ENSMUSG00000049336	15	6	18	0	3	15	3	3	0	3
ENSMUSG00000029423	0	0	0	0	0	0	0	0	0	0
ENSMUSG00000077178	0	0	0	0	0	0	0	0	0	0
ENSMUSG00000029420	3	0	3	0	3	3	10	0	0	11
ENSMUSG00000006218	18	11	30	3	24	12	38	18	21	11
ENSMUSG00000113369	0	0	0	0	0	0	0	0	0	0
ENSMUSG00000113366	0	0	0	0	0	0	0	0	0	0
ENSMUSG00000113367	0	0	0	0	0	0	0	0	0	0
ENSMUSG00000113364	0	0	0	0	0	0	0	0	0	0
ENSMUSG00000113365	0	0	0	0	0	0	0	0	0	0
ENSMUSG00000113362	3	0	0	3	0	0	6	0	0	3
ENSMUSG00000112147	0	0	0	0	0	0	0	0	0	0
ENSMUSG00000113360	0	0	0	0	0	0	0	0	0	0
ENSMUSG00000113361	89	119	76	83	93	76	109	106	102	87
ENSMUSG00000071749	0	0	0	0	0	0	0	0	0	0
ENSMUSG00000109276	0	0	0	0	0	0	0	0	0	0
ENSMUSG00000109275	0	0	0	0	0	0	0	0	0	0
ENSMUSG00000109273	0	0	0	0	0	0	0	0	0	0
ENSMUSG00000109272	0	0	0	0	0	0	0	0	0	0
ENSMUSG00000109271	0	0	0	0	0	0	0	0	0	0
ENSMUSG00000109278	0	0	0	0	0	0	0	0	0	0
ENSMUSG00000106531	0	0	0	3	3	2	0	0	0	0
ENSMUSG00000106534	2	0	0	0	0	0	0	5	1	0
ENSMUSG00000040838	1	6	10	24	5	3	32	17	3	20
ENSMUSG00000050425	6	12	18	12	3	27	80	20	0	12
ENSMUSG00000108062	0	0	0	0	0	0	0	0	0	0
ENSMUSG00000047324	0	0	0	0	0	0	0	0	0	0
ENSMUSG00000050423	1	4	4	2	18	24	2	2	2	1
ENSMUSG00000063529	23	21	6	0	3	27	36	21	15	6
ENSMUSG00000082551	0	0	0	0	0	0	0	0	0	0
ENSMUSG00000082552	0	0	0	0	0	0	0	0	0	0
ENSMUSG00000082553	0	0	0	0	0	0	0	0	0	0
ENSMUSG00000082555	0	0	0	0	0	0	0	0	0	0
ENSMUSG00000082556	0	0	0	0	0	0	0	0	0	0
ENSMUSG00000082558	0	0	0	0	0	0	0	0	0	0
ENSMUSG00000093474	0	0	0	0	0	0	0	0	0	0
ENSMUSG00000085937	0	0	0	0	0	0	0	0	0	0
ENSMUSG00000085934	0	0	0	0	0	0	0	0	0	0
ENSMUSG00000084259	3	0	0	0	0	0	0	0	0	0
ENSMUSG00000080391	0	0	0	0	0	0	0	0	0	0
ENSMUSG00000083206	6	2	12	6	8	3	7	0	0	0
ENSMUSG00000084257	0	0	0	0	0	0	0	0	0	0

ENSMUSG00000083204	0	0	0	0	0	0	0	0	0	0
ENSMUSG00000084251	0	0	0	0	0	0	0	0	0	0
ENSMUSG00000083202	0	0	0	0	0	0	0	3	0	0
ENSMUSG00000084253	3	0	0	0	0	0	0	0	0	0
ENSMUSG00000083200	0	0	0	0	0	0	0	0	0	0
ENSMUSG00000041248	3	0	5	9	0	0	0	0	0	3
ENSMUSG00000085931	0	0	0	0	0	0	0	0	0	0
ENSMUSG00000025215	18	12	36	6	17	12	6	12	29	14
ENSMUSG00000025216	0	0	0	0	5	9	3	5	0	0
ENSMUSG00000080958	0	0	0	0	0	0	0	0	0	0
ENSMUSG00000080959	0	0	0	0	0	0	0	0	0	0
ENSMUSG00000080952	0	0	0	0	0	0	0	0	0	0
ENSMUSG00000080953	0	0	0	0	0	0	0	0	0	0
ENSMUSG00000080954	0	0	0	0	0	0	0	0	0	0
ENSMUSG00000080955	0	0	0	0	2	0	0	0	0	0
ENSMUSG00000099150	0	0	0	0	0	0	0	0	0	0
ENSMUSG00000099152	0	0	0	0	0	0	0	0	0	0
ENSMUSG00000099153	1	0	4	3	1	2	1	2	1	1
ENSMUSG00000099154	0	0	0	0	0	0	0	0	0	0
ENSMUSG00000099158	0	0	0	0	0	0	0	0	1	0
ENSMUSG00000099159	0	0	0	0	0	0	0	0	0	0
ENSMUSG00000085938	0	0	0	0	0	2	0	3	0	0
ENSMUSG00000068574	1	0	2	3	0	3	0	1	8	3
ENSMUSG00000044149	232	303	308	290	246	360	385	310	349	323
ENSMUSG00000080649	0	0	2	0	2	0	1	0	0	0
ENSMUSG00000085129	27	33	44	35	10	14	56	37	44	34
ENSMUSG00000006711	11	0	0	7	3	5	0	6	3	6
ENSMUSG00000085124	0	0	0	0	0	0	0	0	0	0
ENSMUSG00000085126	0	3	0	0	0	0	0	0	6	0
ENSMUSG00000085127	0	9	3	3	3	5	0	0	6	0
ENSMUSG00000085120	0	0	0	0	0	0	0	0	0	0
ENSMUSG00000085121	0	0	0	0	0	0	0	0	0	0
ENSMUSG00000085122	0	0	0	0	0	0	0	0	0	0
ENSMUSG00000085123	0	0	0	0	0	0	0	0	0	0
ENSMUSG00000074665	0	0	0	0	0	0	0	0	0	0
ENSMUSG00000074664	0	0	0	0	0	0	0	0	0	0
ENSMUSG00000079324	0	0	0	0	0	0	0	0	0	0
ENSMUSG00000041930	42	66	48	51	61	20	12	21	22	11
ENSMUSG00000060070	0	0	0	0	0	0	0	0	0	0
ENSMUSG00000045236	0	0	0	0	0	0	0	0	0	0
ENSMUSG00000045232	11	8	5	5	6	8	7	5	9	13
ENSMUSG00000045238	2	4	6	3	12	2	3	0	3	9
ENSMUSG00000036492	74	61	79	85	91	59	96	70	83	48
ENSMUSG00000047496	184	226	258	153	185	416	391	328	389	394
ENSMUSG00000103802	0	0	0	0	0	0	0	0	0	0
ENSMUSG00000103801	0	0	0	0	0	0	0	0	0	0
ENSMUSG00000103807	3	2	0	0	0	0	0	5	0	0
ENSMUSG00000019785	0	0	0	0	0	0	2	0	0	0
ENSMUSG00000047751	0	0	0	0	0	0	0	3	5	0
ENSMUSG00000089922	0	0	0	0	0	0	3	0	0	0
ENSMUSG00000089924	0	0	3	0	0	3	0	0	0	0
ENSMUSG00000089926	0	0	0	0	0	0	0	0	0	0
ENSMUSG00000089927	0	0	0	0	0	0	0	0	0	0
ENSMUSG00000048501	0	0	0	0	0	0	0	0	0	0
ENSMUSG00000048500	0	0	0	0	0	0	0	0	0	0
ENSMUSG00000048503	272	320	368	363	297	308	362	441	480	333
ENSMUSG00000037737	3	3	0	0	0	0	0	3	0	0
ENSMUSG00000031488	265	294	357	452	264	437	874	413	293	244

ENSMUSG00000076983	0	0	0	1	0	0	0	0	0	0
ENSMUSG00000037738	8	13	7	6	11	22	13	11	9	11
ENSMUSG00000067441	0	0	0	0	0	0	0	0	0	0
ENSMUSG00000031738	0	0	0	0	0	0	0	0	0	0
ENSMUSG00000004821	0	0	0	0	0	0	0	0	0	0
ENSMUSG00000082772	0	0	0	0	0	0	0	0	0	0
ENSMUSG00000043308	0	0	0	0	0	0	0	0	0	0
ENSMUSG00000087199	6	0	0	0	0	0	0	0	0	0
ENSMUSG00000087192	0	0	0	0	0	5	6	11	8	4
ENSMUSG00000087196	0	0	0	0	2	2	6	0	0	0
ENSMUSG00000087195	0	0	0	0	0	0	0	0	0	0
ENSMUSG00000085979	0	0	0	0	0	0	0	0	0	0
ENSMUSG00000085978	0	0	0	0	0	0	0	0	0	0
ENSMUSG00000113232	0	0	0	0	0	0	0	0	0	0
ENSMUSG00000094952	0	0	0	0	0	0	0	0	1	0
ENSMUSG00000085973	0	0	0	0	0	0	0	0	0	0
ENSMUSG00000085972	3	0	3	22	6	6	10	24	1	9
ENSMUSG00000037884	0	0	0	0	0	6	0	0	0	0
ENSMUSG00000085970	0	0	3	0	0	0	0	0	0	0
ENSMUSG00000085976	9	6	13	12	4	0	11	12	18	9
ENSMUSG00000094950	0	0	0	0	0	0	0	0	0	0
ENSMUSG00000081918	0	0	0	0	0	0	0	0	0	0
ENSMUSG00000079114	0	0	0	0	0	0	0	0	0	0
ENSMUSG00000015787	0	3	0	0	0	0	0	0	0	0
ENSMUSG00000082618	0	0	0	0	0	0	0	0	0	0
ENSMUSG00000014782	13	2	6	17	6	6	3	6	3	6
ENSMUSG00000040650	0	0	0	0	0	0	0	0	0	0
ENSMUSG00000101516	0	0	0	0	0	0	0	0	0	0
ENSMUSG00000101517	20	14	11	23	8	8	14	8	5	5
ENSMUSG00000040657	0	0	0	0	0	0	0	0	0	0
ENSMUSG00000101515	0	0	0	0	0	0	0	0	0	0
ENSMUSG00000021961	0	0	3	0	0	3	0	0	0	0
ENSMUSG00000100641	0	0	0	0	0	0	0	0	0	0
ENSMUSG00000100640	0	0	0	0	0	0	0	0	0	0
ENSMUSG00000100643	0	0	0	0	0	0	0	0	0	0
ENSMUSG00000100645	0	0	0	0	0	0	0	0	0	0
ENSMUSG00000100644	0	0	0	0	0	0	0	0	0	0
ENSMUSG00000100647	0	0	0	0	0	0	0	0	0	0
ENSMUSG00000100646	0	0	0	0	0	0	0	0	0	0
ENSMUSG00000100649	0	0	0	0	0	0	0	0	0	0
ENSMUSG00000100648	0	0	2	0	2	0	0	0	0	0
ENSMUSG00000059910	0	0	0	0	0	0	0	0	0	0
ENSMUSG00000107918	0	0	0	0	0	0	0	0	0	0
ENSMUSG00000107914	0	0	0	0	0	0	0	0	0	0
ENSMUSG00000107916	0	0	0	0	0	0	0	0	0	0
ENSMUSG00000107917	0	0	3	0	0	0	0	0	0	0
ENSMUSG00000084754	3	0	0	0	0	0	0	0	0	0
ENSMUSG00000084757	0	0	0	0	0	0	0	0	0	0
ENSMUSG00000084750	0	0	0	0	0	0	0	0	0	0
ENSMUSG00000089107	0	0	0	0	0	0	2	0	0	0
ENSMUSG00000084752	0	0	0	0	0	0	0	0	0	0
ENSMUSG00000084753	0	0	0	0	0	0	0	0	0	0
ENSMUSG00000089109	0	0	0	0	0	0	0	0	0	0
ENSMUSG00000084758	2	0	7	10	6	7	5	7	0	7
ENSMUSG00000075270	347	172	229	123	183	128	89	160	124	223
ENSMUSG00000021359	0	3	6	5	3	0	0	0	5	0
ENSMUSG00000022431	10	3	3	13	3	8	6	12	11	4
ENSMUSG00000022435	0	0	0	0	0	0	0	0	0	0

ENSMUSG00000032113	77	51	81	105	60	54	147	73	79	64
ENSMUSG00000032110	0	0	9	0	6	0	0	0	0	0
ENSMUSG00000032118	203	148	266	131	154	236	358	258	282	296
ENSMUSG00000020364	275	303	317	257	265	283	310	328	442	330
ENSMUSG00000088722	0	0	0	0	0	0	0	0	0	0
ENSMUSG00000113111	0	0	0	0	0	0	0	0	0	0
ENSMUSG00000113110	0	1	0	0	0	2	0	1	3	2
ENSMUSG00000060811	0	0	0	0	0	0	0	0	0	0
ENSMUSG00000102739	0	0	0	0	0	0	0	0	0	0
ENSMUSG00000102738	0	0	0	0	0	0	0	0	0	0
ENSMUSG00000112218	8	10	14	0	0	0	0	5	3	3
ENSMUSG00000112215	0	0	0	0	0	0	0	0	0	0
ENSMUSG00000112217	0	0	0	0	0	0	0	0	0	0
ENSMUSG00000112216	2	2	5	2	2	1	1	3	2	2
ENSMUSG00000112211	0	0	0	0	0	0	0	0	0	0
ENSMUSG00000112213	7	0	3	3	1	4	0	0	2	0
ENSMUSG00000112212	0	1	0	0	0	2	0	0	0	0
ENSMUSG00000036912	7	5	3	3	0	3	18	7	9	6
ENSMUSG00000036916	536	638	638	598	489	540	772	691	640	635
ENSMUSG00000036915	0	0	11	0	3	3	3	0	6	0
ENSMUSG00000028176	8	0	3	0	0	2	13	3	0	3
ENSMUSG00000028177	16	23	25	22	30	22	10	22	9	26
ENSMUSG00000028174	0	0	0	0	0	0	0	0	0	0
ENSMUSG00000028175	40	36	64	25	30	35	118	26	34	40
ENSMUSG00000028172	6	0	3	0	0	3	6	3	9	6
ENSMUSG00000028179	189	131	167	116	126	244	110	198	203	198
ENSMUSG00000039691	17	13	14	6	6	4	0	12	0	47
ENSMUSG00000105422	0	0	0	0	0	0	3	0	0	0
ENSMUSG00000105423	0	0	0	0	0	0	0	0	0	0
ENSMUSG00000026065	43	26	15	35	29	28	37	25	13	33
ENSMUSG00000105426	0	0	0	0	0	0	0	0	0	0
ENSMUSG00000105424	0	0	0	0	0	0	0	0	0	0
ENSMUSG00000105425	0	0	0	0	0	0	3	0	0	0
ENSMUSG00000024295	3	5	3	0	2	6	4	2	7	2
ENSMUSG00000082275	0	0	0	3	0	0	3	0	3	0
ENSMUSG00000103023	0	0	0	0	0	0	0	0	0	0
ENSMUSG00000113169	0	0	0	0	0	0	0	0	0	0
ENSMUSG00000113164	0	0	0	0	0	0	0	0	0	0
ENSMUSG00000113166	0	0	0	0	0	0	0	0	0	0
ENSMUSG00000113167	0	0	0	0	0	0	0	0	0	3
ENSMUSG00000113160	0	0	0	0	0	0	0	0	0	0
ENSMUSG00000113161	4	13	3	6	4	7	11	1	2	3
ENSMUSG00000113162	0	0	0	0	0	0	0	0	0	0
ENSMUSG00000113163	0	0	0	0	0	3	0	0	3	0
ENSMUSG00000069038	0	0	0	0	0	0	0	0	0	0
ENSMUSG00000103026	0	0	3	0	0	3	0	3	6	0
ENSMUSG00000069036	0	0	0	0	0	0	0	0	0	0
ENSMUSG00000103027	0	0	0	0	0	0	0	0	0	0
ENSMUSG00000039963	6	15	6	3	9	5	17	25	9	6
ENSMUSG00000039962	0	0	0	0	0	0	0	0	0	0
ENSMUSG00000021187	60	92	100	113	71	196	270	116	247	184
ENSMUSG00000050097	12	6	12	17	0	9	27	4	15	21
ENSMUSG00000024128	0	0	0	0	0	0	0	0	0	0
ENSMUSG00000008813	0	0	0	0	0	0	0	0	0	0
ENSMUSG00000106738	7	0	0	3	0	2	0	2	0	0
ENSMUSG00000106735	46	62	76	36	29	38	22	32	65	69
ENSMUSG00000106733	0	0	0	0	0	0	0	0	0	0
ENSMUSG00000106730	0	0	0	0	0	0	0	0	0	0

ENSMUSG00000051062	0	0	0	0	0	3	0	0	0	0
ENSMUSG00000059280	0	0	0	0	0	0	0	0	0	0
ENSMUSG00000059136	0	1	0	0	0	3	0	0	0	3
ENSMUSG00000059134	0	0	0	0	0	0	3	0	0	0
ENSMUSG00000051839	17	14	64	65	107	23	21	60	19	6
ENSMUSG00000055884	449	355	369	332	309	575	743	505	379	565
ENSMUSG00000051706	0	0	0	0	0	0	0	0	0	0
ENSMUSG00000055882	0	7	10	6	6	14	14	7	25	6
ENSMUSG00000105289	5	3	2	5	7	11	3	17	6	2
ENSMUSG00000109706	0	0	0	0	0	0	0	0	0	0
ENSMUSG00000109705	0	0	0	0	0	0	0	0	0	0
ENSMUSG00000109704	6	5	6	5	0	3	3	15	0	0
ENSMUSG00000109703	0	0	0	0	0	0	0	0	0	0
ENSMUSG00000109702	0	0	0	0	0	0	0	0	0	0
ENSMUSG00000105280	0	0	0	0	0	0	0	0	0	0
ENSMUSG00000105281	2	3	6	5	2	0	0	5	0	1
ENSMUSG00000089487	0	0	0	0	0	0	0	0	0	0
ENSMUSG00000106888	10	7	9	8	6	9	3	8	15	11
ENSMUSG00000089485	0	0	0	0	0	0	0	0	0	0
ENSMUSG00000089484	2	0	0	0	0	0	0	0	0	0
ENSMUSG00000089481	0	0	0	0	0	0	0	0	0	0
ENSMUSG00000089480	0	0	0	0	0	0	0	0	0	0
ENSMUSG00000106880	0	0	0	0	0	0	0	0	0	0
ENSMUSG00000106883	5	0	0	0	0	0	0	2	0	0
ENSMUSG00000106884	0	0	0	0	0	0	0	0	0	0
ENSMUSG00000106887	0	0	0	0	0	0	0	0	0	0
ENSMUSG00000089488	0	0	0	0	0	0	0	0	0	0
ENSMUSG00000104779	0	0	0	0	0	0	0	0	0	0
ENSMUSG00000104778	0	0	0	0	0	0	0	0	0	0
ENSMUSG00000108258	6	14	8	9	18	3	9	0	6	12
ENSMUSG00000108259	0	0	0	0	0	0	0	0	0	0
ENSMUSG00000022986	0	0	0	0	0	0	0	0	0	0
ENSMUSG00000108252	0	0	0	0	0	0	0	0	0	0
ENSMUSG00000104770	0	0	0	0	0	0	0	0	0	0
ENSMUSG00000108250	0	0	0	0	0	0	0	0	0	0
ENSMUSG00000104772	0	0	0	0	0	0	0	0	0	0
ENSMUSG00000104775	0	0	0	0	0	0	0	0	0	0
ENSMUSG00000108257	6	6	3	0	0	0	2	0	8	5
ENSMUSG00000108254	0	0	0	0	0	0	0	0	3	0
ENSMUSG00000104482	0	0	0	0	0	0	2	2	0	0
ENSMUSG00000104481	0	0	5	0	3	3	0	0	5	0
ENSMUSG00000104480	0	0	0	0	0	0	0	0	0	0
ENSMUSG00000104487	0	0	0	0	0	0	0	0	0	0
ENSMUSG00000104489	0	0	0	0	0	0	0	0	0	0
ENSMUSG00000114304	0	0	0	0	0	0	0	0	0	0
ENSMUSG00000114307	2	0	0	0	0	0	0	0	0	0
ENSMUSG00000096295	0	0	0	0	0	0	0	0	0	0
ENSMUSG00000114301	1	0	0	0	0	0	4	0	0	1
ENSMUSG00000096293	0	0	0	0	0	0	0	0	0	0
ENSMUSG00000114303	0	0	0	0	0	0	0	0	0	0
ENSMUSG00000096291	0	0	0	0	0	0	0	0	1	0
ENSMUSG00000114308	0	0	0	0	0	0	0	0	0	0
ENSMUSG00000096298	0	0	0	0	0	0	0	0	0	0
ENSMUSG00000096299	11	8	1	7	0	14	15	5	8	15
ENSMUSG00000097749	0	2	0	3	0	0	0	3	2	5
ENSMUSG00000097742	0	1	0	0	0	1	3	0	3	0
ENSMUSG00000097744	3	0	3	0	0	0	0	0	0	0
ENSMUSG00000050074	2	0	10	0	0	0	3	0	0	3

ENSMUSG00000054757	0	0	0	0	0	0	0	0	4	0
ENSMUSG00000054753	0	0	0	0	0	0	0	0	0	0
ENSMUSG00000054759	0	0	0	0	0	0	0	0	0	0
ENSMUSG00000091745	3	0	11	8	0	17	8	8	4	7
ENSMUSG00000091744	0	0	0	0	0	0	0	0	0	0
ENSMUSG00000091741	0	0	0	0	0	0	0	0	0	0
ENSMUSG00000091740	0	0	0	0	0	0	0	0	0	0
ENSMUSG00000091743	0	0	0	0	0	4	0	0	1	0
ENSMUSG00000103410	0	0	0	0	0	0	0	0	0	0
ENSMUSG00000091749	2	0	0	0	0	0	0	0	0	0
ENSMUSG00000025983	9	25	15	15	25	28	10	14	21	35
ENSMUSG00000058665	0	0	3	0	6	0	3	0	3	0
ENSMUSG00000058669	3	9	3	3	6	6	20	12	3	3
ENSMUSG00000009292	235	150	120	506	238	148	294	135	285	109
ENSMUSG00000044309	9	3	9	11	0	6	60	9	48	6
ENSMUSG00000110534	0	3	0	0	6	26	39	23	20	3
ENSMUSG00000110535	0	0	0	0	0	0	0	0	0	0
ENSMUSG00000110533	0	0	0	0	0	0	0	0	0	0
ENSMUSG00000027584	3	3	12	0	0	25	0	9	11	15
ENSMUSG00000026182	0	0	5	0	0	4	0	0	0	0
ENSMUSG00000038599	0	0	1	0	0	0	0	0	0	0
ENSMUSG00000080427	0	0	0	0	0	0	0	0	0	0
ENSMUSG00000080428	0	0	0	0	0	0	2	0	0	0
ENSMUSG00000095248	0	0	0	0	0	0	0	0	0	0
ENSMUSG00000095245	0	0	0	0	0	0	2	0	0	0
ENSMUSG00000095246	0	0	0	1	0	1	0	0	0	0
ENSMUSG00000095247	0	7	9	14	8	10	11	7	8	5
ENSMUSG00000095240	0	0	0	0	0	0	0	0	0	0
ENSMUSG00000095241	0	0	0	0	0	0	0	0	0	0
ENSMUSG00000095242	0	0	0	0	0	0	0	0	0	0
ENSMUSG00000095243	0	0	0	0	0	0	0	0	0	0
ENSMUSG00000113945	0	0	0	0	0	0	0	0	0	0
ENSMUSG00000113940	22	51	47	56	64	33	42	26	45	29
ENSMUSG00000113941	3	3	5	9	2	12	6	5	9	0
ENSMUSG00000047544	0	0	0	0	0	0	0	0	0	0
ENSMUSG00000047545	0	0	0	0	0	0	0	0	0	0
ENSMUSG00000094596	0	0	0	0	0	0	0	0	0	0
ENSMUSG00000094594	0	4	2	2	2	0	2	0	2	10
ENSMUSG00000114468	0	0	0	0	0	0	0	0	0	0
ENSMUSG00000086860	0	0	0	2	0	4	0	0	2	0
ENSMUSG00000086861	0	0	0	0	0	0	0	0	0	0
ENSMUSG00000086862	3	0	3	3	0	9	3	0	0	0
ENSMUSG00000086868	0	0	0	0	0	0	0	0	0	0
ENSMUSG00000081486	0	0	0	0	0	0	0	0	0	0
ENSMUSG00000048745	0	0	0	0	0	0	0	0	0	0
ENSMUSG00000081484	0	0	0	0	0	0	0	0	0	0
ENSMUSG00000081483	0	0	0	0	0	0	0	0	0	0
ENSMUSG00000081481	0	0	0	0	0	0	0	0	0	0
ENSMUSG00000077619	0	0	0	0	0	0	0	0	0	0
ENSMUSG00000077618	0	0	0	0	0	0	0	0	0	0
ENSMUSG00000077616	0	0	0	0	0	0	0	0	0	0
ENSMUSG00000077615	0	0	0	0	0	0	0	0	0	0
ENSMUSG00000077612	0	0	0	0	0	0	0	0	0	0
ENSMUSG00000094625	0	0	0	0	0	0	0	0	0	0
ENSMUSG00000094624	0	0	0	0	0	0	0	0	0	0
ENSMUSG00000094626	0	3	0	0	3	3	6	0	12	3
ENSMUSG00000094621	0	1	0	0	0	0	0	0	0	0
ENSMUSG00000094620	3	0	0	0	0	0	0	0	0	0

ENSMUSG00000094628	23	19	47	34	16	45	28	42	38	57
ENSMUSG00000013083	0	0	0	0	5	4	6	0	0	0
ENSMUSG00000044899	0	0	0	0	0	0	0	0	0	0
ENSMUSG00000090900	0	0	0	0	0	1	0	0	0	0
ENSMUSG00000044897	3	0	0	0	0	0	0	0	0	0
ENSMUSG00000091185	1	2	1	1	2	2	5	2	0	1
ENSMUSG00000090907	0	0	0	3	0	0	0	0	0	0
ENSMUSG00000048299	0	0	0	0	0	0	0	0	0	0
ENSMUSG00000105178	0	0	0	0	0	0	0	0	0	0
ENSMUSG00000048292	3	8	9	3	3	7	2	1	12	6
ENSMUSG00000048294	0	0	0	0	0	0	0	0	4	0
ENSMUSG00000092502	0	0	0	0	0	0	0	0	0	0
ENSMUSG00000092503	0	0	0	0	0	0	0	0	0	0
ENSMUSG00000099772	0	0	0	0	0	0	0	0	0	0
ENSMUSG00000099774	0	0	0	0	0	0	0	0	0	0
ENSMUSG00000099776	0	0	0	0	0	0	0	0	0	0
ENSMUSG00000099777	0	0	0	0	0	0	0	0	0	0
ENSMUSG00000099778	0	0	0	0	0	0	0	0	0	0
ENSMUSG00000099779	0	0	0	0	0	0	0	5	0	3
ENSMUSG00000092508	0	0	0	0	0	0	0	0	0	0
ENSMUSG00000092509	0	0	0	5	0	0	0	1	0	0
ENSMUSG00000086284	0	0	0	0	0	0	0	0	0	0
ENSMUSG00000046793	5	11	9	3	9	5	0	9	13	6
ENSMUSG00000086286	0	0	0	0	0	0	0	0	0	0
ENSMUSG00000086283	0	0	11	3	0	18	30	9	24	14
ENSMUSG00000007646	102	98	103	154	74	74	153	108	80	115
ENSMUSG00000097798	0	0	0	0	0	0	0	0	0	0
ENSMUSG00000086138	0	0	0	0	0	0	0	0	0	0
ENSMUSG00000086139	0	6	1	2	0	2	5	4	0	5
ENSMUSG00000086136	0	2	2	0	0	0	0	0	2	0
ENSMUSG00000086137	0	0	0	0	0	0	0	0	0	0
ENSMUSG00000043670	3	0	3	0	0	0	6	3	3	9
ENSMUSG00000043671	232	263	325	246	239	277	498	266	366	301
ENSMUSG00000086132	0	0	0	0	0	0	0	0	0	0
ENSMUSG00000105174	0	0	0	0	0	0	0	0	0	0
ENSMUSG00000098829	0	0	0	0	0	0	0	0	0	0
ENSMUSG00000098828	0	0	0	0	0	0	0	0	0	0
ENSMUSG00000076251	0	0	1	2	0	0	1	0	2	1
ENSMUSG00000000037	53	54	44	41	27	45	105	67	30	74
ENSMUSG00000076253	0	0	0	0	0	0	0	0	0	0
ENSMUSG00000098823	0	0	0	1	0	0	0	1	0	1
ENSMUSG00000098821	0	0	0	0	0	0	0	0	0	0
ENSMUSG00000098820	0	0	0	0	0	0	0	0	0	0
ENSMUSG00000070828	0	0	0	0	0	0	0	0	0	0
ENSMUSG00000087425	0	0	0	0	0	0	0	0	0	0
ENSMUSG00000070821	0	0	3	0	0	0	0	0	0	0
ENSMUSG00000070820	0	0	0	0	0	0	0	0	0	0
ENSMUSG00000070823	9	5	2	3	0	6	0	8	5	18
ENSMUSG00000087428	0	0	0	0	0	0	0	0	0	0
ENSMUSG00000041138	12	9	24	15	12	18	0	18	11	9
ENSMUSG00000049168	0	0	0	0	0	0	0	0	0	0
ENSMUSG00000037577	91	84	171	91	96	152	238	165	231	204
ENSMUSG00000083502	0	0	4	0	0	0	1	4	0	1
ENSMUSG00000083505	0	3	2	6	11	6	6	4	1	8
ENSMUSG00000083507	3	0	0	0	0	0	0	0	0	0
ENSMUSG00000049160	2	0	0	0	5	0	7	0	10	5
ENSMUSG00000083509	0	0	0	0	0	0	0	0	0	0
ENSMUSG00000037578	0	0	0	0	0	0	0	0	0	0

ENSMUSG00000037579	14	0	18	0	11	7	23	11	10	9
ENSMUSG00000026841	5	3	6	0	6	3	5	0	9	0
ENSMUSG00000079423	0	0	0	0	0	0	0	0	0	0
ENSMUSG00000079421	0	0	0	0	0	0	0	0	0	0
ENSMUSG00000079424	0	0	0	0	0	0	0	0	0	0
ENSMUSG00000050917	0	0	0	0	0	0	0	0	0	0
ENSMUSG00000110847	0	0	0	6	0	0	0	0	0	0
ENSMUSG00000005649	0	0	0	0	0	0	0	0	0	0
ENSMUSG00000071158	10	0	0	0	0	0	0	0	0	0
ENSMUSG00000084193	0	0	0	0	0	0	0	3	0	0
ENSMUSG00000084191	0	0	0	0	0	0	0	0	0	0
ENSMUSG00000084196	0	0	0	0	0	0	0	0	0	0
ENSMUSG00000084197	0	0	0	0	0	0	0	0	0	0
ENSMUSG00000052865	0	0	0	0	0	0	0	0	0	0
ENSMUSG00000084198	0	0	0	0	0	0	0	0	0	2
ENSMUSG00000084199	0	0	1	0	0	0	0	0	0	0
ENSMUSG00000000690	8	6	3	21	3	6	22	3	18	15
ENSMUSG00000084873	0	0	0	0	0	0	0	0	0	0
ENSMUSG00000099370	5	1	3	4	5	4	0	2	8	6
ENSMUSG00000080852	0	0	0	0	0	0	0	0	0	0
ENSMUSG00000080850	56	50	62	31	35	55	36	58	54	68
ENSMUSG00000105172	0	0	0	0	0	0	0	0	0	0
ENSMUSG00000110848	0	0	0	0	0	0	0	0	0	0
ENSMUSG00000105236	0	0	0	0	0	0	0	0	0	0
ENSMUSG00000104820	0	2	2	0	0	0	0	0	0	0
ENSMUSG00000105173	5	3	6	3	0	6	0	0	0	0
ENSMUSG00000075707	3	4	13	0	6	15	53	22	23	33
ENSMUSG00000104824	0	10	24	0	17	0	18	0	14	0
ENSMUSG00000104826	0	0	0	0	0	0	0	0	0	0
ENSMUSG00000074259	6	0	3	6	3	0	7	3	17	11
ENSMUSG00000034532	20	37	14	42	21	46	48	42	15	34
ENSMUSG00000074252	3	0	2	8	7	6	4	7	3	8
ENSMUSG00000078773	81	29	42	74	25	8	44	27	32	24
ENSMUSG00000074254	6	0	0	39	0	0	3	0	0	6
ENSMUSG00000032053	16	0	6	27	6	68	270	95	138	128
ENSMUSG00000020411	5	15	12	3	12	13	9	0	4	11
ENSMUSG00000081812	0	0	0	0	0	0	0	0	0	0
ENSMUSG00000081814	0	0	0	0	0	0	0	0	0	0
ENSMUSG00000065151	0	0	0	0	0	0	0	0	0	0
ENSMUSG00000082096	0	0	0	0	0	0	0	0	0	0
ENSMUSG00000065155	0	0	2	1	0	0	0	0	0	0
ENSMUSG00000073705	33	28	58	59	31	51	113	62	54	28
ENSMUSG00000006527	688	808	910	921	754	810	1006	862	980	854
ENSMUSG00000006522	29	33	58	72	44	124	154	66	159	94
ENSMUSG00000006529	0	0	0	0	0	0	0	0	6	0
ENSMUSG00000075605	0	0	0	0	0	0	0	0	0	3
ENSMUSG00000079180	0	0	0	0	0	0	2	0	4	0
ENSMUSG00000079183	0	0	0	0	0	0	0	0	0	0
ENSMUSG00000098016	0	0	0	0	0	0	0	3	0	0
ENSMUSG00000098017	0	0	0	0	0	0	0	0	0	0
ENSMUSG00000098015	0	0	0	0	0	0	0	0	0	0
ENSMUSG00000098012	0	0	0	0	0	0	0	0	0	0
ENSMUSG00000098013	0	0	0	0	0	0	0	0	0	0
ENSMUSG00000098010	0	0	0	0	0	0	0	0	0	0
ENSMUSG00000077938	0	0	0	0	0	0	0	0	0	0
ENSMUSG00000088577	0	0	0	0	0	0	0	0	0	0
ENSMUSG00000063216	0	0	0	0	0	0	0	0	0	0
ENSMUSG00000104188	0	8	3	8	4	3	0	0	0	0

ENSMUSG00000111037	0	0	0	0	0	0	0	0	0	0
ENSMUSG00000086655	0	0	0	0	0	3	0	6	0	0
ENSMUSG00000099601	0	0	0	0	0	0	0	0	0	0
ENSMUSG00000108407	0	0	0	3	0	0	0	0	0	0
ENSMUSG00000035910	0	0	0	0	0	0	0	3	0	0
ENSMUSG00000035916	0	11	6	0	9	29	79	21	47	5
ENSMUSG00000035031	0	0	9	0	0	0	0	3	0	0
ENSMUSG00000035914	198	205	236	161	165	259	389	284	286	277
ENSMUSG00000104187	8	13	23	18	9	12	15	18	21	11
ENSMUSG00000108403	0	0	0	0	0	0	0	0	0	3
ENSMUSG00000078938	0	0	0	0	0	0	0	3	6	0
ENSMUSG00000084942	6	0	0	0	0	0	0	0	0	2
ENSMUSG00000084945	0	0	0	0	0	0	0	0	0	0
ENSMUSG00000084944	0	0	0	0	0	0	0	0	0	0
ENSMUSG00000084947	0	18	0	10	6	6	5	18	5	0
ENSMUSG00000084949	0	0	0	0	0	0	0	0	0	0
ENSMUSG00000038891	0	0	0	0	0	0	0	0	0	0
ENSMUSG00000073380	18	3	14	14	22	9	8	9	12	13
ENSMUSG00000110095	0	0	0	0	0	0	0	0	0	0
ENSMUSG00000030368	0	0	0	0	0	0	0	0	0	0
ENSMUSG00000030366	0	0	0	0	0	0	0	0	0	0
ENSMUSG00000030364	0	0	0	0	0	0	0	0	0	3
ENSMUSG00000030363	0	0	0	0	0	0	0	0	0	0
ENSMUSG00000110090	0	0	2	0	0	0	0	0	0	0
ENSMUSG00000030651	3	3	3	0	0	0	6	0	6	3
ENSMUSG00000088796	0	0	0	0	0	0	0	0	0	0
ENSMUSG00000101495	0	0	0	0	0	0	0	0	0	0
ENSMUSG00000088792	0	0	0	0	0	0	0	0	0	0
ENSMUSG00000101721	0	0	0	0	0	0	0	0	0	0
ENSMUSG00000101720	0	0	0	0	0	0	0	0	0	0
ENSMUSG00000101723	0	0	0	0	0	0	0	0	0	0
ENSMUSG00000101722	0	0	3	0	0	0	8	0	0	0
ENSMUSG00000101725	0	0	0	0	0	0	0	0	0	0
ENSMUSG00000101727	0	0	0	0	0	0	0	0	0	0
ENSMUSG00000101726	0	0	0	0	0	0	0	0	0	0
ENSMUSG00000101728	0	0	0	0	0	0	0	0	0	0
ENSMUSG00000061845	0	0	0	0	0	0	0	0	0	0
ENSMUSG00000061847	0	0	0	0	0	0	0	0	0	0
ENSMUSG00000061843	0	0	0	0	0	0	0	3	0	0
ENSMUSG00000088463	0	0	0	0	0	0	0	0	0	0
ENSMUSG00000088460	0	0	0	0	0	0	0	0	0	0
ENSMUSG00000095407	18	18	18	8	11	21	34	27	6	30
ENSMUSG00000058270	0	0	0	0	0	0	3	0	0	0
ENSMUSG00000088469	0	0	0	0	0	0	0	0	0	0
ENSMUSG00000058275	0	0	0	0	0	0	0	0	0	0
ENSMUSG00000107891	0	3	2	2	3	2	0	0	0	6
ENSMUSG00000107890	0	0	0	0	0	0	0	0	0	0
ENSMUSG00000107893	0	0	0	0	0	0	0	0	0	0
ENSMUSG00000059891	3	0	2	2	0	7	8	4	14	0
ENSMUSG00000107894	8	6	3	9	8	6	33	3	23	8
ENSMUSG00000107898	0	0	0	0	0	0	0	0	0	0
ENSMUSG00000059898	0	3	6	12	0	9	39	15	3	6
ENSMUSG00000023391	0	0	0	0	4	0	0	0	3	0
ENSMUSG00000029379	0	3	0	0	3	12	10	10	22	10
ENSMUSG00000107769	0	0	0	0	0	0	3	0	0	0
ENSMUSG00000107762	0	0	0	0	0	0	0	0	0	0
ENSMUSG00000107766	2	4	2	3	0	5	3	3	0	0
ENSMUSG00000107764	0	0	0	0	0	0	0	0	0	0

ENSMUSG00000103658	0	0	2	0	0	0	0	0	0	0
ENSMUSG00000111979	0	0	0	0	0	0	0	0	0	0
ENSMUSG00000111978	0	0	0	0	0	0	0	0	0	0
ENSMUSG00000111975	0	0	0	0	0	0	0	0	0	0
ENSMUSG00000103652	0	0	0	0	0	0	0	0	0	0
ENSMUSG00000036036	94	76	114	102	131	99	146	87	88	102
ENSMUSG00000103650	0	0	0	0	0	0	0	0	0	0
ENSMUSG00000036030	29	18	9	19	13	19	62	18	21	37
ENSMUSG00000111970	0	0	3	0	0	0	0	0	0	0
ENSMUSG00000111973	0	0	0	0	2	0	3	0	0	0
ENSMUSG00000111972	0	0	0	0	0	0	0	0	0	3
ENSMUSG00000058856	0	0	0	0	0	0	0	0	0	0
ENSMUSG00000094021	0	0	0	0	0	0	0	0	0	0
ENSMUSG00000094020	0	0	0	0	0	0	0	0	0	0
ENSMUSG00000094027	0	0	4	0	0	0	0	0	0	7
ENSMUSG00000094026	0	0	0	0	0	0	0	0	0	0
ENSMUSG00000094025	0	0	0	0	0	0	0	0	0	0
ENSMUSG00000094024	0	0	0	0	0	0	0	0	0	0
ENSMUSG00000095998	0	0	0	0	0	0	0	0	0	0
ENSMUSG00000089732	0	0	0	0	0	0	0	0	0	0
ENSMUSG00000089737	0	0	6	0	3	0	10	0	4	3
ENSMUSG00000095992	0	0	0	0	0	0	0	0	0	0
ENSMUSG00000095993	0	0	0	0	0	0	0	3	0	0
ENSMUSG00000095994	0	0	0	0	0	0	0	0	0	0
ENSMUSG00000095996	0	0	0	0	0	0	0	0	0	0
ENSMUSG00000095997	0	0	0	0	0	0	0	0	0	0
ENSMUSG00000106388	0	0	0	0	0	0	0	0	0	0
ENSMUSG00000106389	0	0	0	0	0	0	0	0	0	0
ENSMUSG00000095262	6	9	5	8	0	1	0	0	5	5
ENSMUSG00000106382	0	0	0	0	0	0	0	0	0	0
ENSMUSG00000106383	55	102	107	56	58	62	79	111	55	50
ENSMUSG00000106381	0	0	0	0	0	0	0	0	0	0
ENSMUSG00000106386	24	13	19	25	13	20	29	23	11	28
ENSMUSG00000106387	0	0	0	0	0	0	0	0	0	0
ENSMUSG00000106384	3	9	8	12	9	23	22	27	11	24
ENSMUSG00000028707	0	0	0	0	0	0	0	0	0	0
ENSMUSG00000112409	0	0	0	0	0	2	0	0	0	3
ENSMUSG00000112404	0	0	0	0	0	0	9	0	0	0
ENSMUSG00000112407	0	0	0	0	0	0	0	0	0	0
ENSMUSG00000112400	0	3	0	3	0	6	3	0	3	0
ENSMUSG00000112401	0	0	0	0	0	0	0	0	0	0
ENSMUSG00000112402	0	0	0	0	0	0	0	0	3	0
ENSMUSG00000112403	0	0	0	0	0	0	0	0	0	0
ENSMUSG00000114806	0	0	0	0	0	0	0	0	0	0
ENSMUSG00000114807	0	0	0	0	0	0	0	0	0	0
ENSMUSG00000114804	3	0	0	0	0	0	2	0	0	0
ENSMUSG00000114805	0	9	7	8	3	7	0	3	6	1
ENSMUSG00000114802	0	0	0	0	4	0	0	0	0	0
ENSMUSG00000114800	77	115	109	86	123	48	9	78	44	75
ENSMUSG00000114808	0	0	0	0	0	0	0	0	0	0
ENSMUSG00000114809	0	0	0	0	0	0	0	0	0	0
ENSMUSG00000110314	0	0	0	0	0	0	0	0	2	0
ENSMUSG00000110315	0	0	0	0	0	0	0	0	0	0
ENSMUSG00000110310	0	0	0	0	0	0	0	0	0	0
ENSMUSG00000099501	0	0	0	0	0	0	0	0	0	0
ENSMUSG00000092073	0	0	0	0	0	0	0	0	0	3
ENSMUSG00000092077	2	3	3	0	11	0	3	3	4	1
ENSMUSG00000038135	22	23	16	32	15	26	41	18	34	16

ENSMUSG00000092075	0	0	2	0	0	0	0	0	0	0
ENSMUSG00000109000	0	0	0	0	0	0	0	0	0	0
ENSMUSG00000109001	0	0	0	0	0	0	0	0	0	0
ENSMUSG00000109003	0	0	0	0	0	0	1	0	0	3
ENSMUSG00000109004	0	0	0	0	0	0	0	0	0	0
ENSMUSG00000075033	153	176	206	181	171	205	400	195	266	253
ENSMUSG00000109007	0	0	0	0	0	0	0	0	0	0
ENSMUSG00000109009	6	3	8	0	0	0	4	0	0	6
ENSMUSG00000096444	0	0	0	0	0	0	0	0	0	0
ENSMUSG00000046807	8	76	38	34	54	16	168	72	12	45
ENSMUSG00000046804	0	0	0	0	0	0	0	0	2	0
ENSMUSG00000029074	4	2	3	0	0	0	0	3	0	0
ENSMUSG00000106968	0	0	0	0	0	0	6	0	0	0
ENSMUSG00000066189	0	0	0	0	0	0	0	0	0	0
ENSMUSG00000106963	0	0	0	0	2	0	0	0	0	0
ENSMUSG00000106960	0	0	0	0	0	0	0	0	0	0
ENSMUSG00000088926	0	0	0	0	0	0	0	0	0	0
ENSMUSG00000088925	0	0	0	0	0	0	0	0	0	0
ENSMUSG00000088924	0	0	0	0	0	0	0	0	0	0
ENSMUSG00000088923	0	0	0	0	0	0	0	0	0	0
ENSMUSG00000088928	0	0	0	0	0	0	0	0	0	0
ENSMUSG00000096318	0	0	0	0	0	0	0	0	0	0
ENSMUSG00000096313	0	0	0	0	0	0	0	0	0	0
ENSMUSG00000096311	0	0	0	0	0	0	0	0	0	0
ENSMUSG00000096316	0	0	0	0	0	0	0	0	0	0
ENSMUSG00000096314	0	0	0	0	0	0	0	0	0	0
ENSMUSG00000082924	0	0	0	0	0	0	0	0	0	0
ENSMUSG00000113464	0	0	0	0	0	0	0	0	0	0
ENSMUSG00000113461	0	0	0	0	0	0	0	0	0	2
ENSMUSG00000113460	0	0	0	0	0	0	0	0	0	0
ENSMUSG00000113463	0	0	0	0	0	0	0	0	0	0
ENSMUSG00000034303	323	238	294	262	274	293	407	361	312	317
ENSMUSG00000108357	0	0	0	0	0	0	0	0	0	0
ENSMUSG00000108355	0	0	0	0	0	0	0	0	0	0
ENSMUSG00000034308	306	227	349	300	249	353	415	337	403	370
ENSMUSG00000113468	0	0	0	0	0	0	0	0	0	0
ENSMUSG00000108351	0	0	0	0	0	0	0	0	0	0
ENSMUSG00000111199	0	0	0	0	0	0	0	0	0	0
ENSMUSG00000093848	0	0	0	0	0	0	0	0	0	0
ENSMUSG00000109914	0	0	0	0	0	0	0	0	0	0
ENSMUSG00000111190	0	0	0	0	0	0	0	0	0	0
ENSMUSG00000114118	0	0	0	0	0	0	0	0	0	4
ENSMUSG00000104528	2	6	20	6	6	9	6	8	12	0
ENSMUSG00000104529	0	0	0	0	0	0	0	0	0	0
ENSMUSG00000104522	0	0	0	0	0	0	0	0	0	0
ENSMUSG00000098136	0	0	0	0	0	0	0	0	0	0
ENSMUSG00000098131	0	0	0	0	0	0	0	0	0	0
ENSMUSG00000023243	94	133	149	142	138	58	163	120	146	149
ENSMUSG00000095502	0	0	0	2	0	0	0	0	0	0
ENSMUSG00000098138	0	5	0	0	6	2	0	3	2	3
ENSMUSG00000096256	2	2	3	3	3	2	8	6	4	4
ENSMUSG00000114344	0	15	4	2	0	0	8	3	3	0
ENSMUSG00000114347	0	0	0	0	0	0	0	0	0	0
ENSMUSG00000026394	0	0	0	6	0	0	0	0	0	0
ENSMUSG00000106699	0	0	0	0	0	0	0	0	0	0
ENSMUSG00000082021	0	0	0	0	0	0	0	0	0	0
ENSMUSG00000026398	64	47	76	34	86	62	149	142	120	171
ENSMUSG00000082022	0	0	0	0	0	0	0	0	0	0

ENSMUSG00000044515	0	0	0	0	0	0	0	0	0	0
ENSMUSG00000082024	0	0	0	0	0	0	0	0	0	2
ENSMUSG00000044518	0	0	0	0	0	0	0	0	0	0
ENSMUSG00000044519	0	0	0	0	0	0	3	0	0	0
ENSMUSG00000082027	0	0	0	0	0	0	0	0	0	0
ENSMUSG00000109077	0	0	0	0	0	0	0	0	0	0
ENSMUSG00000095033	0	0	0	0	0	0	0	0	0	0
ENSMUSG00000045648	0	0	0	0	0	0	0	0	0	0
ENSMUSG00000090003	0	0	0	0	0	0	0	0	0	0
ENSMUSG00000095030	0	0	0	0	0	0	0	0	0	0
ENSMUSG00000095037	0	0	0	0	0	0	0	0	0	0
ENSMUSG00000045009	27	47	29	30	45	42	62	41	40	58
ENSMUSG00000095035	0	0	0	0	0	0	0	0	0	0
ENSMUSG00000095034	0	0	0	0	0	0	0	0	0	0
ENSMUSG00000095236	0	0	0	0	0	0	0	0	0	0
ENSMUSG00000053117	0	0	3	10	10	8	2	11	17	5
ENSMUSG00000080383	0	0	0	0	0	0	1	0	0	0
ENSMUSG00000107579	0	0	0	0	0	0	0	0	0	0
ENSMUSG00000080657	0	0	0	0	0	1	0	0	0	0
ENSMUSG00000080653	0	0	0	0	0	0	0	1	0	0
ENSMUSG00000106849	0	0	0	0	0	0	0	0	0	0
ENSMUSG00000080659	0	0	0	0	0	1	0	0	0	0
ENSMUSG00000005364	0	0	0	3	3	10	41	26	24	19
ENSMUSG00000099506	0	0	0	0	0	0	0	0	0	0
ENSMUSG00000081381	0	0	0	0	0	0	0	0	0	0
ENSMUSG00000081383	0	0	0	0	0	0	0	0	0	0
ENSMUSG00000081384	0	0	0	0	0	0	0	0	0	0
ENSMUSG00000081386	0	0	0	0	0	0	0	0	0	0
ENSMUSG00000073906	2	3	6	0	0	3	5	2	2	9
ENSMUSG00000098254	0	0	0	0	0	0	0	0	0	0
ENSMUSG00000081389	0	0	0	0	0	0	0	0	0	0
ENSMUSG00000098252	2	0	0	0	0	0	0	1	0	0
ENSMUSG00000028883	404	334	264	406	393	680	509	504	596	490
ENSMUSG00000035836	0	0	6	0	0	0	0	0	0	6
ENSMUSG00000101306	0	0	0	0	0	0	0	0	0	0
ENSMUSG00000101802	0	0	0	0	0	0	0	0	0	0
ENSMUSG00000098784	0	0	0	0	0	0	0	0	0	0
ENSMUSG00000061525	0	0	0	0	0	0	3	0	0	0
ENSMUSG00000098782	0	0	0	0	0	0	0	0	0	0
ENSMUSG00000073909	0	0	0	0	0	0	0	1	0	0
ENSMUSG00000098780	0	0	0	0	0	0	0	0	0	0
ENSMUSG00000047730	0	3	3	0	0	0	0	3	0	0
ENSMUSG00000031210	14	18	12	3	31	25	4	14	6	10
ENSMUSG00000031212	0	0	0	0	0	0	0	0	0	0
ENSMUSG00000101806	0	0	0	0	0	0	0	0	0	0
ENSMUSG00000093060	0	0	2	0	0	0	0	0	3	0
ENSMUSG00000093064	0	0	0	0	0	0	0	0	0	0
ENSMUSG00000093065	0	0	0	0	0	0	0	0	0	0
ENSMUSG00000052790	0	0	0	0	0	0	0	0	0	0
ENSMUSG00000081524	0	0	0	2	2	0	0	0	0	2
ENSMUSG00000081525	0	0	0	0	0	0	0	0	0	0
ENSMUSG00000081526	0	0	0	0	0	0	0	0	0	0
ENSMUSG00000081523	0	0	0	0	0	0	0	0	0	0
ENSMUSG00000081529	0	0	4	3	0	0	0	0	0	0
ENSMUSG00000077408	0	0	0	0	0	0	0	0	0	0
ENSMUSG00000077409	0	1	0	1	1	1	2	1	2	2
ENSMUSG00000077406	0	0	0	0	0	0	0	0	0	2
ENSMUSG00000077407	0	0	0	0	0	0	0	0	0	0

ENSMUSG00000077404	0	0	0	0	0	0	0	0	0	0
ENSMUSG00000077402	0	1	2	2	0	0	0	0	0	0
ENSMUSG00000077403	0	0	0	0	0	0	0	0	0	0
ENSMUSG00000040424	19	29	13	35	13	34	28	33	17	28
ENSMUSG00000106467	0	0	0	0	0	0	0	0	0	0
ENSMUSG00000004415	9	9	11	6	14	10	13	9	7	9
ENSMUSG00000095316	0	0	0	0	0	0	0	0	0	0
ENSMUSG00000030167	29	24	19	29	19	21	63	38	65	29
ENSMUSG00000030160	0	0	3	23	0	0	3	0	0	6
ENSMUSG00000030162	9	21	29	42	24	26	40	41	55	40
ENSMUSG00000048155	0	0	0	0	0	0	0	0	0	0
ENSMUSG00000048153	0	0	3	0	0	0	3	0	2	3
ENSMUSG00000092331	0	0	0	0	0	0	0	0	0	0
ENSMUSG00000092330	0	0	0	0	0	0	0	0	0	0
ENSMUSG00000092332	0	0	0	0	0	0	0	0	0	0
ENSMUSG00000092336	0	0	0	0	0	0	0	0	0	0
ENSMUSG00000092339	0	0	0	0	0	0	0	0	0	0
ENSMUSG00000092338	4	2	4	4	1	4	2	3	8	4
ENSMUSG00000106958	0	0	0	0	0	0	0	0	0	0
ENSMUSG00000092290	0	0	0	0	0	0	0	0	0	0
ENSMUSG00000072584	8	0	8	12	3	0	2	7	3	0
ENSMUSG00000070985	3	4	0	4	3	0	0	0	0	0
ENSMUSG00000070983	0	0	0	0	0	0	0	0	0	0
ENSMUSG00000070980	3	0	0	0	0	0	0	0	0	0
ENSMUSG00000040284	0	0	0	0	0	0	0	0	0	0
ENSMUSG00000082517	0	0	0	0	0	0	0	0	2	0
ENSMUSG00000043880	0	0	0	0	0	0	0	0	0	0
ENSMUSG00000041771	29	10	6	3	27	25	18	6	8	17
ENSMUSG00000100031	0	0	0	0	0	0	0	0	0	0
ENSMUSG00000100036	0	0	0	0	0	0	0	0	0	0
ENSMUSG00000068806	3	0	3	3	4	0	6	0	1	4
ENSMUSG00000068808	0	0	0	0	0	0	0	0	0	0
ENSMUSG00000070677	0	0	0	0	0	0	0	0	0	0
ENSMUSG00000095492	0	0	0	0	0	0	0	0	0	0
ENSMUSG00000064165	0	0	0	0	0	0	0	0	0	0
ENSMUSG00000095494	0	0	0	0	0	0	0	0	0	0
ENSMUSG00000084548	0	0	0	0	0	0	0	0	0	0
ENSMUSG00000084546	0	0	0	0	0	0	0	0	0	0
ENSMUSG00000075002	0	0	0	0	0	0	0	0	0	0
ENSMUSG00000024734	0	0	4	0	0	0	6	5	0	0
ENSMUSG00000085630	0	0	0	0	0	0	0	0	0	0
ENSMUSG00000107569	0	0	0	0	0	0	0	0	0	0
ENSMUSG00000107563	0	0	0	0	0	0	0	0	0	0
ENSMUSG00000107565	0	0	0	0	0	0	0	0	0	0
ENSMUSG00000107564	0	0	0	0	0	0	0	0	0	0
ENSMUSG00000065698	0	0	0	2	0	0	0	0	0	0
ENSMUSG00000033533	0	0	13	15	0	0	0	0	3	3
ENSMUSG00000065694	0	0	0	2	2	1	1	0	1	0
ENSMUSG00000061536	278	276	276	311	219	351	441	310	471	375
ENSMUSG00000101813	2	1	2	1	2	1	2	2	1	1
ENSMUSG00000101812	0	1	0	0	0	0	0	0	0	0
ENSMUSG00000101811	0	0	0	0	0	0	0	0	0	0
ENSMUSG00000101810	0	0	0	0	0	0	0	0	0	0
ENSMUSG00000101817	0	0	0	0	0	0	0	0	0	0
ENSMUSG00000101816	0	0	0	0	0	0	1	0	0	0
ENSMUSG00000101815	0	0	0	0	0	0	0	0	0	0
ENSMUSG00000101819	1	1	1	0	1	1	0	0	0	1
ENSMUSG00000101818	0	0	0	0	0	0	0	0	0	0

ENSMUSG00000074359	0	0	0	0	0	0	0	0	0	0
ENSMUSG00000089873	0	0	0	0	0	0	0	0	0	0
ENSMUSG00000103718	0	0	0	0	0	0	0	0	0	0
ENSMUSG00000103719	0	0	0	0	0	0	0	0	0	0
ENSMUSG00000075918	0	0	0	0	0	0	0	0	0	0
ENSMUSG00000103712	0	0	0	0	0	0	0	0	0	0
ENSMUSG00000103713	0	0	0	0	0	0	0	0	0	0
ENSMUSG00000103716	0	0	0	0	0	0	0	0	0	0
ENSMUSG00000103714	0	0	0	0	0	0	0	0	0	0
ENSMUSG00000065340	0	0	0	0	0	0	0	0	0	0
ENSMUSG00000068117	0	0	3	0	0	0	0	3	0	0
ENSMUSG00000065344	0	0	0	0	0	0	0	0	0	0
ENSMUSG00000068113	6	6	3	10	0	0	0	0	0	3
ENSMUSG00000065348	0	0	0	0	0	0	0	0	0	0
ENSMUSG00000075088	0	0	0	0	0	0	0	0	0	0
ENSMUSG00000078520	0	0	0	0	0	0	0	0	0	0
ENSMUSG00000078521	18	17	15	18	22	9	25	18	11	15
ENSMUSG00000089311	0	0	0	0	0	0	0	0	0	0
ENSMUSG00000089310	0	0	0	0	0	0	0	0	0	0
ENSMUSG00000083957	0	0	0	0	0	0	0	0	0	0
ENSMUSG00000030945	24	0	109	190	0	0	62	0	3	28
ENSMUSG00000089630	0	0	0	0	0	0	0	0	0	0
ENSMUSG00000089634	3	2	5	7	6	7	5	5	3	0
ENSMUSG00000083955	0	0	0	0	0	0	0	0	0	0
ENSMUSG00000083954	0	0	0	0	0	0	0	0	0	0
ENSMUSG00000097208	12	17	17	44	20	40	34	8	20	61
ENSMUSG00000097209	0	3	0	3	0	0	0	3	3	0
ENSMUSG00000073510	0	0	0	0	0	0	0	0	0	0
ENSMUSG00000097201	0	0	0	0	0	0	0	0	0	0
ENSMUSG00000063971	0	0	0	0	0	0	3	0	0	0
ENSMUSG00000083958	0	0	0	0	0	0	0	0	0	0
ENSMUSG00000075085	0	0	0	2	0	0	0	0	0	0
ENSMUSG00000035270	16	18	15	26	6	24	37	17	33	22
ENSMUSG00000035277	0	0	0	0	0	0	6	0	0	0
ENSMUSG00000062372	6	3	0	0	0	0	0	3	0	3
ENSMUSG00000048830	0	0	0	0	0	0	0	0	0	0
ENSMUSG00000048834	12	9	6	5	9	9	3	3	12	8
ENSMUSG00000077872	0	0	0	0	0	0	0	0	0	0
ENSMUSG00000069441	9	11	5	11	3	10	12	9	6	5
ENSMUSG00000102048	0	0	0	0	0	0	0	0	0	0
ENSMUSG00000102041	0	0	0	0	0	0	0	0	0	0
ENSMUSG00000102043	0	0	0	0	0	0	0	0	0	0
ENSMUSG00000102042	0	0	0	0	0	0	0	0	0	0
ENSMUSG00000102045	0	0	0	0	0	0	0	0	0	0
ENSMUSG00000102044	0	0	0	0	0	0	0	0	0	0
ENSMUSG00000102047	5	1	0	3	0	0	5	0	0	5
ENSMUSG00000102046	5	0	0	0	0	0	0	0	0	0
ENSMUSG00000096159	0	0	0	0	0	0	0	0	0	0
ENSMUSG00000096158	0	0	0	0	0	0	0	0	0	0
ENSMUSG00000096151	0	0	0	0	0	0	0	0	0	0
ENSMUSG00000089697	0	0	0	0	0	0	6	0	0	0
ENSMUSG00000096153	0	0	0	0	0	0	0	0	0	0
ENSMUSG00000096152	0	0	0	0	0	0	0	0	0	0
ENSMUSG00000096154	0	0	0	0	0	0	0	0	0	0
ENSMUSG00000096157	0	2	2	3	0	2	0	2	0	3
ENSMUSG00000091401	0	0	0	0	0	0	2	0	0	0
ENSMUSG00000063394	0	0	0	0	0	0	0	0	0	0
ENSMUSG00000069996	0	0	0	1	0	0	0	0	0	0

ENSMUSG00000063020	0	0	0	0	0	0	0	3	0	0
ENSMUSG00000032726	0	0	0	0	0	0	6	6	3	0
ENSMUSG00000021278	8	3	8	16	0	0	10	10	0	3
ENSMUSG00000105905	0	0	0	0	0	0	0	0	0	0
ENSMUSG00000105907	0	0	0	0	0	0	0	0	0	0
ENSMUSG00000105900	0	0	0	0	0	0	0	0	0	0
ENSMUSG00000105903	0	0	0	0	0	0	0	0	0	0
ENSMUSG00000057074	3	0	0	3	3	3	6	3	6	6
ENSMUSG00000020891	54	65	28	39	29	25	33	32	16	0
ENSMUSG00000060613	0	0	3	0	0	0	0	6	0	0
ENSMUSG00000104051	0	0	0	0	0	0	0	0	0	0
ENSMUSG00000101020	0	0	0	0	0	0	0	0	0	0
ENSMUSG00000101021	0	0	0	0	0	0	0	0	0	0
ENSMUSG00000101026	3	0	0	0	0	0	0	0	0	0
ENSMUSG00000101027	0	0	0	0	0	0	0	0	0	0
ENSMUSG00000060615	0	0	0	0	0	0	0	0	0	0
ENSMUSG00000101025	0	0	0	0	0	0	0	0	0	0
ENSMUSG00000104058	0	0	0	0	0	0	0	0	0	0
ENSMUSG00000104059	0	0	0	0	0	0	0	0	0	0
ENSMUSG00000101028	20	18	17	15	19	22	28	33	33	31
ENSMUSG00000101029	0	0	0	0	0	0	0	0	0	0
ENSMUSG00000089699	0	0	0	0	0	0	0	0	0	0
ENSMUSG00000059460	0	0	0	0	0	0	0	0	0	0
ENSMUSG00000091311	0	0	0	0	0	3	0	0	0	0
ENSMUSG00000083397	5	7	3	2	9	20	5	3	8	0
ENSMUSG00000091318	0	0	0	0	0	0	0	0	0	0
ENSMUSG00000109960	0	0	0	0	0	0	0	0	0	0
ENSMUSG00000102542	6	0	2	0	2	0	0	0	0	3
ENSMUSG00000094043	0	0	0	0	0	0	0	0	0	0
ENSMUSG00000108889	0	0	0	0	0	0	0	0	0	0
ENSMUSG00000110026	0	0	0	0	0	0	0	0	0	0
ENSMUSG00000108882	0	0	0	0	0	0	0	0	0	0
ENSMUSG00000112359	0	0	0	0	0	0	0	0	0	0
ENSMUSG00000056306	37	56	63	18	52	21	12	77	6	37
ENSMUSG00000026809	8	3	7	20	6	1	6	0	7	10
ENSMUSG00000026805	3	5	3	5	0	6	9	7	3	6
ENSMUSG00000026807	0	3	20	8	6	10	12	10	0	9
ENSMUSG00000021852	9	11	6	6	0	0	18	19	7	12
ENSMUSG00000100373	0	0	0	0	0	0	0	0	0	0
ENSMUSG00000100372	0	0	0	0	0	0	0	0	0	0
ENSMUSG00000100375	0	0	0	0	0	0	0	0	0	0
ENSMUSG00000100377	0	0	0	0	0	0	0	0	0	0
ENSMUSG00000100376	0	0	0	0	2	0	0	0	0	0
ENSMUSG00000100379	0	0	0	0	0	0	0	0	0	0
ENSMUSG00000095284	0	0	0	0	0	0	0	0	0	0
ENSMUSG00000110902	6	5	0	9	8	23	16	20	14	13
ENSMUSG00000110901	0	0	0	0	0	0	3	0	0	0
ENSMUSG00000105182	0	0	0	0	0	0	0	1	0	0
ENSMUSG00000110909	0	0	0	0	0	0	0	0	0	0
ENSMUSG00000013150	388	347	377	401	265	343	426	419	382	381
ENSMUSG00000068889	0	0	0	0	0	0	0	0	0	0
ENSMUSG00000088344	0	2	0	0	0	0	0	1	0	0
ENSMUSG00000088343	0	0	0	0	0	0	0	0	0	0
ENSMUSG00000088340	0	0	0	0	0	0	0	0	0	0
ENSMUSG00000046723	0	0	0	0	0	0	0	0	0	0
ENSMUSG00000086415	0	0	0	0	0	0	0	0	0	0
ENSMUSG00000083778	0	0	0	0	0	0	0	0	0	0
ENSMUSG00000086417	66	31	99	90	62	49	59	84	81	58

ENSMUSG00000086416	0	0	0	0	3	0	0	0	3	0
ENSMUSG00000086410	0	0	0	0	0	0	2	0	2	1
ENSMUSG00000086413	0	0	0	0	0	0	0	0	0	0
ENSMUSG00000095543	0	0	0	0	0	0	0	0	0	0
ENSMUSG00000095542	0	0	0	0	0	0	0	0	0	0
ENSMUSG00000095541	0	0	0	0	0	1	0	0	0	0
ENSMUSG00000095540	0	0	0	0	0	0	0	0	0	0
ENSMUSG00000095546	0	0	0	0	0	0	0	0	0	0
ENSMUSG00000095544	0	0	0	0	0	0	0	0	0	0
ENSMUSG00000095549	0	0	0	0	0	0	0	0	0	0
ENSMUSG00000093260	0	0	0	0	0	0	0	0	0	0
ENSMUSG00000093261	0	0	0	0	0	0	0	0	0	0
ENSMUSG00000027869	0	0	0	6	6	0	0	0	0	0
ENSMUSG00000027868	85	44	94	39	63	233	275	158	408	157
ENSMUSG00000052554	0	0	0	0	0	0	0	0	0	0
ENSMUSG00000052557	851	905	1057	917	794	1097	1430	1033	1120	1001
ENSMUSG00000097949	0	0	0	0	0	0	0	0	0	0
ENSMUSG00000097945	0	0	0	0	0	0	0	0	0	0
ENSMUSG00000097947	0	0	0	0	0	0	0	0	0	0
ENSMUSG00000097941	0	0	0	0	0	0	0	0	0	0
ENSMUSG00000097940	0	0	0	0	0	0	0	0	0	0
ENSMUSG00000097943	0	0	2	5	0	0	0	2	0	2
ENSMUSG00000103983	201	199	214	255	150	111	225	170	262	174
ENSMUSG00000112679	0	0	0	0	0	0	0	0	0	0
ENSMUSG00000112678	0	0	0	0	0	0	0	0	0	0
ENSMUSG00000004651	12	21	3	0	6	9	19	15	3	6
ENSMUSG00000112672	0	0	0	0	0	0	0	0	0	0
ENSMUSG00000112671	0	0	0	0	0	0	0	0	0	0
ENSMUSG00000112670	0	9	3	0	11	0	3	0	5	1
ENSMUSG00000112677	5	0	0	11	3	3	0	3	3	0
ENSMUSG00000004654	0	0	0	0	0	0	0	0	0	0
ENSMUSG00000055022	12	9	9	3	17	19	16	9	6	3
ENSMUSG00000024986	113	191	146	144	103	110	226	111	165	187
ENSMUSG00000018973	0	0	0	0	0	0	0	0	0	0
ENSMUSG00000060084	0	0	0	0	0	0	0	0	0	0
ENSMUSG00000024989	75	78	95	81	64	45	135	57	53	35
ENSMUSG00000106029	0	0	0	0	0	0	0	0	0	0
ENSMUSG00000106028	0	0	0	0	0	0	0	0	2	0
ENSMUSG00000106027	0	0	1	0	0	0	0	1	0	0
ENSMUSG00000106026	0	0	0	0	0	0	0	0	0	0
ENSMUSG00000106024	21	18	5	9	0	11	25	13	11	12
ENSMUSG00000106023	0	0	0	0	0	0	0	0	0	0
ENSMUSG00000106021	0	0	0	0	0	0	0	0	0	0
ENSMUSG00000106020	0	0	0	3	0	0	0	0	0	0
ENSMUSG00000028519	6	0	0	0	0	0	6	0	0	0
ENSMUSG00000110293	0	0	0	0	0	0	0	0	0	0
ENSMUSG00000110292	0	0	0	0	0	0	0	0	0	0
ENSMUSG00000110297	0	2	0	0	0	0	0	0	0	0
ENSMUSG00000110296	0	0	0	0	0	0	0	0	0	0
ENSMUSG00000110294	0	0	0	0	0	0	0	0	0	0
ENSMUSG00000110125	0	0	0	0	0	0	0	0	0	0
ENSMUSG00000110126	2	0	0	1	2	0	2	1	3	0
ENSMUSG00000110121	0	0	0	0	0	0	0	0	0	0
ENSMUSG00000025163	11	21	9	39	24	12	36	47	32	24
ENSMUSG00000110123	17	18	9	18	20	12	36	18	15	18
ENSMUSG00000110122	3	0	0	0	0	3	0	0	0	0
ENSMUSG00000100391	0	0	0	0	0	0	0	0	0	0
ENSMUSG00000077869	0	0	0	0	0	0	0	0	0	0

ENSMUSG00000097683	0	0	0	0	0	0	0	0	0	0
ENSMUSG00000087084	0	0	0	0	0	0	0	0	0	0
ENSMUSG00000003354	9	8	39	11	19	23	12	21	14	33
ENSMUSG00000051237	0	0	3	0	0	0	0	0	0	0
ENSMUSG00000105598	4	5	6	9	1	5	3	2	4	4
ENSMUSG00000003352	228	296	281	191	182	261	548	353	462	355
ENSMUSG00000105592	0	0	0	0	0	0	0	0	0	0
ENSMUSG00000105593	0	0	0	0	0	0	0	0	0	0
ENSMUSG00000105590	6	0	6	1	9	6	6	9	3	0
ENSMUSG00000105591	0	0	0	3	0	0	0	0	0	0
ENSMUSG00000114636	0	0	0	0	0	0	0	0	0	0
ENSMUSG00000114630	3	0	1	1	0	1	2	2	0	0
ENSMUSG00000114639	0	0	0	0	0	0	0	3	0	3
ENSMUSG00000082882	0	0	0	0	0	0	0	0	0	0
ENSMUSG00000060081	0	0	0	0	0	0	0	0	0	0
ENSMUSG00000082886	0	0	0	0	0	0	0	0	0	0
ENSMUSG00000082887	0	0	0	0	0	0	0	0	0	0
ENSMUSG00000082889	0	0	0	0	0	0	0	0	0	0
ENSMUSG00000050545	183	192	203	245	218	215	225	234	208	226
ENSMUSG00000070471	4	3	6	0	10	8	14	0	2	0
ENSMUSG00000108147	0	0	0	0	0	0	0	0	0	0
ENSMUSG00000108144	0	0	0	0	0	0	0	0	0	0
ENSMUSG00000108145	7	0	15	0	0	0	0	3	3	3
ENSMUSG00000108140	0	0	0	0	0	0	0	0	0	0
ENSMUSG00000105048	3	0	0	0	3	0	0	0	0	0
ENSMUSG00000105040	0	0	0	0	0	0	0	0	0	0
ENSMUSG00000105041	15	2	3	3	0	9	9	3	2	6
ENSMUSG00000105043	0	0	0	0	0	0	0	0	0	0
ENSMUSG00000105044	14	4	11	10	5	12	18	18	15	6
ENSMUSG00000105045	3	0	0	0	0	7	0	0	2	0
ENSMUSG00000105047	0	3	0	0	0	1	0	1	0	0
ENSMUSG00000111656	2	0	3	23	9	7	15	1	12	6
ENSMUSG00000111657	0	0	0	0	0	2	0	0	0	0
ENSMUSG00000101666	0	0	0	0	0	0	0	0	0	0
ENSMUSG00000111652	6	0	0	3	0	0	0	0	0	0
ENSMUSG00000111658	0	0	0	0	0	0	0	0	0	0
ENSMUSG00000057534	0	0	0	0	5	0	0	0	0	0
ENSMUSG00000037995	73	70	64	113	48	66	75	70	96	79
ENSMUSG00000025838	0	0	0	0	0	0	0	0	0	0
ENSMUSG00000025839	0	0	0	0	0	0	0	0	0	0
ENSMUSG00000085824	0	0	0	0	0	0	0	0	0	0
ENSMUSG00000104317	0	0	0	0	0	0	0	2	3	0
ENSMUSG00000104314	0	0	0	0	0	0	0	0	0	0
ENSMUSG00000104313	0	0	0	0	0	0	0	0	0	0
ENSMUSG00000104312	0	0	0	0	0	0	0	0	0	0
ENSMUSG00000104311	0	0	0	0	0	0	0	0	0	0
ENSMUSG00000104310	0	0	0	0	0	0	0	0	0	0
ENSMUSG00000104319	0	0	0	0	0	0	0	0	0	0
ENSMUSG00000017922	0	0	0	0	0	0	0	0	0	0
ENSMUSG00000076117	0	0	1	0	0	0	0	0	1	1
ENSMUSG00000089253	1	1	0	2	0	0	1	2	0	0
ENSMUSG00000044724	0	0	0	0	0	0	17	0	0	0
ENSMUSG00000044726	0	0	3	0	2	0	0	0	0	0
ENSMUSG00000053091	288	364	280	310	229	294	327	365	177	385
ENSMUSG00000083828	0	0	0	0	0	0	0	0	0	0
ENSMUSG00000001444	11	21	18	24	11	18	39	20	36	27
ENSMUSG00000083821	0	0	0	0	0	0	0	0	0	0
ENSMUSG00000083825	0	0	0	0	0	0	0	0	0	0

ENSMUSG00000042894	0	0	0	0	0	0	0	0	0	0
ENSMUSG00000027478	148	126	124	149	101	88	146	140	165	150
ENSMUSG00000092906	0	0	0	0	0	0	0	0	0	0
ENSMUSG00000027470	15	12	8	11	14	31	84	25	41	31
ENSMUSG00000078057	0	0	0	0	0	0	0	0	0	0
ENSMUSG00000099496	0	0	0	0	0	0	0	0	0	0
ENSMUSG00000090215	62	7	31	87	26	19	34	30	67	40
ENSMUSG00000077972	0	0	0	0	0	0	0	0	0	0
ENSMUSG00000086824	3	0	0	3	3	12	17	12	2	10
ENSMUSG00000098429	0	0	0	0	0	0	0	0	0	0
ENSMUSG00000098423	0	0	0	0	0	0	0	0	0	0
ENSMUSG00000098421	0	0	0	0	0	0	0	0	0	0
ENSMUSG00000098420	0	0	0	0	0	0	0	0	0	0
ENSMUSG00000098425	0	2	0	0	2	0	0	0	1	0
ENSMUSG00000098424	55	0	0	27	37	22	19	37	27	35
ENSMUSG00000042761	3	3	3	0	6	0	0	8	0	0
ENSMUSG00000032978	10	3	0	15	0	0	0	0	12	0
ENSMUSG00000095514	0	0	0	0	0	0	0	0	0	0
ENSMUSG00000074882	0	0	0	0	0	0	0	0	0	0
ENSMUSG00000046650	0	0	0	0	0	0	0	0	0	0
ENSMUSG00000074887	0	0	0	0	0	0	0	0	0	0
ENSMUSG00000074885	9	0	0	3	6	21	5	13	6	2
ENSMUSG00000086995	0	0	0	0	0	0	14	0	9	17
ENSMUSG00000086996	0	0	0	0	0	0	0	0	0	0
ENSMUSG00000086997	4	2	9	5	4	12	17	5	6	3
ENSMUSG00000086999	0	0	0	0	0	0	0	0	0	0
ENSMUSG00000052058	0	0	0	0	0	0	0	0	0	0
ENSMUSG00000111095	0	0	0	0	0	0	0	0	0	0
ENSMUSG00000093747	0	0	0	0	0	0	0	0	0	0
ENSMUSG00000093746	1	0	1	0	1	0	0	0	0	0
ENSMUSG00000043388	0	0	3	11	3	32	35	9	3	23
ENSMUSG00000093743	0	0	0	0	0	0	0	0	0	0
ENSMUSG00000093741	0	0	0	0	0	0	0	0	0	0
ENSMUSG00000093740	4	0	0	0	0	2	0	6	0	0
ENSMUSG00000043383	0	0	0	0	0	0	0	0	0	0
ENSMUSG00000049985	53	27	76	62	34	55	90	42	56	42
ENSMUSG00000043385	9	9	8	9	9	12	6	19	9	2
ENSMUSG00000101899	0	0	0	0	0	0	0	0	0	0
ENSMUSG00000101898	0	0	0	0	0	0	0	0	0	0
ENSMUSG00000028813	0	0	0	0	0	0	0	0	0	0
ENSMUSG00000049670	67	66	45	48	45	81	110	110	82	81
ENSMUSG00000049676	23	20	21	41	14	27	73	38	75	44
ENSMUSG00000033316	0	3	6	12	3	0	6	6	6	0
ENSMUSG00000103620	5	3	8	2	0	2	4	3	0	6
ENSMUSG00000101220	0	0	0	0	0	0	0	0	0	0
ENSMUSG00000101221	0	0	0	0	0	0	0	0	0	0
ENSMUSG00000101222	0	0	0	0	0	0	0	0	0	0
ENSMUSG00000101223	4	10	1	19	14	8	4	3	14	4
ENSMUSG00000101224	0	0	0	0	0	0	0	0	2	0
ENSMUSG00000101226	0	0	0	0	0	0	3	0	0	3
ENSMUSG00000101227	0	0	0	0	0	0	0	0	0	0
ENSMUSG00000101229	0	0	0	0	0	0	0	0	0	0
ENSMUSG00000096852	8	2	3	30	0	0	5	0	0	0
ENSMUSG00000088890	0	0	0	0	0	0	0	0	0	0
ENSMUSG00000088896	0	0	0	0	0	0	0	0	0	0
ENSMUSG00000051528	0	0	0	0	0	0	0	3	0	0
ENSMUSG00000087112	0	0	7	0	0	0	0	0	0	3
ENSMUSG00000087117	0	0	0	0	0	0	0	0	3	0

ENSMUSG00000087116	0	0	0	0	0	0	0	0	0	0
ENSMUSG00000087115	0	0	0	0	0	0	0	0	0	0
ENSMUSG00000087114	0	0	0	0	0	0	0	0	0	0
ENSMUSG00000099464	0	0	0	0	0	0	0	0	0	0
ENSMUSG00000101598	0	0	0	0	0	0	0	0	0	0
ENSMUSG00000099467	0	0	0	0	0	0	0	0	0	0
ENSMUSG00000099460	0	0	0	0	0	0	0	0	0	0
ENSMUSG00000099461	0	0	0	0	0	0	0	0	0	0
ENSMUSG00000099462	0	0	0	0	0	0	0	0	0	0
ENSMUSG00000099463	0	0	0	0	0	0	0	0	0	0
ENSMUSG00000101592	0	0	0	0	0	0	0	0	0	0
ENSMUSG00000101593	0	0	0	0	0	0	0	0	0	0
ENSMUSG00000101590	0	0	0	0	0	0	0	0	0	0
ENSMUSG00000099468	0	0	0	0	0	0	0	0	0	0
ENSMUSG00000099469	0	0	0	0	3	0	0	0	0	0
ENSMUSG00000014704	33	18	21	27	9	44	52	31	71	56
ENSMUSG00000101595	0	0	0	6	0	0	3	0	9	0
ENSMUSG00000096591	4	5	1	2	3	1	7	4	2	4
ENSMUSG00000094661	11	8	11	14	10	19	31	12	39	13
ENSMUSG00000082699	0	0	0	7	0	3	0	0	0	0
ENSMUSG00000082698	0	0	0	0	0	0	0	0	0	0
ENSMUSG00000082696	0	0	0	0	0	0	0	0	0	0
ENSMUSG00000100175	0	0	2	3	0	0	0	0	0	0
ENSMUSG00000100174	20	12	6	9	11	35	18	18	50	12
ENSMUSG00000089094	0	0	0	0	0	0	0	0	0	0
ENSMUSG00000030468	3	6	12	5	12	20	79	31	53	65
ENSMUSG00000100171	0	0	0	0	0	0	0	0	0	0
ENSMUSG00000082690	0	0	0	0	0	0	0	0	0	0
ENSMUSG00000094662	0	0	0	0	0	0	0	0	0	0
ENSMUSG00000094889	0	0	0	0	0	0	0	0	0	0
ENSMUSG00000042031	0	0	0	0	0	0	0	0	0	0
ENSMUSG00000083128	0	0	0	0	0	0	0	0	0	0
ENSMUSG00000083129	0	0	0	0	0	0	0	0	0	0
ENSMUSG00000083126	0	0	0	0	0	0	0	0	0	0
ENSMUSG00000083124	0	0	0	0	0	0	0	0	0	0
ENSMUSG00000050635	0	0	0	0	0	3	3	6	3	0
ENSMUSG00000089187	0	0	0	0	0	0	0	0	0	0
ENSMUSG00000083120	0	0	0	0	0	0	0	0	0	0
ENSMUSG00000000183	33	33	69	15	22	113	227	120	152	56
ENSMUSG00000094886	0	0	0	0	0	0	1	0	0	0
ENSMUSG00000094881	0	0	0	0	0	0	0	0	0	0
ENSMUSG00000041523	6	0	0	3	0	3	3	0	0	0
ENSMUSG00000094880	0	0	0	0	0	1	1	0	0	0
ENSMUSG00000111034	3	0	4	3	2	11	3	0	6	4
ENSMUSG00000064739	0	0	0	0	0	0	0	0	0	0
ENSMUSG00000060890	1	5	4	0	0	7	15	1	5	2
ENSMUSG00000064732	0	0	0	0	0	0	0	0	0	0
ENSMUSG00000064735	0	0	0	0	0	0	0	0	0	0
ENSMUSG00000064737	0	0	0	0	0	0	0	0	0	0
ENSMUSG00000101016	0	0	0	0	0	0	0	0	1	0
ENSMUSG00000107334	0	0	5	0	0	5	3	0	3	0
ENSMUSG00000107336	38	17	35	50	29	59	101	65	71	27
ENSMUSG00000107337	0	0	0	0	0	0	0	0	0	0
ENSMUSG00000079598	51	39	30	32	36	40	91	84	14	9
ENSMUSG00000079594	0	0	0	0	0	0	0	0	0	0
ENSMUSG00000079595	0	0	0	0	0	0	0	0	0	0
ENSMUSG00000087801	0	0	0	0	0	0	2	0	0	0
ENSMUSG00000087800	0	0	0	0	0	0	0	0	0	0

ENSMUSG00000087802	0	0	0	0	0	0	0	0	0	0
ENSMUSG00000087805	0	0	0	0	0	0	1	0	0	0
ENSMUSG00000087804	0	0	0	0	0	0	0	0	0	0
ENSMUSG00000087806	0	0	0	0	0	0	0	0	0	0
ENSMUSG00000085407	16	3	15	0	11	15	21	3	14	6
ENSMUSG00000091497	0	0	0	0	0	0	0	0	0	0
ENSMUSG00000085405	0	0	0	0	0	0	0	0	0	0
ENSMUSG00000085404	0	0	0	0	0	0	0	0	0	0
ENSMUSG00000074146	0	0	3	0	0	0	0	0	3	0
ENSMUSG00000091491	0	0	0	0	0	0	0	0	0	0
ENSMUSG00000015890	0	0	3	12	3	0	6	6	6	0
ENSMUSG00000091490	0	3	0	0	0	0	0	0	0	0
ENSMUSG00000036449	0	0	0	3	4	0	0	0	4	4
ENSMUSG00000079042	6	3	3	6	3	6	3	5	3	0
ENSMUSG00000088750	0	0	0	0	0	0	0	0	0	0
ENSMUSG00000088751	0	0	0	0	0	0	0	0	0	0
ENSMUSG00000088754	0	0	0	0	0	0	0	0	0	0
ENSMUSG00000094968	0	0	0	0	0	0	0	0	0	0
ENSMUSG00000094963	0	0	0	0	0	0	0	0	0	0
ENSMUSG00000094960	0	0	0	0	0	0	0	0	0	0
ENSMUSG00000077799	1	0	0	0	0	0	0	0	0	0
ENSMUSG00000094967	0	0	0	0	0	0	0	0	0	0
ENSMUSG00000094964	0	0	0	0	0	0	0	0	0	0
ENSMUSG00000094965	0	0	0	0	0	0	0	0	0	0
ENSMUSG00000104277	9	6	4	3	2	17	23	17	2	12
ENSMUSG00000062497	0	0	0	0	0	0	0	0	0	0
ENSMUSG00000050504	0	0	0	0	0	0	0	0	0	0
ENSMUSG00000061769	0	0	0	0	3	0	0	0	6	0
ENSMUSG00000105915	0	0	0	0	0	0	0	0	0	0
ENSMUSG00000059203	0	0	0	0	0	0	0	0	0	0
ENSMUSG00000059206	0	0	0	0	0	0	0	0	0	0
ENSMUSG00000063728	0	0	0	0	0	0	0	0	0	0
ENSMUSG00000078315	0	0	0	0	0	0	0	0	0	0
ENSMUSG00000078314	0	2	11	3	0	2	2	9	6	9
ENSMUSG00000024215	6	0	0	5	0	0	3	3	3	3
ENSMUSG00000024211	0	6	6	0	3	3	2	0	3	0
ENSMUSG00000103528	0	0	0	0	0	0	0	0	0	0
ENSMUSG00000103521	0	0	0	0	0	0	0	0	0	0
ENSMUSG00000107626	0	0	0	0	0	0	0	0	0	0
ENSMUSG00000103523	0	0	0	0	0	0	0	0	0	0
ENSMUSG00000103524	0	0	0	0	0	0	0	0	0	0
ENSMUSG00000103526	0	0	0	0	0	0	0	0	0	2
ENSMUSG00000022900	3	3	5	9	3	14	29	24	20	6
ENSMUSG00000022902	0	0	0	0	0	6	0	0	0	0
ENSMUSG00000097435	0	0	0	0	5	3	6	3	5	0
ENSMUSG00000097434	3	0	0	0	0	0	6	0	0	0
ENSMUSG00000097437	0	0	0	0	0	0	0	0	0	0
ENSMUSG00000051037	182	150	199	271	164	250	371	194	285	203
ENSMUSG00000051036	0	0	0	0	0	0	0	0	0	0
ENSMUSG00000051034	361	388	512	390	302	325	445	418	382	387
ENSMUSG00000051038	0	0	0	0	0	0	0	0	0	0
ENSMUSG00000100951	0	0	0	0	0	0	0	0	0	0
ENSMUSG00000105396	0	0	0	0	0	0	0	0	0	0
ENSMUSG00000100953	0	0	0	0	0	0	0	0	0	0
ENSMUSG00000100952	0	0	0	0	0	0	0	0	0	0
ENSMUSG00000114437	0	0	0	0	0	0	0	0	0	0
ENSMUSG00000100957	0	0	0	0	0	0	0	0	0	0
ENSMUSG00000114434	0	0	0	0	0	0	0	0	0	0

ENSMUSG00000100959	0	0	0	0	0	0	0	0	3	0
ENSMUSG00000114439	0	0	0	0	0	0	0	0	0	0
ENSMUSG00000058550	0	0	0	0	0	0	0	0	0	0
ENSMUSG00000050783	0	0	0	0	0	0	0	0	0	0
ENSMUSG00000054200	9	0	0	0	11	15	124	15	59	21
ENSMUSG00000050788	2	21	9	9	15	6	11	20	11	32
ENSMUSG00000093827	4	1	0	0	0	4	0	0	0	0
ENSMUSG00000051788	0	0	0	0	0	0	0	0	0	0
ENSMUSG00000105206	0	0	0	0	0	0	0	0	0	0
ENSMUSG00000105205	0	0	0	0	0	0	0	0	0	0
ENSMUSG00000114389	0	0	0	5	0	0	0	0	3	3
ENSMUSG00000114388	0	0	0	0	0	0	0	0	0	0
ENSMUSG00000114385	0	0	0	0	0	0	0	0	0	0
ENSMUSG00000114384	0	0	0	0	0	0	0	0	0	0
ENSMUSG00000093824	0	0	0	0	0	0	0	0	0	0
ENSMUSG00000114381	0	0	0	0	0	0	0	0	0	0
ENSMUSG00000114383	0	0	0	0	0	0	0	0	0	0
ENSMUSG00000105209	0	0	0	0	0	0	0	0	0	0
ENSMUSG00000066803	0	0	0	0	0	0	0	0	0	0
ENSMUSG00000066804	0	0	0	0	0	0	0	0	0	0
ENSMUSG00000091873	0	0	0	0	0	0	0	0	0	0
ENSMUSG00000065570	0	1	0	1	2	0	4	0	1	1
ENSMUSG00000091879	0	0	0	0	0	0	0	0	0	0
ENSMUSG00000086791	0	0	0	0	0	0	0	0	0	0
ENSMUSG00000080300	0	0	0	0	0	0	0	0	0	0
ENSMUSG00000065572	0	0	0	0	0	0	0	0	0	0
ENSMUSG00000071714	78	64	126	187	77	364	267	218	402	154
ENSMUSG00000106395	2	5	2	2	1	4	4	7	3	1
ENSMUSG00000065574	0	1	0	0	0	0	0	0	0	1
ENSMUSG00000096473	0	0	1	0	0	0	0	1	2	1
ENSMUSG00000096470	0	0	0	0	0	0	0	0	0	0
ENSMUSG00000096476	0	0	0	0	0	0	0	0	0	0
ENSMUSG00000096477	0	0	0	0	0	0	0	1	0	0
ENSMUSG00000096475	0	0	0	0	0	0	0	0	0	0
ENSMUSG00000096479	0	2	4	0	0	2	0	0	0	0
ENSMUSG00000108434	20	30	12	21	9	15	15	11	24	11
ENSMUSG00000108432	0	0	0	0	0	0	0	0	0	0
ENSMUSG00000108433	0	0	0	0	0	0	0	0	0	0
ENSMUSG00000108430	0	0	0	0	0	0	0	0	0	0
ENSMUSG00000108431	0	0	0	0	0	0	0	0	0	0
ENSMUSG00000108438	0	0	2	0	0	0	0	0	0	0
ENSMUSG00000071716	29	3	11	26	0	12	67	22	46	17
ENSMUSG00000026109	50	85	46	38	26	97	225	78	115	72
ENSMUSG00000021337	0	0	0	0	0	0	0	0	0	0
ENSMUSG00000021336	0	3	0	0	0	0	0	0	0	0
ENSMUSG00000021335	0	0	3	11	0	0	0	0	4	11
ENSMUSG00000026100	67	17	70	6	45	129	45	72	20	67
ENSMUSG00000081442	0	0	0	0	0	0	0	0	0	0
ENSMUSG00000106677	0	0	0	0	0	0	0	0	0	0
ENSMUSG00000091296	0	0	0	0	0	0	0	0	0	0
ENSMUSG00000091293	0	0	0	0	0	0	0	0	0	2
ENSMUSG00000045030	0	0	0	0	3	0	0	0	0	0
ENSMUSG00000000982	9	15	36	74	24	112	35	31	64	14
ENSMUSG00000045034	9	6	5	0	5	0	8	0	5	3
ENSMUSG00000111265	0	0	0	0	0	0	0	0	0	0
ENSMUSG00000111267	0	0	0	0	0	0	0	0	0	0
ENSMUSG00000111266	0	0	0	0	0	0	0	0	0	0
ENSMUSG00000111263	0	0	0	0	0	0	0	0	0	0

ENSMUSG00000111262	0	0	0	0	0	0	0	0	0	0
ENSMUSG00000027270	0	0	0	0	0	0	0	0	0	0
ENSMUSG00000027273	0	0	0	0	3	0	3	6	3	0
ENSMUSG00000091129	0	0	0	0	0	0	0	0	0	0
ENSMUSG00000017000	0	0	0	0	0	0	0	0	0	0
ENSMUSG00000017003	0	0	0	0	0	0	0	0	0	0
ENSMUSG00000017004	0	0	0	0	0	0	0	3	9	0
ENSMUSG00000111454	0	0	0	0	0	0	0	0	0	0
ENSMUSG00000098172	0	0	0	0	0	0	3	0	0	0
ENSMUSG00000099730	0	0	0	0	0	0	0	0	0	0
ENSMUSG00000014603	0	0	0	0	0	0	6	0	0	0
ENSMUSG00000090457	0	0	0	0	0	0	0	0	0	0
ENSMUSG00000090455	0	0	0	0	0	0	0	0	0	0
ENSMUSG00000081779	1	0	0	0	0	0	0	0	0	0
ENSMUSG00000081778	0	0	0	0	0	0	0	0	0	0
ENSMUSG00000090451	3	3	5	3	6	2	3	4	9	2
ENSMUSG00000081775	0	0	0	0	0	0	2	0	0	0
ENSMUSG00000081774	0	0	0	0	0	0	0	0	0	0
ENSMUSG00000081777	2	3	2	0	1	0	0	3	0	0
ENSMUSG00000081771	0	6	0	0	0	0	6	3	6	0
ENSMUSG00000081770	0	0	0	0	0	0	0	0	0	0
ENSMUSG00000103213	0	0	0	0	0	0	6	0	0	0
ENSMUSG00000053522	0	13	4	4	9	20	67	22	8	18
ENSMUSG00000053528	0	0	0	0	0	0	0	0	0	0
ENSMUSG00000042567	0	17	8	3	14	9	29	12	14	12
ENSMUSG00000094513	0	0	0	0	0	0	0	0	0	0
ENSMUSG00000094511	0	0	0	0	0	0	0	0	0	0
ENSMUSG00000094510	0	0	0	0	0	0	0	0	0	0
ENSMUSG00000094516	0	0	0	0	0	0	0	0	0	0
ENSMUSG00000094515	0	0	0	0	0	0	0	0	0	0
ENSMUSG00000094514	0	0	0	0	0	0	3	2	3	0
ENSMUSG00000111841	0	6	0	0	0	0	0	0	0	0
ENSMUSG00000111843	0	0	0	0	0	0	0	0	0	0
ENSMUSG00000044814	3	5	0	9	0	13	17	8	9	0
ENSMUSG00000111849	0	0	0	0	0	0	0	0	0	0
ENSMUSG00000090989	0	0	0	0	0	0	0	0	0	0
ENSMUSG00000044819	0	0	0	0	0	0	0	0	0	3
ENSMUSG00000008601	8	7	0	0	0	5	6	0	4	0
ENSMUSG00000051176	0	0	0	0	0	0	0	0	0	0
ENSMUSG00000093187	0	0	0	0	0	0	0	0	0	0
ENSMUSG00000046719	26	18	15	42	11	6	93	18	22	23
ENSMUSG00000093180	0	0	0	0	0	0	0	0	0	0
ENSMUSG00000093183	0	0	0	0	0	0	0	0	0	0
ENSMUSG00000086204	0	0	0	0	0	0	0	0	0	0
ENSMUSG00000086205	0	0	0	0	3	0	0	2	0	0
ENSMUSG00000086207	0	0	0	0	0	0	0	0	0	0
ENSMUSG00000046716	0	0	0	0	0	0	0	0	0	0
ENSMUSG00000046717	0	0	0	0	0	0	0	0	0	0
ENSMUSG00000086202	0	0	0	0	0	0	0	0	0	0
ENSMUSG00000086203	0	0	0	0	0	0	0	0	0	0
ENSMUSG00000051172	0	0	0	0	0	0	0	0	0	0
ENSMUSG00000081252	0	4	0	0	0	0	0	0	0	0
ENSMUSG00000028236	0	0	0	0	0	0	0	0	0	0
ENSMUSG00000028238	9	9	13	32	6	67	32	24	80	14
ENSMUSG00000109538	0	0	0	0	0	0	0	0	0	0
ENSMUSG00000029544	248	332	366	163	178	246	322	244	337	451
ENSMUSG00000029546	0	0	0	0	0	0	0	0	0	0
ENSMUSG00000029541	2	2	0	3	0	0	2	2	3	0

ENSMUSG00000114576	0	0	0	0	0	5	4	2	0	2
ENSMUSG00000092588	0	0	0	0	0	0	0	0	0	0
ENSMUSG00000092583	0	0	0	0	0	0	0	0	0	0
ENSMUSG00000092580	0	0	0	0	0	0	0	0	0	0
ENSMUSG00000114574	0	0	0	0	0	0	0	0	0	0
ENSMUSG00000092584	0	0	0	0	0	0	0	0	0	0
ENSMUSG00000100850	0	0	0	0	0	0	0	0	0	0
ENSMUSG00000114573	22	24	14	16	12	23	13	16	27	17
ENSMUSG00000057195	0	0	0	0	0	0	0	0	0	0
ENSMUSG00000114570	0	0	0	0	0	0	0	0	0	0
ENSMUSG00000071665	0	0	0	0	0	0	0	0	0	0
ENSMUSG00000096808	0	2	0	0	3	1	1	6	0	4
ENSMUSG00000096803	0	0	0	0	0	0	0	0	0	0
ENSMUSG00000114838	0	0	0	0	0	4	0	4	0	5
ENSMUSG00000096800	0	0	0	0	0	0	0	0	0	0
ENSMUSG00000096804	0	0	0	0	0	0	0	0	0	0
ENSMUSG00000076126	0	0	0	0	0	0	0	0	0	0
ENSMUSG00000076120	0	0	0	0	0	0	0	0	0	0
ENSMUSG00000106276	0	0	0	0	0	0	0	0	0	0
ENSMUSG00000106274	0	0	0	0	0	0	0	0	0	0
ENSMUSG00000106275	0	0	0	0	0	0	0	0	0	0
ENSMUSG00000081361	0	0	0	0	0	0	0	0	0	0
ENSMUSG00000106270	0	0	0	0	0	0	0	0	0	0
ENSMUSG00000047048	0	9	6	3	0	0	1	3	0	4
ENSMUSG00000047046	0	0	0	0	0	0	0	0	0	0
ENSMUSG00000103219	9	0	6	8	0	8	5	5	4	9
ENSMUSG00000026830	0	0	0	0	5	3	9	0	3	0
ENSMUSG00000109426	0	0	0	0	0	0	0	0	0	0
ENSMUSG00000109424	0	0	0	3	0	0	3	0	0	0
ENSMUSG00000109422	3	3	0	0	0	0	9	0	3	0
ENSMUSG00000109423	0	0	0	0	0	0	0	0	0	0
ENSMUSG00000109421	0	0	0	0	0	0	0	0	0	0
ENSMUSG00000102661	0	0	0	0	0	0	0	0	0	0
ENSMUSG00000026832	199	206	180	531	138	383	659	325	520	372
ENSMUSG00000082459	0	0	0	0	0	0	0	0	0	0
ENSMUSG00000020545	0	0	0	0	0	0	0	0	0	0
ENSMUSG00000026835	0	0	0	0	3	0	2	0	0	0
ENSMUSG00000082451	0	0	0	0	0	0	0	0	0	0
ENSMUSG00000082450	0	0	0	0	0	0	0	0	0	0
ENSMUSG00000082453	0	0	0	0	0	0	0	0	0	0
ENSMUSG00000026834	86	36	18	6	24	221	1849	276	1136	372
ENSMUSG00000050996	0	0	0	0	0	0	0	0	0	0
ENSMUSG00000050994	13	21	17	25	20	34	37	36	41	15
ENSMUSG00000102667	0	0	0	0	0	0	0	0	0	0
ENSMUSG00000098875	0	0	0	0	0	0	0	0	0	0
ENSMUSG00000084118	0	0	0	0	0	0	0	0	0	0
ENSMUSG00000084119	0	0	0	0	0	0	0	0	0	0
ENSMUSG00000083329	0	0	0	0	0	0	0	0	0	0
ENSMUSG00000084112	0	0	0	0	0	0	0	0	0	0
ENSMUSG00000084110	2	0	0	2	0	0	2	0	0	0
ENSMUSG00000083320	0	0	0	0	0	0	0	0	0	0
ENSMUSG00000084117	0	0	0	0	0	3	0	0	0	0
ENSMUSG00000084114	0	0	0	0	0	0	0	0	0	0
ENSMUSG00000084115	6	0	0	5	3	12	6	6	3	0
ENSMUSG00000112800	72	100	59	42	92	112	74	107	37	62
ENSMUSG00000061959	50	22	37	19	18	38	14	28	24	40
ENSMUSG00000100369	0	0	0	0	0	0	0	0	0	0
ENSMUSG00000054523	0	0	0	0	0	0	0	0	1	0

ENSMUSG00000099059	1	1	1	1	1	1	1	1	1	1
ENSMUSG00000108669	0	0	0	0	0	0	0	0	0	0
ENSMUSG00000070203	0	0	0	0	0	0	0	0	0	0
ENSMUSG00000099054	0	0	0	0	0	0	0	0	0	0
ENSMUSG00000099057	0	0	0	0	0	0	0	0	0	0
ENSMUSG00000112807	0	0	0	0	0	0	0	0	0	0
ENSMUSG00000099051	7	4	6	3	5	4	16	8	3	3
ENSMUSG00000099053	0	0	0	0	0	0	0	0	0	0
ENSMUSG00000100363	0	0	0	0	0	0	0	0	0	0
ENSMUSG00000040907	18	27	12	89	22	60	195	41	56	82
ENSMUSG00000040901	0	0	0	0	0	0	0	0	0	0
ENSMUSG00000108666	0	0	0	0	0	0	0	0	0	0
ENSMUSG00000000617	0	0	0	0	0	0	3	0	0	0
ENSMUSG00000068431	0	0	0	0	0	0	0	0	0	0
ENSMUSG00000044021	0	3	9	9	0	3	14	0	0	3
ENSMUSG00000044026	253	273	319	330	232	344	318	422	445	424
ENSMUSG00000044024	18	8	3	17	0	0	25	15	8	4
ENSMUSG00000044025	0	0	0	0	0	0	0	0	0	0
ENSMUSG00000044029	3	3	3	0	0	3	3	0	0	0
ENSMUSG00000085248	0	0	0	0	0	0	0	0	0	0
ENSMUSG00000070258	0	0	0	0	0	0	0	0	0	0
ENSMUSG00000093974	0	0	0	0	0	0	0	0	0	0
ENSMUSG00000093976	0	0	0	0	0	0	0	0	0	0
ENSMUSG00000093977	0	0	0	0	0	0	0	0	0	0
ENSMUSG00000093971	0	0	0	0	0	0	0	0	0	0
ENSMUSG00000093973	0	0	2	24	0	3	2	0	0	0
ENSMUSG00000026668	15	12	17	13	24	33	151	49	20	58
ENSMUSG00000093979	23	22	25	26	14	40	49	32	41	62
ENSMUSG00000105321	0	0	0	0	0	0	0	0	0	0
ENSMUSG00000105320	0	0	0	0	0	0	0	0	0	0
ENSMUSG00000105325	0	0	0	0	0	0	0	0	0	0
ENSMUSG00000080142	0	0	0	0	0	0	0	0	0	0
ENSMUSG00000045378	0	0	0	0	0	0	0	0	0	0
ENSMUSG00000081891	0	0	0	0	0	0	0	0	0	0
ENSMUSG00000081892	5	3	0	6	6	6	2	3	8	5
ENSMUSG00000033900	149	215	206	158	136	264	409	352	258	266
ENSMUSG00000033906	107	128	107	104	89	144	360	256	261	184
ENSMUSG00000033904	422	516	462	437	303	557	718	605	707	608
ENSMUSG00000081898	0	0	3	0	0	0	0	0	0	3
ENSMUSG00000098096	0	0	0	0	0	0	0	0	0	0
ENSMUSG00000098094	0	0	0	0	0	0	0	0	0	0
ENSMUSG00000098095	0	0	0	0	0	0	0	0	0	0
ENSMUSG00000098092	0	11	2	6	0	2	8	3	10	2
ENSMUSG00000098091	29	28	59	44	43	31	33	40	25	44
ENSMUSG00000098099	0	0	0	0	0	0	0	0	0	0
ENSMUSG00000063522	0	5	3	6	3	8	1	0	9	9
ENSMUSG00000099941	0	0	0	0	0	0	0	0	0	0
ENSMUSG00000099940	0	0	0	0	0	0	0	0	0	0
ENSMUSG00000099946	0	0	0	0	0	0	0	0	0	0
ENSMUSG00000099945	0	0	0	0	0	0	0	0	0	0
ENSMUSG00000099944	0	0	0	0	0	0	0	0	0	0
ENSMUSG00000074436	0	0	0	0	0	0	0	0	0	0
ENSMUSG00000074437	0	0	0	0	0	0	0	0	0	0
ENSMUSG00000074434	0	0	0	0	0	0	0	0	0	0
ENSMUSG00000074435	0	0	0	0	0	0	0	0	0	0
ENSMUSG00000074433	0	0	0	0	0	0	0	0	0	0
ENSMUSG00000074439	0	0	0	0	0	0	0	0	0	0
ENSMUSG00000081234	0	0	0	0	0	0	0	0	0	0

ENSMUSG00000081235	0	0	0	0	0	0	0	0	0	0
ENSMUSG00000081237	0	0	0	0	0	0	0	0	0	0
ENSMUSG00000081231	0	0	0	0	0	0	0	0	0	0
ENSMUSG00000063297	82	60	98	32	119	84	36	75	51	90
ENSMUSG00000076212	0	0	0	0	0	0	0	0	0	0
ENSMUSG00000087468	0	0	0	0	0	0	0	0	0	0
ENSMUSG00000072874	3	0	0	3	0	0	0	0	2	0
ENSMUSG00000072878	3	0	6	0	0	0	0	0	0	0
ENSMUSG00000048469	0	0	0	0	0	0	0	0	0	0
ENSMUSG00000087462	0	0	0	0	0	0	0	0	0	0
ENSMUSG00000077316	0	0	0	0	0	0	0	0	0	0
ENSMUSG00000077317	0	0	0	0	0	0	0	0	0	0
ENSMUSG00000077314	0	0	0	0	0	0	0	0	1	0
ENSMUSG00000077315	0	0	0	0	0	0	0	0	0	0
ENSMUSG00000086487	6	0	3	3	0	5	0	6	0	3
ENSMUSG00000114631	0	0	0	0	0	0	0	0	0	0
ENSMUSG00000102940	0	0	0	0	0	0	0	0	0	0
ENSMUSG00000102941	3	0	0	0	0	0	3	9	0	0
ENSMUSG00000102942	0	0	0	0	0	0	0	0	0	0
ENSMUSG00000102943	0	0	0	0	0	0	0	0	0	0
ENSMUSG00000102946	0	0	0	0	1	8	6	3	0	0
ENSMUSG00000102948	0	0	5	3	0	0	0	0	0	0
ENSMUSG00000102949	0	0	0	0	0	0	0	0	0	0
ENSMUSG00000043020	0	0	0	0	0	0	0	0	3	0
ENSMUSG00000088353	0	0	0	0	1	0	0	0	0	0
ENSMUSG00000015665	0	0	0	0	0	0	0	0	3	0
ENSMUSG00000033122	0	0	0	0	0	0	0	0	0	0
ENSMUSG00000067998	0	0	0	0	0	0	0	0	2	2
ENSMUSG00000082155	0	0	0	0	0	0	0	0	0	0
ENSMUSG00000082156	0	0	0	0	0	0	0	0	0	0
ENSMUSG00000082157	0	0	0	1	0	5	0	0	2	0
ENSMUSG00000087306	0	0	6	0	0	0	0	0	0	3
ENSMUSG00000088175	0	0	0	0	0	0	0	0	0	0
ENSMUSG00000082152	0	2	2	3	0	3	0	0	0	2
ENSMUSG00000088177	0	0	0	0	0	0	0	0	0	0
ENSMUSG00000088179	0	0	0	0	0	0	0	0	0	0
ENSMUSG00000087308	2	0	0	0	1	0	0	1	0	0
ENSMUSG00000087309	0	0	0	0	0	0	0	0	0	0
ENSMUSG00000082158	0	0	0	0	0	0	0	0	0	0
ENSMUSG00000082159	0	0	0	0	0	0	0	0	0	0
ENSMUSG00000101419	0	0	0	0	0	0	0	0	0	0
ENSMUSG00000101418	0	0	0	0	0	0	0	0	0	0
ENSMUSG00000101417	0	0	0	0	0	0	0	0	0	0
ENSMUSG00000101411	0	0	0	0	0	0	0	0	0	0
ENSMUSG00000037636	3	12	3	34	7	22	52	25	14	26
ENSMUSG00000037638	51	285	372	408	294	82	69	334	479	309
ENSMUSG00000103308	0	0	5	0	3	3	0	0	3	0
ENSMUSG00000103305	0	0	0	0	0	0	0	0	0	0
ENSMUSG00000103306	0	0	0	0	0	0	0	0	0	0
ENSMUSG00000096621	0	0	0	0	0	0	0	0	0	0
ENSMUSG00000096620	0	0	0	0	0	0	0	0	0	0
ENSMUSG00000060176	12	21	15	10	6	5	29	19	12	24
ENSMUSG00000060177	0	0	0	0	0	0	0	0	0	0
ENSMUSG00000040046	6	15	19	10	6	9	80	11	11	5
ENSMUSG00000107819	0	0	0	0	0	0	0	0	0	0
ENSMUSG00000107818	0	0	0	0	0	0	0	0	0	0
ENSMUSG00000107810	97	152	198	148	123	179	185	109	235	172
ENSMUSG00000059816	0	0	0	0	6	0	0	0	0	0

ENSMUSG00000107815	0	0	0	0	0	0	0	0	0	0
ENSMUSG00000107817	0	0	0	0	0	0	0	0	0	0
ENSMUSG00000107816	0	0	0	0	0	0	0	0	0	0
ENSMUSG00000084650	0	0	0	0	0	0	0	0	0	0
ENSMUSG00000084653	0	2	0	1	0	2	1	1	4	1
ENSMUSG00000084652	0	0	0	0	0	0	0	0	0	3
ENSMUSG00000084654	0	0	0	0	0	0	0	0	0	0
ENSMUSG00000089229	0	0	0	0	0	0	0	0	0	0
ENSMUSG00000089228	0	0	0	0	0	0	0	0	0	0
ENSMUSG00000089227	0	0	0	0	0	0	0	0	0	0
ENSMUSG00000089226	0	0	0	0	0	0	0	0	0	0
ENSMUSG00000089220	0	0	0	0	0	0	0	0	0	0
ENSMUSG00000100458	0	0	0	0	0	0	0	0	0	0
ENSMUSG00000100459	0	0	0	0	0	0	0	0	0	0
ENSMUSG00000036949	39	10	15	24	35	17	45	55	20	23
ENSMUSG00000100450	0	0	0	0	0	0	0	0	0	0
ENSMUSG00000100451	0	0	0	0	0	0	0	0	0	0
ENSMUSG00000100456	0	0	0	0	0	0	0	0	0	0
ENSMUSG00000103694	14	13	18	23	16	28	44	26	30	27
ENSMUSG00000022376	94	53	82	59	76	32	13	32	21	19
ENSMUSG00000032657	132	92	117	89	93	84	176	108	112	115
ENSMUSG00000031847	32	27	26	11	32	25	54	11	45	27
ENSMUSG00000095910	0	0	0	0	0	0	0	0	0	0
ENSMUSG00000021720	127	151	162	139	133	127	215	158	177	140
ENSMUSG00000095912	4	5	6	11	0	12	10	10	3	0
ENSMUSG00000095916	0	0	0	0	0	0	0	0	0	0
ENSMUSG00000094831	0	0	0	0	0	0	0	0	0	0
ENSMUSG00000075427	0	0	0	0	0	5	0	0	0	2
ENSMUSG00000102163	0	0	0	0	0	0	0	2	0	0
ENSMUSG00000035560	0	0	0	0	0	0	0	2	0	0
ENSMUSG00000102166	0	0	0	0	0	0	0	0	0	0
ENSMUSG00000102164	0	0	0	0	0	0	0	0	0	0
ENSMUSG00000107106	0	3	9	0	3	0	11	3	0	3
ENSMUSG00000052909	0	0	3	0	0	0	0	0	0	0
ENSMUSG00000107108	0	0	0	3	0	0	0	0	6	0
ENSMUSG00000065450	0	0	0	0	0	0	0	0	0	0
ENSMUSG00000080890	0	0	0	0	0	0	0	0	0	0
ENSMUSG00000065456	0	0	0	0	0	0	1	0	0	0
ENSMUSG00000065457	0	0	0	0	0	0	0	0	0	0
ENSMUSG00000065454	0	0	0	0	0	0	0	0	0	0
ENSMUSG00000065458	0	0	0	1	1	1	1	1	0	1
ENSMUSG00000079544	0	0	0	0	0	0	0	0	0	0
ENSMUSG00000021553	0	0	0	0	0	0	0	0	0	0
ENSMUSG00000029788	1	0	0	0	0	0	0	0	0	0
ENSMUSG00000028789	122	127	170	132	101	282	459	268	362	274
ENSMUSG00000028784	3	0	0	0	3	0	0	0	0	0
ENSMUSG00000028782	94	158	135	77	82	166	252	220	163	109
ENSMUSG00000069171	208	233	119	191	211	248	264	218	260	292
ENSMUSG00000029260	0	0	6	0	0	0	0	0	3	0
ENSMUSG00000063887	5	0	6	0	3	0	20	6	3	6
ENSMUSG00000039809	3	0	9	0	0	0	9	0	8	0
ENSMUSG00000069280	5	0	0	0	0	0	0	0	0	0
ENSMUSG00000063881	0	0	0	0	0	0	0	0	0	0
ENSMUSG00000069289	0	0	0	0	0	0	0	0	0	0
ENSMUSG00000035387	0	0	0	0	0	0	0	0	0	0
ENSMUSG00000024008	156	138	66	33	126	78	24	50	17	19
ENSMUSG00000106478	0	0	0	3	0	0	0	1	0	0
ENSMUSG00000106474	0	0	0	0	0	0	0	0	0	1

ENSMUSG00000029269	0	0	0	0	0	0	0	0	0	0
ENSMUSG00000113757	0	3	0	0	0	5	0	0	4	0
ENSMUSG00000113755	0	0	0	0	0	0	0	0	0	0
ENSMUSG00000113754	0	0	0	0	0	0	0	0	0	0
ENSMUSG00000113759	0	0	0	0	0	0	0	0	0	0
ENSMUSG00000113758	0	0	3	0	0	0	0	0	0	0
ENSMUSG00000112077	0	0	0	0	0	0	0	0	0	0
ENSMUSG00000109628	2	0	6	3	0	0	0	0	0	0
ENSMUSG00000109629	6	0	0	3	0	0	3	3	0	0
ENSMUSG00000093767	0	0	0	0	0	2	0	0	0	0
ENSMUSG00000111445	0	0	4	0	0	3	3	0	4	0
ENSMUSG00000109625	3	6	4	3	3	18	8	0	15	8
ENSMUSG00000109626	0	0	0	0	0	0	0	0	3	0
ENSMUSG00000109627	0	0	0	0	0	0	0	0	0	0
ENSMUSG00000111441	0	0	0	0	0	0	0	0	0	0
ENSMUSG00000109621	0	0	0	0	0	0	0	0	0	0
ENSMUSG00000111443	0	0	0	0	0	0	0	0	0	0
ENSMUSG00000066101	0	0	0	0	0	0	0	0	0	0
ENSMUSG00000066100	0	0	0	0	0	0	0	0	0	0
ENSMUSG00000099917	0	0	0	0	0	0	0	0	0	0
ENSMUSG00000011350	0	0	0	0	0	0	0	2	0	0
ENSMUSG00000050875	0	0	6	0	3	0	17	3	12	0
ENSMUSG00000034384	0	0	0	0	0	0	0	0	0	0
ENSMUSG00000034382	16	36	51	45	45	56	52	91	87	72
ENSMUSG00000093258	0	0	0	0	0	0	0	0	0	0
ENSMUSG00000111200	0	0	0	0	0	0	0	0	0	0
ENSMUSG00000111057	0	0	0	0	0	3	3	0	0	0
ENSMUSG00000069824	0	0	0	0	0	0	0	0	0	0
ENSMUSG00000069823	0	0	0	0	0	0	0	0	0	0
ENSMUSG00000093254	0	0	0	0	0	0	0	0	0	0
ENSMUSG00000051596	0	1	0	0	3	9	91	12	9	6
ENSMUSG00000051590	27	26	27	9	3	14	52	30	18	20
ENSMUSG00000051591	0	0	0	0	0	0	0	0	0	0
ENSMUSG00000051592	0	0	0	0	0	3	0	0	0	0
ENSMUSG00000051593	0	0	0	0	0	0	0	0	0	0
ENSMUSG00000096393	0	0	0	0	0	0	0	0	0	0
ENSMUSG00000096392	0	0	0	0	0	0	0	0	0	0
ENSMUSG00000094204	0	0	0	0	0	0	0	0	0	0
ENSMUSG00000111050	0	0	0	0	0	0	0	0	0	0
ENSMUSG00000099918	0	0	0	0	0	0	0	0	0	0
ENSMUSG00000096399	0	0	0	0	0	0	0	0	0	0
ENSMUSG00000096398	0	2	0	0	0	0	0	0	0	0
ENSMUSG00000097668	0	0	0	0	0	0	0	0	0	0
ENSMUSG00000112934	0	0	0	0	0	0	0	0	0	0
ENSMUSG00000112935	0	0	0	0	0	0	0	0	0	0
ENSMUSG00000054418	46	29	63	70	27	65	216	93	134	78
ENSMUSG00000112937	6	0	0	0	0	3	0	0	0	0
ENSMUSG00000112930	0	0	0	0	0	0	0	0	0	0
ENSMUSG00000112931	0	0	0	0	0	0	0	0	0	0
ENSMUSG00000112932	4	5	0	2	0	0	1	6	0	0
ENSMUSG00000112933	0	0	0	0	0	0	0	0	0	0
ENSMUSG00000091620	0	0	0	0	0	0	0	0	0	0
ENSMUSG00000109996	0	0	0	0	0	0	0	0	0	0
ENSMUSG00000109994	0	0	0	0	0	0	0	0	0	0
ENSMUSG00000109995	0	0	0	0	0	0	0	0	0	0
ENSMUSG00000114198	0	0	0	0	0	0	0	0	0	0
ENSMUSG00000114199	0	0	0	0	0	0	0	0	0	0
ENSMUSG00000109990	0	0	0	0	0	0	0	0	0	0

ENSMUSG00000109991	0	0	0	0	0	0	0	0	0	0
ENSMUSG00000114194	0	0	1	6	0	2	0	1	0	0
ENSMUSG00000114195	0	0	0	0	0	0	0	0	0	0
ENSMUSG00000114197	0	0	0	0	0	0	0	0	0	0
ENSMUSG00000114191	0	0	0	0	0	0	0	0	0	0
ENSMUSG00000114192	0	0	0	0	0	0	0	0	0	0
ENSMUSG00000109999	0	0	0	0	0	0	0	0	0	0
ENSMUSG00000058743	48	46	30	45	23	117	255	84	152	88
ENSMUSG00000058741	0	0	0	0	0	0	0	0	0	0
ENSMUSG00000110473	0	0	0	0	0	0	0	1	1	0
ENSMUSG00000110470	0	0	0	0	0	0	0	0	0	0
ENSMUSG00000110479	0	0	0	0	0	0	0	0	0	0
ENSMUSG00000056812	0	0	0	0	0	0	0	0	0	0
ENSMUSG00000097958	2	0	0	0	0	0	0	0	0	0
ENSMUSG00000096537	0	0	0	0	0	0	0	0	0	0
ENSMUSG00000055994	408	326	319	480	273	303	346	247	295	305
ENSMUSG00000096535	0	0	0	0	0	1	1	1	0	0
ENSMUSG00000097959	0	0	0	0	0	0	0	0	0	0
ENSMUSG00000096533	0	0	0	0	0	0	0	0	0	0
ENSMUSG00000096532	0	0	0	0	0	0	0	0	0	0
ENSMUSG00000096530	0	0	0	0	0	0	81	0	0	0
ENSMUSG00000110984	0	0	0	0	0	0	0	0	0	0
ENSMUSG00000044594	0	0	0	0	0	0	0	0	0	0
ENSMUSG00000096538	0	0	0	0	0	0	0	0	2	0
ENSMUSG00000107508	0	0	0	0	0	2	3	0	0	0
ENSMUSG00000105394	0	0	0	0	0	0	0	0	0	0
ENSMUSG00000060523	0	0	0	0	0	0	0	0	0	0
ENSMUSG00000045179	0	0	0	0	0	0	0	0	0	0
ENSMUSG00000045178	0	0	0	0	0	0	0	0	0	0
ENSMUSG00000088333	0	0	0	0	0	0	0	0	0	0
ENSMUSG00000045174	0	0	0	3	0	0	0	0	0	0
ENSMUSG00000109837	0	0	0	0	0	0	0	0	0	0
ENSMUSG00000026312	0	0	0	0	0	0	0	0	0	0
ENSMUSG00000057149	0	0	0	0	0	0	0	0	0	0
ENSMUSG00000033187	0	0	0	8	0	0	0	4	0	0
ENSMUSG00000113393	0	0	0	0	0	0	0	0	0	0
ENSMUSG00000113391	2	6	2	3	0	0	0	0	0	0
ENSMUSG00000059595	0	0	0	0	0	0	0	0	0	0
ENSMUSG00000088331	0	0	0	0	0	0	0	0	0	0
ENSMUSG00000001819	0	0	0	0	3	0	3	0	0	0
ENSMUSG00000113394	0	0	0	0	0	0	0	0	0	0
ENSMUSG00000114076	0	0	0	0	0	0	0	0	0	0
ENSMUSG00000112978	0	0	0	0	0	0	0	0	0	0
ENSMUSG00000042331	398	342	431	449	289	580	930	640	665	637
ENSMUSG00000077156	0	0	0	0	0	0	0	0	0	0
ENSMUSG00000093570	0	0	0	0	0	0	0	0	0	0
ENSMUSG00000094700	0	0	0	0	0	0	0	0	0	0
ENSMUSG00000093573	0	0	0	0	0	0	0	0	0	0
ENSMUSG00000094706	0	0	0	0	0	0	0	0	0	0
ENSMUSG00000094707	36	23	11	21	9	32	71	27	47	39
ENSMUSG00000094704	0	0	0	0	0	0	0	0	0	0
ENSMUSG00000094709	0	0	1	0	0	0	0	1	0	0
ENSMUSG00000099638	0	0	0	0	0	0	0	0	0	0
ENSMUSG00000092629	0	0	0	0	0	0	0	0	0	0
ENSMUSG00000092627	3	0	3	9	0	0	3	6	8	9
ENSMUSG00000099637	0	0	0	0	0	0	0	0	0	0
ENSMUSG00000092624	0	3	3	0	11	0	3	0	0	3
ENSMUSG00000104625	0	0	0	3	0	0	0	3	0	1

ENSMUSG00000092621	0	0	0	0	0	0	0	0	0	0
ENSMUSG00000092620	0	0	0	0	0	0	0	0	0	0
ENSMUSG00000004948	3	0	0	0	0	0	0	0	0	3
ENSMUSG00000112169	6	2	2	0	0	0	3	0	6	3
ENSMUSG00000101476	0	5	0	0	0	0	0	2	0	3
ENSMUSG00000112162	0	0	0	0	0	0	5	0	2	0
ENSMUSG00000112161	0	0	0	0	0	0	0	0	0	0
ENSMUSG00000055730	0	0	0	0	0	0	0	3	0	0
ENSMUSG00000112166	3	0	0	0	0	0	0	0	0	3
ENSMUSG00000055732	0	0	0	0	0	0	0	0	0	0
ENSMUSG00000112164	0	0	0	0	0	0	0	0	0	0
ENSMUSG00000029999	185	265	267	228	192	234	519	225	190	224
ENSMUSG00000102576	0	0	0	0	0	0	0	0	0	0
ENSMUSG00000101472	0	0	0	0	0	0	0	0	0	0
ENSMUSG00000068348	0	0	0	0	0	0	0	0	0	0
ENSMUSG00000101950	0	0	0	0	0	0	0	0	0	0
ENSMUSG00000077484	0	0	0	0	0	0	0	0	0	0
ENSMUSG00000077480	0	0	0	0	0	0	0	0	0	0
ENSMUSG00000077488	0	2	3	0	0	2	0	0	0	0
ENSMUSG00000093394	0	0	12	0	0	0	6	0	0	0
ENSMUSG00000093397	0	0	0	0	0	0	0	0	0	0
ENSMUSG00000065111	0	0	1	0	0	0	0	0	0	0
ENSMUSG00000086072	0	0	0	0	0	0	0	0	0	0
ENSMUSG00000086071	0	0	0	0	0	0	3	0	0	0
ENSMUSG00000086070	0	0	0	0	0	0	0	0	0	0
ENSMUSG00000086077	0	0	0	0	0	0	0	0	3	0
ENSMUSG00000086074	2	3	1	0	1	0	0	6	2	3
ENSMUSG00000098949	0	0	0	0	0	0	0	0	0	0
ENSMUSG00000049202	0	0	0	0	0	0	0	0	0	0
ENSMUSG00000098944	0	0	2	4	1	0	0	0	0	0
ENSMUSG00000098946	2	2	2	3	1	2	8	1	2	4
ENSMUSG00000098947	0	0	2	0	0	0	0	0	0	0
ENSMUSG00000076355	0	0	0	0	0	0	0	0	0	0
ENSMUSG00000076357	0	0	0	0	0	0	0	0	0	0
ENSMUSG00000082791	0	2	0	0	0	0	0	0	6	2
ENSMUSG00000082796	0	0	0	0	0	0	0	3	0	0
ENSMUSG00000082797	0	0	0	0	0	0	0	0	0	0
ENSMUSG00000082794	3	0	3	0	12	0	3	0	2	3
ENSMUSG00000082795	0	0	0	0	0	0	0	0	0	0
ENSMUSG00000082798	0	0	0	0	0	0	0	0	0	0
ENSMUSG00000082799	0	0	0	0	0	0	0	0	0	0
ENSMUSG00000045952	0	0	0	2	0	0	0	0	0	0
ENSMUSG00000037437	42	23	39	26	18	49	63	31	45	61
ENSMUSG00000004231	0	0	0	3	0	2	0	0	0	0
ENSMUSG00000087678	6	3	36	8	0	7	0	17	9	6
ENSMUSG00000075141	0	0	0	0	0	0	0	0	0	0
ENSMUSG00000109175	2	0	0	4	0	2	3	0	0	2
ENSMUSG00000087674	3	0	11	0	6	0	3	7	3	8
ENSMUSG00000087675	0	0	0	0	0	0	0	0	0	0
ENSMUSG00000070904	0	3	0	0	0	0	0	0	0	0
ENSMUSG00000070902	0	0	0	0	0	0	0	0	0	0
ENSMUSG00000070908	5	8	6	6	2	7	15	7	10	6
ENSMUSG00000071361	0	0	0	0	0	0	0	0	0	0
ENSMUSG00000057805	0	0	0	0	0	0	0	0	0	0
ENSMUSG00000057801	0	0	0	0	0	0	0	0	0	0
ENSMUSG00000057802	50	30	55	30	27	34	45	20	33	29
ENSMUSG00000109178	3	0	3	0	0	0	3	3	0	0
ENSMUSG00000082139	0	0	0	0	0	0	0	0	0	0

ENSMUSG00000084320	2	0	13	3	0	4	6	5	0	0
ENSMUSG00000084325	9	6	3	5	0	3	3	9	11	20
ENSMUSG00000084324	0	0	0	0	0	0	0	0	0	0
ENSMUSG00000084327	0	0	0	0	0	0	0	0	0	0
ENSMUSG00000084326	0	0	0	0	0	0	0	0	0	0
ENSMUSG00000084791	0	0	0	0	0	0	0	0	0	0
ENSMUSG00000025504	336	363	349	271	207	356	309	330	244	358
ENSMUSG00000025507	49	29	42	60	19	43	39	39	36	60
ENSMUSG00000099233	0	1	0	0	0	0	0	0	0	0
ENSMUSG00000099392	0	0	0	0	0	0	0	0	0	0
ENSMUSG00000099393	0	0	0	0	0	0	0	0	0	0
ENSMUSG00000099390	0	0	0	0	0	0	0	0	0	0
ENSMUSG00000099391	0	0	0	0	0	0	0	0	0	0
ENSMUSG00000099396	0	0	0	0	0	0	0	0	0	0
ENSMUSG00000099394	0	0	0	0	0	0	0	0	0	0
ENSMUSG00000099395	0	0	0	0	0	0	0	0	0	0
ENSMUSG00000099399	0	0	0	0	0	0	0	0	0	0
ENSMUSG00000099239	0	0	0	0	0	0	0	0	0	0
ENSMUSG00000065613	0	0	0	0	0	0	0	0	0	0
ENSMUSG00000065616	0	0	0	0	0	0	0	0	0	0
ENSMUSG00000065617	0	1	0	0	0	0	1	1	2	3
ENSMUSG00000032269	6	12	6	0	3	3	3	0	9	0
ENSMUSG00000032268	10	15	23	15	15	9	13	14	10	24
ENSMUSG00000110335	0	0	0	0	0	0	0	0	3	0
ENSMUSG00000032262	11	26	12	9	35	18	48	27	36	31
ENSMUSG00000062042	0	0	0	0	0	0	0	0	0	0
ENSMUSG00000062044	3	6	3	3	6	5	0	0	0	0
ENSMUSG00000062046	0	0	0	0	0	0	3	0	0	0
ENSMUSG00000081914	0	0	0	0	0	0	0	0	0	0
ENSMUSG00000081917	0	0	0	0	0	0	0	0	0	0
ENSMUSG00000081916	0	0	0	0	0	0	0	0	0	0
ENSMUSG00000081910	0	0	0	0	0	0	0	0	0	0
ENSMUSG00000081913	0	0	0	0	0	0	0	0	0	0
ENSMUSG00000081912	0	0	0	0	0	0	0	0	0	0
ENSMUSG00000087034	4	0	0	0	0	0	0	0	2	0
ENSMUSG00000110280	0	0	0	0	0	0	0	0	0	0
ENSMUSG00000084918	3	0	7	2	3	9	5	2	19	2
ENSMUSG00000102632	33	17	23	27	21	27	28	36	12	35
ENSMUSG00000098119	0	0	0	0	0	0	0	0	0	0
ENSMUSG00000098118	0	0	0	0	0	0	0	0	0	0
ENSMUSG00000098763	0	0	0	0	0	0	0	0	0	0
ENSMUSG00000110284	0	0	0	0	0	0	0	0	0	0
ENSMUSG00000098116	0	0	0	0	0	0	0	0	0	0
ENSMUSG00000098115	0	0	0	0	0	0	0	0	0	0
ENSMUSG00000098114	0	0	0	0	0	0	0	0	0	0
ENSMUSG00000037034	0	0	0	0	0	0	3	3	0	0
ENSMUSG00000087036	18	17	30	16	13	21	21	30	33	35
ENSMUSG00000083666	0	0	0	0	0	0	0	0	0	0
ENSMUSG00000032841	199	132	144	266	177	194	414	194	299	215
ENSMUSG00000032842	268	270	344	233	233	324	382	400	290	321
ENSMUSG00000035818	6	3	0	0	3	16	0	13	9	13
ENSMUSG00000035811	0	0	0	0	0	0	0	0	3	0
ENSMUSG00000032092	73	94	102	137	123	146	258	130	108	90
ENSMUSG00000110135	0	0	0	0	0	0	0	0	0	0
ENSMUSG00000103977	0	0	0	0	0	0	0	0	0	0
ENSMUSG00000103971	0	0	0	3	0	0	0	0	0	0
ENSMUSG00000103978	0	0	0	0	0	0	0	0	0	0
ENSMUSG00000034145	3	23	27	12	6	39	68	46	55	57

ENSMUSG00000038997	0	0	3	0	0	0	0	0	3	0
ENSMUSG00000056035	0	0	3	0	0	0	0	0	6	0
ENSMUSG00000089859	0	0	0	0	0	0	0	0	0	0
ENSMUSG00000056031	2	10	0	11	10	7	29	14	20	15
ENSMUSG00000086494	0	0	0	7	5	0	0	0	0	5
ENSMUSG00000014813	437	317	288	551	369	842	497	396	307	417
ENSMUSG00000086497	0	0	0	0	0	0	0	0	0	0
ENSMUSG00000091422	0	0	0	0	0	0	0	0	0	0
ENSMUSG00000091425	0	0	0	0	0	0	0	0	0	0
ENSMUSG00000086491	0	0	0	0	0	0	0	0	0	0
ENSMUSG00000018126	13	9	3	6	0	6	6	4	0	13
ENSMUSG00000093731	0	0	0	0	0	0	0	0	0	0
ENSMUSG00000086490	0	0	0	0	0	0	0	0	0	0
ENSMUSG00000101369	0	0	0	0	0	0	0	0	0	0
ENSMUSG00000086498	0	2	0	0	3	0	0	2	7	3
ENSMUSG00000108803	0	0	0	0	0	0	0	0	0	0
ENSMUSG00000108800	0	0	0	0	0	0	0	0	0	0
ENSMUSG00000108805	0	0	0	0	0	0	0	3	0	0
ENSMUSG00000108804	0	0	0	0	0	0	0	0	0	0
ENSMUSG00000108809	0	0	0	0	0	0	0	0	0	0
ENSMUSG00000067561	0	0	0	0	0	0	0	0	0	0
ENSMUSG00000105988	0	0	0	0	0	0	0	0	0	0
ENSMUSG00000044912	0	0	0	0	0	0	0	3	0	0
ENSMUSG00000051228	63	63	99	19	68	82	21	86	74	88
ENSMUSG00000048062	0	0	0	0	0	0	0	0	0	0
ENSMUSG00000099812	3	0	6	0	10	0	6	9	12	0
ENSMUSG00000101608	0	0	0	0	0	0	0	0	0	0
ENSMUSG00000101606	0	0	0	0	0	0	0	0	0	0
ENSMUSG00000101607	0	0	0	0	0	0	0	0	0	0
ENSMUSG00000101605	0	0	0	0	0	0	0	0	0	0
ENSMUSG00000101602	0	0	0	0	0	0	0	0	0	0
ENSMUSG00000101600	0	0	0	0	0	0	0	0	0	0
ENSMUSG00000060030	0	0	0	0	0	0	0	0	0	0
ENSMUSG00000085806	0	0	0	0	0	0	0	0	0	0
ENSMUSG00000085805	0	0	0	0	0	0	0	0	0	0
ENSMUSG00000085804	0	0	0	0	0	0	0	0	0	0
ENSMUSG00000085803	0	0	0	0	0	0	0	0	0	0
ENSMUSG00000085801	0	0	0	0	0	0	0	0	0	0
ENSMUSG00000085808	0	0	0	0	0	3	0	0	0	0
ENSMUSG00000061928	0	0	0	0	0	0	6	0	0	0
ENSMUSG00000061923	0	0	5	0	0	0	3	0	0	3
ENSMUSG00000099653	0	0	0	0	0	0	0	0	0	0
ENSMUSG00000087531	0	0	0	0	0	0	2	2	0	0
ENSMUSG00000087534	0	0	0	0	0	0	0	0	0	0
ENSMUSG00000087537	0	0	0	0	0	0	0	0	0	0
ENSMUSG00000087536	0	0	0	0	0	0	0	0	0	0
ENSMUSG00000087539	0	0	0	0	0	0	0	0	0	0
ENSMUSG00000063380	0	0	0	0	0	0	0	0	0	0
ENSMUSG00000101247	0	0	0	0	0	0	0	0	0	0
ENSMUSG00000107609	0	0	0	0	0	0	0	0	0	0
ENSMUSG00000107601	0	0	0	0	0	0	0	0	0	0
ENSMUSG00000107602	0	0	0	0	0	0	0	0	0	0
ENSMUSG00000107605	14	14	9	10	27	41	15	11	21	23
ENSMUSG00000089358	0	0	0	0	0	0	0	0	0	0
ENSMUSG00000056648	0	0	0	0	11	0	14	0	0	3
ENSMUSG00000103110	0	0	0	0	0	0	0	0	0	0
ENSMUSG00000103111	0	0	0	0	0	0	0	0	0	0
ENSMUSG00000103112	0	0	0	0	0	0	0	0	0	0

ENSMUSG00000036172	3	10	20	15	12	21	40	3	9	10
ENSMUSG00000103114	0	5	9	9	3	12	16	5	15	21
ENSMUSG00000103115	27	16	23	14	10	24	30	52	35	12
ENSMUSG00000103116	0	0	0	0	0	0	0	0	0	0
ENSMUSG00000101245	0	0	0	0	0	0	0	0	0	0
ENSMUSG00000095895	2	0	2	3	0	4	3	4	2	2
ENSMUSG00000095894	0	0	0	0	0	0	0	0	0	0
ENSMUSG00000095896	0	0	0	0	0	0	0	0	0	0
ENSMUSG00000095890	0	0	0	0	0	0	0	0	0	0
ENSMUSG00000095893	0	0	0	0	0	0	0	0	0	0
ENSMUSG00000095899	0	0	0	0	0	0	0	0	0	0
ENSMUSG00000095898	0	0	0	0	0	0	0	0	0	0
ENSMUSG00000065555	2	1	5	1	2	4	3	2	5	4
ENSMUSG00000065551	1	3	0	0	0	2	1	0	0	1
ENSMUSG00000055609	0	0	0	0	0	0	0	0	0	0
ENSMUSG00000065559	0	0	0	0	0	0	0	0	0	0
ENSMUSG00000022501	0	0	0	3	3	0	3	0	0	0
ENSMUSG00000108138	0	0	0	3	9	3	0	0	3	0
ENSMUSG00000089078	0	0	0	0	0	0	0	0	0	0
ENSMUSG00000089079	0	0	0	0	0	0	0	0	0	0
ENSMUSG00000089077	0	0	0	0	0	0	0	0	0	0
ENSMUSG00000089075	0	0	0	0	0	0	0	0	0	0
ENSMUSG00000089073	0	0	0	0	0	0	0	0	0	0
ENSMUSG00000089071	0	0	0	0	0	2	0	0	0	1
ENSMUSG00000102360	0	0	0	0	0	0	0	0	0	0
ENSMUSG00000050577	0	0	0	0	0	0	0	0	0	0
ENSMUSG00000105948	0	0	0	0	0	0	0	0	0	0
ENSMUSG00000050578	151	60	157	237	146	50	141	92	76	68
ENSMUSG00000102365	5	15	18	3	8	6	14	6	11	15
ENSMUSG00000112361	0	0	3	0	0	0	0	0	0	0
ENSMUSG00000112360	0	0	0	0	0	3	0	0	0	0
ENSMUSG00000112363	0	0	0	0	0	0	0	0	0	0
ENSMUSG00000102367	0	0	0	0	0	0	0	0	0	0
ENSMUSG00000112365	3	3	3	6	10	0	4	0	0	0
ENSMUSG00000094277	0	0	0	0	0	0	0	0	0	0
ENSMUSG00000094274	0	0	0	0	0	0	0	0	0	0
ENSMUSG00000112366	0	0	0	0	0	0	3	0	0	0
ENSMUSG00000102646	0	0	2	0	3	1	0	0	3	1
ENSMUSG00000102643	0	0	0	0	0	0	0	0	0	0
ENSMUSG00000102641	0	0	0	0	0	0	0	0	3	1
ENSMUSG00000087940	0	0	0	0	0	0	0	0	0	0
ENSMUSG00000024901	180	197	265	165	176	192	331	247	245	261
ENSMUSG00000102648	0	0	0	0	0	0	0	0	0	0
ENSMUSG00000098258	0	1	1	0	0	0	0	0	0	1
ENSMUSG00000062528	0	0	0	0	0	0	0	0	0	0
ENSMUSG00000113098	0	0	0	0	0	0	0	0	0	0
ENSMUSG00000110212	0	0	0	0	0	0	0	0	0	0
ENSMUSG00000110211	0	0	0	0	0	0	0	0	0	0
ENSMUSG00000110210	0	0	0	0	0	0	0	0	0	0
ENSMUSG00000110217	0	0	0	0	0	0	0	0	0	0
ENSMUSG00000110216	0	3	0	0	0	0	0	3	3	0
ENSMUSG00000110215	0	0	0	0	0	0	0	0	0	0
ENSMUSG00000110214	0	0	0	0	0	0	0	0	0	0
ENSMUSG00000113090	0	0	0	0	0	0	0	0	0	0
ENSMUSG00000113091	0	0	0	0	0	0	0	0	0	0
ENSMUSG00000113092	0	0	0	0	0	0	0	0	0	0
ENSMUSG00000113093	0	0	0	0	0	0	0	0	0	0
ENSMUSG00000028590	0	2	0	0	0	0	0	0	3	0

ENSMUSG00000113095	0	0	0	0	0	0	0	0	0	0
ENSMUSG00000113096	0	0	0	0	0	2	0	3	2	0
ENSMUSG00000113097	0	0	0	0	0	0	0	2	0	0
ENSMUSG00000097670	0	0	0	0	0	0	0	0	0	0
ENSMUSG00000035735	216	260	240	285	225	320	399	308	347	313
ENSMUSG00000105516	6	0	3	3	0	0	0	0	7	0
ENSMUSG00000105515	0	0	0	0	0	0	0	0	0	0
ENSMUSG00000087575	3	0	0	6	0	2	0	8	0	0
ENSMUSG00000107084	0	0	0	0	0	0	0	0	0	0
ENSMUSG00000105511	0	0	0	0	0	0	0	0	0	0
ENSMUSG00000088846	0	0	0	0	0	0	0	0	0	0
ENSMUSG00000088840	0	0	0	0	0	0	0	0	0	0
ENSMUSG00000088842	0	0	0	0	0	0	0	0	0	0
ENSMUSG00000069324	0	0	0	0	0	0	0	0	0	0
ENSMUSG00000094858	0	0	0	3	0	0	0	0	0	0
ENSMUSG00000021749	0	0	0	0	0	0	0	0	0	0
ENSMUSG00000094856	3	0	0	0	0	0	2	0	0	0
ENSMUSG00000021747	156	287	247	140	121	287	169	255	275	265
ENSMUSG00000094854	0	0	0	0	0	0	0	0	0	0
ENSMUSG00000094855	0	0	0	0	0	0	0	0	0	0
ENSMUSG00000094852	0	0	0	2	0	0	0	2	0	0
ENSMUSG00000021743	0	0	0	0	0	0	0	0	0	0
ENSMUSG00000094850	0	0	0	0	0	0	0	0	0	0
ENSMUSG00000095213	0	0	0	0	0	0	0	0	0	0
ENSMUSG00000083455	0	0	0	0	0	0	0	0	0	0
ENSMUSG00000066319	37	62	66	79	33	48	30	72	42	49
ENSMUSG00000082802	0	0	0	0	0	0	0	0	0	0
ENSMUSG00000082807	0	0	0	0	0	0	0	0	0	0
ENSMUSG00000113542	6	0	0	9	0	0	0	12	2	3
ENSMUSG00000113543	0	0	0	0	0	0	0	0	0	0
ENSMUSG00000038760	0	0	0	0	0	0	3	0	0	0
ENSMUSG00000113541	0	1	1	0	1	0	1	0	1	1
ENSMUSG00000113546	0	0	0	0	0	0	0	0	0	0
ENSMUSG00000113547	0	0	0	1	0	0	0	0	0	0
ENSMUSG00000034467	0	0	0	0	0	0	3	3	0	0
ENSMUSG00000038765	0	12	3	0	5	0	3	6	6	3
ENSMUSG00000038768	0	0	0	0	0	0	0	0	0	0
ENSMUSG00000113549	0	0	0	0	0	0	0	0	0	0
ENSMUSG00000055972	8	23	17	14	0	14	22	7	17	12
ENSMUSG00000055971	0	0	0	0	0	0	0	0	0	0
ENSMUSG00000055976	18	3	18	3	9	15	23	20	21	20
ENSMUSG00000114011	0	0	0	0	0	0	0	0	0	0
ENSMUSG00000114010	0	0	0	0	0	0	0	0	0	0
ENSMUSG00000114013	0	0	0	0	0	0	0	0	0	0
ENSMUSG00000114012	0	0	0	0	0	0	0	0	0	0
ENSMUSG00000114015	0	0	0	0	0	0	0	0	0	0
ENSMUSG00000114017	0	0	0	0	0	0	0	0	0	0
ENSMUSG00000086462	0	3	0	0	0	0	0	0	0	0
ENSMUSG00000114018	6	0	13	6	0	5	12	5	15	0
ENSMUSG00000065619	0	0	0	0	0	0	0	0	0	0
ENSMUSG00000059626	0	0	0	0	2	0	0	0	0	0
ENSMUSG00000059625	0	0	0	0	0	0	3	0	0	0
ENSMUSG00000059623	3	0	0	0	0	0	0	0	0	0
ENSMUSG00000101407	0	0	0	0	0	0	0	0	0	0
ENSMUSG00000091987	0	0	0	0	0	0	0	0	0	0
ENSMUSG00000087330	0	3	3	4	0	0	0	0	0	0
ENSMUSG00000091983	21	3	3	6	3	3	8	4	5	14
ENSMUSG00000091980	0	0	0	0	0	0	0	0	0	0

ENSMUSG00000087337	0	0	0	0	0	0	0	0	0	0
ENSMUSG00000067047	0	0	0	0	0	0	0	0	0	0
ENSMUSG00000067049	0	0	0	0	0	0	0	2	0	5
ENSMUSG00000089114	0	0	0	0	0	0	0	0	0	0
ENSMUSG00000020598	61	80	48	35	35	63	132	70	45	86
ENSMUSG00000020599	0	0	0	12	5	25	42	16	17	12
ENSMUSG00000054594	13	10	16	30	24	30	28	18	30	29
ENSMUSG00000075485	0	0	0	0	0	0	0	0	0	0
ENSMUSG00000104397	0	0	0	3	0	0	0	0	0	0
ENSMUSG00000104396	0	0	0	0	0	0	0	0	0	0
ENSMUSG00000104395	0	0	0	0	0	0	0	0	0	0
ENSMUSG00000104393	0	0	0	0	0	0	0	0	0	0
ENSMUSG00000054598	3	3	0	9	3	15	5	2	0	2
ENSMUSG00000083160	0	0	0	0	0	0	6	0	0	0
ENSMUSG00000056995	0	0	0	0	0	0	0	0	0	0
ENSMUSG00000049410	3	8	0	0	9	0	6	3	18	3
ENSMUSG00000026295	0	0	3	6	0	0	0	0	0	9
ENSMUSG00000083651	0	0	0	0	0	0	0	0	0	0
ENSMUSG00000098333	0	0	0	0	0	0	0	0	0	0
ENSMUSG00000113857	0	0	0	0	0	0	0	0	0	0
ENSMUSG00000027748	8	22	27	23	18	28	17	21	18	18
ENSMUSG00000027744	0	6	8	0	5	6	0	0	4	0
ENSMUSG00000098336	0	0	0	0	0	0	0	0	0	0
ENSMUSG00000089111	0	0	0	0	0	0	0	0	0	0
ENSMUSG00000046300	0	0	0	0	0	0	0	0	0	0
ENSMUSG00000092988	0	0	0	0	0	0	0	0	0	0
ENSMUSG00000042817	23	15	34	28	30	51	184	74	137	51
ENSMUSG00000042816	32	22	12	15	12	15	44	12	26	30
ENSMUSG00000089113	0	0	0	0	0	2	0	0	0	0
ENSMUSG00000080511	0	0	0	0	0	0	0	0	0	0
ENSMUSG00000080519	0	0	0	0	0	0	0	0	0	0
ENSMUSG00000074802	622	433	572	399	309	459	857	578	624	562
ENSMUSG00000074804	0	0	0	0	0	0	0	0	0	0
ENSMUSG00000090166	0	0	0	0	0	0	0	0	3	0
ENSMUSG00000098358	0	0	0	0	0	0	0	0	0	0
ENSMUSG00000090162	0	0	0	0	0	0	0	0	0	0
ENSMUSG00000098351	0	0	0	0	0	0	0	0	0	0
ENSMUSG00000098354	0	0	0	0	0	0	0	0	0	0
ENSMUSG00000098357	0	0	0	0	0	1	0	0	1	0
ENSMUSG00000098356	0	0	1	0	0	0	0	0	0	0
ENSMUSG00000111965	0	0	0	0	0	0	0	0	0	0
ENSMUSG00000006179	0	0	6	4	9	3	8	12	3	3
ENSMUSG00000114498	0	3	0	3	0	3	6	2	0	1
ENSMUSG00000086917	0	8	1	0	0	0	0	0	0	8
ENSMUSG00000086910	0	0	0	0	0	0	0	0	0	3
ENSMUSG00000086911	0	3	0	0	0	0	0	0	0	3
ENSMUSG00000086912	0	0	0	0	0	0	0	0	0	0
ENSMUSG00000086913	0	0	0	0	0	4	3	0	0	3
ENSMUSG00000095662	0	3	2	1	1	1	1	2	0	0
ENSMUSG00000095663	0	0	4	0	0	0	0	0	0	0
ENSMUSG00000090292	0	0	0	0	1	0	0	0	0	0
ENSMUSG00000095661	0	1	0	0	0	2	2	0	1	3
ENSMUSG00000095666	0	0	0	0	0	0	0	0	0	0
ENSMUSG00000095667	0	0	0	0	0	0	0	0	0	0
ENSMUSG00000095664	0	0	0	0	0	0	0	0	0	0
ENSMUSG00000090298	0	0	0	0	0	0	0	0	0	0
ENSMUSG00000094482	0	0	0	0	0	0	0	0	0	0
ENSMUSG00000039578	219	174	214	230	154	205	200	256	165	215

ENSMUSG00000100567	0	0	0	0	0	0	0	0	0	0
ENSMUSG00000021538	0	0	0	0	0	0	0	0	0	0
ENSMUSG00000053909	0	0	0	0	0	0	0	0	0	0
ENSMUSG00000094131	0	1	0	0	2	3	1	2	1	0
ENSMUSG00000094133	0	0	0	0	0	0	0	0	0	0
ENSMUSG00000094132	13	10	16	10	14	20	17	13	2	15
ENSMUSG00000094135	0	0	0	0	0	3	0	0	0	0
ENSMUSG00000042102	12	0	6	3	0	0	0	0	3	0
ENSMUSG00000094137	0	0	0	0	0	0	0	0	0	0
ENSMUSG00000094488	0	0	0	0	0	0	0	0	0	0
ENSMUSG00000094139	0	0	0	0	0	0	0	0	0	0
ENSMUSG00000081641	0	0	0	0	0	0	0	0	0	0
ENSMUSG00000095310	0	0	0	0	0	0	0	0	0	0
ENSMUSG00000093451	0	0	0	0	0	0	0	0	0	0
ENSMUSG00000081647	0	0	0	0	0	0	4	0	4	0
ENSMUSG00000081646	0	0	0	0	0	0	0	0	0	0
ENSMUSG00000081649	0	0	0	0	0	0	0	0	0	0
ENSMUSG00000077728	0	0	0	0	0	0	0	0	0	0
ENSMUSG00000100797	0	0	0	0	0	0	0	0	0	0
ENSMUSG00000049902	0	0	0	0	0	0	0	0	0	0
ENSMUSG00000107162	0	3	0	0	3	0	0	3	2	0
ENSMUSG00000057654	0	0	0	0	2	0	0	0	0	1
ENSMUSG00000063251	0	0	0	0	0	0	0	0	0	0
ENSMUSG00000090839	0	0	0	0	0	0	0	0	0	0
ENSMUSG00000100792	0	0	0	0	0	0	0	0	0	0
ENSMUSG00000100791	0	0	0	0	0	0	0	0	0	0
ENSMUSG00000092476	0	0	0	0	0	0	0	0	0	0
ENSMUSG00000092477	0	0	0	0	0	0	0	0	0	0
ENSMUSG00000092473	0	0	0	0	0	0	0	0	0	0
ENSMUSG00000101644	0	0	0	0	0	0	0	0	0	0
ENSMUSG00000046486	0	0	0	0	0	0	0	0	0	0
ENSMUSG00000043472	0	0	0	0	0	0	0	0	0	0
ENSMUSG00000030752	396	328	369	337	317	341	367	389	354	399
ENSMUSG00000030757	53	17	32	56	23	67	62	93	50	67
ENSMUSG00000100799	0	0	0	0	3	0	0	0	0	0
ENSMUSG00000103626	0	0	0	0	0	0	0	0	0	0
ENSMUSG00000112749	0	0	0	0	0	0	0	0	0	0
ENSMUSG00000082344	0	0	0	0	0	0	0	0	0	0
ENSMUSG00000082340	0	0	0	0	0	0	0	0	0	0
ENSMUSG00000082342	3	5	0	0	5	1	1	3	4	0
ENSMUSG00000101773	0	0	0	0	0	0	0	0	0	0
ENSMUSG00000101770	0	0	0	0	0	0	0	0	0	0
ENSMUSG00000101771	0	0	0	0	0	0	0	0	0	0
ENSMUSG00000005883	3	3	0	6	3	0	12	3	3	3
ENSMUSG00000098484	0	0	0	0	0	0	0	0	0	0
ENSMUSG00000101853	0	0	0	0	0	0	0	0	0	0
ENSMUSG00000032446	12	6	9	6	9	6	34	20	38	15
ENSMUSG00000098481	0	0	0	0	0	0	0	0	0	0
ENSMUSG00000072774	268	171	313	232	196	266	521	328	336	353
ENSMUSG00000087737	0	0	0	2	0	0	0	1	0	0
ENSMUSG00000087736	0	0	0	0	0	0	0	0	0	0
ENSMUSG00000087732	0	0	0	0	0	0	0	0	0	0
ENSMUSG00000070732	9	6	3	18	6	9	10	22	3	3
ENSMUSG00000084428	0	0	0	0	0	0	0	0	0	0
ENSMUSG00000083438	0	0	0	0	0	0	0	1	0	0
ENSMUSG00000083439	0	0	0	0	0	0	0	0	0	0
ENSMUSG00000083434	0	0	0	0	0	0	0	0	0	0
ENSMUSG00000083436	0	0	2	0	0	0	1	0	0	0

ENSMUSG00000083437	0	0	0	0	0	0	0	0	0	0
ENSMUSG00000084426	0	0	0	0	0	0	0	0	0	0
ENSMUSG00000083431	0	0	0	0	0	0	0	0	0	0
ENSMUSG00000084425	1	0	0	2	0	0	9	0	0	0
ENSMUSG00000049015	0	0	0	0	0	0	0	0	0	0
ENSMUSG00000049011	0	0	0	0	0	0	0	0	0	0
ENSMUSG00000079516	0	0	0	0	1	0	0	0	6	0
ENSMUSG00000079513	0	0	0	0	0	0	0	0	0	0
ENSMUSG00000106530	0	0	0	0	0	0	0	0	0	0
ENSMUSG00000087880	0	0	0	0	0	0	0	0	0	0
ENSMUSG00000031936	0	9	35	9	12	61	228	36	135	63
ENSMUSG00000031937	32	45	33	20	14	20	44	37	53	23
ENSMUSG00000106533	0	0	0	0	0	0	0	0	0	0
ENSMUSG00000031932	0	3	9	0	6	8	12	16	3	6
ENSMUSG00000079519	0	0	0	0	0	0	0	0	0	0
ENSMUSG00000094698	0	0	0	0	0	0	0	0	0	0
ENSMUSG00000106532	0	0	0	0	0	0	0	0	0	0
ENSMUSG00000085770	0	0	0	0	0	0	0	0	0	0
ENSMUSG00000085773	0	0	0	0	0	0	0	0	0	0
ENSMUSG00000085772	25	32	51	33	6	29	27	27	89	39
ENSMUSG00000085775	0	0	0	0	0	0	0	0	0	0
ENSMUSG00000085774	0	3	0	0	0	4	0	3	5	0
ENSMUSG00000064044	0	0	0	0	0	0	0	0	0	0
ENSMUSG00000066756	0	0	0	0	0	0	0	0	0	0
ENSMUSG00000107447	0	0	0	0	0	0	2	0	0	0
ENSMUSG00000107445	0	0	0	0	0	0	0	0	0	0
ENSMUSG00000107443	2	1	1	2	1	3	4	1	1	1
ENSMUSG00000033470	27	18	17	60	36	0	45	80	31	40
ENSMUSG00000065795	0	0	0	0	0	0	0	0	0	0
ENSMUSG00000065794	0	0	0	0	0	0	0	0	0	0
ENSMUSG00000065797	0	0	0	0	0	0	0	0	0	0
ENSMUSG00000065791	0	0	0	0	0	0	0	0	0	0
ENSMUSG00000065792	0	0	0	0	0	2	2	0	0	0
ENSMUSG00000094151	4	0	3	0	0	0	0	0	0	0
ENSMUSG00000094150	0	0	0	0	0	0	0	0	0	0
ENSMUSG00000094156	0	0	0	0	0	0	0	0	0	0
ENSMUSG00000036045	0	0	0	0	0	0	0	0	0	0
ENSMUSG00000101760	0	0	0	0	0	0	0	0	0	0
ENSMUSG00000094155	0	0	0	0	0	0	0	0	0	0
ENSMUSG00000084289	0	0	0	0	0	0	0	0	0	0
ENSMUSG00000084286	0	0	0	0	0	0	0	0	0	0
ENSMUSG00000084287	0	0	0	0	0	0	0	0	0	0
ENSMUSG00000084285	0	0	0	0	0	0	0	0	0	0
ENSMUSG00000084282	0	0	0	0	0	0	0	0	0	0
ENSMUSG00000086937	3	0	0	2	0	0	9	5	0	0
ENSMUSG00000068011	42	73	66	49	44	62	65	82	79	94
ENSMUSG00000092866	0	0	0	0	1	0	0	0	0	0
ENSMUSG00000085483	6	3	0	3	6	0	0	5	3	6
ENSMUSG00000100421	0	0	0	0	0	0	0	0	0	0
ENSMUSG00000085489	0	0	0	0	0	0	0	0	0	0
ENSMUSG00000085488	0	0	0	0	0	0	0	0	0	0
ENSMUSG00000100423	0	0	0	0	0	0	0	0	0	0
ENSMUSG00000001763	133	102	199	175	89	159	78	120	107	119
ENSMUSG00000100422	0	0	0	0	0	0	0	0	0	0
ENSMUSG00000100429	0	0	0	0	0	0	0	0	0	0
ENSMUSG00000100428	0	0	0	0	0	0	0	0	0	0
ENSMUSG00000086939	0	0	0	0	0	0	0	0	0	0
ENSMUSG00000114424	0	0	2	0	3	3	3	11	0	6

ENSMUSG00000110539	0	0	0	0	0	0	0	0	0	0
ENSMUSG00000076376	2	1	2	3	2	9	1	6	5	4
ENSMUSG00000103424	0	0	0	0	0	0	0	0	3	3
ENSMUSG00000103399	0	0	0	1	0	0	0	0	0	0
ENSMUSG00000103425	0	0	0	0	0	0	0	0	0	0
ENSMUSG00000106410	0	0	0	0	0	0	8	0	0	0
ENSMUSG00000114426	0	0	0	0	0	0	0	0	0	0
ENSMUSG00000073413	29	28	23	22	28	12	25	23	43	45
ENSMUSG00000102250	0	0	0	0	0	0	0	0	0	0
ENSMUSG00000102251	0	0	0	0	0	0	0	0	0	0
ENSMUSG00000092517	6	7	11	9	9	0	7	0	6	18
ENSMUSG00000102255	0	0	0	0	0	0	0	0	0	0
ENSMUSG00000102256	0	0	0	0	0	0	0	1	0	0
ENSMUSG00000022849	223	206	318	231	172	230	337	240	331	299
ENSMUSG00000038560	72	0	0	0	0	0	0	0	0	0
ENSMUSG00000101768	0	5	12	9	0	3	3	3	6	0
ENSMUSG00000083839	0	0	0	0	0	0	0	0	0	0
ENSMUSG00000096743	0	0	0	0	0	0	0	0	0	0
ENSMUSG00000096744	0	0	0	0	0	0	0	0	0	0
ENSMUSG00000096748	0	0	0	0	0	0	0	0	0	0
ENSMUSG00000096749	0	0	0	0	0	0	0	0	0	0
ENSMUSG00000031169	156	194	142	223	140	252	332	220	279	187
ENSMUSG00000114503	0	0	6	0	0	0	0	2	3	0
ENSMUSG00000050702	0	2	2	0	0	0	4	0	0	2
ENSMUSG00000031162	3	0	3	107	24	9	13	49	24	3
ENSMUSG00000050700	6	15	3	6	0	23	29	9	9	21
ENSMUSG00000031160	0	0	0	0	0	0	0	0	0	0
ENSMUSG00000050704	0	0	0	0	0	0	0	0	0	0
ENSMUSG00000083835	0	0	0	0	0	0	0	0	0	0
ENSMUSG00000063106	0	0	0	0	0	0	0	0	0	0
ENSMUSG00000067684	0	0	0	0	0	0	0	0	0	0
ENSMUSG00000102870	0	0	2	0	0	0	0	1	0	0
ENSMUSG00000102872	0	0	0	0	0	0	0	0	0	0
ENSMUSG00000102878	0	0	0	0	0	0	0	0	0	0
ENSMUSG00000018776	4	0	12	0	3	0	0	3	0	0
ENSMUSG00000097740	39	46	8	27	23	8	3	18	12	34
ENSMUSG00000104158	2	0	0	0	0	0	0	0	0	0
ENSMUSG00000095960	0	0	0	0	0	0	0	0	0	0
ENSMUSG00000104155	0	0	0	0	0	3	9	3	4	3
ENSMUSG00000104157	0	0	0	2	0	0	3	3	0	0
ENSMUSG00000089768	3	2	2	10	8	16	18	12	26	0
ENSMUSG00000104152	0	0	0	0	0	0	0	0	0	0
ENSMUSG00000111716	0	0	0	0	0	0	0	0	2	0
ENSMUSG00000059708	618	626	664	574	454	629	767	687	686	753
ENSMUSG00000091212	0	0	0	0	0	0	0	0	0	0
ENSMUSG00000010505	0	0	0	0	0	0	0	0	3	0
ENSMUSG00000101185	0	0	0	0	0	0	0	0	0	0
ENSMUSG00000060480	0	0	0	0	0	0	0	0	0	0
ENSMUSG00000111438	0	0	0	0	0	0	0	0	0	0
ENSMUSG00000056468	0	6	25	8	15	2	47	7	9	38
ENSMUSG00000110821	0	0	0	0	0	0	0	0	0	0
ENSMUSG00000110823	0	0	0	0	0	0	0	0	0	0
ENSMUSG00000110827	22	20	12	8	23	9	32	10	17	13
ENSMUSG00000110829	0	0	0	0	0	0	0	0	0	0
ENSMUSG00000104849	0	0	0	0	0	0	0	0	0	0
ENSMUSG00000104843	0	0	0	0	0	0	0	0	0	0
ENSMUSG00000104841	0	6	0	0	0	0	3	0	0	0
ENSMUSG00000104840	0	0	0	0	0	0	0	0	0	0

ENSMUSG00000104845	0	0	0	0	0	0	0	0	0	0
ENSMUSG00000097604	3	3	3	0	3	6	11	11	5	15
ENSMUSG00000088264	0	0	0	0	0	0	0	0	0	0
ENSMUSG00000088266	0	0	0	0	0	0	0	0	0	0
ENSMUSG00000094211	0	0	0	0	0	0	0	0	0	0
ENSMUSG00000058078	0	0	0	0	0	0	0	0	0	0
ENSMUSG00000088261	0	0	0	0	0	0	0	0	0	0
ENSMUSG00000058071	0	0	0	0	0	0	0	0	0	0
ENSMUSG00000027400	0	0	0	0	0	0	0	6	0	0
ENSMUSG00000020722	13	13	3	6	3	17	14	12	11	3
ENSMUSG00000013367	8	11	12	9	18	5	24	13	6	14
ENSMUSG00000097602	2	6	6	7	2	4	5	0	3	4
ENSMUSG00000022696	54	13	33	48	29	31	79	29	41	18
ENSMUSG00000083205	0	0	0	0	0	0	0	0	0	0
ENSMUSG00000095462	0	0	0	0	0	0	0	0	0	0
ENSMUSG00000084256	0	0	0	0	0	0	0	0	0	0
ENSMUSG00000095465	0	0	0	0	0	0	0	0	0	0
ENSMUSG00000095466	0	0	0	0	0	0	0	0	0	0
ENSMUSG00000095467	0	0	0	0	0	0	0	0	0	0
ENSMUSG00000095469	0	0	0	0	0	0	0	0	0	0
ENSMUSG00000083201	0	0	0	0	0	0	0	0	0	0
ENSMUSG00000100161	0	0	0	0	0	0	0	0	0	0
ENSMUSG00000025219	0	0	3	0	0	0	0	0	0	0
ENSMUSG00000095939	0	0	0	0	0	0	0	0	0	0
ENSMUSG00000022715	0	3	0	0	0	0	0	0	0	0
ENSMUSG00000095806	0	0	0	0	0	0	0	0	0	0
ENSMUSG00000093108	2	0	0	0	0	0	0	0	0	0
ENSMUSG00000093105	1	0	1	0	0	0	0	0	2	2
ENSMUSG00000094336	3	4	3	4	3	4	6	5	3	4
ENSMUSG00000093101	0	0	0	0	0	0	0	0	0	0
ENSMUSG00000112269	0	0	0	0	0	0	0	0	2	0
ENSMUSG00000093102	0	0	0	0	0	0	0	0	0	0
ENSMUSG00000036322	0	0	0	0	0	0	0	0	0	0
ENSMUSG00000002055	164	111	124	171	127	84	245	107	125	131
ENSMUSG00000002057	0	0	0	0	0	6	0	0	0	0
ENSMUSG00000097823	12	14	19	0	2	46	94	18	19	15
ENSMUSG00000097821	0	0	0	0	0	0	0	0	3	3
ENSMUSG00000097824	0	0	0	0	0	0	0	0	0	0
ENSMUSG00000097825	0	0	0	0	0	0	3	0	0	3
ENSMUSG00000109235	0	0	0	0	0	0	0	0	0	0
ENSMUSG00000112539	0	0	1	0	0	0	2	0	0	0
ENSMUSG00000041247	0	0	0	8	0	27	6	0	0	0
ENSMUSG00000055341	75	57	58	39	48	81	80	50	98	62
ENSMUSG00000112531	0	0	0	0	0	0	0	0	0	0
ENSMUSG00000112532	21	18	6	9	27	43	30	18	10	6
ENSMUSG00000112533	3	5	0	0	6	0	0	0	6	4
ENSMUSG00000112534	0	0	0	0	0	0	0	0	0	0
ENSMUSG00000039315	13	8	3	23	3	13	30	12	14	9
ENSMUSG00000112536	0	0	0	0	0	0	0	0	0	0
ENSMUSG00000112537	0	0	0	0	0	0	0	0	0	0
ENSMUSG00000106148	0	0	0	0	0	0	0	0	0	0
ENSMUSG00000106144	3	0	3	4	6	2	0	3	0	0
ENSMUSG00000106146	0	0	0	0	0	0	0	0	3	0
ENSMUSG00000106140	83	63	81	54	58	60	31	77	34	45
ENSMUSG00000106141	0	0	0	0	0	0	0	0	0	0
ENSMUSG00000106143	0	0	0	0	0	0	0	0	0	0
ENSMUSG00000028417	38	15	35	9	30	50	14	34	21	24
ENSMUSG00000092275	0	3	0	0	0	0	0	0	0	0

ENSMUSG00000092276	0	0	0	0	0	0	0	0	0	0
ENSMUSG00000092270	0	0	0	4	0	0	0	0	0	0
ENSMUSG00000092271	0	0	0	0	0	0	0	0	0	0
ENSMUSG00000092272	0	0	0	0	0	0	3	0	0	0
ENSMUSG00000109649	0	0	0	0	0	0	0	0	0	0
ENSMUSG00000071952	0	0	0	0	0	0	0	0	0	0
ENSMUSG00000090667	10	0	0	0	6	8	33	12	18	9
ENSMUSG00000090225	0	0	0	0	0	0	0	0	0	0
ENSMUSG00000101347	0	0	0	0	0	0	0	0	0	0
ENSMUSG00000090227	0	0	0	3	0	0	0	0	0	0
ENSMUSG00000035678	21	78	71	21	65	26	69	50	66	52
ENSMUSG00000109643	3	12	3	5	3	0	0	0	5	0
ENSMUSG00000020627	89	76	100	55	53	231	407	187	228	191
ENSMUSG00000020624	0	0	0	0	0	0	0	0	0	0
ENSMUSG00000020622	3	0	0	0	0	4	0	0	3	0
ENSMUSG00000110002	0	0	0	0	0	0	0	0	0	0
ENSMUSG00000110003	0	0	0	0	0	0	0	0	0	0
ENSMUSG00000110000	0	0	0	0	0	0	0	0	0	0
ENSMUSG00000110006	0	0	0	0	0	0	0	0	0	0
ENSMUSG00000110004	0	0	0	0	0	0	0	0	0	0
ENSMUSG00000110005	0	0	0	0	0	0	0	0	0	0
ENSMUSG00000110008	0	16	12	3	1	11	0	0	0	5
ENSMUSG00000113667	0	0	0	0	0	0	0	0	0	0
ENSMUSG00000113666	0	0	0	0	0	3	0	0	3	0
ENSMUSG00000113664	0	1	0	0	0	0	0	0	0	0
ENSMUSG00000113663	0	0	0	0	0	0	0	0	0	0
ENSMUSG00000105147	0	0	0	0	0	0	0	0	0	0
ENSMUSG00000113661	0	0	0	0	0	0	0	0	0	0
ENSMUSG00000113669	20	21	10	12	17	0	38	24	31	25
ENSMUSG00000022085	0	0	3	0	0	2	0	0	0	0
ENSMUSG00000109138	0	0	0	0	0	0	0	0	0	0
ENSMUSG00000109139	0	0	0	0	0	0	0	0	0	0
ENSMUSG00000105493	0	0	0	0	0	0	0	0	0	0
ENSMUSG00000105492	0	0	0	0	0	0	0	0	0	0
ENSMUSG00000105490	0	0	0	0	0	0	0	0	0	0
ENSMUSG00000096883	3	8	0	0	3	12	39	9	3	0
ENSMUSG00000046974	0	0	0	0	0	0	6	0	3	0
ENSMUSG00000046975	0	0	0	0	0	0	0	0	0	0
ENSMUSG00000096885	0	0	0	0	0	0	0	0	0	0
ENSMUSG00000046971	0	0	4	2	0	0	0	0	0	0
ENSMUSG00000104971	0	0	0	0	0	0	0	0	0	0
ENSMUSG00000043453	0	0	0	0	0	0	0	0	0	0
ENSMUSG00000025396	0	0	0	0	0	0	3	0	0	0
ENSMUSG00000104976	0	0	0	0	0	0	0	0	0	0
ENSMUSG00000070533	0	0	0	0	0	0	0	0	0	0
ENSMUSG00000070532	3	3	9	15	3	14	13	12	6	3
ENSMUSG00000070531	0	0	0	0	0	0	0	0	0	0
ENSMUSG00000070530	0	0	0	0	0	0	0	0	0	0
ENSMUSG00000114249	3	0	0	3	0	5	5	5	0	0
ENSMUSG00000054128	3	0	3	3	3	0	0	0	0	0
ENSMUSG00000105725	0	0	0	0	0	0	0	0	0	0
ENSMUSG00000105727	0	0	0	0	0	0	0	0	0	0
ENSMUSG00000105726	11	8	0	4	5	3	12	15	5	2
ENSMUSG00000105720	6	8	2	8	3	5	9	5	15	4
ENSMUSG00000105723	0	0	0	0	0	0	0	0	1	0
ENSMUSG00000105722	0	0	0	0	0	0	0	0	0	0
ENSMUSG00000105729	0	0	1	0	0	0	3	3	0	0
ENSMUSG00000096225	0	0	0	0	0	0	14	3	5	12

ENSMUSG00000096223	0	0	0	0	0	0	0	0	0	0
ENSMUSG00000096222	0	0	0	0	0	0	0	0	0	0
ENSMUSG00000094575	0	0	0	0	0	0	0	0	0	0
ENSMUSG00000096229	0	0	0	1	0	0	0	0	0	0
ENSMUSG00000096228	0	0	0	0	0	0	0	0	0	4
ENSMUSG00000080566	0	0	0	0	0	0	0	0	0	0
ENSMUSG00000111753	0	0	0	0	0	0	0	0	0	0
ENSMUSG00000111752	0	0	0	0	0	0	3	0	0	0
ENSMUSG00000111755	0	0	0	0	0	0	0	0	0	0
ENSMUSG00000111754	0	0	0	0	0	0	0	0	0	0
ENSMUSG00000100278	0	0	0	0	0	0	0	0	0	0
ENSMUSG00000104454	6	0	4	19	20	8	16	18	21	13
ENSMUSG00000104456	0	0	0	0	0	0	0	0	0	0
ENSMUSG00000112476	0	0	0	0	0	0	0	0	0	0
ENSMUSG00000108770	0	0	0	0	0	0	0	0	0	0
ENSMUSG00000108773	0	0	0	0	0	0	3	0	0	0
ENSMUSG00000108779	0	0	0	0	0	0	0	0	0	0
ENSMUSG00000108778	0	0	0	0	0	0	0	0	0	0
ENSMUSG00000100274	0	0	0	0	0	0	0	0	0	0
ENSMUSG00000112953	0	0	0	0	0	0	0	0	0	0
ENSMUSG00000108512	0	3	0	3	0	2	0	0	0	0
ENSMUSG00000112956	0	0	0	0	0	0	0	0	0	0
ENSMUSG00000109436	0	9	8	0	3	0	13	6	7	9
ENSMUSG00000108517	0	0	0	0	0	0	0	0	0	0
ENSMUSG00000001566	0	0	0	0	0	0	0	0	0	0
ENSMUSG00000082270	0	0	0	0	0	0	0	0	0	0
ENSMUSG00000110587	0	0	0	0	0	0	0	0	0	0
ENSMUSG00000110585	0	2	0	0	2	3	3	0	0	0
ENSMUSG00000110583	0	0	0	0	0	0	0	0	0	0
ENSMUSG00000110582	0	0	0	0	0	0	0	0	0	0
ENSMUSG00000027530	3	0	0	12	0	0	6	0	0	0
ENSMUSG00000102110	0	0	0	0	0	0	0	0	0	0
ENSMUSG00000071489	2	0	0	0	0	0	0	0	3	0
ENSMUSG00000020913	0	0	0	0	0	0	3	0	0	0
ENSMUSG00000090315	0	0	0	0	0	0	0	0	0	0
ENSMUSG00000095146	0	0	0	0	0	0	0	0	0	0
ENSMUSG00000048978	6	0	0	0	0	0	0	3	0	0
ENSMUSG00000095141	0	0	0	0	0	0	0	0	0	0
ENSMUSG00000095140	0	0	0	0	0	0	0	0	0	0
ENSMUSG00000090318	0	0	0	0	0	1	0	0	0	0
ENSMUSG00000095149	0	0	0	0	0	0	0	0	0	0
ENSMUSG00000095148	0	0	0	0	0	0	0	0	0	0
ENSMUSG00000042604	163	185	150	150	115	254	498	257	202	130
ENSMUSG00000046119	0	0	0	0	0	0	0	0	0	0
ENSMUSG00000080495	0	0	0	0	0	0	0	0	0	0
ENSMUSG00000080499	3	0	1	2	1	2	1	2	0	4
ENSMUSG00000080498	0	0	0	0	0	0	0	0	0	0
ENSMUSG00000112340	6	2	3	3	0	2	0	7	6	0
ENSMUSG00000051198	0	0	0	0	0	0	0	0	0	0
ENSMUSG00000086892	0	0	0	0	0	0	0	0	0	0
ENSMUSG00000099253	0	0	3	1	0	0	2	0	3	0
ENSMUSG00000059256	0	0	0	0	0	0	0	0	0	0
ENSMUSG00000052151	163	136	178	240	139	188	382	231	245	215
ENSMUSG00000052157	38	50	35	49	61	12	27	50	51	27
ENSMUSG00000094431	0	0	0	0	0	0	0	0	0	0
ENSMUSG00000080720	6	7	13	13	8	12	18	11	21	20
ENSMUSG00000080722	0	0	0	0	0	0	0	0	0	0
ENSMUSG00000080725	0	0	0	0	0	0	0	0	0	0

ENSMUSG00000080726	0	0	0	0	0	0	0	0	0	0
ENSMUSG00000080729	0	0	0	0	0	0	0	0	0	0
ENSMUSG00000080728	0	0	0	0	0	0	0	0	0	0
ENSMUSG00000096084	0	0	0	0	0	0	0	0	0	0
ENSMUSG00000093664	0	0	0	0	0	0	0	0	0	0
ENSMUSG00000093665	0	0	0	0	0	0	0	0	0	0
ENSMUSG00000093666	0	0	0	0	0	0	0	0	0	0
ENSMUSG00000093660	0	0	0	0	0	0	0	0	0	0
ENSMUSG00000093662	0	0	0	0	0	0	0	0	0	0
ENSMUSG00000093663	0	0	0	0	0	0	0	0	0	0
ENSMUSG00000078840	0	0	0	0	0	0	0	0	0	0
ENSMUSG00000093669	0	0	0	0	0	0	0	0	0	0
ENSMUSG00000086765	30	3	8	16	37	60	3	12	38	17
ENSMUSG00000086764	0	0	0	0	0	0	0	0	0	0
ENSMUSG00000020890	33	18	5	3	8	23	15	11	13	8
ENSMUSG00000098678	14	3	8	21	6	17	6	11	8	5
ENSMUSG00000098679	2	1	1	1	1	3	1	1	2	0
ENSMUSG00000031364	0	0	0	6	0	0	0	0	0	0
ENSMUSG00000077397	0	0	0	0	0	0	0	0	0	0
ENSMUSG00000047643	11	6	10	5	3	9	15	3	14	11
ENSMUSG00000047642	0	3	0	0	3	0	7	0	3	3
ENSMUSG00000047641	0	0	0	2	0	0	3	7	0	3
ENSMUSG00000041583	0	0	0	0	0	0	0	0	0	0
ENSMUSG00000099257	0	0	0	0	0	0	0	0	0	0
ENSMUSG00000077398	0	0	0	0	0	0	0	0	0	0
ENSMUSG00000077399	0	0	0	0	0	0	0	0	0	0
ENSMUSG00000096558	0	0	0	0	0	0	0	0	0	0
ENSMUSG00000033213	28	26	44	77	64	71	115	55	91	52
ENSMUSG00000049737	0	0	0	0	0	0	0	0	0	0
ENSMUSG00000033210	0	0	0	0	0	0	0	0	0	0
ENSMUSG00000033214	32	18	6	15	9	6	3	9	14	0
ENSMUSG00000030091	255	254	316	379	208	372	479	380	469	342
ENSMUSG00000033219	2	0	0	0	0	0	0	0	0	0
ENSMUSG00000030093	3	12	0	0	0	6	12	9	0	0
ENSMUSG00000043165	0	14	3	0	0	3	6	5	3	1
ENSMUSG00000099255	0	0	0	0	0	0	0	1	0	0
ENSMUSG00000082336	2	0	2	0	0	0	0	3	0	0
ENSMUSG00000076448	0	0	0	0	0	0	0	0	0	0
ENSMUSG00000099781	0	0	0	0	0	0	0	0	0	0
ENSMUSG00000099780	0	0	0	0	0	0	0	0	0	0
ENSMUSG00000099783	0	0	0	0	0	0	0	0	0	0
ENSMUSG00000099782	0	0	0	0	0	0	0	0	0	0
ENSMUSG00000099785	0	0	0	0	0	0	0	0	0	0
ENSMUSG00000099784	0	0	0	0	0	0	0	0	0	0
ENSMUSG00000099787	0	0	0	0	0	0	0	0	0	0
ENSMUSG00000099786	0	0	0	0	0	0	0	0	0	0
ENSMUSG00000099789	0	0	0	0	0	0	0	0	0	0
ENSMUSG00000105680	0	0	0	0	0	0	0	0	0	0
ENSMUSG00000101487	0	0	0	0	0	0	0	0	0	0
ENSMUSG00000030543	0	3	3	3	0	0	20	0	0	3
ENSMUSG00000087388	0	0	0	0	0	0	0	0	0	0
ENSMUSG00000030544	0	0	0	0	0	0	3	0	6	0
ENSMUSG00000087382	3	12	18	37	0	27	56	6	61	26
ENSMUSG00000087383	0	0	0	0	0	0	0	6	0	0
ENSMUSG00000030549	0	0	0	7	0	0	0	0	0	0
ENSMUSG00000099258	0	0	0	0	0	0	0	0	0	0
ENSMUSG00000083603	0	2	0	1	4	0	6	0	1	6
ENSMUSG00000083602	0	0	0	0	0	0	0	0	0	0

ENSMUSG00000083601	0	0	0	0	0	0	0	0	0	0
ENSMUSG00000082333	0	0	0	0	0	0	0	0	0	0
ENSMUSG00000100726	0	0	0	0	0	0	0	0	0	0
ENSMUSG00000100727	0	0	0	0	0	0	0	0	0	0
ENSMUSG00000100724	0	0	0	0	0	0	0	0	0	0
ENSMUSG00000100722	0	0	0	0	0	0	0	0	0	0
ENSMUSG00000100723	0	0	0	0	0	0	0	0	0	0
ENSMUSG00000083611	0	0	0	0	0	0	0	0	0	0
ENSMUSG00000100721	0	0	0	0	0	0	0	0	0	0
ENSMUSG00000100728	0	0	0	0	0	0	2	0	0	0
ENSMUSG00000102470	0	0	0	0	0	0	0	0	0	0
ENSMUSG00000102473	0	0	0	0	0	0	0	0	0	0
ENSMUSG00000102478	5	15	23	24	15	9	35	15	26	19
ENSMUSG00000102479	0	0	0	0	0	0	0	0	0	0
ENSMUSG00000106480	0	0	0	0	0	0	0	0	0	0
ENSMUSG00000075139	0	0	0	0	0	0	0	0	0	0
ENSMUSG00000075136	0	0	0	0	0	0	0	0	0	0
ENSMUSG00000075137	0	0	0	0	0	0	0	0	0	0
ENSMUSG00000079495	0	0	7	12	0	0	0	0	5	0
ENSMUSG00000079494	0	0	5	0	0	0	2	6	0	0
ENSMUSG00000088970	0	0	0	0	0	0	0	0	0	0
ENSMUSG00000041399	0	0	0	0	0	1	0	0	0	0
ENSMUSG00000088971	0	0	0	0	0	0	0	0	0	0
ENSMUSG00000087967	0	0	0	0	0	0	0	0	0	0
ENSMUSG00000087964	0	0	0	0	0	0	0	0	0	0
ENSMUSG00000087960	0	0	0	0	0	0	0	0	0	0
ENSMUSG00000087961	0	0	0	0	0	0	0	0	0	0
ENSMUSG00000085528	0	0	0	0	0	0	0	0	0	0
ENSMUSG00000108234	15	3	13	6	2	5	17	18	20	9
ENSMUSG00000088973	0	0	2	1	0	0	2	0	0	0
ENSMUSG00000094069	0	0	0	0	0	0	0	0	0	0
ENSMUSG00000111984	0	0	0	0	0	0	0	0	0	0
ENSMUSG00000088975	0	0	0	0	0	0	0	0	0	0
ENSMUSG00000064588	0	0	0	0	0	0	0	0	0	0
ENSMUSG00000064585	0	0	0	0	0	0	3	0	2	0
ENSMUSG00000064584	0	0	0	0	0	0	0	0	0	0
ENSMUSG00000101405	0	0	0	0	0	0	0	0	0	0
ENSMUSG00000064581	0	0	0	0	0	0	0	0	0	0
ENSMUSG00000111985	0	4	9	7	3	11	8	7	15	15
ENSMUSG00000079722	0	0	0	0	2	0	3	0	0	0
ENSMUSG00000065185	0	0	0	0	0	0	0	0	0	0
ENSMUSG00000065187	0	0	0	0	0	0	0	0	0	0
ENSMUSG00000065181	0	0	0	0	0	0	0	0	0	0
ENSMUSG00000101929	0	0	0	0	0	0	0	0	0	0
ENSMUSG00000101928	0	0	0	0	0	0	0	0	0	0
ENSMUSG00000101927	0	0	0	0	0	0	0	0	0	0
ENSMUSG00000088654	0	0	0	0	0	0	0	0	0	0
ENSMUSG00000088657	0	0	0	0	0	0	0	0	0	0
ENSMUSG00000088656	0	0	0	0	0	0	0	0	0	0
ENSMUSG00000101923	0	0	0	0	0	0	0	0	0	0
ENSMUSG00000101922	0	0	0	0	0	0	0	0	0	0
ENSMUSG00000088653	0	0	0	0	0	0	0	0	0	0
ENSMUSG00000101920	0	0	0	0	0	0	0	0	0	0
ENSMUSG00000095394	0	0	0	0	0	0	0	0	0	0
ENSMUSG00000107189	0	1	0	0	0	0	0	0	0	0
ENSMUSG00000107184	0	0	0	4	0	0	0	0	0	6
ENSMUSG00000107183	0	0	0	0	0	0	0	0	0	0
ENSMUSG00000107181	0	0	0	0	0	0	0	0	1	0

ENSMUSG00000036658	0	0	0	0	0	0	0	0	0	0
ENSMUSG00000095931	0	0	0	0	0	0	0	0	0	0
ENSMUSG00000103406	0	0	0	0	0	0	3	0	0	0
ENSMUSG00000103407	0	0	0	0	0	0	0	0	0	0
ENSMUSG00000103404	0	0	3	3	2	5	17	2	3	0
ENSMUSG00000103402	0	0	0	0	0	0	0	0	0	0
ENSMUSG00000065238	0	0	0	0	0	0	0	0	0	0
ENSMUSG00000065239	0	0	0	0	0	0	0	0	0	0
ENSMUSG00000065237	0	0	0	0	0	0	0	0	0	0
ENSMUSG00000065235	0	0	0	0	0	0	0	0	0	0
ENSMUSG00000034656	189	176	201	227	153	229	331	317	246	250
ENSMUSG00000065233	0	0	0	0	0	0	0	0	0	0
ENSMUSG00000086066	0	0	0	0	0	0	0	0	0	0
ENSMUSG00000063804	15	21	15	12	15	11	6	14	6	26
ENSMUSG00000039555	0	0	0	0	0	0	0	0	0	0
ENSMUSG00000039552	0	0	10	3	2	3	17	0	3	11
ENSMUSG00000113429	0	0	0	0	0	0	0	0	0	0
ENSMUSG00000029700	0	0	28	38	3	2	3	0	3	12
ENSMUSG00000029706	0	0	0	0	0	0	0	0	0	0
ENSMUSG00000024552	0	0	6	0	0	3	0	0	0	0
ENSMUSG00000024553	27	3	8	15	18	6	0	6	6	9
ENSMUSG00000103612	0	0	0	0	0	0	0	0	0	0
ENSMUSG00000097376	0	0	0	0	0	3	0	0	8	12
ENSMUSG00000097377	28	15	3	11	6	9	16	21	8	9
ENSMUSG00000097374	23	12	6	5	11	0	0	3	4	9
ENSMUSG00000073207	17	51	45	41	24	54	64	37	42	54
ENSMUSG00000097372	15	15	3	26	12	14	14	6	15	0
ENSMUSG00000022860	28	37	12	0	6	46	130	52	53	13
ENSMUSG00000022861	95	80	100	99	71	104	122	176	96	127
ENSMUSG00000073208	0	0	0	0	0	0	0	0	0	0
ENSMUSG00000073209	6	6	9	15	9	9	3	5	0	3
ENSMUSG00000097378	17	32	15	21	17	21	60	25	20	22
ENSMUSG00000103298	0	0	0	0	0	0	0	0	0	0
ENSMUSG00000020704	0	0	3	0	0	5	0	9	0	0
ENSMUSG00000020701	35	18	38	21	15	31	54	55	48	26
ENSMUSG00000020703	0	0	0	0	0	0	0	0	0	0
ENSMUSG00000020702	6	3	0	3	0	2	0	3	0	6
ENSMUSG00000054320	52	38	45	23	51	74	57	80	63	45
ENSMUSG00000054321	591	487	608	624	559	635	517	622	582	625
ENSMUSG00000054325	0	0	0	0	0	0	0	0	0	0
ENSMUSG00000105123	0	0	0	0	0	0	0	0	0	3
ENSMUSG00000105122	0	0	0	0	0	0	0	0	0	0
ENSMUSG00000105121	0	0	0	0	0	0	0	0	0	0
ENSMUSG00000114269	0	0	0	0	0	0	3	0	0	0
ENSMUSG00000105126	0	0	0	0	0	0	0	0	0	0
ENSMUSG00000105125	0	0	0	0	0	0	0	0	0	0
ENSMUSG00000105124	0	2	0	0	0	0	0	0	0	0
ENSMUSG00000114262	0	0	0	0	0	0	0	0	0	0
ENSMUSG00000114263	0	0	0	0	0	0	0	0	0	0
ENSMUSG00000105129	0	0	0	0	5	0	3	0	0	0
ENSMUSG00000114261	0	0	0	0	0	0	0	0	0	0
ENSMUSG00000114265	0	0	0	0	0	0	0	0	0	0
ENSMUSG00000050138	0	3	6	3	0	0	8	0	0	9
ENSMUSG00000054498	0	0	0	0	0	0	0	0	0	0
ENSMUSG00000050132	36	21	32	27	29	14	17	33	37	33
ENSMUSG00000067276	44	148	82	69	32	87	285	127	138	123
ENSMUSG00000057674	0	0	0	0	0	0	0	0	0	0
ENSMUSG00000093421	0	0	0	0	0	0	0	0	0	0

ENSMUSG00000111364	0	0	0	0	0	0	0	0	0	3
ENSMUSG00000095154	0	0	0	0	0	0	0	0	0	0
ENSMUSG00000111365	2	0	0	0	6	0	8	0	3	0
ENSMUSG00000073811	0	0	0	0	0	0	0	0	0	0
ENSMUSG00000108570	0	0	0	0	0	0	0	0	0	0
ENSMUSG00000108577	0	0	0	0	0	0	0	0	0	0
ENSMUSG00000108576	0	0	0	0	0	0	0	0	0	0
ENSMUSG00000104294	0	0	0	0	0	0	0	0	0	0
ENSMUSG00000104295	0	0	0	0	0	0	0	0	0	0
ENSMUSG00000039224	0	9	0	0	0	0	3	0	0	3
ENSMUSG00000108579	0	0	0	0	0	0	0	0	0	0
ENSMUSG00000104299	9	3	6	6	6	6	11	6	3	0
ENSMUSG00000021070	77	71	75	99	41	77	171	68	91	58
ENSMUSG00000065319	0	0	0	0	1	0	0	2	0	0
ENSMUSG00000111862	0	0	3	0	0	5	20	11	8	0
ENSMUSG00000101216	0	0	0	0	0	0	0	0	0	0
ENSMUSG00000101118	0	0	0	0	0	0	0	0	0	0
ENSMUSG00000101119	0	0	0	0	0	0	0	0	0	0
ENSMUSG00000101116	0	0	0	0	0	0	0	0	0	0
ENSMUSG00000060726	3	0	3	3	0	0	0	0	0	0
ENSMUSG00000101114	0	0	0	0	0	0	0	0	0	0
ENSMUSG00000060724	0	0	0	0	0	0	0	0	0	0
ENSMUSG00000101112	0	0	0	0	0	0	0	0	5	0
ENSMUSG00000101113	0	0	0	0	0	0	0	3	0	0
ENSMUSG00000101110	0	0	0	0	0	0	0	0	0	0
ENSMUSG00000111342	0	0	0	0	0	0	0	0	0	0
ENSMUSG00000111343	9	23	29	9	26	11	44	25	70	20
ENSMUSG00000111344	0	0	0	0	0	0	0	0	0	0
ENSMUSG00000064915	0	0	0	0	0	0	0	0	0	0
ENSMUSG00000091006	0	0	0	0	0	0	0	0	0	0
ENSMUSG00000091004	0	0	0	0	0	0	0	0	0	0
ENSMUSG00000091005	0	0	0	0	0	0	0	0	1	0
ENSMUSG00000091002	0	6	3	0	0	0	0	3	0	6
ENSMUSG00000091003	3	0	0	0	0	0	0	0	0	0
ENSMUSG00000026931	3	0	1	8	0	0	3	0	1	1
ENSMUSG00000026936	0	0	0	0	0	0	0	0	0	0
ENSMUSG00000026937	0	2	5	7	5	3	0	8	0	0
ENSMUSG00000095785	0	0	0	0	0	0	0	0	0	0
ENSMUSG00000111611	0	0	0	0	0	0	3	0	0	0
ENSMUSG00000081038	0	0	0	0	0	0	0	0	0	0
ENSMUSG00000095786	4	0	0	0	0	0	0	0	0	0
ENSMUSG00000095780	0	0	0	0	0	0	0	0	0	0
ENSMUSG00000095782	0	0	0	0	0	0	0	0	0	0
ENSMUSG00000095789	72	69	79	68	40	84	86	64	90	73
ENSMUSG00000095788	47	40	42	155	39	29	74	18	79	46
ENSMUSG00000022034	99	91	145	145	77	33	169	81	103	56
ENSMUSG00000022033	113	89	130	157	86	45	159	97	43	72
ENSMUSG00000027048	3	6	3	6	0	9	24	3	29	6
ENSMUSG00000042448	0	0	0	0	0	0	0	0	0	0
ENSMUSG00000053773	0	0	0	0	0	0	0	0	0	0
ENSMUSG00000087407	0	0	0	0	0	0	0	0	0	0
ENSMUSG00000094789	0	0	0	0	0	0	0	0	0	0
ENSMUSG00000111159	0	0	0	0	0	0	0	0	0	0
ENSMUSG00000094782	0	0	0	0	0	0	0	0	0	0
ENSMUSG00000094784	0	0	0	0	0	0	0	0	0	0
ENSMUSG00000094785	0	0	0	2	0	0	0	0	0	0
ENSMUSG00000086569	0	0	0	0	0	0	0	0	2	0
ENSMUSG00000086563	0	0	0	0	0	0	0	0	0	0

ENSMUSG00000086564	22	20	19	63	20	25	37	39	89	42
ENSMUSG00000095935	0	0	0	0	0	0	0	0	0	0
ENSMUSG00000062814	0	0	0	0	0	0	0	0	0	0
ENSMUSG00000062818	0	3	0	4	6	0	0	0	3	0
ENSMUSG00000023345	301	256	233	216	210	267	232	187	228	236
ENSMUSG00000087401	0	0	0	0	0	0	0	0	0	0
ENSMUSG00000103394	0	0	3	0	0	7	0	6	2	0
ENSMUSG00000108156	0	0	0	0	0	0	0	0	0	0
ENSMUSG00000103051	0	0	0	0	0	0	0	0	3	0
ENSMUSG00000092168	0	0	0	0	0	0	0	0	0	0
ENSMUSG00000093311	0	0	0	0	0	0	0	0	0	0
ENSMUSG00000100068	0	0	0	0	0	0	0	2	0	0
ENSMUSG00000049532	286	332	314	240	289	319	666	436	489	409
ENSMUSG00000049530	0	0	0	0	0	0	0	0	0	0
ENSMUSG00000049538	0	0	12	0	0	3	15	9	9	0
ENSMUSG00000103699	0	0	0	0	0	0	0	0	0	0
ENSMUSG00000036510	6	29	28	2	9	3	21	12	6	12
ENSMUSG00000099453	0	0	0	0	0	0	0	0	0	0
ENSMUSG00000112706	0	0	0	1	0	0	3	0	0	0
ENSMUSG00000112705	4	0	4	4	0	3	0	0	2	5
ENSMUSG00000112704	0	0	0	0	0	0	0	0	0	0
ENSMUSG00000112703	0	0	0	0	0	0	0	0	0	0
ENSMUSG00000112702	3	0	0	1	2	0	0	0	0	0
ENSMUSG00000099458	0	0	0	0	0	0	0	0	0	0
ENSMUSG00000096921	0	0	0	0	0	0	0	0	0	0
ENSMUSG00000096922	0	0	0	0	0	0	0	0	0	0
ENSMUSG00000049281	126	68	52	154	72	62	354	242	171	59
ENSMUSG00000049280	0	0	0	0	0	0	0	0	0	0
ENSMUSG00000096927	0	0	0	0	0	1	1	0	0	0
ENSMUSG00000106339	8	8	14	10	11	9	31	20	31	8
ENSMUSG00000002578	22	31	40	79	41	27	44	33	47	22
ENSMUSG00000106330	0	0	0	0	0	0	0	0	0	0
ENSMUSG00000106336	0	0	0	0	0	0	0	0	0	0
ENSMUSG00000113252	0	6	0	0	0	0	0	0	0	3
ENSMUSG00000113253	0	0	0	0	0	0	0	0	0	0
ENSMUSG00000113251	0	0	0	0	0	0	0	0	0	0
ENSMUSG00000113254	0	0	0	0	0	0	0	2	0	3
ENSMUSG00000103055	0	0	0	0	0	0	0	0	0	0
ENSMUSG00000113259	0	0	0	0	0	0	0	0	0	0
ENSMUSG00000047253	0	0	0	0	0	0	0	0	0	0
ENSMUSG00000079392	0	0	0	0	0	0	0	0	0	0
ENSMUSG00000047257	3	2	0	0	5	0	8	14	6	3
ENSMUSG00000047259	0	0	0	0	0	0	0	0	0	3
ENSMUSG00000109309	0	0	0	0	0	0	0	0	0	0
ENSMUSG00000109308	0	0	0	0	0	0	0	0	0	0
ENSMUSG00000003227	38	18	45	57	28	97	118	85	88	67
ENSMUSG00000109301	0	0	3	0	0	0	0	0	9	0
ENSMUSG00000109305	6	4	4	0	2	4	14	4	8	0
ENSMUSG00000082712	0	0	0	2	0	0	0	0	0	0
ENSMUSG00000082713	0	0	0	0	0	0	0	0	0	0
ENSMUSG00000082710	0	0	0	0	0	0	0	0	0	0
ENSMUSG00000082717	0	0	0	0	0	0	0	0	0	0
ENSMUSG00000082714	0	0	0	0	0	0	0	0	0	0
ENSMUSG00000083049	7	10	14	8	14	9	9	12	6	12
ENSMUSG00000103056	0	0	0	1	2	2	0	2	2	0
ENSMUSG00000083041	3	16	7	10	5	6	20	19	13	6
ENSMUSG00000083042	0	0	0	0	0	0	0	0	0	0
ENSMUSG00000083044	9	11	24	20	17	17	6	11	12	21

ENSMUSG00000083047	0	0	0	0	0	0	0	0	0	0
ENSMUSG00000083046	0	0	0	0	0	0	0	0	0	0
ENSMUSG00000114701	0	0	3	0	0	3	0	3	0	4
ENSMUSG00000114700	0	0	0	0	0	0	0	0	0	0
ENSMUSG00000114703	0	0	4	2	0	0	0	0	0	0
ENSMUSG00000114702	0	0	0	0	0	0	0	0	0	0
ENSMUSG00000114707	42	71	56	62	24	49	45	69	67	95
ENSMUSG00000114706	0	0	0	0	0	0	0	0	0	0
ENSMUSG00000110633	0	0	3	0	0	3	0	8	0	0
ENSMUSG00000110632	0	0	0	0	0	0	0	0	0	0
ENSMUSG00000110634	0	0	0	0	0	0	0	0	0	3
ENSMUSG00000110636	0	0	0	2	2	0	4	0	2	0
ENSMUSG00000110639	0	0	0	0	0	0	0	0	0	0
ENSMUSG00000110638	3	14	15	22	8	27	26	42	28	24
ENSMUSG00000084459	1	1	1	1	1	0	1	1	1	0
ENSMUSG00000005107	181	157	177	245	98	224	273	281	231	246
ENSMUSG00000022015	36	11	15	9	15	53	54	20	30	14
ENSMUSG00000084456	0	0	0	0	0	1	0	0	0	0
ENSMUSG00000099586	0	0	0	0	0	0	0	0	0	0
ENSMUSG00000025588	59	28	67	37	52	60	39	98	79	57
ENSMUSG00000025582	29	39	21	29	14	27	30	14	18	9
ENSMUSG00000070099	0	0	1	0	0	0	0	0	0	1
ENSMUSG00000082688	9	7	3	1	0	2	4	5	0	5
ENSMUSG00000100169	3	0	0	0	0	0	0	0	0	0
ENSMUSG00000026582	162	150	187	163	104	467	317	204	256	175
ENSMUSG00000026587	114	156	142	121	82	157	234	181	98	215
ENSMUSG00000026589	308	255	297	254	290	519	778	442	698	490
ENSMUSG00000079120	0	0	0	0	0	0	0	0	0	0
ENSMUSG00000068263	117	125	122	115	103	103	221	163	201	205
ENSMUSG00000031481	0	0	2	0	0	0	0	13	0	0
ENSMUSG00000006488	0	0	0	0	0	0	0	0	0	0
ENSMUSG00000082687	0	0	2	0	0	0	0	0	0	0
ENSMUSG00000113790	0	0	0	0	0	0	0	0	0	0
ENSMUSG00000085051	0	4	3	0	6	6	3	0	6	0
ENSMUSG00000085053	0	0	0	0	0	0	0	0	3	0
ENSMUSG00000085057	0	0	0	8	0	0	0	0	3	0
ENSMUSG00000085059	0	0	2	0	0	0	0	0	0	0
ENSMUSG00000026058	0	0	0	3	0	5	0	3	0	0
ENSMUSG00000071311	3	4	0	10	0	15	13	4	10	3
ENSMUSG00000033826	55	65	72	92	61	99	255	106	150	146
ENSMUSG00000081994	0	0	0	0	0	0	0	0	0	0
ENSMUSG00000081991	0	0	0	0	0	0	2	0	0	0
ENSMUSG00000081993	0	0	0	0	0	0	0	0	0	0
ENSMUSG00000098193	0	0	0	0	0	0	0	0	0	0
ENSMUSG00000041849	270	255	340	264	267	382	440	307	374	347
ENSMUSG00000098190	0	0	0	0	0	0	0	0	0	0
ENSMUSG00000098197	0	0	0	0	0	0	0	0	0	0
ENSMUSG00000098195	0	0	0	0	0	0	0	0	0	0
ENSMUSG00000112773	0	0	0	0	0	0	0	0	0	0
ENSMUSG00000041842	29	54	46	15	23	32	93	43	37	46
ENSMUSG00000099860	0	0	0	0	0	0	0	0	0	0
ENSMUSG00000099861	0	0	0	0	0	0	0	0	0	0
ENSMUSG00000099862	0	0	0	0	0	0	0	0	0	0
ENSMUSG00000099863	0	0	0	0	0	0	0	0	0	0
ENSMUSG00000099864	0	0	0	0	0	0	0	0	0	0
ENSMUSG00000099866	0	0	0	0	0	0	0	0	0	0
ENSMUSG00000099867	0	0	0	0	0	0	0	0	0	0
ENSMUSG00000090643	0	0	0	0	0	2	0	0	0	0

ENSMUSG00000112774	0	0	0	0	0	0	0	0	0	3
ENSMUSG00000074575	0	0	0	3	3	0	0	0	0	0
ENSMUSG00000090642	0	0	0	0	0	0	0	0	0	0
ENSMUSG00000074579	292	327	393	358	214	323	274	329	359	331
ENSMUSG00000112775	0	0	0	0	0	0	0	0	0	0
ENSMUSG00000111986	0	0	0	0	0	0	0	0	0	0
ENSMUSG00000026405	0	0	3	0	0	0	0	0	3	0
ENSMUSG00000111981	0	0	0	0	0	0	0	0	0	0
ENSMUSG00000081370	0	0	0	3	0	0	0	2	0	0
ENSMUSG00000109047	1	0	0	0	0	0	0	0	0	0
ENSMUSG00000081377	0	0	0	0	0	0	0	0	0	0
ENSMUSG00000081379	0	0	0	0	0	0	0	0	0	0
ENSMUSG00000109040	0	0	0	0	0	0	0	0	0	0
ENSMUSG00000111983	0	0	0	0	0	0	0	0	0	0
ENSMUSG00000109041	0	0	0	0	0	0	0	0	0	0
ENSMUSG00000019942	225	153	290	216	140	105	320	188	118	92
ENSMUSG00000072934	0	0	3	0	0	0	0	0	0	0
ENSMUSG00000072931	0	0	0	0	0	0	0	0	0	0
ENSMUSG00000072930	0	0	0	0	0	0	0	0	0	0
ENSMUSG00000090505	0	0	0	0	0	0	0	0	0	0
ENSMUSG00000084825	0	0	0	0	0	0	0	0	0	0
ENSMUSG00000090509	0	0	0	0	0	0	0	0	0	0
ENSMUSG00000084829	0	0	0	0	0	0	0	0	0	0
ENSMUSG00000077218	0	0	0	0	0	0	0	0	0	0
ENSMUSG00000038917	5	18	12	0	5	14	24	0	22	2
ENSMUSG00000038916	4	0	6	3	9	9	3	3	1	0
ENSMUSG00000077215	0	0	0	0	0	0	0	0	0	0
ENSMUSG00000077214	0	0	0	0	0	0	0	0	0	0
ENSMUSG00000105494	1	0	0	0	0	0	0	0	0	0
ENSMUSG00000083153	0	0	0	0	0	0	0	0	2	0
ENSMUSG00000110688	0	0	0	0	0	0	0	0	0	0
ENSMUSG00000031535	2	5	0	0	3	0	5	5	0	6
ENSMUSG00000083554	0	0	0	0	0	0	0	0	0	0
ENSMUSG00000083156	0	0	0	0	0	0	0	0	0	0
ENSMUSG00000043144	0	0	3	3	3	0	0	0	0	3
ENSMUSG00000043145	0	0	0	0	0	0	0	0	0	0
ENSMUSG00000110687	0	0	0	0	0	0	0	0	0	0
ENSMUSG00000110680	0	0	0	0	0	0	0	0	0	0
ENSMUSG00000110681	0	0	0	0	0	0	0	0	0	0
ENSMUSG00000025043	0	0	0	0	0	0	0	0	0	0
ENSMUSG00000026560	0	0	0	0	0	0	0	0	0	0
ENSMUSG00000113422	0	0	0	0	0	0	0	0	0	0
ENSMUSG00000087884	0	0	0	0	0	0	0	0	0	0
ENSMUSG00000082051	17	8	5	12	8	8	15	12	12	5
ENSMUSG00000030666	6	28	12	5	6	8	60	26	7	12
ENSMUSG00000082053	0	0	0	0	0	0	0	0	0	0
ENSMUSG00000082052	0	0	0	0	0	3	3	0	0	0
ENSMUSG00000088039	0	0	0	0	0	0	0	0	0	0
ENSMUSG00000082054	0	0	0	0	0	0	0	0	0	0
ENSMUSG00000087029	0	0	3	0	0	0	0	0	0	0
ENSMUSG00000087028	18	24	5	12	19	24	29	34	20	35
ENSMUSG00000082059	0	0	0	0	0	0	0	0	0	0
ENSMUSG00000082058	0	0	0	0	0	0	0	0	0	0
ENSMUSG00000087024	0	3	3	0	0	14	6	10	4	4
ENSMUSG00000088031	0	0	0	0	0	0	0	0	0	3
ENSMUSG00000087022	11	0	3	8	6	13	5	16	3	12
ENSMUSG00000030669	0	0	4	0	0	0	13	0	13	10
ENSMUSG00000087020	41	21	27	40	15	14	19	34	35	21

ENSMUSG00000101354	0	0	0	0	0	0	0	0	0	0
ENSMUSG00000101357	1	0	0	0	0	0	6	0	9	0
ENSMUSG00000101350	0	0	0	0	0	0	0	0	0	0
ENSMUSG00000101351	0	0	0	1	0	0	0	0	0	0
ENSMUSG00000101352	0	0	0	0	0	0	0	0	0	0
ENSMUSG00000101353	0	0	0	0	0	0	0	0	0	0
ENSMUSG00000085889	0	0	0	0	0	0	0	0	0	0
ENSMUSG00000085880	0	0	0	0	0	0	0	0	0	0
ENSMUSG00000103610	0	0	0	0	0	0	0	0	0	0
ENSMUSG00000087886	0	0	0	0	0	2	0	0	0	2
ENSMUSG00000038300	0	0	2	0	0	4	0	0	0	0
ENSMUSG00000103267	0	0	0	0	0	0	0	0	0	0
ENSMUSG00000100089	0	0	0	0	0	0	0	0	0	0
ENSMUSG00000103265	5	0	0	1	0	0	0	4	0	0
ENSMUSG00000103262	0	0	0	0	0	0	0	0	0	0
ENSMUSG00000103263	0	0	0	0	0	0	0	0	0	0
ENSMUSG00000103260	0	0	0	0	0	2	0	0	0	0
ENSMUSG00000103261	17	14	12	2	18	18	11	5	20	5
ENSMUSG00000100083	0	0	0	0	0	0	0	0	0	0
ENSMUSG00000100082	0	0	0	0	0	0	0	0	0	0
ENSMUSG00000100080	0	0	0	0	0	0	0	0	0	0
ENSMUSG00000100087	0	0	0	0	0	0	0	0	0	0
ENSMUSG00000100086	0	0	0	0	0	0	0	0	0	0
ENSMUSG00000100085	0	0	0	0	0	0	0	0	0	0
ENSMUSG00000103269	0	0	0	0	0	0	0	0	0	0
ENSMUSG00000101686	0	0	0	0	0	0	0	0	0	0
ENSMUSG00000040724	170	538	623	458	101	560	375	538	478	625
ENSMUSG00000101684	0	0	0	0	0	0	0	0	0	0
ENSMUSG00000101685	0	0	0	0	2	0	0	0	0	0
ENSMUSG00000101683	0	0	0	0	0	0	0	0	0	0
ENSMUSG00000101681	0	0	0	0	0	0	0	0	0	0
ENSMUSG00000040729	431	410	392	354	399	509	444	558	477	544
ENSMUSG00000101688	0	0	0	0	0	0	0	0	0	0
ENSMUSG00000101689	0	0	0	0	0	0	0	0	0	0
ENSMUSG00000107681	0	0	0	0	0	0	0	0	0	0
ENSMUSG00000107683	0	0	0	0	0	0	0	0	0	0
ENSMUSG00000107684	1	0	0	0	2	0	0	0	0	0
ENSMUSG00000089349	0	0	0	0	0	0	0	0	0	0
ENSMUSG00000084598	0	0	0	0	0	0	0	0	0	0
ENSMUSG00000107687	0	0	0	0	0	0	0	0	0	0
ENSMUSG00000089344	0	0	0	0	0	0	2	0	0	0
ENSMUSG00000089341	0	0	0	0	0	0	0	0	0	0
ENSMUSG00000089342	0	0	0	0	0	0	1	0	0	0
ENSMUSG00000084591	0	0	0	0	0	0	0	0	0	0
ENSMUSG00000103190	0	0	0	0	0	0	0	0	0	0
ENSMUSG00000103193	0	0	0	0	0	0	0	0	1	0
ENSMUSG00000103196	0	0	0	0	0	0	0	0	0	0
ENSMUSG00000069248	0	0	0	0	0	3	0	9	0	0
ENSMUSG00000032750	121	112	131	149	113	137	270	185	215	178
ENSMUSG00000084041	0	0	0	0	0	0	0	0	0	0
ENSMUSG00000084043	0	0	0	3	0	0	0	0	0	0
ENSMUSG00000084044	0	0	0	0	0	0	0	0	0	0
ENSMUSG00000105638	0	0	0	0	0	0	0	0	0	0
ENSMUSG00000084046	0	0	0	0	0	0	0	0	0	0
ENSMUSG00000075302	3	6	15	0	5	0	27	0	0	6
ENSMUSG00000030283	14	30	39	18	12	29	97	57	66	9
ENSMUSG00000095813	0	0	0	0	0	0	0	0	0	0
ENSMUSG00000075304	0	12	0	3	6	0	6	3	13	6

ENSMUSG00000100555	0	3	2	5	0	2	16	6	22	9
ENSMUSG00000065884	0	0	0	0	0	0	0	0	0	0
ENSMUSG00000065881	0	0	0	0	0	0	0	0	0	0
ENSMUSG00000065882	0	0	0	0	0	0	0	0	0	0
ENSMUSG00000065889	0	0	0	0	0	0	0	0	0	0
ENSMUSG00000022589	0	0	0	0	0	0	0	0	0	0
ENSMUSG00000022580	3	6	0	6	3	9	27	6	12	6
ENSMUSG00000022583	0	0	0	0	0	0	6	0	0	0
ENSMUSG00000022582	4	0	0	54	0	13	4	0	13	0
ENSMUSG00000060967	0	0	0	0	0	0	0	0	0	0
ENSMUSG00000096356	0	0	3	0	0	0	0	0	0	0
ENSMUSG00000064428	0	0	0	0	0	0	0	0	0	0
ENSMUSG00000107027	0	0	0	0	0	0	0	0	0	0
ENSMUSG00000107022	0	0	0	0	0	0	6	0	0	0
ENSMUSG00000107028	0	2	0	2	2	0	2	0	5	2
ENSMUSG00000107029	3	0	0	0	0	5	9	0	2	0
ENSMUSG00000023185	0	0	0	0	0	0	0	0	0	0
ENSMUSG00000012282	0	0	0	0	0	3	0	0	0	0
ENSMUSG00000058953	0	0	0	0	0	0	0	0	0	0
ENSMUSG00000103618	0	0	0	0	0	0	0	0	0	0
ENSMUSG00000085316	9	11	6	9	14	9	18	10	22	4
ENSMUSG00000085314	3	3	6	0	12	3	5	0	3	12
ENSMUSG00000085312	0	0	0	0	0	0	0	0	0	0
ENSMUSG00000085318	0	0	0	0	0	0	0	0	0	0
ENSMUSG00000105633	0	0	0	0	6	0	0	0	0	2
ENSMUSG00000033581	235	233	287	281	285	302	271	154	392	192
ENSMUSG00000028661	0	0	0	0	3	0	0	0	3	3
ENSMUSG00000028664	141	97	104	76	74	121	239	131	165	158
ENSMUSG00000088596	0	0	0	0	0	0	0	0	0	0
ENSMUSG00000088597	0	0	0	0	0	0	0	0	0	0
ENSMUSG00000088594	0	0	0	0	0	0	0	0	0	0
ENSMUSG00000088595	0	0	0	0	0	0	0	0	0	0
ENSMUSG00000088598	0	0	0	0	0	0	0	0	0	0
ENSMUSG00000095447	0	0	0	0	0	0	0	0	0	0
ENSMUSG00000050463	0	3	0	0	0	0	0	0	9	0
ENSMUSG00000029134	53	73	59	62	42	64	45	77	51	56
ENSMUSG00000061614	0	0	0	0	0	0	0	0	0	0
ENSMUSG00000113011	0	0	0	0	0	0	0	0	0	0
ENSMUSG00000061616	0	0	0	0	0	0	0	0	0	0
ENSMUSG00000113013	0	0	0	0	0	0	0	0	0	0
ENSMUSG00000113016	0	0	0	0	0	0	0	0	0	0
ENSMUSG00000113017	0	0	0	3	0	0	0	0	0	0
ENSMUSG00000113018	0	0	0	0	0	0	0	0	0	0
ENSMUSG00000059334	0	0	0	0	0	0	0	0	3	0
ENSMUSG00000059336	69	53	88	94	81	132	148	138	117	116
ENSMUSG00000063615	0	0	0	0	0	0	0	0	0	0
ENSMUSG00000109548	0	0	0	0	0	0	0	0	0	0
ENSMUSG00000111528	3	16	15	18	22	17	4	10	9	12
ENSMUSG00000109541	0	0	0	0	0	0	0	0	0	0
ENSMUSG00000109540	0	0	0	0	0	0	0	0	0	0
ENSMUSG00000109543	0	0	0	0	0	0	0	0	0	0
ENSMUSG00000109542	0	0	0	0	0	0	0	0	0	0
ENSMUSG00000109545	0	0	0	0	0	0	0	0	0	0
ENSMUSG00000109544	0	0	0	0	0	0	0	0	0	0
ENSMUSG00000109547	0	0	0	0	0	0	0	0	0	0
ENSMUSG00000109546	0	0	0	0	0	0	0	0	0	0
ENSMUSG00000106642	0	0	0	0	0	0	0	0	0	0
ENSMUSG00000050818	0	0	0	0	0	6	0	0	0	0

ENSMUSG00000106640	0	0	0	0	0	0	0	0	0	0
ENSMUSG00000106647	0	0	0	0	0	0	0	0	0	0
ENSMUSG00000066060	0	0	0	0	0	0	0	3	0	0
ENSMUSG00000106644	0	2	0	0	0	0	0	0	0	2
ENSMUSG00000050813	0	0	0	0	0	0	0	0	0	0
ENSMUSG00000050810	0	0	0	0	0	0	0	0	0	0
ENSMUSG00000106648	38	70	41	38	25	46	39	71	57	71
ENSMUSG00000050815	0	0	0	0	0	0	0	0	0	0
ENSMUSG00000097504	51	77	60	55	60	70	52	85	78	59
ENSMUSG00000038496	58	35	23	33	38	74	64	63	56	73
ENSMUSG00000097502	0	0	0	3	3	3	0	0	0	0
ENSMUSG00000104645	0	0	0	0	0	0	0	0	0	0
ENSMUSG00000104644	0	0	0	0	0	0	0	0	0	0
ENSMUSG00000104647	0	0	0	0	0	0	0	0	0	0
ENSMUSG00000038498	0	0	0	0	0	0	0	0	0	0
ENSMUSG00000104640	0	0	0	0	0	0	0	0	0	0
ENSMUSG00000104643	0	0	0	0	0	0	0	0	0	0
ENSMUSG00000051499	138	98	149	121	107	90	96	169	132	105
ENSMUSG00000051498	102	91	162	227	174	186	289	134	167	146
ENSMUSG00000051490	0	0	0	26	0	6	0	9	9	0
ENSMUSG00000051493	0	0	0	0	0	0	0	0	0	0
ENSMUSG00000051497	0	0	18	50	0	0	6	6	0	12
ENSMUSG00000114549	0	0	0	0	0	0	0	0	0	0
ENSMUSG00000114548	0	0	0	0	0	0	0	0	0	0
ENSMUSG00000114546	0	0	0	0	0	0	0	0	0	0
ENSMUSG00000114544	2	1	8	0	2	0	5	20	4	9
ENSMUSG00000114543	0	0	0	0	0	0	0	0	0	0
ENSMUSG00000114542	0	0	0	0	0	0	0	0	0	0
ENSMUSG00000114541	0	0	0	0	0	0	0	0	0	0
ENSMUSG00000066392	5	8	8	23	9	36	113	29	12	24
ENSMUSG00000111484	0	0	0	0	0	0	0	0	0	0
ENSMUSG00000112878	0	0	0	0	0	0	0	0	0	0
ENSMUSG00000100936	0	0	0	0	0	0	2	0	0	0
ENSMUSG00000112871	0	0	0	0	0	0	0	0	0	0
ENSMUSG00000112870	0	0	0	0	0	0	0	0	0	0
ENSMUSG00000112873	0	0	0	0	0	0	0	0	0	0
ENSMUSG00000112872	0	0	0	0	0	0	0	0	0	0
ENSMUSG00000112876	7	3	0	0	0	3	5	3	4	0
ENSMUSG00000092222	0	0	0	0	0	0	0	0	0	0
ENSMUSG00000091908	2	5	0	0	2	4	4	2	2	2
ENSMUSG00000091903	0	0	0	0	0	0	0	0	0	0
ENSMUSG00000091902	0	0	0	0	0	0	0	0	0	0
ENSMUSG00000109892	0	0	0	0	0	0	0	0	0	0
ENSMUSG00000109891	0	0	0	0	0	0	0	0	0	0
ENSMUSG00000109890	0	0	0	0	0	0	0	3	0	0
ENSMUSG00000109897	0	0	0	2	0	0	0	0	0	0
ENSMUSG00000109896	0	0	0	0	0	0	0	0	0	0
ENSMUSG00000109895	0	0	0	0	0	0	0	0	0	0
ENSMUSG00000114091	0	0	0	0	0	0	0	0	0	0
ENSMUSG00000114090	0	0	0	0	0	0	0	0	0	0
ENSMUSG00000114092	0	0	0	0	0	0	3	0	0	0
ENSMUSG00000080684	2	0	0	0	0	2	0	0	2	0
ENSMUSG00000114097	0	0	0	0	0	0	0	0	0	0
ENSMUSG00000114096	0	0	0	0	0	0	0	0	0	0
ENSMUSG00000025747	240	201	274	267	189	188	482	243	317	273
ENSMUSG00000110734	0	0	0	0	0	0	0	0	0	0
ENSMUSG00000110736	0	0	0	0	0	0	0	0	0	0
ENSMUSG00000058172	0	0	0	0	0	0	0	0	0	0

ENSMUSG00000110739	0	0	0	0	0	0	0	0	0	0
ENSMUSG00000050366	0	0	0	3	0	0	0	0	3	0
ENSMUSG00000056910	0	0	0	0	0	0	0	0	0	0
ENSMUSG00000056912	0	0	0	0	0	0	0	0	0	0
ENSMUSG00000092227	0	0	0	0	0	0	0	0	0	0
ENSMUSG00000023964	18	3	7	3	4	3	2	6	0	0
ENSMUSG00000096676	0	0	0	0	0	0	0	0	0	0
ENSMUSG00000096677	0	0	0	0	0	0	0	0	0	0
ENSMUSG00000096673	0	0	0	0	0	0	0	0	0	0
ENSMUSG00000105777	0	0	0	0	0	0	0	0	0	0
ENSMUSG00000080689	0	0	0	0	0	0	0	0	0	0
ENSMUSG00000023968	7	13	9	20	25	43	47	14	15	4
ENSMUSG00000096679	0	0	0	0	0	0	0	0	0	0
ENSMUSG00000060404	2	3	3	0	2	0	2	3	2	0
ENSMUSG00000080222	0	0	0	0	0	0	5	0	0	0
ENSMUSG00000080223	0	0	0	0	5	8	14	9	7	8
ENSMUSG00000080227	0	1	2	0	1	2	0	0	0	3
ENSMUSG00000107301	0	0	0	0	0	0	0	0	0	0
ENSMUSG00000100876	104	133	146	130	111	188	176	169	191	153
ENSMUSG00000087224	0	0	0	0	1	0	0	0	0	0
ENSMUSG00000082614	0	0	0	0	0	0	0	0	0	0
ENSMUSG00000105775	12	8	11	6	14	4	6	6	5	5
ENSMUSG00000082613	0	0	0	0	0	0	0	0	0	0
ENSMUSG00000090050	0	0	0	0	0	0	0	0	0	0
ENSMUSG00000082612	0	0	0	0	0	0	0	0	0	0
ENSMUSG00000113832	0	0	0	0	0	0	0	0	0	0
ENSMUSG00000113833	0	0	0	0	0	0	3	0	0	0
ENSMUSG00000113830	2	0	0	0	0	0	0	0	1	1
ENSMUSG00000100877	0	0	0	0	0	0	0	0	0	0
ENSMUSG00000082611	0	0	0	0	0	0	0	0	0	0
ENSMUSG00000113834	0	0	0	0	0	0	0	0	0	0
ENSMUSG00000113835	0	0	0	0	0	0	0	0	0	0
ENSMUSG00000113838	0	0	0	0	0	0	0	0	0	0
ENSMUSG00000113839	0	0	0	0	0	0	0	0	0	0
ENSMUSG00000080592	0	0	0	0	0	0	0	0	0	0
ENSMUSG00000080590	0	0	0	0	0	0	0	0	0	0
ENSMUSG00000080596	0	0	0	0	0	0	0	0	0	0
ENSMUSG00000080597	0	0	0	0	0	0	1	1	0	0
ENSMUSG00000080594	0	0	0	0	0	0	0	0	0	0
ENSMUSG00000080595	0	0	0	0	0	0	0	0	0	0
ENSMUSG00000059493	94	120	99	99	57	130	242	165	201	159
ENSMUSG00000095371	0	0	0	0	0	0	0	0	0	0
ENSMUSG00000095372	0	0	0	0	0	0	0	0	0	0
ENSMUSG00000095374	0	0	0	0	0	0	0	0	0	0
ENSMUSG00000082619	0	0	0	4	0	0	0	0	0	0
ENSMUSG00000111069	1	3	6	0	3	3	0	0	0	0
ENSMUSG00000111068	0	0	0	0	0	0	0	0	0	0
ENSMUSG00000111066	0	0	0	0	0	0	0	3	0	0
ENSMUSG00000111064	0	0	0	0	0	0	0	0	0	0
ENSMUSG00000111062	0	0	0	0	0	0	0	0	3	0
ENSMUSG00000111061	0	0	6	0	0	0	0	0	0	0
ENSMUSG00000111060	0	0	0	0	0	0	0	0	0	0
ENSMUSG00000101512	0	0	0	0	0	0	0	0	0	0
ENSMUSG00000101513	0	0	0	0	0	0	0	0	0	0
ENSMUSG00000081175	0	0	0	0	0	0	0	0	0	0
ENSMUSG00000098225	3	0	0	0	0	0	0	3	3	0
ENSMUSG00000104567	0	0	0	0	0	0	0	0	0	0
ENSMUSG00000108020	0	0	0	0	0	0	0	0	0	0

ENSMUSG00000108544	0	0	0	0	0	0	0	0	0	0
ENSMUSG00000098226	0	0	0	0	0	0	0	0	0	0
ENSMUSG00000101514	0	0	0	0	0	0	0	0	0	0
ENSMUSG00000104563	4	1	1	1	1	1	5	1	2	2
ENSMUSG00000098223	0	0	0	0	0	6	0	9	0	0
ENSMUSG00000037771	0	0	0	0	0	0	0	0	0	0
ENSMUSG00000098222	5	3	5	2	8	3	2	5	12	3
ENSMUSG00000101518	0	0	0	0	0	0	0	0	0	0
ENSMUSG00000108545	0	0	0	0	0	0	0	0	0	0
ENSMUSG00000095594	0	0	0	0	0	0	0	0	0	0
ENSMUSG00000065074	0	0	0	0	0	0	0	0	0	0
ENSMUSG00000095591	0	0	0	0	0	0	0	0	0	0
ENSMUSG00000103629	0	0	0	0	0	0	0	0	0	0
ENSMUSG00000081037	0	0	0	0	0	0	0	0	0	0
ENSMUSG00000095599	0	0	0	0	0	0	0	0	0	0
ENSMUSG00000077059	0	2	0	0	0	0	0	0	0	0
ENSMUSG00000077058	0	0	0	0	0	0	0	0	0	0
ENSMUSG00000053980	3	0	0	5	6	15	18	9	5	6
ENSMUSG00000053985	415	292	354	274	366	253	346	335	339	354
ENSMUSG00000082073	0	0	0	0	0	0	1	0	0	0
ENSMUSG00000094429	0	0	0	0	0	0	0	0	0	0
ENSMUSG00000094428	0	0	0	0	0	0	0	0	0	0
ENSMUSG00000096766	0	0	0	0	0	0	0	0	0	0
ENSMUSG00000094422	0	0	0	0	0	0	0	0	0	0
ENSMUSG00000056004	31	20	32	58	25	33	29	57	39	30
ENSMUSG00000088767	0	0	0	0	0	0	0	0	0	0
ENSMUSG00000056008	0	0	0	0	0	0	0	3	0	3
ENSMUSG00000113278	82	42	56	69	63	49	37	39	26	59
ENSMUSG00000088766	0	0	0	0	0	0	0	0	0	0
ENSMUSG00000052581	7	0	3	0	0	3	0	0	3	0
ENSMUSG00000093804	0	0	0	0	0	0	0	0	0	0
ENSMUSG00000111152	0	0	0	0	0	0	0	0	0	0
ENSMUSG00000093806	0	2	0	0	0	0	0	0	0	0
ENSMUSG00000097991	0	2	0	0	6	4	3	3	10	1
ENSMUSG00000055679	0	0	0	0	0	0	0	0	0	0
ENSMUSG00000112001	0	1	0	0	0	2	0	0	0	0
ENSMUSG00000112002	0	0	0	0	0	0	0	0	0	0
ENSMUSG00000042184	0	0	0	0	0	0	0	3	3	0
ENSMUSG00000112005	0	0	0	0	0	0	0	0	0	0
ENSMUSG00000111156	2	0	0	0	0	0	0	0	0	0
ENSMUSG00000113270	0	0	0	0	0	0	0	0	0	0
ENSMUSG00000093802	0	0	0	0	0	0	0	0	0	0
ENSMUSG00000106358	0	0	0	0	0	0	0	0	0	0
ENSMUSG00000111154	0	0	0	0	0	0	0	0	0	0
ENSMUSG00000054988	0	0	0	0	0	0	9	0	0	0
ENSMUSG00000093501	0	0	0	0	0	0	0	0	0	0
ENSMUSG00000054986	0	0	0	0	0	18	0	0	0	0
ENSMUSG00000094736	0	0	0	0	0	0	0	0	0	0
ENSMUSG00000051802	0	0	0	0	0	0	0	0	0	0
ENSMUSG00000086339	0	0	0	0	0	0	0	0	0	0
ENSMUSG00000093292	0	0	0	0	0	0	0	0	0	0
ENSMUSG00000051804	0	0	0	0	0	0	0	0	0	0
ENSMUSG00000086335	0	0	0	4	0	0	0	0	0	0
ENSMUSG00000086336	1	0	0	2	0	0	0	0	0	0
ENSMUSG00000086337	0	0	0	0	0	0	0	0	0	0
ENSMUSG00000076010	0	0	0	0	0	0	0	2	0	0
ENSMUSG00000076014	0	0	2	0	0	0	0	0	0	0
ENSMUSG00000113356	0	0	0	0	0	0	0	0	0	0

ENSMUSG00000113354	0	0	0	0	0	0	0	0	0	3
ENSMUSG00000113352	0	0	0	0	3	0	3	0	0	0
ENSMUSG00000113351	0	0	0	0	0	0	0	0	0	0
ENSMUSG00000113350	0	0	0	0	0	0	0	0	0	0
ENSMUSG00000113359	0	0	0	0	0	0	0	1	0	0
ENSMUSG00000113358	0	0	0	0	0	0	0	0	0	0
ENSMUSG00000049098	0	0	0	0	0	0	0	0	0	0
ENSMUSG00000049094	0	0	0	0	0	0	0	0	0	0
ENSMUSG00000049097	67	79	91	96	79	76	62	121	100	94
ENSMUSG00000049093	61	17	58	23	29	33	84	27	42	89
ENSMUSG00000082528	0	3	0	0	0	0	0	0	0	0
ENSMUSG00000082523	0	0	0	0	0	0	0	0	0	0
ENSMUSG00000082524	0	0	0	0	0	0	0	0	0	0
ENSMUSG00000040121	47	48	42	55	50	69	78	57	77	78
ENSMUSG00000040127	0	0	0	0	0	0	0	0	0	0
ENSMUSG00000062170	27	19	28	26	12	21	109	39	56	24
ENSMUSG00000077597	0	0	0	0	0	0	0	0	0	0
ENSMUSG00000048697	0	0	0	0	0	0	0	0	0	0
ENSMUSG00000084206	0	0	0	0	0	0	0	0	0	0
ENSMUSG00000084204	0	0	0	0	0	0	0	0	0	0
ENSMUSG00000084205	0	0	0	0	0	0	0	0	0	0
ENSMUSG00000084202	0	0	0	0	0	0	0	0	0	0
ENSMUSG00000084203	2	0	0	0	2	0	0	2	0	3
ENSMUSG00000084200	0	0	0	0	0	0	0	0	0	0
ENSMUSG00000075543	0	0	0	0	0	0	0	0	0	0
ENSMUSG00000084208	0	0	0	0	0	0	0	0	0	0
ENSMUSG00000084209	0	0	0	0	0	0	0	0	0	0
ENSMUSG00000041293	3	0	0	0	0	0	0	0	0	0
ENSMUSG00000105807	0	0	0	0	0	0	0	0	0	0
ENSMUSG00000081553	0	0	6	0	0	0	2	0	3	0
ENSMUSG00000105800	0	0	0	0	0	0	0	0	0	0
ENSMUSG00000103531	0	0	0	0	0	0	0	0	0	0
ENSMUSG00000080962	0	0	0	2	0	3	0	0	0	2
ENSMUSG00000080961	0	0	0	0	0	0	0	0	0	0
ENSMUSG00000080960	0	0	0	0	0	0	0	0	0	0
ENSMUSG00000080965	0	0	0	0	0	0	0	0	0	0
ENSMUSG00000080964	0	0	0	0	0	0	0	0	0	0
ENSMUSG00000105208	0	0	0	0	0	0	0	0	0	0
ENSMUSG00000078640	61	90	43	66	49	90	169	134	74	68
ENSMUSG00000103538	0	0	3	0	0	0	0	0	0	0
ENSMUSG00000065719	0	0	0	0	0	0	0	0	0	0
ENSMUSG00000065718	0	0	0	0	0	0	0	0	0	0
ENSMUSG00000068547	0	0	0	0	0	0	9	0	0	0
ENSMUSG00000070111	0	0	0	0	0	0	3	0	6	3
ENSMUSG00000099128	0	0	0	0	0	0	0	0	0	0
ENSMUSG00000099120	0	0	0	0	0	0	0	0	0	0
ENSMUSG00000099124	0	0	0	0	0	0	0	0	0	0
ENSMUSG00000099126	0	3	0	0	0	0	0	2	0	0
ENSMUSG00000032343	5	0	6	3	6	6	0	3	3	7
ENSMUSG00000001496	0	0	0	0	0	5	0	0	0	0
ENSMUSG00000001497	7	13	14	6	9	12	18	9	3	3
ENSMUSG00000068099	0	6	0	0	3	0	3	0	0	0
ENSMUSG00000089105	0	0	0	0	0	0	0	0	0	0
ENSMUSG00000054450	0	2	0	4	0	6	0	0	0	0
ENSMUSG00000006724	14	13	13	3	2	4	22	7	6	4
ENSMUSG00000006720	18	33	87	29	17	28	85	43	58	86
ENSMUSG00000054951	180	164	191	163	104	186	229	241	243	189
ENSMUSG00000035187	3	0	0	0	0	3	3	0	0	6

ENSMUSG00000112649	0	0	0	0	0	0	0	0	0	0
ENSMUSG00000073494	0	9	8	7	0	0	2	3	4	5
ENSMUSG00000073497	0	0	0	0	0	0	0	0	0	0
ENSMUSG00000029630	0	0	4	0	0	0	0	0	0	0
ENSMUSG00000108982	0	0	0	0	0	0	0	0	0	0
ENSMUSG00000024207	0	0	0	0	0	0	0	0	0	0
ENSMUSG00000093681	0	0	0	0	0	0	0	0	0	0
ENSMUSG00000104798	0	0	0	0	0	0	0	0	0	0
ENSMUSG00000111268	0	0	0	0	0	0	0	0	0	0
ENSMUSG00000103833	0	0	0	0	0	0	0	0	0	0
ENSMUSG00000103832	3	0	0	0	0	0	0	0	0	0
ENSMUSG00000103831	0	0	0	0	0	0	0	0	0	0
ENSMUSG00000103837	3	1	0	0	2	3	1	3	3	8
ENSMUSG00000103836	0	0	0	0	0	0	0	0	0	0
ENSMUSG00000103839	91	66	53	43	52	63	81	81	71	50
ENSMUSG00000022439	139	110	131	118	110	120	251	116	194	85
ENSMUSG00000019756	0	0	0	0	0	0	0	0	0	0
ENSMUSG00000070138	1	3	1	0	0	1	3	2	5	2
ENSMUSG00000063188	0	0	0	0	0	0	0	0	0	0
ENSMUSG00000089952	12	0	1	5	2	6	9	15	8	0
ENSMUSG00000089957	0	0	0	0	0	0	0	0	0	0
ENSMUSG00000089956	0	0	0	0	0	0	0	0	0	0
ENSMUSG00000109822	3	0	0	2	4	0	2	0	0	0
ENSMUSG00000076977	0	0	0	0	0	0	0	0	0	0
ENSMUSG00000076976	0	0	0	0	0	0	0	0	0	0
ENSMUSG00000108921	0	0	0	0	0	0	0	0	0	0
ENSMUSG00000108922	0	0	0	0	0	0	0	0	0	0
ENSMUSG00000108924	0	0	0	0	0	0	0	0	0	0
ENSMUSG00000108926	11	1	10	5	3	3	1	15	8	11
ENSMUSG00000108928	0	0	0	0	0	0	0	0	0	0
ENSMUSG00000108929	0	5	7	43	23	0	11	56	2	27
ENSMUSG00000034959	139	156	179	215	136	197	325	200	327	161
ENSMUSG00000105869	0	0	0	0	0	0	0	0	0	0
ENSMUSG00000099667	0	0	0	0	0	0	0	0	0	0
ENSMUSG00000043333	0	9	5	0	0	20	40	35	45	15
ENSMUSG00000067604	0	0	0	0	0	0	0	0	0	0
ENSMUSG00000043331	0	0	0	0	0	0	0	0	0	0
ENSMUSG00000099664	0	0	0	0	0	0	0	0	0	0
ENSMUSG00000030257	127	91	132	96	120	159	441	239	309	211
ENSMUSG00000030254	225	173	220	224	182	231	422	318	385	316
ENSMUSG00000109826	0	0	0	0	0	0	0	0	0	0
ENSMUSG00000099660	0	0	0	0	0	0	0	0	0	0
ENSMUSG00000111260	0	0	0	0	0	0	0	0	0	0
ENSMUSG00000109700	0	9	3	8	6	8	3	0	9	0
ENSMUSG00000036855	0	0	0	0	0	3	5	0	3	0
ENSMUSG00000101528	0	0	0	0	0	0	0	0	0	0
ENSMUSG00000101522	0	0	0	0	0	0	0	0	0	0
ENSMUSG00000101521	0	0	0	0	0	0	0	0	0	0
ENSMUSG00000101520	0	0	0	0	0	0	0	0	0	0
ENSMUSG00000101527	0	0	0	0	0	0	0	0	0	0
ENSMUSG00000060371	0	0	0	0	0	3	3	0	0	0
ENSMUSG00000101525	0	0	0	0	0	0	0	0	0	0
ENSMUSG00000085925	1	0	0	13	1	0	30	27	0	22
ENSMUSG00000085926	0	0	0	0	0	0	0	0	0	0
ENSMUSG00000085921	0	0	0	0	0	0	0	0	0	0
ENSMUSG00000085922	2	0	0	6	0	0	0	0	0	0
ENSMUSG00000085928	0	0	0	0	0	0	0	0	0	3
ENSMUSG00000085929	12	13	6	13	9	6	22	23	17	12

ENSMUSG00000110614	0	0	0	0	9	9	0	0	0	3
ENSMUSG00000087278	0	0	0	0	0	0	0	0	0	0
ENSMUSG00000087279	0	0	3	3	0	0	0	0	3	5
ENSMUSG00000087276	0	0	0	0	0	0	0	0	0	0
ENSMUSG00000087277	9	13	13	6	6	3	12	10	10	17
ENSMUSG00000087273	0	0	0	0	0	0	0	0	3	0
ENSMUSG00000037705	0	5	0	3	9	3	3	6	0	0
ENSMUSG00000037708	28	26	25	37	12	27	30	30	25	23
ENSMUSG00000021953	3	0	0	0	0	0	0	3	0	0
ENSMUSG00000033633	85	67	91	92	74	59	20	33	35	67
ENSMUSG00000095072	0	0	0	0	0	0	0	0	0	0
ENSMUSG00000103015	0	6	0	0	3	6	0	0	5	3
ENSMUSG00000103017	0	0	0	0	0	0	0	0	0	0
ENSMUSG00000103010	0	0	0	0	0	0	0	0	0	0
ENSMUSG00000103012	0	0	0	0	0	0	0	0	0	0
ENSMUSG00000112777	0	0	0	0	0	0	0	0	0	0
ENSMUSG00000062224	0	0	0	0	0	0	0	0	0	0
ENSMUSG00000059964	0	0	0	0	0	0	0	1	0	0
ENSMUSG00000114425	0	0	0	0	0	0	0	0	0	0
ENSMUSG00000114428	0	0	0	0	0	0	6	0	0	0
ENSMUSG00000100947	0	0	0	0	0	0	0	0	0	0
ENSMUSG00000089138	0	0	0	0	0	0	0	0	0	0
ENSMUSG00000100940	0	0	0	0	0	0	0	0	0	0
ENSMUSG00000089132	0	0	0	0	0	0	0	0	0	0
ENSMUSG00000089130	0	0	0	0	0	0	0	0	0	0
ENSMUSG00000089137	0	0	0	0	0	0	0	0	0	0
ENSMUSG00000089136	0	0	0	0	0	0	0	0	0	0
ENSMUSG00000089134	0	0	0	0	0	0	0	0	0	0
ENSMUSG00000018634	0	0	0	0	0	0	0	0	0	0
ENSMUSG00000020335	154	184	224	181	153	178	177	200	162	230
ENSMUSG00000028148	0	0	0	3	0	0	0	0	0	0
ENSMUSG00000022798	0	0	0	0	0	0	0	0	0	0
ENSMUSG00000022790	97	57	73	77	52	113	72	67	80	48
ENSMUSG00000112246	0	0	0	0	0	0	6	2	6	0
ENSMUSG00000102765	0	0	0	0	0	3	186	8	32	13
ENSMUSG00000112244	0	0	0	0	0	0	0	0	0	0
ENSMUSG00000029819	21	36	18	9	27	27	58	3	24	18
ENSMUSG00000102761	0	0	0	0	0	0	3	0	0	0
ENSMUSG00000112240	0	0	0	0	0	0	0	0	0	0
ENSMUSG00000102763	0	0	0	0	0	0	0	0	0	0
ENSMUSG00000029813	7	7	7	0	9	3	9	0	3	12
ENSMUSG00000029811	0	0	0	0	0	3	3	6	0	0
ENSMUSG00000102768	0	0	0	0	0	0	0	0	0	0
ENSMUSG00000102769	0	0	0	0	0	0	0	0	0	0
ENSMUSG00000112248	0	0	0	0	0	0	0	0	0	0
ENSMUSG00000112249	86	110	80	99	90	134	166	112	122	65
ENSMUSG00000062444	3	0	0	8	6	0	8	0	3	3
ENSMUSG00000024795	270	207	266	361	241	204	454	203	270	214
ENSMUSG00000060816	0	0	0	0	0	3	0	0	0	0
ENSMUSG00000024798	104	104	104	78	77	121	110	141	61	92
ENSMUSG00000028492	5	0	0	0	10	9	6	11	0	6
ENSMUSG00000112210	0	0	0	0	0	0	0	0	0	0
ENSMUSG00000039391	0	0	0	0	3	0	0	0	0	0
ENSMUSG00000102735	3	0	0	0	0	0	0	4	0	0
ENSMUSG00000039396	72	70	106	69	50	24	47	35	14	49
ENSMUSG00000039032	5	0	3	0	0	0	0	4	0	4
ENSMUSG00000105411	0	0	0	0	0	0	0	0	0	0
ENSMUSG00000105410	0	0	0	0	0	0	0	0	0	0

ENSMUSG00000105416	0	0	0	4	0	0	0	0	3	0
ENSMUSG00000105419	65	65	82	64	62	115	137	90	79	110
ENSMUSG00000105418	0	0	0	0	0	0	0	0	0	0
ENSMUSG00000110089	0	2	0	0	0	0	3	0	0	0
ENSMUSG00000110082	0	0	0	0	0	0	0	0	0	0
ENSMUSG00000110080	3	0	0	0	0	0	3	0	3	0
ENSMUSG00000110081	0	0	0	0	0	0	0	0	0	0
ENSMUSG00000113194	0	0	0	0	0	0	0	0	0	0
ENSMUSG00000113193	0	0	0	0	0	0	2	0	4	0
ENSMUSG00000113192	0	0	0	0	0	0	0	0	0	0
ENSMUSG00000113199	0	0	0	0	0	0	0	0	0	0
ENSMUSG00000110809	0	0	0	0	0	0	0	0	0	0
ENSMUSG00000021175	54	36	12	54	25	30	114	45	68	43
ENSMUSG00000029369	0	0	0	0	0	0	2	0	3	0
ENSMUSG00000029361	36	30	54	24	15	27	35	23	33	38
ENSMUSG00000051758	0	0	0	0	0	0	0	0	0	0
ENSMUSG00000114358	0	0	0	0	0	0	0	0	0	0
ENSMUSG00000114356	0	0	0	0	0	0	0	0	0	0
ENSMUSG00000058052	0	0	0	0	0	0	0	0	0	0
ENSMUSG00000114352	0	0	0	0	0	0	0	0	0	0
ENSMUSG00000114353	0	0	0	0	0	0	0	0	0	0
ENSMUSG00000114350	0	0	0	0	0	0	0	0	0	0
ENSMUSG00000114351	0	0	0	0	0	0	0	0	0	0
ENSMUSG00000106853	0	0	4	0	0	0	0	0	0	0
ENSMUSG00000105271	0	0	0	0	0	0	0	0	0	0
ENSMUSG00000108262	0	0	0	0	0	0	0	0	0	0
ENSMUSG00000108261	0	0	0	0	0	0	0	0	0	0
ENSMUSG00000108265	0	0	0	0	0	0	0	0	0	0
ENSMUSG00000108264	0	0	0	0	0	0	0	0	0	0
ENSMUSG00000059106	0	0	0	0	0	0	0	0	0	0
ENSMUSG00000059105	0	0	0	0	0	0	0	0	0	0
ENSMUSG00000110018	0	3	0	0	2	8	8	3	11	0
ENSMUSG00000105279	0	0	0	0	0	0	0	0	0	0
ENSMUSG00000020491	348	215	262	239	212	196	313	318	327	253
ENSMUSG00000025468	3	0	4	12	6	0	0	6	3	5
ENSMUSG00000025461	156	129	162	85	85	158	288	240	136	247
ENSMUSG00000097714	14	25	15	44	18	109	58	53	15	54
ENSMUSG00000097717	0	4	2	0	3	0	2	0	0	0
ENSMUSG00000097710	0	0	0	0	0	0	0	0	0	0
ENSMUSG00000097711	8	5	11	12	12	8	3	2	8	6
ENSMUSG00000097712	29	23	18	4	10	9	28	30	10	6
ENSMUSG00000097713	5	0	19	4	0	4	3	5	8	6
ENSMUSG00000097719	0	0	0	0	0	0	0	0	0	0
ENSMUSG00000108487	0	0	0	0	0	0	0	0	0	0
ENSMUSG00000108485	0	0	0	0	0	0	0	0	0	0
ENSMUSG00000104455	0	0	0	0	0	0	0	0	0	0
ENSMUSG00000108483	0	2	0	0	0	0	0	0	0	0
ENSMUSG00000108482	0	0	0	0	0	0	0	0	0	0
ENSMUSG00000108481	0	0	0	0	0	0	0	0	0	0
ENSMUSG00000108480	0	0	0	0	0	0	0	0	0	0
ENSMUSG00000091712	102	96	85	98	47	84	68	109	69	63
ENSMUSG00000091713	2	0	0	0	0	0	0	0	0	0
ENSMUSG00000108488	0	0	0	0	0	0	0	0	0	0
ENSMUSG00000083940	0	0	0	0	0	0	0	0	0	0
ENSMUSG00000083941	0	0	0	0	0	0	0	0	0	0
ENSMUSG00000083942	0	0	0	0	0	0	0	0	0	0
ENSMUSG00000083944	0	0	0	0	0	0	0	0	0	0
ENSMUSG00000083945	0	0	0	0	0	0	0	0	0	0

ENSMUSG00000083946	0	0	0	0	0	0	0	0	0	0
ENSMUSG00000083947	0	0	0	0	0	0	0	0	0	0
ENSMUSG00000083948	0	0	0	0	0	0	0	0	0	0
ENSMUSG00000083949	0	0	0	0	0	0	0	0	0	0
ENSMUSG00000106426	0	0	3	0	0	0	0	0	9	0
ENSMUSG00000010066	370	390	210	178	194	343	724	293	138	158
ENSMUSG00000044359	0	0	0	0	0	3	0	0	0	0
ENSMUSG00000044352	26	17	18	29	12	38	9	27	35	33
ENSMUSG00000054702	2	14	17	12	20	5	28	18	10	0
ENSMUSG00000056706	0	0	0	0	0	0	0	0	0	0
ENSMUSG00000046262	0	0	0	0	0	0	0	0	0	0
ENSMUSG00000045083	0	0	0	3	0	0	0	3	0	0
ENSMUSG00000040726	0	6	11	5	6	12	15	7	12	0
ENSMUSG00000044681	0	0	0	0	0	0	0	0	0	3
ENSMUSG00000110507	0	3	0	0	0	0	0	0	0	0
ENSMUSG00000110506	0	0	0	0	0	0	0	0	0	0
ENSMUSG00000104451	0	2	0	0	0	0	0	0	0	0
ENSMUSG00000110503	0	0	0	0	0	0	0	0	0	0
ENSMUSG00000110502	0	0	0	0	0	0	0	0	0	0
ENSMUSG00000044689	3	0	6	0	6	6	58	14	24	9
ENSMUSG00000023433	0	0	4	0	0	0	0	0	0	0
ENSMUSG00000080419	0	0	0	0	0	0	0	0	0	0
ENSMUSG00000080415	0	0	0	0	0	0	1	0	0	0
ENSMUSG00000080413	1	1	1	1	1	1	1	1	1	1
ENSMUSG00000080412	0	0	0	0	0	0	0	0	0	0
ENSMUSG00000080411	0	2	0	0	2	0	4	0	0	3
ENSMUSG00000080410	0	0	0	0	0	0	0	0	0	0
ENSMUSG00000074928	0	0	0	0	0	0	0	0	0	0
ENSMUSG00000113974	0	0	0	0	0	0	6	0	0	3
ENSMUSG00000113973	0	0	0	0	0	0	0	0	0	0
ENSMUSG00000113972	0	0	0	0	0	0	0	0	0	0
ENSMUSG00000113971	0	0	0	0	0	0	0	0	0	0
ENSMUSG00000078972	0	0	0	0	0	0	0	0	0	0
ENSMUSG00000053287	0	0	0	0	0	0	0	0	0	0
ENSMUSG00000113979	0	0	0	0	0	0	0	0	0	0
ENSMUSG00000106428	0	0	0	0	0	0	0	0	0	0
ENSMUSG00000086810	3	38	39	21	18	0	9	18	27	3
ENSMUSG00000086817	0	0	0	0	0	0	0	0	6	2
ENSMUSG00000037469	0	0	0	0	0	0	0	0	0	0
ENSMUSG00000090396	0	0	0	0	0	0	0	0	0	0
ENSMUSG00000045559	0	0	0	0	0	0	0	0	0	0
ENSMUSG00000098541	0	0	0	0	0	0	0	0	0	0
ENSMUSG00000028804	9	3	0	6	0	0	0	6	9	0
ENSMUSG00000053868	0	0	0	0	0	0	0	0	0	0
ENSMUSG00000053863	0	0	0	0	0	0	0	0	0	0
ENSMUSG00000094678	0	3	0	0	0	0	0	0	0	0
ENSMUSG00000094679	0	0	0	0	0	0	0	0	0	0
ENSMUSG00000094670	0	0	0	0	0	0	0	0	0	0
ENSMUSG00000101788	0	0	0	0	0	0	0	0	0	0
ENSMUSG00000098741	0	0	0	0	0	0	1	0	0	2
ENSMUSG00000106622	0	0	0	0	0	0	0	0	0	0
ENSMUSG00000081458	0	0	0	0	0	0	0	0	0	0
ENSMUSG00000090975	0	0	0	0	0	0	0	0	0	0
ENSMUSG00000081450	0	0	0	0	0	0	0	0	0	0
ENSMUSG00000081452	0	0	0	1	0	2	0	0	3	0
ENSMUSG00000081455	19	17	12	31	15	16	18	8	23	10
ENSMUSG00000099709	0	0	0	0	0	0	0	0	0	0
ENSMUSG00000092538	0	0	0	0	0	0	0	0	0	0

ENSMUSG00000099701	0	0	0	0	0	0	0	0	0	0
ENSMUSG00000092532	0	0	0	0	0	0	0	0	0	0
ENSMUSG00000092530	0	0	0	0	0	0	0	0	0	0
ENSMUSG00000099704	0	0	0	0	0	0	0	0	0	0
ENSMUSG00000099707	0	0	0	0	0	0	0	0	0	0
ENSMUSG00000077572	0	0	0	0	0	0	0	0	0	0
ENSMUSG00000077576	0	0	0	0	0	0	0	0	0	0
ENSMUSG00000077579	0	0	0	0	0	0	0	0	0	0
ENSMUSG00000082228	0	0	0	0	0	0	0	0	0	0
ENSMUSG00000082229	5	12	12	15	3	0	15	9	9	0
ENSMUSG00000030017	0	0	0	0	0	0	0	0	0	0
ENSMUSG00000082222	0	0	0	0	0	0	0	0	0	0
ENSMUSG00000082227	0	0	0	0	0	0	0	0	0	0
ENSMUSG00000082225	0	0	0	0	0	0	0	0	0	0
ENSMUSG00000076190	0	0	0	0	0	0	0	0	0	0
ENSMUSG00000107985	0	0	3	0	0	0	5	6	0	0
ENSMUSG00000076199	0	0	0	0	0	0	0	0	0	0
ENSMUSG00000032528	113	72	140	122	84	208	288	219	143	181
ENSMUSG00000105899	0	0	0	0	0	0	0	0	0	0
ENSMUSG00000106626	0	0	0	0	0	0	0	0	0	0
ENSMUSG00000096480	0	0	0	0	0	0	1	0	0	0
ENSMUSG00000086105	0	0	0	0	0	0	18	3	3	8
ENSMUSG00000086104	0	0	0	0	0	0	0	0	0	0
ENSMUSG00000086103	0	0	0	0	3	0	0	0	0	0
ENSMUSG00000086109	15	0	0	7	0	3	6	0	2	5
ENSMUSG00000072437	177	160	115	141	100	123	195	153	156	117
ENSMUSG00000076222	0	0	0	0	0	0	1	0	0	0
ENSMUSG00000041649	64	90	47	63	77	57	157	80	84	75
ENSMUSG00000082489	0	0	0	0	0	0	0	0	0	0
ENSMUSG00000082486	0	0	0	0	0	0	0	0	0	0
ENSMUSG00000041644	0	0	3	13	0	0	22	2	0	9
ENSMUSG00000082485	0	0	3	0	0	0	0	0	0	0
ENSMUSG00000082482	0	0	0	0	0	0	0	0	0	0
ENSMUSG00000087473	0	0	0	0	0	0	0	0	0	0
ENSMUSG00000107988	0	4	0	0	0	0	0	0	0	3
ENSMUSG00000087649	0	0	0	0	0	0	0	0	0	0
ENSMUSG00000083570	0	0	0	0	0	0	0	0	0	0
ENSMUSG00000083573	0	0	0	0	0	0	0	0	0	0
ENSMUSG00000083572	0	0	0	0	0	0	0	0	0	0
ENSMUSG00000037542	9	3	12	18	9	4	9	0	3	0
ENSMUSG00000037541	21	34	20	14	32	36	55	36	33	14
ENSMUSG00000083576	0	0	0	0	0	0	0	0	0	0
ENSMUSG00000049112	43	59	45	21	24	30	155	73	77	61
ENSMUSG00000100806	0	0	0	0	0	0	0	0	0	0
ENSMUSG00000000394	0	0	0	0	0	0	0	0	0	0
ENSMUSG00000079414	6	12	3	9	8	12	6	16	27	12
ENSMUSG00000031870	9	6	6	0	8	12	9	3	5	3
ENSMUSG00000031872	125	112	138	88	94	138	182	81	152	86
ENSMUSG00000031876	0	0	0	0	0	34	10	0	0	0
ENSMUSG00000080889	0	0	0	0	0	0	0	0	0	0
ENSMUSG00000080880	0	0	0	0	0	0	0	1	0	0
ENSMUSG00000080884	0	0	0	0	0	0	0	0	0	0
ENSMUSG00000080886	0	0	0	0	0	0	0	0	0	0
ENSMUSG00000080887	0	0	0	0	0	0	0	0	0	0
ENSMUSG00000083681	0	0	0	0	0	0	0	0	0	0
ENSMUSG00000083680	0	0	0	0	0	0	0	0	0	0
ENSMUSG00000103079	0	0	0	0	0	0	0	0	0	3
ENSMUSG00000083685	0	0	0	0	0	0	0	0	0	0

ENSMUSG00000083684	0	0	0	0	0	0	0	0	0	0
ENSMUSG00000033730	44	68	72	49	29	54	126	56	75	88
ENSMUSG00000033737	0	3	0	0	9	3	0	0	3	3
ENSMUSG00000061336	0	0	0	0	0	0	0	0	0	0
ENSMUSG00000064675	0	0	0	0	0	0	0	0	0	0
ENSMUSG00000064677	0	0	0	0	0	0	0	0	0	0
ENSMUSG00000064671	0	1	0	0	0	1	0	1	0	0
ENSMUSG00000064670	0	0	0	0	0	0	0	0	0	0
ENSMUSG00000064673	0	0	0	0	0	0	0	0	0	0
ENSMUSG00000107279	0	0	0	0	0	0	0	0	0	0
ENSMUSG00000107278	28	30	38	23	27	18	53	55	47	55
ENSMUSG00000107270	0	0	0	0	0	0	0	0	0	0
ENSMUSG00000107272	0	3	0	0	4	0	3	0	0	2
ENSMUSG00000058030	0	0	0	0	0	0	0	0	0	0
ENSMUSG00000034402	0	0	0	0	0	0	0	0	0	3
ENSMUSG00000061489	0	0	0	0	0	0	0	0	0	3
ENSMUSG00000061486	0	0	0	0	0	2	6	0	3	0
ENSMUSG00000000938	0	0	3	0	3	3	0	0	0	0
ENSMUSG00000065102	0	0	0	0	0	0	0	0	0	0
ENSMUSG00000094319	0	0	0	0	0	0	0	2	0	0
ENSMUSG00000099089	0	0	0	0	0	0	0	0	0	0
ENSMUSG00000099086	0	0	0	0	0	0	0	0	0	0
ENSMUSG00000099087	0	0	0	0	0	0	0	0	0	0
ENSMUSG00000099085	0	0	0	0	0	0	0	0	0	0
ENSMUSG00000071089	0	3	0	0	0	3	0	0	12	3
ENSMUSG00000074261	9	0	9	14	9	3	0	3	15	17
ENSMUSG00000074268	8	0	0	0	0	0	0	0	0	0
ENSMUSG00000030830	190	180	225	386	170	272	286	297	364	300
ENSMUSG00000099808	0	0	0	0	0	0	0	0	0	0
ENSMUSG00000030834	0	4	0	0	0	0	0	0	0	0
ENSMUSG00000099806	0	0	0	0	0	0	0	0	0	0
ENSMUSG00000081869	0	0	0	0	0	0	0	0	0	0
ENSMUSG00000094313	0	0	0	0	0	0	0	0	0	0
ENSMUSG00000099802	0	0	0	0	0	0	0	0	0	0
ENSMUSG00000074486	0	7	4	4	3	4	4	0	3	0
ENSMUSG00000074483	0	0	0	0	0	0	5	0	0	0
ENSMUSG00000074480	33	55	55	44	61	48	86	35	45	43
ENSMUSG00000009248	3	6	4	9	6	12	10	5	16	6
ENSMUSG00000111153	0	0	0	0	0	0	0	0	0	0
ENSMUSG00000006574	69	56	75	122	269	54	45	106	33	21
ENSMUSG00000006570	0	0	3	0	0	0	0	0	0	0
ENSMUSG00000102358	0	0	0	0	0	0	0	0	0	0
ENSMUSG00000102355	0	0	0	0	0	0	0	0	0	0
ENSMUSG00000102354	0	0	0	0	0	0	0	0	0	0
ENSMUSG00000102350	0	0	0	0	0	0	0	0	0	0
ENSMUSG00000102353	13	22	6	14	14	11	14	17	24	14
ENSMUSG00000072884	6	0	3	11	10	7	10	12	7	0
ENSMUSG00000072888	0	0	0	0	0	0	0	0	0	0
ENSMUSG00000098027	0	0	0	0	0	0	0	0	0	0
ENSMUSG00000098025	0	0	0	0	0	0	0	0	0	0
ENSMUSG00000098023	0	0	0	0	0	0	0	0	0	0
ENSMUSG00000098022	296	235	239	299	232	198	162	244	257	61
ENSMUSG00000077903	0	0	0	0	0	0	0	0	0	0
ENSMUSG00000077902	0	0	0	0	0	0	0	0	0	0
ENSMUSG00000098029	0	0	0	0	0	0	0	0	0	0
ENSMUSG00000098028	0	0	2	2	3	0	2	2	3	0
ENSMUSG00000069755	356	410	399	416	341	342	565	437	353	448
ENSMUSG00000035923	0	0	0	0	0	0	0	0	3	0

ENSMUSG00000096029	2	0	0	0	0	0	0	0	0	0
ENSMUSG00000096028	0	0	0	0	0	0	0	0	0	0
ENSMUSG00000096024	0	0	0	0	0	0	0	0	0	0
ENSMUSG00000096027	0	0	0	0	0	0	0	0	0	0
ENSMUSG00000096021	0	0	0	2	0	0	0	0	0	0
ENSMUSG00000096023	0	0	0	0	0	0	0	0	0	0
ENSMUSG00000096022	2	1	0	3	0	1	1	1	0	3
ENSMUSG00000034031	0	0	0	0	0	1	0	2	0	0
ENSMUSG00000063225	0	0	0	0	0	0	0	0	0	0
ENSMUSG00000034039	0	0	0	0	0	0	0	0	0	0
ENSMUSG00000105284	3	0	3	0	0	0	0	0	0	0
ENSMUSG00000101492	0	0	0	0	0	0	0	0	0	0
ENSMUSG00000066767	0	0	0	0	0	0	0	0	0	0
ENSMUSG00000066760	0	0	0	3	0	0	0	3	0	0
ENSMUSG00000105283	8	3	8	14	5	8	11	14	13	18
ENSMUSG00000104219	0	0	0	0	0	0	0	0	0	0
ENSMUSG00000109701	0	0	0	0	0	0	2	2	0	0
ENSMUSG00000104211	0	9	0	5	0	0	6	0	0	3
ENSMUSG00000104216	0	0	0	0	0	0	0	0	0	0
ENSMUSG00000104215	0	0	0	0	0	0	0	0	0	0
ENSMUSG00000101196	0	0	0	0	0	0	0	0	0	0
ENSMUSG00000106889	0	0	0	0	0	0	0	0	0	0
ENSMUSG00000101194	0	0	0	0	0	0	0	0	0	0
ENSMUSG00000101195	0	0	0	0	0	0	0	0	0	0
ENSMUSG00000101192	0	0	0	0	0	0	0	0	0	0
ENSMUSG00000101193	0	0	0	0	0	0	0	0	0	0
ENSMUSG00000101190	0	0	0	0	0	0	0	0	0	0
ENSMUSG00000089486	0	0	0	0	0	2	0	0	0	0
ENSMUSG00000091556	0	0	0	0	3	0	0	0	0	0
ENSMUSG00000091557	0	0	0	0	0	0	0	0	0	0
ENSMUSG00000091550	0	0	0	0	0	3	2	3	2	0
ENSMUSG00000101198	0	0	0	0	0	0	0	0	0	0
ENSMUSG00000091553	0	0	0	0	0	0	0	0	0	0
ENSMUSG00000081358	0	0	0	0	0	0	0	0	0	0
ENSMUSG00000030351	95	207	73	95	72	134	127	66	108	177
ENSMUSG00000020961	714	811	805	904	596	802	889	980	819	1243
ENSMUSG00000107536	0	0	0	0	0	0	0	0	0	0
ENSMUSG00000020963	6	20	9	0	6	35	428	52	125	45
ENSMUSG00000064992	0	0	0	0	0	0	0	0	0	0
ENSMUSG00000049018	0	0	0	0	0	0	0	0	0	0
ENSMUSG00000056544	0	0	0	0	0	0	0	0	0	0
ENSMUSG00000089489	0	0	0	0	0	0	0	0	0	0
ENSMUSG00000091080	0	0	0	0	0	0	0	0	0	0
ENSMUSG00000104964	0	0	7	6	6	0	0	9	6	0
ENSMUSG00000108251	0	0	0	0	0	0	0	0	0	0
ENSMUSG00000073947	0	0	0	0	0	0	0	0	0	0
ENSMUSG00000100245	0	0	0	0	0	0	0	0	0	0
ENSMUSG00000100244	0	0	0	0	0	0	0	0	0	0
ENSMUSG00000100247	0	3	0	0	0	0	0	0	0	0
ENSMUSG00000088182	0	0	0	0	0	0	0	0	0	2
ENSMUSG00000100241	0	0	0	0	0	0	0	0	0	0
ENSMUSG00000100240	0	0	0	0	0	0	0	0	0	0
ENSMUSG00000100243	0	0	0	0	0	0	0	0	0	0
ENSMUSG00000029875	52	45	58	48	39	63	63	36	48	24
ENSMUSG00000100249	0	0	0	0	0	0	0	0	0	0
ENSMUSG00000100248	0	0	0	0	0	0	0	0	0	0
ENSMUSG00000058244	0	0	0	0	0	0	0	0	0	0
ENSMUSG00000058248	38	47	87	33	55	84	122	62	94	71

ENSMUSG00000064333	0	0	0	0	0	0	0	0	0	0
ENSMUSG00000095705	0	0	0	0	0	0	0	0	0	0
ENSMUSG00000095704	3	5	0	4	0	0	0	1	2	2
ENSMUSG00000095706	0	0	0	0	0	0	0	0	0	0
ENSMUSG00000095703	0	0	0	0	0	0	0	0	0	0
ENSMUSG00000095702	0	0	0	0	0	0	0	0	0	0
ENSMUSG00000088459	0	0	0	0	0	0	1	1	2	0
ENSMUSG00000088458	0	0	0	0	0	0	0	0	0	0
ENSMUSG00000088450	0	0	0	0	0	0	0	0	0	0
ENSMUSG00000022613	12	0	38	127	3	3	22	0	0	7
ENSMUSG00000094074	0	0	0	0	0	0	0	0	0	0
ENSMUSG00000107734	0	0	0	3	0	0	0	0	2	2
ENSMUSG00000107735	0	0	0	0	0	0	0	0	0	0
ENSMUSG00000107737	0	0	0	0	0	0	0	0	2	0
ENSMUSG00000107730	2	0	0	0	0	0	3	0	0	0
ENSMUSG00000107731	0	0	0	0	0	0	0	0	0	0
ENSMUSG00000107732	0	0	0	0	0	0	0	6	4	3
ENSMUSG00000107738	0	0	0	0	0	0	0	0	0	0
ENSMUSG00000107739	0	0	0	0	0	0	0	0	0	0
ENSMUSG00000111927	0	0	0	0	0	0	0	0	0	0
ENSMUSG00000111924	0	0	0	0	0	0	0	0	0	0
ENSMUSG00000111925	0	0	0	0	0	0	0	0	0	0
ENSMUSG00000111922	3	6	2	2	0	4	6	0	6	5
ENSMUSG00000111923	0	0	0	0	6	2	0	0	7	6
ENSMUSG00000111920	6	2	3	3	0	0	7	0	9	3
ENSMUSG00000103603	0	0	0	0	0	0	0	0	0	0
ENSMUSG00000103608	0	0	0	0	0	0	0	0	0	0
ENSMUSG00000103609	0	9	0	3	0	6	2	3	3	0
ENSMUSG00000111928	0	2	1	1	3	1	1	3	2	0
ENSMUSG00000085640	0	0	0	0	0	0	0	0	0	0
ENSMUSG00000106295	0	0	0	0	0	0	0	0	0	0
ENSMUSG00000062892	0	0	0	0	0	0	0	0	0	0
ENSMUSG00000036598	0	0	0	0	0	0	0	0	0	3
ENSMUSG00000036596	51	36	62	39	43	76	272	90	110	118
ENSMUSG00000028758	4	21	17	6	12	3	12	8	19	23
ENSMUSG00000096297	0	0	0	0	0	0	0	0	0	0
ENSMUSG00000028753	0	0	0	0	0	0	0	0	0	0
ENSMUSG00000028751	0	11	21	3	10	0	9	8	0	0
ENSMUSG00000114300	0	0	0	0	0	0	0	0	0	0
ENSMUSG00000039215	0	0	0	0	0	0	0	0	0	0
ENSMUSG00000024619	0	0	0	0	2	7	5	0	0	0
ENSMUSG00000096290	0	0	0	0	0	0	0	0	0	0
ENSMUSG00000077270	0	0	0	0	0	0	0	0	0	0
ENSMUSG00000087053	0	0	0	0	0	0	0	0	0	0
ENSMUSG00000020102	929	1021	1049	868	878	1032	1533	1165	924	1062
ENSMUSG00000087051	0	0	0	0	0	0	0	0	2	0
ENSMUSG00000110329	0	3	0	0	0	0	0	0	0	0
ENSMUSG00000110328	0	0	0	0	0	0	0	0	0	0
ENSMUSG00000087056	0	0	0	4	0	0	0	0	0	0
ENSMUSG00000110324	0	0	0	0	0	0	0	0	0	0
ENSMUSG00000110323	0	2	0	0	0	0	0	0	2	2
ENSMUSG00000110322	0	0	0	0	0	0	0	0	0	0
ENSMUSG00000110321	0	0	0	0	0	0	0	0	0	0
ENSMUSG00000110320	0	0	0	0	1	2	9	0	1	0
ENSMUSG00000113784	0	0	0	0	0	0	0	0	0	0
ENSMUSG00000092042	0	0	0	0	0	1	0	0	0	0
ENSMUSG00000097037	0	0	0	0	0	0	0	0	0	0
ENSMUSG00000092040	0	0	0	0	0	0	0	0	0	0

ENSMUSG00000092047	0	1	0	0	0	0	0	0	0	0
ENSMUSG00000087054	0	0	0	0	0	3	0	0	0	0
ENSMUSG00000092045	0	0	0	0	0	0	0	0	0	0
ENSMUSG00000113783	0	0	0	0	0	0	0	0	0	0
ENSMUSG00000092048	0	0	0	0	0	0	0	0	0	0
ENSMUSG00000097039	91	99	76	144	112	192	245	144	201	149
ENSMUSG00000097038	0	0	0	0	0	0	0	0	0	0
ENSMUSG00000055150	141	178	148	74	105	92	297	164	197	91
ENSMUSG00000112786	0	0	0	0	0	0	2	0	0	0
ENSMUSG00000112783	0	3	6	3	0	0	1	0	0	0
ENSMUSG00000114837	0	0	0	0	0	0	0	9	0	3
ENSMUSG00000114833	0	8	0	0	0	6	5	0	0	8
ENSMUSG00000114832	0	0	0	0	0	0	0	0	0	0
ENSMUSG00000114831	0	0	0	0	0	0	0	0	0	0
ENSMUSG00000025058	126	126	148	222	148	154	236	121	260	75
ENSMUSG00000025056	0	0	0	0	0	0	0	0	0	0
ENSMUSG00000105141	0	0	0	0	0	0	0	0	0	0
ENSMUSG00000106957	0	0	0	0	0	11	0	0	0	0
ENSMUSG00000106955	0	0	0	0	0	0	0	0	0	0
ENSMUSG00000106954	0	0	0	0	0	0	0	0	0	0
ENSMUSG00000108380	0	0	0	0	0	0	0	0	0	0
ENSMUSG00000113437	0	0	0	0	0	0	0	0	0	0
ENSMUSG00000108382	0	0	0	0	0	2	0	4	0	0
ENSMUSG00000108383	0	0	0	0	0	0	0	0	0	0
ENSMUSG00000108384	0	0	0	0	0	0	0	2	0	0
ENSMUSG00000108385	0	0	0	0	0	0	0	0	0	0
ENSMUSG00000108387	0	0	0	0	0	0	0	0	0	0
ENSMUSG00000099507	0	0	0	0	0	0	2	0	0	0
ENSMUSG00000069258	0	0	0	0	0	0	0	0	0	0
ENSMUSG00000069259	0	0	0	0	0	0	0	0	0	0
ENSMUSG00000105602	0	0	0	0	0	0	0	0	0	0
ENSMUSG00000105603	0	0	0	0	0	0	0	0	6	0
ENSMUSG00000109381	3	0	0	0	0	0	0	0	0	0
ENSMUSG00000109385	0	0	0	0	0	0	0	0	0	0
ENSMUSG00000105605	0	0	0	0	0	0	0	0	0	0
ENSMUSG00000114781	0	0	0	0	0	0	0	0	0	0
ENSMUSG00000090427	0	0	0	0	0	0	0	0	0	0
ENSMUSG00000114783	0	0	0	0	0	0	2	0	0	2
ENSMUSG00000114784	10	5	28	33	8	6	26	18	19	15
ENSMUSG00000114787	0	5	0	0	3	2	0	3	0	0
ENSMUSG00000114786	0	0	0	0	0	0	0	0	0	0
ENSMUSG00000096344	0	0	0	0	0	0	0	0	0	0
ENSMUSG00000096345	0	0	0	0	0	0	0	0	0	0
ENSMUSG00000096347	0	0	0	0	0	0	0	0	0	0
ENSMUSG00000096340	0	0	0	0	0	0	0	0	0	0
ENSMUSG00000081948	0	0	0	0	0	0	0	0	0	0
ENSMUSG00000096342	0	0	0	0	0	0	0	0	0	0
ENSMUSG00000096348	0	0	0	0	0	0	0	0	0	0
ENSMUSG00000050478	0	0	0	0	0	0	0	2	0	0
ENSMUSG00000070368	0	0	0	0	0	0	0	12	6	0
ENSMUSG00000082975	0	0	0	0	0	0	0	0	0	0
ENSMUSG00000082973	0	0	0	0	0	0	0	0	0	0
ENSMUSG00000082970	0	0	0	0	0	0	0	0	0	0
ENSMUSG00000082971	0	0	0	0	0	0	0	0	0	0
ENSMUSG00000070360	0	0	0	0	0	0	0	0	0	0
ENSMUSG00000050473	2	3	9	0	3	2	0	9	0	0
ENSMUSG00000070366	0	3	0	0	0	2	0	0	0	0
ENSMUSG00000105142	0	0	0	0	0	0	0	0	0	2

ENSMUSG00000111498	3	0	5	4	1	6	6	1	1	3
ENSMUSG00000111499	0	0	0	0	0	0	0	0	0	0
ENSMUSG00000111494	0	0	0	0	0	0	0	0	0	0
ENSMUSG00000081765	0	0	0	0	0	0	0	0	0	0
ENSMUSG00000111493	0	0	0	0	0	0	0	0	0	0
ENSMUSG00000111490	9	5	13	15	2	5	2	15	5	1
ENSMUSG00000109924	0	0	0	0	0	0	0	0	0	0
ENSMUSG00000109922	0	0	0	0	0	0	0	0	0	0
ENSMUSG00000109921	0	0	0	0	0	0	0	0	0	0
ENSMUSG00000109920	0	0	0	0	0	0	0	0	0	0
ENSMUSG00000109929	0	0	0	0	0	0	0	0	0	0
ENSMUSG00000109928	0	0	0	0	0	0	0	0	0	0
ENSMUSG00000108032	0	0	0	0	0	0	0	0	0	0
ENSMUSG00000104553	0	0	0	0	0	0	0	0	0	0
ENSMUSG00000104555	0	0	0	0	0	0	0	0	0	0
ENSMUSG00000104556	0	3	0	0	0	0	0	0	0	0
ENSMUSG00000104558	0	0	0	0	0	0	0	0	1	0
ENSMUSG00000108038	0	0	0	0	0	0	0	0	0	0
ENSMUSG00000108039	0	0	0	0	1	2	0	1	0	0
ENSMUSG00000040866	0	0	0	0	0	0	0	9	3	0
ENSMUSG00000040867	16	37	22	33	10	41	25	33	45	34
ENSMUSG00000093839	0	0	0	0	0	0	0	0	0	0
ENSMUSG00000093831	3	5	0	5	2	6	5	5	5	9
ENSMUSG00000093830	0	0	0	0	0	0	0	0	0	0
ENSMUSG00000111144	0	0	0	0	0	2	0	0	0	0
ENSMUSG00000111146	0	0	0	0	0	0	0	0	0	0
ENSMUSG00000111147	0	6	0	8	2	6	0	3	0	3
ENSMUSG00000044526	0	0	0	0	0	0	0	0	0	0
ENSMUSG00000022144	36	27	14	42	46	12	51	32	29	26
ENSMUSG00000084505	0	0	0	0	0	0	0	0	0	0
ENSMUSG00000109909	6	10	16	8	5	9	6	8	10	16
ENSMUSG00000094506	1	0	1	1	0	0	0	0	0	0
ENSMUSG00000056162	0	3	3	8	0	0	12	6	0	3
ENSMUSG00000104801	34	18	37	70	27	70	58	43	85	40
ENSMUSG00000078212	0	0	0	0	0	0	0	0	0	0
ENSMUSG00000078213	0	0	0	0	0	0	0	0	0	0
ENSMUSG00000078210	0	0	0	0	0	0	0	0	0	0
ENSMUSG00000078214	0	0	0	0	0	0	0	0	0	0
ENSMUSG00000046016	0	0	0	0	0	0	0	0	0	0
ENSMUSG00000095064	0	0	0	0	0	0	0	0	0	3
ENSMUSG00000095065	0	0	0	0	0	0	0	0	0	0
ENSMUSG00000095066	0	0	0	0	0	0	0	0	0	0
ENSMUSG00000095060	0	0	0	0	0	0	0	0	0	0
ENSMUSG00000095063	0	0	0	0	0	0	0	0	0	0
ENSMUSG00000095069	0	0	0	0	0	0	1	0	0	0
ENSMUSG00000053129	0	0	0	0	0	0	0	0	0	0
ENSMUSG00000100074	0	0	0	0	0	0	0	0	0	0
ENSMUSG00000058662	0	0	0	0	0	0	0	0	0	0
ENSMUSG00000092876	0	0	0	0	0	0	0	0	0	0
ENSMUSG00000027368	51	69	45	71	24	104	123	60	85	62
ENSMUSG00000092879	0	0	0	0	0	0	0	0	0	0
ENSMUSG00000090588	0	0	0	0	0	0	0	0	0	0
ENSMUSG00000090585	0	2	0	1	1	0	3	1	2	0
ENSMUSG00000090581	0	0	0	0	0	0	0	0	0	0
ENSMUSG00000105227	3	0	0	0	0	0	0	0	0	0
ENSMUSG00000073043	0	0	0	0	0	0	3	0	0	0
ENSMUSG00000073045	0	0	0	0	0	0	0	0	0	0
ENSMUSG00000080609	0	0	0	0	0	0	0	0	0	0

ENSMUSG00000080607	0	0	1	0	0	0	0	0	0	0
ENSMUSG00000080600	0	0	0	0	0	0	0	0	0	0
ENSMUSG00000093581	0	0	0	0	0	0	0	0	0	0
ENSMUSG00000093580	0	0	0	3	0	0	0	0	0	0
ENSMUSG00000093582	0	0	0	0	0	0	0	0	0	0
ENSMUSG00000111858	0	3	7	0	2	0	0	15	7	10
ENSMUSG00000093587	6	13	21	29	8	13	26	15	14	12
ENSMUSG00000093589	0	0	0	0	0	0	0	0	0	0
ENSMUSG00000093588	0	0	0	0	0	0	0	0	0	0
ENSMUSG00000078962	0	0	0	0	0	0	1	0	4	3
ENSMUSG00000078964	0	0	0	0	0	0	0	0	0	0
ENSMUSG00000052749	46	60	81	472	124	107	132	80	98	77
ENSMUSG00000111857	12	0	5	3	0	0	3	3	6	6
ENSMUSG00000109116	0	0	0	0	0	0	0	0	0	0
ENSMUSG00000109117	0	0	0	0	0	0	0	0	0	0
ENSMUSG00000077293	0	0	0	0	0	0	0	0	0	0
ENSMUSG00000077291	0	0	0	0	0	0	0	0	0	0
ENSMUSG00000077290	0	0	0	0	0	0	0	0	0	0
ENSMUSG00000111850	0	0	0	0	0	0	0	0	0	0
ENSMUSG00000077295	0	0	0	0	0	0	0	0	0	0
ENSMUSG00000031241	0	0	0	0	0	0	0	0	0	0
ENSMUSG00000077299	0	0	0	0	0	0	0	0	0	0
ENSMUSG00000077298	0	0	0	0	0	0	0	0	0	0
ENSMUSG00000114919	0	0	0	0	0	0	0	0	0	0
ENSMUSG00000093059	0	0	0	0	0	0	0	0	0	0
ENSMUSG00000093056	0	0	0	2	0	0	0	0	0	0
ENSMUSG00000055010	0	0	0	0	0	0	0	0	0	0
ENSMUSG00000114915	0	0	0	0	0	0	0	0	0	0
ENSMUSG00000096241	0	0	0	0	0	0	0	0	0	0
ENSMUSG00000073658	0	0	0	0	0	0	0	0	0	0
ENSMUSG00000114912	0	0	0	0	0	0	0	0	0	0
ENSMUSG00000114913	0	0	0	0	0	0	0	0	0	0
ENSMUSG00000040413	3	0	3	9	3	0	9	0	0	0
ENSMUSG00000040412	3	0	3	0	0	12	3	0	0	6
ENSMUSG00000077435	0	0	0	0	0	0	0	0	0	0
ENSMUSG00000037979	38	52	48	135	60	53	95	74	63	120
ENSMUSG00000077433	0	0	0	0	0	0	0	0	0	0
ENSMUSG00000077430	0	0	0	0	0	0	0	0	0	0
ENSMUSG00000037973	0	0	0	0	0	0	0	0	0	0
ENSMUSG00000088546	0	0	0	0	0	0	0	0	0	0
ENSMUSG00000077439	0	0	0	0	0	0	0	0	0	0
ENSMUSG00000055210	58	29	39	21	15	14	19	61	26	40
ENSMUSG00000033080	0	0	3	0	0	0	0	0	0	0
ENSMUSG00000105308	0	0	0	0	0	0	0	0	0	0
ENSMUSG00000030196	0	0	0	0	0	0	0	0	0	0
ENSMUSG00000073959	0	0	0	0	0	0	0	0	0	0
ENSMUSG00000073950	0	0	0	0	0	0	0	0	0	0
ENSMUSG00000073951	0	0	0	0	0	0	0	0	0	0
ENSMUSG00000073952	0	0	0	0	0	0	0	0	0	0
ENSMUSG00000073953	0	0	0	0	0	0	0	0	0	0
ENSMUSG00000073954	0	0	0	0	0	0	0	0	0	0
ENSMUSG00000073955	0	0	0	0	0	0	0	0	0	0
ENSMUSG00000073956	0	0	0	0	0	0	0	0	0	0
ENSMUSG00000105307	0	0	0	0	0	0	0	0	0	0
ENSMUSG00000071553	3	0	0	0	0	0	9	0	9	0
ENSMUSG00000071550	4	20	11	3	2	0	11	5	11	8
ENSMUSG00000071551	116	122	176	103	99	157	185	195	181	207
ENSMUSG00000095914	0	0	0	0	0	0	0	0	0	0

ENSMUSG00000099518	1	1	1	2	2	3	2	1	2	1
ENSMUSG00000099511	0	0	0	0	0	0	0	0	0	0
ENSMUSG00000099513	0	0	0	0	0	0	0	0	0	0
ENSMUSG00000099514	0	0	0	0	0	0	0	0	0	0
ENSMUSG00000099516	0	0	0	0	0	0	0	0	0	0
ENSMUSG00000083729	0	0	0	0	0	0	0	0	0	0
ENSMUSG00000102019	0	0	0	0	0	0	0	0	0	0
ENSMUSG00000083720	0	0	0	3	0	0	0	0	0	3
ENSMUSG00000083721	0	0	0	0	0	0	0	0	0	0
ENSMUSG00000083722	0	0	0	0	0	0	0	0	0	0
ENSMUSG00000083725	3	3	6	6	2	0	0	2	0	0
ENSMUSG00000043850	0	0	0	0	0	6	28	0	6	0
ENSMUSG00000043857	27	26	93	9	11	94	224	115	188	75
ENSMUSG00000043855	0	0	0	0	0	0	0	0	0	0
ENSMUSG00000043859	0	0	0	0	0	0	0	0	0	0
ENSMUSG00000100003	0	0	0	0	0	0	0	0	0	0
ENSMUSG00000100001	0	0	0	0	0	0	0	0	0	0
ENSMUSG00000100000	0	0	0	0	0	2	0	0	0	0
ENSMUSG00000072601	0	0	0	0	0	0	0	0	0	0
ENSMUSG00000100004	0	0	0	0	0	0	0	0	0	0
ENSMUSG00000070601	3	0	3	0	0	3	5	0	9	3
ENSMUSG00000070606	0	0	0	0	0	0	0	0	0	0
ENSMUSG00000102538	0	0	0	0	0	0	0	1	0	0
ENSMUSG00000102535	0	0	0	0	0	0	0	0	0	0
ENSMUSG00000102534	0	0	0	0	0	0	0	0	0	0
ENSMUSG00000102536	0	0	0	0	0	0	0	0	0	0
ENSMUSG00000102531	24	32	34	46	30	42	51	42	30	35
ENSMUSG00000102530	0	0	0	0	0	0	0	0	0	0
ENSMUSG00000102533	63	47	67	40	34	31	49	53	57	47
ENSMUSG00000102532	0	0	0	0	0	0	0	0	0	0
ENSMUSG00000075079	0	0	0	0	0	0	0	0	0	0
ENSMUSG00000075078	0	0	0	0	0	0	0	0	0	0
ENSMUSG00000084515	0	0	0	0	0	0	0	0	0	0
ENSMUSG00000084512	0	0	0	0	0	2	6	0	2	0
ENSMUSG00000084513	0	0	0	0	0	0	0	0	0	0
ENSMUSG00000084511	0	0	0	0	0	0	0	0	0	0
ENSMUSG00000075073	0	0	0	0	0	0	0	0	0	0
ENSMUSG00000075072	0	0	0	0	0	0	0	0	0	0
ENSMUSG00000075075	0	0	0	0	12	0	0	0	0	0
ENSMUSG00000075074	0	0	0	0	0	0	0	0	0	0
ENSMUSG00000084518	0	0	0	0	0	0	0	0	0	0
ENSMUSG00000000215	0	0	0	0	0	0	5	0	0	0
ENSMUSG00000000214	0	0	0	0	0	0	8	0	0	0
ENSMUSG00000000216	0	3	0	6	0	3	0	0	0	0
ENSMUSG00000065807	0	0	0	0	0	0	0	0	0	0
ENSMUSG00000065803	0	0	0	0	0	0	0	0	0	0
ENSMUSG00000065802	0	0	0	0	0	0	0	0	0	0
ENSMUSG00000088621	0	0	0	0	0	0	0	0	0	0
ENSMUSG00000074388	2	0	3	0	3	0	3	3	3	2
ENSMUSG00000075383	0	0	0	0	0	0	0	0	0	0
ENSMUSG00000075382	0	0	0	0	0	0	0	0	0	0
ENSMUSG00000079600	0	0	0	0	0	0	0	0	0	0
ENSMUSG00000075380	0	0	0	0	0	0	0	0	0	0
ENSMUSG00000075387	0	0	0	0	0	0	0	0	0	0
ENSMUSG00000079604	3	0	0	0	0	0	3	2	0	0
ENSMUSG00000075384	0	0	0	0	0	0	0	0	0	0
ENSMUSG00000088518	0	0	0	0	0	0	0	0	0	0
ENSMUSG00000101848	0	0	0	3	0	0	8	0	0	0

ENSMUSG00000077829	0	0	0	0	0	0	0	0	0	0
ENSMUSG00000101844	0	0	0	0	0	0	0	0	0	0
ENSMUSG00000088510	0	0	0	0	0	0	0	0	0	0
ENSMUSG00000035020	3	12	9	14	8	11	6	17	12	6
ENSMUSG00000107803	0	0	0	0	0	0	0	0	0	0
ENSMUSG00000088517	0	0	0	0	0	0	0	0	0	0
ENSMUSG00000101842	0	0	0	0	0	0	0	0	0	0
ENSMUSG00000101843	0	0	0	0	2	0	0	0	0	0
ENSMUSG00000085399	60	41	75	52	38	77	93	85	95	85
ENSMUSG00000085394	0	0	0	2	0	0	3	0	0	0
ENSMUSG00000085393	0	0	0	0	0	0	0	0	0	0
ENSMUSG00000085390	0	0	0	0	0	0	0	0	0	0
ENSMUSG00000033502	412	410	539	547	314	562	813	618	614	529
ENSMUSG00000033501	0	0	0	0	0	0	0	0	0	0
ENSMUSG00000103723	2	0	0	0	2	0	0	1	3	0
ENSMUSG00000103727	0	0	1	0	0	0	3	3	1	1
ENSMUSG00000103725	0	0	2	0	0	0	0	0	0	0
ENSMUSG00000065373	0	0	0	0	0	0	0	0	1	0
ENSMUSG00000065372	0	0	0	0	0	0	0	0	0	0
ENSMUSG00000065371	0	0	3	0	0	0	0	0	3	2
ENSMUSG00000103728	0	0	0	0	0	0	0	0	0	0
ENSMUSG00000024459	8	0	3	8	3	9	5	3	0	0
ENSMUSG00000078554	0	3	0	0	0	0	0	3	10	4
ENSMUSG00000028593	0	0	0	0	0	0	0	0	0	0
ENSMUSG00000084611	2	2	3	3	3	1	5	0	2	3
ENSMUSG00000030976	0	0	0	0	0	0	0	0	0	0
ENSMUSG00000087504	0	0	0	0	0	0	0	0	0	0
ENSMUSG00000073565	12	5	8	8	10	11	80	5	26	0
ENSMUSG00000097236	56	51	42	50	27	52	62	42	66	58
ENSMUSG00000073568	0	0	0	0	0	3	3	0	0	0
ENSMUSG00000082076	0	0	0	0	0	0	0	0	0	0
ENSMUSG00000035226	276	234	327	223	230	212	172	209	226	241
ENSMUSG00000039760	0	0	0	6	0	0	5	6	18	0
ENSMUSG00000089642	0	0	0	0	0	0	0	0	0	0
ENSMUSG00000089645	0	2	0	0	2	0	0	2	0	3
ENSMUSG00000100826	2	3	3	5	0	3	28	8	0	0
ENSMUSG00000100821	0	0	0	0	0	0	0	0	0	0
ENSMUSG00000100820	0	0	0	0	0	0	0	0	0	0
ENSMUSG00000097584	0	0	0	0	0	0	0	0	0	0
ENSMUSG00000102092	0	0	0	0	0	0	0	0	0	0
ENSMUSG00000102090	0	0	0	0	0	0	0	0	0	0
ENSMUSG00000102096	0	0	0	0	0	0	0	0	0	0
ENSMUSG00000102095	4	9	10	22	4	0	0	7	4	9
ENSMUSG00000062342	0	0	0	0	0	0	0	0	0	0
ENSMUSG00000102098	51	47	41	54	17	38	19	39	22	50
ENSMUSG00000102099	2	0	0	3	0	0	0	3	0	0
ENSMUSG00000062345	12	6	10	108	48	21	12	38	24	64
ENSMUSG00000096182	0	0	0	0	0	0	0	0	0	0
ENSMUSG00000096183	0	0	0	0	0	0	0	0	0	0
ENSMUSG00000096181	0	0	0	0	0	0	0	0	0	0
ENSMUSG00000096186	0	0	0	0	0	0	0	0	0	0
ENSMUSG00000096184	0	0	0	0	0	0	0	0	0	0
ENSMUSG00000096185	0	0	0	0	0	0	0	0	0	0
ENSMUSG00000096189	0	0	0	0	0	0	0	0	0	0
ENSMUSG00000113946	2	4	3	2	0	3	1	8	6	5
ENSMUSG00000113947	0	6	0	0	0	0	0	0	0	0
ENSMUSG00000113944	0	0	0	0	0	0	0	0	0	0
ENSMUSG00000101315	0	0	0	0	0	0	2	0	0	0

ENSMUSG00000057228	0	0	3	35	0	0	0	0	0	0
ENSMUSG00000113943	0	0	0	0	0	0	0	0	0	0
ENSMUSG00000096109	0	0	0	0	0	0	0	0	0	0
ENSMUSG00000105372	0	0	0	0	0	0	0	0	0	0
ENSMUSG00000105373	2	5	8	4	5	8	6	6	2	6
ENSMUSG00000105370	2	0	0	0	0	0	0	0	0	0
ENSMUSG00000067331	0	2	0	0	0	0	0	0	0	0
ENSMUSG00000105377	0	0	3	3	0	3	3	6	14	0
ENSMUSG00000105375	0	0	0	0	0	0	0	0	0	0
ENSMUSG00000110748	0	0	0	0	0	0	0	0	0	0
ENSMUSG00000029576	174	157	116	155	96	158	267	165	199	180
ENSMUSG00000108630	0	0	0	0	0	0	0	0	0	0
ENSMUSG00000055811	0	0	0	0	0	0	0	0	0	0
ENSMUSG00000108634	28	59	65	58	42	36	66	44	70	59
ENSMUSG00000050671	7	12	7	7	7	12	34	3	0	9
ENSMUSG00000108638	0	0	2	2	0	2	0	0	0	0
ENSMUSG00000108639	0	0	0	0	0	0	0	0	0	0
ENSMUSG00000109092	0	0	0	0	0	0	0	0	0	0
ENSMUSG00000067591	0	9	3	7	8	7	17	10	16	3
ENSMUSG00000021240	434	458	511	490	349	515	691	620	617	558
ENSMUSG00000067594	6	0	0	0	0	0	8	0	0	0
ENSMUSG00000067597	3	0	0	0	0	3	6	0	0	0
ENSMUSG00000105959	0	0	0	0	0	0	0	0	0	0
ENSMUSG00000105955	0	0	0	0	0	0	0	0	0	0
ENSMUSG00000105324	0	0	0	0	0	0	0	0	0	0
ENSMUSG00000057021	0	0	0	0	0	0	0	0	0	0
ENSMUSG00000101850	0	0	0	0	0	0	0	0	0	0
ENSMUSG00000104029	0	0	3	3	0	0	0	1	0	0
ENSMUSG00000101019	0	0	0	0	0	0	0	0	0	0
ENSMUSG00000103949	1	0	0	0	0	0	0	0	0	0
ENSMUSG00000104021	0	0	0	0	0	0	0	0	0	0
ENSMUSG00000101012	0	0	0	0	0	0	0	0	0	0
ENSMUSG00000101011	0	0	0	0	2	0	0	0	0	0
ENSMUSG00000104022	0	0	0	0	0	0	0	1	0	0
ENSMUSG00000104025	5	2	8	0	3	0	8	0	0	2
ENSMUSG00000094599	0	0	0	0	0	0	0	0	0	0
ENSMUSG00000101015	0	0	0	0	0	0	0	0	0	0
ENSMUSG00000103947	0	0	0	0	0	0	0	0	0	0
ENSMUSG00000094598	0	0	0	0	0	0	0	0	0	0
ENSMUSG00000039714	0	0	0	0	0	0	38	3	16	9
ENSMUSG00000103944	0	0	0	0	0	0	0	0	0	0
ENSMUSG00000103943	0	0	0	0	0	0	0	0	0	0
ENSMUSG00000010435	0	0	0	0	0	0	0	0	0	0
ENSMUSG00000094593	0	0	0	1	0	0	0	0	0	0
ENSMUSG00000094590	0	0	0	0	0	0	0	0	0	0
ENSMUSG00000094597	0	0	0	0	0	0	0	0	0	0
ENSMUSG00000094595	14	24	31	52	38	46	79	64	17	88
ENSMUSG00000103245	0	0	0	0	0	0	0	0	0	0
ENSMUSG00000059411	3	0	0	0	3	0	0	0	0	0
ENSMUSG00000059410	0	0	0	0	0	0	0	0	0	0
ENSMUSG00000064818	0	0	0	0	0	0	0	0	0	0
ENSMUSG00000091325	0	0	0	0	0	0	0	0	0	0
ENSMUSG00000103240	0	0	0	0	0	0	0	0	0	0
ENSMUSG00000103242	0	0	1	0	0	0	0	0	3	0
ENSMUSG00000110958	0	0	0	0	0	0	0	0	3	0
ENSMUSG00000110956	0	0	0	0	0	0	0	0	0	0
ENSMUSG00000110957	0	0	0	0	0	0	0	0	0	0
ENSMUSG00000110951	0	0	0	0	0	0	0	0	0	0

ENSMUSG00000110952	0	0	0	0	0	0	0	0	0	0
ENSMUSG00000110953	0	0	0	0	0	0	0	0	0	0
ENSMUSG00000056089	0	0	0	0	0	2	0	0	0	0
ENSMUSG00000095518	0	0	0	0	0	0	0	0	0	0
ENSMUSG00000090742	0	0	0	0	0	0	0	0	0	0
ENSMUSG00000090740	0	0	0	0	0	0	0	0	0	0
ENSMUSG00000095510	0	0	0	3	3	0	0	0	0	0
ENSMUSG00000090747	0	0	0	0	0	0	0	0	0	0
ENSMUSG00000090744	0	0	0	0	0	0	3	0	0	3
ENSMUSG00000036760	0	0	0	0	0	0	0	0	0	0
ENSMUSG00000042279	0	0	0	2	0	0	0	0	0	5
ENSMUSG00000112086	0	0	0	0	0	0	0	0	0	0
ENSMUSG00000112087	0	0	0	0	0	0	0	2	0	0
ENSMUSG00000112089	0	0	0	0	0	0	0	0	0	0
ENSMUSG00000034783	110	131	82	36	70	226	66	104	30	14
ENSMUSG00000086445	0	0	0	0	0	0	0	0	0	0
ENSMUSG00000086443	0	0	0	0	0	0	0	0	0	0
ENSMUSG00000086441	0	0	0	0	0	0	0	0	0	0
ENSMUSG00000086449	0	0	0	0	0	0	0	0	5	0
ENSMUSG00000105487	0	0	0	0	0	0	0	0	0	0
ENSMUSG00000096818	0	0	0	0	0	0	0	4	3	0
ENSMUSG00000109180	0	0	0	0	0	0	0	0	0	0
ENSMUSG00000108798	0	0	0	0	0	0	0	0	0	0
ENSMUSG00000108796	0	0	0	0	0	0	0	0	0	0
ENSMUSG00000060057	0	0	0	0	0	0	0	0	0	0
ENSMUSG00000111316	0	0	0	0	0	0	0	0	0	0
ENSMUSG00000108794	0	0	0	0	0	0	0	0	0	0
ENSMUSG00000093211	0	0	0	0	0	0	0	0	0	0
ENSMUSG00000103754	4	15	5	8	5	7	7	9	4	2
ENSMUSG00000051885	0	0	0	0	0	0	0	0	0	0
ENSMUSG00000096810	27	43	58	40	40	50	44	41	43	32
ENSMUSG00000109188	0	0	0	0	0	0	0	0	0	0
ENSMUSG00000103752	0	0	0	0	0	0	0	0	0	0
ENSMUSG00000104471	0	0	0	0	0	0	0	0	0	0
ENSMUSG00000052504	77	106	87	51	70	127	158	147	63	79
ENSMUSG00000036251	0	0	1	0	0	0	2	0	0	0
ENSMUSG00000065309	0	0	0	0	0	0	0	0	0	0
ENSMUSG00000052508	0	0	0	0	0	0	0	0	0	0
ENSMUSG00000028386	8	15	9	2	3	15	5	15	9	14
ENSMUSG00000097914	4	0	5	10	6	3	17	12	2	3
ENSMUSG00000097911	8	8	0	15	3	12	12	24	10	13
ENSMUSG00000112624	0	0	0	0	0	0	0	0	0	0
ENSMUSG00000112625	0	0	0	0	0	0	0	0	0	0
ENSMUSG00000112626	0	0	0	0	0	0	0	0	0	0
ENSMUSG00000112620	0	0	0	0	0	0	0	0	2	0
ENSMUSG00000112622	0	0	8	0	3	4	2	2	4	2
ENSMUSG00000112623	2	0	0	0	0	0	1	6	0	0
ENSMUSG00000049362	3	10	4	11	3	12	3	3	26	11
ENSMUSG00000112180	0	0	0	0	0	0	0	0	0	0
ENSMUSG00000112628	0	0	3	0	0	0	5	0	0	0
ENSMUSG00000073973	4	3	0	3	0	0	6	0	5	3
ENSMUSG00000054905	0	0	0	0	3	0	0	0	0	0
ENSMUSG00000047356	0	0	0	0	0	0	0	0	0	0
ENSMUSG00000047352	0	0	0	0	0	0	0	0	0	0
ENSMUSG00000109229	0	0	0	0	0	0	0	0	0	0
ENSMUSG00000003309	0	0	3	0	3	0	5	0	0	0
ENSMUSG00000109223	0	0	0	0	0	6	0	0	2	0
ENSMUSG00000051262	87	74	39	48	25	79	63	65	28	61

ENSMUSG00000109226	0	0	0	0	0	0	0	0	0	0
ENSMUSG00000109227	0	0	0	0	0	0	0	0	0	0
ENSMUSG00000045394	9	12	3	3	0	0	8	9	21	18
ENSMUSG00000106566	0	0	0	0	0	0	0	0	0	0
ENSMUSG00000106564	0	0	2	3	0	0	1	0	0	0
ENSMUSG00000106569	0	0	0	0	0	0	0	0	0	0
ENSMUSG00000114668	0	0	0	0	0	0	0	0	0	0
ENSMUSG00000114669	0	0	0	0	0	0	0	0	0	0
ENSMUSG00000114666	0	0	0	0	0	0	0	0	0	0
ENSMUSG00000114667	0	0	0	0	0	0	0	0	0	0
ENSMUSG00000114664	6	0	13	3	12	17	30	3	9	16
ENSMUSG00000114665	9	3	33	13	29	24	28	23	7	38
ENSMUSG00000114663	0	0	0	0	0	0	0	0	0	0
ENSMUSG00000114660	23	27	18	8	23	18	22	18	22	21
ENSMUSG00000110178	0	0	0	0	0	0	0	0	0	0
ENSMUSG00000110179	6	6	0	0	7	4	0	8	3	3
ENSMUSG00000110176	0	0	0	0	0	0	0	0	0	0
ENSMUSG00000110174	0	0	0	0	0	0	0	0	0	0
ENSMUSG00000110172	0	1	0	5	0	0	2	0	0	5
ENSMUSG00000110171	0	0	0	0	0	0	0	0	0	0
ENSMUSG00000101093	0	0	0	0	0	0	0	0	0	0
ENSMUSG00000070448	0	0	0	0	0	0	0	0	0	0
ENSMUSG00000108177	0	0	0	0	0	0	0	0	0	0
ENSMUSG00000108174	0	0	0	0	0	0	0	0	0	0
ENSMUSG00000108172	0	0	0	0	0	0	0	0	0	0
ENSMUSG00000091601	0	0	0	0	0	0	0	0	0	0
ENSMUSG00000108178	0	0	0	0	0	0	0	0	0	0
ENSMUSG00000105032	0	0	0	0	0	0	0	0	0	3
ENSMUSG00000105035	0	0	0	0	3	0	0	0	3	0
ENSMUSG00000105034	0	0	0	0	0	0	0	0	0	0
ENSMUSG00000105037	0	0	0	0	0	0	0	0	0	0
ENSMUSG00000105036	3	0	0	3	0	0	2	0	0	0
ENSMUSG00000105039	0	0	0	0	0	0	0	0	0	0
ENSMUSG00000105038	0	0	0	0	0	0	0	0	0	0
ENSMUSG00000101094	0	0	0	0	0	0	0	0	0	0
ENSMUSG00000041219	338	291	397	253	261	377	460	398	405	330
ENSMUSG00000102203	0	0	0	0	0	0	0	0	0	0
ENSMUSG00000091453	0	0	0	0	0	0	0	0	0	0
ENSMUSG00000044199	113	95	131	203	124	54	189	110	155	134
ENSMUSG00000083250	0	0	0	0	0	0	3	0	0	0
ENSMUSG00000083251	0	0	0	0	0	0	0	0	0	0
ENSMUSG00000083252	0	0	0	0	0	0	0	0	0	0
ENSMUSG00000083253	0	0	0	0	0	0	0	0	0	0
ENSMUSG00000083254	0	0	0	0	0	0	0	0	0	0
ENSMUSG00000083255	0	0	0	0	0	0	0	0	0	0
ENSMUSG00000083256	0	0	0	0	0	0	0	0	0	0
ENSMUSG00000083257	0	0	0	0	0	0	0	0	0	0
ENSMUSG00000083258	3	0	3	2	3	2	0	0	0	0
ENSMUSG00000085158	0	0	0	0	0	3	0	0	0	0
ENSMUSG00000085155	0	0	0	0	0	0	0	0	0	0
ENSMUSG00000085157	0	0	0	0	0	0	0	0	0	0
ENSMUSG00000085150	0	0	0	0	0	0	0	0	0	0
ENSMUSG00000085152	6	6	4	14	5	13	0	0	3	4
ENSMUSG00000040138	9	15	3	0	6	12	18	5	3	0
ENSMUSG00000079378	0	0	0	0	0	0	0	0	0	0
ENSMUSG00000079379	0	0	0	0	0	0	0	0	0	0
ENSMUSG00000079374	0	0	0	0	0	0	0	0	0	0
ENSMUSG00000041961	592	552	602	396	480	629	503	409	521	684

ENSMUSG00000041966	731	702	838	818	623	366	990	956	1134	449
ENSMUSG00000088779	0	0	0	0	0	0	0	0	0	0
ENSMUSG00000074639	0	0	6	3	0	0	10	0	0	6
ENSMUSG00000083871	0	0	0	0	0	0	0	0	0	0
ENSMUSG00000083872	0	0	0	0	0	0	0	0	0	0
ENSMUSG00000083873	0	0	0	0	0	0	3	0	0	0
ENSMUSG00000109723	0	0	0	0	0	0	0	0	0	0
ENSMUSG00000053375	0	0	0	0	0	0	3	0	0	0
ENSMUSG00000027444	0	0	0	0	0	0	0	0	0	0
ENSMUSG00000027443	0	3	7	0	0	8	6	0	0	0
ENSMUSG00000027442	3	0	3	3	0	0	0	0	3	6
ENSMUSG00000092931	0	0	0	0	0	0	0	3	0	0
ENSMUSG00000110076	0	0	0	0	0	0	0	0	0	0
ENSMUSG00000078025	0	0	0	0	0	0	0	0	0	1
ENSMUSG00000078024	0	0	0	0	0	0	0	0	0	0
ENSMUSG00000092939	0	0	0	0	0	0	0	0	0	0
ENSMUSG00000078026	0	0	0	2	0	0	0	0	0	0
ENSMUSG00000110072	0	0	0	0	0	0	0	0	0	0
ENSMUSG00000090265	0	0	0	0	0	0	0	0	0	0
ENSMUSG00000090268	0	0	0	0	0	0	0	0	0	0
ENSMUSG00000098476	0	0	0	0	0	0	0	0	0	0
ENSMUSG00000098471	0	0	0	0	0	0	0	0	0	0
ENSMUSG00000098472	0	0	0	0	0	0	0	0	0	0
ENSMUSG00000098478	0	0	0	0	0	0	0	0	0	0
ENSMUSG00000098479	0	0	0	0	0	0	0	0	0	0
ENSMUSG00000094623	0	0	0	0	0	0	0	0	0	0
ENSMUSG00000046607	85	66	52	73	38	85	40	65	36	81
ENSMUSG00000046160	6	3	0	0	2	3	5	0	8	0
ENSMUSG00000048559	0	0	0	0	0	0	0	0	0	0
ENSMUSG00000095839	0	0	0	0	0	0	0	0	0	0
ENSMUSG00000105464	0	0	0	0	0	0	0	0	0	0
ENSMUSG00000034829	6	0	0	9	0	3	37	3	0	4
ENSMUSG00000100109	0	0	0	0	0	0	0	0	0	0
ENSMUSG00000062082	45	52	44	137	48	55	115	64	66	33
ENSMUSG00000072100	0	0	0	0	0	0	0	0	0	0
ENSMUSG00000108853	0	0	0	0	0	0	0	0	0	0
ENSMUSG00000004894	0	0	0	4	0	6	3	3	0	0
ENSMUSG00000004892	18	14	12	13	15	16	19	16	2	20
ENSMUSG00000049605	0	0	0	0	0	0	0	0	0	0
ENSMUSG00000049604	6	0	0	0	0	0	0	0	3	0
ENSMUSG00000087148	3	0	3	0	0	0	0	2	0	0
ENSMUSG00000087149	0	0	6	0	0	0	6	0	0	0
ENSMUSG00000082398	0	0	0	0	0	0	0	0	0	0
ENSMUSG00000082396	7	1	16	8	5	3	15	12	10	6
ENSMUSG00000087145	0	0	0	0	0	0	0	0	0	0
ENSMUSG00000087146	0	0	0	0	0	0	0	0	0	0
ENSMUSG00000087147	0	0	0	0	0	0	0	0	0	0
ENSMUSG00000082392	0	0	0	0	0	0	0	0	0	0
ENSMUSG00000030789	176	165	151	178	121	262	423	200	327	176
ENSMUSG00000082390	0	0	2	0	0	0	0	0	0	0
ENSMUSG00000087143	94	115	86	109	100	133	128	176	157	154
ENSMUSG00000101259	0	0	0	0	0	0	0	0	0	0
ENSMUSG00000031789	66	73	77	65	17	88	85	63	114	88
ENSMUSG00000101251	0	0	0	0	0	0	0	0	0	0
ENSMUSG00000101250	0	0	0	0	0	0	0	0	0	0
ENSMUSG00000101253	0	0	0	0	0	0	0	0	0	0
ENSMUSG00000101252	0	0	3	0	0	0	3	0	0	0
ENSMUSG00000101255	0	0	0	0	0	0	0	0	0	0

ENSMUSG00000101254	0	0	0	0	0	0	0	0	0	0
ENSMUSG00000101257	18	23	32	39	29	26	0	11	10	7
ENSMUSG00000031780	20	0	12	5	8	26	55	14	37	12
ENSMUSG00000037833	77	65	94	156	105	76	161	144	67	92
ENSMUSG00000071454	271	287	290	407	253	243	590	339	462	372
ENSMUSG00000071452	0	0	0	0	0	0	0	0	0	0
ENSMUSG00000067860	0	3	0	6	0	0	3	0	0	0
ENSMUSG00000082649	0	0	0	0	0	0	0	0	0	0
ENSMUSG00000082644	0	0	0	0	0	0	0	0	0	0
ENSMUSG00000099770	0	0	0	0	0	0	0	0	0	0
ENSMUSG00000099771	0	0	0	0	0	0	0	0	0	0
ENSMUSG00000102596	0	0	1	0	1	0	0	0	0	0
ENSMUSG00000040601	0	0	0	0	0	0	0	0	0	0
ENSMUSG00000037780	0	0	0	0	0	0	0	6	0	0
ENSMUSG00000037784	503	453	552	385	402	617	1077	718	728	634
ENSMUSG00000107965	0	0	0	0	1	0	0	0	0	0
ENSMUSG00000107964	0	3	3	0	0	0	6	0	0	6
ENSMUSG00000107967	0	0	0	0	0	0	0	0	0	0
ENSMUSG00000046130	0	0	0	0	0	0	0	0	0	0
ENSMUSG00000103099	0	0	0	0	0	0	0	0	0	0
ENSMUSG00000103095	0	0	0	0	0	0	0	0	0	0
ENSMUSG00000103097	0	0	0	0	0	0	0	0	0	0
ENSMUSG00000103096	0	0	0	0	0	0	0	0	0	0
ENSMUSG00000103091	0	0	0	0	0	0	0	0	0	0
ENSMUSG00000063240	0	0	0	0	0	0	0	0	0	0
ENSMUSG00000032496	19	17	7	111	13	29	3	12	23	18
ENSMUSG00000032494	0	9	4	0	3	6	13	6	8	0
ENSMUSG00000032493	0	0	3	0	0	0	0	0	3	0
ENSMUSG00000032491	105	73	117	117	86	141	212	142	163	114
ENSMUSG00000042854	447	435	414	421	411	332	392	404	413	303
ENSMUSG00000083116	0	6	0	0	9	8	12	0	14	0
ENSMUSG00000075220	0	0	0	0	0	0	0	0	0	0
ENSMUSG00000075221	0	0	0	0	0	0	0	0	0	0
ENSMUSG00000075222	0	0	0	0	1	0	0	1	3	0
ENSMUSG00000075223	0	0	0	1	3	0	0	3	0	0
ENSMUSG00000075224	13	26	3	7	9	28	20	40	27	35
ENSMUSG00000084728	0	0	0	0	0	0	0	0	0	0
ENSMUSG00000083119	0	0	0	0	0	0	0	0	0	0
ENSMUSG00000100610	0	0	0	0	0	0	0	1	0	0
ENSMUSG00000100611	0	0	0	0	0	0	0	0	0	0
ENSMUSG00000100616	0	0	0	0	0	0	0	0	0	0
ENSMUSG00000100617	0	0	0	0	0	0	0	0	0	0
ENSMUSG00000100614	0	0	0	0	0	0	0	0	0	0
ENSMUSG00000100618	0	0	0	0	0	3	0	0	0	0
ENSMUSG00000100619	0	0	0	0	0	0	0	0	0	0
ENSMUSG00000064748	0	0	0	0	0	0	0	0	0	0
ENSMUSG00000107341	0	0	0	2	0	0	1	0	0	0
ENSMUSG00000099049	0	0	0	0	0	0	0	0	0	0
ENSMUSG00000107344	0	0	0	0	0	0	0	0	0	0
ENSMUSG00000107346	0	0	0	0	0	0	0	0	0	0
ENSMUSG00000107349	0	0	3	0	0	0	0	0	0	0
ENSMUSG00000107348	0	0	0	0	0	0	0	0	0	0
ENSMUSG00000100818	0	0	0	0	0	0	0	0	0	0
ENSMUSG00000000402	3	0	12	12	15	9	15	12	0	6
ENSMUSG00000100819	11	6	8	13	13	10	4	14	11	13
ENSMUSG00000000409	29	64	38	71	30	77	187	97	176	36
ENSMUSG00000113114	0	0	0	0	2	0	2	0	0	0
ENSMUSG00000102080	0	0	1	0	0	4	3	3	3	0

ENSMUSG00000085450	0	0	0	0	0	0	0	0	0	0
ENSMUSG00000085451	3	0	3	0	0	0	0	0	0	0
ENSMUSG00000085453	0	0	0	0	0	0	0	0	0	0
ENSMUSG00000114532	0	0	0	0	0	0	0	0	0	0
ENSMUSG00000114533	0	0	0	0	0	0	0	0	0	0
ENSMUSG00000100812	0	0	0	0	0	0	0	0	0	0
ENSMUSG00000079031	0	0	0	0	0	0	0	0	0	0
ENSMUSG00000114531	0	3	6	0	3	0	5	0	0	0
ENSMUSG00000065533	0	0	0	0	0	0	0	0	0	0
ENSMUSG00000100814	0	0	0	0	0	0	0	0	0	0
ENSMUSG00000100816	0	0	0	0	0	0	0	0	0	0
ENSMUSG00000101633	0	0	0	0	0	0	0	0	0	0
ENSMUSG00000038676	33	21	19	42	25	27	31	35	38	28
ENSMUSG00000069044	3	2	0	0	0	0	0	3	61	0
ENSMUSG00000069041	32	27	22	9	6	40	19	110	3	32
ENSMUSG00000021464	101	119	104	151	92	136	246	140	184	144
ENSMUSG00000021469	43	42	43	20	33	34	19	16	15	25
ENSMUSG00000105752	0	0	0	0	0	0	0	0	2	0
ENSMUSG00000096193	0	0	0	0	0	0	0	0	0	0
ENSMUSG00000113115	0	0	0	0	0	0	0	0	0	0
ENSMUSG00000113116	0	0	0	0	0	0	0	0	0	0
ENSMUSG00000038331	24	28	6	26	12	39	17	20	38	40
ENSMUSG00000038330	0	0	0	0	0	0	0	0	0	0
ENSMUSG00000113112	0	0	0	0	0	0	0	0	0	0
ENSMUSG00000059279	3	0	0	0	0	7	5	3	2	10
ENSMUSG00000015443	0	0	0	0	0	0	0	0	0	0
ENSMUSG00000063713	0	0	0	0	0	0	0	0	0	0
ENSMUSG00000063715	0	0	0	0	0	0	0	0	0	0
ENSMUSG00000029674	353	436	481	513	459	486	672	480	557	488
ENSMUSG00000029673	384	395	419	379	278	332	368	451	389	382
ENSMUSG00000029671	55	46	21	0	22	41	24	26	28	14
ENSMUSG00000029678	0	0	0	3	0	0	0	3	3	2
ENSMUSG00000029679	0	6	0	0	0	0	0	6	5	0
ENSMUSG00000106762	0	0	0	0	0	0	0	0	0	0
ENSMUSG00000106763	3	0	0	6	0	3	1	0	0	3
ENSMUSG00000106764	1	2	0	0	0	0	0	2	0	2
ENSMUSG00000106768	0	0	0	0	0	3	0	0	0	0
ENSMUSG00000038591	3	0	0	0	0	6	29	6	0	0
ENSMUSG00000104722	0	0	0	0	0	0	0	0	0	0
ENSMUSG00000038598	0	0	0	3	0	3	0	3	0	0
ENSMUSG00000104726	0	0	0	0	0	0	0	0	0	0
ENSMUSG00000104727	3	0	0	0	0	3	0	0	0	0
ENSMUSG00000104725	0	3	0	3	0	0	0	0	0	0
ENSMUSG00000059187	0	0	0	0	0	3	0	3	6	9
ENSMUSG00000059189	0	0	0	0	0	0	0	0	0	0
ENSMUSG00000024173	11	6	11	8	8	18	40	8	6	13
ENSMUSG00000024171	0	0	0	0	0	0	0	0	0	0
ENSMUSG00000024176	40	18	57	20	33	11	99	79	79	65
ENSMUSG00000024174	492	373	397	448	271	344	572	500	564	397
ENSMUSG00000024175	12	3	0	10	9	20	23	21	0	12
ENSMUSG00000114464	0	0	0	0	0	0	0	0	0	0
ENSMUSG00000114465	0	0	0	0	0	0	0	0	0	0
ENSMUSG00000114466	0	0	0	0	0	0	0	0	0	0
ENSMUSG00000114460	0	0	0	0	0	0	0	0	0	0
ENSMUSG00000114461	0	0	0	0	0	0	0	0	0	0
ENSMUSG00000114462	0	0	0	0	0	0	0	0	0	0
ENSMUSG00000054258	0	2	0	6	4	0	0	0	6	0
ENSMUSG00000097794	0	0	0	0	0	0	0	0	0	0

ENSMUSG00000097795	0	5	0	12	0	4	6	0	0	0
ENSMUSG00000054256	20	8	0	6	11	14	32	17	11	9
ENSMUSG00000097797	6	4	11	1	9	0	24	4	11	3
ENSMUSG00000091827	0	0	0	0	3	0	0	0	0	0
ENSMUSG00000091821	0	0	0	0	0	0	0	0	0	0
ENSMUSG00000109750	0	9	0	3	3	0	6	2	0	0
ENSMUSG00000109752	0	0	0	0	0	0	0	0	0	0
ENSMUSG00000109753	0	0	0	0	0	0	0	0	0	0
ENSMUSG00000109756	0	3	2	12	9	0	3	0	0	0
ENSMUSG00000109757	0	0	0	0	0	0	0	0	0	0
ENSMUSG00000105237	0	0	0	0	0	0	0	0	0	0
ENSMUSG00000109759	0	0	0	0	0	0	3	0	0	0
ENSMUSG00000105235	0	0	0	0	0	0	0	0	0	0
ENSMUSG00000105234	0	0	0	0	0	0	0	0	0	0
ENSMUSG00000108933	0	0	0	0	0	0	0	0	0	0
ENSMUSG00000020419	0	13	0	0	3	5	0	8	6	6
ENSMUSG00000008153	28	11	28	9	3	30	124	46	28	33
ENSMUSG00000058631	0	0	0	0	0	0	0	0	0	0
ENSMUSG00000108409	0	0	0	0	0	0	0	0	0	0
ENSMUSG00000104186	0	0	0	0	0	0	0	0	0	0
ENSMUSG00000108406	0	0	0	0	0	0	0	0	0	0
ENSMUSG00000104185	0	0	0	0	0	0	0	0	0	0
ENSMUSG00000104182	0	0	0	0	0	0	0	0	0	0
ENSMUSG00000104183	0	0	3	6	0	6	15	8	7	9
ENSMUSG00000104180	0	0	0	0	0	0	0	0	0	0
ENSMUSG00000108400	0	0	0	0	0	0	0	0	0	0
ENSMUSG00000081296	0	0	0	0	0	0	0	0	0	0
ENSMUSG00000081297	0	0	0	0	0	0	0	0	0	0
ENSMUSG00000043673	32	33	47	28	49	80	241	58	67	79
ENSMUSG00000091794	0	0	0	0	3	0	0	0	0	0
ENSMUSG00000086135	0	0	0	0	0	0	0	0	0	0
ENSMUSG00000091792	0	0	0	0	0	0	0	0	0	0
ENSMUSG00000026009	23	10	6	9	18	29	62	52	59	44
ENSMUSG00000096447	0	0	0	0	0	0	0	0	0	0
ENSMUSG00000056782	0	0	0	0	0	0	0	0	0	0
ENSMUSG00000095239	0	0	0	0	0	0	0	0	0	0
ENSMUSG00000095238	0	0	0	0	0	0	0	0	0	0
ENSMUSG00000045005	538	620	649	599	488	450	745	482	507	494
ENSMUSG00000095234	0	0	4	0	0	0	11	5	7	0
ENSMUSG00000098825	0	0	0	1	1	3	2	0	1	2
ENSMUSG00000095232	0	0	0	0	0	0	0	0	0	0
ENSMUSG00000111236	0	0	0	0	0	0	0	0	0	0
ENSMUSG00000111234	0	0	0	0	0	0	0	0	0	0
ENSMUSG00000111235	0	0	0	0	0	0	0	0	0	0
ENSMUSG00000111232	0	0	0	0	0	0	0	0	0	0
ENSMUSG00000111233	0	3	8	0	0	0	3	0	6	0
ENSMUSG00000010601	14	9	4	19	9	5	32	17	16	3
ENSMUSG00000093942	0	0	1	0	3	3	7	0	3	0
ENSMUSG00000047535	0	0	0	0	0	0	0	0	0	0
ENSMUSG00000047534	90	128	138	120	109	72	113	122	73	82
ENSMUSG00000047531	0	0	0	0	0	0	0	0	0	0
ENSMUSG00000093948	0	0	0	0	0	0	0	0	0	0
ENSMUSG00000095491	0	0	0	0	0	0	0	0	0	0
ENSMUSG00000095490	0	0	0	0	0	0	0	0	0	0
ENSMUSG00000095499	0	0	0	0	0	1	0	0	1	0
ENSMUSG00000090466	0	0	0	0	0	0	0	0	0	0
ENSMUSG00000090465	0	0	0	0	0	0	0	0	0	0
ENSMUSG00000090462	0	0	0	0	0	0	0	0	0	0

ENSMUSG00000090461	0	0	0	0	0	0	0	0	0	0
ENSMUSG00000081724	0	0	0	0	0	0	0	3	0	0
ENSMUSG00000026873	129	132	147	179	127	127	199	193	94	142
ENSMUSG00000081721	10	6	10	16	9	6	7	8	6	12
ENSMUSG00000022101	3	5	0	3	3	0	5	3	3	0
ENSMUSG00000022103	97	134	77	92	78	118	192	121	66	104
ENSMUSG00000094548	0	0	0	0	0	0	1	0	0	0
ENSMUSG00000108876	0	0	0	0	2	0	0	0	0	0
ENSMUSG00000094545	0	0	0	0	0	0	0	0	0	0
ENSMUSG00000094540	0	0	0	0	0	0	0	0	0	0
ENSMUSG00000094542	0	0	0	0	0	0	0	0	0	0
ENSMUSG00000094543	0	0	0	0	0	0	0	0	0	0
ENSMUSG00000056128	0	0	0	0	0	0	0	3	0	0
ENSMUSG00000044847	638	710	679	677	564	639	787	636	725	755
ENSMUSG00000088310	0	0	0	0	0	0	0	0	0	0
ENSMUSG00000097899	7	0	2	24	4	14	3	9	8	13
ENSMUSG00000097898	0	0	0	0	0	0	0	0	0	0
ENSMUSG00000088311	0	0	0	0	0	0	0	0	0	0
ENSMUSG00000028264	5	11	0	3	8	9	41	19	15	9
ENSMUSG00000028262	24	17	3	3	24	37	50	65	57	32
ENSMUSG00000097896	0	0	0	0	0	0	1	0	0	0
ENSMUSG00000088317	0	0	0	0	0	0	0	0	0	0
ENSMUSG00000091970	0	0	0	0	0	0	0	0	0	0
ENSMUSG00000077640	0	0	0	0	0	0	0	0	0	0
ENSMUSG00000077641	0	0	0	0	0	0	0	0	0	0
ENSMUSG00000077642	0	0	0	0	0	0	0	0	0	0
ENSMUSG00000077643	0	0	0	0	0	0	0	0	0	0
ENSMUSG00000077644	0	0	0	0	0	0	0	0	0	0
ENSMUSG00000077645	0	0	0	0	0	0	0	0	0	0
ENSMUSG00000083501	0	0	0	0	0	0	0	0	0	0
ENSMUSG00000077648	0	0	0	0	0	0	0	2	0	0
ENSMUSG00000084283	0	0	0	0	0	0	0	0	0	0
ENSMUSG00000112171	0	0	0	0	0	0	0	0	0	0
ENSMUSG00000089182	0	0	0	0	0	0	0	0	0	0
ENSMUSG00000086189	0	6	11	11	3	0	6	0	3	0
ENSMUSG00000086188	31	29	22	9	11	23	6	26	43	27
ENSMUSG00000086187	0	6	10	2	3	0	3	3	0	6
ENSMUSG00000086185	0	0	0	0	0	0	0	0	0	0
ENSMUSG00000076118	0	0	0	0	0	0	0	0	0	0
ENSMUSG00000089183	0	0	0	0	0	0	0	0	0	0
ENSMUSG00000102456	0	0	0	0	0	0	0	0	0	0
ENSMUSG00000098695	0	0	0	0	0	0	0	0	0	0
ENSMUSG00000045824	0	0	0	0	0	0	0	0	0	0
ENSMUSG00000049191	225	281	105	255	203	72	483	326	344	155
ENSMUSG00000105943	0	0	0	0	0	0	0	0	0	0
ENSMUSG00000098691	0	0	0	0	0	0	0	0	0	1
ENSMUSG00000096854	5	10	10	10	4	6	22	15	9	17
ENSMUSG00000096856	378	500	441	490	371	379	515	440	358	413
ENSMUSG00000096850	28	27	11	25	31	49	25	19	31	18
ENSMUSG00000096851	0	0	0	0	0	0	0	0	0	0
ENSMUSG00000098876	0	0	0	0	0	0	0	0	0	0
ENSMUSG00000098877	0	0	0	0	0	0	0	0	0	0
ENSMUSG00000108083	0	0	0	0	0	0	0	0	0	0
ENSMUSG00000096858	0	0	0	0	0	0	0	0	0	0
ENSMUSG00000096859	0	0	0	0	0	0	0	0	0	0
ENSMUSG00000106245	0	0	0	0	0	3	0	0	0	0
ENSMUSG00000106243	0	0	0	0	0	0	0	0	6	0
ENSMUSG00000113077	0	0	0	0	0	0	0	0	0	0

ENSMUSG00000106248	0	0	0	0	0	0	0	0	0	0
ENSMUSG00000022097	0	0	0	0	0	801	0	0	8	0
ENSMUSG00000047094	0	0	0	3	0	0	0	0	0	3
ENSMUSG00000088824	0	0	0	0	0	0	0	0	0	0
ENSMUSG00000004341	3	0	0	0	0	0	0	0	0	0
ENSMUSG00000088825	0	0	0	0	0	0	0	0	0	0
ENSMUSG00000004344	0	0	0	0	0	0	0	0	0	0
ENSMUSG00000109417	0	0	0	0	0	0	0	0	0	0
ENSMUSG00000109415	0	0	0	0	0	0	0	0	0	0
ENSMUSG00000109414	0	0	0	0	0	0	0	0	0	0
ENSMUSG00000109413	0	0	0	0	0	0	0	0	0	0
ENSMUSG00000109412	0	0	0	0	0	0	0	0	0	0
ENSMUSG00000109411	0	0	0	0	0	0	0	0	0	0
ENSMUSG00000109410	0	0	0	0	0	0	0	0	0	0
ENSMUSG00000109419	84	95	87	66	48	62	53	100	79	90
ENSMUSG00000109418	0	0	0	0	0	0	0	0	0	0
ENSMUSG00000082406	0	0	0	0	0	0	0	0	0	0
ENSMUSG00000082407	0	0	0	0	0	0	0	0	0	0
ENSMUSG00000082404	0	0	0	0	0	0	0	2	0	0
ENSMUSG00000082405	0	0	0	0	0	0	0	0	0	0
ENSMUSG00000082403	0	0	0	0	0	0	0	0	0	0
ENSMUSG00000070870	0	0	0	0	0	0	0	0	0	0
ENSMUSG00000070877	0	0	0	6	0	5	2	4	6	5
ENSMUSG00000082408	0	0	0	0	0	0	0	0	3	0
ENSMUSG00000070875	0	0	0	0	0	0	0	0	0	0
ENSMUSG00000005696	18	7	7	14	6	12	35	10	44	4
ENSMUSG00000040373	0	0	3	0	0	0	0	0	0	3
ENSMUSG00000084123	0	0	0	0	0	0	0	0	0	0
ENSMUSG00000084120	0	0	0	0	0	0	0	0	0	0
ENSMUSG00000084124	0	2	0	0	0	0	0	0	0	0
ENSMUSG00000087037	0	0	0	0	0	0	0	0	0	0
ENSMUSG00000103161	3	0	12	3	3	3	8	0	6	9
ENSMUSG00000080800	1	0	0	0	0	0	0	0	0	0
ENSMUSG00000080802	0	0	0	0	0	0	0	0	0	0
ENSMUSG00000080803	0	0	0	0	0	0	0	0	0	0
ENSMUSG00000080805	0	0	0	0	0	0	0	0	0	0
ENSMUSG00000080806	0	0	0	0	0	0	0	0	0	0
ENSMUSG00000080807	0	3	0	0	0	6	0	1	3	0
ENSMUSG00000080808	0	0	0	0	0	3	4	0	12	0
ENSMUSG00000080809	0	0	0	0	0	0	0	0	0	0
ENSMUSG00000099006	0	0	0	0	0	0	1	0	0	0
ENSMUSG00000099000	0	0	0	0	0	0	0	0	0	0
ENSMUSG00000099009	27	5	3	11	3	29	31	6	0	12
ENSMUSG00000022099	459	490	505	524	396	479	714	466	720	476
ENSMUSG00000041020	89	113	140	106	130	214	326	157	143	106
ENSMUSG00000082778	10	12	14	17	21	16	9	10	7	16
ENSMUSG00000044014	0	0	0	0	0	0	0	0	0	0
ENSMUSG00000104014	0	0	0	0	0	0	0	0	0	0
ENSMUSG00000085292	0	0	0	0	0	0	0	0	0	0
ENSMUSG00000085293	0	0	0	0	0	0	0	0	0	0
ENSMUSG00000085290	0	0	0	0	0	0	0	0	0	0
ENSMUSG00000101065	0	2	0	0	0	0	0	0	0	0
ENSMUSG00000109569	0	0	0	0	0	0	0	0	0	0
ENSMUSG00000085295	4	6	6	8	5	6	5	0	3	0
ENSMUSG00000111508	0	0	0	0	0	0	0	0	0	0
ENSMUSG00000062608	0	0	0	0	0	0	0	0	0	0
ENSMUSG00000109566	0	0	0	0	0	0	0	0	0	0
ENSMUSG00000104013	0	0	0	0	0	0	0	0	0	0

ENSMUSG00000079235	3	0	8	3	0	6	9	3	3	8
ENSMUSG00000074404	0	0	0	0	0	0	0	0	0	0
ENSMUSG00000078182	0	0	0	0	0	0	0	0	0	0
ENSMUSG00000074402	0	0	0	0	0	0	0	0	0	0
ENSMUSG00000074401	0	0	0	0	0	0	0	0	0	0
ENSMUSG00000114087	0	0	0	0	0	0	0	0	0	0
ENSMUSG00000033952	379	255	393	379	270	204	416	220	194	186
ENSMUSG00000073759	0	0	12	0	0	14	17	12	10	24
ENSMUSG00000071150	0	0	0	0	0	0	0	0	0	0
ENSMUSG00000084192	3	0	11	0	3	2	0	4	5	3
ENSMUSG00000077981	0	0	0	0	0	0	0	0	0	0
ENSMUSG00000077980	2	1	0	2	0	1	3	0	2	2
ENSMUSG00000111018	0	0	0	0	0	0	0	0	0	0
ENSMUSG00000106624	0	0	0	0	0	0	0	0	0	0
ENSMUSG00000099914	0	0	0	0	0	0	0	0	0	0
ENSMUSG00000099915	0	0	0	0	0	0	0	0	0	0
ENSMUSG00000099916	0	0	0	0	0	0	0	0	0	0
ENSMUSG00000099910	0	0	0	0	0	0	0	0	0	0
ENSMUSG00000106627	0	0	0	0	6	2	0	0	0	0
ENSMUSG00000099912	0	0	0	0	0	0	0	0	0	0
ENSMUSG00000099951	0	0	0	0	0	0	0	0	0	0
ENSMUSG00000099955	0	0	0	0	0	0	0	0	0	0
ENSMUSG00000099957	0	0	0	0	0	0	0	0	0	0
ENSMUSG00000031085	0	0	0	0	0	0	0	0	0	0
ENSMUSG00000031089	21	17	6	6	3	35	8	36	6	18
ENSMUSG00000074446	0	0	0	0	0	0	0	0	0	0
ENSMUSG00000103487	0	0	0	0	0	0	0	0	0	0
ENSMUSG00000048456	0	0	0	0	0	0	0	0	0	0
ENSMUSG00000048455	0	0	0	0	0	0	0	0	0	0
ENSMUSG00000038860	237	272	248	232	156	275	372	321	282	291
ENSMUSG00000049815	0	0	0	0	0	0	0	0	0	0
ENSMUSG00000077327	0	0	0	0	0	0	0	0	0	0
ENSMUSG00000077325	0	0	0	0	0	0	0	0	0	0
ENSMUSG00000077320	0	0	0	0	0	0	0	0	0	0
ENSMUSG00000077328	0	0	0	0	0	2	0	0	0	0
ENSMUSG00000096019	0	0	0	0	0	0	0	0	0	0
ENSMUSG00000084913	0	0	0	0	0	0	0	0	0	0
ENSMUSG00000104668	0	0	0	2	0	0	0	0	6	0
ENSMUSG00000093851	0	0	0	0	0	0	0	0	0	0
ENSMUSG00000031621	0	0	3	0	6	0	3	0	0	3
ENSMUSG00000103482	0	0	0	0	0	0	0	0	0	0
ENSMUSG00000102933	0	0	0	0	0	0	0	0	0	0
ENSMUSG00000102932	0	0	0	0	0	0	0	0	0	0
ENSMUSG00000102934	0	0	0	0	0	0	0	0	0	0
ENSMUSG00000102937	0	0	0	0	0	0	3	0	0	0
ENSMUSG00000102936	0	0	0	0	0	0	0	0	0	0
ENSMUSG00000071860	6	3	32	12	3	1	23	20	30	9
ENSMUSG00000015653	133	164	196	163	160	265	733	262	450	345
ENSMUSG00000015652	3	3	6	0	0	9	14	5	12	24
ENSMUSG00000043569	0	0	0	0	0	0	0	0	0	0
ENSMUSG00000087339	6	8	2	8	3	3	0	0	0	0
ENSMUSG00000087333	15	6	21	0	15	32	34	28	39	6
ENSMUSG00000087332	0	0	0	0	0	0	0	0	0	0
ENSMUSG00000088103	0	0	0	0	0	0	1	0	2	0
ENSMUSG00000088102	0	0	0	0	0	0	0	1	0	0
ENSMUSG00000088105	0	0	0	0	0	0	0	0	0	0
ENSMUSG00000087335	0	0	0	0	0	0	0	0	0	0
ENSMUSG00000087334	0	0	0	0	0	4	0	0	0	0

ENSMUSG00000068818	0	0	0	3	0	0	0	0	0	0
ENSMUSG00000096598	0	0	0	0	0	0	0	0	0	0
ENSMUSG00000100795	0	0	0	0	0	0	0	0	0	0
ENSMUSG00000100790	0	0	0	0	0	0	0	0	0	0
ENSMUSG00000082243	0	0	0	0	0	0	0	0	0	0
ENSMUSG00000100798	0	0	0	0	0	0	0	0	0	9
ENSMUSG00000040035	0	15	0	6	0	6	20	0	12	9
ENSMUSG00000101777	0	0	0	0	0	0	0	0	0	0
ENSMUSG00000101774	0	0	0	0	0	0	0	0	0	0
ENSMUSG00000101775	0	0	0	0	0	0	0	0	0	0
ENSMUSG00000101779	0	0	0	0	0	0	0	0	0	0
ENSMUSG00000059845	0	0	0	0	0	0	0	0	0	0
ENSMUSG00000113884	0	0	0	0	0	0	0	0	0	0
ENSMUSG00000093859	0	0	0	0	0	0	0	0	0	3
ENSMUSG00000103688	0	0	0	2	0	0	0	0	0	0
ENSMUSG00000103689	0	0	0	0	0	1	0	0	0	0
ENSMUSG00000103684	0	0	0	0	0	0	3	0	0	0
ENSMUSG00000103685	12	6	9	3	15	6	20	3	7	6
ENSMUSG00000103686	3	10	0	16	6	7	14	3	3	1
ENSMUSG00000103681	0	0	0	0	0	0	0	0	0	0
ENSMUSG00000100420	0	0	0	0	0	0	0	0	0	0
ENSMUSG00000100425	0	0	0	0	0	0	0	0	0	0
ENSMUSG00000100424	0	0	0	0	0	0	0	0	0	0
ENSMUSG00000100426	0	0	5	0	0	3	0	3	0	3
ENSMUSG00000012443	372	290	422	402	376	208	507	316	273	213
ENSMUSG00000022347	24	5	19	6	3	11	8	21	23	11
ENSMUSG00000022346	131	114	106	192	101	236	349	201	170	241
ENSMUSG00000022342	3	0	0	0	0	0	0	0	3	0
ENSMUSG00000020279	8	0	6	6	0	9	61	28	48	36
ENSMUSG00000095968	0	0	0	0	0	0	0	0	0	0
ENSMUSG00000089760	0	0	0	0	0	0	0	0	3	0
ENSMUSG00000089761	0	0	0	0	0	0	0	0	0	0
ENSMUSG00000089767	0	0	0	0	0	0	0	0	0	0
ENSMUSG00000095961	0	0	0	0	0	0	0	0	0	0
ENSMUSG00000020270	0	0	0	0	0	0	0	0	0	0
ENSMUSG00000095963	0	0	0	0	0	0	0	0	0	1
ENSMUSG00000095962	0	0	0	0	0	0	0	0	0	0
ENSMUSG00000095965	0	0	0	0	0	0	0	0	0	0
ENSMUSG00000095964	0	0	0	0	0	0	0	0	0	0
ENSMUSG00000075416	9	17	12	22	11	9	27	28	33	11
ENSMUSG00000065469	0	0	0	0	0	0	0	0	0	0
ENSMUSG00000028068	197	154	205	244	142	95	465	193	123	115
ENSMUSG00000028064	489	544	498	826	401	522	1002	641	559	521
ENSMUSG00000102481	0	0	0	0	0	0	0	0	0	0
ENSMUSG00000102480	0	0	0	0	0	0	0	0	0	0
ENSMUSG00000102483	0	0	0	0	0	0	0	0	0	0
ENSMUSG00000102482	0	0	0	0	0	0	0	0	0	0
ENSMUSG00000102485	0	0	0	0	0	0	0	0	0	0
ENSMUSG00000102484	0	0	0	0	0	0	0	0	0	0
ENSMUSG00000102487	0	0	0	0	0	0	0	0	0	0
ENSMUSG00000102486	0	0	0	0	0	0	0	0	0	0
ENSMUSG00000102489	0	0	0	0	0	0	0	0	0	0
ENSMUSG00000102488	0	0	0	0	0	0	0	0	0	0
ENSMUSG00000024694	27	0	39	78	0	0	17	0	0	25
ENSMUSG00000020182	176	214	152	173	177	115	55	171	61	112
ENSMUSG00000020185	101	139	118	167	78	122	171	107	120	148
ENSMUSG00000102159	0	0	0	0	0	0	0	0	0	0
ENSMUSG00000102158	4	3	0	8	9	0	13	9	7	0

ENSMUSG00000112479	0	0	0	0	0	0	0	0	0	0
ENSMUSG00000112478	0	14	10	0	18	10	9	7	14	12
ENSMUSG00000112475	0	0	0	0	0	0	0	0	0	0
ENSMUSG00000102152	17	7	19	14	12	18	12	6	8	8
ENSMUSG00000112477	0	0	0	0	0	0	0	0	0	0
ENSMUSG00000039296	0	0	0	0	0	0	0	0	0	0
ENSMUSG00000112470	0	0	0	0	0	0	0	0	0	0
ENSMUSG00000102155	0	3	0	7	0	0	0	0	0	2
ENSMUSG00000110940	0	0	0	0	0	0	0	0	0	0
ENSMUSG00000088688	0	0	0	0	0	0	0	0	0	0
ENSMUSG00000088686	0	0	0	0	0	0	0	0	0	0
ENSMUSG00000088687	0	0	0	0	0	0	0	0	0	0
ENSMUSG00000088684	0	0	0	0	0	0	0	0	0	0
ENSMUSG00000088680	0	0	0	0	0	0	0	0	0	0
ENSMUSG00000021587	13	38	63	23	38	18	35	36	48	49
ENSMUSG00000105688	0	0	0	0	0	0	0	0	0	0
ENSMUSG00000105689	0	0	0	0	1	0	4	3	0	0
ENSMUSG00000100144	0	0	0	0	0	0	0	0	0	0
ENSMUSG00000105682	0	0	0	0	0	0	0	0	0	0
ENSMUSG00000105683	0	0	0	0	0	0	0	0	0	0
ENSMUSG00000021638	84	139	160	180	51	256	269	226	229	153
ENSMUSG00000105687	0	0	2	2	0	0	2	0	0	0
ENSMUSG00000105684	0	0	0	0	0	0	0	0	0	0
ENSMUSG00000105685	0	0	0	0	0	0	0	0	0	0
ENSMUSG00000024034	0	0	0	0	0	0	0	0	0	0
ENSMUSG00000024033	0	9	24	15	12	21	42	58	49	41
ENSMUSG00000113997	0	0	0	0	0	0	0	0	0	0
ENSMUSG00000024039	30	64	56	54	66	62	95	58	43	73
ENSMUSG00000106485	0	0	0	0	0	0	0	0	0	0
ENSMUSG00000106487	0	0	0	0	0	0	0	0	0	0
ENSMUSG00000106486	0	0	0	0	0	0	0	0	0	0
ENSMUSG00000106481	0	0	0	0	0	2	0	0	0	0
ENSMUSG00000113709	0	0	0	0	0	0	0	0	0	0
ENSMUSG00000106483	0	0	0	0	0	0	0	0	0	0
ENSMUSG00000113704	0	0	0	0	0	0	0	0	0	0
ENSMUSG00000113705	0	0	0	0	0	0	0	0	0	0
ENSMUSG00000088972	0	0	0	0	0	0	0	0	0	0
ENSMUSG00000113707	0	0	0	0	0	0	0	0	0	2
ENSMUSG00000113700	0	0	0	0	0	0	0	0	0	0
ENSMUSG00000106488	0	0	1	0	0	0	1	0	0	0
ENSMUSG00000088976	7	0	3	0	3	0	0	0	0	0
ENSMUSG00000113703	0	0	0	0	0	0	0	0	0	0
ENSMUSG00000078776	0	5	3	7	0	11	0	9	9	5
ENSMUSG00000092167	4	13	8	14	12	21	39	25	18	0
ENSMUSG00000111416	1	0	0	0	0	0	0	0	1	0
ENSMUSG00000111417	3	0	0	0	0	0	0	0	2	0
ENSMUSG00000111413	0	0	0	0	0	0	0	0	0	0
ENSMUSG00000111411	0	0	0	0	0	0	0	0	0	0
ENSMUSG00000111418	0	0	0	0	0	0	0	0	0	0
ENSMUSG00000111419	0	0	0	0	0	0	0	0	0	0
ENSMUSG00000108300	0	0	0	0	0	0	0	0	0	0
ENSMUSG00000062651	0	0	0	0	0	0	0	0	0	0
ENSMUSG00000108302	0	0	0	0	0	0	0	0	0	0
ENSMUSG00000108304	0	0	0	0	0	4	0	0	0	0
ENSMUSG00000108306	0	0	0	0	0	0	0	0	0	0
ENSMUSG00000108307	0	2	0	0	0	0	0	0	0	0
ENSMUSG00000108308	11	8	0	0	6	6	8	0	12	0
ENSMUSG00000059022	108	114	192	81	99	151	122	116	73	153

ENSMUSG00000059023	0	0	0	0	0	0	0	0	0	0
ENSMUSG00000056078	0	0	0	0	0	0	0	0	0	0
ENSMUSG00000059027	86	92	126	135	91	126	264	131	144	118
ENSMUSG00000111288	0	0	0	0	0	0	3	0	0	0
ENSMUSG00000009900	0	0	0	0	0	0	0	0	0	0
ENSMUSG00000067285	0	0	0	0	0	0	0	0	0	0
ENSMUSG00000097612	3	8	0	2	1	11	4	0	4	3
ENSMUSG00000097615	0	0	0	0	0	0	0	0	0	0
ENSMUSG00000097618	2	2	3	5	0	2	1	2	7	0
ENSMUSG00000091618	0	0	0	0	0	3	8	3	0	0
ENSMUSG00000105850	6	7	0	7	6	4	8	9	12	5
ENSMUSG00000099412	0	0	0	0	0	0	0	0	0	0
ENSMUSG00000095508	0	0	0	0	0	0	0	0	0	0
ENSMUSG00000091617	11	19	17	21	0	28	10	20	23	15
ENSMUSG00000091615	0	0	0	0	0	0	0	0	0	0
ENSMUSG00000091614	0	0	0	0	0	0	0	0	0	0
ENSMUSG00000095507	0	0	0	0	0	0	0	0	0	0
ENSMUSG00000070890	0	0	0	0	0	0	0	0	0	0
ENSMUSG00000067750	0	0	6	3	0	0	0	0	6	0
ENSMUSG00000033981	0	0	0	0	6	0	2	3	3	0
ENSMUSG00000070891	14	14	2	9	6	12	8	6	16	6
ENSMUSG00000114164	0	0	0	0	0	0	0	0	0	0
ENSMUSG00000114167	0	0	0	0	0	0	0	0	0	0
ENSMUSG00000114166	0	0	0	0	0	0	0	0	0	0
ENSMUSG00000114161	0	0	0	0	0	0	0	0	0	0
ENSMUSG00000114163	0	3	0	3	0	0	0	0	0	0
ENSMUSG00000114162	0	0	0	0	0	0	0	0	0	0
ENSMUSG00000044254	30	17	12	15	14	50	15	25	20	24
ENSMUSG00000090750	0	0	0	0	0	0	0	0	0	0
ENSMUSG00000054422	0	0	0	3	0	6	0	0	0	6
ENSMUSG00000027670	6	3	0	3	3	9	3	0	0	8
ENSMUSG00000050215	0	0	0	0	0	0	3	6	6	0
ENSMUSG00000027674	118	97	101	92	78	132	151	73	129	136
ENSMUSG00000110759	0	0	0	0	0	0	0	0	0	0
ENSMUSG00000104086	0	0	0	0	0	0	0	0	0	0
ENSMUSG00000110429	0	0	0	0	0	0	0	0	0	0
ENSMUSG00000110425	0	0	0	0	0	0	0	0	0	0
ENSMUSG00000110426	0	0	0	0	0	2	0	0	2	0
ENSMUSG00000110427	0	0	0	0	0	0	0	0	0	0
ENSMUSG00000110420	0	0	0	0	0	0	0	0	0	0
ENSMUSG00000110421	0	0	0	0	0	0	0	0	0	0
ENSMUSG00000080358	0	0	0	0	1	0	1	0	2	0
ENSMUSG00000080359	0	0	0	0	0	0	0	0	0	0
ENSMUSG00000080356	3	0	0	0	1	1	3	3	1	0
ENSMUSG00000104080	3	0	0	0	0	0	3	0	0	0
ENSMUSG00000080350	0	0	0	0	0	0	1	0	0	0
ENSMUSG00000046095	0	0	0	0	0	0	0	0	0	0
ENSMUSG00000001864	180	267	204	187	93	217	205	269	201	195
ENSMUSG00000046093	0	3	18	6	9	6	0	15	0	25
ENSMUSG00000114908	0	0	0	0	0	0	0	0	0	0
ENSMUSG00000113414	0	0	0	0	0	0	0	0	0	0
ENSMUSG00000080686	0	0	0	0	2	0	4	0	0	1
ENSMUSG00000080687	0	3	0	0	3	0	2	2	2	4
ENSMUSG00000106331	0	0	0	0	0	0	0	0	0	0
ENSMUSG00000080680	0	0	0	0	0	0	0	0	0	0
ENSMUSG00000090059	0	0	0	0	0	0	0	0	3	0
ENSMUSG00000094810	0	0	0	0	0	0	0	0	0	0
ENSMUSG00000090051	0	0	0	0	0	0	0	0	0	0

ENSMUSG00000090057	0	2	0	0	3	0	0	0	0	0
ENSMUSG00000113578	0	0	0	0	0	0	0	0	0	0
ENSMUSG00000098229	0	0	0	0	0	0	0	0	0	0
ENSMUSG00000098228	0	0	0	0	0	0	0	0	0	0
ENSMUSG00000098227	0	0	0	0	0	0	0	0	0	0
ENSMUSG00000093509	0	0	0	0	0	0	0	0	0	0
ENSMUSG00000094739	0	0	0	0	0	0	0	0	0	0
ENSMUSG00000094738	0	0	0	0	0	0	0	0	0	0
ENSMUSG00000042345	18	6	15	28	21	20	52	52	54	12
ENSMUSG00000042349	185	196	151	230	157	285	478	248	373	222
ENSMUSG00000094732	0	0	0	0	0	0	0	0	0	0
ENSMUSG00000094730	0	0	0	0	0	0	0	0	0	0
ENSMUSG00000093504	0	0	0	0	0	0	0	0	0	0
ENSMUSG00000093507	4	14	18	10	0	1	0	4	5	9
ENSMUSG00000094734	0	0	0	0	0	0	0	0	0	0
ENSMUSG00000112095	24	18	35	52	12	36	66	23	57	27
ENSMUSG00000112094	0	0	0	0	0	0	0	0	0	0
ENSMUSG00000113576	0	0	2	0	0	0	0	0	0	0
ENSMUSG00000052217	0	0	0	0	0	0	0	0	0	0
ENSMUSG00000081089	0	0	0	0	0	0	0	0	0	0
ENSMUSG00000081088	0	0	0	0	0	0	0	0	0	0
ENSMUSG00000052212	3	6	0	93	20	29	3	9	18	3
ENSMUSG00000081083	0	0	0	0	0	0	2	0	0	0
ENSMUSG00000081082	0	0	0	0	0	0	0	0	0	0
ENSMUSG00000081081	0	0	0	0	2	0	0	0	0	0
ENSMUSG00000081080	0	0	0	0	0	0	0	0	0	0
ENSMUSG00000081084	0	0	0	0	0	0	0	0	0	0
ENSMUSG00000081555	0	0	0	0	0	0	0	0	0	0
ENSMUSG00000081557	0	0	0	0	0	0	0	2	0	0
ENSMUSG00000081551	0	1	0	0	0	0	0	1	0	1
ENSMUSG00000048693	0	0	0	0	0	0	0	3	0	0
ENSMUSG00000048699	0	0	0	0	0	0	6	0	0	0
ENSMUSG00000086684	0	0	0	0	0	0	0	0	0	0
ENSMUSG00000086685	0	0	0	0	0	0	0	0	0	0
ENSMUSG00000086687	0	3	0	0	4	0	5	0	0	0
ENSMUSG00000086689	2	9	0	4	7	0	0	0	4	0
ENSMUSG00000099666	0	0	0	0	0	0	0	0	0	0
ENSMUSG00000099665	0	0	0	0	0	0	0	0	0	0
ENSMUSG00000099661	0	0	0	0	0	0	0	0	0	0
ENSMUSG00000099668	0	0	0	0	0	0	0	0	0	0
ENSMUSG00000099669	0	0	0	0	3	0	0	0	0	0
ENSMUSG00000112134	0	0	6	9	0	0	0	0	0	0
ENSMUSG00000112135	3	4	0	2	3	1	2	2	1	3
ENSMUSG00000112136	0	0	0	0	0	0	0	0	0	0
ENSMUSG00000112137	6	0	0	0	5	0	2	0	3	0
ENSMUSG00000112130	0	0	0	0	0	0	0	0	0	0
ENSMUSG00000112131	0	0	0	0	0	0	0	0	0	0
ENSMUSG00000112132	0	2	3	0	0	0	3	0	0	0
ENSMUSG00000112133	0	0	0	0	0	0	1	0	2	2
ENSMUSG00000112139	0	3	0	0	0	0	0	0	0	0
ENSMUSG00000030114	6	9	6	9	21	15	32	12	12	0
ENSMUSG00000030117	15	24	24	27	8	25	51	14	53	15
ENSMUSG00000030111	255	348	257	415	207	454	242	220	179	210
ENSMUSG00000030110	596	735	614	711	452	357	278	378	344	509
ENSMUSG00000105529	0	1	0	0	0	0	0	0	0	0
ENSMUSG00000092362	0	0	0	0	0	0	0	0	0	0
ENSMUSG00000092363	0	0	0	0	0	0	0	0	1	1
ENSMUSG00000099592	0	0	0	0	0	0	0	0	0	0

ENSMUSG00000099593	0	0	0	0	0	0	0	0	0	0
ENSMUSG00000099594	0	0	0	0	0	0	0	0	0	0
ENSMUSG00000099595	0	0	0	0	0	0	0	0	0	0
ENSMUSG00000099596	0	2	0	0	0	0	0	0	0	2
ENSMUSG00000099598	0	0	0	0	0	0	0	0	0	0
ENSMUSG00000099599	0	0	0	0	0	0	0	0	0	0
ENSMUSG00000092369	0	0	0	0	0	0	0	0	0	0
ENSMUSG00000100034	0	0	0	0	0	0	0	0	0	0
ENSMUSG00000055298	0	0	0	0	0	0	3	0	0	0
ENSMUSG00000051977	110	109	106	138	122	175	191	162	136	196
ENSMUSG00000016179	19	43	0	21	41	49	109	87	52	23
ENSMUSG00000003411	28	49	35	30	24	95	82	102	47	26
ENSMUSG00000043760	9	0	15	39	0	0	7	3	3	0
ENSMUSG00000086025	0	0	0	0	0	0	3	0	0	0
ENSMUSG00000086026	0	0	0	0	0	0	0	0	0	0
ENSMUSG00000086020	0	0	0	0	0	0	0	0	0	0
ENSMUSG00000086021	0	4	0	0	0	0	0	0	0	0
ENSMUSG00000086022	0	3	0	3	0	0	3	3	6	0
ENSMUSG00000094868	0	0	0	0	0	0	0	0	0	0
ENSMUSG00000086028	0	0	0	0	0	0	0	0	0	0
ENSMUSG00000076309	1	1	1	1	1	1	1	1	1	1
ENSMUSG00000041722	0	0	0	0	0	0	0	0	0	0
ENSMUSG00000072686	0	0	0	0	0	0	0	0	0	0
ENSMUSG00000076305	0	0	0	0	0	0	0	0	0	0
ENSMUSG00000072683	2	0	0	0	0	0	6	0	0	0
ENSMUSG00000076303	0	0	0	0	0	0	1	0	0	0
ENSMUSG00000076300	0	0	0	0	0	0	0	0	0	0
ENSMUSG00000005718	183	151	214	271	162	288	272	286	313	280
ENSMUSG00000064616	0	0	0	0	0	0	0	0	0	0
ENSMUSG00000070933	0	3	0	0	0	0	5	0	0	5
ENSMUSG00000005716	58	86	93	74	48	88	57	87	58	58
ENSMUSG00000037466	52	38	31	78	60	20	103	59	57	63
ENSMUSG00000037463	15	6	9	18	9	8	25	25	18	20
ENSMUSG00000088726	0	0	0	0	0	0	0	0	0	0
ENSMUSG00000087643	0	0	0	0	0	0	0	0	0	0
ENSMUSG00000087641	0	0	0	0	0	0	0	0	0	0
ENSMUSG00000087647	0	0	0	0	0	0	0	0	0	0
ENSMUSG00000087646	0	0	0	0	0	0	3	0	0	0
ENSMUSG00000070683	0	0	2	3	0	3	3	0	0	0
ENSMUSG00000087648	0	0	0	0	6	0	3	0	0	0
ENSMUSG00000070687	0	0	0	0	0	0	0	0	0	6
ENSMUSG00000070686	0	0	0	0	0	0	0	0	0	6
ENSMUSG00000040231	4	4	8	6	2	3	6	7	10	2
ENSMUSG00000095745	0	0	0	0	0	0	0	0	0	0
ENSMUSG00000087909	0	0	0	0	0	0	0	0	0	0
ENSMUSG00000064137	0	0	0	0	0	0	0	0	0	0
ENSMUSG00000107533	0	0	0	0	0	0	0	0	0	0
ENSMUSG00000107530	0	0	0	0	0	0	0	0	0	0
ENSMUSG00000071332	6	0	9	0	6	0	3	9	0	0
ENSMUSG00000093227	0	0	0	0	0	0	0	0	1	1
ENSMUSG00000046790	0	0	0	0	0	0	0	0	0	0
ENSMUSG00000065660	0	0	0	0	0	0	0	0	0	0
ENSMUSG00000061561	0	0	0	0	3	0	12	0	0	2
ENSMUSG00000065665	0	0	0	0	0	0	0	0	0	0
ENSMUSG00000085649	0	0	0	0	0	0	0	0	0	0
ENSMUSG00000085648	0	0	0	0	0	0	0	0	0	0
ENSMUSG00000085645	0	0	0	0	6	0	3	3	3	0
ENSMUSG00000085646	0	0	0	0	0	0	0	2	0	0

ENSMUSG00000085641	0	0	0	0	0	0	0	0	0	0
ENSMUSG00000085642	39	42	65	40	49	55	51	87	85	43
ENSMUSG00000074300	0	0	0	0	0	0	0	0	0	0
ENSMUSG00000074302	0	0	0	0	0	0	0	0	0	0
ENSMUSG00000081946	2	2	0	3	0	0	2	0	0	0
ENSMUSG00000081945	0	0	0	0	0	0	0	0	0	0
ENSMUSG00000081942	0	0	0	0	0	0	0	0	0	0
ENSMUSG00000081943	0	0	0	0	0	0	0	0	0	0
ENSMUSG00000081940	0	0	0	0	0	0	0	0	0	0
ENSMUSG00000081941	0	0	0	0	0	0	0	0	0	0
ENSMUSG00000109797	0	0	0	0	0	0	0	0	0	0
ENSMUSG00000062037	0	0	0	0	0	0	0	0	0	0
ENSMUSG00000062036	0	0	4	0	0	0	0	0	0	0
ENSMUSG00000111591	0	3	0	0	0	0	0	0	0	0
ENSMUSG00000068167	0	0	0	0	0	0	3	0	0	0
ENSMUSG00000073656	0	0	5	0	0	12	4	0	0	0
ENSMUSG00000073650	33	45	50	35	29	55	56	44	70	47
ENSMUSG00000111625	0	0	0	0	0	0	0	0	0	0
ENSMUSG00000102013	0	0	0	0	0	0	0	0	0	0
ENSMUSG00000102010	0	0	0	0	0	0	0	0	0	0
ENSMUSG00000102011	0	0	0	0	0	0	0	0	0	0
ENSMUSG00000102016	0	0	0	0	0	0	0	0	0	0
ENSMUSG00000102017	0	0	0	0	0	0	0	0	0	0
ENSMUSG00000102015	0	0	0	0	0	0	0	0	0	0
ENSMUSG00000109840	0	0	0	0	0	0	0	0	0	0
ENSMUSG00000098011	0	0	0	0	0	0	0	0	0	0
ENSMUSG00000098145	3	0	2	0	2	0	0	6	4	4
ENSMUSG00000098147	0	0	0	0	0	0	0	0	0	0
ENSMUSG00000098141	2	0	2	0	0	0	2	0	0	0
ENSMUSG00000098143	0	0	0	0	0	0	0	0	0	0
ENSMUSG00000109843	0	0	6	3	0	1	0	0	0	9
ENSMUSG00000098149	0	0	2	0	2	3	2	2	2	2
ENSMUSG00000109848	0	0	0	0	0	0	0	0	0	0
ENSMUSG00000096107	0	3	0	4	0	3	0	0	3	5
ENSMUSG00000103942	0	0	0	0	0	0	0	0	0	0
ENSMUSG00000034171	54	31	31	44	12	21	158	38	73	79
ENSMUSG00000034177	18	14	12	18	9	21	27	23	51	20
ENSMUSG00000019992	11	10	16	19	12	5	32	10	0	4
ENSMUSG00000082169	0	0	0	0	0	0	0	0	0	0
ENSMUSG00000082168	0	0	0	0	0	0	0	0	0	0
ENSMUSG00000111085	3	0	18	0	0	9	3	0	6	11
ENSMUSG00000084891	0	0	0	0	0	0	0	0	0	3
ENSMUSG00000084890	3	0	0	0	0	0	0	0	3	0
ENSMUSG00000066640	569	636	591	559	727	629	819	717	716	597
ENSMUSG00000084898	0	0	0	0	0	0	0	0	0	0
ENSMUSG00000111081	0	0	0	0	0	0	0	0	0	0
ENSMUSG00000091459	0	0	0	0	0	0	0	0	0	0
ENSMUSG00000101092	0	0	0	0	0	0	0	0	0	0
ENSMUSG00000101091	0	0	0	0	0	0	0	0	0	0
ENSMUSG00000101090	0	0	0	0	0	0	0	0	0	0
ENSMUSG00000101097	0	0	0	0	0	0	0	0	0	0
ENSMUSG00000101096	0	0	0	0	0	0	0	0	0	0
ENSMUSG00000101095	0	0	0	0	0	0	0	0	0	0
ENSMUSG00000050534	3	0	3	3	0	9	3	0	0	3
ENSMUSG00000091450	0	0	0	0	0	0	0	0	0	0
ENSMUSG00000101099	0	0	0	0	0	0	0	0	0	0
ENSMUSG00000101098	0	0	0	0	0	0	0	0	0	0
ENSMUSG00000091455	26	30	35	60	44	29	182	39	45	68

ENSMUSG00000067519	0	0	0	0	0	0	0	0	0	0
ENSMUSG00000057337	176	145	158	119	108	156	227	194	222	224
ENSMUSG00000067513	0	0	0	0	0	0	0	0	0	0
ENSMUSG00000114260	0	0	0	0	0	0	0	0	0	0
ENSMUSG00000112635	0	0	0	0	0	0	0	0	0	0
ENSMUSG00000101404	0	0	0	0	0	0	0	0	0	0
ENSMUSG00000113817	0	0	0	0	0	0	0	0	0	0
ENSMUSG00000108859	0	0	0	0	0	0	3	0	0	0
ENSMUSG00000108854	26	44	46	14	26	15	25	22	17	24
ENSMUSG00000104692	0	0	0	0	0	0	0	0	0	0
ENSMUSG00000108856	0	0	0	0	0	0	0	0	4	2
ENSMUSG00000108857	0	0	0	0	0	0	0	0	0	0
ENSMUSG00000064890	0	0	0	0	0	0	0	0	0	0
ENSMUSG00000108852	2	2	1	1	1	3	4	0	0	0
ENSMUSG00000101403	0	0	0	0	0	0	0	0	0	0
ENSMUSG00000105540	0	3	0	3	6	0	0	0	0	3
ENSMUSG00000101901	0	0	0	0	0	0	0	0	0	0
ENSMUSG00000112638	0	0	0	0	0	0	0	0	0	0
ENSMUSG00000101637	0	0	0	0	0	0	0	0	0	0
ENSMUSG00000101634	6	38	24	19	9	20	6	6	14	24
ENSMUSG00000056379	0	0	0	0	0	0	0	0	0	0
ENSMUSG00000101632	0	0	0	0	0	0	0	0	0	0
ENSMUSG00000101631	0	0	0	0	0	0	0	0	0	0
ENSMUSG00000101906	0	0	0	0	0	0	0	0	0	0
ENSMUSG00000056370	0	0	0	0	0	93	0	0	6	0
ENSMUSG00000101639	0	0	0	0	0	0	0	1	0	0
ENSMUSG00000101638	0	0	0	0	0	0	0	0	0	0
ENSMUSG00000100322	0	0	0	0	0	0	0	0	0	0
ENSMUSG00000061911	13	0	4	2	12	0	15	0	3	5
ENSMUSG00000100321	0	0	0	0	0	0	0	0	0	0
ENSMUSG00000100326	0	0	0	0	0	0	0	0	0	0
ENSMUSG00000100324	0	0	0	0	0	0	0	0	0	0
ENSMUSG00000100325	0	0	0	0	0	0	0	0	0	0
ENSMUSG00000100328	0	0	0	0	0	0	0	0	0	0
ENSMUSG00000100329	0	0	0	0	0	0	0	0	0	0
ENSMUSG00000058360	0	0	0	0	0	3	0	0	0	0
ENSMUSG00000058368	0	0	0	0	0	0	0	0	0	0
ENSMUSG00000102285	0	0	0	0	0	0	0	0	0	0
ENSMUSG00000026875	250	204	192	275	107	207	451	246	320	279
ENSMUSG00000026874	3	0	12	6	3	80	45	3	6	9
ENSMUSG00000088318	1	0	0	0	0	0	0	0	2	0
ENSMUSG00000082427	0	0	0	0	0	0	0	0	0	0
ENSMUSG00000107671	0	0	0	0	0	0	0	0	0	0
ENSMUSG00000107673	0	0	0	0	0	0	0	0	0	0
ENSMUSG00000107672	0	0	0	0	0	0	0	0	0	0
ENSMUSG00000107675	0	0	0	0	0	0	0	0	0	0
ENSMUSG00000107677	0	0	0	0	0	0	0	0	0	0
ENSMUSG00000107676	5	14	7	8	6	6	11	21	4	20
ENSMUSG00000107679	0	0	0	0	0	0	0	0	0	0
ENSMUSG00000107678	0	0	0	0	0	0	0	0	0	0
ENSMUSG00000103160	18	9	6	4	14	17	59	14	44	32
ENSMUSG00000103167	0	0	0	0	0	0	0	0	0	0
ENSMUSG00000103168	0	0	0	0	0	0	0	0	0	0
ENSMUSG00000061000	0	0	0	0	0	0	0	0	0	0
ENSMUSG00000102409	0	0	0	0	0	0	0	0	0	0
ENSMUSG00000103311	0	0	0	0	0	0	0	0	0	0
ENSMUSG00000058981	0	0	0	0	0	0	0	0	0	0
ENSMUSG00000089047	0	0	0	0	0	0	0	0	1	0

ENSMUSG00000089046	0	0	0	0	0	0	0	0	0	0
ENSMUSG00000102280	0	0	0	0	0	0	0	0	3	0
ENSMUSG00000089043	0	0	0	0	0	0	0	0	0	2
ENSMUSG00000089041	0	0	1	0	0	0	0	0	0	0
ENSMUSG00000089040	0	0	0	0	0	0	0	0	0	0
ENSMUSG00000089049	0	0	0	0	0	0	0	0	0	0
ENSMUSG00000106070	2	0	0	0	0	0	0	0	0	0
ENSMUSG00000106071	0	0	0	0	0	0	0	0	0	0
ENSMUSG00000106072	0	0	0	0	0	0	0	0	0	0
ENSMUSG00000106074	0	0	0	0	0	0	0	0	0	0
ENSMUSG00000106076	0	2	0	0	0	0	0	0	2	0
ENSMUSG00000106077	0	0	0	0	0	0	0	0	0	0
ENSMUSG00000106078	0	0	0	0	0	0	0	0	0	0
ENSMUSG00000106079	0	0	0	0	0	0	0	0	0	2
ENSMUSG00000094243	0	0	0	0	0	0	0	0	0	0
ENSMUSG00000094240	0	0	0	0	0	0	0	0	0	0
ENSMUSG00000094247	0	0	0	0	0	0	0	0	0	0
ENSMUSG00000018924	9	3	0	6	20	29	79	42	30	157
ENSMUSG00000018925	0	3	3	3	0	3	11	5	18	0
ENSMUSG00000020020	5	0	7	3	0	0	4	0	4	6
ENSMUSG00000110249	0	3	0	0	2	7	15	5	14	3
ENSMUSG00000110246	0	0	0	0	0	0	6	0	0	0
ENSMUSG00000051276	0	0	0	0	0	0	0	0	0	0
ENSMUSG00000110241	0	0	0	0	0	0	0	0	0	0
ENSMUSG00000092162	0	0	0	0	0	0	0	0	0	0
ENSMUSG00000092164	0	3	0	0	3	0	3	3	0	0
ENSMUSG00000092166	0	0	0	0	0	0	0	0	0	0
ENSMUSG00000109232	0	0	1	0	0	4	0	0	0	0
ENSMUSG00000004609	616	424	627	939	475	755	845	525	657	719
ENSMUSG00000003657	0	0	0	0	0	0	0	0	0	0
ENSMUSG00000035704	163	156	200	188	161	161	283	172	186	182
ENSMUSG00000109234	0	0	0	0	0	0	0	0	0	0
ENSMUSG00000106694	2	3	6	3	0	3	0	0	0	0
ENSMUSG00000092587	0	0	0	0	0	0	0	0	0	0
ENSMUSG00000075153	0	0	0	0	0	0	0	0	0	0
ENSMUSG00000106690	0	0	0	0	0	0	0	0	0	0
ENSMUSG00000106692	0	0	0	0	0	0	0	0	0	0
ENSMUSG00000113573	0	0	0	0	0	0	0	0	0	0
ENSMUSG00000113572	0	0	0	0	0	3	0	0	0	0
ENSMUSG00000038751	6	14	9	27	15	20	3	32	15	17
ENSMUSG00000113570	26	9	18	6	5	6	25	14	15	9
ENSMUSG00000038756	0	0	0	3	0	0	0	3	0	3
ENSMUSG00000038754	0	3	0	0	0	0	0	0	0	6
ENSMUSG00000069355	0	0	0	0	0	0	9	5	0	3
ENSMUSG00000094867	0	0	0	0	0	0	0	0	0	0
ENSMUSG00000094866	0	0	0	0	0	0	0	0	0	0
ENSMUSG00000094865	0	0	0	0	0	0	0	0	0	0
ENSMUSG00000094864	0	0	0	0	0	0	0	0	0	0
ENSMUSG00000094861	0	0	0	0	0	0	0	0	0	0
ENSMUSG00000094860	0	0	0	0	0	0	0	0	0	0
ENSMUSG00000105527	0	0	0	0	0	0	0	0	0	0
ENSMUSG00000105525	0	0	0	0	0	0	0	0	0	0
ENSMUSG00000105524	0	0	1	0	0	0	0	0	0	0
ENSMUSG00000105523	0	0	0	0	0	0	0	8	0	0
ENSMUSG00000105522	0	0	0	0	0	0	0	0	0	0
ENSMUSG00000094869	0	0	0	0	0	0	0	0	0	0
ENSMUSG00000105520	0	2	0	0	0	0	0	0	0	0
ENSMUSG00000110309	0	0	0	0	0	0	0	0	0	0

ENSMUSG00000029219	12	0	0	0	0	3	0	0	3	0
ENSMUSG00000082833	0	0	3	4	0	0	0	4	0	0
ENSMUSG00000082832	0	0	0	0	0	0	0	0	0	0
ENSMUSG00000082831	0	0	0	0	0	0	0	0	0	0
ENSMUSG00000066364	9	0	0	9	3	9	173	19	63	26
ENSMUSG00000082835	0	0	0	0	0	0	0	0	0	0
ENSMUSG00000066366	0	0	0	9	0	0	3	0	0	0
ENSMUSG00000066368	0	0	0	0	0	0	0	0	0	0
ENSMUSG00000082839	0	0	0	0	0	0	0	0	0	0
ENSMUSG00000102403	3	0	0	0	0	0	0	0	0	0
ENSMUSG00000084646	0	0	0	0	0	0	0	0	0	0
ENSMUSG00000111598	0	0	0	0	0	0	0	0	0	0
ENSMUSG00000010803	33	9	12	26	21	48	23	35	42	15
ENSMUSG00000111590	0	0	0	0	3	0	0	0	0	0
ENSMUSG00000111597	0	0	0	0	0	0	0	0	0	0
ENSMUSG00000111596	0	0	0	0	0	0	0	0	0	0
ENSMUSG00000111594	121	78	85	91	58	88	101	98	84	105
ENSMUSG00000109844	0	0	0	0	0	0	0	0	0	0
ENSMUSG00000109845	0	0	0	0	0	0	0	0	0	0
ENSMUSG00000111627	0	0	0	4	0	0	1	0	0	0
ENSMUSG00000111621	0	0	0	0	0	0	0	0	0	0
ENSMUSG00000111620	0	2	2	7	2	3	0	5	11	0
ENSMUSG00000111622	0	0	0	0	0	0	0	0	0	0
ENSMUSG00000111629	0	3	5	0	3	0	0	3	2	3
ENSMUSG00000109849	0	0	0	6	0	0	0	3	3	6
ENSMUSG00000026468	3	0	0	0	0	3	12	9	8	0
ENSMUSG00000084643	0	0	0	0	0	0	0	0	0	0
ENSMUSG00000047938	0	0	0	3	0	0	0	0	3	0
ENSMUSG00000104698	1	0	0	0	0	0	0	0	0	6
ENSMUSG00000104695	0	5	1	0	0	1	6	0	0	3
ENSMUSG00000047935	0	0	0	0	0	2	0	0	0	6
ENSMUSG00000050538	53	59	44	45	49	57	103	59	59	60
ENSMUSG00000104691	0	0	0	0	0	0	0	0	0	0
ENSMUSG00000055942	0	0	0	0	0	0	0	0	0	0
ENSMUSG00000104473	0	0	0	0	0	0	0	0	0	0
ENSMUSG00000055944	0	0	0	0	0	0	0	0	0	0
ENSMUSG00000057540	0	0	0	0	0	0	0	0	0	0
ENSMUSG00000075188	0	0	0	0	0	0	0	0	3	0
ENSMUSG00000086975	0	0	0	0	0	0	0	0	0	0
ENSMUSG00000104340	0	5	0	3	0	0	0	0	0	0
ENSMUSG00000104341	0	0	0	1	0	0	0	0	0	0
ENSMUSG00000104342	16	8	9	9	8	3	16	8	15	14
ENSMUSG00000104343	0	6	0	3	0	0	3	6	3	8
ENSMUSG00000104345	0	0	0	0	0	0	0	0	0	0
ENSMUSG00000030851	0	0	0	0	0	6	0	3	0	0
ENSMUSG00000110264	0	6	0	0	5	3	0	3	0	6
ENSMUSG00000056632	0	0	0	6	0	0	0	0	0	0
ENSMUSG00000091504	0	0	0	0	0	0	0	0	0	0
ENSMUSG00000020226	0	0	0	3	0	0	3	0	0	3
ENSMUSG00000045797	0	0	0	0	0	0	0	0	0	0
ENSMUSG00000095380	0	0	0	0	0	0	0	0	0	0
ENSMUSG00000095383	0	0	0	0	0	0	0	0	0	0
ENSMUSG00000045794	0	0	0	0	3	0	6	0	2	0
ENSMUSG00000045792	0	0	0	0	0	0	0	0	0	0
ENSMUSG00000095386	0	0	0	0	0	0	0	0	0	0
ENSMUSG00000095389	0	0	0	0	0	0	0	0	0	0
ENSMUSG00000044772	0	0	0	0	0	0	0	0	0	0
ENSMUSG00000053044	26	9	27	23	9	14	122	70	125	48

ENSMUSG00000053046	56	49	51	54	21	55	59	34	68	22
ENSMUSG00000053040	187	169	167	285	159	254	277	276	207	238
ENSMUSG00000045421	0	0	0	0	0	0	3	0	0	0
ENSMUSG00000095659	0	0	0	0	0	0	0	0	0	0
ENSMUSG00000095658	0	0	0	0	0	0	0	0	0	0
ENSMUSG00000095653	0	0	0	0	0	0	0	0	0	0
ENSMUSG00000095650	0	0	0	0	0	0	0	0	0	0
ENSMUSG00000095657	0	0	0	0	0	0	0	0	0	0
ENSMUSG00000095655	0	1	1	1	1	1	1	2	0	0
ENSMUSG00000098382	0	0	0	0	0	0	0	0	0	0
ENSMUSG00000098383	0	0	0	0	0	0	0	0	0	0
ENSMUSG00000098386	0	0	0	0	0	0	0	0	0	1
ENSMUSG00000098388	0	0	0	0	1	0	0	1	0	0
ENSMUSG00000006143	0	0	0	0	0	0	0	6	10	0
ENSMUSG00000080529	0	0	0	0	0	0	0	0	0	0
ENSMUSG00000080522	0	0	0	0	0	0	0	0	0	0
ENSMUSG00000080526	0	0	0	0	0	0	0	0	0	0
ENSMUSG00000097315	0	0	0	0	0	0	0	0	0	0
ENSMUSG00000030361	0	15	9	0	0	3	3	10	4	0
ENSMUSG00000040489	64	58	30	58	18	39	23	47	33	43
ENSMUSG00000028976	3	3	6	18	6	10	42	10	3	0
ENSMUSG00000093719	0	0	0	0	0	0	0	0	0	0
ENSMUSG00000020916	0	0	0	0	0	3	0	0	3	0
ENSMUSG00000093711	0	0	0	0	0	0	0	0	0	0
ENSMUSG00000093714	1	0	0	0	0	0	0	0	0	0
ENSMUSG00000110169	0	0	0	0	0	0	0	0	0	0
ENSMUSG00000110168	0	0	0	0	0	0	0	0	0	0
ENSMUSG00000052025	0	0	0	0	0	0	0	0	0	0
ENSMUSG00000052026	21	0	6	18	17	45	153	79	77	72
ENSMUSG00000002240	0	0	0	0	0	0	0	0	0	0
ENSMUSG00000047150	18	0	11	16	6	5	18	3	11	3
ENSMUSG00000047155	3	0	3	3	0	0	3	0	0	0
ENSMUSG00000095928	0	0	0	0	0	0	0	0	0	0
ENSMUSG00000077776	0	0	0	0	0	0	0	0	0	0
ENSMUSG00000077771	0	0	0	0	0	0	0	0	0	0
ENSMUSG00000077772	0	0	0	0	0	0	0	0	0	0
ENSMUSG00000109383	9	0	5	5	5	0	5	5	7	6
ENSMUSG00000112063	0	0	0	0	0	0	0	0	0	0
ENSMUSG00000055555	6	6	3	8	0	0	3	8	6	12
ENSMUSG00000049685	0	0	0	0	0	0	0	0	0	0
ENSMUSG00000095927	0	0	0	0	0	0	0	0	0	0
ENSMUSG00000048080	0	0	0	0	0	0	0	0	0	0
ENSMUSG00000092447	0	0	0	0	0	0	0	0	0	0
ENSMUSG00000099416	0	0	0	0	0	0	0	0	0	0
ENSMUSG00000099411	105	84	89	102	58	89	30	59	44	102
ENSMUSG00000099410	0	0	0	0	0	0	0	0	0	0
ENSMUSG00000099413	2	0	12	21	15	1	8	9	18	17
ENSMUSG00000100464	0	0	0	0	0	0	0	0	0	0
ENSMUSG00000113033	0	0	0	0	0	0	0	0	0	0
ENSMUSG00000100467	0	0	0	0	0	0	0	0	0	0
ENSMUSG00000092448	0	0	0	0	0	0	9	0	0	0
ENSMUSG00000100466	0	0	0	0	0	0	0	0	0	0
ENSMUSG00000100461	0	0	0	0	0	0	0	0	0	0
ENSMUSG00000041301	3	10	34	18	9	12	21	21	3	12
ENSMUSG00000101497	0	0	0	0	0	0	0	0	0	0
ENSMUSG00000062152	0	0	2	0	0	0	0	2	0	3
ENSMUSG00000030701	276	242	139	258	119	153	227	268	175	338
ENSMUSG00000082316	0	0	0	0	0	0	0	0	0	0

ENSMUSG00000082312	0	0	0	0	0	0	0	0	0	0
ENSMUSG00000082310	1	0	0	0	0	0	0	0	0	0
ENSMUSG00000082311	0	0	0	0	0	0	0	0	0	0
ENSMUSG00000048337	45	66	54	45	39	80	129	80	92	98
ENSMUSG00000048334	3	2	5	2	8	1	1	5	5	6
ENSMUSG00000062151	36	33	6	9	6	15	51	14	35	15
ENSMUSG00000100469	0	0	0	0	0	0	0	0	0	0
ENSMUSG00000101490	0	0	0	0	0	0	0	0	0	0
ENSMUSG00000102162	0	2	0	2	0	0	0	0	0	1
ENSMUSG00000100120	0	0	0	0	0	0	5	13	6	5
ENSMUSG00000072726	0	0	0	0	0	0	0	0	0	0
ENSMUSG00000100123	0	0	0	0	0	0	0	0	0	0
ENSMUSG00000100124	0	0	0	0	0	0	0	0	0	0
ENSMUSG00000100125	0	0	0	0	0	0	0	0	0	0
ENSMUSG00000072722	0	0	0	0	0	0	0	0	0	0
ENSMUSG00000072723	0	9	6	16	7	16	21	14	27	25
ENSMUSG00000100129	0	0	2	0	0	2	0	0	0	0
ENSMUSG00000101498	0	0	0	0	0	0	0	0	0	0
ENSMUSG00000043531	26	43	47	18	33	36	91	54	45	65
ENSMUSG00000084413	2	2	2	0	3	2	3	2	0	0
ENSMUSG00000084412	0	0	0	0	0	0	1	0	0	0
ENSMUSG00000084417	0	0	0	0	0	0	0	0	0	0
ENSMUSG00000043537	0	0	0	0	0	0	0	0	0	0
ENSMUSG00000084415	0	0	0	0	0	0	0	0	0	0
ENSMUSG00000084414	0	0	0	0	0	0	0	0	0	0
ENSMUSG00000113779	2	0	0	0	1	0	0	0	0	0
ENSMUSG00000100698	0	0	0	0	0	0	0	0	0	0
ENSMUSG00000100699	0	0	0	0	0	0	0	0	0	0
ENSMUSG00000061633	0	0	0	2	0	0	0	0	0	0
ENSMUSG00000100692	0	0	0	0	0	0	0	0	0	0
ENSMUSG00000100693	0	0	0	0	0	0	0	0	0	0
ENSMUSG00000100690	0	0	0	0	0	0	0	0	0	0
ENSMUSG00000100696	0	0	0	3	0	0	0	0	0	0
ENSMUSG00000100694	0	0	0	0	0	0	0	0	0	0
ENSMUSG00000100695	0	0	0	0	0	0	0	0	0	0
ENSMUSG00000087852	0	0	0	0	0	0	0	0	0	0
ENSMUSG00000087853	0	0	0	0	0	0	0	0	0	0
ENSMUSG00000087850	0	0	0	0	0	0	0	0	0	0
ENSMUSG00000087855	0	0	0	0	0	0	0	0	0	0
ENSMUSG00000087858	0	0	0	0	0	0	0	0	0	0
ENSMUSG00000037167	0	0	0	2	0	2	2	3	0	3
ENSMUSG00000037161	43	27	38	36	27	3	48	20	42	22
ENSMUSG00000107479	0	0	0	0	3	0	0	9	0	0
ENSMUSG00000107477	16	5	3	3	0	8	3	3	9	0
ENSMUSG00000107475	0	0	0	0	0	0	0	0	0	0
ENSMUSG00000083191	0	3	0	2	0	3	0	4	0	0
ENSMUSG00000083190	0	0	0	0	0	0	0	0	0	0
ENSMUSG00000102197	0	0	0	2	0	0	0	2	0	2
ENSMUSG00000079528	0	0	0	0	0	1	0	0	0	0
ENSMUSG00000079525	0	0	0	0	0	0	0	0	0	0
ENSMUSG00000102195	0	0	0	0	0	0	0	0	0	0
ENSMUSG00000079521	0	0	0	0	0	0	0	0	0	0
ENSMUSG00000046687	2	0	3	5	2	5	12	3	7	7
ENSMUSG00000061246	0	0	0	0	0	0	0	0	0	0
ENSMUSG00000099970	0	0	0	0	0	0	0	0	1	0
ENSMUSG00000046683	20	29	43	29	22	40	23	38	24	35
ENSMUSG00000107078	0	0	0	0	0	0	0	0	0	0
ENSMUSG00000062914	0	0	0	0	0	2	0	0	0	0

ENSMUSG00000036412	77	116	83	91	51	65	143	88	120	70
ENSMUSG00000107110	0	0	0	0	3	0	0	0	0	0
ENSMUSG00000101439	47	40	76	42	33	37	50	53	58	42
ENSMUSG00000065031	0	0	0	0	0	0	0	0	0	0
ENSMUSG00000078698	0	0	0	0	0	0	0	0	0	0
ENSMUSG00000078691	0	0	0	0	0	0	0	0	0	0
ENSMUSG00000103579	0	0	0	0	0	0	0	0	0	0
ENSMUSG00000101234	6	5	6	0	3	3	2	5	6	3
ENSMUSG00000103571	0	0	0	0	0	0	0	0	0	0
ENSMUSG00000103575	0	0	0	0	0	0	0	0	0	0
ENSMUSG00000105105	0	0	0	0	0	0	0	0	0	2
ENSMUSG00000035148	4	0	0	0	3	9	6	12	3	0
ENSMUSG00000105106	0	0	0	0	0	0	0	0	0	0
ENSMUSG00000078346	0	0	0	0	0	0	0	0	0	0
ENSMUSG00000105102	0	0	0	0	0	0	0	0	0	0
ENSMUSG00000100900	0	0	0	0	0	0	0	0	0	0
ENSMUSG00000100904	0	0	0	0	0	0	0	0	0	0
ENSMUSG00000089458	0	0	0	0	0	0	0	0	0	0
ENSMUSG00000089459	0	0	0	0	0	0	0	0	0	0
ENSMUSG00000100908	0	0	0	0	0	0	0	0	0	0
ENSMUSG00000097468	26	0	0	0	0	0	33	24	21	0
ENSMUSG00000097463	6	28	23	6	7	19	22	19	23	38
ENSMUSG00000097466	0	0	0	0	0	0	0	0	3	0
ENSMUSG00000097465	0	0	0	0	0	0	0	0	0	3
ENSMUSG00000063796	3	9	0	24	3	0	18	4	0	9
ENSMUSG00000076030	0	0	0	0	0	0	0	0	0	0
ENSMUSG00000083243	0	0	0	3	0	0	0	0	0	0
ENSMUSG00000096042	0	0	0	0	0	0	0	0	0	0
ENSMUSG00000086452	0	0	0	0	0	0	0	0	0	0
ENSMUSG00000058508	0	0	0	0	0	0	0	0	0	0
ENSMUSG00000069620	0	0	0	0	0	0	0	0	0	0
ENSMUSG00000102229	0	0	0	0	0	0	0	0	0	0
ENSMUSG00000102221	0	0	0	0	0	0	0	0	0	0
ENSMUSG00000102223	0	0	0	0	0	0	0	0	0	0
ENSMUSG00000102224	0	4	0	0	3	3	0	3	0	0
ENSMUSG00000102227	0	0	0	0	0	0	0	0	0	0
ENSMUSG00000096738	0	0	0	0	0	0	0	0	0	0
ENSMUSG00000096731	0	0	0	0	0	0	0	0	0	0
ENSMUSG00000096730	0	0	0	0	0	0	0	0	0	0
ENSMUSG00000096733	0	0	0	0	0	0	0	0	0	0
ENSMUSG00000096732	0	0	0	0	0	0	0	0	0	0
ENSMUSG00000096735	0	0	2	0	0	0	0	0	0	0
ENSMUSG00000096734	0	0	0	0	0	0	0	0	0	0
ENSMUSG00000096737	0	0	0	0	0	0	0	0	0	0
ENSMUSG00000031130	3	3	3	0	0	0	0	0	0	6
ENSMUSG00000031131	0	0	0	0	0	0	3	0	0	0
ENSMUSG00000031132	6	0	3	0	0	0	7	0	14	0
ENSMUSG00000031138	0	0	6	0	0	6	3	9	0	0
ENSMUSG00000031139	3	0	6	0	4	6	6	24	0	0
ENSMUSG00000063137	0	5	0	0	0	0	22	1	0	9
ENSMUSG00000063133	0	0	0	0	0	0	0	0	0	0
ENSMUSG00000086558	0	0	0	0	0	0	0	0	0	0
ENSMUSG00000063130	12	0	0	9	0	3	15	0	2	3
ENSMUSG00000021362	0	0	0	0	0	3	0	0	0	0
ENSMUSG00000021363	23	10	11	0	15	17	5	22	37	9
ENSMUSG00000021364	12	9	8	3	6	3	0	3	3	6
ENSMUSG00000057706	174	218	179	119	157	130	193	199	206	145
ENSMUSG00000050089	0	0	0	0	0	0	0	0	0	0

ENSMUSG00000050085	0	0	0	0	0	0	0	0	0	0
ENSMUSG00000104105	0	0	0	0	0	0	0	0	0	0
ENSMUSG00000104102	0	0	0	0	0	2	0	0	0	0
ENSMUSG00000104103	5	6	4	6	1	11	10	5	5	13
ENSMUSG00000104100	0	0	0	0	0	0	0	0	0	0
ENSMUSG00000104101	14	7	0	10	3	4	3	4	12	6
ENSMUSG00000044186	0	0	0	0	0	0	0	0	0	0
ENSMUSG00000104109	17	6	0	6	6	6	0	3	11	6
ENSMUSG00000099762	0	0	0	0	0	3	7	5	0	0
ENSMUSG00000088465	0	0	0	0	0	0	0	0	0	0
ENSMUSG00000088468	0	0	0	0	0	0	0	0	0	0
ENSMUSG00000091174	0	0	2	0	0	0	0	0	0	0
ENSMUSG00000091175	0	0	0	0	0	0	0	0	0	0
ENSMUSG00000021986	3	12	6	17	0	12	18	9	9	8
ENSMUSG00000110858	0	0	0	0	0	0	0	0	0	0
ENSMUSG00000110851	0	0	0	0	0	0	0	0	0	0
ENSMUSG00000110853	0	0	0	0	0	0	0	0	0	0
ENSMUSG00000110855	0	0	0	0	0	0	0	0	0	0
ENSMUSG00000110854	0	0	0	0	0	0	0	0	0	0
ENSMUSG00000110857	0	0	0	0	0	0	0	0	0	0
ENSMUSG00000110856	0	0	0	0	0	0	0	0	0	0
ENSMUSG00000027202	9	0	0	17	0	0	3	3	0	0
ENSMUSG00000104817	0	0	0	0	0	0	0	0	0	0
ENSMUSG00000104811	0	0	0	0	0	0	0	0	0	0
ENSMUSG00000028972	0	3	6	0	0	3	3	6	0	3
ENSMUSG00000104819	0	0	0	0	0	0	0	0	0	0
ENSMUSG00000095412	0	0	0	0	0	0	0	0	0	0
ENSMUSG00000058084	0	0	0	0	0	0	0	0	0	0
ENSMUSG00000022180	181	200	260	354	271	397	337	212	217	248
ENSMUSG00000096593	0	0	0	0	0	0	0	0	0	0
ENSMUSG00000022187	0	0	0	0	0	0	0	0	0	0
ENSMUSG00000096595	0	0	0	0	0	0	0	0	0	0
ENSMUSG00000107895	0	0	0	0	0	0	0	0	0	0
ENSMUSG00000087758	0	0	0	0	0	0	0	0	0	0
ENSMUSG00000027973	0	0	0	0	0	0	0	0	0	0
ENSMUSG00000027971	0	0	0	3	0	3	0	0	0	0
ENSMUSG00000027977	24	24	29	14	7	12	25	24	19	18
ENSMUSG00000086278	0	0	0	0	0	0	0	0	0	0
ENSMUSG00000086275	0	0	0	0	0	0	0	0	3	0
ENSMUSG00000086273	0	0	0	3	0	0	0	0	0	0
ENSMUSG00000086272	0	0	0	0	0	0	0	0	0	0
ENSMUSG00000111810	0	0	0	0	0	0	0	0	0	0
ENSMUSG00000111811	0	0	0	0	0	0	0	0	0	0
ENSMUSG00000111816	0	0	0	0	0	0	0	0	0	0
ENSMUSG00000052625	0	0	0	0	0	0	0	0	0	0
ENSMUSG00000111818	0	0	3	0	0	0	3	6	0	3
ENSMUSG00000111819	0	0	0	0	0	0	0	1	0	9
ENSMUSG00000023274	121	52	62	66	92	71	242	90	186	137
ENSMUSG00000097814	0	0	3	6	0	0	6	0	3	0
ENSMUSG00000112541	0	0	0	0	0	0	0	0	0	0
ENSMUSG00000103389	0	0	0	0	0	0	0	0	0	0
ENSMUSG00000112543	3	3	0	0	0	0	0	3	0	0
ENSMUSG00000112542	0	0	0	0	0	0	0	0	0	0
ENSMUSG00000096196	0	0	1	0	0	0	0	0	0	0
ENSMUSG00000039364	0	0	0	0	0	0	0	0	0	0
ENSMUSG00000112549	0	0	0	0	0	0	0	0	0	0
ENSMUSG00000108559	0	0	0	0	0	0	0	0	0	0
ENSMUSG00000104279	24	26	36	33	16	28	25	29	16	36

ENSMUSG00000104275	0	0	0	0	0	0	0	0	0	0
ENSMUSG00000092285	0	0	0	0	0	0	0	0	0	0
ENSMUSG00000092287	0	0	0	0	0	0	0	0	0	0
ENSMUSG00000092286	14	9	16	6	14	16	26	5	10	18
ENSMUSG00000092281	12	17	32	23	19	15	25	30	29	28
ENSMUSG00000047013	6	0	22	3	9	15	26	3	3	9
ENSMUSG00000047014	0	1	2	3	0	0	0	0	0	0
ENSMUSG00000004031	26	18	15	25	12	35	146	33	64	32
ENSMUSG00000071613	0	0	0	0	0	4	0	0	0	0
ENSMUSG00000024727	46	75	54	40	63	101	277	143	158	151
ENSMUSG00000049420	6	0	9	3	8	36	109	24	39	26
ENSMUSG00000024728	0	0	0	0	0	0	0	0	0	0
ENSMUSG00000020617	0	0	0	0	0	0	0	0	0	0
ENSMUSG00000097103	17	2	12	8	13	12	8	14	7	2
ENSMUSG00000097107	0	0	0	0	0	0	0	0	3	0
ENSMUSG00000106448	0	0	0	0	0	0	0	0	0	0
ENSMUSG00000046942	0	0	0	0	0	0	0	0	0	0
ENSMUSG00000110031	0	0	0	0	0	0	0	0	0	0
ENSMUSG00000110035	0	0	0	0	3	0	0	0	0	5
ENSMUSG00000070568	0	0	2	0	0	0	0	0	0	0
ENSMUSG00000070564	0	5	3	0	0	0	9	0	0	0
ENSMUSG00000106445	11	0	0	0	0	3	3	6	0	0
ENSMUSG00000054117	0	0	0	0	0	0	0	0	0	0
ENSMUSG00000077049	0	0	0	0	2	0	0	0	0	0
ENSMUSG00000105778	0	0	0	0	0	0	0	0	0	0
ENSMUSG00000109497	0	0	0	0	0	0	0	0	0	0
ENSMUSG00000109496	0	0	0	0	0	0	0	0	0	0
ENSMUSG00000109495	0	2	0	0	0	3	0	0	4	0
ENSMUSG00000109494	0	0	0	0	0	0	0	0	0	0
ENSMUSG00000105773	0	1	1	1	1	0	1	0	1	1
ENSMUSG00000101772	0	0	0	0	0	0	0	0	0	0
ENSMUSG00000031478	197	227	272	213	228	318	295	284	305	271
ENSMUSG00000037106	35	47	36	59	58	19	49	37	29	32
ENSMUSG00000107768	0	9	14	3	6	6	46	12	16	13
ENSMUSG00000108728	0	0	0	0	0	0	5	3	0	0
ENSMUSG00000108726	0	3	0	0	0	0	0	9	0	3
ENSMUSG00000108727	0	0	0	0	0	0	0	0	0	0
ENSMUSG00000108724	0	0	0	4	2	0	3	3	3	0
ENSMUSG00000108725	0	0	0	0	0	0	0	0	0	0
ENSMUSG00000108722	0	0	2	0	0	0	6	5	0	4
ENSMUSG00000108723	16	6	7	3	6	0	23	0	2	2
ENSMUSG00000108721	0	0	0	0	0	0	0	0	0	0
ENSMUSG00000083359	0	5	4	9	1	9	12	3	0	6
ENSMUSG00000083358	0	0	0	0	0	0	0	0	0	0
ENSMUSG00000083355	11	6	17	9	0	8	19	16	24	0
ENSMUSG00000031471	6	3	3	0	3	0	0	6	3	0
ENSMUSG00000083356	0	0	0	6	0	0	0	0	0	0
ENSMUSG00000083351	0	0	0	0	0	0	0	0	0	0
ENSMUSG00000083350	0	0	0	0	0	0	0	0	0	0
ENSMUSG00000083352	0	0	0	0	0	0	0	0	0	0
ENSMUSG00000107761	0	4	7	0	4	1	8	6	1	0
ENSMUSG00000089029	0	0	0	0	0	0	0	0	0	0
ENSMUSG00000107767	0	0	0	0	0	0	0	0	0	0
ENSMUSG00000104919	0	3	0	0	0	0	0	3	0	0
ENSMUSG00000107765	0	0	0	0	0	0	0	0	0	0
ENSMUSG00000101776	4	0	0	0	0	2	0	0	3	0
ENSMUSG00000092743	0	0	0	0	0	0	0	0	0	0
ENSMUSG00000093905	0	0	0	0	0	0	0	0	0	0

ENSMUSG00000026614	3	0	0	0	0	9	5	3	3	0
ENSMUSG00000093907	1	0	0	0	0	0	0	0	0	0
ENSMUSG00000026616	52	40	60	53	45	52	182	99	121	65
ENSMUSG00000093901	0	0	0	0	0	0	0	0	0	0
ENSMUSG00000093900	0	0	0	0	0	0	0	0	0	0
ENSMUSG00000093903	0	0	0	0	0	0	0	0	0	0
ENSMUSG00000093902	0	0	0	0	0	0	0	0	0	0
ENSMUSG00000064468	0	0	0	0	0	0	0	0	0	0
ENSMUSG00000093909	0	0	0	0	21	0	0	42	0	0
ENSMUSG00000044633	0	3	0	0	0	0	3	3	0	0
ENSMUSG00000044639	0	0	0	0	0	0	0	0	0	0
ENSMUSG00000111976	0	0	0	0	0	0	0	0	0	0
ENSMUSG00000108266	0	0	0	0	0	0	0	0	0	0
ENSMUSG00000045322	40	23	114	116	51	49	151	94	125	87
ENSMUSG00000101170	0	0	0	0	0	0	0	0	0	0
ENSMUSG00000103655	0	0	0	0	0	0	0	0	0	0
ENSMUSG00000045328	252	217	301	397	259	106	363	233	217	159
ENSMUSG00000103654	0	0	0	0	0	0	0	0	0	0
ENSMUSG00000051618	0	0	0	0	0	0	0	0	0	0
ENSMUSG00000079773	0	0	2	6	0	0	5	3	7	4
ENSMUSG00000111453	0	0	0	0	0	0	0	0	0	0
ENSMUSG00000065381	0	0	0	0	0	0	0	0	0	0
ENSMUSG00000111450	0	0	0	0	2	0	0	0	0	0
ENSMUSG00000027564	0	0	0	0	0	0	0	0	0	0
ENSMUSG00000109612	0	0	0	0	0	0	0	0	0	0
ENSMUSG00000027560	33	36	58	33	18	30	124	40	29	46
ENSMUSG00000109615	0	0	0	0	0	0	0	0	0	0
ENSMUSG00000027568	6	0	3	0	3	0	0	0	0	0
ENSMUSG00000109614	0	0	0	0	0	0	0	0	0	0
ENSMUSG00000051611	0	0	0	0	0	6	0	3	0	0
ENSMUSG00000109616	0	0	0	0	0	0	3	3	9	6
ENSMUSG00000090346	0	0	0	0	0	0	0	0	0	0
ENSMUSG00000090342	0	0	0	0	0	0	0	0	0	0
ENSMUSG00000090341	0	0	0	0	0	0	0	0	0	0
ENSMUSG00000048981	0	0	0	0	0	0	0	0	0	0
ENSMUSG00000081240	0	0	0	0	0	0	0	0	0	0
ENSMUSG00000081243	0	0	0	1	0	0	0	0	0	0
ENSMUSG00000048982	0	12	3	3	0	3	9	0	3	0
ENSMUSG00000048988	6	12	27	12	12	12	12	34	4	12
ENSMUSG00000042671	0	0	0	0	3	0	3	0	0	3
ENSMUSG00000099999	0	0	0	0	0	0	0	0	0	0
ENSMUSG00000042677	324	322	305	298	296	345	400	291	348	275
ENSMUSG00000042678	19	0	4	3	3	3	10	5	6	0
ENSMUSG00000099996	0	0	0	0	0	0	0	0	5	2
ENSMUSG00000099990	0	0	0	0	0	0	0	0	0	0
ENSMUSG00000099991	0	0	0	0	0	0	0	0	0	0
ENSMUSG00000099992	0	0	0	0	0	0	0	0	0	0
ENSMUSG00000099993	0	0	0	0	0	0	0	0	0	0
ENSMUSG00000072188	0	0	0	0	5	0	0	3	0	0
ENSMUSG00000046167	5	0	0	0	15	9	26	3	12	14
ENSMUSG00000084999	0	0	0	0	0	0	0	0	0	5
ENSMUSG00000084992	3	3	11	42	21	3	7	30	9	10
ENSMUSG00000084990	0	0	0	0	0	0	3	0	0	0
ENSMUSG00000084996	0	0	0	0	0	0	0	8	0	0
ENSMUSG00000084995	0	0	0	0	0	8	0	2	2	0
ENSMUSG00000098649	0	0	0	0	0	0	0	0	0	0
ENSMUSG00000098642	0	1	0	0	0	0	0	0	0	0
ENSMUSG00000098640	0	0	0	0	0	0	0	0	0	0

ENSMUSG00000098647	0	0	0	0	0	0	0	0	0	0
ENSMUSG00000098644	0	0	0	0	0	0	0	0	0	0
ENSMUSG00000073130	3	3	3	3	6	6	3	13	3	0
ENSMUSG00000114415	3	3	0	3	3	0	0	0	5	0
ENSMUSG00000111427	0	0	0	0	0	0	0	0	0	0
ENSMUSG00000106996	0	0	0	0	0	0	0	0	0	0
ENSMUSG00000078815	57	60	38	43	20	43	9	40	10	52
ENSMUSG00000086736	37	30	36	70	42	34	43	42	52	53
ENSMUSG00000078817	0	2	5	92	21	30	2	18	18	7
ENSMUSG00000078810	111	26	111	888	208	89	26	526	170	257
ENSMUSG00000086732	0	0	0	0	0	0	0	0	0	0
ENSMUSG00000086733	0	0	0	6	0	0	0	0	0	0
ENSMUSG00000063354	0	26	27	48	26	72	67	53	69	41
ENSMUSG00000114414	0	0	0	0	0	0	0	0	0	0
ENSMUSG00000086738	0	0	0	0	0	0	0	0	0	0
ENSMUSG00000086739	0	0	0	0	0	0	0	0	0	0
ENSMUSG00000092506	0	0	0	0	0	0	0	0	0	0
ENSMUSG00000099415	0	0	0	0	0	0	0	0	0	0
ENSMUSG00000099414	0	0	0	0	0	0	0	0	0	0
ENSMUSG00000049761	0	0	3	6	6	0	2	0	0	2
ENSMUSG00000082291	0	0	0	0	0	0	0	2	0	0
ENSMUSG00000082297	0	0	0	0	0	0	0	0	0	0
ENSMUSG00000082295	0	0	0	0	0	0	0	0	0	0
ENSMUSG00000082294	0	0	0	0	0	0	0	0	0	0
ENSMUSG00000089731	0	0	0	0	0	0	1	0	0	0
ENSMUSG00000092441	0	0	0	0	0	0	0	0	0	0
ENSMUSG00000092440	0	0	0	0	0	0	0	0	0	0
ENSMUSG00000099418	2	0	0	0	0	3	4	4	4	0
ENSMUSG00000043692	0	0	0	0	0	0	0	0	0	0
ENSMUSG00000067941	3	0	3	0	0	0	0	0	0	0
ENSMUSG00000030577	18	23	16	14	17	38	231	79	153	119
ENSMUSG00000114413	0	0	0	0	0	0	6	0	0	0
ENSMUSG00000101440	0	0	0	0	0	0	0	0	0	0
ENSMUSG00000101441	0	0	0	0	0	0	0	0	0	0
ENSMUSG00000101442	0	0	0	0	0	0	0	0	0	0
ENSMUSG00000101443	0	0	0	0	0	0	0	0	0	0
ENSMUSG00000101444	0	0	0	0	0	0	0	0	0	0
ENSMUSG00000020286	0	0	0	0	0	0	2	0	0	3
ENSMUSG00000101446	0	0	0	0	0	0	0	0	0	0
ENSMUSG00000101447	0	0	0	0	0	0	0	0	0	0
ENSMUSG00000101448	0	0	0	0	0	0	0	0	0	0
ENSMUSG00000080829	0	0	0	0	2	0	0	0	3	0
ENSMUSG00000040525	15	17	15	19	9	18	7	7	12	11
ENSMUSG00000019489	0	0	0	0	0	0	0	0	0	0
ENSMUSG00000083830	0	0	0	0	0	0	0	0	0	0
ENSMUSG00000107848	0	0	0	0	0	0	0	0	0	0
ENSMUSG00000107849	0	2	0	0	3	0	0	0	0	0
ENSMUSG00000107842	0	0	0	0	0	0	2	0	0	0
ENSMUSG00000107840	0	0	0	0	0	0	0	0	0	0
ENSMUSG00000107841	14	18	12	12	6	14	6	9	10	16
ENSMUSG00000107847	0	0	0	0	0	0	0	0	0	0
ENSMUSG00000107844	0	0	0	0	0	0	0	0	0	0
ENSMUSG00000107845	0	0	0	0	0	0	0	0	0	0
ENSMUSG00000103358	0	6	0	0	0	0	0	0	9	0
ENSMUSG00000103352	0	0	0	0	0	0	0	0	0	0
ENSMUSG00000103353	0	0	0	0	0	0	0	0	0	0
ENSMUSG00000103350	0	0	0	0	0	0	0	0	0	0
ENSMUSG00000103351	0	0	0	0	0	0	0	0	0	0

ENSMUSG00000103357	6	2	1	2	0	0	4	8	1	2
ENSMUSG00000103354	0	0	6	0	0	3	3	0	0	0
ENSMUSG00000082125	0	0	0	0	0	0	0	0	0	0
ENSMUSG00000082126	0	0	0	0	0	0	0	0	0	0
ENSMUSG00000082122	0	0	0	0	0	0	0	0	0	0
ENSMUSG00000082129	0	0	0	0	0	0	1	0	0	0
ENSMUSG00000099205	0	0	0	0	0	0	0	0	0	0
ENSMUSG00000099201	0	0	0	0	0	0	0	0	0	0
ENSMUSG00000099203	0	0	0	0	0	0	0	0	0	0
ENSMUSG00000099208	0	0	0	0	0	0	0	0	0	0
ENSMUSG00000014164	604	497	532	621	452	566	466	584	439	510
ENSMUSG00000043485	0	0	0	0	0	0	0	0	0	0
ENSMUSG00000032623	0	0	0	0	0	0	0	0	0	0
ENSMUSG00000089270	1	0	0	1	2	0	0	0	0	0
ENSMUSG00000083655	9	13	5	10	27	16	14	14	18	25
ENSMUSG00000089272	0	0	0	0	0	0	0	0	0	0
ENSMUSG00000089273	2	3	0	0	0	0	5	0	4	0
ENSMUSG00000089274	0	0	0	0	0	0	0	0	0	0
ENSMUSG00000084607	4	2	0	1	0	1	3	1	2	6
ENSMUSG00000084604	0	1	0	1	0	0	1	1	1	0
ENSMUSG00000089277	0	0	0	0	0	0	0	0	0	0
ENSMUSG00000089278	0	0	0	0	0	0	0	0	0	0
ENSMUSG00000089279	0	0	0	0	0	0	0	0	0	0
ENSMUSG00000084608	0	0	0	0	0	0	0	0	0	0
ENSMUSG00000084609	0	0	0	0	0	0	0	0	0	0
ENSMUSG00000083659	0	0	0	0	0	0	0	0	0	0
ENSMUSG00000100150	4	0	3	3	4	3	9	13	21	7
ENSMUSG00000064939	0	0	0	0	0	0	0	0	0	0
ENSMUSG00000096386	0	0	0	0	0	0	0	0	0	0
ENSMUSG00000100718	0	0	0	0	0	0	0	0	0	0
ENSMUSG00000100717	0	0	0	0	0	0	0	1	0	0
ENSMUSG00000100716	0	0	0	0	0	0	0	0	0	0
ENSMUSG00000100715	0	0	0	0	0	0	0	0	0	0
ENSMUSG00000114218	0	0	0	0	0	0	0	0	0	0
ENSMUSG00000100713	0	0	0	0	0	0	0	0	0	0
ENSMUSG00000100712	0	0	0	0	0	0	0	0	0	0
ENSMUSG00000100711	0	0	0	0	0	0	0	0	0	0
ENSMUSG00000100710	0	0	0	0	0	0	0	0	0	0
ENSMUSG00000102408	0	0	0	0	0	0	0	0	0	0
ENSMUSG00000102405	0	0	0	0	0	3	2	0	6	3
ENSMUSG00000102407	0	0	0	0	0	0	0	0	0	0
ENSMUSG00000102406	0	0	0	0	0	0	0	0	0	0
ENSMUSG00000087946	0	0	0	0	0	0	0	0	0	0
ENSMUSG00000000365	96	56	113	96	62	55	148	126	68	141
ENSMUSG00000097677	0	0	0	0	0	0	0	0	0	0
ENSMUSG00000082314	0	0	0	2	0	0	0	0	0	0
ENSMUSG00000041347	3	3	3	1	0	22	6	14	10	0
ENSMUSG00000087957	0	0	0	0	0	0	0	0	0	0
ENSMUSG00000087954	0	0	0	0	0	0	0	0	0	0
ENSMUSG00000087958	0	0	0	0	0	0	0	0	0	0
ENSMUSG00000085551	0	0	0	0	0	0	0	0	0	0
ENSMUSG00000085553	0	0	0	0	0	0	0	0	2	0
ENSMUSG00000085552	0	0	0	0	0	0	0	0	0	0
ENSMUSG00000085555	2	0	0	0	0	0	0	0	0	0
ENSMUSG00000085556	0	0	0	0	0	0	0	0	0	0
ENSMUSG00000064550	0	0	0	0	0	0	0	0	0	0
ENSMUSG00000085558	65	60	103	98	68	138	175	72	116	61
ENSMUSG00000064554	0	0	0	0	0	0	0	0	0	0

ENSMUSG00000064555	0	0	0	0	0	0	0	0	0	0
ENSMUSG00000107159	0	0	0	0	0	0	0	0	3	0
ENSMUSG00000112408	0	0	0	0	0	0	0	0	0	0
ENSMUSG00000107153	0	0	0	0	0	0	0	6	0	0
ENSMUSG00000020798	12	3	28	21	3	5	68	15	14	12
ENSMUSG00000107157	1	1	0	0	0	8	6	2	1	5
ENSMUSG00000020799	6	0	0	0	6	0	3	5	0	13
ENSMUSG00000023073	6	0	0	27	0	0	0	0	0	0
ENSMUSG00000023070	69	33	48	64	48	51	26	29	15	50
ENSMUSG00000088603	0	0	0	0	0	0	0	0	0	0
ENSMUSG00000088601	0	0	0	2	0	0	0	0	0	0
ENSMUSG00000095768	0	0	0	0	0	0	0	0	0	0
ENSMUSG00000023083	3	10	0	2	5	7	0	0	8	14
ENSMUSG00000021506	0	0	0	0	0	0	0	0	0	0
ENSMUSG00000084725	0	0	0	0	0	0	0	0	0	0
ENSMUSG00000021509	3	0	0	0	0	0	15	6	3	0
ENSMUSG00000074720	0	0	0	0	0	0	0	0	0	0
ENSMUSG00000095764	0	0	0	0	0	0	0	0	0	0
ENSMUSG00000061457	0	0	0	0	0	0	0	0	0	0
ENSMUSG00000029736	6	0	0	2	0	0	0	0	0	0
ENSMUSG00000029735	246	208	190	255	152	154	197	105	307	269
ENSMUSG00000112250	0	0	0	0	0	0	0	0	0	0
ENSMUSG00000032415	108	129	130	143	98	117	97	111	84	78
ENSMUSG00000103438	0	0	0	0	0	0	2	0	0	0
ENSMUSG00000103437	0	0	0	0	0	0	0	3	0	0
ENSMUSG00000103434	0	0	0	0	0	0	0	0	0	0
ENSMUSG00000103433	0	0	0	0	0	0	0	0	0	1
ENSMUSG00000106400	0	0	0	0	0	0	0	0	0	0
ENSMUSG00000103431	0	0	0	0	0	0	0	0	0	0
ENSMUSG00000106402	0	0	0	0	0	0	0	0	0	0
ENSMUSG00000097349	0	0	0	0	0	0	0	0	0	0
ENSMUSG00000022857	0	0	0	0	0	0	0	0	0	0
ENSMUSG00000097346	0	3	0	0	0	0	42	0	2	0
ENSMUSG00000097344	0	3	6	3	3	0	9	0	0	3
ENSMUSG00000069785	4	6	5	0	0	2	0	0	0	0
ENSMUSG00000070136	0	0	0	0	0	0	2	0	0	0
ENSMUSG00000029086	153	188	177	232	171	164	162	85	100	181
ENSMUSG00000066158	0	0	0	0	0	0	0	0	0	0
ENSMUSG00000066153	0	0	0	0	0	0	0	0	0	2
ENSMUSG00000066154	0	0	11	0	0	0	0	6	6	11
ENSMUSG00000097691	0	0	0	0	0	0	0	0	0	0
ENSMUSG00000097695	0	1	1	1	1	0	0	0	1	3
ENSMUSG00000097694	0	0	3	17	3	0	5	0	3	1
ENSMUSG00000097697	0	6	3	9	3	24	89	26	35	14
ENSMUSG00000034087	0	0	0	0	0	0	0	0	0	0
ENSMUSG00000069873	0	0	0	0	0	0	0	0	0	0
ENSMUSG00000051652	64	55	59	29	47	60	91	72	68	80
ENSMUSG00000099104	0	6	1	3	1	16	0	3	7	0
ENSMUSG00000109659	0	0	0	0	0	0	0	0	0	0
ENSMUSG00000102389	12	5	10	0	0	3	18	9	13	9
ENSMUSG00000102386	0	0	0	0	0	0	0	0	0	0
ENSMUSG00000109654	6	7	17	28	20	8	18	8	23	12
ENSMUSG00000102385	2	0	2	0	0	3	3	2	3	3
ENSMUSG00000102382	0	0	0	0	0	0	0	0	0	0
ENSMUSG00000105171	0	0	3	0	0	0	0	0	0	0
ENSMUSG00000114250	0	0	0	0	0	0	0	0	0	0
ENSMUSG00000114257	0	0	0	0	0	0	0	3	6	0
ENSMUSG00000114259	0	0	0	0	0	0	0	0	0	0

ENSMUSG00000114258	0	2	0	0	0	0	0	0	0	0	
ENSMUSG00000112949	0	0	0	0	0	0	0	0	0	0	
ENSMUSG00000108529	0	0	0	0	0	0	0	0	0	0	
ENSMUSG00000108525	9	0	3	1	0	5	3	3	3	2	
ENSMUSG00000108520	24	17	18	12	15	5	23	9	18	16	
ENSMUSG00000112940	0	0	0	0	0	0	0	0	0	0	
ENSMUSG00000112943	0	0	0	0	0	0	0	0	0	0	
ENSMUSG00000067201	0	0	0	0	0	15	70	18	55	21	
ENSMUSG00000110319	0	0	0	0	0	0	0	0	0	0	
ENSMUSG00000072721	7	2	0	4	0	0	0	0	4	0	
ENSMUSG00000100126	0	0	0	0	0	0	0	0	0	0	
ENSMUSG00000100128	0	0	0	0	0	0	0	3	0	0	
ENSMUSG00000050296	1593	889	1092	910	1192	716	433	783	458	686	875.2
ENSMUSG00000044217	5	0	5	3	6	8	182	26	27	18	
ENSMUSG00000091692	0	0	0	0	0	0	0	0	0	0	
ENSMUSG00000091698	0	0	0	0	0	0	0	0	0	0	
ENSMUSG00000096569	0	0	0	0	0	0	0	0	0	0	
ENSMUSG00000021087	92	101	90	122	68	93	210	95	71	143	
ENSMUSG00000100719	0	0	0	0	0	0	0	0	0	0	
ENSMUSG00000057116	0	0	0	0	0	0	0	0	0	0	
ENSMUSG00000110462	3	0	6	4	3	3	0	6	0	0	
ENSMUSG00000110463	0	0	0	0	0	0	0	0	0	0	
ENSMUSG00000110464	0	0	0	0	0	0	0	0	0	0	
ENSMUSG00000101127	0	0	0	0	0	0	0	0	0	0	
ENSMUSG00000101126	0	0	0	6	0	0	0	3	0	0	
ENSMUSG00000101125	0	0	0	0	0	0	0	0	0	0	
ENSMUSG00000101124	0	0	0	0	0	0	0	0	0	0	
ENSMUSG00000101123	0	0	0	0	0	0	0	0	0	0	
ENSMUSG00000101120	0	0	0	0	0	0	0	0	0	0	
ENSMUSG00000056863	0	0	0	0	0	0	0	0	0	0	
ENSMUSG00000101128	0	0	0	0	0	0	0	0	0	0	
ENSMUSG00000026368	0	0	0	9	0	0	9	0	0	0	
ENSMUSG00000045126	0	0	0	0	0	0	0	0	0	0	
ENSMUSG00000111373	0	0	3	2	0	0	0	0	0	0	
ENSMUSG00000064961	0	0	0	0	0	0	0	0	0	0	
ENSMUSG00000060260	476	509	538	543	441	368	497	446	500	415	
ENSMUSG00000091034	0	0	2	0	1	1	5	0	1	2	
ENSMUSG00000091031	0	0	0	0	0	0	0	0	0	0	
ENSMUSG00000045693	0	0	0	0	0	0	0	0	0	0	
ENSMUSG00000091039	0	0	0	0	0	0	0	0	0	0	
ENSMUSG00000026943	0	0	0	0	0	0	0	0	0	0	
ENSMUSG00000100714	0	0	0	0	0	0	3	0	0	0	
ENSMUSG00000056821	0	0	0	0	0	0	3	0	0	3	
ENSMUSG00000081001	0	0	0	0	0	0	0	0	0	0	
ENSMUSG00000081000	0	0	0	0	0	0	0	0	0	0	
ENSMUSG00000081007	0	0	0	0	0	0	0	0	0	0	
ENSMUSG00000081006	0	0	0	0	0	0	0	0	2	0	
ENSMUSG00000081004	1	5	4	1	3	3	8	0	1	2	
ENSMUSG00000081009	0	0	0	0	0	0	0	0	0	0	
ENSMUSG00000042477	6	8	0	6	0	3	2	9	0	3	
ENSMUSG00000027015	88	131	218	68	86	145	274	170	55	150	
ENSMUSG00000042474	6	6	8	9	6	4	85	39	56	43	
ENSMUSG00000022061	0	0	0	0	3	0	0	3	0	0	
ENSMUSG00000113630	2	3	12	11	5	27	21	11	28	10	
ENSMUSG00000111993	3	0	0	3	0	0	3	0	0	0	
ENSMUSG00000111990	0	0	0	0	0	0	0	0	0	0	
ENSMUSG00000111999	0	0	0	0	0	0	0	0	0	0	
ENSMUSG00000111998	0	0	0	0	0	0	0	0	0	0	

ENSMUSG00000053742	0	0	0	0	0	0	0	0	0	0
ENSMUSG00000053747	0	0	0	0	0	0	0	0	0	0
ENSMUSG00000096889	0	0	1	0	0	0	0	0	0	0
ENSMUSG00000094404	0	0	0	0	0	0	0	0	0	0
ENSMUSG00000015314	11	13	23	20	20	15	83	31	45	43
ENSMUSG00000084418	0	0	0	0	0	0	1	0	0	0
ENSMUSG00000094407	0	0	0	0	0	0	0	0	0	0
ENSMUSG00000096524	0	0	0	0	0	0	0	0	0	0
ENSMUSG00000104939	0	0	0	0	0	0	0	0	0	0
ENSMUSG00000077125	0	0	0	0	0	0	0	0	0	0
ENSMUSG00000055748	8	0	0	0	0	0	6	0	3	0
ENSMUSG00000055746	0	0	0	0	0	0	0	0	0	0
ENSMUSG00000049565	0	5	3	0	3	15	18	21	8	3
ENSMUSG00000049560	0	0	0	0	0	0	0	0	0	0
ENSMUSG00000049561	0	0	0	0	0	0	0	0	0	0
ENSMUSG00000049562	309	324	386	445	320	295	494	366	404	333
ENSMUSG00000072575	0	0	0	0	0	0	0	0	0	0
ENSMUSG00000072573	14	38	31	19	25	18	26	26	27	43
ENSMUSG00000082301	0	0	0	0	0	0	0	0	0	0
ENSMUSG00000092674	0	1	0	0	0	0	0	0	0	0
ENSMUSG00000086501	0	0	3	0	0	1	0	5	0	0
ENSMUSG00000100697	0	0	0	0	0	0	0	0	0	0
ENSMUSG00000045903	0	6	3	0	0	6	6	0	12	9
ENSMUSG00000104757	0	0	0	0	0	0	0	0	0	0
ENSMUSG00000096950	7	2	14	16	6	14	25	14	11	12
ENSMUSG00000096955	0	0	0	0	0	0	0	0	1	0
ENSMUSG00000098938	0	0	0	0	0	0	0	0	0	0
ENSMUSG00000098935	0	0	0	0	0	0	0	0	0	0
ENSMUSG00000098937	0	0	0	0	0	0	0	0	0	0
ENSMUSG00000112513	0	0	5	0	0	0	0	0	0	0
ENSMUSG00000106365	2	0	9	13	6	0	12	12	15	11
ENSMUSG00000113268	0	3	0	9	3	3	6	0	3	0
ENSMUSG00000106360	0	0	0	0	0	0	0	0	0	0
ENSMUSG00000106361	0	0	0	0	0	0	0	0	0	0
ENSMUSG00000106363	0	0	0	0	0	0	0	0	0	0
ENSMUSG00000054836	174	138	166	137	107	155	181	186	179	153
ENSMUSG00000113260	0	0	0	0	0	0	0	0	0	0
ENSMUSG00000113267	0	0	0	2	0	0	0	0	0	0
ENSMUSG00000113266	0	0	0	0	0	0	0	0	0	0
ENSMUSG00000113265	0	0	0	0	0	0	0	0	0	0
ENSMUSG00000071000	0	0	0	0	0	0	0	0	0	0
ENSMUSG00000100554	0	0	0	0	0	2	0	0	0	0
ENSMUSG00000109354	0	0	0	0	0	0	0	0	0	0
ENSMUSG00000109355	0	0	0	0	0	0	0	0	0	0
ENSMUSG00000109356	0	0	0	0	0	0	0	0	0	0
ENSMUSG00000109351	0	0	0	0	0	0	0	0	3	0
ENSMUSG00000109359	0	0	0	0	0	0	0	0	0	0
ENSMUSG00000082723	0	0	0	0	0	0	0	0	0	0
ENSMUSG00000076385	0	0	0	0	0	0	0	0	0	0
ENSMUSG00000082727	0	0	0	0	0	0	0	0	0	0
ENSMUSG00000082728	0	0	0	0	0	0	0	0	0	0
ENSMUSG00000076389	0	0	0	0	0	0	0	0	0	0
ENSMUSG00000047228	2	3	0	0	3	3	12	0	0	0
ENSMUSG00000110288	0	0	0	0	0	0	0	0	0	0
ENSMUSG00000092097	12	9	12	7	4	8	9	11	8	5
ENSMUSG00000092090	5	0	2	0	0	0	0	3	0	0
ENSMUSG00000092093	0	0	3	1	0	0	0	0	0	3
ENSMUSG00000047220	3	0	3	4	0	0	3	3	0	0

ENSMUSG00000047222	0	0	0	0	3	0	3	3	0	0
ENSMUSG00000047225	0	0	0	0	0	0	0	0	0	0
ENSMUSG00000083093	2	0	0	1	0	0	0	0	0	2
ENSMUSG00000083090	0	0	0	0	2	0	0	0	0	0
ENSMUSG00000083091	0	0	0	0	0	0	0	0	0	0
ENSMUSG00000083096	0	0	0	0	0	0	0	0	0	0
ENSMUSG00000083094	0	0	0	0	0	0	0	0	0	0
ENSMUSG00000083098	0	0	0	0	0	0	0	0	0	0
ENSMUSG00000083099	0	0	0	0	0	0	0	0	0	0
ENSMUSG00000098437	0	0	0	0	0	0	0	0	0	0
ENSMUSG00000093223	0	1	0	0	0	2	0	0	0	0
ENSMUSG00000099322	0	0	0	0	0	0	0	0	0	0
ENSMUSG00000099321	0	0	0	0	0	0	0	0	0	0
ENSMUSG00000099326	0	0	0	0	0	0	0	0	0	0
ENSMUSG00000005131	20	32	11	20	12	47	81	14	36	26
ENSMUSG00000099328	0	0	0	0	0	0	0	0	0	0
ENSMUSG00000084378	0	0	0	0	0	0	0	0	0	0
ENSMUSG00000084379	0	0	0	0	0	3	0	0	0	0
ENSMUSG00000084372	0	0	0	0	0	0	0	0	0	0
ENSMUSG00000084370	0	0	0	0	0	0	8	0	0	0
ENSMUSG00000084371	0	0	0	0	0	0	0	0	0	0
ENSMUSG00000084376	0	0	0	0	0	0	0	0	0	0
ENSMUSG00000084377	0	0	0	0	0	0	0	2	0	0
ENSMUSG00000084374	0	0	0	0	0	0	0	0	0	0
ENSMUSG00000084375	0	0	0	0	0	0	0	0	0	0
ENSMUSG00000000560	0	0	0	0	0	0	0	0	0	0
ENSMUSG00000099848	0	0	0	0	0	0	0	0	0	0
ENSMUSG00000083197	0	0	0	0	0	0	0	0	0	0
ENSMUSG00000025577	268	336	373	315	334	268	366	384	366	367
ENSMUSG00000025576	5	0	0	6	3	8	0	13	3	3
ENSMUSG00000025573	2	2	3	0	0	0	5	7	2	6
ENSMUSG00000082929	0	0	0	0	0	0	0	0	0	0
ENSMUSG00000025578	380	420	370	300	292	244	404	396	423	421
ENSMUSG00000100552	0	0	0	0	0	0	0	0	0	0
ENSMUSG00000107473	0	0	0	0	0	0	0	0	0	0
ENSMUSG00000107471	0	0	0	0	0	0	0	0	0	0
ENSMUSG00000065028	0	0	0	0	0	0	0	0	0	0
ENSMUSG00000079174	0	0	0	0	0	0	0	0	0	0
ENSMUSG00000079175	0	0	0	0	0	0	0	0	0	0
ENSMUSG00000079170	0	0	0	0	0	0	0	0	0	0
ENSMUSG00000079171	0	0	0	0	0	0	0	0	0	0
ENSMUSG00000085021	3	2	0	0	0	0	0	0	0	0
ENSMUSG00000085020	0	3	0	0	0	0	4	6	0	0
ENSMUSG00000085025	14	20	12	28	18	15	24	17	17	10
ENSMUSG00000085027	4	0	0	0	0	0	0	0	0	0
ENSMUSG00000085026	0	0	0	0	0	0	0	0	0	0
ENSMUSG00000065042	0	0	0	0	0	0	0	0	0	0
ENSMUSG00000107130	0	0	0	0	0	0	0	0	0	0
ENSMUSG00000033850	0	0	0	0	0	0	0	0	0	0
ENSMUSG00000113462	0	0	0	0	0	0	0	0	0	0
ENSMUSG00000033854	3	3	0	6	0	5	21	9	0	0
ENSMUSG00000033857	300	226	341	334	333	374	399	383	423	539
ENSMUSG00000079522	0	0	0	0	0	0	0	0	0	0
ENSMUSG00000093335	0	0	0	0	0	0	0	0	0	0
ENSMUSG00000108356	0	0	0	0	0	0	0	0	0	0
ENSMUSG00000084476	0	0	0	0	0	0	0	0	0	1
ENSMUSG00000101267	0	0	0	0	0	0	0	0	0	0
ENSMUSG00000113469	0	0	0	0	0	0	0	0	0	0

ENSMUSG00000112490	0	0	0	0	0	0	0	0	0	0
ENSMUSG00000099810	0	0	0	0	0	0	0	2	0	0
ENSMUSG00000099815	0	0	0	0	0	0	0	0	0	0
ENSMUSG00000099814	0	0	0	0	0	0	0	0	0	0
ENSMUSG00000099817	0	0	0	0	0	0	0	0	0	0
ENSMUSG00000099816	0	0	0	0	0	0	0	0	0	0
ENSMUSG00000099818	0	0	0	0	0	0	0	0	0	0
ENSMUSG00000059077	0	0	0	0	0	0	0	0	0	0
ENSMUSG00000100558	0	3	0	0	0	0	0	0	0	0
ENSMUSG00000106303	0	0	0	0	0	0	0	0	0	0
ENSMUSG00000019913	0	0	0	6	0	0	0	0	0	0
ENSMUSG00000104110	0	0	0	0	0	0	0	0	0	0
ENSMUSG00000072962	3	0	2	2	3	6	0	3	0	6
ENSMUSG00000072963	3	3	0	0	0	9	3	8	0	3
ENSMUSG00000020213	0	0	0	0	0	0	0	0	0	0
ENSMUSG00000072968	0	3	0	0	0	0	0	0	0	0
ENSMUSG00000094400	0	0	0	0	0	0	0	0	0	0
ENSMUSG00000077244	0	0	0	0	0	0	0	0	0	0
ENSMUSG00000077246	0	0	0	0	0	0	0	0	0	0
ENSMUSG00000077240	0	0	0	0	0	0	0	0	0	0
ENSMUSG00000077241	0	0	0	0	0	0	0	0	0	0
ENSMUSG00000082666	0	0	0	0	0	0	0	0	0	0
ENSMUSG00000077243	0	0	0	0	0	0	0	0	0	0
ENSMUSG00000077248	0	0	0	0	0	0	1	0	1	0
ENSMUSG00000035842	251	169	168	173	158	140	270	234	242	197
ENSMUSG00000032818	0	0	0	0	0	0	0	0	0	0
ENSMUSG00000109919	0	0	0	0	0	0	0	0	0	0
ENSMUSG00000089828	0	0	0	0	0	0	0	0	0	0
ENSMUSG00000084818	0	0	0	0	0	0	0	0	0	0
ENSMUSG00000089826	0	0	0	0	0	0	0	0	0	0
ENSMUSG00000111195	0	0	0	0	0	0	0	0	0	0
ENSMUSG00000084811	0	0	0	0	0	0	3	0	0	0
ENSMUSG00000089822	0	0	0	0	0	0	0	0	0	0
ENSMUSG00000031549	3	0	6	6	6	3	2	3	0	4
ENSMUSG00000037537	0	3	0	0	0	0	0	0	0	2
ENSMUSG00000094165	2	0	0	0	0	0	5	0	0	0
ENSMUSG00000114115	0	0	0	0	0	0	0	3	0	0
ENSMUSG00000114111	0	0	0	0	0	0	0	0	0	0
ENSMUSG00000093957	0	0	0	0	0	0	0	0	0	0
ENSMUSG00000093113	0	0	0	0	0	0	0	0	0	0
ENSMUSG00000101578	0	0	0	0	0	0	0	0	0	0
ENSMUSG00000088067	0	0	0	0	0	0	0	0	0	0
ENSMUSG00000088064	0	0	0	0	0	0	0	0	0	0
ENSMUSG00000088063	0	0	0	0	0	0	0	0	0	0
ENSMUSG00000088060	0	0	0	0	0	0	0	0	0	0
ENSMUSG00000082660	0	0	0	0	0	0	0	0	0	0
ENSMUSG00000085858	0	0	0	0	0	0	0	3	3	0
ENSMUSG00000111334	8	17	18	7	10	3	26	23	5	25
ENSMUSG00000085522	0	0	0	0	0	0	0	0	0	0
ENSMUSG00000111871	0	0	0	3	0	0	0	3	3	0
ENSMUSG00000109099	0	0	0	0	0	0	0	0	0	0
ENSMUSG00000085856	0	0	0	0	0	0	0	0	0	0
ENSMUSG00000093116	0	0	0	0	0	0	2	0	0	0
ENSMUSG00000112722	0	0	0	0	0	0	0	0	0	0
ENSMUSG00000106309	0	0	0	0	0	0	0	0	0	0
ENSMUSG00000033036	3	5	4	6	2	3	12	8	5	5
ENSMUSG00000103259	0	3	0	0	0	0	4	2	0	0
ENSMUSG00000061798	0	0	0	0	0	0	0	0	0	0

ENSMUSG00000103257	0	0	0	0	0	0	0	0	0	0
ENSMUSG00000103250	0	0	0	0	0	0	0	0	0	0
ENSMUSG00000087561	0	0	0	0	0	0	0	0	0	0
ENSMUSG00000088398	0	3	0	0	0	0	0	0	0	1
ENSMUSG00000087568	0	0	0	0	2	0	0	0	0	0
ENSMUSG00000040614	0	0	0	0	0	0	0	0	0	0
ENSMUSG00000013611	0	0	0	0	0	0	0	3	3	3
ENSMUSG00000082664	0	0	0	0	0	0	0	0	0	0
ENSMUSG00000081044	0	0	0	0	0	0	5	0	0	0
ENSMUSG00000065034	0	0	0	0	0	0	0	0	0	0
ENSMUSG00000051726	0	0	6	0	0	0	3	3	0	0
ENSMUSG00000100508	0	0	0	0	0	0	0	0	0	0
ENSMUSG00000100509	0	0	0	0	0	0	0	0	0	0
ENSMUSG00000100506	0	0	0	0	0	0	0	0	0	0
ENSMUSG00000100507	0	0	0	0	0	0	0	0	0	0
ENSMUSG00000100504	0	0	0	0	0	0	0	0	0	0
ENSMUSG00000100505	35	40	38	31	34	63	73	51	45	52
ENSMUSG00000100502	3	7	5	0	0	4	3	2	5	0
ENSMUSG00000100503	0	0	0	0	0	0	0	0	0	0
ENSMUSG00000100500	0	0	0	0	0	0	0	0	0	0
ENSMUSG00000100501	0	0	0	0	0	0	0	0	0	0
ENSMUSG00000022269	3	9	3	9	3	3	4	6	3	9
ENSMUSG00000019146	0	0	0	0	0	0	0	0	0	0
ENSMUSG00000022262	0	6	3	0	0	0	18	15	21	0
ENSMUSG00000084031	0	0	0	0	0	0	0	0	0	0
ENSMUSG00000084032	1	0	0	0	0	0	0	0	0	0
ENSMUSG00000084034	0	0	0	0	0	0	0	0	0	0
ENSMUSG00000084039	0	0	0	0	0	0	0	0	0	0
ENSMUSG00000084038	0	0	0	0	0	0	0	0	0	0
ENSMUSG00000111148	0	0	0	0	0	0	0	0	0	0
ENSMUSG00000065509	0	5	2	2	2	5	8	2	1	5
ENSMUSG00000095843	0	0	0	0	0	0	0	0	0	0
ENSMUSG00000095841	0	0	0	0	0	0	0	0	0	0
ENSMUSG00000061080	653	272	233	163	469	569	367	509	310	212
ENSMUSG00000095849	0	0	0	0	0	0	0	0	0	0
ENSMUSG00000106314	0	0	0	0	0	0	0	0	0	0
ENSMUSG00000037263	17	24	20	20	39	28	41	36	20	14
ENSMUSG00000024957	2	3	0	3	0	8	3	4	0	4
ENSMUSG00000058904	0	0	0	0	0	0	0	0	0	0
ENSMUSG00000102618	0	0	0	0	3	0	0	0	0	0
ENSMUSG00000102619	7	5	6	3	2	3	3	11	9	0
ENSMUSG00000102610	0	0	0	0	0	0	0	0	0	0
ENSMUSG00000112333	0	0	0	0	0	0	0	0	0	0
ENSMUSG00000112330	0	0	0	0	0	0	0	0	0	0
ENSMUSG00000102613	0	0	0	0	0	0	0	0	0	0
ENSMUSG00000102614	0	0	0	0	0	0	0	0	0	0
ENSMUSG00000112337	0	0	0	0	3	0	0	0	0	4
ENSMUSG00000039154	8	19	11	26	6	33	18	48	38	12
ENSMUSG00000039155	0	2	0	3	10	0	9	12	0	2
ENSMUSG00000035780	0	0	0	0	0	0	0	0	0	3
ENSMUSG00000035785	0	0	0	0	0	0	0	0	0	0
ENSMUSG00000036872	0	3	3	0	0	4	3	0	3	0
ENSMUSG00000036875	139	90	177	196	94	141	211	161	141	142
ENSMUSG00000028655	3	0	3	0	0	8	12	0	8	3
ENSMUSG00000028654	28	33	25	18	25	66	259	65	153	54
ENSMUSG00000093251	0	0	0	1	0	0	0	0	0	1
ENSMUSG00000038209	0	0	0	0	0	0	0	0	0	0
ENSMUSG00000106228	0	0	0	0	0	0	0	0	0	0

ENSMUSG00000038203	0	0	0	0	0	0	0	3	0	6
ENSMUSG00000113062	0	0	0	0	0	0	0	0	0	0
ENSMUSG00000034785	0	0	0	0	0	0	0	0	0	3
ENSMUSG00000113066	0	0	0	0	0	2	0	0	0	0
ENSMUSG00000063626	0	3	3	3	0	9	3	0	0	0
ENSMUSG00000051190	0	0	0	0	0	0	0	0	0	0
ENSMUSG00000114599	0	0	0	0	0	0	0	4	0	0
ENSMUSG00000111511	0	0	0	0	0	0	3	0	0	0
ENSMUSG00000111510	0	0	0	0	0	0	0	0	0	0
ENSMUSG00000111517	0	0	0	0	0	0	0	0	0	0
ENSMUSG00000109595	0	0	0	0	0	0	0	0	0	0
ENSMUSG00000114590	0	0	0	0	0	3	0	0	0	0
ENSMUSG00000114591	3	2	6	5	1	3	13	3	5	9
ENSMUSG00000109598	0	0	0	0	0	3	0	0	0	0
ENSMUSG00000111518	0	0	0	0	0	0	0	0	0	0
ENSMUSG00000114594	0	0	0	0	0	0	0	0	0	0
ENSMUSG00000114597	0	0	0	0	0	0	0	0	0	0
ENSMUSG00000106618	0	0	0	0	0	0	0	0	0	0
ENSMUSG00000106619	0	0	0	0	0	0	0	0	0	0
ENSMUSG00000106616	0	0	0	0	0	0	0	0	0	0
ENSMUSG00000050822	4	0	3	12	19	0	3	20	23	19
ENSMUSG00000067795	0	0	0	0	0	3	0	0	4	0
ENSMUSG00000050824	0	0	0	0	0	0	0	0	0	0
ENSMUSG00000095603	0	0	0	0	0	0	0	0	0	0
ENSMUSG00000051401	10	24	22	17	25	17	27	46	47	14
ENSMUSG00000104616	0	0	2	0	0	0	2	0	0	0
ENSMUSG00000104617	0	0	0	0	0	0	0	0	0	0
ENSMUSG00000104614	0	0	0	0	0	0	0	0	0	0
ENSMUSG00000104610	0	0	0	0	0	0	0	0	0	0
ENSMUSG00000059695	0	0	0	0	0	0	0	0	0	0
ENSMUSG00000104619	0	0	0	0	0	0	0	0	0	0
ENSMUSG00000096048	0	0	0	0	0	0	0	0	0	0
ENSMUSG00000096907	0	0	0	0	0	0	0	0	0	0
ENSMUSG00000114897	0	0	0	0	0	0	0	0	0	2
ENSMUSG00000065048	0	0	0	0	0	0	0	0	0	0
ENSMUSG00000096905	0	0	0	1	0	0	0	0	0	0
ENSMUSG00000097559	24	23	17	32	21	22	46	18	43	43
ENSMUSG00000097557	0	10	4	2	2	8	0	6	3	7
ENSMUSG00000097552	0	0	0	0	0	0	0	0	0	0
ENSMUSG00000097550	0	0	0	0	0	0	0	0	0	0
ENSMUSG00000097551	0	0	0	0	0	0	0	0	0	0
ENSMUSG00000067010	0	0	0	0	0	0	0	0	0	0
ENSMUSG00000114042	0	3	0	0	0	0	3	0	0	0
ENSMUSG00000114043	0	0	0	0	0	0	0	0	0	0
ENSMUSG00000114041	6	0	0	0	0	0	11	2	0	0
ENSMUSG00000114047	0	0	0	0	0	0	0	3	0	0
ENSMUSG00000114044	0	0	0	0	0	0	0	0	0	0
ENSMUSG00000110380	0	0	0	0	0	3	0	0	0	0
ENSMUSG00000114049	0	0	3	0	0	3	2	0	3	5
ENSMUSG00000025777	3	7	0	6	3	3	15	4	0	8
ENSMUSG00000054672	0	0	6	0	6	11	18	16	18	6
ENSMUSG00000054679	3	0	3	9	15	6	18	3	9	0
ENSMUSG00000042306	3	0	0	7	6	3	6	0	0	7
ENSMUSG00000107870	0	0	0	0	0	1	1	0	1	0
ENSMUSG00000110388	32	36	21	22	20	9	52	35	27	32
ENSMUSG00000110749	0	0	0	0	0	0	0	0	0	0
ENSMUSG00000044441	0	3	0	0	0	0	0	3	0	0
ENSMUSG00000044444	0	0	0	0	0	0	0	0	0	0

ENSMUSG00000093544	0	0	0	0	0	0	0	0	0	0
ENSMUSG00000110741	0	0	0	3	0	0	0	0	2	0
ENSMUSG00000103042	1	0	2	1	0	2	1	1	1	1
ENSMUSG00000058124	0	0	0	0	0	0	0	0	0	0
ENSMUSG00000110745	0	0	0	0	0	0	0	0	0	0
ENSMUSG00000093547	0	0	0	0	0	0	0	0	0	0
ENSMUSG00000042848	0	0	0	0	0	0	0	0	0	0
ENSMUSG00000042849	0	0	0	0	0	0	0	0	0	0
ENSMUSG00000080250	0	0	0	0	0	0	0	0	0	0
ENSMUSG00000087752	0	1	0	1	1	2	3	0	0	0
ENSMUSG00000042845	0	0	0	0	0	0	0	0	0	0
ENSMUSG00000046352	6	0	9	12	8	14	12	6	8	3
ENSMUSG00000046354	0	0	0	0	0	0	0	0	0	0
ENSMUSG00000060548	384	339	315	307	156	346	251	499	233	448
ENSMUSG00000021298	56	45	60	62	42	63	129	62	88	56
ENSMUSG00000057093	125	190	142	156	105	118	242	156	182	143
ENSMUSG00000103040	31	38	23	24	15	21	15	20	3	21
ENSMUSG00000024245	46	47	42	27	34	72	51	40	71	68
ENSMUSG00000100606	0	0	0	0	0	0	0	0	0	0
ENSMUSG00000095309	0	0	0	0	0	0	0	0	0	0
ENSMUSG00000095301	0	0	0	0	0	0	0	0	0	0
ENSMUSG00000095300	0	0	0	4	0	0	0	0	0	0
ENSMUSG00000095302	0	0	0	0	0	0	0	0	0	0
ENSMUSG00000095305	0	0	0	0	0	0	0	0	0	0
ENSMUSG00000095307	0	0	0	0	0	0	0	0	0	0
ENSMUSG00000057321	0	0	0	0	0	0	2	0	0	0
ENSMUSG00000113809	0	0	0	0	0	0	0	0	0	0
ENSMUSG00000113808	0	0	0	0	0	0	0	0	0	0
ENSMUSG00000113803	0	0	0	0	0	0	0	0	0	0
ENSMUSG00000108942	2	0	0	1	0	0	0	3	3	1
ENSMUSG00000113801	0	0	0	0	0	0	0	0	0	0
ENSMUSG00000113800	11	0	9	3	9	3	4	8	0	5
ENSMUSG00000098306	0	0	0	0	0	0	0	0	0	0
ENSMUSG00000098307	0	0	0	0	0	0	0	0	0	0
ENSMUSG00000113805	0	0	0	0	0	0	0	0	0	0
ENSMUSG00000089454	0	0	0	0	0	0	0	0	0	0
ENSMUSG00000100907	0	0	0	0	0	0	0	0	0	0
ENSMUSG00000100905	0	0	0	0	0	0	0	0	0	0
ENSMUSG00000108948	0	0	0	0	0	0	0	0	0	0
ENSMUSG00000108949	6	10	18	8	5	6	6	6	7	11
ENSMUSG00000093744	3	0	0	0	0	3	3	0	0	0
ENSMUSG00000105664	4	3	2	3	0	1	5	0	0	0
ENSMUSG00000081610	0	0	0	0	0	0	0	0	0	0
ENSMUSG00000081611	0	0	0	2	0	0	7	2	5	3
ENSMUSG00000081616	0	0	0	0	0	0	0	0	0	0
ENSMUSG00000081618	0	0	0	0	0	0	0	0	0	0
ENSMUSG00000114769	0	0	0	0	0	0	2	0	0	0
ENSMUSG00000077081	0	0	0	0	0	0	0	0	0	0
ENSMUSG00000077085	0	0	0	0	0	0	0	0	0	0
ENSMUSG00000042156	273	271	342	283	320	323	684	480	455	452
ENSMUSG00000105845	0	0	0	0	0	0	0	0	0	0
ENSMUSG00000086969	18	24	17	10	13	18	10	17	27	20
ENSMUSG00000102814	0	0	0	0	0	0	0	0	0	0
ENSMUSG00000093460	0	0	0	0	3	0	0	0	0	0
ENSMUSG00000093461	0	0	0	0	0	0	0	0	0	0
ENSMUSG00000109322	0	0	0	0	0	0	0	0	0	0
ENSMUSG00000086960	0	0	0	0	0	0	0	0	0	0
ENSMUSG00000086963	0	0	0	0	0	0	0	0	0	0

ENSMUSG00000093469	0	0	0	0	0	0	0	0	0	0
ENSMUSG00000103604	0	0	0	0	0	0	0	0	0	0
ENSMUSG00000048004	192	256	230	179	106	342	467	329	317	345
ENSMUSG00000114767	0	0	0	0	0	0	0	0	0	0
ENSMUSG00000090849	0	0	0	0	0	0	0	0	0	0
ENSMUSG00000048001	0	3	0	3	7	0	9	3	6	0
ENSMUSG00000048003	0	6	6	2	0	3	0	2	3	0
ENSMUSG00000092636	0	0	0	0	0	0	0	0	0	0
ENSMUSG00000112071	8	9	11	26	16	6	18	12	18	9
ENSMUSG00000094163	0	0	0	0	0	0	0	0	0	0
ENSMUSG00000094160	0	0	0	0	0	0	0	3	0	0
ENSMUSG00000094161	0	0	0	0	0	0	0	0	0	0
ENSMUSG00000112075	3	0	0	0	0	3	3	0	2	0
ENSMUSG00000094167	0	0	0	0	0	0	0	0	0	0
ENSMUSG00000093796	0	0	0	0	0	0	0	0	0	2
ENSMUSG00000112076	0	0	0	0	0	0	5	0	0	0
ENSMUSG00000112079	0	0	0	0	0	0	0	0	0	0
ENSMUSG00000112078	0	0	0	0	0	0	0	0	0	0
ENSMUSG00000094168	0	0	0	0	0	0	0	0	0	0
ENSMUSG00000017499	90	98	62	111	40	44	91	48	64	57
ENSMUSG00000109582	0	0	0	0	0	0	0	0	0	0
ENSMUSG00000092633	0	1	0	0	0	3	0	0	0	1
ENSMUSG00000103606	0	0	0	0	0	0	0	0	0	0
ENSMUSG00000099499	0	0	0	0	0	0	0	0	0	0
ENSMUSG00000099498	0	0	0	0	0	0	0	0	0	0
ENSMUSG00000099495	0	0	0	0	0	0	0	0	0	0
ENSMUSG00000099494	0	0	0	0	0	0	0	0	0	0
ENSMUSG00000099497	0	0	0	0	0	0	0	0	0	0
ENSMUSG00000103607	0	0	0	0	0	0	0	0	0	0
ENSMUSG00000099491	0	0	0	0	0	3	0	0	0	0
ENSMUSG00000099490	0	0	0	0	0	0	0	0	0	0
ENSMUSG00000099493	0	0	0	0	0	0	0	0	0	0
ENSMUSG00000107376	0	0	0	0	0	0	6	0	0	0
ENSMUSG00000046438	0	0	0	0	0	0	0	0	0	0
ENSMUSG00000086349	0	0	0	0	0	0	0	0	0	0
ENSMUSG00000086348	2	0	0	0	0	0	0	1	0	0
ENSMUSG00000046435	0	0	0	0	0	0	0	0	0	0
ENSMUSG00000110654	6	6	8	6	6	6	11	3	9	6
ENSMUSG00000112172	0	0	0	0	0	0	0	0	0	0
ENSMUSG00000046431	0	0	0	0	0	0	0	0	0	0
ENSMUSG00000097923	0	0	0	0	0	0	0	0	0	0
ENSMUSG00000112175	0	0	0	0	3	0	0	0	0	0
ENSMUSG00000095032	0	0	0	0	0	0	0	0	0	0
ENSMUSG00000112177	0	0	0	0	0	0	0	0	0	0
ENSMUSG00000095031	0	0	0	0	0	0	0	0	0	0
ENSMUSG00000090007	7	19	9	10	8	13	7	17	13	16
ENSMUSG00000021381	3	0	0	0	0	0	0	0	0	0
ENSMUSG00000000159	3	8	9	11	0	0	3	0	0	6
ENSMUSG00000049062	0	0	0	0	0	0	0	0	0	0
ENSMUSG00000000157	0	0	0	18	3	0	0	0	5	0
ENSMUSG00000000154	65	55	79	77	67	42	149	69	73	78
ENSMUSG00000087760	0	0	0	0	0	0	0	0	0	0
ENSMUSG00000087761	0	0	0	0	0	0	0	0	0	0
ENSMUSG00000087764	0	0	0	0	0	0	0	0	0	0
ENSMUSG00000087765	0	0	0	0	0	0	0	0	0	0
ENSMUSG00000087766	0	0	0	0	0	0	0	0	0	0
ENSMUSG00000087769	0	0	0	0	0	0	0	0	0	0
ENSMUSG00000114355	0	5	5	4	2	0	0	6	3	0

ENSMUSG00000000411	3	10	0	3	0	9	6	0	0	3
ENSMUSG00000101540	0	0	3	0	0	0	0	0	0	3
ENSMUSG00000047384	0	0	0	0	0	1	3	0	3	3
ENSMUSG00000047383	0	6	9	2	4	0	4	4	3	3
ENSMUSG00000083449	3	2	8	3	2	2	2	2	2	0
ENSMUSG00000083448	0	0	0	0	0	0	0	0	0	0
ENSMUSG00000083445	1	0	1	0	0	0	1	1	0	0
ENSMUSG00000083444	0	0	0	0	0	0	0	0	0	0
ENSMUSG00000037259	161	112	174	125	111	126	234	172	176	163
ENSMUSG00000083446	0	0	0	0	0	0	0	0	0	0
ENSMUSG00000083441	0	0	0	0	0	0	0	0	0	0
ENSMUSG00000060673	0	0	0	0	0	0	0	0	0	0
ENSMUSG00000031949	286	274	253	326	249	286	362	321	265	352
ENSMUSG00000044528	6	21	21	30	20	14	39	30	24	42
ENSMUSG00000107874	0	0	0	0	0	0	0	0	0	0
ENSMUSG00000108059	0	0	3	2	0	0	0	0	0	0
ENSMUSG00000042918	67	95	91	105	74	256	114	117	131	145
ENSMUSG00000071275	0	0	0	0	0	0	0	0	0	0
ENSMUSG00000084239	0	0	0	0	0	0	0	0	0	0
ENSMUSG00000084238	0	0	0	0	0	0	0	0	0	0
ENSMUSG00000084237	0	0	0	0	0	0	0	0	0	0
ENSMUSG00000084236	0	0	0	0	0	0	0	0	0	0
ENSMUSG00000084234	0	0	0	0	0	0	0	0	0	0
ENSMUSG00000075598	0	0	0	0	0	0	0	0	0	0
ENSMUSG00000075599	0	0	0	0	0	0	0	0	0	0
ENSMUSG00000085728	0	0	0	0	0	0	0	0	0	0
ENSMUSG00000080938	0	0	0	0	0	0	0	0	0	0
ENSMUSG00000080939	0	0	0	0	0	0	3	0	3	3
ENSMUSG00000085722	0	0	0	0	0	0	0	0	0	0
ENSMUSG00000085721	3	0	2	4	2	4	8	3	0	0
ENSMUSG00000080930	0	0	0	0	0	0	0	0	0	0
ENSMUSG00000080933	0	2	0	0	0	0	0	0	0	0
ENSMUSG00000078616	106	70	107	248	93	68	89	30	54	46
ENSMUSG00000078612	16	17	26	15	16	23	31	23	13	18
ENSMUSG00000078618	0	0	0	0	0	0	0	0	0	0
ENSMUSG00000065746	0	0	0	0	1	0	0	0	0	0
ENSMUSG00000065747	0	0	0	0	0	0	0	0	0	0
ENSMUSG00000065745	0	0	0	0	0	0	0	0	0	0
ENSMUSG00000065742	0	0	0	0	0	0	0	0	0	0
ENSMUSG00000065743	0	2	0	2	0	0	1	0	0	1
ENSMUSG00000044121	0	0	0	0	0	0	0	0	0	0
ENSMUSG00000044120	0	0	0	0	0	0	0	0	0	0
ENSMUSG00000065749	1	0	1	0	0	0	0	0	0	0
ENSMUSG00000032373	3	9	0	9	4	16	9	0	0	13
ENSMUSG00000087891	0	0	0	0	0	0	0	0	0	0
ENSMUSG00000062773	0	0	0	0	0	0	3	0	0	0
ENSMUSG00000044522	40	83	83	82	59	84	64	69	66	57
ENSMUSG00000062778	7	0	8	2	3	11	0	0	3	6
ENSMUSG00000068048	0	0	0	0	0	0	0	0	0	0
ENSMUSG00000085188	0	0	0	0	0	0	0	0	0	0
ENSMUSG00000085189	4	0	3	2	0	7	6	0	8	2
ENSMUSG00000085187	0	0	0	0	0	0	0	0	0	0
ENSMUSG00000085184	5	24	5	11	23	17	20	11	29	25
ENSMUSG00000085182	10	6	4	7	6	0	0	3	5	13
ENSMUSG00000085183	0	0	0	0	0	0	0	0	0	0
ENSMUSG00000085180	118	60	65	38	52	72	172	104	111	71
ENSMUSG00000085181	0	0	0	5	0	0	0	0	12	0
ENSMUSG00000053930	41	33	54	23	57	43	18	24	15	23

ENSMUSG00000100988	0	0	0	0	0	0	0	0	0	0
ENSMUSG00000100989	0	0	0	0	0	0	0	0	0	0
ENSMUSG00000100982	0	0	0	0	0	0	0	0	0	0
ENSMUSG00000100983	0	0	0	0	0	0	0	0	0	0
ENSMUSG00000100981	0	3	0	0	0	0	0	0	0	0
ENSMUSG00000100986	0	0	0	0	0	0	0	0	0	0
ENSMUSG00000100987	0	0	0	0	0	0	9	9	6	0
ENSMUSG00000100984	0	0	0	0	0	0	0	0	0	0
ENSMUSG00000087252	16	19	18	17	29	14	42	17	23	9
ENSMUSG00000006269	0	2	3	4	6	0	6	11	2	0
ENSMUSG00000094486	0	0	0	0	0	0	0	0	0	0
ENSMUSG00000079006	0	0	0	0	0	0	0	0	0	0
ENSMUSG00000079007	0	0	0	0	0	0	0	0	0	0
ENSMUSG00000032999	0	3	3	0	0	0	0	0	3	0
ENSMUSG00000103860	0	0	0	0	0	0	0	0	0	0
ENSMUSG00000103862	0	0	0	0	0	0	0	0	0	0
ENSMUSG00000103863	0	0	0	0	0	0	0	0	0	0
ENSMUSG00000081388	0	0	0	0	0	0	0	0	0	0
ENSMUSG00000019893	0	0	0	3	3	2	0	2	2	0
ENSMUSG00000019892	0	0	0	0	0	0	3	3	3	0
ENSMUSG00000019894	15	7	18	10	9	23	45	24	24	26
ENSMUSG00000034239	0	0	0	0	0	0	0	0	0	0
ENSMUSG00000098257	0	0	0	0	0	0	0	0	0	0
ENSMUSG00000079008	0	0	6	0	0	6	0	0	0	0
ENSMUSG00000079009	0	0	3	0	0	3	3	0	0	3
ENSMUSG00000089304	0	0	0	0	0	0	0	0	0	0
ENSMUSG00000105626	0	0	0	0	0	0	0	0	0	0
ENSMUSG00000019768	224	228	188	213	142	201	971	382	429	351
ENSMUSG00000114112	0	0	0	0	0	0	0	0	0	0
ENSMUSG00000019762	3	0	0	3	9	12	3	3	5	0
ENSMUSG00000019767	78	47	57	63	57	72	83	68	47	73
ENSMUSG00000037727	6	5	0	0	0	9	2	3	3	0
ENSMUSG00000037725	140	152	197	116	155	116	412	204	145	89
ENSMUSG00000094765	0	0	0	0	0	0	0	0	0	0
ENSMUSG00000076927	0	0	0	0	1	0	1	1	1	0
ENSMUSG00000076920	0	1	0	1	0	0	1	0	1	0
ENSMUSG00000108959	3	0	3	0	0	0	3	0	6	0
ENSMUSG00000108958	0	0	0	0	0	0	0	0	0	0
ENSMUSG00000034906	154	138	189	243	129	148	245	172	123	118
ENSMUSG00000094767	0	0	0	0	0	0	0	0	0	0
ENSMUSG00000108952	0	0	0	0	0	0	0	0	0	0
ENSMUSG00000108955	26	8	14	4	5	4	13	11	10	23
ENSMUSG00000108957	100	76	85	115	90	77	130	89	125	116
ENSMUSG00000108956	3	0	0	3	3	15	11	9	0	7
ENSMUSG00000091248	0	0	0	0	0	0	0	0	0	0
ENSMUSG00000018387	75	81	82	105	103	77	189	117	188	114
ENSMUSG00000105853	0	0	0	2	0	0	0	0	0	0
ENSMUSG00000102804	0	0	0	0	0	0	0	0	0	0
ENSMUSG00000043366	107	194	190	122	144	184	100	200	130	207
ENSMUSG00000030268	139	260	157	143	174	235	294	201	185	101
ENSMUSG00000102801	0	6	0	0	0	0	0	0	3	0
ENSMUSG00000102803	0	2	0	0	0	3	0	0	0	0
ENSMUSG00000102802	0	0	0	0	0	0	0	0	3	0
ENSMUSG00000105859	0	0	0	0	0	0	0	0	0	0
ENSMUSG00000113226	1	4	3	1	4	2	1	5	3	6
ENSMUSG00000105702	0	0	0	0	0	0	0	0	0	0
ENSMUSG00000101574	0	0	0	3	0	0	0	0	0	0
ENSMUSG00000101575	0	0	0	0	0	0	0	0	0	0

ENSMUSG00000101576	0	0	0	0	0	0	0	0	0	0
ENSMUSG00000101570	0	0	0	0	0	0	0	0	0	0
ENSMUSG00000101571	0	0	0	0	0	0	0	0	0	0
ENSMUSG00000101573	0	0	0	0	0	0	0	0	0	0
ENSMUSG00000056436	0	38	5	12	0	9	6	9	0	3
ENSMUSG00000101579	0	0	0	0	0	0	0	0	0	0
ENSMUSG00000021415	0	0	0	0	0	0	1	0	0	1
ENSMUSG00000100803	0	0	0	0	0	0	0	0	0	0
ENSMUSG00000094922	0	0	0	0	0	0	0	0	0	0
ENSMUSG00000087246	0	0	0	0	0	0	0	0	0	3
ENSMUSG00000088210	0	0	0	0	0	0	0	0	0	0
ENSMUSG00000087241	0	0	0	0	0	0	2	0	0	0
ENSMUSG00000058005	0	0	0	0	0	0	0	0	0	0
ENSMUSG00000088218	0	0	0	0	0	0	0	0	0	0
ENSMUSG00000104898	0	0	0	0	0	0	0	0	0	0
ENSMUSG00000029660	6	5	5	3	0	20	8	6	4	27
ENSMUSG00000104895	0	0	0	0	0	0	0	0	0	0
ENSMUSG00000104892	0	0	0	0	0	0	0	0	0	0
ENSMUSG00000021418	307	327	321	401	337	278	362	401	380	351
ENSMUSG00000060530	15	3	13	5	0	8	4	18	8	12
ENSMUSG00000106482	0	0	0	0	0	0	0	0	0	0
ENSMUSG00000113122	0	0	0	0	0	0	0	0	0	0
ENSMUSG00000089164	0	0	0	0	0	0	0	0	0	0
ENSMUSG00000111898	0	0	0	0	0	0	0	0	0	0
ENSMUSG00000113123	0	3	5	13	0	0	5	9	5	0
ENSMUSG00000089161	0	0	0	0	0	0	0	0	0	0
ENSMUSG00000089162	0	0	0	0	0	0	0	0	0	0
ENSMUSG00000089163	0	0	0	0	0	0	0	0	0	0
ENSMUSG00000088718	0	0	0	0	0	0	0	0	0	0
ENSMUSG00000111891	0	0	0	0	0	0	0	0	0	0
ENSMUSG00000002006	322	326	298	275	209	445	570	375	503	436
ENSMUSG00000111894	0	0	0	0	0	0	0	0	0	0
ENSMUSG00000106139	25	38	42	67	48	40	78	55	8	57
ENSMUSG00000106138	0	0	0	0	0	0	0	0	0	0
ENSMUSG00000009580	0	0	0	0	0	0	0	0	0	0
ENSMUSG00000106134	0	0	0	0	0	0	0	0	0	0
ENSMUSG00000106136	0	0	0	0	0	0	0	0	3	0
ENSMUSG00000106131	0	0	3	0	0	0	0	0	0	0
ENSMUSG00000106130	0	1	0	0	0	0	0	0	0	0
ENSMUSG00000009588	0	0	0	0	0	2	8	0	0	0
ENSMUSG00000028197	3	11	6	3	8	16	47	9	11	11
ENSMUSG00000088715	0	0	0	0	0	0	0	0	0	0
ENSMUSG00000089307	0	0	0	0	0	0	0	0	0	0
ENSMUSG00000088716	0	0	0	0	0	0	0	0	0	0
ENSMUSG00000094360	2	2	2	2	0	2	4	6	2	4
ENSMUSG00000094361	0	0	0	0	0	0	0	0	0	0
ENSMUSG00000094362	0	0	0	0	0	0	0	0	0	0
ENSMUSG00000094363	0	0	0	0	0	0	0	0	0	0
ENSMUSG00000094364	0	0	0	0	0	0	0	0	0	0
ENSMUSG00000094366	0	0	0	4	1	0	0	0	0	0
ENSMUSG00000022762	13	6	0	6	4	9	12	6	9	15
ENSMUSG00000112277	0	0	0	0	0	0	0	0	0	0
ENSMUSG00000094369	0	0	0	0	0	0	0	0	0	0
ENSMUSG00000112275	0	0	0	0	0	0	0	0	0	0
ENSMUSG00000112272	0	0	0	0	0	0	0	0	0	0
ENSMUSG00000112271	0	0	0	0	0	0	0	0	0	0
ENSMUSG00000112270	0	0	0	0	0	0	0	0	0	0
ENSMUSG00000017652	128	137	133	199	77	98	175	134	190	131

ENSMUSG00000092932	0	0	0	0	0	0	0	0	0	0
ENSMUSG00000052848	0	0	0	0	0	0	0	0	0	0
ENSMUSG00000020690	0	0	0	0	0	0	0	0	0	0
ENSMUSG00000092208	0	0	0	0	0	0	0	0	0	0
ENSMUSG00000028444	68	108	127	82	82	132	122	125	79	118
ENSMUSG00000092204	0	0	0	0	0	0	0	0	0	0
ENSMUSG00000035626	0	0	0	0	0	0	0	0	0	0
ENSMUSG00000108470	0	5	0	0	0	0	0	3	3	6
ENSMUSG00000063764	0	0	0	0	0	0	0	0	0	0
ENSMUSG00000015879	17	24	38	35	0	32	51	39	39	32
ENSMUSG00000114944	0	0	0	0	0	0	0	0	0	0
ENSMUSG00000114949	0	0	0	0	2	0	0	0	0	0
ENSMUSG00000114948	0	0	0	0	0	0	0	0	0	0
ENSMUSG00000106791	9	3	9	20	20	24	0	3	6	9
ENSMUSG00000113639	0	0	0	0	0	0	0	0	0	0
ENSMUSG00000106794	0	0	0	0	0	0	0	0	0	0
ENSMUSG00000038010	80	96	80	74	78	67	112	126	160	91
ENSMUSG00000038011	57	33	22	50	18	26	43	39	48	19
ENSMUSG00000113632	0	0	0	0	0	0	0	0	0	0
ENSMUSG00000097182	6	21	6	10	11	17	17	21	23	21
ENSMUSG00000113635	0	0	0	0	0	0	0	0	0	0
ENSMUSG00000113636	5	3	3	2	2	0	0	2	0	0
ENSMUSG00000097186	0	0	1	0	0	0	0	0	0	0
ENSMUSG00000016524	0	0	0	6	6	0	3	9	0	0
ENSMUSG00000016526	24	12	37	3	20	29	39	21	42	38
ENSMUSG00000094985	3	0	0	0	0	0	0	0	0	0
ENSMUSG00000094986	0	0	0	0	0	0	0	0	0	0
ENSMUSG00000094987	0	0	0	0	0	0	0	0	0	0
ENSMUSG00000109149	9	8	9	5	11	18	12	3	11	6
ENSMUSG00000109148	0	0	0	0	0	0	0	0	0	0
ENSMUSG00000094983	0	1	0	0	1	0	0	0	0	0
ENSMUSG00000109145	0	0	0	0	0	3	6	0	0	3
ENSMUSG00000105446	1	1	0	0	0	0	0	0	0	0
ENSMUSG00000105440	0	0	0	0	0	0	0	0	0	0
ENSMUSG00000105441	0	0	0	0	0	0	0	0	0	0
ENSMUSG00000109143	4	0	2	0	0	0	0	0	0	0
ENSMUSG00000109142	0	1	0	0	0	0	0	0	0	0
ENSMUSG00000082146	0	0	0	0	0	0	0	0	0	0
ENSMUSG00000106869	0	0	3	2	0	0	0	0	0	0
ENSMUSG00000106863	0	0	0	0	0	0	0	0	0	0
ENSMUSG00000106862	0	0	0	0	0	6	2	0	0	0
ENSMUSG00000106860	0	0	0	0	0	0	0	0	0	0
ENSMUSG00000066242	0	0	0	0	3	0	0	0	0	0
ENSMUSG00000066240	0	0	0	0	0	0	0	0	0	0
ENSMUSG00000066241	0	0	0	0	0	0	0	0	0	0
ENSMUSG00000024897	739	809	857	788	826	968	1186	947	1015	891
ENSMUSG00000021123	9	15	35	56	27	38	43	27	47	61
ENSMUSG00000111702	0	0	0	0	0	0	0	0	0	0
ENSMUSG00000111707	0	0	0	0	0	0	0	0	0	0
ENSMUSG00000111704	0	0	0	0	0	0	0	0	0	0
ENSMUSG00000096270	0	0	0	0	0	0	0	0	0	0
ENSMUSG00000096272	0	0	0	0	0	0	0	0	0	0
ENSMUSG00000096275	0	0	0	0	0	0	0	0	0	0
ENSMUSG00000096276	0	0	0	0	0	0	0	0	0	0
ENSMUSG00000096277	0	0	0	0	0	0	0	0	0	0
ENSMUSG00000114367	0	0	0	0	0	0	0	0	0	0
ENSMUSG00000114365	0	0	0	0	0	0	0	0	0	0
ENSMUSG00000114364	0	0	0	0	0	0	0	0	0	0

ENSMUSG00000114363	0	0	0	0	0	0	0	0	0	0
ENSMUSG00000114361	0	0	0	0	0	0	0	0	0	0
ENSMUSG00000114360	0	0	0	0	2	0	0	0	2	0
ENSMUSG00000104799	0	0	0	0	0	0	0	0	0	0
ENSMUSG00000104793	6	2	0	0	6	0	3	3	0	0
ENSMUSG00000108235	0	0	0	3	0	0	6	0	0	0
ENSMUSG00000104797	0	0	1	0	0	0	0	0	1	1
ENSMUSG00000104796	0	0	0	0	1	0	0	0	0	0
ENSMUSG00000093985	9	10	4	7	16	47	31	12	20	38
ENSMUSG00000026694	529	492	503	541	496	582	777	709	627	616
ENSMUSG00000093981	0	0	0	0	0	0	0	0	0	0
ENSMUSG00000093980	0	0	0	0	0	0	0	0	0	0
ENSMUSG00000026692	18	3	11	15	3	28	74	30	52	15
ENSMUSG00000111821	0	0	0	0	0	0	0	0	0	0
ENSMUSG00000057424	0	0	0	0	0	0	0	0	0	0
ENSMUSG00000057425	0	0	3	6	0	0	0	0	0	0
ENSMUSG00000104425	0	0	0	0	0	0	0	0	0	0
ENSMUSG00000104424	0	0	0	0	0	0	0	0	0	0
ENSMUSG00000113699	0	0	0	0	0	0	0	0	0	0
ENSMUSG00000104420	2	12	19	21	20	27	43	21	10	23
ENSMUSG00000104423	0	0	0	0	0	0	0	0	0	0
ENSMUSG00000104422	0	0	0	0	0	0	0	0	0	0
ENSMUSG00000104429	0	0	0	0	0	0	0	0	0	0
ENSMUSG00000104428	0	0	0	0	0	0	0	0	0	0
ENSMUSG00000083939	0	0	0	0	0	0	0	0	0	0
ENSMUSG00000083938	0	0	0	0	0	0	0	0	0	0
ENSMUSG00000091723	0	0	0	0	0	0	0	0	0	0
ENSMUSG00000091722	0	0	0	0	0	0	0	0	0	0
ENSMUSG00000091721	0	0	0	0	0	3	0	0	0	0
ENSMUSG00000083931	0	0	0	0	0	0	0	0	0	0
ENSMUSG00000083930	0	0	0	0	0	0	0	0	0	0
ENSMUSG00000083933	0	0	0	0	0	0	0	0	2	0
ENSMUSG00000083934	0	0	0	0	0	0	0	0	0	0
ENSMUSG00000083937	0	0	0	0	0	0	0	0	0	0
ENSMUSG00000083936	0	1	3	0	6	0	3	3	0	3
ENSMUSG00000090474	0	0	0	0	0	0	0	0	0	0
ENSMUSG00000025927	6	0	3	0	9	3	8	0	9	11
ENSMUSG00000025929	0	0	0	0	0	3	0	0	0	0
ENSMUSG00000074555	0	0	1	9	0	3	3	6	3	2
ENSMUSG00000098303	0	0	0	0	0	0	0	0	0	0
ENSMUSG00000078276	0	0	0	0	0	0	0	0	0	0
ENSMUSG00000056771	66	55	103	73	60	112	54	101	85	58
ENSMUSG00000056777	0	0	0	0	0	0	0	2	0	0
ENSMUSG00000046258	0	0	0	0	0	0	0	0	0	0
ENSMUSG00000078270	0	0	0	0	0	0	0	0	0	0
ENSMUSG00000073988	76	65	92	73	43	127	140	102	76	91
ENSMUSG00000074952	0	0	0	0	0	0	0	0	0	0
ENSMUSG00000074955	0	0	0	0	0	0	0	0	0	0
ENSMUSG00000077400	0	2	1	0	0	1	2	3	0	0
ENSMUSG00000045508	0	0	0	0	0	0	0	0	0	0
ENSMUSG00000045509	18	3	0	3	0	5	8	6	3	6
ENSMUSG00000095110	0	0	0	0	0	0	0	0	0	0
ENSMUSG00000095113	0	0	0	0	0	0	0	0	0	0
ENSMUSG00000045502	9	3	12	87	12	61	184	54	132	29
ENSMUSG00000095116	0	0	0	0	0	0	0	0	0	0
ENSMUSG00000079853	0	0	0	3	5	3	0	0	0	14
ENSMUSG00000079852	9	12	5	23	0	15	17	0	9	4
ENSMUSG00000062226	0	0	0	0	0	0	0	0	0	0

ENSMUSG00000080773	0	3	5	7	0	0	2	0	9	3
ENSMUSG00000080770	0	0	0	0	0	0	0	0	0	0
ENSMUSG00000080771	0	0	0	0	0	0	0	0	0	0
ENSMUSG00000080774	6	0	0	3	2	2	0	0	0	2
ENSMUSG00000080778	0	0	0	0	0	0	0	0	0	0
ENSMUSG00000098538	0	0	0	0	0	0	0	0	0	0
ENSMUSG00000111285	0	0	0	0	0	0	0	0	0	0
ENSMUSG00000111284	0	0	0	0	0	0	0	0	0	0
ENSMUSG00000111283	12	15	17	12	20	2	6	23	17	25
ENSMUSG00000111282	0	0	0	0	0	0	0	0	0	3
ENSMUSG00000048905	0	3	18	30	26	13	30	3	32	6
ENSMUSG00000053856	0	0	0	0	0	0	0	0	0	0
ENSMUSG00000053852	0	0	0	0	0	0	0	0	0	0
ENSMUSG00000094647	0	0	0	0	0	0	0	0	0	0
ENSMUSG00000093616	0	0	0	0	0	0	0	0	0	0
ENSMUSG00000094643	0	0	0	0	0	0	0	0	0	0
ENSMUSG00000094642	0	0	0	0	0	0	0	0	0	0
ENSMUSG00000094641	0	0	0	0	0	0	0	0	0	0
ENSMUSG00000094649	0	0	0	0	3	11	17	12	14	3
ENSMUSG00000094648	2	2	1	2	4	2	2	2	3	4
ENSMUSG00000081796	0	0	0	0	0	0	0	0	0	0
ENSMUSG00000081793	3	3	0	3	0	3	0	0	0	3
ENSMUSG00000081791	0	0	0	5	0	0	0	0	0	0
ENSMUSG00000030165	17	0	24	31	21	25	88	39	97	24
ENSMUSG00000031330	2	0	0	0	0	3	0	3	0	0
ENSMUSG00000081798	5	3	0	0	0	3	0	10	6	3
ENSMUSG00000006930	128	83	141	187	149	77	160	100	85	117
ENSMUSG00000046652	2	14	2	6	1	7	3	2	3	0
ENSMUSG00000055452	40	37	47	35	41	38	60	0	51	62
ENSMUSG00000055456	0	0	0	0	0	0	0	0	0	0
ENSMUSG00000081423	0	0	0	0	0	0	0	0	0	0
ENSMUSG00000081422	0	1	1	0	0	1	0	2	3	7
ENSMUSG00000081424	1	0	3	0	0	0	0	0	0	0
ENSMUSG00000081426	0	0	0	0	0	0	0	0	0	0
ENSMUSG00000092566	0	0	0	0	0	0	0	0	0	0
ENSMUSG00000092567	0	0	0	0	0	0	0	0	0	0
ENSMUSG00000092561	0	0	0	0	0	0	0	0	0	0
ENSMUSG00000092562	0	0	0	0	0	0	0	0	0	0
ENSMUSG00000077543	0	0	0	0	0	0	0	0	0	0
ENSMUSG00000077541	0	0	0	0	0	0	0	0	0	0
ENSMUSG00000092568	0	0	0	0	0	0	0	0	0	0
ENSMUSG00000056959	0	0	0	0	0	0	0	0	0	0
ENSMUSG00000099847	3	5	0	6	4	3	3	0	0	5
ENSMUSG00000092813	0	0	0	0	0	0	0	0	0	0
ENSMUSG00000082212	0	0	0	0	0	0	0	0	0	0
ENSMUSG00000082211	0	0	0	0	0	0	0	0	0	0
ENSMUSG00000082217	0	0	0	0	0	0	0	0	0	0
ENSMUSG00000082216	0	0	0	0	0	0	0	0	0	0
ENSMUSG00000082215	0	0	0	0	0	0	0	0	0	0
ENSMUSG00000094354	0	0	0	0	0	0	0	0	0	0
ENSMUSG00000082218	0	0	0	0	0	0	0	0	0	0
ENSMUSG00000111276	0	0	0	0	0	0	0	0	0	0
ENSMUSG00000112266	0	3	0	0	0	3	0	0	0	0
ENSMUSG00000099285	0	0	0	0	0	0	0	0	0	0
ENSMUSG00000099287	3	0	2	4	0	3	5	3	3	1
ENSMUSG00000112261	0	0	0	0	0	0	0	0	0	0
ENSMUSG00000032517	17	5	14	24	6	15	26	9	8	0
ENSMUSG00000112262	0	0	0	2	0	0	0	0	0	0

ENSMUSG00000037593	36	48	59	42	26	34	48	70	43	29
ENSMUSG00000043614	82	41	24	64	27	33	95	76	92	57
ENSMUSG00000087482	0	0	0	0	0	0	0	0	0	0
ENSMUSG00000098880	0	0	0	0	0	0	0	0	0	0
ENSMUSG00000098883	0	0	0	0	0	0	0	0	0	0
ENSMUSG00000098887	0	0	0	0	0	0	0	0	0	0
ENSMUSG00000098888	0	0	0	0	0	0	0	0	0	0
ENSMUSG00000068940	0	0	0	0	0	0	0	0	0	0
ENSMUSG00000068947	0	0	0	6	0	0	0	0	0	0
ENSMUSG00000068948	0	0	0	0	0	0	0	0	0	0
ENSMUSG00000084688	0	0	0	0	0	0	0	0	0	0
ENSMUSG00000084689	0	0	0	0	0	0	0	0	0	0
ENSMUSG00000084682	0	0	0	0	0	0	0	0	0	0
ENSMUSG00000084683	0	0	0	0	0	0	0	0	0	0
ENSMUSG00000084684	0	0	0	0	0	0	0	0	0	0
ENSMUSG00000075149	0	0	0	0	0	0	0	0	3	0
ENSMUSG00000075148	0	0	0	0	0	0	0	0	0	0
ENSMUSG00000084849	0	0	0	0	0	0	0	0	0	0
ENSMUSG00000075145	0	0	0	0	0	0	0	0	0	0
ENSMUSG00000075144	0	0	0	0	0	0	0	0	0	0
ENSMUSG00000075146	0	0	0	0	0	0	0	0	0	0
ENSMUSG00000075140	0	0	0	0	0	0	0	0	0	0
ENSMUSG00000075143	0	0	0	0	0	0	0	0	0	0
ENSMUSG00000075142	0	0	0	0	0	0	0	0	0	0
ENSMUSG00000109743	0	12	0	0	0	0	0	0	6	0
ENSMUSG00000109742	0	0	0	0	0	0	0	0	0	0
ENSMUSG00000070390	112	125	162	172	87	126	163	186	225	75
ENSMUSG00000061361	0	0	0	0	0	0	0	0	0	0
ENSMUSG00000102290	0	0	0	0	0	0	0	0	0	0
ENSMUSG00000102291	5	5	9	8	0	3	8	3	5	0
ENSMUSG00000064626	0	0	0	0	0	0	0	0	0	0
ENSMUSG00000099362	0	0	0	0	0	0	0	0	0	0
ENSMUSG00000107226	11	9	8	3	0	9	0	3	3	6
ENSMUSG00000092219	0	0	0	0	0	0	0	0	0	0
ENSMUSG00000107223	0	0	0	0	0	0	0	2	0	3
ENSMUSG00000107220	0	0	0	0	0	0	0	0	0	0
ENSMUSG00000033740	0	0	3	3	0	0	3	0	0	6
ENSMUSG00000081947	0	0	0	0	0	0	0	0	0	0
ENSMUSG00000104815	0	0	0	0	0	0	0	0	0	0
ENSMUSG00000104816	0	0	0	0	0	0	0	3	0	0
ENSMUSG00000101971	0	0	0	0	0	0	0	0	0	0
ENSMUSG00000101974	0	0	0	0	0	0	0	0	0	0
ENSMUSG00000101975	0	0	0	0	0	0	0	0	0	0
ENSMUSG00000101977	0	0	0	0	0	0	0	0	0	0
ENSMUSG00000101978	0	0	0	0	0	0	0	0	0	0
ENSMUSG00000092210	0	0	0	8	0	0	0	0	0	0
ENSMUSG00000078799	0	0	0	0	0	0	0	0	0	0
ENSMUSG00000092211	7	0	0	0	0	0	0	0	0	0
ENSMUSG00000104812	0	0	0	0	0	0	0	0	0	0
ENSMUSG00000078793	0	0	0	0	0	0	0	0	0	0
ENSMUSG00000092216	0	0	0	3	0	0	6	0	3	0
ENSMUSG00000074236	0	0	0	0	0	0	0	0	0	0
ENSMUSG00000074237	0	0	0	0	0	0	0	0	0	0
ENSMUSG00000078796	0	0	0	0	0	0	0	0	0	0
ENSMUSG00000078795	3	6	0	0	3	6	12	18	12	6
ENSMUSG00000092217	0	0	0	0	0	0	0	0	0	0
ENSMUSG00000092214	0	0	0	0	0	0	0	0	0	0
ENSMUSG00000020428	0	0	0	0	0	0	0	0	0	0

ENSMUSG00000078597	0	0	0	0	0	0	3	0	0	0
ENSMUSG00000020429	0	0	0	0	0	0	0	0	0	0
ENSMUSG00000078591	3	3	12	17	0	14	25	15	35	24
ENSMUSG00000065283	0	0	0	0	0	0	0	0	0	0
ENSMUSG00000081863	0	0	0	0	0	0	0	0	0	0
ENSMUSG00000050087	9	0	3	3	3	6	4	11	6	5
ENSMUSG00000078420	0	0	0	0	0	0	0	0	0	0
ENSMUSG00000062105	3	0	0	0	0	0	3	0	0	6
ENSMUSG00000089558	0	0	0	0	0	0	0	0	0	0
ENSMUSG00000062103	0	0	0	0	0	0	0	0	0	0
ENSMUSG00000089556	0	0	0	0	2	0	0	0	0	0
ENSMUSG00000089551	0	0	0	0	0	0	0	0	0	0
ENSMUSG00000089550	0	0	0	0	0	0	0	0	0	0
ENSMUSG00000089553	0	0	0	0	0	0	0	0	0	0
ENSMUSG00000089552	0	0	0	0	0	0	0	0	0	0
ENSMUSG00000095413	1	1	3	0	1	1	1	1	0	1
ENSMUSG00000063851	0	0	3	3	0	4	6	2	0	3
ENSMUSG00000095414	0	0	0	3	0	0	0	1	0	0
ENSMUSG00000035355	13	17	23	34	8	18	5	9	13	26
ENSMUSG00000102307	2	4	8	4	3	5	3	5	7	6
ENSMUSG00000102300	0	0	0	0	0	0	0	0	0	0
ENSMUSG00000102308	0	0	0	0	0	0	0	0	0	0
ENSMUSG00000102309	6	21	9	12	3	3	3	6	3	6
ENSMUSG00000038656	0	0	0	0	0	0	0	0	0	0
ENSMUSG00000038651	0	0	0	0	0	0	0	0	0	0
ENSMUSG00000069706	0	0	0	0	0	0	0	0	0	0
ENSMUSG00000069707	0	0	0	0	0	0	0	0	0	0
ENSMUSG00000069708	0	0	0	0	0	0	0	0	0	0
ENSMUSG00000096076	0	0	0	0	0	0	0	0	0	0
ENSMUSG00000096075	0	0	0	1	0	1	0	0	0	0
ENSMUSG00000096072	0	0	0	0	0	0	0	0	0	0
ENSMUSG00000096073	0	0	0	0	0	0	0	0	0	0
ENSMUSG00000096070	0	0	0	0	0	0	0	0	0	0
ENSMUSG00000096071	0	0	0	0	0	0	0	0	0	0
ENSMUSG00000031073	0	0	0	0	0	0	0	0	0	0
ENSMUSG00000034009	160	245	33	40	122	124	476	449	142	9
ENSMUSG00000034000	0	0	3	0	0	0	0	0	0	0
ENSMUSG00000063277	0	5	0	8	0	9	0	6	0	8
ENSMUSG00000042532	36	52	51	39	42	29	45	48	39	60
ENSMUSG00000104223	0	0	0	0	0	0	0	0	0	0
ENSMUSG00000104220	3	0	0	3	3	0	0	0	3	6
ENSMUSG00000104229	0	0	0	0	0	0	0	0	0	0
ENSMUSG00000104228	0	0	0	0	0	0	0	0	0	0
ENSMUSG00000106784	0	0	0	0	0	0	0	0	0	0
ENSMUSG00000060771	638	596	650	574	445	573	797	655	804	821
ENSMUSG00000094256	15	6	11	6	9	6	5	11	12	15
ENSMUSG00000059562	0	9	1	7	3	6	11	3	3	5
ENSMUSG00000100032	0	0	0	0	0	0	0	0	0	0
ENSMUSG00000088783	0	0	0	0	0	0	0	0	0	0
ENSMUSG00000061829	0	0	0	0	0	0	0	0	0	0
ENSMUSG00000100219	0	0	0	0	0	0	0	0	0	0
ENSMUSG00000100216	0	0	0	0	0	0	0	0	0	0
ENSMUSG00000100217	0	0	0	0	0	2	0	0	0	0
ENSMUSG00000061825	0	0	0	30	0	0	6	7	3	0
ENSMUSG00000104467	21	18	42	24	28	37	35	38	43	15
ENSMUSG00000100212	3	2	0	1	2	0	5	2	9	2
ENSMUSG00000109158	0	0	0	0	0	0	0	0	0	0
ENSMUSG00000100211	0	0	12	7	3	0	0	15	8	0

ENSMUSG00000043029	15	12	20	18	13	26	52	39	26	41
ENSMUSG00000105470	5	13	9	6	3	4	6	3	14	5
ENSMUSG00000068809	0	0	0	0	0	0	0	0	0	0
ENSMUSG00000095757	0	0	0	0	0	0	0	3	0	0
ENSMUSG00000095754	0	0	0	0	0	0	0	0	0	0
ENSMUSG00000095755	4	0	0	0	0	4	0	0	0	0
ENSMUSG00000095752	2	0	0	2	0	0	3	2	0	0
ENSMUSG00000095758	0	0	0	0	0	0	0	0	0	0
ENSMUSG00000095759	0	0	0	0	0	0	0	0	0	0
ENSMUSG00000088405	0	0	0	0	0	0	0	0	0	0
ENSMUSG00000088407	0	0	0	0	0	0	0	0	0	0
ENSMUSG00000088402	1	1	0	0	0	0	0	0	0	1
ENSMUSG00000088403	0	0	0	0	0	0	0	0	0	0
ENSMUSG00000058216	6	0	0	0	3	10	9	8	22	3
ENSMUSG00000105476	0	0	0	0	0	0	0	0	0	0
ENSMUSG00000088408	0	0	0	0	0	0	0	0	0	0
ENSMUSG00000088409	0	0	0	0	0	0	0	0	0	0
ENSMUSG00000049894	0	0	0	0	0	0	0	0	0	0
ENSMUSG00000105782	0	0	0	0	0	0	0	0	0	0
ENSMUSG00000065956	0	0	0	0	0	0	0	0	0	0
ENSMUSG00000094085	0	0	0	0	0	0	0	0	0	0
ENSMUSG00000094084	0	0	0	0	0	0	0	0	0	0
ENSMUSG00000064926	0	0	0	0	0	0	0	0	0	0
ENSMUSG00000094086	0	0	0	0	0	0	1	0	0	0
ENSMUSG00000094081	0	0	0	0	0	0	0	0	0	0
ENSMUSG00000094080	0	0	0	0	0	0	0	0	0	0
ENSMUSG00000094083	0	7	5	0	0	4	0	0	0	0
ENSMUSG00000094082	0	0	0	0	0	0	0	0	0	0
ENSMUSG00000086530	0	0	0	0	0	0	0	0	0	0
ENSMUSG00000086531	0	0	0	0	0	0	0	0	0	0
ENSMUSG00000062844	0	0	0	0	0	0	0	0	0	0
ENSMUSG00000111919	0	0	0	0	0	0	0	0	0	0
ENSMUSG00000111917	0	0	0	0	0	2	0	3	3	0
ENSMUSG00000111915	0	0	0	0	0	0	0	0	0	0
ENSMUSG00000036095	201	257	232	155	207	336	396	317	203	299
ENSMUSG00000111911	0	0	0	0	0	0	0	0	0	0
ENSMUSG00000111910	0	0	0	0	0	0	0	0	0	0
ENSMUSG00000023333	0	0	0	0	0	0	0	0	0	0
ENSMUSG00000106367	0	0	0	0	1	0	0	0	0	0
ENSMUSG00000093368	0	0	0	0	0	0	0	0	0	0
ENSMUSG00000039246	68	93	120	116	132	140	249	135	176	147
ENSMUSG00000093362	0	0	0	0	0	0	0	0	0	0
ENSMUSG00000009628	24	12	21	15	0	12	21	15	35	22
ENSMUSG00000022763	6	0	6	6	6	6	6	14	12	0
ENSMUSG00000002633	3	0	0	0	0	0	0	3	0	0
ENSMUSG00000052471	3	0	0	0	0	3	0	0	2	0
ENSMUSG00000113942	0	0	0	0	0	0	0	0	0	0
ENSMUSG00000045989	0	0	0	0	6	0	0	0	2	0
ENSMUSG00000112750	0	0	0	0	0	0	0	0	0	0
ENSMUSG00000112752	0	0	0	0	0	0	0	0	0	0
ENSMUSG00000112753	0	0	0	0	0	3	0	0	0	0
ENSMUSG00000055125	14	19	25	14	3	11	8	11	24	9
ENSMUSG00000112757	0	0	0	3	0	3	4	2	0	0
ENSMUSG00000024647	0	0	0	0	0	0	3	0	0	0
ENSMUSG00000020159	0	0	0	0	0	0	0	0	3	0
ENSMUSG00000031512	2	6	6	0	6	7	0	3	3	2
ENSMUSG00000092019	0	0	0	0	0	0	0	0	0	0
ENSMUSG00000097068	31	43	35	31	18	62	59	25	16	46

ENSMUSG00000097066	0	0	0	0	0	0	0	0	0	0
ENSMUSG00000096439	0	0	0	0	0	0	0	0	0	0
ENSMUSG00000097063	0	0	0	0	0	0	1	0	0	0
ENSMUSG00000096436	0	0	0	0	0	0	3	0	0	0
ENSMUSG00000096434	0	0	0	0	0	0	0	0	0	0
ENSMUSG00000038020	37	48	60	42	69	49	19	53	66	49
ENSMUSG00000110663	0	0	0	0	0	0	0	0	0	0
ENSMUSG00000110666	0	0	0	0	0	0	0	0	0	0
ENSMUSG00000110664	21	6	7	43	7	3	2	6	0	12
ENSMUSG00000110665	0	0	0	0	0	0	0	0	0	0
ENSMUSG00000113406	0	0	0	0	0	0	0	0	0	0
ENSMUSG00000113404	12	3	0	9	5	3	6	0	3	0
ENSMUSG00000113403	3	4	0	0	3	2	3	2	0	2
ENSMUSG00000113402	0	0	2	0	0	0	0	0	0	2
ENSMUSG00000113400	0	0	0	0	0	0	0	0	0	0
ENSMUSG00000113409	3	9	0	2	2	2	2	3	0	6
ENSMUSG00000113408	0	0	2	1	0	0	1	0	1	0
ENSMUSG00000003273	194	202	194	148	180	240	355	235	220	227
ENSMUSG00000105639	0	3	2	1	9	3	0	0	6	4
ENSMUSG00000003271	3	7	0	9	0	0	3	19	6	3
ENSMUSG00000051354	14	37	31	6	9	5	57	30	29	12
ENSMUSG00000003279	65	50	51	48	20	90	57	76	54	87
ENSMUSG00000105635	1	2	2	0	1	0	1	1	0	2
ENSMUSG00000114753	3	6	0	0	3	0	3	3	0	0
ENSMUSG00000114750	0	0	0	0	0	0	0	0	0	0
ENSMUSG00000093158	0	0	0	0	0	2	3	2	0	1
ENSMUSG00000114754	0	0	0	0	0	0	0	0	0	0
ENSMUSG00000095431	0	0	0	0	0	0	0	0	0	0
ENSMUSG00000082949	0	0	0	0	0	0	0	0	0	0
ENSMUSG00000023279	8	12	6	5	5	12	32	36	15	18
ENSMUSG00000082947	0	0	2	2	0	0	0	0	0	0
ENSMUSG00000082945	0	0	0	0	0	0	0	0	0	0
ENSMUSG00000082943	0	0	0	0	0	0	0	0	0	0
ENSMUSG00000082940	0	0	0	0	0	0	0	0	0	0
ENSMUSG00000108009	0	0	0	0	0	0	0	0	0	0
ENSMUSG00000113868	0	0	0	0	0	0	0	0	0	0
ENSMUSG00000108003	0	0	0	0	0	0	0	0	0	0
ENSMUSG00000108002	0	0	0	0	0	0	0	0	0	0
ENSMUSG00000108000	0	0	0	0	0	0	0	0	0	1
ENSMUSG00000108007	0	0	0	0	0	0	0	0	0	0
ENSMUSG00000108005	0	0	0	3	0	0	0	0	0	0
ENSMUSG00000108004	10	0	16	15	9	3	8	4	19	3
ENSMUSG00000083018	0	0	0	0	0	0	0	0	0	0
ENSMUSG00000036357	0	0	0	0	0	0	0	3	0	0
ENSMUSG00000083010	0	0	0	0	0	0	0	0	0	0
ENSMUSG00000083016	0	0	3	2	2	3	0	0	2	2
ENSMUSG00000083014	3	0	5	0	0	2	5	0	0	0
ENSMUSG00000083015	7	3	7	0	6	0	6	3	2	3
ENSMUSG00000085638	3	0	0	0	0	0	0	0	3	3
ENSMUSG00000052629	6	2	1	0	0	0	6	5	2	3
ENSMUSG00000098105	0	5	10	3	5	3	2	5	3	2
ENSMUSG00000085635	0	0	0	0	0	0	0	0	0	0
ENSMUSG00000040856	0	6	0	0	5	0	0	6	0	0
ENSMUSG00000111135	0	0	0	0	0	0	0	0	0	0
ENSMUSG00000111137	0	0	0	0	0	0	0	0	0	0
ENSMUSG00000108672	0	0	0	0	0	0	0	0	0	0
ENSMUSG00000097819	8	9	12	33	9	19	33	32	27	24
ENSMUSG00000096245	0	0	0	0	0	0	0	0	0	0

ENSMUSG00000001656	0	0	0	0	0	0	0	0	0	0
ENSMUSG00000001657	0	0	3	0	2	3	0	0	0	3
ENSMUSG00000001655	0	0	0	0	0	0	0	0	0	0
ENSMUSG00000026085	0	0	0	0	0	0	0	0	0	0
ENSMUSG00000026080	61	23	57	29	34	34	122	50	79	95
ENSMUSG00000095202	0	0	0	0	0	0	0	0	0	0
ENSMUSG00000096248	0	0	0	0	0	0	0	0	0	0
ENSMUSG00000045075	3	3	3	10	9	9	9	6	5	9
ENSMUSG00000109987	0	0	0	0	0	0	0	0	0	0
ENSMUSG00000108209	0	0	0	0	0	0	0	0	0	0
ENSMUSG00000104789	0	0	0	0	0	0	0	0	0	0
ENSMUSG00000081323	0	0	0	0	0	0	0	0	0	0
ENSMUSG00000081321	0	0	0	0	0	0	0	0	0	0
ENSMUSG00000081327	0	0	0	0	0	0	0	0	0	0
ENSMUSG00000081324	0	0	0	0	0	0	0	0	0	0
ENSMUSG00000095209	0	0	0	0	0	0	0	0	0	0
ENSMUSG00000099899	0	0	0	0	0	0	0	0	0	0
ENSMUSG00000099898	0	0	0	0	0	0	0	0	0	0
ENSMUSG00000092849	0	0	0	0	0	0	0	0	0	0
ENSMUSG00000092848	0	0	0	0	0	0	0	0	0	0
ENSMUSG00000112540	0	0	0	0	0	0	0	0	0	0
ENSMUSG00000092843	0	0	0	0	0	0	0	1	0	0
ENSMUSG00000099890	0	3	0	0	0	0	0	3	2	0
ENSMUSG00000092840	0	0	0	0	0	0	0	0	0	0
ENSMUSG00000092847	0	0	0	0	0	0	0	0	0	0
ENSMUSG00000027356	0	3	0	3	0	0	2	0	0	0
ENSMUSG00000027355	0	0	0	0	0	0	0	0	0	0
ENSMUSG00000099896	0	0	0	0	0	0	0	0	0	0
ENSMUSG00000104780	0	0	0	0	0	0	0	0	0	0
ENSMUSG00000104782	3	0	0	0	0	0	0	0	0	0
ENSMUSG00000046041	0	0	0	0	0	0	0	9	0	0
ENSMUSG00000104783	0	0	0	0	0	0	0	0	0	0
ENSMUSG00000104785	0	0	0	0	0	0	0	0	0	0
ENSMUSG00000098768	0	0	0	0	0	0	0	0	0	0
ENSMUSG00000090574	0	0	0	0	0	0	0	0	0	1
ENSMUSG00000090577	0	0	0	0	0	0	0	0	0	0
ENSMUSG00000035805	0	0	0	0	3	8	12	0	3	0
ENSMUSG00000098760	6	0	3	0	0	0	0	2	0	0
ENSMUSG00000090578	0	0	0	1	0	1	0	0	0	0
ENSMUSG00000053461	10	14	9	14	11	5	7	13	6	12
ENSMUSG00000073077	12	9	3	18	8	34	43	31	38	39
ENSMUSG00000053465	0	0	0	0	0	0	0	0	0	0
ENSMUSG00000032852	0	0	0	0	0	0	0	0	0	0
ENSMUSG00000080635	0	0	0	0	0	0	0	0	0	0
ENSMUSG00000032899	19	24	12	15	23	9	38	15	14	6
ENSMUSG00000080631	0	0	0	0	0	0	0	0	0	0
ENSMUSG00000032890	27	14	18	30	15	18	39	15	32	17
ENSMUSG00000032894	0	0	0	0	0	0	0	0	0	0
ENSMUSG00000078912	0	0	0	0	0	0	0	0	0	0
ENSMUSG00000078917	0	3	0	0	3	0	2	3	2	2
ENSMUSG00000052736	8	12	8	35	15	24	11	11	34	23
ENSMUSG00000065095	0	0	0	0	0	0	0	0	0	0
ENSMUSG00000037910	0	0	0	0	0	0	0	0	0	0
ENSMUSG00000031271	0	0	0	0	0	0	0	0	0	0
ENSMUSG00000031270	0	0	0	0	0	0	0	0	0	0
ENSMUSG00000101378	3	0	0	2	0	5	3	7	0	0
ENSMUSG00000093000	0	0	0	0	0	0	0	0	0	0
ENSMUSG00000093999	0	0	0	0	0	0	0	0	0	0

ENSMUSG00000026683	126	105	150	220	119	70	233	106	64	64
ENSMUSG00000037942	3	0	3	3	0	3	3	6	6	0
ENSMUSG00000037946	395	412	506	653	378	319	680	448	631	405
ENSMUSG00000037944	9	20	18	27	12	14	110	42	48	29
ENSMUSG00000030638	67	24	58	61	68	67	75	37	60	56
ENSMUSG00000030636	0	0	3	0	0	0	0	1	0	0
ENSMUSG00000073920	0	0	0	0	0	0	0	0	0	0
ENSMUSG00000073923	0	0	0	0	0	3	0	0	0	0
ENSMUSG00000073922	0	0	0	0	0	0	0	0	0	0
ENSMUSG00000073925	0	0	0	0	0	0	0	0	0	0
ENSMUSG00000101328	0	0	0	0	0	0	4	0	0	0
ENSMUSG00000073927	0	0	0	0	3	6	3	0	0	0
ENSMUSG00000101325	0	0	0	0	0	0	0	0	0	0
ENSMUSG00000073928	0	0	0	0	0	0	0	0	0	0
ENSMUSG00000101321	0	0	0	0	0	0	0	0	0	0
ENSMUSG00000101323	0	0	0	0	0	0	0	0	0	0
ENSMUSG00000071562	0	0	0	12	0	0	0	0	0	0
ENSMUSG00000071561	0	0	0	37	0	0	0	0	0	2
ENSMUSG00000033177	0	0	0	2	0	3	8	0	0	0
ENSMUSG00000087071	0	0	0	0	0	0	0	0	0	0
ENSMUSG00000087074	0	0	0	0	0	0	0	0	0	0
ENSMUSG00000087075	2	3	12	0	0	0	11	5	5	10
ENSMUSG00000087076	8	0	7	6	9	5	5	20	0	13
ENSMUSG00000087077	0	0	0	0	0	0	0	0	0	0
ENSMUSG00000082009	0	0	0	0	0	0	0	0	0	0
ENSMUSG00000099560	0	0	0	0	0	0	0	0	0	0
ENSMUSG00000099562	0	0	0	0	0	0	0	0	0	0
ENSMUSG00000099565	0	0	0	0	0	0	0	0	0	0
ENSMUSG00000099564	0	0	0	0	3	0	3	0	2	3
ENSMUSG00000099566	0	0	0	0	0	0	0	0	0	0
ENSMUSG00000019230	20	3	0	11	13	19	29	35	17	9
ENSMUSG00000019232	16	13	12	8	6	15	12	13	2	6
ENSMUSG00000083751	0	0	0	2	0	0	0	0	0	0
ENSMUSG00000089338	0	0	0	0	0	0	0	0	0	0
ENSMUSG00000089335	0	0	0	0	0	0	0	0	0	0
ENSMUSG00000089334	0	0	0	0	0	0	0	0	0	0
ENSMUSG00000084524	0	0	0	0	0	0	0	0	0	0
ENSMUSG00000089331	0	0	0	0	0	0	0	0	0	0
ENSMUSG00000089333	0	0	0	0	0	0	0	0	0	0
ENSMUSG00000084520	0	0	0	0	0	0	0	0	0	0
ENSMUSG00000100058	0	0	0	0	0	0	0	0	0	0
ENSMUSG00000100059	0	0	0	0	0	0	0	0	0	0
ENSMUSG00000100054	0	0	0	0	0	0	0	0	0	0
ENSMUSG00000100055	0	0	0	0	0	0	0	0	0	0
ENSMUSG00000041791	0	0	0	0	0	2	0	6	0	3
ENSMUSG00000100057	0	0	0	0	0	0	0	0	0	0
ENSMUSG00000100050	0	0	0	0	0	0	0	0	0	0
ENSMUSG00000100051	0	0	0	0	0	0	0	0	0	0
ENSMUSG00000100052	0	0	0	0	0	0	0	0	0	0
ENSMUSG00000102566	0	6	3	0	0	0	0	3	0	5
ENSMUSG00000102561	0	0	0	0	0	0	3	0	6	0
ENSMUSG00000107582	0	0	0	0	0	0	0	0	0	0
ENSMUSG00000107581	0	0	0	0	0	0	0	0	0	0
ENSMUSG00000107580	3	0	5	0	3	0	0	3	3	0
ENSMUSG00000107587	0	0	0	0	0	0	0	0	0	0
ENSMUSG00000107585	5	0	0	6	0	0	0	7	0	0
ENSMUSG00000107584	0	0	0	0	0	0	0	0	0	0
ENSMUSG00000000247	6	0	0	0	0	6	9	5	0	0

ENSMUSG00000000244	166	138	172	251	113	168	218	201	236	135
ENSMUSG00000087695	0	0	3	0	0	0	0	0	0	3
ENSMUSG00000100584	0	0	0	0	0	0	0	0	0	0
ENSMUSG00000100582	0	0	0	0	0	0	0	0	0	0
ENSMUSG00000065853	2	0	0	1	0	3	0	1	3	0
ENSMUSG00000100580	0	0	0	0	0	0	0	0	0	0
ENSMUSG00000087693	0	0	0	0	0	3	0	0	0	0
ENSMUSG00000100588	0	0	0	0	0	0	0	0	0	0
ENSMUSG00000100589	0	0	0	0	0	0	0	0	0	0
ENSMUSG00000112091	0	0	0	0	0	0	0	0	0	0
ENSMUSG00000085696	6	11	7	16	6	21	56	11	42	37
ENSMUSG00000085693	0	0	0	0	0	0	0	0	0	0
ENSMUSG00000085690	0	0	0	0	0	0	0	0	0	0
ENSMUSG00000085691	0	0	0	0	0	0	0	0	0	0
ENSMUSG00000102690	0	0	0	0	0	0	0	0	0	0
ENSMUSG00000102692	116	129	149	94	120	246	951	373	582	320
ENSMUSG00000102693	0	0	0	0	0	0	0	0	0	0
ENSMUSG00000102694	2	2	0	3	3	0	2	1	0	5
ENSMUSG00000102696	0	0	0	0	0	0	0	0	0	0
ENSMUSG00000102699	0	0	0	0	0	0	0	2	0	0
ENSMUSG00000033634	3	0	12	6	0	0	0	0	3	0
ENSMUSG00000079639	0	0	0	0	0	0	0	0	0	0
ENSMUSG00000079638	0	0	0	0	0	0	0	0	0	0
ENSMUSG00000079633	0	0	0	0	0	0	0	0	0	0
ENSMUSG00000079630	0	0	0	0	0	0	0	0	0	0
ENSMUSG00000079637	0	6	0	0	0	3	5	0	4	4
ENSMUSG00000079636	0	0	0	0	0	0	0	0	0	0
ENSMUSG00000079635	0	6	0	0	0	0	2	0	0	3
ENSMUSG00000065582	0	0	0	0	0	0	0	0	1	1
ENSMUSG00000065341	1	0	1	1	0	0	0	2	0	0
ENSMUSG00000065585	0	0	0	0	0	0	0	0	0	0
ENSMUSG00000065586	0	0	0	0	0	0	0	0	0	0
ENSMUSG00000068758	173	228	200	329	220	198	293	189	212	166
ENSMUSG00000085348	0	0	0	0	0	0	0	0	0	0
ENSMUSG00000088521	0	0	0	0	0	0	0	0	0	0
ENSMUSG00000088526	0	0	0	0	0	0	0	0	0	0
ENSMUSG00000088525	0	0	0	0	0	0	0	0	0	0
ENSMUSG00000088528	1	0	0	2	0	0	1	0	2	0
ENSMUSG00000074003	3	10	2	13	8	0	7	8	3	12
ENSMUSG00000074006	18	12	3	9	2	6	6	10	12	2
ENSMUSG00000074004	6	3	5	9	5	0	11	9	9	0
ENSMUSG00000077851	0	0	0	0	0	0	0	0	0	0
ENSMUSG00000065320	0	0	0	0	0	1	0	2	1	0
ENSMUSG00000065321	0	0	0	0	0	0	0	0	0	0
ENSMUSG00000034706	5	29	14	11	19	9	27	4	30	9
ENSMUSG00000022132	11	21	19	7	21	28	78	21	24	21
ENSMUSG00000024485	0	0	0	7	0	0	4	0	0	3
ENSMUSG00000113363	0	0	0	0	0	0	0	0	0	0
ENSMUSG00000089364	0	0	0	0	0	0	0	0	0	0
ENSMUSG00000103775	0	0	0	0	0	0	2	0	0	0
ENSMUSG00000103773	0	0	0	0	0	0	0	0	0	0
ENSMUSG00000097268	0	9	0	0	0	0	0	0	6	0
ENSMUSG00000097266	0	0	0	0	0	0	2	0	0	0
ENSMUSG00000097260	0	0	0	0	0	0	0	0	0	0
ENSMUSG00000097262	0	0	0	0	6	0	2	0	0	7
ENSMUSG00000097104	0	0	0	0	0	0	0	0	0	0
ENSMUSG00000029168	21	27	22	28	23	36	19	14	18	15
ENSMUSG00000097106	0	0	0	0	3	0	0	4	0	0

ENSMUSG00000089696	0	0	0	0	0	0	0	0	0	0
ENSMUSG00000089694	23	24	23	17	10	13	37	13	7	13
ENSMUSG00000109518	0	0	0	0	0	0	0	0	0	0
ENSMUSG00000109519	0	0	0	0	0	0	0	0	0	3
ENSMUSG00000109513	0	0	0	0	0	0	0	0	0	0
ENSMUSG00000109516	0	0	0	0	0	0	0	0	0	0
ENSMUSG00000062314	0	0	0	0	0	6	0	0	0	0
ENSMUSG00000114512	0	0	0	0	0	0	0	0	0	0
ENSMUSG00000062310	52	74	52	83	31	34	23	34	78	18
ENSMUSG00000100878	0	0	0	0	0	0	0	0	0	0
ENSMUSG00000114517	0	0	0	0	0	0	0	0	0	0
ENSMUSG00000114518	0	0	0	0	0	0	0	0	0	0
ENSMUSG00000114519	0	0	0	3	0	0	0	0	0	0
ENSMUSG00000100874	0	0	0	0	0	0	0	0	0	0
ENSMUSG00000100875	0	0	0	0	0	0	0	0	0	0
ENSMUSG00000100872	4	3	2	0	0	0	0	3	2	3
ENSMUSG00000100873	0	0	0	0	0	0	0	0	0	0
ENSMUSG00000100870	0	0	0	0	0	0	0	0	0	0
ENSMUSG00000058435	2	0	0	0	0	0	0	0	6	6
ENSMUSG00000020562	0	0	0	0	0	0	0	0	0	0
ENSMUSG00000108649	0	0	1	0	0	3	0	0	0	0
ENSMUSG00000091415	61	11	39	38	24	9	3	22	3	29
ENSMUSG00000108641	0	0	0	0	0	0	0	0	0	0
ENSMUSG00000112823	72	49	49	75	33	54	45	58	45	67
ENSMUSG00000050645	6	6	3	7	0	14	0	7	0	0
ENSMUSG00000112821	0	0	0	0	0	0	0	0	0	0
ENSMUSG00000112826	0	0	0	0	0	0	0	0	0	0
ENSMUSG00000112827	0	0	0	0	0	0	5	0	0	0
ENSMUSG00000108647	0	0	0	0	0	0	0	0	0	0
ENSMUSG00000108646	0	0	0	0	0	0	0	0	0	0
ENSMUSG00000081514	0	0	0	0	0	0	0	0	0	0
ENSMUSG00000105343	0	0	0	0	0	0	0	0	0	0
ENSMUSG00000054727	0	0	0	0	0	0	0	0	0	0
ENSMUSG00000105346	0	0	0	0	0	3	0	0	0	0
ENSMUSG00000067360	0	0	0	0	0	0	0	0	0	0
ENSMUSG00000105344	6	0	2	0	6	0	5	2	8	3
ENSMUSG00000096605	0	0	0	0	0	0	0	0	0	0
ENSMUSG00000023914	20	0	15	147	9	0	15	9	0	5
ENSMUSG00000096607	0	0	0	0	0	0	6	0	0	0
ENSMUSG00000096606	151	172	239	233	183	211	185	181	155	136
ENSMUSG00000023911	29	30	21	30	18	44	89	51	33	42
ENSMUSG00000023918	8	19	14	9	15	38	45	26	29	9
ENSMUSG00000050394	90	132	103	132	43	74	157	117	86	114
ENSMUSG00000091950	0	0	0	0	3	0	0	0	1	0
ENSMUSG00000091956	23	15	22	34	0	11	21	44	21	24
ENSMUSG00000064928	0	0	0	0	0	0	0	0	0	0
ENSMUSG00000105967	0	1	1	0	0	1	1	1	1	1
ENSMUSG00000105964	0	0	0	0	0	0	0	0	0	0
ENSMUSG00000105963	0	0	0	0	0	0	0	0	0	0
ENSMUSG00000105961	0	0	0	0	0	0	3	0	0	0
ENSMUSG00000105968	0	0	0	0	0	0	0	0	0	0
ENSMUSG00000097202	0	0	0	0	0	0	0	0	0	0
ENSMUSG00000048709	2	1	0	0	1	1	1	0	0	0
ENSMUSG00000101044	0	0	0	0	0	0	0	0	0	0
ENSMUSG00000101045	0	0	0	0	0	0	0	0	0	0
ENSMUSG00000101046	0	0	0	0	0	0	0	0	0	0
ENSMUSG00000101040	0	0	0	0	0	0	0	0	0	0
ENSMUSG00000101041	0	0	0	0	0	0	0	0	0	0

ENSMUSG00000101042	0	0	0	0	0	0	0	0	0	0
ENSMUSG00000073514	11	2	3	3	6	21	27	3	14	13
ENSMUSG00000056946	0	0	0	0	0	0	0	0	0	0
ENSMUSG00000110039	0	0	0	0	0	0	0	0	0	0
ENSMUSG00000021214	0	0	0	0	2	0	0	5	6	9
ENSMUSG00000021216	0	0	0	0	0	0	0	0	0	0
ENSMUSG00000021217	246	303	335	265	321	503	713	453	478	433
ENSMUSG00000021210	0	0	0	0	0	0	0	0	0	0
ENSMUSG00000026247	16	0	9	10	0	0	3	1	3	0
ENSMUSG00000024125	0	0	0	0	0	0	0	0	0	0
ENSMUSG00000021213	127	97	148	214	128	175	256	180	239	165
ENSMUSG00000111033	0	0	0	0	0	0	0	0	0	0
ENSMUSG00000111035	0	0	1	0	2	3	0	0	0	0
ENSMUSG00000113883	6	0	0	3	0	0	3	0	0	0
ENSMUSG00000110033	0	0	0	0	0	0	0	0	0	0
ENSMUSG00000113881	3	0	0	0	0	0	4	0	0	0
ENSMUSG00000113880	1	7	3	9	0	4	3	2	5	3
ENSMUSG00000113887	0	0	0	0	0	0	0	0	0	0
ENSMUSG00000110032	0	0	0	0	0	0	0	0	0	0
ENSMUSG00000113888	0	0	0	0	0	0	0	0	0	0
ENSMUSG00000024124	27	3	12	13	6	15	19	9	5	9
ENSMUSG00000089090	0	0	0	0	0	0	0	0	0	0
ENSMUSG00000063975	0	0	0	0	0	0	0	0	3	2
ENSMUSG00000089091	0	0	0	0	0	0	0	0	0	0
ENSMUSG00000110037	0	0	0	0	0	0	0	0	0	0
ENSMUSG00000064842	0	0	0	0	0	0	0	0	0	0
ENSMUSG00000064840	0	0	0	0	0	0	0	0	0	0
ENSMUSG00000110036	0	0	0	0	0	0	0	0	0	0
ENSMUSG00000091375	0	0	0	6	5	0	0	5	3	0
ENSMUSG00000091376	0	0	0	0	0	0	0	0	0	0
ENSMUSG00000084061	0	0	0	0	0	0	0	0	0	0
ENSMUSG00000110034	0	0	0	0	0	0	0	0	0	0
ENSMUSG00000089096	1	2	1	1	0	0	2	4	0	0
ENSMUSG00000081122	0	0	0	0	0	0	0	0	0	0
ENSMUSG00000081124	0	0	0	0	0	0	0	0	0	0
ENSMUSG00000081127	0	0	0	0	0	0	0	0	1	0
ENSMUSG00000081129	0	0	0	0	0	0	0	0	0	0
ENSMUSG00000095220	0	0	0	0	0	0	0	1	0	0
ENSMUSG00000105210	0	0	0	0	0	0	0	0	0	0
ENSMUSG00000035274	82	85	111	52	79	86	266	176	180	130
ENSMUSG00000090773	0	0	0	0	0	0	0	0	0	0
ENSMUSG00000090772	0	0	0	0	0	0	0	0	0	0
ENSMUSG00000081698	0	0	0	0	0	0	0	0	0	0
ENSMUSG00000081699	0	0	0	0	0	0	0	0	0	0
ENSMUSG00000090777	2	4	0	8	0	0	0	0	0	0
ENSMUSG00000090774	0	0	0	0	0	0	0	0	0	0
ENSMUSG00000081693	0	0	0	0	0	0	0	0	0	0
ENSMUSG00000081690	7	4	7	4	3	6	8	4	2	11
ENSMUSG00000081696	0	0	0	0	0	0	0	0	0	0
ENSMUSG00000081697	0	0	0	0	0	0	0	0	0	0
ENSMUSG00000081694	0	0	0	0	0	0	0	0	0	0
ENSMUSG00000058399	0	0	0	0	0	0	0	0	0	0
ENSMUSG00000058398	3	2	0	0	0	0	0	0	8	4
ENSMUSG00000094471	0	0	0	0	0	0	0	0	0	0
ENSMUSG00000094472	0	0	0	0	0	0	0	0	0	0
ENSMUSG00000094473	0	0	0	0	0	0	0	0	0	0
ENSMUSG00000094474	0	0	0	0	0	0	0	0	0	0
ENSMUSG00000094476	0	0	0	0	0	0	0	0	0	0

ENSMUSG00000094477	0	0	0	0	1	2	0	0	0	0
ENSMUSG00000056055	27	37	33	34	9	64	46	29	32	16
ENSMUSG00000086478	0	0	0	0	0	0	0	0	0	0
ENSMUSG00000086474	0	0	0	0	0	0	0	0	0	0
ENSMUSG00000088721	0	0	0	0	0	0	0	0	0	0
ENSMUSG00000105215	0	0	0	0	0	0	0	0	0	0
ENSMUSG00000046755	0	0	0	0	0	0	0	0	0	0
ENSMUSG00000044976	9	32	9	33	18	31	12	51	9	32
ENSMUSG00000052537	0	0	0	0	0	0	0	0	0	0
ENSMUSG00000077001	0	0	0	0	0	2	1	0	0	0
ENSMUSG00000105214	0	0	0	0	0	0	0	0	0	0
ENSMUSG00000055629	8	8	5	5	0	11	16	30	5	15
ENSMUSG00000055621	0	0	0	0	0	0	0	0	0	0
ENSMUSG00000094294	0	0	0	0	0	0	0	0	0	0
ENSMUSG00000094295	0	0	0	0	0	0	0	0	0	0
ENSMUSG00000094296	0	0	0	0	0	0	0	0	0	0
ENSMUSG00000094297	0	0	0	0	0	0	0	0	0	0
ENSMUSG00000094290	0	0	0	0	0	0	0	0	0	0
ENSMUSG00000094291	0	0	0	0	0	0	0	0	0	0
ENSMUSG00000027801	3	6	11	12	3	0	3	8	0	5
ENSMUSG00000094293	0	0	0	0	0	5	0	0	0	0
ENSMUSG00000093411	0	0	0	0	0	0	0	0	0	0
ENSMUSG00000094298	0	0	0	0	0	0	0	0	0	0
ENSMUSG00000028351	15	7	3	9	6	12	11	14	0	12
ENSMUSG00000028356	6	4	0	6	0	0	0	0	6	7
ENSMUSG00000028357	0	0	5	3	0	0	5	0	0	6
ENSMUSG00000097929	0	0	0	0	0	0	0	0	3	4
ENSMUSG00000028358	64	46	68	31	75	88	119	69	57	110
ENSMUSG00000097924	28	25	36	32	26	38	29	12	28	15
ENSMUSG00000097921	14	16	17	55	20	15	9	9	22	11
ENSMUSG00000067555	13	0	0	0	0	1	0	0	0	0
ENSMUSG00000016239	371	275	322	351	266	179	279	167	287	184
ENSMUSG00000105993	0	0	0	0	0	0	0	0	0	0
ENSMUSG00000055088	0	0	0	0	0	0	0	0	0	0
ENSMUSG00000105996	0	0	0	0	0	0	0	0	0	0
ENSMUSG00000049353	7	5	12	17	5	35	5	2	15	10
ENSMUSG00000049350	3	10	0	0	4	0	0	8	0	0
ENSMUSG00000105994	0	0	0	0	0	0	0	0	0	0
ENSMUSG00000029409	0	0	0	0	0	0	0	0	0	0
ENSMUSG00000109490	0	0	0	0	0	0	0	0	0	0
ENSMUSG00000113308	0	0	0	0	0	0	2	0	0	0
ENSMUSG00000113309	0	0	0	0	0	0	2	0	0	0
ENSMUSG00000106083	0	0	0	0	0	0	0	0	0	0
ENSMUSG00000106085	0	0	0	0	0	0	0	0	0	0
ENSMUSG00000106084	0	0	0	0	0	0	0	0	0	0
ENSMUSG00000113300	0	0	0	0	0	0	0	0	0	0
ENSMUSG00000106088	0	0	0	0	0	0	0	0	0	0
ENSMUSG00000113302	15	21	17	0	6	9	37	15	19	20
ENSMUSG00000113303	0	0	6	3	8	0	9	3	0	3
ENSMUSG00000113304	0	0	0	0	0	0	0	0	0	0
ENSMUSG00000113305	0	0	0	0	0	0	0	0	0	0
ENSMUSG00000113306	0	0	0	0	0	0	0	0	0	0
ENSMUSG00000054976	0	0	0	0	0	0	0	0	7	0
ENSMUSG00000035420	0	0	0	0	0	0	0	0	0	0
ENSMUSG00000071769	0	0	0	5	0	4	8	0	3	0
ENSMUSG00000071763	0	0	0	0	0	0	0	0	0	0
ENSMUSG00000071764	0	0	0	0	0	0	0	0	0	0
ENSMUSG00000071767	0	0	0	0	0	0	0	0	0	0

ENSMUSG00000071766	0	0	0	0	0	0	0	0	0	0
ENSMUSG00000109251	13	3	13	23	9	8	23	21	15	27
ENSMUSG00000109250	0	0	0	0	0	0	0	0	0	0
ENSMUSG00000109254	0	0	0	0	0	0	0	0	0	0
ENSMUSG00000051251	0	0	0	0	0	0	0	0	0	0
ENSMUSG00000109258	0	0	0	0	0	0	0	0	0	0
ENSMUSG00000106551	0	0	0	0	0	0	0	3	0	0
ENSMUSG00000106557	3	5	5	7	2	6	8	2	5	12
ENSMUSG00000106554	7	3	0	5	8	7	6	3	0	0
ENSMUSG00000092191	0	0	0	0	0	0	0	0	0	0
ENSMUSG00000092193	0	0	0	0	0	0	0	0	0	0
ENSMUSG00000092195	0	0	0	0	0	0	0	0	0	0
ENSMUSG00000092197	0	0	0	0	0	0	0	0	0	0
ENSMUSG00000092196	0	0	0	0	0	0	0	0	0	0
ENSMUSG00000092199	0	0	0	0	0	0	0	0	0	0
ENSMUSG00000088691	0	0	0	0	0	0	0	0	0	0
ENSMUSG00000105316	0	0	3	3	3	0	0	3	2	0
ENSMUSG00000105159	0	0	0	0	0	0	0	0	0	0
ENSMUSG00000025188	167	167	185	211	182	203	460	304	396	251
ENSMUSG00000025189	17	6	0	12	6	21	21	15	6	8
ENSMUSG00000082578	0	0	3	0	0	0	0	0	0	0
ENSMUSG00000082579	0	0	0	0	0	0	0	0	0	0
ENSMUSG00000114653	1	0	0	0	3	0	2	6	11	6
ENSMUSG00000114652	0	0	0	0	0	0	6	3	0	0
ENSMUSG00000114651	0	0	0	0	0	0	0	0	0	0
ENSMUSG00000082573	0	0	0	0	0	0	0	0	0	0
ENSMUSG00000105317	0	0	0	0	0	0	0	0	0	0
ENSMUSG00000082577	0	0	0	0	0	0	0	0	0	0
ENSMUSG00000082574	0	0	0	0	0	0	0	0	0	0
ENSMUSG00000114658	0	0	0	0	0	0	0	0	0	0
ENSMUSG00000110147	0	0	0	0	0	0	0	0	0	0
ENSMUSG00000110145	0	0	0	0	0	0	0	0	0	0
ENSMUSG00000110143	0	0	0	0	0	0	0	0	0	0
ENSMUSG00000110140	0	0	0	3	0	4	6	2	4	3
ENSMUSG00000110149	0	0	0	0	0	0	0	0	0	0
ENSMUSG00000083229	0	0	0	0	0	0	0	0	0	0
ENSMUSG00000083228	0	0	0	0	0	0	0	0	0	0
ENSMUSG00000083221	0	0	0	0	0	0	0	0	0	0
ENSMUSG00000083223	0	0	2	0	0	2	0	0	0	1
ENSMUSG00000083222	0	0	0	0	0	0	0	0	0	0
ENSMUSG00000083225	0	0	0	0	0	0	0	0	0	0
ENSMUSG00000083224	0	0	0	0	0	0	0	0	0	0
ENSMUSG00000083227	0	0	0	0	0	0	0	0	0	0
ENSMUSG00000083226	0	0	2	3	0	0	0	3	0	2
ENSMUSG00000070498	0	6	6	0	0	10	6	9	12	6
ENSMUSG00000025231	423	514	521	505	430	645	690	572	570	624
ENSMUSG00000070495	9	9	23	17	11	10	13	10	36	36
ENSMUSG00000099178	0	0	0	0	0	0	0	0	0	0
ENSMUSG00000070141	0	0	1	0	0	0	0	0	1	0
ENSMUSG00000035896	10	18	9	30	33	18	29	62	3	12
ENSMUSG00000099173	0	0	0	0	0	0	0	0	0	0
ENSMUSG00000099170	5	0	0	0	0	0	0	0	6	0
ENSMUSG00000099176	0	0	0	0	0	0	0	0	0	0
ENSMUSG00000101673	0	0	0	0	0	0	0	0	0	0
ENSMUSG00000101672	0	0	0	0	0	0	0	0	0	0
ENSMUSG00000035057	0	0	0	0	0	0	0	0	0	0
ENSMUSG00000105313	0	0	0	0	0	0	0	0	0	0
ENSMUSG00000075512	0	0	0	2	0	0	0	0	0	0

ENSMUSG00000075511	19	24	17	28	15	38	31	31	22	23
ENSMUSG00000075517	0	0	0	0	0	0	0	0	0	0
ENSMUSG00000101676	0	0	0	0	0	0	0	0	0	0
ENSMUSG00000000730	22	28	19	51	39	34	35	32	19	26
ENSMUSG00000000731	0	0	0	0	0	5	0	2	0	2
ENSMUSG00000006777	0	0	3	6	0	9	3	3	0	3
ENSMUSG00000085107	0	0	0	0	0	0	0	0	0	0
ENSMUSG00000085104	0	0	0	0	0	0	0	0	0	0
ENSMUSG00000085817	0	0	0	0	0	0	0	0	0	0
ENSMUSG00000085101	0	0	0	0	0	0	0	0	0	0
ENSMUSG00000085108	0	0	0	0	0	0	0	0	0	0
ENSMUSG00000026765	60	18	51	58	12	20	27	0	6	26
ENSMUSG00000026764	324	252	220	289	214	372	902	392	529	348
ENSMUSG00000079342	0	0	0	0	0	0	0	0	0	0
ENSMUSG00000079346	0	0	0	0	0	0	0	0	0	0
ENSMUSG00000079349	0	0	0	0	0	0	0	0	3	0
ENSMUSG00000020297	14	0	13	21	17	15	32	18	6	21
ENSMUSG00000045217	0	0	0	0	0	0	0	0	0	0
ENSMUSG00000045215	43	93	33	24	57	51	82	48	91	42
ENSMUSG00000079966	0	0	0	0	0	0	0	0	0	0
ENSMUSG00000079963	0	0	0	0	0	0	0	0	0	0
ENSMUSG00000019817	174	280	267	432	133	282	553	357	238	209
ENSMUSG00000007827	753	681	727	776	504	718	944	816	877	807
ENSMUSG00000086838	0	0	0	0	0	0	0	0	0	0
ENSMUSG00000032915	241	172	262	330	178	204	268	238	343	316
ENSMUSG00000089905	0	0	0	0	0	0	0	0	6	0
ENSMUSG00000048562	0	0	0	0	0	0	3	0	0	0
ENSMUSG00000107935	0	0	0	0	0	0	0	0	0	0
ENSMUSG00000069998	0	0	0	0	0	0	0	0	0	0
ENSMUSG00000095128	0	0	0	0	0	0	0	0	0	0
ENSMUSG00000031758	279	257	291	268	243	266	352	297	273	241
ENSMUSG00000107933	0	0	0	0	0	0	0	0	0	0
ENSMUSG00000031756	75	74	80	67	66	75	80	66	50	65
ENSMUSG00000031757	0	0	0	0	0	0	0	0	0	0
ENSMUSG00000061584	0	0	0	0	0	0	3	0	0	0
ENSMUSG00000004842	3	0	0	0	4	6	0	9	3	0
ENSMUSG00000102889	0	0	0	0	0	0	0	0	0	0
ENSMUSG00000102885	0	0	0	0	0	0	0	0	0	0
ENSMUSG00000102884	0	0	0	0	0	0	0	0	0	0
ENSMUSG00000102887	0	0	0	0	0	0	0	3	0	0
ENSMUSG00000102886	0	0	0	0	0	0	3	0	5	3
ENSMUSG00000102880	0	0	0	0	0	0	3	0	4	2
ENSMUSG00000102883	0	0	0	0	0	0	0	0	0	0
ENSMUSG00000101282	0	0	0	0	0	0	0	0	0	0
ENSMUSG00000034987	138	89	171	148	118	102	151	211	143	173
ENSMUSG00000101280	0	0	0	0	0	0	0	0	0	0
ENSMUSG00000101281	3	0	0	0	0	0	0	0	4	0
ENSMUSG00000101286	0	0	0	0	0	0	0	0	0	0
ENSMUSG00000101287	0	0	0	0	0	0	0	0	0	0
ENSMUSG00000101284	0	0	0	0	0	0	0	0	0	0
ENSMUSG00000101285	0	0	0	0	0	0	0	0	0	0
ENSMUSG00000101288	0	0	0	0	0	0	0	0	0	0
ENSMUSG00000101289	0	0	0	0	0	0	0	0	0	0
ENSMUSG00000098861	0	0	0	0	0	0	0	0	0	0
ENSMUSG00000085951	0	0	0	0	0	0	0	0	0	0
ENSMUSG00000085950	0	0	0	0	0	0	0	0	0	0
ENSMUSG00000085953	3	0	0	0	0	0	0	0	0	0
ENSMUSG00000085952	0	0	0	0	3	0	0	0	0	0

ENSMUSG00000030406	27	19	26	15	9	20	55	16	50	20
ENSMUSG00000082635	0	0	0	0	0	0	0	0	0	0
ENSMUSG00000030402	0	7	3	3	0	7	27	3	0	12
ENSMUSG00000082637	0	0	0	0	0	0	0	0	0	0
ENSMUSG00000082636	0	0	0	0	0	0	0	0	0	0
ENSMUSG00000082639	0	0	0	0	0	0	0	0	0	0
ENSMUSG00000082638	0	0	0	0	0	0	0	0	0	0
ENSMUSG00000088296	0	0	0	0	0	0	0	0	0	0
ENSMUSG00000088291	0	0	0	1	0	1	0	0	0	0
ENSMUSG00000040632	0	6	3	0	6	0	0	0	0	3
ENSMUSG00000060303	0	0	0	0	0	0	0	0	0	0
ENSMUSG00000037759	48	32	38	35	27	78	115	69	102	44
ENSMUSG00000107938	0	0	0	0	0	0	0	0	0	0
ENSMUSG00000100628	0	0	0	0	0	0	0	0	0	0
ENSMUSG00000100627	4	4	7	8	0	20	17	16	0	10
ENSMUSG00000100626	0	1	1	2	0	1	1	2	0	0
ENSMUSG00000100625	0	0	0	0	0	0	0	0	0	0
ENSMUSG00000100624	0	0	0	0	0	0	0	0	0	0
ENSMUSG00000094654	0	0	0	0	0	0	0	0	0	0
ENSMUSG00000086788	0	0	0	0	0	0	0	0	0	0
ENSMUSG00000093908	38	34	44	26	36	76	71	52	53	50
ENSMUSG00000050625	9	0	6	0	5	3	0	6	0	3
ENSMUSG00000059939	262	311	244	252	209	335	387	239	368	289
ENSMUSG00000103294	0	0	0	0	0	0	0	0	0	0
ENSMUSG00000059934	0	0	0	0	0	0	0	0	0	0
ENSMUSG00000093607	0	0	0	0	0	0	0	0	0	0
ENSMUSG00000084776	0	0	0	0	0	0	0	0	0	0
ENSMUSG00000084774	0	0	0	0	0	0	0	0	0	0
ENSMUSG00000084775	6	15	12	3	12	18	8	0	21	12
ENSMUSG00000084772	2	3	0	3	0	2	0	7	0	0
ENSMUSG00000084773	0	0	0	0	0	0	0	0	0	0
ENSMUSG00000050623	18	20	16	21	17	15	20	9	12	8
ENSMUSG00000086785	2	0	0	3	0	1	0	3	0	0
ENSMUSG00000032131	9	23	9	9	12	9	61	28	30	25
ENSMUSG00000022456	20	40	27	36	32	95	195	107	106	54
ENSMUSG00000022455	0	0	0	0	0	0	0	0	0	3
ENSMUSG00000022454	0	3	0	0	0	3	6	0	0	3
ENSMUSG00000095282	0	0	0	0	0	0	0	0	0	0
ENSMUSG00000102715	0	4	0	0	0	0	0	0	0	2
ENSMUSG00000102716	0	0	0	0	0	0	0	0	0	0
ENSMUSG00000102710	0	0	0	0	0	0	0	0	0	0
ENSMUSG00000102712	0	2	3	0	5	0	0	3	0	1
ENSMUSG00000024857	0	0	0	0	0	0	0	0	0	0
ENSMUSG00000102718	0	0	0	0	0	0	0	0	0	0
ENSMUSG00000012350	23	3	3	14	12	7	32	0	21	12
ENSMUSG00000060878	6	8	2	5	5	5	11	13	14	9
ENSMUSG00000062434	0	0	0	0	0	0	0	0	0	0
ENSMUSG00000062433	0	0	0	0	0	0	0	0	0	0
ENSMUSG00000062432	21	16	13	18	3	8	46	17	35	24
ENSMUSG00000113038	0	0	0	0	0	0	0	0	0	0
ENSMUSG00000038259	0	0	0	0	0	3	6	0	3	0
ENSMUSG00000021439	0	0	0	0	0	0	0	0	0	0
ENSMUSG00000094904	0	1	1	0	0	1	1	0	0	1
ENSMUSG00000094905	0	0	0	0	0	0	0	0	0	0
ENSMUSG00000094901	0	0	0	0	0	0	0	0	0	0
ENSMUSG00000094903	0	0	0	0	0	0	0	0	0	0
ENSMUSG00000095283	0	0	0	0	0	0	0	0	0	0
ENSMUSG00000038253	46	50	48	53	54	36	185	75	98	51

ENSMUSG00000029646	0	0	0	0	0	0	0	0	0	0
ENSMUSG00000029644	0	0	0	0	0	0	0	0	0	0
ENSMUSG00000036931	69	23	37	58	44	50	74	54	73	67
ENSMUSG00000036938	0	0	0	0	0	0	0	0	0	0
ENSMUSG00000034774	3	0	0	0	0	6	0	0	0	0
ENSMUSG00000113146	0	0	0	0	0	0	3	0	3	3
ENSMUSG00000113144	0	0	0	0	0	0	0	0	0	0
ENSMUSG00000113145	0	0	0	0	0	0	0	0	0	0
ENSMUSG00000113034	0	0	0	0	0	0	0	0	0	0
ENSMUSG00000001510	0	0	0	0	5	0	6	0	0	6
ENSMUSG00000113035	4	0	0	4	2	6	4	11	6	6
ENSMUSG00000113148	0	0	0	0	0	0	0	0	0	0
ENSMUSG00000034482	2	0	0	0	5	4	2	9	0	2
ENSMUSG00000051095	0	2	1	0	0	0	0	0	0	0
ENSMUSG00000097291	0	0	0	1	0	0	0	6	3	0
ENSMUSG00000106718	0	0	0	0	0	0	3	0	3	3
ENSMUSG00000106710	0	3	6	3	0	0	6	4	3	3
ENSMUSG00000106713	0	0	0	0	0	0	0	0	0	0
ENSMUSG00000106714	6	13	9	11	8	18	15	16	14	14
ENSMUSG00000038094	0	0	0	0	0	0	3	0	3	0
ENSMUSG00000104712	3	0	0	0	0	0	0	0	0	0
ENSMUSG00000104711	0	0	0	0	0	0	0	0	0	0
ENSMUSG00000104710	2	0	3	0	0	3	9	0	6	9
ENSMUSG00000104717	0	0	0	0	0	0	0	0	0	0
ENSMUSG00000104716	0	0	0	0	0	0	0	0	3	0
ENSMUSG00000104715	0	0	0	0	0	0	0	0	0	0
ENSMUSG00000104714	0	0	0	0	0	0	0	0	0	0
ENSMUSG00000097299	0	0	0	0	0	0	2	0	0	0
ENSMUSG00000104718	0	0	0	0	0	0	0	0	0	0
ENSMUSG00000020897	103	81	134	146	116	34	134	51	83	49
ENSMUSG00000111788	0	0	0	0	0	0	0	0	0	0
ENSMUSG00000111782	0	0	0	0	0	0	0	0	2	0
ENSMUSG00000109720	0	0	0	0	0	0	0	0	0	0
ENSMUSG00000111780	0	0	0	0	0	0	0	0	0	0
ENSMUSG00000111781	0	0	0	0	0	0	0	0	0	0
ENSMUSG00000111786	0	0	0	0	0	0	0	0	0	0
ENSMUSG00000111787	0	0	0	0	0	0	0	0	0	0
ENSMUSG00000109726	0	0	0	0	0	0	0	0	0	0
ENSMUSG00000114495	0	0	0	0	0	0	0	0	0	0
ENSMUSG00000114494	0	0	0	0	0	0	0	0	0	0
ENSMUSG00000114497	0	0	0	0	0	0	0	0	0	0
ENSMUSG00000114491	0	0	0	0	0	0	0	0	0	0
ENSMUSG00000114490	0	0	0	0	0	0	0	0	0	0
ENSMUSG00000114499	0	0	0	0	0	0	0	0	0	0
ENSMUSG00000095193	33	60	32	39	37	20	80	75	91	78
ENSMUSG00000097767	6	3	17	13	6	15	68	20	18	15
ENSMUSG00000095883	0	0	0	0	0	0	0	0	0	0
ENSMUSG00000095880	0	0	0	0	0	0	0	0	0	0
ENSMUSG00000104050	0	0	0	0	0	0	0	0	0	0
ENSMUSG00000109796	0	0	0	0	0	0	0	0	0	0
ENSMUSG00000101023	0	0	0	0	0	0	0	0	0	0
ENSMUSG00000025431	0	0	0	0	0	9	0	0	0	6
ENSMUSG00000104054	0	0	0	0	0	0	0	0	0	0
ENSMUSG00000104055	0	0	0	0	0	0	0	0	0	0
ENSMUSG00000050058	0	0	0	0	0	0	0	2	0	0
ENSMUSG00000044320	6	12	3	2	10	19	13	11	11	17
ENSMUSG00000044322	0	0	0	0	0	0	6	3	0	0
ENSMUSG00000110550	0	0	0	0	0	0	0	0	0	0

ENSMUSG00000110552	0	0	0	0	0	0	0	0	0	0
ENSMUSG00000110554	0	0	3	0	0	0	0	0	0	0
ENSMUSG00000110557	0	0	0	0	0	0	0	0	0	0
ENSMUSG00000110559	0	0	0	0	0	0	3	0	0	0
ENSMUSG00000059463	0	0	0	0	0	0	0	0	0	0
ENSMUSG00000060469	0	0	0	0	0	0	0	0	0	0
ENSMUSG00000050933	0	0	0	0	0	0	0	0	0	0
ENSMUSG00000026167	3	3	3	0	0	6	18	9	3	0
ENSMUSG00000026166	0	0	0	0	0	0	0	0	0	0
ENSMUSG00000061048	36	61	38	43	36	70	239	114	75	110
ENSMUSG00000096414	0	0	0	0	0	0	0	0	0	0
ENSMUSG00000096412	0	0	0	0	0	0	0	0	0	0
ENSMUSG00000096413	0	0	0	0	0	0	0	0	0	0
ENSMUSG00000090951	1	0	0	0	0	0	0	3	0	0
ENSMUSG00000080443	0	0	0	0	0	0	0	0	0	0
ENSMUSG00000013562	6	4	0	19	0	2	0	2	7	1
ENSMUSG00000045052	0	0	0	3	0	0	0	0	3	0
ENSMUSG00000045053	0	3	0	0	3	0	3	0	0	0
ENSMUSG00000113924	0	0	0	0	0	1	0	1	0	0
ENSMUSG00000113925	0	0	0	0	0	0	1	0	0	0
ENSMUSG00000113927	0	0	0	0	0	0	0	0	0	0
ENSMUSG00000099728	0	0	0	0	0	0	0	0	0	0
ENSMUSG00000095192	0	0	0	0	0	0	0	0	0	0
ENSMUSG00000095195	8	8	8	0	3	21	7	9	0	12
ENSMUSG00000095196	0	0	0	1	0	2	1	0	0	0
ENSMUSG00000111207	0	0	0	0	0	0	0	3	0	0
ENSMUSG00000111205	9	3	0	4	12	5	0	4	4	8
ENSMUSG00000111204	8	9	0	5	2	4	3	8	8	4
ENSMUSG00000111203	0	0	0	0	0	0	0	0	2	0
ENSMUSG00000111202	15	18	22	14	11	24	17	22	13	15
ENSMUSG00000111201	0	0	0	0	0	0	0	0	0	0
ENSMUSG00000045589	9	12	8	3	6	18	6	20	6	11
ENSMUSG00000077553	0	0	0	0	0	0	0	0	1	0
ENSMUSG00000081719	0	0	0	0	0	0	0	0	0	0
ENSMUSG00000081718	2	0	0	2	0	0	0	0	0	0
ENSMUSG00000081717	0	0	0	0	0	0	0	0	0	0
ENSMUSG00000099723	0	0	0	0	0	0	0	0	0	0
ENSMUSG00000081714	0	0	0	0	0	0	0	0	0	0
ENSMUSG00000081713	0	0	0	0	0	0	0	0	0	0
ENSMUSG00000081711	2	0	0	0	0	0	0	0	0	0
ENSMUSG00000077555	0	0	0	0	0	0	0	0	0	0
ENSMUSG00000102707	0	0	0	0	0	0	0	0	0	0
ENSMUSG00000028938	0	0	0	0	0	0	3	3	0	3
ENSMUSG00000086848	0	0	0	0	0	0	0	0	0	0
ENSMUSG00000086842	3	6	6	0	0	0	0	3	0	0
ENSMUSG00000018470	10	5	24	12	17	10	22	31	13	16
ENSMUSG00000086840	0	0	0	0	0	0	0	0	0	0
ENSMUSG00000086847	0	9	9	0	0	5	0	4	4	4
ENSMUSG00000086844	16	15	18	9	8	13	17	17	12	23
ENSMUSG00000086845	0	0	0	0	6	3	0	0	6	0
ENSMUSG00000052182	0	0	0	0	0	0	0	0	0	0
ENSMUSG00000052180	3	0	6	0	0	3	15	5	0	0
ENSMUSG00000052187	0	0	0	3	0	0	0	0	0	0
ENSMUSG00000090927	0	0	0	0	0	0	0	0	0	0
ENSMUSG00000090925	0	2	3	0	0	0	0	0	0	3
ENSMUSG00000048763	119	43	78	44	91	107	206	121	184	143
ENSMUSG00000048764	0	0	0	0	0	0	0	0	0	0
ENSMUSG00000048766	0	0	0	0	0	0	0	0	0	0

ENSMUSG00000077633	0	0	0	0	1	0	0	1	0	0
ENSMUSG00000103142	0	0	0	0	0	0	0	0	0	0
ENSMUSG00000093699	0	0	0	0	0	0	0	0	0	0
ENSMUSG00000042050	390	449	513	412	442	624	776	615	707	580
ENSMUSG00000093696	0	0	0	0	0	0	0	0	0	0
ENSMUSG00000093692	0	0	0	0	0	0	0	0	0	0
ENSMUSG00000101022	0	0	0	0	0	0	0	0	0	0
ENSMUSG00000109522	0	0	0	0	0	0	0	0	0	0
ENSMUSG00000099758	0	0	0	0	0	0	0	0	0	0
ENSMUSG00000099759	80	25	89	14	54	36	111	85	43	98
ENSMUSG00000099752	0	2	0	0	0	0	0	0	0	0
ENSMUSG00000049797	0	0	0	0	0	0	0	0	0	0
ENSMUSG00000099750	0	0	0	0	0	0	3	0	0	0
ENSMUSG00000099751	0	0	0	0	0	0	0	0	0	0
ENSMUSG00000099756	0	0	0	0	0	0	0	0	0	0
ENSMUSG00000108881	0	0	0	0	0	0	0	0	0	0
ENSMUSG00000099754	0	0	0	0	0	0	0	0	0	0
ENSMUSG00000099755	0	0	0	0	0	0	0	0	0	0
ENSMUSG00000108887	0	0	0	0	0	0	0	0	0	0
ENSMUSG00000109524	0	0	0	0	0	0	0	0	0	0
ENSMUSG00000108885	0	0	0	0	0	0	0	0	0	0
ENSMUSG00000046774	6	3	9	0	3	5	36	0	0	0
ENSMUSG00000108884	3	0	8	18	0	0	0	0	3	0
ENSMUSG00000100847	0	0	0	0	0	0	0	0	0	0
ENSMUSG00000100845	0	0	0	0	0	0	0	0	0	0
ENSMUSG00000049498	0	0	0	0	0	0	0	0	0	0
ENSMUSG00000086159	0	0	0	0	0	0	0	0	0	0
ENSMUSG00000100844	0	0	0	0	0	0	0	0	0	0
ENSMUSG00000086150	10	22	13	22	12	7	28	10	9	9
ENSMUSG00000049491	0	0	0	0	0	0	0	0	3	0
ENSMUSG00000086152	0	0	0	0	0	0	0	0	0	0
ENSMUSG00000049493	45	27	60	499	127	30	44	293	118	174
ENSMUSG00000086154	0	2	0	8	2	0	0	0	6	3
ENSMUSG00000100841	0	0	0	0	0	0	0	0	0	0
ENSMUSG00000049148	57	96	66	90	74	174	242	142	180	97
ENSMUSG00000049149	5	3	5	3	3	5	3	5	9	7
ENSMUSG00000098807	2	0	0	3	0	0	0	0	0	0
ENSMUSG00000076272	0	1	0	1	0	1	1	0	0	1
ENSMUSG00000087409	0	0	0	0	0	0	0	0	0	0
ENSMUSG00000076274	0	0	0	0	0	0	0	0	0	0
ENSMUSG00000082437	0	0	0	0	0	0	0	0	0	0
ENSMUSG00000082435	0	0	0	0	0	0	0	0	0	0
ENSMUSG00000077205	0	0	0	0	0	0	0	0	0	0
ENSMUSG00000082432	0	0	0	0	0	0	0	0	0	0
ENSMUSG00000087403	110	123	138	124	72	167	230	190	212	208
ENSMUSG00000083528	0	0	2	2	0	0	2	0	1	2
ENSMUSG00000083522	0	0	0	0	0	0	0	0	0	0
ENSMUSG00000045877	4	28	3	11	14	23	0	14	14	11
ENSMUSG00000083520	0	0	0	0	0	0	0	0	0	3
ENSMUSG00000083527	0	3	0	0	0	0	0	0	0	3
ENSMUSG00000083524	0	0	0	0	0	0	0	0	0	0
ENSMUSG00000045871	219	204	196	87	239	140	100	150	104	267
ENSMUSG00000004317	618	725	703	706	551	970	1335	806	898	972
ENSMUSG00000111366	0	0	0	0	0	0	0	3	0	0
ENSMUSG00000112376	21	35	43	52	20	40	63	69	43	36
ENSMUSG00000070841	0	0	0	0	0	0	0	0	0	0
ENSMUSG00000070847	0	0	0	0	0	0	2	3	0	0
ENSMUSG00000070844	0	0	0	0	0	0	0	0	0	0

ENSMUSG00000071138	0	0	0	0	0	2	0	0	0	0
ENSMUSG00000040340	0	3	0	0	0	0	0	0	2	0
ENSMUSG00000021850	3	2	3	9	0	0	1	7	2	3
ENSMUSG00000102650	0	0	4	0	3	3	0	3	0	1
ENSMUSG00000084179	0	0	0	0	0	0	0	0	0	0
ENSMUSG00000083388	0	0	0	0	0	0	0	0	0	0
ENSMUSG00000083389	0	0	0	0	0	0	0	0	0	0
ENSMUSG00000083386	0	0	6	3	0	0	2	0	3	3
ENSMUSG00000083387	0	0	0	0	0	0	0	0	0	0
ENSMUSG00000083384	0	0	0	0	0	0	0	0	0	0
ENSMUSG00000083385	0	0	0	0	0	0	0	0	0	0
ENSMUSG00000083382	13	7	5	9	8	16	16	7	4	8
ENSMUSG00000084171	0	0	0	0	0	0	0	0	0	0
ENSMUSG00000084172	0	0	0	0	1	0	6	0	0	0
ENSMUSG00000084173	0	0	0	0	0	0	0	0	0	0
ENSMUSG00000091929	0	0	0	0	0	0	0	0	0	0
ENSMUSG00000025375	407	328	456	441	397	497	560	490	460	609
ENSMUSG00000112812	0	0	0	0	0	0	0	0	0	0
ENSMUSG00000080871	0	0	0	0	0	0	0	0	0	0
ENSMUSG00000080870	5	3	15	11	20	11	17	6	9	2
ENSMUSG00000080872	0	0	0	0	0	0	0	0	0	0
ENSMUSG00000080874	0	0	0	0	0	0	0	0	0	0
ENSMUSG00000080876	0	0	0	0	0	0	0	0	0	0
ENSMUSG00000108691	0	0	3	2	0	0	0	6	2	8
ENSMUSG00000100378	0	0	0	0	0	0	0	0	0	0
ENSMUSG00000108696	0	9	1	3	3	0	0	7	1	0
ENSMUSG00000112379	3	0	0	0	0	0	0	2	0	0
ENSMUSG00000040966	3	3	3	27	0	0	0	3	0	0
ENSMUSG00000040963	41	33	26	26	11	15	39	34	27	29
ENSMUSG00000001095	0	0	0	0	0	0	2	0	0	0
ENSMUSG00000040969	0	0	6	9	0	5	9	0	0	3
ENSMUSG00000112819	0	0	0	0	0	0	0	0	0	0
ENSMUSG00000075726	0	0	0	0	0	0	0	0	0	0
ENSMUSG00000044040	0	0	0	0	0	0	0	0	0	0
ENSMUSG00000044041	0	0	0	0	0	0	0	0	0	0
ENSMUSG00000044042	134	118	162	145	124	153	784	193	721	512
ENSMUSG00000070240	0	0	0	0	0	2	0	0	0	0
ENSMUSG00000090089	0	0	0	0	0	0	0	0	0	0
ENSMUSG00000070263	0	0	0	0	0	0	0	0	0	0
ENSMUSG00000105291	0	0	0	0	0	0	0	0	0	0
ENSMUSG00000099034	106	106	77	110	81	79	150	90	117	123
ENSMUSG00000099031	0	1	0	0	0	1	1	1	2	0
ENSMUSG00000032033	18	6	18	12	12	17	18	11	0	0
ENSMUSG00000070249	0	0	0	0	0	0	0	0	0	0
ENSMUSG00000030889	504	544	667	453	506	782	241	418	405	493
ENSMUSG00000086326	0	0	0	0	0	0	0	0	0	0
ENSMUSG00000099013	0	0	0	0	0	0	0	0	0	0
ENSMUSG00000034339	0	0	0	0	0	0	0	1	0	6
ENSMUSG00000000942	12	6	6	3	9	11	46	6	19	24
ENSMUSG00000065173	0	0	0	0	0	0	0	0	0	0
ENSMUSG00000099014	0	0	0	0	0	0	0	0	0	0
ENSMUSG00000085285	0	0	0	0	0	0	0	0	0	0
ENSMUSG00000105310	0	0	0	0	0	0	0	0	0	0
ENSMUSG00000067352	0	0	0	0	0	0	0	0	0	0
ENSMUSG00000110209	0	0	0	0	0	0	0	0	0	0
ENSMUSG00000062184	58	88	51	21	59	67	100	84	66	66
ENSMUSG00000062186	0	0	0	0	0	0	0	0	0	0
ENSMUSG00000062181	12	6	9	9	0	5	0	3	12	0

ENSMUSG00000081832	0	0	0	0	0	0	0	0	0	0
ENSMUSG00000081831	0	0	0	0	0	0	0	0	0	0
ENSMUSG00000081836	0	0	0	0	0	0	0	0	0	0
ENSMUSG00000081834	0	1	0	0	0	0	0	0	0	0
ENSMUSG00000081835	3	0	2	0	0	2	9	0	5	0
ENSMUSG00000073761	0	0	0	0	0	3	0	0	0	3
ENSMUSG00000073765	0	0	0	0	0	3	0	0	3	0
ENSMUSG00000073764	0	0	0	0	0	0	0	0	0	0
ENSMUSG00000073769	0	0	0	0	0	0	0	0	0	3
ENSMUSG00000073768	0	0	0	0	0	0	0	0	0	3
ENSMUSG00000063582	0	0	0	0	0	0	0	0	0	0
ENSMUSG00000063583	0	0	0	0	0	0	0	0	0	0
ENSMUSG00000098078	0	0	1	0	0	0	0	0	4	0
ENSMUSG00000098079	0	0	0	0	0	0	0	0	0	0
ENSMUSG00000098070	0	0	0	0	0	0	0	0	0	0
ENSMUSG00000098071	0	0	0	0	0	0	0	0	0	0
ENSMUSG00000098072	0	0	0	0	3	0	0	0	0	0
ENSMUSG00000098073	0	0	0	0	0	0	0	0	0	0
ENSMUSG00000098074	0	0	0	0	0	0	0	0	0	0
ENSMUSG00000098075	0	0	0	0	0	0	0	0	0	0
ENSMUSG00000098077	0	0	0	0	0	0	0	0	0	0
ENSMUSG00000110718	0	0	0	0	0	0	0	0	0	0
ENSMUSG00000087491	0	0	0	0	0	0	0	0	0	0
ENSMUSG00000102633	3	3	6	0	3	3	3	8	6	3
ENSMUSG00000110710	27	9	11	6	32	19	16	17	15	13
ENSMUSG00000066912	0	0	0	0	0	0	0	0	0	0
ENSMUSG00000110717	0	0	0	0	0	0	0	0	0	0
ENSMUSG00000084923	0	2	5	0	0	0	0	0	0	2
ENSMUSG00000084921	0	0	0	0	0	0	0	0	0	0
ENSMUSG00000084920	0	0	0	0	0	0	0	0	0	0
ENSMUSG00000084927	0	0	0	0	0	0	2	0	0	1
ENSMUSG00000084924	0	0	0	0	0	0	0	0	0	0
ENSMUSG00000043073	0	0	3	0	0	0	0	3	0	3
ENSMUSG00000091528	0	0	0	0	0	0	0	0	0	0
ENSMUSG00000086730	0	0	0	0	0	0	9	0	2	0
ENSMUSG00000091526	0	0	0	0	0	0	0	0	5	0
ENSMUSG00000071855	58	60	99	84	69	49	157	84	111	99
ENSMUSG00000043087	0	0	0	0	0	0	0	0	0	0
ENSMUSG00000043089	2	0	0	0	0	0	0	0	0	0
ENSMUSG00000043088	41	56	57	42	27	59	87	76	41	67
ENSMUSG00000030302	140	87	93	64	52	117	62	163	82	64
ENSMUSG00000030307	3	0	0	0	0	0	0	3	0	0
ENSMUSG00000038527	156	101	148	198	94	273	410	211	301	216
ENSMUSG00000100296	0	0	0	0	0	0	0	0	0	0
ENSMUSG00000100297	0	0	0	0	0	0	0	0	0	0
ENSMUSG00000100294	0	0	0	0	0	0	0	0	0	0
ENSMUSG00000100295	0	0	0	0	0	0	0	0	0	0
ENSMUSG00000087360	4	0	3	5	3	7	2	0	9	0
ENSMUSG00000100290	0	0	0	0	0	0	0	0	0	0
ENSMUSG00000087363	0	0	0	3	0	4	0	3	3	0
ENSMUSG00000087369	0	0	0	0	6	0	0	0	0	3
ENSMUSG00000100298	0	0	0	0	0	0	0	0	0	0
ENSMUSG00000100299	0	0	0	0	0	0	0	0	0	0
ENSMUSG00000104998	0	0	0	0	0	0	0	0	0	0
ENSMUSG00000101748	0	0	0	0	0	0	0	0	0	0
ENSMUSG00000101743	0	0	0	0	0	0	0	0	0	0
ENSMUSG00000101742	0	0	0	0	0	0	0	0	0	0
ENSMUSG00000101741	3	2	0	3	0	0	0	3	0	3

ENSMUSG00000101740	0	0	0	0	0	0	0	0	0	0
ENSMUSG00000101747	0	0	0	0	0	0	0	0	0	0
ENSMUSG00000104994	9	9	8	9	7	5	15	14	8	9
ENSMUSG00000101745	0	0	0	0	0	0	0	0	0	0
ENSMUSG00000101744	0	0	0	0	0	0	0	0	0	0
ENSMUSG00000037653	95	54	48	73	57	71	44	29	60	50
ENSMUSG00000093499	0	0	0	2	0	0	0	0	0	0
ENSMUSG00000066097	0	0	0	26	0	0	0	3	0	0
ENSMUSG00000033147	319	424	449	535	236	516	742	517	656	475
ENSMUSG00000008932	13	46	3	19	7	25	76	77	39	42
ENSMUSG00000096999	0	0	0	0	0	0	6	0	0	0
ENSMUSG00000088484	0	4	0	0	0	2	0	1	1	0
ENSMUSG00000088485	3	4	2	6	5	2	1	1	2	2
ENSMUSG00000088480	0	0	0	0	0	0	0	0	0	0
ENSMUSG00000088481	0	0	0	0	0	0	0	0	0	0
ENSMUSG00000088482	1	0	0	0	0	0	1	0	0	0
ENSMUSG00000088483	0	0	0	0	0	0	0	0	0	0
ENSMUSG00000059877	0	0	0	0	0	0	0	0	0	0
ENSMUSG00000059873	0	1	0	6	0	3	6	0	3	0
ENSMUSG00000103675	0	0	0	0	0	0	0	0	0	0
ENSMUSG00000036013	2	0	0	0	0	0	0	0	0	0
ENSMUSG00000103673	0	0	0	0	0	0	0	0	2	0
ENSMUSG00000103678	0	0	0	0	0	0	0	0	0	0
ENSMUSG00000089280	0	0	0	0	0	0	0	0	0	0
ENSMUSG00000089282	0	0	0	0	0	0	0	0	0	0
ENSMUSG00000089285	0	0	0	0	0	0	0	0	0	0
ENSMUSG00000107740	0	0	2	0	0	0	0	0	2	0
ENSMUSG00000089287	0	0	0	0	0	0	0	0	0	0
ENSMUSG00000089289	0	0	0	0	0	0	0	0	0	0
ENSMUSG00000089288	0	0	0	0	0	0	0	0	0	0
ENSMUSG00000107748	0	0	0	0	0	0	0	0	0	0
ENSMUSG00000065435	0	0	0	0	0	0	0	0	0	0
ENSMUSG00000094822	0	0	0	0	0	0	0	0	0	0
ENSMUSG00000065438	0	0	1	1	0	0	1	0	1	1
ENSMUSG00000021730	226	319	102	130	211	777	1279	502	199	197
ENSMUSG00000094009	0	0	0	1	0	0	0	0	0	0
ENSMUSG00000094008	0	0	0	0	0	0	0	0	0	0
ENSMUSG00000094005	0	0	1	0	0	1	1	1	1	1
ENSMUSG00000094004	0	0	0	0	0	0	0	0	0	0
ENSMUSG00000094007	0	0	0	0	0	0	2	0	0	0
ENSMUSG00000094001	0	0	0	0	0	0	0	0	0	0
ENSMUSG00000094002	0	0	0	3	0	0	0	0	0	0
ENSMUSG00000089759	21	9	25	13	15	64	40	20	56	17
ENSMUSG00000089753	17	25	32	32	20	18	61	17	30	23
ENSMUSG00000089754	0	0	0	0	0	0	0	0	0	0
ENSMUSG00000102104	0	0	0	0	0	0	0	0	0	0
ENSMUSG00000102105	0	0	0	0	0	0	0	0	0	0
ENSMUSG00000102106	0	0	0	0	0	0	0	0	0	0
ENSMUSG00000112425	1	0	0	0	0	0	6	0	0	7
ENSMUSG00000102100	0	0	0	0	0	0	0	0	0	0
ENSMUSG00000112420	0	2	6	0	0	0	9	3	3	3
ENSMUSG00000102103	0	0	0	0	0	0	0	0	0	0
ENSMUSG00000102108	0	0	0	0	0	0	0	0	0	0
ENSMUSG00000102109	0	0	0	0	0	0	0	0	0	0
ENSMUSG00000112428	0	0	0	0	0	0	0	0	0	0
ENSMUSG00000114860	3	6	10	0	0	0	11	0	4	0
ENSMUSG00000114861	0	0	0	0	0	0	0	2	0	0
ENSMUSG00000114862	0	0	0	0	0	0	0	0	0	0

ENSMUSG00000114864	0	0	0	3	3	0	0	0	0	0
ENSMUSG00000114865	7	29	3	15	8	7	15	18	9	7
ENSMUSG00000114866	0	0	0	0	0	0	0	1	0	0
ENSMUSG00000114867	0	0	0	0	0	0	0	0	0	0
ENSMUSG00000114868	0	0	0	0	0	0	0	0	0	0
ENSMUSG00000114869	8	3	11	6	3	8	19	21	17	7
ENSMUSG00000111552	0	0	0	0	0	0	0	0	0	0
ENSMUSG00000110370	0	0	0	0	6	0	0	0	0	0
ENSMUSG00000110371	9	15	30	20	18	12	14	5	14	15
ENSMUSG00000110375	0	0	0	0	0	0	0	0	0	0
ENSMUSG00000110377	20	20	6	8	12	11	21	19	23	18
ENSMUSG00000109028	3	0	3	0	3	6	6	0	11	6
ENSMUSG00000109029	22	27	24	22	32	4	0	6	8	7
ENSMUSG00000109022	0	0	0	0	0	0	0	0	0	0
ENSMUSG00000109023	0	0	0	0	0	0	0	0	0	0
ENSMUSG00000109020	0	0	0	0	0	0	0	0	0	0
ENSMUSG00000109021	0	0	0	3	0	8	3	9	0	3
ENSMUSG00000109026	0	0	0	0	0	0	0	0	0	0
ENSMUSG00000109027	0	0	0	0	0	0	0	0	0	0
ENSMUSG00000109024	0	0	0	0	0	0	0	0	0	0
ENSMUSG00000109025	0	0	0	0	0	0	0	0	0	0
ENSMUSG00000029054	0	9	0	3	5	20	6	29	5	9
ENSMUSG00000029055	0	3	3	0	0	14	3	9	12	3
ENSMUSG00000106909	0	0	0	0	0	0	0	0	0	0
ENSMUSG00000106900	0	0	0	0	0	0	0	0	0	0
ENSMUSG00000106905	0	0	0	0	0	0	0	0	0	3
ENSMUSG00000106906	0	0	0	0	0	0	0	0	0	0
ENSMUSG00000038115	3	6	6	2	3	9	6	3	6	6
ENSMUSG00000079479	0	0	0	0	0	0	0	0	0	0
ENSMUSG00000088903	0	0	0	0	0	0	0	0	0	0
ENSMUSG00000088905	0	0	0	0	0	0	0	0	0	0
ENSMUSG00000088904	0	0	0	0	0	0	0	0	0	0
ENSMUSG00000088907	0	0	0	1	0	0	0	0	0	0
ENSMUSG00000038112	25	45	33	29	30	128	201	78	145	40
ENSMUSG00000088909	0	0	0	1	0	0	0	0	0	0
ENSMUSG00000088908	0	0	0	0	0	0	0	0	0	0
ENSMUSG00000069227	26	3	15	15	13	32	34	30	9	23
ENSMUSG00000021600	0	0	0	3	0	0	3	0	0	0
ENSMUSG00000051687	0	0	0	0	0	0	0	0	0	0
ENSMUSG00000051680	0	0	0	0	0	3	0	6	0	0
ENSMUSG00000111429	0	0	0	3	0	0	6	6	0	0
ENSMUSG00000111428	0	0	1	0	1	0	0	1	0	1
ENSMUSG00000109686	0	0	0	0	0	0	0	0	0	0
ENSMUSG00000111426	0	0	0	0	0	0	0	0	0	0
ENSMUSG00000109682	0	0	0	0	0	0	0	0	0	0
ENSMUSG00000111422	0	0	0	0	0	0	0	0	0	7
ENSMUSG00000111421	19	27	3	28	12	14	8	20	19	18
ENSMUSG00000111420	0	0	0	0	0	0	0	0	0	0
ENSMUSG00000079471	0	6	6	0	0	0	0	0	0	0
ENSMUSG00000096337	17	12	10	15	17	19	25	17	13	17
ENSMUSG00000114287	0	0	0	0	0	0	0	0	0	0
ENSMUSG00000114280	0	0	0	0	3	0	2	0	2	0
ENSMUSG00000096330	7	5	1	7	0	0	5	9	5	0
ENSMUSG00000096332	0	0	0	0	0	3	0	0	0	3
ENSMUSG00000096339	0	0	0	0	0	0	0	0	0	0
ENSMUSG00000096338	0	0	0	0	0	0	0	0	0	0
ENSMUSG00000108379	0	0	0	0	0	0	2	0	0	0
ENSMUSG00000103149	49	47	72	69	62	36	79	78	66	54

ENSMUSG00000113489	0	0	0	0	0	0	0	0	0	0
ENSMUSG00000108370	0	0	2	2	0	0	0	0	0	0
ENSMUSG00000034324	36	42	30	8	45	18	71	51	21	26
ENSMUSG00000108372	3	3	6	0	1	0	4	6	7	6
ENSMUSG00000108375	0	0	3	0	0	0	0	0	0	0
ENSMUSG00000113482	0	0	0	0	0	0	0	0	0	0
ENSMUSG00000113481	0	0	0	0	0	0	0	0	0	0
ENSMUSG00000113480	0	0	0	5	0	0	0	0	0	3
ENSMUSG00000109978	0	0	0	0	0	0	0	0	0	0
ENSMUSG00000109979	0	0	0	0	0	0	0	0	0	0
ENSMUSG00000093868	0	0	0	0	0	0	0	0	0	0
ENSMUSG00000093869	0	0	0	0	0	0	0	0	0	0
ENSMUSG00000109971	0	0	0	0	0	0	0	0	0	0
ENSMUSG00000109972	0	0	0	0	0	0	0	0	0	0
ENSMUSG00000026556	70	113	98	78	89	103	152	158	111	182
ENSMUSG00000093867	3	0	6	0	0	0	0	0	3	0
ENSMUSG00000093865	5	6	0	6	9	4	9	6	9	0
ENSMUSG00000114134	0	0	0	0	0	0	0	0	0	0
ENSMUSG00000114132	0	0	0	0	0	0	0	0	0	0
ENSMUSG00000114130	0	0	0	0	0	0	0	0	0	0
ENSMUSG00000066477	0	0	0	0	0	0	0	0	0	0
ENSMUSG00000104502	0	0	0	0	0	0	0	0	0	3
ENSMUSG00000108082	0	0	0	0	0	0	0	0	0	0
ENSMUSG00000104500	0	0	0	0	0	0	0	0	0	0
ENSMUSG00000047889	0	0	0	0	0	0	0	0	0	0
ENSMUSG00000108086	0	0	0	0	0	0	0	0	0	0
ENSMUSG00000108085	0	0	0	0	0	0	0	0	0	0
ENSMUSG00000108084	0	0	5	0	0	3	24	3	3	0
ENSMUSG00000108089	2	2	2	3	1	0	2	1	0	0
ENSMUSG00000108088	0	0	0	0	0	0	0	0	0	0
ENSMUSG00000091648	15	21	8	2	14	11	22	16	9	17
ENSMUSG00000069825	0	3	0	0	0	0	3	0	0	0
ENSMUSG00000067704	0	0	0	0	0	0	0	0	0	0
ENSMUSG00000067700	0	0	0	0	0	0	4	6	0	0
ENSMUSG00000067702	0	0	3	0	0	0	0	0	5	0
ENSMUSG00000061356	0	3	0	12	0	0	22	7	3	12
ENSMUSG00000044286	0	0	3	0	0	0	0	0	0	0
ENSMUSG00000044288	27	33	9	3	18	30	81	24	36	20
ENSMUSG00000096102	0	0	0	0	0	0	0	0	0	0
ENSMUSG00000111174	0	0	0	0	0	0	0	0	0	0
ENSMUSG00000112999	3	4	0	0	2	3	0	0	0	0
ENSMUSG00000112994	0	0	0	0	0	0	0	0	0	0
ENSMUSG00000112992	0	0	0	0	0	0	0	0	0	0
ENSMUSG00000112993	0	0	0	0	0	0	0	0	0	0
ENSMUSG00000112991	12	10	34	22	8	12	20	7	5	19
ENSMUSG00000109808	0	0	0	0	0	0	0	0	0	0
ENSMUSG00000109218	3	2	2	3	0	3	5	3	0	0
ENSMUSG00000097948	1	3	0	3	0	0	3	6	3	1
ENSMUSG00000048904	0	0	0	0	0	0	0	0	0	0
ENSMUSG00000045620	0	0	0	5	3	0	11	0	2	0
ENSMUSG00000098059	0	0	0	0	0	0	0	0	0	0
ENSMUSG00000090063	6	0	12	3	12	9	69	0	24	9
ENSMUSG00000090062	0	0	0	0	0	9	2	0	0	2
ENSMUSG00000090061	0	0	0	3	3	0	6	6	3	0
ENSMUSG00000095011	0	0	0	0	0	0	0	0	0	0
ENSMUSG00000095010	0	0	0	0	0	0	0	0	0	0
ENSMUSG00000095019	0	0	0	0	0	0	0	0	0	0
ENSMUSG00000098275	15	3	0	9	20	27	24	12	12	7

ENSMUSG00000098272	0	0	0	0	0	0	0	0	0	0
ENSMUSG00000098273	0	0	1	0	0	0	1	2	1	1
ENSMUSG00000098271	0	1	0	1	0	0	2	2	0	4
ENSMUSG00000067949	0	0	3	3	0	0	0	3	0	0
ENSMUSG00000096105	0	0	0	0	0	0	0	0	0	0
ENSMUSG00000096741	0	0	0	0	0	0	0	0	0	0
ENSMUSG00000047716	0	3	0	0	0	0	0	0	0	0
ENSMUSG00000053790	0	0	0	0	0	0	0	0	0	0
ENSMUSG00000094763	0	0	0	0	0	0	0	0	0	0
ENSMUSG00000094768	0	0	0	0	0	0	0	0	0	0
ENSMUSG00000111045	3	0	1	0	0	0	0	0	0	3
ENSMUSG00000101030	0	0	0	0	0	0	0	0	0	0
ENSMUSG00000052221	10	5	4	7	3	19	12	3	0	9
ENSMUSG00000093089	1	0	0	0	0	0	0	1	0	0
ENSMUSG00000093085	3	6	1	0	1	1	2	1	1	2
ENSMUSG00000055026	22	21	30	18	9	24	18	40	6	18
ENSMUSG00000093082	0	0	0	0	0	0	0	0	0	0
ENSMUSG00000093083	0	0	0	1	0	0	0	1	0	1
ENSMUSG00000026890	111	224	250	244	251	210	288	267	511	273
ENSMUSG00000090685	0	6	6	3	4	6	6	0	9	0
ENSMUSG00000090684	0	0	0	0	0	1	0	0	0	0
ENSMUSG00000112675	0	0	0	0	0	0	0	0	0	0
ENSMUSG00000081508	0	0	0	0	0	0	0	0	0	0
ENSMUSG00000081506	0	0	0	0	0	0	0	0	0	0
ENSMUSG00000081507	0	0	0	0	0	0	0	0	0	0
ENSMUSG00000081505	0	0	0	0	0	0	0	0	0	0
ENSMUSG00000081502	0	0	0	0	0	0	0	0	0	0
ENSMUSG00000081500	0	0	0	0	0	0	0	0	0	0
ENSMUSG00000090688	0	2	0	0	0	0	0	0	0	3
ENSMUSG00000077468	0	0	0	0	0	0	0	0	0	0
ENSMUSG00000092685	0	0	0	0	0	0	0	0	0	0
ENSMUSG00000099659	0	0	0	0	0	0	0	0	0	0
ENSMUSG00000099657	0	0	0	0	0	0	0	0	0	0
ENSMUSG00000099656	0	0	0	0	0	0	0	0	0	0
ENSMUSG00000077462	0	0	0	0	0	0	0	0	0	0
ENSMUSG00000099654	0	0	0	0	0	0	0	0	0	0
ENSMUSG00000077464	0	0	0	0	0	0	0	0	0	0
ENSMUSG00000099651	0	0	0	0	0	0	0	0	0	0
ENSMUSG00000099650	0	0	0	0	0	0	0	0	0	0
ENSMUSG00000101449	0	0	0	0	0	0	0	0	0	0
ENSMUSG00000049843	0	0	0	0	0	0	0	0	0	0
ENSMUSG00000049848	6	3	3	3	0	6	6	0	9	3
ENSMUSG00000048138	8	11	6	0	6	0	21	0	27	29
ENSMUSG00000086612	0	0	3	0	0	0	1	2	0	3
ENSMUSG00000030145	234	241	234	200	119	219	434	294	326	301
ENSMUSG00000030142	6	13	26	160	33	142	65	45	68	60
ENSMUSG00000030143	0	0	0	0	0	0	0	0	0	0
ENSMUSG00000030149	122	69	62	172	27	108	282	157	207	130
ENSMUSG00000092359	1	0	0	0	0	0	0	0	0	0
ENSMUSG00000092353	0	3	11	7	4	6	6	13	13	12
ENSMUSG00000092352	0	0	0	0	0	0	0	0	0	0
ENSMUSG00000092351	0	0	0	0	0	0	0	0	0	0
ENSMUSG00000092350	0	0	0	0	0	0	0	0	0	0
ENSMUSG00000092357	0	0	0	0	0	0	0	0	0	0
ENSMUSG00000087137	0	0	0	0	0	0	0	0	0	0
ENSMUSG00000051940	10	8	16	0	19	0	18	18	13	13
ENSMUSG00000096742	0	0	0	0	0	0	0	0	0	0
ENSMUSG00000043715	0	0	0	0	0	0	0	0	0	0

ENSMUSG00000082088	17	21	37	45	26	21	33	40	28	21
ENSMUSG00000082089	0	0	0	0	0	0	0	0	0	0
ENSMUSG00000082082	0	0	0	0	0	0	0	0	0	0
ENSMUSG00000082083	0	0	0	0	0	0	0	0	0	0
ENSMUSG00000082080	0	0	0	0	0	0	0	0	0	0
ENSMUSG00000082081	0	0	0	0	0	0	0	0	0	0
ENSMUSG00000082084	0	0	0	0	0	0	0	0	0	0
ENSMUSG00000082085	0	0	0	0	0	0	0	0	0	0
ENSMUSG00000076337	0	0	0	0	0	0	0	0	0	0
ENSMUSG00000076335	0	0	0	0	0	1	0	0	0	1
ENSMUSG00000041710	0	0	0	0	0	0	3	0	0	0
ENSMUSG00000076338	0	0	0	0	0	1	0	1	1	0
ENSMUSG00000093846	0	4	11	0	3	0	14	5	7	18
ENSMUSG00000032783	17	5	15	27	29	14	45	33	21	23
ENSMUSG00000037492	22	17	33	44	20	28	6	31	23	40
ENSMUSG00000113605	0	0	0	0	0	0	3	0	0	0
ENSMUSG00000093847	0	2	0	0	0	0	0	0	0	0
ENSMUSG00000072653	111	79	108	94	55	161	269	175	259	165
ENSMUSG00000087618	0	0	0	0	0	0	0	0	0	0
ENSMUSG00000087617	0	0	0	0	0	0	0	0	3	0
ENSMUSG00000087613	0	0	0	0	0	0	0	0	0	0
ENSMUSG00000075023	11	0	5	6	0	0	6	0	3	6
ENSMUSG00000075020	22	30	29	30	24	25	21	32	26	23
ENSMUSG00000097682	0	0	0	0	0	0	0	0	0	0
ENSMUSG00000075024	0	0	0	0	0	0	0	0	0	0
ENSMUSG00000110330	0	0	0	0	0	0	0	0	0	0
ENSMUSG00000107503	0	0	0	0	0	0	0	0	0	0
ENSMUSG00000107501	0	0	0	0	0	2	0	1	0	0
ENSMUSG00000107500	0	0	0	0	0	0	0	0	0	0
ENSMUSG00000107506	0	0	0	0	0	0	0	0	0	0
ENSMUSG00000101839	0	0	0	0	0	0	0	0	0	0
ENSMUSG00000101835	0	0	0	0	0	0	0	0	0	0
ENSMUSG00000101837	0	0	0	0	0	0	0	0	0	0
ENSMUSG00000101836	0	0	0	0	0	0	0	0	0	0
ENSMUSG00000101831	0	0	0	0	0	0	0	0	0	0
ENSMUSG00000068289	0	0	0	0	0	0	0	3	0	0
ENSMUSG00000101833	0	0	0	0	0	0	0	0	0	0
ENSMUSG00000085616	0	0	0	0	0	0	0	0	0	0
ENSMUSG00000074378	0	0	0	0	0	0	0	0	0	0
ENSMUSG00000085610	0	0	0	0	0	0	0	0	0	0
ENSMUSG00000074373	0	0	0	0	0	0	0	0	0	0
ENSMUSG00000074375	0	0	0	0	0	0	0	0	0	0
ENSMUSG00000074377	0	0	0	0	0	0	0	0	0	0
ENSMUSG00000085619	0	0	0	0	0	0	0	0	0	0
ENSMUSG00000032598	411	495	581	539	494	566	536	496	517	492
ENSMUSG00000090727	0	0	0	0	0	0	0	0	0	0
ENSMUSG00000075973	0	0	0	0	0	0	0	0	0	0
ENSMUSG00000081976	0	0	0	0	0	0	2	0	0	0
ENSMUSG00000081973	0	0	0	0	0	0	0	0	0	0
ENSMUSG00000081972	3	4	8	8	2	3	6	5	9	6
ENSMUSG00000081971	0	0	0	0	0	0	0	0	0	0
ENSMUSG00000078503	6	0	0	0	9	6	9	3	15	6
ENSMUSG00000024409	0	0	0	0	0	0	0	0	0	0
ENSMUSG00000078506	0	0	0	0	0	0	0	0	0	0
ENSMUSG00000078507	0	0	0	0	0	0	0	0	0	0
ENSMUSG00000078504	0	0	0	0	0	0	0	0	0	0
ENSMUSG00000078505	0	0	0	0	0	0	0	0	0	0
ENSMUSG00000024402	0	2	0	6	3	0	6	12	15	6

ENSMUSG00000078508	0	0	0	0	0	0	0	0	0	0
ENSMUSG00000024401	37	42	100	134	52	62	104	62	121	64
ENSMUSG00000024406	0	0	0	0	2	0	0	0	0	0
ENSMUSG00000089618	0	0	0	0	0	0	0	0	0	0
ENSMUSG00000089619	0	0	0	0	0	0	0	0	0	0
ENSMUSG00000089610	0	0	0	0	0	0	0	0	0	0
ENSMUSG00000089616	0	0	0	0	0	0	0	0	0	0
ENSMUSG00000089614	0	0	0	0	0	0	0	0	0	0
ENSMUSG00000089615	0	0	0	0	0	0	0	0	0	0
ENSMUSG00000084907	0	0	0	0	0	0	0	0	0	0
ENSMUSG00000084906	0	0	0	0	0	0	0	0	0	0
ENSMUSG00000032204	3	6	12	46	6	20	20	15	17	9
ENSMUSG00000102023	0	0	0	0	0	0	0	0	0	0
ENSMUSG00000102022	0	0	0	0	0	0	0	0	0	0
ENSMUSG00000102020	0	0	0	0	0	0	0	0	0	0
ENSMUSG00000102027	0	0	0	0	0	0	9	0	0	0
ENSMUSG00000102026	0	0	0	0	0	0	0	0	0	0
ENSMUSG00000102025	0	0	0	0	0	0	0	0	0	0
ENSMUSG00000102028	0	0	0	0	0	0	0	0	0	0
ENSMUSG00000062391	30	37	30	35	26	24	9	29	30	60
ENSMUSG00000062393	0	0	0	0	0	0	3	6	6	0
ENSMUSG00000103998	0	0	0	31	31	0	43	34	0	0
ENSMUSG00000103990	0	0	0	0	0	0	0	0	0	0
ENSMUSG00000103991	10	12	15	0	5	6	0	18	0	10
ENSMUSG00000103992	0	0	0	0	0	0	0	0	0	0
ENSMUSG00000103993	0	0	0	0	0	0	0	0	0	0
ENSMUSG00000103994	0	0	0	0	0	0	0	0	0	0
ENSMUSG00000103995	0	0	0	2	0	6	5	6	9	1
ENSMUSG00000103997	0	0	0	0	0	0	0	0	0	0
ENSMUSG00000073532	4	2	6	2	3	3	11	4	5	5
ENSMUSG00000083654	0	0	0	0	0	0	0	0	0	0
ENSMUSG00000073530	100	131	115	91	150	202	396	119	84	69
ENSMUSG00000110290	0	0	0	0	0	0	0	0	0	0
ENSMUSG00000077857	0	0	0	0	0	0	0	0	0	0
ENSMUSG00000077858	0	0	0	0	0	0	0	0	0	0
ENSMUSG00000084600	0	0	0	0	0	0	0	0	0	0
ENSMUSG00000069421	0	0	0	0	0	0	0	0	1	0
ENSMUSG00000083657	2	0	0	0	0	0	0	0	0	0
ENSMUSG00000042216	60	60	61	60	34	100	110	62	84	73
ENSMUSG00000089275	0	0	0	0	0	0	0	0	0	0
ENSMUSG00000094402	0	0	0	0	0	0	0	0	0	0
ENSMUSG00000057402	0	0	0	0	0	0	0	0	0	0
ENSMUSG00000084605	0	0	0	0	0	0	0	0	0	0
ENSMUSG00000042212	0	0	0	0	0	0	0	0	0	0
ENSMUSG00000096133	0	0	0	0	0	0	0	0	0	0
ENSMUSG00000096131	0	0	0	0	0	0	0	0	0	0
ENSMUSG00000025165	18	0	3	38	35	0	11	0	16	3
ENSMUSG00000096137	0	0	0	0	0	0	0	0	0	0
ENSMUSG00000096136	0	0	0	0	0	0	0	0	0	0
ENSMUSG00000096135	0	0	0	0	0	0	0	0	0	0
ENSMUSG00000096134	0	0	0	0	0	0	0	0	0	0
ENSMUSG00000096139	0	0	0	0	0	0	0	0	0	0
ENSMUSG00000094406	0	0	0	0	0	0	0	0	0	0
ENSMUSG00000078430	0	0	0	0	0	0	0	0	0	0
ENSMUSG00000034122	0	0	0	0	0	0	0	0	0	0
ENSMUSG00000032080	3	0	0	0	0	0	0	0	0	0
ENSMUSG00000103236	0	0	0	0	0	0	0	0	0	0
ENSMUSG00000024532	0	0	0	0	0	0	0	0	0	0

ENSMUSG00000023999	221	260	205	214	181	175	154	202	151	194
ENSMUSG00000096688	6	0	14	0	0	0	6	0	15	0
ENSMUSG00000096685	0	0	0	0	0	0	0	0	0	0
ENSMUSG00000096686	0	0	0	0	0	0	0	0	0	0
ENSMUSG00000096680	0	0	0	0	0	0	0	0	0	0
ENSMUSG00000096683	0	0	0	0	0	0	1	0	0	0
ENSMUSG00000096682	0	3	0	0	0	0	0	0	0	0
ENSMUSG00000066671	0	0	0	0	0	0	0	0	0	0
ENSMUSG00000041698	9	0	3	11	0	0	3	0	0	0
ENSMUSG00000024534	252	288	311	326	214	289	569	383	261	392
ENSMUSG00000071073	5	9	28	13	12	9	34	36	49	20
ENSMUSG00000091483	0	0	0	0	0	0	0	0	0	0
ENSMUSG00000091481	0	0	0	0	0	0	0	0	0	0
ENSMUSG00000091486	0	0	0	0	0	0	0	0	0	0
ENSMUSG00000020875	0	0	0	0	15	0	0	0	0	0
ENSMUSG00000020872	23	31	9	7	10	19	32	18	28	13
ENSMUSG00000020871	6	0	0	0	0	0	6	6	11	3
ENSMUSG00000105844	0	0	0	0	0	0	0	0	0	0
ENSMUSG00000104072	0	0	0	0	0	0	0	0	0	0
ENSMUSG00000104073	0	0	0	0	0	0	0	0	0	0
ENSMUSG00000104070	0	0	0	0	0	0	0	0	0	0
ENSMUSG00000104071	0	0	0	0	0	0	0	0	0	0
ENSMUSG00000104076	0	0	0	0	0	0	3	0	0	0
ENSMUSG00000060630	0	0	0	0	0	0	0	0	0	0
ENSMUSG00000104074	6	3	3	0	8	0	5	2	6	2
ENSMUSG00000104075	0	0	0	0	0	0	0	0	0	0
ENSMUSG00000099873	0	0	0	0	0	0	0	0	0	0
ENSMUSG00000015476	44	46	60	25	52	37	78	67	106	50
ENSMUSG00000048077	0	0	0	0	0	0	0	0	0	0
ENSMUSG00000056328	6	7	0	25	0	18	134	41	83	18
ENSMUSG00000056329	0	0	0	0	0	12	0	2	0	0
ENSMUSG00000100318	0	0	0	0	0	0	0	0	0	0
ENSMUSG00000114634	3	0	0	0	0	0	0	0	0	0
ENSMUSG00000100312	0	0	0	0	0	0	0	0	0	0
ENSMUSG00000100311	0	0	0	0	0	0	0	0	0	0
ENSMUSG00000061947	0	0	0	0	0	0	0	0	3	0
ENSMUSG00000100317	0	0	0	0	0	0	0	0	0	0
ENSMUSG00000100316	0	0	0	0	0	0	0	0	0	0
ENSMUSG00000100315	0	0	3	0	0	0	0	0	0	0
ENSMUSG00000100314	0	0	0	0	0	0	0	0	0	0
ENSMUSG00000110929	0	0	0	0	0	0	0	0	0	0
ENSMUSG00000102400	0	0	0	0	0	0	0	0	0	0
ENSMUSG00000110925	0	0	0	0	0	0	0	0	0	0
ENSMUSG00000110924	0	0	0	0	0	0	0	0	0	0
ENSMUSG00000110927	0	0	0	0	0	0	0	0	0	0
ENSMUSG00000110920	0	0	0	0	0	0	0	0	0	0
ENSMUSG00000110923	0	0	0	0	0	0	0	0	0	0
ENSMUSG00000110922	56	63	61	73	26	45	13	43	52	83
ENSMUSG00000102402	0	0	0	0	0	0	0	0	0	0
ENSMUSG00000114632	0	0	0	0	0	0	0	0	0	0
ENSMUSG00000102404	0	0	0	0	0	0	0	0	0	0
ENSMUSG00000112260	0	0	0	0	0	0	0	0	0	0
ENSMUSG00000026826	135	130	143	152	108	154	297	203	275	183
ENSMUSG00000026828	6	9	3	18	0	13	3	17	21	24
ENSMUSG00000088361	0	0	0	0	0	0	0	0	0	1
ENSMUSG00000088360	0	0	0	0	0	0	0	0	0	0
ENSMUSG00000088363	1	1	0	0	1	1	1	1	0	0
ENSMUSG00000088365	0	2	0	0	0	0	0	0	0	0

ENSMUSG00000088367	0	0	0	0	0	0	0	0	1	0
ENSMUSG00000088369	0	0	0	0	0	0	0	0	0	0
ENSMUSG00000077038	1	0	2	0	0	0	2	0	0	0
ENSMUSG00000036198	3	9	12	12	15	6	5	9	3	0
ENSMUSG00000095569	0	0	0	0	0	0	0	0	0	0
ENSMUSG00000036192	227	168	238	162	166	346	402	313	386	379
ENSMUSG00000095561	0	0	0	0	0	0	0	0	0	0
ENSMUSG00000101895	0	0	0	0	0	0	6	14	0	3
ENSMUSG00000095563	0	0	0	0	0	0	0	0	0	0
ENSMUSG00000112025	0	0	0	0	0	1	0	6	0	0
ENSMUSG00000112389	0	0	0	0	0	0	0	0	0	0
ENSMUSG00000094218	0	0	0	0	0	0	0	0	0	0
ENSMUSG00000094219	0	0	0	0	0	0	0	0	0	0
ENSMUSG00000027889	450	490	464	368	302	421	797	539	598	542
ENSMUSG00000094214	1	0	0	0	0	0	0	0	0	0
ENSMUSG00000027886	0	0	2	0	0	0	0	0	0	0
ENSMUSG00000093240	0	0	0	0	0	0	0	0	0	0
ENSMUSG00000112380	0	0	0	0	0	0	0	0	0	0
ENSMUSG00000112387	0	0	0	0	0	0	0	0	0	0
ENSMUSG00000112386	0	0	3	7	0	3	0	0	3	9
ENSMUSG00000112385	0	0	0	0	0	0	0	0	0	0
ENSMUSG00000027880	0	0	0	0	0	0	3	0	0	0
ENSMUSG00000112029	0	0	0	0	0	0	2	0	0	0
ENSMUSG00000036223	45	3	35	42	26	12	54	24	9	5
ENSMUSG00000113382	0	0	0	0	0	0	0	0	0	0
ENSMUSG00000113383	0	0	0	0	0	0	8	4	0	0
ENSMUSG00000113384	0	0	0	0	0	0	0	0	0	0
ENSMUSG00000028681	85	70	88	76	45	97	144	113	204	127
ENSMUSG00000113387	0	0	0	0	0	0	0	3	0	0
ENSMUSG00000113388	0	0	0	0	0	0	1	0	2	0
ENSMUSG00000108149	0	0	0	0	0	0	0	0	0	0
ENSMUSG00000102377	0	0	0	0	0	0	0	0	0	0
ENSMUSG00000018919	11	25	16	0	16	27	35	13	10	21
ENSMUSG00000087956	0	0	0	0	0	0	0	0	0	0
ENSMUSG00000017723	5	24	27	11	18	14	30	17	13	15
ENSMUSG00000018916	0	0	15	3	3	0	8	0	7	3
ENSMUSG00000018914	0	0	0	0	0	0	0	0	0	0
ENSMUSG00000020051	110	54	233	118	18	48	15	36	44	93
ENSMUSG00000106008	0	0	0	0	0	0	0	0	0	0
ENSMUSG00000009471	0	0	6	0	0	3	0	0	0	9
ENSMUSG00000106001	0	0	0	0	0	0	0	0	0	0
ENSMUSG00000106000	0	3	0	0	0	0	0	0	3	3
ENSMUSG00000106003	0	0	0	0	0	0	0	0	0	0
ENSMUSG00000106002	0	0	0	0	0	0	0	0	0	0
ENSMUSG00000106005	9	27	2	21	8	5	12	6	3	6
ENSMUSG00000106004	0	0	0	0	0	0	0	0	0	0
ENSMUSG00000106007	0	0	0	0	0	0	0	0	0	0
ENSMUSG00000028539	0	27	8	3	3	13	19	5	6	18
ENSMUSG00000028536	3	0	0	18	4	0	0	3	6	0
ENSMUSG00000028533	0	0	0	0	0	0	0	0	0	0
ENSMUSG00000092116	8	8	0	0	6	3	12	5	0	0
ENSMUSG00000039476	23	58	22	13	26	47	181	62	118	55
ENSMUSG00000112653	0	0	0	0	0	0	0	0	0	0
ENSMUSG00000112659	0	0	0	0	0	0	0	4	0	0
ENSMUSG00000112658	0	0	0	0	0	0	0	0	0	0
ENSMUSG00000025105	70	175	193	90	115	166	528	267	222	156
ENSMUSG00000113524	0	0	3	0	0	0	0	0	0	0
ENSMUSG00000113526	0	0	0	0	0	0	0	0	0	0

ENSMUSG00000113527	9	9	10	20	5	33	16	5	17	24
ENSMUSG00000038700	20	7	6	6	14	3	23	0	15	12
ENSMUSG00000113522	0	0	0	0	0	0	0	0	0	0
ENSMUSG00000113523	0	0	0	0	0	0	0	0	0	0
ENSMUSG00000038709	0	0	0	0	0	0	0	0	0	0
ENSMUSG00000085559	0	0	0	0	0	0	0	0	0	0
ENSMUSG00000064551	0	0	0	0	0	0	0	0	0	0
ENSMUSG00000082868	0	0	0	0	0	0	0	0	0	0
ENSMUSG00000082869	0	0	0	0	0	0	0	0	0	0
ENSMUSG00000082864	0	0	0	0	0	0	0	0	0	0
ENSMUSG00000082865	3	0	0	0	0	0	0	0	0	0
ENSMUSG00000082861	2	2	4	3	3	0	7	1	2	0
ENSMUSG00000082862	0	0	0	0	0	0	0	0	0	0
ENSMUSG00000082863	0	0	0	0	0	0	0	0	0	0
ENSMUSG00000108128	0	0	0	0	0	0	0	0	0	0
ENSMUSG00000108129	0	3	0	0	0	0	0	5	3	4
ENSMUSG00000086146	0	0	4	1	0	3	0	0	3	0
ENSMUSG00000070417	0	0	0	0	0	0	0	0	0	0
ENSMUSG00000070419	0	0	0	0	0	0	0	0	0	0
ENSMUSG00000108127	0	0	0	0	0	0	0	0	0	0
ENSMUSG00000105067	0	0	0	0	0	0	0	0	0	0
ENSMUSG00000105064	0	0	0	0	0	0	0	0	0	0
ENSMUSG00000105065	13	15	6	5	0	9	18	3	5	13
ENSMUSG00000109133	0	0	0	0	0	0	0	0	0	0
ENSMUSG00000111678	0	0	0	0	0	0	0	0	0	0
ENSMUSG00000026458	265	245	314	417	169	245	495	315	370	249
ENSMUSG00000111674	0	1	0	0	0	0	1	3	0	0
ENSMUSG00000111675	0	0	0	0	0	0	0	0	0	0
ENSMUSG00000111672	0	0	0	0	0	0	0	0	0	0
ENSMUSG00000088136	0	0	0	0	0	0	0	0	0	0
ENSMUSG00000111670	0	0	0	0	0	0	0	0	0	0
ENSMUSG00000026450	3	3	0	0	0	0	3	0	0	0
ENSMUSG00000079998	0	0	0	0	0	0	0	0	0	0
ENSMUSG00000036960	5	6	17	25	21	8	61	38	32	34
ENSMUSG00000088132	3	0	0	0	0	0	0	0	0	2
ENSMUSG00000104331	0	0	0	0	0	2	0	3	1	1
ENSMUSG00000104330	25	42	56	27	18	29	30	22	46	24
ENSMUSG00000104333	6	3	6	2	5	0	2	5	0	6
ENSMUSG00000104335	0	0	0	0	0	0	0	0	0	0
ENSMUSG00000104334	0	0	0	0	0	0	0	0	0	0
ENSMUSG00000104337	0	0	0	0	0	0	0	0	0	0
ENSMUSG00000104338	0	0	0	0	0	0	0	0	0	0
ENSMUSG00000036961	15	19	12	9	8	3	0	15	3	5
ENSMUSG00000079774	0	0	0	0	0	0	0	18	17	0
ENSMUSG00000083803	0	0	0	0	0	0	0	0	3	0
ENSMUSG00000045291	0	0	0	0	0	0	0	0	0	0
ENSMUSG00000083809	0	0	0	0	0	0	0	0	0	0
ENSMUSG00000044748	0	0	3	9	0	3	3	0	3	3
ENSMUSG00000044743	0	0	0	0	0	0	0	0	0	0
ENSMUSG00000098347	0	0	0	0	0	0	0	0	0	0
ENSMUSG00000113844	0	0	0	0	0	0	0	0	0	0
ENSMUSG00000098343	0	0	0	0	0	0	0	0	0	0
ENSMUSG00000090180	0	0	0	0	0	0	0	0	0	0
ENSMUSG00000027419	9	3	6	0	9	6	12	0	7	3
ENSMUSG00000092961	0	1	0	0	0	0	0	0	0	0
ENSMUSG00000092965	0	0	0	0	0	0	0	0	0	0
ENSMUSG00000027416	0	0	0	0	0	0	21	0	12	0
ENSMUSG00000113849	0	0	0	0	0	0	0	0	0	0

ENSMUSG00000100310	0	0	0	0	0	0	0	0	0	0
ENSMUSG00000088010	0	0	0	0	0	0	0	0	0	1
ENSMUSG00000046678	0	0	0	0	0	0	0	0	0	0
ENSMUSG00000095604	1	0	0	1	0	0	0	0	0	0
ENSMUSG00000095605	0	0	1	0	0	1	1	1	1	0
ENSMUSG00000095606	0	0	0	0	0	0	0	0	0	0
ENSMUSG00000095600	0	0	0	0	0	0	0	0	0	0
ENSMUSG00000095601	0	2	0	0	0	0	0	0	0	0
ENSMUSG00000095602	0	0	0	0	0	0	0	0	0	0
ENSMUSG00000090235	3	25	17	2	14	16	25	6	16	14
ENSMUSG00000095608	0	0	0	0	0	0	0	0	0	0
ENSMUSG00000088017	0	0	0	0	0	0	0	0	0	0
ENSMUSG00000080577	0	0	0	0	0	0	0	0	0	0
ENSMUSG00000113371	0	0	0	0	0	0	0	0	0	0
ENSMUSG00000092493	0	0	0	0	0	0	0	0	0	0
ENSMUSG00000092494	3	10	0	0	0	0	3	0	12	3
ENSMUSG00000052075	0	0	0	0	0	0	0	0	0	0
ENSMUSG00000098405	0	0	0	0	0	0	0	0	0	0
ENSMUSG00000028946	6	6	5	6	11	0	0	0	6	5
ENSMUSG00000098407	0	0	0	0	0	0	0	0	0	0
ENSMUSG00000098406	2	0	0	0	0	0	2	0	1	0
ENSMUSG00000098401	0	0	0	0	0	2	3	2	0	2
ENSMUSG00000098400	0	0	0	0	0	0	0	0	0	0
ENSMUSG00000028940	6	0	0	0	3	0	3	9	0	6
ENSMUSG00000098409	0	0	0	0	0	0	0	0	0	0
ENSMUSG00000113378	0	0	0	0	0	0	0	0	0	0
ENSMUSG00000093761	0	0	0	0	0	0	0	0	0	0
ENSMUSG00000093763	0	0	0	0	0	0	0	0	0	0
ENSMUSG00000093762	0	0	0	0	0	0	1	0	0	0
ENSMUSG00000093765	0	0	4	0	3	5	3	3	9	6
ENSMUSG00000093764	0	0	0	0	0	0	0	0	0	0
ENSMUSG00000093766	0	0	0	0	0	0	0	0	0	0
ENSMUSG00000110381	0	0	0	0	0	0	0	0	0	0
ENSMUSG00000047104	0	0	3	0	0	2	30	0	13	0
ENSMUSG00000031424	0	0	0	0	0	0	0	0	0	0
ENSMUSG00000047109	0	0	0	0	0	0	0	0	0	0
ENSMUSG00000031428	17	29	12	8	21	44	59	29	57	25
ENSMUSG00000037868	120	128	182	98	91	186	436	167	233	152
ENSMUSG00000049653	0	5	3	5	6	0	11	8	3	7
ENSMUSG00000112355	0	0	0	0	0	0	0	0	0	0
ENSMUSG00000105550	0	9	0	6	15	4	19	9	9	0
ENSMUSG00000112011	0	0	0	0	0	0	0	0	0	0
ENSMUSG00000095699	0	0	0	0	0	0	0	0	0	1
ENSMUSG00000095698	0	0	0	0	0	0	0	0	0	0
ENSMUSG00000101202	0	0	0	0	0	0	0	0	0	0
ENSMUSG00000101203	0	0	0	0	0	0	0	0	0	0
ENSMUSG00000101200	0	0	0	0	0	0	0	0	0	0
ENSMUSG00000110804	0	0	0	0	0	0	0	0	0	0
ENSMUSG00000101206	0	0	0	0	0	0	0	0	0	0
ENSMUSG00000101207	0	0	0	0	0	0	0	0	0	0
ENSMUSG00000101204	0	0	0	0	0	0	0	0	0	0
ENSMUSG00000095693	0	0	0	0	0	0	0	0	0	0
ENSMUSG00000087139	0	0	0	0	0	0	0	0	0	0
ENSMUSG00000082325	0	0	0	0	0	0	0	0	0	0
ENSMUSG00000082323	0	0	0	0	0	0	0	0	0	0
ENSMUSG00000048304	5	0	0	6	6	8	0	0	6	5
ENSMUSG00000099446	0	0	0	0	0	0	0	0	0	0
ENSMUSG00000099444	0	0	0	0	0	0	0	0	0	0

ENSMUSG00000099445	0	0	0	0	0	0	0	0	0	0
ENSMUSG00000099443	0	2	1	1	1	1	1	2	0	0
ENSMUSG00000050520	0	0	0	0	0	0	3	0	0	0
ENSMUSG00000037280	100	107	94	146	73	94	205	131	81	88
ENSMUSG00000084449	0	0	0	0	0	0	0	0	0	0
ENSMUSG00000083499	0	0	0	0	0	0	0	0	0	0
ENSMUSG00000084444	0	0	0	0	0	0	0	0	0	0
ENSMUSG00000084445	0	0	0	0	0	0	0	0	0	0
ENSMUSG00000084446	0	0	0	0	0	0	0	0	2	0
ENSMUSG00000083495	0	0	0	2	0	0	0	0	0	0
ENSMUSG00000083492	0	0	0	0	0	0	0	0	0	0
ENSMUSG00000083493	0	0	0	0	0	0	0	0	0	0
ENSMUSG00000084442	0	0	0	0	0	0	0	0	0	0
ENSMUSG00000083491	0	0	0	0	0	0	0	0	0	0
ENSMUSG00000100159	0	0	0	0	1	0	0	0	1	0
ENSMUSG00000110490	0	0	0	5	0	3	6	0	3	4
ENSMUSG00000100151	0	0	0	0	0	0	0	0	0	0
ENSMUSG00000030486	155	221	199	270	166	244	339	275	384	270
ENSMUSG00000101762	0	0	0	0	0	0	0	0	0	0
ENSMUSG00000030483	151	47	167	132	85	113	71	72	196	157
ENSMUSG00000100157	0	0	0	0	3	0	0	3	3	5
ENSMUSG00000034660	0	0	0	0	0	0	0	0	0	0
ENSMUSG00000037139	131	162	254	171	179	282	337	293	418	251
ENSMUSG00000083140	0	0	0	0	0	0	0	0	1	0
ENSMUSG00000065203	0	0	0	0	0	0	0	0	0	0
ENSMUSG00000083142	0	0	0	0	0	0	0	0	0	0
ENSMUSG00000083144	0	0	0	0	0	0	0	0	0	0
ENSMUSG00000037130	0	0	0	0	0	0	0	0	0	0
ENSMUSG00000087043	0	0	0	0	0	0	0	0	0	0
ENSMUSG00000065201	0	0	0	0	0	0	0	0	0	0
ENSMUSG00000102296	0	0	0	0	0	0	0	0	0	0
ENSMUSG00000087792	0	0	0	0	0	0	0	0	0	0
ENSMUSG00000041505	0	0	0	0	0	0	0	0	0	0
ENSMUSG00000087799	0	0	0	0	0	0	0	0	0	0
ENSMUSG00000057612	0	0	0	0	0	0	0	0	0	0
ENSMUSG00000106504	0	0	0	0	0	0	0	0	0	0
ENSMUSG00000085798	0	0	0	0	0	0	0	0	0	0
ENSMUSG00000085797	0	0	0	0	3	0	0	0	0	0
ENSMUSG00000106506	0	0	0	0	0	0	0	0	0	0
ENSMUSG00000085794	0	2	0	0	0	0	0	0	3	0
ENSMUSG00000106507	0	0	0	0	0	0	0	0	0	0
ENSMUSG00000102799	0	0	0	0	0	0	0	0	0	0
ENSMUSG00000064717	0	0	0	0	0	0	0	0	0	0
ENSMUSG00000064714	0	0	0	0	0	0	0	0	0	0
ENSMUSG00000102795	0	0	3	0	0	0	0	0	0	0
ENSMUSG00000064718	0	0	0	0	0	0	0	0	0	0
ENSMUSG00000102796	3	7	11	6	0	3	0	13	9	12
ENSMUSG00000102791	0	0	0	0	0	0	0	0	0	0
ENSMUSG00000102790	0	0	0	0	1	0	0	0	0	0
ENSMUSG00000102793	0	0	0	0	0	0	0	0	0	0
ENSMUSG00000102792	0	0	0	0	0	0	0	0	0	0
ENSMUSG00000087869	0	0	0	0	0	0	0	0	0	0
ENSMUSG00000031998	0	0	0	0	0	0	0	0	0	0
ENSMUSG00000087863	0	0	0	0	0	0	0	0	0	0
ENSMUSG00000087862	0	0	0	0	0	0	0	0	0	0
ENSMUSG00000031995	52	54	64	44	48	140	185	77	213	87
ENSMUSG00000087867	0	0	0	0	0	0	0	0	0	0
ENSMUSG00000031991	0	0	0	0	0	0	0	0	0	0

ENSMUSG00000066748	0	0	0	0	0	0	0	0	0	0
ENSMUSG00000005824	38	20	49	233	46	63	51	39	110	56
ENSMUSG00000074128	0	0	0	0	0	0	4	0	0	0
ENSMUSG00000085424	0	0	2	0	0	0	2	0	0	0
ENSMUSG00000085427	34	60	75	56	58	81	57	74	26	44
ENSMUSG00000085426	0	0	0	0	0	0	0	0	0	0
ENSMUSG00000085421	0	0	0	0	0	0	0	0	0	0
ENSMUSG00000085420	0	0	0	0	0	0	0	0	0	0
ENSMUSG00000074121	15	25	15	13	18	12	11	9	29	9
ENSMUSG00000074123	0	0	0	0	0	0	0	0	0	0
ENSMUSG00000085429	0	0	2	0	0	0	0	0	0	0
ENSMUSG00000085428	0	0	0	0	0	0	0	0	2	0
ENSMUSG00000074127	0	0	0	0	0	0	0	0	0	0
ENSMUSG00000107312	4	6	0	0	9	0	5	0	0	0
ENSMUSG00000107310	29	13	18	13	23	32	29	23	20	19
ENSMUSG00000107315	0	0	0	0	0	0	0	0	0	0
ENSMUSG00000036422	2	6	12	0	0	3	0	0	9	6
ENSMUSG00000079061	0	0	0	0	0	0	0	0	0	1
ENSMUSG00000079064	0	0	0	0	0	0	0	0	0	0
ENSMUSG00000101361	0	2	0	0	0	0	0	0	0	0
ENSMUSG00000103692	0	3	0	0	0	0	0	0	0	0
ENSMUSG00000100411	0	0	0	0	0	0	0	0	0	0
ENSMUSG00000103542	0	0	0	0	0	0	0	0	0	0
ENSMUSG00000103541	0	0	0	0	0	0	0	0	0	0
ENSMUSG00000103540	0	0	0	0	0	0	0	0	0	0
ENSMUSG00000103545	0	0	0	0	0	0	0	0	0	0
ENSMUSG00000103549	0	0	0	0	0	0	0	0	0	0
ENSMUSG00000059229	0	0	0	0	0	0	0	0	0	0
ENSMUSG00000039672	0	0	0	0	3	6	12	0	3	3
ENSMUSG00000035191	0	0	0	0	0	3	0	0	0	2
ENSMUSG00000039676	3	0	12	0	6	6	9	6	0	0
ENSMUSG00000097453	0	0	0	0	0	0	0	0	0	0
ENSMUSG00000097452	0	0	0	0	0	0	0	0	0	0
ENSMUSG00000097450	0	0	0	0	0	1	0	0	1	0
ENSMUSG00000097455	12	6	18	22	5	18	9	17	18	6
ENSMUSG00000097458	0	0	0	0	0	0	0	0	0	0
ENSMUSG00000077635	0	0	0	0	0	0	0	0	0	0
ENSMUSG00000106401	0	0	0	0	0	0	0	0	0	0
ENSMUSG00000081653	0	0	0	0	0	0	0	0	0	0
ENSMUSG00000100938	0	0	0	0	0	0	0	0	0	0
ENSMUSG00000100932	0	0	3	0	0	0	2	3	3	3
ENSMUSG00000089423	0	0	0	0	0	0	0	0	0	0
ENSMUSG00000100930	0	0	0	0	0	0	0	0	0	0
ENSMUSG00000114411	0	0	0	0	0	0	0	0	0	0
ENSMUSG00000114410	0	0	0	0	0	0	0	0	0	0
ENSMUSG00000089427	0	0	0	0	0	0	3	0	0	0
ENSMUSG00000077738	0	0	0	0	0	0	0	0	0	0
ENSMUSG00000109159	0	0	0	0	0	0	0	0	0	0
ENSMUSG00000020447	8	9	3	11	3	0	3	5	6	6
ENSMUSG00000050765	3	0	1	0	0	0	3	6	0	3
ENSMUSG00000050766	0	0	0	0	0	0	0	0	0	0
ENSMUSG00000050762	7	5	2	0	0	0	13	12	5	3
ENSMUSG00000050763	0	0	0	0	0	0	0	6	0	0
ENSMUSG00000105260	0	2	0	0	0	0	0	0	0	0
ENSMUSG00000105261	6	3	3	3	0	13	4	0	9	4
ENSMUSG00000105262	0	0	0	0	0	0	2	0	0	0
ENSMUSG00000105263	57	80	85	47	51	92	57	83	62	64
ENSMUSG00000105264	3	0	0	0	0	6	0	0	0	8

ENSMUSG00000105265	21	12	5	6	5	3	2	10	1	0
ENSMUSG00000105266	0	0	0	0	0	0	0	0	0	0
ENSMUSG00000105267	0	0	0	0	0	0	0	0	0	0
ENSMUSG00000105269	0	0	0	0	0	0	0	0	0	0
ENSMUSG00000073894	0	0	0	0	0	0	0	0	0	0
ENSMUSG00000066820	0	0	0	0	0	0	0	0	0	0
ENSMUSG00000077736	0	0	0	0	0	0	0	0	0	0
ENSMUSG00000058685	0	0	0	0	0	0	0	0	0	0
ENSMUSG00000066828	0	0	0	0	0	0	0	0	0	0
ENSMUSG00000081720	4	3	2	8	0	0	4	3	4	0
ENSMUSG00000091859	0	0	0	0	0	0	0	0	0	0
ENSMUSG00000105885	0	0	0	0	2	0	0	0	0	0
ENSMUSG00000105887	0	0	0	0	0	0	0	0	0	0
ENSMUSG00000105880	0	0	0	0	0	0	0	0	0	0
ENSMUSG00000105882	0	0	0	0	0	0	0	0	0	0
ENSMUSG00000057754	0	0	0	0	0	0	0	0	0	0
ENSMUSG00000096496	0	0	0	0	0	0	0	0	0	0
ENSMUSG00000096497	0	0	0	0	0	0	0	0	0	0
ENSMUSG00000105888	0	0	0	0	0	0	0	0	0	0
ENSMUSG00000105889	0	0	0	0	0	0	0	0	0	0
ENSMUSG00000096492	1	0	1	1	0	0	0	1	0	1
ENSMUSG00000020940	6	2	3	7	2	2	3	0	5	5
ENSMUSG00000108450	0	0	0	0	0	0	0	0	0	0
ENSMUSG00000108451	0	0	0	0	0	0	0	0	0	0
ENSMUSG00000108454	0	0	0	0	0	0	0	0	0	0
ENSMUSG00000108456	15	23	11	32	0	21	29	27	24	41
ENSMUSG00000108457	0	0	0	0	0	0	0	0	0	0
ENSMUSG00000104137	0	0	0	0	0	0	0	0	0	0
ENSMUSG00000108459	0	0	0	0	0	0	0	0	0	0
ENSMUSG00000104135	0	0	0	0	0	0	0	0	0	0
ENSMUSG00000104134	0	0	0	0	0	0	0	0	0	0
ENSMUSG00000104132	0	0	0	0	0	3	2	0	0	0
ENSMUSG00000104131	0	0	0	0	0	0	0	0	0	0
ENSMUSG00000021314	147	112	185	123	104	199	141	85	78	142
ENSMUSG00000057280	18	55	61	44	27	74	137	109	72	73
ENSMUSG00000059768	0	0	0	0	0	0	0	0	0	0
ENSMUSG00000059763	0	0	0	0	0	0	6	0	0	0
ENSMUSG00000059762	0	0	0	0	0	0	0	0	0	0
ENSMUSG00000102518	0	0	0	0	0	0	0	0	0	0
ENSMUSG00000102417	0	0	0	0	0	0	0	0	0	0
ENSMUSG00000110882	0	6	0	0	0	0	0	0	0	0
ENSMUSG00000110880	0	0	0	0	0	0	0	0	0	0
ENSMUSG00000110881	0	0	0	0	0	0	0	0	0	0
ENSMUSG00000110886	0	0	0	0	0	0	0	0	0	0
ENSMUSG00000110884	0	0	2	0	0	0	0	0	6	0
ENSMUSG00000110888	0	0	0	0	0	12	3	0	9	12
ENSMUSG00000110889	0	0	0	0	0	0	0	0	0	0
ENSMUSG00000091148	0	0	0	0	0	0	1	0	0	0
ENSMUSG00000065410	0	1	0	0	0	0	0	0	0	0
ENSMUSG00000091142	0	0	0	0	0	0	0	0	0	0
ENSMUSG00000091140	0	0	0	0	0	0	0	0	0	0
ENSMUSG00000091146	0	0	0	0	0	0	0	0	0	0
ENSMUSG00000056155	10	11	23	20	5	6	9	10	11	22
ENSMUSG00000056158	6	6	7	0	7	3	0	0	0	0
ENSMUSG00000111565	0	0	0	0	0	0	0	0	0	0
ENSMUSG00000053508	0	0	0	0	0	0	0	0	0	2
ENSMUSG00000042501	0	0	0	0	7	0	0	3	0	0
ENSMUSG00000094539	0	3	0	0	0	0	0	0	0	0

ENSMUSG00000094538	0	0	0	0	0	0	0	0	0	0
ENSMUSG00000022155	0	0	0	3	0	0	0	0	0	0
ENSMUSG00000094535	0	0	0	0	0	0	0	0	0	0
ENSMUSG00000094534	0	0	0	0	0	0	0	0	3	0
ENSMUSG00000111823	0	0	0	0	0	0	0	0	0	0
ENSMUSG00000111822	0	0	0	0	0	0	0	0	0	0
ENSMUSG00000111825	0	0	0	0	0	0	0	0	0	0
ENSMUSG00000065906	0	0	0	0	0	0	0	0	0	0
ENSMUSG00000065907	0	0	0	0	0	0	0	0	0	0
ENSMUSG00000065903	0	0	0	0	0	0	0	0	0	0
ENSMUSG00000094391	0	0	0	0	0	0	0	0	0	0
ENSMUSG00000027925	0	0	0	0	0	0	0	0	0	0
ENSMUSG00000027927	0	0	0	0	0	0	0	0	3	0
ENSMUSG00000093169	0	1	0	1	1	2	1	1	1	3
ENSMUSG00000027923	0	0	0	0	0	0	0	0	0	0
ENSMUSG00000094399	0	0	0	0	0	0	0	0	0	0
ENSMUSG00000093164	0	0	0	0	0	0	0	0	0	0
ENSMUSG00000093163	0	1	0	0	0	0	1	0	0	0
ENSMUSG00000093161	0	0	0	0	0	0	0	0	0	0
ENSMUSG00000093160	0	0	0	0	0	0	0	0	0	0
ENSMUSG00000086226	0	0	0	0	0	0	0	0	0	0
ENSMUSG00000086227	0	0	0	0	0	0	0	0	0	0
ENSMUSG00000086224	0	0	0	0	0	0	0	0	3	0
ENSMUSG00000017688	0	0	0	0	0	0	3	0	0	0
ENSMUSG00000086228	25	17	26	0	4	3	10	15	27	24
ENSMUSG00000097848	6	3	3	12	6	18	0	3	3	6
ENSMUSG00000097840	0	0	6	0	0	0	7	0	3	3
ENSMUSG00000097841	0	0	0	0	0	0	0	0	0	0
ENSMUSG00000090219	0	0	0	0	0	0	0	0	0	0
ENSMUSG00000017978	410	306	387	468	341	434	723	372	464	492
ENSMUSG00000049411	335	299	296	327	218	370	393	382	389	428
ENSMUSG00000029564	0	0	0	8	0	3	0	0	4	0
ENSMUSG00000047021	0	0	0	0	3	0	0	0	0	0
ENSMUSG00000047025	0	0	0	3	0	0	3	0	0	0
ENSMUSG00000084802	0	0	0	0	0	0	0	0	0	0
ENSMUSG00000003070	105	91	110	68	82	55	103	95	91	69
ENSMUSG00000071640	4	0	3	7	5	3	0	0	0	9
ENSMUSG00000046991	0	0	3	0	0	0	0	2	0	6
ENSMUSG00000109195	4	0	4	0	3	4	0	0	0	0
ENSMUSG00000109192	0	0	0	0	0	0	0	0	0	0
ENSMUSG00000046995	0	0	0	0	0	0	0	0	0	0
ENSMUSG00000109190	0	0	0	0	0	0	0	0	0	0
ENSMUSG00000076140	1	0	0	0	0	0	0	0	1	0
ENSMUSG00000106219	7	4	6	9	9	3	5	9	15	6
ENSMUSG00000076142	1	0	1	1	1	1	0	1	0	1
ENSMUSG00000102388	0	0	0	0	0	0	0	0	0	0
ENSMUSG00000106215	0	0	0	0	0	0	0	0	0	0
ENSMUSG00000109657	0	0	0	0	0	0	0	0	0	0
ENSMUSG00000005952	9	3	6	3	24	2	35	29	27	22
ENSMUSG00000109651	0	0	3	0	0	6	11	0	8	0
ENSMUSG00000107263	0	3	0	0	0	0	3	3	0	5
ENSMUSG00000109650	0	0	0	0	0	0	0	0	0	0
ENSMUSG00000109443	0	0	0	0	0	0	0	0	0	0
ENSMUSG00000109444	0	0	0	0	0	0	0	0	0	0
ENSMUSG00000109446	0	0	0	0	0	0	0	3	0	0
ENSMUSG00000109447	0	0	0	0	0	0	0	0	0	0
ENSMUSG00000096821	0	0	0	0	0	0	0	0	0	0
ENSMUSG00000096823	0	0	0	0	0	0	0	0	0	0

ENSMUSG00000096822	0	0	0	0	0	0	0	0	0	0
ENSMUSG00000104909	0	0	0	0	0	0	0	0	0	0
ENSMUSG00000096829	0	0	0	0	0	0	0	0	0	0
ENSMUSG00000030742	75	25	47	106	47	44	127	138	135	102
ENSMUSG00000110064	0	0	0	0	0	0	0	0	0	0
ENSMUSG00000110066	0	0	0	3	0	0	0	0	0	0
ENSMUSG00000110061	0	3	0	0	2	0	3	5	0	0
ENSMUSG00000110063	0	0	3	0	0	0	0	0	2	0
ENSMUSG00000104904	9	10	7	2	11	10	4	8	6	10
ENSMUSG00000054146	0	0	0	0	0	0	0	0	0	0
ENSMUSG00000054141	0	0	0	0	0	0	0	0	0	0
ENSMUSG00000054142	0	0	0	0	0	0	0	0	0	0
ENSMUSG00000104902	0	0	0	0	0	0	0	0	0	0
ENSMUSG00000110311	0	1	1	1	0	1	0	1	1	1
ENSMUSG00000058755	23	23	9	28	27	36	33	11	25	8
ENSMUSG00000105746	16	8	6	0	4	0	21	23	6	0
ENSMUSG00000105745	26	23	27	0	12	22	16	12	13	14
ENSMUSG00000105744	2	2	0	0	4	0	0	3	0	1
ENSMUSG00000105749	0	0	0	0	0	0	0	0	0	0
ENSMUSG00000082354	0	0	0	0	0	0	0	0	0	0
ENSMUSG00000083805	0	0	0	0	0	0	0	0	0	0
ENSMUSG00000005045	76	81	79	34	42	44	102	79	60	40
ENSMUSG00000108716	0	0	0	0	0	0	0	0	0	0
ENSMUSG00000108713	0	0	0	0	0	0	0	0	0	0
ENSMUSG00000108712	0	0	0	3	0	0	0	0	0	0
ENSMUSG00000108711	0	1	1	0	0	0	0	0	0	1
ENSMUSG00000108719	0	0	0	0	0	0	0	0	0	0
ENSMUSG00000108718	0	0	0	0	0	0	0	0	0	0
ENSMUSG00000083804	0	0	0	0	0	0	0	0	0	0
ENSMUSG00000111560	0	0	0	0	0	0	0	0	0	0
ENSMUSG00000083303	0	0	0	0	0	0	0	0	0	0
ENSMUSG00000083300	0	0	0	0	0	0	1	0	0	0
ENSMUSG00000099738	0	0	0	0	0	0	0	0	0	0
ENSMUSG00000083309	0	0	0	0	0	0	0	0	0	0
ENSMUSG00000000673	12	5	14	36	4	7	50	16	35	27
ENSMUSG00000041078	0	3	0	1	0	3	3	0	0	0
ENSMUSG00000083807	0	0	0	0	0	0	0	0	0	0
ENSMUSG00000112446	2	8	12	5	2	10	15	12	17	17
ENSMUSG00000112447	3	0	0	0	0	0	0	0	0	0
ENSMUSG00000085223	0	0	4	5	2	5	9	0	3	2
ENSMUSG00000102126	0	0	0	0	0	0	0	0	0	0
ENSMUSG00000085221	0	0	0	0	0	0	0	0	0	0
ENSMUSG00000100268	0	0	0	0	0	0	0	0	0	0
ENSMUSG00000085226	0	1	6	0	0	0	0	0	0	0
ENSMUSG00000085225	0	0	0	6	0	0	0	0	0	0
ENSMUSG00000085224	0	0	0	0	0	0	0	0	0	0
ENSMUSG00000026648	568	613	674	661	534	665	742	656	612	639
ENSMUSG00000026646	202	227	216	218	169	269	265	224	250	196
ENSMUSG00000102122	0	0	0	0	0	0	0	0	0	0
ENSMUSG00000026644	42	15	8	45	17	15	18	20	28	27
ENSMUSG00000026645	0	3	0	0	0	0	0	0	0	0
ENSMUSG00000093952	0	0	0	0	0	0	0	0	0	0
ENSMUSG00000093950	0	0	0	0	0	0	0	0	0	0
ENSMUSG00000079263	0	0	0	0	0	0	0	0	0	0
ENSMUSG00000079265	0	0	0	0	0	0	0	0	0	0
ENSMUSG00000079266	0	0	0	0	0	0	0	0	0	0
ENSMUSG00000100262	0	0	0	0	0	0	0	0	0	0
ENSMUSG00000079269	0	0	0	0	0	0	0	0	0	0

ENSMUSG00000083801	0	0	0	0	0	0	0	0	0	0
ENSMUSG00000100260	0	0	0	0	0	0	0	0	0	0
ENSMUSG00000100266	0	0	0	0	0	0	0	0	3	0
ENSMUSG00000100265	0	0	0	0	0	0	0	0	0	0
ENSMUSG00000100264	0	0	0	0	0	0	0	0	0	0
ENSMUSG00000045318	55	78	47	46	72	107	98	93	42	67
ENSMUSG00000112941	34	22	34	22	26	27	41	27	13	20
ENSMUSG00000045314	0	2	0	0	6	0	2	0	0	3
ENSMUSG00000113760	0	0	0	0	0	0	0	0	0	0
ENSMUSG00000070178	0	0	0	0	0	0	0	0	0	0
ENSMUSG00000114843	0	0	0	0	0	0	0	0	0	0
ENSMUSG00000114840	0	0	0	0	0	0	0	0	0	0
ENSMUSG00000071493	0	0	0	0	0	0	0	0	0	0
ENSMUSG00000114846	0	0	0	0	0	0	0	0	3	0
ENSMUSG00000102196	0	0	0	0	0	0	0	0	0	0
ENSMUSG00000114845	0	0	0	0	0	0	0	0	0	0
ENSMUSG00000063506	242	253	265	339	178	246	489	224	346	298
ENSMUSG00000063507	0	0	0	0	0	0	0	0	0	0
ENSMUSG00000074454	0	0	0	0	0	0	0	0	0	0
ENSMUSG00000078137	161	111	88	133	162	145	57	57	44	73
ENSMUSG00000078132	0	0	0	0	0	0	0	0	0	0
ENSMUSG00000078131	0	0	0	0	0	0	0	0	0	0
ENSMUSG00000078130	0	0	0	0	0	0	0	0	0	0
ENSMUSG00000107674	0	0	0	0	0	0	0	0	0	0
ENSMUSG00000081218	0	0	0	0	0	0	0	0	0	0
ENSMUSG00000081216	0	0	0	0	0	0	0	0	0	0
ENSMUSG00000081215	0	0	0	0	0	0	0	0	0	0
ENSMUSG00000081211	0	0	0	0	0	0	0	0	0	0
ENSMUSG00000110352	0	0	2	0	0	0	0	2	0	0
ENSMUSG00000073103	0	0	0	0	0	0	0	0	0	0
ENSMUSG00000073102	0	12	0	0	0	6	9	3	0	0
ENSMUSG00000099965	0	0	0	0	0	0	0	0	0	0
ENSMUSG00000099964	0	0	0	0	0	0	0	0	0	0
ENSMUSG00000099967	0	0	0	0	0	0	0	0	0	0
ENSMUSG00000099961	0	0	0	0	0	0	0	0	0	0
ENSMUSG00000099960	0	0	0	2	0	0	0	0	0	0
ENSMUSG00000099963	0	0	0	0	0	0	0	0	0	0
ENSMUSG00000099962	0	0	0	0	0	0	0	0	0	0
ENSMUSG00000099969	0	0	0	0	0	0	0	0	0	0
ENSMUSG00000099968	0	0	0	0	0	0	0	0	0	0
ENSMUSG00000101303	0	0	0	0	0	0	0	0	0	0
ENSMUSG00000048400	0	0	0	0	0	0	0	0	0	0
ENSMUSG00000048402	84	108	101	96	68	182	271	195	166	151
ENSMUSG00000077378	0	0	0	0	0	0	0	0	0	0
ENSMUSG00000077379	0	0	0	0	0	0	0	0	0	0
ENSMUSG00000113688	3	12	9	6	0	2	5	9	24	0
ENSMUSG00000098611	0	0	0	0	0	0	0	0	0	0
ENSMUSG00000098612	0	0	0	0	0	0	0	0	0	0
ENSMUSG00000031383	0	3	15	0	0	0	9	6	7	18
ENSMUSG00000031381	318	311	291	282	242	195	485	313	324	350
ENSMUSG00000098619	0	0	0	0	0	0	0	0	0	0
ENSMUSG00000031385	208	321	316	193	200	207	137	278	156	211
ENSMUSG00000031384	0	0	0	0	0	0	0	0	0	0
ENSMUSG00000030382	8	6	0	13	0	0	6	9	0	7
ENSMUSG00000102968	0	0	0	0	0	0	0	0	0	0
ENSMUSG00000102963	6	0	6	2	9	3	2	12	0	14
ENSMUSG00000043003	347	245	335	176	253	300	280	270	152	205
ENSMUSG00000102966	0	0	0	0	0	0	0	0	0	0

ENSMUSG00000102965	0	0	0	0	0	0	0	0	0	0
ENSMUSG00000112149	5	0	0	0	3	5	2	0	0	0
ENSMUSG00000049758	0	0	0	0	0	0	0	0	0	0
ENSMUSG00000041592	113	141	152	146	155	241	314	170	150	168
ENSMUSG00000031654	29	36	39	15	12	17	12	30	12	15
ENSMUSG00000031651	0	0	0	0	0	0	0	0	0	0
ENSMUSG00000101305	0	0	0	0	0	0	0	0	0	0
ENSMUSG00000093745	0	0	0	0	0	0	0	0	0	1
ENSMUSG00000067971	0	0	0	0	0	0	0	0	0	0
ENSMUSG00000030525	22	9	16	13	23	18	36	18	16	18
ENSMUSG00000088159	0	0	0	0	0	0	0	0	0	0
ENSMUSG00000082178	0	0	0	0	0	0	0	0	0	0
ENSMUSG00000030523	21	22	1	17	3	3	19	19	10	8
ENSMUSG00000088152	0	0	0	0	0	0	0	0	0	0
ENSMUSG00000082177	0	0	0	0	0	0	0	0	0	0
ENSMUSG00000088150	0	0	0	0	0	0	0	0	0	0
ENSMUSG00000082175	0	0	0	0	0	0	0	0	0	0
ENSMUSG00000082172	0	0	0	0	0	0	0	0	0	0
ENSMUSG00000088154	0	0	0	0	0	0	0	0	1	3
ENSMUSG00000088155	0	0	0	0	0	0	0	0	0	0
ENSMUSG00000101433	0	2	0	0	0	0	0	2	0	0
ENSMUSG00000101435	0	0	0	0	0	0	0	0	0	0
ENSMUSG00000101434	0	0	0	0	0	0	0	0	0	0
ENSMUSG00000101436	0	0	0	0	0	0	0	0	0	0
ENSMUSG00000021208	33	11	63	92	44	16	27	11	30	32
ENSMUSG00000107873	0	0	0	0	0	0	0	0	0	6
ENSMUSG00000107876	5	0	0	0	0	0	6	0	4	6
ENSMUSG00000107879	164	152	177	143	122	250	187	186	223	191
ENSMUSG00000103362	0	0	0	0	0	0	0	0	0	0
ENSMUSG00000103361	0	0	0	0	0	0	0	0	0	0
ENSMUSG00000103365	0	0	0	0	0	0	0	0	0	0
ENSMUSG00000103369	0	0	0	0	0	0	0	0	0	0
ENSMUSG00000103368	0	0	0	0	0	3	39	0	12	3
ENSMUSG00000040061	213	186	197	241	124	198	212	219	174	191
ENSMUSG00000040065	0	0	3	0	0	0	0	0	0	0
ENSMUSG00000089208	0	1	0	0	0	0	0	0	0	0
ENSMUSG00000084639	0	0	0	0	0	3	0	0	0	0
ENSMUSG00000084633	0	0	0	0	0	0	0	0	0	0
ENSMUSG00000084631	0	0	0	0	0	0	0	0	0	2
ENSMUSG00000084637	0	0	0	0	0	0	0	0	0	0
ENSMUSG00000084636	0	0	0	0	0	0	0	0	0	0
ENSMUSG00000084635	0	0	0	0	0	0	0	0	0	0
ENSMUSG00000089206	0	0	0	0	0	0	0	0	0	0
ENSMUSG00000075198	0	0	0	0	0	0	0	0	0	0
ENSMUSG00000075199	0	3	0	0	0	0	0	0	0	0
ENSMUSG00000075196	0	0	0	0	0	0	0	0	0	0
ENSMUSG00000075197	0	0	0	0	0	0	0	0	0	0
ENSMUSG00000075194	0	0	0	0	0	0	0	0	0	0
ENSMUSG00000075195	0	0	0	0	0	0	0	0	0	0
ENSMUSG00000075192	0	0	0	0	0	0	0	0	0	0
ENSMUSG00000075193	0	0	0	0	0	0	0	0	0	0
ENSMUSG00000075190	0	0	0	0	0	0	0	0	3	0
ENSMUSG00000022311	0	0	0	0	3	0	8	0	0	0
ENSMUSG00000022315	0	0	0	0	0	0	0	0	0	0
ENSMUSG00000021081	0	0	0	15	0	0	0	0	0	0
ENSMUSG00000064697	0	0	0	0	0	0	0	0	0	0
ENSMUSG00000064691	0	0	0	0	0	0	0	0	0	0
ENSMUSG00000064698	0	0	0	0	0	0	0	0	0	0

ENSMUSG00000095938	0	0	0	0	0	0	0	0	0	0
ENSMUSG00000020229	0	0	0	0	0	4	0	0	0	0
ENSMUSG00000049028	0	0	0	0	0	0	0	0	3	3
ENSMUSG00000095936	0	0	0	0	0	0	0	0	0	0
ENSMUSG00000100472	0	0	0	0	0	0	0	0	1	0
ENSMUSG00000100473	0	0	0	0	0	0	0	0	0	0
ENSMUSG00000041333	0	0	0	0	0	0	0	0	0	0
ENSMUSG00000100471	0	0	0	0	0	0	0	0	0	0
ENSMUSG00000100476	0	0	0	0	0	0	0	0	0	0
ENSMUSG00000100477	0	0	0	0	0	0	0	0	0	0
ENSMUSG00000100474	0	0	0	0	0	0	0	0	0	0
ENSMUSG00000100475	0	0	0	0	0	0	0	0	0	0
ENSMUSG00000096564	0	0	0	0	0	0	0	0	0	0
ENSMUSG00000100478	0	0	0	0	0	0	0	0	0	0
ENSMUSG00000100479	0	0	0	0	0	0	0	0	0	0
ENSMUSG00000068999	0	0	0	0	0	0	0	0	0	0
ENSMUSG00000096565	0	0	0	0	0	0	0	0	0	0
ENSMUSG00000096566	0	0	0	0	0	0	0	0	0	0
ENSMUSG00000113716	0	0	0	0	0	0	0	0	2	0
ENSMUSG00000113714	0	0	0	0	0	0	0	0	0	0
ENSMUSG00000102188	0	0	0	0	0	0	0	0	0	0
ENSMUSG00000102189	0	0	0	0	3	6	0	6	1	0
ENSMUSG00000064527	0	0	0	0	0	0	0	0	0	0
ENSMUSG00000064526	0	0	0	0	0	0	0	0	0	0
ENSMUSG00000064529	0	0	0	0	0	0	0	0	0	0
ENSMUSG00000102185	0	0	0	0	0	0	0	0	0	0
ENSMUSG00000035547	333	382	354	406	292	356	591	418	529	457
ENSMUSG00000102187	0	0	0	0	0	0	0	0	0	0
ENSMUSG00000102180	0	0	0	0	0	0	2	0	0	0
ENSMUSG00000035540	3	0	26	2	0	0	0	0	3	15
ENSMUSG00000102182	0	0	0	0	0	0	0	0	0	0
ENSMUSG00000088966	0	0	0	0	0	0	0	0	0	0
ENSMUSG00000107122	0	0	0	0	0	0	0	0	0	0
ENSMUSG00000107125	0	0	0	0	0	0	0	0	0	0
ENSMUSG00000113711	0	0	0	0	0	0	0	0	0	0
ENSMUSG00000107129	0	0	0	0	0	0	0	0	0	0
ENSMUSG00000113710	3	0	0	0	0	3	0	0	3	0
ENSMUSG00000079741	0	0	0	0	0	0	0	0	0	0
ENSMUSG00000079740	0	0	0	0	0	0	0	0	0	0
ENSMUSG00000023047	20	6	23	8	12	21	48	22	7	7
ENSMUSG00000023045	15	16	2	46	14	17	33	26	60	32
ENSMUSG00000023041	0	0	0	0	0	0	0	0	0	0
ENSMUSG00000028012	0	22	6	0	0	9	40	14	11	16
ENSMUSG00000078481	0	0	0	0	0	0	0	0	0	0
ENSMUSG00000069117	18	23	20	43	22	29	0	30	24	32
ENSMUSG00000096180	0	0	0	0	0	0	0	0	0	0
ENSMUSG00000069118	0	0	0	0	0	0	0	0	0	0
ENSMUSG00000074715	9	15	11	12	12	41	20	43	6	35
ENSMUSG00000029769	174	116	151	80	146	218	418	276	255	192
ENSMUSG00000029766	0	0	0	0	0	0	0	0	0	0
ENSMUSG00000101981	0	0	0	0	0	0	0	0	0	0
ENSMUSG00000101980	0	0	0	0	0	0	0	0	0	0
ENSMUSG00000101983	0	0	0	0	0	0	0	0	0	0
ENSMUSG00000101982	0	0	0	0	0	0	0	0	0	0
ENSMUSG00000101985	0	0	0	0	0	0	0	0	0	0
ENSMUSG00000101984	0	0	0	0	0	0	0	0	0	0
ENSMUSG00000101987	0	0	0	0	0	0	0	0	0	0
ENSMUSG00000088638	0	0	0	0	0	0	0	0	0	0

ENSMUSG00000101989	0	0	0	0	0	0	0	0	0	0
ENSMUSG00000101988	0	0	0	0	0	0	0	0	1	0
ENSMUSG00000088635	0	0	0	0	0	0	0	0	0	0
ENSMUSG00000088633	0	0	0	0	0	0	0	0	0	0
ENSMUSG00000061469	0	0	0	0	0	0	0	0	0	0
ENSMUSG00000088630	0	0	0	0	0	0	0	0	0	0
ENSMUSG00000021680	3	0	3	0	0	0	0	0	0	0
ENSMUSG00000021685	0	0	0	0	0	0	0	0	0	0
ENSMUSG00000063535	49	47	36	67	50	31	145	115	111	43
ENSMUSG00000024064	6	0	3	3	3	3	3	3	0	3
ENSMUSG00000088988	0	0	0	0	0	0	0	0	0	0
ENSMUSG00000106454	0	0	0	0	0	0	0	0	0	0
ENSMUSG00000106455	10	15	2	16	1	18	5	10	5	17
ENSMUSG00000038199	0	2	0	0	0	3	0	0	0	0
ENSMUSG00000113778	0	0	0	0	0	0	0	2	0	0
ENSMUSG00000106450	3	7	5	9	6	9	10	4	8	6
ENSMUSG00000111400	0	0	0	0	0	0	0	0	0	0
ENSMUSG00000088981	0	0	0	0	0	0	1	0	0	0
ENSMUSG00000088980	0	0	0	0	0	0	0	0	0	0
ENSMUSG00000113777	0	0	0	0	0	0	0	0	0	0
ENSMUSG00000088985	0	0	0	0	0	0	0	0	0	3
ENSMUSG00000113770	0	0	2	0	0	0	3	6	0	0
ENSMUSG00000113773	0	0	0	0	0	0	0	0	0	0
ENSMUSG00000113772	0	0	0	0	0	0	0	0	0	0
ENSMUSG00000049674	0	0	0	0	0	0	0	0	0	0
ENSMUSG00000089589	0	0	0	0	0	0	0	0	0	0
ENSMUSG00000089587	0	0	0	0	0	0	0	0	0	0
ENSMUSG00000089584	0	0	0	0	0	0	0	0	0	0
ENSMUSG00000089585	0	0	0	0	0	0	0	0	0	0
ENSMUSG00000089583	0	0	0	0	0	0	0	0	0	0
ENSMUSG00000089580	0	0	0	0	0	0	0	0	0	0
ENSMUSG00000089581	1	1	1	2	0	2	6	3	4	3
ENSMUSG00000097398	0	0	0	0	0	0	0	0	0	0
ENSMUSG00000097399	8	3	0	3	5	7	5	4	6	3
ENSMUSG00000106982	0	0	0	0	0	0	0	0	0	0
ENSMUSG00000106986	2	3	2	3	2	2	1	2	1	2
ENSMUSG00000106987	0	0	0	0	0	0	3	0	3	0
ENSMUSG00000097391	84	88	25	107	46	61	101	48	87	71
ENSMUSG00000022886	0	0	0	0	0	0	0	0	0	0
ENSMUSG00000097642	0	0	0	0	0	0	0	2	0	0
ENSMUSG00000054383	33	35	17	22	31	34	47	36	38	31
ENSMUSG00000104588	0	0	0	0	0	0	3	0	3	3
ENSMUSG00000097646	0	0	0	0	0	2	0	0	0	0
ENSMUSG00000097645	8	16	20	13	5	12	27	9	21	18
ENSMUSG00000104582	0	0	0	0	0	0	0	0	0	0
ENSMUSG00000097648	0	0	0	0	0	0	0	0	0	0
ENSMUSG00000104586	0	6	0	3	0	3	3	6	0	0
ENSMUSG00000104587	0	0	0	0	0	0	0	0	0	0
ENSMUSG00000104584	0	0	0	0	0	0	0	0	0	0
ENSMUSG00000101129	0	0	0	0	0	0	0	0	0	0
ENSMUSG00000069806	109	123	134	141	91	223	258	179	208	156
ENSMUSG00000105185	0	0	0	0	0	0	0	0	0	0
ENSMUSG00000105184	3	6	6	4	0	3	27	15	20	15
ENSMUSG00000105186	2	2	3	2	2	4	2	2	2	4
ENSMUSG00000114208	0	0	0	0	0	0	0	0	0	0
ENSMUSG00000109603	9	3	0	0	0	3	14	3	0	3
ENSMUSG00000105183	0	0	0	0	0	0	0	0	0	0
ENSMUSG00000107432	4	4	6	0	0	1	5	0	0	3

ENSMUSG00000114204	0	0	0	0	0	0	0	0	0	0
ENSMUSG00000114206	0	0	0	5	0	0	0	3	0	0
ENSMUSG00000114207	3	0	0	0	0	0	0	0	0	0
ENSMUSG00000105189	0	0	3	3	2	8	0	5	2	2
ENSMUSG00000114201	0	0	0	0	0	0	3	0	0	0
ENSMUSG00000114203	0	0	0	0	0	0	2	0	0	0
ENSMUSG00000107434	0	0	3	0	0	3	3	3	0	0
ENSMUSG00000112917	0	0	2	0	1	0	0	0	0	0
ENSMUSG00000112914	3	0	0	9	2	3	10	3	6	0
ENSMUSG00000112915	0	0	0	0	0	0	0	0	0	0
ENSMUSG00000112912	0	0	0	0	0	0	0	0	0	0
ENSMUSG00000112913	0	0	0	0	0	0	0	0	0	0
ENSMUSG00000112910	0	0	0	0	0	0	0	0	0	0
ENSMUSG00000112919	1	5	3	4	3	1	1	8	7	2
ENSMUSG00000107438	0	0	0	0	0	0	0	0	0	0
ENSMUSG00000058728	0	0	0	0	0	0	5	4	0	0
ENSMUSG00000058729	301	394	390	359	337	303	456	357	446	435
ENSMUSG00000044201	18	16	20	38	18	3	42	0	6	12
ENSMUSG00000058725	0	0	0	0	0	0	0	0	0	0
ENSMUSG00000110455	0	0	0	0	0	0	0	0	0	0
ENSMUSG00000110454	0	0	0	0	0	0	0	0	0	0
ENSMUSG00000110450	0	0	0	0	0	0	0	0	0	0
ENSMUSG00000060780	0	3	0	0	0	0	0	3	0	0
ENSMUSG00000050266	0	0	3	0	0	4	3	0	0	0
ENSMUSG00000060787	0	0	0	0	0	0	0	0	0	0
ENSMUSG00000098508	0	0	0	0	0	0	0	0	0	0
ENSMUSG00000096510	0	1	1	0	1	0	0	1	0	0
ENSMUSG00000096513	0	0	0	0	0	0	0	0	0	0
ENSMUSG00000096512	1	0	1	1	0	0	0	2	0	1
ENSMUSG00000096514	0	0	0	0	0	0	0	0	0	0
ENSMUSG00000096516	0	0	0	0	0	0	0	0	0	0
ENSMUSG00000023868	223	359	327	377	177	396	492	437	392	475
ENSMUSG00000111378	0	0	1	6	0	0	0	3	0	0
ENSMUSG00000101228	0	0	0	0	0	0	0	0	0	0
ENSMUSG00000111568	0	0	0	0	0	0	0	0	0	0
ENSMUSG00000060257	0	0	6	0	3	0	2	0	3	0
ENSMUSG00000060256	0	0	0	0	0	0	0	0	0	0
ENSMUSG00000060254	0	0	0	0	0	0	0	0	0	0
ENSMUSG00000059030	0	0	0	0	0	0	0	0	0	0
ENSMUSG00000056832	238	310	368	176	237	372	533	445	448	328
ENSMUSG00000062873	0	0	0	0	0	0	0	0	0	0
ENSMUSG00000045150	0	0	0	0	0	0	0	0	0	0
ENSMUSG00000026336	0	0	0	0	0	2	0	0	0	0
ENSMUSG00000009356	0	0	0	0	0	0	0	3	0	0
ENSMUSG00000101789	0	0	0	0	0	0	0	0	0	0
ENSMUSG00000026331	0	0	0	0	0	0	0	0	0	0
ENSMUSG00000103940	51	26	17	30	23	28	33	43	50	16
ENSMUSG00000065789	0	0	0	0	0	0	0	0	0	0
ENSMUSG00000095095	0	0	0	0	0	0	0	0	0	0
ENSMUSG00000095094	0	0	0	0	0	0	0	0	0	0
ENSMUSG00000111326	0	0	0	0	0	0	0	0	0	0
ENSMUSG00000111327	0	0	0	0	0	0	0	0	3	0
ENSMUSG00000111320	0	0	0	0	0	0	0	0	0	0
ENSMUSG00000095093	0	0	0	0	0	0	0	0	0	0
ENSMUSG00000095092	0	0	1	0	1	0	1	0	2	3
ENSMUSG00000081054	0	0	0	0	0	0	0	0	0	0
ENSMUSG00000081055	0	0	0	0	0	0	0	0	0	0
ENSMUSG00000081057	0	0	0	0	0	0	0	0	0	0

ENSMUSG00000047799	0	0	0	0	0	0	0	0	0	0
ENSMUSG00000047798	12	15	17	301	37	58	25	37	65	42
ENSMUSG00000047794	0	0	0	0	0	0	0	0	0	0
ENSMUSG00000047797	0	0	3	0	0	0	9	0	0	0
ENSMUSG00000085763	9	3	0	16	0	9	15	14	15	0
ENSMUSG00000022057	3	0	0	0	0	0	0	0	9	3
ENSMUSG00000109952	0	0	0	0	0	0	0	0	0	0
ENSMUSG00000022054	13	23	22	0	15	34	42	23	25	26
ENSMUSG00000090607	0	0	0	2	0	0	0	0	0	0
ENSMUSG00000043969	0	0	0	6	5	3	3	0	0	0
ENSMUSG00000090603	0	0	0	0	0	0	0	0	0	0
ENSMUSG00000081586	0	0	0	0	0	0	0	0	0	0
ENSMUSG00000081587	0	0	0	0	0	0	0	0	0	0
ENSMUSG00000081584	0	0	0	0	0	0	0	0	0	0
ENSMUSG00000048643	0	0	0	0	0	0	0	0	0	0
ENSMUSG00000081580	0	0	0	0	0	0	0	0	0	0
ENSMUSG00000114151	0	0	0	0	0	0	0	0	1	0
ENSMUSG00000093552	0	0	0	0	0	0	0	0	0	0
ENSMUSG00000093551	0	0	0	0	0	0	0	0	0	0
ENSMUSG00000093557	0	0	0	0	0	0	0	0	0	0
ENSMUSG00000114402	0	0	0	0	0	0	0	0	0	0
ENSMUSG00000063478	0	0	0	0	0	0	0	0	0	0
ENSMUSG00000092608	0	0	0	0	0	0	0	0	0	0
ENSMUSG00000092601	0	0	0	0	0	0	0	0	0	0
ENSMUSG00000092600	0	6	0	0	0	0	3	6	11	0
ENSMUSG00000092603	0	0	0	0	0	0	2	0	0	0
ENSMUSG00000092602	0	0	0	4	9	0	0	2	0	12
ENSMUSG00000092606	0	0	0	0	0	0	0	0	0	0
ENSMUSG00000077176	0	0	0	0	0	0	0	0	0	0
ENSMUSG00000077177	0	0	0	0	0	0	0	0	0	0
ENSMUSG00000077174	0	0	0	0	0	0	0	0	0	0
ENSMUSG00000077172	0	0	0	0	0	0	0	0	0	0
ENSMUSG00000055717	14	17	33	18	12	29	60	26	66	26
ENSMUSG00000086588	0	3	0	3	0	0	0	9	5	0
ENSMUSG00000112141	6	3	5	3	0	15	6	0	4	6
ENSMUSG00000112143	0	0	0	0	0	0	0	0	0	0
ENSMUSG00000112142	0	0	0	0	3	0	2	9	0	8
ENSMUSG00000086580	0	0	0	0	0	0	0	0	0	0
ENSMUSG00000086587	82	95	73	166	106	96	242	184	96	114
ENSMUSG00000086055	0	0	0	0	0	0	0	0	0	0
ENSMUSG00000086057	0	0	0	0	0	0	0	0	0	0
ENSMUSG00000086056	0	0	0	0	0	0	0	0	0	0
ENSMUSG00000049593	0	0	0	0	0	0	0	0	0	0
ENSMUSG00000086052	0	0	0	0	0	0	0	0	0	3
ENSMUSG00000049598	2	8	21	10	19	4	7	13	5	21
ENSMUSG00000111437	0	0	0	0	0	0	0	0	0	0
ENSMUSG00000057037	0	0	0	0	0	0	0	0	0	0
ENSMUSG00000099466	0	0	0	0	0	0	0	0	0	0
ENSMUSG00000111430	0	0	0	0	0	0	0	0	0	0
ENSMUSG00000098966	1	0	0	0	0	0	0	0	1	0
ENSMUSG00000098964	0	0	0	0	0	0	0	0	0	0
ENSMUSG00000098965	0	0	1	0	0	0	0	0	0	0
ENSMUSG00000098962	0	0	0	0	0	0	0	0	0	0
ENSMUSG00000098963	0	0	0	0	0	0	0	0	0	0
ENSMUSG00000098960	0	0	0	0	0	0	0	0	0	0
ENSMUSG00000098961	0	0	0	0	0	0	0	0	0	0
ENSMUSG00000082775	0	0	0	0	0	0	0	0	0	0
ENSMUSG00000082776	6	8	3	0	10	0	12	3	12	15

ENSMUSG00000082777	0	0	0	0	0	0	0	0	0	0
ENSMUSG00000082770	0	0	0	0	0	0	0	0	0	0
ENSMUSG00000082771	0	2	6	2	0	5	3	0	3	3
ENSMUSG00000098968	0	0	0	0	0	0	0	0	0	0
ENSMUSG00000082773	0	0	0	0	0	0	0	0	0	0
ENSMUSG00000113235	0	0	0	0	0	0	0	0	0	0
ENSMUSG00000113236	0	0	0	0	0	0	0	0	0	0
ENSMUSG00000113230	0	0	0	0	0	0	0	0	0	0
ENSMUSG00000113231	0	0	0	0	0	0	3	0	0	0
ENSMUSG00000084536	0	0	0	0	0	0	0	0	0	0
ENSMUSG00000113233	0	0	0	0	0	0	0	0	0	0
ENSMUSG00000101596	0	0	0	0	0	0	0	0	0	0
ENSMUSG00000113238	24	8	20	20	17	26	23	37	19	16
ENSMUSG00000114404	0	0	0	0	0	0	3	0	2	0
ENSMUSG00000101594	0	0	0	0	0	0	0	0	0	0
ENSMUSG00000079380	0	3	3	1	0	0	8	4	0	0
ENSMUSG00000070960	16	21	9	25	22	31	23	39	42	33
ENSMUSG00000040264	239	35	76	532	152	22	196	91	226	101
ENSMUSG00000092433	0	0	0	0	0	0	0	0	0	0
ENSMUSG00000114890	0	0	0	0	0	0	0	0	0	0
ENSMUSG00000084532	0	0	0	0	0	0	0	0	0	0
ENSMUSG00000085497	0	0	2	4	2	5	0	3	2	5
ENSMUSG00000048534	111	32	89	266	124	182	281	129	220	87
ENSMUSG00000084341	1	0	0	0	0	0	0	0	0	0
ENSMUSG00000084346	0	0	0	0	0	0	0	0	0	0
ENSMUSG00000084344	0	0	0	0	0	0	0	0	0	0
ENSMUSG00000084348	0	0	0	2	2	1	1	0	0	0
ENSMUSG00000025528	0	0	0	0	0	0	3	0	3	0
ENSMUSG00000025529	42	50	36	24	18	44	76	90	53	58
ENSMUSG00000025527	0	0	0	3	0	0	2	4	0	0
ENSMUSG00000070385	3	0	0	0	0	3	3	3	0	6
ENSMUSG00000070380	0	0	0	0	0	0	0	0	0	0
ENSMUSG00000099379	0	0	0	0	0	0	0	0	0	0
ENSMUSG00000070382	0	0	0	0	0	0	0	0	0	0
ENSMUSG00000070383	0	0	0	0	0	0	0	0	0	0
ENSMUSG00000099374	0	0	0	0	0	0	0	0	0	0
ENSMUSG00000099375	52	63	41	44	21	70	94	142	85	83
ENSMUSG00000099376	0	0	0	0	0	0	0	0	0	0
ENSMUSG00000070388	9	13	3	15	0	0	3	8	3	6
ENSMUSG00000099371	0	0	0	0	0	0	0	0	0	0
ENSMUSG00000099372	0	0	0	0	0	0	0	0	0	0
ENSMUSG00000078586	0	0	0	0	0	0	0	0	0	0
ENSMUSG00000075604	0	0	0	0	0	0	0	0	0	0
ENSMUSG00000079186	3	0	0	0	0	0	6	3	0	0
ENSMUSG00000111633	3	3	0	3	3	0	0	6	0	0
ENSMUSG00000030463	3	0	0	0	0	0	0	0	0	0
ENSMUSG00000075600	284	272	354	463	300	387	387	382	375	392
ENSMUSG00000065630	0	0	0	0	0	0	0	0	0	0
ENSMUSG00000100178	0	0	0	0	0	0	0	0	0	0
ENSMUSG00000065636	0	0	0	0	0	0	0	0	0	0
ENSMUSG00000065635	0	0	0	0	0	0	0	0	0	0
ENSMUSG00000075067	0	0	0	0	0	0	0	0	0	0
ENSMUSG00000080046	0	0	0	0	0	0	0	0	0	0
ENSMUSG00000100172	0	0	0	0	0	0	0	0	0	0
ENSMUSG00000062061	2	0	0	0	0	3	6	0	3	0
ENSMUSG00000062064	0	0	0	0	0	0	0	4	0	0
ENSMUSG00000033882	7	9	6	10	7	6	13	3	0	0
ENSMUSG00000073601	0	0	0	0	0	0	0	0	0	0

ENSMUSG00000073602	0	0	0	0	0	0	0	0	0	0
ENSMUSG00000112765	3	6	0	9	0	16	10	19	16	9
ENSMUSG00000095985	0	0	0	0	0	0	0	0	0	0
ENSMUSG00000055134	54	65	93	49	71	79	60	61	95	101
ENSMUSG00000054457	0	0	0	0	0	0	0	0	0	0
ENSMUSG00000111997	7	19	16	27	16	10	25	25	10	6
ENSMUSG00000111996	0	0	0	0	0	0	0	0	0	0
ENSMUSG00000109058	0	0	0	0	0	0	0	0	0	0
ENSMUSG00000090634	0	8	0	0	3	0	2	0	3	0
ENSMUSG00000054453	29	15	15	42	20	25	88	15	31	14
ENSMUSG00000098132	187	214	227	129	149	239	387	379	377	329
ENSMUSG00000092374	3	0	3	0	0	0	0	0	0	2
ENSMUSG00000098139	0	0	0	0	0	0	0	0	0	0
ENSMUSG00000109054	29	16	24	15	3	15	32	35	31	20
ENSMUSG00000084731	0	0	0	0	0	0	0	0	0	0
ENSMUSG00000111991	0	0	0	0	0	0	0	0	0	0
ENSMUSG00000112769	0	0	0	0	0	0	0	0	0	0
ENSMUSG00000035831	0	0	0	0	0	0	0	0	0	0
ENSMUSG00000109050	0	0	0	0	0	0	0	0	0	0
ENSMUSG00000048616	36	22	15	14	21	15	6	16	21	3
ENSMUSG00000072955	24	19	18	14	11	13	68	32	21	30
ENSMUSG00000072952	0	12	0	6	9	0	11	6	6	0
ENSMUSG00000072950	0	0	0	0	0	0	0	0	0	0
ENSMUSG00000103911	0	0	0	0	0	0	0	0	0	1
ENSMUSG00000103912	0	0	0	0	0	0	0	0	0	0
ENSMUSG00000103913	0	0	0	0	0	0	0	0	0	0
ENSMUSG00000103914	0	0	0	0	0	0	0	0	0	0
ENSMUSG00000103915	0	0	0	0	0	0	0	0	0	0
ENSMUSG00000103917	0	0	0	0	0	0	0	3	0	0
ENSMUSG00000103918	0	0	0	0	0	0	0	0	0	0
ENSMUSG00000103919	0	0	0	0	0	0	0	0	0	0
ENSMUSG00000100142	0	0	0	0	0	0	0	0	0	0
ENSMUSG00000058295	0	0	0	0	0	0	0	0	0	0
ENSMUSG00000038973	2	5	3	5	2	2	10	1	0	0
ENSMUSG00000032191	2	0	9	0	0	7	11	0	0	6
ENSMUSG00000084734	0	0	0	0	0	0	0	0	0	0
ENSMUSG00000057370	5	2	3	8	5	2	5	3	5	0
ENSMUSG00000076994	0	0	0	0	0	0	0	0	0	0
ENSMUSG00000089876	30	55	39	46	32	37	74	59	44	60
ENSMUSG00000089874	2	0	0	0	0	3	3	0	0	0
ENSMUSG00000089189	0	0	0	0	0	0	0	0	0	0
ENSMUSG00000089871	0	0	0	0	0	0	0	0	0	0
ENSMUSG00000111175	0	0	0	0	0	0	0	0	0	0
ENSMUSG00000089879	0	0	0	0	0	3	0	0	0	0
ENSMUSG00000091402	11	6	0	0	5	0	6	4	0	0
ENSMUSG00000091400	0	0	0	0	0	0	0	0	0	0
ENSMUSG00000098662	1	1	2	2	1	1	1	1	1	1
ENSMUSG00000110697	13	16	21	25	19	26	51	20	26	27
ENSMUSG00000089184	0	1	1	1	0	0	2	2	0	2
ENSMUSG00000110696	30	34	17	35	33	30	69	45	65	51
ENSMUSG00000111177	0	0	0	0	0	0	0	0	0	3
ENSMUSG00000110694	0	0	0	0	0	0	0	0	0	0
ENSMUSG00000059663	0	0	0	0	0	0	0	0	0	0
ENSMUSG00000000182	3	18	6	8	9	11	23	0	14	0
ENSMUSG00000108828	0	0	0	0	0	0	0	0	0	0
ENSMUSG00000108824	0	0	0	0	0	0	0	0	0	0
ENSMUSG00000108827	0	0	0	0	0	0	0	0	0	0
ENSMUSG00000108821	0	0	0	0	0	0	0	0	0	0

ENSMUSG00000108820	3	3	6	0	6	2	6	0	3	9
ENSMUSG00000108822	0	0	0	0	0	0	0	0	0	0
ENSMUSG00000043219	3	0	6	0	3	0	22	3	17	6
ENSMUSG00000067543	0	0	0	0	0	0	0	0	0	0
ENSMUSG00000067541	0	0	3	0	0	0	0	0	3	0
ENSMUSG00000067545	0	1	0	0	0	0	0	0	0	0
ENSMUSG00000100392	0	0	0	0	3	0	12	0	0	7
ENSMUSG00000087083	0	0	0	0	0	6	0	0	0	0
ENSMUSG00000100390	0	0	0	0	0	0	0	0	0	0
ENSMUSG00000087085	0	0	0	0	0	0	0	0	0	0
ENSMUSG00000100396	9	0	6	4	3	18	43	8	8	9
ENSMUSG00000100395	0	0	0	0	0	0	0	0	0	0
ENSMUSG00000087089	0	0	0	0	0	0	0	0	0	0
ENSMUSG00000100399	0	0	0	0	0	0	0	0	0	0
ENSMUSG00000100398	0	0	0	0	0	0	0	0	0	0
ENSMUSG00000088052	0	0	0	0	0	0	0	0	0	0
ENSMUSG00000088051	0	0	0	0	0	0	0	0	0	0
ENSMUSG00000088050	0	0	0	0	0	0	0	0	0	0
ENSMUSG00000101668	0	0	0	0	0	0	0	0	0	0
ENSMUSG00000101669	0	0	0	0	0	0	0	0	0	0
ENSMUSG00000101661	0	0	0	0	0	0	0	0	0	0
ENSMUSG00000101664	0	0	0	0	0	0	0	0	0	0
ENSMUSG00000101665	0	0	0	0	0	0	0	0	0	0
ENSMUSG00000060017	0	0	0	0	0	0	0	0	0	0
ENSMUSG00000101667	0	0	0	0	0	0	0	0	0	0
ENSMUSG00000037994	14	17	14	16	14	50	109	51	75	35
ENSMUSG00000085820	0	0	0	0	0	0	0	0	0	0
ENSMUSG00000085822	0	0	0	0	0	0	0	0	0	0
ENSMUSG00000037991	48	15	12	68	45	18	58	56	44	48
ENSMUSG00000085827	0	0	0	0	0	0	3	0	0	0
ENSMUSG00000108907	0	0	0	0	0	0	0	0	0	0
ENSMUSG00000033061	0	0	0	0	0	0	0	0	0	0
ENSMUSG00000033063	0	6	0	0	0	6	12	0	8	0
ENSMUSG00000084076	0	0	0	0	0	0	0	0	0	0
ENSMUSG00000087559	0	0	0	0	0	0	0	0	0	0
ENSMUSG00000110439	0	0	0	0	0	0	0	0	0	0
ENSMUSG00000087553	0	0	0	0	0	0	0	0	0	0
ENSMUSG00000114656	3	0	16	0	0	0	9	6	11	3
ENSMUSG00000087550	0	0	0	0	0	0	0	0	0	0
ENSMUSG00000013643	3	9	0	0	0	3	0	9	0	0
ENSMUSG00000040749	200	150	140	172	151	171	192	194	206	220
ENSMUSG00000103288	0	0	0	0	0	0	0	0	0	0
ENSMUSG00000103289	0	3	0	0	0	0	0	0	0	0
ENSMUSG00000103280	5	9	14	0	3	11	5	15	7	13
ENSMUSG00000103282	0	0	0	0	0	0	0	0	0	0
ENSMUSG00000103287	0	0	0	0	0	0	0	0	0	0
ENSMUSG00000036110	0	0	0	0	0	0	0	3	0	0
ENSMUSG00000103136	0	0	0	4	0	0	11	0	0	0
ENSMUSG00000105624	8	6	10	5	2	11	7	3	8	21
ENSMUSG00000105625	0	0	0	0	0	0	0	0	0	0
ENSMUSG00000100537	0	0	0	0	0	0	0	0	0	0
ENSMUSG00000100535	0	0	0	0	0	0	0	0	0	0
ENSMUSG00000100534	0	0	0	0	0	0	0	0	3	0
ENSMUSG00000100533	0	0	0	0	0	0	0	0	0	0
ENSMUSG00000100532	0	0	0	0	0	0	0	0	0	0
ENSMUSG00000100531	0	0	0	0	0	0	0	0	0	0
ENSMUSG00000100530	0	0	0	0	0	0	0	0	0	0
ENSMUSG00000105627	0	0	0	0	0	0	0	3	0	0

ENSMUSG00000100539	0	0	0	0	0	0	0	0	0	0
ENSMUSG00000100538	0	0	0	0	0	0	0	0	0	0
ENSMUSG00000064223	0	0	0	0	0	0	0	0	0	0
ENSMUSG00000064224	0	0	0	0	0	0	0	0	0	0
ENSMUSG00000107622	0	6	5	0	0	5	11	5	3	12
ENSMUSG00000107623	0	0	0	0	0	0	0	0	0	0
ENSMUSG00000107620	6	3	3	0	1	2	3	3	0	1
ENSMUSG00000084072	0	0	0	0	0	0	0	0	0	0
ENSMUSG00000107625	0	0	0	0	0	0	0	0	0	0
ENSMUSG00000095879	0	0	0	0	0	0	0	0	0	0
ENSMUSG00000095878	0	0	0	0	0	0	0	1	0	0
ENSMUSG00000095874	0	0	0	0	0	0	0	0	0	0
ENSMUSG00000095873	0	0	0	0	0	0	0	0	0	0
ENSMUSG00000095872	0	0	0	0	0	0	0	0	0	0
ENSMUSG00000095870	0	0	0	0	0	0	0	0	0	0
ENSMUSG00000065577	0	0	0	1	0	0	0	0	0	1
ENSMUSG00000111179	0	0	0	0	0	0	0	0	0	0
ENSMUSG00000089018	0	0	0	0	0	0	0	0	0	0
ENSMUSG00000089019	0	0	0	0	0	0	0	0	0	0
ENSMUSG00000100135	0	0	0	0	0	0	0	0	0	0
ENSMUSG00000077127	0	0	0	0	0	0	0	0	0	0
ENSMUSG00000089016	0	0	0	0	0	0	0	0	0	0
ENSMUSG00000089017	0	0	0	0	0	0	0	0	0	0
ENSMUSG00000028602	6	3	8	9	12	9	45	21	14	21
ENSMUSG00000112303	0	0	8	3	0	6	8	0	0	0
ENSMUSG00000102663	0	0	0	0	0	0	0	0	0	0
ENSMUSG00000112300	16	23	12	32	3	22	17	12	42	22
ENSMUSG00000112307	24	6	15	3	21	15	22	24	8	16
ENSMUSG00000112306	0	0	0	0	0	0	0	0	0	0
ENSMUSG00000112305	0	0	0	0	0	0	0	0	0	0
ENSMUSG00000102668	0	0	0	0	0	0	0	0	0	0
ENSMUSG00000112309	6	13	18	16	6	0	13	11	22	5
ENSMUSG00000036800	0	0	0	0	0	0	0	0	0	0
ENSMUSG00000036805	0	0	0	0	0	0	0	0	0	0
ENSMUSG00000110235	25	56	70	27	23	15	61	48	48	86
ENSMUSG00000110236	0	0	0	0	0	0	0	0	0	0
ENSMUSG00000110231	0	5	0	0	0	0	0	0	0	0
ENSMUSG00000110232	0	0	0	0	0	0	0	0	0	0
ENSMUSG00000114080	0	0	0	0	0	0	0	0	0	0
ENSMUSG00000084078	0	0	0	0	0	0	0	0	0	0
ENSMUSG00000043102	37	20	26	26	24	39	27	37	21	36
ENSMUSG00000105570	0	0	0	0	0	0	3	3	0	0
ENSMUSG00000105571	0	0	0	0	0	0	0	0	0	0
ENSMUSG00000039785	0	0	0	0	0	0	0	0	0	0
ENSMUSG00000105573	0	0	0	0	0	0	0	0	0	0
ENSMUSG00000105575	0	0	0	0	0	0	0	0	0	0
ENSMUSG00000105576	0	0	0	0	0	0	0	0	1	0
ENSMUSG00000105577	0	0	0	0	0	0	0	0	0	0
ENSMUSG00000105578	0	0	2	0	0	0	0	2	0	0
ENSMUSG00000105579	0	0	7	3	0	3	0	0	3	0
ENSMUSG00000101381	0	0	0	0	0	0	0	0	0	0
ENSMUSG00000088868	0	0	0	0	0	0	0	0	0	0
ENSMUSG00000088867	0	0	0	0	0	0	0	0	0	0
ENSMUSG00000088864	0	0	0	0	0	0	0	0	0	0
ENSMUSG00000088865	0	0	0	0	0	0	0	0	0	0
ENSMUSG00000088862	0	0	0	0	0	0	0	0	0	0
ENSMUSG00000088863	0	0	0	0	0	0	0	0	0	0
ENSMUSG00000031340	86	71	111	51	57	119	154	132	146	126

ENSMUSG00000088861	0	0	0	0	0	0	0	0	0	0
ENSMUSG00000094838	0	0	0	0	0	0	0	0	0	0
ENSMUSG00000108016	0	0	0	0	0	0	2	0	0	0
ENSMUSG00000016458	182	255	280	270	198	223	499	409	297	216
ENSMUSG00000021721	0	0	0	0	0	0	0	0	0	0
ENSMUSG00000094833	0	0	0	0	0	0	0	0	0	0
ENSMUSG00000094834	0	0	0	0	0	0	0	0	0	0
ENSMUSG00000094836	0	0	0	0	0	0	0	0	0	0
ENSMUSG00000094837	0	0	0	0	0	0	0	0	0	0
ENSMUSG00000100542	0	0	0	0	0	0	0	0	0	0
ENSMUSG00000111549	0	0	0	0	0	0	0	0	0	0
ENSMUSG00000111544	0	0	0	0	3	0	0	0	0	0
ENSMUSG00000111546	0	0	0	0	0	0	7	0	0	5
ENSMUSG00000111541	0	0	0	0	0	0	0	0	0	0
ENSMUSG00000111543	0	0	0	0	0	0	0	0	0	0
ENSMUSG00000101387	0	0	3	0	0	0	1	0	0	0
ENSMUSG00000029268	0	0	0	0	0	0	0	0	0	0
ENSMUSG00000005503	0	0	0	0	0	0	0	0	0	0
ENSMUSG00000050876	0	3	0	0	0	0	0	0	0	0
ENSMUSG00000050870	0	0	0	0	0	0	0	0	0	0
ENSMUSG00000038782	23	18	22	16	25	24	11	28	25	28
ENSMUSG00000058935	3	0	6	3	0	3	0	0	0	3
ENSMUSG00000059397	0	0	0	0	0	0	0	0	0	0
ENSMUSG00000059395	48	33	35	47	43	67	69	58	76	30
ENSMUSG00000109835	0	0	0	0	0	0	6	0	0	0
ENSMUSG00000109834	0	0	0	0	0	0	0	0	0	0
ENSMUSG00000114079	0	0	0	0	0	0	0	0	0	0
ENSMUSG00000114078	0	0	0	0	0	0	0	0	0	0
ENSMUSG00000105390	0	0	0	0	0	0	0	0	0	0
ENSMUSG00000109830	0	0	0	0	0	0	0	0	0	0
ENSMUSG00000109833	0	0	0	0	0	0	0	0	0	0
ENSMUSG00000109832	0	0	0	0	0	0	0	0	0	0
ENSMUSG00000114073	15	24	24	61	27	100	18	120	52	66
ENSMUSG00000114071	0	0	0	0	0	0	0	0	0	0
ENSMUSG00000109839	0	0	0	0	0	0	0	0	0	0
ENSMUSG00000109838	0	0	0	0	0	0	0	0	0	0
ENSMUSG00000114075	0	0	0	0	0	0	0	0	0	0
ENSMUSG00000114074	0	0	0	0	0	0	0	0	0	0
ENSMUSG00000059648	0	0	0	0	0	0	0	2	0	0
ENSMUSG00000047969	0	0	0	0	0	0	0	0	0	0
ENSMUSG00000097563	0	0	0	0	0	0	0	0	0	3
ENSMUSG00000097562	0	0	0	0	0	0	0	0	0	0
ENSMUSG00000104628	0	0	0	0	0	0	0	0	0	0
ENSMUSG00000104626	3	0	1	3	0	3	3	3	6	3
ENSMUSG00000047960	0	0	0	0	0	0	0	0	0	0
ENSMUSG00000059645	0	0	3	0	0	0	5	3	0	0
ENSMUSG00000097569	18	21	30	25	13	21	43	13	21	24
ENSMUSG00000067064	0	0	0	0	0	0	0	0	0	0
ENSMUSG00000112899	0	0	0	0	0	0	0	0	0	0
ENSMUSG00000112898	0	0	0	0	0	0	0	0	0	0
ENSMUSG00000112893	0	0	0	0	0	0	0	1	0	0
ENSMUSG00000112892	0	0	0	0	0	3	0	0	0	0
ENSMUSG00000112891	29	21	29	43	20	36	53	54	56	33
ENSMUSG00000112890	0	0	0	0	0	0	0	0	0	0
ENSMUSG00000112896	0	0	0	0	0	0	0	0	0	0
ENSMUSG00000027761	0	0	0	3	8	4	3	0	0	0
ENSMUSG00000086677	0	15	3	8	9	0	15	6	12	6
ENSMUSG00000083889	0	0	0	0	0	0	0	0	0	0

ENSMUSG00000083888	0	0	0	0	0	0	0	0	0	0
ENSMUSG00000083884	0	0	0	0	0	0	0	0	0	0
ENSMUSG00000083886	0	0	0	0	0	0	0	0	0	0
ENSMUSG00000083882	0	0	0	0	0	0	0	0	0	0
ENSMUSG00000057592	0	0	0	0	0	0	0	0	0	0
ENSMUSG00000113565	0	0	0	0	0	0	2	0	1	0
ENSMUSG00000110792	0	0	0	0	0	0	0	0	0	0
ENSMUSG00000110793	0	0	0	0	0	0	0	0	0	0
ENSMUSG00000058114	0	0	0	0	0	0	0	0	0	0
ENSMUSG00000025892	87	80	60	20	49	88	81	37	50	30
ENSMUSG00000110794	0	2	4	4	1	6	2	6	0	3
ENSMUSG00000110795	0	0	0	0	3	0	3	3	6	0
ENSMUSG00000110799	0	0	0	0	0	0	0	0	0	0
ENSMUSG00000058119	0	0	0	0	0	0	0	0	0	0
ENSMUSG00000069668	0	0	3	0	0	0	0	0	0	0
ENSMUSG00000027490	112	57	97	95	86	87	208	97	97	93
ENSMUSG00000080283	0	0	0	0	0	1	0	0	0	3
ENSMUSG00000046367	0	0	0	0	0	0	0	0	0	0
ENSMUSG00000056643	0	0	0	0	0	0	0	3	3	0
ENSMUSG00000082313	3	9	2	8	0	0	2	6	3	3
ENSMUSG00000053030	0	9	6	5	6	9	36	0	6	11
ENSMUSG00000048652	117	99	138	117	64	108	94	93	92	91
ENSMUSG00000111381	0	0	0	0	0	0	0	0	0	0
ENSMUSG00000090102	0	0	0	0	0	0	0	0	0	0
ENSMUSG00000090109	0	0	0	0	0	0	0	0	0	0
ENSMUSG00000098212	0	0	0	0	0	0	0	0	0	3
ENSMUSG00000113854	3	0	3	0	2	2	0	0	0	0
ENSMUSG00000113855	0	0	0	0	0	0	0	0	0	0
ENSMUSG00000113856	6	13	8	4	6	2	26	22	19	29
ENSMUSG00000098330	0	0	1	0	0	0	0	0	0	0
ENSMUSG00000113850	0	0	0	0	0	0	0	0	0	0
ENSMUSG00000098335	0	0	0	0	0	0	0	0	0	0
ENSMUSG00000113858	0	0	0	0	0	0	0	0	0	0
ENSMUSG00000113859	0	0	0	0	0	0	3	0	0	0
ENSMUSG00000053695	0	0	0	0	0	0	0	0	0	0
ENSMUSG00000093929	0	0	0	0	0	0	0	0	0	0
ENSMUSG00000094481	0	0	0	0	0	0	0	0	0	0
ENSMUSG00000094485	0	0	0	0	0	0	0	0	0	0
ENSMUSG00000094484	0	0	0	0	0	0	0	0	0	0
ENSMUSG00000093458	0	0	0	0	0	0	0	0	0	0
ENSMUSG00000094489	0	0	0	0	0	0	0	0	0	0
ENSMUSG00000093453	3	0	1	0	0	0	0	0	0	0
ENSMUSG00000045471	6	0	0	0	0	0	0	0	0	0
ENSMUSG00000045474	0	0	0	0	0	0	0	0	0	0
ENSMUSG00000045475	0	0	0	0	0	0	0	0	0	0
ENSMUSG00000095684	0	0	0	0	0	0	0	0	0	0
ENSMUSG00000095685	0	0	0	0	0	0	0	0	0	0
ENSMUSG00000095686	0	0	0	0	0	0	0	0	0	0
ENSMUSG00000045479	0	0	0	0	0	0	0	0	0	0
ENSMUSG00000095681	35	41	32	48	30	75	25	37	43	33
ENSMUSG00000095683	0	0	0	0	0	0	0	0	0	0
ENSMUSG00000098485	0	0	0	0	0	0	0	0	0	0
ENSMUSG00000047189	0	0	0	0	0	0	0	0	0	0
ENSMUSG00000053961	0	0	0	0	0	0	0	0	0	0
ENSMUSG00000100460	0	0	0	0	0	0	0	0	0	0
ENSMUSG00000094159	1	0	1	1	0	0	0	2	0	1
ENSMUSG00000088753	0	0	0	0	0	0	0	0	0	0
ENSMUSG00000042124	0	0	0	0	0	0	0	0	0	0

ENSMUSG00000094152	0	0	0	0	0	0	8	0	0	0
ENSMUSG00000094157	0	0	0	0	0	0	0	0	0	0
ENSMUSG00000086936	0	0	0	0	0	0	0	0	0	0
ENSMUSG00000017195	9	0	0	3	0	4	4	0	7	6
ENSMUSG00000100463	0	0	0	0	0	0	0	0	0	0
ENSMUSG00000081628	0	0	0	0	0	0	0	0	0	0
ENSMUSG00000081623	0	0	0	0	0	0	0	0	0	0
ENSMUSG00000081622	7	8	5	9	12	16	16	13	15	20
ENSMUSG00000081626	0	0	0	0	0	0	0	0	0	0
ENSMUSG00000081625	0	0	0	0	0	0	0	0	0	0
ENSMUSG00000081624	0	0	0	0	0	0	0	0	0	0
ENSMUSG00000100915	0	0	0	0	0	0	0	0	0	0
ENSMUSG00000100462	0	0	0	0	0	0	0	0	0	0
ENSMUSG00000093817	0	0	0	0	0	0	0	0	0	0
ENSMUSG00000077707	0	0	0	0	0	0	0	0	0	0
ENSMUSG00000077706	0	0	0	0	0	0	0	0	0	0
ENSMUSG00000077701	0	0	0	0	0	0	0	0	0	0
ENSMUSG00000077700	0	0	0	0	0	0	0	0	0	0
ENSMUSG00000077703	0	0	0	0	0	0	0	0	0	0
ENSMUSG00000100914	0	0	0	0	0	0	0	0	0	0
ENSMUSG00000093814	0	0	0	0	0	0	0	0	0	0
ENSMUSG00000044985	0	0	0	0	0	0	0	0	0	0
ENSMUSG00000090816	0	0	0	0	0	0	0	2	0	0
ENSMUSG00000044988	0	0	0	0	0	0	0	0	0	0
ENSMUSG00000100917	0	0	0	0	0	0	0	0	0	0
ENSMUSG00000113261	1	3	1	4	1	3	1	2	0	1
ENSMUSG00000092419	0	0	0	0	0	0	0	0	0	0
ENSMUSG00000111840	5	9	0	0	3	10	161	15	67	78
ENSMUSG00000094748	0	0	0	0	0	0	0	0	0	0
ENSMUSG00000048388	206	223	176	169	145	299	574	315	398	412
ENSMUSG00000092410	0	0	0	0	0	0	0	0	0	0
ENSMUSG00000092411	0	0	0	0	0	0	0	0	0	0
ENSMUSG00000048385	0	3	0	0	0	0	0	0	6	3
ENSMUSG00000092413	3	0	0	0	0	0	0	0	0	0
ENSMUSG00000092414	0	0	0	0	0	0	0	0	0	0
ENSMUSG00000094197	0	0	0	0	0	0	0	0	0	0
ENSMUSG00000094747	0	0	0	0	0	0	0	0	0	0
ENSMUSG00000113264	0	0	0	0	0	0	0	0	0	0
ENSMUSG00000093536	5	5	9	9	4	5	17	12	8	5
ENSMUSG00000003545	61	70	56	43	52	95	258	152	200	163
ENSMUSG00000051860	0	0	0	0	0	3	1	0	0	0
ENSMUSG00000055523	12	10	8	15	10	24	30	24	17	20
ENSMUSG00000093530	0	0	0	0	0	0	0	0	0	0
ENSMUSG00000043456	398	339	305	377	283	357	226	406	322	373
ENSMUSG00000030775	3	0	3	0	0	3	20	0	0	3
ENSMUSG00000093533	0	0	0	0	0	0	0	0	0	0
ENSMUSG00000112767	0	0	0	0	0	0	0	0	0	0
ENSMUSG00000072754	0	0	0	0	0	0	0	0	0	0
ENSMUSG00000072753	0	0	0	0	0	0	0	0	0	0
ENSMUSG00000062496	0	0	0	0	0	0	3	0	0	0
ENSMUSG00000071481	0	0	0	0	0	0	0	0	0	0
ENSMUSG00000043982	0	0	0	0	0	0	0	0	0	0
ENSMUSG00000049387	14	12	11	0	11	5	12	9	0	5
ENSMUSG00000100913	0	0	0	0	0	0	0	2	0	0
ENSMUSG00000043986	0	0	0	0	0	3	0	0	3	0
ENSMUSG00000112761	0	3	0	6	0	0	4	0	0	9
ENSMUSG00000111844	6	0	3	3	2	3	3	3	3	8
ENSMUSG00000087718	0	0	0	0	0	0	0	0	0	0

ENSMUSG00000085864	0	0	0	0	0	0	0	0	0	0
ENSMUSG00000087710	0	0	0	0	1	0	0	0	0	3
ENSMUSG00000087712	0	0	0	0	0	0	0	0	0	0
ENSMUSG00000087715	0	0	0	0	0	2	0	0	0	0
ENSMUSG00000087717	0	0	0	0	0	0	0	0	0	0
ENSMUSG00000087716	0	0	0	0	0	0	0	0	0	0
ENSMUSG00000040187	221	171	293	128	191	203	304	327	353	222
ENSMUSG00000040183	322	272	335	289	277	366	425	422	351	400
ENSMUSG00000037202	33	12	33	87	16	44	58	34	54	16
ENSMUSG00000083417	0	0	0	0	0	0	0	0	0	0
ENSMUSG00000083414	0	0	0	0	0	0	0	0	0	0
ENSMUSG00000049036	9	9	0	12	3	3	6	0	14	11
ENSMUSG00000083419	0	0	0	0	0	0	0	0	0	0
ENSMUSG00000079577	0	0	0	0	0	0	0	0	0	0
ENSMUSG00000079578	0	0	0	0	0	0	0	0	0	0
ENSMUSG00000079579	0	0	0	0	0	0	0	0	0	0
ENSMUSG00000031919	0	0	0	4	0	2	8	0	0	0
ENSMUSG00000107421	0	0	0	0	0	0	0	0	0	0
ENSMUSG00000107423	0	0	0	0	0	0	0	0	0	0
ENSMUSG00000107424	0	0	0	0	0	0	0	0	0	0
ENSMUSG00000107425	0	0	0	0	0	0	0	0	0	0
ENSMUSG00000071229	3	3	0	0	0	0	3	0	0	0
ENSMUSG00000089678	0	0	9	12	0	0	0	0	0	2
ENSMUSG00000061210	0	0	0	0	0	0	0	0	0	0
ENSMUSG00000105817	0	0	0	0	0	0	0	0	0	0
ENSMUSG00000063434	3	3	3	3	0	0	12	0	0	0
ENSMUSG00000102848	0	0	0	0	0	0	0	0	0	0
ENSMUSG00000065771	0	0	0	0	0	0	0	0	0	0
ENSMUSG00000065770	0	0	0	0	0	0	0	0	0	0
ENSMUSG00000085712	4	0	0	0	0	0	0	0	0	0
ENSMUSG00000085717	0	0	0	0	0	0	0	0	0	0
ENSMUSG00000085715	15	6	39	14	9	21	3	25	14	18
ENSMUSG00000064793	0	0	0	0	0	0	0	0	0	0
ENSMUSG00000087316	0	0	0	0	0	0	0	0	0	0
ENSMUSG00000109055	0	0	0	2	0	0	0	2	0	0
ENSMUSG00000107398	0	0	0	0	0	0	0	0	0	0
ENSMUSG00000107399	0	0	0	0	0	0	0	0	0	0
ENSMUSG00000107392	0	0	0	0	0	0	0	0	0	0
ENSMUSG00000107390	6	29	30	33	9	27	55	11	19	32
ENSMUSG00000107396	0	0	0	0	0	0	0	0	0	0
ENSMUSG00000107397	0	0	0	0	0	0	6	0	0	0
ENSMUSG00000107394	0	0	0	0	0	0	0	0	0	0
ENSMUSG00000082722	0	0	0	0	0	0	0	0	0	0
ENSMUSG00000082721	0	0	0	0	0	0	0	0	0	0
ENSMUSG00000079395	0	0	0	0	0	0	0	0	0	0
ENSMUSG00000079396	7	23	5	6	6	21	20	25	15	20
ENSMUSG00000068078	0	0	0	3	0	0	0	0	0	0
ENSMUSG00000079391	2	5	8	7	5	10	15	13	12	0
ENSMUSG00000075833	0	1	0	0	0	1	0	2	0	0
ENSMUSG00000075832	0	0	0	0	0	0	0	0	0	0
ENSMUSG00000068073	0	0	0	0	0	0	0	0	0	0
ENSMUSG00000068071	0	0	0	0	0	0	0	0	0	0
ENSMUSG00000068075	0	0	0	0	0	0	0	0	0	0
ENSMUSG00000068074	0	0	0	0	0	0	0	0	0	0
ENSMUSG00000093243	0	1	0	0	0	0	1	0	0	0
ENSMUSG00000107904	0	0	0	0	0	0	0	0	0	0
ENSMUSG00000093934	0	0	0	0	0	0	0	0	0	0
ENSMUSG00000082725	0	0	0	0	0	0	0	0	0	0

ENSMUSG00000078627	3	4	0	6	6	0	6	0	2	3
ENSMUSG00000078626	0	0	0	0	0	0	0	0	0	0
ENSMUSG00000078625	0	0	0	0	0	0	0	0	0	0
ENSMUSG00000078624	0	0	0	0	0	0	0	0	0	0
ENSMUSG00000078621	0	0	0	0	0	0	0	0	0	0
ENSMUSG00000059205	0	0	0	0	0	0	0	0	0	0
ENSMUSG00000089117	0	0	0	0	0	0	0	0	0	0
ENSMUSG00000006784	16	30	32	144	28	22	29	57	33	36
ENSMUSG00000056219	0	0	0	0	0	0	2	0	0	0
ENSMUSG00000032327	53	52	71	27	27	48	77	102	70	52
ENSMUSG00000032323	3	3	6	9	3	22	37	9	11	0
ENSMUSG00000062296	18	3	11	27	0	3	64	9	11	21
ENSMUSG00000086699	0	0	0	0	0	0	0	0	0	0
ENSMUSG00000102278	2	0	3	6	0	6	0	0	3	0
ENSMUSG00000102279	0	0	0	0	0	0	0	0	0	0
ENSMUSG00000102272	0	0	0	0	0	0	0	0	0	0
ENSMUSG00000102273	0	0	0	0	0	0	0	0	0	0
ENSMUSG00000102271	0	0	6	15	5	0	3	6	9	3
ENSMUSG00000102276	0	0	0	0	0	0	0	0	0	0
ENSMUSG00000102277	7	3	32	16	22	4	21	22	10	19
ENSMUSG00000102274	0	0	0	0	0	0	0	0	0	0
ENSMUSG00000103895	0	5	0	0	0	0	6	0	0	0
ENSMUSG00000103894	0	0	0	0	0	0	0	0	0	0
ENSMUSG00000103896	0	0	0	0	0	0	0	0	0	0
ENSMUSG00000103890	0	0	0	0	0	0	0	0	0	0
ENSMUSG00000103893	0	0	0	0	0	0	0	0	0	0
ENSMUSG00000103892	0	0	0	0	0	0	0	0	0	0
ENSMUSG00000038541	3	6	14	17	9	6	0	0	3	5
ENSMUSG00000069670	54	54	62	52	23	96	136	98	78	90
ENSMUSG00000096769	0	0	0	0	0	0	0	0	0	0
ENSMUSG00000096763	0	0	0	0	0	1	0	0	2	0
ENSMUSG00000096760	0	0	0	0	0	0	0	0	0	0
ENSMUSG00000096761	0	0	0	0	0	0	0	0	0	0
ENSMUSG00000019737	18	7	14	19	10	8	24	3	14	7
ENSMUSG00000031142	13	5	6	32	4	6	17	4	2	5
ENSMUSG00000063163	0	0	3	0	3	0	0	0	6	0
ENSMUSG00000063166	0	2	0	2	0	0	0	0	0	0
ENSMUSG00000094980	0	0	0	0	0	0	0	0	0	0
ENSMUSG00000105809	0	0	0	0	0	0	0	0	0	0
ENSMUSG00000102858	0	0	0	0	0	0	0	0	0	0
ENSMUSG00000102859	0	0	0	0	0	0	0	0	0	0
ENSMUSG00000102857	0	0	0	0	0	0	0	0	0	0
ENSMUSG00000105806	0	0	0	0	0	0	0	3	0	0
ENSMUSG00000102855	0	0	0	0	0	0	0	0	0	0
ENSMUSG00000102852	0	4	0	0	3	0	12	11	0	2
ENSMUSG00000102853	0	0	0	0	0	0	0	0	0	0
ENSMUSG00000102851	0	1	0	2	0	0	0	0	0	0
ENSMUSG00000076915	0	0	1	0	0	0	0	0	0	0
ENSMUSG00000078235	0	0	0	0	0	0	3	0	0	0
ENSMUSG00000091239	0	0	0	0	0	0	0	0	0	0
ENSMUSG00000108983	0	0	0	3	0	0	0	0	0	0
ENSMUSG00000108980	3	0	3	0	2	2	0	4	6	4
ENSMUSG00000108981	0	0	0	0	0	0	0	0	0	0
ENSMUSG00000108987	0	0	0	0	0	0	0	0	0	3
ENSMUSG00000091230	0	0	0	0	0	0	2	0	3	0
ENSMUSG00000108988	0	0	0	0	0	0	0	0	0	0
ENSMUSG00000103031	0	0	0	0	0	0	0	0	0	0
ENSMUSG00000051537	25	32	20	12	24	49	24	64	21	34

ENSMUSG00000030235	12	30	15	14	26	9	22	23	18	3
ENSMUSG00000030236	0	0	3	0	0	4	3	0	0	3
ENSMUSG00000030237	105	164	82	118	81	166	193	131	93	128
ENSMUSG00000030230	0	2	0	6	6	0	0	0	0	4
ENSMUSG00000077445	0	0	0	0	0	0	0	0	0	0
ENSMUSG00000097870	0	3	3	0	0	6	6	0	0	3
ENSMUSG00000064728	0	0	0	0	0	0	0	0	0	0
ENSMUSG00000110808	0	0	0	0	0	0	0	0	0	0
ENSMUSG00000110802	0	0	0	0	0	0	0	0	0	0
ENSMUSG00000104865	0	0	0	0	0	0	0	0	0	0
ENSMUSG00000104861	0	0	0	0	0	0	3	0	0	0
ENSMUSG00000104860	6	0	0	0	0	0	0	3	0	0
ENSMUSG00000104869	34	14	24	43	24	47	17	19	28	13
ENSMUSG00000104868	0	0	0	0	0	0	0	0	0	0
ENSMUSG00000088248	2	0	0	0	2	2	0	0	0	0
ENSMUSG00000088244	0	0	0	0	0	0	0	0	0	0
ENSMUSG00000088245	0	0	0	0	0	0	0	0	0	0
ENSMUSG00000088243	0	0	0	0	0	0	0	0	0	0
ENSMUSG00000088240	0	0	0	0	0	0	0	0	0	0
ENSMUSG00000088241	0	0	0	0	0	0	0	0	0	0
ENSMUSG00000108073	0	0	0	0	0	0	0	0	0	0
ENSMUSG00000022484	0	0	0	0	0	0	0	0	0	0
ENSMUSG00000022485	0	0	3	0	6	2	38	8	11	15
ENSMUSG00000022487	0	0	0	0	5	0	4	2	0	9
ENSMUSG00000022483	6	3	10	6	261	12	388	61	26	21
ENSMUSG00000107987	0	0	0	0	0	0	0	0	0	0
ENSMUSG00000107986	0	0	0	0	0	0	0	0	0	0
ENSMUSG00000095448	0	0	0	0	0	0	0	0	0	0
ENSMUSG00000107984	0	0	0	0	0	0	0	0	0	0
ENSMUSG00000107983	0	0	0	0	0	0	0	0	0	0
ENSMUSG00000107982	0	0	0	0	0	0	0	0	0	0
ENSMUSG00000107981	0	0	0	0	0	0	0	0	0	0
ENSMUSG00000107980	0	0	0	0	0	0	0	0	0	0
ENSMUSG00000095440	178	237	198	211	156	254	383	238	337	253
ENSMUSG00000095446	0	0	0	0	0	0	0	0	0	0
ENSMUSG00000062235	0	0	6	0	0	0	0	0	0	0
ENSMUSG00000095445	0	1	1	1	1	1	1	2	3	0
ENSMUSG00000103077	0	0	0	0	0	0	0	0	0	0
ENSMUSG00000114430	0	0	0	0	0	0	0	0	0	0
ENSMUSG00000103073	0	0	0	0	0	0	0	0	0	0
ENSMUSG00000103070	0	0	3	0	0	0	0	0	1	1
ENSMUSG00000065987	100	18	127	20	35	105	179	138	42	90
ENSMUSG00000103078	0	0	0	0	0	0	0	0	0	0
ENSMUSG00000089409	0	0	0	0	0	0	0	0	3	0
ENSMUSG00000061150	0	0	0	0	0	0	0	0	0	0
ENSMUSG00000094312	0	0	0	0	0	0	0	0	0	0
ENSMUSG00000094314	0	0	0	0	0	0	0	0	0	0
ENSMUSG00000094317	0	0	0	0	0	0	0	0	0	0
ENSMUSG00000094316	0	0	0	0	0	0	0	0	0	0
ENSMUSG00000089404	0	0	0	0	0	1	4	0	3	0
ENSMUSG00000113046	0	0	0	0	0	0	0	0	0	0
ENSMUSG00000023289	0	0	0	0	0	0	0	0	0	0
ENSMUSG00000036306	270	334	392	338	361	418	267	365	390	449
ENSMUSG00000036304	23	30	35	26	11	38	20	27	39	33
ENSMUSG00000106164	48	20	65	53	43	39	31	54	66	52
ENSMUSG00000106165	0	0	0	0	0	0	3	0	2	0
ENSMUSG00000106162	0	0	0	0	0	0	0	0	0	0
ENSMUSG00000106160	0	0	0	0	0	0	0	0	0	0

ENSMUSG00000106168	0	0	0	0	0	0	0	0	0	0
ENSMUSG00000092855	0	0	0	0	0	0	0	0	0	0
ENSMUSG00000029878	0	0	0	3	0	2	14	0	0	0
ENSMUSG00000024883	238	171	259	365	263	264	476	266	342	278
ENSMUSG00000017607	70	68	80	76	44	46	125	75	120	100
ENSMUSG00000060827	0	0	0	0	0	0	0	0	0	0
ENSMUSG00000020396	6	6	0	3	11	12	3	12	9	3
ENSMUSG00000106292	1	0	0	0	0	0	0	0	0	3
ENSMUSG00000106293	0	0	0	0	3	0	0	0	0	3
ENSMUSG00000097935	0	0	2	5	2	0	6	4	3	5
ENSMUSG00000106297	0	0	0	0	0	0	0	0	0	0
ENSMUSG00000106298	0	0	0	0	0	0	0	0	0	0
ENSMUSG00000028475	667	579	504	694	752	1051	751	749	818	741
ENSMUSG00000092256	0	0	0	0	0	0	0	0	0	0
ENSMUSG00000102746	0	0	0	0	0	0	0	0	0	0
ENSMUSG00000092255	0	0	0	0	0	0	0	0	0	0
ENSMUSG00000092250	0	0	0	0	0	0	0	0	0	0
ENSMUSG00000092251	0	0	0	0	0	0	0	0	0	0
ENSMUSG00000092259	0	0	0	0	0	0	0	0	0	0
ENSMUSG00000035651	0	0	0	0	0	0	0	0	0	0
ENSMUSG00000092375	0	0	0	0	0	0	0	0	0	0
ENSMUSG00000112511	0	0	0	0	3	0	0	0	0	0
ENSMUSG00000112516	0	0	0	2	0	0	0	0	0	0
ENSMUSG00000039337	0	0	0	0	0	0	0	0	0	0
ENSMUSG00000112514	0	0	0	0	0	0	0	0	0	1
ENSMUSG00000039335	0	0	0	0	0	0	0	0	0	0
ENSMUSG00000024770	0	1	0	0	0	0	0	6	0	5
ENSMUSG00000024771	0	0	0	0	0	0	0	0	0	0
ENSMUSG00000112518	0	0	0	0	0	0	0	0	0	0
ENSMUSG00000112519	0	0	0	0	0	3	0	0	0	0
ENSMUSG00000024774	0	0	0	30	0	0	0	0	14	0
ENSMUSG00000099581	0	0	0	0	0	0	0	0	0	0
ENSMUSG00000092376	0	0	0	0	0	0	0	0	0	0
ENSMUSG00000092370	0	0	0	0	0	0	0	0	0	0
ENSMUSG00000113641	0	0	0	0	0	0	0	0	0	0
ENSMUSG00000097156	6	0	0	5	0	2	0	0	0	0
ENSMUSG00000113644	0	0	0	0	0	0	0	0	0	0
ENSMUSG00000038067	0	0	6	0	3	173	11	3	3	0
ENSMUSG00000097155	0	0	0	0	0	6	0	3	3	3
ENSMUSG00000114918	0	0	0	0	0	0	5	0	0	0
ENSMUSG00000114916	0	0	0	0	0	0	0	0	0	0
ENSMUSG00000114910	0	0	0	0	0	0	0	0	0	0
ENSMUSG00000114911	0	0	0	0	0	0	0	0	0	0
ENSMUSG00000104027	0	0	0	0	0	0	0	0	0	0
ENSMUSG00000005339	21	31	21	12	8	44	103	15	12	28
ENSMUSG00000066272	9	7	3	4	5	2	10	30	17	15
ENSMUSG00000106832	3	12	5	6	6	6	5	6	0	3
ENSMUSG00000106833	0	0	0	0	0	0	0	0	0	0
ENSMUSG00000070511	0	0	0	0	0	0	0	0	0	0
ENSMUSG00000110817	0	3	0	0	3	0	0	0	0	6
ENSMUSG00000021118	59	60	63	63	46	45	79	36	58	34
ENSMUSG00000096200	0	0	0	0	0	0	0	0	0	0
ENSMUSG00000096202	0	0	0	0	0	0	0	0	0	0
ENSMUSG00000096209	0	0	0	0	0	0	0	0	0	0
ENSMUSG00000096208	0	0	0	0	0	0	0	0	0	0
ENSMUSG00000100008	0	0	0	0	0	0	0	0	0	0
ENSMUSG00000111739	0	0	0	0	0	0	0	0	0	0
ENSMUSG00000111738	22	20	5	21	19	24	23	14	9	29

ENSMUSG00000111733	0	0	0	0	0	0	0	0	0	0
ENSMUSG00000111732	0	0	0	0	0	0	0	0	0	0
ENSMUSG00000111730	0	9	0	2	0	0	0	2	0	0
ENSMUSG00000104852	51	54	44	46	37	57	63	69	82	80
ENSMUSG00000111734	0	3	0	0	0	0	0	0	0	0
ENSMUSG00000057417	0	0	0	0	0	0	0	0	3	0
ENSMUSG00000104855	0	0	0	0	0	0	0	0	0	0
ENSMUSG00000025912	164	220	209	173	160	302	608	275	398	236
ENSMUSG00000025916	6	9	9	20	10	0	18	6	29	20
ENSMUSG00000094592	0	0	0	0	0	0	0	0	0	0
ENSMUSG00000104478	5	0	0	0	3	8	0	3	0	0
ENSMUSG00000104479	21	4	14	12	19	29	22	18	9	24
ENSMUSG00000108797	0	0	0	0	0	0	0	0	0	0
ENSMUSG00000104477	0	0	0	0	0	0	0	0	0	0
ENSMUSG00000108795	0	0	0	0	0	3	0	4	0	0
ENSMUSG00000104472	0	0	0	0	0	0	0	0	0	0
ENSMUSG00000108792	0	0	0	0	0	0	0	0	0	0
ENSMUSG00000104470	3	0	0	0	0	0	0	0	0	0
ENSMUSG00000108790	6	3	3	6	7	4	0	0	0	1
ENSMUSG00000027220	3	0	3	54	9	0	26	9	12	18
ENSMUSG00000045395	0	0	0	0	0	0	0	0	0	0
ENSMUSG00000095714	0	0	0	0	0	0	0	0	0	0
ENSMUSG00000044664	0	0	3	0	0	0	0	0	3	0
ENSMUSG00000044667	11	6	14	17	12	18	12	24	15	14
ENSMUSG00000083430	0	0	0	0	0	0	0	0	0	0
ENSMUSG00000114009	0	1	0	0	0	0	0	0	0	0
ENSMUSG00000073834	0	0	5	0	0	0	0	0	0	0
ENSMUSG00000079806	0	0	0	0	0	0	0	0	0	0
ENSMUSG00000079800	5	0	4	0	0	3	15	7	6	0
ENSMUSG00000112591	0	0	0	0	0	0	0	0	0	0
ENSMUSG00000027517	6	0	0	0	0	0	0	0	0	0
ENSMUSG00000026542	0	0	0	0	0	0	0	0	0	0
ENSMUSG00000046282	0	0	0	0	0	0	4	0	0	0
ENSMUSG00000027518	0	0	0	3	0	0	0	0	0	0
ENSMUSG00000046287	0	0	0	0	0	0	0	0	0	0
ENSMUSG00000046133	0	0	0	0	0	0	0	0	0	0
ENSMUSG00000074987	9	15	4	12	13	19	29	4	15	10
ENSMUSG00000095163	0	0	0	0	0	0	0	0	0	0
ENSMUSG00000095168	0	0	0	0	0	3	0	2	0	0
ENSMUSG00000045539	0	0	0	0	0	0	0	0	0	0
ENSMUSG00000086666	0	0	0	0	0	0	0	0	0	0
ENSMUSG00000052131	0	0	0	0	0	0	12	0	3	5
ENSMUSG00000052135	80	80	63	45	38	68	76	66	73	80
ENSMUSG00000028864	220	243	261	260	202	427	645	443	510	372
ENSMUSG00000028865	4	8	4	9	3	6	25	22	19	3
ENSMUSG00000081295	0	1	1	1	0	0	0	0	2	0
ENSMUSG00000081292	0	0	0	0	0	0	0	0	0	0
ENSMUSG00000081293	0	0	0	0	0	0	0	0	0	0
ENSMUSG00000081290	0	0	0	0	0	0	0	0	0	0
ENSMUSG00000091874	0	0	0	0	0	0	0	0	0	0
ENSMUSG00000081298	0	0	0	0	0	0	0	0	0	0
ENSMUSG00000080749	0	0	0	0	0	0	0	1	0	0
ENSMUSG00000080748	0	0	0	0	0	0	0	0	0	0
ENSMUSG00000038327	0	0	0	0	0	0	0	0	0	0
ENSMUSG00000080743	0	0	0	0	0	0	0	3	0	0
ENSMUSG00000080742	0	0	0	0	0	0	0	0	0	0
ENSMUSG00000094496	0	0	0	0	0	0	0	0	0	0
ENSMUSG00000114000	0	0	0	0	0	0	0	0	0	0

ENSMUSG00000093648	0	0	0	0	0	0	0	0	0	0
ENSMUSG00000093649	0	0	0	0	0	0	0	0	0	0
ENSMUSG00000093642	0	0	0	0	0	2	0	0	0	0
ENSMUSG00000086743	0	0	0	5	0	0	5	0	12	0
ENSMUSG00000048480	0	0	3	21	5	0	6	6	0	2
ENSMUSG00000048483	0	0	0	3	3	12	3	16	5	6
ENSMUSG00000048484	9	23	11	6	3	5	8	8	12	5
ENSMUSG00000048485	13	11	17	55	18	36	64	17	12	21
ENSMUSG00000098697	0	0	0	0	0	0	0	0	0	0
ENSMUSG00000047661	0	0	0	0	0	0	0	0	0	0
ENSMUSG00000079048	0	3	0	6	8	16	14	2	6	8
ENSMUSG00000047667	0	0	0	0	0	0	0	0	0	0
ENSMUSG00000098756	0	0	0	0	0	0	0	0	0	0
ENSMUSG00000033233	413	367	343	397	337	324	412	317	437	393
ENSMUSG00000098983	0	0	0	0	0	0	0	0	0	0
ENSMUSG00000073686	0	0	0	0	0	3	3	0	0	0
ENSMUSG00000022496	44	8	30	27	12	12	47	43	33	24
ENSMUSG00000113177	0	0	0	0	0	0	0	0	0	0
ENSMUSG00000112650	0	0	0	0	0	0	0	0	0	0
ENSMUSG00000077514	0	0	0	0	0	0	0	0	0	0
ENSMUSG00000077517	0	0	0	0	0	0	0	0	0	0
ENSMUSG00000077511	0	0	0	0	0	0	0	0	0	0
ENSMUSG00000040576	0	0	0	0	0	0	0	0	0	0
ENSMUSG00000077518	0	0	0	0	0	0	0	0	0	0
ENSMUSG00000077519	0	0	0	0	0	0	0	0	0	0
ENSMUSG00000113172	0	3	0	0	0	0	0	0	0	3
ENSMUSG00000082249	0	0	0	0	0	0	0	0	0	0
ENSMUSG00000082244	0	0	2	0	0	0	0	0	0	0
ENSMUSG00000082247	2	0	3	0	0	8	5	1	7	0
ENSMUSG00000082240	0	0	0	0	0	0	0	0	0	0
ENSMUSG00000082242	0	0	0	0	0	0	0	0	0	0
ENSMUSG00000048226	0	0	0	0	0	0	0	0	0	0
ENSMUSG00000099274	0	0	0	0	0	0	0	0	0	0
ENSMUSG00000099270	0	0	0	0	0	0	0	0	0	0
ENSMUSG00000099273	0	0	0	0	0	0	0	0	0	0
ENSMUSG00000099279	0	0	0	0	0	0	0	0	0	0
ENSMUSG00000099278	0	0	0	0	0	0	0	0	0	0
ENSMUSG00000083599	0	0	0	0	0	0	0	0	0	0
ENSMUSG00000083598	0	0	0	0	0	0	0	0	0	0
ENSMUSG00000083592	0	0	0	0	0	0	0	0	0	0
ENSMUSG00000083590	0	0	0	0	0	0	0	0	0	0
ENSMUSG00000083597	0	0	0	0	0	0	0	0	0	0
ENSMUSG00000083594	0	0	0	0	0	0	0	1	0	0
ENSMUSG00000093032	0	0	0	0	0	0	0	0	0	0
ENSMUSG00000062791	0	0	0	0	0	0	0	0	0	0
ENSMUSG00000100740	0	0	0	0	0	0	0	0	0	0
ENSMUSG00000100741	0	0	0	0	0	0	0	0	0	0
ENSMUSG00000100742	0	0	0	0	0	0	0	0	0	0
ENSMUSG00000100743	0	0	0	0	0	0	0	0	0	0
ENSMUSG00000100744	2	0	0	0	0	0	0	0	0	0
ENSMUSG00000100745	0	0	0	0	0	0	0	0	0	0
ENSMUSG00000100746	0	0	0	0	0	0	0	0	0	0
ENSMUSG00000100749	0	0	0	0	0	0	0	0	0	0
ENSMUSG00000041620	0	0	0	0	0	0	0	0	0	0
ENSMUSG00000112488	0	0	0	0	0	0	0	0	0	0
ENSMUSG00000102458	0	0	0	0	0	0	0	0	0	0
ENSMUSG00000064619	0	0	0	0	0	0	0	0	0	0
ENSMUSG00000064618	0	0	0	0	0	0	0	0	0	0

ENSMUSG00000064617	0	0	0	0	0	0	0	0	0	0
ENSMUSG00000102453	0	0	0	0	0	0	0	0	0	0
ENSMUSG00000102450	0	0	0	0	0	0	0	0	0	0
ENSMUSG00000064613	0	0	0	0	0	0	0	0	0	0
ENSMUSG00000083625	0	0	0	0	0	0	0	0	0	0
ENSMUSG00000083620	0	0	0	0	0	0	0	0	0	0
ENSMUSG00000083623	9	0	0	0	0	0	0	0	0	0
ENSMUSG00000083629	0	0	0	0	0	0	0	0	0	0
ENSMUSG00000083628	0	0	0	0	0	0	0	0	0	0
ENSMUSG00000087908	0	0	0	0	0	0	0	0	0	0
ENSMUSG00000075117	0	0	0	0	0	0	0	0	0	0
ENSMUSG00000075114	0	0	0	0	0	0	0	0	0	0
ENSMUSG00000075115	0	0	0	0	0	0	0	0	0	0
ENSMUSG00000075112	0	0	0	0	0	0	0	0	0	0
ENSMUSG00000075113	0	0	0	0	0	0	0	0	0	0
ENSMUSG00000075110	0	0	0	0	0	0	0	0	0	0
ENSMUSG00000075111	0	0	0	0	0	0	0	0	0	0
ENSMUSG00000031893	4	0	1	4	3	4	2	3	3	7
ENSMUSG00000087902	0	0	0	0	0	0	0	0	0	0
ENSMUSG00000087904	0	0	0	0	0	0	0	0	0	0
ENSMUSG00000087905	0	0	0	0	0	0	0	0	0	0
ENSMUSG00000075119	0	0	0	0	0	0	0	0	0	0
ENSMUSG00000061397	8	0	0	3	0	15	51	15	20	13
ENSMUSG00000061393	226	266	316	249	253	322	227	373	292	258
ENSMUSG00000061392	0	0	0	0	0	0	0	0	0	0
ENSMUSG00000085502	0	0	0	0	0	0	0	0	0	0
ENSMUSG00000085500	0	0	0	0	0	0	0	0	0	0
ENSMUSG00000085507	0	0	0	0	0	0	0	0	0	0
ENSMUSG00000085504	0	0	0	0	0	0	0	0	0	0
ENSMUSG00000085508	0	0	0	0	0	0	0	0	0	0
ENSMUSG00000029348	92	89	75	116	64	99	207	98	164	83
ENSMUSG00000107219	0	0	0	0	0	0	0	0	6	0
ENSMUSG00000107218	0	0	0	0	0	0	0	0	0	0
ENSMUSG00000107217	0	0	0	0	0	0	0	0	0	0
ENSMUSG00000107214	0	0	0	0	0	0	0	0	0	0
ENSMUSG00000107213	0	0	0	0	0	0	0	0	0	0
ENSMUSG00000107212	0	0	0	0	0	0	0	0	0	0
ENSMUSG00000033794	0	0	0	0	0	0	0	0	0	0
ENSMUSG00000101909	0	0	0	0	0	0	0	0	0	0
ENSMUSG00000101908	0	0	0	0	0	0	0	0	0	0
ENSMUSG00000101900	0	0	0	0	0	0	0	0	0	0
ENSMUSG00000101903	0	14	12	11	5	7	14	18	14	0
ENSMUSG00000101902	0	0	0	0	0	0	0	0	0	0
ENSMUSG00000101907	0	0	0	0	0	0	0	0	0	0
ENSMUSG00000110298	37	22	38	27	9	69	30	67	46	33
ENSMUSG00000083171	0	0	0	0	0	0	0	0	0	0
ENSMUSG00000036672	197	216	229	238	157	208	345	320	288	264
ENSMUSG00000103460	2	2	0	2	0	3	2	0	2	0
ENSMUSG00000103461	0	0	0	0	0	0	0	0	0	0
ENSMUSG00000103462	0	0	0	0	0	0	0	0	0	0
ENSMUSG00000103463	0	0	0	0	0	0	0	0	0	0
ENSMUSG00000103464	0	0	0	0	0	0	1	0	2	2
ENSMUSG00000103465	0	0	0	0	0	0	0	0	0	0
ENSMUSG00000103466	0	0	0	0	0	0	0	1	0	0
ENSMUSG00000103468	0	0	0	0	0	0	0	0	0	0
ENSMUSG00000065254	0	0	2	0	0	0	2	0	2	0
ENSMUSG00000065255	0	0	0	0	0	0	0	0	0	0
ENSMUSG00000065256	0	0	0	0	0	0	0	0	0	0

ENSMUSG00000093112	0	0	0	0	0	0	2	0	0	0
ENSMUSG00000094325	0	0	0	0	0	0	0	0	0	0
ENSMUSG00000063821	24	13	15	3	8	7	43	19	12	25
ENSMUSG00000063823	0	0	0	0	0	0	0	0	0	0
ENSMUSG00000063827	0	0	0	0	0	0	0	0	0	0
ENSMUSG00000079698	0	0	0	0	0	0	0	0	0	0
ENSMUSG00000030854	0	0	0	3	2	3	9	3	8	0
ENSMUSG00000093115	0	0	0	0	0	0	0	0	0	0
ENSMUSG00000024578	0	0	0	3	0	0	0	0	0	0
ENSMUSG00000024579	87	84	107	111	74	118	155	128	72	119
ENSMUSG00000089506	0	0	0	0	0	0	0	0	0	0
ENSMUSG00000089507	0	0	0	0	0	1	0	0	0	0
ENSMUSG00000097310	12	3	5	6	6	6	17	0	12	6
ENSMUSG00000097311	0	0	0	0	0	0	0	0	0	0
ENSMUSG00000097313	0	14	0	24	0	12	15	0	20	12
ENSMUSG00000022805	3	3	11	5	3	3	6	9	24	0
ENSMUSG00000073226	0	0	0	0	0	0	0	0	0	0
ENSMUSG00000022809	0	0	0	3	3	0	0	2	3	0
ENSMUSG00000038665	0	3	6	6	16	12	11	30	8	12
ENSMUSG00000062142	0	0	0	0	0	0	0	0	0	0
ENSMUSG00000062157	195	218	297	233	255	369	237	327	263	292
ENSMUSG00000062154	0	0	0	0	0	0	0	0	0	0
ENSMUSG00000069733	6	6	0	11	0	8	6	3	15	3
ENSMUSG00000102339	0	0	0	0	0	0	0	0	0	0
ENSMUSG00000102338	0	0	0	0	0	0	0	0	0	0
ENSMUSG00000102337	0	0	0	0	0	0	0	0	0	0
ENSMUSG00000102336	101	126	145	134	109	143	118	171	142	144
ENSMUSG00000102335	0	0	0	0	0	0	0	0	0	0
ENSMUSG00000102334	0	0	0	0	0	0	0	0	0	0
ENSMUSG00000105101	0	0	0	0	0	0	0	0	0	0
ENSMUSG00000102332	0	0	0	0	0	0	0	0	0	0
ENSMUSG00000102331	3	13	6	6	1	7	35	14	14	15
ENSMUSG00000102330	0	0	0	0	0	0	0	0	3	3
ENSMUSG00000060402	9	9	6	33	6	9	18	33	24	15
ENSMUSG00000096049	0	0	0	0	0	0	0	0	0	0
ENSMUSG00000096046	0	0	0	0	0	0	0	0	0	0
ENSMUSG00000096045	8	5	12	0	14	6	11	11	8	0
ENSMUSG00000096044	0	0	0	0	0	0	0	0	0	0
ENSMUSG00000096043	0	0	0	0	0	0	0	0	0	0
ENSMUSG00000096041	0	0	0	0	0	0	0	0	0	0
ENSMUSG00000094116	0	0	0	0	0	0	0	0	0	0
ENSMUSG00000066704	0	0	0	0	0	0	0	0	0	0
ENSMUSG00000060407	0	0	0	0	0	0	0	0	0	0
ENSMUSG00000096289	0	0	0	0	0	0	0	0	0	0
ENSMUSG00000096288	0	0	0	0	0	0	0	0	0	0
ENSMUSG00000094115	0	0	0	0	0	0	0	0	0	0
ENSMUSG00000105292	0	0	0	0	0	0	0	0	0	0
ENSMUSG00000105294	0	0	0	0	0	0	0	0	0	0
ENSMUSG00000021056	0	0	0	0	0	0	0	0	0	0
ENSMUSG00000021057	211	257	381	106	122	341	317	529	358	657
ENSMUSG00000057650	0	0	0	0	0	0	0	0	0	0
ENSMUSG00000105299	0	0	0	0	0	0	0	0	0	0
ENSMUSG00000022657	16	14	24	27	6	21	62	64	36	38
ENSMUSG00000114331	0	0	0	0	0	0	0	0	2	0
ENSMUSG00000102753	0	0	0	0	0	0	0	0	0	0
ENSMUSG00000103594	0	6	0	0	0	0	0	0	0	0
ENSMUSG00000114332	0	0	0	0	0	3	0	0	0	0
ENSMUSG00000091297	10	5	20	13	11	8	12	6	7	4

ENSMUSG00000050150	7	3	2	9	5	3	34	6	21	9
ENSMUSG00000104278	0	0	0	0	0	0	0	0	0	0
ENSMUSG00000108558	0	0	0	0	0	0	0	0	0	0
ENSMUSG00000108554	1	0	0	0	0	0	0	0	4	0
ENSMUSG00000104276	0	0	0	0	0	0	0	0	0	0
ENSMUSG00000050158	0	0	0	0	0	0	0	0	0	0
ENSMUSG00000104270	0	0	0	0	0	0	0	0	0	0
ENSMUSG00000108550	0	0	0	0	0	2	0	0	0	0
ENSMUSG00000108553	0	0	0	0	0	0	0	0	0	0
ENSMUSG00000108552	0	0	0	0	0	0	0	0	0	0
ENSMUSG00000071322	0	9	3	0	6	3	18	0	3	0
ENSMUSG00000096287	0	0	0	0	0	0	0	0	0	0
ENSMUSG00000101178	0	0	0	0	0	0	1	0	0	0
ENSMUSG00000101179	0	0	0	3	0	0	0	0	0	0
ENSMUSG00000107526	0	0	0	2	0	0	0	3	0	0
ENSMUSG00000060701	0	0	0	0	0	0	0	0	0	0
ENSMUSG00000101171	0	0	0	0	0	0	0	0	0	0
ENSMUSG00000101173	0	0	0	0	0	0	0	0	0	0
ENSMUSG00000101177	0	0	0	0	0	0	0	0	0	0
ENSMUSG00000010751	127	117	98	99	108	116	170	151	136	140
ENSMUSG00000084269	0	0	0	0	0	0	0	0	0	0
ENSMUSG00000101338	0	0	0	0	0	0	0	0	0	0
ENSMUSG00000064932	0	0	0	0	0	0	0	0	0	0
ENSMUSG00000101339	0	0	0	0	0	0	0	0	0	0
ENSMUSG00000091061	0	0	0	0	0	0	0	0	0	0
ENSMUSG00000073938	0	0	0	0	0	0	0	0	0	0
ENSMUSG00000056215	0	3	9	9	6	18	11	6	19	3
ENSMUSG00000101332	0	0	0	0	0	0	0	0	0	0
ENSMUSG00000095769	0	0	0	0	0	0	0	0	0	0
ENSMUSG00000101333	0	0	0	0	0	0	0	0	0	0
ENSMUSG00000082616	0	0	0	0	0	0	0	6	0	0
ENSMUSG00000095765	0	0	0	0	0	0	0	0	0	0
ENSMUSG00000026919	0	0	0	0	0	0	0	6	0	0
ENSMUSG00000095763	0	0	0	0	0	0	0	0	0	0
ENSMUSG00000104746	0	0	0	0	0	0	0	0	0	0
ENSMUSG00000100227	0	0	0	0	0	0	0	0	0	0
ENSMUSG00000100226	0	0	0	0	0	0	0	0	0	0
ENSMUSG00000108246	1	1	0	0	0	0	9	0	0	1
ENSMUSG00000100223	0	0	0	0	0	0	0	0	0	0
ENSMUSG00000100221	0	0	0	0	0	0	0	0	0	0
ENSMUSG00000101336	0	0	0	0	0	0	0	0	0	0
ENSMUSG00000100229	0	0	0	0	0	0	0	0	0	0
ENSMUSG00000027068	29	35	23	157	32	69	208	54	97	23
ENSMUSG00000042428	387	368	404	331	268	290	382	367	214	393
ENSMUSG00000061577	18	18	19	8	12	38	76	41	56	11
ENSMUSG00000102655	0	0	0	0	0	0	0	0	0	0
ENSMUSG00000113729	0	0	0	0	0	0	0	0	0	0
ENSMUSG00000086503	51	21	38	12	6	7	21	49	12	36
ENSMUSG00000062833	0	0	0	0	0	0	0	0	0	0
ENSMUSG00000086500	7	2	2	0	3	2	8	3	0	8
ENSMUSG00000086504	0	0	4	0	0	0	0	0	0	0
ENSMUSG00000086509	0	0	0	0	0	0	0	0	0	0
ENSMUSG00000102657	20	30	14	11	22	25	54	36	44	24
ENSMUSG00000114171	0	0	0	0	0	0	0	0	0	0
ENSMUSG00000092248	0	0	0	0	0	0	0	0	0	0
ENSMUSG00000112497	0	0	0	0	0	0	0	0	0	0
ENSMUSG00000112496	0	4	0	0	0	5	0	0	0	0
ENSMUSG00000112495	0	2	1	5	1	1	1	3	4	1

ENSMUSG00000112494	0	0	0	0	3	0	0	0	0	0
ENSMUSG00000112492	0	0	0	0	2	3	0	3	0	0
ENSMUSG00000112491	0	0	0	0	0	0	0	0	0	0
ENSMUSG00000093331	0	0	0	0	0	0	0	0	0	1
ENSMUSG00000049515	6	0	12	0	9	7	9	10	0	12
ENSMUSG00000049511	24	26	2	0	9	7	6	2	6	34
ENSMUSG00000067144	3	0	3	21	0	0	0	0	3	6
ENSMUSG00000093338	0	0	0	0	0	0	1	0	0	0
ENSMUSG00000112498	0	0	0	0	0	0	0	0	0	0
ENSMUSG00000009670	20	23	39	51	16	18	46	15	29	8
ENSMUSG00000112721	0	0	0	6	3	0	0	0	0	3
ENSMUSG00000112720	0	0	0	0	0	0	0	0	0	0
ENSMUSG00000112723	0	0	4	0	0	0	2	0	0	0
ENSMUSG00000109098	0	0	0	0	0	0	0	0	0	0
ENSMUSG00000112725	0	0	0	0	0	0	0	0	0	0
ENSMUSG00000112724	0	0	0	0	0	0	0	0	0	0
ENSMUSG00000112727	2	2	2	5	7	0	4	3	2	0
ENSMUSG00000112726	0	0	0	0	0	0	0	0	0	0
ENSMUSG00000109093	0	0	12	26	3	0	0	0	0	4
ENSMUSG00000112728	0	0	0	0	0	0	0	0	0	0
ENSMUSG00000109091	0	0	0	0	0	0	0	0	0	0
ENSMUSG00000109097	11	14	5	0	6	11	9	6	17	18
ENSMUSG00000109096	12	0	0	0	0	0	0	0	6	9
ENSMUSG00000109095	0	0	0	0	0	0	0	0	0	0
ENSMUSG00000017832	6	13	13	24	9	26	23	21	21	20
ENSMUSG00000106315	0	0	0	0	0	0	0	0	0	0
ENSMUSG00000087045	0	0	0	0	0	0	0	0	0	0
ENSMUSG00000106316	0	0	0	0	0	0	0	3	0	0
ENSMUSG00000106311	0	0	0	0	0	0	0	0	0	0
ENSMUSG00000106310	0	0	0	0	0	6	0	0	0	0
ENSMUSG00000106313	1	0	0	0	0	1	0	7	3	0
ENSMUSG00000106319	0	0	0	0	0	0	1	0	0	0
ENSMUSG00000106318	0	0	0	0	0	0	0	0	0	0
ENSMUSG00000097055	40	20	38	39	19	19	29	35	46	34
ENSMUSG00000097054	0	0	0	0	0	0	0	0	0	0
ENSMUSG00000097050	0	0	0	0	0	0	3	0	9	0
ENSMUSG00000110097	0	0	0	0	0	0	0	0	0	0
ENSMUSG00000051362	0	0	0	0	0	0	0	0	0	0
ENSMUSG00000051361	0	2	0	0	0	0	0	0	0	0
ENSMUSG00000087042	0	7	0	3	0	6	0	3	0	0
ENSMUSG00000051367	184	249	478	182	289	150	271	278	298	401
ENSMUSG00000004296	2	10	0	0	0	0	47	15	12	0
ENSMUSG00000096902	0	0	0	0	0	0	0	0	0	0
ENSMUSG00000096903	0	0	0	0	0	0	0	0	0	0
ENSMUSG00000114893	0	0	4	12	10	0	3	3	6	4
ENSMUSG00000096901	0	0	1	0	0	1	2	1	0	2
ENSMUSG00000096906	0	0	0	0	0	0	0	0	0	0
ENSMUSG00000114894	2	0	0	0	0	0	0	0	0	0
ENSMUSG00000096904	9	11	5	14	10	16	17	21	12	18
ENSMUSG00000114896	0	0	0	3	0	0	0	0	0	0
ENSMUSG00000114899	0	0	0	0	0	0	0	0	0	0
ENSMUSG00000046899	0	0	0	0	0	0	0	0	0	0
ENSMUSG00000110383	0	0	0	0	0	0	0	0	0	0
ENSMUSG00000110382	27	24	12	17	15	20	0	6	0	3
ENSMUSG00000110385	0	0	0	0	0	0	0	0	0	0
ENSMUSG00000110384	0	0	0	0	0	0	0	0	0	0
ENSMUSG00000110386	29	7	26	34	26	30	16	4	11	28
ENSMUSG00000110389	0	0	0	0	0	0	0	0	0	0

ENSMUSG00000099578	0	0	0	0	0	0	0	0	0	0
ENSMUSG00000099577	0	0	0	0	0	0	0	0	0	0
ENSMUSG00000039321	12	32	58	30	18	21	112	57	119	97
ENSMUSG00000109325	0	0	0	0	0	0	0	2	0	0
ENSMUSG00000105665	0	0	0	0	0	0	0	0	0	0
ENSMUSG00000105666	0	0	0	0	0	0	0	0	0	0
ENSMUSG00000099575	0	0	0	0	0	0	0	0	0	0
ENSMUSG00000105660	0	3	0	2	3	0	0	0	7	0
ENSMUSG00000039323	39	26	43	6	29	53	148	106	40	24
ENSMUSG00000105662	0	0	0	0	0	0	0	0	0	0
ENSMUSG00000081950	0	0	0	0	0	0	2	0	0	0
ENSMUSG00000114762	0	0	0	0	0	3	0	0	0	0
ENSMUSG00000102116	0	0	0	0	0	0	0	0	0	0
ENSMUSG00000109329	0	0	0	0	0	0	0	0	0	0
ENSMUSG00000114766	0	0	0	0	0	0	0	0	0	0
ENSMUSG00000114765	0	0	0	0	0	0	0	0	0	0
ENSMUSG00000114764	0	0	0	0	0	0	0	0	0	0
ENSMUSG00000110659	0	0	0	0	0	0	0	0	3	0
ENSMUSG00000025038	24	3	20	18	9	12	28	16	2	22
ENSMUSG00000099570	0	0	0	0	0	0	0	1	0	0
ENSMUSG00000110653	0	0	0	0	0	3	0	0	0	0
ENSMUSG00000081955	0	0	0	0	0	0	0	0	0	0
ENSMUSG00000091779	0	0	0	0	0	0	0	0	0	0
ENSMUSG00000070304	116	128	119	89	93	174	221	152	119	100
ENSMUSG00000070305	318	249	273	235	202	321	405	302	273	259
ENSMUSG00000070306	0	0	0	2	0	0	3	0	3	0
ENSMUSG00000108054	0	0	0	0	0	0	0	0	0	0
ENSMUSG00000108056	0	0	0	0	0	0	0	0	0	0
ENSMUSG00000108057	1	0	0	0	0	0	0	0	0	0
ENSMUSG00000108050	0	0	0	0	0	0	0	0	0	0
ENSMUSG00000108052	0	0	0	0	0	0	0	0	0	0
ENSMUSG00000108058	0	0	0	0	0	0	0	0	0	0
ENSMUSG00000083023	0	0	0	0	0	0	0	0	0	0
ENSMUSG00000083020	0	0	0	0	0	0	0	0	0	0
ENSMUSG00000083026	0	0	0	0	0	0	0	0	0	0
ENSMUSG00000083025	0	0	0	0	0	0	0	0	0	3
ENSMUSG00000083024	0	0	0	0	0	0	0	0	0	0
ENSMUSG00000083029	0	0	0	0	0	0	0	0	0	0
ENSMUSG00000092377	0	0	0	0	0	0	0	0	0	0
ENSMUSG00000082991	0	0	0	0	0	0	0	0	0	0
ENSMUSG00000082993	0	0	0	0	0	0	0	0	0	0
ENSMUSG00000082994	0	0	0	0	0	0	0	0	0	0
ENSMUSG00000082995	0	0	0	0	0	0	0	0	0	0
ENSMUSG00000082997	0	0	0	0	0	0	0	0	0	0
ENSMUSG00000102113	0	0	0	0	0	0	0	0	0	0
ENSMUSG00000050069	5	6	0	0	11	38	58	6	39	6
ENSMUSG00000107528	0	0	0	0	0	0	0	0	0	0
ENSMUSG00000079103	2	0	0	0	0	0	9	0	0	3
ENSMUSG00000083748	0	0	0	3	0	0	6	0	3	0
ENSMUSG00000083749	0	0	0	0	0	0	0	0	0	0
ENSMUSG00000089322	0	0	0	0	0	0	0	0	0	0
ENSMUSG00000085079	0	0	0	3	0	0	6	0	0	0
ENSMUSG00000022176	0	0	0	0	0	0	0	0	3	0
ENSMUSG00000085073	0	0	0	0	0	0	0	0	0	0
ENSMUSG00000085070	0	0	0	0	0	0	0	0	0	0
ENSMUSG00000085077	0	0	0	0	0	0	0	0	0	0
ENSMUSG00000085074	0	7	3	0	8	0	5	5	0	2
ENSMUSG00000085075	0	0	0	0	0	0	0	0	0	0

ENSMUSG00000084530	0	0	0	0	0	0	0	1	2	3
ENSMUSG00000001661	3	3	0	0	7	0	15	0	9	2
ENSMUSG00000084531	0	0	0	0	0	0	0	0	0	0
ENSMUSG00000026070	133	189	225	128	163	190	407	232	245	146
ENSMUSG00000026073	14	6	18	237	30	67	22	29	27	12
ENSMUSG00000033805	28	5	12	14	10	16	22	42	15	7
ENSMUSG00000041789	8	16	3	3	3	9	12	20	3	13
ENSMUSG00000053185	0	0	0	0	0	0	3	0	0	0
ENSMUSG00000053184	6	0	0	0	0	3	0	3	0	6
ENSMUSG00000081318	0	0	0	0	0	0	0	0	0	0
ENSMUSG00000081313	0	0	0	0	0	0	0	0	0	0
ENSMUSG00000081312	0	0	0	0	0	0	0	0	0	0
ENSMUSG00000081310	0	0	0	0	0	0	0	0	0	0
ENSMUSG00000081317	0	0	0	0	0	0	0	0	0	0
ENSMUSG00000081316	0	0	0	0	0	0	2	0	0	0
ENSMUSG00000081315	0	0	0	0	0	0	0	0	0	0
ENSMUSG00000081314	0	0	16	2	3	3	0	2	0	0
ENSMUSG00000073028	0	0	0	0	0	0	3	0	2	0
ENSMUSG00000073027	0	0	0	0	0	0	0	0	0	0
ENSMUSG00000099842	0	0	0	0	0	0	0	0	0	0
ENSMUSG00000099840	0	0	0	0	0	0	0	0	0	0
ENSMUSG00000099841	0	0	0	0	0	0	0	0	0	0
ENSMUSG00000099846	0	0	0	0	0	0	0	0	0	0
ENSMUSG00000027301	0	3	0	6	0	0	3	0	0	0
ENSMUSG00000099845	0	0	0	0	0	0	0	0	0	0
ENSMUSG00000099849	0	0	0	0	0	0	0	0	0	0
ENSMUSG00000025991	12	6	46	9	3	27	22	9	5	8
ENSMUSG00000084840	0	0	0	0	0	0	0	0	0	0
ENSMUSG00000084842	2	3	5	2	9	8	0	6	3	8
ENSMUSG00000084844	86	50	63	92	24	49	74	98	103	68
ENSMUSG00000084847	0	2	0	0	0	2	0	0	1	0
ENSMUSG00000090527	0	0	0	0	0	0	0	0	0	0
ENSMUSG00000098713	14	19	6	19	16	8	12	15	4	6
ENSMUSG00000099474	0	0	0	3	0	0	12	3	9	3
ENSMUSG00000098717	0	0	0	0	0	0	0	0	0	0
ENSMUSG00000088941	0	0	0	0	0	0	0	0	0	0
ENSMUSG00000090981	0	0	0	0	0	0	0	0	0	0
ENSMUSG00000109101	54	22	35	52	27	28	18	31	28	28
ENSMUSG00000111845	0	1	0	0	0	0	0	0	0	0
ENSMUSG00000044816	4	0	7	3	0	0	3	11	9	1
ENSMUSG00000043164	7	0	0	0	0	6	7	3	0	0
ENSMUSG00000105483	0	0	0	0	0	0	0	0	0	0
ENSMUSG00000109105	12	14	13	7	5	6	13	12	7	16
ENSMUSG00000043168	0	3	0	0	0	0	0	3	6	6
ENSMUSG00000105486	0	0	0	0	0	0	0	0	0	0
ENSMUSG00000077235	0	0	0	0	0	0	0	0	0	0
ENSMUSG00000096890	0	0	0	0	0	0	0	0	0	0
ENSMUSG00000077237	0	0	0	0	0	0	0	0	0	0
ENSMUSG00000077231	0	0	0	0	0	0	0	0	0	0
ENSMUSG00000077230	0	0	0	0	0	0	0	0	0	0
ENSMUSG00000096891	0	0	0	0	0	0	0	0	0	0
ENSMUSG00000094078	0	0	0	0	0	0	0	0	0	0
ENSMUSG00000096893	0	0	0	0	0	0	0	0	0	0
ENSMUSG00000096894	1	0	0	0	0	0	0	0	0	1
ENSMUSG00000096896	0	0	0	0	0	0	0	0	0	0
ENSMUSG00000081877	0	0	0	0	0	0	0	0	0	0
ENSMUSG00000114906	0	2	0	0	0	0	0	0	0	0
ENSMUSG00000072049	0	0	0	0	0	0	0	0	0	0

ENSMUSG00000104532	0	0	0	0	0	0	0	0	0	0
ENSMUSG00000107598	0	0	0	0	0	0	0	0	0	0
ENSMUSG00000046908	30	25	38	57	34	72	86	55	64	55
ENSMUSG00000082039	0	0	0	0	3	0	0	0	0	0
ENSMUSG00000082038	0	0	0	0	0	0	0	0	0	0
ENSMUSG00000082033	0	0	0	0	0	0	0	0	0	0
ENSMUSG00000082032	0	0	0	0	0	1	0	0	0	1
ENSMUSG00000087003	0	2	0	0	0	0	0	0	0	0
ENSMUSG00000082030	0	0	0	0	0	0	0	0	0	0
ENSMUSG00000082037	0	0	0	0	0	0	0	0	0	0
ENSMUSG00000082036	3	13	4	3	0	1	0	3	0	3
ENSMUSG00000087007	0	0	2	0	1	0	0	0	0	0
ENSMUSG00000101376	0	0	0	0	0	0	0	0	0	0
ENSMUSG00000101377	0	0	0	0	0	0	0	0	0	0
ENSMUSG00000101374	0	0	0	0	0	0	0	0	0	0
ENSMUSG00000101375	0	0	0	0	0	0	0	0	0	0
ENSMUSG00000101372	0	0	0	0	0	0	0	0	0	0
ENSMUSG00000101373	0	0	0	0	0	0	0	0	0	0
ENSMUSG00000101371	0	6	0	0	0	0	0	0	0	0
ENSMUSG00000037913	22	13	24	32	0	3	31	18	51	19
ENSMUSG00000071531	18	8	11	16	15	15	18	0	33	24
ENSMUSG00000103201	0	3	6	3	12	3	3	0	3	3
ENSMUSG00000103202	0	3	0	0	0	0	0	0	0	3
ENSMUSG00000103204	0	0	0	3	0	5	0	0	0	3
ENSMUSG00000043833	41	25	24	37	23	56	80	49	67	37
ENSMUSG00000103209	0	0	0	0	0	0	0	0	0	0
ENSMUSG00000095741	0	0	0	0	0	0	0	0	0	0
ENSMUSG00000055643	0	0	0	0	0	0	0	0	0	0
ENSMUSG00000113938	0	0	0	0	0	0	0	0	0	0
ENSMUSG00000070526	132	115	118	136	56	107	171	95	112	125
ENSMUSG00000093184	0	0	0	0	0	0	0	0	0	0
ENSMUSG00000089366	0	0	0	0	0	0	0	0	0	0
ENSMUSG00000075099	0	0	0	0	0	0	0	0	0	0
ENSMUSG00000089363	0	0	0	0	0	0	0	0	0	0
ENSMUSG00000075093	0	0	0	0	0	0	3	0	0	0
ENSMUSG00000075092	0	0	0	0	0	0	0	0	0	0
ENSMUSG00000075091	0	0	0	0	0	0	0	0	0	0
ENSMUSG00000075090	0	0	0	0	0	0	0	0	0	0
ENSMUSG00000075097	0	0	0	0	0	0	0	0	0	0
ENSMUSG00000075095	0	0	0	0	0	0	0	0	0	0
ENSMUSG00000075094	0	0	0	0	0	0	0	0	0	0
ENSMUSG00000022218	46	39	41	86	37	69	52	37	55	43
ENSMUSG00000022211	174	157	173	168	150	144	104	123	116	147
ENSMUSG00000022212	3	0	0	3	5	0	6	10	11	5
ENSMUSG00000102552	0	0	0	0	0	0	0	0	0	0
ENSMUSG00000102551	0	0	0	0	0	0	0	0	0	0
ENSMUSG00000102550	2	6	0	0	0	0	0	0	2	2
ENSMUSG00000084066	0	0	0	0	0	0	0	0	0	0
ENSMUSG00000089095	0	0	0	0	0	0	0	0	0	0
ENSMUSG00000089097	0	0	0	0	0	0	0	0	0	0
ENSMUSG00000089098	0	0	0	0	0	0	0	0	0	0
ENSMUSG00000084068	0	0	0	0	0	0	0	0	0	0
ENSMUSG00000084069	2	0	0	0	1	0	0	0	2	0
ENSMUSG00000068890	0	0	0	0	0	0	0	0	0	0
ENSMUSG00000065866	0	0	0	0	0	0	0	0	0	0
ENSMUSG00000065865	0	0	0	0	0	0	0	0	0	0
ENSMUSG00000099022	0	0	0	1	0	0	0	0	0	0
ENSMUSG00000095744	0	0	0	0	0	0	0	0	0	0

ENSMUSG00000035429	6	0	0	3	0	3	0	0	0	0
ENSMUSG00000064408	0	0	0	0	0	0	0	0	0	0
ENSMUSG00000060988	0	0	0	0	0	0	0	0	0	3
ENSMUSG00000064407	0	0	0	0	0	0	0	0	0	0
ENSMUSG00000060985	0	6	0	6	3	3	0	0	3	0
ENSMUSG00000035427	0	0	0	0	0	0	0	0	0	0
ENSMUSG00000064400	0	0	0	0	0	0	0	0	0	0
ENSMUSG00000107008	0	0	0	0	0	0	6	6	0	0
ENSMUSG00000107006	0	0	0	0	0	0	0	0	0	0
ENSMUSG00000107004	0	0	0	0	0	0	0	0	0	0
ENSMUSG00000107005	0	0	0	0	0	0	6	0	0	0
ENSMUSG00000079666	0	0	0	0	0	0	0	0	0	0
ENSMUSG00000023169	390	729	501	535	442	668	1636	881	650	606
ENSMUSG00000023165	0	0	0	0	0	0	0	0	0	0
ENSMUSG00000113932	0	0	0	0	0	0	0	0	0	0
ENSMUSG00000104474	0	0	0	2	0	0	0	0	0	0
ENSMUSG00000058975	146	55	180	54	72	107	97	72	156	127
ENSMUSG00000058976	0	0	0	0	0	0	3	0	0	0
ENSMUSG00000085330	0	0	0	3	0	0	0	0	0	0
ENSMUSG00000085333	0	0	0	0	0	0	0	3	0	0
ENSMUSG00000097181	0	0	0	0	0	0	0	0	0	0
ENSMUSG00000062585	176	137	128	286	138	165	258	154	264	207
ENSMUSG00000074059	0	0	0	0	0	0	0	0	3	0
ENSMUSG00000113930	7	6	15	21	6	4	8	11	9	13
ENSMUSG00000088578	0	0	0	0	0	0	0	0	0	0
ENSMUSG00000088579	0	0	0	0	0	0	0	0	0	0
ENSMUSG00000088574	0	0	0	0	0	0	0	0	0	0
ENSMUSG00000088575	0	0	1	0	0	0	1	0	0	1
ENSMUSG00000088570	0	0	0	0	0	0	0	0	0	0
ENSMUSG00000088571	0	0	0	0	0	0	0	0	0	0
ENSMUSG00000088572	0	0	0	0	0	0	0	0	0	0
ENSMUSG00000113937	0	0	0	0	0	0	0	0	0	0
ENSMUSG00000069385	0	0	0	0	0	0	0	0	0	0
ENSMUSG00000077834	0	0	0	0	0	0	0	0	1	0
ENSMUSG00000036882	16	6	10	5	13	10	21	19	8	15
ENSMUSG00000103785	0	0	0	0	2	0	0	1	0	0
ENSMUSG00000103787	0	0	0	0	0	0	0	0	0	0
ENSMUSG00000113039	0	0	0	0	0	0	0	0	0	0
ENSMUSG00000103781	0	0	0	0	0	0	0	0	0	0
ENSMUSG00000103782	0	0	0	0	0	0	0	0	0	0
ENSMUSG00000061637	0	0	0	0	0	0	0	0	0	0
ENSMUSG00000034773	11	6	21	24	12	15	13	7	6	6
ENSMUSG00000113031	3	0	0	0	0	3	2	2	0	4
ENSMUSG00000103788	0	0	0	0	0	0	0	0	0	0
ENSMUSG00000034777	3	0	3	1	3	0	6	0	0	3
ENSMUSG00000038255	0	0	0	0	0	0	0	0	0	0
ENSMUSG00000034486	0	3	0	9	2	3	0	3	15	3
ENSMUSG00000063672	0	0	0	0	0	3	0	0	3	0
ENSMUSG00000034488	200	112	192	144	174	136	111	134	146	139
ENSMUSG00000016982	0	0	3	4	4	5	6	7	3	0
ENSMUSG00000051160	0	0	0	0	0	0	0	0	0	0
ENSMUSG00000059319	0	0	0	2	0	0	0	0	0	0
ENSMUSG00000029151	1	0	0	3	0	6	9	9	9	8
ENSMUSG00000029155	20	6	6	9	14	30	18	17	26	38
ENSMUSG00000029154	0	0	0	3	3	0	0	0	3	0
ENSMUSG00000106669	0	0	0	0	0	0	0	0	0	0
ENSMUSG00000106667	0	0	0	0	0	0	4	0	0	0
ENSMUSG00000106660	0	0	0	0	0	0	0	0	0	0

ENSMUSG00000114569	0	0	0	0	0	0	0	0	0	0
ENSMUSG00000109521	0	0	0	0	0	0	0	0	0	0
ENSMUSG00000109520	0	0	0	0	0	0	0	0	0	0
ENSMUSG00000109527	0	0	0	0	0	0	0	0	0	0
ENSMUSG00000109526	0	0	0	0	0	0	0	0	0	0
ENSMUSG00000109525	0	0	0	0	0	0	0	0	0	0
ENSMUSG00000113950	0	0	0	0	0	0	0	0	0	0
ENSMUSG00000114561	0	0	0	0	0	0	3	2	0	0
ENSMUSG00000114560	0	0	0	0	0	0	0	0	0	0
ENSMUSG00000109529	0	0	0	0	3	0	0	0	0	0
ENSMUSG00000109528	0	0	0	0	0	0	0	0	0	0
ENSMUSG00000114565	0	0	0	0	0	0	0	0	0	0
ENSMUSG00000114564	0	0	0	0	0	0	0	0	0	0
ENSMUSG00000114567	0	0	0	0	0	0	0	0	0	0
ENSMUSG00000114566	0	3	0	0	0	0	0	0	0	0
ENSMUSG00000100846	0	0	0	0	0	0	0	0	0	3
ENSMUSG00000100843	3	0	3	0	0	0	5	0	0	0
ENSMUSG00000100842	0	0	0	0	0	0	0	0	0	0
ENSMUSG00000100849	0	0	0	0	0	0	0	0	0	0
ENSMUSG00000100848	0	0	0	0	0	0	0	0	0	0
ENSMUSG00000108692	0	0	0	0	0	0	0	0	0	0
ENSMUSG00000108693	7	16	13	9	7	13	21	17	20	18
ENSMUSG00000112811	5	2	6	11	2	3	2	6	6	0
ENSMUSG00000112810	0	3	0	0	3	0	0	0	0	2
ENSMUSG00000112817	0	0	0	0	0	0	0	0	0	0
ENSMUSG00000108697	0	0	0	0	0	0	0	0	0	0
ENSMUSG00000112815	2	0	1	0	0	0	0	0	0	0
ENSMUSG00000108695	0	0	0	0	0	1	0	0	0	9
ENSMUSG00000091923	0	0	0	0	0	0	0	0	0	0
ENSMUSG00000091924	0	0	0	0	0	0	0	0	0	0
ENSMUSG00000105318	0	0	0	0	0	0	0	0	0	0
ENSMUSG00000105319	0	0	0	0	0	0	0	0	0	0
ENSMUSG00000105314	0	0	0	0	0	0	0	0	0	0
ENSMUSG00000105315	0	0	0	0	0	0	3	0	0	0
ENSMUSG00000110712	3	0	0	0	3	0	0	0	0	0
ENSMUSG00000110713	0	0	0	0	0	0	0	0	0	0
ENSMUSG00000066914	0	0	0	0	0	0	0	0	0	0
ENSMUSG00000058194	0	0	0	0	0	0	0	0	0	0
ENSMUSG00000058192	483	668	275	465	178	475	724	616	663	604
ENSMUSG00000110714	0	0	0	0	0	0	0	0	0	0
ENSMUSG00000050343	0	0	0	0	0	0	0	0	0	0
ENSMUSG00000096114	0	0	0	0	0	0	0	0	0	0
ENSMUSG00000095509	0	0	0	0	0	0	0	0	0	0
ENSMUSG00000096117	0	0	0	0	0	0	0	0	0	0
ENSMUSG00000096658	0	0	0	0	0	0	0	0	0	0
ENSMUSG00000023949	0	0	0	0	0	0	0	0	0	0
ENSMUSG00000096654	0	0	0	0	0	0	0	0	0	0
ENSMUSG00000023945	0	3	0	6	26	0	0	0	0	0
ENSMUSG00000096652	0	0	0	0	0	0	0	0	0	0
ENSMUSG00000023943	0	0	0	0	0	0	0	0	0	0
ENSMUSG00000096650	0	0	0	0	0	0	0	0	0	0
ENSMUSG00000096651	1	0	0	0	0	0	0	0	0	0
ENSMUSG00000111748	15	15	15	29	15	3	49	22	35	12
ENSMUSG00000111749	0	0	0	0	0	0	0	0	0	0
ENSMUSG00000111747	0	0	0	0	0	0	0	0	0	0
ENSMUSG00000026272	0	0	0	0	0	3	0	0	0	0
ENSMUSG00000026271	91	87	63	249	51	101	109	63	106	71
ENSMUSG00000113870	0	0	0	0	0	0	0	0	0	0

ENSMUSG00000095505	0	0	0	0	0	0	0	0	0	0
ENSMUSG00000064876	0	0	0	0	0	0	0	0	0	0
ENSMUSG00000064872	0	0	0	0	0	0	0	0	0	0
ENSMUSG00000083779	0	0	0	0	0	0	0	0	3	0
ENSMUSG00000111049	0	0	0	0	0	0	0	0	0	0
ENSMUSG00000111048	0	0	0	0	0	0	0	0	0	0
ENSMUSG00000095358	0	0	0	0	0	0	0	0	0	0
ENSMUSG00000097036	0	0	0	0	0	0	0	0	2	0
ENSMUSG00000111396	0	0	0	0	0	0	0	0	0	0
ENSMUSG00000103256	0	0	0	0	0	0	0	0	0	0
ENSMUSG00000111041	0	0	0	0	0	0	0	0	0	0
ENSMUSG00000095353	0	0	0	0	0	0	0	0	0	0
ENSMUSG00000095356	0	0	0	0	0	0	0	0	0	0
ENSMUSG00000095357	0	0	0	0	0	0	0	0	0	0
ENSMUSG00000111047	0	0	0	0	0	0	0	0	0	0
ENSMUSG00000111046	0	0	0	0	0	0	0	0	0	0
ENSMUSG00000081151	0	0	0	0	0	0	0	0	0	0
ENSMUSG00000081150	0	0	0	0	0	0	0	0	0	0
ENSMUSG00000081155	0	0	0	0	0	0	0	0	0	0
ENSMUSG00000081154	0	0	0	0	0	0	0	0	0	0
ENSMUSG00000107044	3	3	0	3	3	0	6	0	0	3
ENSMUSG00000087563	0	0	0	0	0	0	0	0	0	0
ENSMUSG00000088394	0	0	0	0	0	0	0	0	0	0
ENSMUSG00000056399	0	18	3	3	0	0	9	0	3	5
ENSMUSG00000088395	0	0	0	0	0	0	0	0	0	0
ENSMUSG00000107049	3	0	0	0	0	0	0	0	0	0
ENSMUSG00000090728	0	0	0	0	0	0	0	0	0	0
ENSMUSG00000088397	0	0	0	0	0	0	0	0	0	0
ENSMUSG00000090722	12	15	20	4	15	18	27	10	8	6
ENSMUSG00000094409	0	0	0	0	0	0	0	0	0	0
ENSMUSG00000094408	0	0	0	0	0	0	0	0	0	0
ENSMUSG00000042219	0	0	0	0	0	0	0	0	0	0
ENSMUSG00000094401	0	0	0	0	0	0	0	0	0	0
ENSMUSG00000056023	0	0	3	0	0	0	3	0	3	0
ENSMUSG00000044903	3	0	3	3	0	0	0	0	0	3
ENSMUSG00000108760	0	0	0	0	0	0	0	0	0	0
ENSMUSG00000090897	0	0	0	0	0	0	0	0	0	0
ENSMUSG00000090894	0	0	0	0	0	0	0	0	0	0
ENSMUSG00000028362	29	12	26	15	6	29	72	20	64	29
ENSMUSG00000108767	12	13	5	0	0	2	3	14	3	2
ENSMUSG00000077039	0	0	0	0	0	0	0	0	0	0
ENSMUSG00000055612	129	130	135	138	75	79	189	204	139	153
ENSMUSG00000112020	0	2	0	0	2	3	8	3	3	3
ENSMUSG00000055610	0	0	0	0	0	0	0	0	0	0
ENSMUSG00000112027	1	2	0	3	2	1	1	4	1	2
ENSMUSG00000112024	0	0	0	0	0	0	0	0	0	0
ENSMUSG00000112028	0	0	0	0	0	0	2	0	0	0
ENSMUSG00000046463	56	67	84	67	26	61	106	81	103	35
ENSMUSG00000086315	0	11	6	0	0	0	6	4	3	0
ENSMUSG00000038165	0	0	0	0	0	0	0	0	0	0
ENSMUSG00000086319	0	0	0	0	0	0	0	3	0	0
ENSMUSG00000092767	0	0	0	0	0	0	0	0	0	0
ENSMUSG00000092762	1	1	1	1	1	1	0	1	0	1
ENSMUSG00000092763	1	0	1	0	1	1	0	0	1	0
ENSMUSG00000092761	0	0	1	0	0	0	1	0	0	0
ENSMUSG00000077784	0	0	0	0	0	0	0	0	0	0
ENSMUSG00000077786	0	0	0	0	0	0	0	0	0	0
ENSMUSG00000077783	0	0	0	0	0	0	0	0	0	0

ENSMUSG00000096806	0	0	0	0	0	0	0	0	0	0
ENSMUSG00000049307	150	137	137	203	141	127	258	131	201	205
ENSMUSG00000034730	6	9	15	32	5	19	14	12	5	24
ENSMUSG00000034732	12	7	11	9	13	10	47	15	14	14
ENSMUSG00000092490	41	38	54	41	33	35	68	48	62	68
ENSMUSG00000113373	0	0	0	0	0	0	0	0	0	0
ENSMUSG00000113372	0	0	0	0	0	0	0	0	0	0
ENSMUSG00000113375	0	0	3	0	6	0	0	0	0	0
ENSMUSG00000113374	0	0	0	0	0	0	0	0	0	0
ENSMUSG00000113377	0	0	0	0	0	0	1	0	0	0
ENSMUSG00000113376	0	0	0	0	0	0	0	0	0	0
ENSMUSG00000092499	0	0	0	0	0	0	0	0	0	0
ENSMUSG00000109602	7	12	0	15	6	3	12	0	11	8
ENSMUSG00000076129	0	0	0	0	0	0	0	0	0	0
ENSMUSG00000106505	0	0	0	0	0	0	0	0	0	0
ENSMUSG00000106501	3	0	0	0	0	0	0	0	0	0
ENSMUSG00000106502	0	0	0	0	0	0	0	0	0	0
ENSMUSG00000106503	0	0	0	0	0	0	0	0	0	0
ENSMUSG00000106509	0	0	0	0	0	0	0	0	0	0
ENSMUSG00000100386	0	0	0	0	4	0	0	0	0	0
ENSMUSG00000082543	0	2	3	4	3	2	2	0	0	5
ENSMUSG00000082540	0	0	0	0	0	0	0	0	0	0
ENSMUSG00000082547	0	0	0	0	0	0	0	0	0	0
ENSMUSG00000082546	0	0	0	0	0	0	0	0	0	0
ENSMUSG00000082545	0	0	0	6	0	0	0	0	3	0
ENSMUSG00000082544	0	0	0	0	0	0	0	0	3	0
ENSMUSG00000082548	0	0	0	0	0	0	0	0	0	0
ENSMUSG00000108404	0	0	0	0	0	0	0	0	0	0
ENSMUSG00000084262	0	0	0	0	0	0	0	0	0	0
ENSMUSG00000084267	0	0	0	0	0	0	0	0	0	0
ENSMUSG00000091468	0	0	0	0	0	0	0	0	0	0
ENSMUSG00000100389	9	0	3	7	3	13	4	11	1	9
ENSMUSG00000025202	3	18	22	4	6	12	42	6	30	15
ENSMUSG00000041272	66	48	55	42	44	44	61	79	85	40
ENSMUSG00000080949	0	0	0	0	0	0	0	0	0	0
ENSMUSG00000080948	0	3	0	0	0	0	0	0	0	0
ENSMUSG00000080944	0	0	0	0	0	0	0	0	0	0
ENSMUSG00000080947	0	0	0	0	0	0	0	0	0	0
ENSMUSG00000080943	0	0	0	0	0	0	0	0	0	0
ENSMUSG00000099146	0	0	0	0	6	0	0	7	2	0
ENSMUSG00000099149	0	0	0	0	0	0	0	0	0	0
ENSMUSG00000099148	0	0	0	5	0	0	9	0	11	3
ENSMUSG00000057179	0	0	0	0	0	0	0	0	0	0
ENSMUSG00000112082	3	0	0	0	3	0	0	4	11	0
ENSMUSG00000075524	0	0	0	0	0	0	0	0	0	0
ENSMUSG00000044170	0	0	0	0	0	0	0	0	0	0
ENSMUSG00000000706	3	6	2	3	0	3	6	0	3	6
ENSMUSG00000044172	3	20	9	8	6	22	106	42	37	15
ENSMUSG00000044176	2	0	0	0	0	0	0	0	0	0
ENSMUSG00000070173	0	0	0	0	0	0	0	0	0	0
ENSMUSG00000070175	0	0	0	0	0	0	0	0	0	0
ENSMUSG00000085139	18	55	33	26	30	41	82	48	14	33
ENSMUSG00000085135	13	14	0	2	0	4	8	5	3	3
ENSMUSG00000085130	0	0	0	0	0	0	0	0	0	0
ENSMUSG00000074695	0	0	0	0	0	0	0	0	0	0
ENSMUSG00000109425	0	0	0	0	0	0	0	0	0	0
ENSMUSG00000067522	0	0	0	0	0	3	0	6	0	0
ENSMUSG00000039329	0	0	3	0	0	0	3	0	0	3

ENSMUSG00000102304	0	0	0	0	0	0	0	0	0	0
ENSMUSG00000082458	0	0	0	0	0	0	0	0	0	0
ENSMUSG00000045225	1	0	6	1	0	1	3	0	0	0
ENSMUSG00000084030	0	0	0	0	0	0	0	0	0	0
ENSMUSG00000102303	0	0	0	0	0	0	0	0	0	0
ENSMUSG00000103815	0	0	0	0	0	0	0	0	0	0
ENSMUSG00000103814	0	0	0	0	0	0	2	0	0	0
ENSMUSG00000103817	0	0	0	0	0	0	0	0	0	0
ENSMUSG00000103816	0	0	0	0	0	0	0	0	0	0
ENSMUSG00000103811	3	18	13	15	6	8	20	15	12	7
ENSMUSG00000103810	0	0	0	0	0	0	0	0	0	0
ENSMUSG00000103813	0	0	0	0	0	0	0	0	0	0
ENSMUSG00000103812	0	0	0	0	0	0	0	0	0	0
ENSMUSG00000034057	87	100	104	56	106	97	49	100	70	63
ENSMUSG00000099652	0	0	0	0	0	0	0	0	0	0
ENSMUSG00000095845	12	3	0	4	3	5	3	0	5	9
ENSMUSG00000089934	0	0	0	0	0	2	0	5	0	3
ENSMUSG00000031497	146	202	153	277	246	197	360	356	238	170
ENSMUSG00000031492	0	0	0	3	0	0	0	0	0	0
ENSMUSG00000031491	0	0	0	0	0	0	0	0	0	0
ENSMUSG00000020912	0	0	0	0	0	0	0	0	0	0
ENSMUSG00000061082	0	0	0	0	0	0	0	0	6	0
ENSMUSG00000110406	0	0	0	0	0	0	0	0	0	0
ENSMUSG00000065505	1	1	1	0	0	1	1	1	1	2
ENSMUSG00000108843	0	0	0	0	0	0	0	0	0	0
ENSMUSG00000089667	0	0	0	0	0	0	0	0	0	0
ENSMUSG00000108908	0	0	0	0	0	0	0	0	0	0
ENSMUSG00000108909	0	0	0	0	0	0	0	0	3	0
ENSMUSG00000031727	0	0	0	0	0	0	3	0	3	0
ENSMUSG00000108902	0	0	0	3	0	0	0	0	0	0
ENSMUSG00000108903	0	0	0	0	0	0	0	0	0	0
ENSMUSG00000108900	0	0	3	0	0	3	9	3	0	0
ENSMUSG00000108906	0	0	0	0	0	0	3	0	0	0
ENSMUSG00000108904	0	0	0	0	0	0	0	0	0	0
ENSMUSG00000108905	0	0	0	0	0	0	0	0	0	0
ENSMUSG00000093860	0	0	0	0	0	0	0	0	0	0
ENSMUSG00000089664	0	0	0	0	2	0	0	0	0	0
ENSMUSG00000089663	35	42	52	64	89	56	46	41	63	43
ENSMUSG00000043314	0	0	0	0	0	0	0	0	0	0
ENSMUSG00000043310	0	0	0	0	0	0	0	0	0	0
ENSMUSG00000043312	0	0	0	0	0	0	0	0	0	0
ENSMUSG00000102384	0	0	0	0	0	0	0	0	0	4
ENSMUSG00000043319	0	0	0	0	0	0	0	0	0	0
ENSMUSG00000083324	0	0	0	0	0	0	0	0	0	0
ENSMUSG00000085909	0	0	0	0	0	0	0	0	0	0
ENSMUSG00000085905	0	0	0	0	0	0	0	0	0	0
ENSMUSG00000033383	0	3	3	0	0	6	6	0	3	3
ENSMUSG00000033386	318	289	338	369	215	310	345	323	441	317
ENSMUSG00000110403	9	9	10	6	5	10	12	11	9	14
ENSMUSG00000087219	0	0	0	3	3	0	0	0	0	0
ENSMUSG00000087210	0	0	0	0	0	0	0	0	0	0
ENSMUSG00000087211	0	0	0	0	0	0	0	0	0	0
ENSMUSG00000087214	0	0	0	0	0	0	0	0	0	0
ENSMUSG00000087215	0	0	0	0	0	0	0	0	0	0
ENSMUSG00000087216	0	0	0	0	3	0	0	0	0	0
ENSMUSG00000087217	0	0	0	3	0	0	0	2	0	3
ENSMUSG00000101505	3	0	0	0	0	2	1	1	6	0
ENSMUSG00000101504	0	0	0	0	0	0	0	0	0	0

ENSMUSG00000101507	0	0	0	0	0	0	0	0	0	0
ENSMUSG00000101506	0	0	0	0	0	0	0	0	0	0
ENSMUSG00000101501	1	3	0	0	7	3	2	3	10	8
ENSMUSG00000101500	0	0	0	0	0	0	0	0	0	0
ENSMUSG00000040663	66	71	71	52	70	62	110	81	74	81
ENSMUSG00000037761	551	414	585	518	466	451	562	600	513	561
ENSMUSG00000101508	0	0	0	0	0	0	0	0	0	0
ENSMUSG00000099765	0	0	0	0	0	0	0	0	0	0
ENSMUSG00000100674	0	0	0	0	0	0	0	0	0	0
ENSMUSG00000100675	0	0	0	0	0	0	0	0	0	0
ENSMUSG00000100676	0	0	0	0	1	0	0	0	1	0
ENSMUSG00000100677	0	0	0	0	0	0	0	0	0	0
ENSMUSG00000100670	0	0	0	0	0	0	0	0	0	0
ENSMUSG00000100671	0	0	0	0	0	0	0	0	0	0
ENSMUSG00000100673	0	0	0	0	0	0	0	0	0	0
ENSMUSG00000100678	0	0	0	0	0	0	0	0	0	0
ENSMUSG00000059901	101	136	118	103	149	182	116	168	124	177
ENSMUSG00000112473	0	0	0	0	0	0	0	0	0	0
ENSMUSG00000107908	0	0	0	0	0	0	0	0	0	0
ENSMUSG00000107907	0	0	0	0	0	0	0	0	0	0
ENSMUSG00000107906	0	0	0	0	0	2	2	2	2	1
ENSMUSG00000107905	0	0	0	0	0	0	0	0	0	0
ENSMUSG00000059908	0	0	12	0	0	4	0	0	0	3
ENSMUSG00000107903	0	0	0	0	0	0	0	0	0	0
ENSMUSG00000107902	0	0	0	0	0	0	0	2	0	0
ENSMUSG00000107901	0	0	0	0	0	0	0	0	0	0
ENSMUSG00000107900	0	0	0	0	0	0	0	0	0	0
ENSMUSG00000084747	0	0	0	0	0	0	0	0	0	0
ENSMUSG00000084746	0	0	0	0	0	0	0	0	0	0
ENSMUSG00000089116	0	0	0	0	0	0	0	0	0	0
ENSMUSG00000084740	0	0	0	0	0	0	0	0	0	0
ENSMUSG00000089119	0	0	0	0	0	0	0	0	0	0
ENSMUSG00000089118	0	0	0	0	0	0	0	0	0	0
ENSMUSG00000084748	0	0	0	0	0	0	0	0	0	0
ENSMUSG00000016942	10	13	10	6	20	23	24	22	10	26
ENSMUSG00000022408	15	11	15	18	15	15	18	36	17	12
ENSMUSG00000095155	0	0	0	0	0	0	0	0	0	0
ENSMUSG00000018654	154	158	147	396	177	221	286	247	295	189
ENSMUSG00000018656	3	3	0	0	0	0	7	0	4	5
ENSMUSG00000020317	0	0	0	0	0	0	0	0	0	0
ENSMUSG00000020310	0	3	0	0	0	3	0	3	0	0
ENSMUSG00000090308	0	0	0	0	0	0	0	0	2	0
ENSMUSG00000081282	2	3	1	12	2	15	11	0	6	6
ENSMUSG00000112221	0	0	0	0	0	0	0	0	0	0
ENSMUSG00000112222	0	0	0	0	0	0	0	0	0	1
ENSMUSG00000112223	0	0	0	0	0	0	0	0	0	0
ENSMUSG00000112224	0	0	0	0	0	0	0	0	0	0
ENSMUSG00000112225	0	0	0	0	0	0	0	0	0	0
ENSMUSG00000112226	11	12	10	8	2	3	9	6	3	6
ENSMUSG00000112227	0	0	0	0	0	0	0	0	0	0
ENSMUSG00000112228	0	0	0	0	0	0	0	0	0	0
ENSMUSG00000102747	0	0	0	3	0	0	0	0	6	0
ENSMUSG00000102745	0	0	3	3	0	3	11	3	5	2
ENSMUSG00000102743	0	0	0	0	0	0	0	0	0	0
ENSMUSG00000102740	0	0	0	0	0	0	0	0	0	0
ENSMUSG00000102741	0	0	0	0	0	0	0	0	0	0
ENSMUSG00000099056	0	0	0	0	0	0	0	0	0	0
ENSMUSG00000062464	0	0	0	4	0	0	0	0	0	0

ENSMUSG00000052818	0	0	0	0	0	0	0	0	0	0
ENSMUSG00000052819	0	0	0	0	0	0	6	0	0	0
ENSMUSG00000036962	0	0	0	0	0	3	3	0	0	2
ENSMUSG00000015829	29	15	46	28	29	31	30	24	47	26
ENSMUSG00000112598	0	0	0	0	0	0	0	0	0	0
ENSMUSG00000114523	0	0	0	0	0	0	0	0	0	0
ENSMUSG00000112590	0	0	0	0	0	0	0	0	0	0
ENSMUSG00000114522	0	0	0	0	0	4	0	0	0	0
ENSMUSG00000112596	22	6	10	14	17	22	18	16	26	38
ENSMUSG00000112597	0	0	0	0	0	0	0	0	6	0
ENSMUSG00000029699	37	31	24	38	47	49	49	50	54	38
ENSMUSG00000105439	0	0	0	0	0	0	0	0	0	2
ENSMUSG00000105438	1	0	0	0	0	1	1	0	0	0
ENSMUSG00000105435	0	0	0	0	0	0	0	0	0	0
ENSMUSG00000105437	0	0	2	0	0	0	0	3	0	0
ENSMUSG00000105436	0	0	0	0	0	0	0	0	0	0
ENSMUSG00000029695	154	85	135	142	105	106	429	168	174	130
ENSMUSG00000029697	3	0	0	0	0	0	0	0	0	0
ENSMUSG00000113179	0	0	0	2	0	0	0	0	0	0
ENSMUSG00000038357	14	0	0	30	5	8	4	5	0	0
ENSMUSG00000113175	0	0	0	0	0	0	0	0	0	0
ENSMUSG00000038354	194	247	292	139	185	283	432	317	397	397
ENSMUSG00000113173	0	0	0	0	0	0	0	0	0	0
ENSMUSG00000038352	90	79	75	70	63	83	118	70	145	66
ENSMUSG00000113171	0	0	0	0	0	0	0	0	0	0
ENSMUSG00000020573	542	494	578	613	502	518	1048	593	873	664
ENSMUSG00000042631	0	0	0	0	0	0	0	0	0	0
ENSMUSG00000016552	101	131	98	172	130	216	311	152	176	247
ENSMUSG00000021194	0	0	0	0	0	0	46	0	6	0
ENSMUSG00000039913	52	75	81	53	21	83	62	67	49	100
ENSMUSG00000024114	0	0	0	0	0	0	0	0	0	0
ENSMUSG00000024116	0	0	0	0	0	0	0	0	0	0
ENSMUSG00000034528	0	0	0	0	0	0	0	0	0	0
ENSMUSG00000103595	0	0	0	0	0	0	0	0	0	0
ENSMUSG00000103597	0	1	0	2	1	0	3	6	0	3
ENSMUSG00000103592	0	0	1	0	3	0	2	2	4	3
ENSMUSG00000103598	0	0	0	0	0	0	0	0	0	0
ENSMUSG00000102616	0	0	0	0	0	0	0	0	0	0
ENSMUSG00000112335	0	0	0	0	0	0	1	0	0	0
ENSMUSG00000094327	0	0	0	0	0	2	0	0	0	0
ENSMUSG00000055891	0	0	0	0	0	0	0	0	0	0
ENSMUSG00000051732	0	0	0	0	0	0	0	0	0	0
ENSMUSG00000055895	0	0	0	0	0	0	0	0	0	0
ENSMUSG00000114338	0	0	0	0	0	0	0	0	0	0
ENSMUSG00000114339	0	0	0	0	0	0	0	0	0	0
ENSMUSG00000105295	0	0	0	0	0	0	0	0	0	0
ENSMUSG00000105297	0	0	0	0	0	0	0	7	0	0
ENSMUSG00000105296	10	17	22	10	12	7	22	8	14	3
ENSMUSG00000096281	0	0	0	0	0	0	0	0	0	0
ENSMUSG00000105298	2	0	0	0	0	0	0	0	0	0
ENSMUSG00000096283	0	0	0	0	0	0	0	0	0	0
ENSMUSG00000114334	0	0	0	0	0	0	0	0	0	0
ENSMUSG00000114335	0	0	0	0	0	0	0	0	0	0
ENSMUSG00000114336	0	0	0	0	0	0	0	0	0	0
ENSMUSG00000096286	0	0	0	0	0	0	1	0	0	0
ENSMUSG00000081639	0	0	0	0	0	0	0	0	0	0
ENSMUSG00000041052	6	3	18	54	0	0	12	6	11	11
ENSMUSG00000097484	0	0	0	0	3	3	0	5	0	5

ENSMUSG00000097486	0	0	0	3	3	0	0	0	0	0
ENSMUSG00000108249	0	0	0	0	0	0	0	0	0	0
ENSMUSG00000097481	9	3	5	0	2	0	23	15	13	24
ENSMUSG00000097482	6	4	15	7	10	14	7	12	17	16
ENSMUSG00000097483	0	0	0	0	0	0	0	0	0	0
ENSMUSG00000104744	0	0	0	0	0	0	0	0	0	0
ENSMUSG00000108244	0	0	0	0	0	0	0	0	0	0
ENSMUSG00000108247	0	0	0	0	0	0	0	0	0	0
ENSMUSG00000104747	0	0	0	0	0	0	0	0	0	0
ENSMUSG00000108241	0	0	0	0	0	0	0	0	0	0
ENSMUSG00000108240	0	0	0	0	0	0	0	0	0	0
ENSMUSG00000104742	0	0	0	0	0	0	0	0	0	0
ENSMUSG00000108242	15	8	2	0	0	0	0	0	3	0
ENSMUSG00000091888	0	0	0	0	0	0	0	0	0	0
ENSMUSG00000091887	0	0	0	0	0	0	0	0	0	0
ENSMUSG00000100332	0	0	0	0	0	0	0	0	0	0
ENSMUSG00000083330	0	0	0	0	0	0	0	0	0	0
ENSMUSG00000002897	571	578	555	1163	575	1325	1283	936	1212	789
ENSMUSG00000097738	4	4	0	3	0	0	4	0	2	4
ENSMUSG00000097739	0	0	0	0	0	0	0	0	0	0
ENSMUSG00000097736	13	0	3	3	0	0	10	0	0	0
ENSMUSG00000097735	0	0	0	0	0	0	0	0	2	0
ENSMUSG00000050066	0	3	3	0	0	1	10	7	0	0
ENSMUSG00000050063	0	0	0	0	0	0	0	0	0	0
ENSMUSG00000091771	0	0	0	0	0	0	0	0	0	0
ENSMUSG00000091775	0	0	0	0	0	0	0	0	0	0
ENSMUSG00000091776	0	0	0	0	0	0	1	0	0	0
ENSMUSG00000083969	0	0	0	0	0	0	0	0	0	0
ENSMUSG00000083963	0	0	0	0	0	0	0	0	0	0
ENSMUSG00000083960	0	0	0	0	0	0	0	0	0	0
ENSMUSG00000083961	1	1	0	0	0	0	0	0	4	0
ENSMUSG00000083966	1	2	5	8	0	1	7	10	3	0
ENSMUSG00000083967	0	0	0	0	0	0	0	0	0	0
ENSMUSG00000114592	0	0	0	0	0	0	0	0	0	0
ENSMUSG00000057499	4	2	2	3	4	2	0	2	3	3
ENSMUSG00000058656	99	115	100	104	100	125	150	81	75	142
ENSMUSG00000044375	15	15	3	11	3	11	24	0	16	9
ENSMUSG00000054763	13	3	20	9	0	12	18	13	15	9
ENSMUSG00000054764	0	0	0	0	0	0	0	0	0	0
ENSMUSG00000058659	0	0	0	0	0	3	0	0	0	0
ENSMUSG00000044378	0	0	0	0	0	0	2	0	0	3
ENSMUSG00000043668	141	143	134	116	127	137	205	126	167	139
ENSMUSG00000046203	0	0	0	0	0	0	0	0	0	0
ENSMUSG00000046204	9	3	6	3	5	15	12	17	11	14
ENSMUSG00000086128	0	0	0	0	0	0	0	0	0	0
ENSMUSG00000056728	0	0	0	0	0	0	0	0	0	0
ENSMUSG00000010044	7	3	5	12	2	5	6	0	8	5
ENSMUSG00000110521	17	8	0	7	13	14	0	7	16	6
ENSMUSG00000110520	0	0	0	0	0	0	0	0	0	0
ENSMUSG00000110522	0	0	0	0	0	0	0	0	0	0
ENSMUSG00000110524	6	3	0	8	3	7	6	3	11	3
ENSMUSG00000110527	0	0	0	0	0	0	0	0	0	0
ENSMUSG00000110526	0	0	0	0	0	0	0	0	0	0
ENSMUSG00000080430	0	0	0	0	0	0	0	0	0	0
ENSMUSG00000080434	0	0	0	0	0	0	0	0	0	0
ENSMUSG00000080437	0	0	0	0	0	0	0	0	0	0
ENSMUSG00000095259	0	0	0	0	0	0	0	0	0	0
ENSMUSG00000095257	0	0	0	0	0	0	0	0	0	0

ENSMUSG00000095256	0	0	0	0	0	0	0	0	0	0
ENSMUSG00000095255	0	0	0	0	0	0	0	0	0	0
ENSMUSG00000095251	0	0	0	0	0	0	0	0	0	0
ENSMUSG00000095250	0	0	1	0	1	0	2	0	0	0
ENSMUSG00000113951	0	0	0	0	0	0	0	0	0	0
ENSMUSG00000113953	0	0	0	0	0	0	0	0	0	0
ENSMUSG00000113952	0	0	0	0	0	0	0	0	0	0
ENSMUSG00000113955	0	0	0	0	0	0	0	0	0	0
ENSMUSG00000113954	0	0	0	0	0	0	0	0	0	0
ENSMUSG00000113957	0	0	0	0	0	0	0	0	0	0
ENSMUSG00000113956	0	0	0	0	0	0	0	0	0	0
ENSMUSG00000113959	5	18	9	6	24	23	39	46	37	37
ENSMUSG00000026840	148	124	109	93	91	106	300	92	89	125
ENSMUSG00000080732	0	0	0	0	0	0	0	0	0	0
ENSMUSG00000098561	0	0	2	0	0	0	0	0	0	0
ENSMUSG00000098565	0	0	0	0	0	0	0	0	0	0
ENSMUSG00000026669	78	63	106	164	100	8	105	32	44	13
ENSMUSG00000094618	7	5	3	3	11	13	6	9	4	12
ENSMUSG00000094610	0	0	0	0	0	0	0	0	0	0
ENSMUSG00000094612	0	0	0	0	0	0	0	0	0	0
ENSMUSG00000094613	0	0	0	0	0	13	0	0	0	20
ENSMUSG00000042002	3	0	0	0	0	0	0	3	3	0
ENSMUSG00000094616	0	0	0	0	0	0	0	0	0	0
ENSMUSG00000086870	0	0	0	0	0	0	0	0	0	0
ENSMUSG00000086875	0	0	0	0	0	0	0	0	0	0
ENSMUSG00000084806	12	9	8	0	6	7	46	12	26	11
ENSMUSG00000081470	0	0	3	0	0	0	0	0	0	0
ENSMUSG00000081477	0	0	0	0	0	0	0	0	0	0
ENSMUSG00000081474	0	0	0	0	0	0	0	0	0	0
ENSMUSG00000081475	0	0	0	2	0	0	0	0	0	0
ENSMUSG00000081478	0	0	0	0	0	0	0	0	0	0
ENSMUSG00000088307	0	0	0	0	0	0	0	0	1	0
ENSMUSG00000090919	74	96	60	39	41	72	112	68	72	79
ENSMUSG00000092515	0	0	0	1	0	0	0	0	0	0
ENSMUSG00000092514	0	0	0	0	0	0	0	0	0	0
ENSMUSG00000099761	0	0	0	0	0	0	0	0	0	0
ENSMUSG00000077598	2	0	0	0	0	0	0	0	0	0
ENSMUSG00000077599	0	0	0	0	0	0	0	0	0	0
ENSMUSG00000092513	0	0	0	0	0	0	0	0	0	0
ENSMUSG00000092512	1	2	0	6	4	2	1	7	0	0
ENSMUSG00000077594	0	0	0	0	0	0	0	0	0	0
ENSMUSG00000077595	0	0	0	0	2	0	1	3	3	2
ENSMUSG00000099768	0	0	0	0	0	0	0	0	0	0
ENSMUSG00000092519	0	0	0	0	0	0	0	0	0	0
ENSMUSG00000092518	0	0	0	0	0	0	0	2	0	0
ENSMUSG00000055403	0	0	3	3	0	0	0	0	0	0
ENSMUSG00000055408	0	0	0	0	0	0	0	0	0	0
ENSMUSG00000055409	0	0	16	0	0	0	0	0	0	0
ENSMUSG00000086296	26	15	27	15	29	33	26	38	22	36
ENSMUSG00000086291	25	15	20	7	4	21	18	11	12	13
ENSMUSG00000030031	392	366	436	376	405	228	284	323	408	276
ENSMUSG00000072419	0	0	0	0	0	0	0	0	0	0
ENSMUSG00000094323	0	0	0	0	0	0	0	0	0	0
ENSMUSG00000022542	0	0	0	0	0	0	0	3	0	0
ENSMUSG00000086124	0	0	0	0	0	0	0	6	6	0
ENSMUSG00000086127	0	3	0	3	3	0	0	0	6	0
ENSMUSG00000086126	0	0	0	0	0	0	0	0	0	0
ENSMUSG00000043664	37	53	32	67	27	19	67	49	52	56

ENSMUSG00000043661	10	3	12	6	2	6	8	12	15	0
ENSMUSG00000098685	0	0	0	0	0	0	0	0	0	0
ENSMUSG00000000003	0	0	0	0	0	0	0	0	0	0
ENSMUSG00000098683	0	0	0	0	0	0	0	0	0	0
ENSMUSG00000076241	0	0	0	1	0	0	0	1	0	0
ENSMUSG00000084184	2	0	0	0	0	0	0	0	0	0
ENSMUSG00000113064	0	0	0	0	0	0	0	0	0	0
ENSMUSG00000087457	0	0	0	0	0	0	0	0	0	0
ENSMUSG00000087454	0	1	0	0	0	0	0	0	0	0
ENSMUSG00000087455	0	0	0	0	0	0	0	0	0	2
ENSMUSG00000087452	0	0	0	0	0	0	0	0	0	0
ENSMUSG00000087450	0	0	0	0	0	0	0	3	0	3
ENSMUSG00000070815	0	0	0	0	0	0	0	0	0	0
ENSMUSG00000070816	0	0	0	0	0	0	0	0	0	0
ENSMUSG00000070817	0	0	0	0	0	0	0	0	0	0
ENSMUSG00000070810	0	0	0	0	0	0	0	0	0	0
ENSMUSG00000070811	0	0	0	0	0	0	0	0	0	0
ENSMUSG00000087459	0	0	0	0	0	0	0	0	0	0
ENSMUSG00000037568	35	25	49	20	30	16	38	47	40	42
ENSMUSG00000083513	0	0	0	0	0	0	0	0	0	0
ENSMUSG00000083510	0	0	0	0	0	0	0	0	0	0
ENSMUSG00000083517	0	0	0	0	0	0	0	0	0	0
ENSMUSG00000045842	3	8	9	6	6	6	11	9	2	3
ENSMUSG00000083515	0	0	0	0	0	0	0	0	0	0
ENSMUSG00000083514	0	0	0	0	0	0	0	0	0	0
ENSMUSG00000049173	0	0	0	0	2	0	1	2	0	0
ENSMUSG00000083519	0	0	0	0	0	0	0	0	0	0
ENSMUSG00000083518	0	0	3	0	0	0	0	0	0	2
ENSMUSG00000049176	3	3	2	0	12	9	6	3	0	0
ENSMUSG00000088830	0	0	0	0	0	0	0	0	0	0
ENSMUSG00000079439	0	0	0	0	0	0	0	0	0	0
ENSMUSG00000079438	0	0	0	0	0	0	0	0	0	0
ENSMUSG00000105978	0	0	0	0	0	0	0	0	0	0
ENSMUSG00000079433	0	0	0	0	0	0	0	0	0	0
ENSMUSG00000079432	0	0	0	0	0	0	0	0	0	0
ENSMUSG00000087981	0	0	0	0	0	0	0	0	0	0
ENSMUSG00000087982	0	3	0	0	0	0	1	0	0	0
ENSMUSG00000087983	2	0	0	0	0	0	0	0	0	0
ENSMUSG00000087985	0	0	0	0	0	0	0	0	0	0
ENSMUSG00000040314	0	3	0	0	0	0	9	5	0	0
ENSMUSG00000088839	0	0	0	0	0	0	0	0	0	0
ENSMUSG00000040312	202	216	297	232	153	238	464	222	335	182
ENSMUSG00000071149	0	0	0	0	0	0	0	0	0	0
ENSMUSG00000084185	0	0	0	0	0	0	0	0	0	0
ENSMUSG00000040456	0	0	0	3	0	3	0	3	0	0
ENSMUSG00000084189	0	0	0	0	0	0	0	0	0	0
ENSMUSG00000033717	0	6	6	0	3	3	19	3	15	17
ENSMUSG00000103154	0	0	0	0	0	0	0	0	0	0
ENSMUSG00000103155	0	0	0	0	0	0	0	0	0	0
ENSMUSG00000103156	3	0	9	16	0	2	6	7	7	18
ENSMUSG00000081893	0	0	0	0	0	0	0	0	0	0
ENSMUSG00000103150	0	0	0	0	0	0	0	0	0	0
ENSMUSG00000101070	0	0	0	0	0	0	0	0	0	0
ENSMUSG00000112358	10	22	14	29	29	29	24	15	11	18
ENSMUSG00000103153	0	0	0	0	0	0	0	0	0	0
ENSMUSG00000079299	0	0	0	0	0	0	0	0	0	0
ENSMUSG00000079298	42	36	26	82	18	76	143	85	87	64
ENSMUSG00000101074	0	0	0	0	0	0	0	0	0	0

ENSMUSG00000085588	0	0	0	0	0	0	0	0	0	0
ENSMUSG00000109593	0	0	0	0	0	0	0	0	0	0
ENSMUSG00000085582	0	3	3	0	0	6	0	9	0	3
ENSMUSG00000085580	0	0	0	0	0	0	0	0	0	0
ENSMUSG00000085584	18	14	9	20	23	26	17	12	12	3
ENSMUSG00000085585	0	0	0	0	0	0	0	0	0	0
[truncated: 433,700 more chars]
