# Supplementary material for: Characterizing a long-term chronic heart failure model by transcriptomic alterations and monitoring of cardiac remodeling
Source: Aging (Albany NY). 2021 Apr 23;13(10):13585–614. doi: 10.18632/aging.202879 (PMC8202904; doi:10.18632/aging.202879)
Supplement: Supplementary Tables 1-9 [file aging-13-202879-s002.pdf]

SUPPLEMENTARY TABLES

Supplementary Table 1. The 86 genes in the green module of the WGCNA analysis

ENSMUSG00000000776	Polr3d
ENSMUSG00000001774	Chordc1
ENSMUSG00000002981	Clptm1
ENSMUSG00000005483	Dnajb1
ENSMUSG00000007617	Homer1
ENSMUSG00000007721	Ccdc124
ENSMUSG00000007777	0610009B22Rik
ENSMUSG00000007877	Tcap
ENSMUSG00000008668	Rps18
ENSMUSG00000013878	Rnf170
ENSMUSG00000014226	Cacybp
ENSMUSG00000014294	Ndufa2
ENSMUSG00000018906	P4ha2
ENSMUSG00000020092	Pald1
ENSMUSG00000020482	Ccdc117
ENSMUSG00000020827	Mink1
ENSMUSG00000021361	Tmem14c
ENSMUSG00000022237	Ankrd33b
ENSMUSG00000022426	Josd1
ENSMUSG00000022433	Csnk1e
ENSMUSG00000024219	Anks1
ENSMUSG00000024247	Pkdcc
ENSMUSG00000024900	Cpt1a
ENSMUSG00000024966	Stip1
ENSMUSG00000024978	Gpam
ENSMUSG00000025261	Huwe1
ENSMUSG00000025558	Dock9
ENSMUSG00000025907	Rb1cc1
ENSMUSG00000026500	Cox20
ENSMUSG00000026977	07-Mar
ENSMUSG00000027255	Arfgap2
ENSMUSG00000028034	Fubp1
ENSMUSG00000028271	Gtf2b
ENSMUSG00000028318	Polr1e
ENSMUSG00000028427	Aqp7
ENSMUSG00000030653	Gm45837
ENSMUSG00000031068	Glrx3
ENSMUSG00000031167	Rbm3
ENSMUSG00000031309	Rps6ka3
ENSMUSG00000031667	Aktip
ENSMUSG00000032295	Man2c1
ENSMUSG00000032942	Ucp3

ENSMUSG00000033020	Polr2f
ENSMUSG00000033191	Tie1
ENSMUSG00000035242	Oaz1
ENSMUSG00000035849	Krt222
ENSMUSG00000036585	Fgf1
ENSMUSG00000037266	Rsrp1
ENSMUSG00000037805	Rpl10a
ENSMUSG00000038550	Ciart
ENSMUSG00000038880	Mrps34
ENSMUSG00000040026	Saa3
ENSMUSG00000040269	Mrps28
ENSMUSG00000040459	Arglu1
ENSMUSG00000046691	Chtf8
ENSMUSG00000048007	Timm8a1
ENSMUSG00000052305	Hbb-bs
ENSMUSG00000054364	Rhob
ENSMUSG00000055116	Arntl
ENSMUSG00000056749	Nfil3
ENSMUSG00000057580	Gm10012
ENSMUSG00000058135	Gstm1
ENSMUSG00000059824	Dbp
ENSMUSG00000060147	Serpinb6a
ENSMUSG00000060152	Pop5
ENSMUSG00000063077	Kif1b
ENSMUSG00000064326	Siva1
ENSMUSG00000064791	Snord14e
ENSMUSG00000064796	Terc
ENSMUSG00000067547	Gm7666
ENSMUSG00000067847	Romo1
ENSMUSG00000069917	Hba-a2
ENSMUSG00000069919	Hba-a1
ENSMUSG00000070803	Cited4
ENSMUSG00000071347	C1qtnf9
ENSMUSG00000071540	3425401B19Rik
ENSMUSG00000072772	Grcc10
ENSMUSG00000073940	Hbb-bt
ENSMUSG00000076128	Mir686
ENSMUSG00000078915	Hsp25-ps1
ENSMUSG00000091562	Crybg3
ENSMUSG00000097656	Gm26712
ENSMUSG00000098462	Gm27694
ENSMUSG00000106362	AL672049.1
ENSMUSG00000107283	Mpv17
ENSMUSG00000107478	Gm45234

Supplementary Table 2. GSEA report for upregulated pathways

NAME	SIZE	ES	NES	NOM *p*-val	FDR *q*-val	FWER *p*-val	LEADING EDGE
**PANTHER_MM_INTEGRIN_SIGNALLING_PATHWAY**	54	0.605	2.231	0.000	0.000	0.000	tags=59%, list=22%, signal=75%
**INOH_MM_INTEGRIN**	35	0.665	2.190	0.000	0.000	0.000	tags=69%, list=23%, signal=88%
**WIKIPATHWAYS_MM_FOCAL_ADHESION-WP306**	54	0.592	2.162	0.000	0.000	0.000	tags=57%, list=22%, signal=72%
**PANTHER_MM_CYTOSKELETAL_REGULATION_BY_RHO_GTPASE**	26	0.636	1.983	0.000	0.002	0.011	tags=73%, list=27%, signal=100%
**NETPATH_MM_EGFR1_SIGNALING_PATHWAY**	136	0.426	1.826	0.000	0.019	0.108	tags=46%, list=27%, signal=61%
**WIKIPATHWAYS_MM_REGULATION_OF_ACTIN_CYTOSKELETON-WP51**	37	0.533	1.776	0.001	0.028	0.182	tags=62%, list=27%, signal=84%
**NETPATH_MM_WNT_SIGNALING_PATHWAY**	21	0.570	1.681	0.018	0.066	0.421	tags=48%, list=21%, signal=60%
**PANTHER_MM_HUNTINGTON_DISEASE**	43	0.472	1.653	0.006	0.078	0.529	tags=77%, list=40%, signal=126%
**PANTHER_MM_ALZHEIMER_DISEASE-PRESENILIN_PATHWAY**	16	0.611	1.651	0.014	0.071	0.534	tags=44%, list=8%, signal=47%

Supplementary Table 3. GSEA report for downregulated pathways

NAME	SIZE	ES	NES	NOM *p*-val	FDR *q*-val	FWER *p*-val	LEADING EDGE
**WIKIPATHWAYS_MM_ELECTRON_TRANSPORT_CHAIN-WP111**	64.000	–0.751	–3.185	0.000	0.000	0.000	tags=86%, list=20%, signal=105%
**WIKIPATHWAYS_MM_OXIDATIVE_PHOSPHORYLATION-WP623**	36.000	–0.764	–2.854	0.000	0.000	0.000	tags=81%, list=14%, signal=93%
**INOH_MM_CITRATE_CYCLE**	21.000	–0.716	–2.302	0.000	0.000	0.000	tags=76%, list=20%, signal=94%
**WIKIPATHWAYS_MM_FATTY_ACID_BETA_OXIDATION-WP143**	21.000	–0.706	–2.243	0.000	0.000	0.000	tags=67%, list=19%, signal=82%
**WIKIPATHWAYS_MM_TCA_CYCLE-WP78**	29.000	–0.627	–2.184	0.000	0.000	0.001	tags=69%, list=20%, signal=85%
**WIKIPATHWAYS_MM_GLYCOLYSIS_AND_GLUCONEOGENESIS-WP534**	27.000	–0.617	–2.156	0.000	0.000	0.002	tags=63%, list=21%, signal=79%

Supplementary Table 4. Gene alteration in WNT signaling pathway

PROBE	RANK IN GENE LIST	RANK METRIC SCORE	RUNNING ES
**SFRP2**	26	1.233115	0.151679
**LRP1**	188	0.748588	0.200758
**KREMEN1**	258	0.660285	0.265666
**CTNNB1**	266	0.650148	0.347624
**YWHAB**	400	0.535507	0.377453
**CCND1**	469	0.498205	0.42171
**RHOA**	494	0.479338	0.476556
**ILK**	519	0.463446	0.529349
**AKT1**	686	0.395137	0.531264
**RAC1**	724	0.381176	0.569574
**CSNK1A1**	1033	0.273293	0.513692
**CDH2**	1216	0.211877	0.487182
**PPP2CA**	1388	0.158804	0.457071
**MAPK1**	1410	0.15206	0.470506
**RYK**	1478	0.129619	0.467419
**JUP**	1635	0.085088	0.432221
**CTBP1**	1651	0.08129	0.438286
**PRKACA**	2156	–0.07241	0.2984
**PPP1CA**	2285	–0.10943	0.27464
**JUN**	2440	–0.15866	0.249543
**RAF1**	2683	–0.25956	0.21143

Supplementary Table 5. Gene alterations in integrin signaling pathway

PROBE	RANK IN GENE LIST	RANK METRIC SCORE	RUNNING ES
**COL8A1**	24	1.250044	0.041699
**ITGBL1**	34	1.204578	0.086106
**COL4A2**	46	1.108793	0.126169
**ITGB5**	51	1.086318	0.167447
**COL5A2**	128	0.846045	0.1778
**COL4A1**	129	0.839928	0.21064
**COL6A2**	131	0.83876	0.243136
**COL6A1**	132	0.838017	0.275902
**ACTN4**	141	0.824349	0.305741
**RHOB**	157	0.788537	0.332087
**FN1**	159	0.787086	0.362562
**COL3A1**	190	0.747269	0.382808
**RHOC**	213	0.717562	0.404286
**LAMC1**	314	0.605321	0.398049
**FLNA**	321	0.59947	0.419694
**CAV1**	363	0.55932	0.429302
**RAP1B**	389	0.540683	0.442966
**ITGB1**	425	0.525241	0.453036
**ARPC1A**	441	0.514583	0.46867
**COL1A2**	453	0.507689	0.485231

**CDC42**	459	0.505299	0.503493
**RHOA**	494	0.479338	0.512067
**ILK**	519	0.463446	0.52301
**TLN1**	521	0.462268	0.540786
**LAMA4**	542	0.455688	0.552622
**ARPC1B**	594	0.432813	0.554293
**ARPC5**	623	0.421344	0.562394
**ARF6**	631	0.418681	0.576671
**LAMB2**	669	0.401626	0.58131
**RRAS**	681	0.396598	0.593527
**RAC1**	724	0.381176	0.595871
**ACTB**	742	0.376136	0.605494
**ARPC3**	871	0.327444	0.580019
**MAP3K3**	883	0.324298	0.58941
**ARPC2**	934	0.305842	0.586416
**VCL**	1071	0.260906	0.555947
**ARL1**	1082	0.256929	0.563002
**ARF1**	1207	0.213645	0.534274
**MAP2K1**	1262	0.198721	0.525896
**CRK**	1363	0.166898	0.502517
**MAPK1**	1410	0.15206	0.494707
**ACTG1**	1494	0.125848	0.474807
**ACTN2**	1513	0.122105	0.474198
**LIMS1**	1678	0.071031	0.427932
**RAP1A**	1680	0.0706	0.430394
**LAMB1**	1707	0.063852	0.425115
**MAP2K2**	2045	–0.0357	0.325733
**LIMS2**	2364	–0.13732	0.236007
**ITGA7**	2570	–0.21246	0.18301
**RAF1**	2683	–0.25956	0.159666
**ARHGAP10**	2686	–0.26164	0.169298
**ARAF**	2689	–0.26324	0.178992
**PIK3R1**	2831	–0.32677	0.149604
**GRB2**	3099	–0.49504	0.089115

Supplementary Table 6. Gene alterations in fatty acid beta oxidation pathway

PROBE	RANK IN GENE LIST	RANK METRIC SCORE	RUNNING ES
**HADH**	1575	0.105087	–0.45296
**TPI1**	2110	–0.05695	–0.60381
**PNPLA2**	2190	–0.0827	–0.61663
**CHKB**	2217	–0.09075	–0.61273
**ACAT1**	2221	–0.0911	–0.60197
**ACSL1**	2484	–0.17422	–0.65729
**CPT1B**	2599	–0.22258	–0.66259
**DLD**	2746	–0.29057	–0.66868
**ACADL**	2764	–0.29748	–0.63569
**LPL**	2801	–0.31471	–0.60612

**ACADS**	2830	–0.32602	–0.57274
**CPT2**	2932	–0.38384	–0.55359
**ACADM**	3000	–0.4278	–0.51874
**GCDH**	3038	–0.45053	–0.47211
**DECR1**	3070	–0.4718	–0.42098
**HADHA**	3173	–0.55233	–0.38058
**SLC25A20**	3197	–0.57364	–0.31407
**CRAT**	3199	–0.58078	–0.24013
**ECHS1**	3263	–0.67054	–0.17307
**ACADVL**	3276	–0.68272	–0.08935
**ECI1**	3351	–0.97703	0.013621

Supplementary Table 7. Gene alterations in glycolysis and gluconeogenesis pathway

PROBE	RANK IN GENE LIST	RANK METRIC SCORE	RUNNING ES
PFKP	744	0.375565	–0.17815
HK1	842	0.339539	–0.16844
PFKL	1186	0.221405	–0.2451
GPI1	1476	0.130336	–0.31606
PGK1	1863	0.017227	–0.42862
TPI1	2110	–0.05695	–0.49514
PGAM1	2240	–0.09476	–0.52267
PKM	2281	–0.10786	–0.52231
PDHX	2291	–0.11455	–0.512
DLAT	2333	–0.12668	–0.5098
PFKM	2696	–0.26618	–0.58703
PGAM2	2704	–0.27234	–0.55824
HK2	2712	–0.27505	–0.52915
LDHA	2719	–0.27801	–0.49942
MDH2	2744	–0.29021	–0.47365
DLD	2746	–0.29057	–0.44102
PDHA1	2846	–0.3355	–0.43237
MDH1	2868	–0.35118	–0.3988
GOT2	3013	–0.4349	–0.39223
SLC2A4	3030	–0.4472	–0.3463
PDHB	3051	–0.45979	–0.30013
ALDOA	3116	–0.50893	–0.26144
ENO3	3147	–0.53595	–0.20961
LDHB	3201	–0.58166	–0.15941
ATP5G1	3220	–0.60351	–0.09636
GM10039	3224	–0.60566	–0.02861
GOT1	3286	–0.70273	0.032928

Supplementary Table 8. Gene alterations in TCA cycle pathway

PROBE	RANK IN GENE LIST	RANK METRIC SCORE	RUNNING ES
**PDK4**	431	0.518681	–0.07767
**PDP1**	899	0.318727	–0.1854
**OGDH**	1073	0.260313	–0.21152
**IDH2**	1230	0.209108	–0.23756
**SUCLG2**	2185	–0.08099	–0.51288
**DLST**	2266	–0.1051	–0.52644
**PDK1**	2288	–0.11027	–0.52199
**PDHX**	2291	–0.11455	–0.51148
**DLAT**	2333	–0.12668	–0.51138
**SDHD**	2725	–0.28036	–0.60027
**MDH2**	2744	–0.29021	–0.57748
**DLD**	2746	–0.29057	–0.54962
**IDH3G**	2754	–0.29351	–0.52326
**PDHA1**	2846	–0.3355	–0.51776
**MDH1**	2868	–0.35118	–0.48996
**ACO2**	2907	–0.37008	–0.46537
**SDHC**	2914	–0.37358	–0.43095
**IDH3A**	2931	–0.38174	–0.3987
**SDHB**	3022	–0.44	–0.38278
**SUCLA2**	3023	–0.44078	–0.34006
**CS**	3027	–0.4455	–0.29778
**IDH3B**	3028	–0.44555	–0.2546
**PDK2**	3041	–0.45338	–0.21423
**PDHB**	3051	–0.45979	–0.17234
**FH1**	3081	–0.47873	–0.13456
**SDHA**	3150	–0.53694	–0.10271
**SUCLG1**	3214	–0.59806	–0.06345
**ATP5G1**	3220	–0.60351	–0.00645
**GM10039**	3224	–0.60566	0.05135

Supplementary Table 9. Ninety five genes simultaneously differentially regulated at both 1 and 8 months after MI

gene	baseMean	baseMean	log2FC_1m	log2FC_8m	padj_1m	padj_8m
9330159F19Rik	72.38382	263.2386	1.338094853	0.772947365	0.017056744	0.013809142
Abca12	469.6926	907.8357	–1.58722346	–1.243272159	3.36E-10	0.000107469
AC022682.2	61.96831	80.27954	–1.994359763	–1.33919269	2.14E-05	0.039376723
AC157516.1	69.37918	179.0611	–2.157162233	–1.636440248	9.17E-06	0.002207262
Adamts20	33.94426	91.08125	1.77092724	1.660193472	0.00091548	3.46E-06
Adamtsl1	165.3911	496.4326	1.451609157	0.537405511	1.54E-07	0.022820712
Adcy8	27.36026	51.14039	–1.622768953	–1.993648065	0.007910621	3.46E-06
Add2	32.75743	39.58893	2.425409933	1.206885392	0.000488474	0.001304079
AL591207.3	17.84717	28.20068	3.507431259	1.499062707	0.000266199	0.02008895
Aldh1l2	338.0175	670.2361	–1.325518758	–0.744502034	8.32E-07	0.028921143
Ankrd63	33.96433	107.6297	–2.036353914	–1.157061168	6.31E-05	0.028436322
Aqp2	20.00909	44.6289	1.914123072	1.709417141	0.015780422	0.004250746
Atp8b4	89.5417	406.8387	1.814641735	–0.657079691	2.93E-08	0.049443001
Azin2	89.13142	209.8742	0.984562372	0.935053131	0.006373882	2.94E-07
B4galnt3	31.85274	75.94876	8.307744623	2.056435946	2.89E-08	1.54E-07
Btla	61.95872	128.2981	0.900534149	1.446972316	0.01685731	6.03E-06
Carmil3	84.0663	146.8175	–1.237885337	–0.736173573	0.004326839	0.007285041
Ccdc88b	102.5025	347.5408	0.963590651	–0.587584473	0.017493028	0.042258024
Ccnj	300.905	602.3457	–0.686261923	–0.381715554	0.000265836	0.022820712
Cd22	24.32314	59.99578	2.062886595	2.350666596	0.002042038	8.27E-07
Cdh3	19.31972	73.22186	7.587282833	1.05376394	3.70E-07	0.011799969
Cenpe	100.7449	246.5119	1.235381018	–0.838841904	0.00786079	0.008075965
Clec18a	42.49937	64.59779	–2.194397745	–1.259674449	0.000188506	0.009800352
Col11a1	150.3847	192.7107	5.530428761	1.319914559	1.78E-09	0.046809408
Col27a1	355.7013	443.4263	0.940474882	0.430679306	0.000897193	0.023879553
Crb1	47.02913	83.74538	–2.044517118	–1.229102818	0.000852805	0.012802213
Csf2rb2	57.18061	179.2012	2.032208005	1.050353439	1.25E-05	0.020314203
Dchs2	190.7537	267.483	3.014351889	1.556945263	9.63E-22	1.12E-06
Ereg	42.28248	116.9084	5.215061922	1.724405437	1.09E-09	1.41E-05
Esr1	75.94608	297.3961	1.41602485	0.764006532	0.003032061	0.047435928
Exoc3l2	59.00953	252.4834	1.083413536	0.730190962	0.008056726	0.008757768
Eya4	88.7854	157.3741	2.052361136	1.067523645	3.76E-06	0.008094616
Faim2	15.37698	87.69929	4.430092583	2.564079235	0.000253018	4.64E-08
Fam171b	119.2217	269.3606	1.146632937	0.709317266	0.004534949	0.000717559
Flt3	16.80729	54.71801	4.447239656	1.473136516	0.000609507	0.002537139
Fmn1	116.176	268.8948	1.599808852	1.346818513	1.48E-05	0.002798257
Gabrb3	21.80811	49.84956	2.087083127	1.684009619	0.002830015	0.002798257
Gabrr2	45.87044	68.02024	–1.866615384	–1.519079251	0.00026071	0.001018617
Gcnt4	45.7202	57.86883	2.894881795	1.358232514	5.21E-09	0.047670774
Gli2	85.43764	131.6428	1.820966739	0.679523708	1.43E-05	0.002270175
Gng8	43.2071	59.78843	3.703775196	2.125041857	6.28E-08	1.47E-05
Gprin3	79.06171	177.0166	0.982998678	1.286644524	0.026854629	0.002537139
Greb1	57.37384	57.11321	1.588753271	0.953314325	0.0055535	0.011017089
Gria3	193.0349	250.5424	1.905922134	0.805614693	1.30E-10	0.013596458
H2-Ob	46.0266	106.5466	1.145888193	1.039805101	0.01422812	0.009800352
Hebp2	48.05972	139.9614	2.209293862	0.740453665	4.19E-07	0.001097243
Hephl1	16.70996	48.75719	4.986008608	2.555201241	0.000360475	0.002237368
Hgf	95.50513	333.1287	1.023382578	0.613775256	0.014562949	1.76E-05

Il1rl2	173.3036	310.0596	0.738374965	0.762135194	0.025622396	0.032075891
Il6	24.57122	44.46563	3.580218916	2.015820222	1.59E-05	0.022246819
Kcna6	58.95865	148.0705	–1.690368067	–1.033712286	0.000326346	0.021063736
Kcnj14	49.22876	78.13598	1.106644663	1.423185549	0.0416764	0.000191724
Kif2c	16.6513	76.90939	2.289965649	–0.883948709	0.017160253	0.047670774
Kif6	109.24	196.2545	–1.022273418	–0.670650265	0.004250182	0.009567669
Klhl29	97.04787	144.4202	2.319237182	1.323077437	1.10E-12	8.27E-07
Lgi3	108.5846	196.035	–1.168515181	–1.149833657	0.001143076	0.000839157
Lrp8	52.63052	68.47234	2.17990467	1.688064528	2.89E-05	0.000705942
Mgat5b	21.88977	74.99011	3.284347677	1.681484684	0.000217058	0.014531136
Mmp12	29.26492	186.795	1.442865736	1.614741137	0.009105975	0.004494978
Mylk2	19.4292	23.98107	7.597182009	1.356109249	7.64E-07	0.01630661
Osgin1	70.18034	163.4612	–0.848248138	–0.766289406	0.045968856	0.038826495
P2ry10	25.68545	109.2988	1.507248036	1.3779083	0.02045988	0.000839157
P4ha3	26.37245	35.69365	8.037336988	2.220418479	2.24E-08	0.006814691
Padi4	28.44141	62.34945	1.469349664	1.206210285	0.027370478	0.008492938
Pak3	81.95312	166.8805	2.832568299	2.024932516	3.36E-10	7.13E-06
Pira2	17.9124	217.0514	3.275330959	–1.325123732	0.010418457	3.91E-05
Pitpnm3	122.0296	145.7451	–1.213835066	–0.642499843	6.31E-05	0.022820712
Rab17	50.76643	89.00408	–1.245182676	–1.058803418	0.00547896	0.007285041
Rasgef1a	37.1663	150.3048	1.412350672	1.577620716	0.004241616	1.36E-05
Rassf10	116.5135	245.6662	–0.585507308	0.525495859	0.043312449	0.049865687
Ret	212.9693	506.869	–1.404154551	–1.082278928	9.69E-10	3.10E-05
RMST_1	92.13399	161.2933	–1.081845815	–0.913073663	0.01617561	0.00097616
Scg2	33.79235	56.83588	8.395686327	4.52288006	3.61E-09	2.83E-13
Sec16b	270.5756	403.384	1.297867542	0.647384726	1.94E-10	0.001215566
Slc6a7	51.69015	41.8191	5.803694687	2.310969091	1.71E-12	0.001225144
Slit2	636.5364	1174.787	0.895635384	0.534440627	2.22E-05	1.71E-06
Sntg2	40.96716	119.7424	8.672300191	2.121286353	4.42E-10	5.02E-09
Specc1	290.5399	501.3615	0.598185465	0.459398396	0.011156035	0.000839157
Sphk1	59.47501	191.2715	1.368656126	0.89744868	0.002773203	0.001185865
Srgap3	131.9378	175.2212	2.65425815	0.822610595	3.61E-09	0.004971559
St14	35.53093	88.13187	3.92784732	1.01547955	7.49E-10	0.009497068
Steap2	119.16	258.9819	1.726286203	0.888052602	2.57E-08	0.001723239
Tbx15	49.93634	140.4692	1.104645046	1.524652685	0.015516497	0.000139045
Tc2n	46.51239	133.5308	2.431350799	0.823009946	2.12E-06	0.009369206
Tmem56	147.233	562.4513	–1.209475891	–1.009392798	0.000664798	3.46E-06
Tmprss13	39.14196	149.0266	–1.575697533	–1.033357272	0.00147481	0.044786353
Tnni2	28.0641	68.64875	–1.14286515	–0.915060635	0.03912346	0.004258662
Trpm6	47.30285	99.68346	2.001789475	1.131149033	3.13E-05	0.000705942
Wscd2	40.32155	83.34609	3.789955949	1.548127677	4.50E-07	0.002942176
Xylt1	244.5109	426.3965	1.122938768	0.540895006	2.19E-06	0.001160318
Zfp30	425.8207	634.8073	–0.752838829	–0.53850983	9.80E-06	0.008757768
Zfp354c	275.2212	512.1063	0.888658091	0.638990311	1.48E-06	0.001798011
Zfp423	154.85	365.9361	1.532558639	0.547260101	7.06E-08	0.001086937
Zfp783	80.43104	135.5416	1.066661879	0.809561367	0.002956994	0.002038413
Zfp995	85.12412	168.2397	0.868114571	0.684070644	0.041180192	0.002653893
