# Supplementary material for: Characterizing a long-term chronic heart failure model by transcriptomic alterations and monitoring of cardiac remodeling
Source: Aging (Albany NY). 2021 Apr 23;13(10):13585–614. doi: 10.18632/aging.202879 (PMC8202904; doi:10.18632/aging.202879)
Supplement: Supplementary Figures [file aging-13-202879-s001.pdf]

SUPPLEMENTARY FIGURES

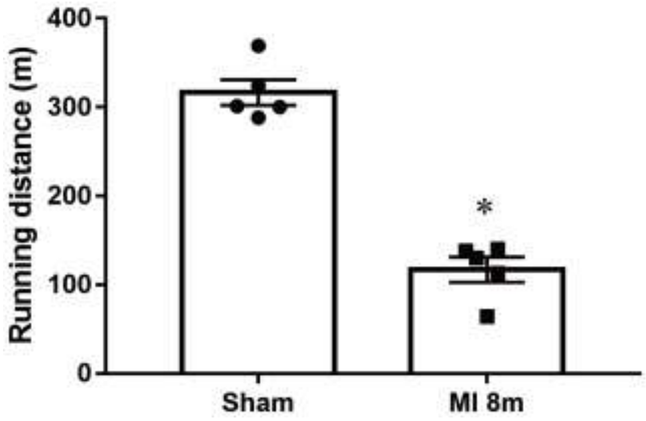

Supplementary Figure 1. Running distance during exercise exhaustion test in mice at 8 months after MI.

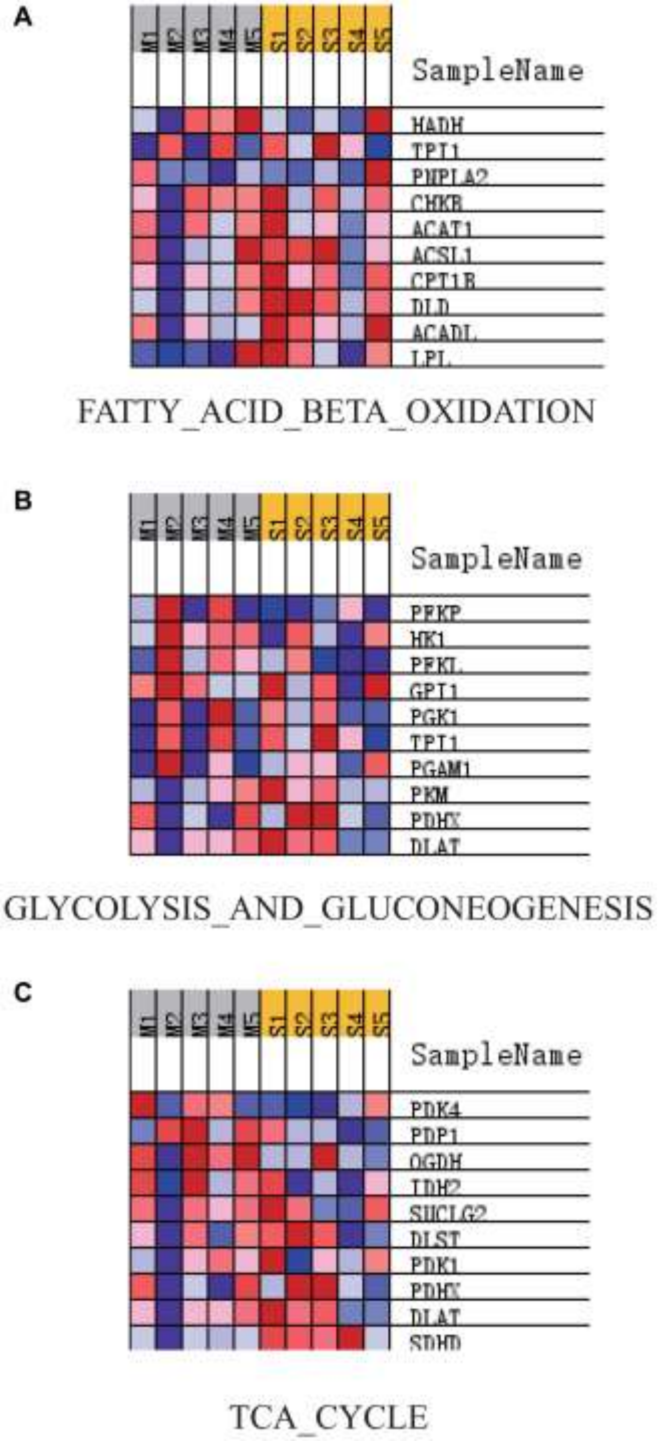

Supplementary Figure 2. Downregulated pathways identified by GSEA analysis. (**A**) Heatmap of the enriched genes in the fatty acid beta oxidation pathway. (**B**) Heatmap of the enriched genes in the glycolysis and gluconeogenesis pathway. (**C**) Heatmap of the enriched genes in the TCA cycle pathway.

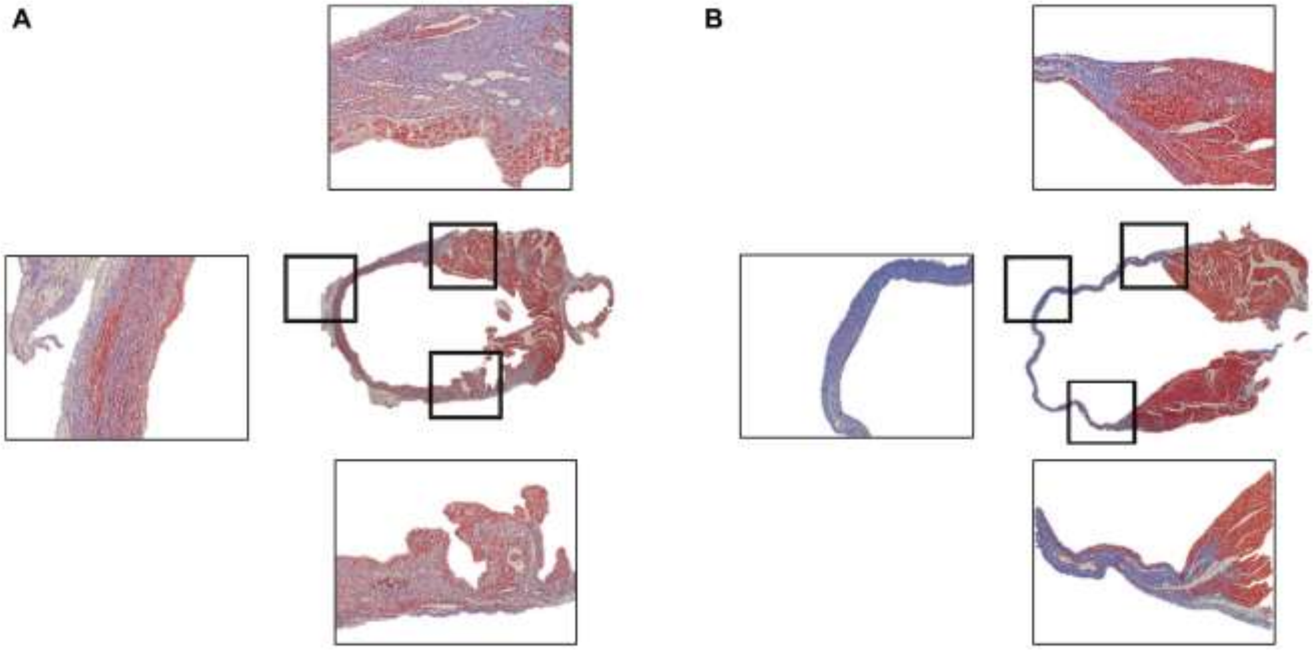

Supplementary Figure 3. Cardiac remodeling validated by detailed morphological and histological analysis. (**A**) Representative longitudinal section pictures of heart stained with Masson's trichrome staining from 4 weeks after myocardial infarction (MI). (**B**) Representative longitudinal section pictures of heart stained with Masson's trichrome staining from 8 months after MI. Zoomed frames indicated the areas around the infarct sites after MI.

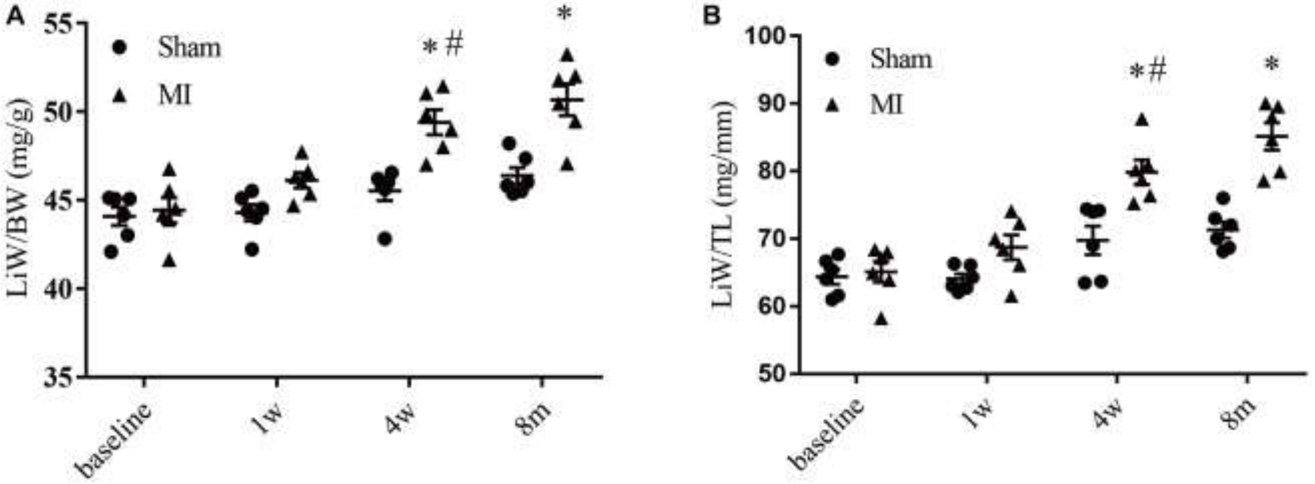

Supplementary Figure 4. The liver weight in mice at 8 months after MI. (**A**) The liver weight (mg) to body weight (g) ratio (LiW/BW). (**B**) The liver weight (mg) to tibia length (mm) ratio (LiW/TL). $^{*}P < 0.05$ vs. the corresponding time point in the sham group. $^{\#}P < 0.05$ compared with the prior time point in the MI group.

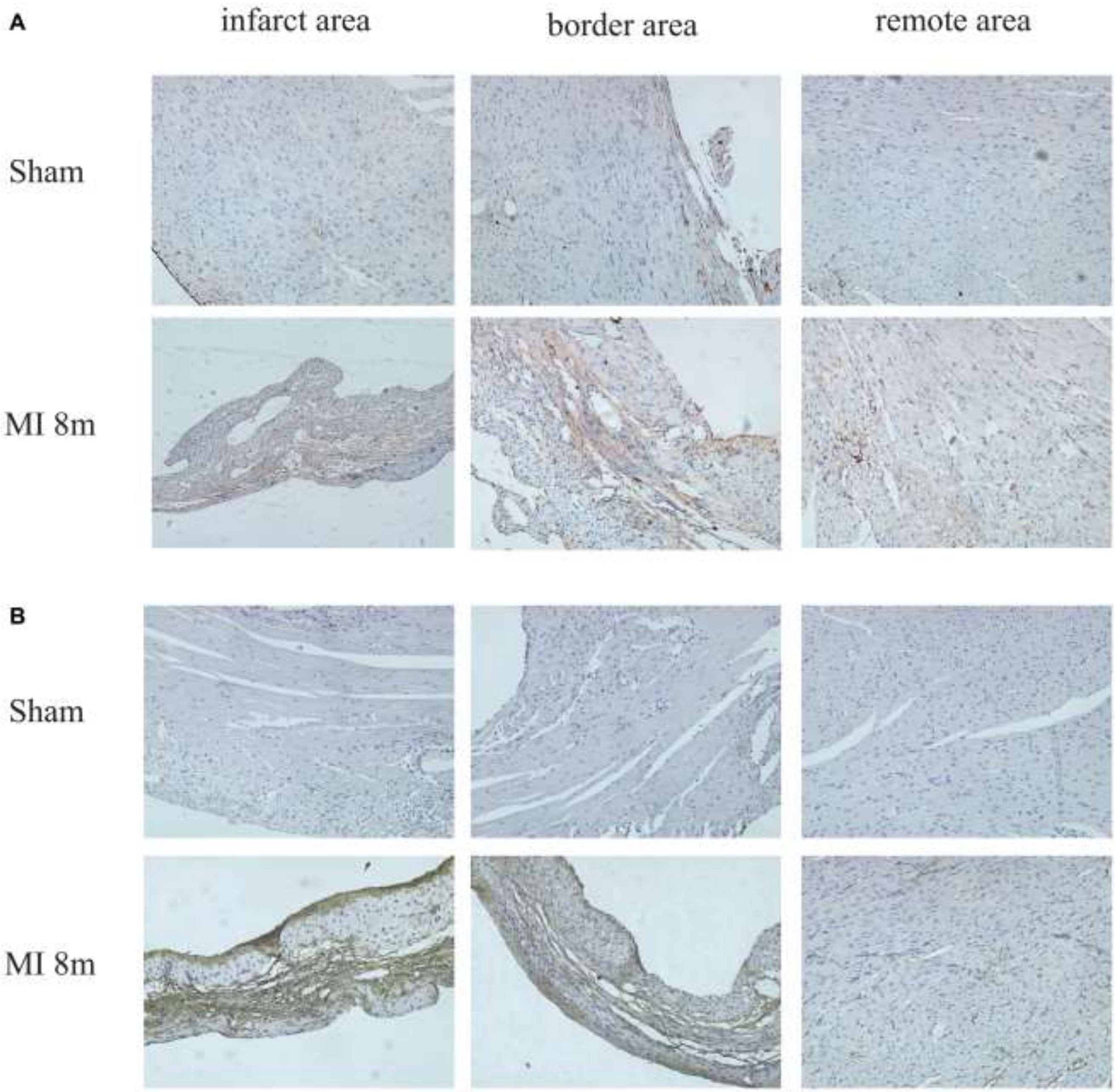

Supplementary Figure 5. Representative immunohistochemical staining graphs in the hearts. (**A**) Upper panel, immunohistochemical staining for β-catenin in different areas of the sham group hearts; Lower panel, immunohistochemical staining for β-catenin in different areas of the MI group hearts. (**B**) Upper panel, immunohistochemical staining for collagen-8 in different areas of the sham group hearts; Lower panel, immunohistochemical staining for collagen-8 in different areas of the MI group hearts. Left: infarct area; Middle: border area; Right: remote area.
